# Estimating the health and economic effects of the proposed US Food and Drug Administration voluntary sodium reformulation: Microsimulation cost-effectiveness analysis

**DOI:** 10.1371/journal.pmed.1002551

**Published:** 2018-04-10

**Authors:** Jonathan Pearson-Stuttard, Chris Kypridemos, Brendan Collins, Dariush Mozaffarian, Yue Huang, Piotr Bandosz, Simon Capewell, Laurie Whitsel, Parke Wilde, Martin O’Flaherty, Renata Micha

**Affiliations:** 1 Department of Public Health and Policy, University of Liverpool, Liverpool, United Kingdom; 2 School of Public Health, Imperial College London, London, United Kingdom; 3 Friedman School of Nutrition Science and Policy, Tufts University, Boston, Massachusetts, United States of America; 4 Department of Preventive Medicine and Education, Medical University of Gdansk, Gdansk, Poland; 5 American Heart Association, Washington, District of Columbia, United States of America; Stanford University, UNITED STATES

## Abstract

**Background:**

Sodium consumption is a modifiable risk factor for higher blood pressure (BP) and cardiovascular disease (CVD). The US Food and Drug Administration (FDA) has proposed voluntary sodium reduction goals targeting processed and commercially prepared foods. We aimed to quantify the potential health and economic impact of this policy.

**Methods and findings:**

We used a microsimulation approach of a close-to-reality synthetic population (US IMPACT Food Policy Model) to estimate CVD deaths and cases prevented or postponed, quality-adjusted life years (QALYs), and cost-effectiveness from 2017 to 2036 of 3 scenarios: (1) optimal, 100% compliance with 10-year reformulation targets; (2) modest, 50% compliance with 10-year reformulation targets; and (3) pessimistic, 100% compliance with 2-year reformulation targets, but with no further progress. We used the National Health and Nutrition Examination Survey and high-quality meta-analyses to inform model inputs. Costs included government costs to administer and monitor the policy, industry reformulation costs, and CVD-related healthcare, productivity, and informal care costs. Between 2017 and 2036, the optimal reformulation scenario achieving the FDA sodium reduction targets could prevent approximately 450,000 CVD cases (95% uncertainty interval: 240,000 to 740,000), gain approximately 2.1 million discounted QALYs (1.7 million to 2.4 million), and produce discounted cost savings (health savings minus policy costs) of approximately $41 billion ($14 billion to $81 billion). In the modest and pessimistic scenarios, health gains would be 1.1 million and 0.7 million QALYS, with savings of $19 billion and $12 billion, respectively. All the scenarios were estimated with more than 80% probability to be cost-effective (incremental cost/QALY < $100,000) by 2021 and to become cost-saving by 2031. Limitations include evaluating only diseases mediated through BP, while decreasing sodium consumption could have beneficial effects upon other health burdens such as gastric cancer. Further, the effect estimates in the model are based on interventional and prospective observational studies. They are therefore subject to biases and confounding that may have influenced also our model estimates.

**Conclusions:**

Implementing and achieving the FDA sodium reformulation targets could generate substantial health gains and net cost savings.

## Abstract 2

Jonathan Pearson-Stuttard and colleagues quantify the potential health and economic impacts of the United States Food & Drug Administration's proposed voluntary sodium reduction goals for processed and commercially prepared foods.

## Author summary

**Why was this study done?:**

- Sodium consumption is a leading modifiable risk factor for high blood pressure and cardiovascular disease in the US and worldwide. The US Food and Drug Administration (FDA) has proposed 2-year and 10-year voluntary sodium reduction targets for processed foods.
- The potential health and economic effects of successful implementation of the FDA’s voluntary reformulation proposal have not been quantified and would be of great interest to policy makers.

**What did the researchers do and find?:**

- We modeled and compared the potential health and economic effects of 3 scenarios of differing implementation of the FDA’s proposed voluntary sodium reformulation policy over a 20-year period.
- We found that the optimal scenario—100% compliance with the 10-year FDA targets—could prevent approximately 450,000 CVD cases, result in the gain of 2.1 million quality-adjusted life years (QALYs), and produce discounted cost savings of approximately $41 billion. In contrast, the modest scenario (50% compliance with the 10-year FDA targets) and the pessimistic scenario (100% compliance with the 2-year targets but no further progress) could yield health and economic gains half and a quarter as large, respectively.
- All 3 scenarios had a greater than 80% probability of being cost-effective by 2021 (incremental cost-effectiveness ratio < $100,000 per QALY) and cost-saving by 2031.

**What do these findings mean?:**

- Implementing the FDA’s sodium reformulation targets could generate substantial health gains and cost savings.
- If the FDA targets are implemented, efforts should focus on ensuring high compliance, to yield maximum health and economic gains for the US population.

## Introduction

Sodium consumption is a leading modifiable risk factor for higher blood pressure (BP) and cardiovascular disease (CVD) [1]. The excess risk associated with sodium consumption appears to be mainly mediated through the deleterious effect of excess sodium consumption on BP [2]. CVD remains the leading cause of mortality and morbidity in the US, generating approximately 800,000 deaths and 6 million hospital admissions annually [3]. These CVD burdens cost $318 billion annually in healthcare costs and an additional $237 billion in lost productivity, with further costs of informal care [4]. Average sodium intake in the US is approximately 3,400 milligrams per person per day, or 8.6 grams of salt, approximately 50% above the recommended upper bound consumption level of 2,300 mg/day [5]. About 75% of sodium intake comes from processed and commercially produced foods, making industry reformulation a major priority for reducing population sodium intake [6,7].

Consistent with World Health Organization (WHO) recommendations and other voluntary reformulation policies that have effectively lowered sodium intake in Finland, Turkey, and the United Kingdom [8], the US Food and Drug Administration (FDA) in 2016 proposed short-term (2 year) and long-term (10 year) voluntary, category-specific sodium reformulation targets for commercially processed, packaged, and prepared foods across 155 food categories [9]. This proposal was designed to support the 2015–2020 US dietary guidelines by encouraging food reformulation and new product development [5].

However, the potential health and economic effects of these proposed targets have not been quantified. In addition, in both the 2017 congressional budget and the current proposed 2018 House of Representatives agriculture appropriations bill, the US Congress has instructed the FDA not “to develop, issue, promote or advance final guidance applicable to food manufacturers for long term population-wide sodium reduction,” at least in part because of uncertainty about potential health effects. Recent studies have estimated the potential health gains of general sodium reduction in the US population, but without mapping the effects of the specific FDA-proposed policies for industry or taking a wider societal perspective [10]. In this study, we quantified the potential reductions in CVD and the economic impact of different levels of compliance with the 2016 proposal over a 20-year period. This investigation was performed as part of the Food-PRICE (Food Policy Review and Intervention Cost-Effectiveness) project.

## Methods

We used and extended the previously validated US IMPACT Food Policy Model [11,12] to assess the potential health and economic effects of the proposed FDA voluntary sodium reformulation policy over a 20-year period (2017 to 2036). We simulated 3 scenarios: (1) optimal, with optimal implementation of the proposed FDA policy, assuming all processed foods will be reformulated to the FDA proposed 2- and 10-year sodium targets; (2) modest, assuming 50% compliance with the proposed upper bound of the 2- and 10-year targets and 50% compliance with the main 2- and 10-year targets; and (3) pessimistic, assuming all processed foods will be reformulated to the 2-year target but with no further reformulation.

We compared these 3 scenarios with a counterfactual “no intervention” (baseline) scenario. For this, we assumed that the recent observed slow declining trends in sodium consumption [13] will continue in the future. In addition, we modeled a very low compliance scenario, with 7.5% of applicable foods compliant with the 10-year reformulation targets, and present these results in S1 Appendix (Extra Scenario; p. 28).

### The US IMPACT Food Policy Model

Our extended US IMPACT Food Policy Model is a stochastic dynamic microsimulation model that simulates the life course of synthetic individuals in a close-to-reality synthetic population under different policy scenarios. Compared to previous versions of the model, it allows for more detailed and flexible simulation of food policies in a competing risk framework, taking into account population heterogeneity and lag times between exposures and outcomes.

**Fig 1:**
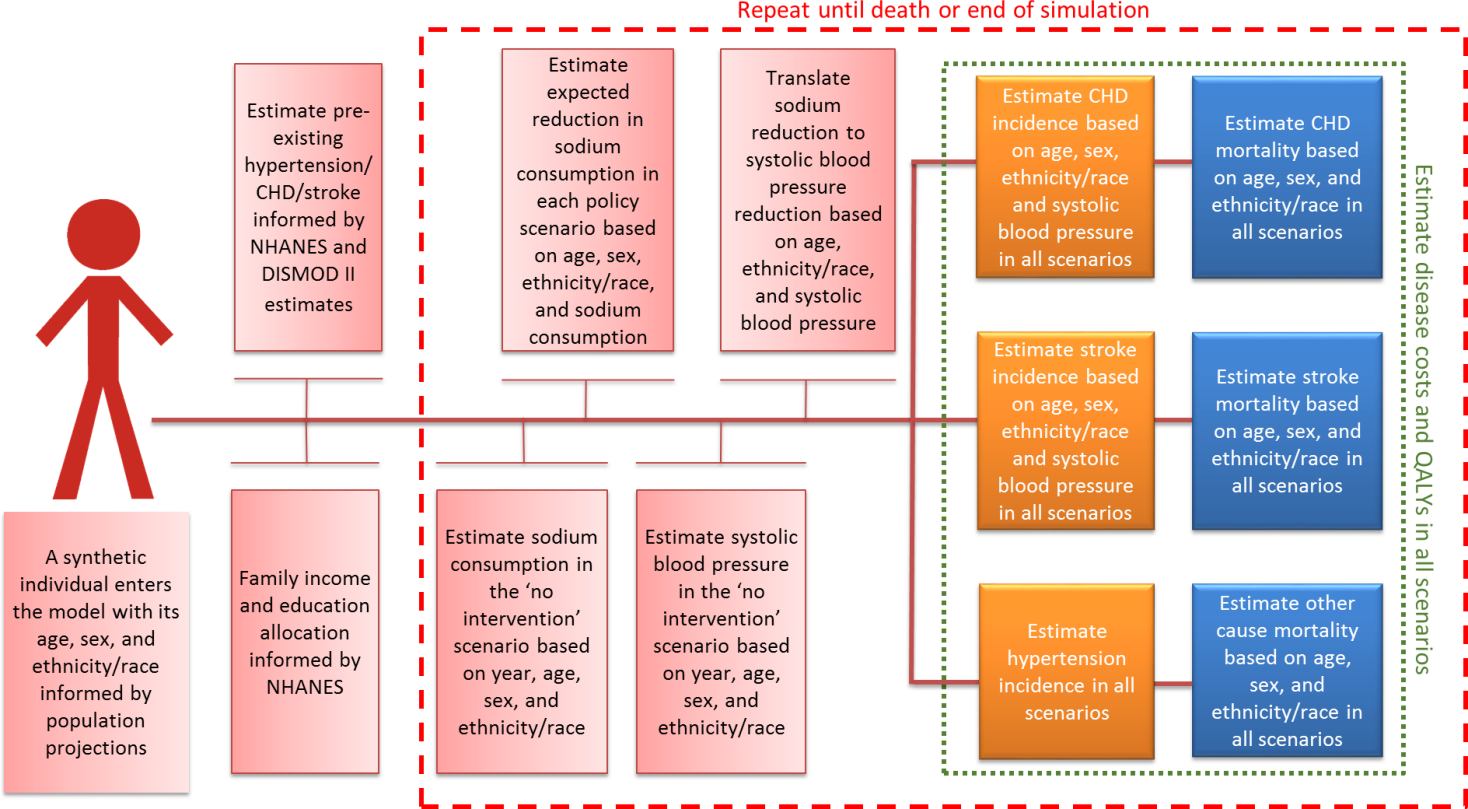
Simplified model structure. CHD, coronary heart disease; NHANES, National Health and Nutrition Examination Survey; QALY, quality-adjusted life year.

Specifically, the model first simulates the life courses of synthetic individuals aged 30 to 84 years under the “no intervention” scenario and records their sodium consumption, systolic BP (SBP), first episode of CVD (coronary heart disease [CHD] or stroke), quality-adjusted life years (QALYs), costs, and death from CVD or any other cause. Then it calculates the life courses of the same synthetic individuals under each of the 3 modeled sodium reformulation scenarios (optimistic, modest, and pessimistic) and records the differences in the aforementioned outcomes (Fig 1). Model data sources are outlined in Table 1. We further describe the model inputs, structure, key assumptions, and outputs below. Detailed description of the model, input sources, and key assumptions are detailed in S1 Appendix.

**Table 1:** The US IMPACT Food Policy Model data sources.

Parameter	Outcome	Details	Comments	Source
Population size estimates	Population	July 1 US resident population from the Vintage 2014 postcensal series, the revised 2000–2009 intercensal series, and the 1990–1999 intercensal series	Stratified by year, age, sex, bridged race, and Hispanic origin	CDC WONDER bridged-race population estimates 1990–2014 [14]
Population projections	Population	2014–2060 US population projections produced by the Census Bureau in 2014	Stratified by year, age, sex, race, and ethnicity	US Census Bureau via CDC WONDER national population projections 2014–2060 [15]
Mortality	Deaths from CHD, stroke, and any other non-modeled causes	Underlying cause of death 1999–2015	Stratified by year, age, sex, race, ethnicity, and cause of death	US Department of Health and Human Services and CDC NCHS via CDC WONDER underlying cause of death 1999–2015 [16]
Sodium exposure	Exposure of individuals	NHANES	Anonymized, individual-level datasets; years 2009–2014	CDC NCHS NHANES data [17]
Systolic blood pressure exposure	Exposure of individuals	NHANES	Anonymized, individual-level datasets; years 1999–2014	CDC NCHS NHANES data [17]
Effect of sodium consumption on systolic blood pressure	Systolic blood pressure change	Meta-analysis/meta-regression of 103 trials	Only trials with duration >7 days were analyzed	Text S1 in Mozaffarian et al. [2]
Reference level of sodium consumption	Ideal sodium consumption below which no risk was considered to occur	Evidence from ecological studies, randomized trials, and meta-analyses of prospective cohort studies	Intake levels associated with the lowest risk ranged from 614 to 2,391 mg/day; in large, well-controlled randomized feeding trials, the lowest tested intake for which blood pressure reductions were clearly documented was 1,500 mg/day	Text S4 and Table S3 in Mozaffarian et al. [2]
Relative risk for systolic blood pressure	CHD and stroke incidence (ICD-10: I20–I25 and I60–I69)	Pooled analysis of 2 individual-level meta-analyses	Stratified by age and sex; adjusted for regression dilution and total blood cholesterol and, where available, lipid fractions (HDL and non-HDL cholesterol), diabetes, weight, alcohol consumption, and smoking at baseline	eTable 5 in Micha et al. [1]
	Mortality from any cause excluding CHD and stroke	Individual-level meta-analysis of 48 prospective cohort studies	Adjusted for age, sex, race or ethnicity, deprivation, smoking, diabetes, inactivity, alcohol, and obesity	Figure 4 in Stringhini et al. [18]
Reference level of systolic blood pressure	Ideal systolic blood pressure below which no risk was considered to occur	Evidence from randomized trials of antihypertensive drugs and the INTERSALT study	There may be health benefits by lowering systolic blood pressure down to 110 mm Hg	Singh et al. [19]
Health state utility values	For CHD, stroke, hypertension, and their combinations	Uses EQ-5D-3L data from MEPS 2000–2002	We used the published regression coefficients to estimate utility values by age, sex, race, ethnicity, income, education, and number of chronic conditions	Tables 2 and 3 in Sullivan et al. [20]
Disease costs	Medical, mortality, and morbidity costs for CHD, stroke, and hypertension	Based on MEPS	Stratified by age, sex, and race; adjusted for comorbidities	Khavjou et al. [4]
	Informal care costs for stroke	Difference-in-differences technique in propensity-score-matched populations		Table 3 in Joo et al. [21]
	Informal care costs for CHD		Costs were extrapolated for US settings	Table 5 in Leal et al. [22]
Government costs to administer the policy		Administration costs for new restaurant menu and vending machine labeling regulation, including cost for outreach, education, review of regulatory issues, developing training for inspectors, and related functions		US Food and Drug Administration [23]
Government costs to monitor and evaluate the policy		UK Food Standards Agency impact assessment of UK salt reduction strategy	We assumed sodium reformulation to have same administrative costs	Collins et al. [24]
Industry costs to reformulate products		Spreadsheet model	The model accounted for variations in product formula complexity, company size, reformulation type, compliance period, and other factors	Muth et al. [25]

CDC, US Centers for Disease Control and Prevention; CHD, coronary heart disease; HDL, high-density lipoprotein; MEPS, Medical Expenditure Panel Survey; NCHS, National Center for Health Statistics; NHANES, National Health and Nutrition Examination Survey.

### Model inputs and structure

#### Demographics, sodium intake, and BP

The model synthesizes information regarding population structure by age, sex, and race/ethnicity [14] and data from the 2 most recent National Health and Nutrition Examination Survey (NHANES) cycles (2011–2014) [13] regarding sodium and SBP exposure, to prime a close-to-reality synthetic population. For this, the model draws the traits of the synthetic individuals from conditional distributions that were estimated from multinomial models fitted in the original survey data. The statistical framework of this method and its extension to modeling have been described elsewhere [26,27], and a detailed description and validation can be found in S1 Appendix. Then, the model projects the recent observed trends in SBP and sodium intake into the future, and uses the projections to evolve the traits of the synthetic individuals over time. We used NHANES 1999–2014 for the SBP projections and NHANES 2009–2014 for the sodium intake projections [13]. The inclusion of exposure trends in our analysis ensures more conservative estimates for the potential impact of the proposed FDA targets compared to an analysis assuming no trends.

#### CVD endpoints

We used the CDC WONDER database [14] to extract mortality rates for CHD (ICD-10: I20–I25), stroke (ICD-10: I60–I69), and any other cause for the years 1999–2015, stratified by age, sex, and race/ethnicity. We forecasted these trends to 2036, again providing a more appropriate and conservative estimate of the potential impact of the proposed FDA targets. Then, we used WHO DISMOD II to model the incidence and prevalence rates for CHD and stroke for 2014 [28]. To account for future trends in CHD and stroke incidence rates that are not attributable to SBP trends, we assumed that half of the forecasted annual change in CHD and stroke mortality rates is due to changes in incidence rates. We based this assumption on observational evidence from England and modeling studies in England and the US [29–32], and we included this assumption in our probabilistic sensitivity analysis (see below). Using a population attributable risk approach, the model calculates the annual risk of the synthetic individuals developing CHD and stroke based on their SBP and incidence rate forecasts using published relative risks. Finally, the model calibrates the annual case fatality for CHD, stroke, and any other cause to the forecasted mortality rates in a competing risk framework. Specifically, for “any other cause” mortality we assumed that hypertensive synthetic individuals had higher mortality rates to account for diseases other than CHD and stroke that we did not explicitly model but that are causally related to hypertension [18].

### Summary of evidence regarding the risks of excess sodium consumption

Excess dietary sodium consumption has been linked to an increased risk of CVD [33]. For CVD, the excess risk appears to be mainly mediated through the deleterious effect of excess sodium consumption on BP [2]. Our methods for evaluating the causality of effects of sodium reduction on BP and of BP reduction on CVD have been previously described [2].

There is some controversy regarding the optimal level of sodium consumption [34]. Some researchers claim that sodium consumption lower than 3,000 mg/day can actually increase the risk of CVD and overall mortality [35,36]. However, it appears that this argument is based on biased measurement methodology [37]. In a recent discussion on the subject, Mozaffarian et al. concluded that the optimal level of sodium consumption, below which further sodium reduction has no further health gains, is somewhere in the range of 614 mg/day to 2,391 mg/day [2]. In our study we incorporated the uncertainty around the ideal sodium consumption in our probabilistic sensitivity analysis.

Evidence that directly links sodium risk reversibility to CVD mortality or morbidity outcomes is lacking. A meta-analysis of several randomized control trials that tested low-sodium diets was underpowered and therefore inconclusive [38]. In comparison, a plethora of evidence exists supporting the effect of a low-sodium diet on BP, which appears to happen within weeks [2,39]. Finally, the cardiovascular risk reversibility of BP has been evident in several randomized control trials and appears to occur within a 5-year period [40].

### Policy effects

The FDA-proposed sodium reformulation policy included specific mean and upper bound sodium concentration targets at 2 and 10 years for 155 food categories [9]. In addition, the FDA also provided data to map these 155 food categories to the NHANES 2009–2010 24-hour recall dietary questionnaire [41]. These data enabled the model to estimate the potential impact of the modeled policies on every synthetic individual based on their age, sex, race/ethnicity, and sodium consumption in the “no intervention” scenario. The model then used the estimated reduction in sodium consumption of the synthetic individuals to calculate the effect upon their SBP using a published meta-regression equation [2]. We assumed a gradual reformulation of food products to targets, and immediate change in sodium intake in synthetic individuals according to reformulation. We also assumed that the reformulated products would sustain their sodium concentration thereafter. Although changes in sodium intake influence SBP within weeks [2,39], we conservatively assumed a median duration of 5 years from change in SBP to health outcomes.

### Model outputs

For each scenario, the model generated the total numbers of relevant events and reported cases and deaths prevented or postponed (CHD or stroke [CVD] or other), QALYs, life years gained, and disaggregated disease costs. We present the results for US adults aged 30 to 84 years from 2017 to 2036 (simulation horizon of 20 years), rounded to 2 significant digits.

### Medical costs and health state utility analysis

We calculated the health state utility values (preference weights) using published equations [20] that used EQ-5D-3L data from the Medical Expenditure Panel Survey (MEPS) 2000–2002 [20]. The disease medical, mortality, and morbidity costs per person-year were derived from an RTI International report that was based on MEPS [4]. We estimated informal care costs using published data [21,22]. All costs were stratified by age, sex, and race/ethnicity except informal care costs. The health state utility values were additionally stratified by income and education.

### Policy costs

Policy costs included government costs to administer and monitor the policy as well as industry costs incurred through reformulating products. By taking this societal perspective, we aimed to understand the impact of sodium reduction on the entire US economy. Specifically, for industry costs we used a reformulation cost model developed by RTI International under contract with the FDA [25]. The model accounts for variations in product formula complexity, company size, reformulation type, compliance period, and other factors, which produces a more accurate cost estimate compared to a standard per-product cost approach. Administrative costs were assumed to occur every year, with monitoring and evaluation costs occurring every year after full policy implementation at year 3. We assumed the industry costs were equal in the 2 rounds of reformulation (2- and 10-year targets) except for the pessimistic scenario (which had no costs for the second round), and divided the costs over the policy implementation years (intervention years 1–3 for the first round, and intervention years 4–10 for the second round). We assumed no policy costs after intervention year 10.

### Cost-effectiveness analysis

To inform cost-effectiveness from different relevant perspectives, we evaluated both societal and healthcare cost perspectives, closely adhering to the recommendations from the Second Panel on Cost-Effectiveness in Health and Medicine [42]. All costs were inflated to 2017 US dollars using the Bureau of Labor Statistics Consumer Price Index and discounted at a 3% annual rate. We also discounted QALYs at the same rate. We assumed a willingness to pay of $100,000 per QALY [43].

### Sensitivity and uncertainty analyses

We performed probabilistic sensitivity analysis via a second-order Monte Carlo approach that allowed the estimated uncertainty of different model parameters and population heterogeneity to be propagated to the outputs [44]. The sources of uncertainty we considered were the sampling errors of baseline sodium intake, baseline SBP, and the relative risk of CHD and stroke based on SBP; the uncertainties around the lowest sodium and SBP exposures below which no risk is observed; the uncertainty around the effect of sodium on SBP; the uncertainty around the true incidence of CHD and stroke; the uncertainty of mortality forecasts; the uncertainty around which foods will be reformulated; the uncertainty around the quality of life decrements used to calculate QALYs; and the uncertainty of all the costs. We summarize the output distributions by reporting the medians and 95% uncertainty intervals (UIs). We also plotted the annual probability that a scenario was cost-effective or cost-saving over the simulation period. Finally, both discount rate and willingness to pay values were included in a 1-way sensitivity analysis and allowed to vary in steps between 0% and 9% and between $50,000 and $150,000, respectively. Please refer to S1 Appendix (specifically Tables D–F) for more information.

## Results

### Health-related outcomes

**Fig 2:**
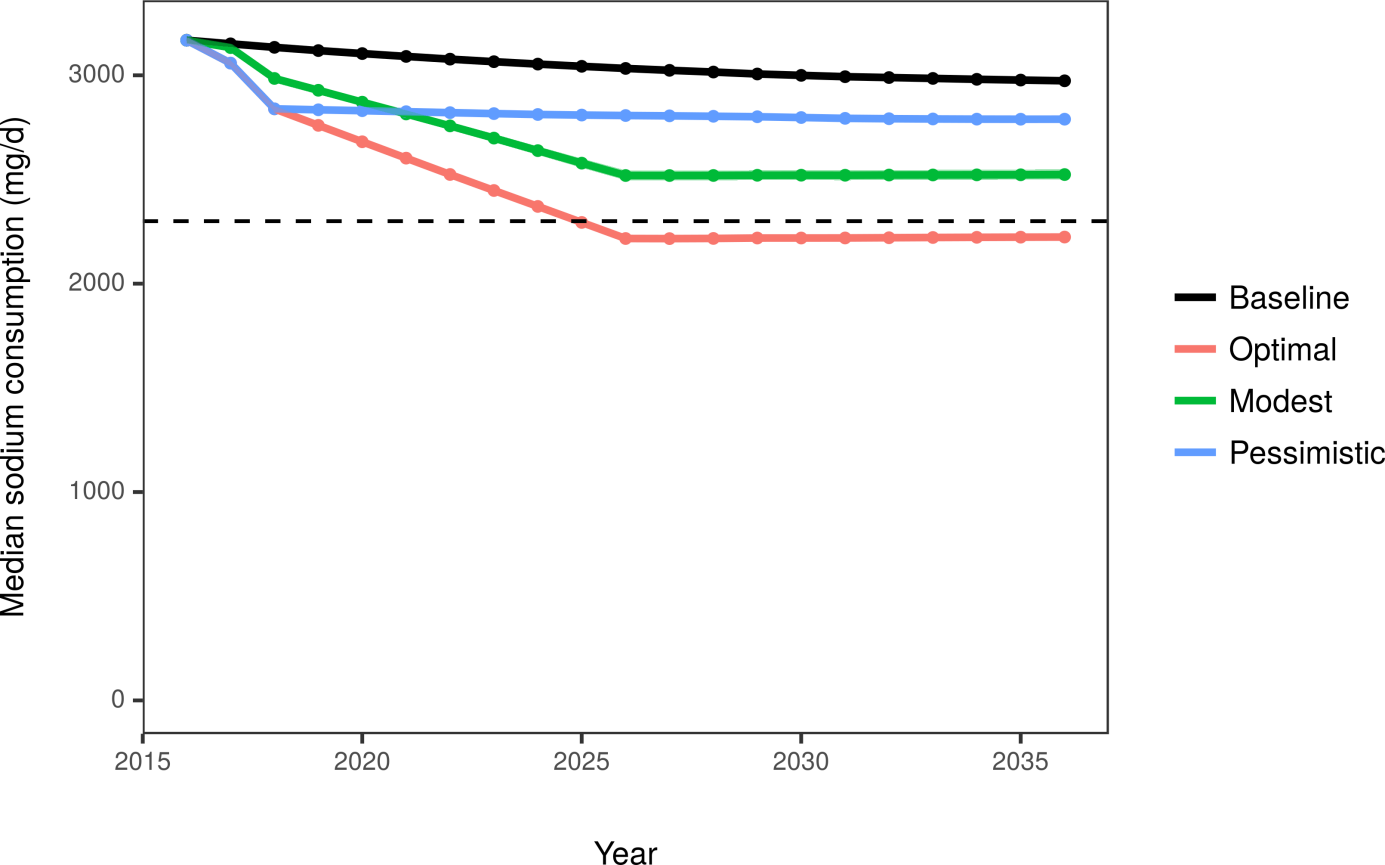
Median US sodium consumption among adults aged 30–84 years under the baseline projection and 3 modeled scenarios. The dashed horizontal line depicts the 2015–2020 Dietary Guidelines for Americans recommended upper bound of 2,300 mg/day [5].

In the baseline scenario, median sodium consumption modestly declined from 3,150 mg/day in 2017 to 2,974 mg/day in 2036. In the optimistic, modest, and pessimistic scenarios, sodium consumption was projected to fall to 2,224 mg/day, 2,524 mg/day, and 2,789 mg/day, respectively, in 2036 (Table 2; Fig 2). The resulting difference in median sodium consumption between the optimal and pessimistic scenarios resulted in a 1.0-mm Hg difference in median SBP. These differences were larger in specific subgroups, for example older adults, those with hypertension, and black individuals.

**Table 2:** Health-related model estimates over the 20-year simulation period from 2017 to 2036, for US adults aged 30 to 84 years.

Outcome	Optimal policy scenario	Modest policy scenario	Pessimistic policy scenario
Median sodium consumption in 2036 (mg/day)	2,224 (2,214 to 2,233)	2,524 (2,500 to 2,550)	2,789 (2,779 to 2,800)
Median SBP in 2036 (mm Hg)	114.0 (113.8 to 114.1)	114.5 (114.4 to 114.7)	115.0 (114.9 to 115.2)
CHD cases prevented or postponed	260,000 (110,000 to 490,000)	120,000 (48,000 to 240,000)	63,000 (17,000 to 130,000)
Stroke cases prevented or postponed	180,000 (78,000 to 340,000)	93,000 (33,000 to 180,000)	52,000 (11,000 to 110,000)
CHD deaths prevented or postponed	22,000 (−3,700* to 54,000)	11,000 (−13,000* to 37,000)	7,400 (−15,000* to 32,000)
Stroke deaths prevented or postponed	13,000 (−3,700* to 32,000)	7,400 (−9,000* to 22,000)	5,600 (−9,000* to 20,000)
Non-CVD deaths prevented or postponed	48,000 (13,000 to 85,000)	24,000 (−5,500* to 54,000)	7,400 (−19,000* to 37,000)
All deaths prevented or postponed	83,000 (50,000 to 120,000)	41,000 (17,000 to 71,000)	22,000 (0 to 45,000)
Life years gained	530,000 (290,000 to 830,000)	260,000 (87,000 to 480,000)	180,000 (26,000 to 370,000)
Discounted QALYs gained(millions)	2.1 m (1.7 m to 2.4 m)	1.1 m (0.91 m to 1.3 m)	0.69 m (0.54 m to 0.86 m)

Values are the median estimate (95% uncertainty interval). Results are rounded to first decimal for SBP, fourth significant digit for sodium consumption, and second significant digit for other outcomes.

*Negative numbers of deaths prevented or postponed for specific causes of death are a direct consequence of the mortality competing risk framework we implemented in the model. They represent synthetic individuals for whom the prevention of their death from a specific disease (i.e., CHD) due to the policy led to their death from another competing cause (i.e., non-CVD) in the same year.

CHD, coronary heart disease; CVD, cardiovascular disease; m, million; QALY, quality-adjusted life year; SBP, systolic blood pressure.

Optimal implementation, achieving the 2015–2020 US dietary guidelines national target of <2,300 mg/day sodium consumption, could potentially prevent or postpone approximately 35,000 CVD deaths (95% UI: 3,700 to 78,000) and 450,000 cases of CVD from 2017 to 2036 (95% UI: 240,000 to 740,000), and potentially generate 2.1 million (95% UI: 1.7 million to 2.4 million) discounted QALYs between 2017 and 2036 (61 QALYs per 100,000 person-years) (Table 3). The modest and pessimistic scenarios might potentially prevent or postpone approximately half as many (220,000) and a quarter as many (120,000) total cases, respectively. Both could still substantially improve the health of the US population, with proportional findings for CVD deaths and QALYs.

**Table 3:** QALYs gained and costs per 100,000 person-years.

Outcome	Optimal scenario	Modest scenario	Pessimistic scenario
**QALYs gained per 100,000 person-years (undiscounted)**	61 (50 to 71)	33 (27 to 40)	19 (14 to 24)
**Net cost per 100,000 person-years (undiscounted, medical perspective)**	−550,000 (−1,200,000 to −28,000)	−240,000 (−570,000 to 69,000)	−120,000 (−350,000 to 73,000)
**Net cost per 100,000 person-years (undiscounted, societal perspective)**	−1,400,000 (−2,700,000 to −640,000)	−680,000 (−1,400,000 to −240,000)	−410,000 (−880,000 to −89,000)

Costs are in 2017 US dollars. Negative costs represent savings. Readers can calculate similar estimates for other outputs by dividing by 4.7 billion (the number of person-years over the 20-year simulated period).

QALY, quality-adjusted life year.

The absolute health benefits from the optimistic scenario would be approximately 50% greater among men than among women, reflecting men’s higher sodium intake and higher CVD burden. The benefit would also be greater among non-Hispanic black individuals than among non-Hispanic white individuals, reflecting the higher SBP, higher CVD burden, and greater sensitivity to sodium changes of black individuals [18]. Finally, the largest number of CVD cases would be prevented in the oldest age group (70–84 years), while middle-aged individuals (50–69 years) would gain the most QALYs (please see S1 Appendix, Tables G–I).

### Costs and cost-effectiveness

**Fig 3:**
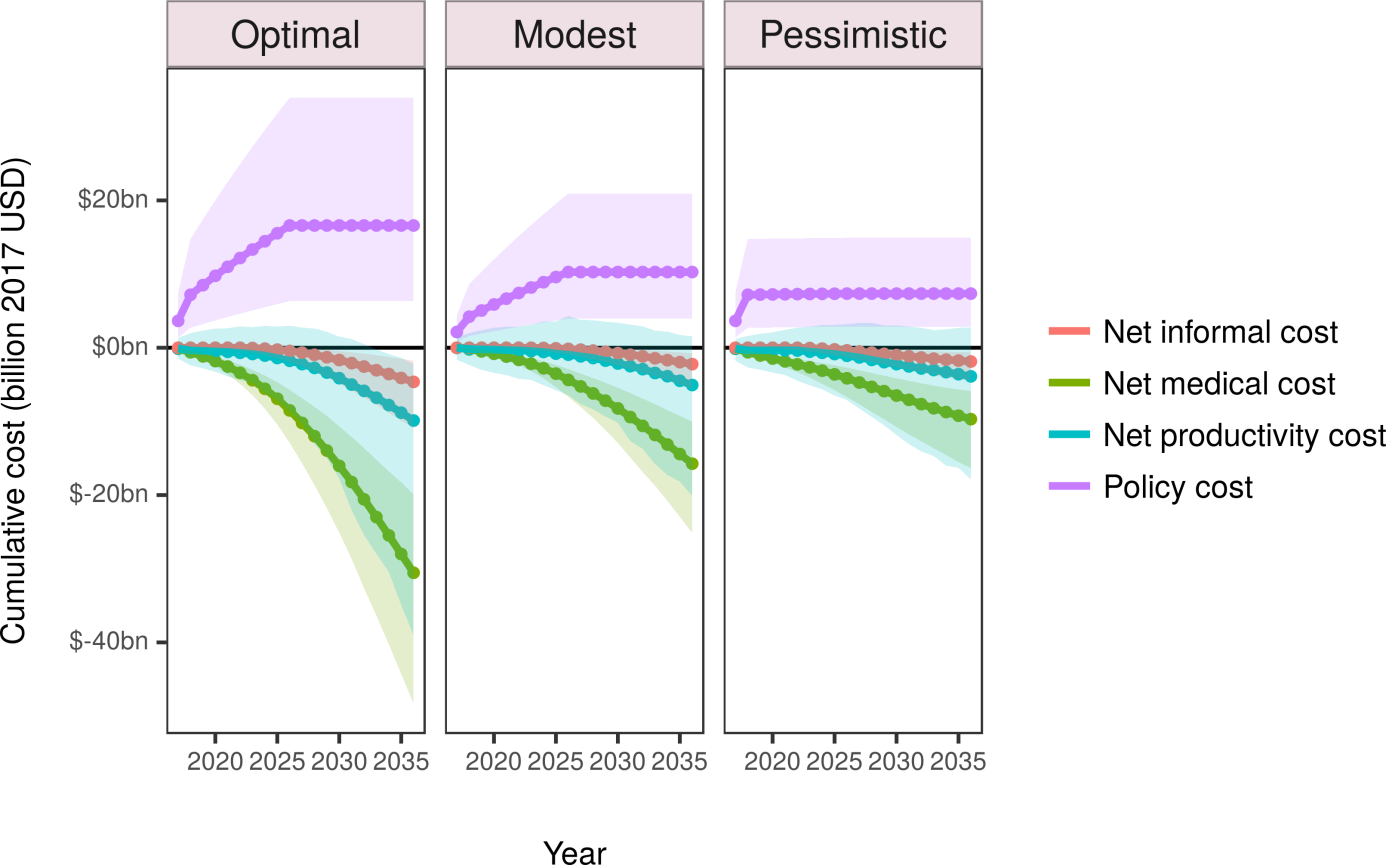
Estimated disaggregated discounted cumulative costs for the simulated period 2017 to 2036. Negative costs represent savings. The shaded areas depict 95% uncertainty intervals. USD, US dollars.

From a healthcare perspective (government and private payers), the optimal scenario would result in an approximately $31 billion (95% UI: $20 billion to $48 billion) reduction in total net costs, a substantial saving over the 20-year period (Table 4; Fig 3). The pessimistic scenario would still yield one-third of the savings, some $9.7 billion (95% UI: $5.9 billion to $16 billion). From the societal perspective, the net savings from 2017 to 2036 would be even larger: an approximately $41 billion (95% UI: $14 billion to $81 billion) reduction in net costs in the optimal scenario. More than 95% of policy costs would be attributable to industry costs of reformulation, with less than 5% attributable to government costs. The largest health-related cost savings for all scenarios would be generated from hypertension medical and productivity costs. The optimal scenario would yield more than 3 times more healthcare and societal savings per 100,000 person-years than the pessimistic scenario (Table 3).

**Table 4:** Impact inventory and cost-effectiveness analysis of model outputs for individuals aged 30 to 84 years, assessed cumulatively over the 20-year simulation period from 2017 to 2036.

Output	Optimal policy scenario	Modest policy scenario	Pessimistic policy scenario
**Change in health-related costs**	−57 bn (−97 bn to −38 bn)	−30 bn (−50 bn to −18 bn)	−19 bn (−35 bn to −9.9 bn)
Hypertension medical costs	−18 bn (−24 bn to −12 bn)	−9.3 bn (−13 bn to −6.4 bn)	−4.4 bn (−6.4 bn to −3 bn)
Hypertension productivity costs	−12 bn (−16 bn to −8.1 bn)	−6.4 bn (−8.8 bn to −4.4 bn)	−3.5 bn (−5 bn to −2.3 bn)
CHD medical costs	−7.1 bn (−16 bn to −2.4 bn)	−3.3 bn (−8 bn to −1 bn)	−2.8 bn (−6.5 bn to −0.76 bn)
CHD mortality productivity costs	−4.8 bn (−26 bn to 0.92 bn)	−2.3 bn (−13 bn to 3.3 bn)	−1.8 bn (−12 bn to 4.1 bn)
CHD morbidity productivity costs	−1.3 bn (−3.4 bn to −0.34 bn)	−0.64 bn (−1.7 bn to −0.14 bn)	−0.5 bn (−1.3 bn to −0.1 bn)
CHD informal care costs	−1.5 bn (−3.5 bn to −0.51 bn)	−0.69 bn (−1.7 bn to −0.2 bn)	−0.58 bn (−1.4 bn to −0.16 bn)
Stroke medical costs	−5.4 bn (−13 bn to −1.9 bn)	−2.9 bn (−6.9 bn to −0.81 bn)	−2.4 bn (−5.8 bn to −0.64 bn)
Stroke mortality productivity costs	−2.3 bn (−12 bn to 1.2 bn)	−1.3 bn (−7.8 bn to 2.3 bn)	−1.0 bn (−7.3 bn to 2.5 bn)
Stroke morbidity productivity costs	−0.76 bn (−1.9 bn to −0.23 bn)	−0.41 bn (−1.1 bn to −0.09 bn)	−0.33 bn (−0.87 bn to −0.051 bn)
Stroke informal care costs	−3.1 bn (−8.1 bn to −0.91 bn)	−1.5 bn (−4.4 bn to −0.35 bn)	−1.2 bn (−3.5 bn to −0.26 bn)
**Change in policy costs**	17 bn (6.3 bn to 34 bn)	10 bn (4.0 bn to 21 bn)	7.3 bn (2.9 bn to 15 bn)
Policy administration costs	0.16 bn (0.12 bn to 0.22 bn)	0.16 bn (0.12 bn to 0.22 bn)	0.16 bn (0.12 bn to 0.22 bn)
Policy monitoring costs	0.029 bn (0.021 bn to 0.039 bn)	0.029 bn (0.021 bn to 0.039 bn)	0.029 bn (0.021 bn to 0.039 bn)
Policy industry costs	16 bn (6.1 bn to 34 bn)	10 bn (3.8 bn to 21 bn)	7.2 bn (2.7 bn to 15 bn)
**Total net cost (medical perspective)**	−31 bn (−48 bn to −20 bn)	−16 bn (−25 bn to −10 bn)	−9.7 bn (−16 bn to −5.9 bn)
**Total net cost (societal perspective)**	−41 bn (−81 bn to −14 bn)	−19 bn (−41 bn to −3.4 bn)	−12 bn (−28 bn to 0.39 bn)
**Net monetary benefit (valuing 1 QALY at $100,000)**	250 bn (190 bn to 300 bn)	130 bn (100 bn to 170 bn)	81 bn (59 bn to 110 bn)
**Incremental cost-effectiveness ratio (2017 US dollars per QALY)**	Dominant (dominant to dominant)	Dominant (dominant to dominant)	Dominant (dominant to 540)

Results are rounded to the second significant digit. Costs are median of each distribution so may not add up to totals. Negative costs represent savings. Costs are presented in billions of discounted 2017 US dollars. Dominant = less costly and more effective than the alternative.

bn, billion; CHD, coronary heart disease; QALY, quality-adjusted life year.

**Fig 4:**
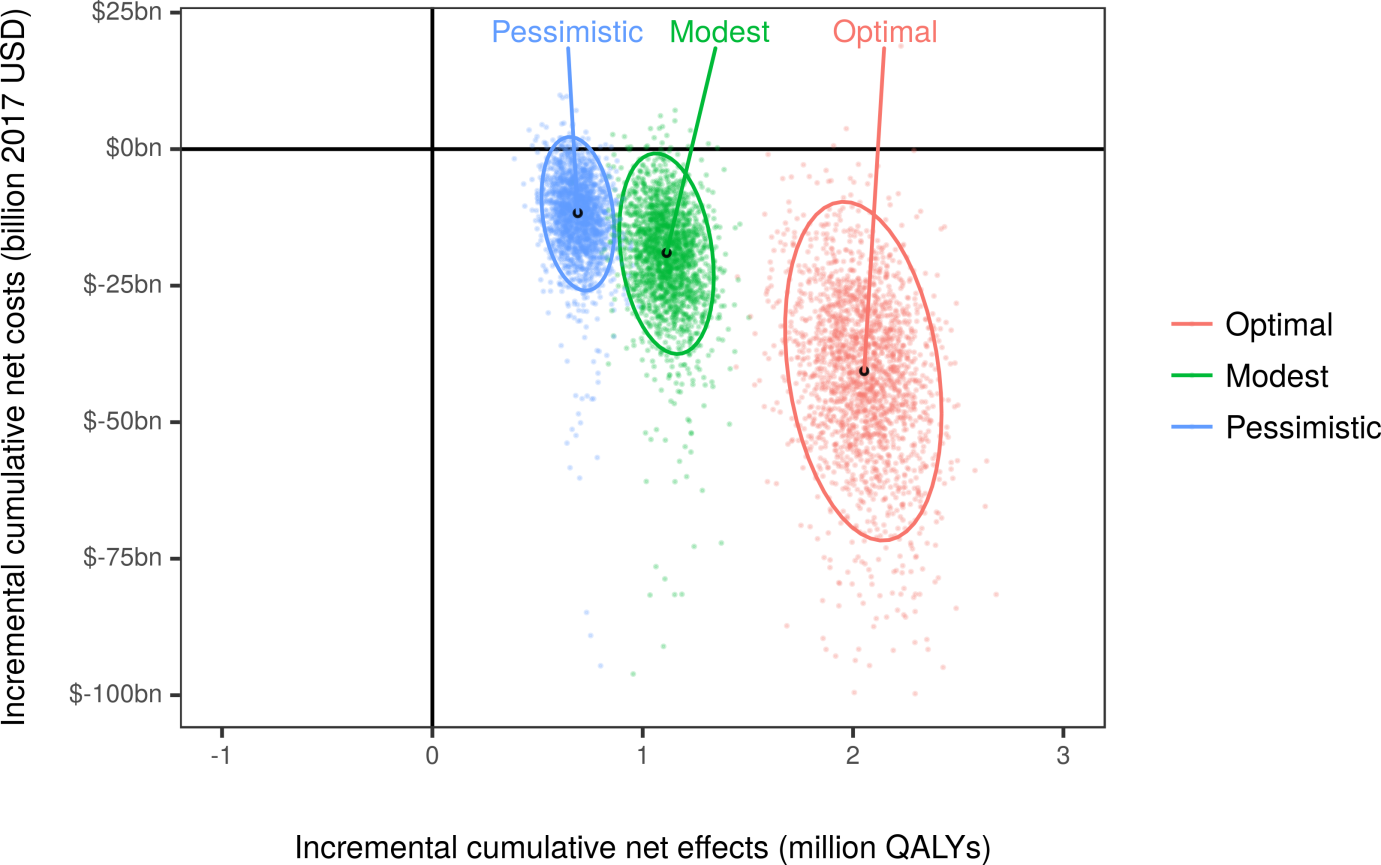
Cost-effectiveness plane by the end of simulation (year 2036). Each colored dot is the result of a stochastic Monte Carlo iteration. The black dots are the median combinations of cumulative discounted net costs (2017 US dollars) and discounted net QALYs for each simulated scenario, and the ellipses depict the 95% uncertainty interval. Negative costs represent savings. QALY, quality-adjusted life year; USD, US dollars.

All reformulation scenarios would be cost-effective, with the optimal and modest scenarios being dominant, i.e., cost-saving and producing more health than the baseline case. The optimal scenario would be approximately twice and 3 times as cost-effective as the modest and pessimistic scenarios, respectively (Fig 4; S1 Animation), generating a net monetary benefit of approximately $250 billion (95% UI: $190 billion to $300 billion), with each QALY gained valued at $100,000.

### Probability of cost-effectiveness and sensitivity analysis

**Fig 5:**
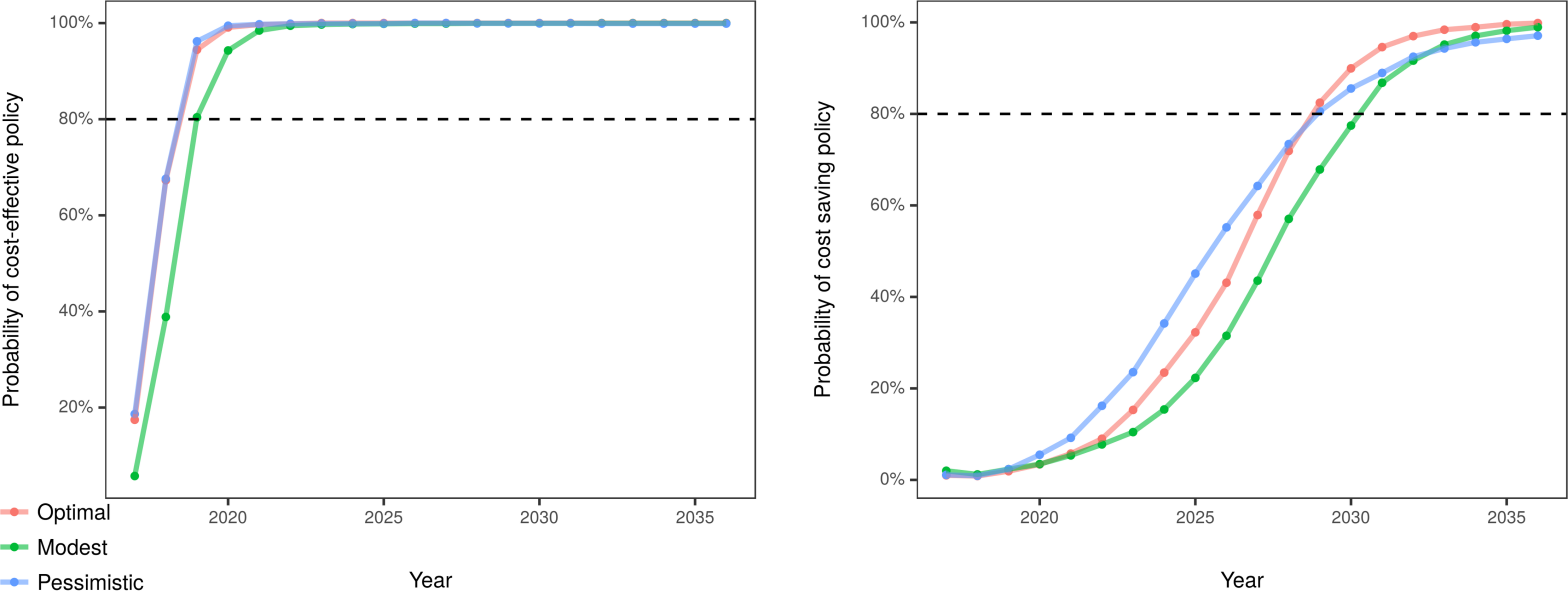
Estimated probability of cost-effective and cost-saving policy over the 20-year simulated period. Cost-effectiveness at the willingness to pay value of $100,000 per quality-adjusted life year.

Including costs, we estimated a probability of near 100% that the optimal and pessimistic scenarios would become cost-effective by 2021, and the modest scenario by 2023. All scenarios were likely to be cost-saving by 2036 (99.9%, 99.0%, and 97.1% probability for optimal, modest, and pessimistic scenarios, respectively). The optimal and pessimistic scenarios would have more than 80% probability of becoming cost-saving by 2029, and the modest scenario by 2031 (Fig 5). In a set of 1-way sensitivity analyses, net monetary benefit remained positive when willingness to pay for a QALY was varied down from $100,000 to $50,000, and when annual discount rates were varied up from 3% to 9% (see S1 Appendix, Tables E and F).

## Discussion

We used a microsimulation approach of a close-to-reality synthetic population (US IMPACT Food Policy Model) to estimate the potential health and economic effects, over a 20-year period, of the FDA’s proposed voluntary sodium reformulation targets under 3 scenarios of differing compliance. Our study suggests that implementation of and full compliance with the FDA voluntary sodium reformulation targets would result in substantial decreases in CVD incidence and mortality whilst also offering impressive cost savings to the healthcare payers and the wider economy. The optimal scenario saved the most lives and generated the most QALYs and economic savings. However, even lower compliance, i.e., with just the 2-year targets or 50% of the 10-year targets, yielded health and cost savings. This finding highlights the substantial health and economic opportunity costs of inaction or poorly sustained efforts to reduce sodium consumption.

Suboptimal diet is a leading cause of mortality and morbidity in the US and worldwide, with excess sodium being a significant contributor [1]. The burden of hypertension continues to grow despite advances in screening and evidence-based medications [45], emphasizing the importance of population initiatives to reduce BP. Due to their well-documented effects on BP, sodium reduction policies have been characterized as a “best buy” government intervention by WHO. Despite this and many efforts, sodium remains overconsumed in the US, highlighting the challenging nature of dietary behavior change. To date, the largest population-wide reductions in sodium consumption have been achieved in Finland, Japan, and the UK via comprehensive “upstream” strategies involving population-wide, multicomponent policies. In contrast, more “downstream” approaches such as individual approaches and worksite or community interventions are much weaker [8], again demonstrating the effectiveness hierarchy of public health interventions [46]. These declines in population sodium consumption in Finland, Japan, and the UK have corresponded with expected reductions in population BP, supporting our findings. In addition, long-term follow-up from the largest randomized control trials of sodium reduction demonstrated expected reductions in risk of CVD events [47]. Gradual sodium reduction achieves mean population sodium intake reductions without noticeable changes to consumers and their palates [48–50]. This is unlikely to trigger compensatory behaviors resulting in additional sodium used by the consumer at the table or in cooking [51–53]. Together with prior results, our investigation therefore supports the potential health and economic benefits of implementing the proposed FDA voluntary sodium reformulation targets.

When stratified by population subgroup, our results suggest the largest beneficial effects in non-Hispanic black individuals, based on higher BP responsiveness and CVD mortality rates [54]. These findings suggest additional benefits of the sodium reformulation targets in reducing disparities, consistent with previous evidence demonstrating that upstream population interventions are more equitable than downstream, individual-focused strategies [55].

Our findings are consistent with previous analyses quantifying potential benefits of general population reduction of sodium consumption [10,56–59] and build upon them significantly. A previous modeling study estimated 194,000 to 392,000 QALYS gained annually in the US with a reduction in salt consumption of 3 g/day [10], whilst another simulation study found that 312,000 QALYs could be generated annually by reducing sodium consumption to the recommended upper bound of 2,300 mg/day [57]. These previous findings are reassuringly consistent with our study, which projected an average 305,000 QALYs generated each year of the study. However, our analysis includes several notable advances. For example, we evaluated and incorporated background trends in sodium intake, SBP, and CVD, which reduces the estimated potential benefits of our policy scenarios, providing the most conservative estimation of benefits. In addition, we specifically modeled the 2016 FDA proposal, providing direct relevance to current policy considerations in the US. Furthermore, we evaluated cost-effectiveness from distinct relevant perspectives, including societal and healthcare perspectives. We are thus able to provide policy makers and health advocacy groups with more accurate and timely real-world estimations of the likely effects of the proposed policy, and the foregone opportunity costs if the desired reformulations are not achieved. By including industry costs, the present study aimed to include all relevant costs and provide objective results for all stakeholders.

This study has potential limitations. The effect estimates in the model are based on interventional and prospective observational studies. They are therefore subject to biases and confounding that may have influenced our model estimates. However, the etiological effects of dietary changes were estimated from meta-analyses with confirmatory validity analyses, including from randomized clinical trials. Our estimates may be conservative and underestimate the full health and economic benefits of sodium reformulation, as (1) our baseline scenario assumed that recent observed declines in sodium intake would continue into the future, moderating the benefit of all reformulation scenarios; (2) we only evaluated diseases mediated through BP, while decreasing sodium consumption could have beneficial effects upon other health burdens such as gastric cancer [60,61]; and (3) reductions in sodium consumption achieved through the proposed policy might additionally increase potassium intake though substitution of NaCl with KCl [62], but we did not include this potential beneficial effect in our model. We did not include unrelated medical costs in the main analysis or a sensitivity analysis as this study focused only on costs for CVD. Our modeling results cannot replace evidence from evaluating the actual policy intervention over time in the US, indicating that any implementation of the FDA policy should be accompanied by robust independent assessment.

In conclusion, our findings suggest that the proposed FDA voluntary sodium reformulation targets could result in substantial health benefits and cost savings across the US population. However, suboptimal compliance or a delay in reaching these targets could result in a significant number of preventable CVD cases, CVD deaths, and costs to the wider economy.

## Supporting information

S1 Animation Model animation. (AVI)

S1 Appendix Supplementary technical appendix. (DOCX)

S1 Table Distributions that were used as inputs for the simulations. (PDF)

## Abbreviations

BP: blood pressure
CHD: coronary heart disease
CVD: cardiovascular disease
FDA: US Food and Drug Administration
MEPS: Medical Expenditure Panel Survey
NHANES: National Health and Nutrition Examination Survey
QALY: quality-adjusted life year
SBP: systolic blood pressure
UI: uncertainty interval
WHO: World Health Organization
