# Supplementary material for: Estimating the health and economic effects of the proposed US Food and Drug Administration voluntary sodium reformulation: Microsimulation cost-effectiveness analysis
Source: PLoS Med. 2018 Apr 10;15(4):e1002551. doi: 10.1371/journal.pmed.1002551 (PMC5892867; doi:10.1371/journal.pmed.1002551)
Supplement: S1 Appendix — (DOCX) [file pmed.1002551.s002.docx]

**Supplementary Technical Appendix**

This appendix has been provided by the authors to give readers additional information about their work.

Supplement to: Estimating the health and economic effects of the proposed US FDA voluntary sodium reformulation.

# Table of Contents

Table of Contents 2

List of Tables 3

List of Figures 5

Summary of evidence about the risks of excess sodium consumption 6

High-level description of the US Sodium Policy model 7

Technical information 7

Population module 9

Estimating exposure to risk factors 9

Generating the ‘close to reality’ synthetic population for the US Sodium Policy model 9

Implementation of individualized risk factor trajectories 10

Demographic and socioeconomic variables 10

Continuous variables 11

Disease module 13

Estimating the annual individualized disease risk and incidence 13

Step 1 13

Step 2 14

Step 3 14

Estimating disease incidence at initial simulation year 14

Estimating disease prevalence at initial simulation year 15

Simulating mortality 15

Health economics module 17

Health state utilities 17

Disease costs 17

Policy costs 18

Policy module 20

Modeling the proposed US FDA voluntary sodium reformulation 20

Step 1 20

Step 2 21

Step 3 21

Evidence supporting the choice of baseline scenario 22

Uncertainty and sensitivity analysis 24

Input uncertainty 25

Outputs 26

One-way sensitivity analysis 27

Extra scenario 28

Calibration and Validation 29

Synthetic population internal validation 31

Tables 35

Additional result from main analysis 52

Model estimates for the baseline scenario 63

Results from the extra scenario (not comparable to main results) 65

References 67

# List of Tables

Table A The US Sodium Policy model data sources. 35

Table B Key modeling assumptions and limitations. 40

Table C Mismatches between the proposed FDA reformulation policy and the FNDDS v5 database that were handled manually. With the exception of imitation cheese (1^st^ row) all other foods that were manually assigned to a relevant FDA food category were not eligible for reformulation. 41

Table D Distributions that were used as inputs for the simulations. Numbers are rounded to second decimal. 48

Table E One-way sensitivity analysis based on different discount rates and willingness to pay for 1 QALY. Results are incremental cost-effectiveness ratio (2017 US Dollars per QALY) over the 20-year simulation period from 2017 to 2036, for US adults age 30 to 84 years. Values are the median estimate (95% UI). Rounded to the second significant digit. 50

Table F One-way sensitivity analysis based on different discount rates and willingness to pay for 1 QALY. Results are net monetary benefit over the 20-year simulation period from 2017 to 2036, of US adults age 30 to 84 years. Values are the median estimate (95% UI). Rounded to the second significant digit. Costs are presented in (discounted) 2017 Billion US Dollars. 51

Table G Health related model estimates, impact Inventory and cost-effectiveness analysis over the 20-year simulation period from 2017 to 2036, for US adults age 30 to 84 years by sex. Values are the median estimate (95% UI). Results are rounded to the first digit for sodium, first decimal for SBP, and second significant digit for other outcomes. Negative costs represent savings. Policy costs were divided proportionally to population size for each sex. Costs are presented in discounted 2017 Billion US Dollars. CHD, coronary heart disease; CVD, cardiovascular disease; QALY, quality adjusted life years; SBP, systolic blood pressure; UI, uncertainty intervals. 52

Table H Health related model estimates, impact Inventory and cost-effectiveness analysis over the 20-year simulation period from 2017 to 2036, for US adults age 30 to 84 years by age group. Values are the median estimate (95% UI). Results are rounded to the first digit for sodium, first decimal for SBP, and second significant digit for other outcomes. Negative costs represent savings. Policy costs were divided proportionally to population size for each age group. Costs are presented in discounted 2017 Billion US Dollars. CHD, coronary heart disease; CVD, cardiovascular disease; QALY, quality adjusted life years; SBP, systolic blood pressure; UI, uncertainty intervals. 55

Table I Health related model estimates, impact Inventory and cost-effectiveness analysis over the 20-year simulation period from 2017 to 2036, for US adults age 30 to 84 years by race/ethnicity (black for non-Hispanic black and white for non-Hispanic white). Values are the median estimate (95% UI). Results are rounded to the first digit for sodium, first decimal for SBP, and second significant digit for other outcomes. Negative costs represent savings. Policy costs were divided proportionally to population size for each race/ethnicity. Costs are presented in discounted 2017 Billion US Dollars. CHD, coronary heart disease; CVD, cardiovascular disease; QALY, quality adjusted life years; SBP, systolic blood pressure; UI, uncertainty intervals. 59

Table J Model estimates for the baseline scenario over the 20-year simulation period from 2017 to 2036, for US adults age 30 to 84 years. Values are the median estimate (95% UI). Results are rounded to the first digit for sodium, first decimal for SBP, and third significant digit for other outcomes. Costs are presented in discounted (3%) 2017 Billion US Dollars. CHD, coronary heart disease; CVD, cardiovascular disease (the sum of CHD and stroke cases, avoiding double counting of cases with coexisting CHD and stroke); QALY, quality adjusted life years; SBP, systolic blood pressure; UI, uncertainty intervals. 63

Table K Health related model estimates, impact Inventory and cost-effectiveness analysis for the 7.5% reformulation scenario over the 20-year simulation period from 2017 to 2036, for US adults age 30 to 84 years. Values are the median estimate (95% UI). Results are rounded to the first digit for sodium, first decimal for SBP, and second significant digit for other outcomes. Negative costs represent savings. Costs are presented in discounted 2017 Billion US Dollars. CHD, coronary heart disease; CVD, cardiovascular disease; QALY, quality adjusted life years; SBP, systolic blood pressure; UI, uncertainty intervals. 65

# List of Figures

Figure A Model logic. The allow between reduction in systolic blood pressure and reduction in all-cause mortality does not imply a causal effect of systolic blood pressure on every mortality cause. 8

Figure B Plot of the percentile rank against the systolic blood pressure of non-Hispanic black male synthetic individuals for age groups 30–34 and 60–64. 12

Figure C Mean sodium concentration across all foods recorded in the FNDDS database over time. 22

Figure D Observed and forecasted coronary heart disease mortality. US population aged 30 to 84. Shaded areas represent 95% prediction intervals. Source for observed mortality CDC WONDER database.^37^ 29

Figure E Observed and forecasted stroke mortality. US population aged 30 to 84. Shaded areas represent 95% prediction intervals. Source for observed mortality CDC WONDER database.^37^ 30

Figure F Observed and forecasted mortality from any-other-cause (excluding coronary heart disease and strokes). US population aged 30 to 84. Shaded areas represent 95% prediction intervals. Source for observed mortality CDC WONDER database.^37^ 30

Figure G Mosaic plot for comparison of age, sex, and race/ethnicity distribution between the synthetic population and the NHANES1114 sample. Green shades depict relative differences close to 0%, and all subgroups were within a ±5% difference compared to the NHANES sample. For Race/ethnicity: b, non-Hispanic black; o, other; w, non-Hispanic white. 32

Figure H Empirical cumulative distributions of sodium consumption in NHANES1114 (sample) and the synthetic population (population), by age group, sex, and race/ethnicity. 33

Figure I Empirical cumulative distributions of systolic blood pressure in NHANES1114 (sample) and the synthetic population (population), by age group, sex, and race/ethnicity. 34

# Summary of evidence about the risks of excess sodium consumption

Excess dietary sodium consumption has been linked to an increased risk of cardiovascular disease (CVD).^1^ For CVD, the excess risk appears to be mainly mediated through the deleterious effect of excess sodium consumption on blood pressure.^2,3^ Our methods for evaluating the causality of effects of sodium reduction on BP and of BP reduction on CVD have been previously described.^3 Text S1-S3^

The World Health Organization (WHO) and the United States (US) national guidelines recommend a daily sodium intake of less than 2,000 mg/d and 2,300 mg/d, respectively, after assessing the totality of evidence.^4,5^ There is some controversy regarding the optimal level of sodium consumption.^6^ Some researchers claim that sodium consumption lower than 3,000 mg/d can actually increase the risk of CVD and overall mortality.^7,8^ However, it appears that this argument is based on biased measurement methodology.^9,10^ A recent discussion on the subject can be found in Mozaffarian *et al*. who concluded that the optimal level of sodium consumption below which no health gains have been observed is somewhere in the range of 614 mg/d to 2391 mg/d.^3 text S4^ In our study we have incorporated the uncertainty around the ideal sodium consumption in our probabilistic sensitivity analysis.

Evidence that directly links sodium risk reversibility to CVD mortality or morbidity outcomes is lacking. A meta-analysis of several randomized control trials that tested low sodium diets was underpowered and therefore inconclusive.^11^ In comparison, a plethora of evidence exists supporting the effect of low sodium diet on blood pressure which appears to happen within weeks.^2,3,12^ Finally, the cardiovascular risk reversibility of blood pressure has been evident in several randomized control trials and appears to occur within a 5-year period.^13^

# High-level description of the US Sodium Policy model

The US Sodium Policy model^[[1]](#footnote-2)^ is a discrete time dynamic stochastic microsimulation model.^14,15^ Within the US Sodium Policy model each unit is a synthetic individual and is represented by a record containing a unique identifier and a set of associated attributes.

For this study, we considered age, sex, race/ethnicity^^[[2]](#footnote-3)^^, education^[[3]](#footnote-4)^, income^[[4]](#footnote-5)^, sodium consumption, and systolic blood pressure (SBP). A set of stochastic rules is then applied to these individuals, such as the probability of developing coronary heart disease (CHD) or dying, as the simulation advances in discrete annual steps. The output is an estimate of the burden of CHD and stroke, in the synthetic population including both total aggregate change and, more importantly, the distributional nature of the change.

The US Sodium Policy model is a complex model that simulates the life course of synthetic individuals and consists of four modules: The ‘population’ module, the ‘disease’ module, the ‘health economics’ module, and the ‘policy’ module. We will fully describe the US Sodium Policy model by describing the processes in each of the modules in the following chapters. The description is from an epidemiological rather than technical perspective. Figure A depicts the logic of the model. Table A and Table B summarize the sources of the input parameters and the main assumptions and limitations, respectively.

**Technical information**

The US Sodium Policy model is being developed in R v3.4.0^16^ and is currently deployed in a 40-core workstation with 192Gb of RAM running Ubuntu v16.4 server edition. The US Sodium Policy model is built around the R package ‘data.table’^17^, which imports a new heavily optimized data structure in R. Most functions that operate on a data table have been coded in C to improve performance. Each iteration for each scenario is running independently in one of the CPU cores, and the R package ‘foreach’^18^ is responsible for the distribution of the jobs and collection of the results. To ensure statistical independence of the pseudo-random number generators running in parallel, the R package ‘doRNG’^19^ was used to produce independent random streams of numbers, generated by L'Ecuyer's combined multiple-recursive generator.^20^

Reduction in stroke incidence

Reduction in coronary heart disease mortality

Reduction in sodium consumption

Reduction in all-cause mortality

Reduction in systolic blood pressure

Processed food sodium reformulation

Reduction in coronary heart disease incidence

Reduction in stroke mortality

Figure S 1 Model logic. The allow between reduction in systolic blood pressure and reduction in all-cause mortality does not imply a causal effect of systolic blood pressure on every mortality cause.

# Population module

Synthetic individuals enter the simulation in the initial year (2014 for this study). The number of synthetic individuals that enter the simulation is user defined and for this study was set to 100,000. The algorithm ensures that the age, sex, and race/ethnicity distribution of the sample is as this of the US population in mid-2014. The exposures to sodium and SBP are being calculated annually (in simulation time) for each synthetic individual until the simulation horizon is reached, or death occurs.

## Estimating exposure to risk factors

The US Sodium Policy model estimates the exposure of the synthetic individual to the modeled risk factors. It is essential the risk profile of each synthetic individual to be similar to the risk profiles that can be observed in the real US population. For this, we first built a ‘close to reality’ synthetic population of US from which we sampled the synthetic individuals. Then, we used generalized linear models (GLM) for sodium consumption and SBP, to simulate individualized risk factor trajectories for all synthetic individuals.

## Generating the ‘close to reality’ synthetic population for the US Sodium Policy model

The ‘close to reality’ synthetic population ensures that the sample of synthetic individuals for the simulation is drawn from a synthetic population similar to the real one in terms of age, sex, race/ethnicity, and risk factors conditional distributions. In our implementation, we used the same statistical framework originally developed by Alfons *et al*.,^21^ and we adapted it to make it compatible with epidemiological principles and frameworks.^22^

In general, this method uses a nationally representative survey of the real population to generate a ‘close to reality’ synthetic population. Therefore, the method expands the, often small, sample of the survey into a significantly larger synthetic population, while preserves the statistical properties and important correlations of the original survey.

The main advantages over other approaches are: 1) it accounts for the hierarchical structure of the sample design of the original survey, and 2) it can generate trait combinations which were not present in the original survey but are likely to exist in the real population. The second is particularly important because it avoids bias from the excessive repetition of combinations of traits present in the original survey that results from multilevel stratification of a relatively small sample. For example, the original survey may have two 35-year-old male participants, one with SBP of 135 mmHg and the other with an SBP of 140 mmHg and no other 35-year-old male participants with SBP between 135 mmHg and 140 mmHg. Unlike other methodologies, the approach proposed by Alfons *et al.* can produce 35-year-old male synthetic individuals with an SBP between 135 mmHg and 140 mmHg. This is possible because the synthetic population is produced by drawing from conditional distributions that were estimated from multinomial models fitted in the original survey data. The detailed statistical framework and justification can be found elsewhere.^21^

All the variables of the synthetic population for this study were informed by the National Health and Nutrition Examination Survey 2011–2014 (NHANES1114).^23^ The R language for statistical computing v3.4.0 and the R package ‘simPop’ v0.6.0 were used to implement the method.^24,25^ For this study we first generated the demographic variables of the synthetic individuals (age, sex, race/ethnicity). Then, conditional on the demographic variables we generated the educational level variable. We generated income level conditional on the demographic and educational level variables. Finally, we generated sodium^[[5]](#footnote-6)^ consumption on all previously generated variables and SBP conditional on sodium consumption and the demographic variables.

The outcome of the method was to create a synthetic population of 50 million with similar characteristics to the non-institutionalized US population in 2011–2014. We validated the synthetic population against the original NHANES1114 sample (internal validation). We present the validation results below, starting from page 31.

## Implementation of individualized risk factor trajectories

the US Sodium Policy model only applies the previous process for the initial year of the simulation (2014 for this study). As the simulation evolves over time, sodium consumption and SBP are recalculated to take into account age and period effects. This feature justifies the classification of the US Sodium Policy model as a dynamic microsimulation. Generally, it uses the continuous NHANES series to capture the time trends by age, sex, and race/ethnicity and project them into the future.

### Demographic and socioeconomic variables

As the simulation progress in annual circles, the age of the synthetic individuals in the model increase by one year in each loop. Their sex and socioeconomic variables remain stable. Therefore, social mobility is not simulated in the current version of the US Sodium Policy model. Every simulated year, a new cohort of 30-year old synthetic individuals enter the simulation. The size of the cohort and the sex and race/ethnicity distribution of the synthetic individuals are informed by the published US population projections.^27^ The educational and income variables are directly standardized based on the initial 30-year old cohort and conditional on sex and race/ethnicity.

### Continuous variables

In the US Sodium Policy model**_,_** the value of each continuous risk factor (sodium, SBP) is calculated in a two-step process for each synthetic individual and each projected year. The first step simulates aging effects, while the second step simulates period effects. We follow this approach mainly for two reasons. Firstly, to simulate physiological mechanisms of aging. For example, the increase of SBP due to age-related stiffening of the arteries. Secondly, because the variance of the risk factor distributions increases with age, and we wanted to model this. Below we describe the steps:

**Step 1:** Instead of tracking the actual continuous risk factor values for the synthetic individuals, we track the percentile ranks^^[[6]](#footnote-7)^^ of the values by age, sex and race/ethnicity. These percentile ranks remain fixed for each synthetic individual throughout the simulation. In each simulated year, the percentile ranks are converted back to actual risk factor values, by matching the percentile ranks of a sample of the initial synthetic population of same age group, sex, and race/ethnicity.

For example, in 2014 a 30-year-old non-Hispanic black male synthetic individual with SBP of 120 mmHg has an SBP percentile rank of 0.52. Thirty years later, the same synthetic individual has retained his percentile score for SBP. However, his SBP is now calculated to 137.6 mmHg to match the SBP of a 60-year old non-Hispanic black man in 2014 with the same percentile rank of 0.52. Figure B illustrates the previous example. Although individuals retain their percentile for the respective risk factor throughout the simulation (vertical position in Figure B), this step remains stochastic because each time this step is implemented a different sample from the synthetic population is drawn. Finally, the distance from the mean for each risk factor is calculated stratified by 5-year age group, sex, and race/ethnicity. For instance, if a synthetic individual has SBP of 140 mmHg and the mean SBP in the respective group of same age group, sex and race/ethnicity is 130 mmHg, the distance from the mean is 140 – 130 = 10 mmHg.

**Step 2:** We fitted regression models to the continuous NHANES data. For sodium, we used NHANES0914^[[7]](#footnote-8)^, and we fitted a GLM with sodium as the dependent variable and year, age, sex, and race/ethnicity as independent variables (including significant quadratic effects and 1^st^ order interactions based on Akaike’s information criterion (AIC)). For SBP we followed a similar approach, but we used the full range of continuous NHANES9914. For both models, we used a logarithmic link function; therefore, we assumed logarithmic declining time trends for both sodium and SBP. These models are used to predict the mean of the relevant group. These predicted means are added then, to the distances calculated in the previous step. The result is the final value of the relevant risk factor that will be used for risk estimation.

**Lag times**


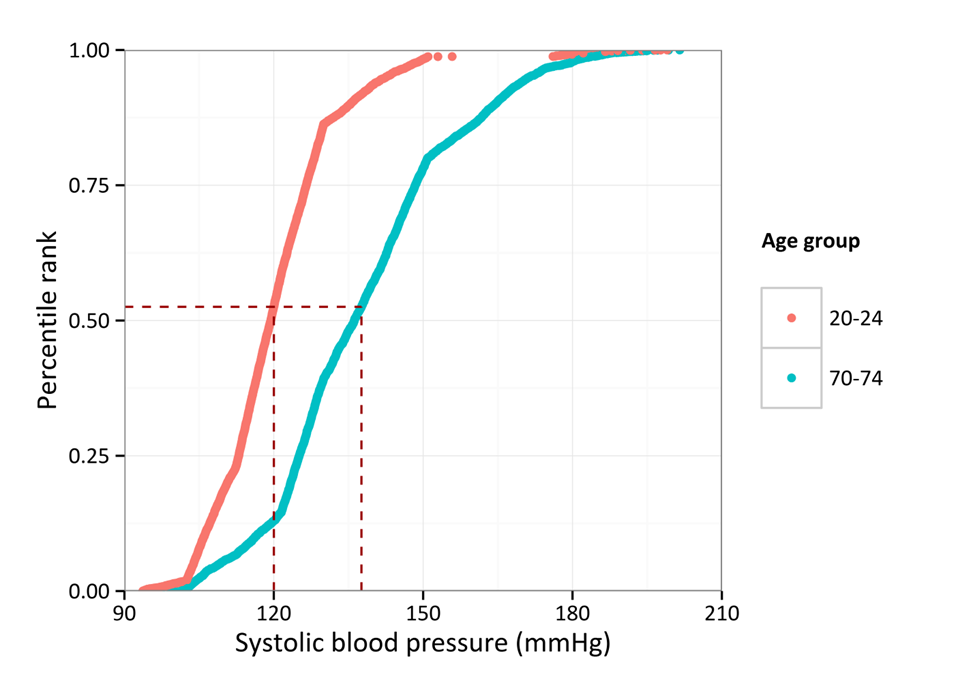


Figure S 2 Plot of the percentile rank against the systolic blood pressure of non-Hispanic black male synthetic individuals for age groups 30–34 and 60–64.

**30–34**

**60–64**

All the functions that have been described above for risk factor trajectories include time and age (in years) as one of the independent variables. Therefore, lag times can be potentially calculated on a per risk factor basis. When the ‘disease’ module of the US Sodium Policy model, uses the exposure to SBP to estimate the risk of a synthetic individual to develop CVD in a specific simulated year, the lag-timed exposure is used. In this study, we assumed that the mean lag time between exposure to high SBP and CVD is 5 years.^13,28,29^ Mean lag times were roughly informed from risk reversibility trials and the median observation times of the cohort studies we used to inform the risk magnitude for SBP. We assumed no lag time between a change in sodium intake and impact on SBP, as this happens within few weeks.^2^

**Disease module**

The risk (probability) for each synthetic individual aged 30–84, to develop each of the modeled diseases is estimated conditional on previous exposure to SBP, age, sex, and race/ethnicity. For every simulated year, the model selects synthetic individuals to develop CHD and/or stroke based on their risk. Finally, the risk of dying from one of the modeled diseases or any other cause is estimated and applied.

**Estimating the annual individualized disease risk and incidence**

To estimate the individualized annual probability of a synthetic individual to develop a specific disease conditional on his/her relevant risk exposures we follow a 3-step approach. Below we describe the general approach that is used for simulations with more than one risk factors. For this study, only one risk factor was included (high SBP). Next, the implementation of the method is described in more detail using CHD as an example. The same process is used for both CHD and stroke.

**Step 1**

The population attributable risk (PAF) is an epidemiological measure that estimates the proportion of the disease attributable to an associated risk factor.^30^ It depends on the relative risk associated with the risk factor and the prevalence of the risk factor in the population. In a microsimulation context where exposure to risk factors are known to the individual level and assuming multiplicative risk factors PAF can be calculated with the formula:

$$PAF= 1-\frac{n}{\sum_{i=1}^{n} (RR_{1}* RR_{2}*\ldots* RR_{k})} ,$$

where $n$ is the number of synthetic individuals in the population, and ${RR}_{1\ldots k}$ is the relative risks of the risk factors associated with CHD. We calculated PAF based on above formula stratified by age, sex, and race/ethnicity only in the initial year of the simulation. Consistent with findings from the respective meta-analyses that were used for the US Sodium Policy model (Table A), SBP below 110 mmHg, was considered to have a relative risk of 1. All the relative risks were taken from published meta-analyses (Table A).

**Step 2**

The incidence of CHD not attributable to the modeled risk factors can be estimated by the formula:

$$I_{Not attributable}= I_{Observed}*\left( 1-PAF \right)$$

Where $I_{Observed}$ is the CHD incidence and $PAF$ is from Step 1. $I_{Not attributable}$ represents CHD incidence if all the modeled risk factors were at optimal levels. The not attributable incidence is calculated by year, age, sex, and race/ethnicity.

To account for future time trend in CHD incidence that is not attributable to the modeled risk factors (in this study SBP), the model updates $I_{Observed}$ every simulated year. For this we assume that half of the forecasted annual change in CHD mortality is attributed to changes in CHD incidence and the other half to changes in CHD case fatality. We based this assumption on observational evidence from England, and modeling studies in the England and the US.^31–34^ Furthermore, we included this assumption in our probabilistic sensitivity analysis (page 24).

**Step 3**

Assuming that $I_{Not attributable}$ is the baseline annual probability of a synthetic individual to develop CHD for a given age, sex, and race/ethnicity due to risk factors not included in the model, the individualized annual probability to develop CHD, $\mathbb{P}\left( \text{CHD | age, sex, race/ethnicity}\text{,}\text{ }\text{exposures} \right)$, given his/her risk factors were estimated by the formula:

$$\mathbb{P}\left( CHD | age,sex\text{, race/ethnicity},exposures \right)= I_{Not attributable}*RR_{1}*RR_{2}*RR_{3}*\ldots*RR_{k}$$

Where $RR_{1 \ldots k}$ the relative risks that are related to the specific risk exposures of the synthetic individual, same as in step 1.

## Estimating disease incidence at initial simulation year

It is obvious that for the method above disease incidence ($I_{Observed}$) in the population, need to be known, at least for the initial year of the simulation. However, the true incidence of CHD (and stroke) in the US, is largely unknown. Several estimates exist nonetheless all have limitations, and the same applies to incidence trends.^35,36^ Therefore, for the estimation of CHD and stroke incidence by age, sex, and race/ethnicity we opted for a modeling solution to synthesize all the available nationally representative sources of information and to minimize bias. Specifically, we used CHD mortality (ICD10 I20–I25) for US in 2014,^37^ self-reported prevalence of CHD from NHANES1314,^23^ and the 1-year risk of CHD for the NHANES1314 participants using the Framingham equation^38^ to inform the WHO DisMod II model.^39^ DisMod II is a multi-state life table model that can estimate the incidence, prevalence, mortality, case fatality and remission of a disease when information about at least three of these variables is available. A similar approach has been followed by the Global Burden of Disease team and others.^40,41^ We considered CHD an incurable chronic disease (i.e. remission rate was set to 0); therefore, the derived DisMod II incidence refers to the first ever episode of CHD excluding any recurrent episodes. For the DisMod II calculations, we assumed that incidence and case-fatality rates had been declining by 2% (relative), over the last 20 years. We used the derived CHD incidence rates by age, sex, and race/ethnicity to inform the US Sodium Policy model. We used the same approach for stroke.

## Estimating disease prevalence at initial simulation year

For the initial year of the simulation, some synthetic individuals need to be allocated as prevalent cases for each of the modeled diseases. We used DisMod II model estimates for prevalence of CHD and stroke by age, sex, and race/ethnicity. At the beginning of each simulation, the estimated number of prevalent cases are sampled independently from the synthetic individuals in the population with weights proportional to their SBP exposures.

## Simulating mortality

All synthetic individuals are exposed to the risk of dying from any of their acquired modeled diseases or any other non-modeled cause in a competing risk framework. The US Sodium Policy model is calibrated to observed CHD, stroke, and any-other-cause mortality for years 2014–2015^37^ and mortality forecasts for years 2016–2036. For years after 2015, coherent functional demographic models by sex and race/ethnicity were fitted to the reported CHD, stroke, and any-other-cause mortality rates from years 1999 to 2015,^37^ and then were projected to the simulation horizon using the R package ‘demography’.^42^ Functional demographic models are generalizations of the Lee-Carter demographic model, influenced by ideas from functional data analysis and non-parametric smoothing.^43^ The coherent approach ensures that subgroup forecasts do not diverge over time.^44^ Finally, we used the observed and forecasted mortality rates to create life tables for each simulated year, by age, sex, race/ethnicity, and disease (CHD, stroke, any-other-cause). We applied the any-other-cause life tables to all synthetic individuals, and the CHD and stroke life tables to prevalent cases of CHD and stroke only, respectively. For the synthetic individual that died of more than one causes in a specific year, a cause was randomly selected to minimize bias.

In reality, hypertensive individuals have a higher risk to die not only of CHD and stroke but from a spectrum of other diseases also. Failure to model this would result in biased estimates in the health economics module (described on page 17) because it would inflate the costs and disutility from hypertension, inappropriately. To account for this and minimize bias the US Sodium Policy model inflates the any-other-cause mortality rates for hypertensive synthetic individuals in the model (Figure A link between SBP and all-cause mortality) while it deflates it for non-hypertensives. The algorithm ensures the total number of hypertensive and non-hypertensive synthetic individuals that die every year from any-other-cause is equal to the defined one in the life table. The algorithm is based on PAF approach, and the relative risk was derived from an individual level meta-analysis by Stringhini *et al*.^45^ In this meta-analysis the relative risk of all-cause mortality for hypertensives was 1.31 (1.24–1.38), and the relative risk of non-CVD-non-cancer mortality was 1.29 (1.21–1.38). Hence, we used a relative risk of 1.3 in the US Sodium Policy model.

# Health economics module

In the previous two modules, the US Sodium Policy model creates synthetic individuals with traits similar to those observed in the US population and tracks their future exposures to sodium and SBP, and important events (first manifestation of CHD and stroke, death from CHD, stroke, or any other cause).

## Health state utilities

We calculated the health state utility values (preference weights) using published censored least absolute deviations regression equations which used EQ-5D-3L data from the Medical Expenditure Panel Survey (MEPS) 2000-2002 for all major chronic conditions in the USA, including CHD, stroke, and hypertension.^46^ The equation uses the main condition, number of coexisting chronic conditions, age, sex, race, ethnicity, income, and education to estimate the health state utilities of the synthetic individuals every simulated year. We found that the most influential parameter in this equation was the number of coexisting chronic conditions, far exceeding its sampling error. Hence, we ignored the sampling error of this equation, and we calibrated the distribution of the number of coexisting chronic conditions in the synthetic population to the distribution reported by Sullivan *et al*.^46^ We further modeled the number of coexisting chronic conditions to increase with age.

## Disease costs

The US Sodium Policy model applies CHD, stroke, and hypertension costs to cases of these diseases, during the simulation. These costs are mean estimates by age, sex, and race/ethnicity.

Disease costs per person-year were derived from a report of projections of CVD costs, prepared for the American Heart Association (AHA) by RTI International which was based on MEPS data.^47^ The AHA report assumed that price increases and new technologies would produce a 2.45% increase in medical costs, above the impact of inflation, demographic change, and disease severity. We assumed an equal annual increase of medical costs. Medical costs per person-year for CHD, stroke, and hypertension were calculated by dividing total medical costs by the number of people with each condition in 2015 and disaggregated by the ratio of the point of service (physician, hospital, prescription, home health, nursing home, and other). The AHA paper included the ratio of medical costs at the point of service for each disease group; the point of service was grouped into physician, hospital, prescription, home health, nursing home, and other.

Productivity costs of morbidity and mortality for CHD and stroke, and hypertension (including workplace productivity and leisure time) were from the same analysis by RTI International and were converted to costs per person-year. For CHD and stroke, we applied morbidity costs to prevalent cases of CHD and stroke, respectively; we applied mortality costs only to deaths from CHD and stroke. For hypertension, we used the productivity costs not decomposing them into mortality and morbidity costs, because the US Sodium Policy model does not track deaths attributed to hypertension. We assumed that productivity costs would increase by 1.29%, annually.

Informal care costs for stroke were from a study by Joo *et al*.^48^, while informal care costs for CHD were based on the ratio of healthcare to informal care costs in Europe from a study by Leal *et al*.^49^ We assumed no informal care costs for hypertension alone as we assumed that most would be mediated through CHD and stroke.

## Policy costs

The policy costs included:

*Government costs to administer and monitor the policy.* For administrative costs, because there has been no previous initiative that is both national in scope and precisely about sodium reduction, we used data from two existing sources. First, we acquired cost data from the National Sodium Reduction Initiative (NSRI), led by New York City’s Health Department.^50^ ^[[8]](#footnote-9)^ Second, we acquired cost data from a different FDA policy, new restaurant menu and vending machine labeling regulation, including the cost of outreach, education, review of regulatory issues, developing training for inspectors, and related functions.^51^ We used the second data source in the analysis because it generated more conservative (higher-cost) estimates. Monitoring and evaluation cost was obtained through UK FSA’s impact assessment and converted to equivalent US dollars^52^. Administrative costs were assumed to occur every year, and monitoring and evaluation costs were assumed to occur every year after full policy implemented in year 3.

*Industry costs to reformulate products.* Industry costs were calculated using a reformulation cost model developed by the Research Triangle Institute under contract with the FDA.^53^ The model accounted for variations in product formula complexity, company size, reformulation type, compliance period and other factors, which produces a more accurate cost estimate compared to a standard per-product cost approach. We calculated the cost of two rounds of reformulation, which corresponded to the FDA’s short term and long-term sodium reduction goals. We assumed the industry cost was equal in the two rounds of reformulation, and divided the costs over the policy implementation years (intervention years 1–3 for the first round, and intervention years 4–10 for the second round). We assumed no policy costs after intervention year 10. All costs were inflated to 2017 dollars and discounted at a 3% rate.

# Policy module

So far, the description of the US Sodium Policy model was for the baseline scenario. The policy module translates the policy scenarios to be modeled by the US Sodium Policy model. Figure A depicts the logic of the model. Changes in sodium consumption are translated into changes in SBP using the meta-regression equation by Mozaffarian *et al*.^3^ by age, race, and hypertensive status.^[[9]](#footnote-10)^ The new SBP is used in the disease module, and updated CHD and stroke risks are calculated for every synthetic individual, with new outcomes. Therefore, new life courses for all synthetic individuals are simulated as a result of the modeled policies. When the simulation ends, the model compares all alternative life courses with the baseline one for each synthetic individual and calculates the outputs (page 26).

## Modeling the proposed US FDA voluntary sodium reformulation

The US Food and Drug Administration (FDA) in 2016 proposed short-term (2 years) and long-term (10 years) voluntary, category-specific sodium reformulation targets for commercially processed, packaged, and prepared foods. This proposal was designed to support the 2015–2020 US Dietary Guidelines by encouraging food reformulation and new product development.^54^ The proposal includes a detailed table with 155 (excluding baby/toddler foods) food categories of commercially processed, packaged, and prepared foods with the baseline (2010) sales-weighted mean sodium concentration the short and long-term target concentrations, and their upper bounds. For example, the ‘Blue/Blue-Veined Cheese (Semi-soft)’ food category has a baseline sales-weighted mean sodium concentration of 1240mg/100g; short-term target 1180mg/100g; short-term upper bound target 1430mg/100g; long-term target 1050mg/100g; and long-term upper bound target 1340mg/100g;

Separately, FDA also published instruction to link the food categories in the proposal, with the food codes that were used in the 24h recall questionnaires for NHANES0710.^55^ Unfortunately the linkage was incompatible with more recent NHANES data that we used to prime the synthetic population. Therefore, to model the effect of the proposed policy to the modeled population we developed the algorithm below:

### Step 1

We use NHANES0910, and for every participant, we access the 24h recall food questionnaire. The questionnaire contains the amount, food type, and sodium concentration that the participant recalls that consumed the previous day. The FNDDS v5 database was used for the coding of foods.^56^ We link these foods with the food categories from the proposed FDA policy. Therefore, we can select which foods in the questionnaires are eligible for reformulation (i.e. their sodium concentration is higher than the proposed FDA targets).^[[10]](#footnote-11)^

### Step 2

Based on the specific scenario assumptions (i.e. 50% compliance with the proposed upper bound of the 2- and 10-year targets and 50% compliance with the main 2- and 10-year targets for the modest scenario) we randomly select eligible foods to be reformulated from the NHANES food questionnaires. We calculate the expected reduction of sodium intake for each NHANES0910 participant, in absolute and relative terms. For this study, we assumed a gradual linear diffusion of the reformulation effect to the population. Once the maximum policy effect was reached, we assumed it would sustain for the rest of the simulation period.

### Step 3

We stochastically match each synthetic individual of the US Sodium Policy model with an NHANES0910 participant based on their age (10-year age group), sex, race/ethnicity, and sodium consumption^[[11]](#footnote-12)^. Then we use the same method described in page 11 to back-project sodium consumption to the year 2010 for all synthetic individuals. Finally, we apply the expected relative sodium reduction of the matched NHANES participant, to the 2010 sodium consumption of the synthetic individuals and we subtract it from the baseline sodium consumption to estimate the net effect of the policy. This net policy effect on every synthetic individual, expressed in a change in sodium consumption every year, is transformed to SBP changes as it was described above. The underlying assumption in this step is that the food composition of US diet has been and will be similar to the one in 2010.

This approach bypasses the incompatibility of the linkage between the FDA proposed policy and more recent NHANES data. It also allows the incorporation of sodium consumption time trends in the calculations and provides enough granularity of the policy effect (by age, sex, race/ethnicity, and sodium consumption) without being too computationally intensive. Yet, it does not address potential behavioral changes of the population as a result of the reformulation and ignores foods prepared in food outlets and restaurant.

Finally, the linkage between the FDA proposed policy and the FNDDS v5 database was not perfect. Of the 4998 different foods that were linked to 155 food categories in the proposed FDA policy, 132 (2.6%) were not a perfect match, and we manually assigned them to one of the 155 food categories. Table C presents these foods. With the notable exception of imitation cheese and feta cheese in the first row of the table, all other foods contain substantially less sodium compared to products in their food category and were not eligible for reformulation. Recipe conversions and combination codes in the NHANES data were not included in the linkage.

## Evidence supporting the choice of baseline scenario

Sodium consumption in NHANES remained stable between 1999 and 2008. Since 2009, an emerging time trend appeared in the data, and mean sodium consumption adjusted for age, sex, and race/ethnicity appears to decrease by almost 18mg per year. Interestingly, the mean sodium concentration of all the foods that were recorded into the FNDDS database, which gets updated every two years, appears to decline since 2009, also (Figure C). Finally, a longitudinal study between 2000 and 2014 using the Nielsen Homescan Consumer Panel data suggests that sodium per capita and food sodium content has been reduced over the period study.^57^


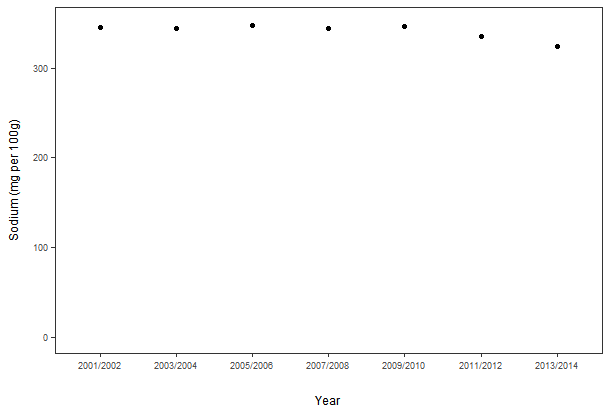
Figure C Mean sodium concentration across all foods recorded in the FNDDS database over time.

Based on the evidence presented above we decided to allow a slowly declining trend in future sodium consumption projections. If the emerging sodium time trend does not continue in the future, will render our results conservative.

# Uncertainty and sensitivity analysis

The US Sodium Policy model implements a 2^nd^ order Monte Carlo approach to estimate uncertainty intervals (UI) for each scenario.^58,59^ Each simulation, which includes all policy scenarios, runs 2000 times. For each iteration, a different set of input parameters is used by sampling from the respective distributions^^[[12]](#footnote-13)^^ of input parameters (see page 25), and a different sample of 100,000 synthetic individuals is drawn from the synthetic population of 50 million. Then, the life course^[[13]](#footnote-14)^ of every synthetic individual is simulated for the baseline, and all policy scenarios and the outcomes are collected and summarized for the population. For instance, if a synthetic individual developed CHD at the age of 50 in the baseline scenario and at the age of 60 in a policy scenario, this is counted as a CHD case postponed, as a result of the policy. Therefore, all model outputs (cases and deaths prevented or postponed, net utility, net costs, etc. described on page 26) are separately estimated for each iteration, and conditional on the set of model inputs.

The framework allows stochastic uncertainty, parameter uncertainty, and individual heterogeneity to be reflected in the reported UI. The following example illustrates the different types of uncertainty that were considered in the US Sodium Policy model. Let us assume that the annual risk of CHD is 5%. If we apply this risk to all individuals and randomly draw from a Bernoulli distribution with $p$ = 5% to select those who will manifest CHD, we only consider stochastic uncertainty. If we allow the annual risk for CHD to be conditional on individual characteristics (i.e. age, sex, exposure to risk factors), then individual heterogeneity is considered. Finally, when the uncertainty of the relative risks due to sampling errors is considered in the estimation of the annual risk for CHD, the parameter uncertainty is considered. From these three types of uncertainty, only the parameter uncertainty can be reduced from better studies in the future.

The structure of the model is grounded on fundamental epidemiological ideas and well-established causal pathways; therefore, we considered this type of uncertainty relatively small and did not study it. However, the discrete-time nature of the model can potentially introduce bias in cases where the synthetic individual dies more than once within a year, and the model cannot identify which event happened first. As we describe in page 15, to minimize this type of bias we randomly select one of the events to be considered as it happened before all others, whenever these cases arise during the simulation.

## Input uncertainty

The sources of uncertainty we considered were:

1. *The sampling error of the baseline sodium intake.* When the model calculates individualized sodium consumptions, it takes into account the sampling error of the regression models that were fitted in the NHANES (see page 11).
2. *The sampling error of the baseline SBP*. Same as above.
3. *The sampling error of the relative risks of SBP on CHD, stroke, and any-other-cause mortality*. We used the reported relative risks and their confidence intervals to construct log-normal (uniform for *any-other-cause mortality*) distributions (Table S 1).
4. *The uncertainty around the lowest exposure to sodium below which no risk is observed.* We used evidence in Mozaffarian *et al.*^3 Text S4^ as parameters for a Pert distribution (Table S 1).
5. *The uncertainty around the lowest exposure to SBP below which no risk is observed*. We used evidence in Singh *et al*.^61^ (Table S 1).
6. *The uncertainty around the effect of sodium on SBP*. We used the meta-regression equation in Mozaffarian *et al*.^3^ Each time the model uses the equation a new set of coefficients was sampled from their respective normal distributions (Table S 1).
7. *The uncertainty around the lag time of SBP exposure and disease outcomes.* The distribution 1 + Binomial(9, (5-1)/9) to vary lag time between 1 and 10 years (median 5 years).
8. *The uncertainty around the true incidence and prevalence rates of CHD and stroke*. We described in page 14 how we used DisMod II to estimate the incidence rate of CHD and stroke. We fitted beta distributions by age, sex, and race/ethnicity assuming the 0.025 percentile to be half of the central estimate, the median the central estimate, and the 0.975 percentile double the central estimate.
9. *The uncertainty of mortality forecasts*. We incorporated the predictive uncertainty of the mortality forecasts to the US Sodium Policy model estimates.
10. *The uncertainty around the assumption that half of the forecasted annual change in CHD and stroke mortality is attributed to changes in CHD and stroke incidence, respectively.* We allowed this assumption to vary, independently for each disease, between 0% and 100% following a uniform distribution.
11. *The uncertainty around which foods will be reformulated.* For policy scenarios that assumed reformulation of less than 100% of eligible products the US Sodium Policy model randomly selected eligible products to be reformulated, in each Monte Carlo iteration.
12. *The uncertainty around the quality of life decrements used to calculate QALY*. The most influential parameter of the equation was the number of coexisting chronic conditions (see page 17). Therefore, we allowed synthetic individuals to have a different number of coexisting chronic conditions in each Monte Carlo iteration.
13. *The uncertainty of all the costs.* The RTI model that was used to estimate the reformulation costs to the industry reported CI. We used these to fit generalized beta of the second kind distributions. For all other costs in the model we fitted generalized beta of the second kind distribution assuming the 0.2 percentile to be 80% of the central estimate, the median the central estimate, and the 0.8 percentile 120% of the central estimate.

## Outputs

We summarize the output distributions of the US Sodium Policy by reporting the medians and 95% uncertainty intervals (UI). We also plotted the annual probability that a scenario was cost effective or cost saving over the simulation period. Table J presents model estimates for the baseline scenario.

*Cases (Deaths) prevented or postponed*, by comparing the life course of each specific individual in the baseline scenario with its life course in the policy scenario.

*Net utility*, by summing the Quality Adjusted Life Years (QALY) through the life course of each specific individual in the baseline scenario and comparing it with the sum of QALY in the policy scenario life course.

*Net disease costs*, by summing the costs through the life course of each specific individual in the baseline scenario and comparing it with the sum of costs in the policy scenario life course.

*Policy costs,* by summing the administrative costs, monitoring and evaluation costs, and industry reformulation costs.

All outputs can be stratified by year, age, sex, race/ethnicity, and disease. Moreover, outputs are scaled to the US population (from the 100,000 sample of synthetic individuals).

Costs were analyzed in terms of incremental cost effectiveness ratio (the difference in costs divided by the difference in QALY from the baseline scenario) and net monetary benefit (NMB; incremental net costs plus the value of incremental QALY). For NMB, a central value of $100,000 per QALY gained was used based on Neumann *et al*.^62^ which was varied from $50,000 to $150,000 in a sensitivity analysis. Costs were also presented in a disaggregated ‘impact inventory’ in line with 2^nd^ US panel recommendations.

From our experience in communicating our results to policy makers and researchers, we realized that they tend to misinterpret 95% UIs as 95% confidence intervals (CI) and overlapping UIs as ‘evidence against statistical significance.' This does not apply to our model outputs because the scenarios share common model inputs as explained above and should be treated as ‘paired’ from a statistical perspective.

## One-way sensitivity analysis

For our main analysis, we assumed 100,000 USD willingness to pay per QALY and 3% annual discount rate. We varied these assumptions in the one-way sensitivity analysis, and we present the results for incremental cost-effectiveness ratio and NMB in Table E and Table F. Overall, all policy scenarios remained cost-effective under all combinations of willingness to pay and discount rates.

# Extra scenario

We present here an additional modeling scenario which assumed that only 7.5% of the eligible processed and commercially produced foods would comply with the proposed upper bound of the 2- and 10-year targets and 7.5% will comply with the main 2- and 10-year targets. These reductions are smaller than in the sodium projection we used in our baseline scenario. Therefore, for this scenario only, we used an alternative baseline that assumes sodium consumption levels will remain at 2010 level throughout the 20-year simulation period. **The change of baseline makes our estimates for this 7.5% scenario not directly comparable with the estimates for our main policy scenarios**. Table K summarizes our model estimates for this scenario.

We roughly based this scenario on two sources. The first is the results from New York City’s National Salt Reduction Initiative showed that industry participation was low; only about 3% of products met reformulation target.^63^ The second is an assumption from FDA that 7.5% of manufacturers will voluntarily reformulate added sugar given this new labeling requirement to label added sugar content on the Nutrition Facts Label.^64^ These examples highlight that setting sodium reformulation targets is necessary, but is effective only as a necessary element of a comprehensive sodium reduction strategy.^65^

# Calibration and Validation

The US Sodium Policy model is calibrated to forecasts of CHD, stroke, and any-other-cause mortality (previously described on page 15). Figure D, Figure E, and Figure F depict the observed and forecasted mortality that was used for the calibration. We included the uncertainty of the forecasts to our probabilistic uncertainty, and it is propagated in our model estimates.


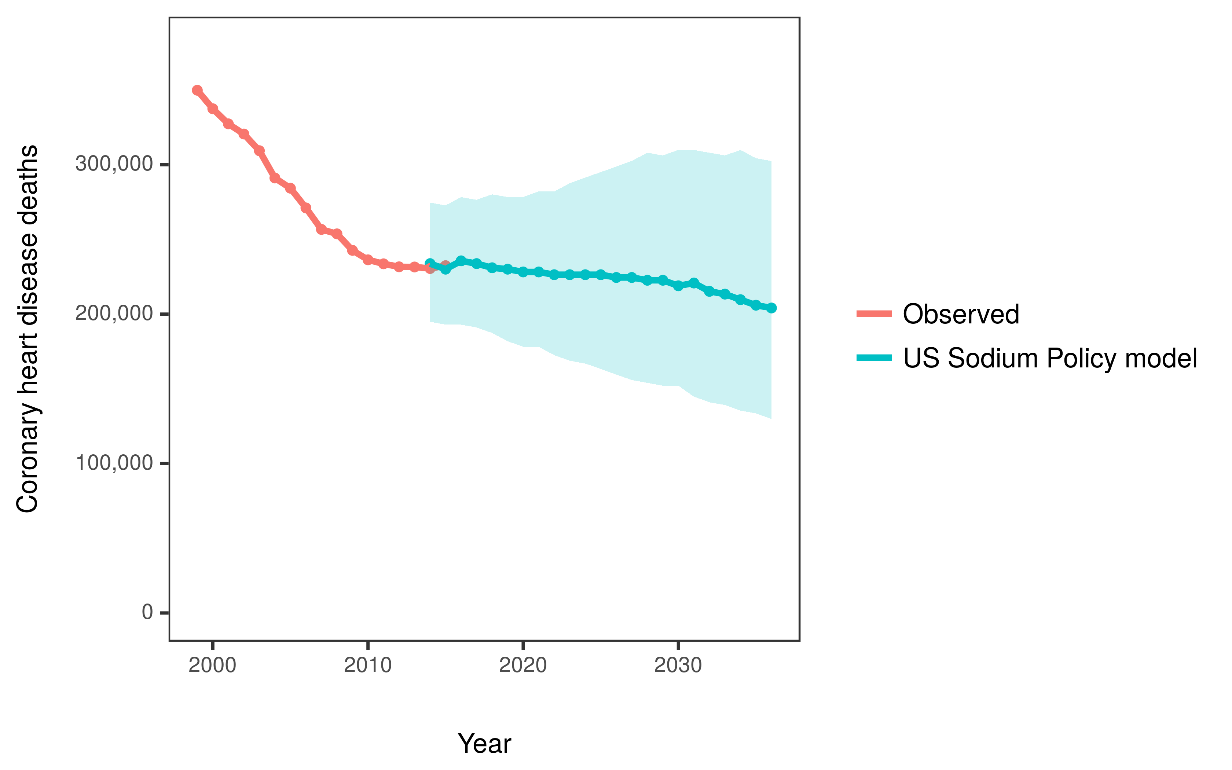


Figure D Observed and forecasted coronary heart disease mortality. US population aged 30 to 84. Shaded areas represent 95% prediction intervals. Source for observed mortality CDC WONDER database.^37^


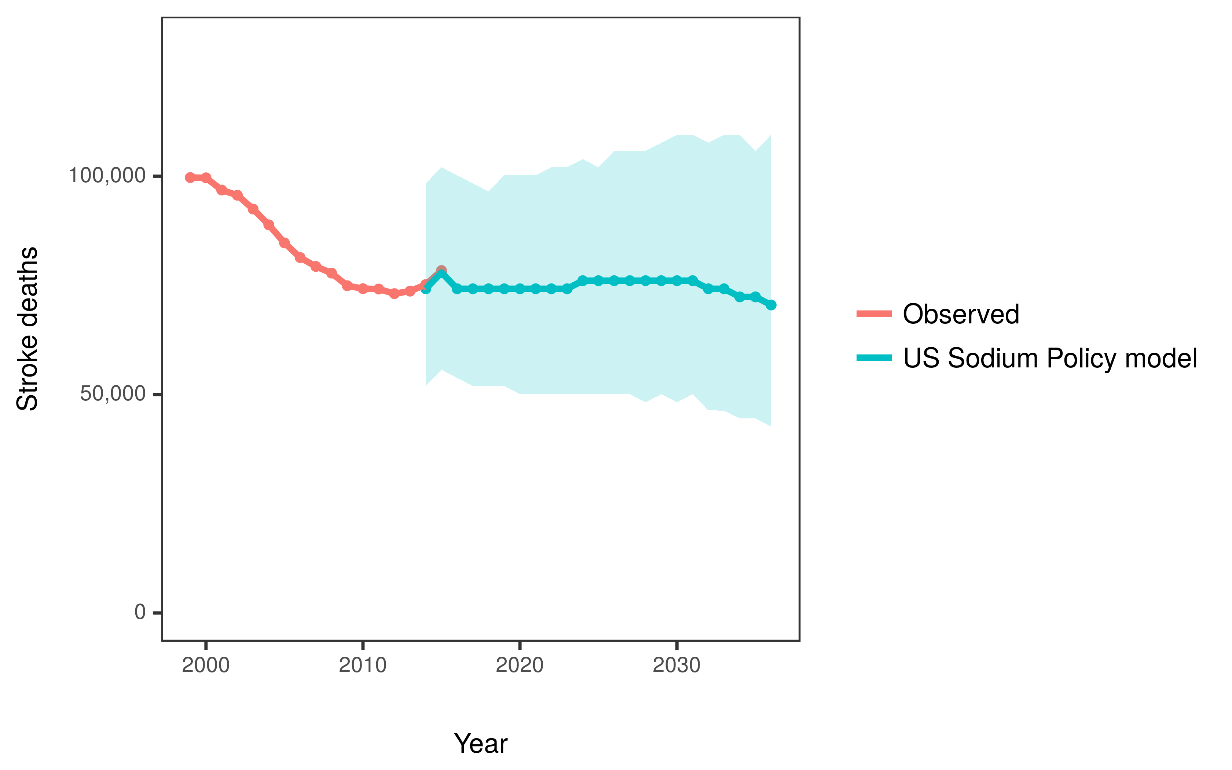


Figure E Observed and forecasted stroke mortality. US population aged 30 to 84. Shaded areas represent 95% prediction intervals. Source for observed mortality CDC WONDER database.^37^


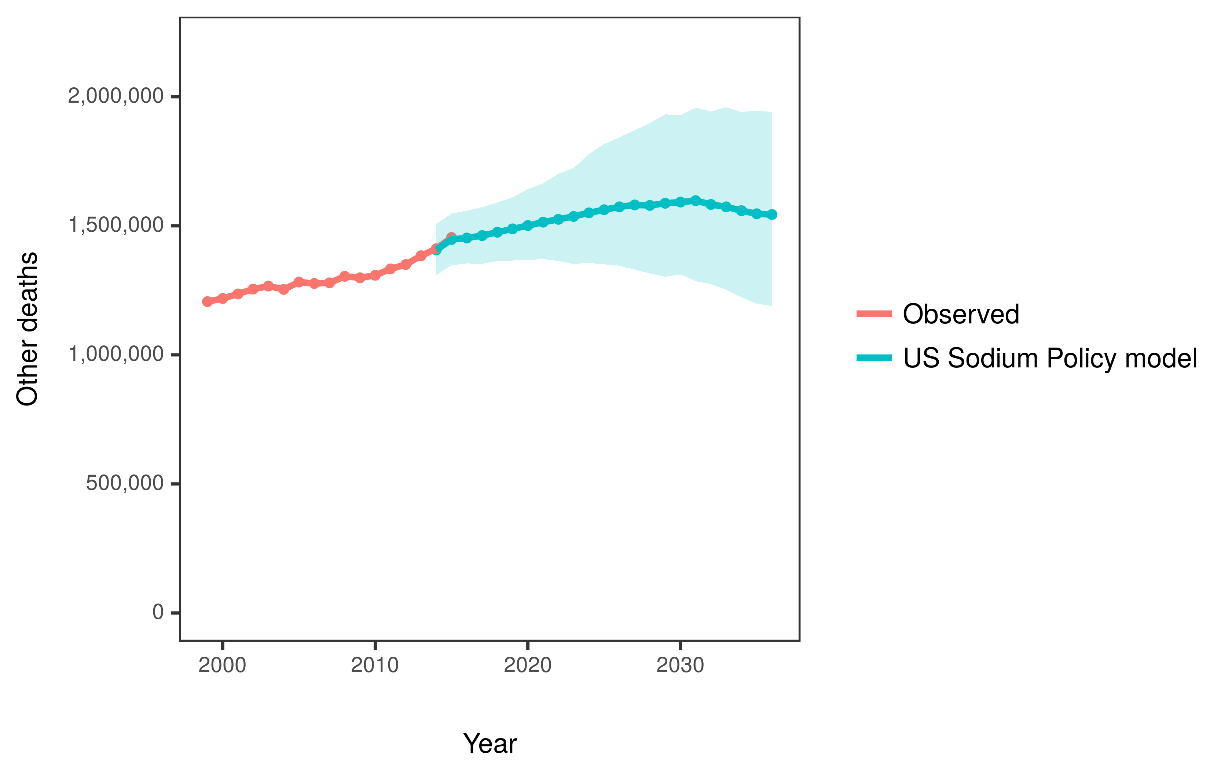


Figure F Observed and forecasted mortality from any-other-cause (excluding coronary heart disease and strokes). US population aged 30 to 84. Shaded areas represent 95% prediction intervals. Source for observed mortality CDC WONDER database.^37^

## Synthetic population internal validation

The following graphs compare a random sample of 1 million synthetic individuals from the synthetic population to the original sample of NHANES1114 (n = 10,907). Mosaic plots^^[[14]](#footnote-15)^^ were used for the categorical variables, and cumulative distribution plots were used for the continuous variables. The area of each tile of the mosaic plots is proportional to the proportion of each subgroup in the respective population. The color of each tile represents the relative difference between NHANES and the synthetic population.

The graphs support the argument that the final synthetic population is close to reality, at least as it was captured through the NHANES1114, and are useful for the internal validation of the method. Alfons *et al*. used a statistical simulation approach to evaluate the process and showed that this method produces synthetic populations very similar to the original survey.^21^ Of course, the method cannot overcome any limitations of the original survey, such as selection bias, or misclassification.


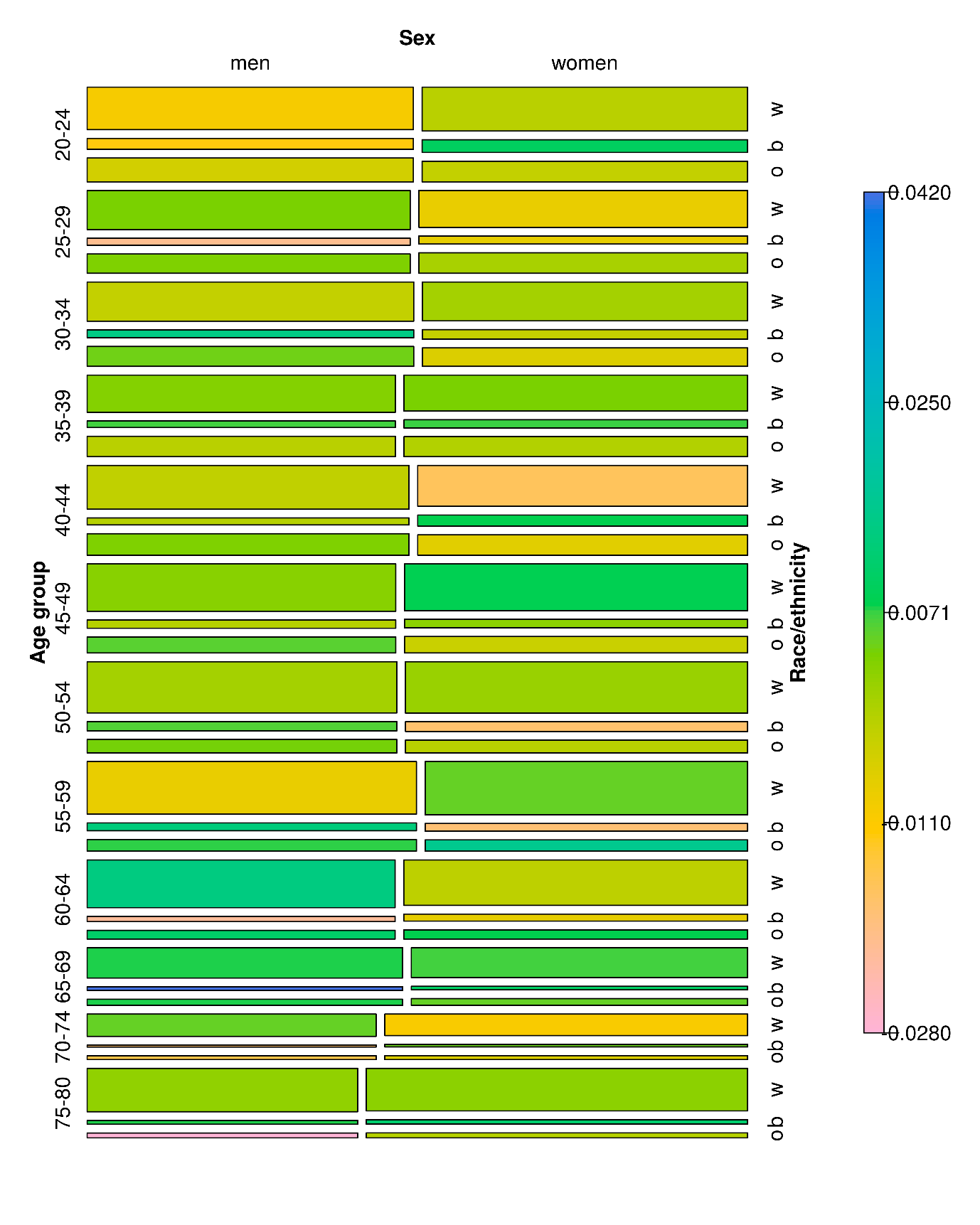


Figure G Mosaic plot for comparison of age, sex, and race/ethnicity distribution between the synthetic population and the NHANES1114 sample. Green shades depict relative differences close to 0%, and all subgroups were within a ±5% difference compared to the NHANES sample. For Race/ethnicity: b, non-Hispanic black; o, other; w, non-Hispanic white.


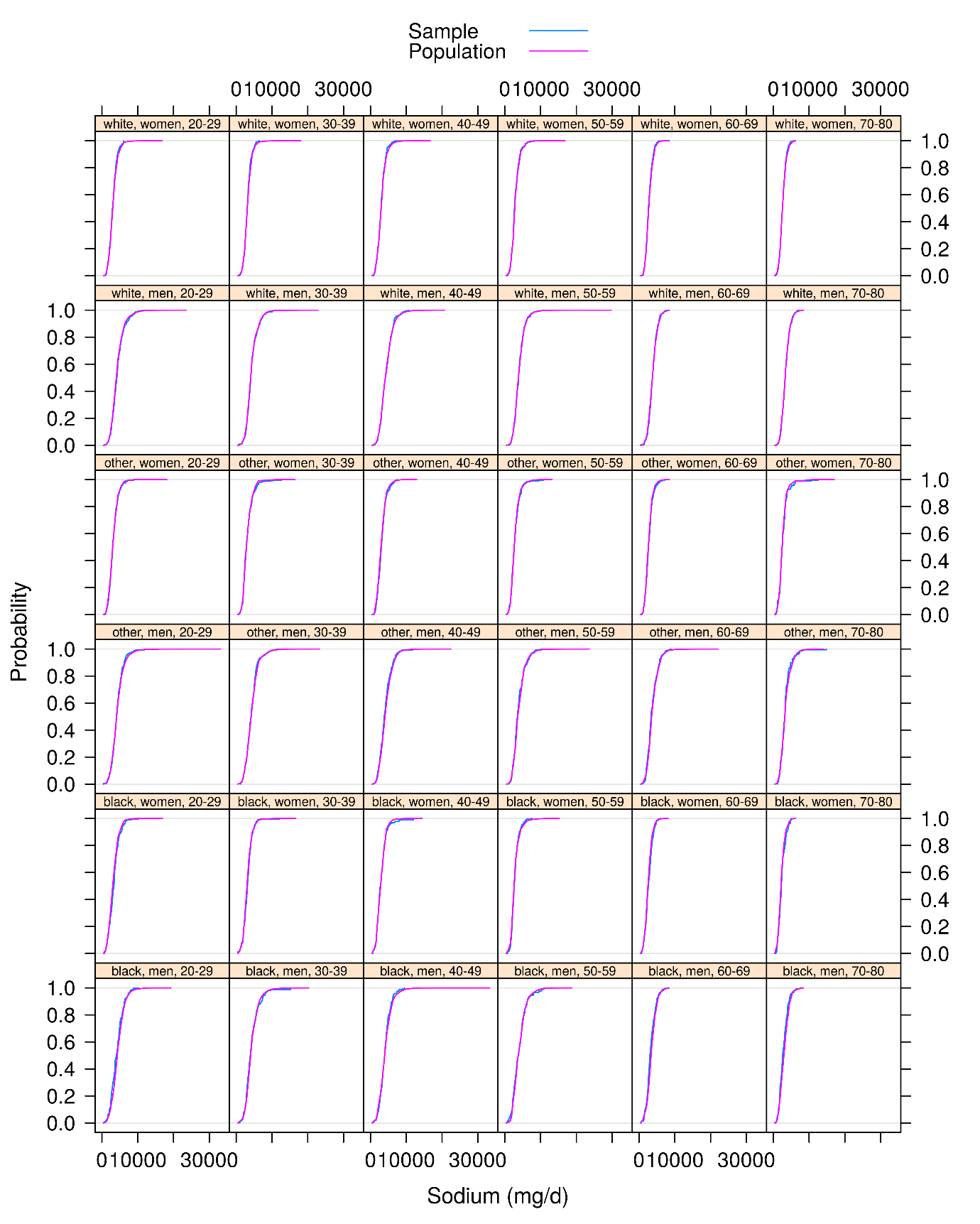


Figure H Empirical cumulative distributions of sodium consumption in NHANES1114 (sample) and the synthetic population (population), by age group, sex, and race/ethnicity.


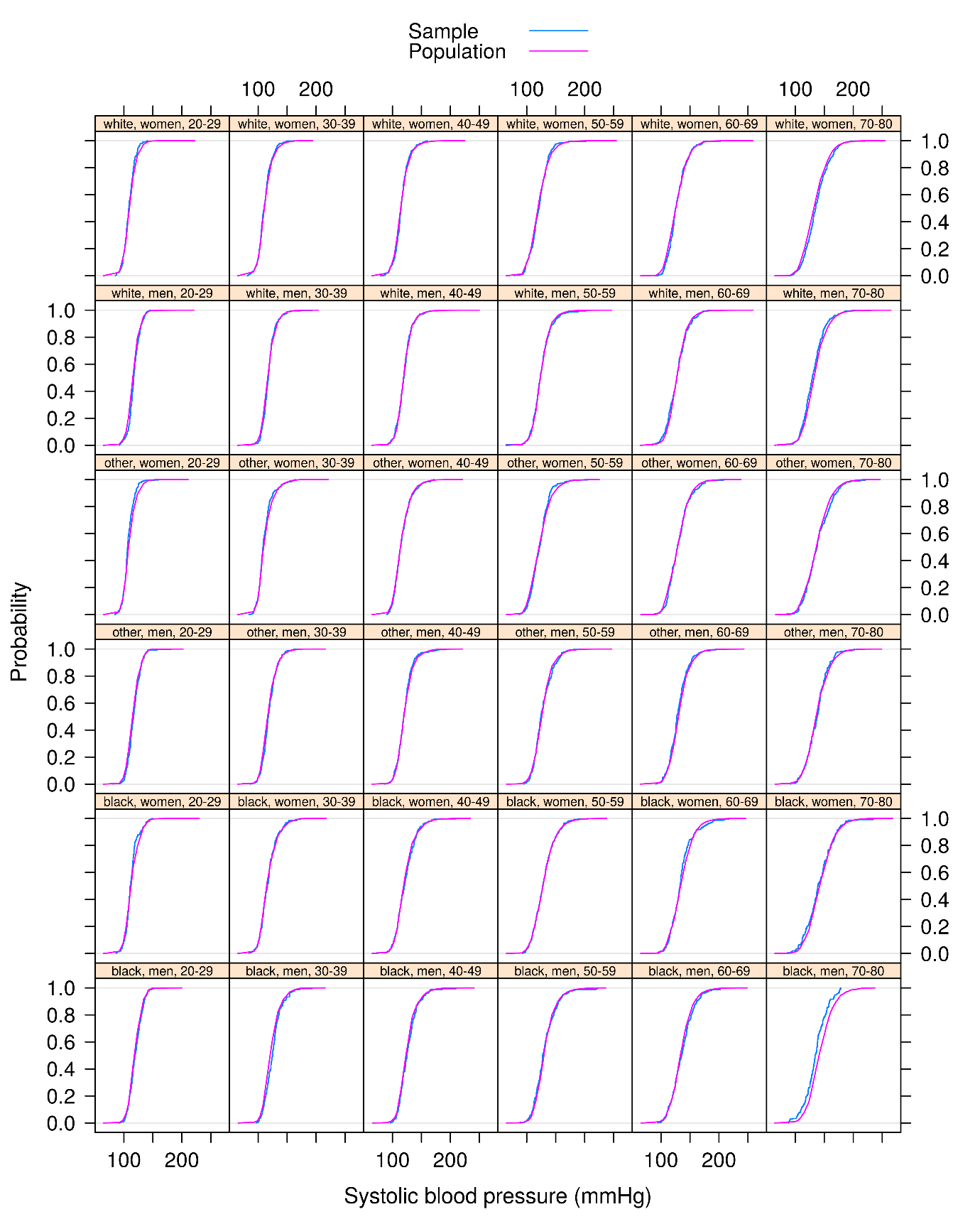


Figure I Empirical cumulative distributions of systolic blood pressure in NHANES1114 (sample) and the synthetic population (population), by age group, sex, and race/ethnicity.

# Tables

Table A The US Sodium Policy model data sources.

| Parameter | Outcome | Details | Comments | Source |
| --- | --- | --- | --- | --- |
| Population size estimates^67^ | Population | July 1 US resident population from the Vintage 2014 postcensal series, the revised 2000-2009 intercensal series, and the 1990-1999 intercensal series | Stratified by year, age, sex, bridged-race, and Hispanic origin | United States Department of Health and Human Services (US DHHS), Centers for Disease Control and Prevention (CDC), National Center for Health Statistics (NCHS). Bridged-race population estimates 1990–2014, United States July 1st resident population by age, sex, bridged-race, and Hispanic origin, on CDC WONDER on-line database [Internet]. 2016 [cited 2017 Feb 11];Available from: https://wonder.cdc.gov/Bridged-Race-v2014.HTML |
| Population projections^27^ | Population | 2014–2060 US population projections produced by the Census Bureau in 2014 | Stratified by year, age, sex, race, and ethnicity | U.S. Census Bureau. National population projections: United States by age, gender, ethnicity and race for years 2014–2060, released by the U.S. Census Bureau on December 10, 2014, on CDC WONDER on-line database [Internet]. 2015 [cited 2017 Feb 11];Available from: https://wonder.cdc.gov/population-projections-2014-2060.html |
| Mortality^37^ | Deaths from CHD, stroke, and any other non-modeled causes | Underlying cause of death 1999–2015 | Stratified by year, age, sex, race, ethnicity, and cause of death | United States Department of Health and Human Services (US DHHS), Centers for Disease Control and Prevention (CDC), National Center for Health Statistics (NCHS). Underlying cause of death 1999–2015 on CDC WONDER online database. Data are compiled from data provided by the 57 vital statistics jurisdictions through the Vital Statistics Cooperative Program [Internet]. 2016 [cited 2017 Apr 18];Available from: https://wonder.cdc.gov/ucd-icd10.html |
| Exposure to sodium^23^ | Exposure of individuals | National Health and Nutrition Examination Survey (NHANES) | Anonymized, individual-level data sets. Years 2009–2014. | Centers for Disease Control and Prevention (CDC), National Center for Health Statistics (NCHS). National Health and Nutrition Examination Survey data. Hyattsville, MD: U.S. Department of Health and Human Services, Centers for Disease Control and Prevention, 1999–2014 [Internet]. [cited 2016 Nov 15];Available from: https://wwwn.cdc.gov/nchs/nhanes/ContinuousNhanes/ |
| Exposure to systolic blood pressure ^23^ | Exposure of individuals | National Health and Nutrition Examination Survey (NHANES) | Anonymized, individual-level data sets. Years 1999–2014. | Centers for Disease Control and Prevention (CDC), National Center for Health Statistics (NCHS). National Health and Nutrition Examination Survey data. Hyattsville, MD: U.S. Department of Health and Human Services, Centers for Disease Control and Prevention, 1999–2014 [Internet]. [cited 2016 Nov 15];Available from: https://wwwn.cdc.gov/nchs/nhanes/ContinuousNhanes/ |
| Effect of sodium consumption on systolic blood pressure^3^ | Systolic blood pressure change | Meta-analysis/meta- regression of 103 trials | Only trials with duration > 7 days were analyzed. | Mozaffarian D, Fahimi S, Singh GM, *et al*. Global sodium consumption and death from cardiovascular causes. New England Journal of Medicine 2014;371(7):624–34. (Text S1 in the appendix) |
| Setting reference level of sodium consumption^3^ | Ideal sodium consumption below which no risk was considered | Evidence from ecologic studies randomized trials and meta-analyses of prospective cohort studies | Intake levels associated with the lowest risk ranged from 614 to 2391 mg/day. In large, well-controlled, randomized feeding trials, the lowest tested intake for which blood pressure reductions were clearly documented was 1500 mg/day. | Mozaffarian D, Fahimi S, Singh GM, *et al*. Global sodium consumption and death from cardiovascular causes. New England Journal of Medicine 2014;371(7):624–34. (Text S4 in the appendix and Table C) |
| Relative risk for systolic blood pressure^45,68^ | CHD and stroke (ICD10: I20–I25 and I60–I69) | Pooled analysis of two individual level meta-analysis | Stratified by age and sex. Adjusted for regression dilution and total blood cholesterol and, where available, lipid fractions (HDL and non-HDL cholesterol), diabetes, weight, alcohol consumption, and smoking at baseline. | Micha R, Peñalvo JL, Cudhea F, Imamura F, Rehm CD, Mozaffarian D. Association between dietary factors and mortality from heart disease, stroke, and type 2 diabetes in the United States. JAMA 2017;317(9):912–24. (eTable5) |
|  | Any other mortality (excluding CHD and stroke) | Individual level meta-analysis of 48 prospective cohort studies | Adjusted for age, sex, race or ethnicity, deprivation, smoking, diabetes, inactivity, alcohol, obesity | Stringhini S, Carmeli C, Jokela M, *et al*. Socioeconomic status and the 25 × 25 risk factors as determinants of premature mortality: a multicohort study and meta-analysis of 1·7 million men and women. The Lancet 2017;389(10075):1229–37. (Figure 4) |
| Setting reference level of systolic blood pressure^61^ | Ideal systolic blood pressure below which no risk was considered | Evidence from evidence from randomized trials of antihypertensive drugs and the Intersalt study | There may be health benefits by lowering systolic blood pressure down to 110mmHg | Singh GM, Danaei G, Farzadfar F, *et al*. The age-specific quantitative effects of metabolic risk factors on cardiovascular diseases and diabetes: a pooled analysis. PLOS ONE 2013;8(7):e65174. |
| Health state utility values^46^ | For CHD, stroke, hypertension, and their combinations | Uses EQ-5D-3L data from the Medical Expenditure Panel Survey (MEPS) 2000-2002 | We used the published regression coefficients to estimate utility values by age, sex, race, ethnicity, income, education, and the number of chronic conditions | Sullivan PW, Ghushchyan V. Preference-Based EQ-5D Index Scores for Chronic Conditions in the United States. Medical Decision Making 2006;26(4):410–20. (Tables 2 and 3) |
| Disease costs^47–49^ | Medical, mortality, and morbidity costs for CHD, stroke, and hypertension | Based on the Medical Expenditure Panel Survey (MEPS) | Stratified by age, sex, and race, adjusted for comorbidities | Khavjou O, Phelps D, Leib A. Projections of cardiovascular disease prevalence and costs: 2015–2035. Technical Report [Internet]. RTI International; 2016 [cited 2017 Jul 10]. Available fromhttps://www.heart.org/idc/groups/heart-public/@wcm/@adv/documents/downloadable/ucm_491513.pdf |
|  | Informal care costs for CHD |  | Costs were extrapolated for US settings | Leal J, Luengo-Fernández R, Gray A, Petersen S, Rayner M. Economic burden of cardiovascular diseases in the enlarged European Union. Eur Heart J 2006;27(13):1610–9. (Table 5) |
|  | Informal care costs for stroke | Difference-in-differences technique to propensity score-matched populations |  | Joo H, Dunet DO, Fang J, Wang G. Cost of informal caregiving associated with stroke among the elderly in the United States. Neurology 2014;83(20):1831–7. (Table 3) |
| Government costs to administer the policy^51^ |  | Administration costs for new restaurant menu and vending machine labeling regulation, including cost for outreach, education, review of regulatory issues, developing training for inspectors, and related functions | We assumed sodium reformulation to have same administrative costs | Food and Drug Administration (FDA), Department of Health and Human Services (DHHS). Food and Drug Administration justification of estimates for appropriations committees. Fiscal year 2012 [Internet]. Food and Drug Administration (FDA); 2012 [cited 2017 Jul 10]. Available from: https://www.fda.gov/downloads/AboutFDA/ReportsManualsForms/­Reports/BudgetReports/UCM243370.pdf |
| Government costs to monitor and evaluate the policy^52^ |  | UK Food Standards Agency impact assessment of UK salt reduction strategy | Costs converted to equivalent US dollars | Collins M, Mason H, O’Flaherty M, Guzman-Castillo M, Critchley J, Capewell S. An economic evaluation of salt reduction policies to reduce coronary heart disease in England: a policy modeling study. Value Health 2014;17(5):517–24. |
| Industry costs to reformulate products^53^ |  | Spreadsheet model | The model accounted for variations in product formula complexity, company size, reformulation type, compliance period and other factors | Mary K. Muth, Samantha Bradley, Jenna Brophy, *et al*. Reformulation Cost Model. Contract No. HHSF-223-2011-10005B, Task Order 20. 2015 |

All data sources are outlined in detail in Table D.

Table B Key modeling assumptions and limitations.

| **Population module** |
| --- |
| We assumed no migration after the age of 30 |
| We assumed NHANES to be representative of the US population |
| **Disease module** |
| We assumed log-linear exposure – response relationship for SBP with 5-year mean lag time |
| We only modeled first ever event of CHD and stroke because we focus on primary prevention |
| For CHD and stroke initial incidence rates (year 2014), we used modeled estimates derived from mortality and NHANES prevalence data |
| We assumed the non-attributable to SBP incidence rate trends for CHD and stroke, to be 50% of the forecasted mortality rates trends |
| We assumed that the risk ratios of SBP on CHD and stroke incidence and mortality are equal and SBP is not modifying CHD and stroke survival |
| We assumed that changes in sodium consumption have an immediate effect on SBP and changes in SBP have a median 5-year time lag to impact the risk of CVD |
| **Policy module** |
| We assumed that the recently observed trends in sodium consumption, SBP, and disease specific mortality would continue in the future (baseline scenario) |
| We assumed that US population diet has and will have similar food composition since 2010 (the most recent year we can link FDA proposed targets to NHANES)  We did not explicitly model food prepared in restaurants and food outlets |
| We assumed that individuals would not change sodium consumption behavior because of the policy |
| We assumed a gradual linear diffusion of the reformulation effect to the population. Once the maximum policy effect was reached, we assumed it would sustain for the rest of the simulation period |
| **Policy micro costing** |
| We estimated the proportion of eligible for reformulation products from the Food and Nutrient Database for Dietary Studies v5 |
| We assumed two rounds of reformulation, one for the 2-year and one for the 10-year target (except for the pessimistic scenario for which only the first cycle was considered) |
| We assumed that the proportion of sodium reduction during reformulation is not related to the cost of reformulation (echoing the assumption of RTI cost model) |

Table C Mismatches between the proposed FDA reformulation policy and the FNDDS v5 database that were handled manually. With the exception of imitation cheese (1^st^ row) all other foods that were manually assigned to a relevant FDA food category were not eligible for reformulation.

| FNDDS food description | FDA food category | Sodium (mg/100g) for the FNDDS food description | Mean sodium in the FDA food category (mg/100g) |
| --- | --- | --- | --- |
| Imitation cheese, American or cheddar type | Feta Cheese (Soft) | 1345 | 1116 |
| Tuna, fresh, raw | Non-Breaded Fish and Other Seafood | 45 | 559 |
| Oysters, raw | Non-Breaded Fish and Other Seafood | 85 | 559 |
| White potato, chips, restructured, reduced fat and reduced sodium | Unflavored Potato and Vegetable Chips | 411 | 550 |
| White potato, chips, unsalted | Unflavored Potato and Vegetable Chips | 8 | 550 |
| White potato, chips, unsalted, reduced fat | Unflavored Potato and Vegetable Chips | 8 | 550 |
| Sweet potato, chips | Unflavored Potato and Vegetable Chips | 35 | 550 |
| Cheese, Cheddar or Colby, low sodium | Cheddar and Colby Cheese (Hard) | 21 | 702 |
| Cheese, Swiss, low sodium | Swiss and Swiss-type Cheese (Hard) | 14 | 263 |
| Butter, stick, unsalted | Butter | 11 | 631 |
| Butter, whipped, tub, unsalted | Butter | 11 | 631 |
| Cucumber salad with creamy dressing | Lettuce/Green Salads: With Additions - Without Dressing | 17 | 339 |
| Artichoke salad in oil | Lettuce/Green Salads: With Additions - Without Dressing | 55 | 339 |
| Cookie, fruit, baby | Toddler Meals and Entrees | 9 | 226 |
| Oatmeal cereal with fruit, baby food, dry, instant, toddler | Toddler Meals and Entrees | 0 | 226 |
| Macaroni, tomatoes, and beef, baby food, junior | Toddler Meals and Entrees | 35 | 226 |
| Macaroni with beef and tomato sauce, baby food, toddler | Toddler Meals and Entrees | 35 | 226 |
| Margarine, stick, unsalted | Margarine and Vegetable Oil Spreads | 2 | 670 |
| Margarine, tub, unsalted | Margarine and Vegetable Oil Spreads | 28 | 670 |
| Margarine-like spread, tub, unsalted | Margarine and Vegetable Oil Spreads | 2 | 670 |
| Cookie, fruit, baby food | Baby/Toddler Snacks: Cookies/Biscuits | 9 | 326 |
| Fruit dressing, made with honey, oil, and water | Salad Dressing | 2 | 867 |
| Milk, vinegar, and sugar dressing | Salad Dressing | 47 | 867 |
| Peppers, hot, cooked, from frozen, fat not added in cooking | Frozen Vegetables and Legumes | 7 | 254 |
| Turnip greens, canned, low sodium, cooked, fat not added in cooking | Canned Vegetables | 29 | 267 |
| Carrots, canned, low sodium, fat not added in cooking | Canned Vegetables | 34 | 267 |
| Peas and carrots, canned, low sodium, fat added in cooking | Canned Vegetables | 16 | 267 |
| Peas and carrots, canned, low sodium, fat not added in cooking | Canned Vegetables | 4 | 267 |
| Sweet potato, canned, NS as to syrup | Canned Vegetables | 44 | 267 |
| Tomatoes, canned, low sodium | Canned Vegetables | 10 | 267 |
| Beans, string, green, canned, low sodium, NS as to fat added in cooking | Canned Vegetables | 21 | 267 |
| Beans, string, green, canned, low sodium, fat not added in cooking | Canned Vegetables | 2 | 267 |
| Beans, string, green, canned, low sodium, fat added in cooking | Canned Vegetables | 21 | 267 |
| Beets, canned, low sodium, fat not added in cooking | Canned Vegetables | 21 | 267 |
| Corn, yellow, canned, low sodium, fat not added in cooking | Canned Vegetables | 1 | 267 |
| Corn, yellow, canned, low sodium, fat added in cooking | Canned Vegetables | 19 | 267 |
| Peas, green, canned, low sodium, fat not added in cooking | Canned Vegetables | 9 | 267 |
| Peas, green, canned, low sodium, fat added in cooking | Canned Vegetables | 21 | 267 |
| Peppers, hot, cooked, from canned, fat not added in cooking | Canned Vegetables | 7 | 267 |
| Peppers, hot, cooked, from canned, fat added in cooking | Canned Vegetables | 7 | 267 |
| Pimiento | Canned Vegetables | 17 | 267 |
| Mixed vegetables (corn, lima beans, peas, green beans, and carrots), | Canned Vegetables | 26 | 267 |
| Mixed vegetables (corn, lima beans, peas, green beans, and carrots), | Canned Vegetables | 42 | 267 |
| Cucumber pickles, dill, reduced salt | Pickled Vegetables | 18 | 433 |
| Carrot juice | Vegetable Juice | 66 | 235 |
| Tomato juice, low sodium | Vegetable Juice | 10 | 235 |
| Tomato and vegetable juice, mostly tomato, low sodium | Vegetable Juice | 70 | 235 |
| Mixed vegetable juice (vegetables other than tomato) | Vegetable Juice | 63 | 235 |
| Celery juice | Vegetable Juice | 91 | 235 |
| Aloe vera juice | Vegetable Juice | 2 | 235 |
| Vegetable and fruit juice blend, 100% juice, with high vitamin C plus | Vegetable Juice | 29 | 235 |
| White potato, boiled, without peel, canned, low sodium, fat not added in | Potato Side Dishes | 5 | 315 |
| Almond paste (Marzipan paste) | Nut/Seed Butters and Pastes | 9 | 335 |
| Peanut butter, low sodium | Nut/Seed Butters and Pastes | 17 | 335 |
| Peanut butter, reduced sodium, and reduced sugar | Nut/Seed Butters and Pastes | 203 | 335 |
| Almonds, unroasted | Nuts and Seeds | 1 | 366 |
| Almonds, dry roasted, without salt | Nuts and Seeds | 3 | 366 |
| Brazil nuts | Nuts and Seeds | 3 | 366 |
| Cashew nuts, roasted, without salt | Nuts and Seeds | 13 | 366 |
| Cashew nuts, dry roasted, without salt | Nuts and Seeds | 16 | 366 |
| Chestnuts, roasted | Nuts and Seeds | 2 | 366 |
| Filberts, hazelnuts | Nuts and Seeds | 0 | 366 |
| Peanuts, roasted, without salt | Nuts and Seeds | 6 | 366 |
| Peanuts, dry roasted, without salt | Nuts and Seeds | 6 | 366 |
| Pecans | Nuts and Seeds | 0 | 366 |
| Pine nuts (Pignolias) | Nuts and Seeds | 2 | 366 |
| Walnuts | Nuts and Seeds | 2 | 366 |
| Walnuts, honey-roasted | Nuts and Seeds | 20 | 366 |
| Nut mixture with dried fruit and seeds | Nuts and Seeds | 7 | 366 |
| Pumpkin and/or squash seeds, hulled, unroasted | Nuts and Seeds | 7 | 366 |
| Pumpkin and/or squash seeds, hulled, roasted, without salt | Nuts and Seeds | 18 | 366 |
| Sunflower seeds, hulled, unroasted | Nuts and Seeds | 9 | 366 |
| Sunflower seeds, hulled, roasted, without salt | Nuts and Seeds | 3 | 366 |
| Sesame seeds | Nuts and Seeds | 39 | 366 |
| Flax seeds | Nuts and Seeds | 30 | 366 |
| Chicken or turkey soup, cream of, canned, reduced sodium, made with | Canned, Condensed Soup | 185 | 358 |
| Chicken rice soup, canned, reduced sodium, prepared with water or | Canned, Condensed Soup | 164 | 358 |
| Tomato soup, canned, reduced sodium, prepared with water | Canned, Condensed Soup | 13 | 358 |
| Tomato soup, canned, reduced sodium, prepared with milk | Canned, Condensed Soup | 33 | 358 |
| Mushroom soup, cream of, canned, reduced sodium, NS as to made with | Canned, Condensed Soup | 207 | 358 |
| Beer soup, made with milk | Canned, Ready-to-Eat Soup | 24 | 311 |
| Beef and mushroom soup, canned, low sodium | Canned, Ready-to-Eat Soup | 25 | 311 |
| Clam chowder, New England, canned, reduced sodium, ready-to-serve | Canned, Ready-to-Eat Soup | 194 | 311 |
| Bean and ham soup, canned, reduced sodium, prepared with water or | Canned, Ready-to-Eat Soup | 187 | 311 |
| Chicken noodle soup, canned, low sodium, ready-to-serve | Canned, Ready-to-Eat Soup | 173 | 311 |
| Chicken noodle soup, canned, reduced sodium, ready-to-serve | Canned, Ready-to-Eat Soup | 193 | 311 |
| Soup, mostly noodles, reduced fat, reduced sodium | Canned, Ready-to-Eat Soup | 212 | 311 |
| Tomato soup, canned, low sodium, ready-to-serve | Canned, Ready-to-Eat Soup | 33 | 311 |
| Vegetable soup, canned, low sodium, prepared with water or ready-to- | Canned, Ready-to-Eat Soup | 198 | 311 |
| Minestrone soup, canned, reduced sodium, ready-to-serve | Canned, Ready-to-Eat Soup | 215 | 311 |
| Vegetable noodle soup, canned, reduced sodium, prepared with water or | Canned, Ready-to-Eat Soup | 196 | 311 |
| Vegetable soup, made from dry mix, low sodium | Dry Mix Soup | 194 | 3342 |
| Chicken broth, canned, low sodium | Shelf Stable Liquid Broth and Stock | 30 | 247 |
| Soy sauce, reduced sodium | Soy Sauce | 3333 | 5637 |
| Cheese, processed, American or Cheddar type, low sodium | Processed Cheese/Cheese Food (Semi-soft) | 7 | 1433 |
| Lemon-butter sauce | Asian-style Sauce | 3 | 1814 |
| Fruit sauce | Asian-style Sauce | 62 | 1814 |
| Tomato catsup, low sodium | Condiments | 20 | 842 |
| 100 % Natural Cereal, with oats, honey and raisins, Quaker | Ready-to-Eat Cereal, Flakes | 54 | 492 |
| Wheat germ, plain | Ready-to-Eat Cereal, Flakes | 4 | 492 |
| Crispy Brown Rice Cereal | Ready-to-Eat Cereal, Puffed | 12 | 529 |
| Froot Loops Cereal Straws | Ready-to-Eat Cereal, Puffed | 49 | 529 |
| Kashi, Puffed | Ready-to-Eat Cereal, Puffed | 10 | 529 |
| Malt-O-Meal Puffed Wheat | Ready-to-Eat Cereal, Puffed | 5 | 529 |
| 100% Natural Cereal, plain, Quaker | Ready-to-Eat Cereal, Puffed | 50 | 529 |
| Wheat, puffed, plain | Ready-to-Eat Cereal, Puffed | 5 | 529 |
| Kashi cereal, NS as to ready to eat or cooked | Prepared Cooked Cereal | 10 | 178 |
| Cornstarch with milk, eaten as a cereal (2 tbsp cornstarch in 2-1/2 cups | Dry Mix Instant Cereal | 46 | 221 |
| Nestum cereal | Dry Mix Instant Cereal | 35 | 221 |
| Flour and water patty | White Bread | 4 | 464 |
| Tortilla, corn | Tortillas and Wraps | 45 | 619 |
| Crackers, saltine, low sodium | Crackers | 198 | 757 |
| Crackers, saltine, fat free, low sodium | Crackers | 716 | 757 |
| Crackers, toast thins (rye, wheat, white flour), low sodium | Crackers | 190 | 757 |
| Cracker, 100% whole wheat, low sodium | Crackers | 186 | 757 |
| Cracker, snack, low sodium | Crackers | 216 | 757 |
| Cracker, cheese, low sodium | Crackers | 458 | 757 |
| Cracker, snack, lowfat, low sodium | Crackers | 292 | 757 |
| Crispbread, wheat, low sodium | Crackers | 324 | 757 |
| Cracker, multigrain, salt free | Crackers | 73 | 757 |
| Crispbread, rye, low sodium | Crackers | 453 | 757 |
| Crackers, matzo | Crackers | 0 | 757 |
| Cake made with glutinous rice | Cake | 12 | 332 |
| Pastry, fruit-filled | Pastries, Pie, and Cobbler | 8 | 251 |
| Ham, sliced, low salt, prepackaged or deli, luncheon meat | Deli Meats - Ham | 969 | 1164 |
| Beef, sandwich steak (flaked, formed, thinly sliced) | Deli Meats - Beef | 74 | 1055 |
| Turkey or chicken breast, low salt, prepackaged or deli, luncheon meat | Deli Meats -Turkey/Chicken | 772 | 1030 |
| Ground beef patty, cooked (for fast food sandwiches) | Deli Meats - Loaves/Mixtures | 77 | 1164 |
| Frankfurter or hot dog, low salt | Frankfurters, Hot Dogs, and Bologna | 314 | 1063 |
| Bologna, beef, lower sodium | Frankfurters, Hot Dogs, and Bologna | 682 | 1063 |
| Pork bacon, smoked or cured, lower sodium | Uncooked Bacon | 1030 |  |
| Cheese, Mozzarella, low sodium | Pasta Filata Cheese (Soft) | 16 | 668 |

Table D Distributions that were used as inputs for the simulations. Numbers are rounded to the third significant digit.

Table D is provided as a separate file: S1 Table.

Table E One-way sensitivity analysis based on different discount rates and willingness to pay for 1 QALY. Results are incremental cost-effectiveness ratio (2017 US Dollars per QALY) over the 20-year simulation period from 2017 to 2036, for US adults age 30 to 84 years. Values are the median estimate (95% UI). Rounded to the second significant digit.

| Willingness to pay | Discount | Optimal policy scenario | Modest policy scenario | Pessimistic policy scenario |
| --- | --- | --- | --- | --- |
| $50,000 | 0% | -23,000 (-43,000 to -11,000) | -21,000 (-41,000 to -7,600) | -22,000 (-47,000 to -4,900) |
|  | 3% | -20,000 (-38,000 to -7,300) | -17,000 (-36,000 to -3,400) | -17,000 (-41,000 to 540) |
|  | 6% | -16,000 (-34,000 to -2,100) | -13,000 (-31,000 to 2,500) | -12,000 (-35,000 to 6,500) |
|  | 9% | -12,000 (-29,000 to 4,600) | -9,200 (-27,000 to 8,700) | -7,600 (-29,000 to 13,000) |
| $100,000 | 0% | -23,000 (-43,000 to -11,000) | -21,000 (-41,000 to -7,600) | -22,000 (-47,000 to -4,900) |
|  | 3% | -20,000 (-38,000 to -7,300) | -17,000 (-36,000 to -3,400) | -17,000 (-41,000 to 540) |
|  | 6% | -16,000 (-34,000 to -2,100) | -13,000 (-31,000 to 2,500) | -12,000 (-35,000 to 6,500) |
|  | 9% | -12,000 (-29,000 to 4,600) | -9,200 (-27,000 to 8,700) | -7,600 (-29,000 to 13,000) |
| $150,000 | 0% | -23,000 (-43,000 to -11,000) | -21,000 (-41,000 to -7,600) | -22,000 (-47,000 to -4,900) |
|  | 3% | -20,000 (-38,000 to -7,300) | -17,000 (-36,000 to -3,400) | -17,000 (-41,000 to 540) |
|  | 6% | -16,000 (-34,000 to -2,100) | -13,000 (-31,000 to 2,500) | -12,000 (-35,000 to 6,500) |
|  | 9% | -12,000 (-29,000 to 4,600) | -9,200 (-27,000 to 8,700) | -7,600 (-29,000 to 13,000) |

Table F One-way sensitivity analysis based on different discount rates and willingness to pay for 1 QALY. Results are net monetary benefit over the 20-year simulation period from 2017 to 2036, of US adults age 30 to 84 years. Values are the median estimate (95% UI). Rounded to the second significant digit. Costs are presented in (discounted) 2017 Billion US Dollars.

| Willingness to pay | Discount | Optimal policy scenario | Modest policy scenario | Pessimistic policy scenario |
| --- | --- | --- | --- | --- |
| $50,000 | 0% | 210bn (160bn to 280bn) | 110bn (82bn to 150bn) | 64bn (43bn to 91bn) |
|  | 3% | 140bn (110bn to 190bn) | 75bn (54bn to 100bn) | 46bn (30bn to 66bn) |
|  | 6% | 98bn (70bn to 130bn) | 51bn (35bn to 70bn) | 34bn (22bn to 50bn) |
|  | 9% | 68bn (46bn to 92bn) | 35bn (22bn to 49bn) | 25bn (15bn to 37bn) |
| $100,000 | 0% | 360bn (280bn to 440bn) | 190bn (150bn to 240bn) | 110bn (78bn to 150bn) |
|  | 3% | 250bn (190bn to 300bn) | 130bn (100bn to 170bn) | 81bn (59bn to 110bn) |
|  | 6% | 170bn (140bn to 210bn) | 92bn (70bn to 120bn) | 61bn (45bn to 82bn) |
|  | 9% | 120bn (94bn to 150bn) | 65bn (49bn to 83bn) | 47bn (34bn to 63bn) |
| $150,000 | 0% | 500bn (400bn to 600bn) | 270bn (210bn to 330bn) | 150bn (110bn to 200bn) |
|  | 3% | 350bn (280bn to 420bn) | 190bn (150bn to 230bn) | 120bn (87bn to 150bn) |
|  | 6% | 250bn (200bn to 300bn) | 130bn (100bn to 160bn) | 89bn (68bn to 110bn) |
|  | 9% | 180bn (140bn to 210bn) | 95bn (74bn to 120bn) | 69bn (53bn to 88bn) |

## Additional result from main analysis

Table G Health related model estimates, impact Inventory and cost-effectiveness analysis over the 20-year simulation period from 2017 to 2036, for US adults age 30 to 84 years by sex. Values are the median estimate (95% UI). Results are rounded to the first digit for sodium, first decimal for SBP, and second significant digit for other outcomes. Negative costs represent savings. Policy costs were divided proportionally to population size for each sex. Costs are presented in discounted 2017 Billion US Dollars. CHD, coronary heart disease; CVD, cardiovascular disease; QALY, quality adjusted life years; SBP, systolic blood pressure; UI, uncertainty intervals.

| Model outputs | Sex | Optimal policy scenario | Modest policy scenario | Pessimistic policy scenario |
| --- | --- | --- | --- | --- |
| Median sodium consumption in 2036 (mg/d) | Men | 2,644 (2,630 to 2,658) | 2,991 (2,950 to 3,031) | 3,350 (3,329 to 3,370) |
|  | Women | 1,882 (1,873 to 1,892) | 2,144 (2,115 to 2,173) | 2,385 (2,373 to 2,397) |
| Median SBP in 2036 (mmHg) | Men | 117.2 (117 to 117.4) | 117.8 (117.6 to 118.1) | 118.4 (118.2 to 118.6) |
|  | Women | 109.9 (109.7 to 110.3) | 110.4 (110.2 to 110.7) | 110.8 (110.5 to 111) |
| CVD cases prevented or postponed | Men | 280,000 (140,000 to 440,000) | 110,000 (52,000 to 200,000) | 37,000 (0 to 89,000) |
|  | Women | 170,000 (89,000 to 300,000) | 100,000 (52,000 to 190,000) | 78,000 (37,000 to 140,000) |
| CVD deaths prevented or postponed | Men | 21,000 (-1,900 to 50,000)* | 9,300 (-13,000 to 32,000)* | 5,600 (-15,000 to 28,000)* |
|  | Women | 13,000 (-3,700 to 33,000)* | 8,400 (-7,400 to 26,000)* | 7,400 (-7,400 to 24,000)* |
| Non-CVD deaths prevented or postponed | Men | 28,000 (1,900 to 56,000) | 11,000 (-11,000 to 35,000)* | 0 (-22,000 to 22,000)* |
|  | Women | 20,000 (1,900 to 41,000) | 13,000 (-3,700 to 30,000)* | 7,400 (-7,400 to 24,000)* |
| All deaths prevented or postponed | Men | 48,000 (26,000 to 78,000) | 20,000 (1,900 to 43,000) | 5,600 (0 to 24,000) |
|  | Women | 33,000 (15,000 to 56,000) | 20,000 (5,600 to 43,000) | 15,000 (3,700 to 30,000) |
| Life years gained | Men | 170,000 (67,000 to 310,000) | 110,000 (26,000 to 220,000) | 89,000 (15,000 to 200,000) |
|  | Women | 320,000 (160,000 to 550,000) | 140,000 (3,700 to 310,000) | 82,000 (0 to 230,000) |
| Discounted QALY gained (million) | Men | 1.2m (1m to 1.5m) | 0.6m (0.46m to 0.76m) | 0.31m (0.18m to 0.44m) |
|  | Women | 0.82m (0.58m to 1m) | 0.51m (0.37m to 0.64m) | 0.38m (0.28m to 0.48m) |
| Change in health-related costs: | Men | -37bn (-63bn to -24bn) | -17bn (-30bn to -8.8bn) | -9.3bn (-20bn to -1.5bn) |
|  | Women | -21bn (-35bn to -12bn) | -13bn (-22bn to -7.3bn) | -10bn (-19bn to -5.5bn) |
| Hypertension medical costs | Men | -10bn (-14bn to -7.1bn) | -4.7bn (-6.8bn to -3.2bn) | -1.4bn (-2.6bn to -0.5bn) |
|  | Women | -7.4bn (-11bn to -4.8bn) | -4.6bn (-6.6bn to -3bn) | -3bn (-4.3bn to -2bn) |
| Hypertension productivity costs | Men | -8.6bn (-12bn to -6bn) | -4.6bn (-6.4bn to -3.1bn) | -2.2bn (-3.4bn to -1.3bn) |
|  | Women | -2.9bn (-4.1bn to -1.8bn) | -1.8bn (-2.6bn to -1.2bn) | -1.3bn (-1.8bn to -0.83bn) |
| CHD medical costs | Men | -3.8bn (-8.7bn to -1.3bn) | -1.4bn (-3.5bn to -0.16bn) | -0.94bn (-2.6bn to 0.0bn) |
|  | Women | -3.3bn (-8.1bn to -0.98bn) | -2bn (-4.9bn to -0.54bn) | -1.8bn (-4.6bn to -0.45bn) |
| CHD mortality productivity costs | Men | -3.8bn (-19bn to 1.7bn) | -1.7bn (-10bn to 3.7bn) | -1.3bn (-9bn to 4.8bn) |
|  | Women | -0.84bn (-6.8bn to 0.98bn) | -0.48bn (-5.1bn to 1.2bn) | -0.45bn (-4bn to 1.2bn) |
| CHD morbidity productivity costs | Men | -0.89bn (-2.3bn to -0.22bn) | -0.39bn (-1.1bn to -0.047bn) | -0.28bn (-0.8bn to 0.0bn) |
|  | Women | -0.39bn (-1.1bn to -0.083bn) | -0.24bn (-0.72bn to -0.033bn) | -0.22bn (-0.66bn to -0.03bn) |
| CHD informal care costs | Men | -0.94bn (-2.2bn to -0.31bn) | -0.36bn (-0.93bn to -0.058bn) | -0.26bn (-0.69bn to 0.0bn) |
|  | Women | -0.55bn (-1.4bn to -0.15bn) | -0.33bn (-0.88bn to -0.092bn) | -0.31bn (-0.79bn to -0.078bn) |
| Stroke medical costs | Men | -3.1bn (-7.2bn to -1bn) | -1.4bn (-3.7bn to -0.26bn) | -1.1bn (-2.9bn to -0.057bn) |
|  | Women | -2.2bn (-5.7bn to -0.7bn) | -1.4bn (-3.6bn to -0.33bn) | -1.2bn (-3.2bn to -0.29bn) |
| Stroke mortality productivity costs | Men | -1.7bn (-8.7bn to 1.6bn) | -0.86bn (-5.7bn to 2.3bn) | -0.71bn (-5.1bn to 2.6bn) |
|  | Women | -0.57bn (-4.1bn to 0.69bn) | -0.33bn (-2.7bn to 0.96bn) | -0.3bn (-3bn to 0.99bn) |
| Stroke morbidity productivity costs | Men | -0.48bn (-1.2bn to -0.13bn) | -0.23bn (-0.66bn to -0.018bn) | -0.18bn (-0.54bn to 0.0bn) |
|  | Women | -0.26bn (-0.74bn to -0.048bn) | -0.16bn (-0.49bn to -0.016bn) | -0.14bn (-0.44bn to -0.0035bn) |
| Stroke informal care costs | Men | -1.8bn (-4.8bn to -0.48bn) | -0.7bn (-2.3bn to -0.086bn) | -0.53bn (-1.8bn to 0.0bn) |
|  | Women | -1.3bn (-3.7bn to -0.33bn) | -0.75bn (-2.2bn to -0.16bn) | -0.69bn (-2bn to -0.14bn) |
| Change in policy costs: | Men | 8.1bn (3.1bn to 17bn) | 5bn (1.9bn to 10bn) | 3.6bn (1.4bn to 7.2bn) |
|  | Women | 8.5bn (3.2bn to 17bn) | 5.3bn (2bn to 11bn) | 3.8bn (1.5bn to 7.7bn) |
| Policy admin costs | Men | 0.079bn (0.057bn to 0.11bn) | 0.079bn (0.057bn to 0.11bn) | 0.079bn (0.057bn to 0.11bn) |
|  | Women | 0.083bn (0.06bn to 0.11bn) | 0.083bn (0.06bn to 0.11bn) | 0.083bn (0.06bn to 0.11bn) |
| Policy monitoring costs | Men | 0.014bn (0.01bn to 0.019bn) | 0.014bn (0.01bn to 0.019bn) | 0.014bn (0.01bn to 0.019bn) |
|  | Women | 0.015bn (0.011bn to 0.02bn) | 0.015bn (0.011bn to 0.02bn) | 0.015bn (0.011bn to 0.02bn) |
| Policy industry costs | Men | 8bn (3bn to 16bn) | 4.9bn (1.8bn to 10bn) | 3.5bn (1.3bn to 7.2bn) |
|  | Women | 8.4bn (3.2bn to 17bn) | 5.2bn (1.9bn to 11bn) | 3.7bn (1.4bn to 7.6bn) |
| Total net cost (medical perspective) | Men | -17bn (-27bn to -12bn) | -7.6bn (-12bn to -4.8bn) | -3.5bn (-6.6bn to -1.6bn) |
|  | Women | -13bn (-22bn to -8bn) | -8.1bn (-13bn to -5bn) | -6.2bn (-11bn to -3.7bn) |
| Total net cost (societal perspective) | Men | -28bn (-54bn to -13bn) | -12bn (-25bn to -2.3bn) | -5.7bn (-16bn to 3.1bn) |
|  | Women | -12bn (-28bn to -0.02bn) | -7.2bn (-17bn to 0.25bn) | -6.1bn (-15bn to -0.34bn) |
| Net monetary benefit in billion (valuing QALY at $100,000) | Men | 150bn (120bn to 190bn) | 72bn (53bn to 97bn) | 37bn (18bn to 58bn) |
|  | Women | 94bn (63bn to 120bn) | 59bn (40bn to 76bn) | 44bn (31bn to 59bn) |
| Incremental cost-effectiveness ratio (2017 US Dollars per QALY) | Men | -23,000 (-44,000 to -11,000) | -19,000 (-42,000 to -4,000) | -18,000 (-54,000 to 13,000) |
|  | Women | -15,000 (-33,000 to -26) | -14,000 (-33,000 to 540) | -16,000 (-37,000 to -950) |
| * Negative number of deaths prevented or postponed for specific causes of death is a direct consequence of the mortality competing risk framework we implemented in the model. They represent synthetic individuals that the prevention of their death from a specific disease (i.e. CHD) due to the policy led to their death from another competing cause (i.e. non-CVD) in the same year. | | | | |

Table H Health related model estimates, impact Inventory and cost-effectiveness analysis over the 20-year simulation period from 2017 to 2036, for US adults age 30 to 84 years by age group. Values are the median estimate (95% UI). Results are rounded to the first digit for sodium, first decimal for SBP, and second significant digit for other outcomes. Negative costs represent savings. Policy costs were divided proportionally to population size for each age group. Costs are presented in discounted 2017 Billion US Dollars. CHD, coronary heart disease; CVD, cardiovascular disease; QALY, quality adjusted life years; SBP, systolic blood pressure; UI, uncertainty intervals.

| Model outputs | Age group | Optimal policy scenario | Modest policy scenario | Pessimistic policy scenario |
| --- | --- | --- | --- | --- |
| Median sodium consumption in 2036 (mg/d) | 30–49 | 2,430 (2,413 to 2,444) | 2,730 (2,690 to 2,774) | 3,038 (3,018 to 3,056) |
|  | 50–69 | 2,220 (2,208 to 2,232) | 2,514 (2,475 to 2,556) | 2,782 (2,766 to 2,797) |
|  | 70–84 | 1,903 (1,887 to 1,920) | 2,165 (2,125 to 2,211) | 2,395 (2,375 to 2,417) |
| Median SBP in 2036 (mmHg) | 30–49 | 112.6 (112.4 to 112.8) | 113.1 (112.9 to 113.3) | 113.4 (113.2 to 113.7) |
|  | 50–69 | 114.2 (114.0 to 114.5) | 114.9 (114.6 to 115.1) | 115.4 (115.2 to 115.7) |
|  | 70–84 | 117.0 (116.5 to 117.4) | 117.7 (117.3 to 118.1) | 118.3 (117.9 to 118.7) |
| CVD cases prevented or postponed | 30–49 | 80,000 (32,000 to 170,000) | 45,000 (13,000 to 91,000) | 26,000 (3,700 to 59,000) |
|  | 50–69 | 210,000 (110,000 to 360,000) | 100,000 (48,000 to 180,000) | 59,000 (20,000 to 110,000) |
|  | 70–84 | 160,000 (87,000 to 240,000) | 71,000 (30,000 to 120,000) | 30,000 (0 to 67,000) |
| CVD deaths prevented or postponed | 30–49 | 1,900 (-1,900 to 9,300)* | 1,900 (-1,900 to 7,400)* | 0 (-3,700 to 5,600)* |
|  | 50–69 | 13,000 (0 to 32,000) | 7,400 (-5,600 to 20,000)* | 5,600 (-5,600 to 19,000)* |
|  | 70–84 | 19,000 (-5,600 to 46,000)* | 9,300 (-15,000 to 33,000)* | 7,400 (-17,000 to 30,000)* |
| Non-CVD deaths prevented or postponed | 30–49 | 3,700 (0 to 13,000) | 1,900 (-1,900 to 9,300)* | 1,900 (-3,700 to 7,400)* |
|  | 50–69 | 19,000 (3,700 to 35,000) | 9,300 (-3,700 to 24,000)* | 3,700 (-9,200 to 19,000)* |
|  | 70–84 | 24,000 (-1,900 to 54,000)* | 11,000 (-13,000 to 37,000)* | 1,900 (-20,000 to 26,000)* |
| All deaths prevented or postponed | 30–49 | 7,400 (0 to 17,000) | 3,700 (0 to 13,000) | 3,700 (0 to 9,200) |
|  | 50–69 | 33,000 (15,000 to 54,000) | 17,000 (1,900 to 34,000) | 9,200 (0 to 24,000) |
|  | 70–84 | 43,000 (20,000 to 71,000) | 20,000 (1,900 to 39,000) | 9,200 (0 to 26,000) |
| Life years gained | 30–49 | 160,000 (59,000 to 300,000) | 83,000 (1,900 to 190,000) | 65,000 (0 to 160,000) |
|  | 50–69 | 270,000 (130,000 to 450,000) | 120,000 (22,000 to 250,000) | 91,000 (0 to 210,000) |
|  | 70–84 | 33,000 (0 to 95,000) | 19,000 (0 to 72,000) | 13,000 (0 to 61,000) |
| Discounted QALY gained (million) | 30–49 | 0.69m (0.53m to 0.89m) | 0.42m (0.31m to 0.54m) | 0.28m (0.19m to 0.39m) |
|  | 50–69 | 0.89m (0.71m to 1.1m) | 0.47m (0.37m to 0.57m) | 0.29m (0.21m to 0.38m) |
|  | 70–84 | 0.47m (0.34m to 0.6m) | 0.23m (0.15m to 0.31m) | 0.12m (0.061m to 0.18m) |
| Change in health-related costs: | 30–49 | -11bn (-20bn to -7.2bn) | -6.6bn (-12bn to -3.3bn) | -4.4bn (-9.2bn to -1.4bn) |
|  | 50–69 | -28bn (-48bn to -17bn) | -14bn (-26bn to -7.9bn) | -9.7bn (-19bn to -3.9bn) |
|  | 70–84 | -19bn (-31bn to -11bn) | -8.7bn (-16bn to -3.5bn) | -5.3bn (-11bn to -0.47bn) |
| Hypertension medical costs | 30–49 | -3.8bn (-5.5bn to -2.5bn) | -2.3bn (-3.3bn to -1.5bn) | -1.4bn (-2.2bn to -0.83bn) |
|  | 50–69 | -8.3bn (-11bn to -5.6bn) | -4.4bn (-6bn to -2.9bn) | -2.2bn (-3.3bn to -1.4bn) |
|  | 70–84 | -5.6bn (-8bn to -3.8bn) | -2.7bn (-3.8bn to -1.7bn) | -0.8bn (-1.4bn to -0.36bn) |
| Hypertension productivity costs | 30–49 | -3.8bn (-5.5bn to -2.5bn) | -2.3bn (-3.4bn to -1.5bn) | -1.5bn (-2.4bn to -0.9bn) |
|  | 50–69 | -5.7bn (-7.9bn to -3.9bn) | -3.1bn (-4.4bn to -2.1bn) | -1.7bn (-2.5bn to -1.1bn) |
|  | 70–84 | -2bn (-2.8bn to -1.3bn) | -0.93bn (-1.4bn to -0.6bn) | -0.26bn (-0.47bn to -0.09bn) |
| CHD medical costs | 30–49 | -0.55bn (-1.5bn to -0.11bn) | -0.3bn (-0.9bn to -0.034bn) | -0.22bn (-0.71bn to 0.0bn) |
|  | 50–69 | -3.1bn (-7.6bn to -0.93bn) | -1.5bn (-3.9bn to -0.37bn) | -1.3bn (-3.2bn to -0.24bn) |
|  | 70–84 | -3.4bn (-7.8bn to -1.2bn) | -1.5bn (-3.7bn to -0.34bn) | -1.2bn (-3.2bn to -0.14bn) |
| CHD mortality productivity costs | 30–49 | -0.58bn (-6.6bn to 1.3bn) | -0.061bn (-4.7bn to 1.8bn) | 0bn (-3.9bn to 2.1bn) |
|  | 50–69 | -2.5bn (-14bn to 1.7bn) | -1.2bn (-8.1bn to 2.7bn) | -1.1bn (-7.5bn to 3.2bn) |
|  | 70–84 | -1.3bn (-6bn to 1.7bn) | -0.56bn (-4.8bn to 2.8bn) | -0.38bn (-4.1bn to 3bn) |
| CHD morbidity productivity costs | 30–49 | -0.41bn (-1.2bn to -0.076bn) | -0.22bn (-0.7bn to -0.02bn) | -0.16bn (-0.55bn to 0.0bn) |
|  | 50–69 | -0.89bn (-2.2bn to -0.25bn) | -0.42bn (-1.1bn to -0.083bn) | -0.34bn (-0.88bn to -0.047bn) |
|  | 70–84 | -0.000000007bn  (-0.000000046bn to 0.0bn) | -0.000000003bn  (-0.000000021bn to 0.0bn) | -0.000000002bn  (-0.000000016bn to 0bn) |
| CHD informal care costs | 30–49 | -0.24bn (-0.73bn to -0.043bn) | -0.12bn (-0.41bn to -0.012bn) | -0.092bn (-0.31bn to 0.0bn) |
|  | 50–69 | -0.73bn (-1.7bn to -0.23bn) | -0.34bn (-0.89bn to -0.073bn) | -0.29bn (-0.74bn to -0.056bn) |
|  | 70–84 | -0.53bn (-1.2bn to -0.18bn) | -0.22bn (-0.55bn to -0.039bn) | -0.19bn (-0.48bn to -0.014bn) |
| Stroke medical costs | 30–49 | -0.3bn (-0.85bn to -0.058bn) | -0.18bn (-0.55bn to -0.0066bn) | -0.15bn (-0.46bn to 0.0bn) |
|  | 50–69 | -2.2bn (-5.2bn to -0.75bn) | -1.2bn (-3bn to -0.3bn) | -1bn (-2.6bn to -0.23bn) |
|  | 70–84 | -2.9bn (-6.9bn to -0.92bn) | -1.4bn (-3.7bn to -0.28bn) | -1.2bn (-3bn to -0.17bn) |
| Stroke mortality productivity costs | 30–49 | 0bn (-3bn to 0.71bn) | 0bn (-2.3bn to 1.4bn) | 0bn (-2bn to 1.4bn) |
|  | 50–69 | -1.1bn (-6.5bn to 1.3bn) | -0.6bn (-4.6bn to 1.8bn) | -0.49bn (-4.6bn to 1.9bn) |
|  | 70–84 | -0.87bn (-3.9bn to 1.6bn) | -0.42bn (-3.1bn to 1.9bn) | -0.31bn (-2.9bn to 2.2bn) |
| Stroke morbidity productivity costs | 30–49 | -0.22bn (-0.62bn to -0.038bn) | -0.12bn (-0.39bn to 0.0021bn) | -0.096bn (-0.33bn to 0.0bn) |
|  | 50–69 | -0.54bn (-1.3bn to -0.16bn) | -0.28bn (-0.74bn to -0.051bn) | -0.23bn (-0.63bn to -0.026bn) |
|  | 70–84 | -0.000000005bn  (-0.000000039bn to 0.0bn) | -0.000000002bn  (-0.000000018bn to 0.0bn) | -0.000000002bn  (-0.000000015bn to 0.0bn) |
| Stroke informal care costs | 30–49 | -0.37bn (-1.2bn to -0.052bn) | -0.2bn (-0.79bn to 0.0041bn) | -0.16bn (-0.67bn to 0.0bn) |
|  | 50–69 | -1.4bn (-3.9bn to -0.38bn) | -0.69bn (-2.1bn to -0.13bn) | -0.58bn (-1.8bn to -0.091bn) |
|  | 70–84 | -1.3bn (-3.4bn to -0.35bn) | -0.58bn (-1.7bn to -0.065bn) | -0.47bn (-1.4bn to -0.023bn) |
| Change in policy costs: | 30–49 | 7.1bn (2.7bn to 15bn) | 4.4bn (1.7bn to 9bn) | 3.2bn (1.2bn to 6.5bn) |
|  | 50–69 | 6.8bn (2.6bn to 14bn) | 4.2bn (1.6bn to 8.5bn) | 3.1bn (1.2bn to 6.2bn) |
|  | 70–84 | 2.7bn (1bn to 5.4bn) | 1.7bn (0.64bn to 3.4bn) | 1.1bn (0.43bn to 2.2bn) |
| Policy admin costs | 30–49 | 0.069bn (0.05bn to 0.093bn) | 0.069bn (0.05bn to 0.093bn) | 0.069bn (0.05bn to 0.093bn) |
|  | 50–69 | 0.065bn (0.048bn to 0.088bn) | 0.065bn (0.048bn to 0.088bn) | 0.065bn (0.048bn to 0.088bn) |
|  | 70–84 | 0.027bn (0.019bn to 0.036bn) | 0.027bn (0.019bn to 0.036bn) | 0.027bn (0.019bn to 0.036bn) |
| Policy monitoring costs | 30–49 | 0.012bn (0.009bn to 0.017bn) | 0.012bn (0.009bn to 0.017bn) | 0.012bn (0.009bn to 0.017bn) |
|  | 50–69 | 0.012bn (0.0084bn to 0.016bn) | 0.012bn (0.0084bn to 0.016bn) | 0.012bn (0.0084bn to 0.016bn) |
|  | 70–84 | 0.0049bn (0.0036bn to 0.0067bn) | 0.0049bn (0.0036bn to 0.0067bn) | 0.0049bn (0.0036bn to 0.0067bn) |
| Policy industry costs | 30–49 | 7.1bn (2.6bn to 15bn) | 4.3bn (1.6bn to 8.9bn) | 3.1bn (1.2bn to 6.4bn) |
|  | 50–69 | 6.7bn (2.5bn to 14bn) | 4.1bn (1.5bn to 8.5bn) | 3bn (1.1bn to 6.2bn) |
|  | 70–84 | 2.6bn (0.98bn to 5.4bn) | 1.6bn (0.61bn to 3.3bn) | 1.1bn (0.39bn to 2.2bn) |
| Total net cost (medical perspective) | 30–49 | -4.7bn (-7.1bn to -3.1bn) | -2.8bn (-4.3bn to -1.8bn) | -1.8bn (-2.9bn to -1.1bn) |
|  | 50–69 | -14bn (-22bn to -8.9bn) | -7.1bn (-11bn to -4.6bn) | -4.5bn (-7.7bn to -2.7bn) |
|  | 70–84 | -12bn (-20bn to -7.5bn) | -5.7bn (-9.8bn to -3.3bn) | -3.3bn (-6.3bn to -1.5bn) |
| Total net cost (societal perspective) | 30–49 | -4.1bn (-14bn to 4.5bn) | -2.2bn (-9.1bn to 3.9bn) | -1.1bn (-6.3bn to 3.5bn) |
|  | 50–69 | -21bn (-42bn to -7.9bn) | -9.9bn (-22bn to -2.3bn) | -6.5bn (-16bn to -0.0031bn) |
|  | 70–84 | -16bn (-28bn to -7.4bn) | -6.9bn (-15bn to -1.5bn) | -4.1bn (-10bn to 0.83bn) |
| Net monetary benefit in billion (valuing QALY at $100,000) | 30–49 | 73bn (54bn to 98bn) | 44bn (30bn to 60bn) | 29bn (17bn to 42bn) |
|  | 50–69 | 110bn (85bn to 140bn) | 57bn (42bn to 75bn) | 36bn (23bn to 51bn) |
|  | 70–84 | 63bn (45bn to 83bn) | 30bn (20bn to 42bn) | 16bn (7.6bn to 26bn) |
| Incremental cost-effectiveness ratio (2017 US Dollars per QALY) | 30–49 | -5,900 (-20,000 to 7,100) | -5,200 (-20,000 to 11,000) | -4,000 (-22,000 to 15,000) |
|  | 50–69 | -23,000 (-46,000 to -9,100) | -21,000 (-46,000 to -5,100) | -23,000 (-56,000 to -10) |
|  | 70–84 | -34,000 (-60,000 to -17,000) | -31,000 (-66,000 to -6,500) | -34,000 (-100,000 to 7,300) |
| * Negative number of deaths prevented or postponed for specific causes of death is a direct consequence of the mortality competing risk framework we implemented in the model. They represent synthetic individuals that the prevention of their death from a specific disease (i.e. CHD) due to the policy led to their death from another competing cause (i.e. non-CVD) in the same year. | | | | |

Table I Health related model estimates, impact Inventory and cost-effectiveness analysis over the 20-year simulation period from 2017 to 2036, for US adults age 30 to 84 years by race/ethnicity (black for non-Hispanic black and white for non-Hispanic white). Values are the median estimate (95% UI). Results are rounded to the first digit for sodium, first decimal for SBP, and second significant digit for other outcomes. Negative costs represent savings. Policy costs were divided proportionally to population size for each race/ethnicity. Costs are presented in discounted 2017 Billion US Dollars. CHD, coronary heart disease; CVD, cardiovascular disease; QALY, quality adjusted life years; SBP, systolic blood pressure; UI, uncertainty intervals.

| Model outputs | Race/ethnicity | Optimal policy scenario | Modest policy scenario | Pessimistic policy scenario |
| --- | --- | --- | --- | --- |
| Median sodium consumption in 2036 (mg/d) | Black | 1,958 (1,927 to 1,989) | 2,276 (2,201 to 2,350) | 2,530 (2,508 to 2,552) |
|  | Other | 2,180 (2,163 to 2,196) | 2,487 (2,439 to 2,536) | 2,765 (2,745 to 2,784) |
|  | White | 2,277 (2,265 to 2,287) | 2,575 (2,545 to 2,609) | 2,846 (2,831 to 2,861) |
| Median SBP in 2036 (mmHg) | Black | 117 (116.5 to 117.6) | 117.8 (117.3 to 118.3) | 118.5 (118.1 to 118.9) |
|  | Other | 112.4 (112.2 to 112.7) | 113 (112.7 to 113.2) | 113.3 (113.1 to 113.6) |
|  | White | 114.1 (113.9 to 114.3) | 114.6 (114.4 to 114.8) | 115.1 (114.9 to 115.4) |
| CVD cases prevented or postponed | Black | 130,000 (65,000 to 220,000) | 87,000 (43,000 to 150,000) | 69,000 (33,000 to 120,000) |
|  | Other | 100,000 (50,000 to 180,000) | 54,000 (19,000 to 110,000) | 33,000 (5,600 to 71,000) |
|  | White | 220,000 (120,000 to 360,000) | 78,000 (32,000 to 140,000) | 13,000 (0 to 54,000) |
| CVD deaths prevented or postponed | Black | 11,000 (-1,900 to 28,000)* | 7,400 (-3,700 to 22,000)* | 7,400 (-3,700 to 19,000)* |
|  | Other | 5,600 (-3,700 to 19,000)* | 3,700 (-7,400 to 15,000)* | 1,900 (-7,400 to 13,000)* |
|  | White | 17,000 (-7,400 to 45,000)* | 5,600 (-17,000 to 28,000)* | 3,700 (-19,000 to 24,000)* |
| Non-CVD deaths prevented or postponed | Black | 13,000 (0 to 30,000) | 9,300 (-3,700 to 24,000)* | 5,600 (-5,600 to 19,000)* |
|  | Other | 9,300 (-1,900 to 22,000)* | 5,600 (-5,600 to 17,000)* | 1,900 (-7,400 to 13,000)* |
|  | White | 26,000 (0 to 56,000) | 9,300 (-13,000 to 33,000) | 0 (-24,000 to 22,000)* |
| All deaths prevented or postponed | Black | 26,000 (11,000 to 45,000) | 18,000 (5,600 to 34,000) | 15,000 (3,700 to 28,000) |
|  | Other | 15,000 (3,700 to 30,000) | 7,400 (0 to 20,000) | 5,600 (0 to 17,000) |
|  | White | 43,000 (20,000 to 67,000) | 15,000 (0 to 33,000) | 1,900 (0 to 29,000) |
| Life years gained | Black | 91,000 (11,000 to 200,000) | 48,000 (0 to 140,000) | 39,000 (0 to 130,000) |
|  | Other | 230,000 (95,000 to 400,000) | 80,000 (0 to 210,000) | 41,000 (0 to 160,000) |
|  | White | 150,000 (50,000 to 280,000) | 100,000 (28,000 to 210,000) | 89,000 (15,000 to 190,000) |
| Discounted QALY gained (million) | Black | 0.62m (0.47m to 0.77m) | 0.44m (0.33m to 0.56m) | 0.35m (0.25m to 0.44m) |
|  | Other | 0.57m (0.44m to 0.72m) | 0.34m (0.25m to 0.45m) | 0.25m (0.16m to 0.35m) |
|  | White | 0.85m (0.66m to 1m) | 0.33m (0.22m to 0.44m) | 0.095m (-0.0043m to 0.19m) |
| Change in health-related costs: | Black | -21bn (-37bn to -13bn) | -15bn (-25bn to -8.7bn) | -12bn (-21bn to -6.8bn) |
|  | Other | -13bn (-22bn to -7.7bn) | -6.7bn (-14bn to -2.4bn) | -4.6bn (-10bn to -0.76bn) |
|  | White | -23bn (-41bn to -14bn) | -8bn (-17bn to -2.1bn) | -2.5bn (-9.3bn to 0.0bn) |
| Hypertension medical costs | Black | -5.5bn (-7.8bn to -3.6bn) | -3.9bn (-5.6bn to -2.5bn) | -2.9bn (-4.1bn to -1.8bn) |
|  | Other | -4.8bn (-6.7bn to -3.2bn) | -2.6bn (-3.8bn to -1.7bn) | -1.5bn (-2.3bn to -0.8bn) |
|  | White | -7.4bn (-10bn to -5.1bn) | -2.8bn (-4bn to -1.8bn) | -0.14bn (-0.78bn to 0.0bn) |
| Hypertension productivity costs | Black | -5.4bn (-7.5bn to -3.6bn) | -3.9bn (-5.5bn to -2.6bn) | -2.9bn (-4.1bn to -1.9bn) |
|  | Other | -2.4bn (-3.6bn to -1.6bn) | -1.4bn (-2.1bn to -0.85bn) | -0.86bn (-1.4bn to -0.45bn) |
|  | White | -3.6bn (-5.3bn to -2.4bn) | -1.1bn (-1.7bn to -0.67bn) | 0.24bn (-0.15bn to 0.0bn) |
| CHD medical costs | Black | -2bn (-4.9bn to -0.65bn) | -1.4bn (-3.4bn to -0.39bn) | -1.3bn (-3.1bn to -0.36bn) |
|  | Other | -1.3bn (-3.3bn to -0.33bn) | -0.63bn (-1.8bn to 0.039bn) | -0.53bn (-1.6bn to 0.0bn) |
|  | White | -3.7bn (-8.8bn to -1.2bn) | -1.3bn (-3.5bn to -0.13bn) | -0.9bn (-2.8bn to 0.0bn) |
| CHD mortality productivity costs | Black | -2.5bn (-10bn to 1.1bn) | -1.7bn (-8.6bn to 1.5bn) | -1.6bn (-7.1bn to 1.4bn) |
|  | Other | -0.46bn (-5bn to 2bn) | -0.079bn (-3.4bn to 2.6bn) | -0.077bn (-3.3bn to 2.8bn) |
|  | White | -1.6bn (-13bn to 2.1bn) | -0.4bn (-6bn to 3.6bn) | -0.14bn (-5.4bn to 4.5bn) |
| CHD morbidity productivity costs | Black | -0.42bn (-1.1bn to -0.1bn) | -0.29bn (-0.77bn to -0.063bn) | -0.26bn (-0.69bn to -0.051bn) |
|  | Other | -0.37bn (-1bn to -0.072bn) | -0.2bn (-0.61bn to -0.0011bn) | -0.17bn (-0.53bn to 0.0bn) |
|  | White | -0.48bn (-1.4bn to -0.1bn) | -0.14bn (-0.53bn to 0.0bn) | -0.066bn (-0.37bn to 0.0bn) |
| CHD informal care costs | Black | -0.43bn (-1bn to -0.13bn) | -0.29bn (-0.71bn to -0.083bn) | -0.27bn (-0.65bn to -0.079bn) |
|  | Other | -0.36bn (-0.9bn to -0.088bn) | -0.18bn (-0.5bn to -0.0092bn) | -0.15bn (-0.45bn to 0.0bn) |
|  | White | -0.7bn (-1.7bn to -0.22bn) | -0.22bn (-0.63bn to 0.0bn) | -0.15bn (-0.49bn to 0.0bn) |
| Stroke medical costs | Black | -1.8bn (-4.5bn to -0.55bn) | -1.2bn (-3.2bn to -0.29bn) | -1.1bn (-2.9bn to -0.26bn) |
|  | Other | -1.3bn (-3.2bn to -0.29bn) | -0.68bn (-2bn to 0.0bn) | -0.6bn (-1.8bn to 0.0bn) |
|  | White | -2.2bn (-5.4bn to -0.71bn) | -0.83bn (-2.4bn to -0.025bn) | -0.6bn (-1.8bn to 0.0bn) |
| Stroke mortality productivity costs | Black | -1.2bn (-5.4bn to 0.99bn) | -0.79bn (-4.3bn to 1.2bn) | -0.7bn (-3.9bn to 1.2bn) |
|  | Other | -0.44bn (-3.7bn to 1.3bn) | -0.12bn (-2.6bn to 1.7bn) | -0.093bn (-2.5bn to 1.8bn) |
|  | White | -0.63bn (-4.3bn to 1.5bn) | -0.16bn (-3bn to 2.1bn) | -0.066bn (-3.1bn to 2.2bn) |
| Stroke morbidity productivity costs | Black | -0.25bn (-0.68bn to -0.065bn) | -0.17bn (-0.49bn to -0.028bn) | -0.15bn (-0.44bn to -0.022bn) |
|  | Other | -0.21bn (-0.56bn to -0.031bn) | -0.11bn (-0.37bn to 0.0bn) | -0.099bn (-0.34bn to 0.0bn) |
|  | White | -0.28bn (-0.78bn to -0.054bn) | -0.11bn (-0.36bn to 0.0bn) | -0.067bn (-0.27bn to 0.0bn) |
| Stroke informal care costs | Black | -0.84bn (-2.4bn to -0.22bn) | -0.57bn (-1.7bn to -0.12bn) | -0.53bn (-1.6bn to -0.1bn) |
|  | Other | -0.68bn (-2.1bn to -0.14bn) | -0.34bn (-1.2bn to 0.027bn) | -0.3bn (-1.1bn to 0.065bn) |
|  | White | -1.5bn (-4.2bn to -0.41bn) | -0.54bn (-1.8bn to 0.0bn) | -0.4bn (-1.3bn to 0.0bn) |
| Change in policy costs: | Black | 2bn (0.74bn to 4bn) | 1.2bn (0.47bn to 2.5bn) | 0.85bn (0.33bn to 1.7bn) |
|  | Other | 3.9bn (1.5bn to 8bn) | 2.4bn (0.93bn to 4.9bn) | 1.7bn (0.65bn to 3.4bn) |
|  | White | 11bn (4.1bn to 22bn) | 6.6bn (2.6bn to 14bn) | 4.8bn (1.9bn to 9.8bn) |
| Policy admin costs | Black | 0.019bn (0.014bn to 0.026bn) | 0.019bn (0.014bn to 0.026bn) | 0.019bn (0.014bn to 0.026bn) |
|  | Other | 0.038bn (0.028bn to 0.052bn) | 0.038bn (0.028bn to 0.052bn) | 0.038bn (0.028bn to 0.052bn) |
|  | White | 0.1bn (0.076bn to 0.14bn) | 0.1bn (0.076bn to 0.14bn) | 0.1bn (0.076bn to 0.14bn) |
| Policy monitoring costs | Black | 0.0034bn (0.0025bn to 0.0047bn) | 0.0034bn (0.0025bn to 0.0047bn) | 0.0034bn (0.0025bn to 0.0047bn) |
|  | Other | 0.007bn (0.0051bn to 0.0095bn) | 0.007bn (0.0051bn to 0.0095bn) | 0.007bn (0.0051bn to 0.0095bn) |
|  | White | 0.018bn (0.013bn to 0.025bn) | 0.018bn (0.013bn to 0.025bn) | 0.018bn (0.013bn to 0.025bn) |
| Policy industry costs | Black | 1.9bn (0.72bn to 4bn) | 1.2bn (0.44bn to 2.4bn) | 0.83bn (0.31bn to 1.7bn) |
|  | Other | 3.9bn (1.4bn to 7.9bn) | 2.4bn (0.89bn to 4.9bn) | 1.6bn (0.6bn to 3.3bn) |
|  | White | 11bn (4bn to 22bn) | 6.5bn (2.4bn to 13bn) | 4.7bn (1.8bn to 9.7bn) |
| Total net cost (medical perspective) | Black | -9.5bn (-15bn to -6bn) | -6.7bn (-11bn to -4.1bn) | -5.4bn (-8.7bn to -3.2bn) |
|  | Other | -7.5bn (-12bn to -4.9bn) | -4bn (-6.6bn to -2.4bn) | -2.7bn (-4.7bn to -1.3bn) |
|  | White | -14bn (-22bn to -8.7bn) | -5bn (-8.7bn to -2.9bn) | -1.7bn (-4bn to -0.12bn) |
| Total net cost (societal perspective) | Black | -19bn (-35bn to -11bn) | -14bn (-24bn to -7.5bn) | -11bn (-20bn to -5.9bn) |
|  | Other | -8.9bn (-19bn to -2.2bn) | -4.2bn (-11bn to 0.69bn) | -2.9bn (-8.7bn to 1.3bn) |
|  | White | -12bn (-31bn to 2.6bn) | -1.2bn (-11bn to 7.7bn) | 2.6bn (-4.8bn to 10bn) |
| Net monetary benefit in billion (valuing QALY at $100,000) | Black | 82bn (61bn to 110bn) | 58bn (42bn to 76bn) | 46bn (33bn to 61bn) |
|  | Other | 66bn (49bn to 87bn) | 38bn (27bn to 53bn) | 28bn (16bn to 41bn) |
|  | White | 98bn (72bn to 130bn) | 34bn (19bn to 51bn) | 6.9bn (-7.5bn to 21bn) |
| Incremental cost-effectiveness ratio (2017 US Dollars per QALY) | Black | -31,000 (-52,000 to -19,000) | -31,000 (-53,000 to -18,000) | -33,000 (-56,000 to -18,000) |
|  | Other | -15,000 (-33,000 to -4,200) | -12,000 (-31,000 to 2,100) | -12,000 (-35,000 to 6,400) |
|  | White | -15,000 (-36,000 to 3,100) | -3,500 (-33,000 to 26,000) | 25,000 (-200,000 to 290,000) |
| * Negative number of deaths prevented or postponed for specific causes of death is a direct consequence of the mortality competing risk framework we implemented in the model. They represent synthetic individuals that the prevention of their death from a specific disease (i.e. CHD) due to the policy led to their death from another competing cause (i.e. non-CVD) in the same year. | | | | |

## Model estimates for the baseline scenario

Table J Model estimates for the baseline scenario over the 20-year simulation period from 2017 to 2036, for US adults age 30 to 84 years. Values are the median estimate (95% UI). Results are rounded to the first digit for sodium, first decimal for SBP, and third significant digit for other outcomes. Costs are presented in discounted (3%) 2017 Billion US Dollars. CHD, coronary heart disease; CVD, cardiovascular disease (the sum of CHD and stroke cases, avoiding double counting of cases with coexisting CHD and stroke); QALY, quality adjusted life years; SBP, systolic blood pressure; UI, uncertainty intervals.

| Model outputs | Baseline scenario |
| --- | --- |
| Population (30 to 84 years) | 219,000,000 (216,000,000 to 222,000,000) |
| Median sodium consumption in 2036 (mg/d) | 2,974 (2,960 to 2,987) |
| Median SBP in 2036 (mmHg) | 114.9 (114.7 to 115.0) |
| New CHD cases (first ever episode, cumulative) | 15,000,000 (7,560,000 to 24,400,000) |
| New stroke cases (first ever episode, cumulative) | 7,910,000 (3,830,000 to 14,100,000) |
| New CVD cases (first ever episode, cumulative) | 23,000,000 (13,900,000 to 33,800,000) |
| CHD prevalence in 2036 | 13,500,000 (5,790,000 to 24,600,000) |
| Stroke prevalence in 2036 | 6,080,000 (2,620,000 to 11,600,000) |
| Hypertension prevalence in 2036 | 50,700,000 (49,400,000 to 52,000,000) |
| CVD prevalence in 2036 | 18,900,000 (10,500,000 to 29,600,000) |
| CVD or hypertension prevalence in 2036 | 61,000,000 (56,300,000 to 67,300,000) |
| CHD deaths (cumulative) | 4,460,000 (3,470,000 to 5,540,000) |
| Stroke deaths (cumulative) | 1,510,000 (1,220,000 to 1,810,000) |
| Any-other deaths (cumulative) | 31,000,000 (27,200,000 to 35,500,000) |
| Hypertension medical cost (cumulative) | 974bn (707bn to 1,320bn) |
| Hypertension productivity cost (cumulative) | 496bn (357bn to 662bn) |
| CHD medical cost (cumulative) | 1,450bn (589bn to 2,880bn) |
| CHD mortality cost (cumulative) | 818bn (512bn to 4,050bn) |
| CHD morbidity cost (cumulative) | 210bn (88.5bn to 408bn) |
| CHD informal cost (cumulative) | 325bn (139bn to 639bn) |
| Stroke medical cost (cumulative) | 617bn (269bn to 1,230bn) |
| Stroke mortality cost (cumulative) | 246bn (155bn to 999bn) |
| Stroke morbidity cost (cumulative) | 79.3bn (32.1bn to 160bn) |
| Stroke informal cost (cumulative) | 423bn (144bn to 1,050bn) |
| QALY (cumulative) | 2,620m (2,600m to 2,630m) |
| QALY lost due to hypertension (cumulative) | 703m (693m to 713m) |
| QALY lost due to CHD (cumulative) | 146m (64.5m to 262m) |
| QALY lost due to stroke (cumulative) | 63m (28m to 117m) |
| QALY lost due to CVD (cumulative) | 202m (114m to 314m) |
| QALY lost due to CVD or hypertension (cumulative) | 794m (755m to 848m) |

**Results from the extra scenario (not comparable to main results)**

Table K Health related model estimates, impact Inventory and cost-effectiveness analysis for the 7.5% reformulation scenario over the 20-year simulation period from 2017 to 2036, for US adults age 30 to 84 years. Values are the median estimate (95% UI). Results are rounded to the first digit for sodium, first decimal for SBP, and second significant digit for other outcomes. Negative costs represent savings. Costs are presented in discounted 2017 Billion US Dollars. CHD, coronary heart disease; CVD, cardiovascular disease; QALY, quality adjusted life years; SBP, systolic blood pressure; UI, uncertainty intervals.

| Model outputs | 7.5% reformulation policy scenario. Not directly comparable with results for other scenarios |
| --- | --- |
| Median sodium consumption in 2036 (mg/d) | 3,085 (95% UI: 3,060 to 3,109) |
| Median SBP in 2036 (mmHg) | 115.6 (95% UI: 115.4 to 115.8) |
| CVD cases prevented or postponed | 100,000 (95% UI: 48,000 to 180,000) |
| CVD deaths prevented or postponed | 7,400 (95% UI: 0 to 33,000) |
| Non-CVD deaths prevented or postponed | 11,000 (95% UI: 0 to 35,000) |
| Life years gained | 110,000 (95% UI: 19,000 to 230,000) |
| Discounted QALY gained (million) | 0.4m (95% UI: 0.32m to 0.49m) |
| Change in health-related costs: | -12bn (95% UI: -24bn to -5.3bn) |
| Hypertension medical costs | -3.6bn (95% UI: -5bn to -2.5bn) |
| Hypertension productivity costs | -2.3bn (95% UI: -3.2bn to -1.6bn) |
| CHD medical costs | -1.6bn (95% UI: -3.9bn to -0.34bn) |
| CHD mortality productivity costs | -1.2bn (95% UI: -10bn to 3.8bn) |
| CHD morbidity productivity costs | -0.27bn (95% UI: -0.81bn to -0.029bn) |
| CHD informal care costs | -0.34bn (95% UI: -0.83bn to -0.077bn) |
| Stroke medical costs | -1.1bn (95% UI: -2.8bn to -0.24bn) |
| Stroke mortality productivity costs | -0.49bn (95% UI: -4.2bn to 2.6bn) |
| Stroke morbidity productivity costs | -0.14bn (95% UI: -0.47bn to 0.000017bn) |
| Stroke informal care costs | -0.64bn (95% UI: -2.0bn to -0.12bn) |
| Change in policy costs: | 2bn (95% UI: 0.84bn to 3.8bn) |
| Policy admin costs | 0.16bn (95% UI: 0.12bn to 0.22bn) |
| Policy monitoring costs | 0.029bn (95% UI: 0.021bn to 0.039bn) |
| Policy industry costs | 1.8bn (95% UI: 0.66bn to 3.6bn) |
| Total net cost (medical perspective) | -6.4bn (95% UI: -11bn to -4bn) |
| Total net cost (societal perspective) | -10bn (95% UI: -22bn to -2.8bn) |
| Net monetary benefit in billion (valuing QALY at $100,000) | 50bn (95% UI: 38bn to 67bn) |
| Incremental cost-effectiveness ratio (2017 US Dollars per QALY) | -25,000 (95% UI: -52,000 to -7,400) |

**References**

1. Strazzullo P, D’Elia L, Kandala N-B, Cappuccio FP. Salt intake, stroke, and cardiovascular disease: meta-analysis of prospective studies. BMJ 2009;339:b4567.

2. He FJ, Li J, MacGregor GA. Effect of longer-term modest salt reduction on blood pressure [Internet]. In: Cochrane Database of Systematic Reviews. John Wiley & Sons, Ltd; 2013 [cited 2015 May 27]. Available from: http://onlinelibrary.wiley.com/doi/10.1002/14651858.CD004937.pub2/abstract

3. Mozaffarian D, Fahimi S, Singh GM, et al. Global sodium consumption and death from cardiovascular causes. N Engl J Med 2014;371(7):624–34.

4. World Health Organisation. Guideline: Sodium Intake for Adults and Children [Internet]. Geneva: World Health Organisation; 2012 [cited 2016 Jul 17]. Available from: http://www.ncbi.nlm.nih.gov/books/NBK133309/

5. U.S. Department of Health and Human Services, U.S. Department of Agriculture. Dietary guidelines for Americans 2015-2020. Eighth edition [Internet]. 2015 [cited 2017 May 31];Available from: https://health.gov/dietaryguidelines/2015/resources/2015-2020_Dietary_Guidelines.pdf

6. Ioannidis JP. Commentary: Salt and the assault of opinion on evidence. Int J Epidemiol 2016;45(1):264–5.

7. Mente A, O’Donnell M, Rangarajan S, et al. Associations of urinary sodium excretion with cardiovascular events in individuals with and without hypertension: a pooled analysis of data from four studies. The Lancet 2016;388:465–75.

8. O’Donnell M, Mente A, Rangarajan S, et al. Urinary sodium and potassium excretion, mortality, and cardiovascular events. N Engl J Med 2014;371(7):612–23.

9. Cogswell ME, Mugavero K, Bowman BA, Frieden TR. Dietary sodium and cardiovascular disease risk — measurement matters. N Engl J Med 2016;375:580--586.

10. Campbell NRC. Dissidents and dietary sodium: concerns about the commentary by O’Donnell et al. Int J Epidemiol 2016;dyw292.

11. Taylor R, Najafi F, Dobson A. Meta-analysis of studies of passive smoking and lung cancer: effects of study type and continent. Int J Epidemiol 2007;36(5):1048–59.

12. Aburto NJ, Ziolkovska A, Hooper L, Elliott P, Cappuccio FP, Meerpohl JJ. Effect of lower sodium intake on health: systematic review and meta-analyses. BMJ 2013;346:f1326.

13. Lawes CMM, Hoorn SV, Law MR, Elliott P, MacMahon S, Rodgers A. Comparative quantification of health risks. Chapter 6: High blood pressure [Internet]. Geneva: World Health Organisation; 2004. Available from: http://www.who.int/publications/cra/en/

14. Williamson P. The role of the International Journal of Microsimulation. Int J Microsimulation 2007;1(1):1–2.

15. Zucchelli E, Jones AM, Rice N. The evaluation of health policies through dynamic microsimulation methods. Int J Microsimulation 2012;5(1):2–20.

16. R Core Team. R: a language and environment for statistical computing [Internet]. R Foundation for Statistical Computing; 2014. Available from: http://www.R-project.org/

17. Dowle M, Short T, Lianoglou S, Srinivasan A. data.table: Extension of data.frame [Internet]. 2015. Available from: https://github.com/Rdatatable/data.table/

18. Revolution Analytics, Weston S. foreach: Foreach looping construct for R [Internet]. 2014. Available from: http://CRAN.R-project.org/package=foreach

19. Gaujoux R. doRNG: Generic Reproducible Parallel Backend for foreach Loops [Internet]. 2014. Available from: http://CRAN.R-project.org/package=doRNG

20. L’Ecuyer P. Good Parameters and Implementations for Combined Multiple Recursive Random Number Generators. Oper Res 1999;47(1):159–64.

21. Alfons A, Kraft S, Templ M, Filzmoser P. Simulation of close-to-reality population data for household surveys with application to EU-SILC. Stat Methods Appl 2011;20(3):383–407.

22. Kypridemos C. Modelling the effectiveness and equity of primary prevention policies in England: a stochastic dynamic microsimulation for the joint prevention of non communicable diseases [Internet]. 2016 [cited 2017 Apr 11];Available from: https://elements.liverpool.ac.uk/repository.html?pub=0&com=get-file&rfurl=http%3A%2F%2Flivrepository.liverpool.ac.uk%2Frt4eprints%2Ffile%2F87606%2F201001644_Oct2016.pdf

23. Centers for Disease Control and Prevention (CDC), National Center for Health Statistics (NCHS). National Health and Nutrition Examination Survey data. Hyattsville, MD: U.S. Department of Health and Human Services, Centers for Disease Control and Prevention [Internet]. 1999 [cited 2016 Nov 15];Available from: https://wwwn.cdc.gov/nchs/nhanes/ContinuousNhanes/

24. R Core Team. R: a language and environment for statistical computing [Internet]. R Foundation for Statistical Computing; 2017. Available from: http://www.R-project.org/

25. Meindl B, Templ M, Alfons A, Kowarik A. simPop: simulation of synthetic populations for survey data considering auxiliary information [Internet]. 2017. Available from: https://CRAN.R-project.org/package=simPop

26. Rhodes DG, Murayi T, Clemens JC, Baer DJ, Sebastian RS, Moshfegh AJ. The USDA Automated Multiple-Pass Method accurately assesses population sodium intakes. Am J Clin Nutr 2013;97(5):958–64.

27. U.S. Census Bureau. National population projections: United States by age, gender, ethnicity and race for years 2014–2060, released by the U.S. Census Bureau on December 10, 2014, on CDC WONDER on-line database [Internet]. 2015 [cited 2017 Feb 11];Available from: https://wonder.cdc.gov/population-projections-2014-2060.html

28. Capewell S, O’Flaherty M. Rapid mortality falls after risk-factor changes in populations. The Lancet 2011;378(9793):752–3.

29. Capewell S, O’Flaherty M. Can dietary changes rapidly decrease cardiovascular mortality rates? Eur Heart J 2011;32(10):1187–9.

30. Levin ML. The occurrence of lung cancer in man. Acta - Unio Int Contra Cancrum 1953;9(3):531–41.

31. Smolina K, Wright FL, Rayner M, Goldacre MJ. Determinants of the decline in mortality from acute myocardial infarction in England between 2002 and 2010: linked national database study. BMJ 2012;344:d8059.

32. Young F, Capewell S, Ford ES, Critchley JA. Coronary mortality declines in the U.S. between 1980 and 2000. Am J Prev Med 2010;39(3):228–34.

33. Unal B, Critchley JA, Capewell S. Explaining the decline in coronary heart disease mortality in England and Wales between 1981 and 2000. Circulation 2004;109(9):1101–7.

34. Ford ES, Ajani UA, Croft JB, et al. Explaining the decrease in U.S. deaths from coronary disease, 1980-2000. N Engl J Med 2007;356(23):2388–98.

35. Benjamin EJ, Blaha MJ, Chiuve SE, et al. Heart Disease and Stroke Statistics—2017 Update: A Report From the American Heart Association. Circulation 2017;135(10):e146–603.

36. Ford ES, Roger VL, Dunlay SM, Go AS, Rosamond WD. Challenges of Ascertaining National Trends in the Incidence of Coronary Heart Disease in the United States. J Am Heart Assoc 2014;3(6):e001097.

37. United States Department of Health and Human Services (US DHHS), Centers for Disease Control and Prevention (CDC), National Center for Health Statistics (NCHS). Underlying cause of death 1999–2015 on CDC WONDER online database. Data are compiled from data provided by the 57 vital statistics jurisdictions through the Vital Statistics Cooperative Program [Internet]. 2016 [cited 2017 Apr 18];Available from: https://wonder.cdc.gov/ucd-icd10.html

38. Wilson PWF, D’Agostino RB, Levy D, Belanger AM, Silbershatz H, Kannel WB. Prediction of coronary heart disease using risk factor categories. Circulation 1998;97(18):1837–47.

39. Barendregt JJ, van Oortmarssen GJ, Vos T, Murray CJ. A generic model for the assessment of disease epidemiology: the computational basis of DisMod II. Popul Health Metr 2003;1:4.

40. Lim SS, Vos T, Flaxman AD, et al. A comparative risk assessment of burden of disease and injury attributable to 67 risk factors and risk factor clusters in 21 regions, 1990–2010: a systematic analysis for the Global Burden of Disease Study 2010. The Lancet 2012;380(9859):2224–60.

41. Boshuizen HC, Lhachimi SK, Baal PHM van, et al. The DYNAMO-HIA Model: An Efficient Implementation of a Risk Factor/Chronic Disease Markov Model for Use in Health Impact Assessment (HIA). Demography 2012;49(4):1259–83.

42. Hyndman RJ. demography: Forecasting mortality, fertility, migration and population data [Internet]. 2017. Available from: http://CRAN.R-project.org/package=demography

43. Hyndman RJ, Shahid Ullah M. Robust forecasting of mortality and fertility rates: A functional data approach. Comput Stat Data Anal 2007;51(10):4942–56.

44. Hyndman RJ, Booth H, Yasmeen F. Coherent mortality forecasting: the product-ratio method with functional time series models. Demography 2013;50(1):261–83.

45. Stringhini S, Carmeli C, Jokela M, et al. Socioeconomic status and the 25 × 25 risk factors as determinants of premature mortality: a multicohort study and meta-analysis of 1·7 million men and women. The Lancet 2017;389(10075):1229–37.

46. Sullivan PW, Ghushchyan V. Preference-Based EQ-5D Index Scores for Chronic Conditions in the United States. Med Decis Making 2006;26(4):410–20.

47. Khavjou O, Phelps D, Leib A. Projections of cardiovascular disease prevalence and costs: 2015–2035. Technical report [Internet]. RTI International; 2016 [cited 2017 Jul 10]. Available from: https://www.heart.org/idc/groups/heart-public/@wcm/@adv/documents/downloadable/ucm_491513.pdf

48. Joo H, Dunet DO, Fang J, Wang G. Cost of informal caregiving associated with stroke among the elderly in the United States. Neurology 2014;83(20):1831–7.

49. Leal J, Luengo-Fernández R, Gray A, Petersen S, Rayner M. Economic burden of cardiovascular diseases in the enlarged European Union. Eur Heart J 2006;27(13):1610–9.

50. New York City Health Department. National Salt Reduction Initiative (NSRI) [Internet]. 2016 [cited 2017 Jul 17];Available from: https://www1.nyc.gov/site/doh/health/health-topics/national-salt-reduction-initiative.page

51. Food and Drug Administration (FDA), Department of Health and Human Services (DHHS). Food and Drug Administration justification of estimates for appropriations committees. Fiscal year 2012 [Internet]. Food and Drug Administration (FDA); 2012 [cited 2017 Jul 10]. Available from: https://www.fda.gov/downloads/AboutFDA/ReportsManualsForms/Reports/BudgetReports/UCM243370.pdf

52. Collins M, Mason H, O’Flaherty M, Guzman-Castillo M, Critchley J, Capewell S. An economic evaluation of salt reduction policies to reduce coronary heart disease in England: a policy modeling study. Value Health J Int Soc Pharmacoeconomics Outcomes Res 2014;17(5):517–24.

53. Mary K. Muth, Samantha Bradley, Jenna Brophy, et al. Reformulation Cost Model. Contract No. HHSF-223-2011-10005B, Task Order 20. 2015;

54. Food and Drug Administration (FDA). Draft guidance for industry: voluntary sodium reduction goals: target mean and upper bound concentrations for sodium in commercially processed, packaged, and prepared foods [Internet]. 2016 [cited 2017 Jul 15];Available from: https://www.fda.gov/downloads/Food/GuidanceRegulation/GuidanceDocumentsRegulatoryInformation/UCM503798.pdf

55. Food and Drug Administration (FDA). FNDDS Mapping File Request 082516 [Internet]. 2014 [cited 2017 Jul 15];Available from: https://www.regulations.gov/document?D=FDA-2014-D-0055-0410

56. U.S. Department of Agriculture, Agricultural Research Service. USDA Food and Nutrient Database for Dietary Studies, 5.0 [Internet]. 2012 [cited 2017 Jul 15];Available from: https://www.ars.usda.gov/northeast-area/beltsville-md/beltsville-human-nutrition-research-center/food-surveys-research-group/

57. Poti JM, Dunford EK, Popkin BM. Sodium reduction in US households’ packaged food and beverage purchases, 2000 to 2014. JAMA Intern Med 2017;177(7):986–94.

58. Koerkamp BG, Stijnen T, Weinstein MC, Hunink MGM. The combined analysis of uncertainty and patient heterogeneity in medical decision models. Med Decis Making 2011;31(4):650–61.

59. Briggs AH, Weinstein MC, Fenwick EAL, Karnon J, Sculpher MJ, Paltiel AD. Model parameter estimation and uncertainty: a report of the ISPOR-SMDM modeling good research practices Task Force-6. Value Health 2012;15(6):835–42.

60. Jones AM, Lomas J, Rice N. Applying beta-type size distributions to healthcare cost regressions. J Appl Econom 2014;29(4):649–70.

61. Singh GM, Danaei G, Farzadfar F, et al. The age-specific quantitative effects of metabolic risk factors on cardiovascular diseases and diabetes: a pooled analysis. PLOS ONE 2013;8(7):e65174.

62. Neumann PJ, Cohen JT, Weinstein MC. Updating cost-effectiveness — the curious resilience of the $50,000-per-QALY threshold. N Engl J Med 2014;371(9):796–7.

63. Curtis CJ, Clapp J, Niederman SA, Ng SW, Angell SY. US food industry progress during the national salt reduction initiative: 2009–2014. Am J Public Health 2016;106(10):1815–9.

64. Food and Drug Administration (FDA). Regulatory impact analysis for final rules on: “food labeling: revision of the nutrition and supplement facts labels” docket no. FDA-2012-N-1210 and “food labeling: serving sizes of foods that can reasonably be consumed at one eating occasion; dualcolumn labeling; updating, modifying, and establishing certain reference amounts customarily consumed; serving size for breath mints; and technical amendments” docket no. FDA-2004-N-0258 (formerly docket no. 2004N-0456) [Internet]. 2014 [cited 2017 Jul 18];Available from: https://www.fda.gov/downloads/AboutFDA/ReportsManualsForms/Reports/EconomicAnalyses/UCM506797.pdf

65. He FJ, Brinsden HC, MacGregor GA. Salt reduction in the United Kingdom: a successful experiment in public health. J Hum Hypertens 2014;28(6):345–52.

66. Friendly M. Mosaic Displays for Multi-Way Contingency Tables. J Am Stat Assoc 1994;89(425):190–200.

67. United States Department of Health and Human Services (US DHHS), Centers for Disease Control and Prevention (CDC), National Center for Health Statistics (NCHS). Bridged-race population estimates 1990–2014, United States July 1st resident population by age, sex, bridged-race, and Hispanic origin, on CDC WONDER on-line database [Internet]. 2016 [cited 2017 Feb 11];Available from: https://wonder.cdc.gov/Bridged-Race-v2014.HTML

68. Micha R, Peñalvo JL, Cudhea F, Imamura F, Rehm CD, Mozaffarian D. Association between dietary factors and mortality from heart disease, stroke, and type 2 diabetes in the United States. JAMA 2017;317(9):912–24.

1. This is an extension of the US IMPACT Food policy model. To avoid confusion with previous versions of the model, in this document we will refer to it as ‘US Sodium Policy’ model. [↑](#footnote-ref-2)
2. Hispanics / non-Hispanic whites / non-Hispanic blacks / other [↑](#footnote-ref-3)
3. Less than 9th grade / 9-11th grade (Includes 12th grade with no diploma) / high school graduate/GED or equivalent / some college or AA degree / College graduate or above [↑](#footnote-ref-4)
4. Based on ratio of family income to poverty: <1.25 / 1.25 – 2 / 2 – 4 / 4+ [↑](#footnote-ref-5)
5. For this study, we assumed that the 24h recall food questionnaire of NHANES is representative of sodium consumption in the US population. An independent validation study with 24h urine collections supports this assumption.^26^ [↑](#footnote-ref-6)
6. For the percentile rank the formula $R_{percentile}= {(R-1)}/{(n-1)}$ is used, where $R_{percentile}$ is the percentile rank and $R=(R_{1},\ldots,R_{n})$ is the rank vector constructed from a random observation vector $(X_{1},\ldots,X_{n})$. In this model specifically, vector $X$ is constructed from the subset of the respective continuous risk factor values, by 5-year age group, sex and race/ethnicity, for each year of the simulation. [↑](#footnote-ref-7)
7. We used only the recent years of NHANES for two reasons: 1) there was a change in the estimation of sodium intake in NHANES since 2009 which renders older NHANES sodium estimates not immediately compatible with most recent ones; 2) Most importantly, while mean sodium intake was almost constant between years 1999 and 2008 a slow declining trend was obvious in more recent years. [↑](#footnote-ref-8)
8. Sonia Angell, personal communication, Feb 6, 2017 [↑](#footnote-ref-9)
9. We apply this equation only to synthetic individuals with sodium consumption above the optimal level of sodium consumption. Hence, the sodium consumption projection of the baseline scenario is not directly used during this calculation. Only the change in sodium consumption is important and is translated in SBP and health outcomes change. [↑](#footnote-ref-10)
10. We assume that the sales-weights are similar to the consumption-weights. [↑](#footnote-ref-11)
11. For NHANES participants we used the observed sodium consumption without the expected effect of reformulation. [↑](#footnote-ref-12)
12. We assumed log-normal distributions for relative risks and hazard ratios, normal distributions for coefficients of regression equations, generalized beta of the second kind for costs, and PERT distributions for other parameters. The cost sources, except industry reformulation costs, did not include any measures of uncertainty like standard error so an estimate of +/-20% was used for uncertainty analyses, fitted to a generalized beta of the second kind distribution, which can account for the skewness of healthcare costs.^60^ [↑](#footnote-ref-13)
13. For this study life course actually starts at the age of 30, because it is unlikely that CVD cases and deaths in younger ages can be prevented by sodium intake reduction. [↑](#footnote-ref-14)
14. Mosaic plots are graphical representations of a contingency table of two or more categorical variables, using tiles with areas proportional to the frequencies in each cell of the table.^66^ [↑](#footnote-ref-15)
