# Supplementary material for: Estimating the health and economic effects of the proposed US Food and Drug Administration voluntary sodium reformulation: Microsimulation cost-effectiveness analysis
Source: PLoS Med. 2018 Apr 10;15(4):e1002551. doi: 10.1371/journal.pmed.1002551 (PMC5892867; doi:10.1371/journal.pmed.1002551)
Supplement: S1 Table — (PDF) [file pmed.1002551.s003.pdf]

Variable	Race/ethnicity	Sex	Age	Distribution
Relative risks of relevant risk factors for coronary heart disease				
Systolic blood pressure	All	Both	30–34	Lognormal(ln(1.81), ln(2.56 / 1.81) / 1.96)
			35–44	Lognormal(ln(1.68), ln(2.20 / 1.68) / 1.96)
			45–54	Lognormal(ln(1.56), ln(1.89 / 1.56) / 1.96)
			55–64	Lognormal(ln(1.45), ln(1.62 / 1.45) / 1.96)
			65–74	Lognormal(ln(1.33), ln(1.38 / 1.33) / 1.96)
			75–84	Lognormal(ln(1.26), ln(1.28 / 1.26) / 1.96)
Relative risks of relevant risk factors for stroke				
Systolic blood pressure	All	Both	30–34	Lognormal(ln(2.30), ln(2.56 / 2.30) / 1.96)
			35–44	Lognormal(ln(2.05), ln(2.22 / 2.05) / 1.96)
			45–54	Lognormal(ln(1.83), ln(1.93 / 1.83) / 1.96)
			55–64	Lognormal(ln(1.63), ln(1.69 / 1.63) / 1.96)
			65–74	Lognormal(ln(1.44), ln(1.44 / 1.50) / 1.96)
			75–84	Lognormal(ln(1.28), ln(1.35 / 1.28) / 1.96)
Relative risks of relevant risk factors for any-other-cause mortality				
Systolic blood pressure	All	Both	30–84	Uniform(1.20, 1.40)
Effect of sodium change on systolic blood pressure				
Intercept	All	Both	30–84	Normal(-3.7, -0.7)
Age coefficient	All	Both	30–84	Normal(-0.1, 0.02)
Coefficient for hypertensives	All	Both	30–84	Normal(-1.9, 0.9)
Coefficient for blacks	Black	Both	30–84	Normal(-2.5, 1.2)
Other inputs				
CVD lag time	All	Both	30–84	1 + Binomial(9, (5-1)/9)
Proportion of CHD annual mortality change attributed to incidence change	All	Both	30–84	Uniform(0, 1)
Proportion of stroke annual mortality change attributed to incidence change	All	Both	30–84	Uniform(0, 1)
Optimal sodium consumption	All	Both	30–84	PERT(min = 614, mode = 1500, max = 2391, shape = 4)
Optimal systolic blood pressure	All	Both	30–84	Normal(Uniform(110, 115), Uniform(4, 6))
CHD medical costs				
	Black	Men	30-44	Betaprime(shape1=41.2, shape2=10100, scale=307000) * (1.0245)^(year-2014)
	Black	Men	45-64	Betaprime(shape1=41.2, shape2=6820, scale=639000) * (1.0245)^(year-2014)
	Black	Men	65-79	Betaprime(shape1=41.3, shape2=5300, scale=803000) * (1.0245)^(year-2014)
	Black	Men	80-84	Betaprime(shape1=41.5, shape2=3150, scale=601000) * (1.0245)^(year-2014)
	Black	Women	30-44	Betaprime(shape1=41.2, shape2=10700, scale=428000) * (1.0245)^(year-2014)
	Black	Women	45-64	Betaprime(shape1=41.2, shape2=6650, scale=817000) * (1.0245)^(year-2014)
	Black	Women	65-79	Betaprime(shape1=41.3, shape2=4390, scale=870000) * (1.0245)^(year-2014)
	Black	Women	80-84	Betaprime(shape1=703, shape2=44.6, scale=642) * (1.0245)^(year-2014)
	Hispanic	Men	30-44	Betaprime(shape1=41.1, shape2=14800, scale=353000) * (1.0245)^(year-2014)
	Hispanic	Men	45-64	Betaprime(shape1=41.1, shape2=8520, scale=624000) * (1.0245)^(year-2014)

Variable	Race/ethnicity	Sex	Age	Distribution
	Hispanic	Men	65-79	Betaprime(shape1=41.3, shape2=4500, scale=532000) * (1.0245)^(year-2014)
	Hispanic	Men	80-84	Betaprime(shape1=41.3, shape2=4900, scale=733000) * (1.0245)^(year-2014)
	Hispanic	Women	30-44	Betaprime(shape1=41.1, shape2=22200, scale=694000) * (1.0245)^(year-2014)
	Hispanic	Women	45-64	Betaprime(shape1=41.1, shape2=13500, scale=1290000) * (1.0245)^(year-2014)
	Hispanic	Women	65-79	Betaprime(shape1=41.3, shape2=5060, scale=783000) * (1.0245)^(year-2014)
	Hispanic	Women	80-84	Betaprime(shape1=41.4, shape2=3870, scale=756000) * (1.0245)^(year-2014)
	White	Men	30-44	Betaprime(shape1=41.1, shape2=18500, scale=513000) * (1.0245)^(year-2014)
	White	Men	45-64	Betaprime(shape1=41.2, shape2=8150, scale=693000) * (1.0245)^(year-2014)
	White	Men	65-79	Betaprime(shape1=41.2, shape2=5610, scale=770000) * (1.0245)^(year-2014)
	White	Men	80-84	Betaprime(shape1=41.4, shape2=4050, scale=702000) * (1.0245)^(year-2014)
	White	Women	30-44	Betaprime(shape1=41.1, shape2=12200, scale=446000) * (1.0245)^(year-2014)
	White	Women	45-64	Betaprime(shape1=41.2, shape2=8320, scale=928000) * (1.0245)^(year-2014)
	White	Women	65-79	Betaprime(shape1=41.3, shape2=5460, scale=984000) * (1.0245)^(year-2014)
	White	Women	80-84	Betaprime(shape1=41.3, shape2=5080, scale=1160000) * (1.0245)^(year-2014)
Stroke medical costs				
	Black	Men	30-44	Betaprime(shape1=41.1, shape2=11900, scale=372000) * (1.0245)^(year-2014)
	Black	Men	45-64	Betaprime(shape1=41.3, shape2=5060, scale=592000) * (1.0245)^(year-2014)
	Black	Men	65-79	Betaprime(shape1=659, shape2=44.8, scale=602) * (1.0245)^(year-2014)
	Black	Men	80-84	Betaprime(shape1=41.4, shape2=4090, scale=1060000) * (1.0245)^(year-2014)
	Black	Women	30-44	Betaprime(shape1=41.1, shape2=9620, scale=315000) * (1.0245)^(year-2014)
	Black	Women	45-64	Betaprime(shape1=41.3, shape2=4880, scale=601000) * (1.0245)^(year-2014)
	Black	Women	65-79	Betaprime(shape1=676, shape2=44.7, scale=618) * (1.0245)^(year-2014)
	Black	Women	80-84	Betaprime(shape1=735, shape2=44.5, scale=672) * (1.0245)^(year-2014)
	Hispanic	Men	30-44	Betaprime(shape1=41.1, shape2=14000, scale=373000) * (1.0245)^(year-2014)
	Hispanic	Men	45-64	Betaprime(shape1=41.4, shape2=5220, scale=519000) * (1.0245)^(year-2014)
	Hispanic	Men	65-79	Betaprime(shape1=611, shape2=45, scale=557) * (1.0245)^(year-2014)
	Hispanic	Men	80-84	Betaprime(shape1=41.6, shape2=2880, scale=637000) * (1.0245)^(year-2014)
	Hispanic	Women	30-44	Betaprime(shape1=41.2, shape2=9280, scale=259000) * (1.0245)^(year-2014)
	Hispanic	Women	45-64	Betaprime(shape1=41.3, shape2=5250, scale=552000) * (1.0245)^(year-2014)
	Hispanic	Women	65-79	Betaprime(shape1=41.3, shape2=5120, scale=1010000) * (1.0245)^(year-2014)
	Hispanic	Women	80-84	Betaprime(shape1=681, shape2=44.7, scale=622) * (1.0245)^(year-2014)
	White	Men	30-44	Betaprime(shape1=41.2, shape2=23000, scale=418000) * (1.0245)^(year-2014)
	White	Men	45-64	Betaprime(shape1=41.1, shape2=9400, scale=644000) * (1.0245)^(year-2014)
	White	Men	65-79	Betaprime(shape1=41.3, shape2=5200, scale=667000) * (1.0245)^(year-2014)
	White	Men	80-84	Betaprime(shape1=41.6, shape2=3300, scale=5e+05) * (1.0245)^(year-2014)
	White	Women	30-44	Betaprime(shape1=40.9, shape2=29200, scale=562000) * (1.0245)^(year-2014)
	White	Women	45-64	Betaprime(shape1=41.2, shape2=7940, scale=573000) * (1.0245)^(year-2014)
	White	Women	65-79	Betaprime(shape1=41.3, shape2=4650, scale=628000) * (1.0245)^(year-2014)
	White	Women	80-84	Betaprime(shape1=41.2, shape2=6780, scale=1090000) * (1.0245)^(year-2014)
Hypertension medical costs				
	Black	Men	30-44	Betaprime(shape1=41.2, shape2=10100, scale=307000) * (1.0245)^(year-2014)
	Black	Men	45-64	Betaprime(shape1=41.2, shape2=6820, scale=639000) * (1.0245)^(year-2014)
	Black	Men	65-79	Betaprime(shape1=41.3, shape2=5300, scale=803000) * (1.0245)^(year-2014)

Variable	Race/ethnicity	Sex	Age	Distribution
	Black	Men	80-84	Betaprime(shape1=41.5, shape2=3150, scale=601000) * (1.0245)^(year-2014)
	Black	Women	30-44	Betaprime(shape1=41.2, shape2=10700, scale=428000) * (1.0245)^(year-2014)
	Black	Women	45-64	Betaprime(shape1=41.2, shape2=6650, scale=817000) * (1.0245)^(year-2014)
	Black	Women	65-79	Betaprime(shape1=41.3, shape2=4390, scale=870000) * (1.0245)^(year-2014)
	Black	Women	80-84	Betaprime(shape1=703, shape2=44.6, scale=642) * (1.0245)^(year-2014)
	Hispanic	Men	30-44	Betaprime(shape1=41.1, shape2=14800, scale=353000) * (1.0245)^(year-2014)
	Hispanic	Men	45-64	Betaprime(shape1=41.1, shape2=8520, scale=624000) * (1.0245)^(year-2014)
	Hispanic	Men	65-79	Betaprime(shape1=41.3, shape2=4500, scale=532000) * (1.0245)^(year-2014)
	Hispanic	Men	80-84	Betaprime(shape1=41.3, shape2=4900, scale=733000) * (1.0245)^(year-2014)
	Hispanic	Women	30-44	Betaprime(shape1=41.1, shape2=22200, scale=694000) * (1.0245)^(year-2014)
	Hispanic	Women	45-64	Betaprime(shape1=41.1, shape2=13500, scale=1290000) * (1.0245)^(year-2014)
	Hispanic	Women	65-79	Betaprime(shape1=41.3, shape2=5060, scale=783000) * (1.0245)^(year-2014)
	Hispanic	Women	80-84	Betaprime(shape1=41.4, shape2=3870, scale=756000) * (1.0245)^(year-2014)
	White	Men	30-44	Betaprime(shape1=41.1, shape2=18500, scale=513000) * (1.0245)^(year-2014)
	White	Men	45-64	Betaprime(shape1=41.2, shape2=8150, scale=693000) * (1.0245)^(year-2014)
	White	Men	65-79	Betaprime(shape1=41.2, shape2=5610, scale=770000) * (1.0245)^(year-2014)
	White	Men	80-84	Betaprime(shape1=41.4, shape2=4050, scale=702000) * (1.0245)^(year-2014)
	White	Women	30-44	Betaprime(shape1=41.1, shape2=12200, scale=446000) * (1.0245)^(year-2014)
	White	Women	45-64	Betaprime(shape1=41.2, shape2=8320, scale=928000) * (1.0245)^(year-2014)
	White	Women	65-79	Betaprime(shape1=41.3, shape2=5460, scale=984000) * (1.0245)^(year-2014)
	White	Women	80-84	Betaprime(shape1=41.3, shape2=5080, scale=1160000) * (1.0245)^(year-2014)
CHD morbidity costs	All	All	30-64	Betaprime(shape1=41.3, shape2=8930, scale=485000) * (1.0129)^(year-2014)
Stroke morbidity costs	All	All	30-64	Betaprime(shape1=41.2, shape2=7300, scale=394000) * (1.0129)^(year-2014)
CHD mortality costs				
	Black	Men	30-44	Betaprime(shape1=464, shape2=1, scale=424) * (1.0129)^(year-2014)
	Black	Men	45-64	Betaprime(shape1=6090, shape2=42.3, scale=5580) * (1.0129)^(year-2014)
	Black	Men	65-79	Betaprime(shape1=3830, shape2=42.5, scale=3500) * (1.0129)^(year-2014)
	Black	Men	80-84	Betaprime(shape1=1230, shape2=43.5, scale=1310) * (1.0129)^(year-2014)
	Black	Women	30-44	Betaprime(shape1=6510, shape2=42.3, scale=5960) * (1.0129)^(year-2014)
	Black	Women	45-64	Betaprime(shape1=290, shape2=1, scale=266) * (1.0129)^(year-2014)
	Black	Women	65-79	Betaprime(shape1=3160, shape2=42.6, scale=2890) * (1.0129)^(year-2014)
	Black	Women	80-84	Betaprime(shape1=1100, shape2=43.6, scale=1000) * (1.0129)^(year-2014)
	Hispanic	Men	30-44	Betaprime(shape1=7120, shape2=42.3, scale=6520) * (1.0129)^(year-2014)
	Hispanic	Men	45-64	Betaprime(shape1=320, shape2=1, scale=293) * (1.0129)^(year-2014)
	Hispanic	Men	65-79	Betaprime(shape1=3450, shape2=42.5, scale=3160) * (1.0129)^(year-2014)
	Hispanic	Men	80-84	Betaprime(shape1=1200, shape2=43.5, scale=1090) * (1.0129)^(year-2014)
	Hispanic	Women	30-44	Betaprime(shape1=5880, shape2=42.3, scale=5380) * (1.0129)^(year-2014)
	Hispanic	Women	45-64	Betaprime(shape1=4540, shape2=42.4, scale=4150) * (1.0129)^(year-2014)
	Hispanic	Women	65-79	Betaprime(shape1=166, shape2=1, scale=152) * (1.0129)^(year-2014)
	Hispanic	Women	80-84	Betaprime(shape1=990, shape2=43.8, scale=906) * (1.0129)^(year-2014)
	White	Men	30-44	Betaprime(shape1=7310, shape2=42.3, scale=6690) * (1.0129)^(year-2014)
	White	Men	45-64	Betaprime(shape1=330, shape2=1, scale=302) * (1.0129)^(year-2014)
	White	Men	65-79	Betaprime(shape1=3550, shape2=42.5, scale=3250) * (1.0129)^(year-2014)

Variable	Race/ethnicity	Sex	Age	Distribution
	White	Men	80-84	Betaprime(shape1=1230, shape2=43.5, scale=1120) * (1.0129)^(year-2014)
	White	Women	30-44	Betaprime(shape1=6030, shape2=42.3, scale=5520) * (1.0129)^(year-2014)
	White	Women	45-64	Betaprime(shape1=4660, shape2=42.4, scale=4260) * (1.0129)^(year-2014)
	White	Women	65-79	Betaprime(shape1=171, shape2=1, scale=157) * (1.0129)^(year-2014)
	White	Women	80-84	Betaprime(shape1=1020, shape2=43.8, scale=930) * (1.0129)^(year-2014)
Stroke mortality costs				
	Black	Men	30-44	Betaprime(shape1=458, shape2=1, scale=419) * (1.0129)^(year-2014)
	Black	Men	45-64	Betaprime(shape1=5820, shape2=42.3, scale=5320) * (1.0129)^(year-2014)
	Black	Men	65-79	Betaprime(shape1=4080, shape2=42.4, scale=3740) * (1.0129)^(year-2014)
	Black	Men	80-84	Betaprime(shape1=1310, shape2=43.4, scale=1400) * (1.0129)^(year-2014)
	Black	Women	30-44	Betaprime(shape1=6340, shape2=42.3, scale=5800) * (1.0129)^(year-2014)
	Black	Women	45-64	Betaprime(shape1=277, shape2=1, scale=254) * (1.0129)^(year-2014)
	Black	Women	65-79	Betaprime(shape1=3320, shape2=42.5, scale=3040) * (1.0129)^(year-2014)
	Black	Women	80-84	Betaprime(shape1=41.7, shape2=2430, scale=1660000) * (1.0129)^(year-2014)
	Hispanic	Men	30-44	Betaprime(shape1=434, shape2=1, scale=397) * (1.0129)^(year-2014)
	Hispanic	Men	45-64	Betaprime(shape1=321, shape2=1, scale=294) * (1.0129)^(year-2014)
	Hispanic	Men	65-79	Betaprime(shape1=3870, shape2=42.5, scale=3540) * (1.0129)^(year-2014)
	Hispanic	Men	80-84	Betaprime(shape1=1240, shape2=43.4, scale=1320) * (1.0129)^(year-2014)
	Hispanic	Women	30-44	Betaprime(shape1=6000, shape2=42.3, scale=5490) * (1.0129)^(year-2014)
	Hispanic	Women	45-64	Betaprime(shape1=4480, shape2=42.4, scale=4100) * (1.0129)^(year-2014)
	Hispanic	Women	65-79	Betaprime(shape1=3150, shape2=42.6, scale=2880) * (1.0129)^(year-2014)
	Hispanic	Women	80-84	Betaprime(shape1=1090, shape2=43.6, scale=998) * (1.0129)^(year-2014)
	White	Men	30-44	Betaprime(shape1=6910, shape2=42.3, scale=6320) * (1.0129)^(year-2014)
	White	Men	45-64	Betaprime(shape1=298, shape2=1, scale=273) * (1.0129)^(year-2014)
	White	Men	65-79	Betaprime(shape1=3620, shape2=42.5, scale=3310) * (1.0129)^(year-2014)
	White	Men	80-84	Betaprime(shape1=1160, shape2=43.5, scale=1240) * (1.0129)^(year-2014)
	White	Women	30-44	Betaprime(shape1=328, shape2=1, scale=300) * (1.0129)^(year-2014)
	White	Women	45-64	Betaprime(shape1=4190, shape2=42.4, scale=3830) * (1.0129)^(year-2014)
	White	Women	65-79	Betaprime(shape1=172, shape2=1, scale=157) * (1.0129)^(year-2014)
	White	Women	80-84	Betaprime(shape1=41.5, shape2=3180, scale=1710000) * (1.0129)^(year-2014)
Hypertension indirect costs				
	Black	Men	30-44	Betaprime(shape1=41, shape2=32400, scale=511000) * (1.0129)^(year-2014)
	Black	Men	45-64	Betaprime(shape1=41.1, shape2=14400, scale=453000) * (1.0129)^(year-2014)
	Black	Men	65-79	Betaprime(shape1=41.3, shape2=15800, scale=451000) * (1.0129)^(year-2014)
	Black	Men	80-84	Betaprime(shape1=41.2, shape2=20000, scale=418000) * (1.0129)^(year-2014)
	Black	Women	30-44	Betaprime(shape1=41, shape2=51100, scale=394000) * (1.0129)^(year-2014)
	Black	Women	45-64	Betaprime(shape1=41, shape2=17900, scale=276000) * (1.0129)^(year-2014)
	Black	Women	65-79	Betaprime(shape1=41.1, shape2=25500, scale=357000) * (1.0129)^(year-2014)
	Black	Women	80-84	Betaprime(shape1=41, shape2=30400, scale=312000) * (1.0129)^(year-2014)
	Hispanic	Men	30-44	Betaprime(shape1=41, shape2=47200, scale=330000) * (1.0129)^(year-2014)
	Hispanic	Men	45-64	Betaprime(shape1=41.1, shape2=12900, scale=181000) * (1.0129)^(year-2014)
	Hispanic	Men	65-79	Betaprime(shape1=41, shape2=30500, scale=388000) * (1.0129)^(year-2014)
	Hispanic	Men	80-84	Betaprime(shape1=41, shape2=35800, scale=332000) * (1.0129)^(year-2014)

Variable	Race/ethnicity	Sex	Age	Distribution
	Hispanic	Women	30-44	Betaprime(shape1=41, shape2=153000, scale=523000) * (1.0129)^(year-2014)
	Hispanic	Women	45-64	Betaprime(shape1=41, shape2=33600, scale=230000) * (1.0129)^(year-2014)
	Hispanic	Women	65-79	Betaprime(shape1=41, shape2=39500, scale=246000) * (1.0129)^(year-2014)
	Hispanic	Women	80-84	Betaprime(shape1=41, shape2=50200, scale=228000) * (1.0129)^(year-2014)
	White	Men	30-44	Betaprime(shape1=41, shape2=28100, scale=217000) * (1.0129)^(year-2014)
	White	Men	45-64	Betaprime(shape1=41, shape2=20200, scale=314000) * (1.0129)^(year-2014)
	White	Men	65-79	Betaprime(shape1=41, shape2=20400, scale=287000) * (1.0129)^(year-2014)
	White	Men	80-84	Betaprime(shape1=41, shape2=25500, scale=262000) * (1.0129)^(year-2014)
	White	Women	30-44	Betaprime(shape1=41, shape2=96300, scale=365000) * (1.0129)^(year-2014)
	White	Women	45-64	Betaprime(shape1=41, shape2=54000, scale=410000) * (1.0129)^(year-2014)
	White	Women	65-79	Betaprime(shape1=41.1, shape2=65800, scale=454000) * (1.0129)^(year-2014)
	White	Women	80-84	Betaprime(shape1=41, shape2=77100, scale=389000) * (1.0129)^(year-2014)
CHD informal costs	All	All	30-84	Betaprime(shape1=41.1, shape2=16500, scale=652000)
Stroke informal costs	All	All	30-84	Betaprime(shape1=9.08, shape2=2550, scale=1380000) * (1)^(year-2014)
Administration costs for modest scenario years 2017-2026				1000000 * Betaprime(shape1=41.1, shape2=161000, scale=72900)
Industry costs for modest scenario years 2017-2018				1000000 * Betaprime(shape1=5.71, shape2=715, scale=283000)
Industry costs for modest scenario years 2019-2026				1000000 * Betaprime(shape1=5.69, shape2=1200, scale=196000)
Monitor costs for modest scenario years 2019-2026				1000000 * Betaprime(shape1=118, shape2=64, scale=2.28)
Administration costs for optimal scenario years 2017-2026				1000000 * Betaprime(shape1=41.1, shape2=161000, scale=72900)
Industry costs for optimal scenario years 2017-2018				1000000 * Betaprime(shape1=5.7, shape2=763, scale=519000)
Industry costs for optimal scenario years 2019-2026				1000000 * Betaprime(shape1=5.68, shape2=1540, scale=394000)
Monitor costs for optimal scenario years 2019-2026				1000000 * Betaprime(shape1=118, shape2=64, scale=2.28)
Administration costs for pessimistic scenario years 2017-2026				1000000 * Betaprime(shape1=41.1, shape2=161000, scale=72900)
Industry costs for pessimistic scenario years 2017-2018				1000000 * Betaprime(shape1=5.7, shape2=763, scale=519000)
Industry costs for pessimistic scenario years 2018-2026				1000000 * Betaprime(shape1=5.7, shape2=763, scale=519000)
CHD incidence rate for 2014				
	White	Men	30	Beta(8.89, 10000)
	White	Men	31	Beta(9.12, 9300)
	White	Men	32	Beta(9.12, 8440)
	White	Men	33	Beta(9.12, 7670)
	White	Men	34	Beta(8.99, 6890)
	White	Men	35	Beta(9.12, 6400)
	White	Men	36	Beta(9.12, 5870)
	White	Men	37	Beta(9.12, 5390)
	White	Men	38	Beta(9.1, 4950)
	White	Men	39	Beta(9.1, 4560)
	White	Men	40	Beta(9.12, 4210)
	White	Men	41	Beta(9.11, 3890)

Variable	Race/ethnicity	Sex	Age	Distribution
	White	Men	42	Beta(9.11, 3610)
	White	Men	43	Beta(9.11, 3350)
	White	Men	44	Beta(9.11, 3110)
	White	Men	45	Beta(9.11, 2900)
	White	Men	46	Beta(9.11, 2700)
	White	Men	47	Beta(9.08, 2520)
	White	Men	48	Beta(9.11, 2360)
	White	Men	49	Beta(8.92, 2170)
	White	Men	50	Beta(9.1, 2080)
	White	Men	51	Beta(9.1, 1950)
	White	Men	52	Beta(8.93, 1800)
	White	Men	53	Beta(9.1, 1730)
	White	Men	54	Beta(9.1, 1640)
	White	Men	55	Beta(9.09, 1550)
	White	Men	56	Beta(9.08, 1460)
	White	Men	57	Beta(9.09, 1390)
	White	Men	58	Beta(9.09, 1320)
	White	Men	59	Beta(9.09, 1250)
	White	Men	60	Beta(9.09, 1190)
	White	Men	61	Beta(9.08, 1140)
	White	Men	62	Beta(8.97, 1070)
	White	Men	63	Beta(9.07, 1040)
	White	Men	64	Beta(9.08, 991)
	White	Men	65	Beta(9.07, 948)
	White	Men	66	Beta(9.04, 904)
	White	Men	67	Beta(8.92, 856)
	White	Men	68	Beta(9.07, 836)
	White	Men	69	Beta(9.06, 802)
	White	Men	70	Beta(9.06, 771)
	White	Men	71	Beta(9.06, 741)
	White	Men	72	Beta(9.06, 713)
	White	Men	73	Beta(9.06, 686)
	White	Men	74	Beta(9.05, 661)
	White	Men	75	Beta(8.92, 626)
	White	Men	76	Beta(9.05, 613)
	White	Men	77	Beta(9.05, 591)
	White	Men	78	Beta(9.03, 569)
	White	Men	79	Beta(9.03, 549)
	White	Men	80	Beta(9, 528)
	White	Men	81	Beta(9.03, 512)
	White	Men	82	Beta(9.03, 494)
	White	Men	83	Beta(9.03, 476)
	White	Men	84	Beta(9.02, 459)

Variable	Race/ethnicity	Sex	Age	Distribution
	White	Women	30	Beta(6.52, 10000)
	White	Women	31	Beta(6.98, 10000)
	White	Women	32	Beta(7.47, 10000)
	White	Women	33	Beta(7.99, 10000)
	White	Women	34	Beta(8.54, 10000)
	White	Women	35	Beta(9.12, 10000)
	White	Women	36	Beta(9.09, 9310)
	White	Women	37	Beta(9.12, 8750)
	White	Women	38	Beta(9.12, 8210)
	White	Women	39	Beta(9.12, 7700)
	White	Women	40	Beta(9.12, 7230)
	White	Women	41	Beta(9.12, 6790)
	White	Women	42	Beta(9.1, 6360)
	White	Women	43	Beta(9.12, 6010)
	White	Women	44	Beta(9.12, 5660)
	White	Women	45	Beta(9.12, 5340)
	White	Women	46	Beta(9.12, 5040)
	White	Women	47	Beta(9.1, 4750)
	White	Women	48	Beta(9.11, 4500)
	White	Women	49	Beta(9.12, 4260)
	White	Women	50	Beta(9.11, 4030)
	White	Women	51	Beta(9.11, 3810)
	White	Women	52	Beta(9.07, 3590)
	White	Women	53	Beta(9.11, 3420)
	White	Women	54	Beta(9.1, 3240)
	White	Women	55	Beta(9.11, 3080)
	White	Women	56	Beta(9.11, 2930)
	White	Women	57	Beta(9.09, 2780)
	White	Women	58	Beta(9.11, 2650)
	White	Women	59	Beta(9.11, 2520)
	White	Women	60	Beta(9.1, 2400)
	White	Women	61	Beta(9.11, 2290)
	White	Women	62	Beta(9.1, 2180)
	White	Women	63	Beta(9.1, 2080)
	White	Women	64	Beta(9.1, 1990)
	White	Women	65	Beta(9.1, 1900)
	White	Women	66	Beta(8.96, 1780)
	White	Women	67	Beta(9.1, 1730)
	White	Women	68	Beta(9.1, 1660)
	White	Women	69	Beta(9.1, 1580)
	White	Women	70	Beta(9.09, 1520)
	White	Women	71	Beta(9.09, 1450)
	White	Women	72	Beta(9.09, 1390)

Variable	Race/ethnicity	Sex	Age	Distribution
	White	Women	73	Beta(9.06, 1330)
	White	Women	74	Beta(9.09, 1280)
	White	Women	75	Beta(9.07, 1220)
	White	Women	76	Beta(9.09, 1170)
	White	Women	77	Beta(9.07, 1120)
	White	Women	78	Beta(9.08, 1080)
	White	Women	79	Beta(9.07, 1040)
	White	Women	80	Beta(9.08, 995)
	White	Women	81	Beta(9.04, 950)
	White	Women	82	Beta(9.06, 914)
	White	Women	83	Beta(9.07, 879)
	White	Women	84	Beta(9.07, 843)
	Black	Men	30	Beta(9.11, 8300)
	Black	Men	31	Beta(9.12, 7630)
	Black	Men	32	Beta(9.11, 7010)
	Black	Men	33	Beta(9.11, 6460)
	Black	Men	34	Beta(9.12, 5980)
	Black	Men	35	Beta(9.12, 5540)
	Black	Men	36	Beta(9.12, 5140)
	Black	Men	37	Beta(9.1, 4770)
	Black	Men	38	Beta(9.12, 4450)
	Black	Men	39	Beta(9.11, 4150)
	Black	Men	40	Beta(9.05, 3840)
	Black	Men	41	Beta(9.11, 3610)
	Black	Men	42	Beta(9.11, 3370)
	Black	Men	43	Beta(9.11, 3150)
	Black	Men	44	Beta(9.11, 2960)
	Black	Men	45	Beta(9.11, 2770)
	Black	Men	46	Beta(9.11, 2600)
	Black	Men	47	Beta(9.1, 2440)
	Black	Men	48	Beta(9.1, 2300)
	Black	Men	49	Beta(9.1, 2160)
	Black	Men	50	Beta(9.1, 2030)
	Black	Men	51	Beta(9.1, 1920)
	Black	Men	52	Beta(8.99, 1780)
	Black	Men	53	Beta(8.98, 1680)
	Black	Men	54	Beta(9.09, 1610)
	Black	Men	55	Beta(9.09, 1520)
	Black	Men	56	Beta(9.09, 1440)
	Black	Men	57	Beta(9.09, 1360)
	Black	Men	58	Beta(9.09, 1290)
	Black	Men	59	Beta(9.09, 1220)
	Black	Men	60	Beta(8.9, 1140)

Variable	Race/ethnicity	Sex	Age	Distribution
	Black	Men	61	Beta(9.08, 1100)
	Black	Men	62	Beta(9.08, 1050)
	Black	Men	63	Beta(9.08, 996)
	Black	Men	64	Beta(9.08, 949)
	Black	Men	65	Beta(9.07, 904)
	Black	Men	66	Beta(9.07, 863)
	Black	Men	67	Beta(9.07, 824)
	Black	Men	68	Beta(9.07, 787)
	Black	Men	69	Beta(9.06, 752)
	Black	Men	70	Beta(9.06, 720)
	Black	Men	71	Beta(9.05, 689)
	Black	Men	72	Beta(9.01, 657)
	Black	Men	73	Beta(9.05, 632)
	Black	Men	74	Beta(9.05, 606)
	Black	Men	75	Beta(9.04, 581)
	Black	Men	76	Beta(9.04, 558)
	Black	Men	77	Beta(9.02, 534)
	Black	Men	78	Beta(8.88, 505)
	Black	Men	79	Beta(9.03, 493)
	Black	Men	80	Beta(9.03, 475)
	Black	Men	81	Beta(9.02, 456)
	Black	Men	82	Beta(9.02, 439)
	Black	Men	83	Beta(8.93, 418)
	Black	Men	84	Beta(9.01, 406)
	Black	Women	30	Beta(9.06, 10000)
	Black	Women	31	Beta(9.12, 9460)
	Black	Women	32	Beta(9.12, 8900)
	Black	Women	33	Beta(9.12, 8380)
	Black	Women	34	Beta(9.12, 7890)
	Black	Women	35	Beta(9.12, 7440)
	Black	Women	36	Beta(8.95, 6880)
	Black	Women	37	Beta(9.12, 6620)
	Black	Women	38	Beta(9.11, 6250)
	Black	Women	39	Beta(9.11, 5910)
	Black	Women	40	Beta(9.12, 5590)
	Black	Women	41	Beta(9.12, 5290)
	Black	Women	42	Beta(9.12, 5010)
	Black	Women	43	Beta(9.12, 4740)
	Black	Women	44	Beta(9.11, 4500)
	Black	Women	45	Beta(9.1, 4250)
	Black	Women	46	Beta(9.11, 4040)
	Black	Women	47	Beta(9.11, 3840)
	Black	Women	48	Beta(9.11, 3640)

Variable	Race/ethnicity	Sex	Age	Distribution
	Black	Women	49	Beta(9.08, 3440)
	Black	Women	50	Beta(9.1, 3280)
	Black	Women	51	Beta(9.11, 3120)
	Black	Women	52	Beta(9.11, 2960)
	Black	Women	53	Beta(9.11, 2820)
	Black	Women	54	Beta(9.11, 2680)
	Black	Women	55	Beta(9.11, 2550)
	Black	Women	56	Beta(9.1, 2430)
	Black	Women	57	Beta(9.1, 2310)
	Black	Women	58	Beta(9.08, 2190)
	Black	Women	59	Beta(8.86, 2040)
	Black	Women	60	Beta(9.08, 1990)
	Black	Women	61	Beta(9.1, 1900)
	Black	Women	62	Beta(9.1, 1810)
	Black	Women	63	Beta(9.05, 1720)
	Black	Women	64	Beta(9.08, 1650)
	Black	Women	65	Beta(9.07, 1570)
	Black	Women	66	Beta(9.09, 1500)
	Black	Women	67	Beta(9.09, 1440)
	Black	Women	68	Beta(9.09, 1370)
	Black	Women	69	Beta(9.09, 1310)
	Black	Women	70	Beta(9.09, 1250)
	Black	Women	71	Beta(9.05, 1190)
	Black	Women	72	Beta(9.08, 1150)
	Black	Women	73	Beta(9.08, 1100)
	Black	Women	74	Beta(9.08, 1050)
	Black	Women	75	Beta(9.08, 1000)
	Black	Women	76	Beta(9.08, 960)
	Black	Women	77	Beta(9.07, 919)
	Black	Women	78	Beta(9.07, 880)
	Black	Women	79	Beta(9.07, 843)
	Black	Women	80	Beta(9.06, 806)
	Black	Women	81	Beta(9.06, 773)
	Black	Women	82	Beta(9.06, 740)
	Black	Women	83	Beta(9.06, 708)
	Black	Women	84	Beta(9.06, 677)
	Hispanic	Men	30	Beta(9.08, 8580)
	Hispanic	Men	31	Beta(9.12, 7910)
	Hispanic	Men	32	Beta(9.12, 7290)
	Hispanic	Men	33	Beta(9.11, 6720)
	Hispanic	Men	34	Beta(9.11, 6210)
	Hispanic	Men	35	Beta(9.09, 5730)
	Hispanic	Men	36	Beta(9.09, 5320)

Variable	Race/ethnicity	Sex	Age	Distribution
	Hispanic	Men	37	Beta(9.1, 4940)
	Hispanic	Men	38	Beta(9.1, 4600)
	Hispanic	Men	39	Beta(9.12, 4290)
	Hispanic	Men	40	Beta(9.11, 4000)
	Hispanic	Men	41	Beta(9.11, 3730)
	Hispanic	Men	42	Beta(9.11, 3480)
	Hispanic	Men	43	Beta(9.11, 3260)
	Hispanic	Men	44	Beta(9.11, 3050)
	Hispanic	Men	45	Beta(9.07, 2840)
	Hispanic	Men	46	Beta(9.11, 2680)
	Hispanic	Men	47	Beta(9.11, 2520)
	Hispanic	Men	48	Beta(9.11, 2360)
	Hispanic	Men	49	Beta(9.1, 2220)
	Hispanic	Men	50	Beta(9.1, 2090)
	Hispanic	Men	51	Beta(9.09, 1970)
	Hispanic	Men	52	Beta(9.1, 1860)
	Hispanic	Men	53	Beta(9.1, 1750)
	Hispanic	Men	54	Beta(9.1, 1660)
	Hispanic	Men	55	Beta(9.08, 1560)
	Hispanic	Men	56	Beta(9.08, 1480)
	Hispanic	Men	57	Beta(9.09, 1400)
	Hispanic	Men	58	Beta(9.05, 1330)
	Hispanic	Men	59	Beta(9.09, 1260)
	Hispanic	Men	60	Beta(9.09, 1200)
	Hispanic	Men	61	Beta(9.08, 1140)
	Hispanic	Men	62	Beta(9.08, 1080)
	Hispanic	Men	63	Beta(9.08, 1030)
	Hispanic	Men	64	Beta(9.08, 983)
	Hispanic	Men	65	Beta(8.93, 922)
	Hispanic	Men	66	Beta(9.06, 893)
	Hispanic	Men	67	Beta(9.07, 853)
	Hispanic	Men	68	Beta(9.07, 815)
	Hispanic	Men	69	Beta(9.04, 777)
	Hispanic	Men	70	Beta(9.06, 745)
	Hispanic	Men	71	Beta(9.01, 709)
	Hispanic	Men	72	Beta(9.06, 683)
	Hispanic	Men	73	Beta(9.04, 654)
	Hispanic	Men	74	Beta(9.05, 628)
	Hispanic	Men	75	Beta(9.05, 602)
	Hispanic	Men	76	Beta(9.04, 578)
	Hispanic	Men	77	Beta(9.04, 554)
	Hispanic	Men	78	Beta(9.04, 533)
	Hispanic	Men	79	Beta(9.03, 512)

Variable	Race/ethnicity	Sex	Age	Distribution
	Hispanic	Men	80	Beta(9.03, 492)
	Hispanic	Men	81	Beta(9.03, 473)
	Hispanic	Men	82	Beta(9.02, 455)
	Hispanic	Men	83	Beta(9.02, 438)
	Hispanic	Men	84	Beta(8.94, 417)
	Hispanic	Women	30	Beta(7.69, 10000)
	Hispanic	Women	31	Beta(8.19, 10000)
	Hispanic	Women	32	Beta(8.72, 10000)
	Hispanic	Women	33	Beta(9.11, 9810)
	Hispanic	Women	34	Beta(9.12, 9220)
	Hispanic	Women	35	Beta(9.1, 8630)
	Hispanic	Women	36	Beta(9.11, 8130)
	Hispanic	Women	37	Beta(9.11, 7640)
	Hispanic	Women	38	Beta(9.12, 7200)
	Hispanic	Women	39	Beta(9.09, 6760)
	Hispanic	Women	40	Beta(9.11, 6390)
	Hispanic	Women	41	Beta(9.12, 6030)
	Hispanic	Women	42	Beta(9.12, 5680)
	Hispanic	Women	43	Beta(9.12, 5370)
	Hispanic	Women	44	Beta(9.12, 5070)
	Hispanic	Women	45	Beta(9.1, 4780)
	Hispanic	Women	46	Beta(9.1, 4520)
	Hispanic	Women	47	Beta(9.12, 4280)
	Hispanic	Women	48	Beta(9.11, 4050)
	Hispanic	Women	49	Beta(9.11, 3840)
	Hispanic	Women	50	Beta(9.11, 3630)
	Hispanic	Women	51	Beta(9.08, 3430)
	Hispanic	Women	52	Beta(9.11, 3260)
	Hispanic	Women	53	Beta(9.11, 3090)
	Hispanic	Women	54	Beta(9.11, 2930)
	Hispanic	Women	55	Beta(9.11, 2780)
	Hispanic	Women	56	Beta(9.09, 2640)
	Hispanic	Women	57	Beta(9.06, 2500)
	Hispanic	Women	58	Beta(9.07, 2370)
	Hispanic	Women	59	Beta(9.04, 2250)
	Hispanic	Women	60	Beta(9.1, 2150)
	Hispanic	Women	61	Beta(9.1, 2050)
	Hispanic	Women	62	Beta(9.1, 1950)
	Hispanic	Women	63	Beta(9.1, 1850)
	Hispanic	Women	64	Beta(9.1, 1760)
	Hispanic	Women	65	Beta(9.1, 1680)
	Hispanic	Women	66	Beta(9.1, 1600)
	Hispanic	Women	67	Beta(9.09, 1520)

Variable	Race/ethnicity	Sex	Age	Distribution
	Hispanic	Women	68	Beta(9.09, 1450)
	Hispanic	Women	69	Beta(9.09, 1380)
	Hispanic	Women	70	Beta(9.09, 1320)
	Hispanic	Women	71	Beta(9.09, 1260)
	Hispanic	Women	72	Beta(9.09, 1200)
	Hispanic	Women	73	Beta(9.08, 1140)
	Hispanic	Women	74	Beta(9, 1080)
	Hispanic	Women	75	Beta(9.08, 1040)
	Hispanic	Women	76	Beta(9.07, 994)
	Hispanic	Women	77	Beta(9.08, 950)
	Hispanic	Women	78	Beta(9.05, 905)
	Hispanic	Women	79	Beta(9.07, 866)
	Hispanic	Women	80	Beta(9.05, 825)
	Hispanic	Women	81	Beta(9.07, 790)
	Hispanic	Women	82	Beta(9.06, 755)
	Hispanic	Women	83	Beta(9.05, 720)
	Hispanic	Women	84	Beta(9.06, 687)
Stroke incidence rate for 2014				
	White	Men	30	Beta(2.03, 8230)
	White	Men	31	Beta(2.59, 10000)
	White	Men	32	Beta(2.81, 10000)
	White	Men	33	Beta(3.06, 10000)
	White	Men	34	Beta(1.66, 4310)
	White	Men	35	Beta(3.66, 10000)
	White	Men	36	Beta(3.98, 10000)
	White	Men	37	Beta(4.36, 10000)
	White	Men	38	Beta(4.75, 10000)
	White	Men	39	Beta(5.19, 10000)
	White	Men	40	Beta(5.66, 10000)
	White	Men	41	Beta(6.16, 10000)
	White	Men	42	Beta(6.72, 10000)
	White	Men	43	Beta(7.31, 10000)
	White	Men	44	Beta(7.91, 10000)
	White	Men	45	Beta(8.59, 10000)
	White	Men	46	Beta(9.12, 9750)
	White	Men	47	Beta(9.11, 8910)
	White	Men	48	Beta(8.96, 8030)
	White	Men	49	Beta(9.12, 7500)
	White	Men	50	Beta(9.09, 6870)
	White	Men	51	Beta(9.07, 6320)
	White	Men	52	Beta(9.12, 5870)
	White	Men	53	Beta(9.11, 5420)
	White	Men	54	Beta(9.1, 4990)

Variable	Race/ethnicity	Sex	Age	Distribution
	White	Men	55	Beta(9.1, 4610)
	White	Men	56	Beta(9.12, 4270)
	White	Men	57	Beta(9.11, 3950)
	White	Men	58	Beta(8.84, 3550)
	White	Men	59	Beta(9.1, 3400)
	White	Men	60	Beta(9.11, 3160)
	White	Men	61	Beta(9.11, 2940)
	White	Men	62	Beta(9.11, 2730)
	White	Men	63	Beta(9.07, 2530)
	White	Men	64	Beta(9.11, 2370)
	White	Men	65	Beta(9.1, 2210)
	White	Men	66	Beta(9.1, 2060)
	White	Men	67	Beta(9.1, 1930)
	White	Men	68	Beta(9.08, 1800)
	White	Men	69	Beta(9.1, 1680)
	White	Men	70	Beta(9.08, 1570)
	White	Men	71	Beta(9.09, 1470)
	White	Men	72	Beta(9.09, 1380)
	White	Men	73	Beta(9.09, 1290)
	White	Men	74	Beta(9.09, 1210)
	White	Men	75	Beta(9.08, 1130)
	White	Men	76	Beta(9.08, 1060)
	White	Men	77	Beta(9.04, 992)
	White	Men	78	Beta(9.06, 934)
	White	Men	79	Beta(9.07, 878)
	White	Men	80	Beta(8.92, 810)
	White	Men	81	Beta(9.06, 773)
	White	Men	82	Beta(9.06, 727)
	White	Men	83	Beta(9.06, 684)
	White	Men	84	Beta(9.01, 640)
	White	Women	30	Beta(4.18, 10000)
	White	Women	31	Beta(4.07, 10000)
	White	Women	32	Beta(3.24, 6590)
	White	Women	33	Beta(4.63, 10000)
	White	Women	34	Beta(5.29, 10000)
	White	Women	35	Beta(5.26, 10000)
	White	Women	36	Beta(5.94, 10000)
	White	Women	37	Beta(5.94, 10000)
	White	Women	38	Beta(6.69, 10000)
	White	Women	39	Beta(6.75, 10000)
	White	Women	40	Beta(7.5, 10000)
	White	Women	41	Beta(7.6, 10000)
	White	Women	42	Beta(8.42, 10000)

Variable	Race/ethnicity	Sex	Age	Distribution
	White	Women	43	Beta(8.57, 10000)
	White	Women	44	Beta(9.12, 9630)
	White	Women	45	Beta(9.12, 9380)
	White	Women	46	Beta(9.12, 8510)
	White	Women	47	Beta(9.12, 8270)
	White	Women	48	Beta(9.12, 7540)
	White	Women	49	Beta(9.12, 7300)
	White	Women	50	Beta(9.12, 6690)
	White	Women	51	Beta(9.12, 6470)
	White	Women	52	Beta(9.11, 5950)
	White	Women	53	Beta(9.12, 5740)
	White	Women	54	Beta(9.12, 5290)
	White	Women	55	Beta(9.12, 5100)
	White	Women	56	Beta(9.11, 4730)
	White	Women	57	Beta(9.1, 4540)
	White	Women	58	Beta(9.11, 4230)
	White	Women	59	Beta(9.11, 4070)
	White	Women	60	Beta(9.11, 3790)
	White	Women	61	Beta(9.11, 3630)
	White	Women	62	Beta(9.1, 3380)
	White	Women	63	Beta(9.11, 3240)
	White	Women	64	Beta(9.09, 3020)
	White	Women	65	Beta(9.11, 2890)
	White	Women	66	Beta(9.11, 2700)
	White	Women	67	Beta(9.09, 2570)
	White	Women	68	Beta(9.07, 2390)
	White	Women	69	Beta(9.11, 2280)
	White	Women	70	Beta(9.1, 2130)
	White	Women	71	Beta(9.1, 2020)
	White	Women	72	Beta(9.1, 1880)
	White	Women	73	Beta(9.07, 1770)
	White	Women	74	Beta(9.1, 1650)
	White	Women	75	Beta(9.09, 1550)
	White	Women	76	Beta(9.09, 1430)
	White	Women	77	Beta(9.09, 1340)
	White	Women	78	Beta(9.09, 1230)
	White	Women	79	Beta(9.08, 1150)
	White	Women	80	Beta(9.08, 1050)
	White	Women	81	Beta(9.04, 978)
	White	Women	82	Beta(9.07, 905)
	White	Women	83	Beta(9.06, 768)
	White	Women	84	Beta(9.06, 679)
	Black	Men	30	Beta(5.25, 10000)

Variable	Race/ethnicity	Sex	Age	Distribution
	Black	Men	31	Beta(5.68, 10000)
	Black	Men	32	Beta(6.13, 10000)
	Black	Men	33	Beta(6.63, 10000)
	Black	Men	34	Beta(7.17, 10000)
	Black	Men	35	Beta(7.74, 10000)
	Black	Men	36	Beta(7.33, 8720)
	Black	Men	37	Beta(9, 10000)
	Black	Men	38	Beta(9.11, 9390)
	Black	Men	39	Beta(9.12, 8730)
	Black	Men	40	Beta(9.12, 8110)
	Black	Men	41	Beta(9.12, 7540)
	Black	Men	42	Beta(9.11, 7000)
	Black	Men	43	Beta(9.12, 6520)
	Black	Men	44	Beta(9.12, 6070)
	Black	Men	45	Beta(8.91, 5530)
	Black	Men	46	Beta(9.12, 5290)
	Black	Men	47	Beta(9.1, 4930)
	Black	Men	48	Beta(9.1, 4610)
	Black	Men	49	Beta(9.11, 4320)
	Black	Men	50	Beta(9.1, 4040)
	Black	Men	51	Beta(9.11, 3780)
	Black	Men	52	Beta(9.1, 3540)
	Black	Men	53	Beta(9.11, 3320)
	Black	Men	54	Beta(9.11, 3110)
	Black	Men	55	Beta(9.11, 2920)
	Black	Men	56	Beta(8.67, 2600)
	Black	Men	57	Beta(9.11, 2570)
	Black	Men	58	Beta(9.11, 2420)
	Black	Men	59	Beta(9.08, 2270)
	Black	Men	60	Beta(8.93, 2100)
	Black	Men	61	Beta(9.1, 2010)
	Black	Men	62	Beta(9.1, 1890)
	Black	Men	63	Beta(9.1, 1780)
	Black	Men	64	Beta(8.91, 1640)
	Black	Men	65	Beta(9.1, 1580)
	Black	Men	66	Beta(9.08, 1490)
	Black	Men	67	Beta(9.09, 1410)
	Black	Men	68	Beta(9.09, 1330)
	Black	Men	69	Beta(9.09, 1250)
	Black	Men	70	Beta(9.09, 1180)
	Black	Men	71	Beta(9.08, 1110)
	Black	Men	72	Beta(9.01, 1040)
	Black	Men	73	Beta(9.08, 992)

Variable	Race/ethnicity	Sex	Age	Distribution
	Black	Men	74	Beta(9.07, 936)
	Black	Men	75	Beta(9.07, 884)
	Black	Men	76	Beta(8.88, 817)
	Black	Men	77	Beta(9.06, 788)
	Black	Men	78	Beta(9.06, 744)
	Black	Men	79	Beta(9.05, 702)
	Black	Men	80	Beta(9.05, 664)
	Black	Men	81	Beta(9.05, 627)
	Black	Men	82	Beta(9.03, 592)
	Black	Men	83	Beta(9.04, 560)
	Black	Men	84	Beta(9.03, 528)
	Black	Women	30	Beta(2.85, 10000)
	Black	Women	31	Beta(3.04, 10000)
	Black	Women	32	Beta(3.23, 10000)
	Black	Women	33	Beta(3.44, 10000)
	Black	Women	34	Beta(3.67, 10000)
	Black	Women	35	Beta(3.91, 10000)
	Black	Women	36	Beta(4.17, 10000)
	Black	Women	37	Beta(4.45, 10000)
	Black	Women	38	Beta(4.75, 10000)
	Black	Women	39	Beta(5.07, 10000)
	Black	Women	40	Beta(5.42, 10000)
	Black	Women	41	Beta(5.79, 10000)
	Black	Women	42	Beta(3.3, 5020)
	Black	Women	43	Beta(6.62, 10000)
	Black	Women	44	Beta(7.08, 10000)
	Black	Women	45	Beta(5.13, 6580)
	Black	Women	46	Beta(8.12, 10000)
	Black	Women	47	Beta(8.7, 10000)
	Black	Women	48	Beta(9.12, 9780)
	Black	Women	49	Beta(9.12, 9100)
	Black	Women	50	Beta(9.11, 8470)
	Black	Women	51	Beta(9.12, 7880)
	Black	Women	52	Beta(9.12, 7330)
	Black	Women	53	Beta(9.12, 6830)
	Black	Women	54	Beta(9.05, 6310)
	Black	Women	55	Beta(9.11, 5910)
	Black	Women	56	Beta(9.12, 5500)
	Black	Women	57	Beta(9.12, 5120)
	Black	Women	58	Beta(9.1, 4760)
	Black	Women	59	Beta(9.12, 4440)
	Black	Women	60	Beta(9.11, 4130)
	Black	Women	61	Beta(9.11, 3840)

Variable	Race/ethnicity	Sex	Age	Distribution
	Black	Women	62	Beta(8.95, 3510)
	Black	Women	63	Beta(9.11, 3330)
	Black	Women	64	Beta(9.11, 3100)
	Black	Women	65	Beta(9.11, 2880)
	Black	Women	66	Beta(9.11, 2680)
	Black	Women	67	Beta(9.11, 2490)
	Black	Women	68	Beta(9.11, 2320)
	Black	Women	69	Beta(9.1, 2160)
	Black	Women	70	Beta(9.1, 2010)
	Black	Women	71	Beta(9.1, 1870)
	Black	Women	72	Beta(9.1, 1740)
	Black	Women	73	Beta(9.1, 1620)
	Black	Women	74	Beta(9.06, 1500)
	Black	Women	75	Beta(9.09, 1400)
	Black	Women	76	Beta(9.08, 1300)
	Black	Women	77	Beta(9.09, 1210)
	Black	Women	78	Beta(9.08, 1130)
	Black	Women	79	Beta(9.08, 1050)
	Black	Women	80	Beta(9.06, 971)
	Black	Women	81	Beta(9.07, 905)
	Black	Women	82	Beta(9.06, 840)
	Black	Women	83	Beta(9.06, 781)
	Black	Women	84	Beta(9.06, 726)
	Hispanic	Men	30	Beta(4.72, 10000)
	Hispanic	Men	31	Beta(5.05, 10000)
	Hispanic	Men	32	Beta(5.4, 10000)
	Hispanic	Men	33	Beta(5.78, 10000)
	Hispanic	Men	34	Beta(6.18, 10000)
	Hispanic	Men	35	Beta(6.61, 10000)
	Hispanic	Men	36	Beta(7.08, 10000)
	Hispanic	Men	37	Beta(7.57, 10000)
	Hispanic	Men	38	Beta(8.1, 10000)
	Hispanic	Men	39	Beta(8.66, 10000)
	Hispanic	Men	40	Beta(9.12, 9850)
	Hispanic	Men	41	Beta(9.1, 9170)
	Hispanic	Men	42	Beta(8.99, 8450)
	Hispanic	Men	43	Beta(9.12, 8020)
	Hispanic	Men	44	Beta(9.11, 7490)
	Hispanic	Men	45	Beta(9.11, 7010)
	Hispanic	Men	46	Beta(9.12, 6560)
	Hispanic	Men	47	Beta(9.12, 6140)
	Hispanic	Men	48	Beta(9.11, 5750)
	Hispanic	Men	49	Beta(9.11, 5380)

Variable	Race/ethnicity	Sex	Age	Distribution
	Hispanic	Men	50	Beta(9.12, 5050)
	Hispanic	Men	51	Beta(9.05, 4690)
	Hispanic	Men	52	Beta(9.11, 4430)
	Hispanic	Men	53	Beta(9.11, 4150)
	Hispanic	Men	54	Beta(8.95, 3820)
	Hispanic	Men	55	Beta(9.11, 3650)
	Hispanic	Men	56	Beta(9.06, 3410)
	Hispanic	Men	57	Beta(9.11, 3220)
	Hispanic	Men	58	Beta(9.11, 3020)
	Hispanic	Men	59	Beta(9.09, 2830)
	Hispanic	Men	60	Beta(9.11, 2670)
	Hispanic	Men	61	Beta(9.11, 2510)
	Hispanic	Men	62	Beta(9.1, 2360)
	Hispanic	Men	63	Beta(9.1, 2220)
	Hispanic	Men	64	Beta(9.1, 2090)
	Hispanic	Men	65	Beta(9.1, 1960)
	Hispanic	Men	66	Beta(9.1, 1850)
	Hispanic	Men	67	Beta(9.08, 1740)
	Hispanic	Men	68	Beta(9.1, 1640)
	Hispanic	Men	69	Beta(9.08, 1540)
	Hispanic	Men	70	Beta(9.09, 1450)
	Hispanic	Men	71	Beta(9.09, 1370)
	Hispanic	Men	72	Beta(9.09, 1290)
	Hispanic	Men	73	Beta(9.09, 1210)
	Hispanic	Men	74	Beta(9.07, 1140)
	Hispanic	Men	75	Beta(9.08, 1080)
	Hispanic	Men	76	Beta(9.08, 1020)
	Hispanic	Men	77	Beta(9.08, 956)
	Hispanic	Men	78	Beta(9, 894)
	Hispanic	Men	79	Beta(9.07, 849)
	Hispanic	Men	80	Beta(9.07, 800)
	Hispanic	Men	81	Beta(9.06, 753)
	Hispanic	Men	82	Beta(9.06, 710)
	Hispanic	Men	83	Beta(9.06, 669)
	Hispanic	Men	84	Beta(9.05, 630)
	Hispanic	Women	30	Beta(2.5, 10000)
	Hispanic	Women	31	Beta(2.67, 10000)
	Hispanic	Women	32	Beta(2.85, 10000)
	Hispanic	Women	33	Beta(3.04, 10000)
	Hispanic	Women	34	Beta(3.24, 10000)
	Hispanic	Women	35	Beta(3.46, 10000)
	Hispanic	Women	36	Beta(3.69, 10000)
	Hispanic	Women	37	Beta(3.94, 10000)

Variable	Race/ethnicity	Sex	Age	Distribution
	Hispanic	Women	38	Beta(4.21, 10000)
	Hispanic	Women	39	Beta(4.5, 10000)
	Hispanic	Women	40	Beta(4.81, 10000)
	Hispanic	Women	41	Beta(5.14, 10000)
	Hispanic	Women	42	Beta(5.5, 10000)
	Hispanic	Women	43	Beta(5.88, 10000)
	Hispanic	Women	44	Beta(6.29, 10000)
	Hispanic	Women	45	Beta(6.73, 10000)
	Hispanic	Women	46	Beta(7.2, 10000)
	Hispanic	Women	47	Beta(7.7, 10000)
	Hispanic	Women	48	Beta(8.25, 10000)
	Hispanic	Women	49	Beta(8.83, 10000)
	Hispanic	Women	50	Beta(9.08, 9600)
	Hispanic	Women	51	Beta(9.12, 8980)
	Hispanic	Women	52	Beta(9.12, 8370)
	Hispanic	Women	53	Beta(9.1, 7780)
	Hispanic	Women	54	Beta(8.86, 7050)
	Hispanic	Women	55	Beta(9.12, 6770)
	Hispanic	Women	56	Beta(9.07, 6280)
	Hispanic	Women	57	Beta(9.1, 5880)
	Hispanic	Women	58	Beta(8.95, 5380)
	Hispanic	Women	59	Beta(9.12, 5110)
	Hispanic	Women	60	Beta(9.1, 4760)
	Hispanic	Women	61	Beta(9.11, 4440)
	Hispanic	Women	62	Beta(9.11, 4150)
	Hispanic	Women	63	Beta(9.08, 3850)
	Hispanic	Women	64	Beta(9.09, 3590)
	Hispanic	Women	65	Beta(9.11, 3360)
	Hispanic	Women	66	Beta(9.1, 3130)
	Hispanic	Women	67	Beta(9.11, 2920)
	Hispanic	Women	68	Beta(9.11, 2720)
	Hispanic	Women	69	Beta(9.11, 2540)
	Hispanic	Women	70	Beta(9.1, 2370)
	Hispanic	Women	71	Beta(9.07, 2200)
	Hispanic	Women	72	Beta(9.1, 2060)
	Hispanic	Women	73	Beta(9.1, 1920)
	Hispanic	Women	74	Beta(9.1, 1790)
	Hispanic	Women	75	Beta(8.87, 1630)
	Hispanic	Women	76	Beta(9.09, 1560)
	Hispanic	Women	77	Beta(9.08, 1450)
	Hispanic	Women	78	Beta(9.08, 1350)
	Hispanic	Women	79	Beta(9.09, 1260)
	Hispanic	Women	80	Beta(9.03, 1170)

Variable	Race/ethnicity	Sex	Age	Distribution
	Hispanic	Women	81	Beta(9.08, 1090)
	Hispanic	Women	82	Beta(9.08, 1020)
	Hispanic	Women	83	Beta(9.08, 948)
	Hispanic	Women	84	Beta(9.07, 883)
CHD prevalence rate for 2014				
	White	Men	30	Beta(9.09, 1250)
	White	Men	31	Beta(9.08, 1090)
	White	Men	32	Beta(9.04, 953)
	White	Men	33	Beta(8.99, 835)
	White	Men	34	Beta(9.06, 745)
	White	Men	35	Beta(8.91, 650)
	White	Men	36	Beta(9.01, 586)
	White	Men	37	Beta(8.87, 515)
	White	Men	38	Beta(9.03, 470)
	White	Men	39	Beta(9.02, 422)
	White	Men	40	Beta(9, 380)
	White	Men	41	Beta(8.99, 343)
	White	Men	42	Beta(8.98, 311)
	White	Men	43	Beta(8.96, 282)
	White	Men	44	Beta(8.88, 254)
	White	Men	45	Beta(8.9, 233)
	White	Men	46	Beta(8.87, 212)
	White	Men	47	Beta(8.87, 194)
	White	Men	48	Beta(8.86, 179)
	White	Men	49	Beta(8.85, 164)
	White	Men	50	Beta(8.84, 151)
	White	Men	51	Beta(8.8, 139)
	White	Men	52	Beta(8.8, 129)
	White	Men	53	Beta(8.78, 119)
	White	Men	54	Beta(8.72, 110)
	White	Men	55	Beta(8.7, 102)
	White	Men	56	Beta(8.65, 94.5)
	White	Men	57	Beta(8.68, 88.5)
	White	Men	58	Beta(8.65, 82.4)
	White	Men	59	Beta(8.63, 76.9)
	White	Men	60	Beta(8.6, 71.8)
	White	Men	61	Beta(8.58, 67.1)
	White	Men	62	Beta(8.55, 62.8)
	White	Men	63	Beta(8.52, 58.8)
	White	Men	64	Beta(8.49, 55.2)
	White	Men	65	Beta(8.47, 51.8)
	White	Men	66	Beta(8.44, 48.6)
	White	Men	67	Beta(8.38, 45.6)

Variable	Race/ethnicity	Sex	Age	Distribution
	White	Men	68	Beta(8.38, 43.1)
	White	Men	69	Beta(8.36, 40.6)
	White	Men	70	Beta(8.33, 38.3)
	White	Men	71	Beta(8.24, 35.9)
	White	Men	72	Beta(8.28, 34.2)
	White	Men	73	Beta(8.25, 32.5)
	White	Men	74	Beta(8.23, 30.8)
	White	Men	75	Beta(8.2, 29.3)
	White	Men	76	Beta(8.12, 27.7)
	White	Men	77	Beta(8.1, 26.4)
	White	Men	78	Beta(8.09, 25.3)
	White	Men	79	Beta(8.07, 24.3)
	White	Men	80	Beta(8.06, 23.4)
	White	Men	81	Beta(8.04, 22.6)
	White	Men	82	Beta(8.03, 21.8)
	White	Men	83	Beta(8.02, 21.3)
	White	Men	84	Beta(8.01, 21)
	White	Women	30	Beta(9.09, 1440)
	White	Women	31	Beta(9.09, 1280)
	White	Women	32	Beta(9.08, 1140)
	White	Women	33	Beta(9.08, 1020)
	White	Women	34	Beta(9.07, 920)
	White	Women	35	Beta(9.04, 828)
	White	Women	36	Beta(9.06, 752)
	White	Women	37	Beta(9.06, 683)
	White	Women	38	Beta(9.04, 621)
	White	Women	39	Beta(8.82, 553)
	White	Women	40	Beta(9.03, 519)
	White	Women	41	Beta(8.51, 448)
	White	Women	42	Beta(9.02, 438)
	White	Women	43	Beta(8.98, 402)
	White	Women	44	Beta(8.99, 371)
	White	Women	45	Beta(8.99, 344)
	White	Women	46	Beta(8.98, 318)
	White	Women	47	Beta(8.93, 293)
	White	Women	48	Beta(8.96, 273)
	White	Women	49	Beta(8.9, 252)
	White	Women	50	Beta(8.9, 235)
	White	Women	51	Beta(8.92, 220)
	White	Women	52	Beta(8.87, 205)
	White	Women	53	Beta(8.87, 191)
	White	Women	54	Beta(8.87, 179)
	White	Women	55	Beta(8.85, 168)

Variable	Race/ethnicity	Sex	Age	Distribution
	White	Women	56	Beta(8.85, 158)
	White	Women	57	Beta(8.82, 147)
	White	Women	58	Beta(8.8, 138)
	White	Women	59	Beta(8.79, 130)
	White	Women	60	Beta(8.78, 122)
	White	Women	61	Beta(8.77, 115)
	White	Women	62	Beta(8.75, 108)
	White	Women	63	Beta(8.71, 102)
	White	Women	64	Beta(8.71, 96.2)
	White	Women	65	Beta(8.68, 90.7)
	White	Women	66	Beta(8.67, 85.8)
	White	Women	67	Beta(8.65, 81.1)
	White	Women	68	Beta(8.63, 76.8)
	White	Women	69	Beta(8.61, 72.7)
	White	Women	70	Beta(8.58, 68.9)
	White	Women	71	Beta(7.63, 58.1)
	White	Women	72	Beta(8.54, 62.2)
	White	Women	73	Beta(8.52, 59.2)
	White	Women	74	Beta(8.5, 56.4)
	White	Women	75	Beta(8.48, 53.8)
	White	Women	76	Beta(8.46, 51.3)
	White	Women	77	Beta(8.44, 49.1)
	White	Women	78	Beta(8.42, 47.1)
	White	Women	79	Beta(8.38, 45.2)
	White	Women	80	Beta(8.39, 43.7)
	White	Women	81	Beta(8.36, 42.2)
	White	Women	82	Beta(8.36, 41)
	White	Women	83	Beta(8.35, 39.9)
	White	Women	84	Beta(8.34, 39)
	Black	Men	30	Beta(9.07, 929)
	Black	Men	31	Beta(9.07, 820)
	Black	Men	32	Beta(9.06, 728)
	Black	Men	33	Beta(9.05, 649)
	Black	Men	34	Beta(9.04, 580)
	Black	Men	35	Beta(9.04, 521)
	Black	Men	36	Beta(9.03, 469)
	Black	Men	37	Beta(9.01, 423)
	Black	Men	38	Beta(8.98, 382)
	Black	Men	39	Beta(8.99, 348)
	Black	Men	40	Beta(8.98, 317)
	Black	Men	41	Beta(8.97, 289)
	Black	Men	42	Beta(8.89, 262)
	Black	Men	43	Beta(8.94, 242)

Variable	Race/ethnicity	Sex	Age	Distribution
	Black	Men	44	Beta(8.92, 223)
	Black	Men	45	Beta(8.87, 204)
	Black	Men	46	Beta(8.87, 188)
	Black	Men	47	Beta(8.86, 174)
	Black	Men	48	Beta(8.85, 161)
	Black	Men	49	Beta(8.67, 146)
	Black	Men	50	Beta(8.8, 138)
	Black	Men	51	Beta(8.8, 129)
	Black	Men	52	Beta(8.72, 119)
	Black	Men	53	Beta(8.7, 111)
	Black	Men	54	Beta(8.69, 104)
	Black	Men	55	Beta(8.71, 97.5)
	Black	Men	56	Beta(8.69, 91.3)
	Black	Men	57	Beta(8.67, 85.6)
	Black	Men	58	Beta(8.65, 80.3)
	Black	Men	59	Beta(8.62, 75.5)
	Black	Men	60	Beta(8.6, 71)
	Black	Men	61	Beta(8.57, 66.8)
	Black	Men	62	Beta(8.55, 63)
	Black	Men	63	Beta(8.53, 59.4)
	Black	Men	64	Beta(8.5, 56.1)
	Black	Men	65	Beta(8.48, 52.9)
	Black	Men	66	Beta(8.45, 49.9)
	Black	Men	67	Beta(8.42, 47.2)
	Black	Men	68	Beta(8.38, 44.5)
	Black	Men	69	Beta(8.36, 42.2)
	Black	Men	70	Beta(8.35, 39.9)
	Black	Men	71	Beta(8.08, 36.7)
	Black	Men	72	Beta(8.3, 35.9)
	Black	Men	73	Beta(8.27, 34.1)
	Black	Men	74	Beta(8.25, 32.4)
	Black	Men	75	Beta(8.23, 30.9)
	Black	Men	76	Beta(8.2, 29.5)
	Black	Men	77	Beta(8.15, 28)
	Black	Men	78	Beta(8.11, 26.8)
	Black	Men	79	Beta(8.09, 25.7)
	Black	Men	80	Beta(8.08, 24.6)
	Black	Men	81	Beta(8.06, 23.7)
	Black	Men	82	Beta(8.05, 22.8)
	Black	Men	83	Beta(8.03, 22.1)
	Black	Men	84	Beta(8.02, 21.5)
	Black	Women	30	Beta(9.06, 871)
	Black	Women	31	Beta(9.07, 786)

Variable	Race/ethnicity	Sex	Age	Distribution
	Black	Women	32	Beta(9.05, 711)
	Black	Women	33	Beta(9.05, 646)
	Black	Women	34	Beta(9.05, 589)
	Black	Women	35	Beta(9.01, 537)
	Black	Women	36	Beta(9.02, 493)
	Black	Women	37	Beta(9.02, 454)
	Black	Women	38	Beta(9.01, 418)
	Black	Women	39	Beta(9, 385)
	Black	Women	40	Beta(9, 356)
	Black	Women	41	Beta(8.98, 329)
	Black	Women	42	Beta(8.95, 304)
	Black	Women	43	Beta(8.93, 282)
	Black	Women	44	Beta(8.95, 263)
	Black	Women	45	Beta(8.94, 245)
	Black	Women	46	Beta(8.93, 229)
	Black	Women	47	Beta(8.87, 212)
	Black	Women	48	Beta(8.87, 199)
	Black	Women	49	Beta(8.87, 187)
	Black	Women	50	Beta(8.86, 175)
	Black	Women	51	Beta(8.85, 165)
	Black	Women	52	Beta(8.85, 155)
	Black	Women	53	Beta(8.8, 145)
	Black	Women	54	Beta(8.81, 137)
	Black	Women	55	Beta(8.8, 129)
	Black	Women	56	Beta(8.75, 121)
	Black	Women	57	Beta(8.76, 115)
	Black	Women	58	Beta(8.75, 109)
	Black	Women	59	Beta(8.68, 102)
	Black	Women	60	Beta(8.71, 97.4)
	Black	Women	61	Beta(8.65, 91.8)
	Black	Women	62	Beta(8.68, 87.5)
	Black	Women	63	Beta(8.66, 83)
	Black	Women	64	Beta(8.64, 78.8)
	Black	Women	65	Beta(8.62, 74.8)
	Black	Women	66	Beta(8.6, 71.1)
	Black	Women	67	Beta(8.58, 67.6)
	Black	Women	68	Beta(8.56, 64.3)
	Black	Women	69	Beta(8.54, 61.3)
	Black	Women	70	Beta(8.52, 58.4)
	Black	Women	71	Beta(8.5, 55.8)
	Black	Women	72	Beta(8.48, 53.3)
	Black	Women	73	Beta(8.46, 51)
	Black	Women	74	Beta(8.43, 48.8)

Variable	Race/ethnicity	Sex	Age	Distribution
	Black	Women	75	Beta(8.39, 46.6)
	Black	Women	76	Beta(8.38, 44.7)
	Black	Women	77	Beta(8.38, 43)
	Black	Women	78	Beta(8.36, 41.4)
	Black	Women	79	Beta(8.35, 39.9)
	Black	Women	80	Beta(8.33, 38.6)
	Black	Women	81	Beta(8.32, 37.3)
	Black	Women	82	Beta(8.3, 36.2)
	Black	Women	83	Beta(8.29, 35.4)
	Black	Women	84	Beta(8.28, 34.9)
	Hispanic	Men	30	Beta(9.06, 962)
	Hispanic	Men	31	Beta(9.07, 847)
	Hispanic	Men	32	Beta(9.06, 749)
	Hispanic	Men	33	Beta(9.05, 664)
	Hispanic	Men	34	Beta(9.05, 591)
	Hispanic	Men	35	Beta(9.03, 528)
	Hispanic	Men	36	Beta(9.03, 474)
	Hispanic	Men	37	Beta(9.02, 426)
	Hispanic	Men	38	Beta(8.72, 371)
	Hispanic	Men	39	Beta(8.99, 347)
	Hispanic	Men	40	Beta(8.98, 315)
	Hispanic	Men	41	Beta(8.51, 271)
	Hispanic	Men	42	Beta(8.88, 258)
	Hispanic	Men	43	Beta(8.91, 236)
	Hispanic	Men	44	Beta(8.92, 217)
	Hispanic	Men	45	Beta(8.87, 198)
	Hispanic	Men	46	Beta(8.87, 182)
	Hispanic	Men	47	Beta(8.85, 167)
	Hispanic	Men	48	Beta(8.85, 154)
	Hispanic	Men	49	Beta(8.8, 142)
	Hispanic	Men	50	Beta(8.31, 123)
	Hispanic	Men	51	Beta(8.78, 121)
	Hispanic	Men	52	Beta(8.58, 110)
	Hispanic	Men	53	Beta(8.69, 104)
	Hispanic	Men	54	Beta(8.71, 96.6)
	Hispanic	Men	55	Beta(8.69, 89.7)
	Hispanic	Men	56	Beta(8.66, 83.4)
	Hispanic	Men	57	Beta(8.63, 77.7)
	Hispanic	Men	58	Beta(8.6, 72.3)
	Hispanic	Men	59	Beta(8.58, 67.5)
	Hispanic	Men	60	Beta(8.55, 63)
	Hispanic	Men	61	Beta(8.52, 58.9)
	Hispanic	Men	62	Beta(8.49, 55.1)

Variable	Race/ethnicity	Sex	Age	Distribution
	Hispanic	Men	63	Beta(8.3, 50.5)
	Hispanic	Men	64	Beta(8.44, 48.3)
	Hispanic	Men	65	Beta(8.38, 45.2)
	Hispanic	Men	66	Beta(8.36, 42.4)
	Hispanic	Men	67	Beta(8.35, 39.9)
	Hispanic	Men	68	Beta(8.32, 37.5)
	Hispanic	Men	69	Beta(8.29, 35.2)
	Hispanic	Men	70	Beta(8.26, 33.1)
	Hispanic	Men	71	Beta(8.23, 31.2)
	Hispanic	Men	72	Beta(8.2, 29.4)
	Hispanic	Men	73	Beta(8.12, 27.6)
	Hispanic	Men	74	Beta(8.1, 26.1)
	Hispanic	Men	75	Beta(8.08, 24.7)
	Hispanic	Men	76	Beta(8.06, 23.4)
	Hispanic	Men	77	Beta(8.04, 22.2)
	Hispanic	Men	78	Beta(8.01, 21.1)
	Hispanic	Men	79	Beta(7.99, 20)
	Hispanic	Men	80	Beta(7.97, 19.1)
	Hispanic	Men	81	Beta(7.95, 18.2)
	Hispanic	Men	82	Beta(7.92, 17.4)
	Hispanic	Men	83	Beta(7.9, 16.7)
	Hispanic	Men	84	Beta(7.88, 16.1)
	Hispanic	Women	30	Beta(9.08, 1070)
	Hispanic	Women	31	Beta(9.06, 961)
	Hispanic	Women	32	Beta(9.07, 867)
	Hispanic	Women	33	Beta(9.07, 783)
	Hispanic	Women	34	Beta(9.05, 709)
	Hispanic	Women	35	Beta(9.05, 645)
	Hispanic	Women	36	Beta(9.05, 588)
	Hispanic	Women	37	Beta(9.01, 535)
	Hispanic	Women	38	Beta(9.03, 491)
	Hispanic	Women	39	Beta(9.02, 450)
	Hispanic	Women	40	Beta(8.95, 411)
	Hispanic	Women	41	Beta(9, 381)
	Hispanic	Women	42	Beta(8.99, 351)
	Hispanic	Women	43	Beta(8.98, 324)
	Hispanic	Women	44	Beta(8.93, 299)
	Hispanic	Women	45	Beta(8.96, 278)
	Hispanic	Women	46	Beta(8.87, 255)
	Hispanic	Women	47	Beta(8.91, 238)
	Hispanic	Women	48	Beta(8.92, 222)
	Hispanic	Women	49	Beta(8.87, 206)
	Hispanic	Women	50	Beta(8.87, 192)

Variable	Race/ethnicity	Sex	Age	Distribution
	Hispanic	Women	51	Beta(8.87, 179)
	Hispanic	Women	52	Beta(8.85, 167)
	Hispanic	Women	53	Beta(8.85, 157)
	Hispanic	Women	54	Beta(8.8, 146)
	Hispanic	Women	55	Beta(8.81, 137)
	Hispanic	Women	56	Beta(8.8, 128)
	Hispanic	Women	57	Beta(8.72, 119)
	Hispanic	Women	58	Beta(8.76, 113)
	Hispanic	Women	59	Beta(8.72, 105)
	Hispanic	Women	60	Beta(8.72, 99.3)
	Hispanic	Women	61	Beta(8.65, 92.8)
	Hispanic	Women	62	Beta(8.68, 87.8)
	Hispanic	Women	63	Beta(8.66, 82.6)
	Hispanic	Women	64	Beta(8.63, 77.8)
	Hispanic	Women	65	Beta(8.61, 73.3)
	Hispanic	Women	66	Beta(8.59, 69.1)
	Hispanic	Women	67	Beta(8.56, 65.2)
	Hispanic	Women	68	Beta(8.54, 61.6)
	Hispanic	Women	69	Beta(8.52, 58.2)
	Hispanic	Women	70	Beta(8.49, 55)
	Hispanic	Women	71	Beta(8.47, 52.1)
	Hispanic	Women	72	Beta(8.44, 49.2)
	Hispanic	Women	73	Beta(8.42, 46.7)
	Hispanic	Women	74	Beta(8.4, 44.3)
	Hispanic	Women	75	Beta(8.37, 42)
	Hispanic	Women	76	Beta(8.35, 39.9)
	Hispanic	Women	77	Beta(8.32, 38)
	Hispanic	Women	78	Beta(8.3, 36.2)
	Hispanic	Women	79	Beta(8.28, 34.6)
	Hispanic	Women	80	Beta(8.26, 33.1)
	Hispanic	Women	81	Beta(8.24, 31.6)
	Hispanic	Women	82	Beta(8.22, 30.4)
	Hispanic	Women	83	Beta(8.2, 29.3)
	Hispanic	Women	84	Beta(8.17, 28.4)
Stroke prevalence rate for 2014				
	White	Men	30	Beta(9.07, 4340)
	White	Men	31	Beta(9.11, 3930)
	White	Men	32	Beta(9.11, 3550)
	White	Men	33	Beta(9.11, 3200)
	White	Men	34	Beta(9.11, 2890)
	White	Men	35	Beta(9.11, 2610)
	White	Men	36	Beta(9.11, 2360)
	White	Men	37	Beta(9.1, 2130)

Variable	Race/ethnicity	Sex	Age	Distribution
	White	Men	38	Beta(9.1, 1930)
	White	Men	39	Beta(9.07, 1740)
	White	Men	40	Beta(9.1, 1590)
	White	Men	41	Beta(9.09, 1440)
	White	Men	42	Beta(9.09, 1300)
	White	Men	43	Beta(9.09, 1180)
	White	Men	44	Beta(9.08, 1070)
	White	Men	45	Beta(9.07, 975)
	White	Men	46	Beta(9, 880)
	White	Men	47	Beta(9.07, 808)
	White	Men	48	Beta(9.05, 735)
	White	Men	49	Beta(9.02, 669)
	White	Men	50	Beta(8.97, 607)
	White	Men	51	Beta(8.96, 553)
	White	Men	52	Beta(8.92, 503)
	White	Men	53	Beta(9.03, 466)
	White	Men	54	Beta(9.02, 426)
	White	Men	55	Beta(8.83, 382)
	White	Men	56	Beta(9, 357)
	White	Men	57	Beta(8.98, 327)
	White	Men	58	Beta(8.93, 299)
	White	Men	59	Beta(8.96, 275)
	White	Men	60	Beta(8.91, 252)
	White	Men	61	Beta(8.91, 232)
	White	Men	62	Beta(8.87, 212)
	White	Men	63	Beta(8.87, 196)
	White	Men	64	Beta(8.87, 181)
	White	Men	65	Beta(8.85, 166)
	White	Men	66	Beta(8.83, 153)
	White	Men	67	Beta(8.8, 141)
	White	Men	68	Beta(8.8, 131)
	White	Men	69	Beta(8.74, 120)
	White	Men	70	Beta(8.7, 111)
	White	Men	71	Beta(8.68, 103)
	White	Men	72	Beta(8.71, 95.6)
	White	Men	73	Beta(8.68, 88.6)
	White	Men	74	Beta(8.65, 82.2)
	White	Men	75	Beta(8.63, 76.3)
	White	Men	76	Beta(8.6, 70.9)
	White	Men	77	Beta(8.57, 65.9)
	White	Men	78	Beta(8.54, 61.4)
	White	Men	79	Beta(8.51, 57.2)
	White	Men	80	Beta(8.48, 53.4)

Variable	Race/ethnicity	Sex	Age	Distribution
	White	Men	81	Beta(8.39, 49.5)
	White	Men	82	Beta(8.4, 46.5)
	White	Men	83	Beta(8.39, 43.6)
	White	Men	84	Beta(8.36, 40.8)
	White	Women	30	Beta(9.09, 1560)
	White	Women	31	Beta(9.09, 1450)
	White	Women	32	Beta(9, 1340)
	White	Women	33	Beta(9.09, 1260)
	White	Women	34	Beta(9.09, 1180)
	White	Women	35	Beta(9.08, 1100)
	White	Women	36	Beta(9.08, 1030)
	White	Women	37	Beta(9.03, 953)
	White	Women	38	Beta(8.94, 880)
	White	Women	39	Beta(9.06, 834)
	White	Women	40	Beta(9.06, 780)
	White	Women	41	Beta(9.06, 729)
	White	Women	42	Beta(9.06, 681)
	White	Women	43	Beta(9.05, 637)
	White	Women	44	Beta(9.05, 596)
	White	Women	45	Beta(9.04, 558)
	White	Women	46	Beta(9.04, 522)
	White	Women	47	Beta(9.03, 489)
	White	Women	48	Beta(9.02, 458)
	White	Women	49	Beta(8.87, 422)
	White	Women	50	Beta(8.93, 398)
	White	Women	51	Beta(9, 377)
	White	Women	52	Beta(8.99, 354)
	White	Women	53	Beta(8.98, 331)
	White	Women	54	Beta(8.98, 311)
	White	Women	55	Beta(8.94, 291)
	White	Women	56	Beta(8.96, 274)
	White	Women	57	Beta(8.87, 255)
	White	Women	58	Beta(8.94, 242)
	White	Women	59	Beta(8.93, 227)
	White	Women	60	Beta(8.87, 212)
	White	Women	61	Beta(8.87, 200)
	White	Women	62	Beta(8.87, 188)
	White	Women	63	Beta(8.86, 177)
	White	Women	64	Beta(8.85, 167)
	White	Women	65	Beta(8.85, 157)
	White	Women	66	Beta(8.82, 148)
	White	Women	67	Beta(8.8, 139)
	White	Women	68	Beta(8.67, 129)

Variable	Race/ethnicity	Sex	Age	Distribution
	White	Women	69	Beta(8.76, 123)
	White	Women	70	Beta(8.77, 117)
	White	Women	71	Beta(8.75, 110)
	White	Women	72	Beta(8.68, 103)
	White	Women	73	Beta(8.71, 97.7)
	White	Women	74	Beta(8.68, 92)
	White	Women	75	Beta(8.67, 86.9)
	White	Women	76	Beta(8.65, 82.1)
	White	Women	77	Beta(8.63, 77.5)
	White	Women	78	Beta(8.61, 73.2)
	White	Women	79	Beta(8.59, 69.1)
	White	Women	80	Beta(8.57, 65.3)
	White	Women	81	Beta(8.54, 61.8)
	White	Women	82	Beta(8.52, 58.4)
	White	Women	83	Beta(8.49, 55.2)
	White	Women	84	Beta(8.47, 52.2)
	Black	Men	30	Beta(9.1, 1600)
	Black	Men	31	Beta(9.08, 1460)
	Black	Men	32	Beta(9.04, 1320)
	Black	Men	33	Beta(9.09, 1220)
	Black	Men	34	Beta(9.08, 1120)
	Black	Men	35	Beta(9.08, 1020)
	Black	Men	36	Beta(9.07, 938)
	Black	Men	37	Beta(9.07, 861)
	Black	Men	38	Beta(9.07, 791)
	Black	Men	39	Beta(9.06, 726)
	Black	Men	40	Beta(9.06, 667)
	Black	Men	41	Beta(9.05, 614)
	Black	Men	42	Beta(9.04, 566)
	Black	Men	43	Beta(9.04, 522)
	Black	Men	44	Beta(9.03, 482)
	Black	Men	45	Beta(9.02, 445)
	Black	Men	46	Beta(8.97, 409)
	Black	Men	47	Beta(9, 380)
	Black	Men	48	Beta(8.99, 352)
	Black	Men	49	Beta(8.98, 326)
	Black	Men	50	Beta(8.96, 302)
	Black	Men	51	Beta(8.96, 281)
	Black	Men	52	Beta(8.88, 258)
	Black	Men	53	Beta(8.94, 242)
	Black	Men	54	Beta(8.91, 225)
	Black	Men	55	Beta(8.87, 209)
	Black	Men	56	Beta(8.87, 195)

Variable	Race/ethnicity	Sex	Age	Distribution
	Black	Men	57	Beta(8.87, 182)
	Black	Men	58	Beta(8.85, 170)
	Black	Men	59	Beta(8.85, 159)
	Black	Men	60	Beta(8.82, 148)
	Black	Men	61	Beta(8.8, 138)
	Black	Men	62	Beta(8.8, 130)
	Black	Men	63	Beta(8.78, 121)
	Black	Men	64	Beta(8.76, 113)
	Black	Men	65	Beta(8.74, 106)
	Black	Men	66	Beta(8.72, 99.2)
	Black	Men	67	Beta(8.65, 92.3)
	Black	Men	68	Beta(8.67, 86.9)
	Black	Men	69	Beta(8.65, 81.3)
	Black	Men	70	Beta(8.63, 76.2)
	Black	Men	71	Beta(8.6, 71.4)
	Black	Men	72	Beta(8.57, 67)
	Black	Men	73	Beta(8.55, 62.9)
	Black	Men	74	Beta(8.52, 59.1)
	Black	Men	75	Beta(8.5, 55.6)
	Black	Men	76	Beta(8.47, 52.2)
	Black	Men	77	Beta(8.36, 48.5)
	Black	Men	78	Beta(8.38, 45.9)
	Black	Men	79	Beta(8.39, 43.3)
	Black	Men	80	Beta(8.33, 40.6)
	Black	Men	81	Beta(8.33, 38.3)
	Black	Men	82	Beta(8.27, 36)
	Black	Men	83	Beta(8.27, 34)
	Black	Men	84	Beta(8.25, 32.2)
	Black	Women	30	Beta(9.1, 2660)
	Black	Women	31	Beta(9.11, 2460)
	Black	Women	32	Beta(9.09, 2270)
	Black	Women	33	Beta(9.1, 2110)
	Black	Women	34	Beta(9.1, 1960)
	Black	Women	35	Beta(9.1, 1820)
	Black	Women	36	Beta(9.1, 1690)
	Black	Women	37	Beta(9.08, 1570)
	Black	Women	38	Beta(9.08, 1460)
	Black	Women	39	Beta(9.08, 1360)
	Black	Women	40	Beta(9.09, 1270)
	Black	Women	41	Beta(9.09, 1180)
	Black	Women	42	Beta(9.08, 1100)
	Black	Women	43	Beta(9.08, 1030)
	Black	Women	44	Beta(9.06, 959)

Variable	Race/ethnicity	Sex	Age	Distribution
	Black	Women	45	Beta(9.07, 898)
	Black	Women	46	Beta(9.06, 839)
	Black	Women	47	Beta(8.9, 769)
	Black	Women	48	Beta(9.05, 732)
	Black	Women	49	Beta(9.06, 685)
	Black	Women	50	Beta(9.05, 641)
	Black	Women	51	Beta(9.05, 600)
	Black	Women	52	Beta(9.04, 562)
	Black	Women	53	Beta(9.03, 526)
	Black	Women	54	Beta(9.03, 492)
	Black	Women	55	Beta(9.02, 460)
	Black	Women	56	Beta(9.02, 429)
	Black	Women	57	Beta(8.72, 387)
	Black	Women	58	Beta(9, 374)
	Black	Women	59	Beta(8.99, 349)
	Black	Women	60	Beta(8.98, 326)
	Black	Women	61	Beta(8.95, 303)
	Black	Women	62	Beta(8.96, 284)
	Black	Women	63	Beta(8.95, 265)
	Black	Women	64	Beta(8.89, 246)
	Black	Women	65	Beta(8.91, 230)
	Black	Women	66	Beta(8.87, 213)
	Black	Women	67	Beta(8.87, 199)
	Black	Women	68	Beta(8.87, 186)
	Black	Women	69	Beta(8.86, 173)
	Black	Women	70	Beta(8.85, 162)
	Black	Women	71	Beta(8.68, 148)
	Black	Women	72	Beta(8.8, 141)
	Black	Women	73	Beta(8.8, 132)
	Black	Women	74	Beta(8.79, 123)
	Black	Women	75	Beta(8.77, 115)
	Black	Women	76	Beta(8.75, 107)
	Black	Women	77	Beta(8.7, 100)
	Black	Women	78	Beta(8.65, 93)
	Black	Women	79	Beta(8.68, 87.4)
	Black	Women	80	Beta(8.65, 81.7)
	Black	Women	81	Beta(8.63, 76.5)
	Black	Women	82	Beta(8.6, 71.6)
	Black	Women	83	Beta(8.58, 67.4)
	Black	Women	84	Beta(8.55, 63.8)
	Hispanic	Men	30	Beta(9.09, 1510)
	Hispanic	Men	31	Beta(9.09, 1390)
	Hispanic	Men	32	Beta(9.08, 1290)

Variable	Race/ethnicity	Sex	Age	Distribution
	Hispanic	Men	33	Beta(9.07, 1180)
	Hispanic	Men	34	Beta(8.81, 1060)
	Hispanic	Men	35	Beta(8.95, 999)
	Hispanic	Men	36	Beta(9.07, 938)
	Hispanic	Men	37	Beta(9.02, 863)
	Hispanic	Men	38	Beta(9.07, 804)
	Hispanic	Men	39	Beta(9.06, 745)
	Hispanic	Men	40	Beta(9.06, 691)
	Hispanic	Men	41	Beta(9.05, 641)
	Hispanic	Men	42	Beta(9.04, 594)
	Hispanic	Men	43	Beta(9.04, 552)
	Hispanic	Men	44	Beta(9.03, 512)
	Hispanic	Men	45	Beta(8.94, 471)
	Hispanic	Men	46	Beta(9.02, 442)
	Hispanic	Men	47	Beta(8.97, 409)
	Hispanic	Men	48	Beta(9, 382)
	Hispanic	Men	49	Beta(9, 356)
	Hispanic	Men	50	Beta(8.98, 331)
	Hispanic	Men	51	Beta(8.97, 308)
	Hispanic	Men	52	Beta(8.57, 274)
	Hispanic	Men	53	Beta(8.92, 267)
	Hispanic	Men	54	Beta(8.94, 250)
	Hispanic	Men	55	Beta(8.9, 232)
	Hispanic	Men	56	Beta(8.92, 218)
	Hispanic	Men	57	Beta(8.87, 202)
	Hispanic	Men	58	Beta(8.87, 189)
	Hispanic	Men	59	Beta(8.86, 177)
	Hispanic	Men	60	Beta(8.85, 165)
	Hispanic	Men	61	Beta(8.85, 154)
	Hispanic	Men	62	Beta(8.79, 144)
	Hispanic	Men	63	Beta(8.74, 134)
	Hispanic	Men	64	Beta(8.79, 126)
	Hispanic	Men	65	Beta(8.77, 118)
	Hispanic	Men	66	Beta(8.75, 110)
	Hispanic	Men	67	Beta(8.68, 102)
	Hispanic	Men	68	Beta(8.71, 96.1)
	Hispanic	Men	69	Beta(8.68, 89.9)
	Hispanic	Men	70	Beta(8.66, 84.1)
	Hispanic	Men	71	Beta(8.64, 78.8)
	Hispanic	Men	72	Beta(8.61, 73.8)
	Hispanic	Men	73	Beta(8.59, 69.1)
	Hispanic	Men	74	Beta(8.56, 64.7)
	Hispanic	Men	75	Beta(8.53, 60.6)

Variable	Race/ethnicity	Sex	Age	Distribution
	Hispanic	Men	76	Beta(8.51, 56.9)
	Hispanic	Men	77	Beta(8.48, 53.3)
	Hispanic	Men	78	Beta(8.45, 50)
	Hispanic	Men	79	Beta(8.38, 46.8)
	Hispanic	Men	80	Beta(8.39, 44.1)
	Hispanic	Men	81	Beta(8.36, 41.5)
	Hispanic	Men	82	Beta(8.34, 39)
	Hispanic	Men	83	Beta(8.31, 36.8)
	Hispanic	Men	84	Beta(8.28, 34.7)
	Hispanic	Women	30	Beta(9.11, 3140)
	Hispanic	Women	31	Beta(9.11, 2890)
	Hispanic	Women	32	Beta(9.11, 2670)
	Hispanic	Women	33	Beta(9.1, 2460)
	Hispanic	Women	34	Beta(9.11, 2270)
	Hispanic	Women	35	Beta(8.87, 2040)
	Hispanic	Women	36	Beta(9.1, 1940)
	Hispanic	Women	37	Beta(9.1, 1790)
	Hispanic	Women	38	Beta(9.1, 1660)
	Hispanic	Women	39	Beta(9.09, 1540)
	Hispanic	Women	40	Beta(9.09, 1430)
	Hispanic	Women	41	Beta(9.09, 1320)
	Hispanic	Women	42	Beta(9.08, 1230)
	Hispanic	Women	43	Beta(9.08, 1140)
	Hispanic	Women	44	Beta(9.08, 1060)
	Hispanic	Women	45	Beta(9.08, 984)
	Hispanic	Women	46	Beta(9.06, 913)
	Hispanic	Women	47	Beta(9.07, 850)
	Hispanic	Women	48	Beta(9.07, 790)
	Hispanic	Women	49	Beta(9.05, 733)
	Hispanic	Women	50	Beta(9.06, 683)
	Hispanic	Women	51	Beta(9.05, 634)
	Hispanic	Women	52	Beta(8.87, 578)
	Hispanic	Women	53	Beta(9.03, 547)
	Hispanic	Women	54	Beta(9.03, 509)
	Hispanic	Women	55	Beta(9.03, 473)
	Hispanic	Women	56	Beta(9.02, 440)
	Hispanic	Women	57	Beta(9.02, 410)
	Hispanic	Women	58	Beta(9, 381)
	Hispanic	Women	59	Beta(9, 354)
	Hispanic	Women	60	Beta(8.98, 329)
	Hispanic	Women	61	Beta(8.96, 306)
	Hispanic	Women	62	Beta(8.97, 285)
	Hispanic	Women	63	Beta(8.95, 266)

Variable	Race/ethnicity	Sex	Age	Distribution
	Hispanic	Women	64	Beta(8.88, 245)
	Hispanic	Women	65	Beta(8.92, 230)
	Hispanic	Women	66	Beta(8.87, 213)
	Hispanic	Women	67	Beta(8.87, 199)
	Hispanic	Women	68	Beta(8.87, 185)
	Hispanic	Women	69	Beta(8.86, 173)
	Hispanic	Women	70	Beta(8.85, 161)
	Hispanic	Women	71	Beta(8.84, 150)
	Hispanic	Women	72	Beta(8.8, 140)
	Hispanic	Women	73	Beta(8.8, 131)
	Hispanic	Women	74	Beta(8.78, 122)
	Hispanic	Women	75	Beta(8.76, 114)
	Hispanic	Women	76	Beta(8.74, 107)
	Hispanic	Women	77	Beta(8.72, 99.8)
	Hispanic	Women	78	Beta(8.65, 92.9)
	Hispanic	Women	79	Beta(8.68, 87.5)
	Hispanic	Women	80	Beta(8.65, 82.1)
	Hispanic	Women	81	Beta(8.63, 77)
	Hispanic	Women	82	Beta(8.61, 72.4)
	Hispanic	Women	83	Beta(8.58, 68.4)
	Hispanic	Women	84	Beta(8.56, 64.9)
	Black	Men	57	0.00149, 0.00177, 0.0021, 0.0025, 0.00297
	Black	Men	58	0.00166, 0.00195, 0.0023, 0.0027, 0.00318
	Black	Men	59	0.00183, 0.00214, 0.00249, 0.00291, 0.00339
	Black	Men	60	0.00201, 0.00232, 0.00269, 0.00311, 0.00361
	Black	Men	61	0.00219, 0.00252, 0.00289, 0.00332, 0.00381
	Black	Men	62	0.00239, 0.00272, 0.00309, 0.00351, 0.004
	Black	Men	63	0.00258, 0.00291, 0.00328, 0.0037, 0.00418
	Black	Men	64	0.00276, 0.0031, 0.00347, 0.00389, 0.00437
	Black	Men	65	0.00294, 0.00329, 0.00367, 0.0041, 0.00459
	Black	Men	66	0.0031, 0.00347, 0.00389, 0.00434, 0.00486
	Black	Men	67	0.00327, 0.00367, 0.00412, 0.00462, 0.0052
	Black	Men	68	0.00345, 0.00389, 0.00439, 0.00495, 0.00558
	Black	Men	69	0.00365, 0.00414, 0.00468, 0.0053, 0.00601
	Black	Men	70	0.00389, 0.00442, 0.00501, 0.00569, 0.00646
	Black	Men	71	0.00416, 0.00473, 0.00537, 0.0061, 0.00694
	Black	Men	72	0.00445, 0.00507, 0.00577, 0.00656, 0.00747
	Black	Men	73	0.00476, 0.00543, 0.00619, 0.00706, 0.00806
	Black	Men	74	0.00509, 0.00582, 0.00665, 0.00759, 0.00868
	Black	Men	75	0.00548, 0.00626, 0.00715, 0.00816, 0.00933
	Black	Men	76	0.00594, 0.00676, 0.0077, 0.00876, 0.00999
	Black	Men	77	0.00645, 0.00732, 0.00831, 0.00942, 0.0107

Variable	Race/ethnicity	Sex	Age	Distribution
	Black	Men	78	0.00699, 0.00793, 0.00898, 0.0102, 0.0115
	Black	Men	79	0.00755, 0.00857, 0.00972, 0.011, 0.0125
	Black	Men	80	0.00812, 0.00925, 0.0105, 0.012, 0.0137
	Black	Men	81	0.00866, 0.00995, 0.0114, 0.0131, 0.015
	Black	Men	82	0.00916, 0.0106, 0.0123, 0.0143, 0.0165
	Black	Men	83	0.00959, 0.0113, 0.0132, 0.0155, 0.0182
	Black	Men	84	0.01, 0.0119, 0.0141, 0.0168, 0.02
	Black	Women	30	0.0000148, 0.0000198, 0.0000263, 0.000035, 0.0000467
	Black	Women	31	0.0000186, 0.000024, 0.000031, 0.00004, 0.0000517
	Black	Women	32	0.0000228, 0.0000288, 0.0000364, 0.0000459, 0.0000581
	Black	Women	33	0.0000277, 0.0000344, 0.0000427, 0.0000529, 0.0000658
	Black	Women	34	0.000033, 0.0000406, 0.0000499, 0.0000612, 0.0000753
	Black	Women	35	0.0000385, 0.0000473, 0.0000579, 0.0000711, 0.0000873
	Black	Women	36	0.000044, 0.0000543, 0.0000669, 0.0000824, 0.000102
	Black	Women	37	0.0000501, 0.0000621, 0.0000767, 0.0000948, 0.000117
	Black	Women	38	0.0000566, 0.0000705, 0.0000874, 0.000109, 0.000135
	Black	Women	39	0.0000637, 0.0000796, 0.0000992, 0.000124, 0.000154
	Black	Women	40	0.0000721, 0.00009, 0.000112, 0.000139, 0.000174
	Black	Women	41	0.0000826, 0.000102, 0.000126, 0.000156, 0.000193
	Black	Women	42	0.0000956, 0.000117, 0.000142, 0.000173, 0.000212
	Black	Women	43	0.000111, 0.000134, 0.000161, 0.000193, 0.000232
	Black	Women	44	0.000129, 0.000153, 0.000182, 0.000216, 0.000257
	Black	Women	45	0.000148, 0.000175, 0.000207, 0.000244, 0.000289
	Black	Women	46	0.000171, 0.000201, 0.000236, 0.000277, 0.000326
	Black	Women	47	0.000197, 0.000231, 0.00027, 0.000315, 0.000369
	Black	Women	48	0.000225, 0.000264, 0.000309, 0.000361, 0.000423
	Black	Women	49	0.000251, 0.000298, 0.000353, 0.000418, 0.000495
	Black	Women	50	0.00028, 0.000336, 0.000402, 0.00048, 0.000575
	Black	Women	51	0.000321, 0.000383, 0.000455, 0.000541, 0.000645
	Black	Women	52	0.000373, 0.000438, 0.000513, 0.000601, 0.000706
	Black	Women	53	0.000424, 0.000494, 0.000576, 0.000671, 0.000782
	Black	Women	54	0.000469, 0.00055, 0.000644, 0.000753, 0.000883
	Black	Women	55	0.000521, 0.000612, 0.000718, 0.000841, 0.000988
	Black	Women	56	0.000586, 0.000684, 0.000797, 0.00093, 0.00109
	Black	Women	57	0.000657, 0.000762, 0.000882, 0.00102, 0.00119
	Black	Women	58	0.000727, 0.000841, 0.000972, 0.00112, 0.0013
	Black	Women	59	0.000792, 0.000919, 0.00106, 0.00123, 0.00143
	Black	Women	60	0.000855, 0.000997, 0.00116, 0.00135, 0.00157
	Black	Women	61	0.000915, 0.00107, 0.00126, 0.00147, 0.00173
	Black	Women	62	0.000971, 0.00115, 0.00135, 0.0016, 0.00189
	Black	Women	63	0.00102, 0.00122, 0.00145, 0.00173, 0.00207
	Black	Women	64	0.00107, 0.00129, 0.00156, 0.00188, 0.00226
	Black	Women	65	0.00112, 0.00136, 0.00166, 0.00203, 0.00248

Variable	Race/ethnicity	Sex	Age	Distribution
	Black	Women	66	0.00117, 0.00145, 0.00179, 0.0022, 0.00272
	Black	Women	67	0.00125, 0.00155, 0.00193, 0.00239, 0.00298
	Black	Women	68	0.00135, 0.00168, 0.00209, 0.00261, 0.00326
	Black	Women	69	0.00147, 0.00184, 0.00229, 0.00285, 0.00356
	Black	Women	70	0.00163, 0.00202, 0.00251, 0.00311, 0.00387
	Black	Women	71	0.0018, 0.00223, 0.00275, 0.00339, 0.0042
	Black	Women	72	0.00198, 0.00244, 0.00301, 0.0037, 0.00456
	Black	Women	73	0.00218, 0.00268, 0.00329, 0.00403, 0.00496
	Black	Women	74	0.00238, 0.00293, 0.00359, 0.0044, 0.0054
	Black	Women	75	0.0026, 0.00319, 0.00392, 0.0048, 0.00591
	Black	Women	76	0.00282, 0.00348, 0.00428, 0.00527, 0.00651
	Black	Women	77	0.00304, 0.00378, 0.00469, 0.00582, 0.00724
	Black	Women	78	0.00327, 0.00411, 0.00515, 0.00645, 0.0081
	Black	Women	79	0.00351, 0.00446, 0.00566, 0.00717, 0.00911
	Black	Women	80	0.00378, 0.00485, 0.00622, 0.00797, 0.0102
	Black	Women	81	0.00408, 0.00528, 0.00683, 0.00883, 0.0114
	Black	Women	82	0.00442, 0.00576, 0.00748, 0.00972, 0.0127
	Black	Women	83	0.0048, 0.00627, 0.00816, 0.0106, 0.0139
	Black	Women	84	0.0052, 0.0068, 0.00887, 0.0116, 0.0151
	Hispanic	Men	30	0.0000124, 0.0000164, 0.0000215, 0.0000282, 0.0000372
	Hispanic	Men	31	0.0000151, 0.0000196, 0.0000255, 0.0000331, 0.0000431
	Hispanic	Men	32	0.0000182, 0.0000235, 0.0000301, 0.0000387, 0.0000497
	Hispanic	Men	33	0.0000223, 0.0000282, 0.0000355, 0.0000448, 0.0000566
	Hispanic	Men	34	0.0000276, 0.000034, 0.0000418, 0.0000515, 0.0000634
	Hispanic	Men	35	0.0000341, 0.0000409, 0.0000491, 0.0000589, 0.0000708
	Hispanic	Men	36	0.0000414, 0.0000488, 0.0000574, 0.0000676, 0.0000798
	Hispanic	Men	37	0.0000492, 0.0000574, 0.000067, 0.000078, 0.0000911
	Hispanic	Men	38	0.0000578, 0.0000671, 0.0000779, 0.0000903, 0.000105
	Hispanic	Men	39	0.0000677, 0.0000783, 0.0000904, 0.000104, 0.000121
	Hispanic	Men	40	0.0000794, 0.0000913, 0.000105, 0.00012, 0.000138
	Hispanic	Men	41	0.0000924, 0.000106, 0.000121, 0.000139, 0.000159
	Hispanic	Men	42	0.000107, 0.000122, 0.00014, 0.00016, 0.000183
	Hispanic	Men	43	0.000123, 0.000141, 0.000161, 0.000184, 0.000211
	Hispanic	Men	44	0.000143, 0.000163, 0.000185, 0.000211, 0.00024
	Hispanic	Men	45	0.000166, 0.000188, 0.000213, 0.000242, 0.000274
	Hispanic	Men	46	0.00019, 0.000216, 0.000246, 0.000279, 0.000318
	Hispanic	Men	47	0.000217, 0.000248, 0.000283, 0.000323, 0.000368
	Hispanic	Men	48	0.000253, 0.000287, 0.000326, 0.00037, 0.00042
	Hispanic	Men	49	0.000295, 0.000333, 0.000375, 0.000423, 0.000477
	Hispanic	Men	50	0.000338, 0.000382, 0.000431, 0.000486, 0.000549
	Hispanic	Men	51	0.000384, 0.000435, 0.000493, 0.000559, 0.000634
	Hispanic	Men	52	0.000434, 0.000494, 0.000562, 0.000639, 0.000728
	Hispanic	Men	53	0.000488, 0.000558, 0.000637, 0.000727, 0.000831

Variable	Race/ethnicity	Sex	Age	Distribution
	Hispanic	Men	54	0.000545, 0.000625, 0.000716, 0.000821, 0.000941
	Hispanic	Men	55	0.00061, 0.000699, 0.000801, 0.000917, 0.00105
	Hispanic	Men	56	0.000682, 0.00078, 0.000889, 0.00101, 0.00116
	Hispanic	Men	57	0.000763, 0.000867, 0.000983, 0.00112, 0.00127
	Hispanic	Men	58	0.00085, 0.00096, 0.00108, 0.00122, 0.00138
	Hispanic	Men	59	0.000939, 0.00106, 0.00119, 0.00134, 0.0015
	Hispanic	Men	60	0.00103, 0.00116, 0.0013, 0.00146, 0.00164
	Hispanic	Men	61	0.00112, 0.00126, 0.00141, 0.00159, 0.00179
	Hispanic	Men	62	0.00121, 0.00136, 0.00153, 0.00173, 0.00195
	Hispanic	Men	63	0.00129, 0.00146, 0.00165, 0.00187, 0.00211
	Hispanic	Men	64	0.00138, 0.00157, 0.00178, 0.00201, 0.00228
	Hispanic	Men	65	0.00147, 0.00168, 0.00191, 0.00217, 0.00247
	Hispanic	Men	66	0.00157, 0.00179, 0.00205, 0.00233, 0.00267
	Hispanic	Men	67	0.00167, 0.00192, 0.0022, 0.00252, 0.0029
	Hispanic	Men	68	0.00178, 0.00206, 0.00238, 0.00274, 0.00317
	Hispanic	Men	69	0.0019, 0.00222, 0.00258, 0.003, 0.00349
	Hispanic	Men	70	0.00204, 0.0024, 0.00281, 0.00329, 0.00386
	Hispanic	Men	71	0.0022, 0.0026, 0.00307, 0.00362, 0.00427
	Hispanic	Men	72	0.00239, 0.00284, 0.00336, 0.00399, 0.00473
	Hispanic	Men	73	0.0026, 0.0031, 0.00369, 0.00439, 0.00523
	Hispanic	Men	74	0.00285, 0.0034, 0.00405, 0.00482, 0.00575
	Hispanic	Men	75	0.00314, 0.00375, 0.00446, 0.0053, 0.00632
	Hispanic	Men	76	0.00348, 0.00414, 0.00492, 0.00584, 0.00694
	Hispanic	Men	77	0.00388, 0.00459, 0.00544, 0.00643, 0.00762
	Hispanic	Men	78	0.00434, 0.00512, 0.00603, 0.00709, 0.00836
	Hispanic	Men	79	0.00485, 0.0057, 0.00669, 0.00785, 0.00922
	Hispanic	Men	80	0.00538, 0.00632, 0.00742, 0.00871, 0.0102
	Hispanic	Men	81	0.00588, 0.00695, 0.00821, 0.00969, 0.0115
	Hispanic	Men	82	0.00634, 0.00757, 0.00902, 0.0108, 0.0128
	Hispanic	Men	83	0.00678, 0.00818, 0.00986, 0.0119, 0.0143
	Hispanic	Men	84	0.0072, 0.00878, 0.0107, 0.013, 0.0159
	Hispanic	Women	30	0.00000338, 0.00000462, 0.00000629, 0.00000856, 0.0000117
	Hispanic	Women	31	0.00000403, 0.00000541, 0.00000725, 0.00000971, 0.000013
	Hispanic	Women	32	0.00000481, 0.00000636, 0.00000838, 0.000011, 0.0000146
	Hispanic	Women	33	0.00000574, 0.00000746, 0.00000969, 0.0000126, 0.0000164
	Hispanic	Women	34	0.00000678, 0.00000873, 0.0000112, 0.0000144, 0.0000185
	Hispanic	Women	35	0.00000791, 0.0000101, 0.0000129, 0.0000165, 0.0000211
	Hispanic	Women	36	0.00000912, 0.0000117, 0.0000149, 0.000019, 0.0000243
	Hispanic	Women	37	0.0000104, 0.0000134, 0.0000171, 0.0000219, 0.000028
	Hispanic	Women	38	0.0000119, 0.0000153, 0.0000196, 0.0000251, 0.0000323
	Hispanic	Women	39	0.0000136, 0.0000175, 0.0000225, 0.0000289, 0.0000371
	Hispanic	Women	40	0.0000157, 0.0000202, 0.0000258, 0.000033, 0.0000424
	Hispanic	Women	41	0.0000183, 0.0000233, 0.0000296, 0.0000376, 0.0000479

Variable	Race/ethnicity	Sex	Age	Distribution
	Hispanic	Women	42	0.0000214, 0.000027, 0.0000339, 0.0000426, 0.0000536
	Hispanic	Women	43	0.0000253, 0.0000314, 0.0000389, 0.0000482, 0.0000598
	Hispanic	Women	44	0.0000301, 0.0000367, 0.0000448, 0.0000546, 0.0000666
	Hispanic	Women	45	0.0000359, 0.0000431, 0.0000516, 0.0000619, 0.0000744
	Hispanic	Women	46	0.0000429, 0.0000506, 0.0000597, 0.0000704, 0.0000831
	Hispanic	Women	47	0.0000512, 0.0000595, 0.0000691, 0.0000801, 0.0000931
	Hispanic	Women	48	0.0000607, 0.0000697, 0.0000799, 0.0000917, 0.000105
	Hispanic	Women	49	0.0000703, 0.0000807, 0.0000926, 0.000106, 0.000122
	Hispanic	Women	50	0.0000795, 0.0000924, 0.000107, 0.000124, 0.000144
	Hispanic	Women	51	0.0000888, 0.000105, 0.000124, 0.000146, 0.000173
	Hispanic	Women	52	0.0000993, 0.000119, 0.000143, 0.000172, 0.000207
	Hispanic	Women	53	0.000112, 0.000136, 0.000166, 0.000201, 0.000245
	Hispanic	Women	54	0.000126, 0.000156, 0.000191, 0.000234, 0.000288
	Hispanic	Women	55	0.000144, 0.000178, 0.00022, 0.000271, 0.000336
	Hispanic	Women	56	0.000164, 0.000204, 0.000253, 0.000313, 0.000389
	Hispanic	Women	57	0.000187, 0.000233, 0.00029, 0.000361, 0.00045
	Hispanic	Women	58	0.000211, 0.000265, 0.000332, 0.000415, 0.000521
	Hispanic	Women	59	0.000237, 0.0003, 0.000378, 0.000477, 0.000603
	Hispanic	Women	60	0.000265, 0.000338, 0.00043, 0.000547, 0.000698
	Hispanic	Women	61	0.000295, 0.00038, 0.000487, 0.000625, 0.000803
	Hispanic	Women	62	0.000328, 0.000424, 0.000548, 0.000707, 0.000916
	Hispanic	Women	63	0.000364, 0.000472, 0.000612, 0.000794, 0.00103
	Hispanic	Women	64	0.000405, 0.000525, 0.000681, 0.000882, 0.00115
	Hispanic	Women	65	0.000452, 0.000584, 0.000755, 0.000974, 0.00126
	Hispanic	Women	66	0.000505, 0.000651, 0.000836, 0.00107, 0.00138
	Hispanic	Women	67	0.000566, 0.000725, 0.000928, 0.00119, 0.00152
	Hispanic	Women	68	0.000633, 0.00081, 0.00103, 0.00132, 0.00168
	Hispanic	Women	69	0.00071, 0.000906, 0.00115, 0.00147, 0.00187
	Hispanic	Women	70	0.000796, 0.00102, 0.00129, 0.00164, 0.00209
	Hispanic	Women	71	0.000895, 0.00114, 0.00145, 0.00184, 0.00235
	Hispanic	Women	72	0.00101, 0.00128, 0.00163, 0.00207, 0.00264
	Hispanic	Women	73	0.00113, 0.00145, 0.00184, 0.00234, 0.00298
	Hispanic	Women	74	0.00128, 0.00163, 0.00208, 0.00265, 0.00338
	Hispanic	Women	75	0.00144, 0.00184, 0.00235, 0.00301, 0.00386
	Hispanic	Women	76	0.00161, 0.00208, 0.00267, 0.00343, 0.00442
	Hispanic	Women	77	0.00181, 0.00235, 0.00304, 0.00393, 0.00509
	Hispanic	Women	78	0.00204, 0.00266, 0.00346, 0.0045, 0.00586
	Hispanic	Women	79	0.0023, 0.00301, 0.00394, 0.00515, 0.00676
	Hispanic	Women	80	0.00258, 0.00341, 0.00449, 0.00591, 0.00781
	Hispanic	Women	81	0.0029, 0.00385, 0.00511, 0.00679, 0.00903
	Hispanic	Women	82	0.00322, 0.00433, 0.0058, 0.00777, 0.0104
	Hispanic	Women	83	0.00354, 0.00482, 0.00654, 0.00887, 0.0121
	Hispanic	Women	84	0.00387, 0.00532, 0.00731, 0.01, 0.0138

Variable	Race/ethnicity	Sex	Age	Distribution
	White	Men	30	0.0000219, 0.0000278, 0.0000351, 0.0000444, 0.0000563
	White	Men	31	0.0000275, 0.0000344, 0.0000429, 0.0000534, 0.0000667
	White	Men	32	0.0000342, 0.0000422, 0.000052, 0.0000641, 0.0000792
	White	Men	33	0.0000419, 0.0000513, 0.0000627, 0.0000767, 0.0000939
	White	Men	34	0.0000507, 0.0000617, 0.0000751, 0.0000913, 0.000111
	White	Men	35	0.0000603, 0.0000734, 0.0000892, 0.000108, 0.000132
	White	Men	36	0.0000712, 0.0000866, 0.000105, 0.000128, 0.000156
	White	Men	37	0.0000841, 0.000102, 0.000124, 0.00015, 0.000182
	White	Men	38	0.000101, 0.000121, 0.000145, 0.000174, 0.000209
	White	Men	39	0.000121, 0.000143, 0.00017, 0.0002, 0.000237
	White	Men	40	0.000146, 0.00017, 0.000197, 0.000229, 0.000266
	White	Men	41	0.000173, 0.000198, 0.000227, 0.000261, 0.000299
	White	Men	42	0.0002, 0.000228, 0.000261, 0.000297, 0.000339
	White	Men	43	0.000229, 0.000261, 0.000297, 0.000338, 0.000384
	White	Men	44	0.000265, 0.000299, 0.000337, 0.000381, 0.00043
	White	Men	45	0.000307, 0.000343, 0.000383, 0.000427, 0.000477
	White	Men	46	0.000356, 0.000393, 0.000434, 0.000479, 0.000529
	White	Men	47	0.00041, 0.000449, 0.000492, 0.000539, 0.000591
	White	Men	48	0.000469, 0.000512, 0.000558, 0.000608, 0.000664
	White	Men	49	0.000534, 0.000581, 0.000632, 0.000687, 0.000748
	White	Men	50	0.000609, 0.000659, 0.000713, 0.000772, 0.000836
	White	Men	51	0.000694, 0.000746, 0.000802, 0.000862, 0.000927
	White	Men	52	0.000783, 0.000838, 0.000896, 0.000959, 0.00103
	White	Men	53	0.000868, 0.00093, 0.000996, 0.00107, 0.00114
	White	Men	54	0.000947, 0.00102, 0.0011, 0.00119, 0.00128
	White	Men	55	0.00102, 0.00111, 0.00121, 0.00131, 0.00142
	White	Men	56	0.0011, 0.0012, 0.00132, 0.00144, 0.00158
	White	Men	57	0.00118, 0.0013, 0.00143, 0.00157, 0.00174
	White	Men	58	0.00126, 0.0014, 0.00155, 0.00171, 0.00189
	White	Men	59	0.00136, 0.00151, 0.00167, 0.00185, 0.00205
	White	Men	60	0.00146, 0.00162, 0.0018, 0.00199, 0.00221
	White	Men	61	0.00157, 0.00174, 0.00193, 0.00214, 0.00238
	White	Men	62	0.00168, 0.00187, 0.00208, 0.00231, 0.00256
	White	Men	63	0.00178, 0.00199, 0.00222, 0.00247, 0.00276
	White	Men	64	0.00188, 0.00211, 0.00236, 0.00265, 0.00297
	White	Men	65	0.00196, 0.00222, 0.00251, 0.00283, 0.0032
	White	Men	66	0.00205, 0.00233, 0.00266, 0.00303, 0.00346
	White	Men	67	0.00213, 0.00246, 0.00283, 0.00326, 0.00376
	White	Men	68	0.00223, 0.0026, 0.00303, 0.00352, 0.0041
	White	Men	69	0.00237, 0.00278, 0.00326, 0.00382, 0.00449
	White	Men	70	0.00255, 0.003, 0.00354, 0.00416, 0.00491
	White	Men	71	0.00277, 0.00326, 0.00385, 0.00454, 0.00535
	White	Men	72	0.00302, 0.00356, 0.0042, 0.00495, 0.00584

Variable	Race/ethnicity	Sex	Age	Distribution
	White	Men	73	0.0033, 0.00389, 0.00459, 0.0054, 0.00637
	White	Men	74	0.0036, 0.00425, 0.00501, 0.00591, 0.00697
	White	Men	75	0.00393, 0.00465, 0.00549, 0.00648, 0.00766
	White	Men	76	0.0043, 0.00509, 0.00602, 0.00713, 0.00845
	White	Men	77	0.0047, 0.00559, 0.00664, 0.00789, 0.00938
	White	Men	78	0.00518, 0.00619, 0.00737, 0.00878, 0.0105
	White	Men	79	0.00576, 0.00689, 0.00822, 0.00981, 0.0117
	White	Men	80	0.00647, 0.00773, 0.00922, 0.011, 0.0132
	White	Men	81	0.00729, 0.0087, 0.0104, 0.0124, 0.0148
	White	Men	82	0.00822, 0.0098, 0.0117, 0.0139, 0.0165
	White	Men	83	0.00923, 0.011, 0.013, 0.0154, 0.0183
	White	Men	84	0.0103, 0.0122, 0.0144, 0.017, 0.0202
	White	Women	30	0.00000547, 0.00000786, 0.0000113, 0.0000161, 0.0000232
	White	Women	31	0.00000706, 0.00000985, 0.0000137, 0.0000191, 0.0000266
	White	Women	32	0.00000918, 0.0000124, 0.0000166, 0.0000223, 0.0000301
	White	Women	33	0.0000119, 0.0000155, 0.0000201, 0.000026, 0.0000339
	White	Women	34	0.0000152, 0.0000192, 0.0000241, 0.0000304, 0.0000383
	White	Women	35	0.000019, 0.0000234, 0.0000288, 0.0000354, 0.0000436
	White	Women	36	0.0000233, 0.0000282, 0.0000342, 0.0000414, 0.0000502
	White	Women	37	0.0000277, 0.0000335, 0.0000403, 0.0000485, 0.0000585
	White	Women	38	0.0000322, 0.000039, 0.0000471, 0.000057, 0.000069
	White	Women	39	0.0000365, 0.0000447, 0.0000546, 0.0000668, 0.0000819
	White	Women	40	0.0000407, 0.0000506, 0.0000628, 0.0000779, 0.0000968
	White	Women	41	0.0000452, 0.000057, 0.0000716, 0.00009, 0.000113
	White	Women	42	0.0000503, 0.0000639, 0.0000811, 0.000103, 0.000131
	White	Women	43	0.0000561, 0.0000717, 0.0000914, 0.000117, 0.000149
	White	Women	44	0.0000628, 0.0000805, 0.000103, 0.000132, 0.000169
	White	Women	45	0.0000708, 0.0000906, 0.000116, 0.000148, 0.000189
	White	Women	46	0.0000811, 0.000103, 0.00013, 0.000164, 0.000208
	White	Women	47	0.0000948, 0.000118, 0.000146, 0.000181, 0.000224
	White	Women	48	0.000112, 0.000136, 0.000164, 0.000197, 0.000239
	White	Women	49	0.000132, 0.000156, 0.000184, 0.000216, 0.000255
	White	Women	50	0.000153, 0.000178, 0.000206, 0.000239, 0.000278
	White	Women	51	0.000174, 0.000201, 0.000232, 0.000268, 0.000309
	White	Women	52	0.000195, 0.000225, 0.00026, 0.000301, 0.000348
	White	Women	53	0.000217, 0.000251, 0.000291, 0.000337, 0.000391
	White	Women	54	0.00024, 0.000279, 0.000324, 0.000377, 0.000438
	White	Women	55	0.000266, 0.00031, 0.00036, 0.000419, 0.000488
	White	Women	56	0.000294, 0.000343, 0.0004, 0.000466, 0.000543
	White	Women	57	0.000327, 0.000381, 0.000444, 0.000517, 0.000602
	White	Women	58	0.000365, 0.000425, 0.000493, 0.000573, 0.000666
	White	Women	59	0.000409, 0.000474, 0.000549, 0.000636, 0.000737
	White	Women	60	0.000453, 0.000526, 0.00061, 0.000708, 0.000823

Variable	Race/ethnicity	Sex	Age	Distribution
	White	Women	61	0.000496, 0.00058, 0.000677, 0.000789, 0.000922
	White	Women	62	0.00054, 0.000636, 0.000746, 0.000877, 0.00103
	White	Women	63	0.000586, 0.000693, 0.000819, 0.000968, 0.00115
	White	Women	64	0.000633, 0.000753, 0.000895, 0.00106, 0.00126
	White	Women	65	0.000685, 0.000819, 0.000977, 0.00117, 0.00139
	White	Women	66	0.000745, 0.000892, 0.00107, 0.00128, 0.00153
	White	Women	67	0.000816, 0.000979, 0.00117, 0.0014, 0.00168
	White	Women	68	0.000901, 0.00108, 0.00129, 0.00154, 0.00185
	White	Women	69	0.000999, 0.0012, 0.00143, 0.00171, 0.00204
	White	Women	70	0.00111, 0.00133, 0.00159, 0.0019, 0.00227
	White	Women	71	0.00123, 0.00148, 0.00177, 0.00212, 0.00254
	White	Women	72	0.00137, 0.00164, 0.00197, 0.00237, 0.00285
	White	Women	73	0.00151, 0.00183, 0.00221, 0.00266, 0.00322
	White	Women	74	0.00168, 0.00204, 0.00247, 0.003, 0.00364
	White	Women	75	0.00186, 0.00227, 0.00277, 0.00338, 0.00412
	White	Women	76	0.00207, 0.00254, 0.00311, 0.00381, 0.00467
	White	Women	77	0.00232, 0.00285, 0.00351, 0.00431, 0.00531
	White	Women	78	0.0026, 0.00322, 0.00397, 0.0049, 0.00606
	White	Women	79	0.00294, 0.00365, 0.00452, 0.0056, 0.00695
	White	Women	80	0.00333, 0.00415, 0.00516, 0.00642, 0.008
	White	Women	81	0.00378, 0.00473, 0.00591, 0.00738, 0.00925
	White	Women	82	0.00429, 0.00539, 0.00676, 0.00849, 0.0107
	White	Women	83	0.00485, 0.00612, 0.0077, 0.0097, 0.0122
	White	Women	84	0.00543, 0.00688, 0.00869, 0.011, 0.0139
CHD mortality rates for 2019 (0.01, 0.2, 0.5, 0.8. 0.99 percentiles of the empirical distribution produced during forecasting)				
	Black	Men	30	0.0000357, 0.0000474, 0.0000626, 0.0000827, 0.00011
	Black	Men	31	0.0000449, 0.0000581, 0.0000751, 0.000097, 0.000126
	Black	Men	32	0.0000561, 0.0000709, 0.0000895, 0.000113, 0.000143
	Black	Men	33	0.0000695, 0.0000859, 0.000106, 0.000131, 0.000161
	Black	Men	34	0.0000845, 0.000103, 0.000124, 0.00015, 0.000183
	Black	Men	35	0.0001, 0.000121, 0.000144, 0.000173, 0.000208
	Black	Men	36	0.000117, 0.00014, 0.000167, 0.000199, 0.000238
	Black	Men	37	0.000134, 0.00016, 0.000191, 0.000228, 0.000273
	Black	Men	38	0.000152, 0.000182, 0.000217, 0.00026, 0.000311
	Black	Men	39	0.000172, 0.000206, 0.000246, 0.000294, 0.000352
	Black	Men	40	0.000193, 0.000232, 0.000277, 0.000332, 0.000398
	Black	Men	41	0.000216, 0.000259, 0.000311, 0.000374, 0.00045
	Black	Men	42	0.000238, 0.000289, 0.000349, 0.000422, 0.000511
	Black	Men	43	0.000262, 0.00032, 0.000391, 0.000477, 0.000583
	Black	Men	44	0.000288, 0.000355, 0.000438, 0.000541, 0.000668
	Black	Men	45	0.000317, 0.000396, 0.000493, 0.000614, 0.000766
	Black	Men	46	0.000353, 0.000444, 0.000557, 0.000698, 0.000878
	Black	Men	47	0.000395, 0.0005, 0.000631, 0.000796, 0.00101

Variable	Race/ethnicity	Sex	Age	Distribution
	Black	Men	48	0.000445, 0.000566, 0.000718, 0.00091, 0.00116
	Black	Men	49	0.000506, 0.000645, 0.000819, 0.00104, 0.00132
	Black	Men	50	0.000583, 0.00074, 0.000935, 0.00118, 0.0015
	Black	Men	51	0.00068, 0.000853, 0.00107, 0.00134, 0.00168
	Black	Men	52	0.000796, 0.000984, 0.00121, 0.0015, 0.00185
	Black	Men	53	0.000922, 0.00113, 0.00137, 0.00168, 0.00205
	Black	Men	54	0.00105, 0.00127, 0.00154, 0.00187, 0.00227
	Black	Men	55	0.00118, 0.00142, 0.00172, 0.00207, 0.00251
	Black	Men	56	0.00132, 0.00158, 0.0019, 0.00228, 0.00274
	Black	Men	57	0.00147, 0.00175, 0.00208, 0.00248, 0.00295
	Black	Men	58	0.00162, 0.00192, 0.00227, 0.00268, 0.00317
	Black	Men	59	0.00179, 0.0021, 0.00246, 0.00288, 0.00338
	Black	Men	60	0.00196, 0.00228, 0.00265, 0.00309, 0.00359
	Black	Men	61	0.00214, 0.00247, 0.00285, 0.00328, 0.00379
	Black	Men	62	0.00233, 0.00266, 0.00304, 0.00347, 0.00397
	Black	Men	63	0.00251, 0.00285, 0.00323, 0.00366, 0.00415
	Black	Men	64	0.00269, 0.00303, 0.00341, 0.00384, 0.00433
	Black	Men	65	0.00285, 0.00321, 0.0036, 0.00404, 0.00454
	Black	Men	66	0.00301, 0.00339, 0.00381, 0.00428, 0.00481
	Black	Men	67	0.00317, 0.00358, 0.00404, 0.00455, 0.00514
	Black	Men	68	0.00334, 0.00379, 0.00429, 0.00486, 0.00552
	Black	Men	69	0.00354, 0.00403, 0.00458, 0.00521, 0.00594
	Black	Men	70	0.00377, 0.0043, 0.00491, 0.00559, 0.00638
	Black	Men	71	0.00403, 0.00461, 0.00526, 0.006, 0.00686
	Black	Men	72	0.00431, 0.00493, 0.00564, 0.00644, 0.00738
	Black	Men	73	0.0046, 0.00528, 0.00605, 0.00693, 0.00795
	Black	Men	74	0.00492, 0.00566, 0.00649, 0.00745, 0.00856
	Black	Men	75	0.0053, 0.00608, 0.00698, 0.008, 0.00918
	Black	Men	76	0.00573, 0.00657, 0.00751, 0.00859, 0.00984
	Black	Men	77	0.00622, 0.00711, 0.0081, 0.00924, 0.0106
	Black	Men	78	0.00675, 0.00769, 0.00876, 0.00998, 0.0114
	Black	Men	79	0.00729, 0.00832, 0.00949, 0.0108, 0.0124
	Black	Men	80	0.00783, 0.00898, 0.0103, 0.0118, 0.0135
	Black	Men	81	0.00836, 0.00965, 0.0111, 0.0128, 0.0148
	Black	Men	82	0.00884, 0.0103, 0.012, 0.014, 0.0163
	Black	Men	83	0.00927, 0.0109, 0.0129, 0.0152, 0.018
	Black	Men	84	0.00966, 0.0115, 0.0138, 0.0165, 0.0197
	Black	Women	30	0.0000144, 0.0000195, 0.0000262, 0.0000351, 0.0000474
	Black	Women	31	0.0000181, 0.0000236, 0.0000308, 0.0000401, 0.0000524
	Black	Women	32	0.0000223, 0.0000284, 0.0000362, 0.0000461, 0.0000588
	Black	Women	33	0.0000271, 0.000034, 0.0000425, 0.0000531, 0.0000666
	Black	Women	34	0.0000323, 0.0000401, 0.0000496, 0.0000615, 0.0000763
	Black	Women	35	0.0000376, 0.0000466, 0.0000577, 0.0000714, 0.0000886

Variable	Race/ethnicity	Sex	Age	Distribution
	Black	Women	36	0.0000429, 0.0000535, 0.0000665, 0.0000827, 0.000103
	Black	Women	37	0.0000488, 0.0000611, 0.0000762, 0.0000952, 0.000119
	Black	Women	38	0.0000552, 0.0000693, 0.0000868, 0.000109, 0.000137
	Black	Women	39	0.000062, 0.0000782, 0.0000984, 0.000124, 0.000156
	Black	Women	40	0.00007, 0.0000882, 0.000111, 0.00014, 0.000176
	Black	Women	41	0.0000799, 0.0001, 0.000125, 0.000156, 0.000195
	Black	Women	42	0.0000924, 0.000114, 0.000141, 0.000173, 0.000214
	Black	Women	43	0.000107, 0.000131, 0.000158, 0.000192, 0.000234
	Black	Women	44	0.000124, 0.000149, 0.000179, 0.000215, 0.000259
	Black	Women	45	0.000143, 0.000171, 0.000204, 0.000243, 0.00029
	Black	Women	46	0.000164, 0.000195, 0.000232, 0.000276, 0.000328
	Black	Women	47	0.00019, 0.000224, 0.000265, 0.000313, 0.000371
	Black	Women	48	0.000217, 0.000257, 0.000304, 0.000359, 0.000425
	Black	Women	49	0.000243, 0.000291, 0.000347, 0.000415, 0.000497
	Black	Women	50	0.000272, 0.000329, 0.000396, 0.000478, 0.000577
	Black	Women	51	0.000313, 0.000375, 0.00045, 0.000539, 0.000647
	Black	Women	52	0.000364, 0.00043, 0.000508, 0.000599, 0.000709
	Black	Women	53	0.000413, 0.000486, 0.00057, 0.000669, 0.000787
	Black	Women	54	0.000457, 0.00054, 0.000638, 0.000752, 0.000889
	Black	Women	55	0.000508, 0.000602, 0.000711, 0.00084, 0.000995
	Black	Women	56	0.000572, 0.000673, 0.00079, 0.000929, 0.00109
	Black	Women	57	0.000642, 0.00075, 0.000875, 0.00102, 0.00119
	Black	Women	58	0.00071, 0.000828, 0.000964, 0.00112, 0.00131
	Black	Women	59	0.000773, 0.000904, 0.00106, 0.00123, 0.00144
	Black	Women	60	0.000834, 0.00098, 0.00115, 0.00135, 0.00158
	Black	Women	61	0.000892, 0.00105, 0.00124, 0.00147, 0.00174
	Black	Women	62	0.000945, 0.00113, 0.00134, 0.0016, 0.0019
	Black	Women	63	0.000992, 0.0012, 0.00144, 0.00173, 0.00208
	Black	Women	64	0.00104, 0.00126, 0.00154, 0.00187, 0.00228
	Black	Women	65	0.00108, 0.00133, 0.00164, 0.00202, 0.00249
	Black	Women	66	0.00113, 0.00141, 0.00176, 0.00219, 0.00273
	Black	Women	67	0.0012, 0.00151, 0.0019, 0.00238, 0.00298
	Black	Women	68	0.0013, 0.00164, 0.00206, 0.00259, 0.00326
	Black	Women	69	0.00142, 0.00179, 0.00225, 0.00282, 0.00355
	Black	Women	70	0.00157, 0.00197, 0.00246, 0.00308, 0.00386
	Black	Women	71	0.00174, 0.00217, 0.0027, 0.00336, 0.00419
	Black	Women	72	0.00191, 0.00238, 0.00295, 0.00366, 0.00455
	Black	Women	73	0.0021, 0.0026, 0.00322, 0.00399, 0.00495
	Black	Women	74	0.00229, 0.00284, 0.00352, 0.00435, 0.00539
	Black	Women	75	0.0025, 0.0031, 0.00384, 0.00476, 0.0059
	Black	Women	76	0.00271, 0.00338, 0.0042, 0.00522, 0.0065
	Black	Women	77	0.00293, 0.00368, 0.0046, 0.00576, 0.00723
	Black	Women	78	0.00315, 0.00399, 0.00505, 0.00639, 0.00811

Variable	Race/ethnicity	Sex	Age	Distribution
	Black	Women	79	0.00338, 0.00434, 0.00555, 0.00711, 0.00913
	Black	Women	80	0.00363, 0.00471, 0.00611, 0.00791, 0.0103
	Black	Women	81	0.00391, 0.00513, 0.00671, 0.00877, 0.0115
	Black	Women	82	0.00423, 0.00558, 0.00735, 0.00967, 0.0128
	Black	Women	83	0.00459, 0.00608, 0.00802, 0.0106, 0.014
	Black	Women	84	0.00497, 0.00659, 0.00871, 0.0115, 0.0153
	Hispanic	Men	30	0.000012, 0.0000161, 0.0000213, 0.0000283, 0.0000378
	Hispanic	Men	31	0.0000146, 0.0000192, 0.0000253, 0.0000332, 0.0000436
	Hispanic	Men	32	0.0000178, 0.0000231, 0.0000299, 0.0000387, 0.0000503
	Hispanic	Men	33	0.0000218, 0.0000277, 0.0000353, 0.0000448, 0.0000571
	Hispanic	Men	34	0.0000269, 0.0000335, 0.0000415, 0.0000515, 0.000064
	Hispanic	Men	35	0.0000332, 0.0000402, 0.0000487, 0.000059, 0.0000715
	Hispanic	Men	36	0.0000402, 0.0000479, 0.0000569, 0.0000677, 0.0000806
	Hispanic	Men	37	0.0000477, 0.0000563, 0.0000663, 0.0000781, 0.0000921
	Hispanic	Men	38	0.0000559, 0.0000657, 0.000077, 0.0000903, 0.000106
	Hispanic	Men	39	0.0000655, 0.0000765, 0.0000893, 0.000104, 0.000122
	Hispanic	Men	40	0.0000767, 0.0000891, 0.000103, 0.00012, 0.000139
	Hispanic	Men	41	0.0000894, 0.000103, 0.000119, 0.000138, 0.000159
	Hispanic	Men	42	0.000103, 0.000119, 0.000137, 0.000158, 0.000183
	Hispanic	Men	43	0.00012, 0.000138, 0.000158, 0.000182, 0.000209
	Hispanic	Men	44	0.000139, 0.000159, 0.000182, 0.000208, 0.000237
	Hispanic	Men	45	0.000161, 0.000184, 0.000209, 0.000238, 0.000271
	Hispanic	Men	46	0.000184, 0.000211, 0.000241, 0.000275, 0.000314
	Hispanic	Men	47	0.000211, 0.000242, 0.000277, 0.000317, 0.000364
	Hispanic	Men	48	0.000246, 0.00028, 0.000319, 0.000363, 0.000415
	Hispanic	Men	49	0.000287, 0.000325, 0.000367, 0.000416, 0.000471
	Hispanic	Men	50	0.00033, 0.000373, 0.000422, 0.000478, 0.000542
	Hispanic	Men	51	0.000375, 0.000426, 0.000484, 0.000551, 0.000627
	Hispanic	Men	52	0.000424, 0.000484, 0.000553, 0.000631, 0.000722
	Hispanic	Men	53	0.000476, 0.000547, 0.000628, 0.00072, 0.000828
	Hispanic	Men	54	0.000531, 0.000613, 0.000707, 0.000816, 0.000942
	Hispanic	Men	55	0.000594, 0.000686, 0.000792, 0.000913, 0.00106
	Hispanic	Men	56	0.000665, 0.000766, 0.00088, 0.00101, 0.00116
	Hispanic	Men	57	0.000745, 0.000852, 0.000973, 0.00111, 0.00127
	Hispanic	Men	58	0.000829, 0.000943, 0.00107, 0.00122, 0.00138
	Hispanic	Men	59	0.000917, 0.00104, 0.00118, 0.00133, 0.00151
	Hispanic	Men	60	0.001, 0.00114, 0.00128, 0.00145, 0.00164
	Hispanic	Men	61	0.00109, 0.00123, 0.0014, 0.00158, 0.00179
	Hispanic	Men	62	0.00117, 0.00133, 0.00151, 0.00171, 0.00194
	Hispanic	Men	63	0.00126, 0.00143, 0.00163, 0.00185, 0.00211
	Hispanic	Men	64	0.00134, 0.00153, 0.00175, 0.00199, 0.00228
	Hispanic	Men	65	0.00143, 0.00164, 0.00187, 0.00214, 0.00246
	Hispanic	Men	66	0.00152, 0.00175, 0.00201, 0.00231, 0.00265

Variable	Race/ethnicity	Sex	Age	Distribution
	Hispanic	Men	67	0.00162, 0.00187, 0.00216, 0.00249, 0.00288
	Hispanic	Men	68	0.00173, 0.00201, 0.00233, 0.00271, 0.00315
	Hispanic	Men	69	0.00184, 0.00216, 0.00253, 0.00296, 0.00347
	Hispanic	Men	70	0.00198, 0.00234, 0.00275, 0.00325, 0.00384
	Hispanic	Men	71	0.00213, 0.00253, 0.00301, 0.00357, 0.00425
	Hispanic	Men	72	0.00231, 0.00276, 0.0033, 0.00394, 0.00471
	Hispanic	Men	73	0.00251, 0.00302, 0.00362, 0.00434, 0.00521
	Hispanic	Men	74	0.00275, 0.00331, 0.00397, 0.00477, 0.00573
	Hispanic	Men	75	0.00303, 0.00364, 0.00437, 0.00524, 0.00629
	Hispanic	Men	76	0.00336, 0.00403, 0.00482, 0.00577, 0.00691
	Hispanic	Men	77	0.00374, 0.00447, 0.00533, 0.00635, 0.00759
	Hispanic	Men	78	0.00419, 0.00498, 0.00591, 0.00701, 0.00834
	Hispanic	Men	79	0.00468, 0.00555, 0.00656, 0.00777, 0.0092
	Hispanic	Men	80	0.00518, 0.00615, 0.00728, 0.00863, 0.0102
	Hispanic	Men	81	0.00565, 0.00675, 0.00805, 0.0096, 0.0115
	Hispanic	Men	82	0.00609, 0.00735, 0.00885, 0.0107, 0.0129
	Hispanic	Men	83	0.00649, 0.00793, 0.00966, 0.0118, 0.0144
	Hispanic	Men	84	0.00689, 0.0085, 0.0105, 0.0129, 0.0159
	Hispanic	Women	30	0.00000324, 0.00000447, 0.00000616, 0.00000849, 0.0000117
	Hispanic	Women	31	0.00000387, 0.00000525, 0.00000711, 0.00000963, 0.0000131
	Hispanic	Women	32	0.00000463, 0.00000618, 0.00000823, 0.0000109, 0.0000146
	Hispanic	Women	33	0.00000554, 0.00000727, 0.00000952, 0.0000125, 0.0000164
	Hispanic	Women	34	0.00000656, 0.00000851, 0.000011, 0.0000143, 0.0000185
	Hispanic	Women	35	0.00000765, 0.00000988, 0.0000127, 0.0000164, 0.0000211
	Hispanic	Women	36	0.00000882, 0.0000114, 0.0000146, 0.0000188, 0.0000243
	Hispanic	Women	37	0.0000101, 0.000013, 0.0000168, 0.0000217, 0.000028
	Hispanic	Women	38	0.0000115, 0.0000149, 0.0000193, 0.0000249, 0.0000323
	Hispanic	Women	39	0.0000131, 0.0000171, 0.0000221, 0.0000285, 0.000037
	Hispanic	Women	40	0.0000151, 0.0000196, 0.0000253, 0.0000326, 0.0000421
	Hispanic	Women	41	0.0000176, 0.0000226, 0.0000289, 0.000037, 0.0000474
	Hispanic	Women	42	0.0000207, 0.0000262, 0.0000331, 0.0000418, 0.0000529
	Hispanic	Women	43	0.0000245, 0.0000305, 0.0000379, 0.0000472, 0.0000588
	Hispanic	Women	44	0.0000291, 0.0000357, 0.0000436, 0.0000534, 0.0000654
	Hispanic	Women	45	0.0000347, 0.0000418, 0.0000503, 0.0000605, 0.0000729
	Hispanic	Women	46	0.0000415, 0.0000492, 0.0000582, 0.0000688, 0.0000814
	Hispanic	Women	47	0.0000497, 0.0000579, 0.0000674, 0.0000783, 0.0000912
	Hispanic	Women	48	0.0000589, 0.0000679, 0.0000781, 0.0000898, 0.000103
	Hispanic	Women	49	0.0000684, 0.0000788, 0.0000905, 0.000104, 0.00012
	Hispanic	Women	50	0.0000776, 0.0000903, 0.000105, 0.000122, 0.000142
	Hispanic	Women	51	0.0000869, 0.000103, 0.000122, 0.000144, 0.000171
	Hispanic	Women	52	0.0000973, 0.000117, 0.000141, 0.00017, 0.000204
	Hispanic	Women	53	0.00011, 0.000134, 0.000163, 0.000199, 0.000243
	Hispanic	Women	54	0.000124, 0.000153, 0.000189, 0.000232, 0.000286

Variable	Race/ethnicity	Sex	Age	Distribution
	Hispanic	Women	55	0.000142, 0.000176, 0.000217, 0.000269, 0.000334
	Hispanic	Women	56	0.000162, 0.000201, 0.00025, 0.000311, 0.000387
	Hispanic	Women	57	0.000184, 0.00023, 0.000287, 0.000359, 0.000449
	Hispanic	Women	58	0.000208, 0.000262, 0.000329, 0.000414, 0.000521
	Hispanic	Women	59	0.000234, 0.000297, 0.000376, 0.000476, 0.000604
	Hispanic	Women	60	0.000262, 0.000335, 0.000428, 0.000546, 0.000699
	Hispanic	Women	61	0.000291, 0.000376, 0.000484, 0.000623, 0.000804
	Hispanic	Women	62	0.000322, 0.000419, 0.000544, 0.000705, 0.000917
	Hispanic	Women	63	0.000357, 0.000466, 0.000607, 0.00079, 0.00103
	Hispanic	Women	64	0.000397, 0.000518, 0.000674, 0.000877, 0.00114
	Hispanic	Women	65	0.000443, 0.000575, 0.000746, 0.000966, 0.00126
	Hispanic	Women	66	0.000495, 0.00064, 0.000825, 0.00106, 0.00137
	Hispanic	Women	67	0.000554, 0.000713, 0.000915, 0.00117, 0.00151
	Hispanic	Women	68	0.00062, 0.000795, 0.00102, 0.0013, 0.00167
	Hispanic	Women	69	0.000694, 0.000889, 0.00114, 0.00145, 0.00186
	Hispanic	Women	70	0.000779, 0.000997, 0.00127, 0.00163, 0.00208
	Hispanic	Women	71	0.000875, 0.00112, 0.00143, 0.00182, 0.00233
	Hispanic	Women	72	0.000985, 0.00126, 0.00161, 0.00205, 0.00262
	Hispanic	Women	73	0.00111, 0.00142, 0.00181, 0.00231, 0.00296
	Hispanic	Women	74	0.00125, 0.0016, 0.00205, 0.00262, 0.00336
	Hispanic	Women	75	0.0014, 0.00181, 0.00232, 0.00298, 0.00384
	Hispanic	Women	76	0.00158, 0.00204, 0.00263, 0.0034, 0.0044
	Hispanic	Women	77	0.00177, 0.0023, 0.00299, 0.00389, 0.00506
	Hispanic	Women	78	0.00199, 0.00261, 0.00341, 0.00445, 0.00584
	Hispanic	Women	79	0.00224, 0.00296, 0.00389, 0.00511, 0.00674
	Hispanic	Women	80	0.00252, 0.00335, 0.00444, 0.00587, 0.0078
	Hispanic	Women	81	0.00283, 0.00379, 0.00506, 0.00675, 0.00903
	Hispanic	Women	82	0.00315, 0.00426, 0.00574, 0.00774, 0.0105
	Hispanic	Women	83	0.00346, 0.00474, 0.00648, 0.00884, 0.0121
	Hispanic	Women	84	0.00378, 0.00524, 0.00725, 0.01, 0.0139
	White	Men	30	0.0000213, 0.0000274, 0.000035, 0.0000449, 0.0000576
	White	Men	31	0.0000269, 0.000034, 0.0000428, 0.0000539, 0.0000682
	White	Men	32	0.0000335, 0.0000417, 0.000052, 0.0000647, 0.0000808
	White	Men	33	0.0000411, 0.0000508, 0.0000627, 0.0000774, 0.0000957
	White	Men	34	0.0000497, 0.0000612, 0.0000751, 0.0000922, 0.000113
	White	Men	35	0.0000592, 0.0000727, 0.0000891, 0.000109, 0.000134
	White	Men	36	0.0000697, 0.0000857, 0.000105, 0.000129, 0.000158
	White	Men	37	0.0000823, 0.000101, 0.000123, 0.000151, 0.000185
	White	Men	38	0.0000981, 0.000119, 0.000144, 0.000175, 0.000212
	White	Men	39	0.000118, 0.000141, 0.000168, 0.000201, 0.00024
	White	Men	40	0.000142, 0.000166, 0.000195, 0.000229, 0.000269
	White	Men	41	0.000168, 0.000194, 0.000225, 0.00026, 0.000301
	White	Men	42	0.000194, 0.000224, 0.000257, 0.000295, 0.000339

Variable	Race/ethnicity	Sex	Age	Distribution
	White	Men	43	0.000223, 0.000255, 0.000292, 0.000334, 0.000382
	White	Men	44	0.000257, 0.000292, 0.000331, 0.000375, 0.000426
	White	Men	45	0.000298, 0.000335, 0.000375, 0.00042, 0.000472
	White	Men	46	0.000346, 0.000383, 0.000425, 0.000471, 0.000523
	White	Men	47	0.000398, 0.000438, 0.000482, 0.00053, 0.000583
	White	Men	48	0.000455, 0.000499, 0.000546, 0.000598, 0.000655
	White	Men	49	0.000519, 0.000567, 0.000619, 0.000675, 0.000738
	White	Men	50	0.000592, 0.000644, 0.000699, 0.00076, 0.000826
	White	Men	51	0.000676, 0.00073, 0.000787, 0.000849, 0.000916
	White	Men	52	0.000764, 0.000821, 0.000881, 0.000945, 0.00101
	White	Men	53	0.000848, 0.000912, 0.00098, 0.00105, 0.00113
	White	Men	54	0.000925, 0.001, 0.00108, 0.00117, 0.00127
	White	Men	55	0.001, 0.00109, 0.00119, 0.0013, 0.00141
	White	Men	56	0.00107, 0.00118, 0.0013, 0.00142, 0.00157
	White	Men	57	0.00115, 0.00127, 0.00141, 0.00156, 0.00172
	White	Men	58	0.00123, 0.00137, 0.00152, 0.00169, 0.00188
	White	Men	59	0.00133, 0.00148, 0.00164, 0.00183, 0.00204
	White	Men	60	0.00143, 0.00159, 0.00177, 0.00197, 0.0022
	White	Men	61	0.00153, 0.00171, 0.0019, 0.00212, 0.00236
	White	Men	62	0.00164, 0.00183, 0.00204, 0.00228, 0.00254
	White	Men	63	0.00173, 0.00194, 0.00218, 0.00244, 0.00273
	White	Men	64	0.00182, 0.00206, 0.00231, 0.00261, 0.00294
	White	Men	65	0.00191, 0.00216, 0.00245, 0.00278, 0.00316
	White	Men	66	0.00199, 0.00227, 0.0026, 0.00297, 0.0034
	White	Men	67	0.00207, 0.00239, 0.00277, 0.00319, 0.0037
	White	Men	68	0.00217, 0.00254, 0.00296, 0.00345, 0.00404
	White	Men	69	0.0023, 0.00271, 0.00319, 0.00375, 0.00442
	White	Men	70	0.00247, 0.00292, 0.00345, 0.00408, 0.00483
	White	Men	71	0.00268, 0.00318, 0.00376, 0.00445, 0.00527
	White	Men	72	0.00293, 0.00347, 0.0041, 0.00485, 0.00575
	White	Men	73	0.0032, 0.00379, 0.00448, 0.0053, 0.00628
	White	Men	74	0.00349, 0.00414, 0.0049, 0.0058, 0.00687
	White	Men	75	0.00381, 0.00453, 0.00536, 0.00635, 0.00754
	White	Men	76	0.00416, 0.00495, 0.00589, 0.00699, 0.00832
	White	Men	77	0.00456, 0.00544, 0.00649, 0.00774, 0.00924
	White	Men	78	0.00502, 0.00602, 0.0072, 0.00861, 0.0103
	White	Men	79	0.00559, 0.00671, 0.00804, 0.00963, 0.0116
	White	Men	80	0.00626, 0.00752, 0.00901, 0.0108, 0.013
	White	Men	81	0.00706, 0.00847, 0.0101, 0.0121, 0.0146
	White	Men	82	0.00796, 0.00952, 0.0114, 0.0136, 0.0163
	White	Men	83	0.00892, 0.0107, 0.0127, 0.0151, 0.0181
	White	Men	84	0.00991, 0.0118, 0.014, 0.0167, 0.0199
	White	Women	30	0.00000535, 0.00000777, 0.0000113, 0.0000163, 0.0000237

Variable	Race/ethnicity	Sex	Age	Distribution
	White	Women	31	0.00000692, 0.00000976, 0.0000137, 0.0000193, 0.0000272
	White	Women	32	0.00000902, 0.0000123, 0.0000167, 0.0000226, 0.0000307
	White	Women	33	0.0000117, 0.0000154, 0.0000201, 0.0000263, 0.0000346
	White	Women	34	0.0000149, 0.000019, 0.0000242, 0.0000307, 0.0000391
	White	Women	35	0.0000187, 0.0000232, 0.0000288, 0.0000358, 0.0000445
	White	Women	36	0.0000228, 0.0000279, 0.0000342, 0.0000418, 0.0000512
	White	Women	37	0.0000271, 0.000033, 0.0000401, 0.0000488, 0.0000595
	White	Women	38	0.0000313, 0.0000383, 0.0000468, 0.0000571, 0.0000699
	White	Women	39	0.0000354, 0.0000438, 0.0000541, 0.0000668, 0.0000826
	White	Women	40	0.0000395, 0.0000495, 0.0000619, 0.0000774, 0.0000971
	White	Women	41	0.0000438, 0.0000556, 0.0000703, 0.000089, 0.000113
	White	Women	42	0.0000486, 0.0000622, 0.0000793, 0.000101, 0.000129
	White	Women	43	0.0000542, 0.0000696, 0.0000891, 0.000114, 0.000147
	White	Women	44	0.0000605, 0.0000778, 0.0000999, 0.000128, 0.000165
	White	Women	45	0.000068, 0.0000874, 0.000112, 0.000144, 0.000185
	White	Women	46	0.0000777, 0.0000989, 0.000126, 0.00016, 0.000203
	White	Women	47	0.0000907, 0.000113, 0.000141, 0.000175, 0.000219
	White	Women	48	0.000107, 0.000131, 0.000158, 0.000192, 0.000233
	White	Women	49	0.000127, 0.00015, 0.000178, 0.00021, 0.000249
	White	Women	50	0.000148, 0.000172, 0.0002, 0.000233, 0.000272
	White	Women	51	0.000168, 0.000195, 0.000225, 0.000261, 0.000303
	White	Women	52	0.000189, 0.000219, 0.000253, 0.000294, 0.00034
	White	Women	53	0.00021, 0.000245, 0.000284, 0.00033, 0.000383
	White	Women	54	0.000234, 0.000273, 0.000317, 0.000369, 0.00043
	White	Women	55	0.000259, 0.000303, 0.000353, 0.000412, 0.000481
	White	Women	56	0.000287, 0.000336, 0.000392, 0.000458, 0.000536
	White	Women	57	0.00032, 0.000374, 0.000436, 0.000509, 0.000595
	White	Women	58	0.000358, 0.000417, 0.000485, 0.000565, 0.000659
	White	Women	59	0.0004, 0.000465, 0.000541, 0.000628, 0.000731
	White	Women	60	0.000443, 0.000517, 0.000602, 0.000701, 0.000817
	White	Women	61	0.000486, 0.00057, 0.000667, 0.000781, 0.000916
	White	Women	62	0.000529, 0.000624, 0.000736, 0.000867, 0.00102
	White	Women	63	0.000573, 0.00068, 0.000806, 0.000956, 0.00114
	White	Women	64	0.000618, 0.000738, 0.00088, 0.00105, 0.00125
	White	Women	65	0.000668, 0.000802, 0.00096, 0.00115, 0.00138
	White	Women	66	0.000726, 0.000873, 0.00105, 0.00126, 0.00152
	White	Women	67	0.000795, 0.000957, 0.00115, 0.00138, 0.00166
	White	Women	68	0.000877, 0.00106, 0.00127, 0.00152, 0.00183
	White	Women	69	0.000972, 0.00117, 0.0014, 0.00168, 0.00203
	White	Women	70	0.00108, 0.0013, 0.00156, 0.00187, 0.00225
	White	Women	71	0.0012, 0.00144, 0.00174, 0.00209, 0.00252
	White	Women	72	0.00133, 0.00161, 0.00194, 0.00234, 0.00283
	White	Women	73	0.00147, 0.00179, 0.00217, 0.00263, 0.00319

Variable	Race/ethnicity	Sex	Age	Distribution
	White	Women	74	0.00163, 0.00199, 0.00243, 0.00296, 0.00361
	White	Women	75	0.00181, 0.00222, 0.00272, 0.00333, 0.00409
	White	Women	76	0.00202, 0.00249, 0.00306, 0.00376, 0.00464
	White	Women	77	0.00226, 0.00279, 0.00345, 0.00426, 0.00527
	White	Women	78	0.00253, 0.00315, 0.00391, 0.00485, 0.00602
	White	Women	79	0.00286, 0.00357, 0.00444, 0.00554, 0.00691
	White	Women	80	0.00324, 0.00406, 0.00508, 0.00635, 0.00797
	White	Women	81	0.00368, 0.00463, 0.00582, 0.00731, 0.0092
	White	Women	82	0.00417, 0.00527, 0.00665, 0.0084, 0.0106
	White	Women	83	0.00471, 0.00598, 0.00757, 0.00959, 0.0122
	White	Women	84	0.00528, 0.00672, 0.00854, 0.0109, 0.0138
CHD mortality rates for 2020 (0.01, 0.2, 0.5, 0.8. 0.99 percentiles of the empirical distribution produced during forecasting)				
	Black	Men	30	0.0000346, 0.0000465, 0.0000622, 0.0000833, 0.000112
	Black	Men	31	0.0000436, 0.0000572, 0.0000747, 0.0000977, 0.000128
	Black	Men	32	0.0000545, 0.0000698, 0.0000891, 0.000114, 0.000146
	Black	Men	33	0.0000676, 0.0000845, 0.000105, 0.000131, 0.000164
	Black	Men	34	0.0000823, 0.000101, 0.000124, 0.000151, 0.000186
	Black	Men	35	0.0000976, 0.000119, 0.000144, 0.000174, 0.000212
	Black	Men	36	0.000113, 0.000137, 0.000166, 0.0002, 0.000242
	Black	Men	37	0.00013, 0.000157, 0.000189, 0.000229, 0.000276
	Black	Men	38	0.000147, 0.000178, 0.000215, 0.00026, 0.000314
	Black	Men	39	0.000166, 0.000201, 0.000243, 0.000293, 0.000355
	Black	Men	40	0.000187, 0.000226, 0.000273, 0.00033, 0.0004
	Black	Men	41	0.000208, 0.000253, 0.000306, 0.000371, 0.00045
	Black	Men	42	0.000231, 0.000281, 0.000342, 0.000417, 0.000508
	Black	Men	43	0.000254, 0.000312, 0.000383, 0.00047, 0.000578
	Black	Men	44	0.000279, 0.000346, 0.000429, 0.000531, 0.000659
	Black	Men	45	0.000308, 0.000386, 0.000482, 0.000602, 0.000754
	Black	Men	46	0.000343, 0.000432, 0.000544, 0.000684, 0.000863
	Black	Men	47	0.000384, 0.000487, 0.000616, 0.00078, 0.000989
	Black	Men	48	0.000434, 0.000552, 0.000701, 0.000891, 0.00113
	Black	Men	49	0.000494, 0.00063, 0.000801, 0.00102, 0.0013
	Black	Men	50	0.000569, 0.000723, 0.000916, 0.00116, 0.00147
	Black	Men	51	0.000664, 0.000835, 0.00105, 0.00131, 0.00165
	Black	Men	52	0.000778, 0.000964, 0.00119, 0.00148, 0.00183
	Black	Men	53	0.000901, 0.00111, 0.00135, 0.00165, 0.00203
	Black	Men	54	0.00103, 0.00125, 0.00152, 0.00185, 0.00225
	Black	Men	55	0.00115, 0.0014, 0.00169, 0.00205, 0.00249
	Black	Men	56	0.00129, 0.00155, 0.00187, 0.00225, 0.00272
	Black	Men	57	0.00143, 0.00172, 0.00205, 0.00245, 0.00294
	Black	Men	58	0.00159, 0.00189, 0.00224, 0.00265, 0.00315
	Black	Men	59	0.00175, 0.00206, 0.00242, 0.00285, 0.00336
	Black	Men	60	0.00192, 0.00224, 0.00261, 0.00305, 0.00357

Variable	Race/ethnicity	Sex	Age	Distribution
	Black	Men	61	0.00209, 0.00242, 0.0028, 0.00325, 0.00376
	Black	Men	62	0.00226, 0.0026, 0.00299, 0.00343, 0.00394
	Black	Men	63	0.00244, 0.00278, 0.00317, 0.00361, 0.00411
	Black	Men	64	0.00261, 0.00296, 0.00334, 0.00378, 0.00429
	Black	Men	65	0.00277, 0.00313, 0.00353, 0.00398, 0.00449
	Black	Men	66	0.00292, 0.0033, 0.00372, 0.0042, 0.00475
	Black	Men	67	0.00307, 0.00348, 0.00395, 0.00447, 0.00507
	Black	Men	68	0.00323, 0.00369, 0.0042, 0.00478, 0.00545
	Black	Men	69	0.00343, 0.00392, 0.00448, 0.00512, 0.00586
	Black	Men	70	0.00365, 0.00419, 0.00479, 0.00549, 0.0063
	Black	Men	71	0.0039, 0.00448, 0.00514, 0.00589, 0.00676
	Black	Men	72	0.00417, 0.00479, 0.0055, 0.00632, 0.00727
	Black	Men	73	0.00445, 0.00513, 0.0059, 0.00679, 0.00783
	Black	Men	74	0.00476, 0.00549, 0.00633, 0.0073, 0.00842
	Black	Men	75	0.00512, 0.0059, 0.0068, 0.00783, 0.00903
	Black	Men	76	0.00554, 0.00637, 0.00732, 0.00841, 0.00968
	Black	Men	77	0.006, 0.00689, 0.0079, 0.00905, 0.0104
	Black	Men	78	0.00651, 0.00746, 0.00854, 0.00978, 0.0112
	Black	Men	79	0.00703, 0.00807, 0.00925, 0.0106, 0.0122
	Black	Men	80	0.00755, 0.00871, 0.01, 0.0115, 0.0133
	Black	Men	81	0.00806, 0.00936, 0.0109, 0.0126, 0.0146
	Black	Men	82	0.00853, 0.01, 0.0117, 0.0137, 0.0161
	Black	Men	83	0.00894, 0.0106, 0.0126, 0.0149, 0.0177
	Black	Men	84	0.00933, 0.0112, 0.0134, 0.0161, 0.0193
	Black	Women	30	0.0000141, 0.0000192, 0.000026, 0.0000353, 0.000048
	Black	Women	31	0.0000177, 0.0000233, 0.0000307, 0.0000403, 0.000053
	Black	Women	32	0.0000218, 0.0000281, 0.0000361, 0.0000463, 0.0000596
	Black	Women	33	0.0000265, 0.0000336, 0.0000423, 0.0000534, 0.0000675
	Black	Women	34	0.0000317, 0.0000396, 0.0000495, 0.0000618, 0.0000773
	Black	Women	35	0.0000368, 0.000046, 0.0000575, 0.0000718, 0.0000898
	Black	Women	36	0.000042, 0.0000528, 0.0000663, 0.0000832, 0.000105
	Black	Women	37	0.0000477, 0.0000603, 0.0000759, 0.0000956, 0.000121
	Black	Women	38	0.0000538, 0.0000683, 0.0000863, 0.000109, 0.000138
	Black	Women	39	0.0000604, 0.0000769, 0.0000977, 0.000124, 0.000158
	Black	Women	40	0.000068, 0.0000867, 0.00011, 0.00014, 0.000178
	Black	Women	41	0.0000776, 0.0000981, 0.000124, 0.000156, 0.000197
	Black	Women	42	0.0000895, 0.000112, 0.000139, 0.000173, 0.000216
	Black	Women	43	0.000104, 0.000128, 0.000156, 0.000192, 0.000235
	Black	Women	44	0.00012, 0.000146, 0.000177, 0.000214, 0.00026
	Black	Women	45	0.000138, 0.000166, 0.0002, 0.000242, 0.000292
	Black	Women	46	0.000158, 0.00019, 0.000228, 0.000274, 0.000329
	Black	Women	47	0.000183, 0.000219, 0.000261, 0.000311, 0.000372
	Black	Women	48	0.00021, 0.000251, 0.000299, 0.000356, 0.000426

Variable	Race/ethnicity	Sex	Age	Distribution
	Black	Women	49	0.000236, 0.000284, 0.000343, 0.000413, 0.000498
	Black	Women	50	0.000265, 0.000322, 0.000391, 0.000475, 0.000578
	Black	Women	51	0.000305, 0.000369, 0.000445, 0.000537, 0.000649
	Black	Women	52	0.000355, 0.000423, 0.000502, 0.000597, 0.000712
	Black	Women	53	0.000403, 0.000477, 0.000565, 0.000668, 0.000791
	Black	Women	54	0.000446, 0.000532, 0.000632, 0.000751, 0.000895
	Black	Women	55	0.000496, 0.000592, 0.000705, 0.000839, 0.001
	Black	Women	56	0.000559, 0.000663, 0.000784, 0.000928, 0.0011
	Black	Women	57	0.000628, 0.000739, 0.000868, 0.00102, 0.0012
	Black	Women	58	0.000694, 0.000815, 0.000956, 0.00112, 0.00132
	Black	Women	59	0.000756, 0.000891, 0.00105, 0.00123, 0.00145
	Black	Women	60	0.000815, 0.000965, 0.00114, 0.00135, 0.0016
	Black	Women	61	0.000871, 0.00104, 0.00123, 0.00147, 0.00175
	Black	Women	62	0.000921, 0.00111, 0.00133, 0.00159, 0.00192
	Black	Women	63	0.000966, 0.00117, 0.00142, 0.00172, 0.0021
	Black	Women	64	0.00101, 0.00124, 0.00152, 0.00186, 0.00229
	Black	Women	65	0.00105, 0.0013, 0.00162, 0.00201, 0.0025
	Black	Women	66	0.0011, 0.00138, 0.00173, 0.00218, 0.00274
	Black	Women	67	0.00117, 0.00148, 0.00187, 0.00236, 0.003
	Black	Women	68	0.00126, 0.0016, 0.00203, 0.00257, 0.00327
	Black	Women	69	0.00138, 0.00175, 0.00221, 0.00281, 0.00356
	Black	Women	70	0.00152, 0.00192, 0.00242, 0.00306, 0.00387
	Black	Women	71	0.00168, 0.00211, 0.00265, 0.00334, 0.0042
	Black	Women	72	0.00185, 0.00232, 0.0029, 0.00363, 0.00456
	Black	Women	73	0.00203, 0.00254, 0.00317, 0.00396, 0.00496
	Black	Women	74	0.00222, 0.00277, 0.00346, 0.00432, 0.0054
	Black	Women	75	0.00241, 0.00302, 0.00378, 0.00472, 0.00591
	Black	Women	76	0.00262, 0.00329, 0.00413, 0.00518, 0.00652
	Black	Women	77	0.00283, 0.00358, 0.00453, 0.00573, 0.00726
	Black	Women	78	0.00304, 0.00389, 0.00497, 0.00636, 0.00814
	Black	Women	79	0.00326, 0.00423, 0.00547, 0.00708, 0.00918
	Black	Women	80	0.00349, 0.00459, 0.00601, 0.00788, 0.0104
	Black	Women	81	0.00376, 0.00499, 0.00661, 0.00875, 0.0116
	Black	Women	82	0.00406, 0.00543, 0.00724, 0.00965, 0.0129
	Black	Women	83	0.00441, 0.00591, 0.00791, 0.0106, 0.0142
	Black	Women	84	0.00477, 0.00641, 0.0086, 0.0115, 0.0155
	Hispanic	Men	30	0.0000117, 0.0000158, 0.0000212, 0.0000285, 0.0000383
	Hispanic	Men	31	0.0000143, 0.0000189, 0.0000251, 0.0000333, 0.0000442
	Hispanic	Men	32	0.0000174, 0.0000227, 0.0000297, 0.0000388, 0.0000508
	Hispanic	Men	33	0.0000213, 0.0000274, 0.0000351, 0.0000449, 0.0000577
	Hispanic	Men	34	0.0000263, 0.000033, 0.0000413, 0.0000516, 0.0000647
	Hispanic	Men	35	0.0000324, 0.0000396, 0.0000484, 0.0000591, 0.0000723
	Hispanic	Men	36	0.0000392, 0.0000471, 0.0000565, 0.0000678, 0.0000815

Variable	Race/ethnicity	Sex	Age	Distribution
	Hispanic	Men	37	0.0000464, 0.0000553, 0.0000657, 0.0000782, 0.0000931
	Hispanic	Men	38	0.0000543, 0.0000644, 0.0000762, 0.0000903, 0.000107
	Hispanic	Men	39	0.0000634, 0.0000748, 0.0000882, 0.000104, 0.000123
	Hispanic	Men	40	0.0000742, 0.000087, 0.000102, 0.000119, 0.00014
	Hispanic	Men	41	0.0000865, 0.000101, 0.000117, 0.000137, 0.000159
	Hispanic	Men	42	0.0001, 0.000116, 0.000135, 0.000157, 0.000182
	Hispanic	Men	43	0.000116, 0.000134, 0.000155, 0.000179, 0.000207
	Hispanic	Men	44	0.000135, 0.000155, 0.000178, 0.000204, 0.000235
	Hispanic	Men	45	0.000156, 0.000179, 0.000205, 0.000234, 0.000268
	Hispanic	Men	46	0.000179, 0.000205, 0.000235, 0.00027, 0.00031
	Hispanic	Men	47	0.000205, 0.000236, 0.000271, 0.000311, 0.000358
	Hispanic	Men	48	0.000238, 0.000273, 0.000312, 0.000356, 0.000408
	Hispanic	Men	49	0.000279, 0.000317, 0.000359, 0.000408, 0.000463
	Hispanic	Men	50	0.000321, 0.000365, 0.000414, 0.000469, 0.000533
	Hispanic	Men	51	0.000366, 0.000417, 0.000475, 0.000541, 0.000618
	Hispanic	Men	52	0.000414, 0.000475, 0.000543, 0.000622, 0.000713
	Hispanic	Men	53	0.000465, 0.000536, 0.000618, 0.000712, 0.000821
	Hispanic	Men	54	0.000519, 0.000602, 0.000697, 0.000808, 0.000938
	Hispanic	Men	55	0.00058, 0.000673, 0.000781, 0.000906, 0.00105
	Hispanic	Men	56	0.00065, 0.000752, 0.000869, 0.001, 0.00116
	Hispanic	Men	57	0.000728, 0.000837, 0.000961, 0.0011, 0.00127
	Hispanic	Men	58	0.000811, 0.000927, 0.00106, 0.00121, 0.00138
	Hispanic	Men	59	0.000896, 0.00102, 0.00116, 0.00132, 0.0015
	Hispanic	Men	60	0.00098, 0.00112, 0.00127, 0.00144, 0.00164
	Hispanic	Men	61	0.00106, 0.00121, 0.00138, 0.00157, 0.00178
	Hispanic	Men	62	0.00114, 0.00131, 0.00149, 0.0017, 0.00194
	Hispanic	Men	63	0.00122, 0.0014, 0.0016, 0.00183, 0.0021
	Hispanic	Men	64	0.0013, 0.0015, 0.00172, 0.00197, 0.00226
	Hispanic	Men	65	0.00139, 0.0016, 0.00184, 0.00211, 0.00243
	Hispanic	Men	66	0.00148, 0.00171, 0.00197, 0.00227, 0.00262
	Hispanic	Men	67	0.00157, 0.00183, 0.00212, 0.00245, 0.00285
	Hispanic	Men	68	0.00167, 0.00196, 0.00228, 0.00267, 0.00312
	Hispanic	Men	69	0.00179, 0.00211, 0.00248, 0.00291, 0.00343
	Hispanic	Men	70	0.00192, 0.00228, 0.0027, 0.0032, 0.0038
	Hispanic	Men	71	0.00207, 0.00247, 0.00295, 0.00352, 0.00421
	Hispanic	Men	72	0.00224, 0.00269, 0.00323, 0.00388, 0.00467
	Hispanic	Men	73	0.00243, 0.00294, 0.00354, 0.00427, 0.00515
	Hispanic	Men	74	0.00267, 0.00322, 0.00389, 0.00469, 0.00567
	Hispanic	Men	75	0.00294, 0.00355, 0.00428, 0.00516, 0.00623
	Hispanic	Men	76	0.00325, 0.00392, 0.00472, 0.00567, 0.00684
	Hispanic	Men	77	0.00362, 0.00435, 0.00522, 0.00626, 0.00751
	Hispanic	Men	78	0.00405, 0.00485, 0.00579, 0.00691, 0.00826
	Hispanic	Men	79	0.00453, 0.0054, 0.00643, 0.00766, 0.00913

Variable	Race/ethnicity	Sex	Age	Distribution
	Hispanic	Men	80	0.00501, 0.00598, 0.00714, 0.00851, 0.0102
	Hispanic	Men	81	0.00546, 0.00657, 0.00789, 0.00948, 0.0114
	Hispanic	Men	82	0.00587, 0.00714, 0.00867, 0.0105, 0.0128
	Hispanic	Men	83	0.00626, 0.0077, 0.00946, 0.0116, 0.0143
	Hispanic	Men	84	0.00663, 0.00826, 0.0103, 0.0127, 0.0158
	Hispanic	Women	30	0.00000313, 0.00000437, 0.00000608, 0.00000846, 0.0000118
	Hispanic	Women	31	0.00000375, 0.00000514, 0.00000702, 0.00000959, 0.0000131
	Hispanic	Women	32	0.00000451, 0.00000606, 0.00000813, 0.0000109, 0.0000147
	Hispanic	Women	33	0.0000054, 0.00000714, 0.00000942, 0.0000124, 0.0000164
	Hispanic	Women	34	0.00000639, 0.00000836, 0.0000109, 0.0000142, 0.0000186
	Hispanic	Women	35	0.00000746, 0.0000097, 0.0000126, 0.0000163, 0.0000212
	Hispanic	Women	36	0.00000859, 0.0000112, 0.0000145, 0.0000188, 0.0000244
	Hispanic	Women	37	0.00000981, 0.0000128, 0.0000166, 0.0000216, 0.0000281
	Hispanic	Women	38	0.0000112, 0.0000146, 0.000019, 0.0000247, 0.0000323
	Hispanic	Women	39	0.0000128, 0.0000167, 0.0000217, 0.0000283, 0.0000369
	Hispanic	Women	40	0.0000147, 0.0000191, 0.0000248, 0.0000322, 0.0000419
	Hispanic	Women	41	0.0000171, 0.000022, 0.0000283, 0.0000364, 0.000047
	Hispanic	Women	42	0.0000201, 0.0000255, 0.0000324, 0.000041, 0.0000521
	Hispanic	Women	43	0.0000238, 0.0000297, 0.000037, 0.0000462, 0.0000577
	Hispanic	Women	44	0.0000282, 0.0000347, 0.0000426, 0.0000522, 0.0000642
	Hispanic	Women	45	0.0000337, 0.0000407, 0.0000491, 0.0000592, 0.0000715
	Hispanic	Women	46	0.0000403, 0.0000478, 0.0000567, 0.0000672, 0.0000798
	Hispanic	Women	47	0.0000483, 0.0000563, 0.0000657, 0.0000766, 0.0000894
	Hispanic	Women	48	0.0000573, 0.0000661, 0.0000762, 0.0000878, 0.000101
	Hispanic	Women	49	0.0000666, 0.0000768, 0.0000884, 0.000102, 0.000118
	Hispanic	Women	50	0.0000756, 0.0000882, 0.000103, 0.00012, 0.00014
	Hispanic	Women	51	0.0000849, 0.000101, 0.000119, 0.000141, 0.000168
	Hispanic	Women	52	0.0000953, 0.000115, 0.000139, 0.000167, 0.000201
	Hispanic	Women	53	0.000108, 0.000132, 0.000161, 0.000196, 0.00024
	Hispanic	Women	54	0.000122, 0.000151, 0.000186, 0.000229, 0.000283
	Hispanic	Women	55	0.000139, 0.000173, 0.000215, 0.000266, 0.000331
	Hispanic	Women	56	0.000159, 0.000198, 0.000247, 0.000308, 0.000384
	Hispanic	Women	57	0.000181, 0.000227, 0.000284, 0.000356, 0.000446
	Hispanic	Women	58	0.000205, 0.000259, 0.000326, 0.00041, 0.000518
	Hispanic	Women	59	0.00023, 0.000293, 0.000372, 0.000472, 0.000601
	Hispanic	Women	60	0.000257, 0.00033, 0.000423, 0.000542, 0.000695
	Hispanic	Women	61	0.000286, 0.000371, 0.000479, 0.000618, 0.0008
	Hispanic	Women	62	0.000317, 0.000413, 0.000537, 0.000699, 0.000911
	Hispanic	Women	63	0.000351, 0.000459, 0.000599, 0.000782, 0.00102
	Hispanic	Women	64	0.000389, 0.000509, 0.000664, 0.000867, 0.00113
	Hispanic	Women	65	0.000434, 0.000565, 0.000734, 0.000954, 0.00124
	Hispanic	Women	66	0.000484, 0.000628, 0.000811, 0.00105, 0.00136
	Hispanic	Women	67	0.000541, 0.000698, 0.000899, 0.00116, 0.00149

Variable	Race/ethnicity	Sex	Age	Distribution
	Hispanic	Women	68	0.000605, 0.000779, 0.001, 0.00128, 0.00165
	Hispanic	Women	69	0.000677, 0.000871, 0.00112, 0.00143, 0.00184
	Hispanic	Women	70	0.00076, 0.000976, 0.00125, 0.0016, 0.00206
	Hispanic	Women	71	0.000855, 0.0011, 0.0014, 0.0018, 0.0023
	Hispanic	Women	72	0.000962, 0.00123, 0.00158, 0.00202, 0.00259
	Hispanic	Women	73	0.00108, 0.00139, 0.00178, 0.00228, 0.00292
	Hispanic	Women	74	0.00122, 0.00157, 0.00201, 0.00258, 0.00332
	Hispanic	Women	75	0.00137, 0.00177, 0.00228, 0.00293, 0.00379
	Hispanic	Women	76	0.00154, 0.00199, 0.00258, 0.00334, 0.00434
	Hispanic	Women	77	0.00173, 0.00225, 0.00294, 0.00383, 0.005
	Hispanic	Women	78	0.00194, 0.00255, 0.00335, 0.00439, 0.00577
	Hispanic	Women	79	0.00219, 0.00289, 0.00382, 0.00504, 0.00666
	Hispanic	Women	80	0.00246, 0.00328, 0.00436, 0.00579, 0.00772
	Hispanic	Women	81	0.00276, 0.00371, 0.00497, 0.00666, 0.00895
	Hispanic	Women	82	0.00307, 0.00417, 0.00565, 0.00764, 0.0104
	Hispanic	Women	83	0.00338, 0.00465, 0.00638, 0.00874, 0.012
	Hispanic	Women	84	0.00369, 0.00514, 0.00714, 0.0099, 0.0138
	White	Men	30	0.0000207, 0.000027, 0.0000349, 0.0000453, 0.0000588
	White	Men	31	0.0000263, 0.0000335, 0.0000427, 0.0000544, 0.0000695
	White	Men	32	0.0000328, 0.0000413, 0.0000519, 0.0000653, 0.0000822
	White	Men	33	0.0000404, 0.0000504, 0.0000627, 0.000078, 0.0000973
	White	Men	34	0.0000488, 0.0000606, 0.000075, 0.0000929, 0.000115
	White	Men	35	0.0000581, 0.000072, 0.000089, 0.00011, 0.000136
	White	Men	36	0.0000684, 0.0000848, 0.000105, 0.00013, 0.000161
	White	Men	37	0.0000806, 0.0000996, 0.000123, 0.000151, 0.000187
	White	Men	38	0.0000958, 0.000117, 0.000143, 0.000175, 0.000215
	White	Men	39	0.000115, 0.000138, 0.000167, 0.000201, 0.000242
	White	Men	40	0.000137, 0.000163, 0.000193, 0.000228, 0.000271
	White	Men	41	0.000163, 0.00019, 0.000221, 0.000258, 0.000302
	White	Men	42	0.000189, 0.000219, 0.000253, 0.000292, 0.000338
	White	Men	43	0.000217, 0.00025, 0.000287, 0.000329, 0.000378
	White	Men	44	0.00025, 0.000285, 0.000325, 0.00037, 0.000421
	White	Men	45	0.000289, 0.000326, 0.000367, 0.000413, 0.000466
	White	Men	46	0.000335, 0.000373, 0.000416, 0.000463, 0.000516
	White	Men	47	0.000386, 0.000427, 0.000471, 0.00052, 0.000574
	White	Men	48	0.000442, 0.000486, 0.000534, 0.000587, 0.000645
	White	Men	49	0.000504, 0.000553, 0.000605, 0.000663, 0.000727
	White	Men	50	0.000576, 0.000628, 0.000685, 0.000746, 0.000814
	White	Men	51	0.000658, 0.000713, 0.000771, 0.000835, 0.000904
	White	Men	52	0.000745, 0.000803, 0.000864, 0.00093, 0.001
	White	Men	53	0.000827, 0.000892, 0.000962, 0.00104, 0.00112
	White	Men	54	0.000903, 0.000981, 0.00106, 0.00116, 0.00126
	White	Men	55	0.000976, 0.00107, 0.00117, 0.00128, 0.0014

Variable	Race/ethnicity	Sex	Age	Distribution
	White	Men	56	0.00105, 0.00116, 0.00128, 0.00141, 0.00155
	White	Men	57	0.00112, 0.00125, 0.00139, 0.00154, 0.00171
	White	Men	58	0.0012, 0.00134, 0.0015, 0.00167, 0.00186
	White	Men	59	0.00129, 0.00145, 0.00162, 0.00181, 0.00202
	White	Men	60	0.00139, 0.00156, 0.00174, 0.00195, 0.00218
	White	Men	61	0.00149, 0.00167, 0.00187, 0.00209, 0.00234
	White	Men	62	0.00159, 0.00179, 0.002, 0.00225, 0.00252
	White	Men	63	0.00169, 0.0019, 0.00214, 0.0024, 0.0027
	White	Men	64	0.00177, 0.00201, 0.00227, 0.00256, 0.0029
	White	Men	65	0.00185, 0.00211, 0.0024, 0.00273, 0.00311
	White	Men	66	0.00193, 0.00222, 0.00254, 0.00292, 0.00335
	White	Men	67	0.00201, 0.00233, 0.0027, 0.00313, 0.00363
	White	Men	68	0.00211, 0.00247, 0.00289, 0.00338, 0.00397
	White	Men	69	0.00223, 0.00264, 0.00311, 0.00367, 0.00434
	White	Men	70	0.0024, 0.00285, 0.00337, 0.004, 0.00474
	White	Men	71	0.0026, 0.00309, 0.00367, 0.00436, 0.00518
	White	Men	72	0.00284, 0.00338, 0.00401, 0.00476, 0.00565
	White	Men	73	0.0031, 0.00369, 0.00438, 0.00519, 0.00617
	White	Men	74	0.00339, 0.00403, 0.00478, 0.00568, 0.00676
	White	Men	75	0.0037, 0.0044, 0.00524, 0.00623, 0.00742
	White	Men	76	0.00403, 0.00482, 0.00575, 0.00685, 0.00818
	White	Men	77	0.00442, 0.0053, 0.00634, 0.00758, 0.00909
	White	Men	78	0.00487, 0.00586, 0.00703, 0.00844, 0.0101
	White	Men	79	0.00541, 0.00652, 0.00785, 0.00944, 0.0114
	White	Men	80	0.00607, 0.00731, 0.0088, 0.0106, 0.0128
	White	Men	81	0.00683, 0.00823, 0.00989, 0.0119, 0.0143
	White	Men	82	0.0077, 0.00925, 0.0111, 0.0133, 0.016
	White	Men	83	0.00862, 0.0103, 0.0124, 0.0148, 0.0178
	White	Men	84	0.00957, 0.0115, 0.0137, 0.0164, 0.0196
	White	Women	30	0.00000525, 0.0000077, 0.0000113, 0.0000164, 0.0000241
	White	Women	31	0.00000681, 0.00000969, 0.0000137, 0.0000194, 0.0000276
	White	Women	32	0.0000089, 0.0000122, 0.0000167, 0.0000228, 0.0000312
	White	Women	33	0.0000115, 0.0000153, 0.0000201, 0.0000266, 0.0000351
	White	Women	34	0.0000147, 0.0000189, 0.0000242, 0.000031, 0.0000397
	White	Women	35	0.0000184, 0.000023, 0.0000288, 0.0000361, 0.0000453
	White	Women	36	0.0000223, 0.0000276, 0.0000341, 0.0000421, 0.000052
	White	Women	37	0.0000265, 0.0000326, 0.00004, 0.000049, 0.0000603
	White	Women	38	0.0000306, 0.0000377, 0.0000465, 0.0000572, 0.0000706
	White	Women	39	0.0000345, 0.000043, 0.0000535, 0.0000666, 0.000083
	White	Women	40	0.0000384, 0.0000485, 0.0000611, 0.0000769, 0.0000971
	White	Women	41	0.0000426, 0.0000543, 0.0000691, 0.0000879, 0.000112
	White	Women	42	0.0000473, 0.0000607, 0.0000777, 0.0000994, 0.000128
	White	Women	43	0.0000526, 0.0000677, 0.000087, 0.000112, 0.000144

Variable	Race/ethnicity	Sex	Age	Distribution
	White	Women	44	0.0000585, 0.0000756, 0.0000973, 0.000125, 0.000162
	White	Women	45	0.0000656, 0.0000846, 0.000109, 0.00014, 0.000181
	White	Women	46	0.0000749, 0.0000957, 0.000122, 0.000155, 0.000198
	White	Women	47	0.0000874, 0.000109, 0.000137, 0.000171, 0.000214
	White	Women	48	0.000104, 0.000126, 0.000153, 0.000187, 0.000227
	White	Women	49	0.000123, 0.000146, 0.000173, 0.000205, 0.000243
	White	Women	50	0.000143, 0.000167, 0.000195, 0.000227, 0.000265
	White	Women	51	0.000163, 0.000189, 0.00022, 0.000255, 0.000296
	White	Women	52	0.000183, 0.000213, 0.000247, 0.000287, 0.000333
	White	Women	53	0.000205, 0.000239, 0.000278, 0.000323, 0.000376
	White	Women	54	0.000228, 0.000266, 0.000311, 0.000362, 0.000423
	White	Women	55	0.000253, 0.000296, 0.000346, 0.000404, 0.000473
	White	Women	56	0.000281, 0.000329, 0.000385, 0.000451, 0.000528
	White	Women	57	0.000313, 0.000366, 0.000429, 0.000502, 0.000588
	White	Women	58	0.00035, 0.000409, 0.000478, 0.000558, 0.000652
	White	Women	59	0.000391, 0.000457, 0.000532, 0.00062, 0.000724
	White	Women	60	0.000434, 0.000507, 0.000593, 0.000692, 0.000809
	White	Women	61	0.000475, 0.000559, 0.000657, 0.000772, 0.000908
	White	Women	62	0.000517, 0.000612, 0.000724, 0.000856, 0.00101
	White	Women	63	0.000559, 0.000667, 0.000793, 0.000943, 0.00112
	White	Women	64	0.000604, 0.000723, 0.000865, 0.00103, 0.00124
	White	Women	65	0.000652, 0.000784, 0.000942, 0.00113, 0.00136
	White	Women	66	0.000707, 0.000854, 0.00103, 0.00124, 0.00149
	White	Women	67	0.000774, 0.000935, 0.00113, 0.00136, 0.00164
	White	Women	68	0.000854, 0.00103, 0.00124, 0.0015, 0.00181
	White	Women	69	0.000946, 0.00114, 0.00137, 0.00166, 0.002
	White	Women	70	0.00105, 0.00127, 0.00153, 0.00184, 0.00222
	White	Women	71	0.00117, 0.00141, 0.0017, 0.00205, 0.00248
	White	Women	72	0.00129, 0.00157, 0.0019, 0.0023, 0.00279
	White	Women	73	0.00143, 0.00175, 0.00212, 0.00258, 0.00315
	White	Women	74	0.00159, 0.00195, 0.00238, 0.00291, 0.00356
	White	Women	75	0.00176, 0.00217, 0.00267, 0.00328, 0.00403
	White	Women	76	0.00197, 0.00243, 0.003, 0.0037, 0.00458
	White	Women	77	0.0022, 0.00273, 0.00338, 0.00419, 0.00521
	White	Women	78	0.00247, 0.00308, 0.00383, 0.00477, 0.00595
	White	Women	79	0.00278, 0.00349, 0.00436, 0.00545, 0.00683
	White	Women	80	0.00315, 0.00397, 0.00498, 0.00625, 0.00787
	White	Women	81	0.00358, 0.00452, 0.0057, 0.00719, 0.00909
	White	Women	82	0.00406, 0.00515, 0.00652, 0.00826, 0.0105
	White	Women	83	0.00458, 0.00584, 0.00742, 0.00944, 0.012
	White	Women	84	0.00513, 0.00656, 0.00837, 0.0107, 0.0137
CHD mortality rates for 2021 (0.01, 0.2, 0.5, 0.8. 0.99 percentiles of the empirical distribution produced during forecasting)				
	Black	Men	30	0.0000336, 0.0000456, 0.0000618, 0.0000837, 0.000114

Variable	Race/ethnicity	Sex	Age	Distribution
	Black	Men	31	0.0000424, 0.0000562, 0.0000743, 0.0000982, 0.00013
	Black	Men	32	0.0000531, 0.0000686, 0.0000886, 0.000114, 0.000148
	Black	Men	33	0.0000659, 0.0000832, 0.000105, 0.000132, 0.000167
	Black	Men	34	0.0000802, 0.0000994, 0.000123, 0.000152, 0.000188
	Black	Men	35	0.0000951, 0.000117, 0.000143, 0.000175, 0.000214
	Black	Men	36	0.00011, 0.000135, 0.000164, 0.0002, 0.000245
	Black	Men	37	0.000126, 0.000154, 0.000188, 0.000229, 0.000279
	Black	Men	38	0.000143, 0.000175, 0.000213, 0.000259, 0.000317
	Black	Men	39	0.000161, 0.000197, 0.00024, 0.000292, 0.000357
	Black	Men	40	0.000181, 0.000221, 0.000269, 0.000328, 0.000401
	Black	Men	41	0.000202, 0.000247, 0.000301, 0.000367, 0.000449
	Black	Men	42	0.000224, 0.000274, 0.000336, 0.000411, 0.000504
	Black	Men	43	0.000246, 0.000304, 0.000375, 0.000462, 0.000571
	Black	Men	44	0.00027, 0.000337, 0.000419, 0.000521, 0.00065
	Black	Men	45	0.000299, 0.000375, 0.000471, 0.00059, 0.000742
	Black	Men	46	0.000333, 0.000421, 0.000531, 0.00067, 0.000847
	Black	Men	47	0.000374, 0.000475, 0.000602, 0.000763, 0.000969
	Black	Men	48	0.000422, 0.000539, 0.000685, 0.000872, 0.00111
	Black	Men	49	0.000482, 0.000615, 0.000784, 0.000998, 0.00127
	Black	Men	50	0.000556, 0.000708, 0.000898, 0.00114, 0.00145
	Black	Men	51	0.00065, 0.000818, 0.00103, 0.00129, 0.00163
	Black	Men	52	0.000761, 0.000946, 0.00117, 0.00146, 0.00181
	Black	Men	53	0.000883, 0.00109, 0.00133, 0.00163, 0.00201
	Black	Men	54	0.00101, 0.00123, 0.0015, 0.00183, 0.00223
	Black	Men	55	0.00113, 0.00138, 0.00167, 0.00203, 0.00247
	Black	Men	56	0.00126, 0.00153, 0.00185, 0.00223, 0.0027
	Black	Men	57	0.00141, 0.00169, 0.00202, 0.00243, 0.00292
	Black	Men	58	0.00156, 0.00186, 0.00221, 0.00262, 0.00313
	Black	Men	59	0.00171, 0.00203, 0.00239, 0.00282, 0.00334
	Black	Men	60	0.00187, 0.0022, 0.00258, 0.00302, 0.00355
	Black	Men	61	0.00204, 0.00237, 0.00276, 0.00321, 0.00374
	Black	Men	62	0.00221, 0.00255, 0.00294, 0.00339, 0.00391
	Black	Men	63	0.00238, 0.00272, 0.00311, 0.00356, 0.00407
	Black	Men	64	0.00254, 0.00289, 0.00328, 0.00373, 0.00424
	Black	Men	65	0.00269, 0.00305, 0.00346, 0.00391, 0.00444
	Black	Men	66	0.00284, 0.00322, 0.00365, 0.00413, 0.00469
	Black	Men	67	0.00298, 0.00339, 0.00386, 0.00439, 0.005
	Black	Men	68	0.00314, 0.00359, 0.00411, 0.00469, 0.00537
	Black	Men	69	0.00332, 0.00382, 0.00438, 0.00503, 0.00578
	Black	Men	70	0.00354, 0.00408, 0.00469, 0.00539, 0.00622
	Black	Men	71	0.00378, 0.00436, 0.00502, 0.00578, 0.00667
	Black	Men	72	0.00404, 0.00466, 0.00538, 0.0062, 0.00717
	Black	Men	73	0.00431, 0.00499, 0.00576, 0.00666, 0.00771

Variable	Race/ethnicity	Sex	Age	Distribution
	Black	Men	74	0.00461, 0.00534, 0.00618, 0.00715, 0.00829
	Black	Men	75	0.00495, 0.00574, 0.00663, 0.00767, 0.00888
	Black	Men	76	0.00536, 0.00619, 0.00714, 0.00824, 0.00952
	Black	Men	77	0.00581, 0.00669, 0.0077, 0.00887, 0.0102
	Black	Men	78	0.00629, 0.00724, 0.00833, 0.00959, 0.011
	Black	Men	79	0.00679, 0.00784, 0.00903, 0.0104, 0.012
	Black	Men	80	0.0073, 0.00846, 0.00979, 0.0113, 0.0131
	Black	Men	81	0.00779, 0.00909, 0.0106, 0.0123, 0.0144
	Black	Men	82	0.00825, 0.00972, 0.0114, 0.0134, 0.0158
	Black	Men	83	0.00865, 0.0103, 0.0123, 0.0146, 0.0174
	Black	Men	84	0.00902, 0.0109, 0.0131, 0.0158, 0.019
	Black	Women	30	0.0000138, 0.0000189, 0.0000259, 0.0000355, 0.0000487
	Black	Women	31	0.0000173, 0.000023, 0.0000305, 0.0000404, 0.0000537
	Black	Women	32	0.0000214, 0.0000278, 0.0000359, 0.0000465, 0.0000603
	Black	Women	33	0.000026, 0.0000332, 0.0000422, 0.0000536, 0.0000683
	Black	Women	34	0.0000311, 0.0000392, 0.0000493, 0.0000621, 0.0000783
	Black	Women	35	0.0000361, 0.0000455, 0.0000573, 0.0000721, 0.000091
	Black	Women	36	0.0000412, 0.0000522, 0.000066, 0.0000835, 0.000106
	Black	Women	37	0.0000467, 0.0000594, 0.0000755, 0.0000958, 0.000122
	Black	Women	38	0.0000526, 0.0000673, 0.0000858, 0.000109, 0.00014
	Black	Women	39	0.0000589, 0.0000757, 0.000097, 0.000124, 0.00016
	Black	Women	40	0.0000662, 0.0000851, 0.000109, 0.00014, 0.00018
	Black	Women	41	0.0000754, 0.0000962, 0.000122, 0.000156, 0.000199
	Black	Women	42	0.000087, 0.000109, 0.000137, 0.000172, 0.000217
	Black	Women	43	0.000101, 0.000125, 0.000154, 0.00019, 0.000236
	Black	Women	44	0.000116, 0.000142, 0.000174, 0.000212, 0.00026
	Black	Women	45	0.000133, 0.000162, 0.000197, 0.000239, 0.000292
	Black	Women	46	0.000153, 0.000185, 0.000224, 0.000271, 0.000329
	Black	Women	47	0.000177, 0.000213, 0.000256, 0.000308, 0.000371
	Black	Women	48	0.000204, 0.000245, 0.000294, 0.000353, 0.000424
	Black	Women	49	0.000229, 0.000278, 0.000337, 0.000409, 0.000496
	Black	Women	50	0.000258, 0.000316, 0.000386, 0.000472, 0.000577
	Black	Women	51	0.000298, 0.000362, 0.000439, 0.000533, 0.000648
	Black	Women	52	0.000347, 0.000415, 0.000497, 0.000594, 0.000711
	Black	Women	53	0.000394, 0.00047, 0.000559, 0.000664, 0.000791
	Black	Women	54	0.000437, 0.000523, 0.000626, 0.000748, 0.000896
	Black	Women	55	0.000486, 0.000583, 0.000698, 0.000836, 0.001
	Black	Women	56	0.000548, 0.000653, 0.000777, 0.000925, 0.0011
	Black	Women	57	0.000615, 0.000728, 0.000861, 0.00102, 0.0012
	Black	Women	58	0.000681, 0.000804, 0.000948, 0.00112, 0.00132
	Black	Women	59	0.000741, 0.000878, 0.00104, 0.00123, 0.00146
	Black	Women	60	0.000798, 0.000951, 0.00113, 0.00134, 0.0016
	Black	Women	61	0.000852, 0.00102, 0.00122, 0.00146, 0.00176

Variable	Race/ethnicity	Sex	Age	Distribution
	Black	Women	62	0.0009, 0.00109, 0.00131, 0.00159, 0.00192
	Black	Women	63	0.000942, 0.00115, 0.00141, 0.00172, 0.0021
	Black	Women	64	0.00098, 0.00121, 0.0015, 0.00185, 0.00229
	Black	Women	65	0.00102, 0.00128, 0.0016, 0.002, 0.0025
	Black	Women	66	0.00107, 0.00135, 0.00171, 0.00216, 0.00273
	Black	Women	67	0.00113, 0.00144, 0.00184, 0.00234, 0.00299
	Black	Women	68	0.00122, 0.00156, 0.00199, 0.00255, 0.00326
	Black	Women	69	0.00133, 0.00171, 0.00218, 0.00278, 0.00355
	Black	Women	70	0.00147, 0.00187, 0.00238, 0.00303, 0.00385
	Black	Women	71	0.00163, 0.00206, 0.00261, 0.0033, 0.00418
	Black	Women	72	0.00179, 0.00226, 0.00285, 0.00359, 0.00454
	Black	Women	73	0.00196, 0.00247, 0.00311, 0.00391, 0.00493
	Black	Women	74	0.00215, 0.0027, 0.00339, 0.00427, 0.00537
	Black	Women	75	0.00234, 0.00295, 0.00371, 0.00466, 0.00588
	Black	Women	76	0.00253, 0.00321, 0.00405, 0.00512, 0.00649
	Black	Women	77	0.00273, 0.00349, 0.00445, 0.00566, 0.00723
	Black	Women	78	0.00294, 0.00379, 0.00488, 0.00629, 0.00811
	Black	Women	79	0.00315, 0.00412, 0.00537, 0.007, 0.00916
	Black	Women	80	0.00338, 0.00447, 0.00591, 0.00781, 0.0103
	Black	Women	81	0.00363, 0.00486, 0.00649, 0.00868, 0.0116
	Black	Women	82	0.00392, 0.00529, 0.00712, 0.00958, 0.0129
	Black	Women	83	0.00425, 0.00576, 0.00778, 0.0105, 0.0142
	Black	Women	84	0.0046, 0.00624, 0.00846, 0.0115, 0.0156
	Hispanic	Men	30	0.0000114, 0.0000155, 0.000021, 0.0000285, 0.0000389
	Hispanic	Men	31	0.0000139, 0.0000187, 0.0000249, 0.0000333, 0.0000447
	Hispanic	Men	32	0.000017, 0.0000224, 0.0000295, 0.0000388, 0.0000513
	Hispanic	Men	33	0.0000209, 0.000027, 0.0000348, 0.000045, 0.0000582
	Hispanic	Men	34	0.0000258, 0.0000326, 0.000041, 0.0000517, 0.0000652
	Hispanic	Men	35	0.0000317, 0.0000391, 0.0000481, 0.0000592, 0.000073
	Hispanic	Men	36	0.0000382, 0.0000463, 0.0000561, 0.0000679, 0.0000824
	Hispanic	Men	37	0.0000451, 0.0000543, 0.0000652, 0.0000782, 0.0000941
	Hispanic	Men	38	0.0000527, 0.0000631, 0.0000755, 0.0000902, 0.000108
	Hispanic	Men	39	0.0000615, 0.0000733, 0.0000872, 0.000104, 0.000124
	Hispanic	Men	40	0.0000719, 0.0000851, 0.0001, 0.000119, 0.00014
	Hispanic	Men	41	0.0000839, 0.0000985, 0.000116, 0.000136, 0.000159
	Hispanic	Men	42	0.0000974, 0.000114, 0.000133, 0.000155, 0.000181
	Hispanic	Men	43	0.000113, 0.000131, 0.000152, 0.000176, 0.000205
	Hispanic	Men	44	0.000131, 0.000152, 0.000175, 0.000201, 0.000232
	Hispanic	Men	45	0.000152, 0.000175, 0.0002, 0.00023, 0.000264
	Hispanic	Men	46	0.000174, 0.0002, 0.00023, 0.000265, 0.000305
	Hispanic	Men	47	0.000199, 0.00023, 0.000265, 0.000305, 0.000352
	Hispanic	Men	48	0.000232, 0.000266, 0.000305, 0.000349, 0.000401
	Hispanic	Men	49	0.000271, 0.000309, 0.000352, 0.0004, 0.000456

Variable	Race/ethnicity	Sex	Age	Distribution
	Hispanic	Men	50	0.000313, 0.000356, 0.000405, 0.000461, 0.000525
	Hispanic	Men	51	0.000357, 0.000408, 0.000466, 0.000532, 0.000609
	Hispanic	Men	52	0.000405, 0.000465, 0.000534, 0.000613, 0.000704
	Hispanic	Men	53	0.000455, 0.000526, 0.000608, 0.000703, 0.000813
	Hispanic	Men	54	0.000507, 0.000591, 0.000687, 0.0008, 0.000931
	Hispanic	Men	55	0.000567, 0.000662, 0.000771, 0.000898, 0.00105
	Hispanic	Men	56	0.000637, 0.00074, 0.000858, 0.000996, 0.00116
	Hispanic	Men	57	0.000713, 0.000824, 0.00095, 0.0011, 0.00126
	Hispanic	Men	58	0.000795, 0.000912, 0.00105, 0.0012, 0.00138
	Hispanic	Men	59	0.000877, 0.001, 0.00115, 0.00131, 0.0015
	Hispanic	Men	60	0.000959, 0.0011, 0.00125, 0.00143, 0.00163
	Hispanic	Men	61	0.00104, 0.00119, 0.00136, 0.00155, 0.00177
	Hispanic	Men	62	0.00112, 0.00128, 0.00147, 0.00168, 0.00192
	Hispanic	Men	63	0.00119, 0.00137, 0.00157, 0.00181, 0.00208
	Hispanic	Men	64	0.00127, 0.00146, 0.00169, 0.00194, 0.00224
	Hispanic	Men	65	0.00135, 0.00156, 0.0018, 0.00208, 0.00241
	Hispanic	Men	66	0.00144, 0.00167, 0.00193, 0.00224, 0.00259
	Hispanic	Men	67	0.00153, 0.00178, 0.00207, 0.00241, 0.00281
	Hispanic	Men	68	0.00163, 0.00191, 0.00224, 0.00262, 0.00308
	Hispanic	Men	69	0.00174, 0.00205, 0.00243, 0.00286, 0.00339
	Hispanic	Men	70	0.00186, 0.00222, 0.00264, 0.00314, 0.00375
	Hispanic	Men	71	0.00201, 0.00241, 0.00289, 0.00346, 0.00416
	Hispanic	Men	72	0.00217, 0.00262, 0.00316, 0.00381, 0.00461
	Hispanic	Men	73	0.00236, 0.00287, 0.00347, 0.0042, 0.00509
	Hispanic	Men	74	0.00259, 0.00314, 0.00381, 0.00461, 0.0056
	Hispanic	Men	75	0.00285, 0.00346, 0.00419, 0.00507, 0.00615
	Hispanic	Men	76	0.00316, 0.00382, 0.00461, 0.00558, 0.00675
	Hispanic	Men	77	0.00351, 0.00424, 0.00511, 0.00615, 0.00742
	Hispanic	Men	78	0.00393, 0.00472, 0.00567, 0.00679, 0.00816
	Hispanic	Men	79	0.00439, 0.00526, 0.0063, 0.00753, 0.00903
	Hispanic	Men	80	0.00485, 0.00583, 0.00699, 0.00838, 0.0101
	Hispanic	Men	81	0.00529, 0.0064, 0.00773, 0.00933, 0.0113
	Hispanic	Men	82	0.00568, 0.00695, 0.00849, 0.0104, 0.0127
	Hispanic	Men	83	0.00605, 0.00749, 0.00926, 0.0115, 0.0142
	Hispanic	Men	84	0.00641, 0.00803, 0.01, 0.0125, 0.0157
	Hispanic	Women	30	0.00000304, 0.00000428, 0.00000602, 0.00000845, 0.0000119
	Hispanic	Women	31	0.00000366, 0.00000505, 0.00000695, 0.00000958, 0.0000132
	Hispanic	Women	32	0.0000044, 0.00000597, 0.00000806, 0.0000109, 0.0000148
	Hispanic	Women	33	0.00000528, 0.00000703, 0.00000934, 0.0000124, 0.0000165
	Hispanic	Women	34	0.00000626, 0.00000824, 0.0000108, 0.0000142, 0.0000187
	Hispanic	Women	35	0.0000073, 0.00000955, 0.0000125, 0.0000163, 0.0000213
	Hispanic	Women	36	0.00000839, 0.000011, 0.0000143, 0.0000187, 0.0000245
	Hispanic	Women	37	0.00000957, 0.0000126, 0.0000164, 0.0000215, 0.0000281

Variable	Race/ethnicity	Sex	Age	Distribution
	Hispanic	Women	38	0.0000109, 0.0000143, 0.0000188, 0.0000246, 0.0000323
	Hispanic	Women	39	0.0000124, 0.0000163, 0.0000214, 0.000028, 0.0000369
	Hispanic	Women	40	0.0000143, 0.0000187, 0.0000244, 0.0000318, 0.0000417
	Hispanic	Women	41	0.0000166, 0.0000215, 0.0000278, 0.0000359, 0.0000465
	Hispanic	Women	42	0.0000195, 0.0000249, 0.0000317, 0.0000403, 0.0000515
	Hispanic	Women	43	0.0000231, 0.000029, 0.0000362, 0.0000453, 0.0000568
	Hispanic	Women	44	0.0000274, 0.0000338, 0.0000416, 0.0000511, 0.000063
	Hispanic	Women	45	0.0000327, 0.0000396, 0.0000479, 0.0000579, 0.0000702
	Hispanic	Women	46	0.0000391, 0.0000466, 0.0000554, 0.0000658, 0.0000783
	Hispanic	Women	47	0.0000469, 0.0000549, 0.0000641, 0.0000749, 0.0000877
	Hispanic	Women	48	0.0000557, 0.0000644, 0.0000744, 0.000086, 0.0000994
	Hispanic	Women	49	0.0000648, 0.0000749, 0.0000865, 0.0000998, 0.000115
	Hispanic	Women	50	0.0000738, 0.0000862, 0.000101, 0.000117, 0.000137
	Hispanic	Women	51	0.000083, 0.0000987, 0.000117, 0.000139, 0.000165
	Hispanic	Women	52	0.0000934, 0.000113, 0.000136, 0.000164, 0.000198
	Hispanic	Women	53	0.000105, 0.000129, 0.000158, 0.000193, 0.000237
	Hispanic	Women	54	0.00012, 0.000148, 0.000183, 0.000226, 0.00028
	Hispanic	Women	55	0.000137, 0.00017, 0.000212, 0.000263, 0.000327
	Hispanic	Women	56	0.000156, 0.000196, 0.000244, 0.000305, 0.000381
	Hispanic	Women	57	0.000178, 0.000224, 0.000281, 0.000352, 0.000443
	Hispanic	Women	58	0.000202, 0.000255, 0.000322, 0.000406, 0.000514
	Hispanic	Women	59	0.000227, 0.000289, 0.000368, 0.000468, 0.000597
	Hispanic	Women	60	0.000253, 0.000326, 0.000418, 0.000537, 0.000691
	Hispanic	Women	61	0.000281, 0.000365, 0.000473, 0.000612, 0.000794
	Hispanic	Women	62	0.000311, 0.000407, 0.00053, 0.000691, 0.000904
	Hispanic	Women	63	0.000344, 0.000452, 0.000591, 0.000773, 0.00101
	Hispanic	Women	64	0.000382, 0.0005, 0.000654, 0.000855, 0.00112
	Hispanic	Women	65	0.000424, 0.000554, 0.000722, 0.00094, 0.00123
	Hispanic	Women	66	0.000473, 0.000615, 0.000797, 0.00103, 0.00134
	Hispanic	Women	67	0.000529, 0.000684, 0.000882, 0.00114, 0.00147
	Hispanic	Women	68	0.000591, 0.000762, 0.000981, 0.00126, 0.00163
	Hispanic	Women	69	0.000661, 0.000852, 0.0011, 0.00141, 0.00181
	Hispanic	Women	70	0.000742, 0.000955, 0.00123, 0.00157, 0.00203
	Hispanic	Women	71	0.000834, 0.00107, 0.00138, 0.00177, 0.00227
	Hispanic	Women	72	0.000938, 0.00121, 0.00155, 0.00198, 0.00255
	Hispanic	Women	73	0.00106, 0.00136, 0.00174, 0.00224, 0.00288
	Hispanic	Women	74	0.00119, 0.00153, 0.00197, 0.00253, 0.00327
	Hispanic	Women	75	0.00133, 0.00173, 0.00223, 0.00288, 0.00373
	Hispanic	Women	76	0.0015, 0.00195, 0.00253, 0.00328, 0.00427
	Hispanic	Women	77	0.00168, 0.0022, 0.00288, 0.00376, 0.00492
	Hispanic	Women	78	0.00189, 0.00249, 0.00328, 0.00431, 0.00567
	Hispanic	Women	79	0.00213, 0.00283, 0.00374, 0.00495, 0.00656
	Hispanic	Women	80	0.0024, 0.00321, 0.00427, 0.00569, 0.0076

Variable	Race/ethnicity	Sex	Age	Distribution
	Hispanic	Women	81	0.00269, 0.00363, 0.00487, 0.00655, 0.00882
	Hispanic	Women	82	0.003, 0.00408, 0.00554, 0.00752, 0.0102
	Hispanic	Women	83	0.0033, 0.00455, 0.00625, 0.0086, 0.0119
	Hispanic	Women	84	0.0036, 0.00503, 0.007, 0.00975, 0.0136
	White	Men	30	0.0000202, 0.0000266, 0.0000348, 0.0000456, 0.0000599
	White	Men	31	0.0000257, 0.0000331, 0.0000426, 0.0000548, 0.0000707
	White	Men	32	0.0000321, 0.0000409, 0.0000518, 0.0000657, 0.0000835
	White	Men	33	0.0000396, 0.0000499, 0.0000626, 0.0000785, 0.0000987
	White	Men	34	0.000048, 0.00006, 0.0000749, 0.0000934, 0.000117
	White	Men	35	0.000057, 0.0000712, 0.0000888, 0.000111, 0.000138
	White	Men	36	0.0000671, 0.0000838, 0.000104, 0.00013, 0.000163
	White	Men	37	0.0000789, 0.0000983, 0.000122, 0.000152, 0.000189
	White	Men	38	0.0000935, 0.000116, 0.000142, 0.000175, 0.000217
	White	Men	39	0.000112, 0.000136, 0.000165, 0.000201, 0.000244
	White	Men	40	0.000133, 0.00016, 0.000191, 0.000228, 0.000272
	White	Men	41	0.000158, 0.000186, 0.000218, 0.000257, 0.000302
	White	Men	42	0.000184, 0.000214, 0.000248, 0.000289, 0.000336
	White	Men	43	0.000211, 0.000244, 0.000281, 0.000325, 0.000375
	White	Men	44	0.000243, 0.000278, 0.000318, 0.000364, 0.000416
	White	Men	45	0.000281, 0.000318, 0.000359, 0.000406, 0.00046
	White	Men	46	0.000325, 0.000364, 0.000407, 0.000454, 0.000508
	White	Men	47	0.000375, 0.000416, 0.000461, 0.00051, 0.000566
	White	Men	48	0.000429, 0.000474, 0.000522, 0.000576, 0.000635
	White	Men	49	0.00049, 0.000539, 0.000592, 0.000651, 0.000716
	White	Men	50	0.00056, 0.000613, 0.000671, 0.000733, 0.000803
	White	Men	51	0.000641, 0.000697, 0.000756, 0.000821, 0.000892
	White	Men	52	0.000727, 0.000786, 0.000848, 0.000916, 0.00099
	White	Men	53	0.000808, 0.000874, 0.000946, 0.00102, 0.00111
	White	Men	54	0.000882, 0.000961, 0.00105, 0.00114, 0.00124
	White	Men	55	0.000954, 0.00105, 0.00115, 0.00126, 0.00139
	White	Men	56	0.00103, 0.00114, 0.00126, 0.00139, 0.00154
	White	Men	57	0.0011, 0.00123, 0.00137, 0.00152, 0.00169
	White	Men	58	0.00118, 0.00132, 0.00148, 0.00165, 0.00185
	White	Men	59	0.00126, 0.00142, 0.00159, 0.00179, 0.00201
	White	Men	60	0.00136, 0.00153, 0.00171, 0.00192, 0.00216
	White	Men	61	0.00146, 0.00164, 0.00184, 0.00207, 0.00233
	White	Men	62	0.00155, 0.00175, 0.00197, 0.00222, 0.0025
	White	Men	63	0.00164, 0.00186, 0.0021, 0.00237, 0.00268
	White	Men	64	0.00173, 0.00196, 0.00222, 0.00252, 0.00286
	White	Men	65	0.0018, 0.00206, 0.00235, 0.00268, 0.00306
	White	Men	66	0.00188, 0.00216, 0.00249, 0.00286, 0.00329
	White	Men	67	0.00195, 0.00227, 0.00264, 0.00307, 0.00357
	White	Men	68	0.00205, 0.00241, 0.00282, 0.00332, 0.0039

Variable	Race/ethnicity	Sex	Age	Distribution
	White	Men	69	0.00217, 0.00257, 0.00304, 0.0036, 0.00426
	White	Men	70	0.00233, 0.00277, 0.0033, 0.00392, 0.00466
	White	Men	71	0.00253, 0.00301, 0.00359, 0.00427, 0.00509
	White	Men	72	0.00276, 0.00329, 0.00391, 0.00466, 0.00556
	White	Men	73	0.00301, 0.00359, 0.00427, 0.00509, 0.00607
	White	Men	74	0.00328, 0.00392, 0.00467, 0.00556, 0.00664
	White	Men	75	0.00358, 0.00428, 0.00511, 0.0061, 0.00729
	White	Men	76	0.00391, 0.00469, 0.00561, 0.00671, 0.00804
	White	Men	77	0.00429, 0.00515, 0.00619, 0.00743, 0.00893
	White	Men	78	0.00472, 0.0057, 0.00686, 0.00826, 0.00997
	White	Men	79	0.00525, 0.00635, 0.00766, 0.00925, 0.0112
	White	Men	80	0.00588, 0.00711, 0.00859, 0.0104, 0.0126
	White	Men	81	0.00662, 0.008, 0.00966, 0.0117, 0.0141
	White	Men	82	0.00745, 0.009, 0.0108, 0.0131, 0.0158
	White	Men	83	0.00835, 0.0101, 0.0121, 0.0145, 0.0175
	White	Men	84	0.00926, 0.0111, 0.0134, 0.016, 0.0193
	White	Women	30	0.00000516, 0.00000763, 0.0000112, 0.0000165, 0.0000245
	White	Women	31	0.00000671, 0.00000961, 0.0000137, 0.0000196, 0.000028
	White	Women	32	0.00000877, 0.0000121, 0.0000167, 0.0000229, 0.0000316
	White	Women	33	0.0000114, 0.0000152, 0.0000201, 0.0000267, 0.0000356
	White	Women	34	0.0000145, 0.0000188, 0.0000242, 0.0000311, 0.0000402
	White	Women	35	0.0000181, 0.0000228, 0.0000288, 0.0000363, 0.0000459
	White	Women	36	0.0000219, 0.0000273, 0.000034, 0.0000423, 0.0000527
	White	Women	37	0.0000259, 0.0000321, 0.0000398, 0.0000492, 0.000061
	White	Women	38	0.0000299, 0.0000371, 0.0000461, 0.0000572, 0.0000712
	White	Women	39	0.0000336, 0.0000423, 0.000053, 0.0000664, 0.0000834
	White	Women	40	0.0000374, 0.0000475, 0.0000602, 0.0000764, 0.000097
	White	Women	41	0.0000414, 0.0000531, 0.0000679, 0.0000869, 0.000111
	White	Women	42	0.000046, 0.0000592, 0.0000761, 0.0000978, 0.000126
	White	Women	43	0.0000511, 0.000066, 0.000085, 0.00011, 0.000141
	White	Women	44	0.0000568, 0.0000735, 0.0000949, 0.000123, 0.000159
	White	Women	45	0.0000636, 0.0000822, 0.000106, 0.000137, 0.000177
	White	Women	46	0.0000725, 0.0000928, 0.000119, 0.000151, 0.000194
	White	Women	47	0.0000846, 0.000106, 0.000133, 0.000166, 0.000209
	White	Women	48	0.0001, 0.000123, 0.000149, 0.000182, 0.000222
	White	Women	49	0.000119, 0.000142, 0.000168, 0.0002, 0.000238
	White	Women	50	0.000139, 0.000162, 0.00019, 0.000222, 0.00026
	White	Women	51	0.000158, 0.000184, 0.000214, 0.000249, 0.00029
	White	Women	52	0.000179, 0.000208, 0.000242, 0.000281, 0.000327
	White	Women	53	0.0002, 0.000233, 0.000272, 0.000317, 0.00037
	White	Women	54	0.000223, 0.000261, 0.000305, 0.000356, 0.000416
	White	Women	55	0.000248, 0.00029, 0.00034, 0.000398, 0.000467
	White	Women	56	0.000275, 0.000323, 0.000379, 0.000444, 0.000522

Variable	Race/ethnicity	Sex	Age	Distribution
	White	Women	57	0.000306, 0.00036, 0.000422, 0.000495, 0.000581
	White	Women	58	0.000343, 0.000402, 0.00047, 0.00055, 0.000645
	White	Women	59	0.000383, 0.000449, 0.000524, 0.000613, 0.000717
	White	Women	60	0.000425, 0.000498, 0.000584, 0.000684, 0.000802
	White	Women	61	0.000465, 0.000549, 0.000647, 0.000762, 0.000899
	White	Women	62	0.000506, 0.000601, 0.000712, 0.000845, 0.001
	White	Women	63	0.000547, 0.000653, 0.000779, 0.000929, 0.00111
	White	Women	64	0.00059, 0.000708, 0.000849, 0.00102, 0.00122
	White	Women	65	0.000636, 0.000767, 0.000924, 0.00111, 0.00134
	White	Women	66	0.00069, 0.000834, 0.00101, 0.00122, 0.00147
	White	Women	67	0.000755, 0.000914, 0.0011, 0.00133, 0.00162
	White	Women	68	0.000832, 0.00101, 0.00122, 0.00147, 0.00178
	White	Women	69	0.000921, 0.00111, 0.00135, 0.00163, 0.00197
	White	Women	70	0.00102, 0.00124, 0.0015, 0.00181, 0.00219
	White	Women	71	0.00114, 0.00138, 0.00167, 0.00202, 0.00244
	White	Women	72	0.00126, 0.00153, 0.00186, 0.00226, 0.00274
	White	Women	73	0.0014, 0.00171, 0.00208, 0.00253, 0.0031
	White	Women	74	0.00155, 0.0019, 0.00233, 0.00285, 0.0035
	White	Women	75	0.00172, 0.00212, 0.00261, 0.00321, 0.00397
	White	Women	76	0.00191, 0.00237, 0.00293, 0.00363, 0.0045
	White	Women	77	0.00214, 0.00266, 0.00331, 0.00411, 0.00512
	White	Women	78	0.0024, 0.003, 0.00375, 0.00468, 0.00585
	White	Women	79	0.00271, 0.0034, 0.00427, 0.00535, 0.00672
	White	Women	80	0.00307, 0.00387, 0.00488, 0.00614, 0.00775
	White	Women	81	0.00348, 0.00441, 0.00558, 0.00706, 0.00896
	White	Women	82	0.00394, 0.00502, 0.00638, 0.00811, 0.0103
	White	Women	83	0.00445, 0.00569, 0.00726, 0.00927, 0.0119
	White	Women	84	0.00498, 0.0064, 0.00819, 0.0105, 0.0135
CHD mortality rates for 2022 (0.01, 0.2, 0.5, 0.8. 0.99 percentiles of the empirical distribution produced during forecasting)				
	Black	Men	30	0.0000327, 0.0000449, 0.0000615, 0.0000842, 0.000116
	Black	Men	31	0.0000413, 0.0000553, 0.0000738, 0.0000986, 0.000132
	Black	Men	32	0.0000518, 0.0000676, 0.0000881, 0.000115, 0.00015
	Black	Men	33	0.0000644, 0.000082, 0.000104, 0.000133, 0.000169
	Black	Men	34	0.0000784, 0.000098, 0.000122, 0.000153, 0.000191
	Black	Men	35	0.0000929, 0.000115, 0.000142, 0.000175, 0.000217
	Black	Men	36	0.000108, 0.000133, 0.000163, 0.000201, 0.000248
	Black	Men	37	0.000123, 0.000151, 0.000186, 0.000229, 0.000282
	Black	Men	38	0.000139, 0.000171, 0.000211, 0.000259, 0.000319
	Black	Men	39	0.000156, 0.000193, 0.000237, 0.000291, 0.000359
	Black	Men	40	0.000175, 0.000216, 0.000265, 0.000326, 0.000401
	Black	Men	41	0.000196, 0.000241, 0.000296, 0.000363, 0.000447
	Black	Men	42	0.000217, 0.000268, 0.000329, 0.000405, 0.0005
	Black	Men	43	0.000239, 0.000296, 0.000367, 0.000454, 0.000564

Variable	Race/ethnicity	Sex	Age	Distribution
	Black	Men	44	0.000263, 0.000328, 0.00041, 0.000512, 0.00064
	Black	Men	45	0.00029, 0.000366, 0.00046, 0.000578, 0.000729
	Black	Men	46	0.000323, 0.00041, 0.000519, 0.000656, 0.000831
	Black	Men	47	0.000364, 0.000463, 0.000588, 0.000746, 0.00095
	Black	Men	48	0.000412, 0.000526, 0.00067, 0.000853, 0.00109
	Black	Men	49	0.00047, 0.000601, 0.000767, 0.000978, 0.00125
	Black	Men	50	0.000543, 0.000692, 0.00088, 0.00112, 0.00142
	Black	Men	51	0.000635, 0.000802, 0.00101, 0.00127, 0.0016
	Black	Men	52	0.000745, 0.000928, 0.00115, 0.00143, 0.00179
	Black	Men	53	0.000864, 0.00107, 0.00131, 0.00161, 0.00199
	Black	Men	54	0.000985, 0.00121, 0.00148, 0.00181, 0.00221
	Black	Men	55	0.00111, 0.00135, 0.00165, 0.00201, 0.00245
	Black	Men	56	0.00124, 0.0015, 0.00182, 0.00221, 0.00268
	Black	Men	57	0.00138, 0.00166, 0.002, 0.0024, 0.00289
	Black	Men	58	0.00153, 0.00182, 0.00218, 0.0026, 0.0031
	Black	Men	59	0.00168, 0.00199, 0.00236, 0.00279, 0.00331
	Black	Men	60	0.00183, 0.00216, 0.00254, 0.00299, 0.00352
	Black	Men	61	0.00199, 0.00233, 0.00272, 0.00317, 0.00371
	Black	Men	62	0.00216, 0.0025, 0.00289, 0.00335, 0.00388
	Black	Men	63	0.00232, 0.00266, 0.00306, 0.00351, 0.00403
	Black	Men	64	0.00247, 0.00282, 0.00322, 0.00367, 0.00419
	Black	Men	65	0.00262, 0.00298, 0.00339, 0.00385, 0.00438
	Black	Men	66	0.00275, 0.00314, 0.00357, 0.00406, 0.00462
	Black	Men	67	0.00289, 0.00331, 0.00378, 0.00431, 0.00493
	Black	Men	68	0.00304, 0.0035, 0.00402, 0.00461, 0.0053
	Black	Men	69	0.00322, 0.00372, 0.00429, 0.00494, 0.0057
	Black	Men	70	0.00343, 0.00397, 0.00458, 0.0053, 0.00613
	Black	Men	71	0.00366, 0.00424, 0.00491, 0.00568, 0.00657
	Black	Men	72	0.00391, 0.00454, 0.00525, 0.00609, 0.00706
	Black	Men	73	0.00417, 0.00485, 0.00563, 0.00653, 0.00759
	Black	Men	74	0.00446, 0.00519, 0.00603, 0.007, 0.00815
	Black	Men	75	0.0048, 0.00558, 0.00647, 0.00751, 0.00873
	Black	Men	76	0.00518, 0.00601, 0.00696, 0.00807, 0.00936
	Black	Men	77	0.00562, 0.0065, 0.00751, 0.00869, 0.0101
	Black	Men	78	0.00608, 0.00704, 0.00813, 0.00939, 0.0109
	Black	Men	79	0.00656, 0.00761, 0.00881, 0.0102, 0.0118
	Black	Men	80	0.00706, 0.00822, 0.00955, 0.0111, 0.0129
	Black	Men	81	0.00754, 0.00883, 0.0103, 0.0121, 0.0142
	Black	Men	82	0.00797, 0.00944, 0.0112, 0.0132, 0.0156
	Black	Men	83	0.00836, 0.01, 0.012, 0.0143, 0.0171
	Black	Men	84	0.00872, 0.0106, 0.0128, 0.0154, 0.0187
	Black	Women	30	0.0000135, 0.0000187, 0.0000258, 0.0000356, 0.0000493
	Black	Women	31	0.000017, 0.0000228, 0.0000304, 0.0000406, 0.0000544

Variable	Race/ethnicity	Sex	Age	Distribution
	Black	Women	32	0.000021, 0.0000274, 0.0000358, 0.0000467, 0.000061
	Black	Women	33	0.0000256, 0.0000328, 0.000042, 0.0000539, 0.0000691
	Black	Women	34	0.0000305, 0.0000388, 0.0000492, 0.0000623, 0.0000793
	Black	Women	35	0.0000354, 0.000045, 0.0000571, 0.0000724, 0.0000921
	Black	Women	36	0.0000404, 0.0000516, 0.0000657, 0.0000838, 0.000107
	Black	Women	37	0.0000458, 0.0000587, 0.0000751, 0.0000961, 0.000123
	Black	Women	38	0.0000515, 0.0000663, 0.0000853, 0.00011, 0.000141
	Black	Women	39	0.0000575, 0.0000745, 0.0000962, 0.000124, 0.000161
	Black	Women	40	0.0000646, 0.0000837, 0.000108, 0.00014, 0.000181
	Black	Women	41	0.0000734, 0.0000944, 0.000121, 0.000155, 0.0002
	Black	Women	42	0.0000846, 0.000107, 0.000136, 0.000171, 0.000217
	Black	Women	43	0.000098, 0.000122, 0.000152, 0.000189, 0.000236
	Black	Women	44	0.000113, 0.000139, 0.000171, 0.000211, 0.00026
	Black	Women	45	0.000129, 0.000158, 0.000194, 0.000237, 0.000291
	Black	Women	46	0.000148, 0.000181, 0.00022, 0.000268, 0.000328
	Black	Women	47	0.000172, 0.000208, 0.000252, 0.000305, 0.00037
	Black	Women	48	0.000198, 0.000239, 0.000289, 0.000349, 0.000422
	Black	Women	49	0.000223, 0.000273, 0.000332, 0.000405, 0.000494
	Black	Women	50	0.000252, 0.00031, 0.00038, 0.000467, 0.000575
	Black	Women	51	0.000291, 0.000355, 0.000433, 0.000529, 0.000646
	Black	Women	52	0.000339, 0.000408, 0.000491, 0.00059, 0.00071
	Black	Women	53	0.000386, 0.000462, 0.000552, 0.00066, 0.000791
	Black	Women	54	0.000428, 0.000515, 0.000619, 0.000744, 0.000896
	Black	Women	55	0.000476, 0.000574, 0.000691, 0.000832, 0.001
	Black	Women	56	0.000537, 0.000643, 0.00077, 0.000921, 0.0011
	Black	Women	57	0.000604, 0.000718, 0.000853, 0.00101, 0.00121
	Black	Women	58	0.000668, 0.000793, 0.00094, 0.00111, 0.00132
	Black	Women	59	0.000727, 0.000866, 0.00103, 0.00122, 0.00146
	Black	Women	60	0.000782, 0.000937, 0.00112, 0.00134, 0.0016
	Black	Women	61	0.000834, 0.00101, 0.00121, 0.00146, 0.00176
	Black	Women	62	0.00088, 0.00107, 0.0013, 0.00158, 0.00192
	Black	Women	63	0.00092, 0.00113, 0.00139, 0.00171, 0.0021
	Black	Women	64	0.000956, 0.00119, 0.00148, 0.00184, 0.00229
	Black	Women	65	0.000992, 0.00125, 0.00157, 0.00198, 0.0025
	Black	Women	66	0.00104, 0.00132, 0.00168, 0.00214, 0.00273
	Black	Women	67	0.0011, 0.00141, 0.00181, 0.00232, 0.00297
	Black	Women	68	0.00119, 0.00153, 0.00196, 0.00252, 0.00324
	Black	Women	69	0.0013, 0.00167, 0.00214, 0.00275, 0.00353
	Black	Women	70	0.00143, 0.00183, 0.00234, 0.00299, 0.00383
	Black	Women	71	0.00158, 0.00201, 0.00256, 0.00326, 0.00416
	Black	Women	72	0.00174, 0.00221, 0.0028, 0.00355, 0.00451
	Black	Women	73	0.00191, 0.00242, 0.00306, 0.00387, 0.0049
	Black	Women	74	0.00208, 0.00264, 0.00333, 0.00421, 0.00534

Variable	Race/ethnicity	Sex	Age	Distribution
	Black	Women	75	0.00227, 0.00287, 0.00364, 0.0046, 0.00584
	Black	Women	76	0.00246, 0.00313, 0.00398, 0.00506, 0.00645
	Black	Women	77	0.00265, 0.00341, 0.00437, 0.00559, 0.00719
	Black	Women	78	0.00285, 0.0037, 0.0048, 0.00622, 0.00808
	Black	Women	79	0.00305, 0.00402, 0.00528, 0.00693, 0.00912
	Black	Women	80	0.00327, 0.00436, 0.00581, 0.00773, 0.0103
	Black	Women	81	0.00351, 0.00474, 0.00639, 0.0086, 0.0116
	Black	Women	82	0.00379, 0.00516, 0.007, 0.0095, 0.0129
	Black	Women	83	0.00411, 0.00562, 0.00765, 0.0104, 0.0143
	Black	Women	84	0.00444, 0.00609, 0.00832, 0.0114, 0.0156
	Hispanic	Men	30	0.0000111, 0.0000153, 0.0000209, 0.0000286, 0.0000394
	Hispanic	Men	31	0.0000136, 0.0000184, 0.0000248, 0.0000334, 0.0000452
	Hispanic	Men	32	0.0000167, 0.0000221, 0.0000294, 0.0000389, 0.0000518
	Hispanic	Men	33	0.0000205, 0.0000267, 0.0000347, 0.000045, 0.0000587
	Hispanic	Men	34	0.0000253, 0.0000322, 0.0000408, 0.0000518, 0.0000658
	Hispanic	Men	35	0.000031, 0.0000386, 0.0000478, 0.0000593, 0.0000737
	Hispanic	Men	36	0.0000373, 0.0000456, 0.0000557, 0.000068, 0.0000832
	Hispanic	Men	37	0.000044, 0.0000534, 0.0000646, 0.0000783, 0.000095
	Hispanic	Men	38	0.0000513, 0.000062, 0.0000747, 0.0000901, 0.000109
	Hispanic	Men	39	0.0000597, 0.0000718, 0.0000862, 0.000103, 0.000124
	Hispanic	Men	40	0.0000697, 0.0000832, 0.0000991, 0.000118, 0.000141
	Hispanic	Men	41	0.0000814, 0.0000963, 0.000114, 0.000134, 0.000159
	Hispanic	Men	42	0.0000946, 0.000111, 0.00013, 0.000153, 0.00018
	Hispanic	Men	43	0.00011, 0.000128, 0.000149, 0.000174, 0.000203
	Hispanic	Men	44	0.000128, 0.000148, 0.000171, 0.000198, 0.000229
	Hispanic	Men	45	0.000148, 0.00017, 0.000196, 0.000226, 0.00026
	Hispanic	Men	46	0.000169, 0.000195, 0.000225, 0.00026, 0.0003
	Hispanic	Men	47	0.000193, 0.000224, 0.000259, 0.000299, 0.000346
	Hispanic	Men	48	0.000226, 0.000259, 0.000298, 0.000342, 0.000394
	Hispanic	Men	49	0.000264, 0.000301, 0.000344, 0.000392, 0.000448
	Hispanic	Men	50	0.000305, 0.000348, 0.000397, 0.000452, 0.000516
	Hispanic	Men	51	0.000349, 0.0004, 0.000457, 0.000523, 0.000599
	Hispanic	Men	52	0.000396, 0.000456, 0.000525, 0.000603, 0.000695
	Hispanic	Men	53	0.000445, 0.000517, 0.000598, 0.000693, 0.000804
	Hispanic	Men	54	0.000497, 0.000581, 0.000677, 0.00079, 0.000923
	Hispanic	Men	55	0.000556, 0.000651, 0.00076, 0.000889, 0.00104
	Hispanic	Men	56	0.000624, 0.000728, 0.000847, 0.000986, 0.00115
	Hispanic	Men	57	0.000699, 0.00081, 0.000938, 0.00109, 0.00126
	Hispanic	Men	58	0.000779, 0.000897, 0.00103, 0.00119, 0.00137
	Hispanic	Men	59	0.000859, 0.000987, 0.00113, 0.0013, 0.00149
	Hispanic	Men	60	0.000938, 0.00108, 0.00123, 0.00141, 0.00162
	Hispanic	Men	61	0.00102, 0.00117, 0.00134, 0.00153, 0.00176
	Hispanic	Men	62	0.00109, 0.00126, 0.00144, 0.00166, 0.00191

Variable	Race/ethnicity	Sex	Age	Distribution
	Hispanic	Men	63	0.00116, 0.00134, 0.00155, 0.00178, 0.00206
	Hispanic	Men	64	0.00124, 0.00143, 0.00166, 0.00191, 0.00221
	Hispanic	Men	65	0.00132, 0.00153, 0.00177, 0.00205, 0.00238
	Hispanic	Men	66	0.0014, 0.00163, 0.00189, 0.0022, 0.00256
	Hispanic	Men	67	0.00149, 0.00174, 0.00203, 0.00237, 0.00277
	Hispanic	Men	68	0.00158, 0.00186, 0.00219, 0.00257, 0.00303
	Hispanic	Men	69	0.00169, 0.002, 0.00237, 0.00281, 0.00334
	Hispanic	Men	70	0.00181, 0.00217, 0.00259, 0.00309, 0.00369
	Hispanic	Men	71	0.00195, 0.00235, 0.00283, 0.0034, 0.00409
	Hispanic	Men	72	0.00211, 0.00256, 0.00309, 0.00374, 0.00454
	Hispanic	Men	73	0.0023, 0.00279, 0.00339, 0.00412, 0.00501
	Hispanic	Men	74	0.00251, 0.00306, 0.00372, 0.00453, 0.00551
	Hispanic	Men	75	0.00277, 0.00337, 0.00409, 0.00497, 0.00605
	Hispanic	Men	76	0.00306, 0.00372, 0.00451, 0.00547, 0.00664
	Hispanic	Men	77	0.00341, 0.00413, 0.00499, 0.00603, 0.0073
	Hispanic	Men	78	0.00382, 0.0046, 0.00554, 0.00667, 0.00804
	Hispanic	Men	79	0.00426, 0.00513, 0.00616, 0.0074, 0.0089
	Hispanic	Men	80	0.00471, 0.00568, 0.00684, 0.00823, 0.00993
	Hispanic	Men	81	0.00513, 0.00623, 0.00756, 0.00917, 0.0112
	Hispanic	Men	82	0.00551, 0.00677, 0.00831, 0.0102, 0.0125
	Hispanic	Men	83	0.00586, 0.00729, 0.00906, 0.0113, 0.014
	Hispanic	Men	84	0.00621, 0.00781, 0.00982, 0.0123, 0.0155
	Hispanic	Women	30	0.00000297, 0.00000421, 0.00000597, 0.00000845, 0.000012
	Hispanic	Women	31	0.00000358, 0.00000498, 0.00000691, 0.00000958, 0.0000133
	Hispanic	Women	32	0.00000431, 0.00000589, 0.00000801, 0.0000109, 0.0000149
	Hispanic	Women	33	0.00000518, 0.00000695, 0.00000928, 0.0000124, 0.0000166
	Hispanic	Women	34	0.00000614, 0.00000813, 0.0000107, 0.0000142, 0.0000188
	Hispanic	Women	35	0.00000716, 0.00000943, 0.0000124, 0.0000163, 0.0000214
	Hispanic	Women	36	0.00000822, 0.0000108, 0.0000142, 0.0000187, 0.0000246
	Hispanic	Women	37	0.00000936, 0.0000124, 0.0000163, 0.0000214, 0.0000282
	Hispanic	Women	38	0.0000106, 0.0000141, 0.0000185, 0.0000245, 0.0000323
	Hispanic	Women	39	0.0000121, 0.000016, 0.0000211, 0.0000278, 0.0000368
	Hispanic	Women	40	0.0000139, 0.0000183, 0.000024, 0.0000315, 0.0000415
	Hispanic	Women	41	0.0000161, 0.000021, 0.0000273, 0.0000355, 0.0000462
	Hispanic	Women	42	0.000019, 0.0000243, 0.0000311, 0.0000397, 0.0000508
	Hispanic	Women	43	0.0000225, 0.0000283, 0.0000355, 0.0000445, 0.0000559
	Hispanic	Women	44	0.0000267, 0.0000329, 0.0000406, 0.0000501, 0.0000619
	Hispanic	Women	45	0.0000317, 0.0000386, 0.0000468, 0.0000567, 0.0000689
	Hispanic	Women	46	0.000038, 0.0000453, 0.000054, 0.0000644, 0.0000768
	Hispanic	Women	47	0.0000456, 0.0000534, 0.0000626, 0.0000733, 0.000086
	Hispanic	Women	48	0.0000542, 0.0000628, 0.0000727, 0.0000841, 0.0000976
	Hispanic	Women	49	0.0000631, 0.0000731, 0.0000845, 0.0000978, 0.000113
	Hispanic	Women	50	0.0000719, 0.0000842, 0.0000985, 0.000115, 0.000135

Variable	Race/ethnicity	Sex	Age	Distribution
	Hispanic	Women	51	0.0000811, 0.0000966, 0.000115, 0.000136, 0.000162
	Hispanic	Women	52	0.0000914, 0.000111, 0.000134, 0.000161, 0.000195
	Hispanic	Women	53	0.000103, 0.000127, 0.000155, 0.00019, 0.000233
	Hispanic	Women	54	0.000118, 0.000146, 0.00018, 0.000223, 0.000276
	Hispanic	Women	55	0.000134, 0.000168, 0.000209, 0.00026, 0.000324
	Hispanic	Women	56	0.000154, 0.000193, 0.000241, 0.000301, 0.000377
	Hispanic	Women	57	0.000175, 0.000221, 0.000277, 0.000348, 0.000439
	Hispanic	Women	58	0.000198, 0.000251, 0.000318, 0.000402, 0.00051
	Hispanic	Women	59	0.000223, 0.000285, 0.000363, 0.000463, 0.000592
	Hispanic	Women	60	0.000249, 0.000321, 0.000413, 0.000531, 0.000685
	Hispanic	Women	61	0.000276, 0.000359, 0.000466, 0.000605, 0.000787
	Hispanic	Women	62	0.000306, 0.0004, 0.000523, 0.000683, 0.000894
	Hispanic	Women	63	0.000338, 0.000444, 0.000581, 0.000762, 0.001
	Hispanic	Women	64	0.000374, 0.000491, 0.000643, 0.000842, 0.0011
	Hispanic	Women	65	0.000415, 0.000543, 0.000708, 0.000924, 0.00121
	Hispanic	Women	66	0.000463, 0.000602, 0.000781, 0.00101, 0.00132
	Hispanic	Women	67	0.000516, 0.000669, 0.000864, 0.00112, 0.00145
	Hispanic	Women	68	0.000576, 0.000745, 0.000961, 0.00124, 0.0016
	Hispanic	Women	69	0.000645, 0.000833, 0.00107, 0.00138, 0.00178
	Hispanic	Women	70	0.000723, 0.000933, 0.0012, 0.00155, 0.00199
	Hispanic	Women	71	0.000813, 0.00105, 0.00135, 0.00173, 0.00223
	Hispanic	Women	72	0.000914, 0.00118, 0.00151, 0.00195, 0.00251
	Hispanic	Women	73	0.00103, 0.00133, 0.00171, 0.00219, 0.00283
	Hispanic	Women	74	0.00116, 0.00149, 0.00193, 0.00248, 0.00321
	Hispanic	Women	75	0.0013, 0.00168, 0.00218, 0.00282, 0.00366
	Hispanic	Women	76	0.00146, 0.0019, 0.00247, 0.00322, 0.00419
	Hispanic	Women	77	0.00164, 0.00215, 0.00281, 0.00368, 0.00483
	Hispanic	Women	78	0.00184, 0.00243, 0.0032, 0.00422, 0.00557
	Hispanic	Women	79	0.00208, 0.00276, 0.00366, 0.00485, 0.00644
	Hispanic	Women	80	0.00234, 0.00313, 0.00418, 0.00558, 0.00747
	Hispanic	Women	81	0.00262, 0.00354, 0.00477, 0.00642, 0.00866
	Hispanic	Women	82	0.00292, 0.00398, 0.00542, 0.00737, 0.0101
	Hispanic	Women	83	0.00321, 0.00444, 0.00612, 0.00843, 0.0117
	Hispanic	Women	84	0.00351, 0.00491, 0.00685, 0.00956, 0.0134
	White	Men	30	0.0000197, 0.0000262, 0.0000347, 0.0000459, 0.000061
	White	Men	31	0.0000251, 0.0000327, 0.0000425, 0.0000551, 0.0000718
	White	Men	32	0.0000315, 0.0000404, 0.0000517, 0.0000661, 0.0000847
	White	Men	33	0.000039, 0.0000494, 0.0000624, 0.000079, 0.0001
	White	Men	34	0.0000472, 0.0000594, 0.0000747, 0.000094, 0.000118
	White	Men	35	0.000056, 0.0000705, 0.0000886, 0.000111, 0.00014
	White	Men	36	0.0000658, 0.0000828, 0.000104, 0.000131, 0.000165
	White	Men	37	0.0000772, 0.000097, 0.000122, 0.000152, 0.000191
	White	Men	38	0.0000913, 0.000114, 0.000141, 0.000176, 0.000219

Variable	Race/ethnicity	Sex	Age	Distribution
	White	Men	39	0.000109, 0.000134, 0.000164, 0.0002, 0.000246
	White	Men	40	0.00013, 0.000156, 0.000188, 0.000227, 0.000273
	White	Men	41	0.000153, 0.000182, 0.000215, 0.000255, 0.000302
	White	Men	42	0.000178, 0.000209, 0.000244, 0.000286, 0.000334
	White	Men	43	0.000206, 0.000238, 0.000276, 0.00032, 0.000371
	White	Men	44	0.000236, 0.000271, 0.000312, 0.000358, 0.000411
	White	Men	45	0.000273, 0.00031, 0.000352, 0.000399, 0.000454
	White	Men	46	0.000315, 0.000354, 0.000397, 0.000446, 0.000501
	White	Men	47	0.000364, 0.000405, 0.00045, 0.0005, 0.000557
	White	Men	48	0.000417, 0.000462, 0.000511, 0.000565, 0.000625
	White	Men	49	0.000476, 0.000526, 0.000579, 0.000639, 0.000705
	White	Men	50	0.000545, 0.000599, 0.000657, 0.00072, 0.000791
	White	Men	51	0.000625, 0.000681, 0.000741, 0.000807, 0.00088
	White	Men	52	0.000709, 0.000769, 0.000833, 0.000902, 0.000977
	White	Men	53	0.000789, 0.000857, 0.000929, 0.00101, 0.00109
	White	Men	54	0.000862, 0.000942, 0.00103, 0.00113, 0.00123
	White	Men	55	0.000933, 0.00103, 0.00113, 0.00125, 0.00138
	White	Men	56	0.001, 0.00112, 0.00124, 0.00137, 0.00153
	White	Men	57	0.00108, 0.0012, 0.00135, 0.0015, 0.00168
	White	Men	58	0.00115, 0.0013, 0.00145, 0.00163, 0.00184
	White	Men	59	0.00124, 0.00139, 0.00157, 0.00177, 0.00199
	White	Men	60	0.00133, 0.0015, 0.00169, 0.0019, 0.00215
	White	Men	61	0.00142, 0.0016, 0.00181, 0.00204, 0.00231
	White	Men	62	0.00151, 0.00171, 0.00194, 0.00219, 0.00247
	White	Men	63	0.0016, 0.00182, 0.00206, 0.00233, 0.00265
	White	Men	64	0.00168, 0.00191, 0.00218, 0.00248, 0.00282
	White	Men	65	0.00175, 0.00201, 0.0023, 0.00263, 0.00302
	White	Men	66	0.00182, 0.00211, 0.00243, 0.00281, 0.00324
	White	Men	67	0.0019, 0.00222, 0.00258, 0.00301, 0.00351
	White	Men	68	0.00199, 0.00234, 0.00276, 0.00325, 0.00383
	White	Men	69	0.00211, 0.0025, 0.00297, 0.00352, 0.00419
	White	Men	70	0.00226, 0.0027, 0.00322, 0.00384, 0.00458
	White	Men	71	0.00246, 0.00294, 0.0035, 0.00418, 0.005
	White	Men	72	0.00268, 0.0032, 0.00382, 0.00456, 0.00546
	White	Men	73	0.00292, 0.00349, 0.00417, 0.00498, 0.00596
	White	Men	74	0.00319, 0.00382, 0.00456, 0.00545, 0.00652
	White	Men	75	0.00348, 0.00417, 0.00499, 0.00597, 0.00716
	White	Men	76	0.0038, 0.00456, 0.00548, 0.00657, 0.00789
	White	Men	77	0.00416, 0.00502, 0.00604, 0.00727, 0.00877
	White	Men	78	0.00458, 0.00554, 0.0067, 0.00809, 0.00979
	White	Men	79	0.00509, 0.00618, 0.00748, 0.00905, 0.011
	White	Men	80	0.0057, 0.00692, 0.00839, 0.0102, 0.0123
	White	Men	81	0.00642, 0.00779, 0.00943, 0.0114, 0.0138

Variable	Race/ethnicity	Sex	Age	Distribution
	White	Men	82	0.00722, 0.00875, 0.0106, 0.0128, 0.0155
	White	Men	83	0.00808, 0.00977, 0.0118, 0.0142, 0.0172
	White	Men	84	0.00896, 0.0108, 0.013, 0.0157, 0.019
	White	Women	30	0.00000507, 0.00000755, 0.0000112, 0.0000166, 0.0000248
	White	Women	31	0.00000661, 0.00000953, 0.0000137, 0.0000197, 0.0000283
	White	Women	32	0.00000865, 0.000012, 0.0000166, 0.000023, 0.000032
	White	Women	33	0.0000112, 0.0000151, 0.0000201, 0.0000269, 0.000036
	White	Women	34	0.0000143, 0.0000186, 0.0000241, 0.0000313, 0.0000407
	White	Women	35	0.0000178, 0.0000226, 0.0000287, 0.0000365, 0.0000464
	White	Women	36	0.0000215, 0.000027, 0.0000339, 0.0000424, 0.0000533
	White	Women	37	0.0000254, 0.0000317, 0.0000395, 0.0000493, 0.0000616
	White	Women	38	0.0000292, 0.0000366, 0.0000457, 0.0000572, 0.0000717
	White	Women	39	0.0000328, 0.0000415, 0.0000524, 0.0000661, 0.0000836
	White	Women	40	0.0000365, 0.0000466, 0.0000594, 0.0000758, 0.0000969
	White	Women	41	0.0000404, 0.000052, 0.0000668, 0.0000859, 0.000111
	White	Women	42	0.0000448, 0.0000579, 0.0000747, 0.0000963, 0.000124
	White	Women	43	0.0000498, 0.0000644, 0.0000832, 0.000107, 0.000139
	White	Women	44	0.0000552, 0.0000716, 0.0000927, 0.00012, 0.000156
	White	Women	45	0.0000617, 0.0000799, 0.000103, 0.000134, 0.000173
	White	Women	46	0.0000703, 0.0000902, 0.000115, 0.000148, 0.00019
	White	Women	47	0.0000821, 0.000103, 0.000129, 0.000162, 0.000204
	White	Women	48	0.0000974, 0.000119, 0.000145, 0.000177, 0.000217
	White	Women	49	0.000116, 0.000138, 0.000164, 0.000195, 0.000233
	White	Women	50	0.000135, 0.000158, 0.000185, 0.000217, 0.000255
	White	Women	51	0.000154, 0.00018, 0.00021, 0.000244, 0.000284
	White	Women	52	0.000174, 0.000203, 0.000237, 0.000276, 0.000321
	White	Women	53	0.000195, 0.000228, 0.000267, 0.000311, 0.000364
	White	Women	54	0.000218, 0.000255, 0.000299, 0.00035, 0.00041
	White	Women	55	0.000242, 0.000285, 0.000334, 0.000392, 0.000461
	White	Women	56	0.000269, 0.000317, 0.000372, 0.000438, 0.000515
	White	Women	57	0.0003, 0.000353, 0.000415, 0.000488, 0.000574
	White	Women	58	0.000336, 0.000395, 0.000463, 0.000543, 0.000639
	White	Women	59	0.000375, 0.000441, 0.000516, 0.000605, 0.00071
	White	Women	60	0.000416, 0.000489, 0.000575, 0.000675, 0.000795
	White	Women	61	0.000455, 0.000539, 0.000637, 0.000752, 0.00089
	White	Women	62	0.000494, 0.000589, 0.0007, 0.000833, 0.000992
	White	Women	63	0.000534, 0.00064, 0.000765, 0.000915, 0.0011
	White	Women	64	0.000576, 0.000693, 0.000833, 0.001, 0.0012
	White	Women	65	0.000621, 0.00075, 0.000905, 0.00109, 0.00132
	White	Women	66	0.000673, 0.000815, 0.000987, 0.00119, 0.00145
	White	Women	67	0.000735, 0.000892, 0.00108, 0.00131, 0.00159
	White	Women	68	0.00081, 0.000982, 0.00119, 0.00144, 0.00175
	White	Women	69	0.000896, 0.00109, 0.00132, 0.00159, 0.00193

Variable	Race/ethnicity	Sex	Age	Distribution
	White	Women	70	0.000995, 0.00121, 0.00146, 0.00177, 0.00215
	White	Women	71	0.00111, 0.00134, 0.00163, 0.00198, 0.0024
	White	Women	72	0.00123, 0.00149, 0.00182, 0.00221, 0.0027
	White	Women	73	0.00136, 0.00166, 0.00203, 0.00248, 0.00304
	White	Women	74	0.0015, 0.00185, 0.00227, 0.00279, 0.00344
	White	Women	75	0.00167, 0.00207, 0.00255, 0.00315, 0.00389
	White	Women	76	0.00186, 0.00231, 0.00287, 0.00356, 0.00442
	White	Women	77	0.00208, 0.0026, 0.00323, 0.00403, 0.00503
	White	Women	78	0.00234, 0.00293, 0.00366, 0.00458, 0.00575
	White	Women	79	0.00263, 0.00332, 0.00417, 0.00524, 0.0066
	White	Women	80	0.00298, 0.00377, 0.00477, 0.00602, 0.00762
	White	Women	81	0.00338, 0.0043, 0.00546, 0.00692, 0.0088
	White	Women	82	0.00383, 0.0049, 0.00624, 0.00795, 0.0102
	White	Women	83	0.00432, 0.00555, 0.0071, 0.00908, 0.0117
	White	Women	84	0.00484, 0.00623, 0.008, 0.0103, 0.0132
CHD mortality rates for 2023 (0.01, 0.2, 0.5, 0.8. 0.99 percentiles of the empirical distribution produced during forecasting)				
	Black	Men	30	0.0000318, 0.0000441, 0.0000611, 0.0000845, 0.000117
	Black	Men	31	0.0000403, 0.0000545, 0.0000734, 0.0000989, 0.000134
	Black	Men	32	0.0000506, 0.0000667, 0.0000876, 0.000115, 0.000152
	Black	Men	33	0.0000629, 0.0000809, 0.000104, 0.000133, 0.000171
	Black	Men	34	0.0000767, 0.0000967, 0.000122, 0.000153, 0.000193
	Black	Men	35	0.0000908, 0.000113, 0.000141, 0.000176, 0.000219
	Black	Men	36	0.000105, 0.000131, 0.000162, 0.000201, 0.00025
	Black	Men	37	0.00012, 0.000149, 0.000185, 0.000229, 0.000285
	Black	Men	38	0.000135, 0.000168, 0.000208, 0.000259, 0.000322
	Black	Men	39	0.000152, 0.000189, 0.000234, 0.00029, 0.000361
	Black	Men	40	0.00017, 0.000211, 0.000261, 0.000324, 0.000402
	Black	Men	41	0.00019, 0.000235, 0.000291, 0.00036, 0.000446
	Black	Men	42	0.000211, 0.000261, 0.000323, 0.0004, 0.000496
	Black	Men	43	0.000232, 0.000289, 0.000359, 0.000447, 0.000556
	Black	Men	44	0.000255, 0.00032, 0.000401, 0.000502, 0.00063
	Black	Men	45	0.000282, 0.000356, 0.000449, 0.000567, 0.000717
	Black	Men	46	0.000314, 0.000399, 0.000506, 0.000642, 0.000816
	Black	Men	47	0.000354, 0.000451, 0.000574, 0.00073, 0.000931
	Black	Men	48	0.000401, 0.000513, 0.000655, 0.000835, 0.00107
	Black	Men	49	0.000459, 0.000587, 0.00075, 0.000958, 0.00123
	Black	Men	50	0.000531, 0.000677, 0.000862, 0.0011, 0.0014
	Black	Men	51	0.000621, 0.000785, 0.00099, 0.00125, 0.00158
	Black	Men	52	0.000729, 0.00091, 0.00113, 0.00141, 0.00176
	Black	Men	53	0.000847, 0.00105, 0.00129, 0.00159, 0.00196
	Black	Men	54	0.000966, 0.00119, 0.00145, 0.00178, 0.00219
	Black	Men	55	0.00109, 0.00133, 0.00162, 0.00198, 0.00243
	Black	Men	56	0.00121, 0.00148, 0.0018, 0.00218, 0.00266

Variable	Race/ethnicity	Sex	Age	Distribution
	Black	Men	57	0.00135, 0.00163, 0.00197, 0.00238, 0.00287
	Black	Men	58	0.0015, 0.00179, 0.00215, 0.00257, 0.00308
	Black	Men	59	0.00164, 0.00196, 0.00233, 0.00276, 0.00329
	Black	Men	60	0.00179, 0.00212, 0.0025, 0.00296, 0.00349
	Black	Men	61	0.00195, 0.00229, 0.00268, 0.00314, 0.00368
	Black	Men	62	0.0021, 0.00245, 0.00284, 0.0033, 0.00384
	Black	Men	63	0.00226, 0.00261, 0.003, 0.00346, 0.00399
	Black	Men	64	0.00241, 0.00276, 0.00316, 0.00361, 0.00414
	Black	Men	65	0.00254, 0.00291, 0.00332, 0.00378, 0.00432
	Black	Men	66	0.00268, 0.00306, 0.00349, 0.00399, 0.00456
	Black	Men	67	0.00281, 0.00322, 0.0037, 0.00423, 0.00486
	Black	Men	68	0.00296, 0.00341, 0.00393, 0.00452, 0.00522
	Black	Men	69	0.00313, 0.00362, 0.00419, 0.00485, 0.00562
	Black	Men	70	0.00333, 0.00387, 0.00448, 0.0052, 0.00604
	Black	Men	71	0.00355, 0.00413, 0.0048, 0.00557, 0.00648
	Black	Men	72	0.00379, 0.00442, 0.00513, 0.00597, 0.00695
	Black	Men	73	0.00405, 0.00472, 0.0055, 0.0064, 0.00746
	Black	Men	74	0.00432, 0.00505, 0.00589, 0.00686, 0.00801
	Black	Men	75	0.00465, 0.00542, 0.00631, 0.00736, 0.00858
	Black	Men	76	0.00502, 0.00584, 0.00679, 0.0079, 0.00919
	Black	Men	77	0.00544, 0.00632, 0.00733, 0.00851, 0.00989
	Black	Men	78	0.00589, 0.00684, 0.00793, 0.0092, 0.0107
	Black	Men	79	0.00635, 0.0074, 0.0086, 0.01, 0.0116
	Black	Men	80	0.00683, 0.00799, 0.00932, 0.0109, 0.0127
	Black	Men	81	0.00729, 0.00858, 0.0101, 0.0119, 0.014
	Black	Men	82	0.00772, 0.00917, 0.0109, 0.0129, 0.0153
	Black	Men	83	0.00809, 0.00973, 0.0117, 0.014, 0.0169
	Black	Men	84	0.00844, 0.0103, 0.0125, 0.0151, 0.0184
	Black	Women	30	0.0000132, 0.0000184, 0.0000257, 0.0000357, 0.00005
	Black	Women	31	0.0000167, 0.0000225, 0.0000303, 0.0000407, 0.000055
	Black	Women	32	0.0000206, 0.0000271, 0.0000357, 0.0000468, 0.0000617
	Black	Women	33	0.0000251, 0.0000325, 0.0000419, 0.0000541, 0.0000699
	Black	Women	34	0.00003, 0.0000384, 0.000049, 0.0000626, 0.0000802
	Black	Women	35	0.0000348, 0.0000445, 0.0000569, 0.0000727, 0.0000931
	Black	Women	36	0.0000396, 0.000051, 0.0000655, 0.0000841, 0.000108
	Black	Women	37	0.0000448, 0.000058, 0.0000747, 0.0000963, 0.000124
	Black	Women	38	0.0000504, 0.0000654, 0.0000847, 0.00011, 0.000142
	Black	Women	39	0.0000562, 0.0000734, 0.0000955, 0.000124, 0.000162
	Black	Women	40	0.000063, 0.0000823, 0.000107, 0.000139, 0.000182
	Black	Women	41	0.0000716, 0.0000927, 0.00012, 0.000155, 0.0002
	Black	Women	42	0.0000824, 0.000105, 0.000134, 0.00017, 0.000217
	Black	Women	43	0.0000954, 0.00012, 0.00015, 0.000187, 0.000235
	Black	Women	44	0.00011, 0.000136, 0.000168, 0.000208, 0.000258

Variable	Race/ethnicity	Sex	Age	Distribution
	Black	Women	45	0.000125, 0.000155, 0.00019, 0.000234, 0.000289
	Black	Women	46	0.000144, 0.000176, 0.000216, 0.000265, 0.000325
	Black	Women	47	0.000167, 0.000203, 0.000247, 0.000301, 0.000367
	Black	Women	48	0.000192, 0.000234, 0.000284, 0.000345, 0.000419
	Black	Women	49	0.000218, 0.000267, 0.000327, 0.0004, 0.00049
	Black	Women	50	0.000246, 0.000304, 0.000375, 0.000462, 0.000571
	Black	Women	51	0.000285, 0.000349, 0.000428, 0.000524, 0.000642
	Black	Women	52	0.000332, 0.000402, 0.000485, 0.000584, 0.000706
	Black	Women	53	0.000378, 0.000455, 0.000546, 0.000655, 0.000788
	Black	Women	54	0.000419, 0.000507, 0.000612, 0.000739, 0.000894
	Black	Women	55	0.000467, 0.000566, 0.000684, 0.000827, 0.001
	Black	Women	56	0.000527, 0.000634, 0.000762, 0.000916, 0.0011
	Black	Women	57	0.000593, 0.000708, 0.000845, 0.00101, 0.0012
	Black	Women	58	0.000656, 0.000782, 0.000931, 0.00111, 0.00132
	Black	Women	59	0.000713, 0.000854, 0.00102, 0.00122, 0.00146
	Black	Women	60	0.000767, 0.000923, 0.00111, 0.00133, 0.0016
	Black	Women	61	0.000817, 0.000991, 0.0012, 0.00145, 0.00176
	Black	Women	62	0.000862, 0.00105, 0.00129, 0.00157, 0.00192
	Black	Women	63	0.000899, 0.00111, 0.00137, 0.00169, 0.00209
	Black	Women	64	0.000933, 0.00117, 0.00146, 0.00182, 0.00228
	Black	Women	65	0.000968, 0.00123, 0.00155, 0.00196, 0.00248
	Black	Women	66	0.00101, 0.0013, 0.00166, 0.00211, 0.00271
	Black	Women	67	0.00107, 0.00138, 0.00178, 0.00229, 0.00295
	Black	Women	68	0.00115, 0.00149, 0.00193, 0.00249, 0.00322
	Black	Women	69	0.00126, 0.00163, 0.0021, 0.00271, 0.0035
	Black	Women	70	0.00139, 0.00179, 0.0023, 0.00295, 0.0038
	Black	Women	71	0.00154, 0.00197, 0.00252, 0.00322, 0.00412
	Black	Women	72	0.00169, 0.00216, 0.00275, 0.0035, 0.00447
	Black	Women	73	0.00185, 0.00236, 0.003, 0.00381, 0.00486
	Black	Women	74	0.00202, 0.00257, 0.00327, 0.00415, 0.00529
	Black	Women	75	0.0022, 0.00281, 0.00357, 0.00454, 0.00579
	Black	Women	76	0.00239, 0.00306, 0.0039, 0.00499, 0.00639
	Black	Women	77	0.00258, 0.00333, 0.00428, 0.00552, 0.00713
	Black	Women	78	0.00277, 0.00361, 0.00471, 0.00613, 0.00801
	Black	Women	79	0.00296, 0.00392, 0.00518, 0.00684, 0.00906
	Black	Women	80	0.00317, 0.00426, 0.0057, 0.00764, 0.0103
	Black	Women	81	0.00341, 0.00463, 0.00627, 0.0085, 0.0116
	Black	Women	82	0.00368, 0.00504, 0.00688, 0.0094, 0.0129
	Black	Women	83	0.00398, 0.00548, 0.00752, 0.0103, 0.0142
	Black	Women	84	0.0043, 0.00594, 0.00818, 0.0113, 0.0156
	Hispanic	Men	30	0.0000108, 0.000015, 0.0000208, 0.0000287, 0.0000399
	Hispanic	Men	31	0.0000133, 0.0000182, 0.0000247, 0.0000335, 0.0000456
	Hispanic	Men	32	0.0000163, 0.0000219, 0.0000292, 0.000039, 0.0000522

Variable	Race/ethnicity	Sex	Age	Distribution
	Hispanic	Men	33	0.0000201, 0.0000264, 0.0000345, 0.0000451, 0.0000592
	Hispanic	Men	34	0.0000249, 0.0000318, 0.0000406, 0.0000519, 0.0000664
	Hispanic	Men	35	0.0000304, 0.0000381, 0.0000476, 0.0000594, 0.0000744
	Hispanic	Men	36	0.0000365, 0.000045, 0.0000554, 0.0000681, 0.000084
	Hispanic	Men	37	0.0000429, 0.0000525, 0.0000641, 0.0000783, 0.0000958
	Hispanic	Men	38	0.0000499, 0.0000609, 0.000074, 0.00009, 0.00011
	Hispanic	Men	39	0.000058, 0.0000704, 0.0000852, 0.000103, 0.000125
	Hispanic	Men	40	0.0000677, 0.0000814, 0.0000978, 0.000117, 0.000141
	Hispanic	Men	41	0.000079, 0.0000941, 0.000112, 0.000133, 0.000159
	Hispanic	Men	42	0.0000919, 0.000109, 0.000128, 0.000151, 0.000178
	Hispanic	Men	43	0.000107, 0.000125, 0.000146, 0.000171, 0.0002
	Hispanic	Men	44	0.000124, 0.000144, 0.000167, 0.000194, 0.000226
	Hispanic	Men	45	0.000143, 0.000166, 0.000192, 0.000221, 0.000256
	Hispanic	Men	46	0.000164, 0.00019, 0.00022, 0.000254, 0.000295
	Hispanic	Men	47	0.000188, 0.000218, 0.000253, 0.000293, 0.00034
	Hispanic	Men	48	0.000219, 0.000253, 0.000291, 0.000335, 0.000386
	Hispanic	Men	49	0.000257, 0.000294, 0.000336, 0.000384, 0.00044
	Hispanic	Men	50	0.000297, 0.00034, 0.000389, 0.000444, 0.000508
	Hispanic	Men	51	0.000341, 0.000391, 0.000448, 0.000514, 0.00059
	Hispanic	Men	52	0.000388, 0.000447, 0.000515, 0.000594, 0.000685
	Hispanic	Men	53	0.000436, 0.000507, 0.000589, 0.000684, 0.000795
	Hispanic	Men	54	0.000487, 0.000571, 0.000667, 0.000781, 0.000914
	Hispanic	Men	55	0.000545, 0.00064, 0.00075, 0.000879, 0.00103
	Hispanic	Men	56	0.000612, 0.000716, 0.000836, 0.000976, 0.00114
	Hispanic	Men	57	0.000686, 0.000797, 0.000926, 0.00107, 0.00125
	Hispanic	Men	58	0.000764, 0.000883, 0.00102, 0.00118, 0.00136
	Hispanic	Men	59	0.000842, 0.00097, 0.00112, 0.00128, 0.00148
	Hispanic	Men	60	0.000919, 0.00106, 0.00122, 0.0014, 0.00161
	Hispanic	Men	61	0.000993, 0.00115, 0.00132, 0.00152, 0.00175
	Hispanic	Men	62	0.00107, 0.00123, 0.00142, 0.00164, 0.00189
	Hispanic	Men	63	0.00114, 0.00132, 0.00152, 0.00176, 0.00204
	Hispanic	Men	64	0.00121, 0.0014, 0.00163, 0.00188, 0.00219
	Hispanic	Men	65	0.00128, 0.00149, 0.00173, 0.00202, 0.00235
	Hispanic	Men	66	0.00136, 0.00159, 0.00185, 0.00216, 0.00252
	Hispanic	Men	67	0.00145, 0.0017, 0.00199, 0.00233, 0.00273
	Hispanic	Men	68	0.00154, 0.00182, 0.00214, 0.00253, 0.00298
	Hispanic	Men	69	0.00164, 0.00195, 0.00232, 0.00276, 0.00329
	Hispanic	Men	70	0.00176, 0.00211, 0.00253, 0.00303, 0.00363
	Hispanic	Men	71	0.0019, 0.00229, 0.00276, 0.00333, 0.00403
	Hispanic	Men	72	0.00205, 0.00249, 0.00303, 0.00367, 0.00447
	Hispanic	Men	73	0.00223, 0.00272, 0.00332, 0.00404, 0.00493
	Hispanic	Men	74	0.00244, 0.00299, 0.00364, 0.00444, 0.00542
	Hispanic	Men	75	0.00269, 0.00328, 0.004, 0.00487, 0.00595

Variable	Race/ethnicity	Sex	Age	Distribution
	Hispanic	Men	76	0.00298, 0.00363, 0.00441, 0.00536, 0.00653
	Hispanic	Men	77	0.00331, 0.00402, 0.00488, 0.00591, 0.00718
	Hispanic	Men	78	0.00371, 0.00448, 0.00542, 0.00654, 0.00792
	Hispanic	Men	79	0.00414, 0.005, 0.00602, 0.00726, 0.00877
	Hispanic	Men	80	0.00457, 0.00553, 0.00669, 0.00808, 0.00979
	Hispanic	Men	81	0.00498, 0.00607, 0.0074, 0.00901, 0.011
	Hispanic	Men	82	0.00534, 0.0066, 0.00813, 0.01, 0.0124
	Hispanic	Men	83	0.00568, 0.0071, 0.00886, 0.0111, 0.0138
	Hispanic	Men	84	0.00602, 0.00761, 0.0096, 0.0121, 0.0153
	Hispanic	Women	30	0.0000029, 0.00000415, 0.00000593, 0.00000847, 0.0000121
	Hispanic	Women	31	0.0000035, 0.00000491, 0.00000687, 0.00000959, 0.0000134
	Hispanic	Women	32	0.00000424, 0.00000582, 0.00000796, 0.0000109, 0.000015
	Hispanic	Women	33	0.00000509, 0.00000687, 0.00000924, 0.0000124, 0.0000168
	Hispanic	Women	34	0.00000604, 0.00000805, 0.0000107, 0.0000142, 0.0000189
	Hispanic	Women	35	0.00000703, 0.00000932, 0.0000123, 0.0000163, 0.0000216
	Hispanic	Women	36	0.00000807, 0.0000107, 0.0000141, 0.0000187, 0.0000247
	Hispanic	Women	37	0.00000917, 0.0000122, 0.0000161, 0.0000214, 0.0000283
	Hispanic	Women	38	0.0000104, 0.0000138, 0.0000184, 0.0000244, 0.0000324
	Hispanic	Women	39	0.0000118, 0.0000157, 0.0000209, 0.0000277, 0.0000368
	Hispanic	Women	40	0.0000135, 0.0000179, 0.0000237, 0.0000312, 0.0000413
	Hispanic	Women	41	0.0000157, 0.0000206, 0.0000268, 0.000035, 0.0000458
	Hispanic	Women	42	0.0000185, 0.0000238, 0.0000305, 0.0000391, 0.0000502
	Hispanic	Women	43	0.0000219, 0.0000276, 0.0000347, 0.0000437, 0.000055
	Hispanic	Women	44	0.0000259, 0.0000321, 0.0000397, 0.0000491, 0.0000608
	Hispanic	Women	45	0.0000308, 0.0000376, 0.0000457, 0.0000555, 0.0000677
	Hispanic	Women	46	0.0000369, 0.0000442, 0.0000528, 0.000063, 0.0000754
	Hispanic	Women	47	0.0000443, 0.0000521, 0.0000611, 0.0000718, 0.0000844
	Hispanic	Women	48	0.0000527, 0.0000612, 0.000071, 0.0000824, 0.0000957
	Hispanic	Women	49	0.0000615, 0.0000713, 0.0000827, 0.0000958, 0.000111
	Hispanic	Women	50	0.0000702, 0.0000823, 0.0000964, 0.000113, 0.000132
	Hispanic	Women	51	0.0000793, 0.0000945, 0.000113, 0.000134, 0.00016
	Hispanic	Women	52	0.0000896, 0.000109, 0.000131, 0.000159, 0.000192
	Hispanic	Women	53	0.000101, 0.000125, 0.000153, 0.000187, 0.00023
	Hispanic	Women	54	0.000115, 0.000143, 0.000177, 0.00022, 0.000273
	Hispanic	Women	55	0.000132, 0.000165, 0.000206, 0.000256, 0.00032
	Hispanic	Women	56	0.000151, 0.00019, 0.000238, 0.000297, 0.000373
	Hispanic	Women	57	0.000172, 0.000217, 0.000273, 0.000344, 0.000434
	Hispanic	Women	58	0.000195, 0.000248, 0.000314, 0.000397, 0.000505
	Hispanic	Women	59	0.000219, 0.000281, 0.000358, 0.000458, 0.000586
	Hispanic	Women	60	0.000245, 0.000316, 0.000407, 0.000525, 0.000678
	Hispanic	Women	61	0.000271, 0.000354, 0.00046, 0.000598, 0.000779
	Hispanic	Women	62	0.0003, 0.000393, 0.000515, 0.000674, 0.000884
	Hispanic	Women	63	0.000331, 0.000435, 0.000572, 0.000751, 0.000988

Variable	Race/ethnicity	Sex	Age	Distribution
	Hispanic	Women	64	0.000366, 0.000481, 0.000631, 0.000828, 0.00109
	Hispanic	Women	65	0.000406, 0.000532, 0.000695, 0.000908, 0.00119
	Hispanic	Women	66	0.000452, 0.000589, 0.000765, 0.000995, 0.0013
	Hispanic	Women	67	0.000504, 0.000654, 0.000846, 0.0011, 0.00142
	Hispanic	Women	68	0.000562, 0.000728, 0.000941, 0.00122, 0.00157
	Hispanic	Women	69	0.000629, 0.000813, 0.00105, 0.00136, 0.00175
	Hispanic	Women	70	0.000705, 0.000911, 0.00118, 0.00152, 0.00196
	Hispanic	Women	71	0.000792, 0.00102, 0.00132, 0.0017, 0.00219
	Hispanic	Women	72	0.000891, 0.00115, 0.00148, 0.00191, 0.00246
	Hispanic	Women	73	0.001, 0.00129, 0.00167, 0.00215, 0.00278
	Hispanic	Women	74	0.00113, 0.00146, 0.00188, 0.00243, 0.00315
	Hispanic	Women	75	0.00126, 0.00164, 0.00213, 0.00276, 0.00359
	Hispanic	Women	76	0.00142, 0.00185, 0.00242, 0.00315, 0.00411
	Hispanic	Women	77	0.00159, 0.0021, 0.00275, 0.0036, 0.00473
	Hispanic	Women	78	0.00179, 0.00237, 0.00313, 0.00413, 0.00546
	Hispanic	Women	79	0.00202, 0.00269, 0.00357, 0.00475, 0.00632
	Hispanic	Women	80	0.00228, 0.00305, 0.00408, 0.00546, 0.00733
	Hispanic	Women	81	0.00255, 0.00345, 0.00466, 0.00628, 0.0085
	Hispanic	Women	82	0.00284, 0.00389, 0.0053, 0.00722, 0.00987
	Hispanic	Women	83	0.00313, 0.00433, 0.00598, 0.00826, 0.0114
	Hispanic	Women	84	0.00341, 0.00479, 0.0067, 0.00936, 0.0131
	White	Men	30	0.0000192, 0.0000258, 0.0000345, 0.0000462, 0.000062
	White	Men	31	0.0000246, 0.0000323, 0.0000423, 0.0000555, 0.0000729
	White	Men	32	0.000031, 0.00004, 0.0000516, 0.0000665, 0.0000859
	White	Men	33	0.0000383, 0.0000489, 0.0000623, 0.0000794, 0.000101
	White	Men	34	0.0000464, 0.0000589, 0.0000745, 0.0000944, 0.00012
	White	Men	35	0.0000551, 0.0000698, 0.0000883, 0.000112, 0.000142
	White	Men	36	0.0000646, 0.0000819, 0.000104, 0.000131, 0.000166
	White	Men	37	0.0000756, 0.0000957, 0.000121, 0.000152, 0.000193
	White	Men	38	0.0000893, 0.000112, 0.00014, 0.000175, 0.00022
	White	Men	39	0.000106, 0.000131, 0.000162, 0.0002, 0.000247
	White	Men	40	0.000126, 0.000153, 0.000186, 0.000225, 0.000274
	White	Men	41	0.000149, 0.000178, 0.000212, 0.000253, 0.000302
	White	Men	42	0.000174, 0.000204, 0.00024, 0.000282, 0.000332
	White	Men	43	0.0002, 0.000233, 0.000271, 0.000315, 0.000367
	White	Men	44	0.00023, 0.000265, 0.000305, 0.000352, 0.000406
	White	Men	45	0.000265, 0.000302, 0.000344, 0.000392, 0.000447
	White	Men	46	0.000306, 0.000345, 0.000389, 0.000438, 0.000493
	White	Men	47	0.000353, 0.000394, 0.00044, 0.000491, 0.000548
	White	Men	48	0.000405, 0.00045, 0.000499, 0.000554, 0.000615
	White	Men	49	0.000463, 0.000513, 0.000567, 0.000627, 0.000694
	White	Men	50	0.000531, 0.000584, 0.000643, 0.000708, 0.000779
	White	Men	51	0.000609, 0.000666, 0.000727, 0.000794, 0.000868

Variable	Race/ethnicity	Sex	Age	Distribution
	White	Men	52	0.000693, 0.000753, 0.000817, 0.000888, 0.000965
	White	Men	53	0.000771, 0.00084, 0.000914, 0.000994, 0.00108
	White	Men	54	0.000842, 0.000924, 0.00101, 0.00111, 0.00122
	White	Men	55	0.000912, 0.00101, 0.00112, 0.00123, 0.00137
	White	Men	56	0.000983, 0.0011, 0.00122, 0.00136, 0.00151
	White	Men	57	0.00105, 0.00118, 0.00133, 0.00149, 0.00167
	White	Men	58	0.00113, 0.00127, 0.00143, 0.00161, 0.00182
	White	Men	59	0.00121, 0.00137, 0.00155, 0.00175, 0.00198
	White	Men	60	0.0013, 0.00147, 0.00166, 0.00188, 0.00213
	White	Men	61	0.00139, 0.00157, 0.00178, 0.00202, 0.00229
	White	Men	62	0.00148, 0.00168, 0.0019, 0.00216, 0.00245
	White	Men	63	0.00156, 0.00178, 0.00202, 0.0023, 0.00262
	White	Men	64	0.00164, 0.00187, 0.00214, 0.00244, 0.00279
	White	Men	65	0.00171, 0.00196, 0.00225, 0.00259, 0.00297
	White	Men	66	0.00178, 0.00206, 0.00238, 0.00275, 0.00319
	White	Men	67	0.00185, 0.00216, 0.00252, 0.00295, 0.00345
	White	Men	68	0.00193, 0.00228, 0.0027, 0.00318, 0.00376
	White	Men	69	0.00205, 0.00244, 0.0029, 0.00345, 0.00412
	White	Men	70	0.0022, 0.00263, 0.00315, 0.00376, 0.0045
	White	Men	71	0.00239, 0.00286, 0.00342, 0.0041, 0.00491
	White	Men	72	0.0026, 0.00312, 0.00373, 0.00447, 0.00536
	White	Men	73	0.00284, 0.0034, 0.00408, 0.00488, 0.00586
	White	Men	74	0.00309, 0.00371, 0.00445, 0.00533, 0.0064
	White	Men	75	0.00338, 0.00406, 0.00487, 0.00584, 0.00703
	White	Men	76	0.00368, 0.00444, 0.00534, 0.00643, 0.00775
	White	Men	77	0.00404, 0.00488, 0.00589, 0.00711, 0.0086
	White	Men	78	0.00445, 0.0054, 0.00654, 0.00792, 0.00961
	White	Men	79	0.00494, 0.00601, 0.0073, 0.00886, 0.0108
	White	Men	80	0.00553, 0.00674, 0.00819, 0.00995, 0.0121
	White	Men	81	0.00622, 0.00757, 0.0092, 0.0112, 0.0136
	White	Men	82	0.007, 0.00851, 0.0103, 0.0125, 0.0152
	White	Men	83	0.00782, 0.0095, 0.0115, 0.0139, 0.0169
	White	Men	84	0.00867, 0.0105, 0.0127, 0.0154, 0.0186
	White	Women	30	0.00000499, 0.00000748, 0.0000112, 0.0000167, 0.0000251
	White	Women	31	0.00000651, 0.00000945, 0.0000137, 0.0000197, 0.0000286
	White	Women	32	0.00000853, 0.0000119, 0.0000166, 0.0000231, 0.0000323
	White	Women	33	0.0000111, 0.0000149, 0.0000201, 0.000027, 0.0000364
	White	Women	34	0.0000141, 0.0000185, 0.0000241, 0.0000314, 0.0000411
	White	Women	35	0.0000175, 0.0000224, 0.0000286, 0.0000366, 0.0000469
	White	Women	36	0.0000211, 0.0000267, 0.0000337, 0.0000425, 0.0000538
	White	Women	37	0.0000249, 0.0000313, 0.0000393, 0.0000493, 0.0000621
	White	Women	38	0.0000285, 0.000036, 0.0000454, 0.0000571, 0.0000721
	White	Women	39	0.000032, 0.0000408, 0.0000518, 0.0000658, 0.0000838

Variable	Race/ethnicity	Sex	Age	Distribution
	White	Women	40	0.0000356, 0.0000457, 0.0000586, 0.0000752, 0.0000967
	White	Women	41	0.0000393, 0.0000509, 0.0000657, 0.0000849, 0.00011
	White	Women	42	0.0000437, 0.0000566, 0.0000733, 0.0000948, 0.000123
	White	Women	43	0.0000485, 0.0000629, 0.0000815, 0.000105, 0.000137
	White	Women	44	0.0000537, 0.0000698, 0.0000905, 0.000117, 0.000153
	White	Women	45	0.00006, 0.0000778, 0.000101, 0.000131, 0.00017
	White	Women	46	0.0000683, 0.0000878, 0.000113, 0.000144, 0.000186
	White	Women	47	0.0000797, 0.0001, 0.000126, 0.000158, 0.000199
	White	Women	48	0.0000946, 0.000116, 0.000142, 0.000173, 0.000212
	White	Women	49	0.000112, 0.000134, 0.00016, 0.000191, 0.000228
	White	Women	50	0.000131, 0.000154, 0.000181, 0.000212, 0.00025
	White	Women	51	0.000151, 0.000176, 0.000205, 0.000239, 0.000279
	White	Women	52	0.00017, 0.000199, 0.000232, 0.00027, 0.000316
	White	Women	53	0.000191, 0.000224, 0.000262, 0.000306, 0.000358
	White	Women	54	0.000213, 0.00025, 0.000294, 0.000344, 0.000404
	White	Women	55	0.000237, 0.000279, 0.000328, 0.000386, 0.000455
	White	Women	56	0.000264, 0.000311, 0.000367, 0.000432, 0.000509
	White	Women	57	0.000294, 0.000347, 0.000409, 0.000482, 0.000568
	White	Women	58	0.000329, 0.000388, 0.000456, 0.000537, 0.000632
	White	Women	59	0.000368, 0.000433, 0.000509, 0.000598, 0.000704
	White	Women	60	0.000407, 0.000481, 0.000566, 0.000667, 0.000787
	White	Women	61	0.000446, 0.000529, 0.000627, 0.000743, 0.000881
	White	Women	62	0.000484, 0.000578, 0.000689, 0.000821, 0.000981
	White	Women	63	0.000522, 0.000627, 0.000752, 0.000901, 0.00108
	White	Women	64	0.000562, 0.000678, 0.000817, 0.000984, 0.00119
	White	Women	65	0.000605, 0.000733, 0.000887, 0.00107, 0.0013
	White	Women	66	0.000656, 0.000797, 0.000966, 0.00117, 0.00142
	White	Women	67	0.000716, 0.000871, 0.00106, 0.00128, 0.00156
	White	Women	68	0.000788, 0.000959, 0.00116, 0.00141, 0.00172
	White	Women	69	0.000872, 0.00106, 0.00129, 0.00156, 0.0019
	White	Women	70	0.000968, 0.00118, 0.00143, 0.00174, 0.00211
	White	Women	71	0.00108, 0.00131, 0.00159, 0.00194, 0.00236
	White	Women	72	0.00119, 0.00146, 0.00178, 0.00217, 0.00265
	White	Women	73	0.00132, 0.00162, 0.00199, 0.00243, 0.00299
	White	Women	74	0.00146, 0.00181, 0.00222, 0.00274, 0.00337
	White	Women	75	0.00163, 0.00201, 0.00249, 0.00308, 0.00382
	White	Women	76	0.00181, 0.00225, 0.0028, 0.00348, 0.00433
	White	Women	77	0.00202, 0.00253, 0.00316, 0.00394, 0.00493
	White	Women	78	0.00227, 0.00285, 0.00358, 0.00449, 0.00564
	White	Women	79	0.00256, 0.00323, 0.00407, 0.00513, 0.00648
	White	Women	80	0.0029, 0.00368, 0.00466, 0.00589, 0.00748
	White	Women	81	0.00329, 0.00419, 0.00533, 0.00678, 0.00864
	White	Women	82	0.00373, 0.00477, 0.0061, 0.00779, 0.00997

Variable	Race/ethnicity	Sex	Age	Distribution
	White	Women	83	0.0042, 0.0054, 0.00693, 0.00889, 0.0114
	White	Women	84	0.0047, 0.00607, 0.00781, 0.0101, 0.013
CHD mortality rates for 2024 (0.01, 0.2, 0.5, 0.8. 0.99 percentiles of the empirical distribution produced during forecasting)				
	Black	Men	30	0.000031, 0.0000435, 0.0000607, 0.0000848, 0.000119
	Black	Men	31	0.0000393, 0.0000537, 0.000073, 0.0000993, 0.000135
	Black	Men	32	0.0000495, 0.0000658, 0.0000872, 0.000115, 0.000153
	Black	Men	33	0.0000616, 0.0000799, 0.000103, 0.000133, 0.000173
	Black	Men	34	0.0000751, 0.0000954, 0.000121, 0.000153, 0.000195
	Black	Men	35	0.0000889, 0.000112, 0.00014, 0.000176, 0.000222
	Black	Men	36	0.000103, 0.000129, 0.000161, 0.000202, 0.000253
	Black	Men	37	0.000117, 0.000146, 0.000183, 0.000229, 0.000287
	Black	Men	38	0.000132, 0.000165, 0.000206, 0.000258, 0.000323
	Black	Men	39	0.000148, 0.000185, 0.000231, 0.000289, 0.000362
	Black	Men	40	0.000165, 0.000206, 0.000257, 0.000321, 0.000402
	Black	Men	41	0.000184, 0.00023, 0.000286, 0.000356, 0.000444
	Black	Men	42	0.000205, 0.000255, 0.000317, 0.000394, 0.000491
	Black	Men	43	0.000226, 0.000282, 0.000352, 0.000439, 0.000549
	Black	Men	44	0.000248, 0.000312, 0.000392, 0.000493, 0.00062
	Black	Men	45	0.000274, 0.000347, 0.000439, 0.000555, 0.000704
	Black	Men	46	0.000306, 0.000389, 0.000495, 0.000628, 0.0008
	Black	Men	47	0.000344, 0.00044, 0.000561, 0.000715, 0.000913
	Black	Men	48	0.000391, 0.000501, 0.00064, 0.000817, 0.00105
	Black	Men	49	0.000448, 0.000574, 0.000734, 0.000938, 0.0012
	Black	Men	50	0.000519, 0.000663, 0.000844, 0.00108, 0.00137
	Black	Men	51	0.000608, 0.000769, 0.000972, 0.00123, 0.00155
	Black	Men	52	0.000714, 0.000893, 0.00111, 0.00139, 0.00174
	Black	Men	53	0.00083, 0.00103, 0.00127, 0.00157, 0.00194
	Black	Men	54	0.000947, 0.00117, 0.00143, 0.00176, 0.00217
	Black	Men	55	0.00107, 0.00131, 0.0016, 0.00196, 0.00241
	Black	Men	56	0.00119, 0.00145, 0.00177, 0.00216, 0.00264
	Black	Men	57	0.00133, 0.00161, 0.00194, 0.00235, 0.00285
	Black	Men	58	0.00147, 0.00177, 0.00212, 0.00254, 0.00306
	Black	Men	59	0.00161, 0.00192, 0.00229, 0.00273, 0.00326
	Black	Men	60	0.00176, 0.00208, 0.00247, 0.00292, 0.00347
	Black	Men	61	0.00191, 0.00224, 0.00264, 0.0031, 0.00365
	Black	Men	62	0.00206, 0.0024, 0.0028, 0.00326, 0.00381
	Black	Men	63	0.0022, 0.00255, 0.00295, 0.00341, 0.00395
	Black	Men	64	0.00234, 0.0027, 0.0031, 0.00356, 0.00409
	Black	Men	65	0.00248, 0.00284, 0.00325, 0.00372, 0.00427
	Black	Men	66	0.0026, 0.00299, 0.00342, 0.00392, 0.00449
	Black	Men	67	0.00273, 0.00314, 0.00362, 0.00416, 0.00479
	Black	Men	68	0.00287, 0.00332, 0.00384, 0.00444, 0.00514
	Black	Men	69	0.00304, 0.00353, 0.0041, 0.00476, 0.00553

Variable	Race/ethnicity	Sex	Age	Distribution
	Black	Men	70	0.00323, 0.00377, 0.00438, 0.0051, 0.00595
	Black	Men	71	0.00345, 0.00402, 0.00469, 0.00546, 0.00638
	Black	Men	72	0.00368, 0.0043, 0.00502, 0.00585, 0.00684
	Black	Men	73	0.00392, 0.00459, 0.00537, 0.00627, 0.00734
	Black	Men	74	0.00419, 0.00491, 0.00574, 0.00672, 0.00787
	Black	Men	75	0.0045, 0.00527, 0.00616, 0.0072, 0.00843
	Black	Men	76	0.00486, 0.00568, 0.00663, 0.00773, 0.00903
	Black	Men	77	0.00527, 0.00614, 0.00715, 0.00833, 0.00971
	Black	Men	78	0.0057, 0.00665, 0.00774, 0.00901, 0.0105
	Black	Men	79	0.00615, 0.00719, 0.00839, 0.0098, 0.0114
	Black	Men	80	0.00661, 0.00776, 0.0091, 0.0107, 0.0125
	Black	Men	81	0.00706, 0.00835, 0.00985, 0.0116, 0.0137
	Black	Men	82	0.00747, 0.00892, 0.0106, 0.0126, 0.0151
	Black	Men	83	0.00784, 0.00946, 0.0114, 0.0137, 0.0166
	Black	Men	84	0.00817, 0.00998, 0.0122, 0.0148, 0.0181
	Black	Women	30	0.0000129, 0.0000182, 0.0000255, 0.0000359, 0.0000506
	Black	Women	31	0.0000163, 0.0000222, 0.0000301, 0.0000409, 0.0000556
	Black	Women	32	0.0000202, 0.0000269, 0.0000355, 0.000047, 0.0000624
	Black	Women	33	0.0000247, 0.0000322, 0.0000418, 0.0000543, 0.0000707
	Black	Women	34	0.0000295, 0.000038, 0.0000489, 0.0000629, 0.000081
	Black	Women	35	0.0000342, 0.0000441, 0.0000567, 0.000073, 0.0000941
	Black	Women	36	0.0000389, 0.0000504, 0.0000652, 0.0000843, 0.000109
	Black	Women	37	0.000044, 0.0000573, 0.0000743, 0.0000965, 0.000126
	Black	Women	38	0.0000493, 0.0000645, 0.0000841, 0.00011, 0.000143
	Black	Women	39	0.000055, 0.0000723, 0.0000947, 0.000124, 0.000163
	Black	Women	40	0.0000616, 0.0000809, 0.000106, 0.000139, 0.000183
	Black	Women	41	0.0000698, 0.000091, 0.000118, 0.000154, 0.000201
	Black	Women	42	0.0000803, 0.000103, 0.000132, 0.000169, 0.000217
	Black	Women	43	0.0000929, 0.000117, 0.000147, 0.000185, 0.000234
	Black	Women	44	0.000107, 0.000133, 0.000165, 0.000206, 0.000257
	Black	Women	45	0.000122, 0.000151, 0.000187, 0.000231, 0.000287
	Black	Women	46	0.00014, 0.000172, 0.000212, 0.000261, 0.000323
	Black	Women	47	0.000162, 0.000198, 0.000243, 0.000297, 0.000364
	Black	Women	48	0.000187, 0.000229, 0.000279, 0.00034, 0.000415
	Black	Women	49	0.000212, 0.000261, 0.000321, 0.000395, 0.000486
	Black	Women	50	0.00024, 0.000298, 0.000369, 0.000457, 0.000566
	Black	Women	51	0.000279, 0.000343, 0.000421, 0.000518, 0.000638
	Black	Women	52	0.000326, 0.000395, 0.000478, 0.000579, 0.000702
	Black	Women	53	0.000371, 0.000448, 0.000539, 0.00065, 0.000784
	Black	Women	54	0.000411, 0.0005, 0.000605, 0.000733, 0.00089
	Black	Women	55	0.000459, 0.000558, 0.000677, 0.000822, 0.000999
	Black	Women	56	0.000518, 0.000625, 0.000754, 0.00091, 0.0011
	Black	Women	57	0.000582, 0.000699, 0.000836, 0.001, 0.0012

Variable	Race/ethnicity	Sex	Age	Distribution
	Black	Women	58	0.000644, 0.000771, 0.000922, 0.0011, 0.00132
	Black	Women	59	0.0007, 0.000842, 0.00101, 0.00121, 0.00146
	Black	Women	60	0.000753, 0.00091, 0.0011, 0.00132, 0.0016
	Black	Women	61	0.000802, 0.000976, 0.00119, 0.00144, 0.00175
	Black	Women	62	0.000844, 0.00104, 0.00127, 0.00156, 0.00191
	Black	Women	63	0.00088, 0.00109, 0.00135, 0.00168, 0.00208
	Black	Women	64	0.000912, 0.00115, 0.00144, 0.0018, 0.00227
	Black	Women	65	0.000945, 0.0012, 0.00153, 0.00194, 0.00247
	Black	Women	66	0.000987, 0.00127, 0.00163, 0.00209, 0.00269
	Black	Women	67	0.00104, 0.00135, 0.00175, 0.00226, 0.00293
	Black	Women	68	0.00112, 0.00146, 0.00189, 0.00245, 0.00319
	Black	Women	69	0.00123, 0.00159, 0.00206, 0.00267, 0.00347
	Black	Women	70	0.00135, 0.00175, 0.00226, 0.00291, 0.00376
	Black	Women	71	0.00149, 0.00192, 0.00247, 0.00317, 0.00408
	Black	Women	72	0.00164, 0.00211, 0.0027, 0.00345, 0.00442
	Black	Women	73	0.0018, 0.0023, 0.00294, 0.00375, 0.0048
	Black	Women	74	0.00196, 0.00251, 0.00321, 0.00409, 0.00523
	Black	Women	75	0.00214, 0.00274, 0.0035, 0.00447, 0.00573
	Black	Women	76	0.00232, 0.00298, 0.00383, 0.00491, 0.00632
	Black	Women	77	0.0025, 0.00325, 0.0042, 0.00544, 0.00705
	Black	Women	78	0.00269, 0.00353, 0.00462, 0.00605, 0.00793
	Black	Women	79	0.00288, 0.00383, 0.00508, 0.00675, 0.00897
	Black	Women	80	0.00308, 0.00416, 0.0056, 0.00753, 0.0102
	Black	Women	81	0.00331, 0.00452, 0.00616, 0.00839, 0.0115
	Black	Women	82	0.00357, 0.00492, 0.00676, 0.00929, 0.0128
	Black	Women	83	0.00386, 0.00535, 0.00739, 0.0102, 0.0141
	Black	Women	84	0.00418, 0.0058, 0.00804, 0.0111, 0.0155
	Hispanic	Men	30	0.0000106, 0.0000148, 0.0000207, 0.0000288, 0.0000403
	Hispanic	Men	31	0.0000131, 0.0000179, 0.0000245, 0.0000336, 0.0000461
	Hispanic	Men	32	0.000016, 0.0000216, 0.0000291, 0.0000391, 0.0000527
	Hispanic	Men	33	0.0000198, 0.0000261, 0.0000344, 0.0000452, 0.0000597
	Hispanic	Men	34	0.0000244, 0.0000315, 0.0000404, 0.000052, 0.000067
	Hispanic	Men	35	0.0000298, 0.0000376, 0.0000473, 0.0000595, 0.0000751
	Hispanic	Men	36	0.0000357, 0.0000444, 0.000055, 0.0000683, 0.0000848
	Hispanic	Men	37	0.0000419, 0.0000517, 0.0000637, 0.0000784, 0.0000966
	Hispanic	Men	38	0.0000487, 0.0000598, 0.0000733, 0.0000899, 0.00011
	Hispanic	Men	39	0.0000564, 0.000069, 0.0000842, 0.000103, 0.000126
	Hispanic	Men	40	0.0000657, 0.0000797, 0.0000965, 0.000117, 0.000142
	Hispanic	Men	41	0.0000767, 0.000092, 0.00011, 0.000132, 0.000158
	Hispanic	Men	42	0.0000894, 0.000106, 0.000126, 0.000149, 0.000177
	Hispanic	Men	43	0.000104, 0.000122, 0.000144, 0.000168, 0.000198
	Hispanic	Men	44	0.000121, 0.000141, 0.000164, 0.000191, 0.000222
	Hispanic	Men	45	0.000139, 0.000162, 0.000187, 0.000217, 0.000252

Variable	Race/ethnicity	Sex	Age	Distribution
	Hispanic	Men	46	0.000159, 0.000185, 0.000215, 0.000249, 0.00029
	Hispanic	Men	47	0.000183, 0.000213, 0.000247, 0.000287, 0.000333
	Hispanic	Men	48	0.000213, 0.000247, 0.000285, 0.000328, 0.000379
	Hispanic	Men	49	0.00025, 0.000287, 0.000329, 0.000377, 0.000432
	Hispanic	Men	50	0.00029, 0.000332, 0.00038, 0.000435, 0.000499
	Hispanic	Men	51	0.000333, 0.000383, 0.00044, 0.000505, 0.000581
	Hispanic	Men	52	0.00038, 0.000439, 0.000506, 0.000584, 0.000675
	Hispanic	Men	53	0.000428, 0.000498, 0.000579, 0.000674, 0.000785
	Hispanic	Men	54	0.000478, 0.000561, 0.000657, 0.00077, 0.000905
	Hispanic	Men	55	0.000535, 0.000629, 0.000739, 0.000868, 0.00102
	Hispanic	Men	56	0.000601, 0.000704, 0.000825, 0.000966, 0.00113
	Hispanic	Men	57	0.000673, 0.000785, 0.000913, 0.00106, 0.00124
	Hispanic	Men	58	0.000749, 0.000869, 0.00101, 0.00116, 0.00135
	Hispanic	Men	59	0.000825, 0.000954, 0.0011, 0.00127, 0.00147
	Hispanic	Men	60	0.000899, 0.00104, 0.0012, 0.00138, 0.0016
	Hispanic	Men	61	0.000971, 0.00112, 0.0013, 0.0015, 0.00174
	Hispanic	Men	62	0.00104, 0.00121, 0.0014, 0.00162, 0.00188
	Hispanic	Men	63	0.00111, 0.00129, 0.0015, 0.00174, 0.00202
	Hispanic	Men	64	0.00118, 0.00137, 0.0016, 0.00185, 0.00216
	Hispanic	Men	65	0.00125, 0.00146, 0.0017, 0.00198, 0.00231
	Hispanic	Men	66	0.00133, 0.00155, 0.00181, 0.00212, 0.00248
	Hispanic	Men	67	0.00141, 0.00166, 0.00194, 0.00228, 0.00269
	Hispanic	Men	68	0.0015, 0.00177, 0.0021, 0.00248, 0.00294
	Hispanic	Men	69	0.0016, 0.00191, 0.00227, 0.00271, 0.00323
	Hispanic	Men	70	0.00171, 0.00206, 0.00247, 0.00297, 0.00358
	Hispanic	Men	71	0.00184, 0.00223, 0.0027, 0.00327, 0.00396
	Hispanic	Men	72	0.002, 0.00243, 0.00296, 0.0036, 0.00439
	Hispanic	Men	73	0.00217, 0.00266, 0.00324, 0.00396, 0.00485
	Hispanic	Men	74	0.00238, 0.00291, 0.00356, 0.00435, 0.00533
	Hispanic	Men	75	0.00261, 0.0032, 0.00391, 0.00477, 0.00584
	Hispanic	Men	76	0.00289, 0.00353, 0.00431, 0.00525, 0.00641
	Hispanic	Men	77	0.00322, 0.00392, 0.00477, 0.00579, 0.00705
	Hispanic	Men	78	0.0036, 0.00437, 0.00529, 0.00641, 0.00778
	Hispanic	Men	79	0.00402, 0.00487, 0.00589, 0.00712, 0.00862
	Hispanic	Men	80	0.00444, 0.00539, 0.00654, 0.00793, 0.00963
	Hispanic	Men	81	0.00483, 0.00592, 0.00723, 0.00884, 0.0108
	Hispanic	Men	82	0.00518, 0.00642, 0.00794, 0.00982, 0.0122
	Hispanic	Men	83	0.00551, 0.00692, 0.00866, 0.0109, 0.0136
	Hispanic	Men	84	0.00584, 0.00741, 0.00938, 0.0119, 0.0151
	Hispanic	Women	30	0.00000284, 0.0000041, 0.0000059, 0.00000849, 0.0000123
	Hispanic	Women	31	0.00000344, 0.00000486, 0.00000683, 0.00000961, 0.0000136
	Hispanic	Women	32	0.00000416, 0.00000576, 0.00000793, 0.0000109, 0.0000151
	Hispanic	Women	33	0.00000501, 0.0000068, 0.0000092, 0.0000124, 0.0000169

Variable	Race/ethnicity	Sex	Age	Distribution
	Hispanic	Women	34	0.00000594, 0.00000797, 0.0000106, 0.0000142, 0.0000191
	Hispanic	Women	35	0.00000692, 0.00000922, 0.0000123, 0.0000163, 0.0000217
	Hispanic	Women	36	0.00000793, 0.0000106, 0.000014, 0.0000187, 0.0000249
	Hispanic	Women	37	0.000009, 0.000012, 0.000016, 0.0000213, 0.0000285
	Hispanic	Women	38	0.0000102, 0.0000136, 0.0000182, 0.0000243, 0.0000325
	Hispanic	Women	39	0.0000115, 0.0000154, 0.0000206, 0.0000275, 0.0000368
	Hispanic	Women	40	0.0000132, 0.0000176, 0.0000233, 0.0000309, 0.0000412
	Hispanic	Women	41	0.0000153, 0.0000201, 0.0000264, 0.0000346, 0.0000454
	Hispanic	Women	42	0.000018, 0.0000232, 0.0000299, 0.0000385, 0.0000496
	Hispanic	Women	43	0.0000213, 0.000027, 0.000034, 0.0000429, 0.0000542
	Hispanic	Women	44	0.0000252, 0.0000313, 0.0000389, 0.0000482, 0.0000598
	Hispanic	Women	45	0.00003, 0.0000366, 0.0000446, 0.0000544, 0.0000665
	Hispanic	Women	46	0.0000359, 0.000043, 0.0000515, 0.0000617, 0.000074
	Hispanic	Women	47	0.0000431, 0.0000507, 0.0000597, 0.0000703, 0.0000828
	Hispanic	Women	48	0.0000513, 0.0000597, 0.0000694, 0.0000807, 0.0000939
	Hispanic	Women	49	0.0000599, 0.0000696, 0.0000808, 0.0000939, 0.000109
	Hispanic	Women	50	0.0000685, 0.0000805, 0.0000944, 0.000111, 0.00013
	Hispanic	Women	51	0.0000775, 0.0000926, 0.00011, 0.000131, 0.000157
	Hispanic	Women	52	0.0000877, 0.000106, 0.000129, 0.000156, 0.000189
	Hispanic	Women	53	0.0000995, 0.000122, 0.00015, 0.000184, 0.000227
	Hispanic	Women	54	0.000113, 0.000141, 0.000175, 0.000217, 0.000269
	Hispanic	Women	55	0.00013, 0.000162, 0.000203, 0.000253, 0.000317
	Hispanic	Women	56	0.000148, 0.000187, 0.000234, 0.000294, 0.000369
	Hispanic	Women	57	0.000169, 0.000214, 0.00027, 0.00034, 0.00043
	Hispanic	Women	58	0.000192, 0.000244, 0.000309, 0.000393, 0.0005
	Hispanic	Women	59	0.000215, 0.000276, 0.000354, 0.000452, 0.00058
	Hispanic	Women	60	0.00024, 0.000311, 0.000402, 0.000518, 0.000671
	Hispanic	Women	61	0.000266, 0.000348, 0.000453, 0.00059, 0.00077
	Hispanic	Women	62	0.000294, 0.000386, 0.000507, 0.000664, 0.000873
	Hispanic	Women	63	0.000324, 0.000427, 0.000562, 0.000739, 0.000974
	Hispanic	Women	64	0.000358, 0.000471, 0.000619, 0.000814, 0.00107
	Hispanic	Women	65	0.000397, 0.00052, 0.000681, 0.000891, 0.00117
	Hispanic	Women	66	0.000441, 0.000576, 0.00075, 0.000976, 0.00127
	Hispanic	Women	67	0.000491, 0.000639, 0.000828, 0.00107, 0.0014
	Hispanic	Women	68	0.000548, 0.000711, 0.00092, 0.00119, 0.00155
	Hispanic	Women	69	0.000612, 0.000794, 0.00103, 0.00133, 0.00172
	Hispanic	Women	70	0.000687, 0.00089, 0.00115, 0.00149, 0.00192
	Hispanic	Women	71	0.000772, 0.000999, 0.00129, 0.00166, 0.00215
	Hispanic	Women	72	0.000867, 0.00112, 0.00145, 0.00187, 0.00242
	Hispanic	Women	73	0.000975, 0.00126, 0.00163, 0.0021, 0.00273
	Hispanic	Women	74	0.00109, 0.00142, 0.00184, 0.00238, 0.00309
	Hispanic	Women	75	0.00123, 0.0016, 0.00208, 0.0027, 0.00352
	Hispanic	Women	76	0.00138, 0.00181, 0.00236, 0.00308, 0.00403

Variable	Race/ethnicity	Sex	Age	Distribution
	Hispanic	Women	77	0.00155, 0.00204, 0.00268, 0.00352, 0.00464
	Hispanic	Women	78	0.00175, 0.00231, 0.00306, 0.00404, 0.00535
	Hispanic	Women	79	0.00197, 0.00262, 0.00349, 0.00464, 0.00619
	Hispanic	Women	80	0.00221, 0.00298, 0.00399, 0.00534, 0.00718
	Hispanic	Women	81	0.00248, 0.00337, 0.00455, 0.00615, 0.00833
	Hispanic	Women	82	0.00276, 0.00379, 0.00517, 0.00706, 0.00968
	Hispanic	Women	83	0.00304, 0.00422, 0.00584, 0.00808, 0.0112
	Hispanic	Women	84	0.00332, 0.00467, 0.00654, 0.00916, 0.0129
	White	Men	30	0.0000188, 0.0000255, 0.0000344, 0.0000465, 0.000063
	White	Men	31	0.0000241, 0.0000319, 0.0000422, 0.0000558, 0.0000739
	White	Men	32	0.0000304, 0.0000396, 0.0000514, 0.0000668, 0.000087
	White	Men	33	0.0000377, 0.0000484, 0.0000621, 0.0000797, 0.000103
	White	Men	34	0.0000456, 0.0000583, 0.0000744, 0.0000948, 0.000121
	White	Men	35	0.0000541, 0.0000691, 0.000088, 0.000112, 0.000143
	White	Men	36	0.0000634, 0.000081, 0.000103, 0.000131, 0.000168
	White	Men	37	0.0000741, 0.0000945, 0.00012, 0.000153, 0.000195
	White	Men	38	0.0000873, 0.00011, 0.000139, 0.000175, 0.000222
	White	Men	39	0.000103, 0.000129, 0.00016, 0.000199, 0.000248
	White	Men	40	0.000123, 0.00015, 0.000184, 0.000224, 0.000275
	White	Men	41	0.000145, 0.000174, 0.000209, 0.000251, 0.000301
	White	Men	42	0.000169, 0.0002, 0.000236, 0.000279, 0.00033
	White	Men	43	0.000194, 0.000228, 0.000266, 0.00031, 0.000363
	White	Men	44	0.000223, 0.000258, 0.000299, 0.000346, 0.000401
	White	Men	45	0.000257, 0.000294, 0.000337, 0.000385, 0.000441
	White	Men	46	0.000297, 0.000336, 0.00038, 0.000429, 0.000486
	White	Men	47	0.000343, 0.000384, 0.00043, 0.000481, 0.000539
	White	Men	48	0.000394, 0.000439, 0.000488, 0.000543, 0.000605
	White	Men	49	0.00045, 0.0005, 0.000555, 0.000615, 0.000683
	White	Men	50	0.000517, 0.000571, 0.00063, 0.000695, 0.000768
	White	Men	51	0.000594, 0.000651, 0.000713, 0.000781, 0.000856
	White	Men	52	0.000676, 0.000737, 0.000803, 0.000874, 0.000952
	White	Men	53	0.000754, 0.000823, 0.000898, 0.00098, 0.00107
	White	Men	54	0.000824, 0.000907, 0.000997, 0.0011, 0.00121
	White	Men	55	0.000893, 0.000991, 0.0011, 0.00122, 0.00135
	White	Men	56	0.000962, 0.00108, 0.0012, 0.00134, 0.0015
	White	Men	57	0.00103, 0.00116, 0.00131, 0.00147, 0.00165
	White	Men	58	0.0011, 0.00125, 0.00141, 0.0016, 0.00181
	White	Men	59	0.00118, 0.00134, 0.00152, 0.00173, 0.00196
	White	Men	60	0.00127, 0.00144, 0.00164, 0.00186, 0.00211
	White	Men	61	0.00135, 0.00154, 0.00175, 0.00199, 0.00227
	White	Men	62	0.00144, 0.00164, 0.00187, 0.00213, 0.00243
	White	Men	63	0.00152, 0.00174, 0.00198, 0.00226, 0.00259
	White	Men	64	0.00159, 0.00183, 0.00209, 0.0024, 0.00275

Variable	Race/ethnicity	Sex	Age	Distribution
	White	Men	65	0.00166, 0.00192, 0.00221, 0.00254, 0.00293
	White	Men	66	0.00173, 0.00201, 0.00233, 0.0027, 0.00314
	White	Men	67	0.0018, 0.00211, 0.00247, 0.00289, 0.00339
	White	Men	68	0.00188, 0.00223, 0.00264, 0.00312, 0.0037
	White	Men	69	0.00199, 0.00238, 0.00284, 0.00338, 0.00404
	White	Men	70	0.00214, 0.00257, 0.00307, 0.00368, 0.00442
	White	Men	71	0.00232, 0.00279, 0.00334, 0.00401, 0.00482
	White	Men	72	0.00252, 0.00304, 0.00365, 0.00438, 0.00527
	White	Men	73	0.00275, 0.00331, 0.00398, 0.00478, 0.00575
	White	Men	74	0.003, 0.00362, 0.00435, 0.00522, 0.00629
	White	Men	75	0.00328, 0.00395, 0.00475, 0.00572, 0.00689
	White	Men	76	0.00358, 0.00432, 0.00521, 0.00629, 0.0076
	White	Men	77	0.00392, 0.00475, 0.00575, 0.00696, 0.00844
	White	Men	78	0.00431, 0.00525, 0.00638, 0.00775, 0.00944
	White	Men	79	0.00479, 0.00585, 0.00712, 0.00868, 0.0106
	White	Men	80	0.00537, 0.00655, 0.00799, 0.00974, 0.0119
	White	Men	81	0.00603, 0.00737, 0.00898, 0.0109, 0.0134
	White	Men	82	0.00678, 0.00827, 0.0101, 0.0123, 0.015
	White	Men	83	0.00758, 0.00923, 0.0112, 0.0137, 0.0166
	White	Men	84	0.00839, 0.0102, 0.0124, 0.0151, 0.0183
	White	Women	30	0.0000049, 0.0000074, 0.0000111, 0.0000168, 0.0000253
	White	Women	31	0.00000641, 0.00000936, 0.0000136, 0.0000198, 0.0000289
	White	Women	32	0.00000842, 0.0000118, 0.0000166, 0.0000232, 0.0000326
	White	Women	33	0.0000109, 0.0000148, 0.00002, 0.0000271, 0.0000367
	White	Women	34	0.0000139, 0.0000183, 0.000024, 0.0000315, 0.0000415
	White	Women	35	0.0000172, 0.0000222, 0.0000286, 0.0000367, 0.0000473
	White	Women	36	0.0000208, 0.0000264, 0.0000336, 0.0000426, 0.0000543
	White	Women	37	0.0000244, 0.0000309, 0.0000391, 0.0000494, 0.0000626
	White	Women	38	0.0000279, 0.0000355, 0.000045, 0.000057, 0.0000725
	White	Women	39	0.0000313, 0.0000401, 0.0000513, 0.0000655, 0.000084
	White	Women	40	0.0000347, 0.0000448, 0.0000578, 0.0000746, 0.0000965
	White	Women	41	0.0000384, 0.0000499, 0.0000647, 0.0000839, 0.000109
	White	Women	42	0.0000426, 0.0000554, 0.0000719, 0.0000934, 0.000122
	White	Women	43	0.0000472, 0.0000615, 0.0000798, 0.000104, 0.000135
	White	Women	44	0.0000523, 0.0000681, 0.0000885, 0.000115, 0.00015
	White	Women	45	0.0000583, 0.0000759, 0.0000984, 0.000128, 0.000166
	White	Women	46	0.0000664, 0.0000855, 0.00011, 0.000141, 0.000182
	White	Women	47	0.0000775, 0.0000977, 0.000123, 0.000155, 0.000195
	White	Women	48	0.000092, 0.000113, 0.000138, 0.000169, 0.000208
	White	Women	49	0.000109, 0.000131, 0.000156, 0.000187, 0.000223
	White	Women	50	0.000128, 0.000151, 0.000177, 0.000208, 0.000245
	White	Women	51	0.000147, 0.000172, 0.000201, 0.000234, 0.000274
	White	Women	52	0.000167, 0.000195, 0.000227, 0.000265, 0.00031

Variable	Race/ethnicity	Sex	Age	Distribution
	White	Women	53	0.000187, 0.000219, 0.000257, 0.000301, 0.000352
	White	Women	54	0.000209, 0.000246, 0.000289, 0.000339, 0.000399
	White	Women	55	0.000233, 0.000274, 0.000323, 0.000381, 0.000449
	White	Women	56	0.000259, 0.000306, 0.000361, 0.000426, 0.000503
	White	Women	57	0.000288, 0.000341, 0.000403, 0.000475, 0.000562
	White	Women	58	0.000323, 0.000381, 0.000449, 0.00053, 0.000626
	White	Women	59	0.00036, 0.000425, 0.000501, 0.000591, 0.000697
	White	Women	60	0.000399, 0.000472, 0.000558, 0.000659, 0.000779
	White	Women	61	0.000436, 0.000519, 0.000617, 0.000733, 0.000872
	White	Women	62	0.000473, 0.000566, 0.000677, 0.000809, 0.000969
	White	Women	63	0.00051, 0.000614, 0.000738, 0.000887, 0.00107
	White	Women	64	0.000549, 0.000664, 0.000801, 0.000967, 0.00117
	White	Women	65	0.000591, 0.000717, 0.000869, 0.00105, 0.00128
	White	Women	66	0.000639, 0.000778, 0.000945, 0.00115, 0.0014
	White	Women	67	0.000697, 0.00085, 0.00103, 0.00126, 0.00153
	White	Women	68	0.000767, 0.000935, 0.00114, 0.00138, 0.00169
	White	Women	69	0.000849, 0.00103, 0.00126, 0.00153, 0.00187
	White	Women	70	0.000942, 0.00115, 0.0014, 0.0017, 0.00207
	White	Women	71	0.00105, 0.00128, 0.00156, 0.0019, 0.00232
	White	Women	72	0.00116, 0.00142, 0.00174, 0.00212, 0.0026
	White	Women	73	0.00128, 0.00158, 0.00194, 0.00238, 0.00293
	White	Women	74	0.00142, 0.00176, 0.00217, 0.00268, 0.00331
	White	Women	75	0.00158, 0.00196, 0.00243, 0.00301, 0.00374
	White	Women	76	0.00176, 0.0022, 0.00273, 0.0034, 0.00425
	White	Women	77	0.00197, 0.00247, 0.00308, 0.00386, 0.00483
	White	Women	78	0.00221, 0.00278, 0.00349, 0.00439, 0.00553
	White	Women	79	0.00249, 0.00315, 0.00398, 0.00502, 0.00636
	White	Women	80	0.00282, 0.00358, 0.00455, 0.00577, 0.00733
	White	Women	81	0.0032, 0.00408, 0.00521, 0.00664, 0.00848
	White	Women	82	0.00362, 0.00465, 0.00595, 0.00762, 0.00978
	White	Women	83	0.00408, 0.00526, 0.00677, 0.0087, 0.0112
	White	Women	84	0.00456, 0.0059, 0.00762, 0.00985, 0.0127
CHD mortality rates for 2025 (0.01, 0.2, 0.5, 0.8. 0.99 percentiles of the empirical distribution produced during forecasting)				
	Black	Men	30	0.0000302, 0.0000428, 0.0000604, 0.0000851, 0.00012
	Black	Men	31	0.0000385, 0.0000529, 0.0000726, 0.0000996, 0.000137
	Black	Men	32	0.0000485, 0.0000649, 0.0000867, 0.000116, 0.000155
	Black	Men	33	0.0000604, 0.0000789, 0.000103, 0.000134, 0.000174
	Black	Men	34	0.0000736, 0.0000943, 0.00012, 0.000154, 0.000197
	Black	Men	35	0.000087, 0.00011, 0.00014, 0.000177, 0.000224
	Black	Men	36	0.0001, 0.000127, 0.00016, 0.000202, 0.000255
	Black	Men	37	0.000114, 0.000144, 0.000182, 0.000229, 0.000289
	Black	Men	38	0.000128, 0.000162, 0.000204, 0.000257, 0.000325
	Black	Men	39	0.000144, 0.000181, 0.000228, 0.000287, 0.000363

Variable	Race/ethnicity	Sex	Age	Distribution
	Black	Men	40	0.00016, 0.000202, 0.000254, 0.000319, 0.000401
	Black	Men	41	0.000179, 0.000224, 0.000281, 0.000352, 0.000441
	Black	Men	42	0.000199, 0.000249, 0.000311, 0.000388, 0.000486
	Black	Men	43	0.000219, 0.000275, 0.000345, 0.000431, 0.000541
	Black	Men	44	0.000241, 0.000304, 0.000383, 0.000483, 0.00061
	Black	Men	45	0.000266, 0.000338, 0.000429, 0.000544, 0.000691
	Black	Men	46	0.000297, 0.000379, 0.000483, 0.000615, 0.000785
	Black	Men	47	0.000335, 0.000429, 0.000548, 0.000699, 0.000895
	Black	Men	48	0.000381, 0.000489, 0.000625, 0.0008, 0.00103
	Black	Men	49	0.000437, 0.000561, 0.000718, 0.000919, 0.00118
	Black	Men	50	0.000507, 0.000648, 0.000827, 0.00106, 0.00135
	Black	Men	51	0.000595, 0.000754, 0.000953, 0.00121, 0.00153
	Black	Men	52	0.0007, 0.000876, 0.0011, 0.00137, 0.00171
	Black	Men	53	0.000814, 0.00101, 0.00125, 0.00155, 0.00192
	Black	Men	54	0.000929, 0.00115, 0.00141, 0.00174, 0.00215
	Black	Men	55	0.00105, 0.00129, 0.00158, 0.00194, 0.00238
	Black	Men	56	0.00117, 0.00143, 0.00175, 0.00213, 0.00261
	Black	Men	57	0.0013, 0.00158, 0.00192, 0.00233, 0.00282
	Black	Men	58	0.00144, 0.00174, 0.00209, 0.00251, 0.00303
	Black	Men	59	0.00158, 0.00189, 0.00226, 0.0027, 0.00324
	Black	Men	60	0.00172, 0.00205, 0.00243, 0.00289, 0.00344
	Black	Men	61	0.00186, 0.0022, 0.0026, 0.00306, 0.00362
	Black	Men	62	0.00201, 0.00235, 0.00275, 0.00322, 0.00377
	Black	Men	63	0.00215, 0.0025, 0.0029, 0.00336, 0.00391
	Black	Men	64	0.00228, 0.00264, 0.00304, 0.0035, 0.00404
	Black	Men	65	0.00241, 0.00277, 0.00318, 0.00366, 0.00421
	Black	Men	66	0.00253, 0.00291, 0.00335, 0.00385, 0.00442
	Black	Men	67	0.00265, 0.00307, 0.00354, 0.00408, 0.00471
	Black	Men	68	0.00279, 0.00324, 0.00376, 0.00436, 0.00506
	Black	Men	69	0.00295, 0.00344, 0.00401, 0.00467, 0.00545
	Black	Men	70	0.00314, 0.00367, 0.00428, 0.005, 0.00585
	Black	Men	71	0.00335, 0.00392, 0.00458, 0.00536, 0.00627
	Black	Men	72	0.00357, 0.00419, 0.0049, 0.00574, 0.00673
	Black	Men	73	0.00381, 0.00447, 0.00524, 0.00614, 0.00721
	Black	Men	74	0.00407, 0.00478, 0.00561, 0.00658, 0.00773
	Black	Men	75	0.00437, 0.00513, 0.00601, 0.00705, 0.00828
	Black	Men	76	0.00471, 0.00553, 0.00647, 0.00757, 0.00887
	Black	Men	77	0.0051, 0.00597, 0.00698, 0.00815, 0.00954
	Black	Men	78	0.00552, 0.00646, 0.00755, 0.00883, 0.0103
	Black	Men	79	0.00596, 0.00699, 0.00819, 0.0096, 0.0113
	Black	Men	80	0.00641, 0.00755, 0.00888, 0.0105, 0.0123
	Black	Men	81	0.00684, 0.00812, 0.00961, 0.0114, 0.0135
	Black	Men	82	0.00724, 0.00867, 0.0104, 0.0124, 0.0148

Variable	Race/ethnicity	Sex	Age	Distribution
	Black	Men	83	0.00759, 0.0092, 0.0111, 0.0134, 0.0163
	Black	Men	84	0.00792, 0.0097, 0.0119, 0.0145, 0.0178
	Black	Women	30	0.0000126, 0.000018, 0.0000254, 0.000036, 0.0000512
	Black	Women	31	0.000016, 0.000022, 0.00003, 0.000041, 0.0000562
	Black	Women	32	0.0000199, 0.0000266, 0.0000354, 0.0000472, 0.000063
	Black	Women	33	0.0000243, 0.0000319, 0.0000417, 0.0000545, 0.0000714
	Black	Women	34	0.000029, 0.0000376, 0.0000487, 0.0000631, 0.0000819
	Black	Women	35	0.0000336, 0.0000436, 0.0000565, 0.0000732, 0.0000951
	Black	Women	36	0.0000382, 0.0000499, 0.0000649, 0.0000845, 0.00011
	Black	Women	37	0.0000432, 0.0000566, 0.0000739, 0.0000966, 0.000127
	Black	Women	38	0.0000484, 0.0000637, 0.0000836, 0.00011, 0.000144
	Black	Women	39	0.0000538, 0.0000712, 0.0000939, 0.000124, 0.000164
	Black	Women	40	0.0000601, 0.0000796, 0.000105, 0.000138, 0.000183
	Black	Women	41	0.0000682, 0.0000894, 0.000117, 0.000153, 0.000201
	Black	Women	42	0.0000784, 0.000101, 0.00013, 0.000167, 0.000216
	Black	Women	43	0.0000906, 0.000115, 0.000145, 0.000183, 0.000232
	Black	Women	44	0.000104, 0.00013, 0.000163, 0.000203, 0.000254
	Black	Women	45	0.000118, 0.000147, 0.000183, 0.000228, 0.000284
	Black	Women	46	0.000136, 0.000168, 0.000208, 0.000258, 0.000319
	Black	Women	47	0.000158, 0.000194, 0.000238, 0.000292, 0.00036
	Black	Women	48	0.000182, 0.000224, 0.000274, 0.000335, 0.000411
	Black	Women	49	0.000207, 0.000256, 0.000316, 0.000389, 0.000481
	Black	Women	50	0.000235, 0.000292, 0.000363, 0.000451, 0.000561
	Black	Women	51	0.000273, 0.000337, 0.000415, 0.000512, 0.000632
	Black	Women	52	0.00032, 0.000389, 0.000472, 0.000573, 0.000697
	Black	Women	53	0.000364, 0.000441, 0.000532, 0.000643, 0.000779
	Black	Women	54	0.000404, 0.000492, 0.000598, 0.000727, 0.000886
	Black	Women	55	0.000451, 0.00055, 0.000669, 0.000815, 0.000994
	Black	Women	56	0.000509, 0.000617, 0.000746, 0.000903, 0.00109
	Black	Women	57	0.000572, 0.000689, 0.000828, 0.000995, 0.0012
	Black	Women	58	0.000633, 0.000761, 0.000913, 0.00109, 0.00132
	Black	Women	59	0.000688, 0.00083, 0.000999, 0.0012, 0.00145
	Black	Women	60	0.000739, 0.000897, 0.00109, 0.00132, 0.0016
	Black	Women	61	0.000786, 0.000961, 0.00117, 0.00143, 0.00175
	Black	Women	62	0.000827, 0.00102, 0.00126, 0.00154, 0.0019
	Black	Women	63	0.000862, 0.00107, 0.00134, 0.00166, 0.00207
	Black	Women	64	0.000891, 0.00113, 0.00142, 0.00178, 0.00225
	Black	Women	65	0.000923, 0.00118, 0.0015, 0.00191, 0.00245
	Black	Women	66	0.000963, 0.00124, 0.0016, 0.00206, 0.00266
	Black	Women	67	0.00102, 0.00132, 0.00172, 0.00223, 0.0029
	Black	Women	68	0.0011, 0.00143, 0.00186, 0.00242, 0.00315
	Black	Women	69	0.0012, 0.00156, 0.00203, 0.00263, 0.00343
	Black	Women	70	0.00132, 0.00171, 0.00222, 0.00287, 0.00372

Variable	Race/ethnicity	Sex	Age	Distribution
	Black	Women	71	0.00146, 0.00188, 0.00242, 0.00312, 0.00403
	Black	Women	72	0.0016, 0.00206, 0.00264, 0.0034, 0.00437
	Black	Women	73	0.00175, 0.00225, 0.00288, 0.00369, 0.00475
	Black	Women	74	0.00191, 0.00245, 0.00314, 0.00402, 0.00517
	Black	Women	75	0.00208, 0.00267, 0.00343, 0.0044, 0.00565
	Black	Women	76	0.00226, 0.00291, 0.00375, 0.00483, 0.00624
	Black	Women	77	0.00243, 0.00317, 0.00412, 0.00535, 0.00697
	Black	Women	78	0.00262, 0.00345, 0.00453, 0.00595, 0.00784
	Black	Women	79	0.0028, 0.00374, 0.00499, 0.00664, 0.00888
	Black	Women	80	0.003, 0.00406, 0.00549, 0.00742, 0.0101
	Black	Women	81	0.00321, 0.00441, 0.00604, 0.00827, 0.0114
	Black	Women	82	0.00347, 0.0048, 0.00663, 0.00916, 0.0127
	Black	Women	83	0.00375, 0.00522, 0.00725, 0.0101, 0.014
	Black	Women	84	0.00406, 0.00567, 0.00789, 0.011, 0.0154
	Hispanic	Men	30	0.0000103, 0.0000146, 0.0000205, 0.0000289, 0.0000408
	Hispanic	Men	31	0.0000128, 0.0000177, 0.0000244, 0.0000337, 0.0000466
	Hispanic	Men	32	0.0000158, 0.0000214, 0.0000289, 0.0000392, 0.0000531
	Hispanic	Men	33	0.0000195, 0.0000258, 0.0000342, 0.0000453, 0.0000602
	Hispanic	Men	34	0.000024, 0.0000311, 0.0000403, 0.0000521, 0.0000675
	Hispanic	Men	35	0.0000293, 0.0000372, 0.0000471, 0.0000597, 0.0000758
	Hispanic	Men	36	0.000035, 0.0000438, 0.0000547, 0.0000684, 0.0000856
	Hispanic	Men	37	0.000041, 0.0000509, 0.0000632, 0.0000784, 0.0000974
	Hispanic	Men	38	0.0000475, 0.0000588, 0.0000726, 0.0000898, 0.000111
	Hispanic	Men	39	0.0000549, 0.0000677, 0.0000832, 0.000102, 0.000126
	Hispanic	Men	40	0.0000639, 0.000078, 0.0000952, 0.000116, 0.000142
	Hispanic	Men	41	0.0000745, 0.00009, 0.000109, 0.000131, 0.000158
	Hispanic	Men	42	0.0000869, 0.000104, 0.000124, 0.000147, 0.000176
	Hispanic	Men	43	0.000101, 0.000119, 0.000141, 0.000166, 0.000196
	Hispanic	Men	44	0.000117, 0.000137, 0.00016, 0.000187, 0.000219
	Hispanic	Men	45	0.000135, 0.000158, 0.000183, 0.000213, 0.000248
	Hispanic	Men	46	0.000155, 0.00018, 0.00021, 0.000244, 0.000285
	Hispanic	Men	47	0.000178, 0.000207, 0.000241, 0.000281, 0.000327
	Hispanic	Men	48	0.000208, 0.00024, 0.000278, 0.000321, 0.000372
	Hispanic	Men	49	0.000244, 0.00028, 0.000322, 0.000369, 0.000424
	Hispanic	Men	50	0.000283, 0.000325, 0.000373, 0.000427, 0.000491
	Hispanic	Men	51	0.000325, 0.000375, 0.000431, 0.000496, 0.000571
	Hispanic	Men	52	0.000372, 0.00043, 0.000497, 0.000575, 0.000665
	Hispanic	Men	53	0.000419, 0.000489, 0.00057, 0.000664, 0.000774
	Hispanic	Men	54	0.000468, 0.000551, 0.000647, 0.00076, 0.000895
	Hispanic	Men	55	0.000525, 0.000619, 0.000729, 0.000858, 0.00101
	Hispanic	Men	56	0.00059, 0.000693, 0.000813, 0.000955, 0.00112
	Hispanic	Men	57	0.000661, 0.000772, 0.000901, 0.00105, 0.00123
	Hispanic	Men	58	0.000735, 0.000854, 0.000992, 0.00115, 0.00134

Variable	Race/ethnicity	Sex	Age	Distribution
	Hispanic	Men	59	0.000809, 0.000938, 0.00109, 0.00126, 0.00146
	Hispanic	Men	60	0.000881, 0.00102, 0.00118, 0.00137, 0.00159
	Hispanic	Men	61	0.00095, 0.0011, 0.00128, 0.00148, 0.00172
	Hispanic	Men	62	0.00102, 0.00118, 0.00137, 0.0016, 0.00186
	Hispanic	Men	63	0.00108, 0.00126, 0.00147, 0.00171, 0.00199
	Hispanic	Men	64	0.00115, 0.00134, 0.00157, 0.00183, 0.00213
	Hispanic	Men	65	0.00122, 0.00143, 0.00167, 0.00195, 0.00228
	Hispanic	Men	66	0.00129, 0.00152, 0.00178, 0.00208, 0.00244
	Hispanic	Men	67	0.00137, 0.00162, 0.0019, 0.00224, 0.00264
	Hispanic	Men	68	0.00146, 0.00173, 0.00205, 0.00243, 0.00289
	Hispanic	Men	69	0.00155, 0.00186, 0.00222, 0.00265, 0.00318
	Hispanic	Men	70	0.00166, 0.00201, 0.00242, 0.00291, 0.00351
	Hispanic	Men	71	0.00179, 0.00218, 0.00264, 0.00321, 0.0039
	Hispanic	Men	72	0.00194, 0.00237, 0.00289, 0.00353, 0.00431
	Hispanic	Men	73	0.00211, 0.00259, 0.00317, 0.00388, 0.00476
	Hispanic	Men	74	0.00231, 0.00284, 0.00347, 0.00426, 0.00523
	Hispanic	Men	75	0.00254, 0.00312, 0.00382, 0.00467, 0.00573
	Hispanic	Men	76	0.00281, 0.00344, 0.00421, 0.00514, 0.00629
	Hispanic	Men	77	0.00313, 0.00382, 0.00465, 0.00567, 0.00692
	Hispanic	Men	78	0.0035, 0.00426, 0.00517, 0.00628, 0.00764
	Hispanic	Men	79	0.0039, 0.00474, 0.00575, 0.00698, 0.00848
	Hispanic	Men	80	0.00431, 0.00525, 0.00639, 0.00777, 0.00947
	Hispanic	Men	81	0.00469, 0.00576, 0.00707, 0.00867, 0.0106
	Hispanic	Men	82	0.00503, 0.00626, 0.00776, 0.00963, 0.012
	Hispanic	Men	83	0.00535, 0.00674, 0.00846, 0.0106, 0.0134
	Hispanic	Men	84	0.00566, 0.00721, 0.00916, 0.0116, 0.0148
	Hispanic	Women	30	0.00000278, 0.00000404, 0.00000587, 0.00000851, 0.0000124
	Hispanic	Women	31	0.00000338, 0.0000048, 0.0000068, 0.00000964, 0.0000137
	Hispanic	Women	32	0.0000041, 0.0000057, 0.0000079, 0.0000109, 0.0000152
	Hispanic	Women	33	0.00000494, 0.00000674, 0.00000917, 0.0000125, 0.000017
	Hispanic	Women	34	0.00000586, 0.00000789, 0.0000106, 0.0000142, 0.0000192
	Hispanic	Women	35	0.00000681, 0.00000913, 0.0000122, 0.0000163, 0.0000219
	Hispanic	Women	36	0.00000779, 0.0000104, 0.000014, 0.0000187, 0.000025
	Hispanic	Women	37	0.00000883, 0.0000119, 0.0000159, 0.0000213, 0.0000286
	Hispanic	Women	38	0.00000997, 0.0000134, 0.000018, 0.0000242, 0.0000325
	Hispanic	Women	39	0.0000113, 0.0000152, 0.0000204, 0.0000273, 0.0000367
	Hispanic	Women	40	0.0000129, 0.0000172, 0.000023, 0.0000307, 0.000041
	Hispanic	Women	41	0.0000149, 0.0000197, 0.000026, 0.0000342, 0.0000451
	Hispanic	Women	42	0.0000176, 0.0000227, 0.0000294, 0.0000379, 0.000049
	Hispanic	Women	43	0.0000208, 0.0000263, 0.0000333, 0.0000421, 0.0000534
	Hispanic	Women	44	0.0000246, 0.0000306, 0.000038, 0.0000472, 0.0000588
	Hispanic	Women	45	0.0000292, 0.0000357, 0.0000436, 0.0000533, 0.0000653
	Hispanic	Women	46	0.0000349, 0.0000419, 0.0000503, 0.0000604, 0.0000727

Variable	Race/ethnicity	Sex	Age	Distribution
	Hispanic	Women	47	0.0000419, 0.0000495, 0.0000583, 0.0000688, 0.0000812
	Hispanic	Women	48	0.0000499, 0.0000582, 0.0000678, 0.000079, 0.0000922
	Hispanic	Women	49	0.0000583, 0.0000679, 0.0000791, 0.000092, 0.000107
	Hispanic	Women	50	0.0000668, 0.0000786, 0.0000924, 0.000109, 0.000128
	Hispanic	Women	51	0.0000758, 0.0000906, 0.000108, 0.000129, 0.000154
	Hispanic	Women	52	0.0000859, 0.000104, 0.000127, 0.000153, 0.000186
	Hispanic	Women	53	0.0000976, 0.00012, 0.000148, 0.000182, 0.000224
	Hispanic	Women	54	0.000111, 0.000138, 0.000172, 0.000214, 0.000266
	Hispanic	Women	55	0.000127, 0.00016, 0.0002, 0.00025, 0.000313
	Hispanic	Women	56	0.000146, 0.000184, 0.000231, 0.00029, 0.000365
	Hispanic	Women	57	0.000166, 0.000211, 0.000266, 0.000336, 0.000425
	Hispanic	Women	58	0.000188, 0.00024, 0.000305, 0.000388, 0.000495
	Hispanic	Women	59	0.000212, 0.000272, 0.000349, 0.000447, 0.000574
	Hispanic	Women	60	0.000236, 0.000306, 0.000396, 0.000512, 0.000664
	Hispanic	Women	61	0.000261, 0.000342, 0.000446, 0.000582, 0.000761
	Hispanic	Women	62	0.000288, 0.000379, 0.000498, 0.000654, 0.000862
	Hispanic	Women	63	0.000317, 0.000419, 0.000552, 0.000727, 0.000961
	Hispanic	Women	64	0.00035, 0.000462, 0.000608, 0.0008, 0.00106
	Hispanic	Women	65	0.000387, 0.000509, 0.000667, 0.000875, 0.00115
	Hispanic	Women	66	0.00043, 0.000563, 0.000734, 0.000957, 0.00125
	Hispanic	Women	67	0.000479, 0.000624, 0.000811, 0.00105, 0.00137
	Hispanic	Women	68	0.000534, 0.000694, 0.0009, 0.00117, 0.00152
	Hispanic	Women	69	0.000597, 0.000775, 0.001, 0.0013, 0.00169
	Hispanic	Women	70	0.000669, 0.000868, 0.00112, 0.00146, 0.00189
	Hispanic	Women	71	0.000751, 0.000974, 0.00126, 0.00163, 0.00211
	Hispanic	Women	72	0.000844, 0.00109, 0.00142, 0.00183, 0.00237
	Hispanic	Women	73	0.000948, 0.00123, 0.00159, 0.00206, 0.00267
	Hispanic	Women	74	0.00107, 0.00138, 0.0018, 0.00233, 0.00302
	Hispanic	Women	75	0.0012, 0.00156, 0.00203, 0.00264, 0.00344
	Hispanic	Women	76	0.00134, 0.00176, 0.0023, 0.00301, 0.00394
	Hispanic	Women	77	0.00151, 0.00199, 0.00262, 0.00344, 0.00454
	Hispanic	Women	78	0.0017, 0.00225, 0.00298, 0.00395, 0.00524
	Hispanic	Women	79	0.00191, 0.00256, 0.00341, 0.00454, 0.00607
	Hispanic	Women	80	0.00215, 0.0029, 0.00389, 0.00523, 0.00704
	Hispanic	Women	81	0.00242, 0.00328, 0.00444, 0.00601, 0.00817
	Hispanic	Women	82	0.00269, 0.00369, 0.00505, 0.00691, 0.00948
	Hispanic	Women	83	0.00296, 0.00411, 0.0057, 0.0079, 0.011
	Hispanic	Women	84	0.00323, 0.00454, 0.00638, 0.00896, 0.0126
	White	Men	30	0.0000184, 0.0000251, 0.0000342, 0.0000467, 0.0000639
	White	Men	31	0.0000236, 0.0000316, 0.000042, 0.000056, 0.0000749
	White	Men	32	0.0000299, 0.0000392, 0.0000513, 0.0000671, 0.0000881
	White	Men	33	0.000037, 0.000048, 0.000062, 0.0000801, 0.000104
	White	Men	34	0.0000449, 0.0000578, 0.0000742, 0.0000952, 0.000122

Variable	Race/ethnicity	Sex	Age	Distribution
	White	Men	35	0.0000532, 0.0000684, 0.0000877, 0.000112, 0.000145
	White	Men	36	0.0000623, 0.0000801, 0.000103, 0.000132, 0.000169
	White	Men	37	0.0000727, 0.0000932, 0.000119, 0.000153, 0.000196
	White	Men	38	0.0000853, 0.000109, 0.000138, 0.000175, 0.000223
	White	Men	39	0.000101, 0.000127, 0.000159, 0.000199, 0.000249
	White	Men	40	0.000119, 0.000147, 0.000181, 0.000223, 0.000275
	White	Men	41	0.000141, 0.00017, 0.000206, 0.000248, 0.000301
	White	Men	42	0.000164, 0.000195, 0.000232, 0.000275, 0.000328
	White	Men	43	0.000189, 0.000222, 0.000261, 0.000306, 0.000359
	White	Men	44	0.000217, 0.000252, 0.000293, 0.00034, 0.000395
	White	Men	45	0.000249, 0.000287, 0.000329, 0.000378, 0.000434
	White	Men	46	0.000288, 0.000327, 0.000371, 0.000421, 0.000478
	White	Men	47	0.000333, 0.000374, 0.00042, 0.000472, 0.00053
	White	Men	48	0.000383, 0.000427, 0.000477, 0.000532, 0.000595
	White	Men	49	0.000438, 0.000488, 0.000542, 0.000603, 0.000672
	White	Men	50	0.000503, 0.000557, 0.000617, 0.000682, 0.000756
	White	Men	51	0.000579, 0.000636, 0.000699, 0.000767, 0.000843
	White	Men	52	0.00066, 0.000722, 0.000788, 0.00086, 0.00094
	White	Men	53	0.000737, 0.000807, 0.000883, 0.000966, 0.00106
	White	Men	54	0.000806, 0.000889, 0.000981, 0.00108, 0.00119
	White	Men	55	0.000873, 0.000973, 0.00108, 0.0012, 0.00134
	White	Men	56	0.000942, 0.00106, 0.00118, 0.00133, 0.00149
	White	Men	57	0.00101, 0.00114, 0.00129, 0.00145, 0.00164
	White	Men	58	0.00108, 0.00123, 0.00139, 0.00158, 0.00179
	White	Men	59	0.00116, 0.00132, 0.0015, 0.00171, 0.00194
	White	Men	60	0.00124, 0.00141, 0.00161, 0.00184, 0.0021
	White	Men	61	0.00132, 0.00151, 0.00173, 0.00197, 0.00225
	White	Men	62	0.00141, 0.00161, 0.00184, 0.0021, 0.0024
	White	Men	63	0.00148, 0.0017, 0.00195, 0.00223, 0.00256
	White	Men	64	0.00155, 0.00179, 0.00205, 0.00236, 0.00271
	White	Men	65	0.00162, 0.00187, 0.00216, 0.00249, 0.00288
	White	Men	66	0.00168, 0.00196, 0.00228, 0.00265, 0.00308
	White	Men	67	0.00175, 0.00205, 0.00241, 0.00283, 0.00333
	White	Men	68	0.00183, 0.00217, 0.00258, 0.00306, 0.00363
	White	Men	69	0.00193, 0.00232, 0.00277, 0.00331, 0.00397
	White	Men	70	0.00208, 0.0025, 0.003, 0.00361, 0.00434
	White	Men	71	0.00225, 0.00271, 0.00327, 0.00393, 0.00474
	White	Men	72	0.00245, 0.00296, 0.00356, 0.00429, 0.00517
	White	Men	73	0.00267, 0.00323, 0.00389, 0.00468, 0.00565
	White	Men	74	0.00292, 0.00352, 0.00424, 0.00511, 0.00617
	White	Men	75	0.00318, 0.00384, 0.00464, 0.0056, 0.00676
	White	Men	76	0.00347, 0.00421, 0.00509, 0.00615, 0.00746
	White	Men	77	0.0038, 0.00462, 0.00561, 0.00681, 0.00828

Variable	Race/ethnicity	Sex	Age	Distribution
	White	Men	78	0.00419, 0.00511, 0.00623, 0.00758, 0.00926
	White	Men	79	0.00465, 0.00569, 0.00695, 0.00849, 0.0104
	White	Men	80	0.00521, 0.00638, 0.0078, 0.00954, 0.0117
	White	Men	81	0.00585, 0.00717, 0.00877, 0.0107, 0.0131
	White	Men	82	0.00657, 0.00805, 0.00983, 0.012, 0.0147
	White	Men	83	0.00734, 0.00898, 0.011, 0.0134, 0.0163
	White	Men	84	0.00813, 0.00992, 0.0121, 0.0147, 0.018
	White	Women	30	0.00000482, 0.00000733, 0.0000111, 0.0000168, 0.0000256
	White	Women	31	0.00000632, 0.00000928, 0.0000136, 0.0000199, 0.0000292
	White	Women	32	0.0000083, 0.0000117, 0.0000165, 0.0000233, 0.0000329
	White	Women	33	0.0000108, 0.0000147, 0.00002, 0.0000272, 0.000037
	White	Women	34	0.0000137, 0.0000182, 0.000024, 0.0000316, 0.0000419
	White	Women	35	0.0000169, 0.000022, 0.0000285, 0.0000368, 0.0000478
	White	Women	36	0.0000204, 0.0000261, 0.0000334, 0.0000427, 0.0000548
	White	Women	37	0.0000239, 0.0000305, 0.0000388, 0.0000494, 0.0000631
	White	Women	38	0.0000273, 0.0000349, 0.0000446, 0.0000569, 0.0000729
	White	Women	39	0.0000305, 0.0000394, 0.0000507, 0.0000652, 0.0000842
	White	Women	40	0.0000338, 0.000044, 0.0000571, 0.000074, 0.0000963
	White	Women	41	0.0000374, 0.0000489, 0.0000637, 0.0000829, 0.000108
	White	Women	42	0.0000415, 0.0000542, 0.0000706, 0.000092, 0.00012
	White	Women	43	0.0000461, 0.0000601, 0.0000781, 0.000102, 0.000133
	White	Women	44	0.0000509, 0.0000665, 0.0000866, 0.000113, 0.000147
	White	Women	45	0.0000567, 0.0000739, 0.0000961, 0.000125, 0.000163
	White	Women	46	0.0000646, 0.0000833, 0.000107, 0.000138, 0.000178
	White	Women	47	0.0000754, 0.0000952, 0.00012, 0.000151, 0.000191
	White	Women	48	0.0000895, 0.00011, 0.000135, 0.000166, 0.000203
	White	Women	49	0.000106, 0.000128, 0.000153, 0.000183, 0.000219
	White	Women	50	0.000125, 0.000147, 0.000173, 0.000204, 0.00024
	White	Women	51	0.000143, 0.000168, 0.000197, 0.00023, 0.000269
	White	Women	52	0.000163, 0.000191, 0.000223, 0.000261, 0.000305
	White	Women	53	0.000183, 0.000215, 0.000252, 0.000296, 0.000347
	White	Women	54	0.000205, 0.000241, 0.000284, 0.000334, 0.000393
	White	Women	55	0.000228, 0.000269, 0.000318, 0.000375, 0.000443
	White	Women	56	0.000254, 0.0003, 0.000355, 0.00042, 0.000497
	White	Women	57	0.000283, 0.000335, 0.000397, 0.000469, 0.000556
	White	Women	58	0.000316, 0.000375, 0.000443, 0.000523, 0.000619
	White	Women	59	0.000353, 0.000418, 0.000494, 0.000583, 0.00069
	White	Women	60	0.000391, 0.000464, 0.000549, 0.00065, 0.000772
	White	Women	61	0.000427, 0.000509, 0.000607, 0.000723, 0.000863
	White	Women	62	0.000463, 0.000555, 0.000666, 0.000798, 0.000958
	White	Women	63	0.000499, 0.000602, 0.000725, 0.000873, 0.00105
	White	Women	64	0.000536, 0.000649, 0.000786, 0.00095, 0.00115
	White	Women	65	0.000576, 0.000701, 0.000851, 0.00103, 0.00126

Variable	Race/ethnicity	Sex	Age	Distribution
	White	Women	66	0.000623, 0.00076, 0.000925, 0.00113, 0.00137
	White	Women	67	0.000679, 0.000829, 0.00101, 0.00123, 0.0015
	White	Women	68	0.000747, 0.000912, 0.00111, 0.00136, 0.00166
	White	Women	69	0.000826, 0.00101, 0.00123, 0.0015, 0.00183
	White	Women	70	0.000916, 0.00112, 0.00137, 0.00167, 0.00204
	White	Women	71	0.00102, 0.00124, 0.00152, 0.00186, 0.00227
	White	Women	72	0.00113, 0.00138, 0.0017, 0.00208, 0.00255
	White	Women	73	0.00125, 0.00154, 0.00189, 0.00233, 0.00287
	White	Women	74	0.00138, 0.00171, 0.00212, 0.00262, 0.00324
	White	Women	75	0.00154, 0.00191, 0.00237, 0.00295, 0.00367
	White	Women	76	0.00171, 0.00214, 0.00267, 0.00333, 0.00416
	White	Women	77	0.00191, 0.0024, 0.00301, 0.00377, 0.00474
	White	Women	78	0.00215, 0.00271, 0.00341, 0.00429, 0.00542
	White	Women	79	0.00242, 0.00307, 0.00388, 0.00491, 0.00623
	White	Women	80	0.00274, 0.00349, 0.00444, 0.00564, 0.00719
	White	Women	81	0.00311, 0.00398, 0.00508, 0.00649, 0.00831
	White	Women	82	0.00352, 0.00452, 0.00581, 0.00745, 0.00959
	White	Women	83	0.00396, 0.00512, 0.0066, 0.00851, 0.011
	White	Women	84	0.00442, 0.00574, 0.00744, 0.00963, 0.0125
CHD mortality rates for 2026 (0.01, 0.2, 0.5, 0.8. 0.99 percentiles of the empirical distribution produced during forecasting)				
	Black	Men	30	0.0000295, 0.0000422, 0.00006, 0.0000854, 0.000122
	Black	Men	31	0.0000377, 0.0000522, 0.0000722, 0.0000998, 0.000138
	Black	Men	32	0.0000476, 0.0000642, 0.0000863, 0.000116, 0.000157
	Black	Men	33	0.0000593, 0.000078, 0.000102, 0.000134, 0.000176
	Black	Men	34	0.0000722, 0.0000932, 0.00012, 0.000154, 0.000199
	Black	Men	35	0.0000854, 0.000109, 0.000139, 0.000177, 0.000226
	Black	Men	36	0.0000983, 0.000125, 0.000159, 0.000202, 0.000257
	Black	Men	37	0.000112, 0.000142, 0.00018, 0.000229, 0.000291
	Black	Men	38	0.000125, 0.000159, 0.000202, 0.000257, 0.000327
	Black	Men	39	0.00014, 0.000178, 0.000226, 0.000286, 0.000364
	Black	Men	40	0.000156, 0.000198, 0.00025, 0.000316, 0.000401
	Black	Men	41	0.000174, 0.000219, 0.000276, 0.000348, 0.000439
	Black	Men	42	0.000193, 0.000243, 0.000305, 0.000383, 0.000481
	Black	Men	43	0.000213, 0.000269, 0.000338, 0.000424, 0.000534
	Black	Men	44	0.000234, 0.000297, 0.000375, 0.000474, 0.0006
	Black	Men	45	0.000259, 0.00033, 0.000419, 0.000533, 0.000679
	Black	Men	46	0.000289, 0.00037, 0.000472, 0.000602, 0.00077
	Black	Men	47	0.000326, 0.000418, 0.000535, 0.000684, 0.000877
	Black	Men	48	0.000371, 0.000477, 0.000611, 0.000783, 0.001
	Black	Men	49	0.000426, 0.000548, 0.000702, 0.0009, 0.00116
	Black	Men	50	0.000495, 0.000634, 0.00081, 0.00103, 0.00133
	Black	Men	51	0.000582, 0.000739, 0.000935, 0.00118, 0.0015
	Black	Men	52	0.000685, 0.00086, 0.00108, 0.00135, 0.00169

Variable	Race/ethnicity	Sex	Age	Distribution
	Black	Men	53	0.000798, 0.000992, 0.00123, 0.00152, 0.00189
	Black	Men	54	0.000911, 0.00113, 0.00139, 0.00172, 0.00212
	Black	Men	55	0.00103, 0.00127, 0.00156, 0.00191, 0.00236
	Black	Men	56	0.00115, 0.00141, 0.00172, 0.00211, 0.00259
	Black	Men	57	0.00128, 0.00156, 0.00189, 0.0023, 0.0028
	Black	Men	58	0.00141, 0.00171, 0.00206, 0.00249, 0.00301
	Black	Men	59	0.00155, 0.00186, 0.00223, 0.00267, 0.00321
	Black	Men	60	0.00169, 0.00201, 0.0024, 0.00286, 0.00341
	Black	Men	61	0.00182, 0.00216, 0.00256, 0.00302, 0.00358
	Black	Men	62	0.00196, 0.00231, 0.00271, 0.00318, 0.00373
	Black	Men	63	0.0021, 0.00244, 0.00285, 0.00331, 0.00386
	Black	Men	64	0.00223, 0.00258, 0.00298, 0.00345, 0.00399
	Black	Men	65	0.00235, 0.00271, 0.00312, 0.00359, 0.00415
	Black	Men	66	0.00246, 0.00284, 0.00328, 0.00378, 0.00436
	Black	Men	67	0.00258, 0.00299, 0.00346, 0.004, 0.00464
	Black	Men	68	0.00271, 0.00316, 0.00367, 0.00427, 0.00498
	Black	Men	69	0.00286, 0.00335, 0.00392, 0.00458, 0.00536
	Black	Men	70	0.00305, 0.00357, 0.00419, 0.00491, 0.00576
	Black	Men	71	0.00325, 0.00382, 0.00448, 0.00525, 0.00617
	Black	Men	72	0.00347, 0.00408, 0.00479, 0.00562, 0.00661
	Black	Men	73	0.00369, 0.00435, 0.00512, 0.00602, 0.00709
	Black	Men	74	0.00394, 0.00465, 0.00547, 0.00644, 0.00759
	Black	Men	75	0.00423, 0.00499, 0.00587, 0.0069, 0.00813
	Black	Men	76	0.00457, 0.00537, 0.00631, 0.00741, 0.00871
	Black	Men	77	0.00495, 0.00581, 0.00681, 0.00798, 0.00937
	Black	Men	78	0.00535, 0.00629, 0.00737, 0.00864, 0.0102
	Black	Men	79	0.00578, 0.0068, 0.008, 0.0094, 0.0111
	Black	Men	80	0.00621, 0.00734, 0.00867, 0.0102, 0.0121
	Black	Men	81	0.00663, 0.00789, 0.00938, 0.0112, 0.0133
	Black	Men	82	0.00701, 0.00843, 0.0101, 0.0121, 0.0146
	Black	Men	83	0.00735, 0.00894, 0.0108, 0.0132, 0.016
	Black	Men	84	0.00767, 0.00943, 0.0116, 0.0142, 0.0174
	Black	Women	30	0.0000124, 0.0000177, 0.0000253, 0.0000362, 0.0000518
	Black	Women	31	0.0000157, 0.0000217, 0.0000299, 0.0000412, 0.0000568
	Black	Women	32	0.0000196, 0.0000263, 0.0000353, 0.0000473, 0.0000637
	Black	Women	33	0.0000239, 0.0000316, 0.0000415, 0.0000547, 0.0000721
	Black	Women	34	0.0000285, 0.0000373, 0.0000486, 0.0000633, 0.0000827
	Black	Women	35	0.0000331, 0.0000432, 0.0000563, 0.0000734, 0.000096
	Black	Women	36	0.0000376, 0.0000493, 0.0000647, 0.0000847, 0.000111
	Black	Women	37	0.0000424, 0.0000559, 0.0000735, 0.0000967, 0.000128
	Black	Women	38	0.0000474, 0.0000628, 0.000083, 0.00011, 0.000145
	Black	Women	39	0.0000527, 0.0000701, 0.0000931, 0.000124, 0.000165
	Black	Women	40	0.0000588, 0.0000783, 0.000104, 0.000138, 0.000184

Variable	Race/ethnicity	Sex	Age	Distribution
	Black	Women	41	0.0000666, 0.0000878, 0.000115, 0.000152, 0.0002
	Black	Women	42	0.0000765, 0.0000992, 0.000128, 0.000166, 0.000215
	Black	Women	43	0.0000884, 0.000112, 0.000143, 0.000181, 0.000231
	Black	Women	44	0.000101, 0.000127, 0.00016, 0.0002, 0.000252
	Black	Women	45	0.000115, 0.000144, 0.00018, 0.000224, 0.000281
	Black	Women	46	0.000132, 0.000164, 0.000204, 0.000254, 0.000316
	Black	Women	47	0.000153, 0.000189, 0.000233, 0.000288, 0.000355
	Black	Women	48	0.000178, 0.000219, 0.000269, 0.00033, 0.000406
	Black	Women	49	0.000202, 0.000251, 0.00031, 0.000383, 0.000475
	Black	Women	50	0.00023, 0.000287, 0.000357, 0.000445, 0.000555
	Black	Women	51	0.000267, 0.000331, 0.000409, 0.000505, 0.000626
	Black	Women	52	0.000313, 0.000382, 0.000465, 0.000566, 0.00069
	Black	Women	53	0.000357, 0.000434, 0.000526, 0.000637, 0.000773
	Black	Women	54	0.000397, 0.000485, 0.000591, 0.00072, 0.00088
	Black	Women	55	0.000443, 0.000542, 0.000662, 0.000808, 0.000989
	Black	Women	56	0.0005, 0.000608, 0.000738, 0.000896, 0.00109
	Black	Women	57	0.000563, 0.00068, 0.000819, 0.000987, 0.00119
	Black	Women	58	0.000622, 0.00075, 0.000903, 0.00109, 0.00131
	Black	Women	59	0.000676, 0.000818, 0.000989, 0.00119, 0.00145
	Black	Women	60	0.000726, 0.000884, 0.00107, 0.00131, 0.00159
	Black	Women	61	0.000772, 0.000946, 0.00116, 0.00142, 0.00174
	Black	Women	62	0.000811, 0.001, 0.00124, 0.00153, 0.00189
	Black	Women	63	0.000844, 0.00106, 0.00132, 0.00164, 0.00206
	Black	Women	64	0.000872, 0.0011, 0.0014, 0.00176, 0.00223
	Black	Women	65	0.000901, 0.00116, 0.00148, 0.00189, 0.00242
	Black	Women	66	0.00094, 0.00122, 0.00157, 0.00203, 0.00263
	Black	Women	67	0.000994, 0.0013, 0.00169, 0.00219, 0.00286
	Black	Women	68	0.00107, 0.0014, 0.00182, 0.00238, 0.00312
	Black	Women	69	0.00117, 0.00152, 0.00199, 0.00259, 0.00339
	Black	Women	70	0.00128, 0.00167, 0.00217, 0.00282, 0.00368
	Black	Women	71	0.00142, 0.00184, 0.00238, 0.00307, 0.00398
	Black	Women	72	0.00156, 0.00201, 0.00259, 0.00334, 0.00431
	Black	Women	73	0.0017, 0.0022, 0.00282, 0.00363, 0.00468
	Black	Women	74	0.00186, 0.00239, 0.00308, 0.00396, 0.0051
	Black	Women	75	0.00202, 0.00261, 0.00336, 0.00432, 0.00558
	Black	Women	76	0.00219, 0.00284, 0.00368, 0.00475, 0.00616
	Black	Women	77	0.00237, 0.0031, 0.00404, 0.00526, 0.00687
	Black	Women	78	0.00255, 0.00337, 0.00444, 0.00585, 0.00774
	Black	Women	79	0.00273, 0.00366, 0.00489, 0.00654, 0.00877
	Black	Women	80	0.00291, 0.00397, 0.00538, 0.00731, 0.00995
	Black	Women	81	0.00313, 0.00431, 0.00593, 0.00814, 0.0112
	Black	Women	82	0.00337, 0.00469, 0.00651, 0.00902, 0.0126
	Black	Women	83	0.00365, 0.0051, 0.00712, 0.00992, 0.0139

Variable	Race/ethnicity	Sex	Age	Distribution
	Black	Women	84	0.00394, 0.00554, 0.00775, 0.0108, 0.0152
	Hispanic	Men	30	0.0000101, 0.0000144, 0.0000204, 0.000029, 0.0000413
	Hispanic	Men	31	0.0000126, 0.0000175, 0.0000243, 0.0000337, 0.000047
	Hispanic	Men	32	0.0000155, 0.0000212, 0.0000288, 0.0000393, 0.0000536
	Hispanic	Men	33	0.0000192, 0.0000256, 0.0000341, 0.0000454, 0.0000607
	Hispanic	Men	34	0.0000236, 0.0000308, 0.0000401, 0.0000522, 0.0000681
	Hispanic	Men	35	0.0000288, 0.0000368, 0.0000469, 0.0000598, 0.0000764
	Hispanic	Men	36	0.0000343, 0.0000432, 0.0000544, 0.0000684, 0.0000863
	Hispanic	Men	37	0.0000401, 0.0000502, 0.0000627, 0.0000784, 0.0000982
	Hispanic	Men	38	0.0000463, 0.0000578, 0.000072, 0.0000896, 0.000112
	Hispanic	Men	39	0.0000535, 0.0000664, 0.0000823, 0.000102, 0.000127
	Hispanic	Men	40	0.0000621, 0.0000764, 0.0000939, 0.000115, 0.000142
	Hispanic	Men	41	0.0000725, 0.0000881, 0.000107, 0.00013, 0.000157
	Hispanic	Men	42	0.0000846, 0.000101, 0.000121, 0.000145, 0.000174
	Hispanic	Men	43	0.0000985, 0.000117, 0.000138, 0.000163, 0.000193
	Hispanic	Men	44	0.000114, 0.000134, 0.000157, 0.000184, 0.000216
	Hispanic	Men	45	0.000131, 0.000154, 0.000179, 0.000209, 0.000244
	Hispanic	Men	46	0.00015, 0.000176, 0.000205, 0.000239, 0.00028
	Hispanic	Men	47	0.000173, 0.000202, 0.000236, 0.000275, 0.000321
	Hispanic	Men	48	0.000202, 0.000234, 0.000272, 0.000315, 0.000365
	Hispanic	Men	49	0.000237, 0.000273, 0.000314, 0.000362, 0.000417
	Hispanic	Men	50	0.000276, 0.000317, 0.000365, 0.000419, 0.000482
	Hispanic	Men	51	0.000318, 0.000367, 0.000423, 0.000487, 0.000562
	Hispanic	Men	52	0.000364, 0.000422, 0.000488, 0.000565, 0.000655
	Hispanic	Men	53	0.000411, 0.00048, 0.00056, 0.000654, 0.000764
	Hispanic	Men	54	0.000459, 0.000541, 0.000637, 0.00075, 0.000884
	Hispanic	Men	55	0.000515, 0.000609, 0.000718, 0.000847, 0.001
	Hispanic	Men	56	0.000579, 0.000682, 0.000802, 0.000943, 0.00111
	Hispanic	Men	57	0.000648, 0.00076, 0.000889, 0.00104, 0.00122
	Hispanic	Men	58	0.000721, 0.000841, 0.000979, 0.00114, 0.00133
	Hispanic	Men	59	0.000793, 0.000922, 0.00107, 0.00124, 0.00145
	Hispanic	Men	60	0.000863, 0.001, 0.00117, 0.00135, 0.00157
	Hispanic	Men	61	0.00093, 0.00108, 0.00126, 0.00146, 0.00171
	Hispanic	Men	62	0.000995, 0.00116, 0.00135, 0.00158, 0.00184
	Hispanic	Men	63	0.00106, 0.00124, 0.00144, 0.00169, 0.00197
	Hispanic	Men	64	0.00112, 0.00131, 0.00154, 0.0018, 0.0021
	Hispanic	Men	65	0.00119, 0.00139, 0.00163, 0.00191, 0.00224
	Hispanic	Men	66	0.00126, 0.00148, 0.00174, 0.00204, 0.0024
	Hispanic	Men	67	0.00133, 0.00158, 0.00186, 0.0022, 0.0026
	Hispanic	Men	68	0.00142, 0.00169, 0.002, 0.00238, 0.00284
	Hispanic	Men	69	0.00151, 0.00181, 0.00217, 0.0026, 0.00312
	Hispanic	Men	70	0.00162, 0.00196, 0.00236, 0.00285, 0.00345
	Hispanic	Men	71	0.00174, 0.00213, 0.00258, 0.00314, 0.00383

Variable	Race/ethnicity	Sex	Age	Distribution
	Hispanic	Men	72	0.00189, 0.00231, 0.00283, 0.00346, 0.00424
	Hispanic	Men	73	0.00205, 0.00252, 0.0031, 0.0038, 0.00467
	Hispanic	Men	74	0.00224, 0.00276, 0.00339, 0.00417, 0.00513
	Hispanic	Men	75	0.00247, 0.00304, 0.00373, 0.00457, 0.00562
	Hispanic	Men	76	0.00273, 0.00335, 0.00411, 0.00503, 0.00617
	Hispanic	Men	77	0.00304, 0.00372, 0.00454, 0.00555, 0.00679
	Hispanic	Men	78	0.0034, 0.00415, 0.00505, 0.00615, 0.0075
	Hispanic	Men	79	0.00379, 0.00462, 0.00562, 0.00683, 0.00832
	Hispanic	Men	80	0.00419, 0.00512, 0.00624, 0.00761, 0.00931
	Hispanic	Men	81	0.00455, 0.00561, 0.0069, 0.00849, 0.0105
	Hispanic	Men	82	0.00489, 0.00609, 0.00758, 0.00944, 0.0118
	Hispanic	Men	83	0.00519, 0.00656, 0.00827, 0.0104, 0.0132
	Hispanic	Men	84	0.00549, 0.00702, 0.00895, 0.0114, 0.0146
	Hispanic	Women	30	0.00000272, 0.000004, 0.00000584, 0.00000854, 0.0000125
	Hispanic	Women	31	0.00000332, 0.00000475, 0.00000677, 0.00000966, 0.0000138
	Hispanic	Women	32	0.00000403, 0.00000564, 0.00000787, 0.000011, 0.0000154
	Hispanic	Women	33	0.00000486, 0.00000668, 0.00000914, 0.0000125, 0.0000172
	Hispanic	Women	34	0.00000577, 0.00000782, 0.0000106, 0.0000143, 0.0000193
	Hispanic	Women	35	0.00000671, 0.00000904, 0.0000122, 0.0000163, 0.000022
	Hispanic	Women	36	0.00000766, 0.0000103, 0.0000139, 0.0000187, 0.0000252
	Hispanic	Women	37	0.00000867, 0.0000117, 0.0000158, 0.0000212, 0.0000287
	Hispanic	Women	38	0.00000977, 0.0000132, 0.0000178, 0.0000241, 0.0000326
	Hispanic	Women	39	0.000011, 0.0000149, 0.0000201, 0.0000271, 0.0000367
	Hispanic	Women	40	0.0000126, 0.0000169, 0.0000227, 0.0000304, 0.0000409
	Hispanic	Women	41	0.0000146, 0.0000193, 0.0000255, 0.0000338, 0.0000448
	Hispanic	Women	42	0.0000171, 0.0000222, 0.0000288, 0.0000373, 0.0000485
	Hispanic	Women	43	0.0000203, 0.0000257, 0.0000326, 0.0000414, 0.0000526
	Hispanic	Women	44	0.0000239, 0.0000299, 0.0000372, 0.0000463, 0.0000578
	Hispanic	Women	45	0.0000284, 0.0000348, 0.0000426, 0.0000522, 0.0000641
	Hispanic	Women	46	0.0000339, 0.0000409, 0.0000492, 0.0000592, 0.0000713
	Hispanic	Women	47	0.0000407, 0.0000482, 0.000057, 0.0000673, 0.0000797
	Hispanic	Women	48	0.0000485, 0.0000568, 0.0000663, 0.0000773, 0.0000904
	Hispanic	Women	49	0.0000568, 0.0000663, 0.0000773, 0.0000901, 0.000105
	Hispanic	Women	50	0.0000652, 0.0000769, 0.0000905, 0.000107, 0.000126
	Hispanic	Women	51	0.0000741, 0.0000887, 0.000106, 0.000127, 0.000152
	Hispanic	Women	52	0.0000841, 0.000102, 0.000124, 0.000151, 0.000183
	Hispanic	Women	53	0.0000957, 0.000118, 0.000145, 0.000179, 0.00022
	Hispanic	Women	54	0.000109, 0.000136, 0.000169, 0.000211, 0.000262
	Hispanic	Women	55	0.000125, 0.000157, 0.000197, 0.000246, 0.000309
	Hispanic	Women	56	0.000143, 0.000181, 0.000228, 0.000286, 0.000361
	Hispanic	Women	57	0.000163, 0.000207, 0.000262, 0.000332, 0.000421
	Hispanic	Women	58	0.000185, 0.000236, 0.000301, 0.000383, 0.000489
	Hispanic	Women	59	0.000208, 0.000268, 0.000344, 0.000441, 0.000568

Variable	Race/ethnicity	Sex	Age	Distribution
	Hispanic	Women	60	0.000232, 0.000301, 0.00039, 0.000505, 0.000657
	Hispanic	Women	61	0.000256, 0.000336, 0.000439, 0.000574, 0.000752
	Hispanic	Women	62	0.000282, 0.000373, 0.00049, 0.000645, 0.000851
	Hispanic	Women	63	0.000311, 0.000411, 0.000542, 0.000716, 0.000947
	Hispanic	Women	64	0.000342, 0.000452, 0.000596, 0.000786, 0.00104
	Hispanic	Women	65	0.000378, 0.000498, 0.000654, 0.000858, 0.00113
	Hispanic	Women	66	0.00042, 0.00055, 0.000718, 0.000938, 0.00123
	Hispanic	Women	67	0.000467, 0.000609, 0.000793, 0.00103, 0.00135
	Hispanic	Women	68	0.00052, 0.000677, 0.00088, 0.00114, 0.00149
	Hispanic	Women	69	0.000581, 0.000756, 0.000982, 0.00127, 0.00166
	Hispanic	Women	70	0.000651, 0.000847, 0.0011, 0.00143, 0.00185
	Hispanic	Women	71	0.000731, 0.00095, 0.00123, 0.0016, 0.00207
	Hispanic	Women	72	0.000822, 0.00107, 0.00138, 0.00179, 0.00233
	Hispanic	Women	73	0.000923, 0.0012, 0.00155, 0.00202, 0.00262
	Hispanic	Women	74	0.00104, 0.00135, 0.00175, 0.00228, 0.00296
	Hispanic	Women	75	0.00116, 0.00152, 0.00198, 0.00258, 0.00337
	Hispanic	Women	76	0.00131, 0.00171, 0.00225, 0.00294, 0.00386
	Hispanic	Women	77	0.00147, 0.00194, 0.00255, 0.00336, 0.00444
	Hispanic	Women	78	0.00165, 0.0022, 0.00291, 0.00386, 0.00513
	Hispanic	Women	79	0.00186, 0.00249, 0.00332, 0.00444, 0.00594
	Hispanic	Women	80	0.00209, 0.00283, 0.0038, 0.00511, 0.00689
	Hispanic	Women	81	0.00235, 0.0032, 0.00433, 0.00588, 0.008
	Hispanic	Women	82	0.00261, 0.00359, 0.00493, 0.00675, 0.00929
	Hispanic	Women	83	0.00287, 0.004, 0.00556, 0.00772, 0.0108
	Hispanic	Women	84	0.00313, 0.00442, 0.00622, 0.00875, 0.0124
	White	Men	30	0.0000179, 0.0000248, 0.0000341, 0.0000469, 0.0000648
	White	Men	31	0.0000232, 0.0000312, 0.0000419, 0.0000563, 0.0000758
	White	Men	32	0.0000293, 0.0000388, 0.0000511, 0.0000674, 0.0000891
	White	Men	33	0.0000364, 0.0000475, 0.0000618, 0.0000804, 0.000105
	White	Men	34	0.0000442, 0.0000572, 0.000074, 0.0000955, 0.000124
	White	Men	35	0.0000524, 0.0000677, 0.0000874, 0.000113, 0.000146
	White	Men	36	0.0000612, 0.0000792, 0.000102, 0.000132, 0.000171
	White	Men	37	0.0000713, 0.000092, 0.000119, 0.000153, 0.000197
	White	Men	38	0.0000835, 0.000107, 0.000137, 0.000175, 0.000224
	White	Men	39	0.0000984, 0.000124, 0.000157, 0.000198, 0.00025
	White	Men	40	0.000116, 0.000144, 0.000179, 0.000222, 0.000275
	White	Men	41	0.000137, 0.000167, 0.000202, 0.000246, 0.0003
	White	Men	42	0.00016, 0.000191, 0.000228, 0.000272, 0.000325
	White	Men	43	0.000184, 0.000217, 0.000256, 0.000301, 0.000355
	White	Men	44	0.000211, 0.000246, 0.000287, 0.000334, 0.00039
	White	Men	45	0.000242, 0.000279, 0.000322, 0.000371, 0.000428
	White	Men	46	0.00028, 0.000319, 0.000363, 0.000413, 0.000471
	White	Men	47	0.000323, 0.000365, 0.000411, 0.000463, 0.000522

Variable	Race/ethnicity	Sex	Age	Distribution
	White	Men	48	0.000372, 0.000417, 0.000466, 0.000522, 0.000585
	White	Men	49	0.000426, 0.000476, 0.000531, 0.000592, 0.000661
	White	Men	50	0.00049, 0.000544, 0.000604, 0.00067, 0.000744
	White	Men	51	0.000565, 0.000622, 0.000685, 0.000754, 0.000831
	White	Men	52	0.000645, 0.000707, 0.000773, 0.000847, 0.000927
	White	Men	53	0.00072, 0.000791, 0.000867, 0.000952, 0.00104
	White	Men	54	0.000788, 0.000873, 0.000965, 0.00107, 0.00118
	White	Men	55	0.000855, 0.000955, 0.00107, 0.00119, 0.00133
	White	Men	56	0.000923, 0.00104, 0.00117, 0.00131, 0.00147
	White	Men	57	0.00099, 0.00112, 0.00127, 0.00143, 0.00162
	White	Men	58	0.00106, 0.00121, 0.00137, 0.00156, 0.00178
	White	Men	59	0.00113, 0.00129, 0.00148, 0.00169, 0.00193
	White	Men	60	0.00121, 0.00139, 0.00159, 0.00182, 0.00208
	White	Men	61	0.00129, 0.00148, 0.0017, 0.00194, 0.00223
	White	Men	62	0.00137, 0.00158, 0.00181, 0.00207, 0.00238
	White	Men	63	0.00145, 0.00166, 0.00191, 0.0022, 0.00253
	White	Men	64	0.00151, 0.00175, 0.00201, 0.00232, 0.00267
	White	Men	65	0.00158, 0.00183, 0.00211, 0.00245, 0.00284
	White	Men	66	0.00164, 0.00191, 0.00223, 0.0026, 0.00303
	White	Men	67	0.0017, 0.002, 0.00236, 0.00278, 0.00327
	White	Men	68	0.00178, 0.00212, 0.00252, 0.00299, 0.00357
	White	Men	69	0.00188, 0.00226, 0.00271, 0.00325, 0.0039
	White	Men	70	0.00202, 0.00244, 0.00293, 0.00353, 0.00426
	White	Men	71	0.00219, 0.00265, 0.00319, 0.00385, 0.00465
	White	Men	72	0.00238, 0.00288, 0.00348, 0.0042, 0.00508
	White	Men	73	0.0026, 0.00314, 0.00379, 0.00458, 0.00554
	White	Men	74	0.00283, 0.00343, 0.00414, 0.005, 0.00605
	White	Men	75	0.00309, 0.00374, 0.00453, 0.00547, 0.00663
	White	Men	76	0.00337, 0.00409, 0.00496, 0.00602, 0.00731
	White	Men	77	0.00369, 0.0045, 0.00548, 0.00666, 0.00812
	White	Men	78	0.00407, 0.00497, 0.00608, 0.00742, 0.00908
	White	Men	79	0.00451, 0.00554, 0.00679, 0.00831, 0.0102
	White	Men	80	0.00505, 0.00621, 0.00761, 0.00934, 0.0115
	White	Men	81	0.00568, 0.00698, 0.00855, 0.0105, 0.0129
	White	Men	82	0.00637, 0.00783, 0.00959, 0.0118, 0.0144
	White	Men	83	0.00711, 0.00873, 0.0107, 0.0131, 0.0161
	White	Men	84	0.00787, 0.00965, 0.0118, 0.0144, 0.0177
	White	Women	30	0.00000473, 0.00000725, 0.0000111, 0.0000169, 0.0000258
	White	Women	31	0.00000622, 0.00000919, 0.0000135, 0.0000199, 0.0000294
	White	Women	32	0.00000818, 0.0000116, 0.0000165, 0.0000233, 0.0000332
	White	Women	33	0.0000106, 0.0000146, 0.0000199, 0.0000272, 0.0000373
	White	Women	34	0.0000135, 0.000018, 0.0000239, 0.0000317, 0.0000422
	White	Women	35	0.0000167, 0.0000218, 0.0000284, 0.0000369, 0.0000482

Variable	Race/ethnicity	Sex	Age	Distribution
	White	Women	36	0.00002, 0.0000258, 0.0000332, 0.0000428, 0.0000552
	White	Women	37	0.0000234, 0.0000301, 0.0000385, 0.0000494, 0.0000635
	White	Women	38	0.0000267, 0.0000344, 0.0000442, 0.0000568, 0.0000732
	White	Women	39	0.0000298, 0.0000387, 0.0000501, 0.0000649, 0.0000843
	White	Women	40	0.000033, 0.0000432, 0.0000563, 0.0000734, 0.000096
	White	Women	41	0.0000365, 0.0000479, 0.0000626, 0.000082, 0.000108
	White	Women	42	0.0000405, 0.0000531, 0.0000693, 0.0000906, 0.000119
	White	Women	43	0.0000449, 0.0000587, 0.0000766, 0.0000998, 0.000131
	White	Women	44	0.0000496, 0.0000649, 0.0000847, 0.00011, 0.000144
	White	Women	45	0.0000552, 0.0000721, 0.0000939, 0.000122, 0.00016
	White	Women	46	0.0000628, 0.0000812, 0.000105, 0.000135, 0.000174
	White	Women	47	0.0000734, 0.0000928, 0.000117, 0.000148, 0.000187
	White	Women	48	0.0000872, 0.000107, 0.000132, 0.000162, 0.000199
	White	Women	49	0.000104, 0.000124, 0.000149, 0.000179, 0.000214
	White	Women	50	0.000121, 0.000144, 0.000169, 0.0002, 0.000236
	White	Women	51	0.00014, 0.000164, 0.000193, 0.000226, 0.000265
	White	Women	52	0.000159, 0.000187, 0.000219, 0.000256, 0.0003
	White	Women	53	0.000179, 0.000211, 0.000248, 0.000291, 0.000342
	White	Women	54	0.000201, 0.000237, 0.000279, 0.000329, 0.000388
	White	Women	55	0.000223, 0.000265, 0.000313, 0.00037, 0.000438
	White	Women	56	0.000249, 0.000295, 0.00035, 0.000414, 0.000492
	White	Women	57	0.000277, 0.000329, 0.000391, 0.000463, 0.00055
	White	Women	58	0.00031, 0.000368, 0.000436, 0.000517, 0.000613
	White	Women	59	0.000346, 0.000411, 0.000486, 0.000576, 0.000683
	White	Women	60	0.000383, 0.000455, 0.000541, 0.000642, 0.000764
	White	Women	61	0.000418, 0.0005, 0.000597, 0.000713, 0.000854
	White	Women	62	0.000452, 0.000545, 0.000654, 0.000786, 0.000946
	White	Women	63	0.000487, 0.000589, 0.000712, 0.000859, 0.00104
	White	Women	64	0.000523, 0.000635, 0.00077, 0.000934, 0.00113
	White	Women	65	0.000562, 0.000685, 0.000833, 0.00101, 0.00124
	White	Women	66	0.000607, 0.000742, 0.000905, 0.0011, 0.00135
	White	Women	67	0.000661, 0.000809, 0.000988, 0.00121, 0.00148
	White	Women	68	0.000727, 0.00089, 0.00109, 0.00133, 0.00163
	White	Women	69	0.000803, 0.000983, 0.0012, 0.00147, 0.0018
	White	Women	70	0.000891, 0.00109, 0.00133, 0.00163, 0.002
	White	Women	71	0.000989, 0.00121, 0.00149, 0.00182, 0.00223
	White	Women	72	0.0011, 0.00135, 0.00166, 0.00203, 0.0025
	White	Women	73	0.00121, 0.0015, 0.00185, 0.00228, 0.00282
	White	Women	74	0.00134, 0.00167, 0.00207, 0.00256, 0.00318
	White	Women	75	0.00149, 0.00186, 0.00232, 0.00288, 0.00359
	White	Women	76	0.00166, 0.00208, 0.0026, 0.00325, 0.00407
	White	Women	77	0.00186, 0.00234, 0.00294, 0.00369, 0.00464
	White	Women	78	0.00209, 0.00264, 0.00333, 0.0042, 0.00531

Variable	Race/ethnicity	Sex	Age	Distribution
	White	Women	79	0.00235, 0.00299, 0.00379, 0.0048, 0.0061
	White	Women	80	0.00266, 0.0034, 0.00433, 0.00552, 0.00705
	White	Women	81	0.00302, 0.00387, 0.00496, 0.00635, 0.00815
	White	Women	82	0.00341, 0.0044, 0.00567, 0.00729, 0.0094
	White	Women	83	0.00384, 0.00498, 0.00644, 0.00833, 0.0108
	White	Women	84	0.00429, 0.00559, 0.00725, 0.00942, 0.0123
CHD mortality rates for 2027 (0.01, 0.2, 0.5, 0.8. 0.99 percentiles of the empirical distribution produced during forecasting)				
	Black	Men	30	0.0000289, 0.0000416, 0.0000597, 0.0000856, 0.000123
	Black	Men	31	0.0000369, 0.0000516, 0.0000719, 0.0001, 0.00014
	Black	Men	32	0.0000467, 0.0000634, 0.0000859, 0.000116, 0.000158
	Black	Men	33	0.0000582, 0.0000771, 0.000102, 0.000134, 0.000178
	Black	Men	34	0.0000709, 0.0000921, 0.000119, 0.000154, 0.000201
	Black	Men	35	0.0000837, 0.000108, 0.000138, 0.000177, 0.000228
	Black	Men	36	0.0000964, 0.000124, 0.000158, 0.000202, 0.000259
	Black	Men	37	0.000109, 0.00014, 0.000179, 0.000229, 0.000293
	Black	Men	38	0.000122, 0.000157, 0.0002, 0.000256, 0.000328
	Black	Men	39	0.000136, 0.000175, 0.000223, 0.000285, 0.000364
	Black	Men	40	0.000152, 0.000194, 0.000247, 0.000314, 0.0004
	Black	Men	41	0.000169, 0.000215, 0.000272, 0.000344, 0.000437
	Black	Men	42	0.000188, 0.000238, 0.000299, 0.000377, 0.000476
	Black	Men	43	0.000208, 0.000262, 0.000331, 0.000417, 0.000526
	Black	Men	44	0.000228, 0.00029, 0.000367, 0.000465, 0.00059
	Black	Men	45	0.000252, 0.000322, 0.00041, 0.000522, 0.000667
	Black	Men	46	0.000281, 0.00036, 0.000461, 0.000589, 0.000755
	Black	Men	47	0.000318, 0.000408, 0.000522, 0.000669, 0.000859
	Black	Men	48	0.000362, 0.000465, 0.000597, 0.000766, 0.000985
	Black	Men	49	0.000416, 0.000535, 0.000687, 0.000881, 0.00113
	Black	Men	50	0.000484, 0.00062, 0.000794, 0.00101, 0.0013
	Black	Men	51	0.000569, 0.000724, 0.000918, 0.00116, 0.00148
	Black	Men	52	0.000672, 0.000844, 0.00106, 0.00132, 0.00166
	Black	Men	53	0.000783, 0.000974, 0.00121, 0.0015, 0.00187
	Black	Men	54	0.000894, 0.00111, 0.00137, 0.00169, 0.0021
	Black	Men	55	0.00101, 0.00124, 0.00153, 0.00189, 0.00234
	Black	Men	56	0.00113, 0.00139, 0.0017, 0.00209, 0.00256
	Black	Men	57	0.00126, 0.00153, 0.00187, 0.00227, 0.00277
	Black	Men	58	0.00139, 0.00168, 0.00203, 0.00246, 0.00298
	Black	Men	59	0.00152, 0.00183, 0.0022, 0.00264, 0.00318
	Black	Men	60	0.00165, 0.00198, 0.00236, 0.00282, 0.00338
	Black	Men	61	0.00179, 0.00212, 0.00252, 0.00299, 0.00355
	Black	Men	62	0.00192, 0.00226, 0.00266, 0.00313, 0.00369
	Black	Men	63	0.00205, 0.00239, 0.0028, 0.00326, 0.00382
	Black	Men	64	0.00217, 0.00252, 0.00292, 0.00339, 0.00394
	Black	Men	65	0.00229, 0.00265, 0.00306, 0.00353, 0.00409

Variable	Race/ethnicity	Sex	Age	Distribution
	Black	Men	66	0.0024, 0.00278, 0.00321, 0.00371, 0.00429
	Black	Men	67	0.00251, 0.00292, 0.00338, 0.00393, 0.00456
	Black	Men	68	0.00263, 0.00308, 0.00359, 0.00419, 0.0049
	Black	Men	69	0.00278, 0.00327, 0.00383, 0.00449, 0.00527
	Black	Men	70	0.00296, 0.00348, 0.00409, 0.00481, 0.00567
	Black	Men	71	0.00316, 0.00372, 0.00438, 0.00515, 0.00607
	Black	Men	72	0.00337, 0.00397, 0.00468, 0.00551, 0.0065
	Black	Men	73	0.00359, 0.00424, 0.005, 0.00589, 0.00697
	Black	Men	74	0.00383, 0.00453, 0.00534, 0.00631, 0.00746
	Black	Men	75	0.00411, 0.00485, 0.00572, 0.00675, 0.00798
	Black	Men	76	0.00443, 0.00523, 0.00615, 0.00725, 0.00854
	Black	Men	77	0.0048, 0.00565, 0.00664, 0.00781, 0.0092
	Black	Men	78	0.00519, 0.00611, 0.00719, 0.00846, 0.00997
	Black	Men	79	0.0056, 0.00662, 0.0078, 0.00921, 0.0109
	Black	Men	80	0.00602, 0.00714, 0.00846, 0.01, 0.0119
	Black	Men	81	0.00642, 0.00768, 0.00916, 0.0109, 0.0131
	Black	Men	82	0.0068, 0.0082, 0.00987, 0.0119, 0.0143
	Black	Men	83	0.00712, 0.00869, 0.0106, 0.0129, 0.0157
	Black	Men	84	0.00743, 0.00917, 0.0113, 0.0139, 0.0171
	Black	Women	30	0.0000121, 0.0000175, 0.0000252, 0.0000363, 0.0000524
	Black	Women	31	0.0000154, 0.0000215, 0.0000298, 0.0000413, 0.0000574
	Black	Women	32	0.0000192, 0.000026, 0.0000352, 0.0000475, 0.0000643
	Black	Women	33	0.0000235, 0.0000313, 0.0000414, 0.0000548, 0.0000728
	Black	Women	34	0.0000281, 0.000037, 0.0000484, 0.0000635, 0.0000835
	Black	Women	35	0.0000325, 0.0000428, 0.0000561, 0.0000736, 0.0000969
	Black	Women	36	0.0000369, 0.0000488, 0.0000644, 0.0000849, 0.000112
	Black	Women	37	0.0000416, 0.0000552, 0.0000731, 0.0000968, 0.000128
	Black	Women	38	0.0000465, 0.000062, 0.0000824, 0.00011, 0.000146
	Black	Women	39	0.0000516, 0.0000691, 0.0000923, 0.000123, 0.000165
	Black	Women	40	0.0000575, 0.000077, 0.000103, 0.000137, 0.000184
	Black	Women	41	0.000065, 0.0000862, 0.000114, 0.000151, 0.0002
	Black	Women	42	0.0000747, 0.0000973, 0.000126, 0.000164, 0.000214
	Black	Women	43	0.0000862, 0.00011, 0.00014, 0.000179, 0.000228
	Black	Women	44	0.0000986, 0.000124, 0.000157, 0.000197, 0.000249
	Black	Women	45	0.000112, 0.000141, 0.000176, 0.000221, 0.000277
	Black	Women	46	0.000128, 0.000161, 0.0002, 0.000249, 0.000312
	Black	Women	47	0.000149, 0.000185, 0.000229, 0.000283, 0.000351
	Black	Women	48	0.000173, 0.000214, 0.000263, 0.000324, 0.0004
	Black	Women	49	0.000197, 0.000245, 0.000304, 0.000377, 0.000469
	Black	Women	50	0.000225, 0.000281, 0.000351, 0.000438, 0.000548
	Black	Women	51	0.000262, 0.000325, 0.000403, 0.000499, 0.000619
	Black	Women	52	0.000307, 0.000376, 0.000458, 0.000559, 0.000684
	Black	Women	53	0.000351, 0.000427, 0.000518, 0.00063, 0.000766

Variable	Race/ethnicity	Sex	Age	Distribution
	Black	Women	54	0.00039, 0.000477, 0.000583, 0.000713, 0.000873
	Black	Women	55	0.000435, 0.000534, 0.000654, 0.000801, 0.000982
	Black	Women	56	0.000492, 0.0006, 0.00073, 0.000888, 0.00108
	Black	Women	57	0.000553, 0.00067, 0.00081, 0.000979, 0.00119
	Black	Women	58	0.000612, 0.00074, 0.000893, 0.00108, 0.0013
	Black	Women	59	0.000664, 0.000807, 0.000978, 0.00119, 0.00144
	Black	Women	60	0.000713, 0.000871, 0.00106, 0.00129, 0.00158
	Black	Women	61	0.000757, 0.000932, 0.00114, 0.00141, 0.00173
	Black	Women	62	0.000795, 0.000987, 0.00122, 0.00152, 0.00188
	Black	Women	63	0.000826, 0.00104, 0.0013, 0.00163, 0.00204
	Black	Women	64	0.000853, 0.00108, 0.00137, 0.00174, 0.00221
	Black	Women	65	0.000881, 0.00113, 0.00145, 0.00186, 0.0024
	Black	Women	66	0.000918, 0.00119, 0.00154, 0.002, 0.0026
	Black	Women	67	0.00097, 0.00127, 0.00166, 0.00216, 0.00283
	Black	Women	68	0.00104, 0.00137, 0.00179, 0.00234, 0.00308
	Black	Women	69	0.00114, 0.00149, 0.00195, 0.00255, 0.00334
	Black	Women	70	0.00125, 0.00164, 0.00213, 0.00278, 0.00363
	Black	Women	71	0.00138, 0.0018, 0.00233, 0.00302, 0.00393
	Black	Women	72	0.00152, 0.00197, 0.00254, 0.00328, 0.00425
	Black	Women	73	0.00166, 0.00215, 0.00277, 0.00357, 0.00461
	Black	Women	74	0.00181, 0.00234, 0.00301, 0.00389, 0.00502
	Black	Women	75	0.00197, 0.00255, 0.00329, 0.00425, 0.00549
	Black	Women	76	0.00214, 0.00278, 0.0036, 0.00467, 0.00607
	Black	Women	77	0.00231, 0.00302, 0.00395, 0.00517, 0.00677
	Black	Women	78	0.00248, 0.00329, 0.00435, 0.00575, 0.00763
	Black	Women	79	0.00265, 0.00357, 0.00479, 0.00643, 0.00865
	Black	Women	80	0.00284, 0.00387, 0.00528, 0.00719, 0.00981
	Black	Women	81	0.00304, 0.00421, 0.00581, 0.00801, 0.0111
	Black	Women	82	0.00328, 0.00458, 0.00638, 0.00888, 0.0124
	Black	Women	83	0.00355, 0.00498, 0.00698, 0.00977, 0.0137
	Black	Women	84	0.00383, 0.00541, 0.0076, 0.0107, 0.015
	Hispanic	Men	30	0.00000991, 0.0000142, 0.0000203, 0.0000291, 0.0000417
	Hispanic	Men	31	0.0000123, 0.0000173, 0.0000242, 0.0000338, 0.0000475
	Hispanic	Men	32	0.0000152, 0.000021, 0.0000287, 0.0000393, 0.0000541
	Hispanic	Men	33	0.0000189, 0.0000253, 0.000034, 0.0000455, 0.0000611
	Hispanic	Men	34	0.0000233, 0.0000305, 0.00004, 0.0000523, 0.0000686
	Hispanic	Men	35	0.0000283, 0.0000364, 0.0000467, 0.0000599, 0.0000771
	Hispanic	Men	36	0.0000336, 0.0000427, 0.0000541, 0.0000685, 0.000087
	Hispanic	Men	37	0.0000392, 0.0000495, 0.0000623, 0.0000784, 0.0000989
	Hispanic	Men	38	0.0000452, 0.0000569, 0.0000713, 0.0000894, 0.000112
	Hispanic	Men	39	0.0000521, 0.0000652, 0.0000814, 0.000102, 0.000127
	Hispanic	Men	40	0.0000604, 0.0000749, 0.0000926, 0.000115, 0.000142
	Hispanic	Men	41	0.0000705, 0.0000862, 0.000105, 0.000128, 0.000157

Variable	Race/ethnicity	Sex	Age	Distribution
	Hispanic	Men	42	0.0000823, 0.0000992, 0.000119, 0.000143, 0.000173
	Hispanic	Men	43	0.0000958, 0.000114, 0.000135, 0.000161, 0.000191
	Hispanic	Men	44	0.000111, 0.000131, 0.000154, 0.000181, 0.000213
	Hispanic	Men	45	0.000128, 0.00015, 0.000175, 0.000205, 0.00024
	Hispanic	Men	46	0.000146, 0.000171, 0.0002, 0.000235, 0.000275
	Hispanic	Men	47	0.000168, 0.000197, 0.00023, 0.000269, 0.000315
	Hispanic	Men	48	0.000197, 0.000229, 0.000265, 0.000308, 0.000358
	Hispanic	Men	49	0.000231, 0.000267, 0.000308, 0.000354, 0.000409
	Hispanic	Men	50	0.000269, 0.00031, 0.000357, 0.000411, 0.000474
	Hispanic	Men	51	0.000311, 0.000359, 0.000415, 0.000479, 0.000553
	Hispanic	Men	52	0.000356, 0.000414, 0.00048, 0.000556, 0.000646
	Hispanic	Men	53	0.000403, 0.000471, 0.000551, 0.000644, 0.000754
	Hispanic	Men	54	0.000451, 0.000532, 0.000627, 0.00074, 0.000874
	Hispanic	Men	55	0.000505, 0.000598, 0.000708, 0.000837, 0.000991
	Hispanic	Men	56	0.000568, 0.000671, 0.000791, 0.000932, 0.0011
	Hispanic	Men	57	0.000636, 0.000748, 0.000877, 0.00103, 0.00121
	Hispanic	Men	58	0.000707, 0.000827, 0.000965, 0.00113, 0.00132
	Hispanic	Men	59	0.000777, 0.000907, 0.00106, 0.00123, 0.00144
	Hispanic	Men	60	0.000845, 0.000986, 0.00115, 0.00134, 0.00156
	Hispanic	Men	61	0.00091, 0.00106, 0.00124, 0.00145, 0.00169
	Hispanic	Men	62	0.000972, 0.00114, 0.00133, 0.00155, 0.00182
	Hispanic	Men	63	0.00103, 0.00121, 0.00142, 0.00166, 0.00195
	Hispanic	Men	64	0.00109, 0.00128, 0.00151, 0.00177, 0.00207
	Hispanic	Men	65	0.00116, 0.00136, 0.0016, 0.00188, 0.00221
	Hispanic	Men	66	0.00123, 0.00145, 0.0017, 0.002, 0.00236
	Hispanic	Men	67	0.0013, 0.00154, 0.00182, 0.00215, 0.00255
	Hispanic	Men	68	0.00138, 0.00164, 0.00196, 0.00234, 0.00279
	Hispanic	Men	69	0.00147, 0.00177, 0.00212, 0.00255, 0.00307
	Hispanic	Men	70	0.00157, 0.00191, 0.00231, 0.0028, 0.00339
	Hispanic	Men	71	0.0017, 0.00207, 0.00253, 0.00308, 0.00376
	Hispanic	Men	72	0.00184, 0.00225, 0.00276, 0.00339, 0.00416
	Hispanic	Men	73	0.002, 0.00246, 0.00302, 0.00372, 0.00458
	Hispanic	Men	74	0.00218, 0.00269, 0.00331, 0.00408, 0.00503
	Hispanic	Men	75	0.0024, 0.00296, 0.00364, 0.00447, 0.00551
	Hispanic	Men	76	0.00265, 0.00326, 0.00401, 0.00492, 0.00605
	Hispanic	Men	77	0.00295, 0.00362, 0.00444, 0.00543, 0.00666
	Hispanic	Men	78	0.0033, 0.00404, 0.00493, 0.00602, 0.00736
	Hispanic	Men	79	0.00368, 0.0045, 0.00548, 0.00669, 0.00817
	Hispanic	Men	80	0.00406, 0.00498, 0.0061, 0.00746, 0.00914
	Hispanic	Men	81	0.00442, 0.00547, 0.00674, 0.00832, 0.0103
	Hispanic	Men	82	0.00474, 0.00593, 0.00741, 0.00924, 0.0116
	Hispanic	Men	83	0.00504, 0.00638, 0.00807, 0.0102, 0.0129
	Hispanic	Men	84	0.00533, 0.00683, 0.00874, 0.0112, 0.0143

Variable	Race/ethnicity	Sex	Age	Distribution
	Hispanic	Women	30	0.00000267, 0.00000395, 0.00000582, 0.00000857, 0.0000127
	Hispanic	Women	31	0.00000326, 0.0000047, 0.00000675, 0.00000969, 0.000014
	Hispanic	Women	32	0.00000397, 0.00000559, 0.00000784, 0.000011, 0.0000155
	Hispanic	Women	33	0.0000048, 0.00000662, 0.00000911, 0.0000125, 0.0000173
	Hispanic	Women	34	0.00000569, 0.00000775, 0.0000105, 0.0000143, 0.0000195
	Hispanic	Women	35	0.00000661, 0.00000896, 0.0000121, 0.0000164, 0.0000222
	Hispanic	Women	36	0.00000754, 0.0000102, 0.0000138, 0.0000187, 0.0000253
	Hispanic	Women	37	0.00000851, 0.0000116, 0.0000157, 0.0000212, 0.0000288
	Hispanic	Women	38	0.00000957, 0.000013, 0.0000177, 0.000024, 0.0000327
	Hispanic	Women	39	0.0000108, 0.0000147, 0.0000199, 0.000027, 0.0000367
	Hispanic	Women	40	0.0000123, 0.0000166, 0.0000224, 0.0000301, 0.0000407
	Hispanic	Women	41	0.0000142, 0.0000189, 0.0000251, 0.0000334, 0.0000444
	Hispanic	Women	42	0.0000167, 0.0000218, 0.0000283, 0.0000368, 0.0000479
	Hispanic	Women	43	0.0000197, 0.0000252, 0.000032, 0.0000407, 0.0000518
	Hispanic	Women	44	0.0000233, 0.0000291, 0.0000364, 0.0000454, 0.0000568
	Hispanic	Women	45	0.0000276, 0.000034, 0.0000417, 0.0000512, 0.000063
	Hispanic	Women	46	0.000033, 0.0000398, 0.000048, 0.0000579, 0.00007
	Hispanic	Women	47	0.0000396, 0.000047, 0.0000557, 0.0000659, 0.0000782
	Hispanic	Women	48	0.0000472, 0.0000554, 0.0000647, 0.0000757, 0.0000887
	Hispanic	Women	49	0.0000553, 0.0000647, 0.0000756, 0.0000883, 0.000103
	Hispanic	Women	50	0.0000636, 0.0000751, 0.0000886, 0.000104, 0.000123
	Hispanic	Women	51	0.0000724, 0.0000869, 0.000104, 0.000124, 0.000149
	Hispanic	Women	52	0.0000824, 0.0001, 0.000122, 0.000148, 0.00018
	Hispanic	Women	53	0.0000938, 0.000116, 0.000143, 0.000176, 0.000217
	Hispanic	Women	54	0.000107, 0.000134, 0.000167, 0.000208, 0.000259
	Hispanic	Women	55	0.000123, 0.000154, 0.000194, 0.000243, 0.000305
	Hispanic	Women	56	0.000141, 0.000178, 0.000224, 0.000283, 0.000357
	Hispanic	Women	57	0.000161, 0.000204, 0.000259, 0.000328, 0.000416
	Hispanic	Women	58	0.000182, 0.000233, 0.000297, 0.000379, 0.000484
	Hispanic	Women	59	0.000204, 0.000263, 0.000339, 0.000436, 0.000562
	Hispanic	Women	60	0.000227, 0.000296, 0.000384, 0.000499, 0.000649
	Hispanic	Women	61	0.000252, 0.00033, 0.000432, 0.000566, 0.000743
	Hispanic	Women	62	0.000277, 0.000366, 0.000482, 0.000635, 0.00084
	Hispanic	Women	63	0.000304, 0.000403, 0.000533, 0.000704, 0.000933
	Hispanic	Women	64	0.000335, 0.000443, 0.000585, 0.000772, 0.00102
	Hispanic	Women	65	0.00037, 0.000487, 0.00064, 0.000842, 0.00111
	Hispanic	Women	66	0.00041, 0.000537, 0.000703, 0.00092, 0.00121
	Hispanic	Women	67	0.000455, 0.000595, 0.000775, 0.00101, 0.00132
	Hispanic	Women	68	0.000507, 0.000661, 0.000861, 0.00112, 0.00146
	Hispanic	Women	69	0.000566, 0.000738, 0.00096, 0.00125, 0.00163
	Hispanic	Women	70	0.000634, 0.000826, 0.00107, 0.0014, 0.00182
	Hispanic	Women	71	0.000712, 0.000927, 0.0012, 0.00156, 0.00203
	Hispanic	Women	72	0.0008, 0.00104, 0.00135, 0.00175, 0.00228

Variable	Race/ethnicity	Sex	Age	Distribution
	Hispanic	Women	73	0.000898, 0.00117, 0.00152, 0.00197, 0.00257
	Hispanic	Women	74	0.00101, 0.00131, 0.00171, 0.00223, 0.0029
	Hispanic	Women	75	0.00113, 0.00148, 0.00193, 0.00252, 0.0033
	Hispanic	Women	76	0.00127, 0.00167, 0.00219, 0.00287, 0.00378
	Hispanic	Women	77	0.00143, 0.00189, 0.00249, 0.00329, 0.00435
	Hispanic	Women	78	0.00161, 0.00214, 0.00284, 0.00377, 0.00502
	Hispanic	Women	79	0.00181, 0.00243, 0.00324, 0.00434, 0.00582
	Hispanic	Women	80	0.00204, 0.00275, 0.00371, 0.00499, 0.00675
	Hispanic	Women	81	0.00228, 0.00311, 0.00423, 0.00575, 0.00783
	Hispanic	Women	82	0.00254, 0.0035, 0.0048, 0.0066, 0.0091
	Hispanic	Women	83	0.00279, 0.0039, 0.00542, 0.00755, 0.0105
	Hispanic	Women	84	0.00304, 0.00431, 0.00607, 0.00855, 0.0121
	White	Men	30	0.0000176, 0.0000245, 0.000034, 0.0000472, 0.0000657
	White	Men	31	0.0000227, 0.0000308, 0.0000418, 0.0000565, 0.0000768
	White	Men	32	0.0000288, 0.0000384, 0.000051, 0.0000677, 0.0000901
	White	Men	33	0.0000359, 0.0000471, 0.0000617, 0.0000807, 0.000106
	White	Men	34	0.0000435, 0.0000567, 0.0000737, 0.0000959, 0.000125
	White	Men	35	0.0000515, 0.0000671, 0.0000871, 0.000113, 0.000147
	White	Men	36	0.0000601, 0.0000783, 0.000102, 0.000132, 0.000172
	White	Men	37	0.0000699, 0.0000909, 0.000118, 0.000153, 0.000199
	White	Men	38	0.0000817, 0.000105, 0.000136, 0.000175, 0.000225
	White	Men	39	0.000096, 0.000122, 0.000155, 0.000197, 0.000251
	White	Men	40	0.000113, 0.000141, 0.000177, 0.00022, 0.000275
	White	Men	41	0.000133, 0.000163, 0.000199, 0.000244, 0.000299
	White	Men	42	0.000155, 0.000187, 0.000224, 0.000269, 0.000323
	White	Men	43	0.000179, 0.000212, 0.000251, 0.000296, 0.000351
	White	Men	44	0.000205, 0.00024, 0.000281, 0.000328, 0.000384
	White	Men	45	0.000235, 0.000272, 0.000315, 0.000364, 0.000422
	White	Men	46	0.000272, 0.000311, 0.000355, 0.000405, 0.000463
	White	Men	47	0.000314, 0.000355, 0.000401, 0.000453, 0.000513
	White	Men	48	0.000361, 0.000406, 0.000456, 0.000512, 0.000575
	White	Men	49	0.000415, 0.000464, 0.000519, 0.00058, 0.00065
	White	Men	50	0.000477, 0.000531, 0.000591, 0.000658, 0.000733
	White	Men	51	0.000551, 0.000609, 0.000672, 0.000742, 0.000819
	White	Men	52	0.00063, 0.000692, 0.000759, 0.000833, 0.000915
	White	Men	53	0.000704, 0.000775, 0.000853, 0.000938, 0.00103
	White	Men	54	0.000771, 0.000856, 0.00095, 0.00105, 0.00117
	White	Men	55	0.000837, 0.000938, 0.00105, 0.00117, 0.00131
	White	Men	56	0.000904, 0.00102, 0.00115, 0.00129, 0.00146
	White	Men	57	0.00097, 0.0011, 0.00125, 0.00142, 0.00161
	White	Men	58	0.00104, 0.00118, 0.00135, 0.00154, 0.00176
	White	Men	59	0.00111, 0.00127, 0.00146, 0.00167, 0.00191
	White	Men	60	0.00119, 0.00136, 0.00156, 0.00179, 0.00206

Variable	Race/ethnicity	Sex	Age	Distribution
	White	Men	61	0.00126, 0.00145, 0.00167, 0.00192, 0.00221
	White	Men	62	0.00134, 0.00154, 0.00178, 0.00204, 0.00235
	White	Men	63	0.00141, 0.00163, 0.00188, 0.00216, 0.0025
	White	Men	64	0.00148, 0.00171, 0.00197, 0.00228, 0.00264
	White	Men	65	0.00154, 0.00178, 0.00207, 0.0024, 0.00279
	White	Men	66	0.00159, 0.00186, 0.00218, 0.00255, 0.00298
	White	Men	67	0.00165, 0.00195, 0.00231, 0.00272, 0.00322
	White	Men	68	0.00173, 0.00206, 0.00246, 0.00293, 0.0035
	White	Men	69	0.00183, 0.0022, 0.00265, 0.00318, 0.00383
	White	Men	70	0.00196, 0.00237, 0.00287, 0.00346, 0.00418
	White	Men	71	0.00213, 0.00258, 0.00312, 0.00377, 0.00457
	White	Men	72	0.00231, 0.00281, 0.0034, 0.00411, 0.00499
	White	Men	73	0.00252, 0.00306, 0.0037, 0.00448, 0.00544
	White	Men	74	0.00275, 0.00334, 0.00404, 0.00489, 0.00594
	White	Men	75	0.003, 0.00364, 0.00442, 0.00536, 0.00651
	White	Men	76	0.00327, 0.00399, 0.00484, 0.00589, 0.00717
	White	Men	77	0.00358, 0.00438, 0.00534, 0.00652, 0.00796
	White	Men	78	0.00395, 0.00484, 0.00593, 0.00726, 0.00891
	White	Men	79	0.00438, 0.00539, 0.00662, 0.00813, 0.01
	White	Men	80	0.0049, 0.00604, 0.00743, 0.00914, 0.0113
	White	Men	81	0.00551, 0.00679, 0.00835, 0.0103, 0.0127
	White	Men	82	0.00618, 0.00761, 0.00936, 0.0115, 0.0142
	White	Men	83	0.00689, 0.00849, 0.0104, 0.0128, 0.0158
	White	Men	84	0.00762, 0.00938, 0.0115, 0.0141, 0.0174
	White	Women	30	0.00000465, 0.00000717, 0.000011, 0.0000169, 0.0000261
	White	Women	31	0.00000613, 0.00000911, 0.0000135, 0.00002, 0.0000297
	White	Women	32	0.00000807, 0.0000115, 0.0000164, 0.0000234, 0.0000335
	White	Women	33	0.0000105, 0.0000145, 0.0000199, 0.0000273, 0.0000376
	White	Women	34	0.0000133, 0.0000178, 0.0000238, 0.0000318, 0.0000426
	White	Women	35	0.0000164, 0.0000216, 0.0000282, 0.000037, 0.0000486
	White	Women	36	0.0000197, 0.0000255, 0.0000331, 0.0000428, 0.0000556
	White	Women	37	0.0000229, 0.0000297, 0.0000383, 0.0000494, 0.0000639
	White	Women	38	0.0000261, 0.0000339, 0.0000438, 0.0000567, 0.0000735
	White	Women	39	0.0000291, 0.0000381, 0.0000496, 0.0000646, 0.0000843
	White	Women	40	0.0000322, 0.0000423, 0.0000555, 0.0000728, 0.0000957
	White	Women	41	0.0000356, 0.0000469, 0.0000616, 0.000081, 0.000107
	White	Women	42	0.0000395, 0.0000519, 0.0000681, 0.0000892, 0.000117
	White	Women	43	0.0000438, 0.0000574, 0.000075, 0.000098, 0.000128
	White	Women	44	0.0000484, 0.0000634, 0.0000828, 0.000108, 0.000142
	White	Women	45	0.0000538, 0.0000703, 0.0000918, 0.00012, 0.000157
	White	Women	46	0.0000611, 0.0000791, 0.000102, 0.000132, 0.000171
	White	Women	47	0.0000714, 0.0000905, 0.000114, 0.000145, 0.000183
	White	Women	48	0.0000849, 0.000105, 0.000129, 0.000158, 0.000195

Variable	Race/ethnicity	Sex	Age	Distribution
	White	Women	49	0.000101, 0.000121, 0.000146, 0.000175, 0.00021
	White	Women	50	0.000118, 0.00014, 0.000166, 0.000196, 0.000232
	White	Women	51	0.000137, 0.000161, 0.000189, 0.000221, 0.00026
	White	Women	52	0.000156, 0.000183, 0.000215, 0.000252, 0.000295
	White	Women	53	0.000176, 0.000207, 0.000243, 0.000286, 0.000337
	White	Women	54	0.000197, 0.000232, 0.000274, 0.000324, 0.000383
	White	Women	55	0.000219, 0.00026, 0.000308, 0.000365, 0.000433
	White	Women	56	0.000244, 0.00029, 0.000345, 0.000409, 0.000486
	White	Women	57	0.000272, 0.000324, 0.000385, 0.000457, 0.000544
	White	Women	58	0.000304, 0.000362, 0.00043, 0.00051, 0.000607
	White	Women	59	0.000339, 0.000404, 0.000479, 0.000569, 0.000677
	White	Women	60	0.000375, 0.000447, 0.000532, 0.000634, 0.000756
	White	Women	61	0.000409, 0.000491, 0.000588, 0.000704, 0.000844
	White	Women	62	0.000443, 0.000534, 0.000643, 0.000775, 0.000935
	White	Women	63	0.000476, 0.000577, 0.000699, 0.000845, 0.00102
	White	Women	64	0.000511, 0.000622, 0.000755, 0.000917, 0.00112
	White	Women	65	0.000548, 0.000669, 0.000816, 0.000995, 0.00121
	White	Women	66	0.000592, 0.000724, 0.000885, 0.00108, 0.00132
	White	Women	67	0.000644, 0.00079, 0.000966, 0.00118, 0.00145
	White	Women	68	0.000707, 0.000867, 0.00106, 0.0013, 0.00159
	White	Women	69	0.000781, 0.000959, 0.00117, 0.00144, 0.00176
	White	Women	70	0.000867, 0.00106, 0.0013, 0.0016, 0.00196
	White	Women	71	0.000962, 0.00118, 0.00145, 0.00178, 0.00219
	White	Women	72	0.00107, 0.00131, 0.00162, 0.00199, 0.00245
	White	Women	73	0.00118, 0.00146, 0.0018, 0.00223, 0.00276
	White	Women	74	0.00131, 0.00162, 0.00202, 0.0025, 0.00311
	White	Women	75	0.00145, 0.00181, 0.00226, 0.00282, 0.00352
	White	Women	76	0.00161, 0.00203, 0.00254, 0.00318, 0.00399
	White	Women	77	0.00181, 0.00228, 0.00286, 0.0036, 0.00454
	White	Women	78	0.00203, 0.00257, 0.00325, 0.0041, 0.0052
	White	Women	79	0.00228, 0.00291, 0.0037, 0.0047, 0.00598
	White	Women	80	0.00258, 0.00331, 0.00423, 0.0054, 0.00691
	White	Women	81	0.00293, 0.00377, 0.00484, 0.00621, 0.00799
	White	Women	82	0.00331, 0.00429, 0.00553, 0.00713, 0.00922
	White	Women	83	0.00373, 0.00485, 0.00628, 0.00814, 0.0106
	White	Women	84	0.00417, 0.00544, 0.00708, 0.00921, 0.012
CHD mortality rates for 2028 (0.01, 0.2, 0.5, 0.8. 0.99 percentiles of the empirical distribution produced during forecasting)				
	Black	Men	30	0.0000283, 0.000041, 0.0000593, 0.0000858, 0.000125
	Black	Men	31	0.0000362, 0.0000509, 0.0000715, 0.0001, 0.000141
	Black	Men	32	0.0000458, 0.0000627, 0.0000855, 0.000117, 0.00016
	Black	Men	33	0.0000572, 0.0000762, 0.000101, 0.000135, 0.000179
	Black	Men	34	0.0000697, 0.0000911, 0.000119, 0.000155, 0.000202
	Black	Men	35	0.0000822, 0.000106, 0.000137, 0.000178, 0.00023

Variable	Race/ethnicity	Sex	Age	Distribution
	Black	Men	36	0.0000945, 0.000122, 0.000157, 0.000202, 0.000261
	Black	Men	37	0.000107, 0.000138, 0.000177, 0.000228, 0.000295
	Black	Men	38	0.000119, 0.000154, 0.000198, 0.000255, 0.000329
	Black	Men	39	0.000133, 0.000171, 0.00022, 0.000283, 0.000365
	Black	Men	40	0.000148, 0.00019, 0.000243, 0.000311, 0.0004
	Black	Men	41	0.000165, 0.00021, 0.000267, 0.00034, 0.000434
	Black	Men	42	0.000183, 0.000232, 0.000294, 0.000372, 0.000471
	Black	Men	43	0.000202, 0.000256, 0.000324, 0.000409, 0.000519
	Black	Men	44	0.000222, 0.000283, 0.000359, 0.000456, 0.00058
	Black	Men	45	0.000245, 0.000313, 0.0004, 0.000511, 0.000655
	Black	Men	46	0.000274, 0.000351, 0.00045, 0.000577, 0.00074
	Black	Men	47	0.000309, 0.000398, 0.00051, 0.000655, 0.000842
	Black	Men	48	0.000353, 0.000454, 0.000583, 0.000749, 0.000965
	Black	Men	49	0.000406, 0.000523, 0.000672, 0.000863, 0.00111
	Black	Men	50	0.000473, 0.000607, 0.000777, 0.000995, 0.00128
	Black	Men	51	0.000557, 0.000709, 0.0009, 0.00114, 0.00145
	Black	Men	52	0.000658, 0.000828, 0.00104, 0.0013, 0.00164
	Black	Men	53	0.000768, 0.000957, 0.00119, 0.00148, 0.00184
	Black	Men	54	0.000878, 0.00109, 0.00135, 0.00167, 0.00207
	Black	Men	55	0.000989, 0.00122, 0.00151, 0.00187, 0.00231
	Black	Men	56	0.00111, 0.00136, 0.00168, 0.00206, 0.00254
	Black	Men	57	0.00123, 0.00151, 0.00184, 0.00225, 0.00275
	Black	Men	58	0.00136, 0.00165, 0.00201, 0.00243, 0.00295
	Black	Men	59	0.00149, 0.0018, 0.00217, 0.00261, 0.00316
	Black	Men	60	0.00162, 0.00194, 0.00233, 0.00279, 0.00335
	Black	Men	61	0.00175, 0.00208, 0.00248, 0.00295, 0.00352
	Black	Men	62	0.00188, 0.00222, 0.00262, 0.00309, 0.00366
	Black	Men	63	0.002, 0.00234, 0.00275, 0.00322, 0.00377
	Black	Men	64	0.00212, 0.00247, 0.00287, 0.00334, 0.00389
	Black	Men	65	0.00223, 0.00258, 0.00299, 0.00347, 0.00403
	Black	Men	66	0.00233, 0.00271, 0.00314, 0.00364, 0.00422
	Black	Men	67	0.00244, 0.00284, 0.00331, 0.00385, 0.00449
	Black	Men	68	0.00256, 0.003, 0.00351, 0.00411, 0.00482
	Black	Men	69	0.0027, 0.00318, 0.00374, 0.0044, 0.00519
	Black	Men	70	0.00287, 0.00339, 0.004, 0.00472, 0.00557
	Black	Men	71	0.00307, 0.00363, 0.00428, 0.00505, 0.00597
	Black	Men	72	0.00327, 0.00387, 0.00457, 0.0054, 0.00639
	Black	Men	73	0.00348, 0.00412, 0.00488, 0.00577, 0.00684
	Black	Men	74	0.00371, 0.0044, 0.00521, 0.00617, 0.00732
	Black	Men	75	0.00399, 0.00472, 0.00558, 0.00661, 0.00783
	Black	Men	76	0.0043, 0.00509, 0.006, 0.00709, 0.00838
	Black	Men	77	0.00465, 0.0055, 0.00648, 0.00764, 0.00903
	Black	Men	78	0.00503, 0.00595, 0.00702, 0.00828, 0.00979

Variable	Race/ethnicity	Sex	Age	Distribution
	Black	Men	79	0.00543, 0.00644, 0.00762, 0.00901, 0.0107
	Black	Men	80	0.00583, 0.00695, 0.00826, 0.00982, 0.0117
	Black	Men	81	0.00623, 0.00747, 0.00894, 0.0107, 0.0128
	Black	Men	82	0.00659, 0.00797, 0.00963, 0.0116, 0.0141
	Black	Men	83	0.0069, 0.00845, 0.0103, 0.0126, 0.0154
	Black	Men	84	0.0072, 0.00891, 0.011, 0.0136, 0.0168
	Black	Women	30	0.0000119, 0.0000173, 0.0000251, 0.0000364, 0.000053
	Black	Women	31	0.0000152, 0.0000213, 0.0000297, 0.0000414, 0.000058
	Black	Women	32	0.0000189, 0.0000258, 0.000035, 0.0000476, 0.0000649
	Black	Women	33	0.0000232, 0.000031, 0.0000413, 0.000055, 0.0000735
	Black	Women	34	0.0000277, 0.0000366, 0.0000483, 0.0000637, 0.0000842
	Black	Women	35	0.000032, 0.0000424, 0.000056, 0.0000738, 0.0000977
	Black	Women	36	0.0000363, 0.0000483, 0.0000641, 0.000085, 0.000113
	Black	Women	37	0.0000409, 0.0000546, 0.0000727, 0.0000968, 0.000129
	Black	Women	38	0.0000456, 0.0000612, 0.0000818, 0.000109, 0.000147
	Black	Women	39	0.0000505, 0.0000681, 0.0000914, 0.000123, 0.000166
	Black	Women	40	0.0000562, 0.0000757, 0.000102, 0.000136, 0.000184
	Black	Women	41	0.0000635, 0.0000847, 0.000113, 0.00015, 0.000199
	Black	Women	42	0.000073, 0.0000954, 0.000124, 0.000162, 0.000212
	Black	Women	43	0.0000842, 0.000108, 0.000138, 0.000176, 0.000226
	Black	Women	44	0.0000961, 0.000122, 0.000154, 0.000194, 0.000246
	Black	Women	45	0.000109, 0.000138, 0.000173, 0.000217, 0.000274
	Black	Women	46	0.000125, 0.000157, 0.000196, 0.000245, 0.000307
	Black	Women	47	0.000145, 0.000181, 0.000224, 0.000278, 0.000346
	Black	Women	48	0.000169, 0.000209, 0.000258, 0.000319, 0.000395
	Black	Women	49	0.000193, 0.00024, 0.000299, 0.000371, 0.000463
	Black	Women	50	0.00022, 0.000276, 0.000345, 0.000432, 0.000541
	Black	Women	51	0.000256, 0.000319, 0.000396, 0.000492, 0.000612
	Black	Women	52	0.000302, 0.000369, 0.000452, 0.000552, 0.000676
	Black	Women	53	0.000345, 0.00042, 0.000511, 0.000622, 0.000759
	Black	Women	54	0.000383, 0.00047, 0.000576, 0.000705, 0.000866
	Black	Women	55	0.000428, 0.000526, 0.000646, 0.000793, 0.000975
	Black	Women	56	0.000483, 0.000591, 0.000721, 0.00088, 0.00108
	Black	Women	57	0.000544, 0.000661, 0.000801, 0.00097, 0.00118
	Black	Women	58	0.000601, 0.00073, 0.000883, 0.00107, 0.0013
	Black	Women	59	0.000653, 0.000795, 0.000967, 0.00118, 0.00143
	Black	Women	60	0.0007, 0.000858, 0.00105, 0.00128, 0.00157
	Black	Women	61	0.000743, 0.000917, 0.00113, 0.00139, 0.00172
	Black	Women	62	0.00078, 0.000971, 0.00121, 0.0015, 0.00187
	Black	Women	63	0.000809, 0.00102, 0.00128, 0.00161, 0.00202
	Black	Women	64	0.000835, 0.00106, 0.00135, 0.00172, 0.00219
	Black	Women	65	0.000861, 0.00111, 0.00143, 0.00184, 0.00237
	Black	Women	66	0.000896, 0.00117, 0.00152, 0.00197, 0.00257

Variable	Race/ethnicity	Sex	Age	Distribution
	Black	Women	67	0.000946, 0.00124, 0.00162, 0.00212, 0.00279
	Black	Women	68	0.00102, 0.00134, 0.00176, 0.0023, 0.00303
	Black	Women	69	0.00111, 0.00146, 0.00191, 0.00251, 0.0033
	Black	Women	70	0.00122, 0.0016, 0.00209, 0.00273, 0.00357
	Black	Women	71	0.00135, 0.00175, 0.00228, 0.00297, 0.00387
	Black	Women	72	0.00148, 0.00192, 0.00249, 0.00322, 0.00419
	Black	Women	73	0.00162, 0.00209, 0.00271, 0.0035, 0.00454
	Black	Women	74	0.00176, 0.00228, 0.00295, 0.00381, 0.00494
	Black	Women	75	0.00192, 0.00249, 0.00322, 0.00417, 0.00541
	Black	Women	76	0.00208, 0.00271, 0.00352, 0.00458, 0.00597
	Black	Women	77	0.00224, 0.00295, 0.00387, 0.00507, 0.00667
	Black	Women	78	0.00241, 0.00321, 0.00426, 0.00565, 0.00751
	Black	Women	79	0.00258, 0.00349, 0.00469, 0.00631, 0.00852
	Black	Women	80	0.00276, 0.00378, 0.00517, 0.00706, 0.00967
	Black	Women	81	0.00296, 0.00411, 0.00569, 0.00788, 0.0109
	Black	Women	82	0.00319, 0.00447, 0.00625, 0.00873, 0.0122
	Black	Women	83	0.00345, 0.00487, 0.00684, 0.00961, 0.0135
	Black	Women	84	0.00373, 0.00528, 0.00744, 0.0105, 0.0149
	Hispanic	Men	30	0.00000971, 0.000014, 0.0000202, 0.0000292, 0.0000422
	Hispanic	Men	31	0.0000121, 0.0000171, 0.0000241, 0.0000339, 0.0000479
	Hispanic	Men	32	0.000015, 0.0000207, 0.0000286, 0.0000394, 0.0000545
	Hispanic	Men	33	0.0000186, 0.0000251, 0.0000338, 0.0000456, 0.0000616
	Hispanic	Men	34	0.0000229, 0.0000302, 0.0000398, 0.0000524, 0.0000692
	Hispanic	Men	35	0.0000278, 0.000036, 0.0000465, 0.00006, 0.0000777
	Hispanic	Men	36	0.000033, 0.0000422, 0.0000538, 0.0000686, 0.0000877
	Hispanic	Men	37	0.0000384, 0.0000488, 0.0000618, 0.0000783, 0.0000995
	Hispanic	Men	38	0.0000442, 0.0000559, 0.0000707, 0.0000892, 0.000113
	Hispanic	Men	39	0.0000508, 0.000064, 0.0000805, 0.000101, 0.000127
	Hispanic	Men	40	0.0000588, 0.0000734, 0.0000913, 0.000114, 0.000142
	Hispanic	Men	41	0.0000685, 0.0000843, 0.000103, 0.000127, 0.000156
	Hispanic	Men	42	0.0000801, 0.0000969, 0.000117, 0.000142, 0.000171
	Hispanic	Men	43	0.0000933, 0.000111, 0.000133, 0.000158, 0.000189
	Hispanic	Men	44	0.000108, 0.000128, 0.00015, 0.000178, 0.00021
	Hispanic	Men	45	0.000124, 0.000146, 0.000171, 0.000201, 0.000237
	Hispanic	Men	46	0.000142, 0.000167, 0.000196, 0.00023, 0.00027
	Hispanic	Men	47	0.000163, 0.000192, 0.000225, 0.000264, 0.000309
	Hispanic	Men	48	0.000191, 0.000223, 0.000259, 0.000302, 0.000352
	Hispanic	Men	49	0.000225, 0.00026, 0.000301, 0.000347, 0.000402
	Hispanic	Men	50	0.000262, 0.000303, 0.00035, 0.000403, 0.000466
	Hispanic	Men	51	0.000304, 0.000352, 0.000406, 0.00047, 0.000544
	Hispanic	Men	52	0.000349, 0.000406, 0.000471, 0.000547, 0.000636
	Hispanic	Men	53	0.000395, 0.000463, 0.000542, 0.000634, 0.000744
	Hispanic	Men	54	0.000442, 0.000523, 0.000618, 0.000729, 0.000863

Variable	Race/ethnicity	Sex	Age	Distribution
	Hispanic	Men	55	0.000496, 0.000588, 0.000697, 0.000826, 0.00098
	Hispanic	Men	56	0.000558, 0.00066, 0.000779, 0.000921, 0.00109
	Hispanic	Men	57	0.000625, 0.000735, 0.000864, 0.00102, 0.0012
	Hispanic	Men	58	0.000694, 0.000813, 0.000952, 0.00111, 0.00131
	Hispanic	Men	59	0.000762, 0.000891, 0.00104, 0.00122, 0.00142
	Hispanic	Men	60	0.000827, 0.000968, 0.00113, 0.00132, 0.00155
	Hispanic	Men	61	0.00089, 0.00104, 0.00122, 0.00143, 0.00167
	Hispanic	Men	62	0.00095, 0.00112, 0.00131, 0.00153, 0.0018
	Hispanic	Men	63	0.00101, 0.00119, 0.00139, 0.00163, 0.00192
	Hispanic	Men	64	0.00107, 0.00126, 0.00148, 0.00174, 0.00204
	Hispanic	Men	65	0.00113, 0.00133, 0.00157, 0.00184, 0.00217
	Hispanic	Men	66	0.00119, 0.00141, 0.00167, 0.00197, 0.00232
	Hispanic	Men	67	0.00126, 0.0015, 0.00178, 0.00211, 0.00251
	Hispanic	Men	68	0.00134, 0.0016, 0.00192, 0.00229, 0.00274
	Hispanic	Men	69	0.00143, 0.00172, 0.00207, 0.0025, 0.00301
	Hispanic	Men	70	0.00153, 0.00186, 0.00226, 0.00274, 0.00333
	Hispanic	Men	71	0.00165, 0.00202, 0.00247, 0.00301, 0.00369
	Hispanic	Men	72	0.00178, 0.0022, 0.0027, 0.00332, 0.00408
	Hispanic	Men	73	0.00194, 0.0024, 0.00295, 0.00364, 0.0045
	Hispanic	Men	74	0.00212, 0.00262, 0.00323, 0.00399, 0.00493
	Hispanic	Men	75	0.00233, 0.00288, 0.00355, 0.00438, 0.0054
	Hispanic	Men	76	0.00258, 0.00318, 0.00391, 0.00481, 0.00593
	Hispanic	Men	77	0.00287, 0.00353, 0.00433, 0.00531, 0.00653
	Hispanic	Men	78	0.00321, 0.00393, 0.00481, 0.00589, 0.00722
	Hispanic	Men	79	0.00358, 0.00438, 0.00535, 0.00655, 0.00802
	Hispanic	Men	80	0.00395, 0.00485, 0.00595, 0.0073, 0.00897
	Hispanic	Men	81	0.00429, 0.00532, 0.00658, 0.00814, 0.0101
	Hispanic	Men	82	0.0046, 0.00578, 0.00723, 0.00905, 0.0114
	Hispanic	Men	83	0.00489, 0.00621, 0.00788, 0.00999, 0.0127
	Hispanic	Men	84	0.00517, 0.00665, 0.00853, 0.0109, 0.0141
	Hispanic	Women	30	0.00000262, 0.0000039, 0.00000579, 0.00000859, 0.0000128
	Hispanic	Women	31	0.00000321, 0.00000465, 0.00000672, 0.00000971, 0.0000141
	Hispanic	Women	32	0.00000391, 0.00000554, 0.00000782, 0.000011, 0.0000156
	Hispanic	Women	33	0.00000473, 0.00000656, 0.00000908, 0.0000126, 0.0000174
	Hispanic	Women	34	0.00000561, 0.00000769, 0.0000105, 0.0000143, 0.0000196
	Hispanic	Women	35	0.00000651, 0.00000887, 0.0000121, 0.0000164, 0.0000223
	Hispanic	Women	36	0.00000742, 0.0000101, 0.0000137, 0.0000187, 0.0000254
	Hispanic	Women	37	0.00000836, 0.0000114, 0.0000156, 0.0000212, 0.0000289
	Hispanic	Women	38	0.00000939, 0.0000128, 0.0000175, 0.0000239, 0.0000327
	Hispanic	Women	39	0.0000106, 0.0000144, 0.0000197, 0.0000268, 0.0000367
	Hispanic	Women	40	0.000012, 0.0000163, 0.0000221, 0.0000299, 0.0000405
	Hispanic	Women	41	0.0000139, 0.0000186, 0.0000247, 0.000033, 0.0000441
	Hispanic	Women	42	0.0000163, 0.0000213, 0.0000278, 0.0000362, 0.0000473

Variable	Race/ethnicity	Sex	Age	Distribution
	Hispanic	Women	43	0.0000193, 0.0000246, 0.0000313, 0.0000399, 0.000051
	Hispanic	Women	44	0.0000227, 0.0000285, 0.0000356, 0.0000446, 0.0000559
	Hispanic	Women	45	0.0000268, 0.0000331, 0.0000408, 0.0000502, 0.0000619
	Hispanic	Women	46	0.0000321, 0.0000388, 0.0000469, 0.0000567, 0.0000687
	Hispanic	Women	47	0.0000385, 0.0000458, 0.0000544, 0.0000645, 0.0000767
	Hispanic	Women	48	0.000046, 0.000054, 0.0000633, 0.0000742, 0.000087
	Hispanic	Women	49	0.0000539, 0.0000632, 0.000074, 0.0000865, 0.000101
	Hispanic	Women	50	0.0000621, 0.0000734, 0.0000868, 0.000102, 0.000121
	Hispanic	Women	51	0.0000708, 0.0000851, 0.000102, 0.000122, 0.000147
	Hispanic	Women	52	0.0000807, 0.0000984, 0.00012, 0.000146, 0.000178
	Hispanic	Women	53	0.000092, 0.000114, 0.00014, 0.000173, 0.000214
	Hispanic	Women	54	0.000105, 0.000131, 0.000164, 0.000205, 0.000256
	Hispanic	Women	55	0.000121, 0.000152, 0.000191, 0.00024, 0.000302
	Hispanic	Women	56	0.000138, 0.000175, 0.000221, 0.000279, 0.000353
	Hispanic	Women	57	0.000158, 0.000201, 0.000255, 0.000323, 0.000411
	Hispanic	Women	58	0.000179, 0.000229, 0.000293, 0.000374, 0.000479
	Hispanic	Women	59	0.0002, 0.000259, 0.000334, 0.00043, 0.000556
	Hispanic	Women	60	0.000223, 0.000291, 0.000379, 0.000492, 0.000642
	Hispanic	Women	61	0.000247, 0.000325, 0.000426, 0.000558, 0.000734
	Hispanic	Women	62	0.000271, 0.000359, 0.000474, 0.000626, 0.000828
	Hispanic	Women	63	0.000298, 0.000395, 0.000523, 0.000692, 0.000919
	Hispanic	Women	64	0.000327, 0.000434, 0.000573, 0.000758, 0.00101
	Hispanic	Women	65	0.000361, 0.000476, 0.000627, 0.000826, 0.00109
	Hispanic	Women	66	0.0004, 0.000525, 0.000688, 0.000901, 0.00118
	Hispanic	Women	67	0.000444, 0.000581, 0.000758, 0.00099, 0.0013
	Hispanic	Women	68	0.000494, 0.000645, 0.000841, 0.0011, 0.00143
	Hispanic	Women	69	0.000551, 0.00072, 0.000938, 0.00122, 0.0016
	Hispanic	Women	70	0.000617, 0.000806, 0.00105, 0.00137, 0.00178
	Hispanic	Women	71	0.000693, 0.000904, 0.00118, 0.00153, 0.002
	Hispanic	Women	72	0.000778, 0.00101, 0.00132, 0.00172, 0.00224
	Hispanic	Women	73	0.000873, 0.00114, 0.00148, 0.00193, 0.00252
	Hispanic	Women	74	0.00098, 0.00128, 0.00167, 0.00218, 0.00284
	Hispanic	Women	75	0.0011, 0.00144, 0.00189, 0.00247, 0.00323
	Hispanic	Women	76	0.00123, 0.00163, 0.00214, 0.00281, 0.0037
	Hispanic	Women	77	0.00139, 0.00184, 0.00243, 0.00321, 0.00426
	Hispanic	Women	78	0.00156, 0.00208, 0.00277, 0.00369, 0.00492
	Hispanic	Women	79	0.00176, 0.00236, 0.00316, 0.00424, 0.0057
	Hispanic	Women	80	0.00198, 0.00268, 0.00362, 0.00488, 0.00661
	Hispanic	Women	81	0.00222, 0.00303, 0.00413, 0.00562, 0.00767
	Hispanic	Women	82	0.00247, 0.00341, 0.00469, 0.00645, 0.0089
	Hispanic	Women	83	0.00271, 0.00379, 0.00529, 0.00737, 0.0103
	Hispanic	Women	84	0.00296, 0.00419, 0.00592, 0.00835, 0.0118
	White	Men	30	0.0000172, 0.0000241, 0.0000338, 0.0000474, 0.0000666

Variable	Race/ethnicity	Sex	Age	Distribution
	White	Men	31	0.0000223, 0.0000305, 0.0000416, 0.0000568, 0.0000777
	White	Men	32	0.0000284, 0.000038, 0.0000508, 0.0000679, 0.0000911
	White	Men	33	0.0000353, 0.0000467, 0.0000615, 0.000081, 0.000107
	White	Men	34	0.0000429, 0.0000562, 0.0000735, 0.0000962, 0.000126
	White	Men	35	0.0000507, 0.0000664, 0.0000868, 0.000113, 0.000149
	White	Men	36	0.0000591, 0.0000774, 0.000101, 0.000132, 0.000174
	White	Men	37	0.0000686, 0.0000897, 0.000117, 0.000153, 0.0002
	White	Men	38	0.00008, 0.000104, 0.000134, 0.000174, 0.000226
	White	Men	39	0.0000937, 0.00012, 0.000154, 0.000196, 0.000252
	White	Men	40	0.00011, 0.000139, 0.000174, 0.000219, 0.000275
	White	Men	41	0.000129, 0.00016, 0.000196, 0.000241, 0.000298
	White	Men	42	0.000151, 0.000183, 0.00022, 0.000265, 0.00032
	White	Men	43	0.000174, 0.000207, 0.000246, 0.000292, 0.000346
	White	Men	44	0.000199, 0.000234, 0.000275, 0.000322, 0.000379
	White	Men	45	0.000229, 0.000265, 0.000308, 0.000357, 0.000415
	White	Men	46	0.000264, 0.000303, 0.000347, 0.000397, 0.000456
	White	Men	47	0.000305, 0.000346, 0.000392, 0.000444, 0.000504
	White	Men	48	0.000351, 0.000396, 0.000446, 0.000501, 0.000565
	White	Men	49	0.000404, 0.000453, 0.000508, 0.000569, 0.000639
	White	Men	50	0.000465, 0.000519, 0.000579, 0.000646, 0.000721
	White	Men	51	0.000537, 0.000595, 0.000659, 0.000729, 0.000807
	White	Men	52	0.000616, 0.000678, 0.000746, 0.00082, 0.000903
	White	Men	53	0.000689, 0.00076, 0.000838, 0.000924, 0.00102
	White	Men	54	0.000755, 0.00084, 0.000935, 0.00104, 0.00116
	White	Men	55	0.00082, 0.000921, 0.00103, 0.00116, 0.0013
	White	Men	56	0.000886, 0.001, 0.00113, 0.00128, 0.00145
	White	Men	57	0.00095, 0.00108, 0.00123, 0.0014, 0.0016
	White	Men	58	0.00102, 0.00116, 0.00133, 0.00152, 0.00175
	White	Men	59	0.00109, 0.00125, 0.00144, 0.00165, 0.0019
	White	Men	60	0.00116, 0.00134, 0.00154, 0.00177, 0.00204
	White	Men	61	0.00124, 0.00143, 0.00164, 0.0019, 0.00219
	White	Men	62	0.00131, 0.00151, 0.00175, 0.00202, 0.00233
	White	Men	63	0.00138, 0.00159, 0.00184, 0.00213, 0.00247
	White	Men	64	0.00144, 0.00167, 0.00193, 0.00224, 0.0026
	White	Men	65	0.0015, 0.00174, 0.00203, 0.00236, 0.00275
	White	Men	66	0.00155, 0.00182, 0.00213, 0.0025, 0.00293
	White	Men	67	0.00161, 0.0019, 0.00225, 0.00267, 0.00316
	White	Men	68	0.00168, 0.00201, 0.0024, 0.00287, 0.00344
	White	Men	69	0.00178, 0.00215, 0.00259, 0.00312, 0.00376
	White	Men	70	0.00191, 0.00231, 0.0028, 0.00339, 0.00411
	White	Men	71	0.00207, 0.00251, 0.00304, 0.00369, 0.00448
	White	Men	72	0.00225, 0.00273, 0.00332, 0.00402, 0.00489
	White	Men	73	0.00245, 0.00298, 0.00362, 0.00439, 0.00534

Variable	Race/ethnicity	Sex	Age	Distribution
	White	Men	74	0.00267, 0.00325, 0.00394, 0.00479, 0.00583
	White	Men	75	0.00291, 0.00355, 0.00431, 0.00524, 0.00638
	White	Men	76	0.00318, 0.00388, 0.00473, 0.00576, 0.00703
	White	Men	77	0.00348, 0.00426, 0.00521, 0.00637, 0.00781
	White	Men	78	0.00383, 0.00471, 0.00578, 0.0071, 0.00873
	White	Men	79	0.00425, 0.00525, 0.00646, 0.00796, 0.00981
	White	Men	80	0.00476, 0.00588, 0.00725, 0.00894, 0.011
	White	Men	81	0.00534, 0.0066, 0.00815, 0.01, 0.0124
	White	Men	82	0.00599, 0.00741, 0.00913, 0.0113, 0.0139
	White	Men	83	0.00668, 0.00825, 0.0102, 0.0125, 0.0155
	White	Men	84	0.00738, 0.00911, 0.0112, 0.0138, 0.0171
	White	Women	30	0.00000457, 0.0000071, 0.000011, 0.000017, 0.0000263
	White	Women	31	0.00000603, 0.00000902, 0.0000134, 0.00002, 0.0000299
	White	Women	32	0.00000796, 0.0000114, 0.0000164, 0.0000235, 0.0000337
	White	Women	33	0.0000104, 0.0000144, 0.0000198, 0.0000274, 0.0000379
	White	Women	34	0.0000131, 0.0000177, 0.0000238, 0.0000319, 0.0000429
	White	Women	35	0.0000162, 0.0000214, 0.0000281, 0.0000371, 0.000049
	White	Women	36	0.0000193, 0.0000253, 0.0000329, 0.0000429, 0.000056
	White	Women	37	0.0000225, 0.0000293, 0.000038, 0.0000494, 0.0000643
	White	Women	38	0.0000256, 0.0000334, 0.0000434, 0.0000565, 0.0000738
	White	Women	39	0.0000285, 0.0000374, 0.000049, 0.0000642, 0.0000844
	White	Women	40	0.0000314, 0.0000416, 0.0000548, 0.0000722, 0.0000955
	White	Women	41	0.0000347, 0.000046, 0.0000607, 0.0000801, 0.000106
	White	Women	42	0.0000386, 0.0000508, 0.0000668, 0.0000879, 0.000116
	White	Women	43	0.0000427, 0.0000561, 0.0000735, 0.0000963, 0.000126
	White	Women	44	0.0000471, 0.0000619, 0.000081, 0.000106, 0.000139
	White	Women	45	0.0000523, 0.0000686, 0.0000897, 0.000117, 0.000154
	White	Women	46	0.0000595, 0.0000772, 0.0000998, 0.000129, 0.000167
	White	Women	47	0.0000695, 0.0000882, 0.000112, 0.000141, 0.000179
	White	Women	48	0.0000827, 0.000102, 0.000126, 0.000155, 0.000191
	White	Women	49	0.0000984, 0.000119, 0.000142, 0.000171, 0.000206
	White	Women	50	0.000116, 0.000137, 0.000162, 0.000192, 0.000227
	White	Women	51	0.000134, 0.000157, 0.000185, 0.000217, 0.000256
	White	Women	52	0.000153, 0.000179, 0.000211, 0.000247, 0.000291
	White	Women	53	0.000172, 0.000203, 0.000239, 0.000281, 0.000332
	White	Women	54	0.000193, 0.000228, 0.00027, 0.000319, 0.000378
	White	Women	55	0.000215, 0.000255, 0.000303, 0.00036, 0.000428
	White	Women	56	0.000239, 0.000285, 0.000339, 0.000404, 0.000481
	White	Women	57	0.000267, 0.000318, 0.000379, 0.000451, 0.000538
	White	Women	58	0.000298, 0.000356, 0.000423, 0.000504, 0.000601
	White	Women	59	0.000333, 0.000397, 0.000472, 0.000562, 0.00067
	White	Women	60	0.000367, 0.000439, 0.000524, 0.000626, 0.000749
	White	Women	61	0.0004, 0.000482, 0.000578, 0.000694, 0.000835

Variable	Race/ethnicity	Sex	Age	Distribution
	White	Women	62	0.000433, 0.000524, 0.000632, 0.000764, 0.000924
	White	Women	63	0.000465, 0.000565, 0.000686, 0.000832, 0.00101
	White	Women	64	0.000499, 0.000608, 0.00074, 0.000901, 0.0011
	White	Women	65	0.000535, 0.000654, 0.000799, 0.000976, 0.00119
	White	Women	66	0.000577, 0.000707, 0.000866, 0.00106, 0.0013
	White	Women	67	0.000627, 0.00077, 0.000944, 0.00116, 0.00142
	White	Women	68	0.000688, 0.000846, 0.00104, 0.00127, 0.00156
	White	Women	69	0.00076, 0.000935, 0.00115, 0.00141, 0.00173
	White	Women	70	0.000843, 0.00104, 0.00127, 0.00156, 0.00192
	White	Women	71	0.000935, 0.00115, 0.00142, 0.00174, 0.00215
	White	Women	72	0.00104, 0.00128, 0.00158, 0.00195, 0.00241
	White	Women	73	0.00115, 0.00142, 0.00176, 0.00218, 0.00271
	White	Women	74	0.00127, 0.00158, 0.00197, 0.00245, 0.00305
	White	Women	75	0.00141, 0.00176, 0.0022, 0.00275, 0.00345
	White	Women	76	0.00157, 0.00197, 0.00248, 0.00311, 0.00391
	White	Women	77	0.00175, 0.00222, 0.00279, 0.00352, 0.00445
	White	Women	78	0.00197, 0.0025, 0.00317, 0.00401, 0.00509
	White	Women	79	0.00222, 0.00283, 0.00361, 0.00459, 0.00586
	White	Women	80	0.00251, 0.00322, 0.00412, 0.00527, 0.00677
	White	Women	81	0.00285, 0.00367, 0.00472, 0.00607, 0.00783
	White	Women	82	0.00322, 0.00417, 0.00539, 0.00697, 0.00903
	White	Women	83	0.00362, 0.00471, 0.00613, 0.00796, 0.0104
	White	Women	84	0.00404, 0.00529, 0.0069, 0.009, 0.0118
CHD mortality rates for 2029 (0.01, 0.2, 0.5, 0.8. 0.99 percentiles of the empirical distribution produced during forecasting)				
	Black	Men	30	0.0000277, 0.0000405, 0.000059, 0.0000861, 0.000126
	Black	Men	31	0.0000355, 0.0000503, 0.0000711, 0.000101, 0.000143
	Black	Men	32	0.000045, 0.000062, 0.0000851, 0.000117, 0.000161
	Black	Men	33	0.0000563, 0.0000754, 0.000101, 0.000135, 0.000181
	Black	Men	34	0.0000685, 0.0000901, 0.000118, 0.000155, 0.000204
	Black	Men	35	0.0000808, 0.000105, 0.000137, 0.000178, 0.000232
	Black	Men	36	0.0000927, 0.00012, 0.000156, 0.000202, 0.000263
	Black	Men	37	0.000105, 0.000136, 0.000176, 0.000228, 0.000296
	Black	Men	38	0.000117, 0.000152, 0.000197, 0.000255, 0.000331
	Black	Men	39	0.00013, 0.000168, 0.000218, 0.000282, 0.000365
	Black	Men	40	0.000144, 0.000186, 0.00024, 0.000309, 0.000399
	Black	Men	41	0.00016, 0.000206, 0.000263, 0.000336, 0.000431
	Black	Men	42	0.000178, 0.000227, 0.000288, 0.000366, 0.000466
	Black	Men	43	0.000197, 0.00025, 0.000317, 0.000402, 0.000511
	Black	Men	44	0.000216, 0.000276, 0.000351, 0.000447, 0.000571
	Black	Men	45	0.000238, 0.000306, 0.000391, 0.000501, 0.000643
	Black	Men	46	0.000266, 0.000343, 0.00044, 0.000564, 0.000726
	Black	Men	47	0.000301, 0.000388, 0.000498, 0.00064, 0.000825
	Black	Men	48	0.000344, 0.000443, 0.00057, 0.000733, 0.000945

Variable	Race/ethnicity	Sex	Age	Distribution
	Black	Men	49	0.000396, 0.000511, 0.000657, 0.000845, 0.00109
	Black	Men	50	0.000462, 0.000594, 0.000761, 0.000976, 0.00125
	Black	Men	51	0.000545, 0.000695, 0.000883, 0.00112, 0.00143
	Black	Men	52	0.000645, 0.000812, 0.00102, 0.00128, 0.00161
	Black	Men	53	0.000753, 0.00094, 0.00117, 0.00146, 0.00182
	Black	Men	54	0.000861, 0.00107, 0.00133, 0.00165, 0.00205
	Black	Men	55	0.000971, 0.0012, 0.00149, 0.00184, 0.00229
	Black	Men	56	0.00109, 0.00134, 0.00165, 0.00204, 0.00251
	Black	Men	57	0.00121, 0.00148, 0.00182, 0.00222, 0.00272
	Black	Men	58	0.00134, 0.00163, 0.00198, 0.0024, 0.00292
	Black	Men	59	0.00146, 0.00177, 0.00214, 0.00258, 0.00313
	Black	Men	60	0.00159, 0.00191, 0.00229, 0.00276, 0.00332
	Black	Men	61	0.00171, 0.00205, 0.00244, 0.00291, 0.00348
	Black	Men	62	0.00183, 0.00217, 0.00257, 0.00305, 0.00362
	Black	Men	63	0.00195, 0.0023, 0.0027, 0.00317, 0.00373
	Black	Men	64	0.00206, 0.00241, 0.00281, 0.00328, 0.00383
	Black	Men	65	0.00217, 0.00253, 0.00293, 0.00341, 0.00397
	Black	Men	66	0.00227, 0.00264, 0.00307, 0.00357, 0.00415
	Black	Men	67	0.00237, 0.00277, 0.00324, 0.00378, 0.00441
	Black	Men	68	0.00249, 0.00293, 0.00343, 0.00403, 0.00474
	Black	Men	69	0.00263, 0.0031, 0.00366, 0.00432, 0.0051
	Black	Men	70	0.00279, 0.00331, 0.00391, 0.00463, 0.00548
	Black	Men	71	0.00298, 0.00353, 0.00418, 0.00495, 0.00587
	Black	Men	72	0.00317, 0.00377, 0.00446, 0.00529, 0.00628
	Black	Men	73	0.00338, 0.00402, 0.00476, 0.00565, 0.00672
	Black	Men	74	0.0036, 0.00429, 0.00509, 0.00604, 0.00718
	Black	Men	75	0.00387, 0.00459, 0.00545, 0.00646, 0.00768
	Black	Men	76	0.00417, 0.00495, 0.00586, 0.00693, 0.00822
	Black	Men	77	0.00451, 0.00535, 0.00632, 0.00748, 0.00886
	Black	Men	78	0.00488, 0.00579, 0.00685, 0.00811, 0.00961
	Black	Men	79	0.00526, 0.00626, 0.00743, 0.00882, 0.0105
	Black	Men	80	0.00566, 0.00676, 0.00806, 0.00962, 0.0115
	Black	Men	81	0.00604, 0.00726, 0.00872, 0.0105, 0.0126
	Black	Men	82	0.00638, 0.00775, 0.0094, 0.0114, 0.0138
	Black	Men	83	0.00669, 0.00822, 0.0101, 0.0123, 0.0152
	Black	Men	84	0.00698, 0.00866, 0.0107, 0.0133, 0.0165
	Black	Women	30	0.0000117, 0.0000171, 0.000025, 0.0000365, 0.0000535
	Black	Women	31	0.0000149, 0.000021, 0.0000296, 0.0000415, 0.0000586
	Black	Women	32	0.0000186, 0.0000255, 0.0000349, 0.0000478, 0.0000655
	Black	Women	33	0.0000228, 0.0000307, 0.0000412, 0.0000552, 0.0000741
	Black	Women	34	0.0000273, 0.0000363, 0.0000482, 0.0000639, 0.000085
	Black	Women	35	0.0000316, 0.000042, 0.0000558, 0.000074, 0.0000985
	Black	Women	36	0.0000357, 0.0000478, 0.0000638, 0.0000851, 0.000114

Variable	Race/ethnicity	Sex	Age	Distribution
	Black	Women	37	0.0000402, 0.000054, 0.0000723, 0.0000968, 0.00013
	Black	Women	38	0.0000447, 0.0000603, 0.0000812, 0.000109, 0.000147
	Black	Women	39	0.0000495, 0.000067, 0.0000906, 0.000122, 0.000166
	Black	Women	40	0.000055, 0.0000745, 0.0001, 0.000136, 0.000184
	Black	Women	41	0.0000621, 0.0000831, 0.000111, 0.000148, 0.000199
	Black	Women	42	0.0000713, 0.0000936, 0.000123, 0.00016, 0.000211
	Black	Women	43	0.0000822, 0.000106, 0.000136, 0.000174, 0.000224
	Black	Women	44	0.0000938, 0.000119, 0.000151, 0.000191, 0.000243
	Black	Women	45	0.000106, 0.000134, 0.00017, 0.000214, 0.00027
	Black	Women	46	0.000122, 0.000153, 0.000192, 0.000241, 0.000303
	Black	Women	47	0.000142, 0.000177, 0.00022, 0.000273, 0.000341
	Black	Women	48	0.000165, 0.000205, 0.000253, 0.000313, 0.000389
	Black	Women	49	0.000188, 0.000235, 0.000293, 0.000365, 0.000456
	Black	Women	50	0.000215, 0.00027, 0.000339, 0.000425, 0.000534
	Black	Women	51	0.000251, 0.000313, 0.00039, 0.000485, 0.000604
	Black	Women	52	0.000296, 0.000363, 0.000445, 0.000545, 0.000669
	Black	Women	53	0.000338, 0.000413, 0.000504, 0.000615, 0.000751
	Black	Women	54	0.000376, 0.000463, 0.000568, 0.000698, 0.000858
	Black	Women	55	0.00042, 0.000518, 0.000638, 0.000785, 0.000967
	Black	Women	56	0.000475, 0.000583, 0.000712, 0.000871, 0.00107
	Black	Women	57	0.000535, 0.000651, 0.000791, 0.000961, 0.00117
	Black	Women	58	0.000591, 0.000719, 0.000873, 0.00106, 0.00129
	Black	Women	59	0.000642, 0.000784, 0.000955, 0.00116, 0.00142
	Black	Women	60	0.000688, 0.000845, 0.00104, 0.00127, 0.00156
	Black	Women	61	0.000729, 0.000903, 0.00112, 0.00138, 0.00171
	Black	Women	62	0.000765, 0.000955, 0.00119, 0.00148, 0.00185
	Black	Women	63	0.000793, 0.001, 0.00126, 0.00159, 0.002
	Black	Women	64	0.000817, 0.00104, 0.00133, 0.00169, 0.00216
	Black	Women	65	0.000842, 0.00109, 0.0014, 0.00181, 0.00234
	Black	Women	66	0.000875, 0.00114, 0.00149, 0.00194, 0.00253
	Black	Women	67	0.000924, 0.00121, 0.00159, 0.00209, 0.00275
	Black	Women	68	0.000992, 0.00131, 0.00172, 0.00226, 0.00299
	Black	Women	69	0.00108, 0.00142, 0.00187, 0.00246, 0.00325
	Black	Women	70	0.00119, 0.00156, 0.00205, 0.00268, 0.00352
	Black	Women	71	0.00131, 0.00171, 0.00223, 0.00291, 0.00381
	Black	Women	72	0.00144, 0.00188, 0.00244, 0.00316, 0.00412
	Black	Women	73	0.00157, 0.00205, 0.00265, 0.00344, 0.00447
	Black	Women	74	0.00171, 0.00223, 0.00289, 0.00374, 0.00486
	Black	Women	75	0.00187, 0.00243, 0.00315, 0.00409, 0.00532
	Black	Women	76	0.00202, 0.00265, 0.00345, 0.00449, 0.00587
	Black	Women	77	0.00218, 0.00288, 0.00379, 0.00498, 0.00656
	Black	Women	78	0.00235, 0.00313, 0.00417, 0.00554, 0.00739
	Black	Women	79	0.00251, 0.0034, 0.00459, 0.00619, 0.00838

Variable	Race/ethnicity	Sex	Age	Distribution
	Black	Women	80	0.00269, 0.00369, 0.00506, 0.00693, 0.00953
	Black	Women	81	0.00288, 0.00401, 0.00557, 0.00773, 0.0108
	Black	Women	82	0.00311, 0.00437, 0.00612, 0.00858, 0.0121
	Black	Women	83	0.00336, 0.00475, 0.0067, 0.00944, 0.0134
	Black	Women	84	0.00363, 0.00515, 0.00729, 0.0103, 0.0147
	Hispanic	Men	30	0.00000952, 0.0000139, 0.0000201, 0.0000292, 0.0000426
	Hispanic	Men	31	0.0000119, 0.0000169, 0.000024, 0.000034, 0.0000483
	Hispanic	Men	32	0.0000148, 0.0000205, 0.0000285, 0.0000395, 0.000055
	Hispanic	Men	33	0.0000183, 0.0000249, 0.0000337, 0.0000457, 0.0000621
	Hispanic	Men	34	0.0000226, 0.00003, 0.0000397, 0.0000525, 0.0000697
	Hispanic	Men	35	0.0000273, 0.0000356, 0.0000463, 0.0000601, 0.0000783
	Hispanic	Men	36	0.0000324, 0.0000417, 0.0000535, 0.0000687, 0.0000884
	Hispanic	Men	37	0.0000376, 0.0000481, 0.0000614, 0.0000783, 0.0001
	Hispanic	Men	38	0.0000432, 0.0000551, 0.00007, 0.000089, 0.000113
	Hispanic	Men	39	0.0000496, 0.0000629, 0.0000795, 0.000101, 0.000128
	Hispanic	Men	40	0.0000573, 0.0000719, 0.0000901, 0.000113, 0.000142
	Hispanic	Men	41	0.0000667, 0.0000825, 0.000102, 0.000126, 0.000156
	Hispanic	Men	42	0.000078, 0.0000948, 0.000115, 0.00014, 0.00017
	Hispanic	Men	43	0.0000908, 0.000109, 0.00013, 0.000155, 0.000186
	Hispanic	Men	44	0.000105, 0.000124, 0.000147, 0.000174, 0.000207
	Hispanic	Men	45	0.000121, 0.000142, 0.000167, 0.000197, 0.000233
	Hispanic	Men	46	0.000138, 0.000162, 0.000191, 0.000225, 0.000265
	Hispanic	Men	47	0.000159, 0.000187, 0.00022, 0.000258, 0.000304
	Hispanic	Men	48	0.000186, 0.000217, 0.000254, 0.000296, 0.000345
	Hispanic	Men	49	0.000219, 0.000254, 0.000294, 0.00034, 0.000394
	Hispanic	Men	50	0.000256, 0.000296, 0.000342, 0.000396, 0.000458
	Hispanic	Men	51	0.000297, 0.000344, 0.000399, 0.000462, 0.000536
	Hispanic	Men	52	0.000341, 0.000398, 0.000462, 0.000538, 0.000626
	Hispanic	Men	53	0.000387, 0.000454, 0.000533, 0.000624, 0.000733
	Hispanic	Men	54	0.000433, 0.000514, 0.000608, 0.000719, 0.000853
	Hispanic	Men	55	0.000487, 0.000579, 0.000687, 0.000815, 0.000969
	Hispanic	Men	56	0.000547, 0.000649, 0.000768, 0.000909, 0.00108
	Hispanic	Men	57	0.000613, 0.000723, 0.000852, 0.001, 0.00118
	Hispanic	Men	58	0.00068, 0.0008, 0.000939, 0.0011, 0.00129
	Hispanic	Men	59	0.000747, 0.000876, 0.00103, 0.0012, 0.00141
	Hispanic	Men	60	0.00081, 0.000951, 0.00111, 0.00131, 0.00153
	Hispanic	Men	61	0.000871, 0.00102, 0.0012, 0.00141, 0.00166
	Hispanic	Men	62	0.000929, 0.00109, 0.00129, 0.00151, 0.00178
	Hispanic	Men	63	0.000985, 0.00116, 0.00137, 0.00161, 0.0019
	Hispanic	Men	64	0.00104, 0.00123, 0.00145, 0.00171, 0.00202
	Hispanic	Men	65	0.0011, 0.0013, 0.00153, 0.00181, 0.00214
	Hispanic	Men	66	0.00116, 0.00138, 0.00163, 0.00193, 0.00228
	Hispanic	Men	67	0.00123, 0.00146, 0.00174, 0.00207, 0.00246

Variable	Race/ethnicity	Sex	Age	Distribution
	Hispanic	Men	68	0.0013, 0.00156, 0.00187, 0.00224, 0.00269
	Hispanic	Men	69	0.00139, 0.00168, 0.00203, 0.00245, 0.00296
	Hispanic	Men	70	0.00149, 0.00182, 0.00221, 0.00268, 0.00327
	Hispanic	Men	71	0.00161, 0.00197, 0.00241, 0.00295, 0.00362
	Hispanic	Men	72	0.00174, 0.00214, 0.00264, 0.00325, 0.004
	Hispanic	Men	73	0.00189, 0.00233, 0.00288, 0.00356, 0.00441
	Hispanic	Men	74	0.00206, 0.00255, 0.00316, 0.0039, 0.00484
	Hispanic	Men	75	0.00227, 0.0028, 0.00346, 0.00428, 0.0053
	Hispanic	Men	76	0.0025, 0.00309, 0.00382, 0.0047, 0.00581
	Hispanic	Men	77	0.00279, 0.00343, 0.00422, 0.00519, 0.0064
	Hispanic	Men	78	0.00311, 0.00383, 0.00469, 0.00576, 0.00708
	Hispanic	Men	79	0.00347, 0.00426, 0.00523, 0.00641, 0.00787
	Hispanic	Men	80	0.00383, 0.00472, 0.00581, 0.00714, 0.0088
	Hispanic	Men	81	0.00417, 0.00518, 0.00643, 0.00797, 0.0099
	Hispanic	Men	82	0.00447, 0.00562, 0.00706, 0.00886, 0.0111
	Hispanic	Men	83	0.00475, 0.00605, 0.00769, 0.00978, 0.0125
	Hispanic	Men	84	0.00502, 0.00647, 0.00832, 0.0107, 0.0138
	Hispanic	Women	30	0.00000257, 0.00000386, 0.00000577, 0.00000862, 0.0000129
	Hispanic	Women	31	0.00000316, 0.00000461, 0.0000067, 0.00000974, 0.0000142
	Hispanic	Women	32	0.00000386, 0.00000549, 0.00000779, 0.0000111, 0.0000157
	Hispanic	Women	33	0.00000467, 0.00000651, 0.00000905, 0.0000126, 0.0000176
	Hispanic	Women	34	0.00000554, 0.00000762, 0.0000105, 0.0000144, 0.0000198
	Hispanic	Women	35	0.00000642, 0.00000879, 0.000012, 0.0000164, 0.0000225
	Hispanic	Women	36	0.0000073, 0.00001, 0.0000137, 0.0000187, 0.0000256
	Hispanic	Women	37	0.00000822, 0.0000113, 0.0000154, 0.0000211, 0.000029
	Hispanic	Women	38	0.00000921, 0.0000127, 0.0000174, 0.0000238, 0.0000328
	Hispanic	Women	39	0.0000103, 0.0000142, 0.0000195, 0.0000267, 0.0000366
	Hispanic	Women	40	0.0000117, 0.000016, 0.0000218, 0.0000296, 0.0000404
	Hispanic	Women	41	0.0000135, 0.0000182, 0.0000243, 0.0000326, 0.0000437
	Hispanic	Women	42	0.0000159, 0.0000209, 0.0000273, 0.0000357, 0.0000468
	Hispanic	Women	43	0.0000188, 0.000024, 0.0000307, 0.0000393, 0.0000503
	Hispanic	Women	44	0.0000221, 0.0000278, 0.0000348, 0.0000437, 0.000055
	Hispanic	Women	45	0.0000261, 0.0000323, 0.0000398, 0.0000492, 0.0000608
	Hispanic	Women	46	0.0000312, 0.0000379, 0.0000459, 0.0000556, 0.0000674
	Hispanic	Women	47	0.0000375, 0.0000447, 0.0000531, 0.0000632, 0.0000753
	Hispanic	Women	48	0.0000448, 0.0000527, 0.0000618, 0.0000726, 0.0000854
	Hispanic	Women	49	0.0000525, 0.0000617, 0.0000723, 0.0000848, 0.0000996
	Hispanic	Women	50	0.0000606, 0.0000718, 0.0000849, 0.0001, 0.000119
	Hispanic	Women	51	0.0000693, 0.0000833, 0.0000999, 0.00012, 0.000144
	Hispanic	Women	52	0.0000791, 0.0000965, 0.000118, 0.000143, 0.000175
	Hispanic	Women	53	0.0000902, 0.000112, 0.000138, 0.00017, 0.000211
	Hispanic	Women	54	0.000103, 0.000129, 0.000161, 0.000202, 0.000252
	Hispanic	Women	55	0.000118, 0.000149, 0.000188, 0.000236, 0.000298

Variable	Race/ethnicity	Sex	Age	Distribution
	Hispanic	Women	56	0.000136, 0.000172, 0.000218, 0.000275, 0.000349
	Hispanic	Women	57	0.000155, 0.000198, 0.000251, 0.000319, 0.000407
	Hispanic	Women	58	0.000176, 0.000225, 0.000288, 0.000369, 0.000474
	Hispanic	Women	59	0.000197, 0.000255, 0.000329, 0.000425, 0.00055
	Hispanic	Women	60	0.000219, 0.000286, 0.000373, 0.000486, 0.000635
	Hispanic	Women	61	0.000242, 0.000319, 0.000419, 0.000551, 0.000725
	Hispanic	Women	62	0.000266, 0.000352, 0.000466, 0.000616, 0.000817
	Hispanic	Women	63	0.000291, 0.000387, 0.000514, 0.000681, 0.000905
	Hispanic	Women	64	0.00032, 0.000425, 0.000562, 0.000744, 0.000989
	Hispanic	Women	65	0.000352, 0.000466, 0.000614, 0.00081, 0.00107
	Hispanic	Women	66	0.00039, 0.000513, 0.000673, 0.000883, 0.00116
	Hispanic	Women	67	0.000432, 0.000567, 0.000741, 0.00097, 0.00127
	Hispanic	Women	68	0.000481, 0.00063, 0.000822, 0.00107, 0.00141
	Hispanic	Women	69	0.000537, 0.000702, 0.000917, 0.0012, 0.00157
	Hispanic	Women	70	0.000601, 0.000786, 0.00103, 0.00134, 0.00175
	Hispanic	Women	71	0.000675, 0.000882, 0.00115, 0.0015, 0.00196
	Hispanic	Women	72	0.000757, 0.000989, 0.00129, 0.00168, 0.00219
	Hispanic	Women	73	0.00085, 0.00111, 0.00145, 0.00189, 0.00246
	Hispanic	Women	74	0.000953, 0.00125, 0.00163, 0.00213, 0.00278
	Hispanic	Women	75	0.00107, 0.0014, 0.00184, 0.00241, 0.00316
	Hispanic	Women	76	0.0012, 0.00158, 0.00208, 0.00274, 0.00362
	Hispanic	Women	77	0.00135, 0.00179, 0.00237, 0.00314, 0.00416
	Hispanic	Women	78	0.00152, 0.00203, 0.0027, 0.0036, 0.00481
	Hispanic	Women	79	0.00171, 0.0023, 0.00309, 0.00414, 0.00557
	Hispanic	Women	80	0.00192, 0.00261, 0.00353, 0.00477, 0.00647
	Hispanic	Women	81	0.00216, 0.00295, 0.00402, 0.00549, 0.00751
	Hispanic	Women	82	0.0024, 0.00331, 0.00457, 0.0063, 0.00872
	Hispanic	Women	83	0.00263, 0.00369, 0.00516, 0.0072, 0.0101
	Hispanic	Women	84	0.00287, 0.00408, 0.00577, 0.00816, 0.0116
	White	Men	30	0.0000168, 0.0000238, 0.0000337, 0.0000476, 0.0000674
	White	Men	31	0.0000219, 0.0000302, 0.0000414, 0.000057, 0.0000785
	White	Men	32	0.0000279, 0.0000376, 0.0000506, 0.0000682, 0.000092
	White	Men	33	0.0000348, 0.0000462, 0.0000613, 0.0000813, 0.000108
	White	Men	34	0.0000422, 0.0000557, 0.0000733, 0.0000964, 0.000127
	White	Men	35	0.0000499, 0.0000658, 0.0000865, 0.000114, 0.00015
	White	Men	36	0.0000581, 0.0000766, 0.000101, 0.000133, 0.000175
	White	Men	37	0.0000673, 0.0000886, 0.000116, 0.000153, 0.000201
	White	Men	38	0.0000783, 0.000102, 0.000133, 0.000174, 0.000227
	White	Men	39	0.0000916, 0.000118, 0.000152, 0.000195, 0.000252
	White	Men	40	0.000107, 0.000136, 0.000172, 0.000217, 0.000275
	White	Men	41	0.000126, 0.000156, 0.000193, 0.000239, 0.000296
	White	Men	42	0.000147, 0.000179, 0.000216, 0.000262, 0.000317
	White	Men	43	0.00017, 0.000203, 0.000241, 0.000287, 0.000342

Variable	Race/ethnicity	Sex	Age	Distribution
	White	Men	44	0.000194, 0.000229, 0.000269, 0.000317, 0.000373
	White	Men	45	0.000222, 0.000259, 0.000301, 0.000351, 0.000409
	White	Men	46	0.000256, 0.000295, 0.000339, 0.000389, 0.000448
	White	Men	47	0.000296, 0.000337, 0.000383, 0.000435, 0.000495
	White	Men	48	0.000342, 0.000386, 0.000435, 0.000491, 0.000555
	White	Men	49	0.000393, 0.000442, 0.000497, 0.000558, 0.000628
	White	Men	50	0.000453, 0.000507, 0.000567, 0.000634, 0.00071
	White	Men	51	0.000524, 0.000582, 0.000646, 0.000716, 0.000795
	White	Men	52	0.000602, 0.000664, 0.000732, 0.000807, 0.00089
	White	Men	53	0.000674, 0.000746, 0.000824, 0.00091, 0.00101
	White	Men	54	0.000739, 0.000825, 0.00092, 0.00103, 0.00114
	White	Men	55	0.000803, 0.000904, 0.00102, 0.00114, 0.00129
	White	Men	56	0.000868, 0.000984, 0.00112, 0.00126, 0.00143
	White	Men	57	0.000932, 0.00106, 0.00121, 0.00138, 0.00158
	White	Men	58	0.000996, 0.00114, 0.00131, 0.00151, 0.00173
	White	Men	59	0.00106, 0.00123, 0.00141, 0.00163, 0.00188
	White	Men	60	0.00113, 0.00131, 0.00152, 0.00175, 0.00203
	White	Men	61	0.00121, 0.0014, 0.00162, 0.00187, 0.00217
	White	Men	62	0.00128, 0.00148, 0.00172, 0.00199, 0.0023
	White	Men	63	0.00134, 0.00156, 0.00181, 0.0021, 0.00244
	White	Men	64	0.0014, 0.00163, 0.0019, 0.0022, 0.00256
	White	Men	65	0.00146, 0.0017, 0.00199, 0.00232, 0.00271
	White	Men	66	0.00151, 0.00177, 0.00208, 0.00245, 0.00288
	White	Men	67	0.00156, 0.00186, 0.0022, 0.00261, 0.0031
	White	Men	68	0.00163, 0.00196, 0.00235, 0.00281, 0.00338
	White	Men	69	0.00173, 0.00209, 0.00253, 0.00305, 0.00369
	White	Men	70	0.00186, 0.00225, 0.00274, 0.00332, 0.00403
	White	Men	71	0.00201, 0.00245, 0.00297, 0.00361, 0.0044
	White	Men	72	0.00219, 0.00266, 0.00324, 0.00394, 0.0048
	White	Men	73	0.00238, 0.0029, 0.00353, 0.0043, 0.00524
	White	Men	74	0.00259, 0.00316, 0.00385, 0.00469, 0.00571
	White	Men	75	0.00283, 0.00345, 0.0042, 0.00512, 0.00626
	White	Men	76	0.00308, 0.00378, 0.00461, 0.00563, 0.00689
	White	Men	77	0.00338, 0.00415, 0.00508, 0.00623, 0.00765
	White	Men	78	0.00372, 0.00459, 0.00564, 0.00694, 0.00856
	White	Men	79	0.00413, 0.00511, 0.0063, 0.00778, 0.00963
	White	Men	80	0.00462, 0.00572, 0.00707, 0.00875, 0.0108
	White	Men	81	0.00518, 0.00643, 0.00795, 0.00983, 0.0122
	White	Men	82	0.00581, 0.0072, 0.00891, 0.011, 0.0137
	White	Men	83	0.00648, 0.00802, 0.00992, 0.0123, 0.0152
	White	Men	84	0.00715, 0.00886, 0.0109, 0.0135, 0.0168
	White	Women	30	0.0000045, 0.00000702, 0.0000109, 0.000017, 0.0000265
	White	Women	31	0.00000594, 0.00000894, 0.0000134, 0.0000201, 0.0000302

Variable	Race/ethnicity	Sex	Age	Distribution
	White	Women	32	0.00000785, 0.0000113, 0.0000163, 0.0000235, 0.000034
	White	Women	33	0.0000102, 0.0000142, 0.0000198, 0.0000274, 0.0000382
	White	Women	34	0.000013, 0.0000175, 0.0000237, 0.0000319, 0.0000432
	White	Women	35	0.0000159, 0.0000212, 0.000028, 0.0000371, 0.0000493
	White	Women	36	0.000019, 0.000025, 0.0000327, 0.0000429, 0.0000564
	White	Women	37	0.0000221, 0.0000289, 0.0000378, 0.0000493, 0.0000646
	White	Women	38	0.000025, 0.0000329, 0.000043, 0.0000564, 0.000074
	White	Women	39	0.0000278, 0.0000368, 0.0000485, 0.0000639, 0.0000844
	White	Women	40	0.0000307, 0.0000408, 0.000054, 0.0000716, 0.0000952
	White	Women	41	0.0000339, 0.000045, 0.0000597, 0.0000791, 0.000105
	White	Women	42	0.0000376, 0.0000498, 0.0000656, 0.0000865, 0.000114
	White	Women	43	0.0000417, 0.0000549, 0.0000721, 0.0000946, 0.000125
	White	Women	44	0.0000459, 0.0000604, 0.0000793, 0.000104, 0.000137
	White	Women	45	0.000051, 0.0000669, 0.0000877, 0.000115, 0.000151
	White	Women	46	0.0000579, 0.0000752, 0.0000975, 0.000126, 0.000164
	White	Women	47	0.0000677, 0.000086, 0.000109, 0.000138, 0.000176
	White	Women	48	0.0000805, 0.0000996, 0.000123, 0.000152, 0.000187
	White	Women	49	0.0000959, 0.000116, 0.000139, 0.000168, 0.000202
	White	Women	50	0.000113, 0.000134, 0.000159, 0.000188, 0.000223
	White	Women	51	0.000131, 0.000154, 0.000181, 0.000213, 0.000251
	White	Women	52	0.000149, 0.000176, 0.000207, 0.000243, 0.000286
	White	Women	53	0.000169, 0.000199, 0.000235, 0.000277, 0.000327
	White	Women	54	0.000189, 0.000224, 0.000265, 0.000314, 0.000373
	White	Women	55	0.000211, 0.000251, 0.000298, 0.000355, 0.000423
	White	Women	56	0.000235, 0.00028, 0.000334, 0.000398, 0.000475
	White	Women	57	0.000262, 0.000313, 0.000374, 0.000446, 0.000533
	White	Women	58	0.000293, 0.00035, 0.000417, 0.000498, 0.000595
	White	Women	59	0.000326, 0.00039, 0.000465, 0.000555, 0.000663
	White	Women	60	0.00036, 0.000431, 0.000516, 0.000618, 0.000741
	White	Women	61	0.000392, 0.000473, 0.000569, 0.000685, 0.000826
	White	Women	62	0.000423, 0.000513, 0.000622, 0.000752, 0.000912
	White	Women	63	0.000455, 0.000554, 0.000673, 0.000818, 0.000997
	White	Women	64	0.000487, 0.000595, 0.000726, 0.000885, 0.00108
	White	Women	65	0.000521, 0.000639, 0.000782, 0.000957, 0.00117
	White	Women	66	0.000562, 0.00069, 0.000847, 0.00104, 0.00128
	White	Women	67	0.000611, 0.000752, 0.000923, 0.00113, 0.0014
	White	Women	68	0.00067, 0.000825, 0.00101, 0.00125, 0.00154
	White	Women	69	0.000739, 0.000911, 0.00112, 0.00138, 0.0017
	White	Women	70	0.00082, 0.00101, 0.00124, 0.00153, 0.00189
	White	Women	71	0.000909, 0.00112, 0.00138, 0.00171, 0.00211
	White	Women	72	0.00101, 0.00125, 0.00154, 0.0019, 0.00236
	White	Women	73	0.00111, 0.00139, 0.00172, 0.00213, 0.00265
	White	Women	74	0.00123, 0.00154, 0.00192, 0.00239, 0.00299

Variable	Race/ethnicity	Sex	Age	Distribution
	White	Women	75	0.00137, 0.00172, 0.00215, 0.00269, 0.00337
	White	Women	76	0.00152, 0.00192, 0.00241, 0.00303, 0.00382
	White	Women	77	0.0017, 0.00216, 0.00272, 0.00344, 0.00435
	White	Women	78	0.00191, 0.00243, 0.00309, 0.00392, 0.00498
	White	Women	79	0.00216, 0.00276, 0.00352, 0.00449, 0.00574
	White	Women	80	0.00244, 0.00314, 0.00402, 0.00516, 0.00663
	White	Women	81	0.00276, 0.00357, 0.0046, 0.00593, 0.00767
	White	Women	82	0.00312, 0.00406, 0.00526, 0.00681, 0.00885
	White	Women	83	0.00351, 0.00459, 0.00597, 0.00778, 0.0102
	White	Women	84	0.00392, 0.00514, 0.00673, 0.0088, 0.0115
CHD mortality rates for 2030 (0.01, 0.2, 0.5, 0.8. 0.99 percentiles of the empirical distribution produced during forecasting)				
	Black	Men	30	0.0000271, 0.00004, 0.0000587, 0.0000863, 0.000127
	Black	Men	31	0.0000348, 0.0000497, 0.0000708, 0.000101, 0.000144
	Black	Men	32	0.0000442, 0.0000613, 0.0000847, 0.000117, 0.000162
	Black	Men	33	0.0000553, 0.0000747, 0.0001, 0.000135, 0.000182
	Black	Men	34	0.0000674, 0.0000892, 0.000118, 0.000155, 0.000206
	Black	Men	35	0.0000794, 0.000104, 0.000136, 0.000178, 0.000233
	Black	Men	36	0.000091, 0.000119, 0.000155, 0.000202, 0.000265
	Black	Men	37	0.000102, 0.000134, 0.000175, 0.000228, 0.000298
	Black	Men	38	0.000114, 0.000149, 0.000195, 0.000254, 0.000332
	Black	Men	39	0.000127, 0.000165, 0.000215, 0.00028, 0.000365
	Black	Men	40	0.00014, 0.000182, 0.000236, 0.000306, 0.000398
	Black	Men	41	0.000156, 0.000201, 0.000259, 0.000332, 0.000429
	Black	Men	42	0.000174, 0.000222, 0.000283, 0.000361, 0.000461
	Black	Men	43	0.000192, 0.000245, 0.000311, 0.000395, 0.000504
	Black	Men	44	0.00021, 0.000269, 0.000343, 0.000438, 0.000561
	Black	Men	45	0.000232, 0.000298, 0.000382, 0.000491, 0.000631
	Black	Men	46	0.000259, 0.000334, 0.000429, 0.000552, 0.000712
	Black	Men	47	0.000293, 0.000378, 0.000487, 0.000626, 0.000808
	Black	Men	48	0.000335, 0.000433, 0.000557, 0.000717, 0.000926
	Black	Men	49	0.000387, 0.000499, 0.000643, 0.000828, 0.00107
	Black	Men	50	0.000451, 0.000581, 0.000746, 0.000957, 0.00123
	Black	Men	51	0.000533, 0.00068, 0.000866, 0.0011, 0.00141
	Black	Men	52	0.000632, 0.000797, 0.001, 0.00126, 0.00159
	Black	Men	53	0.000739, 0.000923, 0.00115, 0.00144, 0.00179
	Black	Men	54	0.000845, 0.00105, 0.00131, 0.00163, 0.00202
	Black	Men	55	0.000954, 0.00118, 0.00147, 0.00182, 0.00226
	Black	Men	56	0.00107, 0.00132, 0.00163, 0.00201, 0.00248
	Black	Men	57	0.00119, 0.00146, 0.00179, 0.00219, 0.00269
	Black	Men	58	0.00131, 0.0016, 0.00195, 0.00237, 0.0029
	Black	Men	59	0.00143, 0.00174, 0.00211, 0.00255, 0.0031
	Black	Men	60	0.00156, 0.00188, 0.00226, 0.00272, 0.00329
	Black	Men	61	0.00168, 0.00201, 0.0024, 0.00288, 0.00345

Variable	Race/ethnicity	Sex	Age	Distribution
	Black	Men	62	0.00179, 0.00213, 0.00253, 0.00301, 0.00358
	Black	Men	63	0.00191, 0.00225, 0.00265, 0.00312, 0.00368
	Black	Men	64	0.00201, 0.00236, 0.00276, 0.00323, 0.00378
	Black	Men	65	0.00211, 0.00247, 0.00287, 0.00335, 0.00391
	Black	Men	66	0.00221, 0.00258, 0.00301, 0.0035, 0.00409
	Black	Men	67	0.00231, 0.00271, 0.00317, 0.0037, 0.00434
	Black	Men	68	0.00242, 0.00285, 0.00336, 0.00395, 0.00466
	Black	Men	69	0.00255, 0.00303, 0.00358, 0.00423, 0.00501
	Black	Men	70	0.00271, 0.00322, 0.00382, 0.00453, 0.00538
	Black	Men	71	0.00289, 0.00344, 0.00408, 0.00485, 0.00576
	Black	Men	72	0.00308, 0.00367, 0.00436, 0.00518, 0.00617
	Black	Men	73	0.00328, 0.00391, 0.00465, 0.00553, 0.00659
	Black	Men	74	0.0035, 0.00417, 0.00497, 0.00591, 0.00705
	Black	Men	75	0.00375, 0.00447, 0.00532, 0.00632, 0.00753
	Black	Men	76	0.00405, 0.00481, 0.00571, 0.00678, 0.00807
	Black	Men	77	0.00438, 0.0052, 0.00617, 0.00732, 0.00869
	Black	Men	78	0.00473, 0.00563, 0.00668, 0.00793, 0.00943
	Black	Men	79	0.00511, 0.00609, 0.00725, 0.00864, 0.0103
	Black	Men	80	0.00548, 0.00658, 0.00787, 0.00942, 0.0113
	Black	Men	81	0.00585, 0.00707, 0.00851, 0.0103, 0.0124
	Black	Men	82	0.00619, 0.00754, 0.00917, 0.0112, 0.0136
	Black	Men	83	0.00649, 0.00799, 0.00983, 0.0121, 0.0149
	Black	Men	84	0.00676, 0.00842, 0.0105, 0.013, 0.0162
	Black	Women	30	0.0000114, 0.0000169, 0.0000249, 0.0000366, 0.0000541
	Black	Women	31	0.0000147, 0.0000208, 0.0000294, 0.0000417, 0.0000591
	Black	Women	32	0.0000183, 0.0000253, 0.0000348, 0.0000479, 0.0000661
	Black	Women	33	0.0000225, 0.0000304, 0.000041, 0.0000553, 0.0000748
	Black	Women	34	0.0000269, 0.000036, 0.000048, 0.000064, 0.0000857
	Black	Women	35	0.0000311, 0.0000416, 0.0000556, 0.0000742, 0.0000993
	Black	Women	36	0.0000352, 0.0000473, 0.0000635, 0.0000852, 0.000115
	Black	Women	37	0.0000395, 0.0000533, 0.0000718, 0.0000968, 0.000131
	Black	Women	38	0.0000439, 0.0000596, 0.0000806, 0.000109, 0.000148
	Black	Women	39	0.0000485, 0.0000661, 0.0000897, 0.000122, 0.000166
	Black	Women	40	0.0000538, 0.0000732, 0.0000993, 0.000135, 0.000183
	Black	Women	41	0.0000607, 0.0000816, 0.00011, 0.000147, 0.000198
	Black	Women	42	0.0000696, 0.0000918, 0.000121, 0.000159, 0.000209
	Black	Women	43	0.0000802, 0.000104, 0.000133, 0.000172, 0.000221
	Black	Women	44	0.0000914, 0.000117, 0.000148, 0.000188, 0.00024
	Black	Women	45	0.000104, 0.000131, 0.000166, 0.00021, 0.000266
	Black	Women	46	0.000119, 0.00015, 0.000188, 0.000237, 0.000298
	Black	Women	47	0.000138, 0.000173, 0.000215, 0.000268, 0.000335
	Black	Women	48	0.000161, 0.0002, 0.000248, 0.000308, 0.000382
	Black	Women	49	0.000184, 0.00023, 0.000287, 0.000359, 0.000449

Variable	Race/ethnicity	Sex	Age	Distribution
	Black	Women	50	0.00021, 0.000265, 0.000333, 0.000418, 0.000526
	Black	Women	51	0.000246, 0.000308, 0.000383, 0.000478, 0.000596
	Black	Women	52	0.00029, 0.000357, 0.000438, 0.000537, 0.000661
	Black	Women	53	0.000332, 0.000407, 0.000497, 0.000607, 0.000743
	Black	Women	54	0.00037, 0.000456, 0.00056, 0.00069, 0.00085
	Black	Women	55	0.000413, 0.00051, 0.000629, 0.000776, 0.000959
	Black	Women	56	0.000467, 0.000574, 0.000704, 0.000862, 0.00106
	Black	Women	57	0.000526, 0.000642, 0.000782, 0.000952, 0.00116
	Black	Women	58	0.000581, 0.000709, 0.000863, 0.00105, 0.00128
	Black	Women	59	0.000631, 0.000772, 0.000944, 0.00115, 0.00141
	Black	Women	60	0.000676, 0.000833, 0.00102, 0.00126, 0.00155
	Black	Women	61	0.000716, 0.000889, 0.0011, 0.00136, 0.00169
	Black	Women	62	0.00075, 0.000939, 0.00117, 0.00147, 0.00184
	Black	Women	63	0.000777, 0.000983, 0.00124, 0.00157, 0.00198
	Black	Women	64	0.000799, 0.00102, 0.00131, 0.00167, 0.00214
	Black	Women	65	0.000823, 0.00107, 0.00138, 0.00178, 0.00231
	Black	Women	66	0.000855, 0.00112, 0.00146, 0.0019, 0.00249
	Black	Women	67	0.000902, 0.00119, 0.00156, 0.00205, 0.0027
	Black	Women	68	0.000967, 0.00128, 0.00169, 0.00222, 0.00294
	Black	Women	69	0.00105, 0.00139, 0.00183, 0.00242, 0.00319
	Black	Women	70	0.00116, 0.00153, 0.002, 0.00263, 0.00346
	Black	Women	71	0.00128, 0.00167, 0.00219, 0.00286, 0.00375
	Black	Women	72	0.0014, 0.00183, 0.00238, 0.00311, 0.00405
	Black	Women	73	0.00153, 0.002, 0.0026, 0.00337, 0.0044
	Black	Women	74	0.00167, 0.00217, 0.00282, 0.00367, 0.00478
	Black	Women	75	0.00182, 0.00237, 0.00308, 0.00401, 0.00523
	Black	Women	76	0.00197, 0.00258, 0.00337, 0.00441, 0.00577
	Black	Women	77	0.00213, 0.00281, 0.0037, 0.00488, 0.00645
	Black	Women	78	0.00229, 0.00306, 0.00408, 0.00543, 0.00727
	Black	Women	79	0.00245, 0.00332, 0.00449, 0.00608, 0.00824
	Black	Women	80	0.00262, 0.00361, 0.00495, 0.0068, 0.00937
	Black	Women	81	0.00281, 0.00392, 0.00545, 0.00759, 0.0106
	Black	Women	82	0.00302, 0.00426, 0.00599, 0.00842, 0.0119
	Black	Women	83	0.00327, 0.00464, 0.00656, 0.00927, 0.0132
	Black	Women	84	0.00353, 0.00503, 0.00714, 0.0101, 0.0144
	Hispanic	Men	30	0.00000933, 0.0000137, 0.00002, 0.0000293, 0.0000431
	Hispanic	Men	31	0.0000117, 0.0000167, 0.0000239, 0.0000341, 0.0000488
	Hispanic	Men	32	0.0000145, 0.0000203, 0.0000284, 0.0000396, 0.0000554
	Hispanic	Men	33	0.000018, 0.0000247, 0.0000336, 0.0000458, 0.0000626
	Hispanic	Men	34	0.0000222, 0.0000297, 0.0000395, 0.0000526, 0.0000703
	Hispanic	Men	35	0.0000269, 0.0000353, 0.0000461, 0.0000602, 0.0000789
	Hispanic	Men	36	0.0000318, 0.0000412, 0.0000532, 0.0000687, 0.000089
	Hispanic	Men	37	0.0000369, 0.0000474, 0.0000609, 0.0000783, 0.000101

Variable	Race/ethnicity	Sex	Age	Distribution
	Hispanic	Men	38	0.0000422, 0.0000542, 0.0000694, 0.0000888, 0.000114
	Hispanic	Men	39	0.0000484, 0.0000617, 0.0000786, 0.0001, 0.000128
	Hispanic	Men	40	0.0000558, 0.0000705, 0.0000889, 0.000112, 0.000142
	Hispanic	Men	41	0.0000649, 0.0000808, 0.0001, 0.000124, 0.000155
	Hispanic	Men	42	0.0000759, 0.0000927, 0.000113, 0.000138, 0.000168
	Hispanic	Men	43	0.0000884, 0.000106, 0.000127, 0.000153, 0.000184
	Hispanic	Men	44	0.000102, 0.000121, 0.000144, 0.000171, 0.000204
	Hispanic	Men	45	0.000117, 0.000139, 0.000164, 0.000193, 0.000229
	Hispanic	Men	46	0.000134, 0.000158, 0.000187, 0.000221, 0.000261
	Hispanic	Men	47	0.000154, 0.000182, 0.000215, 0.000253, 0.000298
	Hispanic	Men	48	0.000181, 0.000212, 0.000248, 0.000289, 0.000339
	Hispanic	Men	49	0.000214, 0.000248, 0.000288, 0.000334, 0.000387
	Hispanic	Men	50	0.00025, 0.000289, 0.000335, 0.000388, 0.00045
	Hispanic	Men	51	0.00029, 0.000337, 0.000391, 0.000453, 0.000527
	Hispanic	Men	52	0.000334, 0.00039, 0.000454, 0.000529, 0.000617
	Hispanic	Men	53	0.000379, 0.000446, 0.000524, 0.000615, 0.000723
	Hispanic	Men	54	0.000425, 0.000505, 0.000598, 0.000709, 0.000842
	Hispanic	Men	55	0.000477, 0.000569, 0.000676, 0.000804, 0.000958
	Hispanic	Men	56	0.000537, 0.000638, 0.000757, 0.000898, 0.00107
	Hispanic	Men	57	0.000602, 0.000712, 0.00084, 0.000992, 0.00117
	Hispanic	Men	58	0.000668, 0.000787, 0.000925, 0.00109, 0.00128
	Hispanic	Men	59	0.000732, 0.000861, 0.00101, 0.00119, 0.0014
	Hispanic	Men	60	0.000794, 0.000934, 0.0011, 0.00129, 0.00152
	Hispanic	Men	61	0.000852, 0.001, 0.00118, 0.00139, 0.00164
	Hispanic	Men	62	0.000908, 0.00107, 0.00126, 0.00149, 0.00176
	Hispanic	Men	63	0.000962, 0.00114, 0.00134, 0.00158, 0.00187
	Hispanic	Men	64	0.00102, 0.0012, 0.00142, 0.00168, 0.00199
	Hispanic	Men	65	0.00107, 0.00127, 0.0015, 0.00178, 0.00211
	Hispanic	Men	66	0.00113, 0.00135, 0.00159, 0.00189, 0.00224
	Hispanic	Men	67	0.0012, 0.00143, 0.0017, 0.00203, 0.00242
	Hispanic	Men	68	0.00127, 0.00152, 0.00183, 0.0022, 0.00264
	Hispanic	Men	69	0.00135, 0.00164, 0.00198, 0.0024, 0.00291
	Hispanic	Men	70	0.00145, 0.00177, 0.00216, 0.00263, 0.00321
	Hispanic	Men	71	0.00156, 0.00192, 0.00235, 0.00289, 0.00355
	Hispanic	Men	72	0.00169, 0.00209, 0.00257, 0.00318, 0.00393
	Hispanic	Men	73	0.00183, 0.00227, 0.00282, 0.00349, 0.00432
	Hispanic	Men	74	0.002, 0.00249, 0.00308, 0.00382, 0.00474
	Hispanic	Men	75	0.0022, 0.00273, 0.00338, 0.00418, 0.00519
	Hispanic	Men	76	0.00243, 0.00301, 0.00372, 0.0046, 0.00569
	Hispanic	Men	77	0.00271, 0.00334, 0.00412, 0.00508, 0.00627
	Hispanic	Men	78	0.00302, 0.00373, 0.00458, 0.00563, 0.00694
	Hispanic	Men	79	0.00337, 0.00415, 0.0051, 0.00627, 0.00771
	Hispanic	Men	80	0.00372, 0.0046, 0.00567, 0.00699, 0.00864

Variable	Race/ethnicity	Sex	Age	Distribution
	Hispanic	Men	81	0.00405, 0.00504, 0.00627, 0.0078, 0.00972
	Hispanic	Men	82	0.00434, 0.00547, 0.00689, 0.00867, 0.0109
	Hispanic	Men	83	0.00461, 0.00589, 0.0075, 0.00957, 0.0122
	Hispanic	Men	84	0.00487, 0.0063, 0.00812, 0.0105, 0.0135
	Hispanic	Women	30	0.00000253, 0.00000382, 0.00000574, 0.00000864, 0.0000131
	Hispanic	Women	31	0.00000311, 0.00000456, 0.00000667, 0.00000976, 0.0000143
	Hispanic	Women	32	0.0000038, 0.00000544, 0.00000777, 0.0000111, 0.0000159
	Hispanic	Women	33	0.0000046, 0.00000646, 0.00000902, 0.0000126, 0.0000177
	Hispanic	Women	34	0.00000546, 0.00000756, 0.0000104, 0.0000144, 0.0000199
	Hispanic	Women	35	0.00000633, 0.00000871, 0.000012, 0.0000164, 0.0000226
	Hispanic	Women	36	0.00000719, 0.0000099, 0.0000136, 0.0000187, 0.0000257
	Hispanic	Women	37	0.00000808, 0.0000111, 0.0000153, 0.0000211, 0.0000291
	Hispanic	Women	38	0.00000903, 0.0000125, 0.0000172, 0.0000237, 0.0000328
	Hispanic	Women	39	0.0000101, 0.000014, 0.0000192, 0.0000265, 0.0000366
	Hispanic	Women	40	0.0000115, 0.0000157, 0.0000215, 0.0000293, 0.0000402
	Hispanic	Women	41	0.0000132, 0.0000178, 0.000024, 0.0000322, 0.0000434
	Hispanic	Women	42	0.0000155, 0.0000204, 0.0000268, 0.0000351, 0.0000462
	Hispanic	Women	43	0.0000183, 0.0000235, 0.0000301, 0.0000386, 0.0000495
	Hispanic	Women	44	0.0000215, 0.0000271, 0.0000341, 0.0000429, 0.000054
	Hispanic	Women	45	0.0000254, 0.0000315, 0.0000389, 0.0000482, 0.0000597
	Hispanic	Women	46	0.0000303, 0.0000369, 0.0000448, 0.0000544, 0.0000662
	Hispanic	Women	47	0.0000365, 0.0000435, 0.0000519, 0.0000618, 0.0000738
	Hispanic	Women	48	0.0000436, 0.0000514, 0.0000604, 0.0000711, 0.0000838
	Hispanic	Women	49	0.0000512, 0.0000602, 0.0000707, 0.0000831, 0.0000977
	Hispanic	Women	50	0.0000591, 0.0000702, 0.0000832, 0.0000986, 0.000117
	Hispanic	Women	51	0.0000677, 0.0000815, 0.000098, 0.000118, 0.000142
	Hispanic	Women	52	0.0000774, 0.0000946, 0.000115, 0.000141, 0.000172
	Hispanic	Women	53	0.0000885, 0.00011, 0.000136, 0.000168, 0.000208
	Hispanic	Women	54	0.000101, 0.000127, 0.000159, 0.000199, 0.000249
	Hispanic	Women	55	0.000116, 0.000147, 0.000185, 0.000233, 0.000294
	Hispanic	Women	56	0.000134, 0.000169, 0.000215, 0.000272, 0.000345
	Hispanic	Women	57	0.000152, 0.000194, 0.000248, 0.000315, 0.000402
	Hispanic	Women	58	0.000172, 0.000222, 0.000284, 0.000365, 0.000469
	Hispanic	Women	59	0.000193, 0.000251, 0.000324, 0.00042, 0.000544
	Hispanic	Women	60	0.000215, 0.000281, 0.000367, 0.00048, 0.000628
	Hispanic	Women	61	0.000237, 0.000313, 0.000412, 0.000543, 0.000717
	Hispanic	Women	62	0.00026, 0.000346, 0.000458, 0.000607, 0.000806
	Hispanic	Women	63	0.000285, 0.00038, 0.000504, 0.00067, 0.000892
	Hispanic	Women	64	0.000313, 0.000416, 0.000551, 0.000731, 0.000972
	Hispanic	Women	65	0.000344, 0.000456, 0.000602, 0.000794, 0.00105
	Hispanic	Women	66	0.00038, 0.000501, 0.000658, 0.000865, 0.00114
	Hispanic	Women	67	0.000421, 0.000554, 0.000725, 0.00095, 0.00125
	Hispanic	Women	68	0.000468, 0.000614, 0.000804, 0.00105, 0.00138

Variable	Race/ethnicity	Sex	Age	Distribution
	Hispanic	Women	69	0.000523, 0.000685, 0.000896, 0.00117, 0.00154
	Hispanic	Women	70	0.000585, 0.000767, 0.001, 0.00131, 0.00172
	Hispanic	Women	71	0.000657, 0.00086, 0.00112, 0.00147, 0.00192
	Hispanic	Women	72	0.000737, 0.000964, 0.00126, 0.00164, 0.00215
	Hispanic	Women	73	0.000827, 0.00108, 0.00141, 0.00184, 0.00241
	Hispanic	Women	74	0.000927, 0.00122, 0.00159, 0.00208, 0.00273
	Hispanic	Women	75	0.00104, 0.00137, 0.00179, 0.00235, 0.0031
	Hispanic	Women	76	0.00117, 0.00154, 0.00203, 0.00268, 0.00354
	Hispanic	Women	77	0.00131, 0.00174, 0.00231, 0.00306, 0.00407
	Hispanic	Women	78	0.00148, 0.00197, 0.00264, 0.00352, 0.00471
	Hispanic	Women	79	0.00166, 0.00224, 0.00301, 0.00405, 0.00546
	Hispanic	Women	80	0.00187, 0.00254, 0.00344, 0.00466, 0.00633
	Hispanic	Women	81	0.0021, 0.00287, 0.00392, 0.00536, 0.00735
	Hispanic	Women	82	0.00233, 0.00323, 0.00446, 0.00616, 0.00853
	Hispanic	Women	83	0.00256, 0.00359, 0.00503, 0.00704, 0.00988
	Hispanic	Women	84	0.00279, 0.00397, 0.00562, 0.00797, 0.0113
	White	Men	30	0.0000165, 0.0000235, 0.0000335, 0.0000477, 0.0000682
	White	Men	31	0.0000215, 0.0000298, 0.0000413, 0.0000572, 0.0000794
	White	Men	32	0.0000274, 0.0000373, 0.0000505, 0.0000684, 0.0000929
	White	Men	33	0.0000343, 0.0000458, 0.0000611, 0.0000815, 0.000109
	White	Men	34	0.0000416, 0.0000552, 0.0000731, 0.0000967, 0.000128
	White	Men	35	0.0000492, 0.0000652, 0.0000862, 0.000114, 0.000151
	White	Men	36	0.0000571, 0.0000758, 0.0001, 0.000133, 0.000176
	White	Men	37	0.000066, 0.0000875, 0.000116, 0.000153, 0.000202
	White	Men	38	0.0000767, 0.000101, 0.000132, 0.000173, 0.000228
	White	Men	39	0.0000894, 0.000116, 0.00015, 0.000195, 0.000253
	White	Men	40	0.000105, 0.000133, 0.00017, 0.000216, 0.000275
	White	Men	41	0.000123, 0.000153, 0.00019, 0.000237, 0.000295
	White	Men	42	0.000143, 0.000175, 0.000212, 0.000258, 0.000314
	White	Men	43	0.000165, 0.000198, 0.000236, 0.000282, 0.000338
	White	Men	44	0.000189, 0.000223, 0.000263, 0.000311, 0.000368
	White	Men	45	0.000216, 0.000252, 0.000295, 0.000344, 0.000402
	White	Men	46	0.000249, 0.000287, 0.000331, 0.000382, 0.000441
	White	Men	47	0.000288, 0.000329, 0.000374, 0.000427, 0.000487
	White	Men	48	0.000332, 0.000376, 0.000426, 0.000482, 0.000545
	White	Men	49	0.000382, 0.000431, 0.000486, 0.000547, 0.000618
	White	Men	50	0.000441, 0.000495, 0.000555, 0.000622, 0.000698
	White	Men	51	0.000512, 0.000569, 0.000633, 0.000704, 0.000784
	White	Men	52	0.000588, 0.00065, 0.000719, 0.000794, 0.000878
	White	Men	53	0.000659, 0.000731, 0.00081, 0.000897, 0.000995
	White	Men	54	0.000723, 0.000809, 0.000905, 0.00101, 0.00113
	White	Men	55	0.000786, 0.000888, 0.001, 0.00113, 0.00128
	White	Men	56	0.000851, 0.000967, 0.0011, 0.00125, 0.00142

Variable	Race/ethnicity	Sex	Age	Distribution
	White	Men	57	0.000913, 0.00105, 0.0012, 0.00137, 0.00157
	White	Men	58	0.000976, 0.00112, 0.00129, 0.00149, 0.00172
	White	Men	59	0.00104, 0.00121, 0.00139, 0.00161, 0.00186
	White	Men	60	0.00111, 0.00129, 0.00149, 0.00173, 0.00201
	White	Men	61	0.00118, 0.00137, 0.00159, 0.00185, 0.00215
	White	Men	62	0.00125, 0.00145, 0.00169, 0.00196, 0.00228
	White	Men	63	0.00131, 0.00153, 0.00178, 0.00206, 0.00241
	White	Men	64	0.00137, 0.0016, 0.00186, 0.00217, 0.00253
	White	Men	65	0.00142, 0.00166, 0.00194, 0.00227, 0.00266
	White	Men	66	0.00147, 0.00173, 0.00204, 0.0024, 0.00283
	White	Men	67	0.00152, 0.00181, 0.00215, 0.00256, 0.00305
	White	Men	68	0.00159, 0.00191, 0.0023, 0.00276, 0.00332
	White	Men	69	0.00168, 0.00204, 0.00247, 0.00299, 0.00362
	White	Men	70	0.0018, 0.0022, 0.00267, 0.00325, 0.00396
	White	Men	71	0.00195, 0.00238, 0.0029, 0.00354, 0.00432
	White	Men	72	0.00212, 0.00259, 0.00316, 0.00386, 0.00471
	White	Men	73	0.00231, 0.00283, 0.00345, 0.0042, 0.00514
	White	Men	74	0.00252, 0.00308, 0.00376, 0.00458, 0.0056
	White	Men	75	0.00274, 0.00336, 0.0041, 0.00501, 0.00613
	White	Men	76	0.003, 0.00367, 0.0045, 0.00551, 0.00675
	White	Men	77	0.00328, 0.00404, 0.00496, 0.00609, 0.0075
	White	Men	78	0.00361, 0.00446, 0.00551, 0.00679, 0.00839
	White	Men	79	0.00401, 0.00497, 0.00615, 0.00761, 0.00944
	White	Men	80	0.00448, 0.00557, 0.0069, 0.00856, 0.0106
	White	Men	81	0.00503, 0.00625, 0.00776, 0.00962, 0.012
	White	Men	82	0.00564, 0.00701, 0.00869, 0.0108, 0.0134
	White	Men	83	0.00628, 0.0078, 0.00968, 0.012, 0.0149
	White	Men	84	0.00693, 0.00861, 0.0107, 0.0132, 0.0164
	White	Women	30	0.00000442, 0.00000695, 0.0000109, 0.000017, 0.0000268
	White	Women	31	0.00000585, 0.00000886, 0.0000133, 0.0000201, 0.0000304
	White	Women	32	0.00000774, 0.0000112, 0.0000163, 0.0000236, 0.0000342
	White	Women	33	0.0000101, 0.0000141, 0.0000197, 0.0000275, 0.0000385
	White	Women	34	0.0000128, 0.0000174, 0.0000236, 0.000032, 0.0000436
	White	Women	35	0.0000157, 0.000021, 0.0000279, 0.0000372, 0.0000497
	White	Women	36	0.0000187, 0.0000247, 0.0000326, 0.000043, 0.0000568
	White	Women	37	0.0000217, 0.0000285, 0.0000375, 0.0000493, 0.000065
	White	Women	38	0.0000245, 0.0000324, 0.0000427, 0.0000562, 0.0000742
	White	Women	39	0.0000272, 0.0000362, 0.0000479, 0.0000635, 0.0000844
	White	Women	40	0.00003, 0.00004, 0.0000533, 0.000071, 0.0000948
	White	Women	41	0.0000331, 0.0000441, 0.0000588, 0.0000782, 0.000104
	White	Women	42	0.0000367, 0.0000487, 0.0000644, 0.0000852, 0.000113
	White	Women	43	0.0000407, 0.0000537, 0.0000706, 0.0000929, 0.000123
	White	Women	44	0.0000448, 0.000059, 0.0000776, 0.000102, 0.000135

Variable	Race/ethnicity	Sex	Age	Distribution
	White	Women	45	0.0000496, 0.0000653, 0.0000857, 0.000112, 0.000148
	White	Women	46	0.0000564, 0.0000734, 0.0000952, 0.000124, 0.000161
	White	Women	47	0.0000659, 0.0000839, 0.000107, 0.000135, 0.000172
	White	Women	48	0.0000784, 0.0000971, 0.00012, 0.000148, 0.000184
	White	Women	49	0.0000935, 0.000113, 0.000136, 0.000164, 0.000198
	White	Women	50	0.00011, 0.000131, 0.000155, 0.000184, 0.000219
	White	Women	51	0.000128, 0.000151, 0.000178, 0.000209, 0.000247
	White	Women	52	0.000146, 0.000172, 0.000203, 0.000239, 0.000282
	White	Women	53	0.000165, 0.000195, 0.000231, 0.000273, 0.000322
	White	Women	54	0.000185, 0.00022, 0.000261, 0.00031, 0.000368
	White	Women	55	0.000207, 0.000247, 0.000294, 0.00035, 0.000417
	White	Women	56	0.000231, 0.000276, 0.000329, 0.000393, 0.00047
	White	Women	57	0.000257, 0.000308, 0.000368, 0.00044, 0.000527
	White	Women	58	0.000287, 0.000344, 0.000411, 0.000491, 0.000589
	White	Women	59	0.00032, 0.000383, 0.000458, 0.000548, 0.000657
	White	Women	60	0.000353, 0.000424, 0.000509, 0.00061, 0.000733
	White	Women	61	0.000384, 0.000464, 0.00056, 0.000676, 0.000817
	White	Women	62	0.000414, 0.000503, 0.000611, 0.000741, 0.000901
	White	Women	63	0.000444, 0.000542, 0.000661, 0.000805, 0.000983
	White	Women	64	0.000475, 0.000582, 0.000711, 0.00087, 0.00106
	White	Women	65	0.000509, 0.000625, 0.000766, 0.000939, 0.00115
	White	Women	66	0.000548, 0.000674, 0.000828, 0.00102, 0.00125
	White	Women	67	0.000595, 0.000733, 0.000902, 0.00111, 0.00137
	White	Women	68	0.000652, 0.000804, 0.000991, 0.00122, 0.00151
	White	Women	69	0.000719, 0.000888, 0.00109, 0.00135, 0.00167
	White	Women	70	0.000797, 0.000985, 0.00122, 0.0015, 0.00185
	White	Women	71	0.000884, 0.00109, 0.00135, 0.00167, 0.00207
	White	Women	72	0.000979, 0.00122, 0.0015, 0.00186, 0.00231
	White	Women	73	0.00108, 0.00135, 0.00168, 0.00209, 0.0026
	White	Women	74	0.0012, 0.0015, 0.00187, 0.00234, 0.00293
	White	Women	75	0.00133, 0.00167, 0.0021, 0.00263, 0.0033
	White	Women	76	0.00148, 0.00187, 0.00235, 0.00297, 0.00374
	White	Women	77	0.00166, 0.0021, 0.00266, 0.00336, 0.00426
	White	Women	78	0.00186, 0.00237, 0.00301, 0.00383, 0.00488
	White	Women	79	0.00209, 0.00268, 0.00343, 0.00438, 0.00562
	White	Women	80	0.00237, 0.00305, 0.00392, 0.00504, 0.00649
	White	Women	81	0.00268, 0.00348, 0.00449, 0.0058, 0.00751
	White	Women	82	0.00303, 0.00395, 0.00513, 0.00666, 0.00867
	White	Women	83	0.00341, 0.00446, 0.00582, 0.0076, 0.00995
	White	Women	84	0.0038, 0.005, 0.00656, 0.0086, 0.0113
CHD mortality rates for 2031 (0.01, 0.2, 0.5, 0.8. 0.99 percentiles of the empirical distribution produced during forecasting)				
	Black	Men	30	0.0000265, 0.0000394, 0.0000584, 0.0000864, 0.000128
	Black	Men	31	0.0000342, 0.0000492, 0.0000704, 0.000101, 0.000145

Variable	Race/ethnicity	Sex	Age	Distribution
	Black	Men	32	0.0000435, 0.0000607, 0.0000843, 0.000117, 0.000164
	Black	Men	33	0.0000545, 0.0000739, 0.0001, 0.000135, 0.000184
	Black	Men	34	0.0000663, 0.0000883, 0.000117, 0.000156, 0.000207
	Black	Men	35	0.0000781, 0.000103, 0.000135, 0.000178, 0.000235
	Black	Men	36	0.0000893, 0.000118, 0.000154, 0.000202, 0.000266
	Black	Men	37	0.0001, 0.000132, 0.000173, 0.000228, 0.000299
	Black	Men	38	0.000112, 0.000147, 0.000193, 0.000253, 0.000333
	Black	Men	39	0.000124, 0.000162, 0.000213, 0.000278, 0.000365
	Black	Men	40	0.000137, 0.000179, 0.000233, 0.000304, 0.000397
	Black	Men	41	0.000152, 0.000197, 0.000254, 0.000329, 0.000426
	Black	Men	42	0.000169, 0.000217, 0.000278, 0.000355, 0.000456
	Black	Men	43	0.000187, 0.000239, 0.000305, 0.000388, 0.000496
	Black	Men	44	0.000205, 0.000263, 0.000336, 0.00043, 0.000552
	Black	Men	45	0.000226, 0.000291, 0.000374, 0.000481, 0.000619
	Black	Men	46	0.000252, 0.000326, 0.00042, 0.00054, 0.000698
	Black	Men	47	0.000286, 0.000369, 0.000476, 0.000613, 0.000792
	Black	Men	48	0.000327, 0.000422, 0.000544, 0.000702, 0.000907
	Black	Men	49	0.000377, 0.000488, 0.000629, 0.000811, 0.00105
	Black	Men	50	0.000441, 0.000568, 0.00073, 0.000938, 0.00121
	Black	Men	51	0.000522, 0.000667, 0.000849, 0.00108, 0.00138
	Black	Men	52	0.000619, 0.000782, 0.000984, 0.00124, 0.00156
	Black	Men	53	0.000725, 0.000907, 0.00113, 0.00141, 0.00177
	Black	Men	54	0.00083, 0.00103, 0.00129, 0.0016, 0.002
	Black	Men	55	0.000937, 0.00117, 0.00145, 0.0018, 0.00223
	Black	Men	56	0.00105, 0.0013, 0.00161, 0.00199, 0.00246
	Black	Men	57	0.00117, 0.00144, 0.00177, 0.00217, 0.00267
	Black	Men	58	0.00129, 0.00158, 0.00192, 0.00235, 0.00287
	Black	Men	59	0.00141, 0.00171, 0.00208, 0.00252, 0.00307
	Black	Men	60	0.00152, 0.00184, 0.00223, 0.00269, 0.00325
	Black	Men	61	0.00164, 0.00197, 0.00237, 0.00284, 0.00341
	Black	Men	62	0.00175, 0.00209, 0.00249, 0.00296, 0.00354
	Black	Men	63	0.00186, 0.0022, 0.0026, 0.00307, 0.00363
	Black	Men	64	0.00196, 0.00231, 0.00271, 0.00317, 0.00373
	Black	Men	65	0.00206, 0.00241, 0.00281, 0.00329, 0.00385
	Black	Men	66	0.00215, 0.00252, 0.00294, 0.00344, 0.00402
	Black	Men	67	0.00225, 0.00264, 0.0031, 0.00363, 0.00427
	Black	Men	68	0.00235, 0.00278, 0.00328, 0.00387, 0.00458
	Black	Men	69	0.00248, 0.00295, 0.0035, 0.00415, 0.00493
	Black	Men	70	0.00264, 0.00314, 0.00374, 0.00444, 0.00529
	Black	Men	71	0.00281, 0.00335, 0.00399, 0.00475, 0.00566
	Black	Men	72	0.003, 0.00357, 0.00426, 0.00507, 0.00605
	Black	Men	73	0.00319, 0.00381, 0.00454, 0.00542, 0.00647
	Black	Men	74	0.0034, 0.00406, 0.00485, 0.00578, 0.00691

Variable	Race/ethnicity	Sex	Age	Distribution
	Black	Men	75	0.00364, 0.00435, 0.00519, 0.00618, 0.00738
	Black	Men	76	0.00393, 0.00468, 0.00557, 0.00663, 0.00791
	Black	Men	77	0.00425, 0.00506, 0.00602, 0.00716, 0.00852
	Black	Men	78	0.00459, 0.00548, 0.00652, 0.00776, 0.00926
	Black	Men	79	0.00495, 0.00593, 0.00708, 0.00845, 0.0101
	Black	Men	80	0.00532, 0.0064, 0.00768, 0.00922, 0.0111
	Black	Men	81	0.00568, 0.00687, 0.00831, 0.01, 0.0122
	Black	Men	82	0.006, 0.00734, 0.00895, 0.0109, 0.0133
	Black	Men	83	0.00629, 0.00777, 0.00959, 0.0118, 0.0146
	Black	Men	84	0.00655, 0.00819, 0.0102, 0.0127, 0.0159
	Black	Women	30	0.0000112, 0.0000167, 0.0000248, 0.0000367, 0.0000546
	Black	Women	31	0.0000144, 0.0000206, 0.0000293, 0.0000418, 0.0000597
	Black	Women	32	0.0000181, 0.0000251, 0.0000347, 0.000048, 0.0000667
	Black	Women	33	0.0000222, 0.0000302, 0.0000409, 0.0000554, 0.0000754
	Black	Women	34	0.0000265, 0.0000357, 0.0000479, 0.0000642, 0.0000864
	Black	Women	35	0.0000306, 0.0000412, 0.0000554, 0.0000743, 0.0001
	Black	Women	36	0.0000346, 0.0000469, 0.0000632, 0.0000853, 0.000115
	Black	Women	37	0.0000388, 0.0000527, 0.0000714, 0.0000967, 0.000131
	Black	Women	38	0.0000431, 0.0000588, 0.0000799, 0.000109, 0.000148
	Black	Women	39	0.0000475, 0.0000651, 0.0000889, 0.000121, 0.000166
	Black	Women	40	0.0000526, 0.000072, 0.0000982, 0.000134, 0.000183
	Black	Women	41	0.0000593, 0.0000802, 0.000108, 0.000146, 0.000197
	Black	Women	42	0.000068, 0.00009, 0.000119, 0.000157, 0.000207
	Black	Women	43	0.0000783, 0.000101, 0.000131, 0.000169, 0.000219
	Black	Women	44	0.0000892, 0.000114, 0.000145, 0.000185, 0.000237
	Black	Women	45	0.000101, 0.000128, 0.000163, 0.000206, 0.000262
	Black	Women	46	0.000116, 0.000146, 0.000184, 0.000232, 0.000293
	Black	Women	47	0.000135, 0.000169, 0.000211, 0.000263, 0.00033
	Black	Women	48	0.000157, 0.000195, 0.000243, 0.000302, 0.000376
	Black	Women	49	0.00018, 0.000225, 0.000282, 0.000353, 0.000442
	Black	Women	50	0.000206, 0.00026, 0.000327, 0.000411, 0.000519
	Black	Women	51	0.000241, 0.000302, 0.000377, 0.00047, 0.000588
	Black	Women	52	0.000285, 0.000351, 0.000431, 0.00053, 0.000652
	Black	Women	53	0.000326, 0.0004, 0.00049, 0.000599, 0.000735
	Black	Women	54	0.000363, 0.000448, 0.000553, 0.000681, 0.000842
	Black	Women	55	0.000406, 0.000503, 0.000621, 0.000768, 0.00095
	Black	Women	56	0.00046, 0.000566, 0.000695, 0.000853, 0.00105
	Black	Women	57	0.000518, 0.000633, 0.000772, 0.000943, 0.00115
	Black	Women	58	0.000572, 0.000699, 0.000852, 0.00104, 0.00127
	Black	Women	59	0.00062, 0.000761, 0.000932, 0.00114, 0.0014
	Black	Women	60	0.000663, 0.00082, 0.00101, 0.00125, 0.00154
	Black	Women	61	0.000703, 0.000874, 0.00109, 0.00135, 0.00168
	Black	Women	62	0.000735, 0.000923, 0.00116, 0.00145, 0.00182

Variable	Race/ethnicity	Sex	Age	Distribution
	Black	Women	63	0.000761, 0.000965, 0.00122, 0.00155, 0.00196
	Black	Women	64	0.000782, 0.001, 0.00128, 0.00164, 0.00211
	Black	Women	65	0.000805, 0.00104, 0.00135, 0.00175, 0.00227
	Black	Women	66	0.000835, 0.00109, 0.00143, 0.00187, 0.00245
	Black	Women	67	0.00088, 0.00116, 0.00153, 0.00201, 0.00266
	Black	Women	68	0.000944, 0.00125, 0.00165, 0.00218, 0.00289
	Black	Women	69	0.00103, 0.00136, 0.0018, 0.00237, 0.00314
	Black	Women	70	0.00113, 0.00149, 0.00196, 0.00258, 0.00341
	Black	Women	71	0.00125, 0.00164, 0.00214, 0.00281, 0.00368
	Black	Women	72	0.00137, 0.00179, 0.00233, 0.00305, 0.00399
	Black	Women	73	0.00149, 0.00195, 0.00254, 0.00331, 0.00432
	Black	Women	74	0.00163, 0.00212, 0.00276, 0.0036, 0.00469
	Black	Women	75	0.00177, 0.00231, 0.00301, 0.00393, 0.00513
	Black	Women	76	0.00192, 0.00252, 0.0033, 0.00432, 0.00567
	Black	Women	77	0.00207, 0.00274, 0.00362, 0.00478, 0.00633
	Black	Women	78	0.00223, 0.00298, 0.00399, 0.00533, 0.00714
	Black	Women	79	0.00238, 0.00324, 0.0044, 0.00596, 0.0081
	Black	Women	80	0.00255, 0.00352, 0.00485, 0.00667, 0.00921
	Black	Women	81	0.00273, 0.00382, 0.00534, 0.00745, 0.0104
	Black	Women	82	0.00294, 0.00416, 0.00586, 0.00826, 0.0117
	Black	Women	83	0.00318, 0.00453, 0.00642, 0.0091, 0.0129
	Black	Women	84	0.00344, 0.00491, 0.00699, 0.00995, 0.0142
	Hispanic	Men	30	0.00000915, 0.0000135, 0.00002, 0.0000294, 0.0000435
	Hispanic	Men	31	0.0000115, 0.0000166, 0.0000238, 0.0000341, 0.0000492
	Hispanic	Men	32	0.0000143, 0.0000202, 0.0000283, 0.0000397, 0.0000558
	Hispanic	Men	33	0.0000178, 0.0000244, 0.0000335, 0.0000459, 0.000063
	Hispanic	Men	34	0.0000219, 0.0000294, 0.0000394, 0.0000527, 0.0000708
	Hispanic	Men	35	0.0000265, 0.0000349, 0.0000459, 0.0000603, 0.0000795
	Hispanic	Men	36	0.0000312, 0.0000407, 0.0000529, 0.0000688, 0.0000897
	Hispanic	Men	37	0.0000361, 0.0000468, 0.0000605, 0.0000782, 0.000101
	Hispanic	Men	38	0.0000413, 0.0000534, 0.0000687, 0.0000886, 0.000114
	Hispanic	Men	39	0.0000472, 0.0000607, 0.0000778, 0.0000997, 0.000128
	Hispanic	Men	40	0.0000544, 0.0000691, 0.0000877, 0.000111, 0.000141
	Hispanic	Men	41	0.0000632, 0.0000791, 0.0000987, 0.000123, 0.000154
	Hispanic	Men	42	0.0000739, 0.0000906, 0.000111, 0.000136, 0.000167
	Hispanic	Men	43	0.0000861, 0.000104, 0.000125, 0.00015, 0.000181
	Hispanic	Men	44	0.0000993, 0.000119, 0.000141, 0.000168, 0.0002
	Hispanic	Men	45	0.000114, 0.000135, 0.00016, 0.00019, 0.000225
	Hispanic	Men	46	0.00013, 0.000154, 0.000183, 0.000216, 0.000256
	Hispanic	Men	47	0.00015, 0.000178, 0.00021, 0.000247, 0.000292
	Hispanic	Men	48	0.000176, 0.000207, 0.000242, 0.000283, 0.000332
	Hispanic	Men	49	0.000208, 0.000242, 0.000281, 0.000327, 0.00038
	Hispanic	Men	50	0.000243, 0.000283, 0.000328, 0.000381, 0.000442

Variable	Race/ethnicity	Sex	Age	Distribution
	Hispanic	Men	51	0.000283, 0.00033, 0.000383, 0.000445, 0.000518
	Hispanic	Men	52	0.000327, 0.000382, 0.000446, 0.00052, 0.000607
	Hispanic	Men	53	0.000372, 0.000438, 0.000515, 0.000605, 0.000713
	Hispanic	Men	54	0.000417, 0.000496, 0.000589, 0.000699, 0.000831
	Hispanic	Men	55	0.000469, 0.000559, 0.000666, 0.000794, 0.000947
	Hispanic	Men	56	0.000527, 0.000628, 0.000746, 0.000887, 0.00106
	Hispanic	Men	57	0.000591, 0.0007, 0.000828, 0.00098, 0.00116
	Hispanic	Men	58	0.000655, 0.000774, 0.000912, 0.00108, 0.00127
	Hispanic	Men	59	0.000718, 0.000847, 0.000997, 0.00117, 0.00139
	Hispanic	Men	60	0.000778, 0.000918, 0.00108, 0.00127, 0.00151
	Hispanic	Men	61	0.000834, 0.000986, 0.00116, 0.00137, 0.00162
	Hispanic	Men	62	0.000888, 0.00105, 0.00124, 0.00147, 0.00174
	Hispanic	Men	63	0.000939, 0.00111, 0.00132, 0.00156, 0.00185
	Hispanic	Men	64	0.000991, 0.00118, 0.00139, 0.00165, 0.00196
	Hispanic	Men	65	0.00105, 0.00124, 0.00147, 0.00174, 0.00207
	Hispanic	Men	66	0.0011, 0.00131, 0.00156, 0.00185, 0.00221
	Hispanic	Men	67	0.00117, 0.00139, 0.00166, 0.00199, 0.00238
	Hispanic	Men	68	0.00123, 0.00149, 0.00179, 0.00215, 0.00259
	Hispanic	Men	69	0.00131, 0.0016, 0.00194, 0.00235, 0.00285
	Hispanic	Men	70	0.00141, 0.00172, 0.00211, 0.00257, 0.00315
	Hispanic	Men	71	0.00152, 0.00187, 0.0023, 0.00283, 0.00349
	Hispanic	Men	72	0.00164, 0.00203, 0.00251, 0.00311, 0.00385
	Hispanic	Men	73	0.00178, 0.00222, 0.00275, 0.00341, 0.00424
	Hispanic	Men	74	0.00195, 0.00242, 0.00301, 0.00373, 0.00464
	Hispanic	Men	75	0.00214, 0.00266, 0.0033, 0.00409, 0.00508
	Hispanic	Men	76	0.00236, 0.00293, 0.00363, 0.00449, 0.00558
	Hispanic	Men	77	0.00263, 0.00325, 0.00402, 0.00496, 0.00614
	Hispanic	Men	78	0.00294, 0.00363, 0.00447, 0.0055, 0.0068
	Hispanic	Men	79	0.00327, 0.00404, 0.00498, 0.00613, 0.00756
	Hispanic	Men	80	0.00361, 0.00448, 0.00553, 0.00684, 0.00847
	Hispanic	Men	81	0.00393, 0.00491, 0.00612, 0.00763, 0.00953
	Hispanic	Men	82	0.00421, 0.00533, 0.00672, 0.00848, 0.0107
	Hispanic	Men	83	0.00447, 0.00573, 0.00732, 0.00936, 0.012
	Hispanic	Men	84	0.00473, 0.00613, 0.00792, 0.0102, 0.0133
	Hispanic	Women	30	0.00000248, 0.00000377, 0.00000572, 0.00000866, 0.0000132
	Hispanic	Women	31	0.00000306, 0.00000452, 0.00000665, 0.00000978, 0.0000145
	Hispanic	Women	32	0.00000375, 0.0000054, 0.00000774, 0.0000111, 0.000016
	Hispanic	Women	33	0.00000454, 0.0000064, 0.000009, 0.0000126, 0.0000178
	Hispanic	Women	34	0.00000539, 0.0000075, 0.0000104, 0.0000144, 0.00002
	Hispanic	Women	35	0.00000624, 0.00000863, 0.0000119, 0.0000164, 0.0000227
	Hispanic	Women	36	0.00000708, 0.0000098, 0.0000135, 0.0000187, 0.0000259
	Hispanic	Women	37	0.00000794, 0.000011, 0.0000152, 0.0000211, 0.0000292
	Hispanic	Women	38	0.00000886, 0.0000123, 0.0000171, 0.0000236, 0.0000329

Variable	Race/ethnicity	Sex	Age	Distribution
	Hispanic	Women	39	0.00000991, 0.0000138, 0.000019, 0.0000263, 0.0000365
	Hispanic	Women	40	0.0000112, 0.0000154, 0.0000212, 0.0000291, 0.00004
	Hispanic	Women	41	0.0000129, 0.0000175, 0.0000236, 0.0000318, 0.0000431
	Hispanic	Women	42	0.0000151, 0.00002, 0.0000263, 0.0000346, 0.0000457
	Hispanic	Women	43	0.0000179, 0.000023, 0.0000295, 0.0000379, 0.0000488
	Hispanic	Women	44	0.000021, 0.0000265, 0.0000334, 0.0000421, 0.0000531
	Hispanic	Women	45	0.0000247, 0.0000307, 0.0000381, 0.0000472, 0.0000586
	Hispanic	Women	46	0.0000295, 0.000036, 0.0000438, 0.0000533, 0.0000649
	Hispanic	Women	47	0.0000355, 0.0000425, 0.0000507, 0.0000605, 0.0000724
	Hispanic	Women	48	0.0000424, 0.0000501, 0.0000591, 0.0000696, 0.0000822
	Hispanic	Women	49	0.0000499, 0.0000588, 0.0000692, 0.0000814, 0.0000959
	Hispanic	Women	50	0.0000577, 0.0000686, 0.0000814, 0.0000967, 0.000115
	Hispanic	Women	51	0.0000662, 0.0000798, 0.0000961, 0.000116, 0.000139
	Hispanic	Women	52	0.0000759, 0.0000928, 0.000113, 0.000138, 0.000169
	Hispanic	Women	53	0.0000868, 0.000108, 0.000133, 0.000165, 0.000205
	Hispanic	Women	54	0.0000994, 0.000125, 0.000156, 0.000196, 0.000246
	Hispanic	Women	55	0.000114, 0.000144, 0.000182, 0.00023, 0.000291
	Hispanic	Women	56	0.000131, 0.000167, 0.000211, 0.000268, 0.000341
	Hispanic	Women	57	0.00015, 0.000191, 0.000244, 0.000311, 0.000398
	Hispanic	Women	58	0.000169, 0.000218, 0.00028, 0.00036, 0.000463
	Hispanic	Women	59	0.00019, 0.000247, 0.00032, 0.000414, 0.000538
	Hispanic	Women	60	0.000211, 0.000277, 0.000362, 0.000473, 0.00062
	Hispanic	Women	61	0.000233, 0.000308, 0.000406, 0.000535, 0.000708
	Hispanic	Women	62	0.000255, 0.000339, 0.00045, 0.000598, 0.000795
	Hispanic	Women	63	0.000279, 0.000372, 0.000495, 0.000658, 0.000878
	Hispanic	Women	64	0.000306, 0.000407, 0.00054, 0.000718, 0.000956
	Hispanic	Women	65	0.000336, 0.000446, 0.000589, 0.000779, 0.00103
	Hispanic	Women	66	0.000371, 0.00049, 0.000644, 0.000848, 0.00112
	Hispanic	Women	67	0.000411, 0.00054, 0.000709, 0.00093, 0.00122
	Hispanic	Women	68	0.000456, 0.0006, 0.000786, 0.00103, 0.00135
	Hispanic	Women	69	0.000509, 0.000668, 0.000876, 0.00115, 0.00151
	Hispanic	Women	70	0.00057, 0.000748, 0.000979, 0.00128, 0.00168
	Hispanic	Women	71	0.000639, 0.000838, 0.0011, 0.00143, 0.00188
	Hispanic	Women	72	0.000717, 0.00094, 0.00123, 0.00161, 0.00211
	Hispanic	Women	73	0.000804, 0.00105, 0.00138, 0.0018, 0.00236
	Hispanic	Women	74	0.000901, 0.00118, 0.00155, 0.00203, 0.00267
	Hispanic	Women	75	0.00101, 0.00133, 0.00175, 0.0023, 0.00303
	Hispanic	Women	76	0.00113, 0.0015, 0.00198, 0.00262, 0.00346
	Hispanic	Women	77	0.00127, 0.0017, 0.00225, 0.00299, 0.00399
	Hispanic	Women	78	0.00143, 0.00192, 0.00257, 0.00344, 0.00461
	Hispanic	Women	79	0.00162, 0.00218, 0.00294, 0.00395, 0.00534
	Hispanic	Women	80	0.00182, 0.00247, 0.00336, 0.00455, 0.0062
	Hispanic	Women	81	0.00204, 0.0028, 0.00383, 0.00524, 0.00719

Variable	Race/ethnicity	Sex	Age	Distribution
	Hispanic	Women	82	0.00226, 0.00314, 0.00435, 0.00602, 0.00835
	Hispanic	Women	83	0.00249, 0.0035, 0.0049, 0.00687, 0.00967
	Hispanic	Women	84	0.00271, 0.00386, 0.00548, 0.00779, 0.0111
	White	Men	30	0.0000161, 0.0000233, 0.0000334, 0.0000479, 0.000069
	White	Men	31	0.0000211, 0.0000295, 0.0000411, 0.0000574, 0.0000802
	White	Men	32	0.000027, 0.0000369, 0.0000503, 0.0000686, 0.0000938
	White	Men	33	0.0000338, 0.0000454, 0.0000609, 0.0000818, 0.00011
	White	Men	34	0.000041, 0.0000547, 0.0000728, 0.000097, 0.000129
	White	Men	35	0.0000484, 0.0000645, 0.0000858, 0.000114, 0.000152
	White	Men	36	0.0000562, 0.000075, 0.0000998, 0.000133, 0.000177
	White	Men	37	0.0000648, 0.0000864, 0.000115, 0.000153, 0.000203
	White	Men	38	0.0000751, 0.0000994, 0.000131, 0.000173, 0.000229
	White	Men	39	0.0000874, 0.000114, 0.000149, 0.000194, 0.000253
	White	Men	40	0.000102, 0.000131, 0.000167, 0.000214, 0.000275
	White	Men	41	0.00012, 0.00015, 0.000187, 0.000234, 0.000294
	White	Men	42	0.00014, 0.000171, 0.000209, 0.000255, 0.000312
	White	Men	43	0.000161, 0.000193, 0.000232, 0.000278, 0.000334
	White	Men	44	0.000183, 0.000218, 0.000258, 0.000305, 0.000362
	White	Men	45	0.00021, 0.000246, 0.000288, 0.000337, 0.000396
	White	Men	46	0.000242, 0.00028, 0.000324, 0.000374, 0.000433
	White	Men	47	0.00028, 0.00032, 0.000366, 0.000418, 0.000478
	White	Men	48	0.000323, 0.000367, 0.000416, 0.000472, 0.000536
	White	Men	49	0.000372, 0.000421, 0.000475, 0.000537, 0.000607
	White	Men	50	0.00043, 0.000484, 0.000544, 0.000611, 0.000687
	White	Men	51	0.000499, 0.000557, 0.000621, 0.000692, 0.000772
	White	Men	52	0.000575, 0.000637, 0.000705, 0.000781, 0.000866
	White	Men	53	0.000645, 0.000717, 0.000796, 0.000884, 0.000982
	White	Men	54	0.000708, 0.000794, 0.00089, 0.000998, 0.00112
	White	Men	55	0.00077, 0.000872, 0.000986, 0.00112, 0.00126
	White	Men	56	0.000834, 0.000951, 0.00108, 0.00123, 0.00141
	White	Men	57	0.000895, 0.00103, 0.00118, 0.00135, 0.00155
	White	Men	58	0.000956, 0.00111, 0.00128, 0.00147, 0.0017
	White	Men	59	0.00102, 0.00118, 0.00137, 0.00159, 0.00185
	White	Men	60	0.00109, 0.00127, 0.00147, 0.00171, 0.00199
	White	Men	61	0.00116, 0.00135, 0.00157, 0.00182, 0.00213
	White	Men	62	0.00122, 0.00142, 0.00166, 0.00193, 0.00225
	White	Men	63	0.00128, 0.00149, 0.00174, 0.00203, 0.00238
	White	Men	64	0.00133, 0.00156, 0.00182, 0.00213, 0.00249
	White	Men	65	0.00138, 0.00162, 0.0019, 0.00223, 0.00262
	White	Men	66	0.00143, 0.00169, 0.00199, 0.00235, 0.00278
	White	Men	67	0.00148, 0.00177, 0.00211, 0.00251, 0.00299
	White	Men	68	0.00155, 0.00186, 0.00224, 0.0027, 0.00326
	White	Men	69	0.00163, 0.00199, 0.00241, 0.00293, 0.00356

Variable	Race/ethnicity	Sex	Age	Distribution
	White	Men	70	0.00175, 0.00214, 0.00261, 0.00318, 0.00389
	White	Men	71	0.0019, 0.00232, 0.00284, 0.00346, 0.00424
	White	Men	72	0.00206, 0.00253, 0.00309, 0.00377, 0.00462
	White	Men	73	0.00225, 0.00275, 0.00336, 0.00411, 0.00504
	White	Men	74	0.00245, 0.003, 0.00367, 0.00448, 0.00549
	White	Men	75	0.00267, 0.00327, 0.004, 0.0049, 0.00601
	White	Men	76	0.00291, 0.00358, 0.00439, 0.00538, 0.00662
	White	Men	77	0.00318, 0.00393, 0.00484, 0.00596, 0.00735
	White	Men	78	0.00351, 0.00434, 0.00537, 0.00664, 0.00823
	White	Men	79	0.00389, 0.00484, 0.006, 0.00745, 0.00926
	White	Men	80	0.00435, 0.00542, 0.00674, 0.00837, 0.0104
	White	Men	81	0.00488, 0.00608, 0.00757, 0.00941, 0.0117
	White	Men	82	0.00547, 0.00682, 0.00848, 0.0105, 0.0131
	White	Men	83	0.00609, 0.00759, 0.00944, 0.0117, 0.0146
	White	Men	84	0.00672, 0.00837, 0.0104, 0.013, 0.0161
	White	Women	30	0.00000435, 0.00000688, 0.0000108, 0.0000171, 0.000027
	White	Women	31	0.00000577, 0.00000877, 0.0000133, 0.0000201, 0.0000306
	White	Women	32	0.00000763, 0.0000111, 0.0000162, 0.0000236, 0.0000345
	White	Women	33	0.00000995, 0.000014, 0.0000196, 0.0000275, 0.0000387
	White	Women	34	0.0000126, 0.0000172, 0.0000235, 0.0000321, 0.0000439
	White	Women	35	0.0000155, 0.0000208, 0.0000278, 0.0000372, 0.00005
	White	Women	36	0.0000184, 0.0000244, 0.0000324, 0.000043, 0.0000572
	White	Women	37	0.0000213, 0.0000282, 0.0000372, 0.0000492, 0.0000653
	White	Women	38	0.000024, 0.0000319, 0.0000423, 0.000056, 0.0000744
	White	Women	39	0.0000266, 0.0000356, 0.0000474, 0.0000632, 0.0000844
	White	Women	40	0.0000293, 0.0000393, 0.0000526, 0.0000704, 0.0000945
	White	Women	41	0.0000323, 0.0000433, 0.0000578, 0.0000773, 0.000104
	White	Women	42	0.0000358, 0.0000477, 0.0000633, 0.000084, 0.000112
	White	Women	43	0.0000397, 0.0000525, 0.0000692, 0.0000913, 0.000121
	White	Women	44	0.0000436, 0.0000576, 0.0000759, 0.0001, 0.000132
	White	Women	45	0.0000484, 0.0000637, 0.0000838, 0.00011, 0.000145
	White	Women	46	0.0000549, 0.0000716, 0.000093, 0.000121, 0.000158
	White	Women	47	0.0000642, 0.0000818, 0.000104, 0.000132, 0.000169
	White	Women	48	0.0000764, 0.0000948, 0.000117, 0.000145, 0.00018
	White	Women	49	0.0000911, 0.00011, 0.000133, 0.000161, 0.000195
	White	Women	50	0.000107, 0.000128, 0.000152, 0.000181, 0.000215
	White	Women	51	0.000125, 0.000147, 0.000174, 0.000205, 0.000243
	White	Women	52	0.000143, 0.000169, 0.000199, 0.000235, 0.000277
	White	Women	53	0.000162, 0.000192, 0.000227, 0.000268, 0.000318
	White	Women	54	0.000182, 0.000216, 0.000257, 0.000305, 0.000363
	White	Women	55	0.000203, 0.000242, 0.000289, 0.000345, 0.000412
	White	Women	56	0.000226, 0.000271, 0.000324, 0.000388, 0.000465
	White	Women	57	0.000252, 0.000303, 0.000363, 0.000434, 0.000521

Variable	Race/ethnicity	Sex	Age	Distribution
	White	Women	58	0.000282, 0.000338, 0.000405, 0.000485, 0.000583
	White	Women	59	0.000314, 0.000377, 0.000452, 0.000541, 0.00065
	White	Women	60	0.000345, 0.000416, 0.000501, 0.000602, 0.000726
	White	Women	61	0.000376, 0.000455, 0.000551, 0.000667, 0.000808
	White	Women	62	0.000405, 0.000494, 0.0006, 0.00073, 0.00089
	White	Women	63	0.000434, 0.000531, 0.000649, 0.000792, 0.000969
	White	Women	64	0.000464, 0.000569, 0.000697, 0.000854, 0.00105
	White	Women	65	0.000496, 0.000611, 0.00075, 0.000921, 0.00113
	White	Women	66	0.000534, 0.000658, 0.00081, 0.000997, 0.00123
	White	Women	67	0.000579, 0.000715, 0.000882, 0.00109, 0.00134
	White	Women	68	0.000634, 0.000784, 0.000968, 0.00119, 0.00148
	White	Women	69	0.0007, 0.000866, 0.00107, 0.00132, 0.00164
	White	Women	70	0.000775, 0.00096, 0.00119, 0.00147, 0.00182
	White	Women	71	0.00086, 0.00107, 0.00132, 0.00163, 0.00203
	White	Women	72	0.000952, 0.00118, 0.00147, 0.00182, 0.00227
	White	Women	73	0.00105, 0.00131, 0.00164, 0.00204, 0.00255
	White	Women	74	0.00116, 0.00146, 0.00183, 0.00229, 0.00287
	White	Women	75	0.00129, 0.00163, 0.00204, 0.00257, 0.00323
	White	Women	76	0.00144, 0.00182, 0.0023, 0.0029, 0.00366
	White	Women	77	0.00161, 0.00204, 0.00259, 0.00328, 0.00417
	White	Women	78	0.00181, 0.00231, 0.00294, 0.00374, 0.00478
	White	Women	79	0.00203, 0.00261, 0.00335, 0.00428, 0.0055
	White	Women	80	0.0023, 0.00297, 0.00383, 0.00493, 0.00636
	White	Women	81	0.00261, 0.00338, 0.00438, 0.00567, 0.00736
	White	Women	82	0.00294, 0.00384, 0.005, 0.00651, 0.00849
	White	Women	83	0.00331, 0.00434, 0.00568, 0.00743, 0.00975
	White	Women	84	0.00369, 0.00486, 0.00639, 0.00841, 0.0111
CHD mortality rates for 2032 (0.01, 0.2, 0.5, 0.8. 0.99 percentiles of the empirical distribution produced during forecasting)				
	Black	Men	30	0.000026, 0.0000389, 0.0000581, 0.0000866, 0.00013
	Black	Men	31	0.0000336, 0.0000486, 0.0000701, 0.000101, 0.000146
	Black	Men	32	0.0000428, 0.00006, 0.000084, 0.000117, 0.000165
	Black	Men	33	0.0000536, 0.0000732, 0.0000996, 0.000136, 0.000185
	Black	Men	34	0.0000653, 0.0000874, 0.000117, 0.000156, 0.000209
	Black	Men	35	0.0000768, 0.000102, 0.000135, 0.000178, 0.000237
	Black	Men	36	0.0000877, 0.000116, 0.000153, 0.000202, 0.000268
	Black	Men	37	0.0000985, 0.00013, 0.000172, 0.000227, 0.000301
	Black	Men	38	0.000109, 0.000145, 0.000191, 0.000252, 0.000334
	Black	Men	39	0.000121, 0.000159, 0.00021, 0.000277, 0.000366
	Black	Men	40	0.000133, 0.000175, 0.00023, 0.000301, 0.000395
	Black	Men	41	0.000148, 0.000193, 0.00025, 0.000325, 0.000423
	Black	Men	42	0.000165, 0.000212, 0.000273, 0.00035, 0.000451
	Black	Men	43	0.000182, 0.000233, 0.000298, 0.000381, 0.000489
	Black	Men	44	0.000199, 0.000256, 0.000329, 0.000422, 0.000542

Variable	Race/ethnicity	Sex	Age	Distribution
	Black	Men	45	0.00022, 0.000284, 0.000365, 0.000471, 0.000608
	Black	Men	46	0.000246, 0.000318, 0.00041, 0.000529, 0.000684
	Black	Men	47	0.000278, 0.00036, 0.000465, 0.000599, 0.000776
	Black	Men	48	0.000319, 0.000412, 0.000532, 0.000687, 0.000889
	Black	Men	49	0.000368, 0.000476, 0.000615, 0.000794, 0.00103
	Black	Men	50	0.000431, 0.000556, 0.000715, 0.00092, 0.00119
	Black	Men	51	0.000511, 0.000653, 0.000833, 0.00106, 0.00136
	Black	Men	52	0.000607, 0.000767, 0.000967, 0.00122, 0.00154
	Black	Men	53	0.000711, 0.000891, 0.00111, 0.00139, 0.00174
	Black	Men	54	0.000815, 0.00102, 0.00127, 0.00158, 0.00197
	Black	Men	55	0.00092, 0.00115, 0.00143, 0.00177, 0.00221
	Black	Men	56	0.00103, 0.00128, 0.00158, 0.00196, 0.00243
	Black	Men	57	0.00115, 0.00142, 0.00174, 0.00214, 0.00264
	Black	Men	58	0.00127, 0.00155, 0.0019, 0.00232, 0.00284
	Black	Men	59	0.00138, 0.00168, 0.00205, 0.00249, 0.00304
	Black	Men	60	0.00149, 0.00181, 0.00219, 0.00266, 0.00322
	Black	Men	61	0.00161, 0.00194, 0.00233, 0.0028, 0.00338
	Black	Men	62	0.00171, 0.00205, 0.00245, 0.00292, 0.0035
	Black	Men	63	0.00182, 0.00216, 0.00255, 0.00302, 0.00359
	Black	Men	64	0.00192, 0.00226, 0.00265, 0.00312, 0.00367
	Black	Men	65	0.00201, 0.00235, 0.00276, 0.00323, 0.00379
	Black	Men	66	0.0021, 0.00246, 0.00288, 0.00337, 0.00395
	Black	Men	67	0.00219, 0.00257, 0.00303, 0.00356, 0.00419
	Black	Men	68	0.00229, 0.00271, 0.00321, 0.0038, 0.0045
	Black	Men	69	0.00241, 0.00287, 0.00342, 0.00406, 0.00484
	Black	Men	70	0.00256, 0.00306, 0.00365, 0.00435, 0.0052
	Black	Men	71	0.00273, 0.00327, 0.0039, 0.00465, 0.00556
	Black	Men	72	0.00291, 0.00348, 0.00416, 0.00497, 0.00594
	Black	Men	73	0.00309, 0.00371, 0.00443, 0.0053, 0.00635
	Black	Men	74	0.0033, 0.00395, 0.00473, 0.00566, 0.00678
	Black	Men	75	0.00353, 0.00423, 0.00506, 0.00605, 0.00724
	Black	Men	76	0.00381, 0.00456, 0.00544, 0.00649, 0.00775
	Black	Men	77	0.00412, 0.00492, 0.00587, 0.007, 0.00836
	Black	Men	78	0.00445, 0.00533, 0.00636, 0.00759, 0.00908
	Black	Men	79	0.0048, 0.00576, 0.00691, 0.00827, 0.00992
	Black	Men	80	0.00516, 0.00622, 0.00749, 0.00902, 0.0109
	Black	Men	81	0.00551, 0.00669, 0.00811, 0.00983, 0.0119
	Black	Men	82	0.00582, 0.00714, 0.00873, 0.0107, 0.0131
	Black	Men	83	0.00609, 0.00756, 0.00935, 0.0116, 0.0143
	Black	Men	84	0.00635, 0.00796, 0.00996, 0.0125, 0.0156
	Black	Women	30	0.000011, 0.0000165, 0.0000247, 0.0000368, 0.0000552
	Black	Women	31	0.0000142, 0.0000204, 0.0000292, 0.0000419, 0.0000602
	Black	Women	32	0.0000178, 0.0000248, 0.0000346, 0.0000481, 0.0000672

Variable	Race/ethnicity	Sex	Age	Distribution
	Black	Women	33	0.0000219, 0.0000299, 0.0000408, 0.0000556, 0.000076
	Black	Women	34	0.0000261, 0.0000354, 0.0000477, 0.0000643, 0.000087
	Black	Women	35	0.0000302, 0.0000409, 0.0000552, 0.0000744, 0.000101
	Black	Women	36	0.0000341, 0.0000464, 0.0000629, 0.0000854, 0.000116
	Black	Women	37	0.0000381, 0.0000521, 0.000071, 0.0000967, 0.000132
	Black	Women	38	0.0000423, 0.000058, 0.0000793, 0.000108, 0.000149
	Black	Women	39	0.0000465, 0.0000641, 0.000088, 0.000121, 0.000166
	Black	Women	40	0.0000515, 0.0000708, 0.000097, 0.000133, 0.000183
	Black	Women	41	0.000058, 0.0000787, 0.000107, 0.000144, 0.000196
	Black	Women	42	0.0000665, 0.0000883, 0.000117, 0.000155, 0.000205
	Black	Women	43	0.0000765, 0.0000993, 0.000129, 0.000167, 0.000216
	Black	Women	44	0.000087, 0.000111, 0.000143, 0.000182, 0.000234
	Black	Women	45	0.0000984, 0.000125, 0.000159, 0.000203, 0.000258
	Black	Women	46	0.000113, 0.000143, 0.00018, 0.000228, 0.000289
	Black	Women	47	0.000131, 0.000165, 0.000206, 0.000258, 0.000324
	Black	Women	48	0.000153, 0.000191, 0.000238, 0.000296, 0.00037
	Black	Women	49	0.000175, 0.00022, 0.000276, 0.000346, 0.000435
	Black	Women	50	0.000201, 0.000254, 0.000321, 0.000404, 0.000511
	Black	Women	51	0.000236, 0.000296, 0.00037, 0.000463, 0.00058
	Black	Women	52	0.000279, 0.000345, 0.000424, 0.000522, 0.000644
	Black	Women	53	0.00032, 0.000393, 0.000482, 0.000591, 0.000726
	Black	Women	54	0.000357, 0.000441, 0.000545, 0.000673, 0.000833
	Black	Women	55	0.000399, 0.000495, 0.000613, 0.000759, 0.000941
	Black	Women	56	0.000452, 0.000557, 0.000686, 0.000844, 0.00104
	Black	Women	57	0.000509, 0.000624, 0.000763, 0.000933, 0.00114
	Black	Women	58	0.000562, 0.000688, 0.000842, 0.00103, 0.00126
	Black	Women	59	0.000609, 0.00075, 0.000921, 0.00113, 0.00139
	Black	Women	60	0.000652, 0.000807, 0.000998, 0.00123, 0.00153
	Black	Women	61	0.000689, 0.00086, 0.00107, 0.00133, 0.00166
	Black	Women	62	0.000721, 0.000907, 0.00114, 0.00143, 0.0018
	Black	Women	63	0.000745, 0.000947, 0.0012, 0.00152, 0.00194
	Black	Women	64	0.000765, 0.000984, 0.00126, 0.00162, 0.00208
	Black	Women	65	0.000787, 0.00102, 0.00133, 0.00172, 0.00224
	Black	Women	66	0.000816, 0.00107, 0.0014, 0.00184, 0.00242
	Black	Women	67	0.000859, 0.00114, 0.0015, 0.00198, 0.00262
	Black	Women	68	0.000921, 0.00122, 0.00162, 0.00214, 0.00284
	Black	Women	69	0.001, 0.00133, 0.00176, 0.00233, 0.00309
	Black	Women	70	0.0011, 0.00146, 0.00192, 0.00253, 0.00335
	Black	Women	71	0.00121, 0.0016, 0.0021, 0.00275, 0.00362
	Black	Women	72	0.00133, 0.00175, 0.00228, 0.00299, 0.00392
	Black	Women	73	0.00145, 0.0019, 0.00248, 0.00324, 0.00424
	Black	Women	74	0.00158, 0.00207, 0.0027, 0.00352, 0.00461
	Black	Women	75	0.00172, 0.00225, 0.00295, 0.00385, 0.00504

Variable	Race/ethnicity	Sex	Age	Distribution
	Black	Women	76	0.00187, 0.00246, 0.00322, 0.00423, 0.00556
	Black	Women	77	0.00202, 0.00268, 0.00354, 0.00468, 0.00622
	Black	Women	78	0.00217, 0.00291, 0.0039, 0.00522, 0.00701
	Black	Women	79	0.00232, 0.00316, 0.0043, 0.00584, 0.00796
	Black	Women	80	0.00248, 0.00344, 0.00474, 0.00654, 0.00905
	Black	Women	81	0.00266, 0.00373, 0.00522, 0.0073, 0.0102
	Black	Women	82	0.00286, 0.00406, 0.00574, 0.0081, 0.0115
	Black	Women	83	0.0031, 0.00442, 0.00628, 0.00892, 0.0127
	Black	Women	84	0.00334, 0.00479, 0.00684, 0.00976, 0.014
	Hispanic	Men	30	0.00000898, 0.0000134, 0.0000199, 0.0000295, 0.0000439
	Hispanic	Men	31	0.0000113, 0.0000164, 0.0000237, 0.0000342, 0.0000496
	Hispanic	Men	32	0.0000141, 0.00002, 0.0000282, 0.0000397, 0.0000563
	Hispanic	Men	33	0.0000175, 0.0000242, 0.0000334, 0.000046, 0.0000635
	Hispanic	Men	34	0.0000216, 0.0000291, 0.0000392, 0.0000528, 0.0000713
	Hispanic	Men	35	0.0000261, 0.0000345, 0.0000457, 0.0000604, 0.0000801
	Hispanic	Men	36	0.0000307, 0.0000402, 0.0000526, 0.0000688, 0.0000903
	Hispanic	Men	37	0.0000354, 0.0000462, 0.0000601, 0.0000782, 0.000102
	Hispanic	Men	38	0.0000404, 0.0000525, 0.0000681, 0.0000883, 0.000115
	Hispanic	Men	39	0.0000461, 0.0000596, 0.0000769, 0.0000992, 0.000128
	Hispanic	Men	40	0.000053, 0.0000678, 0.0000865, 0.00011, 0.000141
	Hispanic	Men	41	0.0000616, 0.0000774, 0.0000971, 0.000122, 0.000153
	Hispanic	Men	42	0.000072, 0.0000887, 0.000109, 0.000134, 0.000165
	Hispanic	Men	43	0.0000838, 0.000101, 0.000122, 0.000148, 0.000179
	Hispanic	Men	44	0.0000966, 0.000116, 0.000138, 0.000165, 0.000197
	Hispanic	Men	45	0.000111, 0.000132, 0.000157, 0.000186, 0.000221
	Hispanic	Men	46	0.000127, 0.00015, 0.000178, 0.000212, 0.000251
	Hispanic	Men	47	0.000146, 0.000173, 0.000205, 0.000242, 0.000287
	Hispanic	Men	48	0.000172, 0.000202, 0.000237, 0.000277, 0.000326
	Hispanic	Men	49	0.000203, 0.000236, 0.000275, 0.00032, 0.000373
	Hispanic	Men	50	0.000238, 0.000276, 0.000321, 0.000373, 0.000435
	Hispanic	Men	51	0.000277, 0.000323, 0.000376, 0.000437, 0.00051
	Hispanic	Men	52	0.00032, 0.000375, 0.000438, 0.000511, 0.000598
	Hispanic	Men	53	0.000364, 0.00043, 0.000506, 0.000596, 0.000703
	Hispanic	Men	54	0.000409, 0.000487, 0.000579, 0.000689, 0.000821
	Hispanic	Men	55	0.00046, 0.00055, 0.000656, 0.000783, 0.000936
	Hispanic	Men	56	0.000518, 0.000617, 0.000735, 0.000876, 0.00104
	Hispanic	Men	57	0.00058, 0.000688, 0.000816, 0.000968, 0.00115
	Hispanic	Men	58	0.000642, 0.000761, 0.000899, 0.00106, 0.00126
	Hispanic	Men	59	0.000704, 0.000832, 0.000983, 0.00116, 0.00137
	Hispanic	Men	60	0.000762, 0.000902, 0.00107, 0.00126, 0.00149
	Hispanic	Men	61	0.000816, 0.000968, 0.00115, 0.00136, 0.00161
	Hispanic	Men	62	0.000868, 0.00103, 0.00122, 0.00145, 0.00172
	Hispanic	Men	63	0.000917, 0.00109, 0.00129, 0.00154, 0.00183

Variable	Race/ethnicity	Sex	Age	Distribution
	Hispanic	Men	64	0.000967, 0.00115, 0.00137, 0.00162, 0.00193
	Hispanic	Men	65	0.00102, 0.00121, 0.00144, 0.00171, 0.00204
	Hispanic	Men	66	0.00107, 0.00128, 0.00153, 0.00182, 0.00217
	Hispanic	Men	67	0.00113, 0.00136, 0.00163, 0.00195, 0.00233
	Hispanic	Men	68	0.0012, 0.00145, 0.00175, 0.00211, 0.00255
	Hispanic	Men	69	0.00128, 0.00156, 0.00189, 0.0023, 0.0028
	Hispanic	Men	70	0.00137, 0.00168, 0.00206, 0.00252, 0.00309
	Hispanic	Men	71	0.00148, 0.00182, 0.00225, 0.00277, 0.00342
	Hispanic	Men	72	0.0016, 0.00198, 0.00245, 0.00304, 0.00378
	Hispanic	Men	73	0.00173, 0.00216, 0.00268, 0.00333, 0.00415
	Hispanic	Men	74	0.00189, 0.00236, 0.00293, 0.00365, 0.00455
	Hispanic	Men	75	0.00208, 0.00259, 0.00322, 0.004, 0.00498
	Hispanic	Men	76	0.0023, 0.00285, 0.00354, 0.00439, 0.00546
	Hispanic	Men	77	0.00255, 0.00317, 0.00392, 0.00485, 0.00602
	Hispanic	Men	78	0.00285, 0.00353, 0.00436, 0.00538, 0.00666
	Hispanic	Men	79	0.00318, 0.00393, 0.00486, 0.00599, 0.00741
	Hispanic	Men	80	0.00351, 0.00436, 0.0054, 0.00669, 0.00831
	Hispanic	Men	81	0.00381, 0.00478, 0.00597, 0.00746, 0.00935
	Hispanic	Men	82	0.00409, 0.00518, 0.00656, 0.00829, 0.0105
	Hispanic	Men	83	0.00434, 0.00557, 0.00714, 0.00915, 0.0118
	Hispanic	Men	84	0.00459, 0.00596, 0.00772, 0.01, 0.013
	Hispanic	Women	30	0.00000244, 0.00000373, 0.00000569, 0.00000868, 0.0000133
	Hispanic	Women	31	0.00000301, 0.00000448, 0.00000662, 0.00000981, 0.0000146
	Hispanic	Women	32	0.0000037, 0.00000535, 0.00000772, 0.0000111, 0.0000161
	Hispanic	Women	33	0.00000448, 0.00000635, 0.00000897, 0.0000127, 0.0000179
	Hispanic	Women	34	0.00000532, 0.00000744, 0.0000104, 0.0000144, 0.0000202
	Hispanic	Women	35	0.00000615, 0.00000856, 0.0000119, 0.0000165, 0.0000229
	Hispanic	Women	36	0.00000697, 0.0000097, 0.0000135, 0.0000187, 0.000026
	Hispanic	Women	37	0.00000781, 0.0000109, 0.0000151, 0.000021, 0.0000293
	Hispanic	Women	38	0.00000869, 0.0000121, 0.0000169, 0.0000235, 0.0000329
	Hispanic	Women	39	0.00000971, 0.0000135, 0.0000188, 0.0000262, 0.0000365
	Hispanic	Women	40	0.000011, 0.0000152, 0.0000209, 0.0000288, 0.0000399
	Hispanic	Women	41	0.0000126, 0.0000171, 0.0000232, 0.0000314, 0.0000427
	Hispanic	Women	42	0.0000148, 0.0000196, 0.0000258, 0.0000341, 0.0000451
	Hispanic	Women	43	0.0000174, 0.0000225, 0.0000289, 0.0000372, 0.000048
	Hispanic	Women	44	0.0000204, 0.0000259, 0.0000327, 0.0000413, 0.0000522
	Hispanic	Women	45	0.0000241, 0.00003, 0.0000372, 0.0000462, 0.0000576
	Hispanic	Women	46	0.0000287, 0.0000351, 0.0000428, 0.0000522, 0.0000637
	Hispanic	Women	47	0.0000345, 0.0000414, 0.0000495, 0.0000593, 0.000071
	Hispanic	Women	48	0.0000413, 0.0000489, 0.0000577, 0.0000682, 0.0000806
	Hispanic	Women	49	0.0000486, 0.0000574, 0.0000677, 0.0000798, 0.0000942
	Hispanic	Women	50	0.0000563, 0.0000671, 0.0000797, 0.0000948, 0.000113
	Hispanic	Women	51	0.0000647, 0.0000782, 0.0000942, 0.000114, 0.000137

Variable	Race/ethnicity	Sex	Age	Distribution
	Hispanic	Women	52	0.0000743, 0.000091, 0.000111, 0.000136, 0.000167
	Hispanic	Women	53	0.0000851, 0.000106, 0.000131, 0.000162, 0.000202
	Hispanic	Women	54	0.0000975, 0.000123, 0.000154, 0.000193, 0.000242
	Hispanic	Women	55	0.000112, 0.000142, 0.000179, 0.000227, 0.000287
	Hispanic	Women	56	0.000129, 0.000164, 0.000208, 0.000265, 0.000337
	Hispanic	Women	57	0.000147, 0.000188, 0.000241, 0.000307, 0.000393
	Hispanic	Women	58	0.000166, 0.000215, 0.000276, 0.000355, 0.000458
	Hispanic	Women	59	0.000187, 0.000243, 0.000315, 0.000409, 0.000532
	Hispanic	Women	60	0.000207, 0.000272, 0.000356, 0.000467, 0.000613
	Hispanic	Women	61	0.000228, 0.000302, 0.000399, 0.000528, 0.000699
	Hispanic	Women	62	0.00025, 0.000333, 0.000443, 0.000588, 0.000784
	Hispanic	Women	63	0.000273, 0.000365, 0.000486, 0.000647, 0.000865
	Hispanic	Women	64	0.000299, 0.000398, 0.00053, 0.000705, 0.00094
	Hispanic	Women	65	0.000328, 0.000436, 0.000577, 0.000764, 0.00101
	Hispanic	Women	66	0.000362, 0.000478, 0.00063, 0.00083, 0.0011
	Hispanic	Women	67	0.0004, 0.000527, 0.000693, 0.00091, 0.0012
	Hispanic	Women	68	0.000444, 0.000585, 0.000768, 0.00101, 0.00133
	Hispanic	Women	69	0.000496, 0.000652, 0.000856, 0.00112, 0.00148
	Hispanic	Women	70	0.000555, 0.000729, 0.000957, 0.00125, 0.00165
	Hispanic	Women	71	0.000622, 0.000817, 0.00107, 0.0014, 0.00184
	Hispanic	Women	72	0.000698, 0.000916, 0.0012, 0.00157, 0.00206
	Hispanic	Women	73	0.000782, 0.00103, 0.00135, 0.00176, 0.00232
	Hispanic	Women	74	0.000876, 0.00115, 0.00151, 0.00199, 0.00261
	Hispanic	Women	75	0.000983, 0.0013, 0.00171, 0.00225, 0.00297
	Hispanic	Women	76	0.0011, 0.00146, 0.00193, 0.00256, 0.00339
	Hispanic	Women	77	0.00124, 0.00165, 0.0022, 0.00292, 0.0039
	Hispanic	Women	78	0.00139, 0.00187, 0.00251, 0.00336, 0.00451
	Hispanic	Women	79	0.00157, 0.00212, 0.00286, 0.00386, 0.00523
	Hispanic	Women	80	0.00177, 0.00241, 0.00327, 0.00445, 0.00606
	Hispanic	Women	81	0.00198, 0.00272, 0.00373, 0.00512, 0.00704
	Hispanic	Women	82	0.0022, 0.00306, 0.00424, 0.00588, 0.00817
	Hispanic	Women	83	0.00241, 0.0034, 0.00478, 0.00671, 0.00946
	Hispanic	Women	84	0.00263, 0.00375, 0.00534, 0.0076, 0.0109
	White	Men	30	0.0000158, 0.000023, 0.0000332, 0.0000481, 0.0000698
	White	Men	31	0.0000207, 0.0000292, 0.000041, 0.0000575, 0.000081
	White	Men	32	0.0000266, 0.0000366, 0.0000502, 0.0000688, 0.0000947
	White	Men	33	0.0000333, 0.000045, 0.0000607, 0.000082, 0.000111
	White	Men	34	0.0000404, 0.0000542, 0.0000726, 0.0000972, 0.00013
	White	Men	35	0.0000477, 0.0000639, 0.0000855, 0.000114, 0.000153
	White	Men	36	0.0000553, 0.0000742, 0.0000993, 0.000133, 0.000178
	White	Men	37	0.0000637, 0.0000853, 0.000114, 0.000152, 0.000204
	White	Men	38	0.0000736, 0.0000979, 0.00013, 0.000172, 0.000229
	White	Men	39	0.0000854, 0.000112, 0.000147, 0.000193, 0.000253

Variable	Race/ethnicity	Sex	Age	Distribution
	White	Men	40	0.0000996, 0.000128, 0.000165, 0.000213, 0.000274
	White	Men	41	0.000116, 0.000147, 0.000184, 0.000232, 0.000292
	White	Men	42	0.000136, 0.000167, 0.000205, 0.000251, 0.000309
	White	Men	43	0.000157, 0.000189, 0.000227, 0.000273, 0.000329
	White	Men	44	0.000179, 0.000212, 0.000252, 0.0003, 0.000357
	White	Men	45	0.000204, 0.00024, 0.000282, 0.000331, 0.00039
	White	Men	46	0.000235, 0.000273, 0.000316, 0.000367, 0.000426
	White	Men	47	0.000272, 0.000312, 0.000357, 0.000409, 0.00047
	White	Men	48	0.000314, 0.000358, 0.000407, 0.000462, 0.000526
	White	Men	49	0.000362, 0.000411, 0.000465, 0.000526, 0.000597
	White	Men	50	0.000419, 0.000473, 0.000532, 0.0006, 0.000676
	White	Men	51	0.000487, 0.000545, 0.000609, 0.00068, 0.00076
	White	Men	52	0.000562, 0.000624, 0.000693, 0.000769, 0.000854
	White	Men	53	0.000631, 0.000703, 0.000782, 0.000871, 0.00097
	White	Men	54	0.000693, 0.00078, 0.000876, 0.000984, 0.00111
	White	Men	55	0.000754, 0.000857, 0.000971, 0.0011, 0.00125
	White	Men	56	0.000817, 0.000934, 0.00107, 0.00122, 0.00139
	White	Men	57	0.000877, 0.00101, 0.00116, 0.00134, 0.00154
	White	Men	58	0.000937, 0.00109, 0.00126, 0.00145, 0.00169
	White	Men	59	0.000999, 0.00116, 0.00135, 0.00157, 0.00183
	White	Men	60	0.00106, 0.00124, 0.00145, 0.00169, 0.00197
	White	Men	61	0.00113, 0.00132, 0.00154, 0.0018, 0.00211
	White	Men	62	0.00119, 0.00139, 0.00163, 0.0019, 0.00223
	White	Men	63	0.00125, 0.00146, 0.00171, 0.002, 0.00234
	White	Men	64	0.0013, 0.00153, 0.00179, 0.00209, 0.00246
	White	Men	65	0.00135, 0.00159, 0.00186, 0.00219, 0.00258
	White	Men	66	0.00139, 0.00165, 0.00195, 0.00231, 0.00273
	White	Men	67	0.00144, 0.00172, 0.00206, 0.00246, 0.00294
	White	Men	68	0.0015, 0.00182, 0.00219, 0.00264, 0.0032
	White	Men	69	0.00159, 0.00194, 0.00236, 0.00287, 0.00349
	White	Men	70	0.00171, 0.00209, 0.00255, 0.00312, 0.00381
	White	Men	71	0.00185, 0.00226, 0.00277, 0.00339, 0.00416
	White	Men	72	0.00201, 0.00246, 0.00302, 0.00369, 0.00453
	White	Men	73	0.00218, 0.00268, 0.00328, 0.00402, 0.00494
	White	Men	74	0.00238, 0.00292, 0.00358, 0.00438, 0.00539
	White	Men	75	0.00259, 0.00318, 0.0039, 0.00479, 0.00589
	White	Men	76	0.00282, 0.00348, 0.00428, 0.00526, 0.00648
	White	Men	77	0.00309, 0.00382, 0.00472, 0.00582, 0.0072
	White	Men	78	0.00341, 0.00423, 0.00524, 0.00649, 0.00806
	White	Men	79	0.00378, 0.00471, 0.00586, 0.00728, 0.00907
	White	Men	80	0.00422, 0.00527, 0.00657, 0.00819, 0.0102
	White	Men	81	0.00474, 0.00592, 0.00738, 0.00921, 0.0115
	White	Men	82	0.0053, 0.00663, 0.00827, 0.0103, 0.0129

Variable	Race/ethnicity	Sex	Age	Distribution
	White	Men	83	0.0059, 0.00738, 0.00921, 0.0115, 0.0144
	White	Men	84	0.00651, 0.00814, 0.0102, 0.0127, 0.0158
	White	Women	30	0.00000427, 0.0000068, 0.0000108, 0.0000171, 0.0000272
	White	Women	31	0.00000568, 0.00000869, 0.0000132, 0.0000202, 0.0000308
	White	Women	32	0.00000753, 0.0000111, 0.0000162, 0.0000236, 0.0000347
	White	Women	33	0.00000982, 0.0000139, 0.0000196, 0.0000276, 0.000039
	White	Women	34	0.0000124, 0.0000171, 0.0000234, 0.0000321, 0.0000442
	White	Women	35	0.0000152, 0.0000206, 0.0000277, 0.0000373, 0.0000504
	White	Women	36	0.0000181, 0.0000242, 0.0000322, 0.000043, 0.0000575
	White	Women	37	0.0000209, 0.0000278, 0.000037, 0.0000492, 0.0000656
	White	Women	38	0.0000235, 0.0000314, 0.0000419, 0.0000558, 0.0000746
	White	Women	39	0.000026, 0.000035, 0.0000469, 0.0000628, 0.0000844
	White	Women	40	0.0000286, 0.0000386, 0.0000519, 0.0000698, 0.0000941
	White	Women	41	0.0000315, 0.0000424, 0.0000569, 0.0000764, 0.000103
	White	Women	42	0.000035, 0.0000467, 0.0000621, 0.0000827, 0.00011
	White	Women	43	0.0000387, 0.0000513, 0.0000678, 0.0000896, 0.000119
	White	Women	44	0.0000425, 0.0000563, 0.0000743, 0.0000981, 0.00013
	White	Women	45	0.0000471, 0.0000622, 0.0000819, 0.000108, 0.000142
	White	Women	46	0.0000535, 0.0000698, 0.0000909, 0.000118, 0.000154
	White	Women	47	0.0000625, 0.0000798, 0.000102, 0.000129, 0.000165
	White	Women	48	0.0000744, 0.0000925, 0.000115, 0.000142, 0.000176
	White	Women	49	0.0000888, 0.000108, 0.00013, 0.000157, 0.000191
	White	Women	50	0.000105, 0.000125, 0.000149, 0.000177, 0.000211
	White	Women	51	0.000122, 0.000144, 0.000171, 0.000202, 0.000239
	White	Women	52	0.00014, 0.000166, 0.000195, 0.000231, 0.000273
	White	Women	53	0.000159, 0.000188, 0.000223, 0.000264, 0.000313
	White	Women	54	0.000178, 0.000212, 0.000253, 0.000301, 0.000359
	White	Women	55	0.000199, 0.000238, 0.000285, 0.00034, 0.000408
	White	Women	56	0.000222, 0.000267, 0.000319, 0.000383, 0.000459
	White	Women	57	0.000248, 0.000298, 0.000357, 0.000429, 0.000516
	White	Women	58	0.000276, 0.000332, 0.000399, 0.000479, 0.000577
	White	Women	59	0.000308, 0.00037, 0.000445, 0.000535, 0.000644
	White	Women	60	0.000339, 0.000409, 0.000493, 0.000595, 0.000718
	White	Women	61	0.000368, 0.000447, 0.000542, 0.000657, 0.000799
	White	Women	62	0.000396, 0.000484, 0.00059, 0.000719, 0.000879
	White	Women	63	0.000424, 0.00052, 0.000637, 0.000779, 0.000955
	White	Women	64	0.000453, 0.000557, 0.000684, 0.000839, 0.00103
	White	Women	65	0.000484, 0.000597, 0.000734, 0.000903, 0.00111
	White	Women	66	0.00052, 0.000643, 0.000792, 0.000977, 0.00121
	White	Women	67	0.000564, 0.000698, 0.000862, 0.00106, 0.00132
	White	Women	68	0.000617, 0.000765, 0.000946, 0.00117, 0.00145
	White	Women	69	0.000681, 0.000844, 0.00104, 0.00129, 0.0016
	White	Women	70	0.000754, 0.000936, 0.00116, 0.00144, 0.00178

Variable	Race/ethnicity	Sex	Age	Distribution
	White	Women	71	0.000836, 0.00104, 0.00129, 0.0016, 0.00199
	White	Women	72	0.000926, 0.00115, 0.00143, 0.00178, 0.00222
	White	Women	73	0.00102, 0.00128, 0.0016, 0.00199, 0.00249
	White	Women	74	0.00113, 0.00142, 0.00178, 0.00223, 0.00281
	White	Women	75	0.00126, 0.00158, 0.00199, 0.00251, 0.00317
	White	Women	76	0.0014, 0.00177, 0.00224, 0.00283, 0.00358
	White	Women	77	0.00156, 0.00199, 0.00253, 0.00321, 0.00408
	White	Women	78	0.00175, 0.00224, 0.00286, 0.00365, 0.00468
	White	Women	79	0.00198, 0.00254, 0.00326, 0.00419, 0.00539
	White	Women	80	0.00223, 0.00289, 0.00373, 0.00481, 0.00623
	White	Women	81	0.00253, 0.00329, 0.00427, 0.00554, 0.00721
	White	Women	82	0.00286, 0.00374, 0.00488, 0.00636, 0.00832
	White	Women	83	0.00321, 0.00422, 0.00554, 0.00726, 0.00955
	White	Women	84	0.00358, 0.00473, 0.00623, 0.00821, 0.0109
CHD mortality rates for 2033 (0.01, 0.2, 0.5, 0.8. 0.99 percentiles of the empirical distribution produced during forecasting)				
	Black	Men	30	0.0000255, 0.0000385, 0.0000578, 0.0000868, 0.000131
	Black	Men	31	0.000033, 0.0000481, 0.0000698, 0.000101, 0.000148
	Black	Men	32	0.0000421, 0.0000594, 0.0000836, 0.000118, 0.000166
	Black	Men	33	0.0000528, 0.0000725, 0.0000992, 0.000136, 0.000186
	Black	Men	34	0.0000643, 0.0000866, 0.000116, 0.000156, 0.00021
	Black	Men	35	0.0000755, 0.000101, 0.000134, 0.000179, 0.000238
	Black	Men	36	0.0000862, 0.000115, 0.000152, 0.000202, 0.00027
	Black	Men	37	0.0000966, 0.000129, 0.000171, 0.000227, 0.000302
	Black	Men	38	0.000107, 0.000143, 0.000189, 0.000251, 0.000334
	Black	Men	39	0.000118, 0.000157, 0.000208, 0.000275, 0.000365
	Black	Men	40	0.00013, 0.000172, 0.000226, 0.000298, 0.000394
	Black	Men	41	0.000144, 0.000189, 0.000246, 0.000321, 0.00042
	Black	Men	42	0.000161, 0.000208, 0.000268, 0.000345, 0.000446
	Black	Men	43	0.000178, 0.000228, 0.000292, 0.000375, 0.000481
	Black	Men	44	0.000194, 0.00025, 0.000322, 0.000413, 0.000533
	Black	Men	45	0.000214, 0.000277, 0.000357, 0.000461, 0.000597
	Black	Men	46	0.000239, 0.00031, 0.0004, 0.000518, 0.000671
	Black	Men	47	0.000271, 0.000351, 0.000454, 0.000586, 0.00076
	Black	Men	48	0.000311, 0.000402, 0.00052, 0.000672, 0.000871
	Black	Men	49	0.000359, 0.000466, 0.000602, 0.000777, 0.00101
	Black	Men	50	0.000421, 0.000544, 0.0007, 0.000902, 0.00116
	Black	Men	51	0.0005, 0.00064, 0.000817, 0.00104, 0.00134
	Black	Men	52	0.000595, 0.000752, 0.00095, 0.0012, 0.00152
	Black	Men	53	0.000697, 0.000875, 0.00109, 0.00137, 0.00172
	Black	Men	54	0.0008, 0.001, 0.00125, 0.00156, 0.00195
	Black	Men	55	0.000903, 0.00113, 0.0014, 0.00175, 0.00218
	Black	Men	56	0.00101, 0.00126, 0.00156, 0.00194, 0.0024
	Black	Men	57	0.00113, 0.00139, 0.00172, 0.00211, 0.00261

Variable	Race/ethnicity	Sex	Age	Distribution
	Black	Men	58	0.00124, 0.00153, 0.00187, 0.00229, 0.00281
	Black	Men	59	0.00136, 0.00166, 0.00202, 0.00246, 0.00301
	Black	Men	60	0.00147, 0.00178, 0.00216, 0.00262, 0.00319
	Black	Men	61	0.00157, 0.0019, 0.00229, 0.00276, 0.00334
	Black	Men	62	0.00168, 0.00201, 0.00241, 0.00288, 0.00346
	Black	Men	63	0.00177, 0.00211, 0.00251, 0.00298, 0.00354
	Black	Men	64	0.00187, 0.00221, 0.0026, 0.00307, 0.00362
	Black	Men	65	0.00196, 0.0023, 0.0027, 0.00317, 0.00373
	Black	Men	66	0.00204, 0.0024, 0.00282, 0.00331, 0.00389
	Black	Men	67	0.00213, 0.00251, 0.00296, 0.00349, 0.00412
	Black	Men	68	0.00222, 0.00264, 0.00314, 0.00372, 0.00442
	Black	Men	69	0.00235, 0.0028, 0.00334, 0.00398, 0.00476
	Black	Men	70	0.00249, 0.00298, 0.00357, 0.00426, 0.00511
	Black	Men	71	0.00266, 0.00318, 0.00381, 0.00456, 0.00546
	Black	Men	72	0.00283, 0.00339, 0.00406, 0.00486, 0.00584
	Black	Men	73	0.003, 0.00361, 0.00433, 0.00519, 0.00623
	Black	Men	74	0.0032, 0.00385, 0.00461, 0.00553, 0.00665
	Black	Men	75	0.00343, 0.00412, 0.00493, 0.00591, 0.0071
	Black	Men	76	0.0037, 0.00443, 0.0053, 0.00634, 0.0076
	Black	Men	77	0.004, 0.00479, 0.00572, 0.00684, 0.00819
	Black	Men	78	0.00432, 0.00518, 0.0062, 0.00743, 0.00891
	Black	Men	79	0.00466, 0.00561, 0.00674, 0.00809, 0.00974
	Black	Men	80	0.00501, 0.00605, 0.00731, 0.00883, 0.0107
	Black	Men	81	0.00534, 0.00651, 0.00791, 0.00962, 0.0117
	Black	Men	82	0.00564, 0.00694, 0.00852, 0.0105, 0.0129
	Black	Men	83	0.00591, 0.00735, 0.00912, 0.0113, 0.0141
	Black	Men	84	0.00616, 0.00774, 0.00971, 0.0122, 0.0153
	Black	Women	30	0.0000108, 0.0000163, 0.0000245, 0.0000369, 0.0000557
	Black	Women	31	0.0000139, 0.0000202, 0.0000291, 0.000042, 0.0000608
	Black	Women	32	0.0000175, 0.0000246, 0.0000344, 0.0000482, 0.0000678
	Black	Women	33	0.0000216, 0.0000296, 0.0000406, 0.0000557, 0.0000766
	Black	Women	34	0.0000258, 0.0000351, 0.0000475, 0.0000645, 0.0000877
	Black	Women	35	0.0000298, 0.0000405, 0.000055, 0.0000746, 0.000101
	Black	Women	36	0.0000336, 0.0000459, 0.0000626, 0.0000854, 0.000117
	Black	Women	37	0.0000375, 0.0000515, 0.0000705, 0.0000966, 0.000133
	Black	Women	38	0.0000415, 0.0000572, 0.0000787, 0.000108, 0.000149
	Black	Women	39	0.0000456, 0.0000631, 0.0000871, 0.00012, 0.000166
	Black	Women	40	0.0000504, 0.0000696, 0.0000959, 0.000132, 0.000182
	Black	Women	41	0.0000567, 0.0000773, 0.000105, 0.000143, 0.000194
	Black	Women	42	0.000065, 0.0000865, 0.000115, 0.000153, 0.000203
	Black	Women	43	0.0000747, 0.0000972, 0.000126, 0.000164, 0.000213
	Black	Women	44	0.0000848, 0.000109, 0.00014, 0.000179, 0.00023
	Black	Women	45	0.0000959, 0.000123, 0.000156, 0.000199, 0.000254

Variable	Race/ethnicity	Sex	Age	Distribution
	Black	Women	46	0.00011, 0.000139, 0.000176, 0.000224, 0.000284
	Black	Women	47	0.000128, 0.000161, 0.000202, 0.000253, 0.000319
	Black	Women	48	0.000149, 0.000187, 0.000233, 0.000291, 0.000364
	Black	Women	49	0.000171, 0.000216, 0.000271, 0.00034, 0.000428
	Black	Women	50	0.000197, 0.000249, 0.000315, 0.000397, 0.000503
	Black	Women	51	0.000232, 0.000291, 0.000364, 0.000455, 0.000571
	Black	Women	52	0.000274, 0.000339, 0.000417, 0.000514, 0.000635
	Black	Women	53	0.000315, 0.000387, 0.000475, 0.000583, 0.000717
	Black	Women	54	0.00035, 0.000434, 0.000537, 0.000665, 0.000824
	Black	Women	55	0.000392, 0.000488, 0.000605, 0.00075, 0.000932
	Black	Women	56	0.000444, 0.000549, 0.000677, 0.000835, 0.00103
	Black	Women	57	0.0005, 0.000614, 0.000753, 0.000923, 0.00113
	Black	Women	58	0.000552, 0.000678, 0.000831, 0.00102, 0.00125
	Black	Women	59	0.000598, 0.000738, 0.000909, 0.00112, 0.00138
	Black	Women	60	0.00064, 0.000794, 0.000984, 0.00122, 0.00151
	Black	Women	61	0.000676, 0.000846, 0.00106, 0.00132, 0.00165
	Black	Women	62	0.000707, 0.000891, 0.00112, 0.00141, 0.00178
	Black	Women	63	0.00073, 0.00093, 0.00118, 0.0015, 0.00191
	Black	Women	64	0.000749, 0.000965, 0.00124, 0.00159, 0.00205
	Black	Women	65	0.000769, 0.001, 0.0013, 0.00169, 0.00221
	Black	Women	66	0.000797, 0.00105, 0.00138, 0.00181, 0.00238
	Black	Women	67	0.000838, 0.00111, 0.00147, 0.00194, 0.00257
	Black	Women	68	0.000898, 0.00119, 0.00158, 0.0021, 0.00279
	Black	Women	69	0.000977, 0.0013, 0.00172, 0.00228, 0.00304
	Black	Women	70	0.00107, 0.00142, 0.00188, 0.00248, 0.00329
	Black	Women	71	0.00118, 0.00156, 0.00205, 0.0027, 0.00356
	Black	Women	72	0.0013, 0.0017, 0.00223, 0.00293, 0.00385
	Black	Women	73	0.00142, 0.00186, 0.00243, 0.00318, 0.00416
	Black	Women	74	0.00154, 0.00202, 0.00264, 0.00345, 0.00452
	Black	Women	75	0.00168, 0.0022, 0.00288, 0.00377, 0.00494
	Black	Women	76	0.00182, 0.0024, 0.00315, 0.00414, 0.00546
	Black	Women	77	0.00196, 0.00261, 0.00346, 0.00459, 0.0061
	Black	Women	78	0.00211, 0.00284, 0.00381, 0.00511, 0.00688
	Black	Women	79	0.00226, 0.00309, 0.0042, 0.00572, 0.00781
	Black	Women	80	0.00242, 0.00335, 0.00463, 0.00641, 0.00889
	Black	Women	81	0.00259, 0.00364, 0.0051, 0.00716, 0.0101
	Black	Women	82	0.00279, 0.00396, 0.00561, 0.00794, 0.0113
	Black	Women	83	0.00301, 0.00431, 0.00614, 0.00875, 0.0125
	Black	Women	84	0.00325, 0.00467, 0.00668, 0.00956, 0.0137
	Hispanic	Men	30	0.00000881, 0.0000132, 0.0000198, 0.0000295, 0.0000443
	Hispanic	Men	31	0.0000111, 0.0000162, 0.0000236, 0.0000343, 0.00005
	Hispanic	Men	32	0.0000139, 0.0000198, 0.0000281, 0.0000398, 0.0000567
	Hispanic	Men	33	0.0000173, 0.000024, 0.0000332, 0.000046, 0.0000639

Variable	Race/ethnicity	Sex	Age	Distribution
	Hispanic	Men	34	0.0000213, 0.0000289, 0.0000391, 0.0000529, 0.0000718
	Hispanic	Men	35	0.0000257, 0.0000342, 0.0000455, 0.0000605, 0.0000806
	Hispanic	Men	36	0.0000302, 0.0000398, 0.0000523, 0.0000689, 0.0000908
	Hispanic	Men	37	0.0000347, 0.0000456, 0.0000597, 0.0000781, 0.000102
	Hispanic	Men	38	0.0000396, 0.0000517, 0.0000675, 0.0000881, 0.000115
	Hispanic	Men	39	0.000045, 0.0000586, 0.000076, 0.0000987, 0.000128
	Hispanic	Men	40	0.0000517, 0.0000665, 0.0000853, 0.00011, 0.000141
	Hispanic	Men	41	0.00006, 0.0000758, 0.0000956, 0.000121, 0.000152
	Hispanic	Men	42	0.0000701, 0.0000867, 0.000107, 0.000132, 0.000163
	Hispanic	Men	43	0.0000817, 0.0000991, 0.00012, 0.000145, 0.000177
	Hispanic	Men	44	0.000094, 0.000113, 0.000135, 0.000162, 0.000194
	Hispanic	Men	45	0.000108, 0.000128, 0.000153, 0.000182, 0.000218
	Hispanic	Men	46	0.000123, 0.000147, 0.000174, 0.000207, 0.000247
	Hispanic	Men	47	0.000142, 0.000169, 0.0002, 0.000237, 0.000281
	Hispanic	Men	48	0.000167, 0.000197, 0.000231, 0.000272, 0.00032
	Hispanic	Men	49	0.000198, 0.000231, 0.000269, 0.000314, 0.000366
	Hispanic	Men	50	0.000232, 0.00027, 0.000315, 0.000366, 0.000427
	Hispanic	Men	51	0.00027, 0.000316, 0.000368, 0.000429, 0.000501
	Hispanic	Men	52	0.000314, 0.000367, 0.00043, 0.000502, 0.000588
	Hispanic	Men	53	0.000357, 0.000422, 0.000497, 0.000587, 0.000693
	Hispanic	Men	54	0.000401, 0.000479, 0.00057, 0.000679, 0.00081
	Hispanic	Men	55	0.000451, 0.00054, 0.000646, 0.000773, 0.000925
	Hispanic	Men	56	0.000508, 0.000607, 0.000724, 0.000864, 0.00103
	Hispanic	Men	57	0.000569, 0.000677, 0.000805, 0.000956, 0.00114
	Hispanic	Men	58	0.00063, 0.000748, 0.000886, 0.00105, 0.00125
	Hispanic	Men	59	0.00069, 0.000818, 0.000968, 0.00115, 0.00136
	Hispanic	Men	60	0.000746, 0.000886, 0.00105, 0.00124, 0.00148
	Hispanic	Men	61	0.000799, 0.00095, 0.00113, 0.00134, 0.00159
	Hispanic	Men	62	0.000849, 0.00101, 0.0012, 0.00143, 0.0017
	Hispanic	Men	63	0.000896, 0.00107, 0.00127, 0.00151, 0.0018
	Hispanic	Men	64	0.000944, 0.00112, 0.00134, 0.00159, 0.0019
	Hispanic	Men	65	0.000994, 0.00118, 0.00141, 0.00168, 0.002
	Hispanic	Men	66	0.00105, 0.00125, 0.00149, 0.00178, 0.00213
	Hispanic	Men	67	0.0011, 0.00133, 0.00159, 0.00191, 0.00229
	Hispanic	Men	68	0.00117, 0.00141, 0.00171, 0.00206, 0.0025
	Hispanic	Men	69	0.00124, 0.00152, 0.00185, 0.00225, 0.00275
	Hispanic	Men	70	0.00133, 0.00164, 0.00201, 0.00247, 0.00303
	Hispanic	Men	71	0.00144, 0.00178, 0.00219, 0.00271, 0.00335
	Hispanic	Men	72	0.00155, 0.00193, 0.0024, 0.00298, 0.0037
	Hispanic	Men	73	0.00168, 0.0021, 0.00262, 0.00326, 0.00407
	Hispanic	Men	74	0.00184, 0.0023, 0.00286, 0.00357, 0.00446
	Hispanic	Men	75	0.00202, 0.00252, 0.00314, 0.00391, 0.00487
	Hispanic	Men	76	0.00223, 0.00278, 0.00345, 0.00429, 0.00535

Variable	Race/ethnicity	Sex	Age	Distribution
	Hispanic	Men	77	0.00248, 0.00308, 0.00382, 0.00474, 0.00589
	Hispanic	Men	78	0.00277, 0.00344, 0.00425, 0.00526, 0.00652
	Hispanic	Men	79	0.00309, 0.00383, 0.00474, 0.00586, 0.00727
	Hispanic	Men	80	0.00341, 0.00424, 0.00527, 0.00654, 0.00814
	Hispanic	Men	81	0.0037, 0.00465, 0.00583, 0.0073, 0.00917
	Hispanic	Men	82	0.00397, 0.00504, 0.0064, 0.00811, 0.0103
	Hispanic	Men	83	0.00421, 0.00542, 0.00697, 0.00895, 0.0115
	Hispanic	Men	84	0.00445, 0.0058, 0.00753, 0.00979, 0.0128
	Hispanic	Women	30	0.00000239, 0.00000369, 0.00000567, 0.0000087, 0.0000134
	Hispanic	Women	31	0.00000296, 0.00000443, 0.0000066, 0.00000983, 0.0000147
	Hispanic	Women	32	0.00000365, 0.0000053, 0.00000769, 0.0000112, 0.0000162
	Hispanic	Women	33	0.00000443, 0.0000063, 0.00000894, 0.0000127, 0.0000181
	Hispanic	Women	34	0.00000525, 0.00000738, 0.0000103, 0.0000145, 0.0000203
	Hispanic	Women	35	0.00000607, 0.00000848, 0.0000118, 0.0000165, 0.000023
	Hispanic	Women	36	0.00000687, 0.0000096, 0.0000134, 0.0000187, 0.0000261
	Hispanic	Women	37	0.00000768, 0.0000108, 0.000015, 0.000021, 0.0000294
	Hispanic	Women	38	0.00000853, 0.000012, 0.0000168, 0.0000234, 0.0000329
	Hispanic	Women	39	0.00000951, 0.0000133, 0.0000186, 0.000026, 0.0000364
	Hispanic	Women	40	0.0000107, 0.0000149, 0.0000206, 0.0000286, 0.0000397
	Hispanic	Women	41	0.0000123, 0.0000168, 0.0000228, 0.0000311, 0.0000424
	Hispanic	Women	42	0.0000144, 0.0000192, 0.0000254, 0.0000336, 0.0000446
	Hispanic	Women	43	0.000017, 0.000022, 0.0000284, 0.0000366, 0.0000473
	Hispanic	Women	44	0.0000199, 0.0000253, 0.000032, 0.0000405, 0.0000513
	Hispanic	Women	45	0.0000234, 0.0000292, 0.0000364, 0.0000453, 0.0000565
	Hispanic	Women	46	0.0000279, 0.0000342, 0.0000418, 0.0000511, 0.0000625
	Hispanic	Women	47	0.0000336, 0.0000404, 0.0000484, 0.000058, 0.0000697
	Hispanic	Women	48	0.0000402, 0.0000477, 0.0000564, 0.0000667, 0.0000791
	Hispanic	Women	49	0.0000474, 0.0000561, 0.0000662, 0.0000782, 0.0000924
	Hispanic	Women	50	0.000055, 0.0000656, 0.0000781, 0.000093, 0.000111
	Hispanic	Women	51	0.0000633, 0.0000765, 0.0000924, 0.000111, 0.000135
	Hispanic	Women	52	0.0000728, 0.0000893, 0.000109, 0.000134, 0.000164
	Hispanic	Women	53	0.0000835, 0.000104, 0.000129, 0.00016, 0.000199
	Hispanic	Women	54	0.0000957, 0.00012, 0.000151, 0.00019, 0.000239
	Hispanic	Women	55	0.00011, 0.00014, 0.000177, 0.000224, 0.000284
	Hispanic	Women	56	0.000127, 0.000161, 0.000205, 0.000261, 0.000333
	Hispanic	Women	57	0.000145, 0.000185, 0.000237, 0.000303, 0.000389
	Hispanic	Women	58	0.000164, 0.000211, 0.000272, 0.000351, 0.000453
	Hispanic	Women	59	0.000183, 0.000239, 0.00031, 0.000404, 0.000526
	Hispanic	Women	60	0.000203, 0.000268, 0.000351, 0.000461, 0.000606
	Hispanic	Women	61	0.000224, 0.000297, 0.000393, 0.00052, 0.00069
	Hispanic	Women	62	0.000245, 0.000327, 0.000435, 0.000579, 0.000774
	Hispanic	Women	63	0.000267, 0.000358, 0.000477, 0.000637, 0.000852
	Hispanic	Women	64	0.000292, 0.00039, 0.000519, 0.000692, 0.000924

Variable	Race/ethnicity	Sex	Age	Distribution
	Hispanic	Women	65	0.00032, 0.000426, 0.000565, 0.000749, 0.000996
	Hispanic	Women	66	0.000353, 0.000467, 0.000616, 0.000813, 0.00108
	Hispanic	Women	67	0.00039, 0.000515, 0.000677, 0.000891, 0.00118
	Hispanic	Women	68	0.000433, 0.000571, 0.00075, 0.000986, 0.0013
	Hispanic	Women	69	0.000483, 0.000636, 0.000836, 0.0011, 0.00145
	Hispanic	Women	70	0.00054, 0.000711, 0.000934, 0.00123, 0.00162
	Hispanic	Women	71	0.000606, 0.000797, 0.00105, 0.00137, 0.00181
	Hispanic	Women	72	0.000679, 0.000893, 0.00117, 0.00154, 0.00202
	Hispanic	Women	73	0.000761, 0.001, 0.00131, 0.00172, 0.00227
	Hispanic	Women	74	0.000852, 0.00112, 0.00148, 0.00194, 0.00256
	Hispanic	Women	75	0.000955, 0.00126, 0.00166, 0.00219, 0.0029
	Hispanic	Women	76	0.00107, 0.00142, 0.00189, 0.0025, 0.00332
	Hispanic	Women	77	0.0012, 0.00161, 0.00214, 0.00286, 0.00382
	Hispanic	Women	78	0.00135, 0.00182, 0.00244, 0.00328, 0.00441
	Hispanic	Women	79	0.00153, 0.00207, 0.00279, 0.00377, 0.00511
	Hispanic	Women	80	0.00172, 0.00235, 0.00319, 0.00435, 0.00593
	Hispanic	Women	81	0.00192, 0.00265, 0.00364, 0.005, 0.00689
	Hispanic	Women	82	0.00214, 0.00298, 0.00413, 0.00574, 0.008
	Hispanic	Women	83	0.00234, 0.00331, 0.00466, 0.00656, 0.00926
	Hispanic	Women	84	0.00255, 0.00365, 0.00521, 0.00743, 0.0106
	White	Men	30	0.0000155, 0.0000227, 0.0000331, 0.0000482, 0.0000705
	White	Men	31	0.0000204, 0.0000289, 0.0000408, 0.0000577, 0.0000818
	White	Men	32	0.0000262, 0.0000362, 0.00005, 0.000069, 0.0000955
	White	Men	33	0.0000328, 0.0000446, 0.0000606, 0.0000822, 0.000112
	White	Men	34	0.0000398, 0.0000538, 0.0000724, 0.0000974, 0.000131
	White	Men	35	0.000047, 0.0000633, 0.0000852, 0.000114, 0.000154
	White	Men	36	0.0000544, 0.0000734, 0.0000988, 0.000133, 0.000179
	White	Men	37	0.0000625, 0.0000843, 0.000113, 0.000152, 0.000205
	White	Men	38	0.0000721, 0.0000965, 0.000129, 0.000172, 0.00023
	White	Men	39	0.0000835, 0.00011, 0.000145, 0.000192, 0.000253
	White	Men	40	0.0000971, 0.000126, 0.000163, 0.000211, 0.000274
	White	Men	41	0.000113, 0.000144, 0.000182, 0.000229, 0.000291
	White	Men	42	0.000132, 0.000163, 0.000201, 0.000248, 0.000306
	White	Men	43	0.000153, 0.000185, 0.000223, 0.000269, 0.000325
	White	Men	44	0.000174, 0.000207, 0.000247, 0.000295, 0.000352
	White	Men	45	0.000198, 0.000234, 0.000275, 0.000325, 0.000383
	White	Men	46	0.000228, 0.000266, 0.000309, 0.000359, 0.000419
	White	Men	47	0.000264, 0.000304, 0.000349, 0.000401, 0.000461
	White	Men	48	0.000306, 0.000349, 0.000397, 0.000453, 0.000517
	White	Men	49	0.000353, 0.000401, 0.000455, 0.000516, 0.000586
	White	Men	50	0.000408, 0.000462, 0.000521, 0.000588, 0.000665
	White	Men	51	0.000476, 0.000533, 0.000597, 0.000668, 0.000749
	White	Men	52	0.000549, 0.000611, 0.00068, 0.000756, 0.000842

Variable	Race/ethnicity	Sex	Age	Distribution
	White	Men	53	0.000618, 0.00069, 0.000769, 0.000858, 0.000957
	White	Men	54	0.000679, 0.000765, 0.000862, 0.000971, 0.00109
	White	Men	55	0.000739, 0.000841, 0.000956, 0.00109, 0.00124
	White	Men	56	0.000801, 0.000918, 0.00105, 0.0012, 0.00138
	White	Men	57	0.00086, 0.000993, 0.00114, 0.00132, 0.00152
	White	Men	58	0.000918, 0.00107, 0.00124, 0.00144, 0.00167
	White	Men	59	0.000979, 0.00114, 0.00133, 0.00155, 0.00182
	White	Men	60	0.00104, 0.00122, 0.00143, 0.00167, 0.00195
	White	Men	61	0.0011, 0.0013, 0.00152, 0.00178, 0.00208
	White	Men	62	0.00116, 0.00137, 0.0016, 0.00188, 0.0022
	White	Men	63	0.00122, 0.00143, 0.00168, 0.00197, 0.00231
	White	Men	64	0.00127, 0.00149, 0.00175, 0.00206, 0.00242
	White	Men	65	0.00131, 0.00155, 0.00182, 0.00215, 0.00254
	White	Men	66	0.00136, 0.00161, 0.00191, 0.00226, 0.00269
	White	Men	67	0.0014, 0.00168, 0.00201, 0.00241, 0.00289
	White	Men	68	0.00146, 0.00177, 0.00214, 0.00259, 0.00314
	White	Men	69	0.00155, 0.00189, 0.0023, 0.00281, 0.00343
	White	Men	70	0.00166, 0.00203, 0.00249, 0.00305, 0.00374
	White	Men	71	0.00179, 0.00221, 0.00271, 0.00332, 0.00408
	White	Men	72	0.00195, 0.0024, 0.00294, 0.00362, 0.00445
	White	Men	73	0.00212, 0.00261, 0.00321, 0.00394, 0.00484
	White	Men	74	0.00231, 0.00284, 0.00349, 0.00429, 0.00528
	White	Men	75	0.00251, 0.0031, 0.00381, 0.00468, 0.00577
	White	Men	76	0.00274, 0.00339, 0.00417, 0.00514, 0.00635
	White	Men	77	0.003, 0.00372, 0.0046, 0.00569, 0.00706
	White	Men	78	0.00331, 0.00412, 0.00511, 0.00635, 0.0079
	White	Men	79	0.00367, 0.00458, 0.00571, 0.00712, 0.00889
	White	Men	80	0.0041, 0.00513, 0.00641, 0.00801, 0.01
	White	Men	81	0.0046, 0.00576, 0.0072, 0.00901, 0.0113
	White	Men	82	0.00515, 0.00645, 0.00807, 0.0101, 0.0127
	White	Men	83	0.00572, 0.00718, 0.00898, 0.0112, 0.0141
	White	Men	84	0.00631, 0.00791, 0.0099, 0.0124, 0.0155
	White	Women	30	0.0000042, 0.00000673, 0.0000107, 0.0000171, 0.0000274
	White	Women	31	0.0000056, 0.00000861, 0.0000132, 0.0000202, 0.0000311
	White	Women	32	0.00000743, 0.000011, 0.0000161, 0.0000237, 0.0000349
	White	Women	33	0.00000969, 0.0000138, 0.0000195, 0.0000276, 0.0000393
	White	Women	34	0.0000123, 0.000017, 0.0000234, 0.0000322, 0.0000444
	White	Women	35	0.000015, 0.0000204, 0.0000276, 0.0000373, 0.0000507
	White	Women	36	0.0000178, 0.0000239, 0.0000321, 0.000043, 0.0000579
	White	Women	37	0.0000205, 0.0000275, 0.0000367, 0.0000491, 0.0000659
	White	Women	38	0.0000231, 0.000031, 0.0000415, 0.0000556, 0.0000748
	White	Women	39	0.0000255, 0.0000344, 0.0000464, 0.0000624, 0.0000844
	White	Women	40	0.0000279, 0.0000379, 0.0000512, 0.0000692, 0.0000938

Variable	Race/ethnicity	Sex	Age	Distribution
	White	Women	41	0.0000308, 0.0000416, 0.000056, 0.0000755, 0.000102
	White	Women	42	0.0000341, 0.0000457, 0.000061, 0.0000814, 0.000109
	White	Women	43	0.0000378, 0.0000502, 0.0000665, 0.0000881, 0.000117
	White	Women	44	0.0000415, 0.000055, 0.0000727, 0.0000962, 0.000128
	White	Women	45	0.0000459, 0.0000607, 0.00008, 0.000106, 0.00014
	White	Women	46	0.0000521, 0.0000681, 0.0000888, 0.000116, 0.000151
	White	Women	47	0.0000609, 0.0000778, 0.0000993, 0.000127, 0.000162
	White	Women	48	0.0000725, 0.0000902, 0.000112, 0.000139, 0.000173
	White	Women	49	0.0000866, 0.000105, 0.000127, 0.000154, 0.000187
	White	Women	50	0.000102, 0.000122, 0.000146, 0.000174, 0.000208
	White	Women	51	0.000119, 0.000141, 0.000167, 0.000198, 0.000235
	White	Women	52	0.000137, 0.000162, 0.000192, 0.000227, 0.000268
	White	Women	53	0.000156, 0.000185, 0.000219, 0.00026, 0.000308
	White	Women	54	0.000175, 0.000209, 0.000249, 0.000296, 0.000354
	White	Women	55	0.000195, 0.000234, 0.00028, 0.000336, 0.000403
	White	Women	56	0.000218, 0.000262, 0.000315, 0.000378, 0.000454
	White	Women	57	0.000243, 0.000293, 0.000352, 0.000423, 0.00051
	White	Women	58	0.000271, 0.000327, 0.000393, 0.000473, 0.000571
	White	Women	59	0.000302, 0.000364, 0.000438, 0.000528, 0.000637
	White	Women	60	0.000332, 0.000402, 0.000486, 0.000587, 0.000711
	White	Women	61	0.00036, 0.000439, 0.000533, 0.000648, 0.00079
	White	Women	62	0.000388, 0.000475, 0.00058, 0.000709, 0.000868
	White	Women	63	0.000415, 0.00051, 0.000625, 0.000766, 0.000942
	White	Women	64	0.000442, 0.000545, 0.00067, 0.000824, 0.00102
	White	Women	65	0.000472, 0.000583, 0.000719, 0.000886, 0.00109
	White	Women	66	0.000507, 0.000627, 0.000775, 0.000957, 0.00118
	White	Women	67	0.000549, 0.000681, 0.000843, 0.00104, 0.00129
	White	Women	68	0.000601, 0.000746, 0.000924, 0.00115, 0.00142
	White	Women	69	0.000662, 0.000823, 0.00102, 0.00127, 0.00157
	White	Women	70	0.000733, 0.000912, 0.00113, 0.00141, 0.00175
	White	Women	71	0.000813, 0.00101, 0.00126, 0.00156, 0.00195
	White	Women	72	0.0009, 0.00112, 0.0014, 0.00174, 0.00218
	White	Women	73	0.000994, 0.00125, 0.00156, 0.00195, 0.00244
	White	Women	74	0.0011, 0.00138, 0.00174, 0.00218, 0.00275
	White	Women	75	0.00122, 0.00154, 0.00194, 0.00245, 0.0031
	White	Women	76	0.00136, 0.00172, 0.00218, 0.00276, 0.00351
	White	Women	77	0.00152, 0.00194, 0.00246, 0.00313, 0.00399
	White	Women	78	0.0017, 0.00218, 0.00279, 0.00357, 0.00458
	White	Women	79	0.00192, 0.00247, 0.00318, 0.00409, 0.00527
	White	Women	80	0.00217, 0.00281, 0.00364, 0.0047, 0.0061
	White	Women	81	0.00246, 0.0032, 0.00416, 0.00541, 0.00706
	White	Women	82	0.00277, 0.00364, 0.00476, 0.00622, 0.00815
	White	Women	83	0.00311, 0.00411, 0.0054, 0.0071, 0.00936

Variable	Race/ethnicity	Sex	Age	Distribution
	White	Women	84	0.00347, 0.0046, 0.00608, 0.00803, 0.0106
CHD mortality rates for 2034 (0.01, 0.2, 0.5, 0.8. 0.99 percentiles of the empirical distribution produced during forecasting)				
	Black	Men	30	0.000025, 0.000038, 0.0000575, 0.000087, 0.000132
	Black	Men	31	0.0000324, 0.0000476, 0.0000695, 0.000101, 0.000149
	Black	Men	32	0.0000414, 0.0000588, 0.0000833, 0.000118, 0.000167
	Black	Men	33	0.000052, 0.0000718, 0.0000988, 0.000136, 0.000188
	Black	Men	34	0.0000633, 0.0000858, 0.000116, 0.000156, 0.000212
	Black	Men	35	0.0000743, 0.0000998, 0.000134, 0.000179, 0.00024
	Black	Men	36	0.0000847, 0.000113, 0.000152, 0.000202, 0.000271
	Black	Men	37	0.0000948, 0.000127, 0.00017, 0.000226, 0.000303
	Black	Men	38	0.000105, 0.00014, 0.000187, 0.00025, 0.000335
	Black	Men	39	0.000115, 0.000154, 0.000205, 0.000273, 0.000365
	Black	Men	40	0.000127, 0.000169, 0.000223, 0.000296, 0.000393
	Black	Men	41	0.000141, 0.000185, 0.000242, 0.000317, 0.000417
	Black	Men	42	0.000157, 0.000203, 0.000263, 0.00034, 0.000441
	Black	Men	43	0.000173, 0.000223, 0.000286, 0.000368, 0.000474
	Black	Men	44	0.000189, 0.000244, 0.000315, 0.000405, 0.000524
	Black	Men	45	0.000208, 0.00027, 0.000349, 0.000451, 0.000586
	Black	Men	46	0.000233, 0.000302, 0.000391, 0.000506, 0.000658
	Black	Men	47	0.000264, 0.000343, 0.000443, 0.000574, 0.000744
	Black	Men	48	0.000303, 0.000393, 0.000508, 0.000658, 0.000853
	Black	Men	49	0.000351, 0.000455, 0.000588, 0.000761, 0.000987
	Black	Men	50	0.000412, 0.000532, 0.000686, 0.000884, 0.00114
	Black	Men	51	0.000489, 0.000627, 0.000801, 0.00102, 0.00131
	Black	Men	52	0.000583, 0.000738, 0.000933, 0.00118, 0.00149
	Black	Men	53	0.000684, 0.000859, 0.00108, 0.00135, 0.00169
	Black	Men	54	0.000785, 0.000983, 0.00123, 0.00154, 0.00192
	Black	Men	55	0.000887, 0.00111, 0.00138, 0.00173, 0.00216
	Black	Men	56	0.000995, 0.00124, 0.00154, 0.00191, 0.00238
	Black	Men	57	0.00111, 0.00137, 0.00169, 0.00209, 0.00258
	Black	Men	58	0.00122, 0.0015, 0.00184, 0.00226, 0.00278
	Black	Men	59	0.00133, 0.00163, 0.00199, 0.00243, 0.00298
	Black	Men	60	0.00144, 0.00175, 0.00213, 0.00259, 0.00316
	Black	Men	61	0.00154, 0.00187, 0.00226, 0.00273, 0.0033
	Black	Men	62	0.00164, 0.00197, 0.00237, 0.00284, 0.00342
	Black	Men	63	0.00173, 0.00207, 0.00246, 0.00293, 0.0035
	Black	Men	64	0.00182, 0.00216, 0.00255, 0.00301, 0.00357
	Black	Men	65	0.00191, 0.00225, 0.00264, 0.00311, 0.00367
	Black	Men	66	0.00199, 0.00234, 0.00276, 0.00324, 0.00382
	Black	Men	67	0.00207, 0.00245, 0.00289, 0.00342, 0.00405
	Black	Men	68	0.00216, 0.00258, 0.00306, 0.00364, 0.00434
	Black	Men	69	0.00228, 0.00273, 0.00326, 0.0039, 0.00467
	Black	Men	70	0.00242, 0.00291, 0.00349, 0.00418, 0.00502

Variable	Race/ethnicity	Sex	Age	Distribution
	Black	Men	71	0.00258, 0.0031, 0.00372, 0.00446, 0.00536
	Black	Men	72	0.00275, 0.0033, 0.00397, 0.00476, 0.00573
	Black	Men	73	0.00292, 0.00351, 0.00422, 0.00508, 0.00611
	Black	Men	74	0.00311, 0.00374, 0.0045, 0.00541, 0.00652
	Black	Men	75	0.00333, 0.00401, 0.00481, 0.00578, 0.00696
	Black	Men	76	0.00359, 0.00431, 0.00517, 0.0062, 0.00745
	Black	Men	77	0.00388, 0.00466, 0.00558, 0.00669, 0.00803
	Black	Men	78	0.00419, 0.00504, 0.00605, 0.00726, 0.00873
	Black	Men	79	0.00452, 0.00546, 0.00657, 0.00792, 0.00955
	Black	Men	80	0.00486, 0.00589, 0.00713, 0.00864, 0.0105
	Black	Men	81	0.00518, 0.00633, 0.00772, 0.00941, 0.0115
	Black	Men	82	0.00547, 0.00675, 0.00831, 0.0102, 0.0126
	Black	Men	83	0.00573, 0.00715, 0.0089, 0.0111, 0.0138
	Black	Men	84	0.00597, 0.00753, 0.00947, 0.0119, 0.015
	Black	Women	30	0.0000106, 0.0000161, 0.0000244, 0.000037, 0.0000562
	Black	Women	31	0.0000137, 0.00002, 0.000029, 0.0000421, 0.0000613
	Black	Women	32	0.0000172, 0.0000244, 0.0000343, 0.0000483, 0.0000683
	Black	Women	33	0.0000213, 0.0000294, 0.0000405, 0.0000558, 0.0000772
	Black	Women	34	0.0000254, 0.0000348, 0.0000474, 0.0000646, 0.0000883
	Black	Women	35	0.0000293, 0.0000401, 0.0000547, 0.0000747, 0.000102
	Black	Women	36	0.0000331, 0.0000455, 0.0000623, 0.0000855, 0.000118
	Black	Women	37	0.0000369, 0.0000509, 0.0000701, 0.0000965, 0.000133
	Black	Women	38	0.0000407, 0.0000565, 0.000078, 0.000108, 0.00015
	Black	Women	39	0.0000447, 0.0000622, 0.0000863, 0.00012, 0.000166
	Black	Women	40	0.0000493, 0.0000685, 0.0000947, 0.000131, 0.000182
	Black	Women	41	0.0000554, 0.0000759, 0.000104, 0.000141, 0.000193
	Black	Women	42	0.0000635, 0.0000848, 0.000113, 0.000151, 0.000201
	Black	Women	43	0.0000729, 0.0000952, 0.000124, 0.000161, 0.000211
	Black	Women	44	0.0000828, 0.000107, 0.000137, 0.000176, 0.000227
	Black	Women	45	0.0000935, 0.00012, 0.000153, 0.000195, 0.00025
	Black	Women	46	0.000107, 0.000136, 0.000173, 0.000219, 0.000279
	Black	Women	47	0.000125, 0.000157, 0.000197, 0.000248, 0.000313
	Black	Women	48	0.000146, 0.000182, 0.000228, 0.000285, 0.000357
	Black	Women	49	0.000167, 0.000211, 0.000265, 0.000334, 0.00042
	Black	Women	50	0.000193, 0.000244, 0.000309, 0.00039, 0.000495
	Black	Women	51	0.000227, 0.000285, 0.000357, 0.000448, 0.000563
	Black	Women	52	0.000269, 0.000333, 0.00041, 0.000507, 0.000626
	Black	Women	53	0.000309, 0.00038, 0.000468, 0.000575, 0.000708
	Black	Women	54	0.000344, 0.000427, 0.00053, 0.000656, 0.000814
	Black	Women	55	0.000386, 0.00048, 0.000596, 0.000741, 0.000922
	Black	Women	56	0.000437, 0.000541, 0.000668, 0.000825, 0.00102
	Black	Women	57	0.000492, 0.000605, 0.000743, 0.000913, 0.00112
	Black	Women	58	0.000543, 0.000668, 0.00082, 0.00101, 0.00124

Variable	Race/ethnicity	Sex	Age	Distribution
	Black	Women	59	0.000588, 0.000727, 0.000897, 0.00111, 0.00137
	Black	Women	60	0.000628, 0.000782, 0.000971, 0.00121, 0.0015
	Black	Women	61	0.000664, 0.000832, 0.00104, 0.0013, 0.00163
	Black	Women	62	0.000693, 0.000876, 0.0011, 0.00139, 0.00176
	Black	Women	63	0.000715, 0.000913, 0.00116, 0.00148, 0.00189
	Black	Women	64	0.000733, 0.000946, 0.00122, 0.00157, 0.00202
	Black	Women	65	0.000752, 0.000981, 0.00128, 0.00166, 0.00217
	Black	Women	66	0.000778, 0.00103, 0.00135, 0.00177, 0.00234
	Black	Women	67	0.000818, 0.00109, 0.00144, 0.0019, 0.00253
	Black	Women	68	0.000876, 0.00117, 0.00155, 0.00206, 0.00275
	Black	Women	69	0.000953, 0.00127, 0.00169, 0.00224, 0.00298
	Black	Women	70	0.00105, 0.00139, 0.00184, 0.00243, 0.00323
	Black	Women	71	0.00115, 0.00152, 0.00201, 0.00264, 0.00349
	Black	Women	72	0.00126, 0.00166, 0.00218, 0.00287, 0.00377
	Black	Women	73	0.00138, 0.00181, 0.00237, 0.00311, 0.00409
	Black	Women	74	0.0015, 0.00197, 0.00258, 0.00338, 0.00444
	Black	Women	75	0.00163, 0.00214, 0.00281, 0.00369, 0.00485
	Black	Women	76	0.00177, 0.00234, 0.00308, 0.00405, 0.00535
	Black	Women	77	0.00191, 0.00254, 0.00338, 0.00449, 0.00598
	Black	Women	78	0.00205, 0.00277, 0.00372, 0.00501, 0.00675
	Black	Women	79	0.0022, 0.00301, 0.00411, 0.0056, 0.00767
	Black	Women	80	0.00235, 0.00327, 0.00453, 0.00628, 0.00872
	Black	Women	81	0.00252, 0.00355, 0.00499, 0.00701, 0.00988
	Black	Women	82	0.00271, 0.00386, 0.00548, 0.00778, 0.0111
	Black	Women	83	0.00293, 0.0042, 0.006, 0.00857, 0.0123
	Black	Women	84	0.00317, 0.00456, 0.00653, 0.00937, 0.0135
	Hispanic	Men	30	0.00000865, 0.0000131, 0.0000197, 0.0000296, 0.0000448
	Hispanic	Men	31	0.0000109, 0.0000161, 0.0000235, 0.0000343, 0.0000504
	Hispanic	Men	32	0.0000137, 0.0000196, 0.000028, 0.0000399, 0.0000571
	Hispanic	Men	33	0.0000171, 0.0000238, 0.0000331, 0.0000461, 0.0000644
	Hispanic	Men	34	0.000021, 0.0000286, 0.0000389, 0.000053, 0.0000723
	Hispanic	Men	35	0.0000253, 0.0000339, 0.0000453, 0.0000606, 0.0000812
	Hispanic	Men	36	0.0000297, 0.0000393, 0.0000521, 0.0000689, 0.0000914
	Hispanic	Men	37	0.0000341, 0.000045, 0.0000592, 0.000078, 0.000103
	Hispanic	Men	38	0.0000387, 0.000051, 0.0000669, 0.0000878, 0.000116
	Hispanic	Men	39	0.000044, 0.0000576, 0.0000752, 0.0000982, 0.000128
	Hispanic	Men	40	0.0000504, 0.0000652, 0.0000842, 0.000109, 0.000141
	Hispanic	Men	41	0.0000584, 0.0000742, 0.0000941, 0.000119, 0.000152
	Hispanic	Men	42	0.0000683, 0.0000848, 0.000105, 0.00013, 0.000162
	Hispanic	Men	43	0.0000795, 0.0000968, 0.000118, 0.000143, 0.000174
	Hispanic	Men	44	0.0000915, 0.00011, 0.000132, 0.000159, 0.000191
	Hispanic	Men	45	0.000105, 0.000125, 0.00015, 0.000179, 0.000214
	Hispanic	Men	46	0.00012, 0.000143, 0.00017, 0.000203, 0.000242

Variable	Race/ethnicity	Sex	Age	Distribution
	Hispanic	Men	47	0.000138, 0.000165, 0.000195, 0.000232, 0.000276
	Hispanic	Men	48	0.000163, 0.000192, 0.000226, 0.000266, 0.000314
	Hispanic	Men	49	0.000192, 0.000225, 0.000263, 0.000307, 0.00036
	Hispanic	Men	50	0.000226, 0.000264, 0.000308, 0.000359, 0.00042
	Hispanic	Men	51	0.000264, 0.000309, 0.000361, 0.000422, 0.000493
	Hispanic	Men	52	0.000307, 0.00036, 0.000422, 0.000494, 0.000579
	Hispanic	Men	53	0.00035, 0.000414, 0.000489, 0.000577, 0.000683
	Hispanic	Men	54	0.000393, 0.00047, 0.000561, 0.000669, 0.0008
	Hispanic	Men	55	0.000443, 0.000531, 0.000636, 0.000762, 0.000915
	Hispanic	Men	56	0.000499, 0.000597, 0.000714, 0.000853, 0.00102
	Hispanic	Men	57	0.000558, 0.000666, 0.000793, 0.000944, 0.00113
	Hispanic	Men	58	0.000618, 0.000735, 0.000873, 0.00104, 0.00123
	Hispanic	Men	59	0.000676, 0.000804, 0.000954, 0.00113, 0.00135
	Hispanic	Men	60	0.000731, 0.00087, 0.00103, 0.00123, 0.00146
	Hispanic	Men	61	0.000782, 0.000932, 0.00111, 0.00132, 0.00157
	Hispanic	Men	62	0.00083, 0.00099, 0.00118, 0.00141, 0.00168
	Hispanic	Men	63	0.000875, 0.00105, 0.00125, 0.00149, 0.00178
	Hispanic	Men	64	0.000921, 0.0011, 0.00131, 0.00157, 0.00187
	Hispanic	Men	65	0.000969, 0.00116, 0.00138, 0.00165, 0.00197
	Hispanic	Men	66	0.00102, 0.00122, 0.00146, 0.00175, 0.00209
	Hispanic	Men	67	0.00107, 0.00129, 0.00155, 0.00187, 0.00225
	Hispanic	Men	68	0.00114, 0.00138, 0.00167, 0.00202, 0.00245
	Hispanic	Men	69	0.00121, 0.00148, 0.00181, 0.0022, 0.0027
	Hispanic	Men	70	0.0013, 0.0016, 0.00196, 0.00242, 0.00298
	Hispanic	Men	71	0.0014, 0.00173, 0.00214, 0.00265, 0.00329
	Hispanic	Men	72	0.00151, 0.00188, 0.00234, 0.00291, 0.00363
	Hispanic	Men	73	0.00164, 0.00205, 0.00256, 0.00319, 0.00399
	Hispanic	Men	74	0.00179, 0.00224, 0.00279, 0.00349, 0.00437
	Hispanic	Men	75	0.00196, 0.00245, 0.00306, 0.00382, 0.00477
	Hispanic	Men	76	0.00217, 0.0027, 0.00337, 0.00419, 0.00523
	Hispanic	Men	77	0.00241, 0.003, 0.00373, 0.00463, 0.00577
	Hispanic	Men	78	0.00269, 0.00334, 0.00415, 0.00514, 0.00639
	Hispanic	Men	79	0.003, 0.00373, 0.00462, 0.00573, 0.00712
	Hispanic	Men	80	0.00331, 0.00413, 0.00514, 0.0064, 0.00798
	Hispanic	Men	81	0.00359, 0.00452, 0.00568, 0.00714, 0.00899
	Hispanic	Men	82	0.00385, 0.00491, 0.00624, 0.00793, 0.0101
	Hispanic	Men	83	0.00408, 0.00527, 0.00679, 0.00875, 0.0113
	Hispanic	Men	84	0.00432, 0.00564, 0.00735, 0.00957, 0.0125
	Hispanic	Women	30	0.00000235, 0.00000365, 0.00000564, 0.00000872, 0.0000135
	Hispanic	Women	31	0.00000292, 0.00000439, 0.00000657, 0.00000985, 0.0000148
	Hispanic	Women	32	0.0000036, 0.00000526, 0.00000767, 0.0000112, 0.0000163
	Hispanic	Women	33	0.00000437, 0.00000625, 0.00000891, 0.0000127, 0.0000182
	Hispanic	Women	34	0.00000519, 0.00000732, 0.0000103, 0.0000145, 0.0000204

Variable	Race/ethnicity	Sex	Age	Distribution
	Hispanic	Women	35	0.00000599, 0.00000841, 0.0000118, 0.0000165, 0.0000231
	Hispanic	Women	36	0.00000677, 0.00000951, 0.0000133, 0.0000187, 0.0000262
	Hispanic	Women	37	0.00000755, 0.0000106, 0.0000149, 0.000021, 0.0000295
	Hispanic	Women	38	0.00000838, 0.0000118, 0.0000166, 0.0000234, 0.0000329
	Hispanic	Women	39	0.00000931, 0.0000131, 0.0000184, 0.0000258, 0.0000364
	Hispanic	Women	40	0.0000105, 0.0000146, 0.0000203, 0.0000283, 0.0000395
	Hispanic	Women	41	0.000012, 0.0000165, 0.0000225, 0.0000307, 0.000042
	Hispanic	Women	42	0.0000141, 0.0000188, 0.0000249, 0.0000331, 0.0000441
	Hispanic	Women	43	0.0000166, 0.0000215, 0.0000278, 0.0000359, 0.0000466
	Hispanic	Women	44	0.0000194, 0.0000247, 0.0000313, 0.0000397, 0.0000505
	Hispanic	Women	45	0.0000228, 0.0000285, 0.0000356, 0.0000444, 0.0000555
	Hispanic	Women	46	0.0000272, 0.0000334, 0.0000408, 0.00005, 0.0000614
	Hispanic	Women	47	0.0000327, 0.0000393, 0.0000473, 0.0000568, 0.0000683
	Hispanic	Women	48	0.0000392, 0.0000465, 0.0000551, 0.0000653, 0.0000776
	Hispanic	Women	49	0.0000462, 0.0000547, 0.0000647, 0.0000766, 0.0000907
	Hispanic	Women	50	0.0000536, 0.0000641, 0.0000764, 0.0000912, 0.000109
	Hispanic	Women	51	0.0000619, 0.0000749, 0.0000905, 0.000109, 0.000132
	Hispanic	Women	52	0.0000713, 0.0000875, 0.000107, 0.000131, 0.000161
	Hispanic	Women	53	0.0000819, 0.000102, 0.000127, 0.000157, 0.000196
	Hispanic	Women	54	0.000094, 0.000118, 0.000149, 0.000187, 0.000236
	Hispanic	Women	55	0.000108, 0.000137, 0.000174, 0.000221, 0.00028
	Hispanic	Women	56	0.000124, 0.000159, 0.000202, 0.000258, 0.000329
	Hispanic	Women	57	0.000142, 0.000182, 0.000234, 0.000299, 0.000384
	Hispanic	Women	58	0.000161, 0.000208, 0.000268, 0.000346, 0.000448
	Hispanic	Women	59	0.00018, 0.000235, 0.000306, 0.000398, 0.00052
	Hispanic	Women	60	0.0002, 0.000263, 0.000346, 0.000454, 0.000599
	Hispanic	Women	61	0.00022, 0.000292, 0.000387, 0.000513, 0.000681
	Hispanic	Women	62	0.00024, 0.000321, 0.000428, 0.00057, 0.000763
	Hispanic	Women	63	0.000261, 0.00035, 0.000468, 0.000626, 0.000839
	Hispanic	Women	64	0.000285, 0.000382, 0.000509, 0.000679, 0.000909
	Hispanic	Women	65	0.000313, 0.000416, 0.000553, 0.000734, 0.000978
	Hispanic	Women	66	0.000344, 0.000456, 0.000603, 0.000797, 0.00106
	Hispanic	Women	67	0.00038, 0.000503, 0.000662, 0.000873, 0.00115
	Hispanic	Women	68	0.000422, 0.000557, 0.000733, 0.000965, 0.00127
	Hispanic	Women	69	0.00047, 0.00062, 0.000817, 0.00108, 0.00142
	Hispanic	Women	70	0.000526, 0.000694, 0.000913, 0.0012, 0.00158
	Hispanic	Women	71	0.00059, 0.000777, 0.00102, 0.00134, 0.00177
	Hispanic	Women	72	0.000661, 0.000871, 0.00114, 0.0015, 0.00198
	Hispanic	Women	73	0.00074, 0.000975, 0.00128, 0.00169, 0.00222
	Hispanic	Women	74	0.000829, 0.00109, 0.00144, 0.0019, 0.0025
	Hispanic	Women	75	0.000929, 0.00123, 0.00162, 0.00214, 0.00284
	Hispanic	Women	76	0.00104, 0.00139, 0.00184, 0.00244, 0.00324
	Hispanic	Women	77	0.00117, 0.00157, 0.00209, 0.00279, 0.00373

Variable	Race/ethnicity	Sex	Age	Distribution
	Hispanic	Women	78	0.00132, 0.00177, 0.00238, 0.0032, 0.00431
	Hispanic	Women	79	0.00148, 0.00201, 0.00272, 0.00369, 0.005
	Hispanic	Women	80	0.00167, 0.00228, 0.00311, 0.00425, 0.00581
	Hispanic	Women	81	0.00187, 0.00258, 0.00355, 0.00489, 0.00674
	Hispanic	Women	82	0.00207, 0.0029, 0.00403, 0.00561, 0.00783
	Hispanic	Women	83	0.00228, 0.00322, 0.00454, 0.0064, 0.00906
	Hispanic	Women	84	0.00248, 0.00355, 0.00508, 0.00725, 0.0104
	White	Men	30	0.0000152, 0.0000224, 0.0000329, 0.0000484, 0.0000713
	White	Men	31	0.00002, 0.0000286, 0.0000407, 0.0000579, 0.0000826
	White	Men	32	0.0000258, 0.0000359, 0.0000498, 0.0000692, 0.0000964
	White	Men	33	0.0000323, 0.0000442, 0.0000604, 0.0000824, 0.000113
	White	Men	34	0.0000393, 0.0000533, 0.0000721, 0.0000976, 0.000132
	White	Men	35	0.0000463, 0.0000628, 0.0000848, 0.000115, 0.000155
	White	Men	36	0.0000535, 0.0000726, 0.0000983, 0.000133, 0.000181
	White	Men	37	0.0000614, 0.0000833, 0.000113, 0.000152, 0.000206
	White	Men	38	0.0000707, 0.0000951, 0.000128, 0.000171, 0.000231
	White	Men	39	0.0000817, 0.000109, 0.000144, 0.000191, 0.000253
	White	Men	40	0.0000948, 0.000124, 0.000161, 0.000209, 0.000273
	White	Men	41	0.000111, 0.000141, 0.000179, 0.000227, 0.000289
	White	Men	42	0.000129, 0.00016, 0.000198, 0.000244, 0.000303
	White	Men	43	0.000149, 0.00018, 0.000218, 0.000264, 0.000321
	White	Men	44	0.000169, 0.000202, 0.000242, 0.000289, 0.000346
	White	Men	45	0.000192, 0.000228, 0.000269, 0.000318, 0.000377
	White	Men	46	0.000222, 0.000259, 0.000302, 0.000352, 0.000411
	White	Men	47	0.000257, 0.000296, 0.000341, 0.000393, 0.000453
	White	Men	48	0.000297, 0.00034, 0.000388, 0.000444, 0.000508
	White	Men	49	0.000343, 0.000391, 0.000445, 0.000506, 0.000576
	White	Men	50	0.000398, 0.000451, 0.00051, 0.000577, 0.000654
	White	Men	51	0.000464, 0.000521, 0.000585, 0.000656, 0.000737
	White	Men	52	0.000537, 0.000599, 0.000668, 0.000744, 0.00083
	White	Men	53	0.000605, 0.000676, 0.000756, 0.000845, 0.000945
	White	Men	54	0.000665, 0.000751, 0.000848, 0.000957, 0.00108
	White	Men	55	0.000724, 0.000826, 0.000942, 0.00107, 0.00122
	White	Men	56	0.000785, 0.000902, 0.00104, 0.00119, 0.00136
	White	Men	57	0.000843, 0.000976, 0.00113, 0.0013, 0.00151
	White	Men	58	0.0009, 0.00105, 0.00122, 0.00142, 0.00165
	White	Men	59	0.000959, 0.00112, 0.00131, 0.00154, 0.0018
	White	Men	60	0.00102, 0.0012, 0.0014, 0.00165, 0.00194
	White	Men	61	0.00108, 0.00127, 0.00149, 0.00175, 0.00206
	White	Men	62	0.00114, 0.00134, 0.00157, 0.00185, 0.00218
	White	Men	63	0.00119, 0.0014, 0.00165, 0.00194, 0.00228
	White	Men	64	0.00124, 0.00146, 0.00172, 0.00202, 0.00238
	White	Men	65	0.00128, 0.00151, 0.00179, 0.00211, 0.0025

Variable	Race/ethnicity	Sex	Age	Distribution
	White	Men	66	0.00132, 0.00157, 0.00187, 0.00222, 0.00264
	White	Men	67	0.00136, 0.00164, 0.00197, 0.00236, 0.00283
	White	Men	68	0.00142, 0.00173, 0.00209, 0.00254, 0.00308
	White	Men	69	0.0015, 0.00184, 0.00225, 0.00275, 0.00336
	White	Men	70	0.00161, 0.00198, 0.00243, 0.00299, 0.00367
	White	Men	71	0.00174, 0.00215, 0.00264, 0.00325, 0.004
	White	Men	72	0.0019, 0.00234, 0.00288, 0.00354, 0.00436
	White	Men	73	0.00206, 0.00254, 0.00313, 0.00385, 0.00475
	White	Men	74	0.00224, 0.00277, 0.00341, 0.00419, 0.00517
	White	Men	75	0.00244, 0.00301, 0.00371, 0.00458, 0.00565
	White	Men	76	0.00266, 0.0033, 0.00407, 0.00503, 0.00622
	White	Men	77	0.00292, 0.00362, 0.00449, 0.00556, 0.00691
	White	Men	78	0.00321, 0.00401, 0.00499, 0.00621, 0.00774
	White	Men	79	0.00356, 0.00446, 0.00557, 0.00696, 0.00872
	White	Men	80	0.00398, 0.00499, 0.00625, 0.00783, 0.00983
	White	Men	81	0.00446, 0.00561, 0.00703, 0.00881, 0.0111
	White	Men	82	0.00499, 0.00627, 0.00787, 0.00987, 0.0124
	White	Men	83	0.00555, 0.00698, 0.00876, 0.011, 0.0138
	White	Men	84	0.00611, 0.00769, 0.00966, 0.0121, 0.0153
	White	Women	30	0.00000413, 0.00000666, 0.0000107, 0.0000171, 0.0000276
	White	Women	31	0.00000552, 0.00000853, 0.0000131, 0.0000202, 0.0000313
	White	Women	32	0.00000733, 0.0000109, 0.000016, 0.0000237, 0.0000352
	White	Women	33	0.00000957, 0.0000137, 0.0000194, 0.0000277, 0.0000395
	White	Women	34	0.0000121, 0.0000168, 0.0000233, 0.0000322, 0.0000447
	White	Women	35	0.0000148, 0.0000202, 0.0000275, 0.0000374, 0.000051
	White	Women	36	0.0000175, 0.0000236, 0.0000319, 0.000043, 0.0000582
	White	Women	37	0.0000201, 0.0000271, 0.0000365, 0.000049, 0.0000661
	White	Women	38	0.0000226, 0.0000305, 0.0000412, 0.0000554, 0.0000749
	White	Women	39	0.0000249, 0.0000339, 0.0000458, 0.0000621, 0.0000843
	White	Women	40	0.0000273, 0.0000372, 0.0000505, 0.0000686, 0.0000934
	White	Women	41	0.00003, 0.0000408, 0.0000551, 0.0000746, 0.000101
	White	Women	42	0.0000333, 0.0000448, 0.0000599, 0.0000802, 0.000108
	White	Women	43	0.0000369, 0.0000491, 0.0000652, 0.0000865, 0.000115
	White	Women	44	0.0000404, 0.0000537, 0.0000712, 0.0000943, 0.000125
	White	Women	45	0.0000447, 0.0000592, 0.0000783, 0.000103, 0.000137
	White	Women	46	0.0000507, 0.0000664, 0.0000867, 0.000113, 0.000148
	White	Women	47	0.0000593, 0.0000759, 0.000097, 0.000124, 0.000159
	White	Women	48	0.0000707, 0.000088, 0.000109, 0.000136, 0.000169
	White	Women	49	0.0000844, 0.000103, 0.000125, 0.000151, 0.000184
	White	Women	50	0.0000998, 0.000119, 0.000143, 0.00017, 0.000204
	White	Women	51	0.000116, 0.000138, 0.000164, 0.000194, 0.000231
	White	Women	52	0.000134, 0.000159, 0.000188, 0.000223, 0.000264
	White	Women	53	0.000152, 0.000181, 0.000215, 0.000256, 0.000304

Variable	Race/ethnicity	Sex	Age	Distribution
	White	Women	54	0.000171, 0.000205, 0.000245, 0.000292, 0.000349
	White	Women	55	0.000191, 0.00023, 0.000276, 0.000331, 0.000398
	White	Women	56	0.000214, 0.000258, 0.00031, 0.000373, 0.000449
	White	Women	57	0.000239, 0.000288, 0.000347, 0.000418, 0.000505
	White	Women	58	0.000266, 0.000321, 0.000388, 0.000467, 0.000565
	White	Women	59	0.000296, 0.000358, 0.000432, 0.000521, 0.00063
	White	Women	60	0.000325, 0.000395, 0.000478, 0.000579, 0.000703
	White	Women	61	0.000353, 0.000431, 0.000525, 0.000639, 0.000781
	White	Women	62	0.000379, 0.000465, 0.00057, 0.000698, 0.000857
	White	Women	63	0.000405, 0.000499, 0.000613, 0.000754, 0.000928
	White	Women	64	0.000432, 0.000533, 0.000657, 0.000809, 0.000999
	White	Women	65	0.00046, 0.00057, 0.000703, 0.000869, 0.00107
	White	Women	66	0.000494, 0.000612, 0.000758, 0.000938, 0.00116
	White	Women	67	0.000535, 0.000664, 0.000823, 0.00102, 0.00127
	White	Women	68	0.000584, 0.000727, 0.000903, 0.00112, 0.00139
	White	Women	69	0.000644, 0.000802, 0.000997, 0.00124, 0.00154
	White	Women	70	0.000713, 0.000889, 0.00111, 0.00138, 0.00171
	White	Women	71	0.000791, 0.000987, 0.00123, 0.00153, 0.00191
	White	Women	72	0.000875, 0.00109, 0.00137, 0.00171, 0.00214
	White	Women	73	0.000967, 0.00121, 0.00152, 0.00191, 0.00239
	White	Women	74	0.00107, 0.00135, 0.0017, 0.00213, 0.00269
	White	Women	75	0.00118, 0.0015, 0.0019, 0.0024, 0.00303
	White	Women	76	0.00132, 0.00168, 0.00213, 0.0027, 0.00343
	White	Women	77	0.00147, 0.00188, 0.0024, 0.00306, 0.00391
	White	Women	78	0.00165, 0.00213, 0.00272, 0.00349, 0.00448
	White	Women	79	0.00186, 0.00241, 0.0031, 0.004, 0.00516
	White	Women	80	0.00211, 0.00274, 0.00355, 0.0046, 0.00597
	White	Women	81	0.00239, 0.00312, 0.00406, 0.00529, 0.00691
	White	Women	82	0.00269, 0.00354, 0.00464, 0.00608, 0.00798
	White	Women	83	0.00302, 0.00399, 0.00526, 0.00694, 0.00916
	White	Women	84	0.00337, 0.00447, 0.00592, 0.00784, 0.0104
CHD mortality rates for 2035 (0.01, 0.2, 0.5, 0.8. 0.99 percentiles of the empirical distribution produced during forecasting)				
	Black	Men	30	0.0000246, 0.0000376, 0.0000572, 0.0000871, 0.000133
	Black	Men	31	0.0000319, 0.000047, 0.0000691, 0.000102, 0.00015
	Black	Men	32	0.0000408, 0.0000583, 0.0000829, 0.000118, 0.000169
	Black	Men	33	0.0000513, 0.0000711, 0.0000984, 0.000136, 0.000189
	Black	Men	34	0.0000624, 0.000085, 0.000115, 0.000156, 0.000213
	Black	Men	35	0.0000732, 0.0000988, 0.000133, 0.000179, 0.000241
	Black	Men	36	0.0000833, 0.000112, 0.000151, 0.000202, 0.000273
	Black	Men	37	0.000093, 0.000125, 0.000168, 0.000226, 0.000304
	Black	Men	38	0.000103, 0.000138, 0.000186, 0.000249, 0.000336
	Black	Men	39	0.000113, 0.000151, 0.000203, 0.000272, 0.000365
	Black	Men	40	0.000124, 0.000165, 0.00022, 0.000293, 0.000392

Variable	Race/ethnicity	Sex	Age	Distribution
	Black	Men	41	0.000137, 0.000181, 0.000238, 0.000314, 0.000414
	Black	Men	42	0.000153, 0.000199, 0.000258, 0.000335, 0.000436
	Black	Men	43	0.000169, 0.000218, 0.000281, 0.000362, 0.000467
	Black	Men	44	0.000184, 0.000238, 0.000308, 0.000398, 0.000515
	Black	Men	45	0.000203, 0.000263, 0.000341, 0.000442, 0.000575
	Black	Men	46	0.000227, 0.000295, 0.000382, 0.000496, 0.000645
	Black	Men	47	0.000257, 0.000334, 0.000433, 0.000561, 0.000729
	Black	Men	48	0.000295, 0.000383, 0.000497, 0.000643, 0.000836
	Black	Men	49	0.000342, 0.000444, 0.000575, 0.000745, 0.000968
	Black	Men	50	0.000402, 0.00052, 0.000672, 0.000867, 0.00112
	Black	Men	51	0.000479, 0.000614, 0.000786, 0.00101, 0.00129
	Black	Men	52	0.000571, 0.000724, 0.000916, 0.00116, 0.00147
	Black	Men	53	0.000671, 0.000844, 0.00106, 0.00133, 0.00167
	Black	Men	54	0.000771, 0.000966, 0.00121, 0.00151, 0.0019
	Black	Men	55	0.000871, 0.00109, 0.00136, 0.0017, 0.00213
	Black	Men	56	0.000978, 0.00122, 0.00152, 0.00189, 0.00235
	Black	Men	57	0.00109, 0.00135, 0.00167, 0.00206, 0.00256
	Black	Men	58	0.0012, 0.00148, 0.00182, 0.00223, 0.00275
	Black	Men	59	0.0013, 0.0016, 0.00196, 0.0024, 0.00295
	Black	Men	60	0.00141, 0.00172, 0.0021, 0.00256, 0.00312
	Black	Men	61	0.00151, 0.00183, 0.00222, 0.00269, 0.00327
	Black	Men	62	0.0016, 0.00193, 0.00233, 0.0028, 0.00337
	Black	Men	63	0.00169, 0.00202, 0.00242, 0.00289, 0.00345
	Black	Men	64	0.00178, 0.00211, 0.0025, 0.00296, 0.00352
	Black	Men	65	0.00186, 0.0022, 0.00259, 0.00305, 0.00361
	Black	Men	66	0.00193, 0.00229, 0.0027, 0.00318, 0.00376
	Black	Men	67	0.00201, 0.00239, 0.00283, 0.00335, 0.00398
	Black	Men	68	0.0021, 0.00251, 0.003, 0.00357, 0.00426
	Black	Men	69	0.00222, 0.00266, 0.00319, 0.00382, 0.00459
	Black	Men	70	0.00235, 0.00283, 0.00341, 0.00409, 0.00492
	Black	Men	71	0.00251, 0.00302, 0.00363, 0.00437, 0.00527
	Black	Men	72	0.00267, 0.00322, 0.00387, 0.00466, 0.00562
	Black	Men	73	0.00283, 0.00342, 0.00412, 0.00497, 0.006
	Black	Men	74	0.00302, 0.00364, 0.00439, 0.00529, 0.00639
	Black	Men	75	0.00323, 0.0039, 0.00469, 0.00565, 0.00682
	Black	Men	76	0.00348, 0.0042, 0.00504, 0.00606, 0.0073
	Black	Men	77	0.00377, 0.00453, 0.00544, 0.00654, 0.00787
	Black	Men	78	0.00407, 0.00491, 0.0059, 0.0071, 0.00856
	Black	Men	79	0.00439, 0.00531, 0.00641, 0.00774, 0.00937
	Black	Men	80	0.00471, 0.00573, 0.00696, 0.00845, 0.0103
	Black	Men	81	0.00502, 0.00616, 0.00753, 0.00921, 0.0113
	Black	Men	82	0.00531, 0.00657, 0.0081, 0.01, 0.0124
	Black	Men	83	0.00555, 0.00695, 0.00868, 0.0108, 0.0136

Variable	Race/ethnicity	Sex	Age	Distribution
	Black	Men	84	0.00578, 0.00732, 0.00923, 0.0117, 0.0147
	Black	Women	30	0.0000104, 0.000016, 0.0000243, 0.0000371, 0.0000567
	Black	Women	31	0.0000135, 0.0000198, 0.0000289, 0.0000421, 0.0000618
	Black	Women	32	0.000017, 0.0000242, 0.0000342, 0.0000484, 0.0000688
	Black	Women	33	0.000021, 0.0000291, 0.0000404, 0.0000559, 0.0000778
	Black	Women	34	0.0000251, 0.0000345, 0.0000472, 0.0000647, 0.0000889
	Black	Women	35	0.0000289, 0.0000398, 0.0000545, 0.0000748, 0.000103
	Black	Women	36	0.0000326, 0.000045, 0.000062, 0.0000855, 0.000118
	Black	Women	37	0.0000363, 0.0000503, 0.0000696, 0.0000964, 0.000134
	Black	Women	38	0.00004, 0.0000557, 0.0000774, 0.000108, 0.00015
	Black	Women	39	0.0000438, 0.0000613, 0.0000854, 0.000119, 0.000166
	Black	Women	40	0.0000483, 0.0000673, 0.0000935, 0.00013, 0.000181
	Black	Women	41	0.0000542, 0.0000745, 0.000102, 0.00014, 0.000192
	Black	Women	42	0.000062, 0.0000832, 0.000111, 0.000149, 0.000199
	Black	Women	43	0.0000712, 0.0000932, 0.000122, 0.000159, 0.000208
	Black	Women	44	0.0000807, 0.000104, 0.000134, 0.000173, 0.000223
	Black	Women	45	0.0000911, 0.000117, 0.00015, 0.000192, 0.000246
	Black	Women	46	0.000104, 0.000133, 0.000169, 0.000215, 0.000274
	Black	Women	47	0.000121, 0.000153, 0.000193, 0.000243, 0.000307
	Black	Women	48	0.000142, 0.000178, 0.000223, 0.00028, 0.000351
	Black	Women	49	0.000163, 0.000206, 0.00026, 0.000327, 0.000413
	Black	Women	50	0.000188, 0.000239, 0.000303, 0.000383, 0.000486
	Black	Women	51	0.000222, 0.00028, 0.000351, 0.000441, 0.000554
	Black	Women	52	0.000264, 0.000327, 0.000404, 0.000499, 0.000618
	Black	Women	53	0.000303, 0.000374, 0.00046, 0.000567, 0.000699
	Black	Women	54	0.000338, 0.000421, 0.000522, 0.000647, 0.000805
	Black	Women	55	0.000379, 0.000473, 0.000588, 0.000732, 0.000912
	Black	Women	56	0.000429, 0.000533, 0.000659, 0.000816, 0.00101
	Black	Women	57	0.000484, 0.000596, 0.000734, 0.000903, 0.00111
	Black	Women	58	0.000534, 0.000658, 0.00081, 0.000996, 0.00123
	Black	Women	59	0.000578, 0.000716, 0.000885, 0.00109, 0.00136
	Black	Women	60	0.000617, 0.00077, 0.000958, 0.00119, 0.00149
	Black	Women	61	0.000651, 0.000818, 0.00103, 0.00129, 0.00162
	Black	Women	62	0.000679, 0.00086, 0.00109, 0.00137, 0.00174
	Black	Women	63	0.0007, 0.000896, 0.00114, 0.00146, 0.00187
	Black	Women	64	0.000717, 0.000927, 0.0012, 0.00154, 0.002
	Black	Women	65	0.000735, 0.00096, 0.00125, 0.00163, 0.00214
	Black	Women	66	0.00076, 0.001, 0.00132, 0.00174, 0.0023
	Black	Women	67	0.000798, 0.00106, 0.00141, 0.00187, 0.00248
	Black	Women	68	0.000854, 0.00114, 0.00152, 0.00202, 0.0027
	Black	Women	69	0.000929, 0.00124, 0.00165, 0.00219, 0.00293
	Black	Women	70	0.00102, 0.00136, 0.0018, 0.00239, 0.00317
	Black	Women	71	0.00112, 0.00149, 0.00196, 0.00259, 0.00343

Variable	Race/ethnicity	Sex	Age	Distribution
	Black	Women	72	0.00123, 0.00162, 0.00214, 0.00281, 0.0037
	Black	Women	73	0.00134, 0.00177, 0.00232, 0.00305, 0.00401
	Black	Women	74	0.00146, 0.00192, 0.00252, 0.00331, 0.00435
	Black	Women	75	0.00159, 0.00209, 0.00275, 0.00361, 0.00475
	Black	Women	76	0.00172, 0.00228, 0.00301, 0.00397, 0.00525
	Black	Women	77	0.00186, 0.00248, 0.0033, 0.00439, 0.00586
	Black	Women	78	0.002, 0.0027, 0.00364, 0.0049, 0.00662
	Black	Women	79	0.00214, 0.00294, 0.00401, 0.00548, 0.00752
	Black	Women	80	0.00229, 0.00319, 0.00443, 0.00614, 0.00856
	Black	Women	81	0.00245, 0.00346, 0.00488, 0.00686, 0.00969
	Black	Women	82	0.00264, 0.00377, 0.00536, 0.00762, 0.0109
	Black	Women	83	0.00285, 0.0041, 0.00586, 0.00839, 0.0121
	Black	Women	84	0.00308, 0.00444, 0.00639, 0.00918, 0.0132
	Hispanic	Men	30	0.00000849, 0.0000129, 0.0000196, 0.0000297, 0.0000452
	Hispanic	Men	31	0.0000108, 0.0000159, 0.0000234, 0.0000344, 0.0000508
	Hispanic	Men	32	0.0000135, 0.0000194, 0.0000279, 0.00004, 0.0000575
	Hispanic	Men	33	0.0000168, 0.0000236, 0.000033, 0.0000462, 0.0000648
	Hispanic	Men	34	0.0000207, 0.0000284, 0.0000388, 0.0000531, 0.0000728
	Hispanic	Men	35	0.0000249, 0.0000336, 0.0000451, 0.0000606, 0.0000817
	Hispanic	Men	36	0.0000292, 0.0000389, 0.0000518, 0.0000689, 0.000092
	Hispanic	Men	37	0.0000334, 0.0000444, 0.0000588, 0.0000779, 0.000103
	Hispanic	Men	38	0.0000379, 0.0000502, 0.0000663, 0.0000875, 0.000116
	Hispanic	Men	39	0.000043, 0.0000566, 0.0000743, 0.0000976, 0.000129
	Hispanic	Men	40	0.0000491, 0.000064, 0.000083, 0.000108, 0.00014
	Hispanic	Men	41	0.0000569, 0.0000727, 0.0000926, 0.000118, 0.000151
	Hispanic	Men	42	0.0000665, 0.000083, 0.000103, 0.000128, 0.00016
	Hispanic	Men	43	0.0000775, 0.0000946, 0.000115, 0.000141, 0.000172
	Hispanic	Men	44	0.000089, 0.000107, 0.00013, 0.000156, 0.000188
	Hispanic	Men	45	0.000102, 0.000122, 0.000146, 0.000175, 0.00021
	Hispanic	Men	46	0.000116, 0.000139, 0.000166, 0.000199, 0.000238
	Hispanic	Men	47	0.000135, 0.00016, 0.000191, 0.000227, 0.000271
	Hispanic	Men	48	0.000158, 0.000187, 0.000221, 0.00026, 0.000308
	Hispanic	Men	49	0.000188, 0.00022, 0.000257, 0.000301, 0.000353
	Hispanic	Men	50	0.000221, 0.000258, 0.000302, 0.000352, 0.000412
	Hispanic	Men	51	0.000258, 0.000303, 0.000354, 0.000414, 0.000485
	Hispanic	Men	52	0.0003, 0.000353, 0.000414, 0.000486, 0.00057
	Hispanic	Men	53	0.000343, 0.000406, 0.000481, 0.000568, 0.000673
	Hispanic	Men	54	0.000386, 0.000462, 0.000552, 0.00066, 0.00079
	Hispanic	Men	55	0.000434, 0.000522, 0.000627, 0.000752, 0.000904
	Hispanic	Men	56	0.00049, 0.000587, 0.000703, 0.000842, 0.00101
	Hispanic	Men	57	0.000548, 0.000655, 0.000781, 0.000932, 0.00111
	Hispanic	Men	58	0.000607, 0.000723, 0.000861, 0.00102, 0.00122
	Hispanic	Men	59	0.000663, 0.00079, 0.00094, 0.00112, 0.00133

Variable	Race/ethnicity	Sex	Age	Distribution
	Hispanic	Men	60	0.000716, 0.000854, 0.00102, 0.00121, 0.00145
	Hispanic	Men	61	0.000765, 0.000915, 0.00109, 0.0013, 0.00156
	Hispanic	Men	62	0.000811, 0.000971, 0.00116, 0.00139, 0.00166
	Hispanic	Men	63	0.000855, 0.00102, 0.00122, 0.00146, 0.00175
	Hispanic	Men	64	0.000898, 0.00108, 0.00129, 0.00154, 0.00184
	Hispanic	Men	65	0.000944, 0.00113, 0.00135, 0.00162, 0.00194
	Hispanic	Men	66	0.000993, 0.00119, 0.00143, 0.00171, 0.00205
	Hispanic	Men	67	0.00105, 0.00126, 0.00152, 0.00183, 0.00221
	Hispanic	Men	68	0.0011, 0.00134, 0.00163, 0.00198, 0.00241
	Hispanic	Men	69	0.00118, 0.00144, 0.00176, 0.00216, 0.00265
	Hispanic	Men	70	0.00126, 0.00156, 0.00192, 0.00236, 0.00292
	Hispanic	Men	71	0.00136, 0.00169, 0.00209, 0.0026, 0.00323
	Hispanic	Men	72	0.00147, 0.00183, 0.00228, 0.00285, 0.00356
	Hispanic	Men	73	0.00159, 0.00199, 0.00249, 0.00312, 0.00391
	Hispanic	Men	74	0.00174, 0.00218, 0.00272, 0.00341, 0.00428
	Hispanic	Men	75	0.00191, 0.00239, 0.00298, 0.00373, 0.00467
	Hispanic	Men	76	0.0021, 0.00263, 0.00328, 0.0041, 0.00512
	Hispanic	Men	77	0.00234, 0.00292, 0.00364, 0.00453, 0.00565
	Hispanic	Men	78	0.00261, 0.00325, 0.00404, 0.00503, 0.00626
	Hispanic	Men	79	0.00291, 0.00363, 0.00451, 0.0056, 0.00698
	Hispanic	Men	80	0.00321, 0.00402, 0.00501, 0.00626, 0.00782
	Hispanic	Men	81	0.00349, 0.0044, 0.00554, 0.00698, 0.00881
	Hispanic	Men	82	0.00374, 0.00477, 0.00608, 0.00775, 0.00991
	Hispanic	Men	83	0.00396, 0.00513, 0.00662, 0.00855, 0.0111
	Hispanic	Men	84	0.00419, 0.00548, 0.00716, 0.00935, 0.0123
	Hispanic	Women	30	0.00000231, 0.00000361, 0.00000562, 0.00000874, 0.0000137
	Hispanic	Women	31	0.00000288, 0.00000435, 0.00000655, 0.00000987, 0.0000149
	Hispanic	Women	32	0.00000355, 0.00000522, 0.00000764, 0.0000112, 0.0000165
	Hispanic	Women	33	0.00000431, 0.0000062, 0.00000888, 0.0000127, 0.0000183
	Hispanic	Women	34	0.00000512, 0.00000726, 0.0000103, 0.0000145, 0.0000206
	Hispanic	Women	35	0.00000591, 0.00000834, 0.0000117, 0.0000165, 0.0000233
	Hispanic	Women	36	0.00000667, 0.00000941, 0.0000132, 0.0000186, 0.0000263
	Hispanic	Women	37	0.00000742, 0.0000105, 0.0000148, 0.0000209, 0.0000296
	Hispanic	Women	38	0.00000822, 0.0000117, 0.0000165, 0.0000233, 0.000033
	Hispanic	Women	39	0.00000912, 0.0000129, 0.0000182, 0.0000257, 0.0000363
	Hispanic	Women	40	0.0000102, 0.0000144, 0.0000201, 0.000028, 0.0000393
	Hispanic	Women	41	0.0000117, 0.0000161, 0.0000221, 0.0000303, 0.0000417
	Hispanic	Women	42	0.0000137, 0.0000184, 0.0000245, 0.0000326, 0.0000435
	Hispanic	Women	43	0.0000162, 0.000021, 0.0000272, 0.0000353, 0.0000459
	Hispanic	Women	44	0.0000189, 0.0000241, 0.0000306, 0.0000389, 0.0000496
	Hispanic	Women	45	0.0000222, 0.0000278, 0.0000348, 0.0000435, 0.0000545
	Hispanic	Women	46	0.0000264, 0.0000325, 0.0000399, 0.000049, 0.0000602
	Hispanic	Women	47	0.0000318, 0.0000384, 0.0000462, 0.0000556, 0.000067

Variable	Race/ethnicity	Sex	Age	Distribution
	Hispanic	Women	48	0.0000381, 0.0000454, 0.0000539, 0.000064, 0.0000761
	Hispanic	Women	49	0.000045, 0.0000534, 0.0000633, 0.000075, 0.000089
	Hispanic	Women	50	0.0000524, 0.0000627, 0.0000748, 0.0000894, 0.000107
	Hispanic	Women	51	0.0000605, 0.0000734, 0.0000888, 0.000107, 0.00013
	Hispanic	Women	52	0.0000698, 0.0000858, 0.000105, 0.000129, 0.000159
	Hispanic	Women	53	0.0000803, 0.0001, 0.000124, 0.000155, 0.000193
	Hispanic	Women	54	0.0000922, 0.000116, 0.000146, 0.000184, 0.000233
	Hispanic	Women	55	0.000106, 0.000135, 0.000171, 0.000217, 0.000277
	Hispanic	Women	56	0.000122, 0.000156, 0.000199, 0.000254, 0.000325
	Hispanic	Women	57	0.00014, 0.000179, 0.00023, 0.000295, 0.00038
	Hispanic	Women	58	0.000158, 0.000205, 0.000264, 0.000342, 0.000443
	Hispanic	Women	59	0.000177, 0.000231, 0.000301, 0.000393, 0.000514
	Hispanic	Women	60	0.000196, 0.000259, 0.000341, 0.000448, 0.000592
	Hispanic	Women	61	0.000215, 0.000287, 0.000381, 0.000505, 0.000673
	Hispanic	Women	62	0.000235, 0.000315, 0.00042, 0.000562, 0.000752
	Hispanic	Women	63	0.000256, 0.000343, 0.00046, 0.000615, 0.000826
	Hispanic	Women	64	0.000279, 0.000374, 0.000499, 0.000667, 0.000893
	Hispanic	Women	65	0.000305, 0.000407, 0.000541, 0.00072, 0.00096
	Hispanic	Women	66	0.000336, 0.000446, 0.00059, 0.00078, 0.00104
	Hispanic	Women	67	0.000371, 0.000491, 0.000647, 0.000854, 0.00113
	Hispanic	Women	68	0.000411, 0.000543, 0.000716, 0.000945, 0.00125
	Hispanic	Women	69	0.000457, 0.000605, 0.000798, 0.00105, 0.00139
	Hispanic	Women	70	0.000512, 0.000676, 0.000892, 0.00118, 0.00155
	Hispanic	Women	71	0.000574, 0.000758, 0.000998, 0.00131, 0.00173
	Hispanic	Women	72	0.000643, 0.000848, 0.00112, 0.00147, 0.00194
	Hispanic	Women	73	0.00072, 0.00095, 0.00125, 0.00165, 0.00217
	Hispanic	Women	74	0.000806, 0.00107, 0.0014, 0.00185, 0.00245
	Hispanic	Women	75	0.000903, 0.0012, 0.00158, 0.00209, 0.00278
	Hispanic	Women	76	0.00101, 0.00135, 0.00179, 0.00238, 0.00317
	Hispanic	Women	77	0.00114, 0.00152, 0.00204, 0.00272, 0.00365
	Hispanic	Women	78	0.00128, 0.00173, 0.00232, 0.00313, 0.00422
	Hispanic	Women	79	0.00144, 0.00196, 0.00266, 0.0036, 0.0049
	Hispanic	Women	80	0.00162, 0.00222, 0.00304, 0.00415, 0.00568
	Hispanic	Women	81	0.00182, 0.00251, 0.00346, 0.00477, 0.0066
	Hispanic	Women	82	0.00202, 0.00282, 0.00393, 0.00548, 0.00766
	Hispanic	Women	83	0.00221, 0.00314, 0.00443, 0.00626, 0.00887
	Hispanic	Women	84	0.00241, 0.00346, 0.00495, 0.00708, 0.0102
	White	Men	30	0.0000149, 0.0000222, 0.0000328, 0.0000485, 0.000072
	White	Men	31	0.0000197, 0.0000283, 0.0000405, 0.000058, 0.0000834
	White	Men	32	0.0000254, 0.0000355, 0.0000496, 0.0000693, 0.0000972
	White	Men	33	0.0000319, 0.0000439, 0.0000602, 0.0000826, 0.000114
	White	Men	34	0.0000387, 0.0000529, 0.0000719, 0.0000978, 0.000133
	White	Men	35	0.0000456, 0.0000622, 0.0000845, 0.000115, 0.000156

Variable	Race/ethnicity	Sex	Age	Distribution
	White	Men	36	0.0000527, 0.0000719, 0.0000978, 0.000133, 0.000182
	White	Men	37	0.0000604, 0.0000823, 0.000112, 0.000152, 0.000207
	White	Men	38	0.0000693, 0.0000938, 0.000127, 0.000171, 0.000231
	White	Men	39	0.0000799, 0.000107, 0.000142, 0.00019, 0.000253
	White	Men	40	0.0000925, 0.000121, 0.000159, 0.000208, 0.000272
	White	Men	41	0.000108, 0.000138, 0.000176, 0.000225, 0.000287
	White	Men	42	0.000126, 0.000156, 0.000194, 0.000241, 0.0003
	White	Men	43	0.000145, 0.000176, 0.000214, 0.00026, 0.000317
	White	Men	44	0.000164, 0.000198, 0.000237, 0.000284, 0.000341
	White	Men	45	0.000187, 0.000222, 0.000263, 0.000312, 0.000371
	White	Men	46	0.000215, 0.000252, 0.000295, 0.000345, 0.000404
	White	Men	47	0.00025, 0.000289, 0.000333, 0.000385, 0.000445
	White	Men	48	0.000289, 0.000332, 0.00038, 0.000435, 0.000498
	White	Men	49	0.000334, 0.000382, 0.000435, 0.000496, 0.000566
	White	Men	50	0.000388, 0.000441, 0.0005, 0.000567, 0.000643
	White	Men	51	0.000453, 0.00051, 0.000574, 0.000645, 0.000726
	White	Men	52	0.000525, 0.000587, 0.000655, 0.000732, 0.000818
	White	Men	53	0.000592, 0.000664, 0.000743, 0.000832, 0.000933
	White	Men	54	0.000651, 0.000737, 0.000834, 0.000944, 0.00107
	White	Men	55	0.00071, 0.000812, 0.000927, 0.00106, 0.00121
	White	Men	56	0.00077, 0.000887, 0.00102, 0.00117, 0.00135
	White	Men	57	0.000827, 0.000959, 0.00111, 0.00129, 0.00149
	White	Men	58	0.000883, 0.00103, 0.0012, 0.0014, 0.00164
	White	Men	59	0.00094, 0.0011, 0.00129, 0.00152, 0.00178
	White	Men	60	0.000999, 0.00118, 0.00138, 0.00163, 0.00192
	White	Men	61	0.00106, 0.00125, 0.00147, 0.00173, 0.00204
	White	Men	62	0.00111, 0.00131, 0.00155, 0.00182, 0.00215
	White	Men	63	0.00116, 0.00137, 0.00162, 0.00191, 0.00225
	White	Men	64	0.00121, 0.00143, 0.00168, 0.00199, 0.00235
	White	Men	65	0.00125, 0.00148, 0.00175, 0.00207, 0.00245
	White	Men	66	0.00129, 0.00153, 0.00183, 0.00217, 0.00259
	White	Men	67	0.00133, 0.0016, 0.00192, 0.00231, 0.00278
	White	Men	68	0.00138, 0.00168, 0.00204, 0.00248, 0.00302
	White	Men	69	0.00146, 0.00179, 0.0022, 0.00269, 0.0033
	White	Men	70	0.00157, 0.00193, 0.00238, 0.00292, 0.0036
	White	Men	71	0.0017, 0.00209, 0.00258, 0.00318, 0.00393
	White	Men	72	0.00184, 0.00228, 0.00281, 0.00346, 0.00428
	White	Men	73	0.002, 0.00247, 0.00305, 0.00377, 0.00466
	White	Men	74	0.00218, 0.00269, 0.00332, 0.0041, 0.00507
	White	Men	75	0.00237, 0.00293, 0.00362, 0.00448, 0.00554
	White	Men	76	0.00259, 0.00321, 0.00397, 0.00491, 0.00609
	White	Men	77	0.00283, 0.00352, 0.00438, 0.00544, 0.00677
	White	Men	78	0.00312, 0.0039, 0.00486, 0.00607, 0.00758

Variable	Race/ethnicity	Sex	Age	Distribution
	White	Men	79	0.00346, 0.00434, 0.00544, 0.00681, 0.00854
	White	Men	80	0.00386, 0.00486, 0.0061, 0.00766, 0.00964
	White	Men	81	0.00433, 0.00545, 0.00685, 0.00861, 0.0108
	White	Men	82	0.00484, 0.0061, 0.00768, 0.00965, 0.0122
	White	Men	83	0.00538, 0.00678, 0.00854, 0.0107, 0.0136
	White	Men	84	0.00592, 0.00748, 0.00941, 0.0119, 0.015
	White	Women	30	0.00000407, 0.00000659, 0.0000106, 0.0000172, 0.0000278
	White	Women	31	0.00000544, 0.00000845, 0.0000131, 0.0000202, 0.0000315
	White	Women	32	0.00000723, 0.0000108, 0.000016, 0.0000237, 0.0000354
	White	Women	33	0.00000945, 0.0000136, 0.0000194, 0.0000277, 0.0000397
	White	Women	34	0.000012, 0.0000167, 0.0000232, 0.0000323, 0.000045
	White	Women	35	0.0000146, 0.00002, 0.0000273, 0.0000374, 0.0000513
	White	Women	36	0.0000172, 0.0000234, 0.0000317, 0.000043, 0.0000585
	White	Women	37	0.0000198, 0.0000268, 0.0000362, 0.000049, 0.0000664
	White	Women	38	0.0000222, 0.0000301, 0.0000408, 0.0000552, 0.0000751
	White	Women	39	0.0000244, 0.0000333, 0.0000453, 0.0000617, 0.0000842
	White	Women	40	0.0000267, 0.0000365, 0.0000498, 0.0000679, 0.000093
	White	Women	41	0.0000293, 0.0000399, 0.0000542, 0.0000737, 0.0001
	White	Women	42	0.0000325, 0.0000438, 0.0000588, 0.000079, 0.000106
	White	Women	43	0.000036, 0.000048, 0.0000639, 0.000085, 0.000113
	White	Women	44	0.0000394, 0.0000525, 0.0000696, 0.0000924, 0.000123
	White	Women	45	0.0000435, 0.0000578, 0.0000765, 0.000101, 0.000134
	White	Women	46	0.0000494, 0.0000648, 0.0000847, 0.000111, 0.000145
	White	Women	47	0.0000578, 0.0000741, 0.0000947, 0.000121, 0.000155
	White	Women	48	0.0000689, 0.0000859, 0.000107, 0.000133, 0.000166
	White	Women	49	0.0000823, 0.0001, 0.000122, 0.000148, 0.00018
	White	Women	50	0.0000974, 0.000117, 0.00014, 0.000167, 0.0002
	White	Women	51	0.000114, 0.000135, 0.000161, 0.000191, 0.000227
	White	Women	52	0.000131, 0.000156, 0.000185, 0.000219, 0.00026
	White	Women	53	0.000149, 0.000178, 0.000212, 0.000252, 0.0003
	White	Women	54	0.000168, 0.000201, 0.000241, 0.000288, 0.000345
	White	Women	55	0.000188, 0.000226, 0.000272, 0.000326, 0.000393
	White	Women	56	0.00021, 0.000253, 0.000305, 0.000368, 0.000444
	White	Women	57	0.000234, 0.000283, 0.000342, 0.000413, 0.000499
	White	Women	58	0.000261, 0.000316, 0.000382, 0.000461, 0.000559
	White	Women	59	0.00029, 0.000352, 0.000425, 0.000515, 0.000624
	White	Women	60	0.000319, 0.000388, 0.000471, 0.000572, 0.000696
	White	Women	61	0.000345, 0.000423, 0.000516, 0.000631, 0.000772
	White	Women	62	0.000371, 0.000456, 0.00056, 0.000688, 0.000846
	White	Women	63	0.000396, 0.000489, 0.000602, 0.000741, 0.000915
	White	Women	64	0.000422, 0.000521, 0.000644, 0.000795, 0.000983
	White	Women	65	0.000449, 0.000557, 0.000689, 0.000852, 0.00106
	White	Women	66	0.000481, 0.000598, 0.000741, 0.000919, 0.00114

Variable	Race/ethnicity	Sex	Age	Distribution
	White	Women	67	0.000521, 0.000648, 0.000805, 0.000999, 0.00124
	White	Women	68	0.000569, 0.000709, 0.000882, 0.0011, 0.00137
	White	Women	69	0.000626, 0.000782, 0.000974, 0.00121, 0.00151
	White	Women	70	0.000694, 0.000866, 0.00108, 0.00135, 0.00168
	White	Women	71	0.000769, 0.000961, 0.0012, 0.0015, 0.00187
	White	Women	72	0.000851, 0.00107, 0.00133, 0.00167, 0.00209
	White	Women	73	0.000939, 0.00118, 0.00148, 0.00186, 0.00235
	White	Women	74	0.00104, 0.00131, 0.00165, 0.00209, 0.00264
	White	Women	75	0.00115, 0.00146, 0.00185, 0.00234, 0.00297
	White	Women	76	0.00128, 0.00163, 0.00207, 0.00264, 0.00336
	White	Women	77	0.00143, 0.00183, 0.00234, 0.00299, 0.00382
	White	Women	78	0.00161, 0.00207, 0.00265, 0.00341, 0.00438
	White	Women	79	0.00181, 0.00234, 0.00303, 0.00391, 0.00505
	White	Women	80	0.00205, 0.00267, 0.00346, 0.00449, 0.00585
	White	Women	81	0.00232, 0.00303, 0.00396, 0.00517, 0.00677
	White	Women	82	0.00261, 0.00344, 0.00452, 0.00594, 0.00782
	White	Women	83	0.00293, 0.00389, 0.00513, 0.00678, 0.00898
	White	Women	84	0.00327, 0.00435, 0.00577, 0.00766, 0.0102
CHD mortality rates for 2036 (0.01, 0.2, 0.5, 0.8. 0.99 percentiles of the empirical distribution produced during forecasting)				
	Black	Men	30	0.0000241, 0.0000371, 0.0000569, 0.0000872, 0.000134
	Black	Men	31	0.0000314, 0.0000465, 0.0000688, 0.000102, 0.000151
	Black	Men	32	0.0000402, 0.0000577, 0.0000826, 0.000118, 0.00017
	Black	Men	33	0.0000505, 0.0000705, 0.000098, 0.000136, 0.00019
	Black	Men	34	0.0000615, 0.0000842, 0.000115, 0.000157, 0.000214
	Black	Men	35	0.0000721, 0.0000978, 0.000132, 0.000179, 0.000243
	Black	Men	36	0.0000819, 0.000111, 0.00015, 0.000202, 0.000274
	Black	Men	37	0.0000913, 0.000124, 0.000167, 0.000226, 0.000306
	Black	Men	38	0.000101, 0.000136, 0.000184, 0.000248, 0.000336
	Black	Men	39	0.00011, 0.000149, 0.000201, 0.00027, 0.000365
	Black	Men	40	0.000121, 0.000162, 0.000217, 0.000291, 0.00039
	Black	Men	41	0.000134, 0.000177, 0.000234, 0.00031, 0.000411
	Black	Men	42	0.000149, 0.000195, 0.000253, 0.00033, 0.000431
	Black	Men	43	0.000164, 0.000213, 0.000275, 0.000355, 0.00046
	Black	Men	44	0.00018, 0.000233, 0.000301, 0.00039, 0.000506
	Black	Men	45	0.000197, 0.000257, 0.000333, 0.000433, 0.000564
	Black	Men	46	0.000221, 0.000287, 0.000373, 0.000485, 0.000632
	Black	Men	47	0.000251, 0.000326, 0.000423, 0.000549, 0.000714
	Black	Men	48	0.000288, 0.000374, 0.000485, 0.000629, 0.000819
	Black	Men	49	0.000334, 0.000434, 0.000563, 0.00073, 0.000949
	Black	Men	50	0.000393, 0.000509, 0.000658, 0.00085, 0.0011
	Black	Men	51	0.000468, 0.000601, 0.00077, 0.000987, 0.00127
	Black	Men	52	0.00056, 0.00071, 0.000899, 0.00114, 0.00144
	Black	Men	53	0.000659, 0.000829, 0.00104, 0.00131, 0.00165

Variable	Race/ethnicity	Sex	Age	Distribution
	Black	Men	54	0.000756, 0.00095, 0.00119, 0.00149, 0.00187
	Black	Men	55	0.000856, 0.00107, 0.00134, 0.00168, 0.00211
	Black	Men	56	0.000961, 0.0012, 0.00149, 0.00186, 0.00232
	Black	Men	57	0.00107, 0.00133, 0.00164, 0.00204, 0.00253
	Black	Men	58	0.00118, 0.00145, 0.00179, 0.00221, 0.00273
	Black	Men	59	0.00128, 0.00157, 0.00193, 0.00237, 0.00292
	Black	Men	60	0.00138, 0.00169, 0.00207, 0.00252, 0.00309
	Black	Men	61	0.00148, 0.0018, 0.00219, 0.00266, 0.00323
	Black	Men	62	0.00157, 0.0019, 0.00229, 0.00276, 0.00333
	Black	Men	63	0.00165, 0.00198, 0.00237, 0.00284, 0.0034
	Black	Men	64	0.00173, 0.00206, 0.00245, 0.00291, 0.00346
	Black	Men	65	0.00181, 0.00214, 0.00253, 0.003, 0.00355
	Black	Men	66	0.00188, 0.00223, 0.00264, 0.00312, 0.00369
	Black	Men	67	0.00196, 0.00233, 0.00277, 0.00328, 0.00391
	Black	Men	68	0.00205, 0.00245, 0.00293, 0.0035, 0.00419
	Black	Men	69	0.00216, 0.00259, 0.00312, 0.00374, 0.00451
	Black	Men	70	0.00229, 0.00276, 0.00333, 0.00401, 0.00484
	Black	Men	71	0.00244, 0.00294, 0.00355, 0.00428, 0.00517
	Black	Men	72	0.00259, 0.00313, 0.00378, 0.00456, 0.00552
	Black	Men	73	0.00275, 0.00333, 0.00402, 0.00486, 0.00588
	Black	Men	74	0.00293, 0.00355, 0.00429, 0.00518, 0.00627
	Black	Men	75	0.00314, 0.00379, 0.00458, 0.00553, 0.00668
	Black	Men	76	0.00338, 0.00408, 0.00492, 0.00593, 0.00715
	Black	Men	77	0.00365, 0.00441, 0.00531, 0.00639, 0.00772
	Black	Men	78	0.00395, 0.00477, 0.00576, 0.00695, 0.00839
	Black	Men	79	0.00426, 0.00517, 0.00625, 0.00757, 0.00919
	Black	Men	80	0.00457, 0.00558, 0.00679, 0.00826, 0.0101
	Black	Men	81	0.00487, 0.00599, 0.00734, 0.00901, 0.0111
	Black	Men	82	0.00515, 0.00639, 0.0079, 0.00979, 0.0121
	Black	Men	83	0.00539, 0.00676, 0.00846, 0.0106, 0.0133
	Black	Men	84	0.00561, 0.00711, 0.009, 0.0114, 0.0145
	Black	Women	30	0.0000102, 0.0000158, 0.0000242, 0.0000371, 0.0000572
	Black	Women	31	0.0000133, 0.0000196, 0.0000287, 0.0000422, 0.0000623
	Black	Women	32	0.0000167, 0.0000239, 0.0000341, 0.0000485, 0.0000694
	Black	Women	33	0.0000207, 0.0000289, 0.0000402, 0.000056, 0.0000783
	Black	Women	34	0.0000248, 0.0000342, 0.0000471, 0.0000648, 0.0000896
	Black	Women	35	0.0000285, 0.0000394, 0.0000543, 0.0000748, 0.000103
	Black	Women	36	0.0000321, 0.0000446, 0.0000617, 0.0000855, 0.000119
	Black	Women	37	0.0000357, 0.0000498, 0.0000692, 0.0000962, 0.000134
	Black	Women	38	0.0000393, 0.000055, 0.0000768, 0.000107, 0.00015
	Black	Women	39	0.000043, 0.0000603, 0.0000845, 0.000118, 0.000166
	Black	Women	40	0.0000473, 0.0000662, 0.0000924, 0.000129, 0.000181
	Black	Women	41	0.000053, 0.0000731, 0.000101, 0.000138, 0.000191

Variable	Race/ethnicity	Sex	Age	Distribution
	Black	Women	42	0.0000606, 0.0000815, 0.000109, 0.000147, 0.000197
	Black	Women	43	0.0000696, 0.0000913, 0.000119, 0.000156, 0.000205
	Black	Women	44	0.0000788, 0.000102, 0.000132, 0.00017, 0.00022
	Black	Women	45	0.0000888, 0.000114, 0.000147, 0.000188, 0.000242
	Black	Women	46	0.000101, 0.00013, 0.000165, 0.000211, 0.000269
	Black	Women	47	0.000118, 0.00015, 0.000189, 0.000238, 0.000302
	Black	Women	48	0.000139, 0.000174, 0.000218, 0.000274, 0.000344
	Black	Women	49	0.00016, 0.000202, 0.000255, 0.000321, 0.000406
	Black	Women	50	0.000184, 0.000234, 0.000297, 0.000376, 0.000478
	Black	Women	51	0.000218, 0.000274, 0.000345, 0.000433, 0.000546
	Black	Women	52	0.000259, 0.000321, 0.000397, 0.000491, 0.000609
	Black	Women	53	0.000298, 0.000368, 0.000453, 0.000559, 0.00069
	Black	Women	54	0.000332, 0.000414, 0.000514, 0.000639, 0.000795
	Black	Women	55	0.000372, 0.000465, 0.00058, 0.000723, 0.000903
	Black	Women	56	0.000422, 0.000524, 0.00065, 0.000806, 0.001
	Black	Women	57	0.000475, 0.000587, 0.000724, 0.000892, 0.0011
	Black	Women	58	0.000524, 0.000648, 0.000799, 0.000985, 0.00122
	Black	Women	59	0.000567, 0.000705, 0.000873, 0.00108, 0.00134
	Black	Women	60	0.000606, 0.000757, 0.000945, 0.00118, 0.00147
	Black	Women	61	0.000639, 0.000804, 0.00101, 0.00127, 0.0016
	Black	Women	62	0.000665, 0.000845, 0.00107, 0.00136, 0.00172
	Black	Women	63	0.000685, 0.000879, 0.00112, 0.00144, 0.00184
	Black	Women	64	0.000701, 0.000908, 0.00117, 0.00152, 0.00197
	Black	Women	65	0.000718, 0.00094, 0.00123, 0.0016, 0.0021
	Black	Women	66	0.000742, 0.000981, 0.00129, 0.00171, 0.00226
	Black	Women	67	0.000779, 0.00104, 0.00138, 0.00183, 0.00244
	Black	Women	68	0.000833, 0.00111, 0.00148, 0.00198, 0.00265
	Black	Women	69	0.000906, 0.00121, 0.00161, 0.00215, 0.00287
	Black	Women	70	0.000994, 0.00132, 0.00176, 0.00234, 0.00311
	Black	Women	71	0.00109, 0.00145, 0.00192, 0.00254, 0.00336
	Black	Women	72	0.0012, 0.00158, 0.00209, 0.00275, 0.00363
	Black	Women	73	0.00131, 0.00172, 0.00227, 0.00298, 0.00393
	Black	Women	74	0.00142, 0.00187, 0.00246, 0.00324, 0.00427
	Black	Women	75	0.00155, 0.00204, 0.00268, 0.00353, 0.00466
	Black	Women	76	0.00168, 0.00222, 0.00294, 0.00388, 0.00514
	Black	Women	77	0.00181, 0.00242, 0.00322, 0.0043, 0.00575
	Black	Women	78	0.00195, 0.00263, 0.00355, 0.00479, 0.00649
	Black	Women	79	0.00208, 0.00286, 0.00392, 0.00537, 0.00737
	Black	Women	80	0.00223, 0.00311, 0.00433, 0.00602, 0.00839
	Black	Women	81	0.00239, 0.00338, 0.00477, 0.00672, 0.00951
	Black	Women	82	0.00257, 0.00367, 0.00524, 0.00746, 0.0107
	Black	Women	83	0.00277, 0.00399, 0.00573, 0.00822, 0.0118
	Black	Women	84	0.003, 0.00433, 0.00624, 0.00899, 0.013

Variable	Race/ethnicity	Sex	Age	Distribution
	Hispanic	Men	30	0.00000834, 0.0000128, 0.0000195, 0.0000297, 0.0000456
	Hispanic	Men	31	0.0000106, 0.0000157, 0.0000233, 0.0000345, 0.0000512
	Hispanic	Men	32	0.0000133, 0.0000193, 0.0000278, 0.00004, 0.0000579
	Hispanic	Men	33	0.0000166, 0.0000234, 0.0000329, 0.0000463, 0.0000652
	Hispanic	Men	34	0.0000204, 0.0000281, 0.0000387, 0.0000531, 0.0000732
	Hispanic	Men	35	0.0000245, 0.0000332, 0.0000449, 0.0000607, 0.0000823
	Hispanic	Men	36	0.0000287, 0.0000385, 0.0000515, 0.0000689, 0.0000925
	Hispanic	Men	37	0.0000328, 0.0000439, 0.0000584, 0.0000778, 0.000104
	Hispanic	Men	38	0.0000372, 0.0000495, 0.0000657, 0.0000872, 0.000116
	Hispanic	Men	39	0.000042, 0.0000556, 0.0000735, 0.0000971, 0.000129
	Hispanic	Men	40	0.0000479, 0.0000628, 0.0000819, 0.000107, 0.00014
	Hispanic	Men	41	0.0000555, 0.0000712, 0.0000911, 0.000117, 0.00015
	Hispanic	Men	42	0.0000648, 0.0000812, 0.000101, 0.000127, 0.000158
	Hispanic	Men	43	0.0000755, 0.0000925, 0.000113, 0.000138, 0.000169
	Hispanic	Men	44	0.0000867, 0.000105, 0.000127, 0.000153, 0.000185
	Hispanic	Men	45	0.0000989, 0.000119, 0.000143, 0.000172, 0.000207
	Hispanic	Men	46	0.000113, 0.000136, 0.000163, 0.000195, 0.000234
	Hispanic	Men	47	0.000131, 0.000156, 0.000186, 0.000222, 0.000266
	Hispanic	Men	48	0.000154, 0.000183, 0.000216, 0.000255, 0.000302
	Hispanic	Men	49	0.000183, 0.000215, 0.000252, 0.000295, 0.000347
	Hispanic	Men	50	0.000215, 0.000252, 0.000295, 0.000346, 0.000405
	Hispanic	Men	51	0.000252, 0.000296, 0.000347, 0.000406, 0.000477
	Hispanic	Men	52	0.000294, 0.000346, 0.000406, 0.000477, 0.000561
	Hispanic	Men	53	0.000336, 0.000399, 0.000472, 0.000559, 0.000664
	Hispanic	Men	54	0.000379, 0.000454, 0.000543, 0.00065, 0.000779
	Hispanic	Men	55	0.000426, 0.000513, 0.000617, 0.000742, 0.000893
	Hispanic	Men	56	0.000481, 0.000578, 0.000693, 0.000831, 0.000999
	Hispanic	Men	57	0.000538, 0.000644, 0.00077, 0.000921, 0.0011
	Hispanic	Men	58	0.000595, 0.000711, 0.000848, 0.00101, 0.00121
	Hispanic	Men	59	0.00065, 0.000777, 0.000926, 0.0011, 0.00132
	Hispanic	Men	60	0.000701, 0.000839, 0.001, 0.0012, 0.00143
	Hispanic	Men	61	0.000749, 0.000898, 0.00107, 0.00128, 0.00154
	Hispanic	Men	62	0.000793, 0.000952, 0.00114, 0.00137, 0.00164
	Hispanic	Men	63	0.000835, 0.001, 0.0012, 0.00144, 0.00173
	Hispanic	Men	64	0.000876, 0.00105, 0.00126, 0.00151, 0.00181
	Hispanic	Men	65	0.00092, 0.0011, 0.00132, 0.00159, 0.0019
	Hispanic	Men	66	0.000967, 0.00116, 0.0014, 0.00168, 0.00202
	Hispanic	Men	67	0.00102, 0.00123, 0.00149, 0.00179, 0.00217
	Hispanic	Men	68	0.00107, 0.00131, 0.00159, 0.00194, 0.00236
	Hispanic	Men	69	0.00114, 0.0014, 0.00172, 0.00211, 0.0026
	Hispanic	Men	70	0.00123, 0.00152, 0.00187, 0.00231, 0.00286
	Hispanic	Men	71	0.00132, 0.00164, 0.00204, 0.00254, 0.00316
	Hispanic	Men	72	0.00143, 0.00179, 0.00223, 0.00279, 0.00349

Variable	Race/ethnicity	Sex	Age	Distribution
	Hispanic	Men	73	0.00155, 0.00194, 0.00243, 0.00305, 0.00383
	Hispanic	Men	74	0.00169, 0.00212, 0.00266, 0.00333, 0.00419
	Hispanic	Men	75	0.00185, 0.00232, 0.00291, 0.00364, 0.00457
	Hispanic	Men	76	0.00204, 0.00256, 0.0032, 0.004, 0.00502
	Hispanic	Men	77	0.00227, 0.00284, 0.00354, 0.00442, 0.00553
	Hispanic	Men	78	0.00254, 0.00317, 0.00394, 0.00491, 0.00613
	Hispanic	Men	79	0.00283, 0.00353, 0.0044, 0.00548, 0.00684
	Hispanic	Men	80	0.00312, 0.00391, 0.00489, 0.00612, 0.00767
	Hispanic	Men	81	0.00339, 0.00428, 0.00541, 0.00682, 0.00863
	Hispanic	Men	82	0.00363, 0.00465, 0.00593, 0.00758, 0.00971
	Hispanic	Men	83	0.00385, 0.00499, 0.00646, 0.00836, 0.0109
	Hispanic	Men	84	0.00406, 0.00533, 0.00698, 0.00914, 0.012
	Hispanic	Women	30	0.00000227, 0.00000357, 0.00000559, 0.00000876, 0.0000138
	Hispanic	Women	31	0.00000283, 0.00000431, 0.00000652, 0.00000988, 0.000015
	Hispanic	Women	32	0.0000035, 0.00000517, 0.00000761, 0.0000112, 0.0000166
	Hispanic	Women	33	0.00000426, 0.00000615, 0.00000886, 0.0000127, 0.0000184
	Hispanic	Women	34	0.00000506, 0.0000072, 0.0000102, 0.0000145, 0.0000207
	Hispanic	Women	35	0.00000583, 0.00000826, 0.0000117, 0.0000165, 0.0000234
	Hispanic	Women	36	0.00000657, 0.00000932, 0.0000132, 0.0000186, 0.0000264
	Hispanic	Women	37	0.0000073, 0.0000104, 0.0000147, 0.0000209, 0.0000297
	Hispanic	Women	38	0.00000807, 0.0000115, 0.0000163, 0.0000232, 0.000033
	Hispanic	Women	39	0.00000894, 0.0000127, 0.000018, 0.0000255, 0.0000362
	Hispanic	Women	40	0.00001, 0.0000141, 0.0000198, 0.0000278, 0.0000391
	Hispanic	Women	41	0.0000115, 0.0000158, 0.0000218, 0.0000299, 0.0000413
	Hispanic	Women	42	0.0000134, 0.000018, 0.000024, 0.0000321, 0.000043
	Hispanic	Women	43	0.0000158, 0.0000205, 0.0000267, 0.0000347, 0.0000452
	Hispanic	Women	44	0.0000184, 0.0000235, 0.00003, 0.0000382, 0.0000488
	Hispanic	Women	45	0.0000216, 0.0000271, 0.000034, 0.0000426, 0.0000535
	Hispanic	Women	46	0.0000257, 0.0000317, 0.000039, 0.0000479, 0.0000591
	Hispanic	Women	47	0.000031, 0.0000374, 0.0000451, 0.0000544, 0.0000657
	Hispanic	Women	48	0.0000371, 0.0000443, 0.0000526, 0.0000626, 0.0000746
	Hispanic	Women	49	0.0000439, 0.0000522, 0.0000619, 0.0000735, 0.0000874
	Hispanic	Women	50	0.0000511, 0.0000613, 0.0000733, 0.0000877, 0.000105
	Hispanic	Women	51	0.0000592, 0.0000718, 0.000087, 0.000105, 0.000128
	Hispanic	Women	52	0.0000684, 0.0000842, 0.000103, 0.000127, 0.000156
	Hispanic	Women	53	0.0000788, 0.0000983, 0.000122, 0.000152, 0.00019
	Hispanic	Women	54	0.0000905, 0.000114, 0.000144, 0.000182, 0.00023
	Hispanic	Women	55	0.000104, 0.000133, 0.000169, 0.000214, 0.000273
	Hispanic	Women	56	0.00012, 0.000154, 0.000196, 0.000251, 0.000321
	Hispanic	Women	57	0.000137, 0.000177, 0.000227, 0.000292, 0.000376
	Hispanic	Women	58	0.000155, 0.000201, 0.000261, 0.000337, 0.000438
	Hispanic	Women	59	0.000173, 0.000227, 0.000297, 0.000388, 0.000508
	Hispanic	Women	60	0.000192, 0.000254, 0.000335, 0.000442, 0.000585

Variable	Race/ethnicity	Sex	Age	Distribution
	Hispanic	Women	61	0.000211, 0.000282, 0.000374, 0.000498, 0.000664
	Hispanic	Women	62	0.00023, 0.000309, 0.000413, 0.000553, 0.000742
	Hispanic	Women	63	0.00025, 0.000337, 0.000451, 0.000605, 0.000813
	Hispanic	Women	64	0.000273, 0.000366, 0.000489, 0.000655, 0.000878
	Hispanic	Women	65	0.000298, 0.000398, 0.00053, 0.000706, 0.000942
	Hispanic	Women	66	0.000328, 0.000435, 0.000577, 0.000764, 0.00102
	Hispanic	Women	67	0.000361, 0.000479, 0.000633, 0.000836, 0.00111
	Hispanic	Women	68	0.0004, 0.00053, 0.0007, 0.000925, 0.00122
	Hispanic	Women	69	0.000445, 0.00059, 0.000779, 0.00103, 0.00136
	Hispanic	Women	70	0.000498, 0.00066, 0.000871, 0.00115, 0.00152
	Hispanic	Women	71	0.000558, 0.000739, 0.000974, 0.00129, 0.0017
	Hispanic	Women	72	0.000626, 0.000827, 0.00109, 0.00144, 0.0019
	Hispanic	Women	73	0.0007, 0.000926, 0.00122, 0.00161, 0.00213
	Hispanic	Women	74	0.000783, 0.00104, 0.00137, 0.00181, 0.0024
	Hispanic	Women	75	0.000878, 0.00117, 0.00154, 0.00205, 0.00272
	Hispanic	Women	76	0.000985, 0.00131, 0.00175, 0.00233, 0.0031
	Hispanic	Women	77	0.00111, 0.00148, 0.00199, 0.00266, 0.00357
	Hispanic	Women	78	0.00124, 0.00168, 0.00227, 0.00305, 0.00413
	Hispanic	Women	79	0.0014, 0.00191, 0.00259, 0.00352, 0.00479
	Hispanic	Women	80	0.00158, 0.00216, 0.00296, 0.00405, 0.00556
	Hispanic	Women	81	0.00177, 0.00245, 0.00338, 0.00466, 0.00646
	Hispanic	Women	82	0.00196, 0.00274, 0.00383, 0.00535, 0.0075
	Hispanic	Women	83	0.00215, 0.00305, 0.00432, 0.00611, 0.00867
	Hispanic	Women	84	0.00234, 0.00336, 0.00482, 0.00692, 0.00995
	White	Men	30	0.0000146, 0.0000219, 0.0000326, 0.0000486, 0.0000727
	White	Men	31	0.0000194, 0.000028, 0.0000403, 0.0000581, 0.0000841
	White	Men	32	0.000025, 0.0000352, 0.0000495, 0.0000695, 0.000098
	White	Men	33	0.0000314, 0.0000435, 0.00006, 0.0000827, 0.000114
	White	Men	34	0.0000382, 0.0000524, 0.0000716, 0.000098, 0.000134
	White	Men	35	0.000045, 0.0000616, 0.0000842, 0.000115, 0.000157
	White	Men	36	0.0000518, 0.0000711, 0.0000973, 0.000133, 0.000182
	White	Men	37	0.0000593, 0.0000813, 0.000111, 0.000152, 0.000208
	White	Men	38	0.0000679, 0.0000925, 0.000125, 0.00017, 0.000232
	White	Men	39	0.0000781, 0.000105, 0.000141, 0.000189, 0.000253
	White	Men	40	0.0000903, 0.000119, 0.000157, 0.000206, 0.000272
	White	Men	41	0.000105, 0.000135, 0.000173, 0.000222, 0.000286
	White	Men	42	0.000123, 0.000153, 0.000191, 0.000238, 0.000297
	White	Men	43	0.000141, 0.000172, 0.00021, 0.000256, 0.000312
	White	Men	44	0.00016, 0.000193, 0.000232, 0.000279, 0.000336
	White	Men	45	0.000182, 0.000217, 0.000258, 0.000306, 0.000365
	White	Men	46	0.000209, 0.000246, 0.000288, 0.000338, 0.000397
	White	Men	47	0.000243, 0.000281, 0.000326, 0.000377, 0.000437
	White	Men	48	0.000281, 0.000323, 0.000371, 0.000426, 0.000489

Variable	Race/ethnicity	Sex	Age	Distribution
	White	Men	49	0.000325, 0.000372, 0.000425, 0.000486, 0.000556
	White	Men	50	0.000378, 0.00043, 0.000489, 0.000556, 0.000633
	White	Men	51	0.000442, 0.000499, 0.000562, 0.000633, 0.000715
	White	Men	52	0.000513, 0.000575, 0.000643, 0.00072, 0.000806
	White	Men	53	0.000579, 0.000651, 0.00073, 0.00082, 0.000921
	White	Men	54	0.000638, 0.000724, 0.000821, 0.000931, 0.00106
	White	Men	55	0.000696, 0.000797, 0.000913, 0.00105, 0.0012
	White	Men	56	0.000755, 0.000871, 0.001, 0.00116, 0.00134
	White	Men	57	0.000811, 0.000943, 0.0011, 0.00127, 0.00148
	White	Men	58	0.000865, 0.00101, 0.00119, 0.00139, 0.00162
	White	Men	59	0.000921, 0.00108, 0.00127, 0.0015, 0.00176
	White	Men	60	0.000978, 0.00116, 0.00136, 0.00161, 0.0019
	White	Men	61	0.00103, 0.00122, 0.00145, 0.00171, 0.00202
	White	Men	62	0.00109, 0.00129, 0.00152, 0.0018, 0.00213
	White	Men	63	0.00113, 0.00134, 0.00159, 0.00188, 0.00223
	White	Men	64	0.00118, 0.00139, 0.00165, 0.00195, 0.00231
	White	Men	65	0.00121, 0.00144, 0.00171, 0.00203, 0.00241
	White	Men	66	0.00125, 0.0015, 0.00179, 0.00213, 0.00255
	White	Men	67	0.00129, 0.00156, 0.00188, 0.00226, 0.00273
	White	Men	68	0.00135, 0.00164, 0.002, 0.00243, 0.00297
	White	Men	69	0.00142, 0.00175, 0.00215, 0.00263, 0.00324
	White	Men	70	0.00152, 0.00188, 0.00232, 0.00286, 0.00353
	White	Men	71	0.00165, 0.00204, 0.00252, 0.00311, 0.00385
	White	Men	72	0.00179, 0.00222, 0.00274, 0.00339, 0.00419
	White	Men	73	0.00195, 0.00241, 0.00298, 0.00368, 0.00456
	White	Men	74	0.00211, 0.00262, 0.00324, 0.00401, 0.00497
	White	Men	75	0.0023, 0.00286, 0.00353, 0.00437, 0.00543
	White	Men	76	0.00251, 0.00312, 0.00387, 0.0048, 0.00597
	White	Men	77	0.00275, 0.00343, 0.00427, 0.00531, 0.00663
	White	Men	78	0.00303, 0.00379, 0.00474, 0.00593, 0.00743
	White	Men	79	0.00336, 0.00422, 0.0053, 0.00665, 0.00837
	White	Men	80	0.00375, 0.00473, 0.00595, 0.00749, 0.00944
	White	Men	81	0.0042, 0.00531, 0.00669, 0.00842, 0.0106
	White	Men	82	0.0047, 0.00594, 0.00749, 0.00944, 0.0119
	White	Men	83	0.00522, 0.0066, 0.00833, 0.0105, 0.0133
	White	Men	84	0.00574, 0.00727, 0.00918, 0.0116, 0.0147
	White	Women	30	0.000004, 0.00000653, 0.0000106, 0.0000172, 0.000028
	White	Women	31	0.00000536, 0.00000838, 0.000013, 0.0000203, 0.0000317
	White	Women	32	0.00000713, 0.0000107, 0.0000159, 0.0000238, 0.0000356
	White	Women	33	0.00000933, 0.0000134, 0.0000193, 0.0000277, 0.00004
	White	Women	34	0.0000118, 0.0000165, 0.0000231, 0.0000323, 0.0000453
	White	Women	35	0.0000144, 0.0000198, 0.0000272, 0.0000374, 0.0000516
	White	Women	36	0.0000169, 0.0000232, 0.0000316, 0.000043, 0.0000588

Variable	Race/ethnicity	Sex	Age	Distribution
	White	Women	37	0.0000194, 0.0000265, 0.000036, 0.0000489, 0.0000666
	White	Women	38	0.0000217, 0.0000297, 0.0000404, 0.000055, 0.0000752
	White	Women	39	0.0000239, 0.0000328, 0.0000448, 0.0000613, 0.0000841
	White	Women	40	0.0000261, 0.0000358, 0.0000491, 0.0000673, 0.0000926
	White	Women	41	0.0000286, 0.0000392, 0.0000534, 0.0000728, 0.0000995
	White	Women	42	0.0000318, 0.0000429, 0.0000578, 0.0000778, 0.000105
	White	Women	43	0.0000351, 0.0000469, 0.0000626, 0.0000835, 0.000112
	White	Women	44	0.0000384, 0.0000513, 0.0000682, 0.0000906, 0.000121
	White	Women	45	0.0000424, 0.0000564, 0.0000748, 0.0000992, 0.000132
	White	Women	46	0.0000481, 0.0000632, 0.0000828, 0.000108, 0.000143
	White	Women	47	0.0000563, 0.0000722, 0.0000925, 0.000119, 0.000152
	White	Women	48	0.0000671, 0.0000838, 0.000104, 0.00013, 0.000163
	White	Women	49	0.0000803, 0.0000979, 0.000119, 0.000145, 0.000177
	White	Women	50	0.000095, 0.000114, 0.000137, 0.000164, 0.000197
	White	Women	51	0.000111, 0.000132, 0.000158, 0.000187, 0.000223
	White	Women	52	0.000129, 0.000153, 0.000181, 0.000215, 0.000256
	White	Women	53	0.000146, 0.000175, 0.000208, 0.000248, 0.000295
	White	Women	54	0.000165, 0.000198, 0.000237, 0.000284, 0.00034
	White	Women	55	0.000184, 0.000222, 0.000268, 0.000322, 0.000388
	White	Women	56	0.000206, 0.000249, 0.000301, 0.000363, 0.000439
	White	Women	57	0.00023, 0.000279, 0.000337, 0.000407, 0.000494
	White	Women	58	0.000256, 0.000311, 0.000376, 0.000456, 0.000553
	White	Women	59	0.000284, 0.000346, 0.000419, 0.000508, 0.000617
	White	Women	60	0.000312, 0.000381, 0.000464, 0.000564, 0.000688
	White	Women	61	0.000338, 0.000415, 0.000508, 0.000622, 0.000763
	White	Women	62	0.000363, 0.000447, 0.00055, 0.000677, 0.000835
	White	Women	63	0.000387, 0.000479, 0.000591, 0.000729, 0.000902
	White	Women	64	0.000412, 0.00051, 0.000631, 0.00078, 0.000967
	White	Women	65	0.000438, 0.000544, 0.000674, 0.000835, 0.00104
	White	Women	66	0.000469, 0.000584, 0.000725, 0.0009, 0.00112
	White	Women	67	0.000507, 0.000632, 0.000786, 0.000978, 0.00122
	White	Women	68	0.000553, 0.000691, 0.000862, 0.00107, 0.00134
	White	Women	69	0.000609, 0.000762, 0.000951, 0.00119, 0.00148
	White	Women	70	0.000675, 0.000844, 0.00105, 0.00132, 0.00165
	White	Women	71	0.000748, 0.000937, 0.00117, 0.00146, 0.00184
	White	Women	72	0.000827, 0.00104, 0.0013, 0.00163, 0.00205
	White	Women	73	0.000913, 0.00115, 0.00145, 0.00182, 0.0023
	White	Women	74	0.00101, 0.00128, 0.00161, 0.00204, 0.00258
	White	Women	75	0.00112, 0.00142, 0.0018, 0.00229, 0.00291
	White	Women	76	0.00125, 0.00159, 0.00202, 0.00258, 0.00329
	White	Women	77	0.00139, 0.00178, 0.00228, 0.00292, 0.00374
	White	Women	78	0.00156, 0.00201, 0.00259, 0.00333, 0.00429
	White	Women	79	0.00176, 0.00228, 0.00295, 0.00382, 0.00495

Variable	Race/ethnicity	Sex	Age	Distribution
	White	Women	80	0.00199, 0.00259, 0.00337, 0.00439, 0.00573
	White	Women	81	0.00225, 0.00295, 0.00386, 0.00505, 0.00663
	White	Women	82	0.00254, 0.00335, 0.00441, 0.0058, 0.00766
	White	Women	83	0.00285, 0.00378, 0.005, 0.00662, 0.00879
	White	Women	84	0.00317, 0.00423, 0.00563, 0.00749, 0.00999
CHD mortality rates for 2037 (0.01, 0.2, 0.5, 0.8. 0.99 percentiles of the empirical distribution produced during forecasting)				
	Black	Men	30	0.0000237, 0.0000367, 0.0000566, 0.0000874, 0.000135
	Black	Men	31	0.0000309, 0.0000461, 0.0000685, 0.000102, 0.000152
	Black	Men	32	0.0000396, 0.0000572, 0.0000822, 0.000118, 0.000171
	Black	Men	33	0.0000498, 0.0000699, 0.0000977, 0.000137, 0.000192
	Black	Men	34	0.0000606, 0.0000834, 0.000114, 0.000157, 0.000216
	Black	Men	35	0.000071, 0.0000968, 0.000132, 0.000179, 0.000244
	Black	Men	36	0.0000806, 0.00011, 0.000149, 0.000202, 0.000275
	Black	Men	37	0.0000897, 0.000122, 0.000166, 0.000225, 0.000307
	Black	Men	38	0.0000986, 0.000134, 0.000182, 0.000247, 0.000337
	Black	Men	39	0.000108, 0.000146, 0.000198, 0.000268, 0.000365
	Black	Men	40	0.000118, 0.000159, 0.000214, 0.000288, 0.000389
	Black	Men	41	0.000131, 0.000174, 0.000231, 0.000306, 0.000407
	Black	Men	42	0.000145, 0.00019, 0.000249, 0.000325, 0.000426
	Black	Men	43	0.00016, 0.000208, 0.00027, 0.000349, 0.000453
	Black	Men	44	0.000175, 0.000227, 0.000295, 0.000382, 0.000497
	Black	Men	45	0.000192, 0.00025, 0.000326, 0.000424, 0.000553
	Black	Men	46	0.000215, 0.00028, 0.000365, 0.000475, 0.000619
	Black	Men	47	0.000244, 0.000318, 0.000413, 0.000537, 0.0007
	Black	Men	48	0.00028, 0.000365, 0.000474, 0.000616, 0.000802
	Black	Men	49	0.000326, 0.000424, 0.000551, 0.000715, 0.00093
	Black	Men	50	0.000384, 0.000498, 0.000644, 0.000833, 0.00108
	Black	Men	51	0.000458, 0.000589, 0.000755, 0.000969, 0.00124
	Black	Men	52	0.000549, 0.000697, 0.000883, 0.00112, 0.00142
	Black	Men	53	0.000646, 0.000814, 0.00102, 0.00129, 0.00162
	Black	Men	54	0.000743, 0.000934, 0.00117, 0.00147, 0.00185
	Black	Men	55	0.00084, 0.00106, 0.00132, 0.00166, 0.00208
	Black	Men	56	0.000944, 0.00118, 0.00147, 0.00184, 0.0023
	Black	Men	57	0.00105, 0.00131, 0.00162, 0.00201, 0.0025
	Black	Men	58	0.00116, 0.00143, 0.00177, 0.00218, 0.0027
	Black	Men	59	0.00126, 0.00155, 0.0019, 0.00234, 0.00289
	Black	Men	60	0.00135, 0.00166, 0.00203, 0.00249, 0.00306
	Black	Men	61	0.00145, 0.00177, 0.00215, 0.00262, 0.0032
	Black	Men	62	0.00153, 0.00186, 0.00225, 0.00272, 0.00329
	Black	Men	63	0.00162, 0.00194, 0.00233, 0.00279, 0.00336
	Black	Men	64	0.00169, 0.00202, 0.0024, 0.00286, 0.00341
	Black	Men	65	0.00176, 0.00209, 0.00248, 0.00294, 0.00349
	Black	Men	66	0.00183, 0.00218, 0.00258, 0.00306, 0.00363

Variable	Race/ethnicity	Sex	Age	Distribution
	Black	Men	67	0.00191, 0.00227, 0.0027, 0.00322, 0.00384
	Black	Men	68	0.00199, 0.00239, 0.00286, 0.00343, 0.00411
	Black	Men	69	0.0021, 0.00253, 0.00305, 0.00367, 0.00442
	Black	Men	70	0.00223, 0.00269, 0.00325, 0.00392, 0.00475
	Black	Men	71	0.00237, 0.00287, 0.00347, 0.00419, 0.00507
	Black	Men	72	0.00252, 0.00305, 0.00369, 0.00447, 0.00541
	Black	Men	73	0.00267, 0.00324, 0.00393, 0.00475, 0.00577
	Black	Men	74	0.00285, 0.00345, 0.00418, 0.00506, 0.00614
	Black	Men	75	0.00305, 0.00369, 0.00447, 0.0054, 0.00655
	Black	Men	76	0.00328, 0.00397, 0.0048, 0.00579, 0.00701
	Black	Men	77	0.00355, 0.00429, 0.00518, 0.00625, 0.00756
	Black	Men	78	0.00383, 0.00464, 0.00562, 0.00679, 0.00823
	Black	Men	79	0.00413, 0.00503, 0.0061, 0.00741, 0.00901
	Black	Men	80	0.00443, 0.00542, 0.00662, 0.00808, 0.00989
	Black	Men	81	0.00473, 0.00583, 0.00716, 0.00881, 0.0109
	Black	Men	82	0.00499, 0.00621, 0.00771, 0.00957, 0.0119
	Black	Men	83	0.00522, 0.00657, 0.00825, 0.0104, 0.013
	Black	Men	84	0.00544, 0.00692, 0.00878, 0.0111, 0.0142
	Black	Women	30	0.0000101, 0.0000156, 0.0000241, 0.0000372, 0.0000577
	Black	Women	31	0.0000131, 0.0000194, 0.0000286, 0.0000423, 0.0000628
	Black	Women	32	0.0000165, 0.0000237, 0.000034, 0.0000486, 0.0000699
	Black	Women	33	0.0000204, 0.0000287, 0.0000401, 0.0000561, 0.0000788
	Black	Women	34	0.0000244, 0.0000339, 0.0000469, 0.0000649, 0.0000901
	Black	Women	35	0.0000281, 0.0000391, 0.0000541, 0.0000749, 0.000104
	Black	Women	36	0.0000316, 0.0000441, 0.0000614, 0.0000855, 0.000119
	Black	Women	37	0.0000351, 0.0000492, 0.0000687, 0.0000961, 0.000135
	Black	Women	38	0.0000386, 0.0000543, 0.0000761, 0.000107, 0.00015
	Black	Women	39	0.0000421, 0.0000594, 0.0000836, 0.000118, 0.000166
	Black	Women	40	0.0000463, 0.0000651, 0.0000912, 0.000128, 0.00018
	Black	Women	41	0.0000518, 0.0000718, 0.0000991, 0.000137, 0.000189
	Black	Women	42	0.0000592, 0.0000799, 0.000108, 0.000145, 0.000195
	Black	Women	43	0.0000679, 0.0000894, 0.000117, 0.000154, 0.000202
	Black	Women	44	0.0000768, 0.0000996, 0.000129, 0.000167, 0.000216
	Black	Women	45	0.0000865, 0.000112, 0.000143, 0.000184, 0.000238
	Black	Women	46	0.0000989, 0.000127, 0.000162, 0.000207, 0.000264
	Black	Women	47	0.000115, 0.000146, 0.000185, 0.000234, 0.000296
	Black	Women	48	0.000135, 0.00017, 0.000214, 0.000269, 0.000338
	Black	Women	49	0.000156, 0.000197, 0.000249, 0.000315, 0.000399
	Black	Women	50	0.00018, 0.000229, 0.000291, 0.00037, 0.00047
	Black	Women	51	0.000213, 0.000269, 0.000338, 0.000426, 0.000537
	Black	Women	52	0.000254, 0.000315, 0.00039, 0.000483, 0.0006
	Black	Women	53	0.000292, 0.000361, 0.000446, 0.00055, 0.000681
	Black	Women	54	0.000326, 0.000407, 0.000506, 0.00063, 0.000786

Variable	Race/ethnicity	Sex	Age	Distribution
	Black	Women	55	0.000366, 0.000458, 0.000572, 0.000713, 0.000893
	Black	Women	56	0.000415, 0.000516, 0.000641, 0.000796, 0.000991
	Black	Women	57	0.000467, 0.000578, 0.000714, 0.000882, 0.00109
	Black	Women	58	0.000515, 0.000638, 0.000788, 0.000974, 0.00121
	Black	Women	59	0.000557, 0.000694, 0.000861, 0.00107, 0.00133
	Black	Women	60	0.000594, 0.000745, 0.000931, 0.00116, 0.00146
	Black	Women	61	0.000627, 0.000791, 0.000996, 0.00125, 0.00158
	Black	Women	62	0.000652, 0.00083, 0.00105, 0.00134, 0.0017
	Black	Women	63	0.000671, 0.000862, 0.0011, 0.00142, 0.00182
	Black	Women	64	0.000686, 0.00089, 0.00115, 0.00149, 0.00194
	Black	Women	65	0.000701, 0.00092, 0.0012, 0.00158, 0.00207
	Black	Women	66	0.000724, 0.000959, 0.00127, 0.00167, 0.00222
	Black	Women	67	0.00076, 0.00101, 0.00135, 0.00179, 0.00239
	Black	Women	68	0.000812, 0.00109, 0.00145, 0.00194, 0.0026
	Black	Women	69	0.000883, 0.00118, 0.00158, 0.00211, 0.00282
	Black	Women	70	0.000969, 0.00129, 0.00172, 0.00229, 0.00306
	Black	Women	71	0.00107, 0.00142, 0.00188, 0.00249, 0.0033
	Black	Women	72	0.00117, 0.00155, 0.00204, 0.00269, 0.00357
	Black	Women	73	0.00127, 0.00168, 0.00221, 0.00292, 0.00386
	Black	Women	74	0.00138, 0.00183, 0.00241, 0.00317, 0.00418
	Black	Women	75	0.0015, 0.00199, 0.00262, 0.00345, 0.00457
	Black	Women	76	0.00163, 0.00216, 0.00287, 0.00379, 0.00504
	Black	Women	77	0.00176, 0.00236, 0.00315, 0.0042, 0.00563
	Black	Women	78	0.00189, 0.00257, 0.00347, 0.00469, 0.00636
	Black	Women	79	0.00203, 0.00279, 0.00383, 0.00525, 0.00723
	Black	Women	80	0.00217, 0.00303, 0.00423, 0.00589, 0.00823
	Black	Women	81	0.00232, 0.00329, 0.00465, 0.00658, 0.00932
	Black	Women	82	0.0025, 0.00358, 0.00511, 0.0073, 0.0105
	Black	Women	83	0.0027, 0.00389, 0.0056, 0.00804, 0.0116
	Black	Women	84	0.00291, 0.00422, 0.00609, 0.0088, 0.0127
	Hispanic	Men	30	0.00000819, 0.0000126, 0.0000194, 0.0000298, 0.000046
	Hispanic	Men	31	0.0000104, 0.0000156, 0.0000232, 0.0000345, 0.0000516
	Hispanic	Men	32	0.0000131, 0.0000191, 0.0000277, 0.0000401, 0.0000583
	Hispanic	Men	33	0.0000164, 0.0000232, 0.0000328, 0.0000463, 0.0000657
	Hispanic	Men	34	0.0000201, 0.0000279, 0.0000385, 0.0000532, 0.0000737
	Hispanic	Men	35	0.0000242, 0.0000329, 0.0000447, 0.0000607, 0.0000828
	Hispanic	Men	36	0.0000282, 0.0000381, 0.0000512, 0.0000689, 0.000093
	Hispanic	Men	37	0.0000322, 0.0000433, 0.000058, 0.0000777, 0.000104
	Hispanic	Men	38	0.0000364, 0.0000488, 0.0000651, 0.0000869, 0.000116
	Hispanic	Men	39	0.0000411, 0.0000547, 0.0000727, 0.0000965, 0.000129
	Hispanic	Men	40	0.0000468, 0.0000616, 0.0000808, 0.000106, 0.00014
	Hispanic	Men	41	0.0000541, 0.0000697, 0.0000897, 0.000115, 0.000149
	Hispanic	Men	42	0.0000632, 0.0000794, 0.0000996, 0.000125, 0.000157

Variable	Race/ethnicity	Sex	Age	Distribution
	Hispanic	Men	43	0.0000735, 0.0000904, 0.000111, 0.000136, 0.000167
	Hispanic	Men	44	0.0000843, 0.000102, 0.000124, 0.00015, 0.000182
	Hispanic	Men	45	0.0000962, 0.000116, 0.00014, 0.000168, 0.000203
	Hispanic	Men	46	0.00011, 0.000132, 0.000159, 0.000191, 0.000229
	Hispanic	Men	47	0.000127, 0.000152, 0.000182, 0.000218, 0.00026
	Hispanic	Men	48	0.00015, 0.000178, 0.000211, 0.00025, 0.000296
	Hispanic	Men	49	0.000178, 0.000209, 0.000246, 0.000289, 0.00034
	Hispanic	Men	50	0.00021, 0.000246, 0.000289, 0.000339, 0.000398
	Hispanic	Men	51	0.000247, 0.00029, 0.00034, 0.000399, 0.000469
	Hispanic	Men	52	0.000288, 0.000339, 0.000399, 0.000469, 0.000553
	Hispanic	Men	53	0.00033, 0.000391, 0.000464, 0.000551, 0.000654
	Hispanic	Men	54	0.000371, 0.000446, 0.000534, 0.000641, 0.000769
	Hispanic	Men	55	0.000418, 0.000505, 0.000608, 0.000731, 0.000882
	Hispanic	Men	56	0.000472, 0.000568, 0.000683, 0.00082, 0.000988
	Hispanic	Men	57	0.000528, 0.000634, 0.000759, 0.000909, 0.00109
	Hispanic	Men	58	0.000584, 0.000699, 0.000836, 0.000999, 0.0012
	Hispanic	Men	59	0.000637, 0.000763, 0.000913, 0.00109, 0.00131
	Hispanic	Men	60	0.000687, 0.000824, 0.000987, 0.00118, 0.00142
	Hispanic	Men	61	0.000733, 0.000881, 0.00106, 0.00127, 0.00152
	Hispanic	Men	62	0.000775, 0.000933, 0.00112, 0.00135, 0.00162
	Hispanic	Men	63	0.000815, 0.000981, 0.00118, 0.00142, 0.0017
	Hispanic	Men	64	0.000855, 0.00103, 0.00124, 0.00148, 0.00179
	Hispanic	Men	65	0.000897, 0.00108, 0.0013, 0.00156, 0.00187
	Hispanic	Men	66	0.000942, 0.00114, 0.00137, 0.00164, 0.00198
	Hispanic	Men	67	0.00099, 0.0012, 0.00145, 0.00176, 0.00213
	Hispanic	Men	68	0.00105, 0.00128, 0.00156, 0.0019, 0.00232
	Hispanic	Men	69	0.00111, 0.00137, 0.00168, 0.00207, 0.00255
	Hispanic	Men	70	0.00119, 0.00148, 0.00183, 0.00226, 0.00281
	Hispanic	Men	71	0.00128, 0.0016, 0.002, 0.00249, 0.0031
	Hispanic	Men	72	0.00139, 0.00174, 0.00218, 0.00273, 0.00342
	Hispanic	Men	73	0.0015, 0.00189, 0.00237, 0.00298, 0.00375
	Hispanic	Men	74	0.00164, 0.00206, 0.00259, 0.00326, 0.0041
	Hispanic	Men	75	0.0018, 0.00226, 0.00284, 0.00356, 0.00448
	Hispanic	Men	76	0.00199, 0.00249, 0.00312, 0.00391, 0.00491
	Hispanic	Men	77	0.00221, 0.00277, 0.00346, 0.00432, 0.00541
	Hispanic	Men	78	0.00246, 0.00308, 0.00385, 0.0048, 0.006
	Hispanic	Men	79	0.00274, 0.00343, 0.00429, 0.00535, 0.0067
	Hispanic	Men	80	0.00303, 0.0038, 0.00477, 0.00598, 0.00751
	Hispanic	Men	81	0.00329, 0.00417, 0.00527, 0.00667, 0.00846
	Hispanic	Men	82	0.00352, 0.00452, 0.00579, 0.00741, 0.00951
	Hispanic	Men	83	0.00373, 0.00485, 0.0063, 0.00817, 0.0106
	Hispanic	Men	84	0.00394, 0.00519, 0.00681, 0.00894, 0.0118
	Hispanic	Women	30	0.00000223, 0.00000354, 0.00000557, 0.00000877, 0.0000139

Variable	Race/ethnicity	Sex	Age	Distribution
	Hispanic	Women	31	0.00000279, 0.00000427, 0.0000065, 0.0000099, 0.0000151
	Hispanic	Women	32	0.00000345, 0.00000513, 0.00000759, 0.0000112, 0.0000167
	Hispanic	Women	33	0.00000421, 0.0000061, 0.00000883, 0.0000128, 0.0000185
	Hispanic	Women	34	0.00000499, 0.00000714, 0.0000102, 0.0000145, 0.0000208
	Hispanic	Women	35	0.00000575, 0.00000819, 0.0000116, 0.0000165, 0.0000235
	Hispanic	Women	36	0.00000647, 0.00000923, 0.0000131, 0.0000186, 0.0000266
	Hispanic	Women	37	0.00000719, 0.0000103, 0.0000146, 0.0000208, 0.0000297
	Hispanic	Women	38	0.00000793, 0.0000113, 0.0000162, 0.0000231, 0.000033
	Hispanic	Women	39	0.00000876, 0.0000125, 0.0000178, 0.0000253, 0.0000361
	Hispanic	Women	40	0.0000098, 0.0000139, 0.0000195, 0.0000275, 0.0000389
	Hispanic	Women	41	0.0000112, 0.0000155, 0.0000214, 0.0000296, 0.000041
	Hispanic	Women	42	0.0000131, 0.0000176, 0.0000236, 0.0000316, 0.0000425
	Hispanic	Women	43	0.0000154, 0.0000201, 0.0000262, 0.0000341, 0.0000445
	Hispanic	Women	44	0.0000179, 0.000023, 0.0000293, 0.0000375, 0.0000479
	Hispanic	Women	45	0.000021, 0.0000265, 0.0000332, 0.0000418, 0.0000526
	Hispanic	Women	46	0.000025, 0.0000309, 0.0000381, 0.0000469, 0.000058
	Hispanic	Women	47	0.0000301, 0.0000365, 0.0000441, 0.0000533, 0.0000645
	Hispanic	Women	48	0.0000362, 0.0000432, 0.0000514, 0.0000613, 0.0000732
	Hispanic	Women	49	0.0000428, 0.0000509, 0.0000606, 0.000072, 0.0000857
	Hispanic	Women	50	0.0000499, 0.0000599, 0.0000717, 0.000086, 0.000103
	Hispanic	Women	51	0.0000579, 0.0000703, 0.0000853, 0.000103, 0.000126
	Hispanic	Women	52	0.000067, 0.0000825, 0.000101, 0.000125, 0.000154
	Hispanic	Women	53	0.0000772, 0.0000965, 0.00012, 0.00015, 0.000187
	Hispanic	Women	54	0.0000888, 0.000112, 0.000142, 0.000179, 0.000226
	Hispanic	Women	55	0.000102, 0.000131, 0.000166, 0.000211, 0.00027
	Hispanic	Women	56	0.000118, 0.000151, 0.000193, 0.000247, 0.000317
	Hispanic	Women	57	0.000135, 0.000174, 0.000224, 0.000288, 0.000371
	Hispanic	Women	58	0.000152, 0.000198, 0.000257, 0.000333, 0.000433
	Hispanic	Women	59	0.00017, 0.000224, 0.000293, 0.000383, 0.000502
	Hispanic	Women	60	0.000189, 0.00025, 0.00033, 0.000436, 0.000578
	Hispanic	Women	61	0.000207, 0.000277, 0.000368, 0.000491, 0.000656
	Hispanic	Women	62	0.000226, 0.000303, 0.000406, 0.000544, 0.000731
	Hispanic	Women	63	0.000245, 0.00033, 0.000443, 0.000595, 0.000801
	Hispanic	Women	64	0.000266, 0.000358, 0.00048, 0.000643, 0.000863
	Hispanic	Women	65	0.000291, 0.000389, 0.000519, 0.000692, 0.000925
	Hispanic	Women	66	0.000319, 0.000425, 0.000564, 0.000749, 0.000996
	Hispanic	Women	67	0.000352, 0.000467, 0.000618, 0.000819, 0.00109
	Hispanic	Women	68	0.00039, 0.000517, 0.000684, 0.000905, 0.0012
	Hispanic	Women	69	0.000434, 0.000576, 0.000762, 0.00101, 0.00134
	Hispanic	Women	70	0.000485, 0.000643, 0.000851, 0.00113, 0.00149
	Hispanic	Women	71	0.000544, 0.00072, 0.000951, 0.00126, 0.00167
	Hispanic	Women	72	0.000609, 0.000806, 0.00106, 0.00141, 0.00186
	Hispanic	Women	73	0.000681, 0.000902, 0.00119, 0.00157, 0.00208

Variable	Race/ethnicity	Sex	Age	Distribution
	Hispanic	Women	74	0.000762, 0.00101, 0.00134, 0.00177, 0.00235
	Hispanic	Women	75	0.000853, 0.00114, 0.00151, 0.002, 0.00266
	Hispanic	Women	76	0.000957, 0.00128, 0.0017, 0.00227, 0.00303
	Hispanic	Women	77	0.00108, 0.00145, 0.00194, 0.0026, 0.00349
	Hispanic	Women	78	0.00121, 0.00164, 0.00221, 0.00298, 0.00404
	Hispanic	Women	79	0.00136, 0.00186, 0.00253, 0.00344, 0.00469
	Hispanic	Women	80	0.00153, 0.00211, 0.00289, 0.00396, 0.00544
	Hispanic	Women	81	0.00172, 0.00238, 0.00329, 0.00455, 0.00632
	Hispanic	Women	82	0.0019, 0.00267, 0.00374, 0.00523, 0.00733
	Hispanic	Women	83	0.00209, 0.00297, 0.00421, 0.00597, 0.00849
	Hispanic	Women	84	0.00227, 0.00327, 0.0047, 0.00675, 0.00974
	White	Men	30	0.0000144, 0.0000217, 0.0000325, 0.0000487, 0.0000734
	White	Men	31	0.000019, 0.0000277, 0.0000402, 0.0000583, 0.0000848
	White	Men	32	0.0000246, 0.0000349, 0.0000493, 0.0000696, 0.0000987
	White	Men	33	0.000031, 0.0000431, 0.0000598, 0.0000829, 0.000115
	White	Men	34	0.0000377, 0.000052, 0.0000714, 0.0000981, 0.000135
	White	Men	35	0.0000443, 0.0000611, 0.0000838, 0.000115, 0.000158
	White	Men	36	0.000051, 0.0000704, 0.0000968, 0.000133, 0.000183
	White	Men	37	0.0000583, 0.0000803, 0.00011, 0.000151, 0.000208
	White	Men	38	0.0000666, 0.0000912, 0.000124, 0.00017, 0.000232
	White	Men	39	0.0000764, 0.000103, 0.000139, 0.000187, 0.000253
	White	Men	40	0.0000882, 0.000117, 0.000155, 0.000204, 0.000271
	White	Men	41	0.000102, 0.000132, 0.000171, 0.00022, 0.000284
	White	Men	42	0.000119, 0.00015, 0.000187, 0.000234, 0.000294
	White	Men	43	0.000138, 0.000168, 0.000206, 0.000252, 0.000308
	White	Men	44	0.000156, 0.000188, 0.000227, 0.000274, 0.000331
	White	Men	45	0.000177, 0.000211, 0.000252, 0.0003, 0.000359
	White	Men	46	0.000203, 0.00024, 0.000282, 0.000331, 0.00039
	White	Men	47	0.000236, 0.000274, 0.000318, 0.000369, 0.000429
	White	Men	48	0.000274, 0.000315, 0.000363, 0.000417, 0.00048
	White	Men	49	0.000317, 0.000363, 0.000416, 0.000476, 0.000546
	White	Men	50	0.000369, 0.000421, 0.000479, 0.000546, 0.000622
	White	Men	51	0.000432, 0.000488, 0.000551, 0.000622, 0.000703
	White	Men	52	0.000502, 0.000563, 0.000632, 0.000708, 0.000794
	White	Men	53	0.000567, 0.000638, 0.000718, 0.000807, 0.000909
	White	Men	54	0.000624, 0.000711, 0.000808, 0.000918, 0.00104
	White	Men	55	0.000682, 0.000783, 0.000899, 0.00103, 0.00119
	White	Men	56	0.00074, 0.000856, 0.00099, 0.00114, 0.00132
	White	Men	57	0.000795, 0.000927, 0.00108, 0.00126, 0.00146
	White	Men	58	0.000848, 0.000996, 0.00117, 0.00137, 0.00161
	White	Men	59	0.000902, 0.00107, 0.00126, 0.00148, 0.00175
	White	Men	60	0.000958, 0.00113, 0.00134, 0.00159, 0.00188
	White	Men	61	0.00101, 0.0012, 0.00142, 0.00168, 0.002

Variable	Race/ethnicity	Sex	Age	Distribution
	White	Men	62	0.00106, 0.00126, 0.00149, 0.00177, 0.0021
	White	Men	63	0.00111, 0.00131, 0.00156, 0.00185, 0.0022
	White	Men	64	0.00115, 0.00136, 0.00162, 0.00192, 0.00228
	White	Men	65	0.00118, 0.00141, 0.00168, 0.00199, 0.00237
	White	Men	66	0.00122, 0.00146, 0.00175, 0.00209, 0.0025
	White	Men	67	0.00126, 0.00152, 0.00184, 0.00222, 0.00268
	White	Men	68	0.00131, 0.0016, 0.00195, 0.00238, 0.00291
	White	Men	69	0.00138, 0.0017, 0.0021, 0.00258, 0.00318
	White	Men	70	0.00148, 0.00183, 0.00227, 0.0028, 0.00347
	White	Men	71	0.0016, 0.00199, 0.00246, 0.00305, 0.00378
	White	Men	72	0.00174, 0.00216, 0.00268, 0.00331, 0.00411
	White	Men	73	0.00189, 0.00235, 0.00291, 0.0036, 0.00447
	White	Men	74	0.00205, 0.00255, 0.00316, 0.00392, 0.00487
	White	Men	75	0.00224, 0.00278, 0.00345, 0.00428, 0.00531
	White	Men	76	0.00244, 0.00304, 0.00378, 0.00469, 0.00584
	White	Men	77	0.00267, 0.00334, 0.00416, 0.00519, 0.00649
	White	Men	78	0.00294, 0.00369, 0.00463, 0.0058, 0.00728
	White	Men	79	0.00326, 0.00411, 0.00517, 0.00651, 0.0082
	White	Men	80	0.00364, 0.0046, 0.00581, 0.00732, 0.00926
	White	Men	81	0.00408, 0.00516, 0.00652, 0.00824, 0.0104
	White	Men	82	0.00456, 0.00578, 0.0073, 0.00923, 0.0117
	White	Men	83	0.00506, 0.00642, 0.00812, 0.0103, 0.013
	White	Men	84	0.00557, 0.00707, 0.00895, 0.0113, 0.0144
	White	Women	30	0.00000394, 0.00000646, 0.0000105, 0.0000172, 0.0000282
	White	Women	31	0.00000528, 0.0000083, 0.000013, 0.0000203, 0.0000319
	White	Women	32	0.00000704, 0.0000106, 0.0000159, 0.0000238, 0.0000358
	White	Women	33	0.00000921, 0.0000133, 0.0000192, 0.0000278, 0.0000402
	White	Women	34	0.0000116, 0.0000164, 0.000023, 0.0000323, 0.0000456
	White	Women	35	0.0000142, 0.0000196, 0.0000271, 0.0000375, 0.0000519
	White	Women	36	0.0000167, 0.0000229, 0.0000314, 0.000043, 0.0000591
	White	Women	37	0.0000191, 0.0000261, 0.0000357, 0.0000488, 0.0000669
	White	Women	38	0.0000213, 0.0000293, 0.0000401, 0.0000548, 0.0000753
	White	Women	39	0.0000234, 0.0000322, 0.0000443, 0.0000609, 0.000084
	White	Women	40	0.0000255, 0.0000352, 0.0000485, 0.0000667, 0.0000922
	White	Women	41	0.000028, 0.0000384, 0.0000525, 0.0000719, 0.0000987
	White	Women	42	0.000031, 0.000042, 0.0000567, 0.0000766, 0.000104
	White	Women	43	0.0000343, 0.0000459, 0.0000614, 0.000082, 0.00011
	White	Women	44	0.0000375, 0.0000501, 0.0000667, 0.0000889, 0.000119
	White	Women	45	0.0000413, 0.000055, 0.0000731, 0.0000971, 0.000129
	White	Women	46	0.0000468, 0.0000616, 0.0000809, 0.000106, 0.00014
	White	Women	47	0.0000548, 0.0000705, 0.0000904, 0.000116, 0.000149
	White	Women	48	0.0000654, 0.0000818, 0.000102, 0.000127, 0.000159
	White	Women	49	0.0000783, 0.0000955, 0.000116, 0.000142, 0.000173

Variable	Race/ethnicity	Sex	Age	Distribution
	White	Women	50	0.0000927, 0.000112, 0.000134, 0.000161, 0.000193
	White	Women	51	0.000109, 0.00013, 0.000154, 0.000184, 0.000219
	White	Women	52	0.000126, 0.00015, 0.000178, 0.000212, 0.000252
	White	Women	53	0.000144, 0.000171, 0.000204, 0.000244, 0.000291
	White	Women	54	0.000161, 0.000194, 0.000233, 0.000279, 0.000336
	White	Women	55	0.000181, 0.000218, 0.000263, 0.000318, 0.000384
	White	Women	56	0.000202, 0.000245, 0.000296, 0.000358, 0.000434
	White	Women	57	0.000226, 0.000274, 0.000332, 0.000402, 0.000488
	White	Women	58	0.000251, 0.000306, 0.000371, 0.00045, 0.000547
	White	Women	59	0.000279, 0.00034, 0.000413, 0.000502, 0.000611
	White	Women	60	0.000306, 0.000374, 0.000456, 0.000557, 0.000681
	White	Women	61	0.000331, 0.000407, 0.0005, 0.000613, 0.000754
	White	Women	62	0.000355, 0.000439, 0.000541, 0.000667, 0.000824
	White	Women	63	0.000378, 0.000469, 0.00058, 0.000717, 0.000889
	White	Women	64	0.000402, 0.000499, 0.000618, 0.000766, 0.000951
	White	Women	65	0.000427, 0.000532, 0.00066, 0.000819, 0.00102
	White	Women	66	0.000457, 0.00057, 0.000709, 0.000882, 0.0011
	White	Women	67	0.000494, 0.000617, 0.000769, 0.000958, 0.0012
	White	Women	68	0.000539, 0.000674, 0.000842, 0.00105, 0.00132
	White	Women	69	0.000593, 0.000743, 0.000929, 0.00116, 0.00146
	White	Women	70	0.000656, 0.000823, 0.00103, 0.00129, 0.00162
	White	Women	71	0.000727, 0.000913, 0.00114, 0.00143, 0.0018
	White	Women	72	0.000804, 0.00101, 0.00127, 0.0016, 0.00201
	White	Women	73	0.000887, 0.00112, 0.00141, 0.00178, 0.00225
	White	Women	74	0.00098, 0.00124, 0.00157, 0.00199, 0.00253
	White	Women	75	0.00109, 0.00138, 0.00176, 0.00223, 0.00284
	White	Women	76	0.00121, 0.00155, 0.00197, 0.00252, 0.00322
	White	Women	77	0.00135, 0.00174, 0.00222, 0.00285, 0.00366
	White	Women	78	0.00152, 0.00196, 0.00252, 0.00325, 0.0042
	White	Women	79	0.00171, 0.00222, 0.00288, 0.00373, 0.00484
	White	Women	80	0.00193, 0.00252, 0.00329, 0.00429, 0.00561
	White	Women	81	0.00219, 0.00287, 0.00377, 0.00494, 0.00649
	White	Women	82	0.00246, 0.00326, 0.0043, 0.00567, 0.0075
	White	Women	83	0.00276, 0.00368, 0.00488, 0.00647, 0.00861
	White	Women	84	0.00308, 0.00411, 0.00549, 0.00732, 0.00978
Stroke mortality rates for 2014 (0.01, 0.2, 0.5, 0.8. 0.99 percentiles of the empirical distribution produced during forecasting)				
	Black	Men	30	0.0000365, 0.0000365, 0.0000365, 0.0000365, 0.0000365
	Black	Men	31	0.0000417, 0.0000417, 0.0000417, 0.0000417, 0.0000417
	Black	Men	32	0.0000476, 0.0000476, 0.0000476, 0.0000476, 0.0000476
	Black	Men	33	0.0000542, 0.0000542, 0.0000542, 0.0000542, 0.0000542
	Black	Men	34	0.0000614, 0.0000614, 0.0000614, 0.0000614, 0.0000614
	Black	Men	35	0.0000692, 0.0000692, 0.0000692, 0.0000692, 0.0000692
	Black	Men	36	0.0000776, 0.0000776, 0.0000776, 0.0000776, 0.0000776

Variable	Race/ethnicity	Sex	Age	Distribution
	Black	Men	37	0.0000866, 0.0000866, 0.0000866, 0.0000866, 0.0000866
	Black	Men	38	0.0000962, 0.0000962, 0.0000962, 0.0000962, 0.0000962
	Black	Men	39	0.000107, 0.000107, 0.000107, 0.000107, 0.000107
	Black	Men	40	0.000118, 0.000118, 0.000118, 0.000118, 0.000118
	Black	Men	41	0.00013, 0.00013, 0.00013, 0.00013, 0.00013
	Black	Men	42	0.000144, 0.000144, 0.000144, 0.000144, 0.000144
	Black	Men	43	0.000159, 0.000159, 0.000159, 0.000159, 0.000159
	Black	Men	44	0.000175, 0.000175, 0.000175, 0.000175, 0.000175
	Black	Men	45	0.000192, 0.000192, 0.000192, 0.000192, 0.000192
	Black	Men	46	0.000212, 0.000212, 0.000212, 0.000212, 0.000212
	Black	Men	47	0.000235, 0.000235, 0.000235, 0.000235, 0.000235
	Black	Men	48	0.00026, 0.00026, 0.00026, 0.00026, 0.00026
	Black	Men	49	0.000289, 0.000289, 0.000289, 0.000289, 0.000289
	Black	Men	50	0.000321, 0.000321, 0.000321, 0.000321, 0.000321
	Black	Men	51	0.000358, 0.000358, 0.000358, 0.000358, 0.000358
	Black	Men	52	0.000398, 0.000398, 0.000398, 0.000398, 0.000398
	Black	Men	53	0.000443, 0.000443, 0.000443, 0.000443, 0.000443
	Black	Men	54	0.000491, 0.000491, 0.000491, 0.000491, 0.000491
	Black	Men	55	0.000543, 0.000543, 0.000543, 0.000543, 0.000543
	Black	Men	56	0.000599, 0.000599, 0.000599, 0.000599, 0.000599
	Black	Men	57	0.000658, 0.000658, 0.000658, 0.000658, 0.000658
	Black	Men	58	0.00072, 0.00072, 0.00072, 0.00072, 0.00072
	Black	Men	59	0.000784, 0.000784, 0.000784, 0.000784, 0.000784
	Black	Men	60	0.000851, 0.000851, 0.000851, 0.000851, 0.000851
	Black	Men	61	0.000921, 0.000921, 0.000921, 0.000921, 0.000921
	Black	Men	62	0.000995, 0.000995, 0.000995, 0.000995, 0.000995
	Black	Men	63	0.00107, 0.00107, 0.00107, 0.00107, 0.00107
	Black	Men	64	0.00116, 0.00116, 0.00116, 0.00116, 0.00116
	Black	Men	65	0.00125, 0.00125, 0.00125, 0.00125, 0.00125
	Black	Men	66	0.00135, 0.00135, 0.00135, 0.00135, 0.00135
	Black	Men	67	0.00146, 0.00146, 0.00146, 0.00146, 0.00146
	Black	Men	68	0.00159, 0.00159, 0.00159, 0.00159, 0.00159
	Black	Men	69	0.00173, 0.00173, 0.00173, 0.00173, 0.00173
	Black	Men	70	0.00188, 0.00188, 0.00188, 0.00188, 0.00188
	Black	Men	71	0.00206, 0.00206, 0.00206, 0.00206, 0.00206
	Black	Men	72	0.00225, 0.00225, 0.00225, 0.00225, 0.00225
	Black	Men	73	0.00246, 0.00246, 0.00246, 0.00246, 0.00246
	Black	Men	74	0.00269, 0.00269, 0.00269, 0.00269, 0.00269
	Black	Men	75	0.00293, 0.00293, 0.00293, 0.00293, 0.00293
	Black	Men	76	0.00319, 0.00319, 0.00319, 0.00319, 0.00319
	Black	Men	77	0.00346, 0.00346, 0.00346, 0.00346, 0.00346
	Black	Men	78	0.00375, 0.00375, 0.00375, 0.00375, 0.00375
	Black	Men	79	0.00406, 0.00406, 0.00406, 0.00406, 0.00406

Variable	Race/ethnicity	Sex	Age	Distribution
	Black	Men	80	0.00437, 0.00437, 0.00437, 0.00437, 0.00437
	Black	Men	81	0.0047, 0.0047, 0.0047, 0.0047, 0.0047
	Black	Men	82	0.00504, 0.00504, 0.00504, 0.00504, 0.00504
	Black	Men	83	0.00538, 0.00538, 0.00538, 0.00538, 0.00538
	Black	Men	84	0.0057, 0.0057, 0.0057, 0.0057, 0.0057
	Black	Women	30	0.0000214, 0.0000214, 0.0000214, 0.0000214, 0.0000214
	Black	Women	31	0.0000232, 0.0000232, 0.0000232, 0.0000232, 0.0000232
	Black	Women	32	0.0000253, 0.0000253, 0.0000253, 0.0000253, 0.0000253
	Black	Women	33	0.000028, 0.000028, 0.000028, 0.000028, 0.000028
	Black	Women	34	0.0000314, 0.0000314, 0.0000314, 0.0000314, 0.0000314
	Black	Women	35	0.0000357, 0.0000357, 0.0000357, 0.0000357, 0.0000357
	Black	Women	36	0.000041, 0.000041, 0.000041, 0.000041, 0.000041
	Black	Women	37	0.0000474, 0.0000474, 0.0000474, 0.0000474, 0.0000474
	Black	Women	38	0.0000549, 0.0000549, 0.0000549, 0.0000549, 0.0000549
	Black	Women	39	0.0000636, 0.0000636, 0.0000636, 0.0000636, 0.0000636
	Black	Women	40	0.0000736, 0.0000736, 0.0000736, 0.0000736, 0.0000736
	Black	Women	41	0.0000849, 0.0000849, 0.0000849, 0.0000849, 0.0000849
	Black	Women	42	0.0000972, 0.0000972, 0.0000972, 0.0000972, 0.0000972
	Black	Women	43	0.00011, 0.00011, 0.00011, 0.00011, 0.00011
	Black	Women	44	0.000124, 0.000124, 0.000124, 0.000124, 0.000124
	Black	Women	45	0.000138, 0.000138, 0.000138, 0.000138, 0.000138
	Black	Women	46	0.000154, 0.000154, 0.000154, 0.000154, 0.000154
	Black	Women	47	0.000171, 0.000171, 0.000171, 0.000171, 0.000171
	Black	Women	48	0.000191, 0.000191, 0.000191, 0.000191, 0.000191
	Black	Women	49	0.000215, 0.000215, 0.000215, 0.000215, 0.000215
	Black	Women	50	0.00024, 0.00024, 0.00024, 0.00024, 0.00024
	Black	Women	51	0.000266, 0.000266, 0.000266, 0.000266, 0.000266
	Black	Women	52	0.000291, 0.000291, 0.000291, 0.000291, 0.000291
	Black	Women	53	0.000314, 0.000314, 0.000314, 0.000314, 0.000314
	Black	Women	54	0.000334, 0.000334, 0.000334, 0.000334, 0.000334
	Black	Women	55	0.000353, 0.000353, 0.000353, 0.000353, 0.000353
	Black	Women	56	0.000374, 0.000374, 0.000374, 0.000374, 0.000374
	Black	Women	57	0.0004, 0.0004, 0.0004, 0.0004, 0.0004
	Black	Women	58	0.000433, 0.000433, 0.000433, 0.000433, 0.000433
	Black	Women	59	0.000472, 0.000472, 0.000472, 0.000472, 0.000472
	Black	Women	60	0.000518, 0.000518, 0.000518, 0.000518, 0.000518
	Black	Women	61	0.000568, 0.000568, 0.000568, 0.000568, 0.000568
	Black	Women	62	0.00062, 0.00062, 0.00062, 0.00062, 0.00062
	Black	Women	63	0.000673, 0.000673, 0.000673, 0.000673, 0.000673
	Black	Women	64	0.000726, 0.000726, 0.000726, 0.000726, 0.000726
	Black	Women	65	0.000782, 0.000782, 0.000782, 0.000782, 0.000782
	Black	Women	66	0.000844, 0.000844, 0.000844, 0.000844, 0.000844
	Black	Women	67	0.000916, 0.000916, 0.000916, 0.000916, 0.000916

Variable	Race/ethnicity	Sex	Age	Distribution
	Black	Women	68	0.001, 0.001, 0.001, 0.001, 0.001
	Black	Women	69	0.00111, 0.00111, 0.00111, 0.00111, 0.00111
	Black	Women	70	0.00123, 0.00123, 0.00123, 0.00123, 0.00123
	Black	Women	71	0.00138, 0.00138, 0.00138, 0.00138, 0.00138
	Black	Women	72	0.00154, 0.00154, 0.00154, 0.00154, 0.00154
	Black	Women	73	0.00172, 0.00172, 0.00172, 0.00172, 0.00172
	Black	Women	74	0.0019, 0.0019, 0.0019, 0.0019, 0.0019
	Black	Women	75	0.00209, 0.00209, 0.00209, 0.00209, 0.00209
	Black	Women	76	0.0023, 0.0023, 0.0023, 0.0023, 0.0023
	Black	Women	77	0.00253, 0.00253, 0.00253, 0.00253, 0.00253
	Black	Women	78	0.00279, 0.00279, 0.00279, 0.00279, 0.00279
	Black	Women	79	0.0031, 0.0031, 0.0031, 0.0031, 0.0031
	Black	Women	80	0.00347, 0.00347, 0.00347, 0.00347, 0.00347
	Black	Women	81	0.0039, 0.0039, 0.0039, 0.0039, 0.0039
	Black	Women	82	0.00438, 0.00438, 0.00438, 0.00438, 0.00438
	Black	Women	83	0.00489, 0.00489, 0.00489, 0.00489, 0.00489
	Black	Women	84	0.00541, 0.00541, 0.00541, 0.00541, 0.00541
	Hispanic	Men	30	0.0000155, 0.0000155, 0.0000155, 0.0000155, 0.0000155
	Hispanic	Men	31	0.0000173, 0.0000173, 0.0000173, 0.0000173, 0.0000173
	Hispanic	Men	32	0.0000195, 0.0000195, 0.0000195, 0.0000195, 0.0000195
	Hispanic	Men	33	0.0000219, 0.0000219, 0.0000219, 0.0000219, 0.0000219
	Hispanic	Men	34	0.0000248, 0.0000248, 0.0000248, 0.0000248, 0.0000248
	Hispanic	Men	35	0.000028, 0.000028, 0.000028, 0.000028, 0.000028
	Hispanic	Men	36	0.0000316, 0.0000316, 0.0000316, 0.0000316, 0.0000316
	Hispanic	Men	37	0.0000355, 0.0000355, 0.0000355, 0.0000355, 0.0000355
	Hispanic	Men	38	0.0000397, 0.0000397, 0.0000397, 0.0000397, 0.0000397
	Hispanic	Men	39	0.000044, 0.000044, 0.000044, 0.000044, 0.000044
	Hispanic	Men	40	0.0000486, 0.0000486, 0.0000486, 0.0000486, 0.0000486
	Hispanic	Men	41	0.0000535, 0.0000535, 0.0000535, 0.0000535, 0.0000535
	Hispanic	Men	42	0.0000587, 0.0000587, 0.0000587, 0.0000587, 0.0000587
	Hispanic	Men	43	0.0000645, 0.0000645, 0.0000645, 0.0000645, 0.0000645
	Hispanic	Men	44	0.000071, 0.000071, 0.000071, 0.000071, 0.000071
	Hispanic	Men	45	0.0000784, 0.0000784, 0.0000784, 0.0000784, 0.0000784
	Hispanic	Men	46	0.000087, 0.000087, 0.000087, 0.000087, 0.000087
	Hispanic	Men	47	0.0000968, 0.0000968, 0.0000968, 0.0000968, 0.0000968
	Hispanic	Men	48	0.000108, 0.000108, 0.000108, 0.000108, 0.000108
	Hispanic	Men	49	0.000121, 0.000121, 0.000121, 0.000121, 0.000121
	Hispanic	Men	50	0.000135, 0.000135, 0.000135, 0.000135, 0.000135
	Hispanic	Men	51	0.000151, 0.000151, 0.000151, 0.000151, 0.000151
	Hispanic	Men	52	0.000168, 0.000168, 0.000168, 0.000168, 0.000168
	Hispanic	Men	53	0.000187, 0.000187, 0.000187, 0.000187, 0.000187
	Hispanic	Men	54	0.000206, 0.000206, 0.000206, 0.000206, 0.000206
	Hispanic	Men	55	0.000227, 0.000227, 0.000227, 0.000227, 0.000227

Variable	Race/ethnicity	Sex	Age	Distribution
	Hispanic	Men	56	0.000249, 0.000249, 0.000249, 0.000249, 0.000249
	Hispanic	Men	57	0.000272, 0.000272, 0.000272, 0.000272, 0.000272
	Hispanic	Men	58	0.000296, 0.000296, 0.000296, 0.000296, 0.000296
	Hispanic	Men	59	0.000322, 0.000322, 0.000322, 0.000322, 0.000322
	Hispanic	Men	60	0.000348, 0.000348, 0.000348, 0.000348, 0.000348
	Hispanic	Men	61	0.000376, 0.000376, 0.000376, 0.000376, 0.000376
	Hispanic	Men	62	0.000406, 0.000406, 0.000406, 0.000406, 0.000406
	Hispanic	Men	63	0.000438, 0.000438, 0.000438, 0.000438, 0.000438
	Hispanic	Men	64	0.000474, 0.000474, 0.000474, 0.000474, 0.000474
	Hispanic	Men	65	0.000515, 0.000515, 0.000515, 0.000515, 0.000515
	Hispanic	Men	66	0.000563, 0.000563, 0.000563, 0.000563, 0.000563
	Hispanic	Men	67	0.000619, 0.000619, 0.000619, 0.000619, 0.000619
	Hispanic	Men	68	0.000684, 0.000684, 0.000684, 0.000684, 0.000684
	Hispanic	Men	69	0.000758, 0.000758, 0.000758, 0.000758, 0.000758
	Hispanic	Men	70	0.000844, 0.000844, 0.000844, 0.000844, 0.000844
	Hispanic	Men	71	0.000942, 0.000942, 0.000942, 0.000942, 0.000942
	Hispanic	Men	72	0.00105, 0.00105, 0.00105, 0.00105, 0.00105
	Hispanic	Men	73	0.00118, 0.00118, 0.00118, 0.00118, 0.00118
	Hispanic	Men	74	0.00132, 0.00132, 0.00132, 0.00132, 0.00132
	Hispanic	Men	75	0.00148, 0.00148, 0.00148, 0.00148, 0.00148
	Hispanic	Men	76	0.00166, 0.00166, 0.00166, 0.00166, 0.00166
	Hispanic	Men	77	0.00186, 0.00186, 0.00186, 0.00186, 0.00186
	Hispanic	Men	78	0.00209, 0.00209, 0.00209, 0.00209, 0.00209
	Hispanic	Men	79	0.00235, 0.00235, 0.00235, 0.00235, 0.00235
	Hispanic	Men	80	0.00264, 0.00264, 0.00264, 0.00264, 0.00264
	Hispanic	Men	81	0.00294, 0.00294, 0.00294, 0.00294, 0.00294
	Hispanic	Men	82	0.00326, 0.00326, 0.00326, 0.00326, 0.00326
	Hispanic	Men	83	0.00359, 0.00359, 0.00359, 0.00359, 0.00359
	Hispanic	Men	84	0.00392, 0.00392, 0.00392, 0.00392, 0.00392
	Hispanic	Women	30	0.0000103, 0.0000103, 0.0000103, 0.0000103, 0.0000103
	Hispanic	Women	31	0.0000108, 0.0000108, 0.0000108, 0.0000108, 0.0000108
	Hispanic	Women	32	0.0000114, 0.0000114, 0.0000114, 0.0000114, 0.0000114
	Hispanic	Women	33	0.0000121, 0.0000121, 0.0000121, 0.0000121, 0.0000121
	Hispanic	Women	34	0.0000132, 0.0000132, 0.0000132, 0.0000132, 0.0000132
	Hispanic	Women	35	0.0000145, 0.0000145, 0.0000145, 0.0000145, 0.0000145
	Hispanic	Women	36	0.0000163, 0.0000163, 0.0000163, 0.0000163, 0.0000163
	Hispanic	Women	37	0.0000185, 0.0000185, 0.0000185, 0.0000185, 0.0000185
	Hispanic	Women	38	0.0000215, 0.0000215, 0.0000215, 0.0000215, 0.0000215
	Hispanic	Women	39	0.0000251, 0.0000251, 0.0000251, 0.0000251, 0.0000251
	Hispanic	Women	40	0.0000296, 0.0000296, 0.0000296, 0.0000296, 0.0000296
	Hispanic	Women	41	0.000035, 0.000035, 0.000035, 0.000035, 0.000035
	Hispanic	Women	42	0.0000411, 0.0000411, 0.0000411, 0.0000411, 0.0000411
	Hispanic	Women	43	0.0000476, 0.0000476, 0.0000476, 0.0000476, 0.0000476

Variable	Race/ethnicity	Sex	Age	Distribution
	Hispanic	Women	44	0.0000542, 0.0000542, 0.0000542, 0.0000542, 0.0000542
	Hispanic	Women	45	0.0000605, 0.0000605, 0.0000605, 0.0000605, 0.0000605
	Hispanic	Women	46	0.0000662, 0.0000662, 0.0000662, 0.0000662, 0.0000662
	Hispanic	Women	47	0.0000714, 0.0000714, 0.0000714, 0.0000714, 0.0000714
	Hispanic	Women	48	0.0000761, 0.0000761, 0.0000761, 0.0000761, 0.0000761
	Hispanic	Women	49	0.0000806, 0.0000806, 0.0000806, 0.0000806, 0.0000806
	Hispanic	Women	50	0.0000854, 0.0000854, 0.0000854, 0.0000854, 0.0000854
	Hispanic	Women	51	0.0000907, 0.0000907, 0.0000907, 0.0000907, 0.0000907
	Hispanic	Women	52	0.0000969, 0.0000969, 0.0000969, 0.0000969, 0.0000969
	Hispanic	Women	53	0.000104, 0.000104, 0.000104, 0.000104, 0.000104
	Hispanic	Women	54	0.000113, 0.000113, 0.000113, 0.000113, 0.000113
	Hispanic	Women	55	0.000123, 0.000123, 0.000123, 0.000123, 0.000123
	Hispanic	Women	56	0.000134, 0.000134, 0.000134, 0.000134, 0.000134
	Hispanic	Women	57	0.000147, 0.000147, 0.000147, 0.000147, 0.000147
	Hispanic	Women	58	0.000161, 0.000161, 0.000161, 0.000161, 0.000161
	Hispanic	Women	59	0.000178, 0.000178, 0.000178, 0.000178, 0.000178
	Hispanic	Women	60	0.000196, 0.000196, 0.000196, 0.000196, 0.000196
	Hispanic	Women	61	0.000217, 0.000217, 0.000217, 0.000217, 0.000217
	Hispanic	Women	62	0.000241, 0.000241, 0.000241, 0.000241, 0.000241
	Hispanic	Women	63	0.000267, 0.000267, 0.000267, 0.000267, 0.000267
	Hispanic	Women	64	0.000295, 0.000295, 0.000295, 0.000295, 0.000295
	Hispanic	Women	65	0.000328, 0.000328, 0.000328, 0.000328, 0.000328
	Hispanic	Women	66	0.000365, 0.000365, 0.000365, 0.000365, 0.000365
	Hispanic	Women	67	0.000408, 0.000408, 0.000408, 0.000408, 0.000408
	Hispanic	Women	68	0.000458, 0.000458, 0.000458, 0.000458, 0.000458
	Hispanic	Women	69	0.000518, 0.000518, 0.000518, 0.000518, 0.000518
	Hispanic	Women	70	0.000589, 0.000589, 0.000589, 0.000589, 0.000589
	Hispanic	Women	71	0.000672, 0.000672, 0.000672, 0.000672, 0.000672
	Hispanic	Women	72	0.000768, 0.000768, 0.000768, 0.000768, 0.000768
	Hispanic	Women	73	0.000878, 0.000878, 0.000878, 0.000878, 0.000878
	Hispanic	Women	74	0.001, 0.001, 0.001, 0.001, 0.001
	Hispanic	Women	75	0.00115, 0.00115, 0.00115, 0.00115, 0.00115
	Hispanic	Women	76	0.00131, 0.00131, 0.00131, 0.00131, 0.00131
	Hispanic	Women	77	0.0015, 0.0015, 0.0015, 0.0015, 0.0015
	Hispanic	Women	78	0.00173, 0.00173, 0.00173, 0.00173, 0.00173
	Hispanic	Women	79	0.00201, 0.00201, 0.00201, 0.00201, 0.00201
	Hispanic	Women	80	0.00233, 0.00233, 0.00233, 0.00233, 0.00233
	Hispanic	Women	81	0.0027, 0.0027, 0.0027, 0.0027, 0.0027
	Hispanic	Women	82	0.00311, 0.00311, 0.00311, 0.00311, 0.00311
	Hispanic	Women	83	0.00355, 0.00355, 0.00355, 0.00355, 0.00355
	Hispanic	Women	84	0.004, 0.004, 0.004, 0.004, 0.004
	White	Men	30	0.0000121, 0.0000121, 0.0000121, 0.0000121, 0.0000121
	White	Men	31	0.0000135, 0.0000135, 0.0000135, 0.0000135, 0.0000135

Variable	Race/ethnicity	Sex	Age	Distribution
	White	Men	32	0.000015, 0.000015, 0.000015, 0.000015, 0.000015
	White	Men	33	0.0000168, 0.0000168, 0.0000168, 0.0000168, 0.0000168
	White	Men	34	0.0000187, 0.0000187, 0.0000187, 0.0000187, 0.0000187
	White	Men	35	0.000021, 0.000021, 0.000021, 0.000021, 0.000021
	White	Men	36	0.0000236, 0.0000236, 0.0000236, 0.0000236, 0.0000236
	White	Men	37	0.0000265, 0.0000265, 0.0000265, 0.0000265, 0.0000265
	White	Men	38	0.0000297, 0.0000297, 0.0000297, 0.0000297, 0.0000297
	White	Men	39	0.0000333, 0.0000333, 0.0000333, 0.0000333, 0.0000333
	White	Men	40	0.0000373, 0.0000373, 0.0000373, 0.0000373, 0.0000373
	White	Men	41	0.0000417, 0.0000417, 0.0000417, 0.0000417, 0.0000417
	White	Men	42	0.0000466, 0.0000466, 0.0000466, 0.0000466, 0.0000466
	White	Men	43	0.000052, 0.000052, 0.000052, 0.000052, 0.000052
	White	Men	44	0.0000582, 0.0000582, 0.0000582, 0.0000582, 0.0000582
	White	Men	45	0.0000651, 0.0000651, 0.0000651, 0.0000651, 0.0000651
	White	Men	46	0.0000729, 0.0000729, 0.0000729, 0.0000729, 0.0000729
	White	Men	47	0.0000814, 0.0000814, 0.0000814, 0.0000814, 0.0000814
	White	Men	48	0.0000909, 0.0000909, 0.0000909, 0.0000909, 0.0000909
	White	Men	49	0.000101, 0.000101, 0.000101, 0.000101, 0.000101
	White	Men	50	0.000113, 0.000113, 0.000113, 0.000113, 0.000113
	White	Men	51	0.000125, 0.000125, 0.000125, 0.000125, 0.000125
	White	Men	52	0.000138, 0.000138, 0.000138, 0.000138, 0.000138
	White	Men	53	0.000153, 0.000153, 0.000153, 0.000153, 0.000153
	White	Men	54	0.000169, 0.000169, 0.000169, 0.000169, 0.000169
	White	Men	55	0.000186, 0.000186, 0.000186, 0.000186, 0.000186
	White	Men	56	0.000204, 0.000204, 0.000204, 0.000204, 0.000204
	White	Men	57	0.000224, 0.000224, 0.000224, 0.000224, 0.000224
	White	Men	58	0.000245, 0.000245, 0.000245, 0.000245, 0.000245
	White	Men	59	0.000268, 0.000268, 0.000268, 0.000268, 0.000268
	White	Men	60	0.000293, 0.000293, 0.000293, 0.000293, 0.000293
	White	Men	61	0.000321, 0.000321, 0.000321, 0.000321, 0.000321
	White	Men	62	0.000352, 0.000352, 0.000352, 0.000352, 0.000352
	White	Men	63	0.000387, 0.000387, 0.000387, 0.000387, 0.000387
	White	Men	64	0.000426, 0.000426, 0.000426, 0.000426, 0.000426
	White	Men	65	0.000471, 0.000471, 0.000471, 0.000471, 0.000471
	White	Men	66	0.000523, 0.000523, 0.000523, 0.000523, 0.000523
	White	Men	67	0.000582, 0.000582, 0.000582, 0.000582, 0.000582
	White	Men	68	0.00065, 0.00065, 0.00065, 0.00065, 0.00065
	White	Men	69	0.000729, 0.000729, 0.000729, 0.000729, 0.000729
	White	Men	70	0.00082, 0.00082, 0.00082, 0.00082, 0.00082
	White	Men	71	0.000925, 0.000925, 0.000925, 0.000925, 0.000925
	White	Men	72	0.00105, 0.00105, 0.00105, 0.00105, 0.00105
	White	Men	73	0.00119, 0.00119, 0.00119, 0.00119, 0.00119
	White	Men	74	0.00135, 0.00135, 0.00135, 0.00135, 0.00135

Variable	Race/ethnicity	Sex	Age	Distribution
	White	Men	75	0.00154, 0.00154, 0.00154, 0.00154, 0.00154
	White	Men	76	0.00175, 0.00175, 0.00175, 0.00175, 0.00175
	White	Men	77	0.002, 0.002, 0.002, 0.002, 0.002
	White	Men	78	0.00227, 0.00227, 0.00227, 0.00227, 0.00227
	White	Men	79	0.00258, 0.00258, 0.00258, 0.00258, 0.00258
	White	Men	80	0.00291, 0.00291, 0.00291, 0.00291, 0.00291
	White	Men	81	0.00326, 0.00326, 0.00326, 0.00326, 0.00326
	White	Men	82	0.00364, 0.00364, 0.00364, 0.00364, 0.00364
	White	Men	83	0.00402, 0.00402, 0.00402, 0.00402, 0.00402
	White	Men	84	0.00441, 0.00441, 0.00441, 0.00441, 0.00441
	White	Women	30	0.0000104, 0.0000104, 0.0000104, 0.0000104, 0.0000104
	White	Women	31	0.0000118, 0.0000118, 0.0000118, 0.0000118, 0.0000118
	White	Women	32	0.0000134, 0.0000134, 0.0000134, 0.0000134, 0.0000134
	White	Women	33	0.0000152, 0.0000152, 0.0000152, 0.0000152, 0.0000152
	White	Women	34	0.0000171, 0.0000171, 0.0000171, 0.0000171, 0.0000171
	White	Women	35	0.0000193, 0.0000193, 0.0000193, 0.0000193, 0.0000193
	White	Women	36	0.0000216, 0.0000216, 0.0000216, 0.0000216, 0.0000216
	White	Women	37	0.000024, 0.000024, 0.000024, 0.000024, 0.000024
	White	Women	38	0.0000267, 0.0000267, 0.0000267, 0.0000267, 0.0000267
	White	Women	39	0.0000295, 0.0000295, 0.0000295, 0.0000295, 0.0000295
	White	Women	40	0.0000325, 0.0000325, 0.0000325, 0.0000325, 0.0000325
	White	Women	41	0.0000358, 0.0000358, 0.0000358, 0.0000358, 0.0000358
	White	Women	42	0.0000394, 0.0000394, 0.0000394, 0.0000394, 0.0000394
	White	Women	43	0.0000435, 0.0000435, 0.0000435, 0.0000435, 0.0000435
	White	Women	44	0.000048, 0.000048, 0.000048, 0.000048, 0.000048
	White	Women	45	0.0000532, 0.0000532, 0.0000532, 0.0000532, 0.0000532
	White	Women	46	0.0000589, 0.0000589, 0.0000589, 0.0000589, 0.0000589
	White	Women	47	0.0000653, 0.0000653, 0.0000653, 0.0000653, 0.0000653
	White	Women	48	0.0000724, 0.0000724, 0.0000724, 0.0000724, 0.0000724
	White	Women	49	0.00008, 0.00008, 0.00008, 0.00008, 0.00008
	White	Women	50	0.0000883, 0.0000883, 0.0000883, 0.0000883, 0.0000883
	White	Women	51	0.0000971, 0.0000971, 0.0000971, 0.0000971, 0.0000971
	White	Women	52	0.000107, 0.000107, 0.000107, 0.000107, 0.000107
	White	Women	53	0.000117, 0.000117, 0.000117, 0.000117, 0.000117
	White	Women	54	0.000127, 0.000127, 0.000127, 0.000127, 0.000127
	White	Women	55	0.000139, 0.000139, 0.000139, 0.000139, 0.000139
	White	Women	56	0.00015, 0.00015, 0.00015, 0.00015, 0.00015
	White	Women	57	0.000163, 0.000163, 0.000163, 0.000163, 0.000163
	White	Women	58	0.000177, 0.000177, 0.000177, 0.000177, 0.000177
	White	Women	59	0.000192, 0.000192, 0.000192, 0.000192, 0.000192
	White	Women	60	0.000209, 0.000209, 0.000209, 0.000209, 0.000209
	White	Women	61	0.000229, 0.000229, 0.000229, 0.000229, 0.000229
	White	Women	62	0.000252, 0.000252, 0.000252, 0.000252, 0.000252

Variable	Race/ethnicity	Sex	Age	Distribution
	White	Women	63	0.000278, 0.000278, 0.000278, 0.000278, 0.000278
	White	Women	64	0.000309, 0.000309, 0.000309, 0.000309, 0.000309
	White	Women	65	0.000345, 0.000345, 0.000345, 0.000345, 0.000345
	White	Women	66	0.000386, 0.000386, 0.000386, 0.000386, 0.000386
	White	Women	67	0.000435, 0.000435, 0.000435, 0.000435, 0.000435
	White	Women	68	0.000491, 0.000491, 0.000491, 0.000491, 0.000491
	White	Women	69	0.000557, 0.000557, 0.000557, 0.000557, 0.000557
	White	Women	70	0.000634, 0.000634, 0.000634, 0.000634, 0.000634
	White	Women	71	0.000725, 0.000725, 0.000725, 0.000725, 0.000725
	White	Women	72	0.000833, 0.000833, 0.000833, 0.000833, 0.000833
	White	Women	73	0.00096, 0.00096, 0.00096, 0.00096, 0.00096
	White	Women	74	0.00111, 0.00111, 0.00111, 0.00111, 0.00111
	White	Women	75	0.00129, 0.00129, 0.00129, 0.00129, 0.00129
	White	Women	76	0.0015, 0.0015, 0.0015, 0.0015, 0.0015
	White	Women	77	0.00175, 0.00175, 0.00175, 0.00175, 0.00175
	White	Women	78	0.00204, 0.00204, 0.00204, 0.00204, 0.00204
	White	Women	79	0.00237, 0.00237, 0.00237, 0.00237, 0.00237
	White	Women	80	0.00275, 0.00275, 0.00275, 0.00275, 0.00275
	White	Women	81	0.00318, 0.00318, 0.00318, 0.00318, 0.00318
	White	Women	82	0.00363, 0.00363, 0.00363, 0.00363, 0.00363
	White	Women	83	0.00412, 0.00412, 0.00412, 0.00412, 0.00412
	White	Women	84	0.00462, 0.00462, 0.00462, 0.00462, 0.00462
Stroke mortality rates for 2015 (0.01, 0.2, 0.5, 0.8. 0.99 percentiles of the empirical distribution produced during forecasting)				
	Black	Men	30	0.0000311, 0.0000311, 0.0000311, 0.0000311, 0.0000311
	Black	Men	31	0.0000362, 0.0000362, 0.0000362, 0.0000362, 0.0000362
	Black	Men	32	0.0000421, 0.0000421, 0.0000421, 0.0000421, 0.0000421
	Black	Men	33	0.0000488, 0.0000488, 0.0000488, 0.0000488, 0.0000488
	Black	Men	34	0.0000564, 0.0000564, 0.0000564, 0.0000564, 0.0000564
	Black	Men	35	0.0000649, 0.0000649, 0.0000649, 0.0000649, 0.0000649
	Black	Men	36	0.0000743, 0.0000743, 0.0000743, 0.0000743, 0.0000743
	Black	Men	37	0.0000847, 0.0000847, 0.0000847, 0.0000847, 0.0000847
	Black	Men	38	0.000096, 0.000096, 0.000096, 0.000096, 0.000096
	Black	Men	39	0.000108, 0.000108, 0.000108, 0.000108, 0.000108
	Black	Men	40	0.000121, 0.000121, 0.000121, 0.000121, 0.000121
	Black	Men	41	0.000135, 0.000135, 0.000135, 0.000135, 0.000135
	Black	Men	42	0.00015, 0.00015, 0.00015, 0.00015, 0.00015
	Black	Men	43	0.000165, 0.000165, 0.000165, 0.000165, 0.000165
	Black	Men	44	0.000182, 0.000182, 0.000182, 0.000182, 0.000182
	Black	Men	45	0.0002, 0.0002, 0.0002, 0.0002, 0.0002
	Black	Men	46	0.000219, 0.000219, 0.000219, 0.000219, 0.000219
	Black	Men	47	0.00024, 0.00024, 0.00024, 0.00024, 0.00024
	Black	Men	48	0.000264, 0.000264, 0.000264, 0.000264, 0.000264
	Black	Men	49	0.00029, 0.00029, 0.00029, 0.00029, 0.00029

Variable	Race/ethnicity	Sex	Age	Distribution
	Black	Men	50	0.00032, 0.00032, 0.00032, 0.00032, 0.00032
	Black	Men	51	0.000352, 0.000352, 0.000352, 0.000352, 0.000352
	Black	Men	52	0.000389, 0.000389, 0.000389, 0.000389, 0.000389
	Black	Men	53	0.000429, 0.000429, 0.000429, 0.000429, 0.000429
	Black	Men	54	0.000475, 0.000475, 0.000475, 0.000475, 0.000475
	Black	Men	55	0.000524, 0.000524, 0.000524, 0.000524, 0.000524
	Black	Men	56	0.000579, 0.000579, 0.000579, 0.000579, 0.000579
	Black	Men	57	0.000639, 0.000639, 0.000639, 0.000639, 0.000639
	Black	Men	58	0.000703, 0.000703, 0.000703, 0.000703, 0.000703
	Black	Men	59	0.000773, 0.000773, 0.000773, 0.000773, 0.000773
	Black	Men	60	0.000847, 0.000847, 0.000847, 0.000847, 0.000847
	Black	Men	61	0.000925, 0.000925, 0.000925, 0.000925, 0.000925
	Black	Men	62	0.00101, 0.00101, 0.00101, 0.00101, 0.00101
	Black	Men	63	0.00109, 0.00109, 0.00109, 0.00109, 0.00109
	Black	Men	64	0.00118, 0.00118, 0.00118, 0.00118, 0.00118
	Black	Men	65	0.00128, 0.00128, 0.00128, 0.00128, 0.00128
	Black	Men	66	0.00138, 0.00138, 0.00138, 0.00138, 0.00138
	Black	Men	67	0.00149, 0.00149, 0.00149, 0.00149, 0.00149
	Black	Men	68	0.00162, 0.00162, 0.00162, 0.00162, 0.00162
	Black	Men	69	0.00175, 0.00175, 0.00175, 0.00175, 0.00175
	Black	Men	70	0.00191, 0.00191, 0.00191, 0.00191, 0.00191
	Black	Men	71	0.00208, 0.00208, 0.00208, 0.00208, 0.00208
	Black	Men	72	0.00228, 0.00228, 0.00228, 0.00228, 0.00228
	Black	Men	73	0.00249, 0.00249, 0.00249, 0.00249, 0.00249
	Black	Men	74	0.00273, 0.00273, 0.00273, 0.00273, 0.00273
	Black	Men	75	0.00298, 0.00298, 0.00298, 0.00298, 0.00298
	Black	Men	76	0.00326, 0.00326, 0.00326, 0.00326, 0.00326
	Black	Men	77	0.00356, 0.00356, 0.00356, 0.00356, 0.00356
	Black	Men	78	0.00388, 0.00388, 0.00388, 0.00388, 0.00388
	Black	Men	79	0.00422, 0.00422, 0.00422, 0.00422, 0.00422
	Black	Men	80	0.00457, 0.00457, 0.00457, 0.00457, 0.00457
	Black	Men	81	0.00493, 0.00493, 0.00493, 0.00493, 0.00493
	Black	Men	82	0.00529, 0.00529, 0.00529, 0.00529, 0.00529
	Black	Men	83	0.00564, 0.00564, 0.00564, 0.00564, 0.00564
	Black	Men	84	0.00597, 0.00597, 0.00597, 0.00597, 0.00597
	Black	Women	30	0.0000247, 0.0000247, 0.0000247, 0.0000247, 0.0000247
	Black	Women	31	0.0000284, 0.0000284, 0.0000284, 0.0000284, 0.0000284
	Black	Women	32	0.0000326, 0.0000326, 0.0000326, 0.0000326, 0.0000326
	Black	Women	33	0.0000372, 0.0000372, 0.0000372, 0.0000372, 0.0000372
	Black	Women	34	0.0000425, 0.0000425, 0.0000425, 0.0000425, 0.0000425
	Black	Women	35	0.0000484, 0.0000484, 0.0000484, 0.0000484, 0.0000484
	Black	Women	36	0.0000549, 0.0000549, 0.0000549, 0.0000549, 0.0000549
	Black	Women	37	0.0000619, 0.0000619, 0.0000619, 0.0000619, 0.0000619

Variable	Race/ethnicity	Sex	Age	Distribution
	Black	Women	38	0.0000694, 0.0000694, 0.0000694, 0.0000694, 0.0000694
	Black	Women	39	0.0000774, 0.0000774, 0.0000774, 0.0000774, 0.0000774
	Black	Women	40	0.000086, 0.000086, 0.000086, 0.000086, 0.000086
	Black	Women	41	0.0000953, 0.0000953, 0.0000953, 0.0000953, 0.0000953
	Black	Women	42	0.000105, 0.000105, 0.000105, 0.000105, 0.000105
	Black	Women	43	0.000117, 0.000117, 0.000117, 0.000117, 0.000117
	Black	Women	44	0.000129, 0.000129, 0.000129, 0.000129, 0.000129
	Black	Women	45	0.000143, 0.000143, 0.000143, 0.000143, 0.000143
	Black	Women	46	0.000158, 0.000158, 0.000158, 0.000158, 0.000158
	Black	Women	47	0.000175, 0.000175, 0.000175, 0.000175, 0.000175
	Black	Women	48	0.000192, 0.000192, 0.000192, 0.000192, 0.000192
	Black	Women	49	0.00021, 0.00021, 0.00021, 0.00021, 0.00021
	Black	Women	50	0.000228, 0.000228, 0.000228, 0.000228, 0.000228
	Black	Women	51	0.000246, 0.000246, 0.000246, 0.000246, 0.000246
	Black	Women	52	0.000264, 0.000264, 0.000264, 0.000264, 0.000264
	Black	Women	53	0.000284, 0.000284, 0.000284, 0.000284, 0.000284
	Black	Women	54	0.000306, 0.000306, 0.000306, 0.000306, 0.000306
	Black	Women	55	0.000332, 0.000332, 0.000332, 0.000332, 0.000332
	Black	Women	56	0.000362, 0.000362, 0.000362, 0.000362, 0.000362
	Black	Women	57	0.000397, 0.000397, 0.000397, 0.000397, 0.000397
	Black	Women	58	0.000436, 0.000436, 0.000436, 0.000436, 0.000436
	Black	Women	59	0.000479, 0.000479, 0.000479, 0.000479, 0.000479
	Black	Women	60	0.000523, 0.000523, 0.000523, 0.000523, 0.000523
	Black	Women	61	0.000568, 0.000568, 0.000568, 0.000568, 0.000568
	Black	Women	62	0.000613, 0.000613, 0.000613, 0.000613, 0.000613
	Black	Women	63	0.000659, 0.000659, 0.000659, 0.000659, 0.000659
	Black	Women	64	0.000708, 0.000708, 0.000708, 0.000708, 0.000708
	Black	Women	65	0.000761, 0.000761, 0.000761, 0.000761, 0.000761
	Black	Women	66	0.000822, 0.000822, 0.000822, 0.000822, 0.000822
	Black	Women	67	0.000893, 0.000893, 0.000893, 0.000893, 0.000893
	Black	Women	68	0.000978, 0.000978, 0.000978, 0.000978, 0.000978
	Black	Women	69	0.00108, 0.00108, 0.00108, 0.00108, 0.00108
	Black	Women	70	0.0012, 0.0012, 0.0012, 0.0012, 0.0012
	Black	Women	71	0.00134, 0.00134, 0.00134, 0.00134, 0.00134
	Black	Women	72	0.0015, 0.0015, 0.0015, 0.0015, 0.0015
	Black	Women	73	0.00168, 0.00168, 0.00168, 0.00168, 0.00168
	Black	Women	74	0.00189, 0.00189, 0.00189, 0.00189, 0.00189
	Black	Women	75	0.00213, 0.00213, 0.00213, 0.00213, 0.00213
	Black	Women	76	0.00241, 0.00241, 0.00241, 0.00241, 0.00241
	Black	Women	77	0.00272, 0.00272, 0.00272, 0.00272, 0.00272
	Black	Women	78	0.00307, 0.00307, 0.00307, 0.00307, 0.00307
	Black	Women	79	0.00345, 0.00345, 0.00345, 0.00345, 0.00345
	Black	Women	80	0.00387, 0.00387, 0.00387, 0.00387, 0.00387

Variable	Race/ethnicity	Sex	Age	Distribution
	Black	Women	81	0.00431, 0.00431, 0.00431, 0.00431, 0.00431
	Black	Women	82	0.00478, 0.00478, 0.00478, 0.00478, 0.00478
	Black	Women	83	0.00525, 0.00525, 0.00525, 0.00525, 0.00525
	Black	Women	84	0.00571, 0.00571, 0.00571, 0.00571, 0.00571
	Hispanic	Men	30	0.0000163, 0.0000163, 0.0000163, 0.0000163, 0.0000163
	Hispanic	Men	31	0.0000183, 0.0000183, 0.0000183, 0.0000183, 0.0000183
	Hispanic	Men	32	0.0000206, 0.0000206, 0.0000206, 0.0000206, 0.0000206
	Hispanic	Men	33	0.000023, 0.000023, 0.000023, 0.000023, 0.000023
	Hispanic	Men	34	0.0000257, 0.0000257, 0.0000257, 0.0000257, 0.0000257
	Hispanic	Men	35	0.0000287, 0.0000287, 0.0000287, 0.0000287, 0.0000287
	Hispanic	Men	36	0.0000321, 0.0000321, 0.0000321, 0.0000321, 0.0000321
	Hispanic	Men	37	0.0000358, 0.0000358, 0.0000358, 0.0000358, 0.0000358
	Hispanic	Men	38	0.00004, 0.00004, 0.00004, 0.00004, 0.00004
	Hispanic	Men	39	0.0000447, 0.0000447, 0.0000447, 0.0000447, 0.0000447
	Hispanic	Men	40	0.0000497, 0.0000497, 0.0000497, 0.0000497, 0.0000497
	Hispanic	Men	41	0.0000552, 0.0000552, 0.0000552, 0.0000552, 0.0000552
	Hispanic	Men	42	0.000061, 0.000061, 0.000061, 0.000061, 0.000061
	Hispanic	Men	43	0.0000671, 0.0000671, 0.0000671, 0.0000671, 0.0000671
	Hispanic	Men	44	0.0000738, 0.0000738, 0.0000738, 0.0000738, 0.0000738
	Hispanic	Men	45	0.000081, 0.000081, 0.000081, 0.000081, 0.000081
	Hispanic	Men	46	0.000089, 0.000089, 0.000089, 0.000089, 0.000089
	Hispanic	Men	47	0.000098, 0.000098, 0.000098, 0.000098, 0.000098
	Hispanic	Men	48	0.000108, 0.000108, 0.000108, 0.000108, 0.000108
	Hispanic	Men	49	0.00012, 0.00012, 0.00012, 0.00012, 0.00012
	Hispanic	Men	50	0.000133, 0.000133, 0.000133, 0.000133, 0.000133
	Hispanic	Men	51	0.000148, 0.000148, 0.000148, 0.000148, 0.000148
	Hispanic	Men	52	0.000164, 0.000164, 0.000164, 0.000164, 0.000164
	Hispanic	Men	53	0.000182, 0.000182, 0.000182, 0.000182, 0.000182
	Hispanic	Men	54	0.000202, 0.000202, 0.000202, 0.000202, 0.000202
	Hispanic	Men	55	0.000223, 0.000223, 0.000223, 0.000223, 0.000223
	Hispanic	Men	56	0.000245, 0.000245, 0.000245, 0.000245, 0.000245
	Hispanic	Men	57	0.000269, 0.000269, 0.000269, 0.000269, 0.000269
	Hispanic	Men	58	0.000294, 0.000294, 0.000294, 0.000294, 0.000294
	Hispanic	Men	59	0.000322, 0.000322, 0.000322, 0.000322, 0.000322
	Hispanic	Men	60	0.000353, 0.000353, 0.000353, 0.000353, 0.000353
	Hispanic	Men	61	0.000388, 0.000388, 0.000388, 0.000388, 0.000388
	Hispanic	Men	62	0.000426, 0.000426, 0.000426, 0.000426, 0.000426
	Hispanic	Men	63	0.000468, 0.000468, 0.000468, 0.000468, 0.000468
	Hispanic	Men	64	0.000514, 0.000514, 0.000514, 0.000514, 0.000514
	Hispanic	Men	65	0.000564, 0.000564, 0.000564, 0.000564, 0.000564
	Hispanic	Men	66	0.000619, 0.000619, 0.000619, 0.000619, 0.000619
	Hispanic	Men	67	0.000681, 0.000681, 0.000681, 0.000681, 0.000681
	Hispanic	Men	68	0.00075, 0.00075, 0.00075, 0.00075, 0.00075

Variable	Race/ethnicity	Sex	Age	Distribution
	Hispanic	Men	69	0.000829, 0.000829, 0.000829, 0.000829, 0.000829
	Hispanic	Men	70	0.000922, 0.000922, 0.000922, 0.000922, 0.000922
	Hispanic	Men	71	0.00103, 0.00103, 0.00103, 0.00103, 0.00103
	Hispanic	Men	72	0.00115, 0.00115, 0.00115, 0.00115, 0.00115
	Hispanic	Men	73	0.00129, 0.00129, 0.00129, 0.00129, 0.00129
	Hispanic	Men	74	0.00146, 0.00146, 0.00146, 0.00146, 0.00146
	Hispanic	Men	75	0.00164, 0.00164, 0.00164, 0.00164, 0.00164
	Hispanic	Men	76	0.00184, 0.00184, 0.00184, 0.00184, 0.00184
	Hispanic	Men	77	0.00207, 0.00207, 0.00207, 0.00207, 0.00207
	Hispanic	Men	78	0.00232, 0.00232, 0.00232, 0.00232, 0.00232
	Hispanic	Men	79	0.00261, 0.00261, 0.00261, 0.00261, 0.00261
	Hispanic	Men	80	0.00292, 0.00292, 0.00292, 0.00292, 0.00292
	Hispanic	Men	81	0.00325, 0.00325, 0.00325, 0.00325, 0.00325
	Hispanic	Men	82	0.00361, 0.00361, 0.00361, 0.00361, 0.00361
	Hispanic	Men	83	0.00396, 0.00396, 0.00396, 0.00396, 0.00396
	Hispanic	Men	84	0.00432, 0.00432, 0.00432, 0.00432, 0.00432
	Hispanic	Women	30	0.00000965, 0.00000965, 0.00000965, 0.00000965, 0.00000965
	Hispanic	Women	31	0.000011, 0.000011, 0.000011, 0.000011, 0.000011
	Hispanic	Women	32	0.0000127, 0.0000127, 0.0000127, 0.0000127, 0.0000127
	Hispanic	Women	33	0.0000145, 0.0000145, 0.0000145, 0.0000145, 0.0000145
	Hispanic	Women	34	0.0000167, 0.0000167, 0.0000167, 0.0000167, 0.0000167
	Hispanic	Women	35	0.0000191, 0.0000191, 0.0000191, 0.0000191, 0.0000191
	Hispanic	Women	36	0.0000217, 0.0000217, 0.0000217, 0.0000217, 0.0000217
	Hispanic	Women	37	0.0000246, 0.0000246, 0.0000246, 0.0000246, 0.0000246
	Hispanic	Women	38	0.0000277, 0.0000277, 0.0000277, 0.0000277, 0.0000277
	Hispanic	Women	39	0.0000309, 0.0000309, 0.0000309, 0.0000309, 0.0000309
	Hispanic	Women	40	0.0000342, 0.0000342, 0.0000342, 0.0000342, 0.0000342
	Hispanic	Women	41	0.0000377, 0.0000377, 0.0000377, 0.0000377, 0.0000377
	Hispanic	Women	42	0.0000415, 0.0000415, 0.0000415, 0.0000415, 0.0000415
	Hispanic	Women	43	0.0000459, 0.0000459, 0.0000459, 0.0000459, 0.0000459
	Hispanic	Women	44	0.0000508, 0.0000508, 0.0000508, 0.0000508, 0.0000508
	Hispanic	Women	45	0.0000563, 0.0000563, 0.0000563, 0.0000563, 0.0000563
	Hispanic	Women	46	0.0000624, 0.0000624, 0.0000624, 0.0000624, 0.0000624
	Hispanic	Women	47	0.000069, 0.000069, 0.000069, 0.000069, 0.000069
	Hispanic	Women	48	0.0000761, 0.0000761, 0.0000761, 0.0000761, 0.0000761
	Hispanic	Women	49	0.0000838, 0.0000838, 0.0000838, 0.0000838, 0.0000838
	Hispanic	Women	50	0.0000921, 0.0000921, 0.0000921, 0.0000921, 0.0000921
	Hispanic	Women	51	0.000101, 0.000101, 0.000101, 0.000101, 0.000101
	Hispanic	Women	52	0.000111, 0.000111, 0.000111, 0.000111, 0.000111
	Hispanic	Women	53	0.000121, 0.000121, 0.000121, 0.000121, 0.000121
	Hispanic	Women	54	0.000131, 0.000131, 0.000131, 0.000131, 0.000131
	Hispanic	Women	55	0.000141, 0.000141, 0.000141, 0.000141, 0.000141
	Hispanic	Women	56	0.000151, 0.000151, 0.000151, 0.000151, 0.000151

Variable	Race/ethnicity	Sex	Age	Distribution
	Hispanic	Women	57	0.000162, 0.000162, 0.000162, 0.000162, 0.000162
	Hispanic	Women	58	0.000174, 0.000174, 0.000174, 0.000174, 0.000174
	Hispanic	Women	59	0.000189, 0.000189, 0.000189, 0.000189, 0.000189
	Hispanic	Women	60	0.000207, 0.000207, 0.000207, 0.000207, 0.000207
	Hispanic	Women	61	0.000229, 0.000229, 0.000229, 0.000229, 0.000229
	Hispanic	Women	62	0.000254, 0.000254, 0.000254, 0.000254, 0.000254
	Hispanic	Women	63	0.000282, 0.000282, 0.000282, 0.000282, 0.000282
	Hispanic	Women	64	0.000312, 0.000312, 0.000312, 0.000312, 0.000312
	Hispanic	Women	65	0.000345, 0.000345, 0.000345, 0.000345, 0.000345
	Hispanic	Women	66	0.00038, 0.00038, 0.00038, 0.00038, 0.00038
	Hispanic	Women	67	0.000418, 0.000418, 0.000418, 0.000418, 0.000418
	Hispanic	Women	68	0.000463, 0.000463, 0.000463, 0.000463, 0.000463
	Hispanic	Women	69	0.000517, 0.000517, 0.000517, 0.000517, 0.000517
	Hispanic	Women	70	0.000583, 0.000583, 0.000583, 0.000583, 0.000583
	Hispanic	Women	71	0.000664, 0.000664, 0.000664, 0.000664, 0.000664
	Hispanic	Women	72	0.000763, 0.000763, 0.000763, 0.000763, 0.000763
	Hispanic	Women	73	0.000882, 0.000882, 0.000882, 0.000882, 0.000882
	Hispanic	Women	74	0.00102, 0.00102, 0.00102, 0.00102, 0.00102
	Hispanic	Women	75	0.00119, 0.00119, 0.00119, 0.00119, 0.00119
	Hispanic	Women	76	0.00139, 0.00139, 0.00139, 0.00139, 0.00139
	Hispanic	Women	77	0.00162, 0.00162, 0.00162, 0.00162, 0.00162
	Hispanic	Women	78	0.00189, 0.00189, 0.00189, 0.00189, 0.00189
	Hispanic	Women	79	0.0022, 0.0022, 0.0022, 0.0022, 0.0022
	Hispanic	Women	80	0.00257, 0.00257, 0.00257, 0.00257, 0.00257
	Hispanic	Women	81	0.00298, 0.00298, 0.00298, 0.00298, 0.00298
	Hispanic	Women	82	0.00343, 0.00343, 0.00343, 0.00343, 0.00343
	Hispanic	Women	83	0.00391, 0.00391, 0.00391, 0.00391, 0.00391
	Hispanic	Women	84	0.00439, 0.00439, 0.00439, 0.00439, 0.00439
	White	Men	30	0.0000112, 0.0000112, 0.0000112, 0.0000112, 0.0000112
	White	Men	31	0.0000125, 0.0000125, 0.0000125, 0.0000125, 0.0000125
	White	Men	32	0.000014, 0.000014, 0.000014, 0.000014, 0.000014
	White	Men	33	0.0000156, 0.0000156, 0.0000156, 0.0000156, 0.0000156
	White	Men	34	0.0000174, 0.0000174, 0.0000174, 0.0000174, 0.0000174
	White	Men	35	0.0000194, 0.0000194, 0.0000194, 0.0000194, 0.0000194
	White	Men	36	0.0000218, 0.0000218, 0.0000218, 0.0000218, 0.0000218
	White	Men	37	0.0000245, 0.0000245, 0.0000245, 0.0000245, 0.0000245
	White	Men	38	0.0000276, 0.0000276, 0.0000276, 0.0000276, 0.0000276
	White	Men	39	0.0000311, 0.0000311, 0.0000311, 0.0000311, 0.0000311
	White	Men	40	0.000035, 0.000035, 0.000035, 0.000035, 0.000035
	White	Men	41	0.0000394, 0.0000394, 0.0000394, 0.0000394, 0.0000394
	White	Men	42	0.0000442, 0.0000442, 0.0000442, 0.0000442, 0.0000442
	White	Men	43	0.0000494, 0.0000494, 0.0000494, 0.0000494, 0.0000494
	White	Men	44	0.0000553, 0.0000553, 0.0000553, 0.0000553, 0.0000553

Variable	Race/ethnicity	Sex	Age	Distribution
	White	Men	45	0.0000619, 0.0000619, 0.0000619, 0.0000619, 0.0000619
	White	Men	46	0.0000694, 0.0000694, 0.0000694, 0.0000694, 0.0000694
	White	Men	47	0.0000778, 0.0000778, 0.0000778, 0.0000778, 0.0000778
	White	Men	48	0.0000873, 0.0000873, 0.0000873, 0.0000873, 0.0000873
	White	Men	49	0.0000979, 0.0000979, 0.0000979, 0.0000979, 0.0000979
	White	Men	50	0.000109, 0.000109, 0.000109, 0.000109, 0.000109
	White	Men	51	0.000122, 0.000122, 0.000122, 0.000122, 0.000122
	White	Men	52	0.000135, 0.000135, 0.000135, 0.000135, 0.000135
	White	Men	53	0.00015, 0.00015, 0.00015, 0.00015, 0.00015
	White	Men	54	0.000165, 0.000165, 0.000165, 0.000165, 0.000165
	White	Men	55	0.000182, 0.000182, 0.000182, 0.000182, 0.000182
	White	Men	56	0.000201, 0.000201, 0.000201, 0.000201, 0.000201
	White	Men	57	0.000222, 0.000222, 0.000222, 0.000222, 0.000222
	White	Men	58	0.000245, 0.000245, 0.000245, 0.000245, 0.000245
	White	Men	59	0.00027, 0.00027, 0.00027, 0.00027, 0.00027
	White	Men	60	0.000298, 0.000298, 0.000298, 0.000298, 0.000298
	White	Men	61	0.000327, 0.000327, 0.000327, 0.000327, 0.000327
	White	Men	62	0.000359, 0.000359, 0.000359, 0.000359, 0.000359
	White	Men	63	0.000392, 0.000392, 0.000392, 0.000392, 0.000392
	White	Men	64	0.000429, 0.000429, 0.000429, 0.000429, 0.000429
	White	Men	65	0.00047, 0.00047, 0.00047, 0.00047, 0.00047
	White	Men	66	0.000517, 0.000517, 0.000517, 0.000517, 0.000517
	White	Men	67	0.000572, 0.000572, 0.000572, 0.000572, 0.000572
	White	Men	68	0.000638, 0.000638, 0.000638, 0.000638, 0.000638
	White	Men	69	0.000716, 0.000716, 0.000716, 0.000716, 0.000716
	White	Men	70	0.000809, 0.000809, 0.000809, 0.000809, 0.000809
	White	Men	71	0.000918, 0.000918, 0.000918, 0.000918, 0.000918
	White	Men	72	0.00104, 0.00104, 0.00104, 0.00104, 0.00104
	White	Men	73	0.00118, 0.00118, 0.00118, 0.00118, 0.00118
	White	Men	74	0.00135, 0.00135, 0.00135, 0.00135, 0.00135
	White	Men	75	0.00153, 0.00153, 0.00153, 0.00153, 0.00153
	White	Men	76	0.00174, 0.00174, 0.00174, 0.00174, 0.00174
	White	Men	77	0.00198, 0.00198, 0.00198, 0.00198, 0.00198
	White	Men	78	0.00226, 0.00226, 0.00226, 0.00226, 0.00226
	White	Men	79	0.00259, 0.00259, 0.00259, 0.00259, 0.00259
	White	Men	80	0.00296, 0.00296, 0.00296, 0.00296, 0.00296
	White	Men	81	0.00338, 0.00338, 0.00338, 0.00338, 0.00338
	White	Men	82	0.00384, 0.00384, 0.00384, 0.00384, 0.00384
	White	Men	83	0.00433, 0.00433, 0.00433, 0.00433, 0.00433
	White	Men	84	0.00482, 0.00482, 0.00482, 0.00482, 0.00482
	White	Women	30	0.0000093, 0.0000093, 0.0000093, 0.0000093, 0.0000093
	White	Women	31	0.0000105, 0.0000105, 0.0000105, 0.0000105, 0.0000105
	White	Women	32	0.0000118, 0.0000118, 0.0000118, 0.0000118, 0.0000118

Variable	Race/ethnicity	Sex	Age	Distribution
	White	Women	33	0.0000135, 0.0000135, 0.0000135, 0.0000135, 0.0000135
	White	Women	34	0.0000154, 0.0000154, 0.0000154, 0.0000154, 0.0000154
	White	Women	35	0.0000177, 0.0000177, 0.0000177, 0.0000177, 0.0000177
	White	Women	36	0.0000202, 0.0000202, 0.0000202, 0.0000202, 0.0000202
	White	Women	37	0.0000231, 0.0000231, 0.0000231, 0.0000231, 0.0000231
	White	Women	38	0.0000263, 0.0000263, 0.0000263, 0.0000263, 0.0000263
	White	Women	39	0.0000297, 0.0000297, 0.0000297, 0.0000297, 0.0000297
	White	Women	40	0.0000334, 0.0000334, 0.0000334, 0.0000334, 0.0000334
	White	Women	41	0.0000373, 0.0000373, 0.0000373, 0.0000373, 0.0000373
	White	Women	42	0.0000415, 0.0000415, 0.0000415, 0.0000415, 0.0000415
	White	Women	43	0.000046, 0.000046, 0.000046, 0.000046, 0.000046
	White	Women	44	0.000051, 0.000051, 0.000051, 0.000051, 0.000051
	White	Women	45	0.0000566, 0.0000566, 0.0000566, 0.0000566, 0.0000566
	White	Women	46	0.0000628, 0.0000628, 0.0000628, 0.0000628, 0.0000628
	White	Women	47	0.0000696, 0.0000696, 0.0000696, 0.0000696, 0.0000696
	White	Women	48	0.0000771, 0.0000771, 0.0000771, 0.0000771, 0.0000771
	White	Women	49	0.0000852, 0.0000852, 0.0000852, 0.0000852, 0.0000852
	White	Women	50	0.0000939, 0.0000939, 0.0000939, 0.0000939, 0.0000939
	White	Women	51	0.000103, 0.000103, 0.000103, 0.000103, 0.000103
	White	Women	52	0.000113, 0.000113, 0.000113, 0.000113, 0.000113
	White	Women	53	0.000123, 0.000123, 0.000123, 0.000123, 0.000123
	White	Women	54	0.000134, 0.000134, 0.000134, 0.000134, 0.000134
	White	Women	55	0.000145, 0.000145, 0.000145, 0.000145, 0.000145
	White	Women	56	0.000157, 0.000157, 0.000157, 0.000157, 0.000157
	White	Women	57	0.000169, 0.000169, 0.000169, 0.000169, 0.000169
	White	Women	58	0.000182, 0.000182, 0.000182, 0.000182, 0.000182
	White	Women	59	0.000196, 0.000196, 0.000196, 0.000196, 0.000196
	White	Women	60	0.000212, 0.000212, 0.000212, 0.000212, 0.000212
	White	Women	61	0.00023, 0.00023, 0.00023, 0.00023, 0.00023
	White	Women	62	0.000251, 0.000251, 0.000251, 0.000251, 0.000251
	White	Women	63	0.000276, 0.000276, 0.000276, 0.000276, 0.000276
	White	Women	64	0.000304, 0.000304, 0.000304, 0.000304, 0.000304
	White	Women	65	0.000339, 0.000339, 0.000339, 0.000339, 0.000339
	White	Women	66	0.000379, 0.000379, 0.000379, 0.000379, 0.000379
	White	Women	67	0.000428, 0.000428, 0.000428, 0.000428, 0.000428
	White	Women	68	0.000487, 0.000487, 0.000487, 0.000487, 0.000487
	White	Women	69	0.000557, 0.000557, 0.000557, 0.000557, 0.000557
	White	Women	70	0.000641, 0.000641, 0.000641, 0.000641, 0.000641
	White	Women	71	0.00074, 0.00074, 0.00074, 0.00074, 0.00074
	White	Women	72	0.000856, 0.000856, 0.000856, 0.000856, 0.000856
	White	Women	73	0.000989, 0.000989, 0.000989, 0.000989, 0.000989
	White	Women	74	0.00114, 0.00114, 0.00114, 0.00114, 0.00114
	White	Women	75	0.00132, 0.00132, 0.00132, 0.00132, 0.00132

Variable	Race/ethnicity	Sex	Age	Distribution
	White	Women	76	0.00152, 0.00152, 0.00152, 0.00152, 0.00152
	White	Women	77	0.00176, 0.00176, 0.00176, 0.00176, 0.00176
	White	Women	78	0.00204, 0.00204, 0.00204, 0.00204, 0.00204
	White	Women	79	0.00238, 0.00238, 0.00238, 0.00238, 0.00238
	White	Women	80	0.00276, 0.00276, 0.00276, 0.00276, 0.00276
	White	Women	81	0.00321, 0.00321, 0.00321, 0.00321, 0.00321
	White	Women	82	0.00371, 0.00371, 0.00371, 0.00371, 0.00371
	White	Women	83	0.00425, 0.00425, 0.00425, 0.00425, 0.00425
	White	Women	84	0.00481, 0.00481, 0.00481, 0.00481, 0.00481
Stroke mortality rates for 2016 (0.01, 0.2, 0.5, 0.8. 0.99 percentiles of the empirical distribution produced during forecasting)				
	Black	Men	30	0.0000197, 0.0000248, 0.0000311, 0.0000391, 0.0000491
	Black	Men	31	0.0000238, 0.0000294, 0.0000363, 0.0000448, 0.0000554
	Black	Men	32	0.0000281, 0.0000345, 0.0000423, 0.0000518, 0.0000637
	Black	Men	33	0.0000325, 0.0000399, 0.0000489, 0.00006, 0.0000738
	Black	Men	34	0.0000373, 0.0000458, 0.0000563, 0.0000691, 0.000085
	Black	Men	35	0.0000427, 0.0000525, 0.0000643, 0.0000788, 0.0000968
	Black	Men	36	0.0000491, 0.0000599, 0.0000729, 0.0000888, 0.000108
	Black	Men	37	0.0000565, 0.0000682, 0.0000822, 0.000099, 0.000119
	Black	Men	38	0.0000652, 0.0000775, 0.000092, 0.000109, 0.00013
	Black	Men	39	0.0000746, 0.0000874, 0.000102, 0.00012, 0.00014
	Black	Men	40	0.0000841, 0.0000977, 0.000113, 0.000132, 0.000153
	Black	Men	41	0.0000939, 0.000109, 0.000126, 0.000145, 0.000168
	Black	Men	42	0.000105, 0.000121, 0.00014, 0.000161, 0.000185
	Black	Men	43	0.000117, 0.000135, 0.000155, 0.000179, 0.000206
	Black	Men	44	0.000128, 0.000149, 0.000173, 0.000201, 0.000233
	Black	Men	45	0.00014, 0.000164, 0.000192, 0.000226, 0.000265
	Black	Men	46	0.000153, 0.000181, 0.000213, 0.000251, 0.000297
	Black	Men	47	0.000169, 0.0002, 0.000236, 0.000279, 0.000329
	Black	Men	48	0.000186, 0.00022, 0.000261, 0.00031, 0.000367
	Black	Men	49	0.000205, 0.000244, 0.00029, 0.000345, 0.000411
	Black	Men	50	0.00023, 0.000273, 0.000324, 0.000383, 0.000455
	Black	Men	51	0.000266, 0.000311, 0.000363, 0.000423, 0.000495
	Black	Men	52	0.000309, 0.000354, 0.000406, 0.000466, 0.000534
	Black	Men	53	0.000354, 0.0004, 0.000453, 0.000512, 0.00058
	Black	Men	54	0.000398, 0.000447, 0.000502, 0.000563, 0.000633
	Black	Men	55	0.00044, 0.000493, 0.000551, 0.000616, 0.000689
	Black	Men	56	0.000482, 0.000538, 0.000601, 0.000671, 0.000751
	Black	Men	57	0.000525, 0.000586, 0.000654, 0.000729, 0.000813
	Black	Men	58	0.000577, 0.00064, 0.00071, 0.000786, 0.000872
	Black	Men	59	0.000636, 0.000701, 0.00077, 0.000847, 0.000932
	Black	Men	60	0.000691, 0.00076, 0.000835, 0.000918, 0.00101
	Black	Men	61	0.000737, 0.000817, 0.000903, 0.001, 0.00111
	Black	Men	62	0.000788, 0.000876, 0.000973, 0.00108, 0.0012

Variable	Race/ethnicity	Sex	Age	Distribution
	Black	Men	63	0.000854, 0.000944, 0.00104, 0.00115, 0.00127
	Black	Men	64	0.000927, 0.00102, 0.00111, 0.00122, 0.00134
	Black	Men	65	0.000993, 0.00109, 0.00119, 0.00131, 0.00143
	Black	Men	66	0.00106, 0.00116, 0.00128, 0.00141, 0.00155
	Black	Men	67	0.00113, 0.00125, 0.00138, 0.00152, 0.00168
	Black	Men	68	0.00124, 0.00137, 0.0015, 0.00165, 0.00181
	Black	Men	69	0.00137, 0.0015, 0.00164, 0.00178, 0.00195
	Black	Men	70	0.00151, 0.00164, 0.00179, 0.00194, 0.00212
	Black	Men	71	0.00165, 0.00179, 0.00195, 0.00212, 0.00231
	Black	Men	72	0.00179, 0.00195, 0.00213, 0.00231, 0.00252
	Black	Men	73	0.00193, 0.00211, 0.00231, 0.00253, 0.00276
	Black	Men	74	0.00206, 0.00227, 0.00251, 0.00277, 0.00305
	Black	Men	75	0.0022, 0.00245, 0.00272, 0.00303, 0.00338
	Black	Men	76	0.00237, 0.00265, 0.00296, 0.00331, 0.00371
	Black	Men	77	0.00258, 0.00289, 0.00323, 0.00361, 0.00404
	Black	Men	78	0.00283, 0.00316, 0.00352, 0.00393, 0.00439
	Black	Men	79	0.00308, 0.00344, 0.00384, 0.00428, 0.00479
	Black	Men	80	0.00332, 0.00372, 0.00417, 0.00467, 0.00523
	Black	Men	81	0.00357, 0.00401, 0.00451, 0.00506, 0.00568
	Black	Men	82	0.00383, 0.0043, 0.00484, 0.00543, 0.00611
	Black	Men	83	0.00408, 0.00459, 0.00516, 0.0058, 0.00652
	Black	Men	84	0.00431, 0.00485, 0.00546, 0.00615, 0.00692
	Black	Women	30	0.0000136, 0.0000183, 0.0000246, 0.0000331, 0.0000446
	Black	Women	31	0.0000151, 0.0000206, 0.0000282, 0.0000385, 0.0000528
	Black	Women	32	0.0000166, 0.0000232, 0.0000322, 0.0000448, 0.0000624
	Black	Women	33	0.0000184, 0.000026, 0.0000368, 0.000052, 0.0000737
	Black	Women	34	0.0000204, 0.0000293, 0.0000419, 0.0000601, 0.0000863
	Black	Women	35	0.0000228, 0.000033, 0.0000477, 0.0000688, 0.0000996
	Black	Women	36	0.0000261, 0.0000376, 0.000054, 0.0000776, 0.000112
	Black	Women	37	0.0000307, 0.0000434, 0.0000611, 0.000086, 0.000122
	Black	Women	38	0.0000367, 0.0000503, 0.0000688, 0.000094, 0.000129
	Black	Women	39	0.0000438, 0.0000583, 0.0000772, 0.000102, 0.000136
	Black	Women	40	0.0000518, 0.000067, 0.0000863, 0.000111, 0.000144
	Black	Women	41	0.0000604, 0.0000763, 0.0000961, 0.000121, 0.000153
	Black	Women	42	0.0000698, 0.0000864, 0.000107, 0.000132, 0.000163
	Black	Women	43	0.0000796, 0.0000971, 0.000118, 0.000144, 0.000175
	Black	Women	44	0.0000894, 0.000108, 0.000131, 0.000158, 0.000191
	Black	Women	45	0.0000993, 0.00012, 0.000144, 0.000174, 0.00021
	Black	Women	46	0.000111, 0.000133, 0.000159, 0.000191, 0.000229
	Black	Women	47	0.000125, 0.000149, 0.000176, 0.000208, 0.000246
	Black	Women	48	0.000143, 0.000166, 0.000193, 0.000225, 0.000262
	Black	Women	49	0.00016, 0.000184, 0.000211, 0.000242, 0.000278
	Black	Women	50	0.000178, 0.000202, 0.00023, 0.000261, 0.000298

Variable	Race/ethnicity	Sex	Age	Distribution
	Black	Women	51	0.000194, 0.00022, 0.000249, 0.000281, 0.000319
	Black	Women	52	0.000212, 0.000238, 0.000268, 0.000301, 0.000339
	Black	Women	53	0.00023, 0.000258, 0.000288, 0.000322, 0.00036
	Black	Women	54	0.000248, 0.000278, 0.00031, 0.000346, 0.000386
	Black	Women	55	0.000267, 0.000299, 0.000334, 0.000374, 0.000419
	Black	Women	56	0.000288, 0.000323, 0.000362, 0.000405, 0.000455
	Black	Women	57	0.000314, 0.000352, 0.000393, 0.00044, 0.000492
	Black	Women	58	0.000346, 0.000385, 0.000428, 0.000476, 0.00053
	Black	Women	59	0.000382, 0.000422, 0.000467, 0.000516, 0.000571
	Black	Women	60	0.000416, 0.00046, 0.000508, 0.000562, 0.000621
	Black	Women	61	0.000448, 0.000497, 0.000551, 0.000612, 0.000679
	Black	Women	62	0.000477, 0.000533, 0.000595, 0.000665, 0.000743
	Black	Women	63	0.000509, 0.000571, 0.00064, 0.000718, 0.000806
	Black	Women	64	0.000546, 0.000613, 0.000688, 0.000771, 0.000865
	Black	Women	65	0.000591, 0.000661, 0.000739, 0.000825, 0.000923
	Black	Women	66	0.000646, 0.000717, 0.000796, 0.000884, 0.000982
	Black	Women	67	0.000712, 0.000784, 0.000863, 0.000951, 0.00105
	Black	Women	68	0.000792, 0.000865, 0.000943, 0.00103, 0.00112
	Black	Women	69	0.000886, 0.000959, 0.00104, 0.00112, 0.00122
	Black	Women	70	0.00099, 0.00107, 0.00115, 0.00124, 0.00134
	Black	Women	71	0.0011, 0.00119, 0.00128, 0.00138, 0.00149
	Black	Women	72	0.00123, 0.00133, 0.00144, 0.00155, 0.00168
	Black	Women	73	0.00137, 0.00148, 0.00161, 0.00175, 0.00189
	Black	Women	74	0.00153, 0.00167, 0.00181, 0.00196, 0.00213
	Black	Women	75	0.00172, 0.00187, 0.00203, 0.00221, 0.00241
	Black	Women	76	0.0019, 0.00209, 0.00229, 0.0025, 0.00274
	Black	Women	77	0.00209, 0.00232, 0.00257, 0.00285, 0.00316
	Black	Women	78	0.0023, 0.00258, 0.00289, 0.00324, 0.00364
	Black	Women	79	0.00254, 0.00287, 0.00325, 0.00368, 0.00416
	Black	Women	80	0.00282, 0.00321, 0.00364, 0.00414, 0.0047
	Black	Women	81	0.00315, 0.00358, 0.00407, 0.00461, 0.00525
	Black	Women	82	0.0035, 0.00397, 0.00451, 0.00512, 0.00581
	Black	Women	83	0.00383, 0.00436, 0.00496, 0.00564, 0.00642
	Black	Women	84	0.00414, 0.00474, 0.00541, 0.00617, 0.00706
	Hispanic	Men	30	0.00000894, 0.0000115, 0.0000147, 0.0000187, 0.000024
	Hispanic	Men	31	0.0000106, 0.0000132, 0.0000166, 0.0000207, 0.000026
	Hispanic	Men	32	0.0000124, 0.0000152, 0.0000187, 0.000023, 0.0000283
	Hispanic	Men	33	0.0000145, 0.0000175, 0.0000212, 0.0000256, 0.0000309
	Hispanic	Men	34	0.0000168, 0.00002, 0.0000239, 0.0000285, 0.0000341
	Hispanic	Men	35	0.0000193, 0.0000229, 0.000027, 0.0000318, 0.0000376
	Hispanic	Men	36	0.0000223, 0.0000261, 0.0000304, 0.0000355, 0.0000415
	Hispanic	Men	37	0.0000258, 0.0000298, 0.0000343, 0.0000394, 0.0000455
	Hispanic	Men	38	0.0000298, 0.0000339, 0.0000385, 0.0000438, 0.0000499

Variable	Race/ethnicity	Sex	Age	Distribution
	Hispanic	Men	39	0.000034, 0.0000384, 0.0000432, 0.0000487, 0.000055
	Hispanic	Men	40	0.0000382, 0.000043, 0.0000484, 0.0000545, 0.0000614
	Hispanic	Men	41	0.0000422, 0.0000478, 0.0000541, 0.0000611, 0.0000692
	Hispanic	Men	42	0.0000464, 0.0000529, 0.0000602, 0.0000686, 0.0000782
	Hispanic	Men	43	0.0000509, 0.0000584, 0.0000669, 0.0000767, 0.000088
	Hispanic	Men	44	0.000056, 0.0000646, 0.0000743, 0.0000855, 0.0000985
	Hispanic	Men	45	0.0000618, 0.0000714, 0.0000824, 0.0000952, 0.00011
	Hispanic	Men	46	0.0000683, 0.0000791, 0.0000915, 0.000106, 0.000123
	Hispanic	Men	47	0.0000758, 0.0000878, 0.000102, 0.000117, 0.000136
	Hispanic	Men	48	0.0000847, 0.0000978, 0.000113, 0.00013, 0.00015
	Hispanic	Men	49	0.0000952, 0.000109, 0.000125, 0.000143, 0.000164
	Hispanic	Men	50	0.000108, 0.000122, 0.000138, 0.000157, 0.000178
	Hispanic	Men	51	0.000122, 0.000137, 0.000153, 0.000171, 0.000192
	Hispanic	Men	52	0.000137, 0.000152, 0.000169, 0.000187, 0.000208
	Hispanic	Men	53	0.000151, 0.000168, 0.000186, 0.000206, 0.000228
	Hispanic	Men	54	0.000163, 0.000183, 0.000204, 0.000228, 0.000255
	Hispanic	Men	55	0.000175, 0.000198, 0.000223, 0.000252, 0.000284
	Hispanic	Men	56	0.000189, 0.000215, 0.000244, 0.000276, 0.000314
	Hispanic	Men	57	0.000206, 0.000234, 0.000265, 0.000301, 0.000342
	Hispanic	Men	58	0.000228, 0.000257, 0.000289, 0.000325, 0.000366
	Hispanic	Men	59	0.000254, 0.000283, 0.000315, 0.00035, 0.000389
	Hispanic	Men	60	0.000284, 0.000312, 0.000342, 0.000376, 0.000413
	Hispanic	Men	61	0.000314, 0.000342, 0.000372, 0.000405, 0.00044
	Hispanic	Men	62	0.000343, 0.000373, 0.000404, 0.000438, 0.000476
	Hispanic	Men	63	0.000371, 0.000404, 0.000439, 0.000477, 0.00052
	Hispanic	Men	64	0.000399, 0.000436, 0.000477, 0.000521, 0.00057
	Hispanic	Men	65	0.000429, 0.000472, 0.000519, 0.000571, 0.000628
	Hispanic	Men	66	0.000464, 0.000513, 0.000567, 0.000626, 0.000692
	Hispanic	Men	67	0.000503, 0.000559, 0.000621, 0.00069, 0.000767
	Hispanic	Men	68	0.000548, 0.000612, 0.000684, 0.000764, 0.000854
	Hispanic	Men	69	0.000602, 0.000675, 0.000757, 0.000848, 0.000951
	Hispanic	Men	70	0.000669, 0.00075, 0.000841, 0.000943, 0.00106
	Hispanic	Men	71	0.000749, 0.000839, 0.000939, 0.00105, 0.00118
	Hispanic	Men	72	0.000842, 0.000941, 0.00105, 0.00117, 0.00131
	Hispanic	Men	73	0.000946, 0.00106, 0.00118, 0.00131, 0.00146
	Hispanic	Men	74	0.00106, 0.00118, 0.00132, 0.00147, 0.00164
	Hispanic	Men	75	0.00119, 0.00133, 0.00148, 0.00165, 0.00185
	Hispanic	Men	76	0.00133, 0.00149, 0.00166, 0.00186, 0.00208
	Hispanic	Men	77	0.00148, 0.00166, 0.00186, 0.00209, 0.00234
	Hispanic	Men	78	0.00165, 0.00186, 0.00209, 0.00235, 0.00264
	Hispanic	Men	79	0.00184, 0.00207, 0.00234, 0.00264, 0.00298
	Hispanic	Men	80	0.00203, 0.00231, 0.00261, 0.00296, 0.00336
	Hispanic	Men	81	0.00223, 0.00255, 0.0029, 0.00331, 0.00377

Variable	Race/ethnicity	Sex	Age	Distribution
	Hispanic	Men	82	0.00244, 0.0028, 0.0032, 0.00367, 0.00421
	Hispanic	Men	83	0.00264, 0.00305, 0.00351, 0.00405, 0.00467
	Hispanic	Men	84	0.00284, 0.00329, 0.00382, 0.00442, 0.00513
	Hispanic	Women	30	0.00000497, 0.00000709, 0.0000101, 0.0000143, 0.0000204
	Hispanic	Women	31	0.00000574, 0.00000806, 0.0000113, 0.0000158, 0.0000221
	Hispanic	Women	32	0.00000662, 0.00000917, 0.0000127, 0.0000175, 0.0000243
	Hispanic	Women	33	0.00000762, 0.0000105, 0.0000143, 0.0000197, 0.000027
	Hispanic	Women	34	0.00000884, 0.000012, 0.0000163, 0.0000221, 0.00003
	Hispanic	Women	35	0.0000104, 0.0000139, 0.0000185, 0.0000246, 0.000033
	Hispanic	Women	36	0.0000123, 0.0000161, 0.000021, 0.0000274, 0.0000358
	Hispanic	Women	37	0.0000146, 0.0000187, 0.0000238, 0.0000303, 0.0000388
	Hispanic	Women	38	0.0000173, 0.0000216, 0.0000269, 0.0000336, 0.0000419
	Hispanic	Women	39	0.0000202, 0.0000248, 0.0000303, 0.0000372, 0.0000456
	Hispanic	Women	40	0.0000231, 0.0000281, 0.000034, 0.0000412, 0.00005
	Hispanic	Women	41	0.000026, 0.0000314, 0.000038, 0.0000458, 0.0000555
	Hispanic	Women	42	0.0000287, 0.0000348, 0.0000422, 0.0000511, 0.000062
	Hispanic	Women	43	0.0000316, 0.0000385, 0.0000467, 0.0000568, 0.0000691
	Hispanic	Women	44	0.0000352, 0.0000427, 0.0000517, 0.0000626, 0.000076
	Hispanic	Women	45	0.0000397, 0.0000476, 0.0000571, 0.0000684, 0.0000821
	Hispanic	Women	46	0.0000452, 0.0000534, 0.000063, 0.0000743, 0.0000878
	Hispanic	Women	47	0.0000513, 0.0000597, 0.0000693, 0.0000806, 0.0000938
	Hispanic	Women	48	0.0000572, 0.0000661, 0.0000762, 0.0000879, 0.000102
	Hispanic	Women	49	0.0000625, 0.0000724, 0.0000837, 0.0000967, 0.000112
	Hispanic	Women	50	0.0000671, 0.0000785, 0.0000916, 0.000107, 0.000125
	Hispanic	Women	51	0.0000716, 0.0000847, 0.0001, 0.000118, 0.00014
	Hispanic	Women	52	0.0000762, 0.0000914, 0.000109, 0.000131, 0.000157
	Hispanic	Women	53	0.0000816, 0.0000987, 0.000119, 0.000144, 0.000174
	Hispanic	Women	54	0.000088, 0.000107, 0.00013, 0.000157, 0.000191
	Hispanic	Women	55	0.0000957, 0.000116, 0.000141, 0.000171, 0.000208
	Hispanic	Women	56	0.000105, 0.000127, 0.000154, 0.000186, 0.000225
	Hispanic	Women	57	0.000115, 0.000139, 0.000167, 0.000201, 0.000242
	Hispanic	Women	58	0.000127, 0.000152, 0.000182, 0.000218, 0.000262
	Hispanic	Women	59	0.00014, 0.000167, 0.000199, 0.000238, 0.000284
	Hispanic	Women	60	0.000154, 0.000184, 0.000218, 0.000259, 0.000308
	Hispanic	Women	61	0.000171, 0.000202, 0.000239, 0.000283, 0.000335
	Hispanic	Women	62	0.000188, 0.000222, 0.000262, 0.000309, 0.000365
	Hispanic	Women	63	0.000207, 0.000244, 0.000287, 0.000337, 0.000398
	Hispanic	Women	64	0.000226, 0.000266, 0.000314, 0.00037, 0.000436
	Hispanic	Women	65	0.000246, 0.000291, 0.000344, 0.000406, 0.00048
	Hispanic	Women	66	0.00027, 0.00032, 0.000378, 0.000447, 0.000529
	Hispanic	Women	67	0.000299, 0.000354, 0.000418, 0.000494, 0.000585
	Hispanic	Women	68	0.000334, 0.000394, 0.000466, 0.000549, 0.000649
	Hispanic	Women	69	0.000376, 0.000444, 0.000522, 0.000615, 0.000725

Variable	Race/ethnicity	Sex	Age	Distribution
	Hispanic	Women	70	0.000429, 0.000504, 0.00059, 0.000692, 0.000811
	Hispanic	Women	71	0.000497, 0.000578, 0.000671, 0.000779, 0.000906
	Hispanic	Women	72	0.000583, 0.000669, 0.000766, 0.000878, 0.00101
	Hispanic	Women	73	0.000688, 0.000778, 0.000877, 0.00099, 0.00112
	Hispanic	Women	74	0.000809, 0.000903, 0.00101, 0.00112, 0.00125
	Hispanic	Women	75	0.000941, 0.00104, 0.00115, 0.00128, 0.00141
	Hispanic	Women	76	0.00108, 0.0012, 0.00132, 0.00146, 0.00162
	Hispanic	Women	77	0.00124, 0.00138, 0.00152, 0.00168, 0.00186
	Hispanic	Women	78	0.00144, 0.00159, 0.00175, 0.00193, 0.00213
	Hispanic	Women	79	0.00167, 0.00183, 0.00201, 0.00221, 0.00242
	Hispanic	Women	80	0.00193, 0.00211, 0.00231, 0.00252, 0.00276
	Hispanic	Women	81	0.00218, 0.0024, 0.00264, 0.0029, 0.00319
	Hispanic	Women	82	0.00239, 0.00268, 0.00299, 0.00334, 0.00373
	Hispanic	Women	83	0.00257, 0.00294, 0.00336, 0.00384, 0.00439
	Hispanic	Women	84	0.00274, 0.0032, 0.00374, 0.00436, 0.00509
	White	Men	30	0.00000733, 0.00000908, 0.0000112, 0.0000139, 0.0000173
	White	Men	31	0.00000836, 0.0000103, 0.0000126, 0.0000155, 0.0000191
	White	Men	32	0.00000956, 0.0000116, 0.0000142, 0.0000172, 0.000021
	White	Men	33	0.000011, 0.0000132, 0.0000159, 0.0000191, 0.000023
	White	Men	34	0.0000126, 0.000015, 0.0000178, 0.0000211, 0.0000252
	White	Men	35	0.0000145, 0.000017, 0.00002, 0.0000234, 0.0000275
	White	Men	36	0.0000166, 0.0000193, 0.0000224, 0.000026, 0.0000302
	White	Men	37	0.0000189, 0.0000218, 0.0000251, 0.000029, 0.0000335
	White	Men	38	0.0000214, 0.0000246, 0.0000282, 0.0000324, 0.0000372
	White	Men	39	0.0000241, 0.0000277, 0.0000317, 0.0000362, 0.0000415
	White	Men	40	0.0000271, 0.000031, 0.0000354, 0.0000404, 0.0000462
	White	Men	41	0.0000304, 0.0000346, 0.0000395, 0.0000449, 0.0000512
	White	Men	42	0.0000341, 0.0000387, 0.0000439, 0.0000497, 0.0000564
	White	Men	43	0.0000384, 0.0000433, 0.0000487, 0.0000548, 0.0000618
	White	Men	44	0.0000433, 0.0000484, 0.0000541, 0.0000604, 0.0000676
	White	Men	45	0.0000487, 0.0000542, 0.0000601, 0.0000667, 0.0000742
	White	Men	46	0.0000547, 0.0000605, 0.0000669, 0.000074, 0.0000819
	White	Men	47	0.0000611, 0.0000676, 0.0000746, 0.0000824, 0.0000911
	White	Men	48	0.0000681, 0.0000754, 0.0000833, 0.0000921, 0.000102
	White	Men	49	0.0000759, 0.0000841, 0.000093, 0.000103, 0.000114
	White	Men	50	0.0000847, 0.0000938, 0.000104, 0.000115, 0.000127
	White	Men	51	0.0000949, 0.000105, 0.000116, 0.000128, 0.000141
	White	Men	52	0.000106, 0.000117, 0.000129, 0.000141, 0.000156
	White	Men	53	0.000119, 0.00013, 0.000143, 0.000156, 0.000171
	White	Men	54	0.000132, 0.000144, 0.000158, 0.000173, 0.000189
	White	Men	55	0.000145, 0.000159, 0.000174, 0.000191, 0.00021
	White	Men	56	0.000158, 0.000174, 0.000192, 0.000212, 0.000234
	White	Men	57	0.000172, 0.000191, 0.000211, 0.000234, 0.00026

Variable	Race/ethnicity	Sex	Age	Distribution
	White	Men	58	0.000188, 0.000209, 0.000233, 0.000258, 0.000287
	White	Men	59	0.000207, 0.00023, 0.000256, 0.000284, 0.000316
	White	Men	60	0.000229, 0.000254, 0.000281, 0.000312, 0.000346
	White	Men	61	0.000254, 0.000281, 0.000309, 0.000341, 0.000376
	White	Men	62	0.000283, 0.00031, 0.000339, 0.000371, 0.000406
	White	Men	63	0.000316, 0.000343, 0.000372, 0.000404, 0.000438
	White	Men	64	0.000353, 0.00038, 0.000409, 0.000439, 0.000473
	White	Men	65	0.000393, 0.00042, 0.000449, 0.00048, 0.000513
	White	Men	66	0.000438, 0.000466, 0.000496, 0.000527, 0.000561
	White	Men	67	0.000488, 0.000519, 0.000551, 0.000584, 0.000621
	White	Men	68	0.000544, 0.000578, 0.000615, 0.000654, 0.000696
	White	Men	69	0.000606, 0.000648, 0.000691, 0.000738, 0.000788
	White	Men	70	0.000678, 0.000728, 0.00078, 0.000837, 0.000898
	White	Men	71	0.000761, 0.00082, 0.000883, 0.000952, 0.00103
	White	Men	72	0.000857, 0.000927, 0.001, 0.00108, 0.00117
	White	Men	73	0.000969, 0.00105, 0.00113, 0.00123, 0.00133
	White	Men	74	0.0011, 0.00119, 0.00129, 0.00139, 0.00151
	White	Men	75	0.00124, 0.00135, 0.00146, 0.00158, 0.00171
	White	Men	76	0.00141, 0.00153, 0.00166, 0.00179, 0.00195
	White	Men	77	0.00159, 0.00173, 0.00188, 0.00205, 0.00223
	White	Men	78	0.0018, 0.00197, 0.00215, 0.00235, 0.00256
	White	Men	79	0.00204, 0.00224, 0.00246, 0.00269, 0.00296
	White	Men	80	0.0023, 0.00254, 0.00281, 0.0031, 0.00343
	White	Men	81	0.00256, 0.00287, 0.0032, 0.00358, 0.004
	White	Men	82	0.00282, 0.0032, 0.00362, 0.00411, 0.00467
	White	Men	83	0.00306, 0.00353, 0.00407, 0.00469, 0.00541
	White	Men	84	0.0033, 0.00386, 0.00451, 0.00527, 0.00617
	White	Women	30	0.00000629, 0.00000811, 0.0000104, 0.0000134, 0.0000173
	White	Women	31	0.00000715, 0.0000092, 0.0000118, 0.0000152, 0.0000195
	White	Women	32	0.00000821, 0.0000105, 0.0000134, 0.000017, 0.0000218
	White	Women	33	0.00000949, 0.000012, 0.0000151, 0.000019, 0.000024
	White	Women	34	0.000011, 0.0000137, 0.000017, 0.0000211, 0.0000263
	White	Women	35	0.0000127, 0.0000156, 0.0000191, 0.0000234, 0.0000287
	White	Women	36	0.0000146, 0.0000177, 0.0000214, 0.0000258, 0.0000312
	White	Women	37	0.0000168, 0.0000201, 0.0000239, 0.0000284, 0.0000339
	White	Women	38	0.0000192, 0.0000226, 0.0000266, 0.0000313, 0.0000369
	White	Women	39	0.0000217, 0.0000253, 0.0000295, 0.0000344, 0.0000401
	White	Women	40	0.0000244, 0.0000282, 0.0000326, 0.0000377, 0.0000437
	White	Women	41	0.0000272, 0.0000313, 0.000036, 0.0000413, 0.0000475
	White	Women	42	0.0000302, 0.0000346, 0.0000395, 0.0000451, 0.0000516
	White	Women	43	0.0000336, 0.0000382, 0.0000434, 0.0000492, 0.000056
	White	Women	44	0.0000372, 0.0000421, 0.0000476, 0.0000538, 0.0000609
	White	Women	45	0.0000408, 0.0000462, 0.0000523, 0.0000591, 0.000067

Variable	Race/ethnicity	Sex	Age	Distribution
	White	Women	46	0.0000443, 0.0000505, 0.0000574, 0.0000653, 0.0000744
	White	Women	47	0.0000478, 0.000055, 0.0000632, 0.0000725, 0.0000834
	White	Women	48	0.0000513, 0.0000597, 0.0000694, 0.0000807, 0.0000939
	White	Women	49	0.0000551, 0.0000649, 0.0000762, 0.0000896, 0.000106
	White	Women	50	0.0000595, 0.0000706, 0.0000836, 0.000099, 0.000117
	White	Women	51	0.000065, 0.0000772, 0.0000915, 0.000108, 0.000129
	White	Women	52	0.0000719, 0.0000848, 0.0000999, 0.000118, 0.000139
	White	Women	53	0.0000802, 0.0000934, 0.000109, 0.000127, 0.000147
	White	Women	54	0.0000893, 0.000103, 0.000118, 0.000136, 0.000156
	White	Women	55	0.0000991, 0.000113, 0.000128, 0.000145, 0.000165
	White	Women	56	0.00011, 0.000123, 0.000139, 0.000156, 0.000175
	White	Women	57	0.000121, 0.000135, 0.00015, 0.000167, 0.000186
	White	Women	58	0.000135, 0.000149, 0.000164, 0.00018, 0.000199
	White	Women	59	0.000151, 0.000164, 0.000179, 0.000195, 0.000213
	White	Women	60	0.000168, 0.000182, 0.000197, 0.000214, 0.000232
	White	Women	61	0.000187, 0.000202, 0.000218, 0.000236, 0.000255
	White	Women	62	0.000207, 0.000224, 0.000242, 0.000261, 0.000282
	White	Women	63	0.00023, 0.000248, 0.000268, 0.00029, 0.000314
	White	Women	64	0.000255, 0.000276, 0.000298, 0.000323, 0.000349
	White	Women	65	0.000284, 0.000307, 0.000332, 0.000359, 0.000389
	White	Women	66	0.000316, 0.000343, 0.000371, 0.000401, 0.000434
	White	Women	67	0.000355, 0.000385, 0.000416, 0.00045, 0.000487
	White	Women	68	0.000403, 0.000435, 0.00047, 0.000507, 0.000548
	White	Women	69	0.000461, 0.000497, 0.000534, 0.000575, 0.000619
	White	Women	70	0.000532, 0.000571, 0.000612, 0.000657, 0.000705
	White	Women	71	0.000613, 0.000658, 0.000705, 0.000756, 0.00081
	White	Women	72	0.000706, 0.000758, 0.000815, 0.000875, 0.00094
	White	Women	73	0.000811, 0.000874, 0.000942, 0.00102, 0.0011
	White	Women	74	0.000933, 0.00101, 0.00109, 0.00118, 0.00127
	White	Women	75	0.00108, 0.00117, 0.00126, 0.00136, 0.00148
	White	Women	76	0.00124, 0.00135, 0.00146, 0.00158, 0.00172
	White	Women	77	0.00142, 0.00155, 0.00169, 0.00184, 0.00201
	White	Women	78	0.00162, 0.00179, 0.00196, 0.00215, 0.00237
	White	Women	79	0.00186, 0.00206, 0.00228, 0.00252, 0.0028
	White	Women	80	0.00213, 0.00237, 0.00265, 0.00295, 0.0033
	White	Women	81	0.00244, 0.00273, 0.00307, 0.00344, 0.00386
	White	Women	82	0.00278, 0.00313, 0.00353, 0.00397, 0.00448
	White	Women	83	0.00315, 0.00356, 0.00402, 0.00455, 0.00514
	White	Women	84	0.00352, 0.004, 0.00453, 0.00514, 0.00583
Stroke mortality rates for 2017 (0.01, 0.2, 0.5, 0.8. 0.99 percentiles of the empirical distribution produced during forecasting)				
	Black	Men	30	0.000019, 0.0000243, 0.0000309, 0.0000393, 0.0000501
	Black	Men	31	0.000023, 0.0000288, 0.000036, 0.000045, 0.0000563
	Black	Men	32	0.0000272, 0.0000338, 0.0000419, 0.0000519, 0.0000645

Variable	Race/ethnicity	Sex	Age	Distribution
	Black	Men	33	0.0000315, 0.0000391, 0.0000485, 0.0000601, 0.0000746
	Black	Men	34	0.0000362, 0.000045, 0.0000557, 0.0000691, 0.0000859
	Black	Men	35	0.0000415, 0.0000514, 0.0000636, 0.0000787, 0.0000976
	Black	Men	36	0.0000476, 0.0000586, 0.0000721, 0.0000887, 0.000109
	Black	Men	37	0.0000547, 0.0000667, 0.0000811, 0.0000987, 0.00012
	Black	Men	38	0.0000629, 0.0000756, 0.0000906, 0.000109, 0.000131
	Black	Men	39	0.0000718, 0.0000851, 0.000101, 0.000119, 0.000141
	Black	Men	40	0.0000809, 0.0000951, 0.000111, 0.000131, 0.000154
	Black	Men	41	0.0000905, 0.000106, 0.000123, 0.000144, 0.000168
	Black	Men	42	0.000102, 0.000118, 0.000137, 0.000158, 0.000184
	Black	Men	43	0.000113, 0.000131, 0.000152, 0.000176, 0.000203
	Black	Men	44	0.000124, 0.000145, 0.000169, 0.000197, 0.00023
	Black	Men	45	0.000135, 0.00016, 0.000188, 0.000221, 0.000261
	Black	Men	46	0.000149, 0.000176, 0.000209, 0.000247, 0.000293
	Black	Men	47	0.000164, 0.000195, 0.000232, 0.000275, 0.000327
	Black	Men	48	0.00018, 0.000216, 0.000257, 0.000307, 0.000367
	Black	Men	49	0.000198, 0.000239, 0.000287, 0.000344, 0.000414
	Black	Men	50	0.000224, 0.000268, 0.000321, 0.000384, 0.00046
	Black	Men	51	0.000258, 0.000305, 0.00036, 0.000424, 0.000501
	Black	Men	52	0.000301, 0.000349, 0.000404, 0.000467, 0.000541
	Black	Men	53	0.000346, 0.000395, 0.000451, 0.000514, 0.000587
	Black	Men	54	0.00039, 0.000441, 0.000499, 0.000564, 0.000639
	Black	Men	55	0.000431, 0.000487, 0.000548, 0.000617, 0.000696
	Black	Men	56	0.000471, 0.000531, 0.000598, 0.000673, 0.000758
	Black	Men	57	0.000513, 0.000577, 0.000649, 0.000729, 0.000821
	Black	Men	58	0.000563, 0.000629, 0.000703, 0.000786, 0.000879
	Black	Men	59	0.000618, 0.000686, 0.000761, 0.000844, 0.000937
	Black	Men	60	0.000669, 0.000742, 0.000823, 0.000913, 0.00101
	Black	Men	61	0.000712, 0.000795, 0.000888, 0.000991, 0.00111
	Black	Men	62	0.00076, 0.000852, 0.000954, 0.00107, 0.0012
	Black	Men	63	0.000822, 0.000916, 0.00102, 0.00114, 0.00127
	Black	Men	64	0.000893, 0.000987, 0.00109, 0.0012, 0.00133
	Black	Men	65	0.000957, 0.00106, 0.00116, 0.00128, 0.00142
	Black	Men	66	0.00102, 0.00113, 0.00125, 0.00138, 0.00153
	Black	Men	67	0.00109, 0.00121, 0.00135, 0.00149, 0.00166
	Black	Men	68	0.0012, 0.00132, 0.00146, 0.00161, 0.00178
	Black	Men	69	0.00132, 0.00145, 0.00159, 0.00174, 0.00192
	Black	Men	70	0.00145, 0.00158, 0.00173, 0.0019, 0.00208
	Black	Men	71	0.00158, 0.00173, 0.00189, 0.00207, 0.00227
	Black	Men	72	0.00171, 0.00188, 0.00206, 0.00225, 0.00247
	Black	Men	73	0.00185, 0.00203, 0.00224, 0.00246, 0.00271
	Black	Men	74	0.00197, 0.00219, 0.00243, 0.00269, 0.00298
	Black	Men	75	0.0021, 0.00235, 0.00263, 0.00294, 0.0033

Variable	Race/ethnicity	Sex	Age	Distribution
	Black	Men	76	0.00227, 0.00255, 0.00286, 0.00322, 0.00362
	Black	Men	77	0.00247, 0.00278, 0.00312, 0.00351, 0.00394
	Black	Men	78	0.0027, 0.00303, 0.0034, 0.00382, 0.0043
	Black	Men	79	0.00293, 0.0033, 0.00371, 0.00417, 0.0047
	Black	Men	80	0.00316, 0.00357, 0.00403, 0.00455, 0.00514
	Black	Men	81	0.00339, 0.00384, 0.00435, 0.00493, 0.00559
	Black	Men	82	0.00363, 0.00412, 0.00467, 0.0053, 0.00602
	Black	Men	83	0.00386, 0.00439, 0.00498, 0.00565, 0.00643
	Black	Men	84	0.00408, 0.00464, 0.00528, 0.006, 0.00683
	Black	Women	30	0.0000131, 0.0000179, 0.0000245, 0.0000335, 0.000046
	Black	Women	31	0.0000146, 0.0000203, 0.0000281, 0.0000388, 0.0000539
	Black	Women	32	0.0000163, 0.0000229, 0.0000321, 0.000045, 0.0000633
	Black	Women	33	0.000018, 0.0000257, 0.0000366, 0.000052, 0.0000743
	Black	Women	34	0.0000199, 0.0000288, 0.0000416, 0.00006, 0.0000868
	Black	Women	35	0.0000223, 0.0000325, 0.0000472, 0.0000686, 0.0000999
	Black	Women	36	0.0000254, 0.0000369, 0.0000534, 0.0000772, 0.000112
	Black	Women	37	0.0000297, 0.0000423, 0.0000601, 0.0000853, 0.000122
	Black	Women	38	0.0000353, 0.0000489, 0.0000674, 0.000093, 0.000129
	Black	Women	39	0.0000419, 0.0000563, 0.0000753, 0.000101, 0.000135
	Black	Women	40	0.0000492, 0.0000643, 0.0000838, 0.000109, 0.000143
	Black	Women	41	0.000057, 0.0000729, 0.0000929, 0.000119, 0.000152
	Black	Women	42	0.0000656, 0.0000822, 0.000103, 0.000128, 0.000161
	Black	Women	43	0.0000746, 0.000092, 0.000113, 0.000139, 0.000172
	Black	Women	44	0.0000835, 0.000102, 0.000125, 0.000153, 0.000187
	Black	Women	45	0.0000927, 0.000113, 0.000138, 0.000168, 0.000205
	Black	Women	46	0.000104, 0.000126, 0.000152, 0.000184, 0.000223
	Black	Women	47	0.000117, 0.00014, 0.000168, 0.0002, 0.000239
	Black	Women	48	0.000134, 0.000157, 0.000185, 0.000216, 0.000254
	Black	Women	49	0.000151, 0.000175, 0.000202, 0.000234, 0.000271
	Black	Women	50	0.000168, 0.000192, 0.00022, 0.000253, 0.00029
	Black	Women	51	0.000184, 0.00021, 0.000239, 0.000272, 0.000311
	Black	Women	52	0.000201, 0.000228, 0.000258, 0.000292, 0.000331
	Black	Women	53	0.000219, 0.000247, 0.000278, 0.000313, 0.000352
	Black	Women	54	0.000237, 0.000266, 0.000299, 0.000336, 0.000378
	Black	Women	55	0.000254, 0.000287, 0.000323, 0.000364, 0.000411
	Black	Women	56	0.000275, 0.000311, 0.000351, 0.000396, 0.000448
	Black	Women	57	0.000301, 0.00034, 0.000382, 0.00043, 0.000485
	Black	Women	58	0.000333, 0.000373, 0.000418, 0.000467, 0.000523
	Black	Women	59	0.000368, 0.00041, 0.000456, 0.000508, 0.000565
	Black	Women	60	0.000402, 0.000448, 0.000497, 0.000553, 0.000615
	Black	Women	61	0.000432, 0.000483, 0.00054, 0.000603, 0.000674
	Black	Women	62	0.000461, 0.000518, 0.000582, 0.000655, 0.000737
	Black	Women	63	0.000491, 0.000555, 0.000626, 0.000706, 0.000798

Variable	Race/ethnicity	Sex	Age	Distribution
	Black	Women	64	0.000526, 0.000595, 0.000671, 0.000757, 0.000855
	Black	Women	65	0.000569, 0.00064, 0.00072, 0.000809, 0.00091
	Black	Women	66	0.000621, 0.000694, 0.000775, 0.000864, 0.000966
	Black	Women	67	0.000684, 0.000758, 0.000839, 0.000928, 0.00103
	Black	Women	68	0.00076, 0.000835, 0.000916, 0.001, 0.0011
	Black	Women	69	0.000849, 0.000925, 0.00101, 0.0011, 0.00119
	Black	Women	70	0.000948, 0.00103, 0.00112, 0.00121, 0.00131
	Black	Women	71	0.00106, 0.00115, 0.00124, 0.00135, 0.00147
	Black	Women	72	0.00117, 0.00128, 0.00139, 0.00152, 0.00165
	Black	Women	73	0.00131, 0.00143, 0.00156, 0.00171, 0.00186
	Black	Women	74	0.00147, 0.00161, 0.00176, 0.00192, 0.0021
	Black	Women	75	0.00165, 0.00181, 0.00198, 0.00216, 0.00237
	Black	Women	76	0.00183, 0.00202, 0.00223, 0.00245, 0.0027
	Black	Women	77	0.00202, 0.00225, 0.00251, 0.00279, 0.00311
	Black	Women	78	0.00222, 0.00251, 0.00282, 0.00318, 0.00359
	Black	Women	79	0.00245, 0.00279, 0.00317, 0.00361, 0.00411
	Black	Women	80	0.00273, 0.00312, 0.00356, 0.00406, 0.00464
	Black	Women	81	0.00304, 0.00348, 0.00398, 0.00454, 0.0052
	Black	Women	82	0.00337, 0.00386, 0.00441, 0.00505, 0.00578
	Black	Women	83	0.00368, 0.00423, 0.00486, 0.00557, 0.00641
	Black	Women	84	0.00397, 0.00459, 0.0053, 0.00611, 0.00707
	Hispanic	Men	30	0.00000873, 0.0000113, 0.0000147, 0.000019, 0.0000247
	Hispanic	Men	31	0.0000103, 0.0000131, 0.0000166, 0.000021, 0.0000267
	Hispanic	Men	32	0.0000122, 0.0000151, 0.0000188, 0.0000234, 0.0000291
	Hispanic	Men	33	0.0000142, 0.0000174, 0.0000213, 0.000026, 0.0000319
	Hispanic	Men	34	0.0000164, 0.0000199, 0.0000241, 0.0000291, 0.0000353
	Hispanic	Men	35	0.0000189, 0.0000227, 0.0000272, 0.0000326, 0.0000392
	Hispanic	Men	36	0.0000217, 0.0000258, 0.0000306, 0.0000364, 0.0000433
	Hispanic	Men	37	0.000025, 0.0000294, 0.0000345, 0.0000405, 0.0000475
	Hispanic	Men	38	0.0000288, 0.0000334, 0.0000387, 0.0000449, 0.000052
	Hispanic	Men	39	0.0000328, 0.0000377, 0.0000433, 0.0000498, 0.0000573
	Hispanic	Men	40	0.0000368, 0.0000422, 0.0000483, 0.0000554, 0.0000635
	Hispanic	Men	41	0.0000407, 0.0000468, 0.0000537, 0.0000616, 0.0000709
	Hispanic	Men	42	0.0000448, 0.0000516, 0.0000595, 0.0000685, 0.000079
	Hispanic	Men	43	0.0000492, 0.0000569, 0.0000657, 0.0000759, 0.0000878
	Hispanic	Men	44	0.0000539, 0.0000625, 0.0000724, 0.0000839, 0.0000974
	Hispanic	Men	45	0.0000592, 0.0000688, 0.0000799, 0.0000928, 0.000108
	Hispanic	Men	46	0.0000651, 0.0000758, 0.0000882, 0.000103, 0.000119
	Hispanic	Men	47	0.0000719, 0.0000838, 0.0000975, 0.000113, 0.000132
	Hispanic	Men	48	0.00008, 0.000093, 0.000108, 0.000125, 0.000145
	Hispanic	Men	49	0.0000898, 0.000104, 0.00012, 0.000138, 0.000159
	Hispanic	Men	50	0.000102, 0.000116, 0.000133, 0.000152, 0.000173
	Hispanic	Men	51	0.000116, 0.000131, 0.000147, 0.000166, 0.000188

Variable	Race/ethnicity	Sex	Age	Distribution
	Hispanic	Men	52	0.00013, 0.000146, 0.000163, 0.000182, 0.000204
	Hispanic	Men	53	0.000144, 0.000161, 0.00018, 0.000202, 0.000226
	Hispanic	Men	54	0.000156, 0.000176, 0.000198, 0.000224, 0.000253
	Hispanic	Men	55	0.000167, 0.000191, 0.000218, 0.000249, 0.000284
	Hispanic	Men	56	0.00018, 0.000207, 0.000238, 0.000274, 0.000315
	Hispanic	Men	57	0.000197, 0.000226, 0.00026, 0.000298, 0.000343
	Hispanic	Men	58	0.000217, 0.000248, 0.000283, 0.000322, 0.000368
	Hispanic	Men	59	0.000242, 0.000273, 0.000308, 0.000346, 0.000391
	Hispanic	Men	60	0.00027, 0.000301, 0.000334, 0.000372, 0.000414
	Hispanic	Men	61	0.000299, 0.00033, 0.000363, 0.0004, 0.000441
	Hispanic	Men	62	0.000328, 0.00036, 0.000395, 0.000433, 0.000476
	Hispanic	Men	63	0.000355, 0.00039, 0.000429, 0.000471, 0.000518
	Hispanic	Men	64	0.000383, 0.000423, 0.000466, 0.000514, 0.000568
	Hispanic	Men	65	0.000413, 0.000458, 0.000507, 0.000562, 0.000624
	Hispanic	Men	66	0.000447, 0.000498, 0.000554, 0.000617, 0.000688
	Hispanic	Men	67	0.000485, 0.000543, 0.000608, 0.00068, 0.000762
	Hispanic	Men	68	0.000529, 0.000596, 0.00067, 0.000753, 0.000848
	Hispanic	Men	69	0.000582, 0.000658, 0.000742, 0.000836, 0.000944
	Hispanic	Men	70	0.000647, 0.000731, 0.000825, 0.00093, 0.00105
	Hispanic	Men	71	0.000725, 0.000817, 0.00092, 0.00104, 0.00117
	Hispanic	Men	72	0.000814, 0.000916, 0.00103, 0.00116, 0.0013
	Hispanic	Men	73	0.000914, 0.00103, 0.00115, 0.00129, 0.00145
	Hispanic	Men	74	0.00102, 0.00115, 0.00129, 0.00145, 0.00163
	Hispanic	Men	75	0.00115, 0.00129, 0.00145, 0.00163, 0.00183
	Hispanic	Men	76	0.00128, 0.00145, 0.00163, 0.00183, 0.00206
	Hispanic	Men	77	0.00143, 0.00162, 0.00183, 0.00206, 0.00233
	Hispanic	Men	78	0.0016, 0.00181, 0.00205, 0.00232, 0.00263
	Hispanic	Men	79	0.00178, 0.00202, 0.0023, 0.00261, 0.00297
	Hispanic	Men	80	0.00196, 0.00225, 0.00257, 0.00294, 0.00336
	Hispanic	Men	81	0.00216, 0.00249, 0.00286, 0.00329, 0.00379
	Hispanic	Men	82	0.00235, 0.00273, 0.00316, 0.00366, 0.00425
	Hispanic	Men	83	0.00254, 0.00297, 0.00347, 0.00405, 0.00473
	Hispanic	Men	84	0.00273, 0.00321, 0.00377, 0.00443, 0.00521
	Hispanic	Women	30	0.00000481, 0.00000696, 0.00001, 0.0000145, 0.000021
	Hispanic	Women	31	0.00000556, 0.0000079, 0.0000112, 0.0000159, 0.0000225
	Hispanic	Women	32	0.0000064, 0.00000898, 0.0000126, 0.0000176, 0.0000247
	Hispanic	Women	33	0.00000734, 0.0000102, 0.0000142, 0.0000196, 0.0000273
	Hispanic	Women	34	0.00000849, 0.0000117, 0.000016, 0.000022, 0.0000303
	Hispanic	Women	35	0.00000994, 0.0000135, 0.0000182, 0.0000246, 0.0000333
	Hispanic	Women	36	0.0000118, 0.0000156, 0.0000206, 0.0000273, 0.0000362
	Hispanic	Women	37	0.000014, 0.0000181, 0.0000234, 0.0000302, 0.0000392
	Hispanic	Women	38	0.0000165, 0.0000209, 0.0000265, 0.0000335, 0.0000425
	Hispanic	Women	39	0.0000193, 0.000024, 0.0000298, 0.0000371, 0.0000462

Variable	Race/ethnicity	Sex	Age	Distribution
	Hispanic	Women	40	0.0000222, 0.0000273, 0.0000335, 0.0000411, 0.0000506
	Hispanic	Women	41	0.000025, 0.0000306, 0.0000374, 0.0000458, 0.0000561
	Hispanic	Women	42	0.0000278, 0.000034, 0.0000417, 0.000051, 0.0000626
	Hispanic	Women	43	0.0000306, 0.0000377, 0.0000463, 0.0000568, 0.00007
	Hispanic	Women	44	0.000034, 0.0000417, 0.0000512, 0.0000628, 0.0000772
	Hispanic	Women	45	0.0000383, 0.0000466, 0.0000566, 0.0000687, 0.0000835
	Hispanic	Women	46	0.0000437, 0.0000522, 0.0000623, 0.0000744, 0.0000889
	Hispanic	Women	47	0.0000497, 0.0000584, 0.0000685, 0.0000803, 0.0000943
	Hispanic	Women	48	0.0000558, 0.0000648, 0.0000751, 0.0000872, 0.000101
	Hispanic	Women	49	0.0000611, 0.000071, 0.0000823, 0.0000955, 0.000111
	Hispanic	Women	50	0.0000656, 0.0000769, 0.0000901, 0.000105, 0.000124
	Hispanic	Women	51	0.0000698, 0.000083, 0.0000985, 0.000117, 0.000139
	Hispanic	Women	52	0.0000742, 0.0000894, 0.000108, 0.00013, 0.000156
	Hispanic	Women	53	0.0000792, 0.0000966, 0.000118, 0.000143, 0.000174
	Hispanic	Women	54	0.0000852, 0.000105, 0.000128, 0.000157, 0.000193
	Hispanic	Women	55	0.0000926, 0.000114, 0.00014, 0.000172, 0.000211
	Hispanic	Women	56	0.000101, 0.000124, 0.000152, 0.000186, 0.000229
	Hispanic	Women	57	0.000112, 0.000136, 0.000166, 0.000202, 0.000246
	Hispanic	Women	58	0.000123, 0.00015, 0.000181, 0.000219, 0.000266
	Hispanic	Women	59	0.000136, 0.000164, 0.000198, 0.000238, 0.000287
	Hispanic	Women	60	0.00015, 0.00018, 0.000216, 0.000259, 0.000312
	Hispanic	Women	61	0.000165, 0.000198, 0.000236, 0.000282, 0.000338
	Hispanic	Women	62	0.000182, 0.000217, 0.000258, 0.000308, 0.000367
	Hispanic	Women	63	0.000199, 0.000237, 0.000282, 0.000336, 0.0004
	Hispanic	Women	64	0.000216, 0.000258, 0.000308, 0.000367, 0.000438
	Hispanic	Women	65	0.000235, 0.000282, 0.000337, 0.000402, 0.000482
	Hispanic	Women	66	0.000257, 0.000309, 0.00037, 0.000443, 0.000532
	Hispanic	Women	67	0.000284, 0.000341, 0.000409, 0.00049, 0.000589
	Hispanic	Women	68	0.000316, 0.00038, 0.000455, 0.000546, 0.000656
	Hispanic	Women	69	0.000356, 0.000427, 0.000511, 0.000611, 0.000733
	Hispanic	Women	70	0.000408, 0.000486, 0.000577, 0.000687, 0.000818
	Hispanic	Women	71	0.000474, 0.000558, 0.000657, 0.000772, 0.000909
	Hispanic	Women	72	0.000558, 0.000647, 0.00075, 0.000868, 0.00101
	Hispanic	Women	73	0.000659, 0.000752, 0.000858, 0.000978, 0.00112
	Hispanic	Women	74	0.000775, 0.000874, 0.000983, 0.00111, 0.00125
	Hispanic	Women	75	0.000902, 0.00101, 0.00113, 0.00126, 0.00141
	Hispanic	Women	76	0.00104, 0.00116, 0.0013, 0.00144, 0.00161
	Hispanic	Women	77	0.0012, 0.00134, 0.00149, 0.00166, 0.00185
	Hispanic	Women	78	0.00138, 0.00154, 0.00171, 0.0019, 0.00212
	Hispanic	Women	79	0.00161, 0.00178, 0.00197, 0.00218, 0.00241
	Hispanic	Women	80	0.00185, 0.00205, 0.00226, 0.00249, 0.00275
	Hispanic	Women	81	0.00209, 0.00232, 0.00258, 0.00285, 0.00317
	Hispanic	Women	82	0.0023, 0.00259, 0.00292, 0.00328, 0.0037

Variable	Race/ethnicity	Sex	Age	Distribution
	Hispanic	Women	83	0.00247, 0.00284, 0.00327, 0.00377, 0.00434
	Hispanic	Women	84	0.00262, 0.00309, 0.00363, 0.00427, 0.00502
	White	Men	30	0.00000703, 0.00000886, 0.0000111, 0.000014, 0.0000177
	White	Men	31	0.00000807, 0.0000101, 0.0000125, 0.0000156, 0.0000195
	White	Men	32	0.00000926, 0.0000114, 0.0000141, 0.0000173, 0.0000214
	White	Men	33	0.0000107, 0.000013, 0.0000158, 0.0000192, 0.0000234
	White	Men	34	0.0000123, 0.0000148, 0.0000177, 0.0000213, 0.0000256
	White	Men	35	0.0000141, 0.0000167, 0.0000199, 0.0000236, 0.000028
	White	Men	36	0.0000161, 0.0000189, 0.0000223, 0.0000262, 0.0000308
	White	Men	37	0.0000183, 0.0000214, 0.000025, 0.0000291, 0.0000341
	White	Men	38	0.0000207, 0.0000241, 0.000028, 0.0000325, 0.0000378
	White	Men	39	0.0000233, 0.000027, 0.0000313, 0.0000363, 0.0000421
	White	Men	40	0.0000261, 0.0000302, 0.0000349, 0.0000403, 0.0000467
	White	Men	41	0.0000292, 0.0000337, 0.0000388, 0.0000447, 0.0000515
	White	Men	42	0.0000328, 0.0000376, 0.000043, 0.0000492, 0.0000564
	White	Men	43	0.0000369, 0.0000419, 0.0000476, 0.0000541, 0.0000615
	White	Men	44	0.0000415, 0.0000468, 0.0000528, 0.0000594, 0.000067
	White	Men	45	0.0000467, 0.0000523, 0.0000586, 0.0000655, 0.0000734
	White	Men	46	0.0000524, 0.0000584, 0.0000651, 0.0000726, 0.000081
	White	Men	47	0.0000585, 0.0000652, 0.0000726, 0.0000808, 0.00009
	White	Men	48	0.0000652, 0.0000727, 0.000081, 0.0000903, 0.000101
	White	Men	49	0.0000725, 0.000081, 0.0000905, 0.000101, 0.000113
	White	Men	50	0.0000806, 0.0000903, 0.000101, 0.000113, 0.000127
	White	Men	51	0.0000902, 0.000101, 0.000113, 0.000126, 0.000141
	White	Men	52	0.000101, 0.000113, 0.000126, 0.00014, 0.000156
	White	Men	53	0.000113, 0.000126, 0.000139, 0.000154, 0.000171
	White	Men	54	0.000126, 0.000139, 0.000154, 0.000171, 0.000189
	White	Men	55	0.000138, 0.000153, 0.00017, 0.000189, 0.00021
	White	Men	56	0.000151, 0.000168, 0.000188, 0.000209, 0.000233
	White	Men	57	0.000164, 0.000184, 0.000206, 0.000231, 0.000259
	White	Men	58	0.000179, 0.000202, 0.000227, 0.000255, 0.000287
	White	Men	59	0.000197, 0.000222, 0.00025, 0.000281, 0.000316
	White	Men	60	0.000218, 0.000245, 0.000274, 0.000308, 0.000345
	White	Men	61	0.000242, 0.00027, 0.000301, 0.000336, 0.000375
	White	Men	62	0.00027, 0.000299, 0.000331, 0.000366, 0.000405
	White	Men	63	0.000302, 0.000331, 0.000363, 0.000397, 0.000435
	White	Men	64	0.000338, 0.000367, 0.000398, 0.000431, 0.000468
	White	Men	65	0.000377, 0.000406, 0.000437, 0.00047, 0.000506
	White	Men	66	0.000421, 0.000451, 0.000482, 0.000516, 0.000553
	White	Men	67	0.000468, 0.000501, 0.000535, 0.000572, 0.000612
	White	Men	68	0.000522, 0.000559, 0.000598, 0.00064, 0.000686
	White	Men	69	0.000582, 0.000626, 0.000672, 0.000722, 0.000776
	White	Men	70	0.000651, 0.000703, 0.000759, 0.000819, 0.000885

Variable	Race/ethnicity	Sex	Age	Distribution
	White	Men	71	0.00073, 0.000792, 0.000859, 0.000931, 0.00101
	White	Men	72	0.000822, 0.000894, 0.000973, 0.00106, 0.00115
	White	Men	73	0.000927, 0.00101, 0.0011, 0.0012, 0.00131
	White	Men	74	0.00105, 0.00114, 0.00125, 0.00136, 0.00149
	White	Men	75	0.00118, 0.00129, 0.00141, 0.00154, 0.00169
	White	Men	76	0.00133, 0.00146, 0.0016, 0.00176, 0.00193
	White	Men	77	0.00151, 0.00166, 0.00183, 0.00201, 0.00221
	White	Men	78	0.0017, 0.00188, 0.00208, 0.0023, 0.00255
	White	Men	79	0.00192, 0.00214, 0.00238, 0.00265, 0.00295
	White	Men	80	0.00216, 0.00243, 0.00272, 0.00306, 0.00344
	White	Men	81	0.00241, 0.00273, 0.0031, 0.00352, 0.00401
	White	Men	82	0.00265, 0.00306, 0.00351, 0.00404, 0.00465
	White	Men	83	0.00289, 0.00338, 0.00394, 0.0046, 0.00537
	White	Men	84	0.00313, 0.0037, 0.00437, 0.00516, 0.00611
	White	Women	30	0.0000061, 0.00000796, 0.0000104, 0.0000135, 0.0000176
	White	Women	31	0.00000697, 0.00000906, 0.0000117, 0.0000152, 0.0000198
	White	Women	32	0.00000804, 0.0000103, 0.0000133, 0.0000171, 0.000022
	White	Women	33	0.0000093, 0.0000118, 0.000015, 0.0000191, 0.0000243
	White	Women	34	0.0000108, 0.0000135, 0.0000169, 0.0000212, 0.0000267
	White	Women	35	0.0000124, 0.0000154, 0.000019, 0.0000235, 0.0000291
	White	Women	36	0.0000143, 0.0000175, 0.0000213, 0.000026, 0.0000317
	White	Women	37	0.0000164, 0.0000198, 0.0000238, 0.0000286, 0.0000345
	White	Women	38	0.0000187, 0.0000222, 0.0000264, 0.0000314, 0.0000375
	White	Women	39	0.0000211, 0.0000249, 0.0000293, 0.0000345, 0.0000407
	White	Women	40	0.0000236, 0.0000276, 0.0000323, 0.0000377, 0.0000442
	White	Women	41	0.0000264, 0.0000306, 0.0000355, 0.0000411, 0.0000478
	White	Women	42	0.0000293, 0.0000338, 0.0000389, 0.0000447, 0.0000515
	White	Women	43	0.0000326, 0.0000373, 0.0000426, 0.0000486, 0.0000556
	White	Women	44	0.000036, 0.000041, 0.0000466, 0.000053, 0.0000603
	White	Women	45	0.0000395, 0.0000449, 0.0000511, 0.0000581, 0.0000662
	White	Women	46	0.0000429, 0.0000491, 0.0000561, 0.0000642, 0.0000735
	White	Women	47	0.0000464, 0.0000535, 0.0000617, 0.0000711, 0.0000821
	White	Women	48	0.0000499, 0.0000582, 0.0000678, 0.000079, 0.0000922
	White	Women	49	0.0000536, 0.0000632, 0.0000745, 0.0000878, 0.000104
	White	Women	50	0.0000578, 0.0000688, 0.0000818, 0.0000972, 0.000116
	White	Women	51	0.0000629, 0.0000751, 0.0000896, 0.000107, 0.000128
	White	Women	52	0.0000693, 0.0000825, 0.0000979, 0.000116, 0.000138
	White	Women	53	0.0000771, 0.0000908, 0.000107, 0.000125, 0.000148
	White	Women	54	0.0000858, 0.0000998, 0.000116, 0.000135, 0.000157
	White	Women	55	0.0000953, 0.00011, 0.000126, 0.000144, 0.000166
	White	Women	56	0.000106, 0.00012, 0.000136, 0.000155, 0.000176
	White	Women	57	0.000117, 0.000132, 0.000148, 0.000166, 0.000187
	White	Women	58	0.00013, 0.000145, 0.000161, 0.000179, 0.000199

Variable	Race/ethnicity	Sex	Age	Distribution
	White	Women	59	0.000146, 0.00016, 0.000176, 0.000194, 0.000213
	White	Women	60	0.000163, 0.000178, 0.000194, 0.000212, 0.000231
	White	Women	61	0.000181, 0.000197, 0.000214, 0.000233, 0.000254
	White	Women	62	0.0002, 0.000218, 0.000237, 0.000258, 0.00028
	White	Women	63	0.000222, 0.000242, 0.000263, 0.000286, 0.000311
	White	Women	64	0.000246, 0.000268, 0.000291, 0.000317, 0.000345
	White	Women	65	0.000274, 0.000298, 0.000324, 0.000352, 0.000383
	White	Women	66	0.000305, 0.000332, 0.000361, 0.000392, 0.000427
	White	Women	67	0.000342, 0.000373, 0.000405, 0.00044, 0.000479
	White	Women	68	0.000388, 0.000421, 0.000457, 0.000496, 0.000539
	White	Women	69	0.000444, 0.000481, 0.00052, 0.000563, 0.00061
	White	Women	70	0.000511, 0.000552, 0.000596, 0.000643, 0.000695
	White	Women	71	0.000589, 0.000636, 0.000686, 0.00074, 0.000799
	White	Women	72	0.000677, 0.000733, 0.000793, 0.000857, 0.000928
	White	Women	73	0.000777, 0.000844, 0.000916, 0.000995, 0.00108
	White	Women	74	0.000894, 0.000973, 0.00106, 0.00115, 0.00126
	White	Women	75	0.00103, 0.00112, 0.00122, 0.00133, 0.00145
	White	Women	76	0.00119, 0.0013, 0.00141, 0.00154, 0.00168
	White	Women	77	0.00136, 0.00149, 0.00164, 0.00179, 0.00197
	White	Women	78	0.00156, 0.00172, 0.0019, 0.0021, 0.00232
	White	Women	79	0.00178, 0.00198, 0.00221, 0.00246, 0.00274
	White	Women	80	0.00203, 0.00228, 0.00256, 0.00288, 0.00323
	White	Women	81	0.00233, 0.00263, 0.00297, 0.00335, 0.00379
	White	Women	82	0.00266, 0.00302, 0.00342, 0.00387, 0.0044
	White	Women	83	0.00301, 0.00343, 0.0039, 0.00443, 0.00505
	White	Women	84	0.00336, 0.00385, 0.00439, 0.00501, 0.00573
Stroke mortality rates for 2018 (0.01, 0.2, 0.5, 0.8. 0.99 percentiles of the empirical distribution produced during forecasting)				
	Black	Men	30	0.0000185, 0.0000239, 0.0000308, 0.0000397, 0.0000512
	Black	Men	31	0.0000224, 0.0000284, 0.0000359, 0.0000453, 0.0000574
	Black	Men	32	0.0000266, 0.0000333, 0.0000417, 0.0000522, 0.0000655
	Black	Men	33	0.0000308, 0.0000386, 0.0000483, 0.0000603, 0.0000756
	Black	Men	34	0.0000354, 0.0000444, 0.0000554, 0.0000693, 0.0000868
	Black	Men	35	0.0000405, 0.0000507, 0.0000632, 0.0000788, 0.0000985
	Black	Men	36	0.0000464, 0.0000577, 0.0000715, 0.0000887, 0.00011
	Black	Men	37	0.0000532, 0.0000654, 0.0000803, 0.0000986, 0.000121
	Black	Men	38	0.000061, 0.000074, 0.0000896, 0.000108, 0.000132
	Black	Men	39	0.0000695, 0.0000832, 0.0000993, 0.000119, 0.000142
	Black	Men	40	0.0000782, 0.0000927, 0.00011, 0.00013, 0.000154
	Black	Men	41	0.0000875, 0.000103, 0.000121, 0.000142, 0.000168
	Black	Men	42	0.0000983, 0.000115, 0.000134, 0.000156, 0.000183
	Black	Men	43	0.00011, 0.000128, 0.000149, 0.000173, 0.000201
	Black	Men	44	0.000121, 0.000141, 0.000165, 0.000193, 0.000226
	Black	Men	45	0.000132, 0.000156, 0.000184, 0.000217, 0.000257

Variable	Race/ethnicity	Sex	Age	Distribution
	Black	Men	46	0.000145, 0.000172, 0.000204, 0.000243, 0.000288
	Black	Men	47	0.00016, 0.00019, 0.000227, 0.00027, 0.000322
	Black	Men	48	0.000175, 0.00021, 0.000252, 0.000303, 0.000364
	Black	Men	49	0.000193, 0.000233, 0.000282, 0.00034, 0.000412
	Black	Men	50	0.000217, 0.000262, 0.000316, 0.00038, 0.000459
	Black	Men	51	0.000251, 0.000299, 0.000355, 0.000421, 0.0005
	Black	Men	52	0.000294, 0.000342, 0.000398, 0.000463, 0.000539
	Black	Men	53	0.000338, 0.000388, 0.000445, 0.000509, 0.000585
	Black	Men	54	0.000381, 0.000433, 0.000493, 0.00056, 0.000638
	Black	Men	55	0.000422, 0.000478, 0.000541, 0.000613, 0.000695
	Black	Men	56	0.000461, 0.000522, 0.00059, 0.000668, 0.000757
	Black	Men	57	0.000501, 0.000567, 0.000641, 0.000724, 0.000819
	Black	Men	58	0.000548, 0.000617, 0.000694, 0.000779, 0.000877
	Black	Men	59	0.000601, 0.000672, 0.00075, 0.000837, 0.000935
	Black	Men	60	0.000649, 0.000725, 0.00081, 0.000904, 0.00101
	Black	Men	61	0.000689, 0.000776, 0.000872, 0.000979, 0.0011
	Black	Men	62	0.000735, 0.000829, 0.000935, 0.00105, 0.00119
	Black	Men	63	0.000794, 0.000891, 0.000999, 0.00112, 0.00126
	Black	Men	64	0.000863, 0.000959, 0.00106, 0.00118, 0.00131
	Black	Men	65	0.000925, 0.00103, 0.00114, 0.00126, 0.0014
	Black	Men	66	0.000984, 0.00109, 0.00122, 0.00135, 0.00151
	Black	Men	67	0.00106, 0.00118, 0.00131, 0.00146, 0.00163
	Black	Men	68	0.00115, 0.00128, 0.00142, 0.00158, 0.00175
	Black	Men	69	0.00127, 0.0014, 0.00155, 0.00171, 0.00188
	Black	Men	70	0.00139, 0.00153, 0.00169, 0.00186, 0.00204
	Black	Men	71	0.00152, 0.00167, 0.00184, 0.00202, 0.00223
	Black	Men	72	0.00165, 0.00182, 0.002, 0.0022, 0.00242
	Black	Men	73	0.00178, 0.00196, 0.00217, 0.0024, 0.00265
	Black	Men	74	0.0019, 0.00211, 0.00235, 0.00262, 0.00292
	Black	Men	75	0.00202, 0.00227, 0.00255, 0.00287, 0.00322
	Black	Men	76	0.00218, 0.00246, 0.00278, 0.00313, 0.00354
	Black	Men	77	0.00237, 0.00268, 0.00303, 0.00341, 0.00386
	Black	Men	78	0.00259, 0.00292, 0.0033, 0.00372, 0.00421
	Black	Men	79	0.00281, 0.00318, 0.0036, 0.00407, 0.0046
	Black	Men	80	0.00303, 0.00344, 0.00391, 0.00443, 0.00504
	Black	Men	81	0.00325, 0.0037, 0.00422, 0.00481, 0.00549
	Black	Men	82	0.00347, 0.00397, 0.00453, 0.00517, 0.00592
	Black	Men	83	0.00369, 0.00423, 0.00483, 0.00553, 0.00633
	Black	Men	84	0.0039, 0.00447, 0.00512, 0.00587, 0.00673
	Black	Women	30	0.0000128, 0.0000178, 0.0000247, 0.0000342, 0.0000475
	Black	Women	31	0.0000144, 0.0000202, 0.0000283, 0.0000395, 0.0000554
	Black	Women	32	0.0000161, 0.0000228, 0.0000323, 0.0000456, 0.0000648
	Black	Women	33	0.0000178, 0.0000257, 0.0000368, 0.0000527, 0.0000758

Variable	Race/ethnicity	Sex	Age	Distribution
	Black	Women	34	0.0000198, 0.0000288, 0.0000418, 0.0000607, 0.0000884
	Black	Women	35	0.0000221, 0.0000324, 0.0000474, 0.0000693, 0.000102
	Black	Women	36	0.0000252, 0.0000368, 0.0000535, 0.0000779, 0.000114
	Black	Women	37	0.0000293, 0.0000421, 0.0000602, 0.0000861, 0.000124
	Black	Women	38	0.0000347, 0.0000485, 0.0000675, 0.0000939, 0.000131
	Black	Women	39	0.000041, 0.0000556, 0.0000753, 0.000102, 0.000138
	Black	Women	40	0.0000477, 0.0000633, 0.0000837, 0.000111, 0.000147
	Black	Women	41	0.000055, 0.0000715, 0.0000927, 0.00012, 0.000156
	Black	Women	42	0.0000629, 0.0000803, 0.000102, 0.00013, 0.000167
	Black	Women	43	0.0000714, 0.0000898, 0.000113, 0.000142, 0.000178
	Black	Women	44	0.00008, 0.0000998, 0.000124, 0.000155, 0.000193
	Black	Women	45	0.0000889, 0.00011, 0.000137, 0.00017, 0.000211
	Black	Women	46	0.0000995, 0.000123, 0.000151, 0.000186, 0.000229
	Black	Women	47	0.000113, 0.000137, 0.000166, 0.000202, 0.000245
	Black	Women	48	0.000129, 0.000154, 0.000183, 0.000218, 0.00026
	Black	Women	49	0.000145, 0.000171, 0.000201, 0.000236, 0.000277
	Black	Women	50	0.000161, 0.000188, 0.000219, 0.000255, 0.000298
	Black	Women	51	0.000176, 0.000205, 0.000237, 0.000275, 0.000319
	Black	Women	52	0.000193, 0.000223, 0.000256, 0.000295, 0.00034
	Black	Women	53	0.000211, 0.000242, 0.000276, 0.000316, 0.000362
	Black	Women	54	0.000228, 0.000261, 0.000298, 0.00034, 0.000388
	Black	Women	55	0.000245, 0.000281, 0.000321, 0.000367, 0.000421
	Black	Women	56	0.000265, 0.000304, 0.000348, 0.000399, 0.000457
	Black	Women	57	0.00029, 0.000332, 0.000378, 0.000432, 0.000493
	Black	Women	58	0.000322, 0.000365, 0.000413, 0.000467, 0.000529
	Black	Women	59	0.000356, 0.000401, 0.00045, 0.000505, 0.000568
	Black	Women	60	0.00039, 0.000437, 0.000489, 0.000548, 0.000615
	Black	Women	61	0.000419, 0.000472, 0.00053, 0.000596, 0.000671
	Black	Women	62	0.000447, 0.000506, 0.000572, 0.000646, 0.000731
	Black	Women	63	0.000476, 0.000541, 0.000613, 0.000696, 0.000791
	Black	Women	64	0.00051, 0.000579, 0.000657, 0.000745, 0.000846
	Black	Women	65	0.000551, 0.000623, 0.000704, 0.000795, 0.000899
	Black	Women	66	0.0006, 0.000674, 0.000757, 0.000849, 0.000954
	Black	Women	67	0.00066, 0.000736, 0.000819, 0.000911, 0.00102
	Black	Women	68	0.000733, 0.000809, 0.000893, 0.000986, 0.00109
	Black	Women	69	0.000818, 0.000897, 0.000983, 0.00108, 0.00118
	Black	Women	70	0.000913, 0.000997, 0.00109, 0.00119, 0.0013
	Black	Women	71	0.00102, 0.00111, 0.00121, 0.00132, 0.00145
	Black	Women	72	0.00113, 0.00124, 0.00136, 0.00148, 0.00163
	Black	Women	73	0.00126, 0.00139, 0.00152, 0.00167, 0.00183
	Black	Women	74	0.00142, 0.00156, 0.00171, 0.00187, 0.00206
	Black	Women	75	0.00159, 0.00175, 0.00192, 0.00211, 0.00232
	Black	Women	76	0.00176, 0.00195, 0.00216, 0.00239, 0.00264

Variable	Race/ethnicity	Sex	Age	Distribution
	Black	Women	77	0.00194, 0.00217, 0.00243, 0.00272, 0.00304
	Black	Women	78	0.00213, 0.00242, 0.00273, 0.00309, 0.00351
	Black	Women	79	0.00235, 0.00269, 0.00307, 0.00351, 0.00401
	Black	Women	80	0.00261, 0.003, 0.00344, 0.00395, 0.00454
	Black	Women	81	0.00291, 0.00334, 0.00384, 0.00442, 0.00508
	Black	Women	82	0.00321, 0.0037, 0.00426, 0.00491, 0.00566
	Black	Women	83	0.0035, 0.00405, 0.00469, 0.00542, 0.00627
	Black	Women	84	0.00377, 0.00439, 0.00511, 0.00594, 0.00692
	Hispanic	Men	30	0.00000844, 0.0000111, 0.0000145, 0.000019, 0.000025
	Hispanic	Men	31	0.00001, 0.0000128, 0.0000164, 0.000021, 0.000027
	Hispanic	Men	32	0.0000118, 0.0000148, 0.0000186, 0.0000233, 0.0000293
	Hispanic	Men	33	0.0000138, 0.000017, 0.000021, 0.000026, 0.0000322
	Hispanic	Men	34	0.0000159, 0.0000194, 0.0000238, 0.0000291, 0.0000357
	Hispanic	Men	35	0.0000182, 0.0000221, 0.0000268, 0.0000326, 0.0000396
	Hispanic	Men	36	0.0000209, 0.0000251, 0.0000302, 0.0000363, 0.0000438
	Hispanic	Men	37	0.000024, 0.0000286, 0.000034, 0.0000404, 0.000048
	Hispanic	Men	38	0.0000276, 0.0000324, 0.0000381, 0.0000447, 0.0000526
	Hispanic	Men	39	0.0000313, 0.0000365, 0.0000425, 0.0000495, 0.0000578
	Hispanic	Men	40	0.0000351, 0.0000408, 0.0000474, 0.000055, 0.0000639
	Hispanic	Men	41	0.000039, 0.0000453, 0.0000526, 0.000061, 0.000071
	Hispanic	Men	42	0.0000431, 0.0000501, 0.0000582, 0.0000676, 0.0000787
	Hispanic	Men	43	0.0000475, 0.0000553, 0.0000643, 0.0000748, 0.0000871
	Hispanic	Men	44	0.0000523, 0.000061, 0.000071, 0.0000827, 0.0000964
	Hispanic	Men	45	0.0000575, 0.0000672, 0.0000784, 0.0000915, 0.000107
	Hispanic	Men	46	0.0000635, 0.0000742, 0.0000867, 0.000101, 0.000118
	Hispanic	Men	47	0.0000703, 0.0000822, 0.000096, 0.000112, 0.000131
	Hispanic	Men	48	0.0000784, 0.0000914, 0.000106, 0.000124, 0.000144
	Hispanic	Men	49	0.0000881, 0.000102, 0.000118, 0.000137, 0.000158
	Hispanic	Men	50	0.0000998, 0.000114, 0.000131, 0.00015, 0.000172
	Hispanic	Men	51	0.000113, 0.000129, 0.000146, 0.000165, 0.000187
	Hispanic	Men	52	0.000128, 0.000144, 0.000161, 0.000181, 0.000204
	Hispanic	Men	53	0.000141, 0.000158, 0.000178, 0.0002, 0.000226
	Hispanic	Men	54	0.000152, 0.000173, 0.000196, 0.000223, 0.000254
	Hispanic	Men	55	0.000162, 0.000187, 0.000215, 0.000247, 0.000285
	Hispanic	Men	56	0.000175, 0.000203, 0.000235, 0.000272, 0.000316
	Hispanic	Men	57	0.000191, 0.000221, 0.000256, 0.000297, 0.000344
	Hispanic	Men	58	0.00021, 0.000242, 0.000279, 0.000321, 0.000369
	Hispanic	Men	59	0.000234, 0.000266, 0.000303, 0.000345, 0.000392
	Hispanic	Men	60	0.000261, 0.000293, 0.000329, 0.000369, 0.000415
	Hispanic	Men	61	0.000289, 0.000321, 0.000357, 0.000397, 0.000442
	Hispanic	Men	62	0.000316, 0.00035, 0.000387, 0.000428, 0.000474
	Hispanic	Men	63	0.000342, 0.000379, 0.000419, 0.000464, 0.000514
	Hispanic	Men	64	0.000368, 0.000409, 0.000455, 0.000505, 0.000561

Variable	Race/ethnicity	Sex	Age	Distribution
	Hispanic	Men	65	0.000397, 0.000443, 0.000494, 0.000551, 0.000615
	Hispanic	Men	66	0.000429, 0.000481, 0.000538, 0.000603, 0.000676
	Hispanic	Men	67	0.000465, 0.000524, 0.00059, 0.000663, 0.000747
	Hispanic	Men	68	0.000508, 0.000574, 0.000649, 0.000734, 0.00083
	Hispanic	Men	69	0.000559, 0.000634, 0.000719, 0.000814, 0.000924
	Hispanic	Men	70	0.000621, 0.000705, 0.000799, 0.000906, 0.00103
	Hispanic	Men	71	0.000696, 0.000788, 0.000892, 0.00101, 0.00114
	Hispanic	Men	72	0.000781, 0.000883, 0.000997, 0.00113, 0.00127
	Hispanic	Men	73	0.000876, 0.00099, 0.00112, 0.00126, 0.00142
	Hispanic	Men	74	0.000981, 0.00111, 0.00125, 0.00141, 0.0016
	Hispanic	Men	75	0.0011, 0.00124, 0.0014, 0.00158, 0.00179
	Hispanic	Men	76	0.00123, 0.00139, 0.00157, 0.00178, 0.00202
	Hispanic	Men	77	0.00137, 0.00156, 0.00177, 0.002, 0.00227
	Hispanic	Men	78	0.00153, 0.00174, 0.00198, 0.00225, 0.00257
	Hispanic	Men	79	0.0017, 0.00194, 0.00222, 0.00254, 0.0029
	Hispanic	Men	80	0.00187, 0.00216, 0.00248, 0.00285, 0.00328
	Hispanic	Men	81	0.00206, 0.00238, 0.00276, 0.00319, 0.0037
	Hispanic	Men	82	0.00224, 0.00261, 0.00305, 0.00355, 0.00415
	Hispanic	Men	83	0.00241, 0.00284, 0.00334, 0.00393, 0.00462
	Hispanic	Men	84	0.00259, 0.00307, 0.00363, 0.0043, 0.0051
	Hispanic	Women	30	0.00000468, 0.00000685, 0.00000998, 0.0000146, 0.0000213
	Hispanic	Women	31	0.00000545, 0.00000781, 0.0000112, 0.0000159, 0.0000228
	Hispanic	Women	32	0.0000063, 0.0000089, 0.0000125, 0.0000177, 0.000025
	Hispanic	Women	33	0.00000726, 0.0000102, 0.0000142, 0.0000198, 0.0000277
	Hispanic	Women	34	0.0000084, 0.0000116, 0.000016, 0.0000222, 0.0000307
	Hispanic	Women	35	0.00000982, 0.0000134, 0.0000182, 0.0000247, 0.0000337
	Hispanic	Women	36	0.0000116, 0.0000155, 0.0000206, 0.0000274, 0.0000366
	Hispanic	Women	37	0.0000137, 0.0000179, 0.0000233, 0.0000303, 0.0000396
	Hispanic	Women	38	0.0000161, 0.0000206, 0.0000263, 0.0000335, 0.0000428
	Hispanic	Women	39	0.0000187, 0.0000235, 0.0000295, 0.0000369, 0.0000464
	Hispanic	Women	40	0.0000214, 0.0000266, 0.000033, 0.0000408, 0.0000507
	Hispanic	Women	41	0.0000241, 0.0000298, 0.0000367, 0.0000452, 0.0000558
	Hispanic	Women	42	0.0000267, 0.000033, 0.0000407, 0.0000502, 0.000062
	Hispanic	Women	43	0.0000294, 0.0000364, 0.000045, 0.0000557, 0.0000689
	Hispanic	Women	44	0.0000326, 0.0000403, 0.0000498, 0.0000614, 0.0000759
	Hispanic	Women	45	0.0000368, 0.000045, 0.0000549, 0.000067, 0.000082
	Hispanic	Women	46	0.000042, 0.0000505, 0.0000605, 0.0000726, 0.0000872
	Hispanic	Women	47	0.0000479, 0.0000565, 0.0000666, 0.0000784, 0.0000924
	Hispanic	Women	48	0.0000539, 0.0000629, 0.0000731, 0.0000851, 0.0000992
	Hispanic	Women	49	0.0000593, 0.000069, 0.0000803, 0.0000933, 0.000109
	Hispanic	Women	50	0.0000639, 0.0000751, 0.0000881, 0.000103, 0.000121
	Hispanic	Women	51	0.0000681, 0.0000812, 0.0000965, 0.000115, 0.000137
	Hispanic	Women	52	0.0000725, 0.0000877, 0.000106, 0.000128, 0.000154

Variable	Race/ethnicity	Sex	Age	Distribution
	Hispanic	Women	53	0.0000775, 0.0000948, 0.000116, 0.000141, 0.000173
	Hispanic	Women	54	0.0000834, 0.000103, 0.000127, 0.000156, 0.000192
	Hispanic	Women	55	0.0000907, 0.000112, 0.000138, 0.00017, 0.00021
	Hispanic	Women	56	0.0000995, 0.000122, 0.000151, 0.000185, 0.000228
	Hispanic	Women	57	0.00011, 0.000134, 0.000164, 0.000201, 0.000246
	Hispanic	Women	58	0.000121, 0.000147, 0.000179, 0.000217, 0.000265
	Hispanic	Women	59	0.000133, 0.000162, 0.000195, 0.000236, 0.000286
	Hispanic	Women	60	0.000147, 0.000177, 0.000214, 0.000257, 0.000311
	Hispanic	Women	61	0.000162, 0.000194, 0.000233, 0.00028, 0.000337
	Hispanic	Women	62	0.000177, 0.000213, 0.000255, 0.000305, 0.000366
	Hispanic	Women	63	0.000194, 0.000232, 0.000278, 0.000332, 0.000399
	Hispanic	Women	64	0.00021, 0.000253, 0.000303, 0.000363, 0.000436
	Hispanic	Women	65	0.000229, 0.000275, 0.000331, 0.000398, 0.000479
	Hispanic	Women	66	0.00025, 0.000301, 0.000363, 0.000437, 0.000528
	Hispanic	Women	67	0.000275, 0.000332, 0.000401, 0.000484, 0.000585
	Hispanic	Women	68	0.000306, 0.00037, 0.000446, 0.000538, 0.000651
	Hispanic	Women	69	0.000345, 0.000416, 0.0005, 0.000602, 0.000727
	Hispanic	Women	70	0.000395, 0.000473, 0.000565, 0.000676, 0.00081
	Hispanic	Women	71	0.000459, 0.000543, 0.000642, 0.000759, 0.000898
	Hispanic	Women	72	0.00054, 0.000629, 0.000732, 0.000852, 0.000993
	Hispanic	Women	73	0.000637, 0.000731, 0.000837, 0.000958, 0.0011
	Hispanic	Women	74	0.000749, 0.000847, 0.000958, 0.00108, 0.00122
	Hispanic	Women	75	0.000871, 0.000978, 0.0011, 0.00123, 0.00138
	Hispanic	Women	76	0.001, 0.00112, 0.00126, 0.00141, 0.00158
	Hispanic	Women	77	0.00115, 0.00129, 0.00145, 0.00162, 0.00182
	Hispanic	Women	78	0.00133, 0.00149, 0.00166, 0.00186, 0.00208
	Hispanic	Women	79	0.00154, 0.00172, 0.00191, 0.00212, 0.00236
	Hispanic	Women	80	0.00178, 0.00197, 0.00219, 0.00243, 0.00269
	Hispanic	Women	81	0.002, 0.00224, 0.00249, 0.00278, 0.0031
	Hispanic	Women	82	0.0022, 0.00249, 0.00282, 0.00319, 0.00362
	Hispanic	Women	83	0.00235, 0.00273, 0.00316, 0.00366, 0.00424
	Hispanic	Women	84	0.0025, 0.00296, 0.0035, 0.00414, 0.0049
	White	Men	30	0.00000682, 0.00000871, 0.0000111, 0.0000141, 0.0000181
	White	Men	31	0.00000785, 0.00000991, 0.0000125, 0.0000157, 0.0000198
	White	Men	32	0.00000904, 0.0000113, 0.000014, 0.0000174, 0.0000217
	White	Men	33	0.0000104, 0.0000128, 0.0000157, 0.0000193, 0.0000238
	White	Men	34	0.000012, 0.0000146, 0.0000176, 0.0000214, 0.000026
	White	Men	35	0.0000137, 0.0000165, 0.0000198, 0.0000237, 0.0000284
	White	Men	36	0.0000157, 0.0000186, 0.0000221, 0.0000263, 0.0000313
	White	Men	37	0.0000178, 0.000021, 0.0000248, 0.0000292, 0.0000345
	White	Men	38	0.00002, 0.0000236, 0.0000277, 0.0000325, 0.0000383
	White	Men	39	0.0000224, 0.0000263, 0.0000309, 0.0000362, 0.0000425
	White	Men	40	0.0000251, 0.0000294, 0.0000343, 0.0000401, 0.000047

Variable	Race/ethnicity	Sex	Age	Distribution
	White	Men	41	0.0000281, 0.0000327, 0.0000381, 0.0000443, 0.0000516
	White	Men	42	0.0000316, 0.0000365, 0.0000421, 0.0000486, 0.0000562
	White	Men	43	0.0000355, 0.0000407, 0.0000465, 0.0000532, 0.0000609
	White	Men	44	0.00004, 0.0000454, 0.0000515, 0.0000584, 0.0000663
	White	Men	45	0.0000449, 0.0000506, 0.0000571, 0.0000643, 0.0000725
	White	Men	46	0.0000503, 0.0000565, 0.0000634, 0.0000711, 0.0000798
	White	Men	47	0.0000562, 0.000063, 0.0000706, 0.000079, 0.0000886
	White	Men	48	0.0000626, 0.0000702, 0.0000787, 0.0000883, 0.0000991
	White	Men	49	0.0000694, 0.0000782, 0.000088, 0.000099, 0.000111
	White	Men	50	0.000077, 0.0000871, 0.0000983, 0.000111, 0.000125
	White	Men	51	0.000086, 0.0000972, 0.00011, 0.000124, 0.00014
	White	Men	52	0.0000966, 0.000109, 0.000122, 0.000137, 0.000155
	White	Men	53	0.000108, 0.000121, 0.000136, 0.000152, 0.00017
	White	Men	54	0.00012, 0.000135, 0.00015, 0.000168, 0.000188
	White	Men	55	0.000132, 0.000148, 0.000166, 0.000186, 0.000209
	White	Men	56	0.000145, 0.000163, 0.000183, 0.000206, 0.000232
	White	Men	57	0.000157, 0.000178, 0.000201, 0.000228, 0.000258
	White	Men	58	0.000172, 0.000195, 0.000222, 0.000251, 0.000286
	White	Men	59	0.000189, 0.000214, 0.000244, 0.000277, 0.000315
	White	Men	60	0.000208, 0.000236, 0.000268, 0.000303, 0.000344
	White	Men	61	0.000232, 0.000261, 0.000294, 0.000331, 0.000373
	White	Men	62	0.000259, 0.000289, 0.000323, 0.00036, 0.000402
	White	Men	63	0.00029, 0.00032, 0.000354, 0.00039, 0.000431
	White	Men	64	0.000325, 0.000355, 0.000387, 0.000423, 0.000462
	White	Men	65	0.000363, 0.000393, 0.000426, 0.000461, 0.000499
	White	Men	66	0.000405, 0.000436, 0.000469, 0.000505, 0.000545
	White	Men	67	0.00045, 0.000485, 0.000521, 0.00056, 0.000602
	White	Men	68	0.000501, 0.00054, 0.000582, 0.000627, 0.000675
	White	Men	69	0.000559, 0.000605, 0.000654, 0.000707, 0.000764
	White	Men	70	0.000626, 0.00068, 0.000738, 0.000801, 0.000871
	White	Men	71	0.000702, 0.000766, 0.000835, 0.00091, 0.000994
	White	Men	72	0.00079, 0.000864, 0.000946, 0.00103, 0.00113
	White	Men	73	0.000889, 0.000976, 0.00107, 0.00117, 0.00129
	White	Men	74	0.001, 0.0011, 0.00121, 0.00133, 0.00147
	White	Men	75	0.00113, 0.00124, 0.00137, 0.00151, 0.00167
	White	Men	76	0.00127, 0.00141, 0.00156, 0.00172, 0.00191
	White	Men	77	0.00143, 0.00159, 0.00177, 0.00197, 0.00219
	White	Men	78	0.00162, 0.00181, 0.00202, 0.00226, 0.00253
	White	Men	79	0.00182, 0.00205, 0.00231, 0.00261, 0.00294
	White	Men	80	0.00204, 0.00233, 0.00265, 0.00301, 0.00343
	White	Men	81	0.00228, 0.00262, 0.00302, 0.00347, 0.004
	White	Men	82	0.00252, 0.00294, 0.00342, 0.00397, 0.00463
	White	Men	83	0.00276, 0.00325, 0.00383, 0.00451, 0.00532

Variable	Race/ethnicity	Sex	Age	Distribution
	White	Men	84	0.00298, 0.00356, 0.00425, 0.00506, 0.00604
	White	Women	30	0.00000595, 0.00000785, 0.0000103, 0.0000136, 0.0000179
	White	Women	31	0.00000683, 0.00000895, 0.0000117, 0.0000153, 0.0000201
	White	Women	32	0.00000789, 0.0000102, 0.0000133, 0.0000172, 0.0000223
	White	Women	33	0.00000915, 0.0000117, 0.000015, 0.0000192, 0.0000246
	White	Women	34	0.0000106, 0.0000134, 0.0000169, 0.0000213, 0.000027
	White	Women	35	0.0000122, 0.0000152, 0.000019, 0.0000236, 0.0000295
	White	Women	36	0.000014, 0.0000173, 0.0000212, 0.0000261, 0.0000321
	White	Women	37	0.000016, 0.0000195, 0.0000236, 0.0000287, 0.0000349
	White	Women	38	0.0000182, 0.0000219, 0.0000262, 0.0000315, 0.0000379
	White	Women	39	0.0000205, 0.0000244, 0.000029, 0.0000345, 0.0000411
	White	Women	40	0.0000229, 0.0000271, 0.0000319, 0.0000376, 0.0000444
	White	Women	41	0.0000256, 0.0000299, 0.000035, 0.0000409, 0.0000478
	White	Women	42	0.0000285, 0.000033, 0.0000382, 0.0000442, 0.0000513
	White	Women	43	0.0000316, 0.0000364, 0.0000417, 0.0000479, 0.000055
	White	Women	44	0.0000349, 0.0000399, 0.0000456, 0.0000521, 0.0000596
	White	Women	45	0.0000382, 0.0000437, 0.0000499, 0.0000571, 0.0000653
	White	Women	46	0.0000416, 0.0000477, 0.0000548, 0.0000629, 0.0000722
	White	Women	47	0.0000449, 0.000052, 0.0000602, 0.0000696, 0.0000805
	White	Women	48	0.0000484, 0.0000566, 0.0000661, 0.0000772, 0.0000903
	White	Women	49	0.0000521, 0.0000615, 0.0000726, 0.0000857, 0.000101
	White	Women	50	0.0000561, 0.000067, 0.0000798, 0.000095, 0.000113
	White	Women	51	0.0000611, 0.0000732, 0.0000875, 0.000105, 0.000125
	White	Women	52	0.0000673, 0.0000803, 0.0000957, 0.000114, 0.000136
	White	Women	53	0.0000748, 0.0000884, 0.000104, 0.000123, 0.000146
	White	Women	54	0.0000833, 0.0000973, 0.000114, 0.000133, 0.000155
	White	Women	55	0.0000925, 0.000107, 0.000123, 0.000142, 0.000164
	White	Women	56	0.000103, 0.000117, 0.000134, 0.000153, 0.000174
	White	Women	57	0.000114, 0.000129, 0.000145, 0.000164, 0.000185
	White	Women	58	0.000127, 0.000142, 0.000158, 0.000177, 0.000197
	White	Women	59	0.000141, 0.000157, 0.000173, 0.000191, 0.000212
	White	Women	60	0.000158, 0.000173, 0.00019, 0.000209, 0.00023
	White	Women	61	0.000175, 0.000192, 0.00021, 0.00023, 0.000251
	White	Women	62	0.000194, 0.000212, 0.000232, 0.000254, 0.000278
	White	Women	63	0.000215, 0.000235, 0.000257, 0.000281, 0.000307
	White	Women	64	0.000238, 0.00026, 0.000285, 0.000311, 0.00034
	White	Women	65	0.000264, 0.000289, 0.000316, 0.000345, 0.000377
	White	Women	66	0.000295, 0.000322, 0.000352, 0.000384, 0.00042
	White	Women	67	0.00033, 0.000361, 0.000394, 0.00043, 0.00047
	White	Women	68	0.000374, 0.000408, 0.000445, 0.000485, 0.00053
	White	Women	69	0.000427, 0.000465, 0.000506, 0.000551, 0.0006
	White	Women	70	0.000492, 0.000534, 0.00058, 0.00063, 0.000684
	White	Women	71	0.000567, 0.000616, 0.000668, 0.000725, 0.000787

Variable	Race/ethnicity	Sex	Age	Distribution
	White	Women	72	0.000651, 0.000709, 0.000771, 0.000839, 0.000914
	White	Women	73	0.000747, 0.000817, 0.000891, 0.000973, 0.00106
	White	Women	74	0.000859, 0.000941, 0.00103, 0.00113, 0.00123
	White	Women	75	0.00099, 0.00109, 0.00119, 0.0013, 0.00143
	White	Women	76	0.00114, 0.00125, 0.00137, 0.0015, 0.00165
	White	Women	77	0.00131, 0.00144, 0.00159, 0.00175, 0.00192
	White	Women	78	0.0015, 0.00166, 0.00184, 0.00204, 0.00227
	White	Women	79	0.00171, 0.00191, 0.00214, 0.00239, 0.00268
	White	Women	80	0.00195, 0.0022, 0.00249, 0.0028, 0.00317
	White	Women	81	0.00224, 0.00254, 0.00288, 0.00327, 0.00371
	White	Women	82	0.00255, 0.00291, 0.00332, 0.00378, 0.00431
	White	Women	83	0.00289, 0.00331, 0.00378, 0.00433, 0.00495
	White	Women	84	0.00323, 0.00371, 0.00426, 0.00489, 0.00562
Stroke mortality rates for 2019 (0.01, 0.2, 0.5, 0.8. 0.99 percentiles of the empirical distribution produced during forecasting)				
	Black	Men	30	0.000018, 0.0000235, 0.0000306, 0.0000399, 0.0000522
	Black	Men	31	0.0000219, 0.000028, 0.0000357, 0.0000456, 0.0000583
	Black	Men	32	0.000026, 0.0000329, 0.0000415, 0.0000524, 0.0000664
	Black	Men	33	0.0000302, 0.0000381, 0.000048, 0.0000605, 0.0000764
	Black	Men	34	0.0000347, 0.0000438, 0.0000551, 0.0000694, 0.0000876
	Black	Men	35	0.0000397, 0.00005, 0.0000628, 0.0000789, 0.0000994
	Black	Men	36	0.0000453, 0.0000568, 0.0000709, 0.0000887, 0.000111
	Black	Men	37	0.0000518, 0.0000643, 0.0000796, 0.0000985, 0.000122
	Black	Men	38	0.0000593, 0.0000726, 0.0000886, 0.000108, 0.000132
	Black	Men	39	0.0000674, 0.0000814, 0.0000981, 0.000118, 0.000143
	Black	Men	40	0.0000757, 0.0000906, 0.000108, 0.000129, 0.000154
	Black	Men	41	0.0000848, 0.000101, 0.000119, 0.000141, 0.000167
	Black	Men	42	0.0000954, 0.000112, 0.000132, 0.000154, 0.000181
	Black	Men	43	0.000107, 0.000125, 0.000146, 0.00017, 0.000199
	Black	Men	44	0.000117, 0.000138, 0.000162, 0.00019, 0.000223
	Black	Men	45	0.000128, 0.000152, 0.00018, 0.000213, 0.000253
	Black	Men	46	0.000141, 0.000168, 0.0002, 0.000238, 0.000284
	Black	Men	47	0.000155, 0.000186, 0.000222, 0.000265, 0.000318
	Black	Men	48	0.00017, 0.000206, 0.000248, 0.000298, 0.000359
	Black	Men	49	0.000188, 0.000228, 0.000277, 0.000336, 0.000408
	Black	Men	50	0.000212, 0.000257, 0.000311, 0.000376, 0.000455
	Black	Men	51	0.000246, 0.000293, 0.000349, 0.000416, 0.000497
	Black	Men	52	0.000287, 0.000336, 0.000393, 0.000458, 0.000536
	Black	Men	53	0.000331, 0.000382, 0.000439, 0.000505, 0.000581
	Black	Men	54	0.000373, 0.000427, 0.000487, 0.000556, 0.000635
	Black	Men	55	0.000413, 0.000471, 0.000535, 0.000608, 0.000693
	Black	Men	56	0.000452, 0.000514, 0.000584, 0.000663, 0.000755
	Black	Men	57	0.000491, 0.000558, 0.000633, 0.000719, 0.000817
	Black	Men	58	0.000537, 0.000607, 0.000685, 0.000774, 0.000875

Variable	Race/ethnicity	Sex	Age	Distribution
	Black	Men	59	0.000587, 0.00066, 0.00074, 0.00083, 0.000932
	Black	Men	60	0.000633, 0.000711, 0.000798, 0.000895, 0.00101
	Black	Men	61	0.000671, 0.000759, 0.000857, 0.000969, 0.0011
	Black	Men	62	0.000714, 0.00081, 0.000918, 0.00104, 0.00118
	Black	Men	63	0.000771, 0.00087, 0.000979, 0.0011, 0.00124
	Black	Men	64	0.000837, 0.000935, 0.00104, 0.00116, 0.0013
	Black	Men	65	0.000897, 0.000999, 0.00111, 0.00124, 0.00138
	Black	Men	66	0.000954, 0.00107, 0.00119, 0.00133, 0.00149
	Black	Men	67	0.00102, 0.00115, 0.00128, 0.00143, 0.0016
	Black	Men	68	0.00112, 0.00125, 0.00139, 0.00155, 0.00172
	Black	Men	69	0.00123, 0.00136, 0.00151, 0.00167, 0.00186
	Black	Men	70	0.00134, 0.00149, 0.00164, 0.00182, 0.00201
	Black	Men	71	0.00146, 0.00162, 0.00179, 0.00198, 0.00219
	Black	Men	72	0.00159, 0.00176, 0.00195, 0.00215, 0.00238
	Black	Men	73	0.00171, 0.0019, 0.00211, 0.00234, 0.0026
	Black	Men	74	0.00183, 0.00205, 0.00229, 0.00256, 0.00286
	Black	Men	75	0.00195, 0.0022, 0.00248, 0.0028, 0.00316
	Black	Men	76	0.0021, 0.00238, 0.0027, 0.00306, 0.00347
	Black	Men	77	0.00229, 0.0026, 0.00294, 0.00333, 0.00378
	Black	Men	78	0.0025, 0.00283, 0.00321, 0.00364, 0.00413
	Black	Men	79	0.00271, 0.00308, 0.0035, 0.00397, 0.00452
	Black	Men	80	0.00291, 0.00333, 0.0038, 0.00434, 0.00495
	Black	Men	81	0.00313, 0.00358, 0.00411, 0.0047, 0.00539
	Black	Men	82	0.00334, 0.00384, 0.00441, 0.00506, 0.00582
	Black	Men	83	0.00355, 0.00409, 0.0047, 0.00541, 0.00623
	Black	Men	84	0.00375, 0.00432, 0.00498, 0.00575, 0.00663
	Black	Women	30	0.0000125, 0.0000175, 0.0000245, 0.0000343, 0.0000482
	Black	Women	31	0.0000141, 0.0000199, 0.0000281, 0.0000396, 0.000056
	Black	Women	32	0.0000158, 0.0000226, 0.0000321, 0.0000457, 0.0000654
	Black	Women	33	0.0000175, 0.0000254, 0.0000366, 0.0000528, 0.0000764
	Black	Women	34	0.0000195, 0.0000285, 0.0000416, 0.0000608, 0.000089
	Black	Women	35	0.0000218, 0.0000321, 0.0000472, 0.0000693, 0.000102
	Black	Women	36	0.0000247, 0.0000363, 0.0000532, 0.0000778, 0.000114
	Black	Women	37	0.0000287, 0.0000415, 0.0000597, 0.0000859, 0.000124
	Black	Women	38	0.0000339, 0.0000476, 0.0000668, 0.0000936, 0.000132
	Black	Women	39	0.0000398, 0.0000545, 0.0000743, 0.000101, 0.000139
	Black	Women	40	0.0000461, 0.0000617, 0.0000824, 0.00011, 0.000147
	Black	Women	41	0.0000528, 0.0000694, 0.000091, 0.000119, 0.000157
	Black	Women	42	0.0000602, 0.0000778, 0.0001, 0.000129, 0.000167
	Black	Women	43	0.0000683, 0.0000868, 0.00011, 0.00014, 0.000178
	Black	Women	44	0.0000764, 0.0000963, 0.000121, 0.000152, 0.000192
	Black	Women	45	0.000085, 0.000107, 0.000133, 0.000167, 0.000209
	Black	Women	46	0.0000951, 0.000118, 0.000147, 0.000182, 0.000227

Variable	Race/ethnicity	Sex	Age	Distribution
	Black	Women	47	0.000108, 0.000132, 0.000162, 0.000198, 0.000242
	Black	Women	48	0.000123, 0.000148, 0.000178, 0.000214, 0.000257
	Black	Women	49	0.000139, 0.000165, 0.000195, 0.000231, 0.000274
	Black	Women	50	0.000154, 0.000181, 0.000213, 0.000251, 0.000295
	Black	Women	51	0.000169, 0.000198, 0.000232, 0.000271, 0.000317
	Black	Women	52	0.000186, 0.000216, 0.00025, 0.000291, 0.000338
	Black	Women	53	0.000203, 0.000234, 0.00027, 0.000311, 0.00036
	Black	Women	54	0.00022, 0.000253, 0.000291, 0.000335, 0.000386
	Black	Women	55	0.000236, 0.000273, 0.000315, 0.000363, 0.000419
	Black	Women	56	0.000256, 0.000296, 0.000341, 0.000394, 0.000456
	Black	Women	57	0.000281, 0.000323, 0.000372, 0.000427, 0.000492
	Black	Women	58	0.000312, 0.000356, 0.000405, 0.000462, 0.000527
	Black	Women	59	0.000346, 0.000391, 0.000442, 0.000499, 0.000565
	Black	Women	60	0.000378, 0.000427, 0.000481, 0.000541, 0.000611
	Black	Women	61	0.000407, 0.00046, 0.00052, 0.000588, 0.000665
	Black	Women	62	0.000434, 0.000493, 0.00056, 0.000636, 0.000724
	Black	Women	63	0.000462, 0.000527, 0.0006, 0.000684, 0.000781
	Black	Women	64	0.000494, 0.000564, 0.000642, 0.000731, 0.000834
	Black	Women	65	0.000533, 0.000606, 0.000687, 0.000779, 0.000885
	Black	Women	66	0.000581, 0.000655, 0.000738, 0.000831, 0.000938
	Black	Women	67	0.000638, 0.000714, 0.000798, 0.000892, 0.000999
	Black	Women	68	0.000707, 0.000785, 0.00087, 0.000965, 0.00107
	Black	Women	69	0.000788, 0.000869, 0.000957, 0.00105, 0.00116
	Black	Women	70	0.00088, 0.000966, 0.00106, 0.00116, 0.00128
	Black	Women	71	0.000979, 0.00108, 0.00118, 0.0013, 0.00142
	Black	Women	72	0.00109, 0.0012, 0.00132, 0.00145, 0.0016
	Black	Women	73	0.00122, 0.00134, 0.00148, 0.00163, 0.0018
	Black	Women	74	0.00136, 0.00151, 0.00166, 0.00183, 0.00202
	Black	Women	75	0.00153, 0.00169, 0.00187, 0.00206, 0.00228
	Black	Women	76	0.0017, 0.00189, 0.0021, 0.00233, 0.00259
	Black	Women	77	0.00187, 0.00211, 0.00236, 0.00265, 0.00298
	Black	Women	78	0.00206, 0.00234, 0.00266, 0.00302, 0.00344
	Black	Women	79	0.00227, 0.00261, 0.00299, 0.00343, 0.00394
	Black	Women	80	0.00252, 0.00291, 0.00336, 0.00387, 0.00446
	Black	Women	81	0.0028, 0.00324, 0.00374, 0.00432, 0.005
	Black	Women	82	0.00309, 0.00358, 0.00415, 0.0048, 0.00557
	Black	Women	83	0.00337, 0.00392, 0.00456, 0.0053, 0.00617
	Black	Women	84	0.00363, 0.00425, 0.00497, 0.00581, 0.00681
	Hispanic	Men	30	0.00000824, 0.0000109, 0.0000145, 0.0000192, 0.0000255
	Hispanic	Men	31	0.00000981, 0.0000127, 0.0000164, 0.0000212, 0.0000274
	Hispanic	Men	32	0.0000116, 0.0000147, 0.0000186, 0.0000235, 0.0000298
	Hispanic	Men	33	0.0000135, 0.0000169, 0.000021, 0.0000262, 0.0000327
	Hispanic	Men	34	0.0000155, 0.0000192, 0.0000237, 0.0000293, 0.0000363

Variable	Race/ethnicity	Sex	Age	Distribution
	Hispanic	Men	35	0.0000178, 0.0000218, 0.0000268, 0.0000328, 0.0000403
	Hispanic	Men	36	0.0000204, 0.0000248, 0.0000301, 0.0000366, 0.0000446
	Hispanic	Men	37	0.0000234, 0.0000281, 0.0000338, 0.0000406, 0.0000489
	Hispanic	Men	38	0.0000268, 0.0000318, 0.0000378, 0.0000449, 0.0000535
	Hispanic	Men	39	0.0000303, 0.0000358, 0.0000422, 0.0000497, 0.0000586
	Hispanic	Men	40	0.000034, 0.0000399, 0.0000469, 0.000055, 0.0000646
	Hispanic	Men	41	0.0000377, 0.0000442, 0.0000519, 0.0000608, 0.0000713
	Hispanic	Men	42	0.0000417, 0.0000489, 0.0000572, 0.000067, 0.0000785
	Hispanic	Men	43	0.000046, 0.0000539, 0.000063, 0.0000737, 0.0000863
	Hispanic	Men	44	0.0000506, 0.0000593, 0.0000694, 0.0000812, 0.0000951
	Hispanic	Men	45	0.0000557, 0.0000653, 0.0000764, 0.0000895, 0.000105
	Hispanic	Men	46	0.0000614, 0.000072, 0.0000843, 0.0000988, 0.000116
	Hispanic	Men	47	0.000068, 0.0000797, 0.0000933, 0.000109, 0.000128
	Hispanic	Men	48	0.0000758, 0.0000885, 0.000103, 0.000121, 0.000141
	Hispanic	Men	49	0.0000852, 0.0000989, 0.000115, 0.000133, 0.000155
	Hispanic	Men	50	0.0000966, 0.000111, 0.000128, 0.000147, 0.000168
	Hispanic	Men	51	0.00011, 0.000125, 0.000142, 0.000161, 0.000183
	Hispanic	Men	52	0.000124, 0.00014, 0.000158, 0.000177, 0.0002
	Hispanic	Men	53	0.000137, 0.000155, 0.000174, 0.000197, 0.000222
	Hispanic	Men	54	0.000147, 0.000169, 0.000192, 0.00022, 0.000251
	Hispanic	Men	55	0.000158, 0.000183, 0.000211, 0.000244, 0.000283
	Hispanic	Men	56	0.00017, 0.000198, 0.000231, 0.000269, 0.000315
	Hispanic	Men	57	0.000185, 0.000216, 0.000252, 0.000294, 0.000343
	Hispanic	Men	58	0.000204, 0.000237, 0.000274, 0.000318, 0.000368
	Hispanic	Men	59	0.000227, 0.00026, 0.000298, 0.000341, 0.000391
	Hispanic	Men	60	0.000253, 0.000286, 0.000323, 0.000366, 0.000414
	Hispanic	Men	61	0.000279, 0.000313, 0.000351, 0.000393, 0.00044
	Hispanic	Men	62	0.000306, 0.000341, 0.00038, 0.000423, 0.000472
	Hispanic	Men	63	0.000331, 0.000369, 0.000411, 0.000458, 0.00051
	Hispanic	Men	64	0.000356, 0.000399, 0.000445, 0.000497, 0.000556
	Hispanic	Men	65	0.000384, 0.000431, 0.000483, 0.000541, 0.000607
	Hispanic	Men	66	0.000415, 0.000468, 0.000526, 0.000592, 0.000667
	Hispanic	Men	67	0.000451, 0.00051, 0.000576, 0.000651, 0.000737
	Hispanic	Men	68	0.000492, 0.000559, 0.000634, 0.00072, 0.000818
	Hispanic	Men	69	0.000541, 0.000617, 0.000702, 0.000799, 0.000911
	Hispanic	Men	70	0.000602, 0.000686, 0.000781, 0.000889, 0.00101
	Hispanic	Men	71	0.000674, 0.000767, 0.000871, 0.00099, 0.00113
	Hispanic	Men	72	0.000757, 0.000859, 0.000974, 0.0011, 0.00125
	Hispanic	Men	73	0.000848, 0.000962, 0.00109, 0.00124, 0.0014
	Hispanic	Men	74	0.00095, 0.00108, 0.00122, 0.00138, 0.00157
	Hispanic	Men	75	0.00106, 0.00121, 0.00137, 0.00155, 0.00176
	Hispanic	Men	76	0.00119, 0.00135, 0.00154, 0.00174, 0.00198
	Hispanic	Men	77	0.00133, 0.00151, 0.00172, 0.00196, 0.00223

Variable	Race/ethnicity	Sex	Age	Distribution
	Hispanic	Men	78	0.00148, 0.00169, 0.00193, 0.00221, 0.00253
	Hispanic	Men	79	0.00164, 0.00189, 0.00217, 0.00249, 0.00286
	Hispanic	Men	80	0.00182, 0.0021, 0.00242, 0.0028, 0.00324
	Hispanic	Men	81	0.00199, 0.00232, 0.0027, 0.00314, 0.00365
	Hispanic	Men	82	0.00217, 0.00254, 0.00298, 0.00349, 0.0041
	Hispanic	Men	83	0.00234, 0.00277, 0.00327, 0.00386, 0.00458
	Hispanic	Men	84	0.0025, 0.00298, 0.00355, 0.00423, 0.00505
	Hispanic	Women	30	0.00000458, 0.00000675, 0.00000992, 0.0000146, 0.0000215
	Hispanic	Women	31	0.00000535, 0.00000772, 0.0000111, 0.000016, 0.000023
	Hispanic	Women	32	0.0000062, 0.00000882, 0.0000125, 0.0000177, 0.0000252
	Hispanic	Women	33	0.00000716, 0.0000101, 0.0000141, 0.0000198, 0.0000279
	Hispanic	Women	34	0.00000829, 0.0000115, 0.000016, 0.0000222, 0.0000309
	Hispanic	Women	35	0.00000969, 0.0000133, 0.0000181, 0.0000248, 0.0000339
	Hispanic	Women	36	0.0000114, 0.0000153, 0.0000205, 0.0000274, 0.0000368
	Hispanic	Women	37	0.0000135, 0.0000177, 0.0000231, 0.0000303, 0.0000398
	Hispanic	Women	38	0.0000158, 0.0000203, 0.000026, 0.0000334, 0.000043
	Hispanic	Women	39	0.0000182, 0.0000231, 0.0000291, 0.0000368, 0.0000465
	Hispanic	Women	40	0.0000208, 0.000026, 0.0000325, 0.0000405, 0.0000506
	Hispanic	Women	41	0.0000234, 0.0000291, 0.000036, 0.0000447, 0.0000555
	Hispanic	Women	42	0.0000259, 0.0000322, 0.0000399, 0.0000494, 0.0000613
	Hispanic	Women	43	0.0000286, 0.0000355, 0.000044, 0.0000546, 0.0000679
	Hispanic	Women	44	0.0000317, 0.0000393, 0.0000486, 0.0000601, 0.0000746
	Hispanic	Women	45	0.0000357, 0.0000438, 0.0000536, 0.0000656, 0.0000805
	Hispanic	Women	46	0.0000408, 0.0000491, 0.000059, 0.000071, 0.0000855
	Hispanic	Women	47	0.0000466, 0.0000551, 0.000065, 0.0000767, 0.0000906
	Hispanic	Women	48	0.0000525, 0.0000613, 0.0000714, 0.0000833, 0.0000973
	Hispanic	Women	49	0.0000578, 0.0000674, 0.0000785, 0.0000915, 0.000107
	Hispanic	Women	50	0.0000623, 0.0000734, 0.0000862, 0.000101, 0.000119
	Hispanic	Women	51	0.0000666, 0.0000795, 0.0000947, 0.000113, 0.000135
	Hispanic	Women	52	0.000071, 0.000086, 0.000104, 0.000126, 0.000152
	Hispanic	Women	53	0.000076, 0.0000931, 0.000114, 0.000139, 0.000171
	Hispanic	Women	54	0.0000819, 0.000101, 0.000125, 0.000154, 0.00019
	Hispanic	Women	55	0.0000891, 0.00011, 0.000136, 0.000168, 0.000208
	Hispanic	Women	56	0.0000977, 0.000121, 0.000149, 0.000183, 0.000226
	Hispanic	Women	57	0.000108, 0.000132, 0.000162, 0.000198, 0.000243
	Hispanic	Women	58	0.000119, 0.000145, 0.000177, 0.000215, 0.000263
	Hispanic	Women	59	0.000131, 0.000159, 0.000193, 0.000234, 0.000284
	Hispanic	Women	60	0.000144, 0.000174, 0.000211, 0.000255, 0.000308
	Hispanic	Women	61	0.000158, 0.000191, 0.00023, 0.000277, 0.000334
	Hispanic	Women	62	0.000173, 0.000209, 0.000251, 0.000301, 0.000362
	Hispanic	Women	63	0.000189, 0.000227, 0.000273, 0.000328, 0.000394
	Hispanic	Women	64	0.000205, 0.000247, 0.000297, 0.000357, 0.00043
	Hispanic	Women	65	0.000223, 0.000269, 0.000324, 0.000391, 0.000472

Variable	Race/ethnicity	Sex	Age	Distribution
	Hispanic	Women	66	0.000243, 0.000294, 0.000355, 0.000429, 0.000519
	Hispanic	Women	67	0.000267, 0.000324, 0.000392, 0.000474, 0.000575
	Hispanic	Women	68	0.000297, 0.00036, 0.000436, 0.000528, 0.00064
	Hispanic	Women	69	0.000335, 0.000405, 0.000489, 0.00059, 0.000714
	Hispanic	Women	70	0.000383, 0.000461, 0.000552, 0.000662, 0.000796
	Hispanic	Women	71	0.000446, 0.000529, 0.000627, 0.000743, 0.000882
	Hispanic	Women	72	0.000523, 0.000612, 0.000714, 0.000834, 0.000975
	Hispanic	Women	73	0.000617, 0.00071, 0.000816, 0.000937, 0.00108
	Hispanic	Women	74	0.000725, 0.000823, 0.000933, 0.00106, 0.0012
	Hispanic	Women	75	0.000842, 0.000949, 0.00107, 0.0012, 0.00136
	Hispanic	Women	76	0.000969, 0.00109, 0.00123, 0.00138, 0.00155
	Hispanic	Women	77	0.00111, 0.00125, 0.00141, 0.00158, 0.00178
	Hispanic	Women	78	0.00128, 0.00144, 0.00162, 0.00181, 0.00204
	Hispanic	Women	79	0.00149, 0.00166, 0.00186, 0.00208, 0.00232
	Hispanic	Women	80	0.00171, 0.00191, 0.00213, 0.00237, 0.00264
	Hispanic	Women	81	0.00193, 0.00216, 0.00242, 0.00271, 0.00304
	Hispanic	Women	82	0.00211, 0.00241, 0.00274, 0.00312, 0.00355
	Hispanic	Women	83	0.00227, 0.00264, 0.00307, 0.00356, 0.00415
	Hispanic	Women	84	0.00241, 0.00286, 0.0034, 0.00403, 0.0048
	White	Men	30	0.00000661, 0.00000855, 0.000011, 0.0000142, 0.0000184
	White	Men	31	0.00000765, 0.00000975, 0.0000124, 0.0000158, 0.0000201
	White	Men	32	0.00000883, 0.0000111, 0.0000139, 0.0000175, 0.000022
	White	Men	33	0.0000102, 0.0000126, 0.0000157, 0.0000194, 0.0000241
	White	Men	34	0.0000117, 0.0000144, 0.0000175, 0.0000215, 0.0000263
	White	Men	35	0.0000134, 0.0000162, 0.0000196, 0.0000238, 0.0000288
	White	Men	36	0.0000153, 0.0000183, 0.000022, 0.0000263, 0.0000316
	White	Men	37	0.0000173, 0.0000206, 0.0000245, 0.0000293, 0.0000349
	White	Men	38	0.0000194, 0.0000231, 0.0000274, 0.0000325, 0.0000387
	White	Men	39	0.0000217, 0.0000257, 0.0000305, 0.0000361, 0.0000428
	White	Men	40	0.0000242, 0.0000286, 0.0000338, 0.0000399, 0.0000472
	White	Men	41	0.0000271, 0.0000318, 0.0000374, 0.0000439, 0.0000516
	White	Men	42	0.0000304, 0.0000355, 0.0000412, 0.000048, 0.0000559
	White	Men	43	0.0000343, 0.0000395, 0.0000455, 0.0000524, 0.0000604
	White	Men	44	0.0000385, 0.000044, 0.0000503, 0.0000574, 0.0000655
	White	Men	45	0.0000433, 0.0000491, 0.0000556, 0.0000631, 0.0000716
	White	Men	46	0.0000485, 0.0000548, 0.0000618, 0.0000697, 0.0000787
	White	Men	47	0.0000542, 0.0000611, 0.0000688, 0.0000774, 0.0000872
	White	Men	48	0.0000603, 0.0000681, 0.0000767, 0.0000865, 0.0000976
	White	Men	49	0.0000668, 0.0000757, 0.0000857, 0.0000971, 0.00011
	White	Men	50	0.000074, 0.0000843, 0.0000959, 0.000109, 0.000124
	White	Men	51	0.0000826, 0.0000941, 0.000107, 0.000122, 0.000139
	White	Men	52	0.0000928, 0.000105, 0.00012, 0.000136, 0.000154
	White	Men	53	0.000104, 0.000118, 0.000133, 0.00015, 0.00017

Variable	Race/ethnicity	Sex	Age	Distribution
	White	Men	54	0.000116, 0.000131, 0.000147, 0.000166, 0.000187
	White	Men	55	0.000128, 0.000144, 0.000163, 0.000184, 0.000208
	White	Men	56	0.00014, 0.000158, 0.000179, 0.000203, 0.000231
	White	Men	57	0.000152, 0.000173, 0.000197, 0.000225, 0.000257
	White	Men	58	0.000166, 0.00019, 0.000217, 0.000248, 0.000285
	White	Men	59	0.000182, 0.000208, 0.000239, 0.000274, 0.000314
	White	Men	60	0.000201, 0.000229, 0.000262, 0.0003, 0.000343
	White	Men	61	0.000223, 0.000254, 0.000288, 0.000327, 0.000372
	White	Men	62	0.000249, 0.000281, 0.000316, 0.000355, 0.000399
	White	Men	63	0.00028, 0.000311, 0.000346, 0.000384, 0.000427
	White	Men	64	0.000313, 0.000345, 0.000379, 0.000416, 0.000457
	White	Men	65	0.00035, 0.000382, 0.000415, 0.000452, 0.000492
	White	Men	66	0.000391, 0.000423, 0.000458, 0.000496, 0.000537
	White	Men	67	0.000435, 0.00047, 0.000508, 0.000549, 0.000594
	White	Men	68	0.000483, 0.000524, 0.000567, 0.000614, 0.000666
	White	Men	69	0.000539, 0.000586, 0.000637, 0.000693, 0.000754
	White	Men	70	0.000603, 0.000659, 0.000719, 0.000785, 0.000858
	White	Men	71	0.000677, 0.000743, 0.000814, 0.000892, 0.000979
	White	Men	72	0.000761, 0.000838, 0.000922, 0.00101, 0.00112
	White	Men	73	0.000855, 0.000945, 0.00104, 0.00115, 0.00127
	White	Men	74	0.000961, 0.00107, 0.00118, 0.00131, 0.00145
	White	Men	75	0.00108, 0.0012, 0.00134, 0.00149, 0.00165
	White	Men	76	0.00122, 0.00136, 0.00152, 0.00169, 0.00189
	White	Men	77	0.00137, 0.00154, 0.00173, 0.00194, 0.00217
	White	Men	78	0.00154, 0.00175, 0.00197, 0.00223, 0.00252
	White	Men	79	0.00174, 0.00198, 0.00225, 0.00257, 0.00293
	White	Men	80	0.00194, 0.00224, 0.00258, 0.00297, 0.00342
	White	Men	81	0.00217, 0.00253, 0.00294, 0.00342, 0.00399
	White	Men	82	0.0024, 0.00283, 0.00333, 0.00391, 0.0046
	White	Men	83	0.00264, 0.00314, 0.00373, 0.00443, 0.00528
	White	Men	84	0.00286, 0.00344, 0.00413, 0.00497, 0.00598
	White	Women	30	0.0000058, 0.00000773, 0.0000103, 0.0000137, 0.0000182
	White	Women	31	0.00000668, 0.00000883, 0.0000116, 0.0000154, 0.0000203
	White	Women	32	0.00000774, 0.0000101, 0.0000132, 0.0000172, 0.0000225
	White	Women	33	0.00000899, 0.0000116, 0.0000149, 0.0000192, 0.0000248
	White	Women	34	0.0000104, 0.0000133, 0.0000168, 0.0000214, 0.0000272
	White	Women	35	0.000012, 0.0000151, 0.0000189, 0.0000237, 0.0000298
	White	Women	36	0.0000137, 0.000017, 0.0000211, 0.0000262, 0.0000325
	White	Women	37	0.0000156, 0.0000192, 0.0000235, 0.0000288, 0.0000353
	White	Women	38	0.0000177, 0.0000215, 0.000026, 0.0000315, 0.0000383
	White	Women	39	0.0000199, 0.0000239, 0.0000287, 0.0000344, 0.0000414
	White	Women	40	0.0000222, 0.0000265, 0.0000315, 0.0000375, 0.0000446
	White	Women	41	0.0000248, 0.0000292, 0.0000344, 0.0000405, 0.0000478

Variable	Race/ethnicity	Sex	Age	Distribution
	White	Women	42	0.0000277, 0.0000323, 0.0000375, 0.0000437, 0.000051
	White	Women	43	0.0000308, 0.0000355, 0.0000409, 0.0000472, 0.0000544
	White	Women	44	0.0000339, 0.0000389, 0.0000447, 0.0000512, 0.0000588
	White	Women	45	0.0000371, 0.0000426, 0.0000488, 0.000056, 0.0000643
	White	Women	46	0.0000403, 0.0000465, 0.0000535, 0.0000616, 0.000071
	White	Women	47	0.0000437, 0.0000507, 0.0000588, 0.0000681, 0.0000791
	White	Women	48	0.0000471, 0.0000552, 0.0000646, 0.0000755, 0.0000885
	White	Women	49	0.0000507, 0.00006, 0.000071, 0.0000839, 0.0000994
	White	Women	50	0.0000547, 0.0000654, 0.000078, 0.0000931, 0.000111
	White	Women	51	0.0000596, 0.0000715, 0.0000857, 0.000103, 0.000123
	White	Women	52	0.0000657, 0.0000786, 0.0000938, 0.000112, 0.000134
	White	Women	53	0.0000731, 0.0000866, 0.000103, 0.000121, 0.000144
	White	Women	54	0.0000814, 0.0000954, 0.000112, 0.000131, 0.000153
	White	Women	55	0.0000904, 0.000105, 0.000121, 0.00014, 0.000163
	White	Women	56	0.0001, 0.000115, 0.000132, 0.000151, 0.000173
	White	Women	57	0.000111, 0.000126, 0.000143, 0.000162, 0.000184
	White	Women	58	0.000124, 0.000139, 0.000156, 0.000174, 0.000196
	White	Women	59	0.000138, 0.000153, 0.00017, 0.000189, 0.00021
	White	Women	60	0.000154, 0.00017, 0.000187, 0.000206, 0.000228
	White	Women	61	0.00017, 0.000188, 0.000206, 0.000227, 0.00025
	White	Women	62	0.000189, 0.000207, 0.000228, 0.00025, 0.000275
	White	Women	63	0.000208, 0.000229, 0.000252, 0.000277, 0.000304
	White	Women	64	0.000231, 0.000254, 0.000278, 0.000306, 0.000336
	White	Women	65	0.000256, 0.000281, 0.000309, 0.000338, 0.000372
	White	Women	66	0.000285, 0.000313, 0.000343, 0.000376, 0.000413
	White	Women	67	0.000319, 0.000351, 0.000384, 0.000422, 0.000463
	White	Women	68	0.000361, 0.000396, 0.000434, 0.000476, 0.000522
	White	Women	69	0.000412, 0.000451, 0.000494, 0.00054, 0.000591
	White	Women	70	0.000474, 0.000518, 0.000566, 0.000617, 0.000675
	White	Women	71	0.000547, 0.000597, 0.000651, 0.000711, 0.000776
	White	Women	72	0.000628, 0.000688, 0.000752, 0.000822, 0.0009
	White	Women	73	0.000721, 0.000791, 0.000868, 0.000953, 0.00105
	White	Women	74	0.000828, 0.000912, 0.001, 0.0011, 0.00121
	White	Women	75	0.000955, 0.00105, 0.00116, 0.00127, 0.0014
	White	Women	76	0.0011, 0.00121, 0.00134, 0.00147, 0.00162
	White	Women	77	0.00126, 0.0014, 0.00154, 0.00171, 0.00189
	White	Women	78	0.00144, 0.00161, 0.00179, 0.00199, 0.00222
	White	Women	79	0.00164, 0.00185, 0.00208, 0.00234, 0.00263
	White	Women	80	0.00188, 0.00213, 0.00242, 0.00274, 0.00311
	White	Women	81	0.00215, 0.00246, 0.0028, 0.00319, 0.00364
	White	Women	82	0.00246, 0.00282, 0.00322, 0.00369, 0.00423
	White	Women	83	0.00278, 0.0032, 0.00368, 0.00423, 0.00486
	White	Women	84	0.00311, 0.00359, 0.00414, 0.00478, 0.00552

Variable	Race/ethnicity	Sex	Age	Distribution
Stroke mortality rates for 2020 (0.01, 0.2, 0.5, 0.8. 0.99 percentiles of the empirical distribution produced during forecasting)				
	Black	Men	30	0.0000175, 0.0000232, 0.0000305, 0.0000402, 0.0000531
	Black	Men	31	0.0000214, 0.0000276, 0.0000356, 0.0000458, 0.0000592
	Black	Men	32	0.0000254, 0.0000325, 0.0000414, 0.0000527, 0.0000673
	Black	Men	33	0.0000296, 0.0000377, 0.0000478, 0.0000607, 0.0000772
	Black	Men	34	0.000034, 0.0000433, 0.0000549, 0.0000696, 0.0000884
	Black	Men	35	0.0000389, 0.0000493, 0.0000624, 0.000079, 0.0001
	Black	Men	36	0.0000444, 0.000056, 0.0000705, 0.0000887, 0.000112
	Black	Men	37	0.0000506, 0.0000633, 0.0000789, 0.0000984, 0.000123
	Black	Men	38	0.0000577, 0.0000712, 0.0000877, 0.000108, 0.000133
	Black	Men	39	0.0000654, 0.0000797, 0.0000969, 0.000118, 0.000143
	Black	Men	40	0.0000734, 0.0000885, 0.000107, 0.000128, 0.000155
	Black	Men	41	0.0000822, 0.0000983, 0.000117, 0.00014, 0.000167
	Black	Men	42	0.0000926, 0.000109, 0.000129, 0.000152, 0.00018
	Black	Men	43	0.000104, 0.000122, 0.000143, 0.000167, 0.000196
	Black	Men	44	0.000114, 0.000134, 0.000158, 0.000186, 0.00022
	Black	Men	45	0.000124, 0.000148, 0.000176, 0.000209, 0.000249
	Black	Men	46	0.000137, 0.000164, 0.000195, 0.000233, 0.000279
	Black	Men	47	0.000151, 0.000181, 0.000217, 0.00026, 0.000312
	Black	Men	48	0.000166, 0.000201, 0.000242, 0.000293, 0.000354
	Black	Men	49	0.000183, 0.000223, 0.000271, 0.00033, 0.000402
	Black	Men	50	0.000207, 0.000251, 0.000305, 0.00037, 0.00045
	Black	Men	51	0.00024, 0.000287, 0.000343, 0.00041, 0.000491
	Black	Men	52	0.000281, 0.00033, 0.000386, 0.000452, 0.000531
	Black	Men	53	0.000324, 0.000375, 0.000432, 0.000499, 0.000576
	Black	Men	54	0.000366, 0.000419, 0.00048, 0.000549, 0.00063
	Black	Men	55	0.000405, 0.000463, 0.000528, 0.000602, 0.000688
	Black	Men	56	0.000442, 0.000505, 0.000576, 0.000657, 0.00075
	Black	Men	57	0.000481, 0.000549, 0.000625, 0.000712, 0.000812
	Black	Men	58	0.000525, 0.000596, 0.000676, 0.000766, 0.00087
	Black	Men	59	0.000574, 0.000647, 0.000729, 0.000822, 0.000927
	Black	Men	60	0.000617, 0.000697, 0.000785, 0.000885, 0.000999
	Black	Men	61	0.000654, 0.000743, 0.000843, 0.000957, 0.00109
	Black	Men	62	0.000695, 0.000792, 0.000902, 0.00103, 0.00117
	Black	Men	63	0.00075, 0.000849, 0.00096, 0.00109, 0.00123
	Black	Men	64	0.000813, 0.000912, 0.00102, 0.00114, 0.00128
	Black	Men	65	0.000871, 0.000974, 0.00109, 0.00122, 0.00136
	Black	Men	66	0.000925, 0.00104, 0.00116, 0.0013, 0.00146
	Black	Men	67	0.000992, 0.00111, 0.00125, 0.0014, 0.00158
	Black	Men	68	0.00108, 0.00121, 0.00135, 0.00152, 0.0017
	Black	Men	69	0.00119, 0.00132, 0.00147, 0.00164, 0.00183
	Black	Men	70	0.0013, 0.00144, 0.0016, 0.00178, 0.00198
	Black	Men	71	0.00141, 0.00157, 0.00174, 0.00194, 0.00215

Variable	Race/ethnicity	Sex	Age	Distribution
	Black	Men	72	0.00153, 0.00171, 0.00189, 0.0021, 0.00234
	Black	Men	73	0.00165, 0.00184, 0.00205, 0.00229, 0.00256
	Black	Men	74	0.00177, 0.00198, 0.00223, 0.0025, 0.00281
	Black	Men	75	0.00189, 0.00214, 0.00242, 0.00273, 0.0031
	Black	Men	76	0.00203, 0.00231, 0.00263, 0.00299, 0.0034
	Black	Men	77	0.00221, 0.00252, 0.00286, 0.00326, 0.00371
	Black	Men	78	0.00241, 0.00274, 0.00312, 0.00355, 0.00405
	Black	Men	79	0.00261, 0.00298, 0.0034, 0.00388, 0.00444
	Black	Men	80	0.00281, 0.00323, 0.0037, 0.00424, 0.00486
	Black	Men	81	0.00301, 0.00347, 0.004, 0.0046, 0.0053
	Black	Men	82	0.00322, 0.00372, 0.00429, 0.00495, 0.00572
	Black	Men	83	0.00342, 0.00396, 0.00458, 0.00529, 0.00613
	Black	Men	84	0.00361, 0.00419, 0.00485, 0.00562, 0.00653
	Black	Women	30	0.0000122, 0.0000173, 0.0000245, 0.0000347, 0.0000494
	Black	Women	31	0.0000138, 0.0000198, 0.0000281, 0.00004, 0.0000571
	Black	Women	32	0.0000156, 0.0000224, 0.0000322, 0.0000461, 0.0000665
	Black	Women	33	0.0000173, 0.0000253, 0.0000367, 0.0000532, 0.0000775
	Black	Women	34	0.0000193, 0.0000284, 0.0000417, 0.0000612, 0.0000901
	Black	Women	35	0.0000215, 0.0000319, 0.0000472, 0.0000697, 0.000103
	Black	Women	36	0.0000244, 0.0000361, 0.0000531, 0.0000782, 0.000116
	Black	Women	37	0.0000283, 0.0000411, 0.0000596, 0.0000863, 0.000125
	Black	Women	38	0.0000332, 0.0000471, 0.0000665, 0.0000939, 0.000133
	Black	Women	39	0.0000388, 0.0000537, 0.0000739, 0.000102, 0.000141
	Black	Women	40	0.0000447, 0.0000606, 0.0000818, 0.00011, 0.00015
	Black	Women	41	0.000051, 0.0000679, 0.0000902, 0.00012, 0.00016
	Black	Women	42	0.0000579, 0.0000759, 0.0000992, 0.00013, 0.00017
	Black	Women	43	0.0000656, 0.0000846, 0.000109, 0.00014, 0.000181
	Black	Women	44	0.0000735, 0.0000938, 0.00012, 0.000152, 0.000194
	Black	Women	45	0.0000817, 0.000104, 0.000131, 0.000166, 0.000211
	Black	Women	46	0.0000916, 0.000115, 0.000145, 0.000182, 0.000229
	Black	Women	47	0.000104, 0.000129, 0.000159, 0.000197, 0.000244
	Black	Women	48	0.000119, 0.000144, 0.000175, 0.000213, 0.000259
	Black	Women	49	0.000134, 0.000161, 0.000192, 0.000231, 0.000277
	Black	Women	50	0.000148, 0.000177, 0.00021, 0.00025, 0.000298
	Black	Women	51	0.000163, 0.000193, 0.000229, 0.000271, 0.000321
	Black	Women	52	0.000179, 0.000211, 0.000248, 0.000291, 0.000342
	Black	Women	53	0.000196, 0.000229, 0.000267, 0.000312, 0.000364
	Black	Women	54	0.000213, 0.000248, 0.000288, 0.000336, 0.000391
	Black	Women	55	0.000229, 0.000267, 0.000312, 0.000363, 0.000424
	Black	Women	56	0.000248, 0.00029, 0.000338, 0.000394, 0.00046
	Black	Women	57	0.000272, 0.000316, 0.000367, 0.000426, 0.000495
	Black	Women	58	0.000302, 0.000348, 0.0004, 0.00046, 0.000529
	Black	Women	59	0.000336, 0.000383, 0.000436, 0.000496, 0.000565

Variable	Race/ethnicity	Sex	Age	Distribution
	Black	Women	60	0.000368, 0.000418, 0.000473, 0.000537, 0.000609
	Black	Women	61	0.000396, 0.000451, 0.000512, 0.000582, 0.000662
	Black	Women	62	0.000422, 0.000482, 0.00055, 0.000628, 0.000718
	Black	Women	63	0.000449, 0.000515, 0.000589, 0.000675, 0.000774
	Black	Women	64	0.00048, 0.00055, 0.000629, 0.00072, 0.000825
	Black	Women	65	0.000517, 0.00059, 0.000672, 0.000766, 0.000874
	Black	Women	66	0.000563, 0.000637, 0.000722, 0.000817, 0.000926
	Black	Women	67	0.000617, 0.000694, 0.00078, 0.000876, 0.000986
	Black	Women	68	0.000683, 0.000763, 0.00085, 0.000948, 0.00106
	Black	Women	69	0.000761, 0.000844, 0.000935, 0.00103, 0.00115
	Black	Women	70	0.00085, 0.000938, 0.00103, 0.00114, 0.00126
	Black	Women	71	0.000946, 0.00104, 0.00115, 0.00127, 0.0014
	Black	Women	72	0.00105, 0.00116, 0.00129, 0.00142, 0.00158
	Black	Women	73	0.00117, 0.0013, 0.00144, 0.0016, 0.00177
	Black	Women	74	0.00132, 0.00146, 0.00162, 0.00179, 0.00199
	Black	Women	75	0.00148, 0.00164, 0.00182, 0.00202, 0.00224
	Black	Women	76	0.00164, 0.00183, 0.00204, 0.00228, 0.00254
	Black	Women	77	0.00181, 0.00204, 0.0023, 0.00259, 0.00292
	Black	Women	78	0.00199, 0.00227, 0.00259, 0.00295, 0.00337
	Black	Women	79	0.00219, 0.00252, 0.00291, 0.00335, 0.00386
	Black	Women	80	0.00243, 0.00281, 0.00326, 0.00377, 0.00437
	Black	Women	81	0.00269, 0.00313, 0.00363, 0.00422, 0.0049
	Black	Women	82	0.00297, 0.00346, 0.00403, 0.00469, 0.00546
	Black	Women	83	0.00323, 0.00378, 0.00442, 0.00517, 0.00606
	Black	Women	84	0.00348, 0.0041, 0.00482, 0.00567, 0.00668
	Hispanic	Men	30	0.00000803, 0.0000108, 0.0000144, 0.0000193, 0.0000258
	Hispanic	Men	31	0.00000958, 0.0000125, 0.0000163, 0.0000213, 0.0000278
	Hispanic	Men	32	0.0000113, 0.0000145, 0.0000185, 0.0000235, 0.0000301
	Hispanic	Men	33	0.0000132, 0.0000166, 0.0000209, 0.0000262, 0.000033
	Hispanic	Men	34	0.0000152, 0.0000189, 0.0000236, 0.0000294, 0.0000366
	Hispanic	Men	35	0.0000173, 0.0000215, 0.0000266, 0.0000329, 0.0000408
	Hispanic	Men	36	0.0000198, 0.0000244, 0.0000299, 0.0000367, 0.0000451
	Hispanic	Men	37	0.0000227, 0.0000276, 0.0000335, 0.0000406, 0.0000494
	Hispanic	Men	38	0.0000259, 0.0000311, 0.0000374, 0.0000449, 0.000054
	Hispanic	Men	39	0.0000293, 0.0000349, 0.0000416, 0.0000495, 0.0000591
	Hispanic	Men	40	0.0000328, 0.0000389, 0.0000461, 0.0000546, 0.0000649
	Hispanic	Men	41	0.0000364, 0.0000431, 0.0000509, 0.0000602, 0.0000713
	Hispanic	Men	42	0.0000404, 0.0000476, 0.0000561, 0.0000661, 0.000078
	Hispanic	Men	43	0.0000446, 0.0000525, 0.0000617, 0.0000725, 0.0000853
	Hispanic	Men	44	0.0000491, 0.0000578, 0.0000679, 0.0000797, 0.0000938
	Hispanic	Men	45	0.0000541, 0.0000636, 0.0000748, 0.0000879, 0.000103
	Hispanic	Men	46	0.0000597, 0.0000702, 0.0000825, 0.0000969, 0.000114
	Hispanic	Men	47	0.0000662, 0.0000778, 0.0000913, 0.000107, 0.000126

Variable	Race/ethnicity	Sex	Age	Distribution
	Hispanic	Men	48	0.0000739, 0.0000865, 0.000101, 0.000118, 0.000139
	Hispanic	Men	49	0.0000831, 0.0000968, 0.000112, 0.000131, 0.000152
	Hispanic	Men	50	0.0000944, 0.000109, 0.000125, 0.000144, 0.000166
	Hispanic	Men	51	0.000108, 0.000122, 0.000139, 0.000158, 0.000181
	Hispanic	Men	52	0.000121, 0.000137, 0.000155, 0.000175, 0.000198
	Hispanic	Men	53	0.000134, 0.000152, 0.000172, 0.000194, 0.00022
	Hispanic	Men	54	0.000144, 0.000165, 0.00019, 0.000217, 0.000249
	Hispanic	Men	55	0.000154, 0.000179, 0.000208, 0.000242, 0.000281
	Hispanic	Men	56	0.000166, 0.000195, 0.000228, 0.000267, 0.000313
	Hispanic	Men	57	0.000181, 0.000212, 0.000249, 0.000291, 0.000342
	Hispanic	Men	58	0.000199, 0.000232, 0.00027, 0.000315, 0.000367
	Hispanic	Men	59	0.000221, 0.000255, 0.000294, 0.000338, 0.00039
	Hispanic	Men	60	0.000246, 0.00028, 0.000318, 0.000362, 0.000413
	Hispanic	Men	61	0.000272, 0.000306, 0.000345, 0.000388, 0.000438
	Hispanic	Men	62	0.000297, 0.000333, 0.000373, 0.000418, 0.000468
	Hispanic	Men	63	0.000321, 0.00036, 0.000403, 0.000451, 0.000506
	Hispanic	Men	64	0.000345, 0.000388, 0.000436, 0.000489, 0.000549
	Hispanic	Men	65	0.000372, 0.000419, 0.000472, 0.000531, 0.000599
	Hispanic	Men	66	0.000402, 0.000455, 0.000513, 0.00058, 0.000656
	Hispanic	Men	67	0.000436, 0.000495, 0.000562, 0.000637, 0.000724
	Hispanic	Men	68	0.000475, 0.000542, 0.000618, 0.000704, 0.000804
	Hispanic	Men	69	0.000523, 0.000599, 0.000684, 0.000782, 0.000894
	Hispanic	Men	70	0.000582, 0.000666, 0.000761, 0.000869, 0.000994
	Hispanic	Men	71	0.000651, 0.000744, 0.000849, 0.000968, 0.00111
	Hispanic	Men	72	0.000731, 0.000833, 0.000949, 0.00108, 0.00123
	Hispanic	Men	73	0.000819, 0.000933, 0.00106, 0.00121, 0.00138
	Hispanic	Men	74	0.000917, 0.00104, 0.00119, 0.00135, 0.00154
	Hispanic	Men	75	0.00103, 0.00117, 0.00133, 0.00152, 0.00173
	Hispanic	Men	76	0.00115, 0.00131, 0.00149, 0.0017, 0.00194
	Hispanic	Men	77	0.00128, 0.00147, 0.00168, 0.00191, 0.00219
	Hispanic	Men	78	0.00143, 0.00164, 0.00188, 0.00216, 0.00247
	Hispanic	Men	79	0.00158, 0.00183, 0.00211, 0.00243, 0.0028
	Hispanic	Men	80	0.00175, 0.00203, 0.00236, 0.00273, 0.00317
	Hispanic	Men	81	0.00192, 0.00224, 0.00262, 0.00306, 0.00358
	Hispanic	Men	82	0.00209, 0.00246, 0.0029, 0.00341, 0.00402
	Hispanic	Men	83	0.00225, 0.00267, 0.00317, 0.00377, 0.00449
	Hispanic	Men	84	0.0024, 0.00288, 0.00345, 0.00413, 0.00495
	Hispanic	Women	30	0.00000449, 0.00000667, 0.00000988, 0.0000146, 0.0000217
	Hispanic	Women	31	0.00000527, 0.00000765, 0.0000111, 0.000016, 0.0000232
	Hispanic	Women	32	0.00000612, 0.00000875, 0.0000125, 0.0000178, 0.0000254
	Hispanic	Women	33	0.00000707, 0.00001, 0.0000141, 0.0000199, 0.0000281
	Hispanic	Women	34	0.00000819, 0.0000114, 0.000016, 0.0000223, 0.0000311
	Hispanic	Women	35	0.00000956, 0.0000132, 0.0000181, 0.0000248, 0.0000342

Variable	Race/ethnicity	Sex	Age	Distribution
	Hispanic	Women	36	0.0000112, 0.0000152, 0.0000204, 0.0000275, 0.0000371
	Hispanic	Women	37	0.0000132, 0.0000174, 0.000023, 0.0000303, 0.00004
	Hispanic	Women	38	0.0000154, 0.00002, 0.0000258, 0.0000333, 0.0000431
	Hispanic	Women	39	0.0000178, 0.0000227, 0.0000288, 0.0000366, 0.0000466
	Hispanic	Women	40	0.0000203, 0.0000255, 0.000032, 0.0000402, 0.0000506
	Hispanic	Women	41	0.0000228, 0.0000284, 0.0000354, 0.0000442, 0.0000552
	Hispanic	Women	42	0.0000252, 0.0000314, 0.0000391, 0.0000486, 0.0000606
	Hispanic	Women	43	0.0000278, 0.0000346, 0.0000431, 0.0000536, 0.0000668
	Hispanic	Women	44	0.0000308, 0.0000383, 0.0000475, 0.0000589, 0.0000732
	Hispanic	Women	45	0.0000347, 0.0000426, 0.0000523, 0.0000642, 0.000079
	Hispanic	Women	46	0.0000396, 0.0000478, 0.0000576, 0.0000694, 0.0000839
	Hispanic	Women	47	0.0000452, 0.0000536, 0.0000634, 0.000075, 0.0000889
	Hispanic	Women	48	0.000051, 0.0000597, 0.0000697, 0.0000815, 0.0000954
	Hispanic	Women	49	0.0000562, 0.0000657, 0.0000767, 0.0000895, 0.000105
	Hispanic	Women	50	0.0000608, 0.0000717, 0.0000844, 0.0000993, 0.000117
	Hispanic	Women	51	0.0000651, 0.0000778, 0.0000928, 0.000111, 0.000132
	Hispanic	Women	52	0.0000695, 0.0000843, 0.000102, 0.000123, 0.00015
	Hispanic	Women	53	0.0000745, 0.0000914, 0.000112, 0.000137, 0.000168
	Hispanic	Women	54	0.0000803, 0.0000993, 0.000123, 0.000151, 0.000187
	Hispanic	Women	55	0.0000874, 0.000108, 0.000134, 0.000166, 0.000206
	Hispanic	Women	56	0.0000959, 0.000119, 0.000146, 0.00018, 0.000223
	Hispanic	Women	57	0.000106, 0.00013, 0.00016, 0.000196, 0.000241
	Hispanic	Women	58	0.000117, 0.000143, 0.000174, 0.000213, 0.00026
	Hispanic	Women	59	0.000128, 0.000156, 0.00019, 0.000231, 0.000281
	Hispanic	Women	60	0.000141, 0.000171, 0.000207, 0.000251, 0.000305
	Hispanic	Women	61	0.000155, 0.000187, 0.000226, 0.000273, 0.000331
	Hispanic	Women	62	0.00017, 0.000205, 0.000246, 0.000297, 0.000358
	Hispanic	Women	63	0.000185, 0.000223, 0.000268, 0.000322, 0.000389
	Hispanic	Women	64	0.0002, 0.000242, 0.000291, 0.000351, 0.000424
	Hispanic	Women	65	0.000217, 0.000263, 0.000317, 0.000383, 0.000464
	Hispanic	Women	66	0.000237, 0.000287, 0.000348, 0.000421, 0.00051
	Hispanic	Women	67	0.00026, 0.000316, 0.000383, 0.000465, 0.000565
	Hispanic	Women	68	0.000289, 0.000351, 0.000426, 0.000517, 0.000628
	Hispanic	Women	69	0.000325, 0.000395, 0.000478, 0.000578, 0.000701
	Hispanic	Women	70	0.000372, 0.000448, 0.000539, 0.000648, 0.000781
	Hispanic	Women	71	0.000432, 0.000515, 0.000612, 0.000727, 0.000866
	Hispanic	Women	72	0.000508, 0.000595, 0.000697, 0.000816, 0.000957
	Hispanic	Women	73	0.000598, 0.00069, 0.000795, 0.000917, 0.00106
	Hispanic	Women	74	0.000701, 0.000799, 0.000909, 0.00103, 0.00118
	Hispanic	Women	75	0.000814, 0.000921, 0.00104, 0.00118, 0.00133
	Hispanic	Women	76	0.000936, 0.00106, 0.00119, 0.00135, 0.00152
	Hispanic	Women	77	0.00107, 0.00121, 0.00137, 0.00154, 0.00174
	Hispanic	Women	78	0.00124, 0.0014, 0.00157, 0.00177, 0.002

Variable	Race/ethnicity	Sex	Age	Distribution
	Hispanic	Women	79	0.00143, 0.00161, 0.00181, 0.00203, 0.00228
	Hispanic	Women	80	0.00165, 0.00185, 0.00207, 0.00232, 0.0026
	Hispanic	Women	81	0.00186, 0.00209, 0.00236, 0.00265, 0.00299
	Hispanic	Women	82	0.00203, 0.00233, 0.00266, 0.00304, 0.00348
	Hispanic	Women	83	0.00218, 0.00255, 0.00298, 0.00348, 0.00407
	Hispanic	Women	84	0.00232, 0.00277, 0.0033, 0.00393, 0.00469
	White	Men	30	0.00000643, 0.00000842, 0.000011, 0.0000143, 0.0000187
	White	Men	31	0.00000747, 0.00000961, 0.0000124, 0.0000159, 0.0000204
	White	Men	32	0.00000864, 0.000011, 0.0000139, 0.0000176, 0.0000223
	White	Men	33	0.00000998, 0.0000125, 0.0000156, 0.0000195, 0.0000244
	White	Men	34	0.0000115, 0.0000142, 0.0000175, 0.0000215, 0.0000266
	White	Men	35	0.0000131, 0.000016, 0.0000195, 0.0000238, 0.0000291
	White	Men	36	0.0000149, 0.000018, 0.0000218, 0.0000264, 0.000032
	White	Men	37	0.0000168, 0.0000202, 0.0000243, 0.0000293, 0.0000353
	White	Men	38	0.0000188, 0.0000226, 0.0000271, 0.0000325, 0.000039
	White	Men	39	0.000021, 0.0000252, 0.0000301, 0.000036, 0.0000431
	White	Men	40	0.0000234, 0.0000279, 0.0000333, 0.0000397, 0.0000474
	White	Men	41	0.0000262, 0.000031, 0.0000367, 0.0000435, 0.0000515
	White	Men	42	0.0000294, 0.0000345, 0.0000404, 0.0000473, 0.0000555
	White	Men	43	0.0000331, 0.0000384, 0.0000445, 0.0000515, 0.0000597
	White	Men	44	0.0000372, 0.0000428, 0.0000491, 0.0000563, 0.0000647
	White	Men	45	0.0000417, 0.0000476, 0.0000543, 0.0000618, 0.0000706
	White	Men	46	0.0000468, 0.0000531, 0.0000602, 0.0000682, 0.0000774
	White	Men	47	0.0000523, 0.0000592, 0.000067, 0.0000758, 0.0000858
	White	Men	48	0.0000582, 0.000066, 0.0000747, 0.0000847, 0.000096
	White	Men	49	0.0000644, 0.0000734, 0.0000835, 0.0000951, 0.000108
	White	Men	50	0.0000713, 0.0000817, 0.0000935, 0.000107, 0.000123
	White	Men	51	0.0000795, 0.0000913, 0.000105, 0.00012, 0.000138
	White	Men	52	0.0000894, 0.000102, 0.000117, 0.000133, 0.000153
	White	Men	53	0.000101, 0.000114, 0.00013, 0.000148, 0.000168
	White	Men	54	0.000112, 0.000127, 0.000144, 0.000164, 0.000186
	White	Men	55	0.000123, 0.00014, 0.00016, 0.000181, 0.000206
	White	Men	56	0.000135, 0.000154, 0.000176, 0.000201, 0.000229
	White	Men	57	0.000147, 0.000169, 0.000194, 0.000222, 0.000255
	White	Men	58	0.00016, 0.000185, 0.000213, 0.000245, 0.000283
	White	Men	59	0.000175, 0.000203, 0.000234, 0.00027, 0.000312
	White	Men	60	0.000193, 0.000223, 0.000257, 0.000296, 0.000341
	White	Men	61	0.000215, 0.000247, 0.000282, 0.000323, 0.000369
	White	Men	62	0.000241, 0.000273, 0.000309, 0.00035, 0.000397
	White	Men	63	0.00027, 0.000302, 0.000338, 0.000378, 0.000423
	White	Men	64	0.000303, 0.000335, 0.00037, 0.000409, 0.000452
	White	Men	65	0.000339, 0.000371, 0.000406, 0.000444, 0.000486
	White	Men	66	0.000377, 0.000411, 0.000447, 0.000486, 0.000529

Variable	Race/ethnicity	Sex	Age	Distribution
	White	Men	67	0.00042, 0.000456, 0.000495, 0.000538, 0.000585
	White	Men	68	0.000467, 0.000508, 0.000553, 0.000602, 0.000656
	White	Men	69	0.00052, 0.000569, 0.000621, 0.000679, 0.000742
	White	Men	70	0.000582, 0.000639, 0.000701, 0.00077, 0.000845
	White	Men	71	0.000653, 0.00072, 0.000794, 0.000874, 0.000964
	White	Men	72	0.000734, 0.000812, 0.000898, 0.000993, 0.0011
	White	Men	73	0.000824, 0.000916, 0.00102, 0.00113, 0.00125
	White	Men	74	0.000925, 0.00103, 0.00115, 0.00128, 0.00143
	White	Men	75	0.00104, 0.00116, 0.0013, 0.00146, 0.00163
	White	Men	76	0.00117, 0.00131, 0.00148, 0.00166, 0.00187
	White	Men	77	0.00131, 0.00149, 0.00168, 0.0019, 0.00215
	White	Men	78	0.00148, 0.00169, 0.00192, 0.00219, 0.00249
	White	Men	79	0.00166, 0.00191, 0.0022, 0.00253, 0.00291
	White	Men	80	0.00186, 0.00216, 0.00251, 0.00292, 0.0034
	White	Men	81	0.00207, 0.00244, 0.00286, 0.00337, 0.00396
	White	Men	82	0.0023, 0.00273, 0.00324, 0.00385, 0.00457
	White	Men	83	0.00253, 0.00303, 0.00363, 0.00435, 0.00522
	White	Men	84	0.00275, 0.00333, 0.00403, 0.00487, 0.00591
	White	Women	30	0.00000566, 0.00000762, 0.0000102, 0.0000137, 0.0000185
	White	Women	31	0.00000654, 0.00000872, 0.0000116, 0.0000154, 0.0000206
	White	Women	32	0.0000076, 0.00001, 0.0000132, 0.0000173, 0.0000227
	White	Women	33	0.00000885, 0.0000115, 0.0000149, 0.0000193, 0.0000251
	White	Women	34	0.0000102, 0.0000131, 0.0000168, 0.0000215, 0.0000275
	White	Women	35	0.0000118, 0.0000149, 0.0000188, 0.0000238, 0.0000301
	White	Women	36	0.0000135, 0.0000168, 0.000021, 0.0000262, 0.0000328
	White	Women	37	0.0000153, 0.0000189, 0.0000233, 0.0000288, 0.0000356
	White	Women	38	0.0000173, 0.0000211, 0.0000258, 0.0000315, 0.0000386
	White	Women	39	0.0000193, 0.0000234, 0.0000284, 0.0000344, 0.0000417
	White	Women	40	0.0000216, 0.0000259, 0.0000311, 0.0000373, 0.0000448
	White	Women	41	0.0000241, 0.0000286, 0.0000339, 0.0000402, 0.0000478
	White	Women	42	0.0000269, 0.0000315, 0.0000369, 0.0000432, 0.0000506
	White	Women	43	0.0000299, 0.0000346, 0.0000401, 0.0000464, 0.0000538
	White	Women	44	0.0000329, 0.000038, 0.0000437, 0.0000503, 0.000058
	White	Women	45	0.000036, 0.0000415, 0.0000477, 0.0000549, 0.0000633
	White	Women	46	0.0000391, 0.0000453, 0.0000523, 0.0000603, 0.0000698
	White	Women	47	0.0000424, 0.0000494, 0.0000574, 0.0000666, 0.0000776
	White	Women	48	0.0000458, 0.0000538, 0.000063, 0.0000739, 0.0000868
	White	Women	49	0.0000493, 0.0000585, 0.0000693, 0.0000821, 0.0000974
	White	Women	50	0.0000533, 0.0000638, 0.0000763, 0.0000912, 0.000109
	White	Women	51	0.0000581, 0.0000699, 0.0000838, 0.000101, 0.000121
	White	Women	52	0.0000642, 0.0000769, 0.000092, 0.00011, 0.000132
	White	Women	53	0.0000714, 0.0000848, 0.000101, 0.000119, 0.000142
	White	Women	54	0.0000796, 0.0000935, 0.00011, 0.000129, 0.000151

Variable	Race/ethnicity	Sex	Age	Distribution
	White	Women	55	0.0000884, 0.000103, 0.000119, 0.000138, 0.000161
	White	Women	56	0.000098, 0.000113, 0.000129, 0.000149, 0.000171
	White	Women	57	0.000109, 0.000124, 0.000141, 0.00016, 0.000182
	White	Women	58	0.000121, 0.000136, 0.000153, 0.000172, 0.000194
	White	Women	59	0.000135, 0.00015, 0.000167, 0.000187, 0.000208
	White	Women	60	0.00015, 0.000166, 0.000184, 0.000204, 0.000226
	White	Women	61	0.000166, 0.000183, 0.000203, 0.000224, 0.000248
	White	Women	62	0.000183, 0.000203, 0.000224, 0.000247, 0.000273
	White	Women	63	0.000202, 0.000224, 0.000247, 0.000272, 0.000301
	White	Women	64	0.000224, 0.000247, 0.000272, 0.0003, 0.000332
	White	Women	65	0.000248, 0.000274, 0.000302, 0.000332, 0.000366
	White	Women	66	0.000276, 0.000304, 0.000335, 0.000369, 0.000406
	White	Women	67	0.000309, 0.000341, 0.000375, 0.000413, 0.000455
	White	Women	68	0.000349, 0.000384, 0.000423, 0.000466, 0.000513
	White	Women	69	0.000398, 0.000438, 0.000481, 0.000529, 0.000582
	White	Women	70	0.000458, 0.000503, 0.000552, 0.000605, 0.000664
	White	Women	71	0.000528, 0.000579, 0.000635, 0.000697, 0.000765
	White	Women	72	0.000606, 0.000667, 0.000733, 0.000806, 0.000886
	White	Women	73	0.000696, 0.000768, 0.000846, 0.000933, 0.00103
	White	Women	74	0.000799, 0.000884, 0.000976, 0.00108, 0.00119
	White	Women	75	0.000921, 0.00102, 0.00113, 0.00124, 0.00138
	White	Women	76	0.00106, 0.00117, 0.0013, 0.00144, 0.00159
	White	Women	77	0.00122, 0.00135, 0.0015, 0.00167, 0.00185
	White	Women	78	0.00139, 0.00156, 0.00174, 0.00195, 0.00218
	White	Women	79	0.00159, 0.00179, 0.00202, 0.00228, 0.00258
	White	Women	80	0.00181, 0.00207, 0.00235, 0.00268, 0.00305
	White	Women	81	0.00208, 0.00238, 0.00272, 0.00312, 0.00357
	White	Women	82	0.00237, 0.00273, 0.00314, 0.00361, 0.00415
	White	Women	83	0.00268, 0.0031, 0.00358, 0.00413, 0.00477
	White	Women	84	0.003, 0.00348, 0.00403, 0.00467, 0.00542
Stroke mortality rates for 2021 (0.01, 0.2, 0.5, 0.8. 0.99 percentiles of the empirical distribution produced during forecasting)				
	Black	Men	30	0.0000171, 0.0000228, 0.0000304, 0.0000404, 0.000054
	Black	Men	31	0.0000209, 0.0000273, 0.0000354, 0.0000461, 0.00006
	Black	Men	32	0.0000249, 0.0000321, 0.0000412, 0.0000529, 0.0000681
	Black	Men	33	0.0000291, 0.0000372, 0.0000476, 0.0000609, 0.000078
	Black	Men	34	0.0000334, 0.0000428, 0.0000546, 0.0000697, 0.0000892
	Black	Men	35	0.0000382, 0.0000488, 0.0000621, 0.0000791, 0.000101
	Black	Men	36	0.0000434, 0.0000552, 0.00007, 0.0000888, 0.000113
	Black	Men	37	0.0000495, 0.0000623, 0.0000783, 0.0000983, 0.000124
	Black	Men	38	0.0000563, 0.00007, 0.0000868, 0.000108, 0.000134
	Black	Men	39	0.0000636, 0.0000781, 0.0000957, 0.000117, 0.000144
	Black	Men	40	0.0000713, 0.0000866, 0.000105, 0.000127, 0.000155
	Black	Men	41	0.0000798, 0.000096, 0.000115, 0.000138, 0.000166

Variable	Race/ethnicity	Sex	Age	Distribution
	Black	Men	42	0.00009, 0.000107, 0.000127, 0.00015, 0.000178
	Black	Men	43	0.000101, 0.000119, 0.00014, 0.000164, 0.000194
	Black	Men	44	0.000111, 0.000131, 0.000155, 0.000183, 0.000216
	Black	Men	45	0.000121, 0.000144, 0.000172, 0.000205, 0.000244
	Black	Men	46	0.000133, 0.000159, 0.000191, 0.000229, 0.000274
	Black	Men	47	0.000147, 0.000177, 0.000213, 0.000255, 0.000307
	Black	Men	48	0.000162, 0.000196, 0.000237, 0.000287, 0.000348
	Black	Men	49	0.000179, 0.000218, 0.000266, 0.000324, 0.000396
	Black	Men	50	0.000202, 0.000246, 0.000299, 0.000364, 0.000444
	Black	Men	51	0.000235, 0.000282, 0.000338, 0.000404, 0.000485
	Black	Men	52	0.000276, 0.000324, 0.00038, 0.000446, 0.000524
	Black	Men	53	0.000318, 0.000368, 0.000426, 0.000492, 0.00057
	Black	Men	54	0.000359, 0.000412, 0.000473, 0.000543, 0.000624
	Black	Men	55	0.000397, 0.000455, 0.000521, 0.000596, 0.000683
	Black	Men	56	0.000434, 0.000497, 0.000569, 0.00065, 0.000745
	Black	Men	57	0.000472, 0.00054, 0.000617, 0.000705, 0.000807
	Black	Men	58	0.000515, 0.000586, 0.000667, 0.000759, 0.000865
	Black	Men	59	0.000562, 0.000636, 0.000719, 0.000814, 0.000922
	Black	Men	60	0.000603, 0.000684, 0.000774, 0.000876, 0.000993
	Black	Men	61	0.000638, 0.000728, 0.00083, 0.000946, 0.00108
	Black	Men	62	0.000678, 0.000775, 0.000886, 0.00101, 0.00116
	Black	Men	63	0.00073, 0.00083, 0.000943, 0.00107, 0.00122
	Black	Men	64	0.00079, 0.00089, 0.001, 0.00113, 0.00127
	Black	Men	65	0.000846, 0.00095, 0.00106, 0.00119, 0.00134
	Black	Men	66	0.000899, 0.00101, 0.00114, 0.00128, 0.00144
	Black	Men	67	0.000963, 0.00109, 0.00122, 0.00138, 0.00155
	Black	Men	68	0.00105, 0.00118, 0.00132, 0.00149, 0.00167
	Black	Men	69	0.00115, 0.00129, 0.00144, 0.00161, 0.0018
	Black	Men	70	0.00126, 0.0014, 0.00156, 0.00174, 0.00195
	Black	Men	71	0.00137, 0.00153, 0.0017, 0.0019, 0.00212
	Black	Men	72	0.00148, 0.00166, 0.00185, 0.00206, 0.0023
	Black	Men	73	0.0016, 0.00179, 0.002, 0.00224, 0.00251
	Black	Men	74	0.00171, 0.00193, 0.00217, 0.00244, 0.00276
	Black	Men	75	0.00182, 0.00207, 0.00235, 0.00267, 0.00304
	Black	Men	76	0.00196, 0.00224, 0.00256, 0.00292, 0.00333
	Black	Men	77	0.00214, 0.00244, 0.00279, 0.00318, 0.00364
	Black	Men	78	0.00233, 0.00266, 0.00304, 0.00347, 0.00397
	Black	Men	79	0.00252, 0.00289, 0.00332, 0.0038, 0.00436
	Black	Men	80	0.00272, 0.00313, 0.0036, 0.00415, 0.00478
	Black	Men	81	0.00291, 0.00337, 0.00389, 0.0045, 0.00521
	Black	Men	82	0.00311, 0.00361, 0.00418, 0.00485, 0.00562
	Black	Men	83	0.0033, 0.00384, 0.00446, 0.00518, 0.00603
	Black	Men	84	0.00348, 0.00406, 0.00473, 0.00551, 0.00642

Variable	Race/ethnicity	Sex	Age	Distribution
	Black	Women	30	0.0000119, 0.0000171, 0.0000244, 0.000035, 0.0000502
	Black	Women	31	0.0000136, 0.0000195, 0.000028, 0.0000402, 0.0000579
	Black	Women	32	0.0000153, 0.0000222, 0.0000321, 0.0000463, 0.0000672
	Black	Women	33	0.0000171, 0.0000251, 0.0000366, 0.0000534, 0.0000782
	Black	Women	34	0.000019, 0.0000282, 0.0000416, 0.0000613, 0.0000908
	Black	Women	35	0.0000212, 0.0000316, 0.000047, 0.0000698, 0.000104
	Black	Women	36	0.0000241, 0.0000357, 0.0000529, 0.0000782, 0.000116
	Black	Women	37	0.0000278, 0.0000407, 0.0000592, 0.0000862, 0.000126
	Black	Women	38	0.0000325, 0.0000464, 0.000066, 0.0000938, 0.000134
	Black	Women	39	0.0000378, 0.0000527, 0.0000732, 0.000102, 0.000142
	Black	Women	40	0.0000434, 0.0000593, 0.0000808, 0.00011, 0.000151
	Black	Women	41	0.0000492, 0.0000662, 0.0000889, 0.000119, 0.000161
	Black	Women	42	0.0000557, 0.0000738, 0.0000975, 0.000129, 0.00017
	Black	Women	43	0.000063, 0.0000821, 0.000107, 0.000139, 0.000181
	Black	Women	44	0.0000705, 0.000091, 0.000117, 0.000151, 0.000194
	Black	Women	45	0.0000784, 0.000101, 0.000129, 0.000164, 0.000211
	Black	Women	46	0.000088, 0.000112, 0.000141, 0.000179, 0.000228
	Black	Women	47	0.0001, 0.000125, 0.000156, 0.000194, 0.000243
	Black	Women	48	0.000114, 0.00014, 0.000172, 0.00021, 0.000258
	Black	Women	49	0.000129, 0.000156, 0.000188, 0.000227, 0.000275
	Black	Women	50	0.000143, 0.000172, 0.000206, 0.000247, 0.000297
	Black	Women	51	0.000157, 0.000188, 0.000224, 0.000268, 0.00032
	Black	Women	52	0.000173, 0.000205, 0.000243, 0.000288, 0.000341
	Black	Women	53	0.00019, 0.000224, 0.000263, 0.000309, 0.000363
	Black	Women	54	0.000206, 0.000242, 0.000284, 0.000333, 0.000391
	Black	Women	55	0.000222, 0.000261, 0.000307, 0.000361, 0.000424
	Black	Women	56	0.000241, 0.000283, 0.000333, 0.000391, 0.00046
	Black	Women	57	0.000264, 0.00031, 0.000362, 0.000423, 0.000495
	Black	Women	58	0.000294, 0.000341, 0.000394, 0.000456, 0.000528
	Black	Women	59	0.000327, 0.000375, 0.000429, 0.000491, 0.000563
	Black	Women	60	0.000358, 0.000409, 0.000466, 0.000531, 0.000606
	Black	Women	61	0.000386, 0.000441, 0.000503, 0.000575, 0.000657
	Black	Women	62	0.000411, 0.000471, 0.000541, 0.00062, 0.000712
	Black	Women	63	0.000436, 0.000502, 0.000578, 0.000664, 0.000765
	Black	Women	64	0.000466, 0.000536, 0.000616, 0.000708, 0.000814
	Black	Women	65	0.000502, 0.000575, 0.000658, 0.000752, 0.000861
	Black	Women	66	0.000545, 0.00062, 0.000705, 0.000801, 0.000912
	Black	Women	67	0.000598, 0.000675, 0.000762, 0.000859, 0.000971
	Black	Women	68	0.000661, 0.000741, 0.00083, 0.00093, 0.00104
	Black	Women	69	0.000736, 0.00082, 0.000912, 0.00101, 0.00113
	Black	Women	70	0.000821, 0.000911, 0.00101, 0.00112, 0.00124
	Black	Women	71	0.000914, 0.00101, 0.00112, 0.00125, 0.00138
	Black	Women	72	0.00102, 0.00113, 0.00126, 0.00139, 0.00155

Variable	Race/ethnicity	Sex	Age	Distribution
	Black	Women	73	0.00113, 0.00126, 0.00141, 0.00156, 0.00174
	Black	Women	74	0.00127, 0.00142, 0.00158, 0.00175, 0.00195
	Black	Women	75	0.00143, 0.00159, 0.00177, 0.00197, 0.0022
	Black	Women	76	0.00159, 0.00178, 0.00199, 0.00223, 0.0025
	Black	Women	77	0.00175, 0.00198, 0.00224, 0.00253, 0.00287
	Black	Women	78	0.00192, 0.0022, 0.00252, 0.00288, 0.00331
	Black	Women	79	0.00212, 0.00245, 0.00283, 0.00327, 0.00379
	Black	Women	80	0.00234, 0.00273, 0.00317, 0.00369, 0.0043
	Black	Women	81	0.0026, 0.00303, 0.00354, 0.00413, 0.00482
	Black	Women	82	0.00286, 0.00335, 0.00392, 0.00458, 0.00537
	Black	Women	83	0.00311, 0.00366, 0.00431, 0.00506, 0.00596
	Black	Women	84	0.00335, 0.00396, 0.00469, 0.00555, 0.00657
	Hispanic	Men	30	0.00000784, 0.0000106, 0.0000143, 0.0000194, 0.0000262
	Hispanic	Men	31	0.00000938, 0.0000124, 0.0000162, 0.0000214, 0.0000281
	Hispanic	Men	32	0.0000111, 0.0000143, 0.0000184, 0.0000236, 0.0000305
	Hispanic	Men	33	0.000013, 0.0000164, 0.0000208, 0.0000263, 0.0000334
	Hispanic	Men	34	0.0000149, 0.0000187, 0.0000235, 0.0000295, 0.0000371
	Hispanic	Men	35	0.000017, 0.0000212, 0.0000265, 0.000033, 0.0000413
	Hispanic	Men	36	0.0000194, 0.000024, 0.0000297, 0.0000368, 0.0000456
	Hispanic	Men	37	0.0000221, 0.0000272, 0.0000333, 0.0000407, 0.00005
	Hispanic	Men	38	0.0000252, 0.0000306, 0.0000371, 0.0000449, 0.0000545
	Hispanic	Men	39	0.0000284, 0.0000342, 0.0000411, 0.0000494, 0.0000595
	Hispanic	Men	40	0.0000318, 0.000038, 0.0000455, 0.0000544, 0.0000652
	Hispanic	Men	41	0.0000353, 0.0000421, 0.0000501, 0.0000597, 0.0000712
	Hispanic	Men	42	0.0000392, 0.0000465, 0.0000551, 0.0000653, 0.0000775
	Hispanic	Men	43	0.0000433, 0.0000512, 0.0000605, 0.0000713, 0.0000843
	Hispanic	Men	44	0.0000477, 0.0000563, 0.0000664, 0.0000783, 0.0000924
	Hispanic	Men	45	0.0000524, 0.0000619, 0.000073, 0.0000861, 0.000102
	Hispanic	Men	46	0.0000579, 0.0000683, 0.0000805, 0.0000948, 0.000112
	Hispanic	Men	47	0.0000642, 0.0000757, 0.000089, 0.000105, 0.000123
	Hispanic	Men	48	0.0000717, 0.0000842, 0.0000987, 0.000116, 0.000136
	Hispanic	Men	49	0.0000808, 0.0000942, 0.00011, 0.000128, 0.000149
	Hispanic	Men	50	0.0000918, 0.000106, 0.000122, 0.000141, 0.000163
	Hispanic	Men	51	0.000105, 0.00012, 0.000136, 0.000155, 0.000177
	Hispanic	Men	52	0.000119, 0.000134, 0.000152, 0.000172, 0.000194
	Hispanic	Men	53	0.000131, 0.000149, 0.000169, 0.000191, 0.000217
	Hispanic	Men	54	0.000141, 0.000162, 0.000186, 0.000214, 0.000247
	Hispanic	Men	55	0.000151, 0.000176, 0.000205, 0.000239, 0.000279
	Hispanic	Men	56	0.000162, 0.000191, 0.000225, 0.000264, 0.000311
	Hispanic	Men	57	0.000177, 0.000208, 0.000245, 0.000288, 0.00034
	Hispanic	Men	58	0.000195, 0.000228, 0.000266, 0.000312, 0.000365
	Hispanic	Men	59	0.000216, 0.00025, 0.000289, 0.000335, 0.000388
	Hispanic	Men	60	0.00024, 0.000274, 0.000313, 0.000358, 0.00041

Variable	Race/ethnicity	Sex	Age	Distribution
	Hispanic	Men	61	0.000264, 0.0003, 0.000339, 0.000384, 0.000435
	Hispanic	Men	62	0.000289, 0.000325, 0.000366, 0.000412, 0.000465
	Hispanic	Men	63	0.000312, 0.000351, 0.000395, 0.000445, 0.000501
	Hispanic	Men	64	0.000336, 0.000379, 0.000427, 0.000481, 0.000543
	Hispanic	Men	65	0.000361, 0.000409, 0.000462, 0.000522, 0.00059
	Hispanic	Men	66	0.00039, 0.000443, 0.000502, 0.000569, 0.000646
	Hispanic	Men	67	0.000423, 0.000482, 0.000549, 0.000625, 0.000713
	Hispanic	Men	68	0.000461, 0.000528, 0.000604, 0.000691, 0.000791
	Hispanic	Men	69	0.000508, 0.000583, 0.000668, 0.000766, 0.00088
	Hispanic	Men	70	0.000564, 0.000648, 0.000743, 0.000852, 0.000978
	Hispanic	Men	71	0.000631, 0.000724, 0.000829, 0.000949, 0.00109
	Hispanic	Men	72	0.000708, 0.00081, 0.000926, 0.00106, 0.00121
	Hispanic	Men	73	0.000793, 0.000907, 0.00104, 0.00118, 0.00135
	Hispanic	Men	74	0.000888, 0.00102, 0.00116, 0.00132, 0.00151
	Hispanic	Men	75	0.000993, 0.00114, 0.0013, 0.00148, 0.0017
	Hispanic	Men	76	0.00111, 0.00127, 0.00146, 0.00167, 0.00191
	Hispanic	Men	77	0.00124, 0.00142, 0.00163, 0.00187, 0.00215
	Hispanic	Men	78	0.00138, 0.00159, 0.00183, 0.00211, 0.00243
	Hispanic	Men	79	0.00153, 0.00178, 0.00206, 0.00238, 0.00275
	Hispanic	Men	80	0.00169, 0.00197, 0.0023, 0.00268, 0.00312
	Hispanic	Men	81	0.00186, 0.00218, 0.00256, 0.003, 0.00352
	Hispanic	Men	82	0.00202, 0.00239, 0.00283, 0.00334, 0.00396
	Hispanic	Men	83	0.00217, 0.0026, 0.0031, 0.0037, 0.00442
	Hispanic	Men	84	0.00233, 0.0028, 0.00337, 0.00405, 0.00488
	Hispanic	Women	30	0.0000044, 0.00000659, 0.00000983, 0.0000147, 0.000022
	Hispanic	Women	31	0.00000518, 0.00000757, 0.000011, 0.000016, 0.0000234
	Hispanic	Women	32	0.00000603, 0.00000867, 0.0000124, 0.0000178, 0.0000256
	Hispanic	Women	33	0.00000698, 0.00000992, 0.000014, 0.0000199, 0.0000283
	Hispanic	Women	34	0.00000808, 0.0000114, 0.0000159, 0.0000223, 0.0000313
	Hispanic	Women	35	0.00000943, 0.000013, 0.000018, 0.0000248, 0.0000344
	Hispanic	Women	36	0.0000111, 0.000015, 0.0000203, 0.0000275, 0.0000373
	Hispanic	Women	37	0.000013, 0.0000172, 0.0000228, 0.0000303, 0.0000402
	Hispanic	Women	38	0.0000151, 0.0000197, 0.0000256, 0.0000332, 0.0000433
	Hispanic	Women	39	0.0000174, 0.0000223, 0.0000285, 0.0000364, 0.0000467
	Hispanic	Women	40	0.0000197, 0.000025, 0.0000316, 0.0000399, 0.0000505
	Hispanic	Women	41	0.0000221, 0.0000278, 0.0000348, 0.0000437, 0.0000549
	Hispanic	Women	42	0.0000246, 0.0000307, 0.0000384, 0.0000479, 0.0000599
	Hispanic	Women	43	0.0000271, 0.0000338, 0.0000422, 0.0000526, 0.0000658
	Hispanic	Women	44	0.0000299, 0.0000373, 0.0000464, 0.0000577, 0.000072
	Hispanic	Women	45	0.0000337, 0.0000415, 0.0000511, 0.0000629, 0.0000775
	Hispanic	Women	46	0.0000385, 0.0000466, 0.0000562, 0.000068, 0.0000823
	Hispanic	Women	47	0.000044, 0.0000522, 0.0000619, 0.0000734, 0.0000871
	Hispanic	Women	48	0.0000496, 0.0000582, 0.0000681, 0.0000798, 0.0000936

Variable	Race/ethnicity	Sex	Age	Distribution
	Hispanic	Women	49	0.0000548, 0.0000641, 0.000075, 0.0000877, 0.000103
	Hispanic	Women	50	0.0000593, 0.00007, 0.0000826, 0.0000974, 0.000115
	Hispanic	Women	51	0.0000636, 0.0000761, 0.000091, 0.000109, 0.00013
	Hispanic	Women	52	0.0000681, 0.0000826, 0.0001, 0.000121, 0.000147
	Hispanic	Women	53	0.000073, 0.0000897, 0.00011, 0.000135, 0.000166
	Hispanic	Women	54	0.0000788, 0.0000976, 0.000121, 0.000149, 0.000185
	Hispanic	Women	55	0.0000858, 0.000107, 0.000132, 0.000164, 0.000203
	Hispanic	Women	56	0.0000942, 0.000117, 0.000144, 0.000178, 0.000221
	Hispanic	Women	57	0.000104, 0.000128, 0.000157, 0.000193, 0.000238
	Hispanic	Women	58	0.000114, 0.00014, 0.000172, 0.00021, 0.000257
	Hispanic	Women	59	0.000126, 0.000154, 0.000187, 0.000228, 0.000279
	Hispanic	Women	60	0.000138, 0.000168, 0.000204, 0.000248, 0.000302
	Hispanic	Women	61	0.000152, 0.000184, 0.000223, 0.00027, 0.000327
	Hispanic	Women	62	0.000166, 0.000201, 0.000242, 0.000293, 0.000354
	Hispanic	Women	63	0.00018, 0.000218, 0.000263, 0.000317, 0.000384
	Hispanic	Women	64	0.000195, 0.000236, 0.000286, 0.000345, 0.000417
	Hispanic	Women	65	0.000212, 0.000257, 0.000311, 0.000376, 0.000456
	Hispanic	Women	66	0.000231, 0.00028, 0.00034, 0.000412, 0.000501
	Hispanic	Women	67	0.000253, 0.000308, 0.000375, 0.000455, 0.000554
	Hispanic	Women	68	0.000281, 0.000342, 0.000416, 0.000506, 0.000617
	Hispanic	Women	69	0.000316, 0.000385, 0.000467, 0.000566, 0.000688
	Hispanic	Women	70	0.000362, 0.000437, 0.000527, 0.000635, 0.000767
	Hispanic	Women	71	0.00042, 0.000501, 0.000597, 0.000712, 0.00085
	Hispanic	Women	72	0.000492, 0.000579, 0.00068, 0.000799, 0.000939
	Hispanic	Women	73	0.00058, 0.000671, 0.000776, 0.000897, 0.00104
	Hispanic	Women	74	0.000679, 0.000776, 0.000886, 0.00101, 0.00116
	Hispanic	Women	75	0.000787, 0.000894, 0.00101, 0.00115, 0.00131
	Hispanic	Women	76	0.000905, 0.00103, 0.00116, 0.00132, 0.00149
	Hispanic	Women	77	0.00104, 0.00118, 0.00133, 0.00151, 0.00171
	Hispanic	Women	78	0.0012, 0.00136, 0.00153, 0.00173, 0.00196
	Hispanic	Women	79	0.00138, 0.00156, 0.00176, 0.00198, 0.00224
	Hispanic	Women	80	0.00159, 0.00179, 0.00201, 0.00227, 0.00255
	Hispanic	Women	81	0.00179, 0.00203, 0.00229, 0.00259, 0.00294
	Hispanic	Women	82	0.00196, 0.00226, 0.00259, 0.00297, 0.00342
	Hispanic	Women	83	0.00211, 0.00247, 0.0029, 0.0034, 0.00399
	Hispanic	Women	84	0.00224, 0.00268, 0.00321, 0.00384, 0.0046
	White	Men	30	0.00000626, 0.00000828, 0.0000109, 0.0000144, 0.000019
	White	Men	31	0.00000729, 0.00000948, 0.0000123, 0.0000159, 0.0000207
	White	Men	32	0.00000846, 0.0000108, 0.0000138, 0.0000177, 0.0000226
	White	Men	33	0.00000978, 0.0000123, 0.0000155, 0.0000195, 0.0000246
	White	Men	34	0.0000113, 0.000014, 0.0000174, 0.0000216, 0.0000269
	White	Men	35	0.0000128, 0.0000158, 0.0000194, 0.0000239, 0.0000294
	White	Men	36	0.0000145, 0.0000178, 0.0000217, 0.0000265, 0.0000324

Variable	Race/ethnicity	Sex	Age	Distribution
	White	Men	37	0.0000164, 0.0000199, 0.0000242, 0.0000293, 0.0000356
	White	Men	38	0.0000183, 0.0000222, 0.0000268, 0.0000325, 0.0000393
	White	Men	39	0.0000204, 0.0000246, 0.0000297, 0.0000358, 0.0000433
	White	Men	40	0.0000227, 0.0000273, 0.0000328, 0.0000394, 0.0000475
	White	Men	41	0.0000253, 0.0000302, 0.0000361, 0.000043, 0.0000514
	White	Men	42	0.0000285, 0.0000336, 0.0000396, 0.0000467, 0.0000551
	White	Men	43	0.0000321, 0.0000374, 0.0000435, 0.0000507, 0.0000591
	White	Men	44	0.000036, 0.0000416, 0.0000479, 0.0000553, 0.0000639
	White	Men	45	0.0000403, 0.0000462, 0.000053, 0.0000606, 0.0000695
	White	Men	46	0.0000452, 0.0000516, 0.0000587, 0.0000669, 0.0000762
	White	Men	47	0.0000506, 0.0000575, 0.0000653, 0.0000742, 0.0000843
	White	Men	48	0.0000562, 0.000064, 0.0000729, 0.0000829, 0.0000944
	White	Men	49	0.0000622, 0.0000712, 0.0000815, 0.0000932, 0.000107
	White	Men	50	0.0000688, 0.0000793, 0.0000913, 0.000105, 0.000121
	White	Men	51	0.0000768, 0.0000887, 0.000102, 0.000118, 0.000136
	White	Men	52	0.0000864, 0.0000994, 0.000114, 0.000131, 0.000151
	White	Men	53	0.0000973, 0.000111, 0.000127, 0.000146, 0.000167
	White	Men	54	0.000109, 0.000124, 0.000141, 0.000161, 0.000184
	White	Men	55	0.00012, 0.000137, 0.000157, 0.000179, 0.000205
	White	Men	56	0.000131, 0.00015, 0.000173, 0.000198, 0.000228
	White	Men	57	0.000142, 0.000165, 0.00019, 0.000219, 0.000254
	White	Men	58	0.000155, 0.00018, 0.000209, 0.000242, 0.000282
	White	Men	59	0.00017, 0.000198, 0.00023, 0.000267, 0.000311
	White	Men	60	0.000187, 0.000217, 0.000252, 0.000292, 0.00034
	White	Men	61	0.000208, 0.00024, 0.000277, 0.000318, 0.000367
	White	Men	62	0.000233, 0.000266, 0.000303, 0.000345, 0.000393
	White	Men	63	0.000261, 0.000294, 0.000331, 0.000372, 0.000419
	White	Men	64	0.000293, 0.000326, 0.000362, 0.000402, 0.000447
	White	Men	65	0.000328, 0.000361, 0.000396, 0.000436, 0.000479
	White	Men	66	0.000365, 0.000399, 0.000436, 0.000477, 0.000521
	White	Men	67	0.000406, 0.000443, 0.000484, 0.000528, 0.000576
	White	Men	68	0.000451, 0.000494, 0.00054, 0.00059, 0.000646
	White	Men	69	0.000503, 0.000552, 0.000606, 0.000666, 0.000732
	White	Men	70	0.000563, 0.000621, 0.000684, 0.000755, 0.000833
	White	Men	71	0.000631, 0.0007, 0.000774, 0.000857, 0.000949
	White	Men	72	0.000709, 0.000789, 0.000876, 0.000973, 0.00108
	White	Men	73	0.000796, 0.000889, 0.000991, 0.00111, 0.00123
	White	Men	74	0.000892, 0.001, 0.00112, 0.00126, 0.00141
	White	Men	75	0.001, 0.00113, 0.00127, 0.00143, 0.00161
	White	Men	76	0.00112, 0.00127, 0.00144, 0.00163, 0.00184
	White	Men	77	0.00126, 0.00144, 0.00164, 0.00186, 0.00212
	White	Men	78	0.00142, 0.00163, 0.00187, 0.00215, 0.00247
	White	Men	79	0.00159, 0.00185, 0.00214, 0.00248, 0.00288

Variable	Race/ethnicity	Sex	Age	Distribution
	White	Men	80	0.00178, 0.00209, 0.00245, 0.00287, 0.00338
	White	Men	81	0.00199, 0.00236, 0.00279, 0.00331, 0.00393
	White	Men	82	0.00221, 0.00265, 0.00316, 0.00378, 0.00453
	White	Men	83	0.00243, 0.00294, 0.00354, 0.00428, 0.00517
	White	Men	84	0.00264, 0.00322, 0.00393, 0.00478, 0.00583
	White	Women	30	0.00000553, 0.00000751, 0.0000102, 0.0000138, 0.0000187
	White	Women	31	0.00000641, 0.00000862, 0.0000115, 0.0000155, 0.0000208
	White	Women	32	0.00000747, 0.00000991, 0.0000131, 0.0000173, 0.000023
	White	Women	33	0.0000087, 0.0000114, 0.0000148, 0.0000193, 0.0000253
	White	Women	34	0.0000101, 0.000013, 0.0000167, 0.0000215, 0.0000278
	White	Women	35	0.0000116, 0.0000147, 0.0000188, 0.0000238, 0.0000304
	White	Women	36	0.0000132, 0.0000166, 0.0000209, 0.0000263, 0.0000331
	White	Women	37	0.000015, 0.0000186, 0.0000232, 0.0000288, 0.0000359
	White	Women	38	0.0000168, 0.0000208, 0.0000256, 0.0000315, 0.0000388
	White	Women	39	0.0000188, 0.000023, 0.0000281, 0.0000343, 0.0000419
	White	Women	40	0.000021, 0.0000254, 0.0000307, 0.0000371, 0.0000449
	White	Women	41	0.0000234, 0.0000279, 0.0000334, 0.0000398, 0.0000476
	White	Women	42	0.0000261, 0.0000308, 0.0000362, 0.0000426, 0.0000502
	White	Women	43	0.000029, 0.0000338, 0.0000393, 0.0000457, 0.0000532
	White	Women	44	0.000032, 0.000037, 0.0000428, 0.0000494, 0.0000572
	White	Women	45	0.0000349, 0.0000404, 0.0000467, 0.0000539, 0.0000623
	White	Women	46	0.000038, 0.0000441, 0.0000511, 0.0000591, 0.0000686
	White	Women	47	0.0000412, 0.0000481, 0.000056, 0.0000652, 0.0000761
	White	Women	48	0.0000446, 0.0000524, 0.0000616, 0.0000723, 0.0000851
	White	Women	49	0.0000481, 0.0000571, 0.0000678, 0.0000804, 0.0000956
	White	Women	50	0.000052, 0.0000623, 0.0000746, 0.0000893, 0.000107
	White	Women	51	0.0000568, 0.0000684, 0.0000821, 0.0000987, 0.000119
	White	Women	52	0.0000628, 0.0000753, 0.0000902, 0.000108, 0.00013
	White	Women	53	0.00007, 0.0000832, 0.0000988, 0.000117, 0.000139
	White	Women	54	0.0000779, 0.0000917, 0.000108, 0.000127, 0.000149
	White	Women	55	0.0000866, 0.000101, 0.000117, 0.000136, 0.000159
	White	Women	56	0.000096, 0.000111, 0.000127, 0.000147, 0.000169
	White	Women	57	0.000106, 0.000121, 0.000138, 0.000158, 0.00018
	White	Women	58	0.000118, 0.000134, 0.000151, 0.00017, 0.000192
	White	Women	59	0.000131, 0.000147, 0.000165, 0.000185, 0.000207
	White	Women	60	0.000146, 0.000163, 0.000181, 0.000202, 0.000225
	White	Women	61	0.000162, 0.00018, 0.000199, 0.000221, 0.000246
	White	Women	62	0.000178, 0.000198, 0.00022, 0.000244, 0.00027
	White	Women	63	0.000197, 0.000218, 0.000242, 0.000268, 0.000298
	White	Women	64	0.000217, 0.000241, 0.000267, 0.000295, 0.000327
	White	Women	65	0.000241, 0.000267, 0.000295, 0.000326, 0.000361
	White	Women	66	0.000268, 0.000296, 0.000327, 0.000362, 0.0004
	White	Women	67	0.000299, 0.000331, 0.000366, 0.000405, 0.000448

Variable	Race/ethnicity	Sex	Age	Distribution
	White	Women	68	0.000338, 0.000374, 0.000413, 0.000457, 0.000505
	White	Women	69	0.000385, 0.000426, 0.00047, 0.000519, 0.000573
	White	Women	70	0.000443, 0.000488, 0.000538, 0.000593, 0.000655
	White	Women	71	0.00051, 0.000562, 0.00062, 0.000683, 0.000753
	White	Women	72	0.000586, 0.000648, 0.000715, 0.000789, 0.000872
	White	Women	73	0.000672, 0.000745, 0.000825, 0.000914, 0.00101
	White	Women	74	0.000772, 0.000858, 0.000951, 0.00106, 0.00117
	White	Women	75	0.00089, 0.000988, 0.0011, 0.00122, 0.00135
	White	Women	76	0.00102, 0.00114, 0.00127, 0.0014, 0.00156
	White	Women	77	0.00118, 0.00131, 0.00146, 0.00163, 0.00182
	White	Women	78	0.00134, 0.00151, 0.0017, 0.0019, 0.00214
	White	Women	79	0.00153, 0.00174, 0.00197, 0.00223, 0.00254
	White	Women	80	0.00175, 0.002, 0.00229, 0.00262, 0.00299
	White	Women	81	0.00201, 0.00231, 0.00265, 0.00305, 0.00351
	White	Women	82	0.00229, 0.00265, 0.00305, 0.00353, 0.00408
	White	Women	83	0.00259, 0.003, 0.00348, 0.00404, 0.00469
	White	Women	84	0.00289, 0.00337, 0.00392, 0.00457, 0.00533
Stroke mortality rates for 2022 (0.01, 0.2, 0.5, 0.8. 0.99 percentiles of the empirical distribution produced during forecasting)				
	Black	Men	30	0.0000167, 0.0000225, 0.0000303, 0.0000407, 0.0000548
	Black	Men	31	0.0000205, 0.0000269, 0.0000353, 0.0000463, 0.0000609
	Black	Men	32	0.0000244, 0.0000317, 0.000041, 0.0000531, 0.0000689
	Black	Men	33	0.0000286, 0.0000368, 0.0000474, 0.0000611, 0.0000788
	Black	Men	34	0.0000329, 0.0000423, 0.0000544, 0.0000699, 0.0000901
	Black	Men	35	0.0000375, 0.0000482, 0.0000618, 0.0000793, 0.000102
	Black	Men	36	0.0000426, 0.0000545, 0.0000696, 0.0000889, 0.000114
	Black	Men	37	0.0000484, 0.0000614, 0.0000777, 0.0000983, 0.000125
	Black	Men	38	0.0000549, 0.0000688, 0.000086, 0.000107, 0.000135
	Black	Men	39	0.0000619, 0.0000766, 0.0000946, 0.000117, 0.000144
	Black	Men	40	0.0000692, 0.0000848, 0.000104, 0.000127, 0.000155
	Black	Men	41	0.0000775, 0.0000939, 0.000113, 0.000137, 0.000166
	Black	Men	42	0.0000875, 0.000104, 0.000124, 0.000148, 0.000177
	Black	Men	43	0.0000981, 0.000116, 0.000137, 0.000162, 0.000191
	Black	Men	44	0.000108, 0.000128, 0.000151, 0.000179, 0.000213
	Black	Men	45	0.000117, 0.00014, 0.000168, 0.000201, 0.00024
	Black	Men	46	0.000129, 0.000155, 0.000187, 0.000224, 0.000269
	Black	Men	47	0.000143, 0.000173, 0.000208, 0.00025, 0.000301
	Black	Men	48	0.000158, 0.000191, 0.000232, 0.000282, 0.000342
	Black	Men	49	0.000174, 0.000213, 0.000261, 0.000318, 0.00039
	Black	Men	50	0.000197, 0.000241, 0.000294, 0.000358, 0.000437
	Black	Men	51	0.00023, 0.000276, 0.000331, 0.000398, 0.000478
	Black	Men	52	0.00027, 0.000318, 0.000374, 0.00044, 0.000518
	Black	Men	53	0.000312, 0.000362, 0.000419, 0.000486, 0.000564
	Black	Men	54	0.000352, 0.000405, 0.000466, 0.000536, 0.000618

Variable	Race/ethnicity	Sex	Age	Distribution
	Black	Men	55	0.00039, 0.000448, 0.000514, 0.000589, 0.000677
	Black	Men	56	0.000426, 0.000489, 0.000561, 0.000643, 0.000739
	Black	Men	57	0.000462, 0.000531, 0.000609, 0.000698, 0.000801
	Black	Men	58	0.000504, 0.000576, 0.000658, 0.000751, 0.000859
	Black	Men	59	0.000549, 0.000625, 0.000709, 0.000805, 0.000915
	Black	Men	60	0.000589, 0.000671, 0.000762, 0.000866, 0.000986
	Black	Men	61	0.000623, 0.000714, 0.000816, 0.000934, 0.00107
	Black	Men	62	0.000661, 0.000759, 0.000871, 0.000998, 0.00115
	Black	Men	63	0.000711, 0.000812, 0.000925, 0.00105, 0.0012
	Black	Men	64	0.000769, 0.000869, 0.000981, 0.00111, 0.00125
	Black	Men	65	0.000823, 0.000927, 0.00104, 0.00117, 0.00132
	Black	Men	66	0.000873, 0.000986, 0.00111, 0.00126, 0.00142
	Black	Men	67	0.000935, 0.00106, 0.0012, 0.00135, 0.00153
	Black	Men	68	0.00102, 0.00115, 0.00129, 0.00146, 0.00164
	Black	Men	69	0.00111, 0.00125, 0.0014, 0.00157, 0.00177
	Black	Men	70	0.00122, 0.00136, 0.00153, 0.00171, 0.00192
	Black	Men	71	0.00132, 0.00148, 0.00166, 0.00186, 0.00208
	Black	Men	72	0.00143, 0.00161, 0.0018, 0.00202, 0.00226
	Black	Men	73	0.00154, 0.00174, 0.00195, 0.00219, 0.00247
	Black	Men	74	0.00165, 0.00187, 0.00211, 0.00239, 0.00271
	Black	Men	75	0.00176, 0.00201, 0.00229, 0.00261, 0.00298
	Black	Men	76	0.0019, 0.00218, 0.00249, 0.00285, 0.00327
	Black	Men	77	0.00207, 0.00237, 0.00271, 0.00311, 0.00357
	Black	Men	78	0.00225, 0.00258, 0.00296, 0.0034, 0.0039
	Black	Men	79	0.00244, 0.00281, 0.00323, 0.00371, 0.00428
	Black	Men	80	0.00263, 0.00304, 0.00351, 0.00406, 0.00469
	Black	Men	81	0.00281, 0.00327, 0.00379, 0.0044, 0.00512
	Black	Men	82	0.003, 0.0035, 0.00407, 0.00474, 0.00553
	Black	Men	83	0.00319, 0.00372, 0.00435, 0.00507, 0.00593
	Black	Men	84	0.00336, 0.00394, 0.00461, 0.00539, 0.00632
	Black	Women	30	0.0000116, 0.0000169, 0.0000244, 0.0000353, 0.0000512
	Black	Women	31	0.0000133, 0.0000194, 0.000028, 0.0000405, 0.0000588
	Black	Women	32	0.0000151, 0.000022, 0.0000321, 0.0000466, 0.0000681
	Black	Women	33	0.0000169, 0.0000249, 0.0000366, 0.0000537, 0.0000791
	Black	Women	34	0.0000188, 0.000028, 0.0000415, 0.0000616, 0.0000917
	Black	Women	35	0.000021, 0.0000314, 0.0000469, 0.0000701, 0.000105
	Black	Women	36	0.0000237, 0.0000355, 0.0000528, 0.0000785, 0.000117
	Black	Women	37	0.0000274, 0.0000403, 0.000059, 0.0000864, 0.000127
	Black	Women	38	0.0000319, 0.0000458, 0.0000656, 0.0000939, 0.000135
	Black	Women	39	0.0000369, 0.0000519, 0.0000726, 0.000102, 0.000143
	Black	Women	40	0.0000421, 0.0000582, 0.0000801, 0.00011, 0.000152
	Black	Women	41	0.0000476, 0.0000648, 0.0000879, 0.000119, 0.000162
	Black	Women	42	0.0000538, 0.000072, 0.0000962, 0.000129, 0.000172

Variable	Race/ethnicity	Sex	Age	Distribution
	Black	Women	43	0.0000608, 0.0000801, 0.000105, 0.000138, 0.000182
	Black	Women	44	0.000068, 0.0000886, 0.000115, 0.00015, 0.000195
	Black	Women	45	0.0000756, 0.0000979, 0.000126, 0.000163, 0.000211
	Black	Women	46	0.0000848, 0.000109, 0.000139, 0.000178, 0.000228
	Black	Women	47	0.0000966, 0.000122, 0.000153, 0.000193, 0.000243
	Black	Women	48	0.00011, 0.000137, 0.000169, 0.000208, 0.000258
	Black	Women	49	0.000125, 0.000152, 0.000185, 0.000226, 0.000275
	Black	Women	50	0.000138, 0.000168, 0.000203, 0.000245, 0.000298
	Black	Women	51	0.000152, 0.000184, 0.000221, 0.000266, 0.000321
	Black	Women	52	0.000168, 0.000201, 0.00024, 0.000286, 0.000343
	Black	Women	53	0.000185, 0.000219, 0.00026, 0.000308, 0.000365
	Black	Women	54	0.0002, 0.000237, 0.000281, 0.000332, 0.000393
	Black	Women	55	0.000216, 0.000256, 0.000303, 0.000359, 0.000427
	Black	Women	56	0.000234, 0.000278, 0.000329, 0.00039, 0.000462
	Black	Women	57	0.000257, 0.000304, 0.000357, 0.000421, 0.000497
	Black	Women	58	0.000286, 0.000334, 0.000389, 0.000453, 0.000529
	Black	Women	59	0.000318, 0.000367, 0.000423, 0.000488, 0.000562
	Black	Women	60	0.000349, 0.000401, 0.000459, 0.000526, 0.000603
	Black	Women	61	0.000376, 0.000432, 0.000495, 0.000568, 0.000653
	Black	Women	62	0.0004, 0.000461, 0.000531, 0.000612, 0.000706
	Black	Women	63	0.000425, 0.000491, 0.000567, 0.000655, 0.000758
	Black	Women	64	0.000453, 0.000524, 0.000604, 0.000697, 0.000805
	Black	Women	65	0.000488, 0.000561, 0.000644, 0.000739, 0.00085
	Black	Women	66	0.000529, 0.000605, 0.00069, 0.000787, 0.000899
	Black	Women	67	0.000579, 0.000657, 0.000745, 0.000844, 0.000957
	Black	Women	68	0.00064, 0.000721, 0.000811, 0.000913, 0.00103
	Black	Women	69	0.000712, 0.000797, 0.000891, 0.000996, 0.00112
	Black	Women	70	0.000794, 0.000886, 0.000986, 0.0011, 0.00122
	Black	Women	71	0.000884, 0.000986, 0.0011, 0.00122, 0.00136
	Black	Women	72	0.000983, 0.0011, 0.00123, 0.00137, 0.00153
	Black	Women	73	0.0011, 0.00123, 0.00137, 0.00153, 0.00171
	Black	Women	74	0.00123, 0.00138, 0.00154, 0.00172, 0.00192
	Black	Women	75	0.00138, 0.00154, 0.00172, 0.00193, 0.00216
	Black	Women	76	0.00153, 0.00172, 0.00194, 0.00218, 0.00245
	Black	Women	77	0.00169, 0.00192, 0.00218, 0.00247, 0.00281
	Black	Women	78	0.00185, 0.00213, 0.00245, 0.00282, 0.00324
	Black	Women	79	0.00204, 0.00237, 0.00276, 0.0032, 0.00372
	Black	Women	80	0.00226, 0.00264, 0.00309, 0.0036, 0.00422
	Black	Women	81	0.0025, 0.00294, 0.00344, 0.00403, 0.00473
	Black	Women	82	0.00275, 0.00324, 0.00381, 0.00448, 0.00527
	Black	Women	83	0.00299, 0.00354, 0.00418, 0.00495, 0.00585
	Black	Women	84	0.00322, 0.00383, 0.00456, 0.00542, 0.00646
	Hispanic	Men	30	0.00000766, 0.0000105, 0.0000143, 0.0000195, 0.0000266

Variable	Race/ethnicity	Sex	Age	Distribution
	Hispanic	Men	31	0.00000918, 0.0000122, 0.0000162, 0.0000214, 0.0000285
	Hispanic	Men	32	0.0000109, 0.0000141, 0.0000183, 0.0000237, 0.0000308
	Hispanic	Men	33	0.0000127, 0.0000162, 0.0000207, 0.0000264, 0.0000338
	Hispanic	Men	34	0.0000146, 0.0000185, 0.0000234, 0.0000296, 0.0000375
	Hispanic	Men	35	0.0000166, 0.000021, 0.0000263, 0.0000331, 0.0000417
	Hispanic	Men	36	0.0000189, 0.0000237, 0.0000295, 0.0000368, 0.000046
	Hispanic	Men	37	0.0000216, 0.0000267, 0.000033, 0.0000407, 0.0000504
	Hispanic	Men	38	0.0000245, 0.00003, 0.0000367, 0.0000448, 0.0000549
	Hispanic	Men	39	0.0000276, 0.0000335, 0.0000406, 0.0000493, 0.0000599
	Hispanic	Men	40	0.0000308, 0.0000372, 0.0000448, 0.0000541, 0.0000653
	Hispanic	Men	41	0.0000342, 0.0000411, 0.0000493, 0.0000591, 0.0000711
	Hispanic	Men	42	0.000038, 0.0000454, 0.000054, 0.0000644, 0.0000769
	Hispanic	Men	43	0.0000421, 0.00005, 0.0000592, 0.0000702, 0.0000833
	Hispanic	Men	44	0.0000463, 0.0000549, 0.0000649, 0.0000768, 0.0000911
	Hispanic	Men	45	0.0000509, 0.0000603, 0.0000714, 0.0000844, 0.0001
	Hispanic	Men	46	0.0000562, 0.0000666, 0.0000786, 0.0000929, 0.00011
	Hispanic	Men	47	0.0000625, 0.0000738, 0.0000869, 0.000102, 0.000121
	Hispanic	Men	48	0.0000699, 0.0000822, 0.0000965, 0.000113, 0.000133
	Hispanic	Men	49	0.0000787, 0.000092, 0.000107, 0.000125, 0.000146
	Hispanic	Men	50	0.0000896, 0.000104, 0.00012, 0.000138, 0.00016
	Hispanic	Men	51	0.000102, 0.000117, 0.000134, 0.000153, 0.000175
	Hispanic	Men	52	0.000116, 0.000132, 0.000149, 0.000169, 0.000192
	Hispanic	Men	53	0.000128, 0.000146, 0.000166, 0.000189, 0.000215
	Hispanic	Men	54	0.000138, 0.000159, 0.000184, 0.000212, 0.000244
	Hispanic	Men	55	0.000148, 0.000173, 0.000202, 0.000236, 0.000277
	Hispanic	Men	56	0.000159, 0.000188, 0.000221, 0.000261, 0.000308
	Hispanic	Men	57	0.000173, 0.000205, 0.000241, 0.000285, 0.000337
	Hispanic	Men	58	0.00019, 0.000224, 0.000263, 0.000308, 0.000362
	Hispanic	Men	59	0.000211, 0.000245, 0.000285, 0.000331, 0.000385
	Hispanic	Men	60	0.000234, 0.000269, 0.000309, 0.000354, 0.000408
	Hispanic	Men	61	0.000258, 0.000293, 0.000334, 0.000379, 0.000432
	Hispanic	Men	62	0.000281, 0.000318, 0.00036, 0.000407, 0.000461
	Hispanic	Men	63	0.000303, 0.000343, 0.000388, 0.000438, 0.000496
	Hispanic	Men	64	0.000326, 0.000369, 0.000418, 0.000473, 0.000536
	Hispanic	Men	65	0.000351, 0.000398, 0.000452, 0.000512, 0.000582
	Hispanic	Men	66	0.000379, 0.000431, 0.000491, 0.000558, 0.000636
	Hispanic	Men	67	0.00041, 0.000469, 0.000536, 0.000612, 0.000701
	Hispanic	Men	68	0.000447, 0.000514, 0.000589, 0.000676, 0.000777
	Hispanic	Men	69	0.000492, 0.000567, 0.000652, 0.00075, 0.000864
	Hispanic	Men	70	0.000547, 0.00063, 0.000725, 0.000834, 0.000961
	Hispanic	Men	71	0.000612, 0.000704, 0.000808, 0.000929, 0.00107
	Hispanic	Men	72	0.000686, 0.000788, 0.000903, 0.00104, 0.00119
	Hispanic	Men	73	0.000768, 0.000881, 0.00101, 0.00116, 0.00133

Variable	Race/ethnicity	Sex	Age	Distribution
	Hispanic	Men	74	0.000859, 0.000986, 0.00113, 0.0013, 0.00149
	Hispanic	Men	75	0.000961, 0.0011, 0.00127, 0.00145, 0.00167
	Hispanic	Men	76	0.00107, 0.00124, 0.00142, 0.00163, 0.00187
	Hispanic	Men	77	0.0012, 0.00138, 0.00159, 0.00183, 0.00211
	Hispanic	Men	78	0.00134, 0.00155, 0.00179, 0.00206, 0.00238
	Hispanic	Men	79	0.00148, 0.00173, 0.002, 0.00232, 0.0027
	Hispanic	Men	80	0.00164, 0.00192, 0.00224, 0.00262, 0.00306
	Hispanic	Men	81	0.0018, 0.00212, 0.00249, 0.00293, 0.00346
	Hispanic	Men	82	0.00195, 0.00232, 0.00275, 0.00327, 0.00389
	Hispanic	Men	83	0.0021, 0.00252, 0.00302, 0.00361, 0.00434
	Hispanic	Men	84	0.00225, 0.00272, 0.00328, 0.00396, 0.00479
	Hispanic	Women	30	0.00000432, 0.00000652, 0.00000979, 0.0000147, 0.0000222
	Hispanic	Women	31	0.0000051, 0.00000749, 0.000011, 0.0000161, 0.0000236
	Hispanic	Women	32	0.00000595, 0.0000086, 0.0000124, 0.0000178, 0.0000258
	Hispanic	Women	33	0.00000689, 0.00000984, 0.000014, 0.0000199, 0.0000285
	Hispanic	Women	34	0.00000798, 0.0000113, 0.0000159, 0.0000223, 0.0000315
	Hispanic	Women	35	0.0000093, 0.0000129, 0.0000179, 0.0000249, 0.0000346
	Hispanic	Women	36	0.0000109, 0.0000149, 0.0000202, 0.0000275, 0.0000375
	Hispanic	Women	37	0.0000127, 0.000017, 0.0000227, 0.0000302, 0.0000404
	Hispanic	Women	38	0.0000148, 0.0000194, 0.0000253, 0.0000331, 0.0000434
	Hispanic	Women	39	0.0000169, 0.0000219, 0.0000282, 0.0000362, 0.0000468
	Hispanic	Women	40	0.0000192, 0.0000245, 0.0000311, 0.0000396, 0.0000504
	Hispanic	Women	41	0.0000215, 0.0000272, 0.0000343, 0.0000432, 0.0000545
	Hispanic	Women	42	0.0000239, 0.00003, 0.0000376, 0.0000472, 0.0000593
	Hispanic	Women	43	0.0000263, 0.000033, 0.0000413, 0.0000517, 0.0000648
	Hispanic	Women	44	0.0000291, 0.0000364, 0.0000454, 0.0000566, 0.0000707
	Hispanic	Women	45	0.0000327, 0.0000405, 0.0000499, 0.0000616, 0.0000761
	Hispanic	Women	46	0.0000374, 0.0000453, 0.0000549, 0.0000665, 0.0000807
	Hispanic	Women	47	0.0000428, 0.0000509, 0.0000604, 0.0000718, 0.0000854
	Hispanic	Women	48	0.0000483, 0.0000567, 0.0000665, 0.0000781, 0.0000917
	Hispanic	Women	49	0.0000533, 0.0000626, 0.0000733, 0.0000859, 0.000101
	Hispanic	Women	50	0.0000578, 0.0000684, 0.0000808, 0.0000955, 0.000113
	Hispanic	Women	51	0.0000622, 0.0000745, 0.0000891, 0.000107, 0.000128
	Hispanic	Women	52	0.0000667, 0.000081, 0.0000982, 0.000119, 0.000145
	Hispanic	Women	53	0.0000716, 0.0000881, 0.000108, 0.000133, 0.000163
	Hispanic	Women	54	0.0000773, 0.0000959, 0.000119, 0.000147, 0.000182
	Hispanic	Women	55	0.0000842, 0.000105, 0.00013, 0.000161, 0.000201
	Hispanic	Women	56	0.0000925, 0.000115, 0.000142, 0.000176, 0.000218
	Hispanic	Women	57	0.000102, 0.000126, 0.000155, 0.000191, 0.000236
	Hispanic	Women	58	0.000112, 0.000138, 0.000169, 0.000207, 0.000255
	Hispanic	Women	59	0.000124, 0.000151, 0.000185, 0.000225, 0.000276
	Hispanic	Women	60	0.000136, 0.000165, 0.000201, 0.000245, 0.000299
	Hispanic	Women	61	0.000149, 0.000181, 0.000219, 0.000266, 0.000324

Variable	Race/ethnicity	Sex	Age	Distribution
	Hispanic	Women	62	0.000162, 0.000197, 0.000238, 0.000288, 0.00035
	Hispanic	Women	63	0.000176, 0.000214, 0.000258, 0.000312, 0.000379
	Hispanic	Women	64	0.000191, 0.000231, 0.00028, 0.000339, 0.000411
	Hispanic	Women	65	0.000206, 0.000251, 0.000304, 0.000369, 0.000449
	Hispanic	Women	66	0.000225, 0.000274, 0.000332, 0.000404, 0.000492
	Hispanic	Women	67	0.000246, 0.000301, 0.000366, 0.000446, 0.000544
	Hispanic	Women	68	0.000273, 0.000334, 0.000407, 0.000496, 0.000605
	Hispanic	Women	69	0.000308, 0.000375, 0.000456, 0.000554, 0.000676
	Hispanic	Women	70	0.000352, 0.000426, 0.000514, 0.000622, 0.000752
	Hispanic	Women	71	0.000408, 0.000488, 0.000583, 0.000697, 0.000834
	Hispanic	Women	72	0.000478, 0.000564, 0.000664, 0.000782, 0.000922
	Hispanic	Women	73	0.000562, 0.000653, 0.000757, 0.000878, 0.00102
	Hispanic	Women	74	0.000657, 0.000754, 0.000864, 0.00099, 0.00114
	Hispanic	Women	75	0.000762, 0.000868, 0.000988, 0.00112, 0.00128
	Hispanic	Women	76	0.000876, 0.000996, 0.00113, 0.00129, 0.00146
	Hispanic	Women	77	0.00101, 0.00114, 0.0013, 0.00148, 0.00168
	Hispanic	Women	78	0.00116, 0.00132, 0.00149, 0.00169, 0.00192
	Hispanic	Women	79	0.00134, 0.00151, 0.00171, 0.00194, 0.0022
	Hispanic	Women	80	0.00154, 0.00174, 0.00196, 0.00222, 0.00251
	Hispanic	Women	81	0.00173, 0.00197, 0.00223, 0.00254, 0.00289
	Hispanic	Women	82	0.00189, 0.00219, 0.00252, 0.00291, 0.00336
	Hispanic	Women	83	0.00203, 0.0024, 0.00282, 0.00332, 0.00391
	Hispanic	Women	84	0.00216, 0.0026, 0.00312, 0.00375, 0.00451
	White	Men	30	0.00000611, 0.00000816, 0.0000109, 0.0000145, 0.0000194
	White	Men	31	0.00000713, 0.00000935, 0.0000122, 0.000016, 0.000021
	White	Men	32	0.00000829, 0.0000107, 0.0000138, 0.0000177, 0.0000229
	White	Men	33	0.0000096, 0.0000122, 0.0000155, 0.0000196, 0.0000249
	White	Men	34	0.000011, 0.0000138, 0.0000173, 0.0000217, 0.0000272
	White	Men	35	0.0000126, 0.0000156, 0.0000193, 0.000024, 0.0000298
	White	Men	36	0.0000142, 0.0000175, 0.0000216, 0.0000265, 0.0000327
	White	Men	37	0.000016, 0.0000196, 0.000024, 0.0000293, 0.000036
	White	Men	38	0.0000178, 0.0000218, 0.0000266, 0.0000324, 0.0000396
	White	Men	39	0.0000198, 0.0000241, 0.0000293, 0.0000357, 0.0000435
	White	Men	40	0.000022, 0.0000267, 0.0000323, 0.0000391, 0.0000475
	White	Men	41	0.0000245, 0.0000295, 0.0000354, 0.0000426, 0.0000513
	White	Men	42	0.0000276, 0.0000327, 0.0000388, 0.0000461, 0.0000547
	White	Men	43	0.000031, 0.0000364, 0.0000426, 0.0000498, 0.0000584
	White	Men	44	0.0000348, 0.0000404, 0.0000468, 0.0000543, 0.000063
	White	Men	45	0.000039, 0.0000449, 0.0000517, 0.0000595, 0.0000685
	White	Men	46	0.0000437, 0.0000501, 0.0000573, 0.0000655, 0.000075
	White	Men	47	0.0000489, 0.0000558, 0.0000637, 0.0000726, 0.0000829
	White	Men	48	0.0000544, 0.0000622, 0.000071, 0.0000812, 0.0000928
	White	Men	49	0.0000601, 0.0000692, 0.0000795, 0.0000913, 0.000105

Variable	Race/ethnicity	Sex	Age	Distribution
	White	Men	50	0.0000665, 0.0000771, 0.0000891, 0.000103, 0.000119
	White	Men	51	0.0000743, 0.0000862, 0.0000999, 0.000116, 0.000134
	White	Men	52	0.0000837, 0.0000968, 0.000112, 0.000129, 0.00015
	White	Men	53	0.0000943, 0.000109, 0.000125, 0.000144, 0.000165
	White	Men	54	0.000105, 0.000121, 0.000139, 0.000159, 0.000183
	White	Men	55	0.000116, 0.000134, 0.000154, 0.000176, 0.000203
	White	Men	56	0.000127, 0.000147, 0.000169, 0.000196, 0.000226
	White	Men	57	0.000138, 0.000161, 0.000187, 0.000217, 0.000252
	White	Men	58	0.00015, 0.000176, 0.000205, 0.000239, 0.00028
	White	Men	59	0.000165, 0.000193, 0.000225, 0.000264, 0.000309
	White	Men	60	0.000182, 0.000212, 0.000248, 0.000289, 0.000337
	White	Men	61	0.000202, 0.000234, 0.000271, 0.000314, 0.000364
	White	Men	62	0.000226, 0.000259, 0.000297, 0.00034, 0.00039
	White	Men	63	0.000253, 0.000287, 0.000324, 0.000367, 0.000415
	White	Men	64	0.000284, 0.000317, 0.000354, 0.000395, 0.000441
	White	Men	65	0.000318, 0.000351, 0.000388, 0.000428, 0.000473
	White	Men	66	0.000354, 0.000389, 0.000426, 0.000468, 0.000514
	White	Men	67	0.000393, 0.000431, 0.000472, 0.000518, 0.000568
	White	Men	68	0.000436, 0.00048, 0.000527, 0.000579, 0.000637
	White	Men	69	0.000486, 0.000537, 0.000592, 0.000653, 0.000721
	White	Men	70	0.000544, 0.000603, 0.000668, 0.00074, 0.00082
	White	Men	71	0.000611, 0.00068, 0.000756, 0.00084, 0.000935
	White	Men	72	0.000686, 0.000766, 0.000855, 0.000954, 0.00107
	White	Men	73	0.000769, 0.000863, 0.000967, 0.00108, 0.00122
	White	Men	74	0.000861, 0.000971, 0.00109, 0.00123, 0.00139
	White	Men	75	0.000965, 0.00109, 0.00124, 0.0014, 0.00159
	White	Men	76	0.00108, 0.00123, 0.0014, 0.0016, 0.00182
	White	Men	77	0.00122, 0.0014, 0.0016, 0.00183, 0.0021
	White	Men	78	0.00137, 0.00158, 0.00183, 0.00211, 0.00244
	White	Men	79	0.00153, 0.00179, 0.00209, 0.00244, 0.00285
	White	Men	80	0.00171, 0.00202, 0.00239, 0.00283, 0.00334
	White	Men	81	0.00191, 0.00228, 0.00273, 0.00326, 0.00389
	White	Men	82	0.00213, 0.00256, 0.00308, 0.00371, 0.00448
	White	Men	83	0.00234, 0.00285, 0.00346, 0.0042, 0.0051
	White	Men	84	0.00255, 0.00313, 0.00383, 0.00469, 0.00576
	White	Women	30	0.0000054, 0.00000741, 0.0000101, 0.0000139, 0.000019
	White	Women	31	0.00000629, 0.00000852, 0.0000115, 0.0000155, 0.000021
	White	Women	32	0.00000734, 0.0000098, 0.0000131, 0.0000174, 0.0000232
	White	Women	33	0.00000856, 0.0000113, 0.0000148, 0.0000194, 0.0000255
	White	Women	34	0.00000991, 0.0000129, 0.0000167, 0.0000216, 0.000028
	White	Women	35	0.0000114, 0.0000146, 0.0000187, 0.0000239, 0.0000307
	White	Women	36	0.000013, 0.0000164, 0.0000208, 0.0000263, 0.0000334
	White	Women	37	0.0000146, 0.0000184, 0.000023, 0.0000288, 0.0000362

Variable	Race/ethnicity	Sex	Age	Distribution
	White	Women	38	0.0000164, 0.0000204, 0.0000253, 0.0000314, 0.0000391
	White	Women	39	0.0000183, 0.0000226, 0.0000278, 0.0000341, 0.0000421
	White	Women	40	0.0000204, 0.0000248, 0.0000303, 0.0000368, 0.0000449
	White	Women	41	0.0000227, 0.0000273, 0.0000328, 0.0000395, 0.0000475
	White	Women	42	0.0000254, 0.0000301, 0.0000356, 0.0000421, 0.0000498
	White	Women	43	0.0000282, 0.000033, 0.0000385, 0.000045, 0.0000526
	White	Women	44	0.0000311, 0.0000361, 0.0000418, 0.0000485, 0.0000564
	White	Women	45	0.0000339, 0.0000394, 0.0000456, 0.0000528, 0.0000613
	White	Women	46	0.0000369, 0.0000429, 0.0000499, 0.0000579, 0.0000674
	White	Women	47	0.0000401, 0.0000469, 0.0000547, 0.0000639, 0.0000747
	White	Women	48	0.0000434, 0.0000511, 0.0000601, 0.0000708, 0.0000834
	White	Women	49	0.0000468, 0.0000557, 0.0000662, 0.0000787, 0.0000937
	White	Women	50	0.0000507, 0.0000609, 0.000073, 0.0000875, 0.000105
	White	Women	51	0.0000555, 0.0000669, 0.0000804, 0.0000968, 0.000117
	White	Women	52	0.0000615, 0.0000738, 0.0000885, 0.000106, 0.000127
	White	Women	53	0.0000685, 0.0000816, 0.000097, 0.000115, 0.000137
	White	Women	54	0.0000763, 0.00009, 0.000106, 0.000125, 0.000147
	White	Women	55	0.0000848, 0.000099, 0.000115, 0.000134, 0.000157
	White	Women	56	0.000094, 0.000109, 0.000125, 0.000145, 0.000167
	White	Women	57	0.000104, 0.000119, 0.000136, 0.000156, 0.000178
	White	Women	58	0.000116, 0.000131, 0.000148, 0.000168, 0.00019
	White	Women	59	0.000128, 0.000144, 0.000162, 0.000182, 0.000205
	White	Women	60	0.000142, 0.000159, 0.000178, 0.000199, 0.000223
	White	Women	61	0.000157, 0.000176, 0.000196, 0.000218, 0.000244
	White	Women	62	0.000174, 0.000194, 0.000216, 0.00024, 0.000268
	White	Women	63	0.000191, 0.000213, 0.000237, 0.000264, 0.000294
	White	Women	64	0.000211, 0.000235, 0.000261, 0.000291, 0.000323
	White	Women	65	0.000234, 0.00026, 0.000288, 0.00032, 0.000356
	White	Women	66	0.00026, 0.000289, 0.00032, 0.000355, 0.000394
	White	Women	67	0.00029, 0.000322, 0.000358, 0.000397, 0.000441
	White	Women	68	0.000327, 0.000363, 0.000403, 0.000448, 0.000497
	White	Women	69	0.000373, 0.000414, 0.000459, 0.000509, 0.000565
	White	Women	70	0.000428, 0.000475, 0.000525, 0.000582, 0.000645
	White	Women	71	0.000493, 0.000546, 0.000605, 0.000669, 0.000742
	White	Women	72	0.000567, 0.000629, 0.000697, 0.000773, 0.000859
	White	Women	73	0.00065, 0.000723, 0.000804, 0.000895, 0.000996
	White	Women	74	0.000746, 0.000832, 0.000927, 0.00103, 0.00115
	White	Women	75	0.00086, 0.000959, 0.00107, 0.00119, 0.00133
	White	Women	76	0.00099, 0.00111, 0.00123, 0.00137, 0.00153
	White	Women	77	0.00114, 0.00127, 0.00142, 0.00159, 0.00179
	White	Women	78	0.0013, 0.00146, 0.00165, 0.00186, 0.0021
	White	Women	79	0.00148, 0.00169, 0.00192, 0.00218, 0.00249
	White	Women	80	0.00169, 0.00194, 0.00223, 0.00256, 0.00294

Variable	Race/ethnicity	Sex	Age	Distribution
	White	Women	81	0.00194, 0.00224, 0.00258, 0.00298, 0.00344
	White	Women	82	0.00221, 0.00257, 0.00297, 0.00345, 0.004
	White	Women	83	0.0025, 0.00291, 0.00339, 0.00395, 0.0046
	White	Women	84	0.00279, 0.00327, 0.00382, 0.00447, 0.00523
Stroke mortality rates for 2023 (0.01, 0.2, 0.5, 0.8. 0.99 percentiles of the empirical distribution produced during forecasting)				
	Black	Men	30	0.0000163, 0.0000222, 0.0000301, 0.0000409, 0.0000556
	Black	Men	31	0.00002, 0.0000266, 0.0000352, 0.0000465, 0.0000617
	Black	Men	32	0.000024, 0.0000314, 0.0000409, 0.0000533, 0.0000697
	Black	Men	33	0.0000281, 0.0000365, 0.0000473, 0.0000613, 0.0000796
	Black	Men	34	0.0000323, 0.0000419, 0.0000542, 0.0000701, 0.0000909
	Black	Men	35	0.0000368, 0.0000477, 0.0000615, 0.0000794, 0.000103
	Black	Men	36	0.0000418, 0.0000538, 0.0000692, 0.0000889, 0.000115
	Black	Men	37	0.0000474, 0.0000605, 0.0000771, 0.0000982, 0.000125
	Black	Men	38	0.0000536, 0.0000676, 0.0000852, 0.000107, 0.000135
	Black	Men	39	0.0000603, 0.0000752, 0.0000935, 0.000116, 0.000145
	Black	Men	40	0.0000673, 0.000083, 0.000102, 0.000126, 0.000155
	Black	Men	41	0.0000753, 0.0000918, 0.000112, 0.000136, 0.000165
	Black	Men	42	0.000085, 0.000102, 0.000122, 0.000146, 0.000175
	Black	Men	43	0.0000954, 0.000113, 0.000134, 0.000159, 0.000189
	Black	Men	44	0.000105, 0.000125, 0.000148, 0.000176, 0.00021
	Black	Men	45	0.000114, 0.000137, 0.000164, 0.000197, 0.000236
	Black	Men	46	0.000126, 0.000152, 0.000182, 0.000219, 0.000264
	Black	Men	47	0.000139, 0.000168, 0.000203, 0.000245, 0.000296
	Black	Men	48	0.000154, 0.000187, 0.000227, 0.000276, 0.000336
	Black	Men	49	0.00017, 0.000208, 0.000255, 0.000312, 0.000383
	Black	Men	50	0.000193, 0.000236, 0.000288, 0.000351, 0.00043
	Black	Men	51	0.000225, 0.000271, 0.000325, 0.000391, 0.000471
	Black	Men	52	0.000264, 0.000312, 0.000367, 0.000433, 0.000511
	Black	Men	53	0.000306, 0.000355, 0.000413, 0.000479, 0.000557
	Black	Men	54	0.000345, 0.000398, 0.000459, 0.00053, 0.000612
	Black	Men	55	0.000382, 0.00044, 0.000506, 0.000582, 0.000671
	Black	Men	56	0.000418, 0.000481, 0.000553, 0.000636, 0.000733
	Black	Men	57	0.000454, 0.000522, 0.0006, 0.00069, 0.000795
	Black	Men	58	0.000494, 0.000567, 0.000649, 0.000743, 0.000852
	Black	Men	59	0.000538, 0.000614, 0.000699, 0.000797, 0.000909
	Black	Men	60	0.000576, 0.000658, 0.000751, 0.000857, 0.000978
	Black	Men	61	0.000609, 0.0007, 0.000803, 0.000922, 0.00106
	Black	Men	62	0.000645, 0.000744, 0.000856, 0.000985, 0.00113
	Black	Men	63	0.000693, 0.000794, 0.000908, 0.00104, 0.00119
	Black	Men	64	0.000749, 0.000849, 0.000962, 0.00109, 0.00124
	Black	Men	65	0.0008, 0.000904, 0.00102, 0.00115, 0.0013
	Black	Men	66	0.000849, 0.000962, 0.00109, 0.00123, 0.0014
	Black	Men	67	0.000908, 0.00103, 0.00117, 0.00132, 0.0015

Variable	Race/ethnicity	Sex	Age	Distribution
	Black	Men	68	0.000985, 0.00112, 0.00126, 0.00143, 0.00162
	Black	Men	69	0.00108, 0.00122, 0.00137, 0.00154, 0.00174
	Black	Men	70	0.00118, 0.00132, 0.00149, 0.00168, 0.00189
	Black	Men	71	0.00128, 0.00144, 0.00162, 0.00182, 0.00205
	Black	Men	72	0.00139, 0.00156, 0.00176, 0.00198, 0.00222
	Black	Men	73	0.00149, 0.00169, 0.0019, 0.00215, 0.00242
	Black	Men	74	0.0016, 0.00182, 0.00206, 0.00234, 0.00266
	Black	Men	75	0.00171, 0.00195, 0.00223, 0.00255, 0.00292
	Black	Men	76	0.00184, 0.00212, 0.00243, 0.00279, 0.0032
	Black	Men	77	0.002, 0.0023, 0.00265, 0.00304, 0.0035
	Black	Men	78	0.00218, 0.00251, 0.00289, 0.00332, 0.00382
	Black	Men	79	0.00236, 0.00273, 0.00315, 0.00363, 0.0042
	Black	Men	80	0.00254, 0.00295, 0.00342, 0.00397, 0.00461
	Black	Men	81	0.00272, 0.00317, 0.0037, 0.00431, 0.00503
	Black	Men	82	0.0029, 0.0034, 0.00397, 0.00464, 0.00543
	Black	Men	83	0.00308, 0.00361, 0.00424, 0.00496, 0.00583
	Black	Men	84	0.00325, 0.00382, 0.00449, 0.00528, 0.00621
	Black	Women	30	0.0000114, 0.0000167, 0.0000243, 0.0000355, 0.000052
	Black	Women	31	0.0000131, 0.0000192, 0.0000279, 0.0000407, 0.0000596
	Black	Women	32	0.0000149, 0.0000219, 0.000032, 0.0000468, 0.0000688
	Black	Women	33	0.0000167, 0.0000247, 0.0000365, 0.0000539, 0.0000798
	Black	Women	34	0.0000186, 0.0000278, 0.0000414, 0.0000618, 0.0000924
	Black	Women	35	0.0000207, 0.0000312, 0.0000468, 0.0000702, 0.000106
	Black	Women	36	0.0000234, 0.0000351, 0.0000525, 0.0000786, 0.000118
	Black	Women	37	0.0000269, 0.0000398, 0.0000586, 0.0000864, 0.000128
	Black	Women	38	0.0000313, 0.0000452, 0.0000651, 0.0000938, 0.000136
	Black	Women	39	0.0000361, 0.000051, 0.000072, 0.000101, 0.000144
	Black	Women	40	0.0000409, 0.000057, 0.0000791, 0.00011, 0.000153
	Black	Women	41	0.0000461, 0.0000633, 0.0000867, 0.000119, 0.000163
	Black	Women	42	0.0000519, 0.0000702, 0.0000947, 0.000128, 0.000173
	Black	Women	43	0.0000586, 0.000078, 0.000103, 0.000137, 0.000183
	Black	Women	44	0.0000655, 0.0000862, 0.000113, 0.000148, 0.000195
	Black	Women	45	0.0000729, 0.0000952, 0.000124, 0.000161, 0.000211
	Black	Women	46	0.0000818, 0.000106, 0.000136, 0.000176, 0.000227
	Black	Women	47	0.0000932, 0.000118, 0.00015, 0.00019, 0.000241
	Black	Women	48	0.000107, 0.000133, 0.000165, 0.000205, 0.000256
	Black	Women	49	0.00012, 0.000148, 0.000182, 0.000223, 0.000274
	Black	Women	50	0.000134, 0.000163, 0.000199, 0.000243, 0.000297
	Black	Women	51	0.000147, 0.000179, 0.000217, 0.000264, 0.00032
	Black	Women	52	0.000163, 0.000196, 0.000236, 0.000284, 0.000342
	Black	Women	53	0.000179, 0.000214, 0.000256, 0.000305, 0.000365
	Black	Women	54	0.000195, 0.000232, 0.000277, 0.000329, 0.000393
	Black	Women	55	0.00021, 0.000251, 0.000299, 0.000357, 0.000427

Variable	Race/ethnicity	Sex	Age	Distribution
	Black	Women	56	0.000228, 0.000272, 0.000325, 0.000387, 0.000463
	Black	Women	57	0.000251, 0.000297, 0.000353, 0.000418, 0.000496
	Black	Women	58	0.000279, 0.000327, 0.000384, 0.00045, 0.000528
	Black	Women	59	0.00031, 0.00036, 0.000417, 0.000483, 0.000561
	Black	Women	60	0.000341, 0.000393, 0.000452, 0.000521, 0.0006
	Black	Women	61	0.000367, 0.000423, 0.000487, 0.000562, 0.000648
	Black	Women	62	0.00039, 0.000452, 0.000522, 0.000604, 0.0007
	Black	Women	63	0.000414, 0.00048, 0.000557, 0.000646, 0.000749
	Black	Women	64	0.000441, 0.000511, 0.000592, 0.000686, 0.000795
	Black	Women	65	0.000474, 0.000547, 0.00063, 0.000727, 0.000839
	Black	Women	66	0.000514, 0.000589, 0.000675, 0.000773, 0.000886
	Black	Women	67	0.000562, 0.00064, 0.000728, 0.000828, 0.000943
	Black	Women	68	0.00062, 0.000701, 0.000792, 0.000895, 0.00101
	Black	Women	69	0.00069, 0.000775, 0.000871, 0.000978, 0.0011
	Black	Women	70	0.000769, 0.000861, 0.000963, 0.00108, 0.00121
	Black	Women	71	0.000856, 0.000958, 0.00107, 0.0012, 0.00134
	Black	Women	72	0.000952, 0.00107, 0.0012, 0.00134, 0.0015
	Black	Women	73	0.00106, 0.00119, 0.00134, 0.0015, 0.00169
	Black	Women	74	0.00119, 0.00134, 0.0015, 0.00168, 0.00189
	Black	Women	75	0.00133, 0.0015, 0.00168, 0.00189, 0.00212
	Black	Women	76	0.00148, 0.00167, 0.00189, 0.00213, 0.0024
	Black	Women	77	0.00163, 0.00186, 0.00212, 0.00242, 0.00276
	Black	Women	78	0.00179, 0.00207, 0.00239, 0.00275, 0.00318
	Black	Women	79	0.00197, 0.0023, 0.00268, 0.00313, 0.00365
	Black	Women	80	0.00218, 0.00256, 0.00301, 0.00353, 0.00414
	Black	Women	81	0.00242, 0.00285, 0.00335, 0.00394, 0.00465
	Black	Women	82	0.00265, 0.00314, 0.00371, 0.00438, 0.00518
	Black	Women	83	0.00288, 0.00343, 0.00407, 0.00484, 0.00576
	Black	Women	84	0.0031, 0.00371, 0.00443, 0.0053, 0.00635
	Hispanic	Men	30	0.00000749, 0.0000103, 0.0000142, 0.0000195, 0.000027
	Hispanic	Men	31	0.000009, 0.0000121, 0.0000161, 0.0000215, 0.0000288
	Hispanic	Men	32	0.0000107, 0.000014, 0.0000182, 0.0000238, 0.0000311
	Hispanic	Men	33	0.0000125, 0.0000161, 0.0000206, 0.0000265, 0.0000341
	Hispanic	Men	34	0.0000144, 0.0000183, 0.0000233, 0.0000297, 0.0000378
	Hispanic	Men	35	0.0000163, 0.0000207, 0.0000262, 0.0000332, 0.0000421
	Hispanic	Men	36	0.0000186, 0.0000234, 0.0000294, 0.0000369, 0.0000465
	Hispanic	Men	37	0.0000211, 0.0000263, 0.0000327, 0.0000408, 0.0000509
	Hispanic	Men	38	0.0000239, 0.0000295, 0.0000364, 0.0000448, 0.0000553
	Hispanic	Men	39	0.0000268, 0.0000329, 0.0000402, 0.0000491, 0.0000602
	Hispanic	Men	40	0.0000299, 0.0000364, 0.0000442, 0.0000538, 0.0000655
	Hispanic	Men	41	0.0000332, 0.0000402, 0.0000485, 0.0000586, 0.0000709
	Hispanic	Men	42	0.0000369, 0.0000443, 0.0000531, 0.0000635, 0.0000762
	Hispanic	Men	43	0.0000409, 0.0000488, 0.000058, 0.000069, 0.0000823

Variable	Race/ethnicity	Sex	Age	Distribution
	Hispanic	Men	44	0.000045, 0.0000535, 0.0000635, 0.0000754, 0.0000897
	Hispanic	Men	45	0.0000495, 0.0000588, 0.0000697, 0.0000827, 0.0000983
	Hispanic	Men	46	0.0000546, 0.0000648, 0.0000768, 0.000091, 0.000108
	Hispanic	Men	47	0.0000607, 0.0000719, 0.0000849, 0.0001, 0.000119
	Hispanic	Men	48	0.000068, 0.0000801, 0.0000942, 0.000111, 0.000131
	Hispanic	Men	49	0.0000767, 0.0000897, 0.000105, 0.000123, 0.000144
	Hispanic	Men	50	0.0000873, 0.000101, 0.000117, 0.000136, 0.000157
	Hispanic	Men	51	0.0001, 0.000115, 0.000131, 0.00015, 0.000172
	Hispanic	Men	52	0.000113, 0.000129, 0.000146, 0.000166, 0.000189
	Hispanic	Men	53	0.000125, 0.000143, 0.000163, 0.000186, 0.000212
	Hispanic	Men	54	0.000135, 0.000156, 0.000181, 0.000209, 0.000242
	Hispanic	Men	55	0.000145, 0.00017, 0.000199, 0.000233, 0.000274
	Hispanic	Men	56	0.000156, 0.000184, 0.000218, 0.000258, 0.000306
	Hispanic	Men	57	0.000169, 0.000201, 0.000238, 0.000282, 0.000334
	Hispanic	Men	58	0.000186, 0.00022, 0.000259, 0.000305, 0.00036
	Hispanic	Men	59	0.000206, 0.000241, 0.000281, 0.000328, 0.000382
	Hispanic	Men	60	0.000229, 0.000264, 0.000304, 0.000351, 0.000405
	Hispanic	Men	61	0.000252, 0.000288, 0.000328, 0.000375, 0.000429
	Hispanic	Men	62	0.000274, 0.000312, 0.000354, 0.000402, 0.000457
	Hispanic	Men	63	0.000295, 0.000336, 0.000381, 0.000432, 0.00049
	Hispanic	Men	64	0.000317, 0.000361, 0.00041, 0.000465, 0.000529
	Hispanic	Men	65	0.000341, 0.000389, 0.000442, 0.000503, 0.000573
	Hispanic	Men	66	0.000368, 0.00042, 0.00048, 0.000547, 0.000626
	Hispanic	Men	67	0.000398, 0.000457, 0.000524, 0.0006, 0.000689
	Hispanic	Men	68	0.000434, 0.0005, 0.000576, 0.000663, 0.000764
	Hispanic	Men	69	0.000477, 0.000552, 0.000637, 0.000735, 0.00085
	Hispanic	Men	70	0.00053, 0.000613, 0.000708, 0.000817, 0.000945
	Hispanic	Men	71	0.000593, 0.000685, 0.000789, 0.00091, 0.00105
	Hispanic	Men	72	0.000665, 0.000766, 0.000882, 0.00101, 0.00117
	Hispanic	Men	73	0.000745, 0.000857, 0.000986, 0.00113, 0.0013
	Hispanic	Men	74	0.000832, 0.000959, 0.0011, 0.00127, 0.00146
	Hispanic	Men	75	0.000931, 0.00107, 0.00123, 0.00142, 0.00164
	Hispanic	Men	76	0.00104, 0.0012, 0.00138, 0.00159, 0.00184
	Hispanic	Men	77	0.00116, 0.00134, 0.00155, 0.00179, 0.00207
	Hispanic	Men	78	0.0013, 0.0015, 0.00174, 0.00202, 0.00234
	Hispanic	Men	79	0.00144, 0.00168, 0.00195, 0.00227, 0.00265
	Hispanic	Men	80	0.00159, 0.00186, 0.00218, 0.00256, 0.00301
	Hispanic	Men	81	0.00174, 0.00206, 0.00243, 0.00287, 0.0034
	Hispanic	Men	82	0.00189, 0.00225, 0.00269, 0.0032, 0.00382
	Hispanic	Men	83	0.00203, 0.00245, 0.00294, 0.00354, 0.00426
	Hispanic	Men	84	0.00217, 0.00264, 0.0032, 0.00388, 0.00471
	Hispanic	Women	30	0.00000424, 0.00000645, 0.00000975, 0.0000147, 0.0000224
	Hispanic	Women	31	0.00000502, 0.00000742, 0.0000109, 0.0000161, 0.0000238

Variable	Race/ethnicity	Sex	Age	Distribution
	Hispanic	Women	32	0.00000587, 0.00000852, 0.0000123, 0.0000179, 0.000026
	Hispanic	Women	33	0.0000068, 0.00000977, 0.000014, 0.00002, 0.0000287
	Hispanic	Women	34	0.00000788, 0.0000112, 0.0000158, 0.0000224, 0.0000317
	Hispanic	Women	35	0.00000918, 0.0000128, 0.0000179, 0.0000249, 0.0000348
	Hispanic	Women	36	0.0000107, 0.0000147, 0.0000201, 0.0000275, 0.0000377
	Hispanic	Women	37	0.0000125, 0.0000168, 0.0000225, 0.0000302, 0.0000406
	Hispanic	Women	38	0.0000145, 0.0000191, 0.0000251, 0.000033, 0.0000436
	Hispanic	Women	39	0.0000165, 0.0000215, 0.0000278, 0.000036, 0.0000468
	Hispanic	Women	40	0.0000187, 0.000024, 0.0000307, 0.0000392, 0.0000503
	Hispanic	Women	41	0.000021, 0.0000266, 0.0000337, 0.0000427, 0.0000542
	Hispanic	Women	42	0.0000233, 0.0000294, 0.0000369, 0.0000465, 0.0000586
	Hispanic	Women	43	0.0000257, 0.0000323, 0.0000405, 0.0000508, 0.0000639
	Hispanic	Women	44	0.0000284, 0.0000355, 0.0000444, 0.0000555, 0.0000695
	Hispanic	Women	45	0.0000318, 0.0000394, 0.0000488, 0.0000603, 0.0000747
	Hispanic	Women	46	0.0000363, 0.0000442, 0.0000536, 0.0000651, 0.0000792
	Hispanic	Women	47	0.0000416, 0.0000496, 0.000059, 0.0000703, 0.0000838
	Hispanic	Women	48	0.000047, 0.0000553, 0.000065, 0.0000764, 0.00009
	Hispanic	Women	49	0.0000519, 0.0000611, 0.0000717, 0.0000841, 0.0000989
	Hispanic	Women	50	0.0000564, 0.0000669, 0.0000791, 0.0000936, 0.000111
	Hispanic	Women	51	0.0000608, 0.0000729, 0.0000874, 0.000105, 0.000126
	Hispanic	Women	52	0.0000653, 0.0000794, 0.0000964, 0.000117, 0.000142
	Hispanic	Women	53	0.0000702, 0.0000864, 0.000106, 0.000131, 0.000161
	Hispanic	Women	54	0.0000758, 0.0000942, 0.000117, 0.000145, 0.00018
	Hispanic	Women	55	0.0000827, 0.000103, 0.000128, 0.000159, 0.000198
	Hispanic	Women	56	0.0000908, 0.000113, 0.00014, 0.000173, 0.000216
	Hispanic	Women	57	0.0001, 0.000124, 0.000153, 0.000189, 0.000233
	Hispanic	Women	58	0.00011, 0.000136, 0.000167, 0.000205, 0.000252
	Hispanic	Women	59	0.000121, 0.000149, 0.000182, 0.000223, 0.000273
	Hispanic	Women	60	0.000133, 0.000163, 0.000198, 0.000242, 0.000296
	Hispanic	Women	61	0.000145, 0.000177, 0.000216, 0.000263, 0.00032
	Hispanic	Women	62	0.000159, 0.000193, 0.000234, 0.000284, 0.000346
	Hispanic	Women	63	0.000172, 0.000209, 0.000254, 0.000307, 0.000373
	Hispanic	Women	64	0.000186, 0.000226, 0.000275, 0.000333, 0.000405
	Hispanic	Women	65	0.000201, 0.000245, 0.000298, 0.000362, 0.000441
	Hispanic	Women	66	0.000219, 0.000267, 0.000325, 0.000396, 0.000484
	Hispanic	Women	67	0.00024, 0.000293, 0.000358, 0.000437, 0.000534
	Hispanic	Women	68	0.000266, 0.000325, 0.000398, 0.000486, 0.000594
	Hispanic	Women	69	0.000299, 0.000365, 0.000445, 0.000543, 0.000663
	Hispanic	Women	70	0.000342, 0.000415, 0.000502, 0.000609, 0.000739
	Hispanic	Women	71	0.000396, 0.000476, 0.00057, 0.000682, 0.000819
	Hispanic	Women	72	0.000464, 0.000549, 0.000648, 0.000765, 0.000905
	Hispanic	Women	73	0.000545, 0.000635, 0.000738, 0.000859, 0.001
	Hispanic	Women	74	0.000637, 0.000733, 0.000843, 0.000968, 0.00111

Variable	Race/ethnicity	Sex	Age	Distribution
	Hispanic	Women	75	0.000738, 0.000844, 0.000963, 0.0011, 0.00126
	Hispanic	Women	76	0.000848, 0.000968, 0.0011, 0.00126, 0.00143
	Hispanic	Women	77	0.000973, 0.00111, 0.00127, 0.00144, 0.00165
	Hispanic	Women	78	0.00112, 0.00128, 0.00145, 0.00166, 0.00189
	Hispanic	Women	79	0.00129, 0.00147, 0.00167, 0.0019, 0.00216
	Hispanic	Women	80	0.00148, 0.00169, 0.00191, 0.00217, 0.00246
	Hispanic	Women	81	0.00167, 0.00191, 0.00218, 0.00248, 0.00284
	Hispanic	Women	82	0.00183, 0.00212, 0.00246, 0.00284, 0.0033
	Hispanic	Women	83	0.00196, 0.00233, 0.00275, 0.00325, 0.00384
	Hispanic	Women	84	0.00209, 0.00252, 0.00304, 0.00366, 0.00442
	White	Men	30	0.00000596, 0.00000804, 0.0000108, 0.0000146, 0.0000196
	White	Men	31	0.00000698, 0.00000923, 0.0000122, 0.0000161, 0.0000213
	White	Men	32	0.00000813, 0.0000106, 0.0000137, 0.0000178, 0.0000231
	White	Men	33	0.00000942, 0.0000121, 0.0000154, 0.0000197, 0.0000252
	White	Men	34	0.0000108, 0.0000137, 0.0000172, 0.0000217, 0.0000274
	White	Men	35	0.0000123, 0.0000154, 0.0000193, 0.000024, 0.0000301
	White	Men	36	0.0000139, 0.0000173, 0.0000214, 0.0000266, 0.000033
	White	Men	37	0.0000156, 0.0000193, 0.0000238, 0.0000293, 0.0000363
	White	Men	38	0.0000174, 0.0000214, 0.0000263, 0.0000323, 0.0000399
	White	Men	39	0.0000192, 0.0000236, 0.000029, 0.0000356, 0.0000437
	White	Men	40	0.0000213, 0.0000261, 0.0000318, 0.0000389, 0.0000476
	White	Men	41	0.0000237, 0.0000288, 0.0000348, 0.0000421, 0.0000511
	White	Men	42	0.0000267, 0.0000319, 0.0000381, 0.0000454, 0.0000543
	White	Men	43	0.0000301, 0.0000354, 0.0000417, 0.000049, 0.0000577
	White	Men	44	0.0000337, 0.0000393, 0.0000458, 0.0000533, 0.0000621
	White	Men	45	0.0000377, 0.0000437, 0.0000504, 0.0000583, 0.0000674
	White	Men	46	0.0000423, 0.0000487, 0.0000559, 0.0000641, 0.0000737
	White	Men	47	0.0000474, 0.0000543, 0.0000621, 0.0000711, 0.0000814
	White	Men	48	0.0000527, 0.0000605, 0.0000693, 0.0000795, 0.0000912
	White	Men	49	0.0000582, 0.0000673, 0.0000776, 0.0000895, 0.000103
	White	Men	50	0.0000644, 0.000075, 0.0000871, 0.000101, 0.000118
	White	Men	51	0.000072, 0.0000839, 0.0000977, 0.000114, 0.000133
	White	Men	52	0.0000812, 0.0000943, 0.00011, 0.000127, 0.000148
	White	Men	53	0.0000916, 0.000106, 0.000122, 0.000141, 0.000164
	White	Men	54	0.000102, 0.000118, 0.000136, 0.000157, 0.000181
	White	Men	55	0.000113, 0.000131, 0.000151, 0.000174, 0.000201
	White	Men	56	0.000124, 0.000144, 0.000166, 0.000193, 0.000224
	White	Men	57	0.000135, 0.000157, 0.000183, 0.000214, 0.00025
	White	Men	58	0.000146, 0.000172, 0.000202, 0.000236, 0.000278
	White	Men	59	0.00016, 0.000188, 0.000221, 0.00026, 0.000307
	White	Men	60	0.000176, 0.000207, 0.000243, 0.000285, 0.000335
	White	Men	61	0.000196, 0.000229, 0.000266, 0.00031, 0.000362
	White	Men	62	0.000219, 0.000253, 0.000291, 0.000335, 0.000387

Variable	Race/ethnicity	Sex	Age	Distribution
	White	Men	63	0.000246, 0.00028, 0.000318, 0.000361, 0.00041
	White	Men	64	0.000276, 0.000309, 0.000347, 0.000388, 0.000436
	White	Men	65	0.000308, 0.000342, 0.000379, 0.00042, 0.000466
	White	Men	66	0.000343, 0.000378, 0.000417, 0.000459, 0.000506
	White	Men	67	0.00038, 0.000419, 0.000461, 0.000508, 0.000559
	White	Men	68	0.000422, 0.000466, 0.000515, 0.000568, 0.000627
	White	Men	69	0.00047, 0.000522, 0.000578, 0.00064, 0.00071
	White	Men	70	0.000527, 0.000586, 0.000652, 0.000725, 0.000808
	White	Men	71	0.000591, 0.000661, 0.000738, 0.000824, 0.00092
	White	Men	72	0.000664, 0.000744, 0.000834, 0.000935, 0.00105
	White	Men	73	0.000743, 0.000838, 0.000943, 0.00106, 0.0012
	White	Men	74	0.000832, 0.000942, 0.00107, 0.00121, 0.00137
	White	Men	75	0.000932, 0.00106, 0.00121, 0.00137, 0.00156
	White	Men	76	0.00105, 0.0012, 0.00137, 0.00157, 0.00179
	White	Men	77	0.00118, 0.00135, 0.00156, 0.00179, 0.00207
	White	Men	78	0.00132, 0.00153, 0.00178, 0.00207, 0.0024
	White	Men	79	0.00148, 0.00174, 0.00204, 0.00239, 0.00282
	White	Men	80	0.00165, 0.00196, 0.00233, 0.00278, 0.00331
	White	Men	81	0.00184, 0.00221, 0.00266, 0.0032, 0.00385
	White	Men	82	0.00205, 0.00249, 0.00301, 0.00365, 0.00442
	White	Men	83	0.00226, 0.00276, 0.00337, 0.00412, 0.00504
	White	Men	84	0.00246, 0.00303, 0.00373, 0.0046, 0.00568
	White	Women	30	0.00000528, 0.00000731, 0.0000101, 0.0000139, 0.0000193
	White	Women	31	0.00000617, 0.00000842, 0.0000115, 0.0000156, 0.0000213
	White	Women	32	0.00000722, 0.0000097, 0.000013, 0.0000174, 0.0000234
	White	Women	33	0.00000843, 0.0000112, 0.0000147, 0.0000195, 0.0000258
	White	Women	34	0.00000976, 0.0000127, 0.0000166, 0.0000216, 0.0000283
	White	Women	35	0.0000112, 0.0000144, 0.0000186, 0.000024, 0.0000309
	White	Women	36	0.0000127, 0.0000162, 0.0000207, 0.0000264, 0.0000337
	White	Women	37	0.0000143, 0.0000181, 0.0000229, 0.0000288, 0.0000365
	White	Women	38	0.000016, 0.0000201, 0.0000251, 0.0000314, 0.0000393
	White	Women	39	0.0000178, 0.0000222, 0.0000274, 0.000034, 0.0000422
	White	Women	40	0.0000198, 0.0000243, 0.0000298, 0.0000366, 0.000045
	White	Women	41	0.000022, 0.0000267, 0.0000323, 0.0000391, 0.0000474
	White	Women	42	0.0000247, 0.0000294, 0.0000349, 0.0000415, 0.0000494
	White	Women	43	0.0000275, 0.0000322, 0.0000378, 0.0000442, 0.0000519
	White	Women	44	0.0000302, 0.0000352, 0.0000409, 0.0000477, 0.0000555
	White	Women	45	0.0000329, 0.0000383, 0.0000446, 0.0000518, 0.0000603
	White	Women	46	0.0000359, 0.0000418, 0.0000487, 0.0000567, 0.0000662
	White	Women	47	0.000039, 0.0000457, 0.0000534, 0.0000625, 0.0000733
	White	Women	48	0.0000422, 0.0000498, 0.0000588, 0.0000693, 0.0000818
	White	Women	49	0.0000456, 0.0000544, 0.0000648, 0.0000771, 0.0000919
	White	Women	50	0.0000495, 0.0000595, 0.0000715, 0.0000858, 0.000103

Variable	Race/ethnicity	Sex	Age	Distribution
	White	Women	51	0.0000542, 0.0000654, 0.0000788, 0.0000949, 0.000115
	White	Women	52	0.0000602, 0.0000723, 0.0000868, 0.000104, 0.000125
	White	Women	53	0.0000671, 0.00008, 0.0000953, 0.000113, 0.000135
	White	Women	54	0.0000748, 0.0000884, 0.000104, 0.000123, 0.000145
	White	Women	55	0.0000831, 0.0000972, 0.000114, 0.000133, 0.000155
	White	Women	56	0.0000922, 0.000107, 0.000123, 0.000143, 0.000165
	White	Women	57	0.000102, 0.000117, 0.000134, 0.000154, 0.000176
	White	Women	58	0.000113, 0.000129, 0.000146, 0.000166, 0.000189
	White	Women	59	0.000126, 0.000142, 0.00016, 0.00018, 0.000204
	White	Women	60	0.000139, 0.000156, 0.000175, 0.000197, 0.000221
	White	Women	61	0.000154, 0.000172, 0.000193, 0.000216, 0.000242
	White	Women	62	0.000169, 0.000189, 0.000212, 0.000237, 0.000265
	White	Women	63	0.000186, 0.000208, 0.000233, 0.00026, 0.000291
	White	Women	64	0.000206, 0.00023, 0.000256, 0.000286, 0.000319
	White	Women	65	0.000227, 0.000253, 0.000282, 0.000314, 0.00035
	White	Women	66	0.000252, 0.000281, 0.000313, 0.000348, 0.000388
	White	Women	67	0.000281, 0.000314, 0.000349, 0.000389, 0.000434
	White	Women	68	0.000317, 0.000353, 0.000394, 0.000439, 0.00049
	White	Women	69	0.000361, 0.000402, 0.000448, 0.000499, 0.000556
	White	Women	70	0.000414, 0.000461, 0.000513, 0.00057, 0.000635
	White	Women	71	0.000477, 0.000531, 0.00059, 0.000656, 0.00073
	White	Women	72	0.000548, 0.000611, 0.000681, 0.000758, 0.000845
	White	Women	73	0.000629, 0.000703, 0.000785, 0.000876, 0.000979
	White	Women	74	0.000722, 0.000808, 0.000904, 0.00101, 0.00113
	White	Women	75	0.000831, 0.000931, 0.00104, 0.00116, 0.0013
	White	Women	76	0.000958, 0.00107, 0.0012, 0.00134, 0.00151
	White	Women	77	0.0011, 0.00124, 0.00139, 0.00156, 0.00175
	White	Women	78	0.00125, 0.00142, 0.00161, 0.00182, 0.00206
	White	Women	79	0.00143, 0.00164, 0.00187, 0.00214, 0.00244
	White	Women	80	0.00164, 0.00189, 0.00217, 0.0025, 0.00288
	White	Women	81	0.00188, 0.00217, 0.00252, 0.00292, 0.00338
	White	Women	82	0.00214, 0.00249, 0.0029, 0.00337, 0.00393
	White	Women	83	0.00241, 0.00283, 0.0033, 0.00386, 0.00452
	White	Women	84	0.00269, 0.00317, 0.00372, 0.00437, 0.00514
Stroke mortality rates for 2024 (0.01, 0.2, 0.5, 0.8. 0.99 percentiles of the empirical distribution produced during forecasting)				
	Black	Men	30	0.000016, 0.0000219, 0.00003, 0.0000411, 0.0000564
	Black	Men	31	0.0000196, 0.0000263, 0.000035, 0.0000467, 0.0000624
	Black	Men	32	0.0000236, 0.000031, 0.0000407, 0.0000535, 0.0000704
	Black	Men	33	0.0000276, 0.0000361, 0.0000471, 0.0000614, 0.0000804
	Black	Men	34	0.0000318, 0.0000415, 0.000054, 0.0000702, 0.0000916
	Black	Men	35	0.0000362, 0.0000472, 0.0000613, 0.0000796, 0.000104
	Black	Men	36	0.000041, 0.0000532, 0.0000688, 0.000089, 0.000115
	Black	Men	37	0.0000464, 0.0000597, 0.0000765, 0.0000981, 0.000126

Variable	Race/ethnicity	Sex	Age	Distribution
	Black	Men	38	0.0000524, 0.0000666, 0.0000844, 0.000107, 0.000136
	Black	Men	39	0.0000587, 0.0000738, 0.0000924, 0.000116, 0.000145
	Black	Men	40	0.0000655, 0.0000813, 0.000101, 0.000125, 0.000155
	Black	Men	41	0.0000732, 0.0000898, 0.00011, 0.000134, 0.000165
	Black	Men	42	0.0000827, 0.0000997, 0.00012, 0.000144, 0.000174
	Black	Men	43	0.0000928, 0.000111, 0.000132, 0.000156, 0.000186
	Black	Men	44	0.000102, 0.000122, 0.000145, 0.000173, 0.000206
	Black	Men	45	0.000111, 0.000134, 0.00016, 0.000193, 0.000232
	Black	Men	46	0.000122, 0.000148, 0.000178, 0.000215, 0.00026
	Black	Men	47	0.000136, 0.000164, 0.000199, 0.00024, 0.00029
	Black	Men	48	0.00015, 0.000183, 0.000222, 0.00027, 0.00033
	Black	Men	49	0.000166, 0.000204, 0.00025, 0.000306, 0.000376
	Black	Men	50	0.000188, 0.000231, 0.000282, 0.000345, 0.000423
	Black	Men	51	0.00022, 0.000265, 0.000319, 0.000385, 0.000464
	Black	Men	52	0.000259, 0.000306, 0.000361, 0.000426, 0.000504
	Black	Men	53	0.0003, 0.000349, 0.000406, 0.000472, 0.00055
	Black	Men	54	0.000338, 0.000391, 0.000452, 0.000523, 0.000605
	Black	Men	55	0.000375, 0.000433, 0.000499, 0.000575, 0.000664
	Black	Men	56	0.00041, 0.000473, 0.000545, 0.000629, 0.000726
	Black	Men	57	0.000445, 0.000514, 0.000592, 0.000683, 0.000788
	Black	Men	58	0.000484, 0.000557, 0.00064, 0.000735, 0.000845
	Black	Men	59	0.000527, 0.000603, 0.000689, 0.000788, 0.000902
	Black	Men	60	0.000564, 0.000646, 0.00074, 0.000847, 0.000971
	Black	Men	61	0.000595, 0.000686, 0.000791, 0.000911, 0.00105
	Black	Men	62	0.00063, 0.000728, 0.000841, 0.000971, 0.00112
	Black	Men	63	0.000676, 0.000777, 0.000891, 0.00102, 0.00118
	Black	Men	64	0.000729, 0.00083, 0.000943, 0.00107, 0.00122
	Black	Men	65	0.000779, 0.000883, 0.000999, 0.00113, 0.00128
	Black	Men	66	0.000825, 0.000938, 0.00106, 0.00121, 0.00137
	Black	Men	67	0.000882, 0.001, 0.00114, 0.0013, 0.00148
	Black	Men	68	0.000956, 0.00109, 0.00123, 0.0014, 0.00159
	Black	Men	69	0.00104, 0.00118, 0.00134, 0.00151, 0.00171
	Black	Men	70	0.00114, 0.00129, 0.00145, 0.00164, 0.00186
	Black	Men	71	0.00124, 0.0014, 0.00158, 0.00178, 0.00201
	Black	Men	72	0.00134, 0.00152, 0.00171, 0.00194, 0.00219
	Black	Men	73	0.00145, 0.00164, 0.00186, 0.0021, 0.00238
	Black	Men	74	0.00155, 0.00176, 0.00201, 0.00229, 0.00261
	Black	Men	75	0.00165, 0.0019, 0.00218, 0.0025, 0.00287
	Black	Men	76	0.00178, 0.00205, 0.00237, 0.00272, 0.00314
	Black	Men	77	0.00194, 0.00224, 0.00258, 0.00297, 0.00343
	Black	Men	78	0.00211, 0.00244, 0.00281, 0.00325, 0.00375
	Black	Men	79	0.00229, 0.00265, 0.00307, 0.00355, 0.00412
	Black	Men	80	0.00246, 0.00287, 0.00334, 0.00388, 0.00453

Variable	Race/ethnicity	Sex	Age	Distribution
	Black	Men	81	0.00263, 0.00308, 0.00361, 0.00422, 0.00494
	Black	Men	82	0.00281, 0.0033, 0.00387, 0.00454, 0.00534
	Black	Men	83	0.00298, 0.00351, 0.00413, 0.00486, 0.00573
	Black	Men	84	0.00314, 0.00371, 0.00438, 0.00517, 0.00611
	Black	Women	30	0.0000112, 0.0000165, 0.0000243, 0.0000358, 0.0000529
	Black	Women	31	0.0000129, 0.000019, 0.0000279, 0.000041, 0.0000605
	Black	Women	32	0.0000147, 0.0000217, 0.000032, 0.0000471, 0.0000697
	Black	Women	33	0.0000165, 0.0000246, 0.0000365, 0.0000541, 0.0000806
	Black	Women	34	0.0000184, 0.0000276, 0.0000414, 0.000062, 0.0000933
	Black	Women	35	0.0000205, 0.000031, 0.0000467, 0.0000704, 0.000107
	Black	Women	36	0.0000231, 0.0000349, 0.0000524, 0.0000787, 0.000119
	Black	Women	37	0.0000265, 0.0000394, 0.0000584, 0.0000865, 0.000129
	Black	Women	38	0.0000307, 0.0000446, 0.0000647, 0.0000938, 0.000137
	Black	Women	39	0.0000352, 0.0000502, 0.0000714, 0.000101, 0.000145
	Black	Women	40	0.0000399, 0.000056, 0.0000784, 0.00011, 0.000154
	Black	Women	41	0.0000447, 0.000062, 0.0000857, 0.000118, 0.000164
	Black	Women	42	0.0000503, 0.0000686, 0.0000934, 0.000127, 0.000174
	Black	Women	43	0.0000566, 0.0000761, 0.000102, 0.000136, 0.000183
	Black	Women	44	0.0000633, 0.000084, 0.000111, 0.000147, 0.000195
	Black	Women	45	0.0000704, 0.0000927, 0.000122, 0.00016, 0.000211
	Black	Women	46	0.0000791, 0.000103, 0.000134, 0.000174, 0.000226
	Black	Women	47	0.0000901, 0.000115, 0.000147, 0.000188, 0.000241
	Black	Women	48	0.000103, 0.000129, 0.000162, 0.000203, 0.000255
	Black	Women	49	0.000117, 0.000144, 0.000178, 0.000221, 0.000273
	Black	Women	50	0.000129, 0.000159, 0.000196, 0.000241, 0.000296
	Black	Women	51	0.000143, 0.000175, 0.000214, 0.000261, 0.00032
	Black	Women	52	0.000158, 0.000192, 0.000233, 0.000282, 0.000342
	Black	Women	53	0.000175, 0.00021, 0.000252, 0.000303, 0.000365
	Black	Women	54	0.00019, 0.000228, 0.000273, 0.000328, 0.000393
	Black	Women	55	0.000205, 0.000246, 0.000296, 0.000355, 0.000428
	Black	Women	56	0.000222, 0.000267, 0.000321, 0.000385, 0.000463
	Black	Women	57	0.000244, 0.000292, 0.000348, 0.000415, 0.000497
	Black	Women	58	0.000272, 0.000321, 0.000379, 0.000446, 0.000527
	Black	Women	59	0.000303, 0.000353, 0.000411, 0.000479, 0.000559
	Black	Women	60	0.000332, 0.000385, 0.000446, 0.000516, 0.000597
	Black	Women	61	0.000358, 0.000415, 0.00048, 0.000556, 0.000644
	Black	Women	62	0.00038, 0.000442, 0.000514, 0.000597, 0.000694
	Black	Women	63	0.000403, 0.00047, 0.000547, 0.000636, 0.000742
	Black	Women	64	0.000429, 0.0005, 0.000581, 0.000675, 0.000785
	Black	Women	65	0.000461, 0.000534, 0.000618, 0.000714, 0.000827
	Black	Women	66	0.000499, 0.000574, 0.00066, 0.000759, 0.000874
	Black	Women	67	0.000545, 0.000623, 0.000712, 0.000813, 0.00093
	Black	Women	68	0.000601, 0.000683, 0.000775, 0.000879, 0.000998

Variable	Race/ethnicity	Sex	Age	Distribution
	Black	Women	69	0.000668, 0.000754, 0.000851, 0.000959, 0.00108
	Black	Women	70	0.000745, 0.000838, 0.000941, 0.00106, 0.00119
	Black	Women	71	0.000829, 0.000932, 0.00105, 0.00118, 0.00132
	Black	Women	72	0.000922, 0.00104, 0.00117, 0.00131, 0.00148
	Black	Women	73	0.00103, 0.00116, 0.00131, 0.00147, 0.00166
	Black	Women	74	0.00115, 0.0013, 0.00146, 0.00165, 0.00185
	Black	Women	75	0.00129, 0.00145, 0.00164, 0.00184, 0.00208
	Black	Women	76	0.00143, 0.00162, 0.00184, 0.00208, 0.00236
	Black	Women	77	0.00158, 0.00181, 0.00207, 0.00236, 0.00271
	Black	Women	78	0.00173, 0.00201, 0.00233, 0.00269, 0.00312
	Black	Women	79	0.00191, 0.00224, 0.00261, 0.00306, 0.00358
	Black	Women	80	0.00211, 0.00249, 0.00293, 0.00345, 0.00406
	Black	Women	81	0.00233, 0.00276, 0.00326, 0.00386, 0.00456
	Black	Women	82	0.00256, 0.00304, 0.00361, 0.00428, 0.00509
	Black	Women	83	0.00278, 0.00332, 0.00396, 0.00473, 0.00565
	Black	Women	84	0.00298, 0.00359, 0.00431, 0.00518, 0.00623
	Hispanic	Men	30	0.00000733, 0.0000102, 0.0000141, 0.0000196, 0.0000273
	Hispanic	Men	31	0.00000882, 0.0000119, 0.000016, 0.0000216, 0.0000292
	Hispanic	Men	32	0.0000105, 0.0000138, 0.0000182, 0.0000239, 0.0000315
	Hispanic	Men	33	0.0000123, 0.0000159, 0.0000206, 0.0000266, 0.0000345
	Hispanic	Men	34	0.0000141, 0.0000181, 0.0000232, 0.0000297, 0.0000382
	Hispanic	Men	35	0.000016, 0.0000205, 0.0000261, 0.0000333, 0.0000425
	Hispanic	Men	36	0.0000182, 0.0000231, 0.0000292, 0.000037, 0.0000469
	Hispanic	Men	37	0.0000206, 0.0000259, 0.0000325, 0.0000408, 0.0000513
	Hispanic	Men	38	0.0000233, 0.000029, 0.000036, 0.0000447, 0.0000557
	Hispanic	Men	39	0.0000261, 0.0000322, 0.0000397, 0.0000489, 0.0000604
	Hispanic	Men	40	0.000029, 0.0000356, 0.0000436, 0.0000534, 0.0000655
	Hispanic	Men	41	0.0000322, 0.0000392, 0.0000477, 0.000058, 0.0000706
	Hispanic	Men	42	0.0000359, 0.0000433, 0.0000521, 0.0000627, 0.0000756
	Hispanic	Men	43	0.0000398, 0.0000476, 0.0000568, 0.0000679, 0.0000812
	Hispanic	Men	44	0.0000437, 0.0000522, 0.0000622, 0.000074, 0.0000883
	Hispanic	Men	45	0.0000481, 0.0000573, 0.0000682, 0.0000811, 0.0000967
	Hispanic	Men	46	0.0000531, 0.0000632, 0.000075, 0.0000891, 0.000106
	Hispanic	Men	47	0.0000591, 0.00007, 0.0000829, 0.0000981, 0.000116
	Hispanic	Men	48	0.0000661, 0.0000781, 0.000092, 0.000108, 0.000128
	Hispanic	Men	49	0.0000747, 0.0000876, 0.000103, 0.00012, 0.000141
	Hispanic	Men	50	0.0000852, 0.0000989, 0.000115, 0.000133, 0.000155
	Hispanic	Men	51	0.0000976, 0.000112, 0.000128, 0.000147, 0.000169
	Hispanic	Men	52	0.000111, 0.000126, 0.000144, 0.000163, 0.000186
	Hispanic	Men	53	0.000123, 0.00014, 0.00016, 0.000183, 0.000209
	Hispanic	Men	54	0.000132, 0.000153, 0.000178, 0.000206, 0.000239
	Hispanic	Men	55	0.000142, 0.000167, 0.000196, 0.00023, 0.000271
	Hispanic	Men	56	0.000153, 0.000181, 0.000215, 0.000255, 0.000303

Variable	Race/ethnicity	Sex	Age	Distribution
	Hispanic	Men	57	0.000166, 0.000198, 0.000235, 0.000279, 0.000331
	Hispanic	Men	58	0.000183, 0.000216, 0.000255, 0.000302, 0.000357
	Hispanic	Men	59	0.000202, 0.000237, 0.000277, 0.000324, 0.000379
	Hispanic	Men	60	0.000223, 0.000259, 0.0003, 0.000347, 0.000402
	Hispanic	Men	61	0.000246, 0.000282, 0.000323, 0.00037, 0.000425
	Hispanic	Men	62	0.000267, 0.000305, 0.000348, 0.000396, 0.000453
	Hispanic	Men	63	0.000288, 0.000328, 0.000374, 0.000425, 0.000485
	Hispanic	Men	64	0.000309, 0.000352, 0.000402, 0.000458, 0.000522
	Hispanic	Men	65	0.000332, 0.000379, 0.000433, 0.000494, 0.000565
	Hispanic	Men	66	0.000357, 0.00041, 0.000469, 0.000537, 0.000615
	Hispanic	Men	67	0.000387, 0.000445, 0.000512, 0.000588, 0.000677
	Hispanic	Men	68	0.000421, 0.000487, 0.000562, 0.000649, 0.000751
	Hispanic	Men	69	0.000463, 0.000537, 0.000622, 0.00072, 0.000835
	Hispanic	Men	70	0.000515, 0.000597, 0.000691, 0.000801, 0.000928
	Hispanic	Men	71	0.000576, 0.000666, 0.000771, 0.000891, 0.00103
	Hispanic	Men	72	0.000645, 0.000746, 0.00086, 0.000993, 0.00115
	Hispanic	Men	73	0.000722, 0.000834, 0.000962, 0.00111, 0.00128
	Hispanic	Men	74	0.000807, 0.000932, 0.00107, 0.00124, 0.00143
	Hispanic	Men	75	0.000902, 0.00104, 0.0012, 0.00139, 0.0016
	Hispanic	Men	76	0.00101, 0.00117, 0.00135, 0.00156, 0.0018
	Hispanic	Men	77	0.00113, 0.00131, 0.00151, 0.00175, 0.00203
	Hispanic	Men	78	0.00126, 0.00146, 0.0017, 0.00197, 0.0023
	Hispanic	Men	79	0.00139, 0.00163, 0.0019, 0.00223, 0.0026
	Hispanic	Men	80	0.00154, 0.00181, 0.00213, 0.0025, 0.00295
	Hispanic	Men	81	0.00168, 0.002, 0.00237, 0.00281, 0.00333
	Hispanic	Men	82	0.00183, 0.00219, 0.00262, 0.00313, 0.00375
	Hispanic	Men	83	0.00197, 0.00238, 0.00287, 0.00346, 0.00419
	Hispanic	Men	84	0.0021, 0.00256, 0.00312, 0.00379, 0.00462
	Hispanic	Women	30	0.00000417, 0.00000637, 0.00000971, 0.0000148, 0.0000226
	Hispanic	Women	31	0.00000494, 0.00000735, 0.0000109, 0.0000162, 0.000024
	Hispanic	Women	32	0.00000579, 0.00000845, 0.0000123, 0.0000179, 0.0000262
	Hispanic	Women	33	0.00000672, 0.00000969, 0.0000139, 0.00002, 0.0000289
	Hispanic	Women	34	0.00000779, 0.0000111, 0.0000158, 0.0000224, 0.0000319
	Hispanic	Women	35	0.00000905, 0.0000127, 0.0000178, 0.0000249, 0.000035
	Hispanic	Women	36	0.0000106, 0.0000146, 0.00002, 0.0000275, 0.0000379
	Hispanic	Women	37	0.0000123, 0.0000166, 0.0000224, 0.0000302, 0.0000408
	Hispanic	Women	38	0.0000142, 0.0000188, 0.0000249, 0.0000329, 0.0000437
	Hispanic	Women	39	0.0000162, 0.0000211, 0.0000275, 0.0000359, 0.0000468
	Hispanic	Women	40	0.0000183, 0.0000235, 0.0000303, 0.0000389, 0.0000502
	Hispanic	Women	41	0.0000204, 0.0000261, 0.0000332, 0.0000422, 0.0000538
	Hispanic	Women	42	0.0000227, 0.0000287, 0.0000363, 0.0000458, 0.000058
	Hispanic	Women	43	0.000025, 0.0000315, 0.0000396, 0.0000499, 0.0000629
	Hispanic	Women	44	0.0000276, 0.0000347, 0.0000434, 0.0000544, 0.0000683

Variable	Race/ethnicity	Sex	Age	Distribution
	Hispanic	Women	45	0.000031, 0.0000385, 0.0000477, 0.0000591, 0.0000733
	Hispanic	Women	46	0.0000353, 0.0000431, 0.0000524, 0.0000637, 0.0000777
	Hispanic	Women	47	0.0000404, 0.0000483, 0.0000576, 0.0000688, 0.0000822
	Hispanic	Women	48	0.0000457, 0.0000539, 0.0000635, 0.0000748, 0.0000883
	Hispanic	Women	49	0.0000506, 0.0000596, 0.0000701, 0.0000824, 0.0000971
	Hispanic	Women	50	0.000055, 0.0000653, 0.0000774, 0.0000918, 0.000109
	Hispanic	Women	51	0.0000594, 0.0000714, 0.0000856, 0.000103, 0.000123
	Hispanic	Women	52	0.0000639, 0.0000779, 0.0000946, 0.000115, 0.00014
	Hispanic	Women	53	0.0000688, 0.0000849, 0.000104, 0.000128, 0.000158
	Hispanic	Women	54	0.0000744, 0.0000926, 0.000115, 0.000143, 0.000177
	Hispanic	Women	55	0.0000812, 0.000101, 0.000126, 0.000157, 0.000196
	Hispanic	Women	56	0.0000892, 0.000111, 0.000138, 0.000171, 0.000213
	Hispanic	Women	57	0.0000984, 0.000122, 0.000151, 0.000186, 0.000231
	Hispanic	Women	58	0.000108, 0.000134, 0.000164, 0.000202, 0.000249
	Hispanic	Women	59	0.000119, 0.000146, 0.000179, 0.00022, 0.00027
	Hispanic	Women	60	0.00013, 0.00016, 0.000195, 0.000239, 0.000293
	Hispanic	Women	61	0.000142, 0.000174, 0.000212, 0.000259, 0.000317
	Hispanic	Women	62	0.000155, 0.000189, 0.00023, 0.00028, 0.000341
	Hispanic	Women	63	0.000168, 0.000205, 0.000249, 0.000303, 0.000368
	Hispanic	Women	64	0.000182, 0.000221, 0.000269, 0.000327, 0.000399
	Hispanic	Women	65	0.000196, 0.00024, 0.000292, 0.000355, 0.000434
	Hispanic	Women	66	0.000213, 0.000261, 0.000318, 0.000388, 0.000475
	Hispanic	Women	67	0.000233, 0.000286, 0.00035, 0.000428, 0.000524
	Hispanic	Women	68	0.000259, 0.000317, 0.000388, 0.000476, 0.000583
	Hispanic	Women	69	0.000291, 0.000356, 0.000435, 0.000532, 0.000651
	Hispanic	Women	70	0.000332, 0.000404, 0.000491, 0.000596, 0.000725
	Hispanic	Women	71	0.000385, 0.000463, 0.000556, 0.000668, 0.000804
	Hispanic	Women	72	0.00045, 0.000534, 0.000632, 0.000749, 0.000888
	Hispanic	Women	73	0.000529, 0.000618, 0.00072, 0.00084, 0.000982
	Hispanic	Women	74	0.000617, 0.000713, 0.000822, 0.000947, 0.00109
	Hispanic	Women	75	0.000715, 0.00082, 0.000939, 0.00108, 0.00123
	Hispanic	Women	76	0.000821, 0.00094, 0.00108, 0.00123, 0.00141
	Hispanic	Women	77	0.000942, 0.00108, 0.00123, 0.00141, 0.00162
	Hispanic	Women	78	0.00108, 0.00124, 0.00142, 0.00162, 0.00185
	Hispanic	Women	79	0.00125, 0.00143, 0.00163, 0.00186, 0.00212
	Hispanic	Women	80	0.00143, 0.00164, 0.00186, 0.00212, 0.00242
	Hispanic	Women	81	0.00161, 0.00185, 0.00212, 0.00243, 0.00279
	Hispanic	Women	82	0.00177, 0.00206, 0.00239, 0.00278, 0.00324
	Hispanic	Women	83	0.0019, 0.00226, 0.00268, 0.00317, 0.00377
	Hispanic	Women	84	0.00202, 0.00245, 0.00296, 0.00358, 0.00434
	White	Men	30	0.00000582, 0.00000793, 0.0000108, 0.0000146, 0.0000199
	White	Men	31	0.00000684, 0.00000912, 0.0000121, 0.0000161, 0.0000215
	White	Men	32	0.00000798, 0.0000105, 0.0000137, 0.0000178, 0.0000234

Variable	Race/ethnicity	Sex	Age	Distribution
	White	Men	33	0.00000926, 0.0000119, 0.0000153, 0.0000197, 0.0000254
	White	Men	34	0.0000106, 0.0000135, 0.0000172, 0.0000218, 0.0000277
	White	Men	35	0.0000121, 0.0000153, 0.0000192, 0.0000241, 0.0000303
	White	Men	36	0.0000136, 0.0000171, 0.0000213, 0.0000266, 0.0000333
	White	Men	37	0.0000152, 0.000019, 0.0000236, 0.0000293, 0.0000366
	White	Men	38	0.0000169, 0.000021, 0.000026, 0.0000323, 0.0000401
	White	Men	39	0.0000187, 0.0000232, 0.0000286, 0.0000354, 0.0000439
	White	Men	40	0.0000207, 0.0000255, 0.0000314, 0.0000386, 0.0000476
	White	Men	41	0.000023, 0.0000281, 0.0000342, 0.0000417, 0.0000509
	White	Men	42	0.0000259, 0.0000311, 0.0000373, 0.0000448, 0.0000538
	White	Men	43	0.0000292, 0.0000345, 0.0000408, 0.0000482, 0.000057
	White	Men	44	0.0000327, 0.0000383, 0.0000447, 0.0000523, 0.0000612
	White	Men	45	0.0000365, 0.0000425, 0.0000493, 0.0000571, 0.0000664
	White	Men	46	0.000041, 0.0000473, 0.0000545, 0.0000628, 0.0000725
	White	Men	47	0.0000459, 0.0000528, 0.0000606, 0.0000696, 0.00008
	White	Men	48	0.000051, 0.0000588, 0.0000676, 0.0000778, 0.0000896
	White	Men	49	0.0000564, 0.0000654, 0.0000757, 0.0000877, 0.000102
	White	Men	50	0.0000625, 0.000073, 0.0000851, 0.0000992, 0.000116
	White	Men	51	0.0000698, 0.0000818, 0.0000956, 0.000112, 0.000131
	White	Men	52	0.0000788, 0.000092, 0.000107, 0.000125, 0.000146
	White	Men	53	0.0000891, 0.000103, 0.00012, 0.000139, 0.000162
	White	Men	54	0.0000997, 0.000115, 0.000134, 0.000155, 0.000179
	White	Men	55	0.00011, 0.000128, 0.000148, 0.000172, 0.000199
	White	Men	56	0.000121, 0.000141, 0.000164, 0.00019, 0.000222
	White	Men	57	0.000131, 0.000154, 0.00018, 0.000211, 0.000248
	White	Men	58	0.000142, 0.000168, 0.000198, 0.000233, 0.000275
	White	Men	59	0.000156, 0.000184, 0.000218, 0.000257, 0.000304
	White	Men	60	0.000172, 0.000203, 0.000239, 0.000281, 0.000332
	White	Men	61	0.000191, 0.000224, 0.000262, 0.000306, 0.000359
	White	Men	62	0.000213, 0.000247, 0.000286, 0.00033, 0.000383
	White	Men	63	0.000239, 0.000273, 0.000311, 0.000355, 0.000406
	White	Men	64	0.000268, 0.000302, 0.000339, 0.000382, 0.00043
	White	Men	65	0.000299, 0.000333, 0.000371, 0.000412, 0.000459
	White	Men	66	0.000333, 0.000368, 0.000407, 0.00045, 0.000498
	White	Men	67	0.000368, 0.000408, 0.00045, 0.000498, 0.000551
	White	Men	68	0.000409, 0.000453, 0.000502, 0.000557, 0.000618
	White	Men	69	0.000455, 0.000507, 0.000564, 0.000628, 0.000699
	White	Men	70	0.00051, 0.00057, 0.000637, 0.000711, 0.000795
	White	Men	71	0.000572, 0.000642, 0.00072, 0.000807, 0.000906
	White	Men	72	0.000642, 0.000724, 0.000814, 0.000917, 0.00103
	White	Men	73	0.000719, 0.000814, 0.000921, 0.00104, 0.00118
	White	Men	74	0.000804, 0.000915, 0.00104, 0.00118, 0.00135
	White	Men	75	0.0009, 0.00103, 0.00118, 0.00135, 0.00154

Variable	Race/ethnicity	Sex	Age	Distribution
	White	Men	76	0.00101, 0.00116, 0.00134, 0.00153, 0.00177
	White	Men	77	0.00114, 0.00132, 0.00152, 0.00176, 0.00204
	White	Men	78	0.00127, 0.00149, 0.00174, 0.00203, 0.00237
	White	Men	79	0.00143, 0.00169, 0.00199, 0.00235, 0.00278
	White	Men	80	0.00159, 0.0019, 0.00228, 0.00273, 0.00327
	White	Men	81	0.00177, 0.00215, 0.0026, 0.00314, 0.0038
	White	Men	82	0.00198, 0.00241, 0.00294, 0.00358, 0.00437
	White	Men	83	0.00218, 0.00268, 0.00329, 0.00404, 0.00497
	White	Men	84	0.00237, 0.00294, 0.00364, 0.00451, 0.00559
	White	Women	30	0.00000517, 0.00000722, 0.00001, 0.000014, 0.0000195
	White	Women	31	0.00000606, 0.00000833, 0.0000114, 0.0000156, 0.0000215
	White	Women	32	0.0000071, 0.00000961, 0.000013, 0.0000175, 0.0000237
	White	Women	33	0.0000083, 0.0000111, 0.0000147, 0.0000195, 0.000026
	White	Women	34	0.00000961, 0.0000126, 0.0000166, 0.0000217, 0.0000285
	White	Women	35	0.000011, 0.0000143, 0.0000185, 0.000024, 0.0000312
	White	Women	36	0.0000125, 0.000016, 0.0000206, 0.0000264, 0.0000339
	White	Women	37	0.000014, 0.0000179, 0.0000227, 0.0000288, 0.0000367
	White	Women	38	0.0000157, 0.0000198, 0.0000249, 0.0000313, 0.0000395
	White	Women	39	0.0000174, 0.0000218, 0.0000271, 0.0000339, 0.0000424
	White	Women	40	0.0000193, 0.0000238, 0.0000294, 0.0000364, 0.000045
	White	Women	41	0.0000214, 0.0000261, 0.0000318, 0.0000387, 0.0000472
	White	Women	42	0.000024, 0.0000287, 0.0000343, 0.000041, 0.000049
	White	Women	43	0.0000267, 0.0000315, 0.000037, 0.0000435, 0.0000513
	White	Women	44	0.0000293, 0.0000343, 0.0000401, 0.0000468, 0.0000547
	White	Women	45	0.000032, 0.0000374, 0.0000436, 0.0000508, 0.0000594
	White	Women	46	0.0000348, 0.0000407, 0.0000476, 0.0000556, 0.000065
	White	Women	47	0.0000379, 0.0000445, 0.0000522, 0.0000612, 0.0000719
	White	Women	48	0.0000411, 0.0000486, 0.0000574, 0.0000678, 0.0000802
	White	Women	49	0.0000444, 0.0000531, 0.0000633, 0.0000755, 0.0000902
	White	Women	50	0.0000483, 0.0000582, 0.0000699, 0.0000841, 0.000101
	White	Women	51	0.000053, 0.000064, 0.0000772, 0.0000932, 0.000113
	White	Women	52	0.0000589, 0.0000709, 0.0000852, 0.000102, 0.000123
	White	Women	53	0.0000658, 0.0000785, 0.0000936, 0.000112, 0.000133
	White	Women	54	0.0000733, 0.0000867, 0.000102, 0.000121, 0.000143
	White	Women	55	0.0000815, 0.0000955, 0.000112, 0.000131, 0.000153
	White	Women	56	0.0000903, 0.000105, 0.000122, 0.000141, 0.000163
	White	Women	57	0.0001, 0.000115, 0.000132, 0.000152, 0.000175
	White	Women	58	0.000111, 0.000126, 0.000144, 0.000164, 0.000187
	White	Women	59	0.000123, 0.000139, 0.000157, 0.000178, 0.000202
	White	Women	60	0.000136, 0.000153, 0.000173, 0.000195, 0.000219
	White	Women	61	0.00015, 0.000169, 0.00019, 0.000213, 0.00024
	White	Women	62	0.000165, 0.000185, 0.000208, 0.000234, 0.000263
	White	Women	63	0.000181, 0.000204, 0.000229, 0.000256, 0.000288

Variable	Race/ethnicity	Sex	Age	Distribution
	White	Women	64	0.0002, 0.000224, 0.000251, 0.000281, 0.000315
	White	Women	65	0.000221, 0.000247, 0.000276, 0.000309, 0.000345
	White	Women	66	0.000245, 0.000274, 0.000306, 0.000342, 0.000382
	White	Women	67	0.000273, 0.000305, 0.000341, 0.000382, 0.000427
	White	Women	68	0.000307, 0.000344, 0.000385, 0.00043, 0.000482
	White	Women	69	0.000349, 0.000391, 0.000437, 0.000489, 0.000547
	White	Women	70	0.000401, 0.000449, 0.000501, 0.000559, 0.000625
	White	Women	71	0.000462, 0.000516, 0.000576, 0.000643, 0.000719
	White	Women	72	0.000531, 0.000594, 0.000664, 0.000743, 0.000831
	White	Women	73	0.000608, 0.000683, 0.000765, 0.000858, 0.000963
	White	Women	74	0.000699, 0.000785, 0.000881, 0.00099, 0.00111
	White	Women	75	0.000804, 0.000904, 0.00101, 0.00114, 0.00128
	White	Women	76	0.000926, 0.00104, 0.00117, 0.00131, 0.00148
	White	Women	77	0.00106, 0.0012, 0.00135, 0.00153, 0.00172
	White	Women	78	0.00121, 0.00138, 0.00157, 0.00178, 0.00203
	White	Women	79	0.00138, 0.00159, 0.00182, 0.00209, 0.0024
	White	Women	80	0.00158, 0.00183, 0.00212, 0.00245, 0.00283
	White	Women	81	0.00181, 0.00211, 0.00245, 0.00285, 0.00332
	White	Women	82	0.00207, 0.00242, 0.00282, 0.0033, 0.00386
	White	Women	83	0.00233, 0.00274, 0.00322, 0.00378, 0.00444
	White	Women	84	0.0026, 0.00307, 0.00363, 0.00428, 0.00505
Stroke mortality rates for 2025 (0.01, 0.2, 0.5, 0.8. 0.99 percentiles of the empirical distribution produced during forecasting)				
	Black	Men	30	0.0000156, 0.0000216, 0.0000299, 0.0000413, 0.0000572
	Black	Men	31	0.0000193, 0.000026, 0.0000349, 0.0000469, 0.0000632
	Black	Men	32	0.0000231, 0.0000307, 0.0000406, 0.0000537, 0.0000712
	Black	Men	33	0.0000272, 0.0000358, 0.0000469, 0.0000616, 0.0000811
	Black	Men	34	0.0000313, 0.0000411, 0.0000538, 0.0000704, 0.0000924
	Black	Men	35	0.0000356, 0.0000467, 0.000061, 0.0000797, 0.000104
	Black	Men	36	0.0000403, 0.0000526, 0.0000684, 0.000089, 0.000116
	Black	Men	37	0.0000455, 0.0000588, 0.000076, 0.0000981, 0.000127
	Black	Men	38	0.0000512, 0.0000655, 0.0000836, 0.000107, 0.000137
	Black	Men	39	0.0000573, 0.0000724, 0.0000914, 0.000115, 0.000146
	Black	Men	40	0.0000637, 0.0000797, 0.0000994, 0.000124, 0.000155
	Black	Men	41	0.0000712, 0.0000878, 0.000108, 0.000133, 0.000164
	Black	Men	42	0.0000805, 0.0000974, 0.000118, 0.000142, 0.000172
	Black	Men	43	0.0000903, 0.000108, 0.000129, 0.000154, 0.000184
	Black	Men	44	0.000099, 0.000119, 0.000142, 0.00017, 0.000203
	Black	Men	45	0.000108, 0.00013, 0.000157, 0.000189, 0.000228
	Black	Men	46	0.000119, 0.000144, 0.000174, 0.00021, 0.000255
	Black	Men	47	0.000132, 0.00016, 0.000194, 0.000235, 0.000285
	Black	Men	48	0.000146, 0.000178, 0.000217, 0.000265, 0.000323
	Black	Men	49	0.000162, 0.000199, 0.000245, 0.0003, 0.00037
	Black	Men	50	0.000184, 0.000226, 0.000277, 0.000339, 0.000416

Variable	Race/ethnicity	Sex	Age	Distribution
	Black	Men	51	0.000215, 0.00026, 0.000313, 0.000378, 0.000457
	Black	Men	52	0.000254, 0.0003, 0.000355, 0.000419, 0.000496
	Black	Men	53	0.000294, 0.000343, 0.000399, 0.000465, 0.000543
	Black	Men	54	0.000332, 0.000385, 0.000445, 0.000516, 0.000598
	Black	Men	55	0.000368, 0.000426, 0.000492, 0.000568, 0.000658
	Black	Men	56	0.000402, 0.000465, 0.000538, 0.000622, 0.000719
	Black	Men	57	0.000436, 0.000505, 0.000584, 0.000675, 0.000781
	Black	Men	58	0.000475, 0.000548, 0.000631, 0.000727, 0.000839
	Black	Men	59	0.000516, 0.000592, 0.000679, 0.000779, 0.000895
	Black	Men	60	0.000552, 0.000634, 0.000729, 0.000837, 0.000963
	Black	Men	61	0.000581, 0.000673, 0.000778, 0.000899, 0.00104
	Black	Men	62	0.000615, 0.000714, 0.000827, 0.000958, 0.00111
	Black	Men	63	0.000659, 0.00076, 0.000875, 0.00101, 0.00116
	Black	Men	64	0.00071, 0.000811, 0.000924, 0.00105, 0.0012
	Black	Men	65	0.000758, 0.000862, 0.000978, 0.00111, 0.00126
	Black	Men	66	0.000802, 0.000915, 0.00104, 0.00119, 0.00135
	Black	Men	67	0.000857, 0.000978, 0.00112, 0.00127, 0.00145
	Black	Men	68	0.000928, 0.00106, 0.0012, 0.00137, 0.00156
	Black	Men	69	0.00101, 0.00115, 0.00131, 0.00148, 0.00169
	Black	Men	70	0.00111, 0.00125, 0.00142, 0.00161, 0.00183
	Black	Men	71	0.0012, 0.00136, 0.00154, 0.00175, 0.00198
	Black	Men	72	0.0013, 0.00148, 0.00167, 0.0019, 0.00215
	Black	Men	73	0.0014, 0.00159, 0.00181, 0.00206, 0.00234
	Black	Men	74	0.0015, 0.00171, 0.00196, 0.00224, 0.00256
	Black	Men	75	0.0016, 0.00185, 0.00212, 0.00244, 0.00281
	Black	Men	76	0.00173, 0.002, 0.00231, 0.00266, 0.00308
	Black	Men	77	0.00188, 0.00217, 0.00251, 0.00291, 0.00337
	Black	Men	78	0.00204, 0.00237, 0.00274, 0.00318, 0.00368
	Black	Men	79	0.00221, 0.00258, 0.00299, 0.00348, 0.00405
	Black	Men	80	0.00238, 0.00278, 0.00325, 0.0038, 0.00445
	Black	Men	81	0.00255, 0.003, 0.00352, 0.00413, 0.00485
	Black	Men	82	0.00272, 0.0032, 0.00377, 0.00445, 0.00524
	Black	Men	83	0.00288, 0.00341, 0.00403, 0.00476, 0.00563
	Black	Men	84	0.00303, 0.0036, 0.00427, 0.00506, 0.00601
	Black	Women	30	0.0000109, 0.0000163, 0.0000242, 0.000036, 0.0000537
	Black	Women	31	0.0000127, 0.0000188, 0.0000278, 0.0000412, 0.0000612
	Black	Women	32	0.0000145, 0.0000215, 0.0000319, 0.0000473, 0.0000704
	Black	Women	33	0.0000163, 0.0000244, 0.0000364, 0.0000543, 0.0000814
	Black	Women	34	0.0000182, 0.0000274, 0.0000413, 0.0000622, 0.000094
	Black	Women	35	0.0000202, 0.0000308, 0.0000466, 0.0000706, 0.000107
	Black	Women	36	0.0000228, 0.0000346, 0.0000522, 0.0000788, 0.000119
	Black	Women	37	0.0000261, 0.000039, 0.0000581, 0.0000865, 0.000129
	Black	Women	38	0.0000301, 0.0000441, 0.0000643, 0.0000937, 0.000137

Variable	Race/ethnicity	Sex	Age	Distribution
	Black	Women	39	0.0000344, 0.0000495, 0.0000707, 0.000101, 0.000145
	Black	Women	40	0.0000388, 0.0000549, 0.0000775, 0.000109, 0.000155
	Black	Women	41	0.0000434, 0.0000607, 0.0000846, 0.000118, 0.000165
	Black	Women	42	0.0000487, 0.000067, 0.0000921, 0.000126, 0.000174
	Black	Women	43	0.0000548, 0.0000742, 0.0001, 0.000135, 0.000183
	Black	Women	44	0.0000612, 0.0000819, 0.000109, 0.000146, 0.000195
	Black	Women	45	0.000068, 0.0000903, 0.000119, 0.000158, 0.00021
	Black	Women	46	0.0000764, 0.0001, 0.000131, 0.000172, 0.000225
	Black	Women	47	0.0000872, 0.000112, 0.000144, 0.000185, 0.000239
	Black	Women	48	0.0000998, 0.000126, 0.000159, 0.000201, 0.000253
	Black	Women	49	0.000113, 0.000141, 0.000175, 0.000218, 0.000272
	Black	Women	50	0.000126, 0.000156, 0.000192, 0.000238, 0.000295
	Black	Women	51	0.000139, 0.000171, 0.00021, 0.000259, 0.000319
	Black	Women	52	0.000154, 0.000188, 0.000229, 0.00028, 0.000341
	Black	Women	53	0.00017, 0.000206, 0.000249, 0.000301, 0.000364
	Black	Women	54	0.000185, 0.000224, 0.00027, 0.000325, 0.000393
	Black	Women	55	0.0002, 0.000242, 0.000292, 0.000353, 0.000427
	Black	Women	56	0.000217, 0.000262, 0.000317, 0.000383, 0.000463
	Black	Women	57	0.000238, 0.000287, 0.000344, 0.000413, 0.000496
	Black	Women	58	0.000266, 0.000315, 0.000374, 0.000443, 0.000525
	Black	Women	59	0.000296, 0.000347, 0.000406, 0.000475, 0.000556
	Black	Women	60	0.000325, 0.000378, 0.000439, 0.00051, 0.000594
	Black	Women	61	0.000349, 0.000407, 0.000472, 0.000549, 0.000639
	Black	Women	62	0.000371, 0.000433, 0.000505, 0.000589, 0.000688
	Black	Women	63	0.000393, 0.00046, 0.000537, 0.000627, 0.000734
	Black	Women	64	0.000418, 0.000488, 0.000569, 0.000664, 0.000775
	Black	Women	65	0.000448, 0.000521, 0.000605, 0.000702, 0.000816
	Black	Women	66	0.000485, 0.00056, 0.000646, 0.000745, 0.000861
	Black	Women	67	0.000529, 0.000607, 0.000696, 0.000798, 0.000916
	Black	Women	68	0.000583, 0.000665, 0.000757, 0.000862, 0.000984
	Black	Women	69	0.000648, 0.000734, 0.000831, 0.000941, 0.00107
	Black	Women	70	0.000722, 0.000815, 0.00092, 0.00104, 0.00117
	Black	Women	71	0.000803, 0.000907, 0.00102, 0.00115, 0.0013
	Black	Women	72	0.000893, 0.00101, 0.00114, 0.00129, 0.00146
	Black	Women	73	0.000996, 0.00113, 0.00127, 0.00144, 0.00163
	Black	Women	74	0.00112, 0.00126, 0.00143, 0.00161, 0.00182
	Black	Women	75	0.00125, 0.00141, 0.0016, 0.00181, 0.00204
	Black	Women	76	0.00139, 0.00158, 0.00179, 0.00204, 0.00232
	Black	Women	77	0.00153, 0.00176, 0.00201, 0.00231, 0.00266
	Black	Women	78	0.00168, 0.00195, 0.00227, 0.00263, 0.00306
	Black	Women	79	0.00185, 0.00217, 0.00255, 0.00299, 0.00351
	Black	Women	80	0.00204, 0.00241, 0.00285, 0.00337, 0.00399
	Black	Women	81	0.00225, 0.00268, 0.00318, 0.00377, 0.00448

Variable	Race/ethnicity	Sex	Age	Distribution
	Black	Women	82	0.00247, 0.00295, 0.00352, 0.00419, 0.005
	Black	Women	83	0.00268, 0.00322, 0.00386, 0.00463, 0.00555
	Black	Women	84	0.00288, 0.00348, 0.0042, 0.00507, 0.00613
	Hispanic	Men	30	0.00000717, 0.0000101, 0.0000141, 0.0000197, 0.0000276
	Hispanic	Men	31	0.00000865, 0.0000118, 0.000016, 0.0000217, 0.0000295
	Hispanic	Men	32	0.0000103, 0.0000137, 0.0000181, 0.000024, 0.0000318
	Hispanic	Men	33	0.0000121, 0.0000158, 0.0000205, 0.0000267, 0.0000348
	Hispanic	Men	34	0.0000139, 0.0000179, 0.0000231, 0.0000298, 0.0000386
	Hispanic	Men	35	0.0000158, 0.0000203, 0.000026, 0.0000333, 0.0000429
	Hispanic	Men	36	0.0000178, 0.0000228, 0.000029, 0.000037, 0.0000473
	Hispanic	Men	37	0.0000202, 0.0000255, 0.0000323, 0.0000408, 0.0000516
	Hispanic	Men	38	0.0000227, 0.0000285, 0.0000357, 0.0000447, 0.000056
	Hispanic	Men	39	0.0000254, 0.0000316, 0.0000393, 0.0000488, 0.0000607
	Hispanic	Men	40	0.0000282, 0.0000349, 0.000043, 0.0000531, 0.0000656
	Hispanic	Men	41	0.0000313, 0.0000384, 0.0000469, 0.0000574, 0.0000704
	Hispanic	Men	42	0.0000349, 0.0000423, 0.0000511, 0.0000618, 0.0000749
	Hispanic	Men	43	0.0000387, 0.0000465, 0.0000557, 0.0000668, 0.0000802
	Hispanic	Men	44	0.0000425, 0.0000509, 0.0000608, 0.0000727, 0.000087
	Hispanic	Men	45	0.0000467, 0.0000558, 0.0000666, 0.0000795, 0.000095
	Hispanic	Men	46	0.0000516, 0.0000615, 0.0000733, 0.0000872, 0.000104
	Hispanic	Men	47	0.0000574, 0.0000683, 0.000081, 0.000096, 0.000114
	Hispanic	Men	48	0.0000644, 0.0000761, 0.0000899, 0.000106, 0.000126
	Hispanic	Men	49	0.0000728, 0.0000855, 0.0001, 0.000118, 0.000138
	Hispanic	Men	50	0.0000831, 0.0000966, 0.000112, 0.00013, 0.000152
	Hispanic	Men	51	0.0000954, 0.00011, 0.000126, 0.000145, 0.000166
	Hispanic	Men	52	0.000109, 0.000124, 0.000141, 0.000161, 0.000183
	Hispanic	Men	53	0.00012, 0.000138, 0.000157, 0.00018, 0.000206
	Hispanic	Men	54	0.00013, 0.000151, 0.000175, 0.000203, 0.000236
	Hispanic	Men	55	0.000139, 0.000164, 0.000193, 0.000227, 0.000268
	Hispanic	Men	56	0.00015, 0.000178, 0.000212, 0.000252, 0.0003
	Hispanic	Men	57	0.000163, 0.000194, 0.000231, 0.000275, 0.000328
	Hispanic	Men	58	0.000179, 0.000212, 0.000252, 0.000298, 0.000354
	Hispanic	Men	59	0.000198, 0.000233, 0.000273, 0.00032, 0.000376
	Hispanic	Men	60	0.000219, 0.000254, 0.000295, 0.000343, 0.000398
	Hispanic	Men	61	0.00024, 0.000276, 0.000318, 0.000366, 0.000422
	Hispanic	Men	62	0.000261, 0.000299, 0.000342, 0.000391, 0.000448
	Hispanic	Men	63	0.000281, 0.000321, 0.000367, 0.000419, 0.000479
	Hispanic	Men	64	0.000301, 0.000344, 0.000394, 0.00045, 0.000515
	Hispanic	Men	65	0.000323, 0.00037, 0.000424, 0.000485, 0.000556
	Hispanic	Men	66	0.000347, 0.000399, 0.000459, 0.000527, 0.000605
	Hispanic	Men	67	0.000376, 0.000434, 0.0005, 0.000577, 0.000666
	Hispanic	Men	68	0.000409, 0.000474, 0.000549, 0.000636, 0.000738
	Hispanic	Men	69	0.00045, 0.000523, 0.000607, 0.000706, 0.000821

Variable	Race/ethnicity	Sex	Age	Distribution
	Hispanic	Men	70	0.000499, 0.000581, 0.000675, 0.000784, 0.000912
	Hispanic	Men	71	0.000559, 0.000649, 0.000752, 0.000873, 0.00101
	Hispanic	Men	72	0.000626, 0.000726, 0.00084, 0.000973, 0.00113
	Hispanic	Men	73	0.0007, 0.000811, 0.000938, 0.00109, 0.00126
	Hispanic	Men	74	0.000782, 0.000906, 0.00105, 0.00121, 0.00141
	Hispanic	Men	75	0.000874, 0.00101, 0.00117, 0.00136, 0.00157
	Hispanic	Men	76	0.000977, 0.00113, 0.00131, 0.00152, 0.00177
	Hispanic	Men	77	0.00109, 0.00127, 0.00147, 0.00171, 0.00199
	Hispanic	Men	78	0.00122, 0.00142, 0.00166, 0.00193, 0.00225
	Hispanic	Men	79	0.00135, 0.00158, 0.00186, 0.00218, 0.00256
	Hispanic	Men	80	0.00149, 0.00176, 0.00208, 0.00245, 0.0029
	Hispanic	Men	81	0.00163, 0.00194, 0.00231, 0.00275, 0.00327
	Hispanic	Men	82	0.00177, 0.00213, 0.00255, 0.00306, 0.00368
	Hispanic	Men	83	0.00191, 0.00231, 0.0028, 0.00339, 0.00411
	Hispanic	Men	84	0.00204, 0.00249, 0.00304, 0.00371, 0.00454
	Hispanic	Women	30	0.00000409, 0.0000063, 0.00000966, 0.0000148, 0.0000228
	Hispanic	Women	31	0.00000486, 0.00000728, 0.0000109, 0.0000162, 0.0000242
	Hispanic	Women	32	0.00000571, 0.00000838, 0.0000123, 0.0000179, 0.0000263
	Hispanic	Women	33	0.00000664, 0.00000962, 0.0000139, 0.00002, 0.000029
	Hispanic	Women	34	0.00000769, 0.000011, 0.0000157, 0.0000224, 0.0000321
	Hispanic	Women	35	0.00000894, 0.0000126, 0.0000177, 0.0000249, 0.0000352
	Hispanic	Women	36	0.0000104, 0.0000144, 0.0000199, 0.0000275, 0.0000381
	Hispanic	Women	37	0.0000121, 0.0000164, 0.0000222, 0.0000301, 0.0000409
	Hispanic	Women	38	0.0000139, 0.0000185, 0.0000247, 0.0000328, 0.0000438
	Hispanic	Women	39	0.0000158, 0.0000208, 0.0000272, 0.0000357, 0.0000469
	Hispanic	Women	40	0.0000178, 0.0000231, 0.0000299, 0.0000386, 0.0000501
	Hispanic	Women	41	0.0000199, 0.0000255, 0.0000326, 0.0000417, 0.0000535
	Hispanic	Women	42	0.0000221, 0.0000281, 0.0000356, 0.0000451, 0.0000573
	Hispanic	Women	43	0.0000244, 0.0000308, 0.0000388, 0.000049, 0.000062
	Hispanic	Women	44	0.0000269, 0.0000338, 0.0000425, 0.0000534, 0.0000672
	Hispanic	Women	45	0.0000301, 0.0000375, 0.0000466, 0.0000578, 0.000072
	Hispanic	Women	46	0.0000344, 0.000042, 0.0000512, 0.0000624, 0.0000762
	Hispanic	Women	47	0.0000393, 0.0000471, 0.0000563, 0.0000673, 0.0000806
	Hispanic	Women	48	0.0000445, 0.0000526, 0.0000621, 0.0000732, 0.0000866
	Hispanic	Women	49	0.0000493, 0.0000582, 0.0000685, 0.0000807, 0.0000953
	Hispanic	Women	50	0.0000537, 0.0000639, 0.0000758, 0.00009, 0.000107
	Hispanic	Women	51	0.0000581, 0.0000699, 0.0000839, 0.000101, 0.000121
	Hispanic	Women	52	0.0000626, 0.0000763, 0.0000929, 0.000113, 0.000138
	Hispanic	Women	53	0.0000675, 0.0000833, 0.000103, 0.000126, 0.000156
	Hispanic	Women	54	0.000073, 0.000091, 0.000113, 0.00014, 0.000175
	Hispanic	Women	55	0.0000797, 0.0000996, 0.000124, 0.000155, 0.000193
	Hispanic	Women	56	0.0000876, 0.000109, 0.000136, 0.000169, 0.000211
	Hispanic	Women	57	0.0000966, 0.00012, 0.000148, 0.000184, 0.000228

Variable	Race/ethnicity	Sex	Age	Distribution
	Hispanic	Women	58	0.000106, 0.000131, 0.000162, 0.0002, 0.000247
	Hispanic	Women	59	0.000117, 0.000144, 0.000177, 0.000217, 0.000268
	Hispanic	Women	60	0.000128, 0.000157, 0.000192, 0.000236, 0.00029
	Hispanic	Women	61	0.00014, 0.000171, 0.000209, 0.000256, 0.000313
	Hispanic	Women	62	0.000152, 0.000186, 0.000226, 0.000276, 0.000337
	Hispanic	Women	63	0.000164, 0.000201, 0.000244, 0.000298, 0.000363
	Hispanic	Women	64	0.000177, 0.000217, 0.000264, 0.000322, 0.000393
	Hispanic	Women	65	0.000191, 0.000234, 0.000286, 0.000349, 0.000426
	Hispanic	Women	66	0.000208, 0.000255, 0.000311, 0.000381, 0.000466
	Hispanic	Women	67	0.000227, 0.000279, 0.000342, 0.000419, 0.000515
	Hispanic	Women	68	0.000252, 0.000309, 0.00038, 0.000466, 0.000572
	Hispanic	Women	69	0.000283, 0.000347, 0.000425, 0.000521, 0.000639
	Hispanic	Women	70	0.000323, 0.000394, 0.000479, 0.000583, 0.000712
	Hispanic	Women	71	0.000374, 0.000451, 0.000543, 0.000654, 0.000789
	Hispanic	Women	72	0.000437, 0.00052, 0.000617, 0.000733, 0.000872
	Hispanic	Women	73	0.000513, 0.000601, 0.000703, 0.000822, 0.000964
	Hispanic	Women	74	0.000599, 0.000693, 0.000801, 0.000927, 0.00107
	Hispanic	Women	75	0.000693, 0.000797, 0.000915, 0.00105, 0.00121
	Hispanic	Women	76	0.000796, 0.000914, 0.00105, 0.0012, 0.00138
	Hispanic	Women	77	0.000912, 0.00105, 0.0012, 0.00138, 0.00159
	Hispanic	Women	78	0.00105, 0.00121, 0.00138, 0.00158, 0.00182
	Hispanic	Women	79	0.00121, 0.00139, 0.00159, 0.00182, 0.00208
	Hispanic	Women	80	0.00139, 0.00159, 0.00182, 0.00208, 0.00238
	Hispanic	Women	81	0.00156, 0.0018, 0.00207, 0.00238, 0.00274
	Hispanic	Women	82	0.00171, 0.002, 0.00233, 0.00272, 0.00318
	Hispanic	Women	83	0.00184, 0.00219, 0.00261, 0.0031, 0.0037
	Hispanic	Women	84	0.00195, 0.00238, 0.00288, 0.0035, 0.00426
	White	Men	30	0.00000569, 0.00000782, 0.0000107, 0.0000147, 0.0000202
	White	Men	31	0.0000067, 0.00000901, 0.0000121, 0.0000162, 0.0000218
	White	Men	32	0.00000783, 0.0000103, 0.0000136, 0.0000179, 0.0000236
	White	Men	33	0.0000091, 0.0000118, 0.0000153, 0.0000198, 0.0000257
	White	Men	34	0.0000105, 0.0000134, 0.0000171, 0.0000219, 0.000028
	White	Men	35	0.0000119, 0.0000151, 0.0000191, 0.0000241, 0.0000306
	White	Men	36	0.0000134, 0.0000168, 0.0000212, 0.0000266, 0.0000336
	White	Men	37	0.0000149, 0.0000187, 0.0000234, 0.0000293, 0.0000368
	White	Men	38	0.0000165, 0.0000207, 0.0000258, 0.0000322, 0.0000403
	White	Men	39	0.0000182, 0.0000227, 0.0000283, 0.0000352, 0.000044
	White	Men	40	0.0000201, 0.0000249, 0.0000309, 0.0000383, 0.0000476
	White	Men	41	0.0000224, 0.0000275, 0.0000337, 0.0000413, 0.0000507
	White	Men	42	0.0000251, 0.0000304, 0.0000366, 0.0000442, 0.0000533
	White	Men	43	0.0000283, 0.0000337, 0.0000399, 0.0000474, 0.0000563
	White	Men	44	0.0000317, 0.0000372, 0.0000437, 0.0000513, 0.0000603
	White	Men	45	0.0000354, 0.0000413, 0.0000481, 0.000056, 0.0000653

Variable	Race/ethnicity	Sex	Age	Distribution
	White	Men	46	0.0000397, 0.000046, 0.0000532, 0.0000615, 0.0000713
	White	Men	47	0.0000445, 0.0000513, 0.0000591, 0.0000681, 0.0000786
	White	Men	48	0.0000495, 0.0000572, 0.000066, 0.0000761, 0.000088
	White	Men	49	0.0000548, 0.0000637, 0.000074, 0.0000859, 0.0000999
	White	Men	50	0.0000606, 0.0000711, 0.0000831, 0.0000973, 0.000114
	White	Men	51	0.0000678, 0.0000797, 0.0000935, 0.00011, 0.000129
	White	Men	52	0.0000767, 0.0000898, 0.000105, 0.000123, 0.000144
	White	Men	53	0.0000867, 0.000101, 0.000118, 0.000137, 0.00016
	White	Men	54	0.0000971, 0.000113, 0.000131, 0.000152, 0.000177
	White	Men	55	0.000107, 0.000125, 0.000146, 0.000169, 0.000197
	White	Men	56	0.000118, 0.000138, 0.000161, 0.000188, 0.00022
	White	Men	57	0.000128, 0.000151, 0.000177, 0.000208, 0.000245
	White	Men	58	0.000139, 0.000165, 0.000195, 0.00023, 0.000273
	White	Men	59	0.000152, 0.00018, 0.000214, 0.000254, 0.000302
	White	Men	60	0.000167, 0.000198, 0.000235, 0.000278, 0.000329
	White	Men	61	0.000186, 0.000219, 0.000257, 0.000302, 0.000355
	White	Men	62	0.000207, 0.000241, 0.00028, 0.000326, 0.000379
	White	Men	63	0.000232, 0.000266, 0.000305, 0.00035, 0.000401
	White	Men	64	0.00026, 0.000294, 0.000332, 0.000375, 0.000425
	White	Men	65	0.00029, 0.000325, 0.000363, 0.000405, 0.000453
	White	Men	66	0.000323, 0.000359, 0.000398, 0.000442, 0.000491
	White	Men	67	0.000357, 0.000397, 0.00044, 0.000488, 0.000542
	White	Men	68	0.000396, 0.000441, 0.000491, 0.000546, 0.000608
	White	Men	69	0.000441, 0.000493, 0.000551, 0.000616, 0.000688
	White	Men	70	0.000494, 0.000554, 0.000622, 0.000697, 0.000783
	White	Men	71	0.000554, 0.000625, 0.000703, 0.000791, 0.000892
	White	Men	72	0.000622, 0.000704, 0.000795, 0.000898, 0.00102
	White	Men	73	0.000697, 0.000792, 0.000899, 0.00102, 0.00116
	White	Men	74	0.000778, 0.00089, 0.00102, 0.00116, 0.00132
	White	Men	75	0.000871, 0.001, 0.00115, 0.00132, 0.00151
	White	Men	76	0.000977, 0.00113, 0.0013, 0.0015, 0.00174
	White	Men	77	0.0011, 0.00128, 0.00148, 0.00172, 0.00201
	White	Men	78	0.00123, 0.00145, 0.0017, 0.00199, 0.00234
	White	Men	79	0.00138, 0.00164, 0.00194, 0.00231, 0.00274
	White	Men	80	0.00153, 0.00185, 0.00222, 0.00267, 0.00322
	White	Men	81	0.00171, 0.00208, 0.00253, 0.00308, 0.00375
	White	Men	82	0.00191, 0.00234, 0.00287, 0.00351, 0.00431
	White	Men	83	0.00211, 0.0026, 0.00321, 0.00396, 0.0049
	White	Men	84	0.00229, 0.00286, 0.00356, 0.00442, 0.00551
	White	Women	30	0.00000506, 0.00000712, 0.00001, 0.000014, 0.0000198
	White	Women	31	0.00000594, 0.00000823, 0.0000114, 0.0000157, 0.0000217
	White	Women	32	0.00000699, 0.00000951, 0.0000129, 0.0000175, 0.0000239
	White	Women	33	0.00000818, 0.000011, 0.0000146, 0.0000196, 0.0000262

Variable	Race/ethnicity	Sex	Age	Distribution
	White	Women	34	0.00000947, 0.0000125, 0.0000165, 0.0000217, 0.0000287
	White	Women	35	0.0000108, 0.0000142, 0.0000184, 0.0000241, 0.0000314
	White	Women	36	0.0000123, 0.0000159, 0.0000205, 0.0000264, 0.0000342
	White	Women	37	0.0000138, 0.0000176, 0.0000225, 0.0000288, 0.000037
	White	Women	38	0.0000153, 0.0000195, 0.0000247, 0.0000313, 0.0000397
	White	Women	39	0.000017, 0.0000214, 0.0000268, 0.0000337, 0.0000425
	White	Women	40	0.0000187, 0.0000234, 0.000029, 0.0000361, 0.000045
	White	Women	41	0.0000208, 0.0000256, 0.0000313, 0.0000383, 0.000047
	White	Women	42	0.0000233, 0.000028, 0.0000337, 0.0000404, 0.0000486
	White	Women	43	0.000026, 0.0000307, 0.0000363, 0.0000428, 0.0000506
	White	Women	44	0.0000285, 0.0000335, 0.0000392, 0.0000459, 0.0000539
	White	Women	45	0.0000311, 0.0000364, 0.0000426, 0.0000498, 0.0000584
	White	Women	46	0.0000338, 0.0000397, 0.0000465, 0.0000545, 0.0000639
	White	Women	47	0.0000368, 0.0000434, 0.000051, 0.0000599, 0.0000705
	White	Women	48	0.00004, 0.0000474, 0.0000561, 0.0000664, 0.0000787
	White	Women	49	0.0000433, 0.0000518, 0.0000619, 0.0000739, 0.0000885
	White	Women	50	0.0000471, 0.0000568, 0.0000685, 0.0000824, 0.0000995
	White	Women	51	0.0000518, 0.0000627, 0.0000757, 0.0000914, 0.000111
	White	Women	52	0.0000577, 0.0000695, 0.0000836, 0.000101, 0.000121
	White	Women	53	0.0000644, 0.0000771, 0.000092, 0.00011, 0.000131
	White	Women	54	0.0000719, 0.0000852, 0.000101, 0.000119, 0.000141
	White	Women	55	0.0000799, 0.0000938, 0.00011, 0.000129, 0.000151
	White	Women	56	0.0000886, 0.000103, 0.00012, 0.000139, 0.000162
	White	Women	57	0.0000981, 0.000113, 0.00013, 0.00015, 0.000173
	White	Women	58	0.000109, 0.000124, 0.000142, 0.000162, 0.000185
	White	Women	59	0.00012, 0.000137, 0.000155, 0.000176, 0.0002
	White	Women	60	0.000133, 0.00015, 0.00017, 0.000192, 0.000218
	White	Women	61	0.000146, 0.000165, 0.000187, 0.00021, 0.000238
	White	Women	62	0.000161, 0.000182, 0.000205, 0.000231, 0.00026
	White	Women	63	0.000177, 0.000199, 0.000224, 0.000253, 0.000285
	White	Women	64	0.000195, 0.000219, 0.000246, 0.000276, 0.000311
	White	Women	65	0.000215, 0.000241, 0.00027, 0.000303, 0.00034
	White	Women	66	0.000238, 0.000267, 0.000299, 0.000335, 0.000376
	White	Women	67	0.000265, 0.000297, 0.000334, 0.000374, 0.00042
	White	Women	68	0.000298, 0.000335, 0.000376, 0.000422, 0.000474
	White	Women	69	0.000339, 0.00038, 0.000427, 0.000479, 0.000539
	White	Women	70	0.000389, 0.000436, 0.000489, 0.000548, 0.000615
	White	Women	71	0.000447, 0.000502, 0.000563, 0.00063, 0.000707
	White	Women	72	0.000514, 0.000577, 0.000648, 0.000728, 0.000818
	White	Women	73	0.000589, 0.000664, 0.000747, 0.00084, 0.000947
	White	Women	74	0.000676, 0.000763, 0.00086, 0.000969, 0.00109
	White	Women	75	0.000778, 0.000878, 0.000989, 0.00111, 0.00126
	White	Women	76	0.000896, 0.00101, 0.00114, 0.00129, 0.00145

Variable	Race/ethnicity	Sex	Age	Distribution
	White	Women	77	0.00103, 0.00117, 0.00132, 0.00149, 0.00169
	White	Women	78	0.00117, 0.00134, 0.00153, 0.00174, 0.00199
	White	Women	79	0.00134, 0.00154, 0.00178, 0.00204, 0.00236
	White	Women	80	0.00153, 0.00178, 0.00207, 0.00239, 0.00278
	White	Women	81	0.00176, 0.00205, 0.00239, 0.00279, 0.00326
	White	Women	82	0.002, 0.00235, 0.00275, 0.00323, 0.00379
	White	Women	83	0.00226, 0.00266, 0.00314, 0.0037, 0.00436
	White	Women	84	0.00252, 0.00298, 0.00353, 0.00418, 0.00496
Stroke mortality rates for 2026 (0.01, 0.2, 0.5, 0.8. 0.99 percentiles of the empirical distribution produced during forecasting)				
	Black	Men	30	0.0000153, 0.0000214, 0.0000298, 0.0000414, 0.0000579
	Black	Men	31	0.0000189, 0.0000257, 0.0000348, 0.0000471, 0.0000639
	Black	Men	32	0.0000228, 0.0000304, 0.0000405, 0.0000539, 0.0000719
	Black	Men	33	0.0000267, 0.0000354, 0.0000468, 0.0000618, 0.0000818
	Black	Men	34	0.0000308, 0.0000407, 0.0000536, 0.0000706, 0.0000932
	Black	Men	35	0.0000351, 0.0000462, 0.0000607, 0.0000798, 0.000105
	Black	Men	36	0.0000396, 0.000052, 0.000068, 0.0000891, 0.000117
	Black	Men	37	0.0000446, 0.0000581, 0.0000754, 0.000098, 0.000128
	Black	Men	38	0.0000501, 0.0000645, 0.0000828, 0.000106, 0.000137
	Black	Men	39	0.0000559, 0.0000711, 0.0000903, 0.000115, 0.000146
	Black	Men	40	0.0000621, 0.0000781, 0.0000981, 0.000123, 0.000155
	Black	Men	41	0.0000693, 0.0000859, 0.000106, 0.000132, 0.000163
	Black	Men	42	0.0000783, 0.0000952, 0.000116, 0.00014, 0.000171
	Black	Men	43	0.0000879, 0.000105, 0.000126, 0.000151, 0.000182
	Black	Men	44	0.0000963, 0.000116, 0.000139, 0.000167, 0.0002
	Black	Men	45	0.000105, 0.000127, 0.000153, 0.000185, 0.000224
	Black	Men	46	0.000116, 0.00014, 0.00017, 0.000206, 0.00025
	Black	Men	47	0.000129, 0.000156, 0.00019, 0.00023, 0.000279
	Black	Men	48	0.000142, 0.000174, 0.000212, 0.000259, 0.000317
	Black	Men	49	0.000158, 0.000195, 0.000239, 0.000294, 0.000363
	Black	Men	50	0.00018, 0.000221, 0.000271, 0.000332, 0.000409
	Black	Men	51	0.00021, 0.000255, 0.000308, 0.000372, 0.00045
	Black	Men	52	0.000249, 0.000295, 0.000349, 0.000412, 0.000489
	Black	Men	53	0.000288, 0.000337, 0.000393, 0.000458, 0.000535
	Black	Men	54	0.000325, 0.000378, 0.000439, 0.000509, 0.000591
	Black	Men	55	0.000361, 0.000418, 0.000484, 0.000561, 0.000651
	Black	Men	56	0.000394, 0.000458, 0.00053, 0.000614, 0.000713
	Black	Men	57	0.000428, 0.000497, 0.000576, 0.000667, 0.000774
	Black	Men	58	0.000466, 0.000539, 0.000622, 0.000719, 0.000832
	Black	Men	59	0.000505, 0.000582, 0.00067, 0.00077, 0.000888
	Black	Men	60	0.00054, 0.000623, 0.000718, 0.000827, 0.000955
	Black	Men	61	0.000569, 0.00066, 0.000766, 0.000888, 0.00103
	Black	Men	62	0.000601, 0.000699, 0.000813, 0.000944, 0.0011
	Black	Men	63	0.000643, 0.000743, 0.000859, 0.000992, 0.00115

Variable	Race/ethnicity	Sex	Age	Distribution
	Black	Men	64	0.000692, 0.000792, 0.000906, 0.00104, 0.00119
	Black	Men	65	0.000737, 0.000841, 0.000958, 0.00109, 0.00124
	Black	Men	66	0.00078, 0.000892, 0.00102, 0.00116, 0.00133
	Black	Men	67	0.000832, 0.000954, 0.00109, 0.00125, 0.00143
	Black	Men	68	0.000901, 0.00103, 0.00118, 0.00134, 0.00154
	Black	Men	69	0.000983, 0.00112, 0.00128, 0.00145, 0.00166
	Black	Men	70	0.00107, 0.00122, 0.00139, 0.00158, 0.0018
	Black	Men	71	0.00116, 0.00133, 0.00151, 0.00171, 0.00195
	Black	Men	72	0.00126, 0.00144, 0.00163, 0.00186, 0.00211
	Black	Men	73	0.00136, 0.00155, 0.00177, 0.00201, 0.0023
	Black	Men	74	0.00145, 0.00167, 0.00191, 0.00219, 0.00251
	Black	Men	75	0.00155, 0.00179, 0.00207, 0.00239, 0.00276
	Black	Men	76	0.00167, 0.00194, 0.00225, 0.0026, 0.00302
	Black	Men	77	0.00182, 0.00211, 0.00245, 0.00284, 0.0033
	Black	Men	78	0.00198, 0.0023, 0.00267, 0.00311, 0.00361
	Black	Men	79	0.00214, 0.0025, 0.00292, 0.0034, 0.00397
	Black	Men	80	0.0023, 0.00271, 0.00317, 0.00372, 0.00436
	Black	Men	81	0.00247, 0.00291, 0.00343, 0.00404, 0.00476
	Black	Men	82	0.00263, 0.00311, 0.00368, 0.00435, 0.00515
	Black	Men	83	0.00279, 0.00331, 0.00392, 0.00465, 0.00553
	Black	Men	84	0.00293, 0.0035, 0.00416, 0.00495, 0.0059
	Black	Women	30	0.0000107, 0.0000161, 0.0000242, 0.0000362, 0.0000545
	Black	Women	31	0.0000125, 0.0000186, 0.0000278, 0.0000414, 0.000062
	Black	Women	32	0.0000143, 0.0000214, 0.0000319, 0.0000475, 0.0000712
	Black	Women	33	0.0000161, 0.0000242, 0.0000364, 0.0000545, 0.0000821
	Black	Women	34	0.000018, 0.0000273, 0.0000413, 0.0000624, 0.0000947
	Black	Women	35	0.00002, 0.0000306, 0.0000465, 0.0000707, 0.000108
	Black	Women	36	0.0000225, 0.0000343, 0.000052, 0.0000789, 0.00012
	Black	Women	37	0.0000257, 0.0000386, 0.0000578, 0.0000865, 0.00013
	Black	Women	38	0.0000296, 0.0000435, 0.0000638, 0.0000937, 0.000138
	Black	Women	39	0.0000337, 0.0000487, 0.0000702, 0.000101, 0.000146
	Black	Women	40	0.0000378, 0.000054, 0.0000767, 0.000109, 0.000156
	Black	Women	41	0.0000421, 0.0000594, 0.0000835, 0.000117, 0.000166
	Black	Women	42	0.0000472, 0.0000655, 0.0000907, 0.000126, 0.000175
	Black	Women	43	0.0000531, 0.0000724, 0.0000986, 0.000134, 0.000183
	Black	Women	44	0.0000592, 0.0000799, 0.000107, 0.000144, 0.000195
	Black	Women	45	0.0000658, 0.000088, 0.000117, 0.000156, 0.000209
	Black	Women	46	0.000074, 0.0000977, 0.000129, 0.000169, 0.000224
	Black	Women	47	0.0000845, 0.000109, 0.000142, 0.000183, 0.000237
	Black	Women	48	0.0000968, 0.000123, 0.000156, 0.000198, 0.000252
	Black	Women	49	0.00011, 0.000137, 0.000172, 0.000215, 0.00027
	Black	Women	50	0.000122, 0.000152, 0.000189, 0.000235, 0.000293
	Black	Women	51	0.000135, 0.000167, 0.000207, 0.000256, 0.000318

Variable	Race/ethnicity	Sex	Age	Distribution
	Black	Women	52	0.00015, 0.000184, 0.000226, 0.000277, 0.000341
	Black	Women	53	0.000166, 0.000202, 0.000246, 0.000299, 0.000364
	Black	Women	54	0.000181, 0.00022, 0.000266, 0.000323, 0.000393
	Black	Women	55	0.000195, 0.000237, 0.000289, 0.000351, 0.000427
	Black	Women	56	0.000212, 0.000257, 0.000313, 0.00038, 0.000463
	Black	Women	57	0.000233, 0.000282, 0.00034, 0.00041, 0.000495
	Black	Women	58	0.00026, 0.00031, 0.000369, 0.000439, 0.000524
	Black	Women	59	0.000289, 0.000341, 0.0004, 0.000471, 0.000554
	Black	Women	60	0.000317, 0.000371, 0.000433, 0.000505, 0.000591
	Black	Women	61	0.000341, 0.000399, 0.000465, 0.000543, 0.000635
	Black	Women	62	0.000362, 0.000424, 0.000497, 0.000581, 0.000681
	Black	Women	63	0.000383, 0.00045, 0.000527, 0.000618, 0.000726
	Black	Women	64	0.000407, 0.000477, 0.000559, 0.000654, 0.000766
	Black	Women	65	0.000436, 0.000509, 0.000592, 0.00069, 0.000805
	Black	Women	66	0.000471, 0.000546, 0.000632, 0.000732, 0.000848
	Black	Women	67	0.000513, 0.000592, 0.000681, 0.000783, 0.000902
	Black	Women	68	0.000565, 0.000647, 0.00074, 0.000846, 0.000969
	Black	Women	69	0.000628, 0.000715, 0.000813, 0.000924, 0.00105
	Black	Women	70	0.0007, 0.000793, 0.000899, 0.00102, 0.00115
	Black	Women	71	0.000778, 0.000882, 0.000999, 0.00113, 0.00128
	Black	Women	72	0.000865, 0.000982, 0.00111, 0.00126, 0.00143
	Black	Women	73	0.000965, 0.0011, 0.00124, 0.00141, 0.0016
	Black	Women	74	0.00108, 0.00123, 0.00139, 0.00158, 0.00179
	Black	Women	75	0.00121, 0.00137, 0.00156, 0.00177, 0.00201
	Black	Women	76	0.00134, 0.00153, 0.00175, 0.00199, 0.00227
	Black	Women	77	0.00148, 0.00171, 0.00196, 0.00226, 0.0026
	Black	Women	78	0.00162, 0.00189, 0.00221, 0.00257, 0.003
	Black	Women	79	0.00179, 0.00211, 0.00248, 0.00292, 0.00345
	Black	Women	80	0.00197, 0.00234, 0.00278, 0.00329, 0.00391
	Black	Women	81	0.00218, 0.0026, 0.00309, 0.00369, 0.0044
	Black	Women	82	0.00239, 0.00286, 0.00342, 0.0041, 0.00491
	Black	Women	83	0.00259, 0.00312, 0.00376, 0.00452, 0.00546
	Black	Women	84	0.00277, 0.00337, 0.00409, 0.00495, 0.00602
	Hispanic	Men	30	0.00000702, 0.00000993, 0.000014, 0.0000198, 0.000028
	Hispanic	Men	31	0.00000849, 0.0000116, 0.0000159, 0.0000217, 0.0000298
	Hispanic	Men	32	0.0000101, 0.0000135, 0.000018, 0.000024, 0.0000321
	Hispanic	Men	33	0.0000119, 0.0000156, 0.0000204, 0.0000267, 0.0000351
	Hispanic	Men	34	0.0000137, 0.0000178, 0.000023, 0.0000299, 0.0000389
	Hispanic	Men	35	0.0000155, 0.00002, 0.0000259, 0.0000334, 0.0000432
	Hispanic	Men	36	0.0000175, 0.0000225, 0.0000289, 0.0000371, 0.0000477
	Hispanic	Men	37	0.0000197, 0.0000252, 0.000032, 0.0000408, 0.000052
	Hispanic	Men	38	0.0000222, 0.0000281, 0.0000354, 0.0000446, 0.0000563
	Hispanic	Men	39	0.0000248, 0.000031, 0.0000388, 0.0000486, 0.0000609

Variable	Race/ethnicity	Sex	Age	Distribution
	Hispanic	Men	40	0.0000274, 0.0000342, 0.0000424, 0.0000527, 0.0000656
	Hispanic	Men	41	0.0000304, 0.0000375, 0.0000462, 0.0000568, 0.0000701
	Hispanic	Men	42	0.0000339, 0.0000413, 0.0000502, 0.000061, 0.0000742
	Hispanic	Men	43	0.0000377, 0.0000454, 0.0000546, 0.0000657, 0.0000791
	Hispanic	Men	44	0.0000414, 0.0000497, 0.0000595, 0.0000713, 0.0000856
	Hispanic	Men	45	0.0000454, 0.0000544, 0.0000651, 0.0000779, 0.0000934
	Hispanic	Men	46	0.0000502, 0.00006, 0.0000716, 0.0000854, 0.000102
	Hispanic	Men	47	0.0000559, 0.0000665, 0.0000791, 0.000094, 0.000112
	Hispanic	Men	48	0.0000627, 0.0000743, 0.0000878, 0.000104, 0.000123
	Hispanic	Men	49	0.0000709, 0.0000835, 0.0000981, 0.000115, 0.000136
	Hispanic	Men	50	0.000081, 0.0000944, 0.00011, 0.000128, 0.000149
	Hispanic	Men	51	0.0000932, 0.000107, 0.000123, 0.000142, 0.000164
	Hispanic	Men	52	0.000106, 0.000121, 0.000138, 0.000158, 0.000181
	Hispanic	Men	53	0.000118, 0.000135, 0.000155, 0.000177, 0.000204
	Hispanic	Men	54	0.000127, 0.000148, 0.000172, 0.0002, 0.000233
	Hispanic	Men	55	0.000136, 0.000161, 0.00019, 0.000225, 0.000265
	Hispanic	Men	56	0.000147, 0.000175, 0.000209, 0.000249, 0.000297
	Hispanic	Men	57	0.00016, 0.000191, 0.000228, 0.000272, 0.000325
	Hispanic	Men	58	0.000176, 0.000209, 0.000248, 0.000295, 0.00035
	Hispanic	Men	59	0.000194, 0.000229, 0.000269, 0.000316, 0.000373
	Hispanic	Men	60	0.000214, 0.00025, 0.000291, 0.000338, 0.000395
	Hispanic	Men	61	0.000234, 0.000271, 0.000313, 0.000361, 0.000418
	Hispanic	Men	62	0.000254, 0.000293, 0.000336, 0.000386, 0.000444
	Hispanic	Men	63	0.000273, 0.000314, 0.00036, 0.000413, 0.000474
	Hispanic	Men	64	0.000293, 0.000336, 0.000386, 0.000442, 0.000508
	Hispanic	Men	65	0.000314, 0.000361, 0.000415, 0.000476, 0.000548
	Hispanic	Men	66	0.000338, 0.00039, 0.000449, 0.000516, 0.000595
	Hispanic	Men	67	0.000365, 0.000423, 0.000489, 0.000565, 0.000654
	Hispanic	Men	68	0.000397, 0.000462, 0.000537, 0.000623, 0.000725
	Hispanic	Men	69	0.000436, 0.000509, 0.000593, 0.000691, 0.000807
	Hispanic	Men	70	0.000485, 0.000566, 0.000659, 0.000768, 0.000897
	Hispanic	Men	71	0.000542, 0.000632, 0.000735, 0.000855, 0.000996
	Hispanic	Men	72	0.000607, 0.000706, 0.00082, 0.000952, 0.00111
	Hispanic	Men	73	0.000679, 0.000789, 0.000916, 0.00106, 0.00124
	Hispanic	Men	74	0.000758, 0.000881, 0.00102, 0.00119, 0.00138
	Hispanic	Men	75	0.000847, 0.000985, 0.00114, 0.00133, 0.00154
	Hispanic	Men	76	0.000947, 0.0011, 0.00128, 0.00149, 0.00173
	Hispanic	Men	77	0.00106, 0.00123, 0.00144, 0.00167, 0.00195
	Hispanic	Men	78	0.00118, 0.00138, 0.00161, 0.00189, 0.00221
	Hispanic	Men	79	0.00131, 0.00154, 0.00181, 0.00213, 0.00251
	Hispanic	Men	80	0.00144, 0.00171, 0.00203, 0.0024, 0.00284
	Hispanic	Men	81	0.00158, 0.00189, 0.00225, 0.00269, 0.00321
	Hispanic	Men	82	0.00172, 0.00207, 0.00249, 0.003, 0.00362

Variable	Race/ethnicity	Sex	Age	Distribution
	Hispanic	Men	83	0.00185, 0.00225, 0.00273, 0.00332, 0.00404
	Hispanic	Men	84	0.00197, 0.00242, 0.00297, 0.00363, 0.00446
	Hispanic	Women	30	0.00000402, 0.00000624, 0.00000962, 0.0000148, 0.000023
	Hispanic	Women	31	0.00000479, 0.00000721, 0.0000108, 0.0000162, 0.0000244
	Hispanic	Women	32	0.00000563, 0.00000831, 0.0000122, 0.000018, 0.0000265
	Hispanic	Women	33	0.00000656, 0.00000955, 0.0000138, 0.0000201, 0.0000292
	Hispanic	Women	34	0.0000076, 0.0000109, 0.0000157, 0.0000225, 0.0000323
	Hispanic	Women	35	0.00000882, 0.0000125, 0.0000177, 0.000025, 0.0000354
	Hispanic	Women	36	0.0000102, 0.0000143, 0.0000198, 0.0000275, 0.0000383
	Hispanic	Women	37	0.0000119, 0.0000162, 0.0000221, 0.0000301, 0.0000411
	Hispanic	Women	38	0.0000136, 0.0000183, 0.0000244, 0.0000327, 0.0000439
	Hispanic	Women	39	0.0000154, 0.0000204, 0.0000269, 0.0000355, 0.0000469
	Hispanic	Women	40	0.0000174, 0.0000226, 0.0000294, 0.0000383, 0.0000499
	Hispanic	Women	41	0.0000194, 0.000025, 0.0000321, 0.0000412, 0.0000531
	Hispanic	Women	42	0.0000215, 0.0000275, 0.0000349, 0.0000444, 0.0000567
	Hispanic	Women	43	0.0000237, 0.0000301, 0.0000381, 0.0000481, 0.000061
	Hispanic	Women	44	0.0000262, 0.000033, 0.0000416, 0.0000523, 0.000066
	Hispanic	Women	45	0.0000293, 0.0000366, 0.0000455, 0.0000567, 0.0000707
	Hispanic	Women	46	0.0000334, 0.0000409, 0.00005, 0.0000611, 0.0000748
	Hispanic	Women	47	0.0000383, 0.0000459, 0.000055, 0.0000659, 0.000079
	Hispanic	Women	48	0.0000433, 0.0000513, 0.0000606, 0.0000717, 0.0000849
	Hispanic	Women	49	0.000048, 0.0000568, 0.000067, 0.0000791, 0.0000935
	Hispanic	Women	50	0.0000524, 0.0000624, 0.0000742, 0.0000882, 0.000105
	Hispanic	Women	51	0.0000568, 0.0000684, 0.0000823, 0.000099, 0.000119
	Hispanic	Women	52	0.0000613, 0.0000749, 0.0000912, 0.000111, 0.000136
	Hispanic	Women	53	0.0000662, 0.0000818, 0.000101, 0.000124, 0.000154
	Hispanic	Women	54	0.0000717, 0.0000894, 0.000111, 0.000138, 0.000173
	Hispanic	Women	55	0.0000782, 0.0000979, 0.000122, 0.000153, 0.000191
	Hispanic	Women	56	0.0000861, 0.000107, 0.000134, 0.000167, 0.000208
	Hispanic	Women	57	0.0000949, 0.000118, 0.000146, 0.000181, 0.000226
	Hispanic	Women	58	0.000104, 0.000129, 0.00016, 0.000197, 0.000244
	Hispanic	Women	59	0.000115, 0.000141, 0.000174, 0.000215, 0.000265
	Hispanic	Women	60	0.000125, 0.000154, 0.00019, 0.000233, 0.000287
	Hispanic	Women	61	0.000137, 0.000168, 0.000206, 0.000252, 0.00031
	Hispanic	Women	62	0.000149, 0.000182, 0.000223, 0.000272, 0.000333
	Hispanic	Women	63	0.000161, 0.000197, 0.00024, 0.000293, 0.000358
	Hispanic	Women	64	0.000173, 0.000212, 0.000259, 0.000316, 0.000386
	Hispanic	Women	65	0.000187, 0.000229, 0.00028, 0.000342, 0.000419
	Hispanic	Women	66	0.000202, 0.000249, 0.000304, 0.000373, 0.000458
	Hispanic	Women	67	0.000221, 0.000272, 0.000334, 0.000411, 0.000505
	Hispanic	Women	68	0.000245, 0.000302, 0.000371, 0.000456, 0.000562
	Hispanic	Women	69	0.000275, 0.000338, 0.000415, 0.00051, 0.000627
	Hispanic	Women	70	0.000314, 0.000384, 0.000468, 0.000571, 0.000698

Variable	Race/ethnicity	Sex	Age	Distribution
	Hispanic	Women	71	0.000364, 0.00044, 0.000531, 0.00064, 0.000774
	Hispanic	Women	72	0.000425, 0.000506, 0.000603, 0.000717, 0.000855
	Hispanic	Women	73	0.000498, 0.000585, 0.000686, 0.000805, 0.000946
	Hispanic	Women	74	0.00058, 0.000674, 0.000782, 0.000907, 0.00105
	Hispanic	Women	75	0.000671, 0.000775, 0.000893, 0.00103, 0.00119
	Hispanic	Women	76	0.000771, 0.000888, 0.00102, 0.00118, 0.00135
	Hispanic	Women	77	0.000884, 0.00102, 0.00117, 0.00135, 0.00156
	Hispanic	Women	78	0.00102, 0.00117, 0.00135, 0.00155, 0.00179
	Hispanic	Women	79	0.00117, 0.00135, 0.00155, 0.00178, 0.00204
	Hispanic	Women	80	0.00134, 0.00154, 0.00177, 0.00203, 0.00234
	Hispanic	Women	81	0.00151, 0.00174, 0.00201, 0.00233, 0.00269
	Hispanic	Women	82	0.00165, 0.00194, 0.00227, 0.00266, 0.00312
	Hispanic	Women	83	0.00178, 0.00213, 0.00254, 0.00303, 0.00363
	Hispanic	Women	84	0.00189, 0.00231, 0.00281, 0.00342, 0.00418
	White	Men	30	0.00000556, 0.00000772, 0.0000107, 0.0000148, 0.0000205
	White	Men	31	0.00000657, 0.0000089, 0.000012, 0.0000163, 0.000022
	White	Men	32	0.00000769, 0.0000102, 0.0000136, 0.000018, 0.0000239
	White	Men	33	0.00000895, 0.0000117, 0.0000152, 0.0000198, 0.0000259
	White	Men	34	0.0000103, 0.0000133, 0.000017, 0.0000219, 0.0000282
	White	Men	35	0.0000117, 0.0000149, 0.000019, 0.0000242, 0.0000309
	White	Men	36	0.0000131, 0.0000166, 0.0000211, 0.0000267, 0.0000339
	White	Men	37	0.0000146, 0.0000184, 0.0000233, 0.0000293, 0.0000371
	White	Men	38	0.0000161, 0.0000203, 0.0000256, 0.0000321, 0.0000405
	White	Men	39	0.0000177, 0.0000223, 0.000028, 0.0000351, 0.0000441
	White	Men	40	0.0000195, 0.0000244, 0.0000305, 0.000038, 0.0000475
	White	Men	41	0.0000217, 0.0000268, 0.0000331, 0.0000408, 0.0000504
	White	Men	42	0.0000244, 0.0000297, 0.0000359, 0.0000435, 0.0000528
	White	Men	43	0.0000275, 0.0000328, 0.0000391, 0.0000466, 0.0000556
	White	Men	44	0.0000307, 0.0000363, 0.0000427, 0.0000503, 0.0000594
	White	Men	45	0.0000343, 0.0000402, 0.000047, 0.0000549, 0.0000643
	White	Men	46	0.0000385, 0.0000447, 0.0000519, 0.0000602, 0.00007
	White	Men	47	0.0000431, 0.0000499, 0.0000577, 0.0000667, 0.0000771
	White	Men	48	0.000048, 0.0000557, 0.0000644, 0.0000745, 0.0000864
	White	Men	49	0.0000531, 0.000062, 0.0000722, 0.0000842, 0.0000982
	White	Men	50	0.0000589, 0.0000692, 0.0000813, 0.0000954, 0.000112
	White	Men	51	0.000066, 0.0000778, 0.0000915, 0.000108, 0.000127
	White	Men	52	0.0000747, 0.0000877, 0.000103, 0.000121, 0.000142
	White	Men	53	0.0000845, 0.0000989, 0.000115, 0.000135, 0.000158
	White	Men	54	0.0000947, 0.000111, 0.000129, 0.00015, 0.000175
	White	Men	55	0.000105, 0.000123, 0.000143, 0.000167, 0.000195
	White	Men	56	0.000115, 0.000135, 0.000158, 0.000185, 0.000218
	White	Men	57	0.000125, 0.000148, 0.000174, 0.000206, 0.000243
	White	Men	58	0.000136, 0.000161, 0.000192, 0.000227, 0.000271

Variable	Race/ethnicity	Sex	Age	Distribution
	White	Men	59	0.000148, 0.000177, 0.00021, 0.000251, 0.000299
	White	Men	60	0.000163, 0.000194, 0.000231, 0.000274, 0.000327
	White	Men	61	0.000181, 0.000214, 0.000252, 0.000298, 0.000352
	White	Men	62	0.000202, 0.000236, 0.000275, 0.000321, 0.000375
	White	Men	63	0.000226, 0.00026, 0.000299, 0.000344, 0.000396
	White	Men	64	0.000253, 0.000287, 0.000325, 0.000369, 0.000419
	White	Men	65	0.000282, 0.000317, 0.000355, 0.000398, 0.000446
	White	Men	66	0.000313, 0.000349, 0.000389, 0.000433, 0.000483
	White	Men	67	0.000346, 0.000386, 0.00043, 0.000479, 0.000534
	White	Men	68	0.000384, 0.000429, 0.000479, 0.000535, 0.000599
	White	Men	69	0.000427, 0.00048, 0.000538, 0.000604, 0.000678
	White	Men	70	0.000479, 0.000539, 0.000607, 0.000684, 0.000771
	White	Men	71	0.000537, 0.000608, 0.000687, 0.000776, 0.000877
	White	Men	72	0.000603, 0.000684, 0.000776, 0.00088, 0.001
	White	Men	73	0.000675, 0.00077, 0.000877, 0.000999, 0.00114
	White	Men	74	0.000754, 0.000865, 0.000991, 0.00114, 0.0013
	White	Men	75	0.000843, 0.000973, 0.00112, 0.00129, 0.00149
	White	Men	76	0.000945, 0.0011, 0.00127, 0.00147, 0.00171
	White	Men	77	0.00106, 0.00124, 0.00145, 0.00169, 0.00197
	White	Men	78	0.00119, 0.00141, 0.00166, 0.00195, 0.0023
	White	Men	79	0.00133, 0.00159, 0.0019, 0.00226, 0.0027
	White	Men	80	0.00148, 0.0018, 0.00217, 0.00262, 0.00318
	White	Men	81	0.00165, 0.00203, 0.00247, 0.00302, 0.0037
	White	Men	82	0.00185, 0.00227, 0.0028, 0.00344, 0.00424
	White	Men	83	0.00204, 0.00253, 0.00313, 0.00388, 0.00482
	White	Men	84	0.00222, 0.00278, 0.00347, 0.00433, 0.00542
	White	Women	30	0.00000496, 0.00000704, 0.00000995, 0.0000141, 0.00002
	White	Women	31	0.00000584, 0.00000814, 0.0000113, 0.0000157, 0.000022
	White	Women	32	0.00000687, 0.00000942, 0.0000129, 0.0000176, 0.0000241
	White	Women	33	0.00000806, 0.0000109, 0.0000146, 0.0000196, 0.0000264
	White	Women	34	0.00000933, 0.0000124, 0.0000164, 0.0000218, 0.000029
	White	Women	35	0.0000107, 0.000014, 0.0000184, 0.0000241, 0.0000317
	White	Women	36	0.000012, 0.0000157, 0.0000204, 0.0000264, 0.0000344
	White	Women	37	0.0000135, 0.0000174, 0.0000224, 0.0000288, 0.0000372
	White	Women	38	0.000015, 0.0000192, 0.0000245, 0.0000312, 0.0000399
	White	Women	39	0.0000165, 0.000021, 0.0000265, 0.0000336, 0.0000426
	White	Women	40	0.0000183, 0.0000229, 0.0000287, 0.0000359, 0.000045
	White	Women	41	0.0000203, 0.000025, 0.0000308, 0.0000379, 0.0000468
	White	Women	42	0.0000227, 0.0000274, 0.0000331, 0.0000399, 0.0000482
	White	Women	43	0.0000253, 0.00003, 0.0000356, 0.0000421, 0.00005
	White	Women	44	0.0000277, 0.0000326, 0.0000384, 0.0000451, 0.0000531
	White	Women	45	0.0000302, 0.0000355, 0.0000416, 0.0000489, 0.0000575
	White	Women	46	0.0000329, 0.0000387, 0.0000454, 0.0000533, 0.0000628

Variable	Race/ethnicity	Sex	Age	Distribution
	White	Women	47	0.0000358, 0.0000423, 0.0000498, 0.0000587, 0.0000692
	White	Women	48	0.0000389, 0.0000462, 0.0000548, 0.000065, 0.0000772
	White	Women	49	0.0000422, 0.0000506, 0.0000605, 0.0000724, 0.0000868
	White	Women	50	0.000046, 0.0000556, 0.000067, 0.0000808, 0.0000977
	White	Women	51	0.0000507, 0.0000614, 0.0000742, 0.0000897, 0.000109
	White	Women	52	0.0000565, 0.0000681, 0.000082, 0.0000988, 0.000119
	White	Women	53	0.0000632, 0.0000756, 0.0000904, 0.000108, 0.000129
	White	Women	54	0.0000705, 0.0000836, 0.0000991, 0.000118, 0.00014
	White	Women	55	0.0000783, 0.0000922, 0.000108, 0.000127, 0.00015
	White	Women	56	0.0000868, 0.000101, 0.000118, 0.000137, 0.00016
	White	Women	57	0.0000961, 0.000111, 0.000128, 0.000148, 0.000171
	White	Women	58	0.000106, 0.000122, 0.00014, 0.00016, 0.000184
	White	Women	59	0.000118, 0.000134, 0.000153, 0.000174, 0.000199
	White	Women	60	0.00013, 0.000147, 0.000167, 0.00019, 0.000216
	White	Women	61	0.000143, 0.000162, 0.000183, 0.000208, 0.000236
	White	Women	62	0.000157, 0.000178, 0.000201, 0.000228, 0.000258
	White	Women	63	0.000172, 0.000195, 0.00022, 0.000249, 0.000282
	White	Women	64	0.000189, 0.000214, 0.000241, 0.000272, 0.000307
	White	Women	65	0.000209, 0.000235, 0.000265, 0.000298, 0.000335
	White	Women	66	0.000231, 0.00026, 0.000292, 0.000329, 0.00037
	White	Women	67	0.000257, 0.00029, 0.000326, 0.000367, 0.000413
	White	Women	68	0.000289, 0.000326, 0.000367, 0.000414, 0.000467
	White	Women	69	0.000328, 0.00037, 0.000417, 0.00047, 0.00053
	White	Women	70	0.000377, 0.000424, 0.000478, 0.000537, 0.000606
	White	Women	71	0.000433, 0.000488, 0.000549, 0.000618, 0.000696
	White	Women	72	0.000498, 0.000561, 0.000633, 0.000713, 0.000804
	White	Women	73	0.00057, 0.000645, 0.000729, 0.000823, 0.00093
	White	Women	74	0.000655, 0.000741, 0.000838, 0.000948, 0.00107
	White	Women	75	0.000753, 0.000853, 0.000965, 0.00109, 0.00124
	White	Women	76	0.000868, 0.000983, 0.00111, 0.00126, 0.00142
	White	Women	77	0.000995, 0.00113, 0.00129, 0.00146, 0.00166
	White	Women	78	0.00114, 0.0013, 0.00149, 0.00171, 0.00195
	White	Women	79	0.0013, 0.0015, 0.00173, 0.002, 0.00231
	White	Women	80	0.00148, 0.00173, 0.00201, 0.00234, 0.00273
	White	Women	81	0.0017, 0.00199, 0.00233, 0.00273, 0.0032
	White	Women	82	0.00194, 0.00228, 0.00268, 0.00316, 0.00372
	White	Women	83	0.00218, 0.00259, 0.00306, 0.00362, 0.00429
	White	Women	84	0.00243, 0.0029, 0.00344, 0.00409, 0.00487
Stroke mortality rates for 2027 (0.01, 0.2, 0.5, 0.8. 0.99 percentiles of the empirical distribution produced during forecasting)				
	Black	Men	30	0.000015, 0.0000211, 0.0000296, 0.0000416, 0.0000587
	Black	Men	31	0.0000185, 0.0000254, 0.0000346, 0.0000472, 0.0000646
	Black	Men	32	0.0000224, 0.0000301, 0.0000403, 0.000054, 0.0000726
	Black	Men	33	0.0000263, 0.0000351, 0.0000466, 0.0000619, 0.0000826

Variable	Race/ethnicity	Sex	Age	Distribution
	Black	Men	34	0.0000304, 0.0000403, 0.0000534, 0.0000707, 0.0000939
	Black	Men	35	0.0000345, 0.0000457, 0.0000605, 0.0000799, 0.000106
	Black	Men	36	0.0000389, 0.0000514, 0.0000677, 0.0000891, 0.000118
	Black	Men	37	0.0000437, 0.0000573, 0.0000749, 0.0000979, 0.000128
	Black	Men	38	0.000049, 0.0000635, 0.0000821, 0.000106, 0.000138
	Black	Men	39	0.0000545, 0.0000699, 0.0000893, 0.000114, 0.000146
	Black	Men	40	0.0000605, 0.0000766, 0.0000967, 0.000122, 0.000155
	Black	Men	41	0.0000675, 0.0000841, 0.000105, 0.00013, 0.000162
	Black	Men	42	0.0000762, 0.0000931, 0.000114, 0.000138, 0.000169
	Black	Men	43	0.0000856, 0.000103, 0.000124, 0.000149, 0.000179
	Black	Men	44	0.0000937, 0.000113, 0.000136, 0.000163, 0.000197
	Black	Men	45	0.000102, 0.000124, 0.00015, 0.000182, 0.00022
	Black	Men	46	0.000113, 0.000137, 0.000166, 0.000202, 0.000246
	Black	Men	47	0.000125, 0.000153, 0.000185, 0.000225, 0.000274
	Black	Men	48	0.000139, 0.00017, 0.000208, 0.000254, 0.000311
	Black	Men	49	0.000154, 0.00019, 0.000234, 0.000288, 0.000356
	Black	Men	50	0.000175, 0.000216, 0.000265, 0.000326, 0.000402
	Black	Men	51	0.000206, 0.000249, 0.000302, 0.000365, 0.000442
	Black	Men	52	0.000243, 0.000289, 0.000342, 0.000406, 0.000482
	Black	Men	53	0.000282, 0.000331, 0.000386, 0.000451, 0.000528
	Black	Men	54	0.000319, 0.000371, 0.000432, 0.000502, 0.000584
	Black	Men	55	0.000354, 0.000411, 0.000477, 0.000554, 0.000644
	Black	Men	56	0.000387, 0.00045, 0.000523, 0.000607, 0.000706
	Black	Men	57	0.00042, 0.000489, 0.000568, 0.000659, 0.000767
	Black	Men	58	0.000456, 0.00053, 0.000613, 0.000711, 0.000824
	Black	Men	59	0.000495, 0.000572, 0.00066, 0.000762, 0.00088
	Black	Men	60	0.000528, 0.000612, 0.000707, 0.000817, 0.000946
	Black	Men	61	0.000556, 0.000648, 0.000753, 0.000877, 0.00102
	Black	Men	62	0.000587, 0.000685, 0.000799, 0.000931, 0.00109
	Black	Men	63	0.000627, 0.000728, 0.000843, 0.000976, 0.00113
	Black	Men	64	0.000674, 0.000774, 0.000888, 0.00102, 0.00117
	Black	Men	65	0.000718, 0.000821, 0.000938, 0.00107, 0.00123
	Black	Men	66	0.000759, 0.00087, 0.000996, 0.00114, 0.00131
	Black	Men	67	0.000809, 0.000929, 0.00107, 0.00122, 0.00141
	Black	Men	68	0.000875, 0.001, 0.00115, 0.00132, 0.00151
	Black	Men	69	0.000954, 0.00109, 0.00125, 0.00142, 0.00163
	Black	Men	70	0.00104, 0.00119, 0.00135, 0.00155, 0.00177
	Black	Men	71	0.00113, 0.00129, 0.00147, 0.00168, 0.00192
	Black	Men	72	0.00122, 0.0014, 0.00159, 0.00182, 0.00208
	Black	Men	73	0.00132, 0.00151, 0.00172, 0.00197, 0.00226
	Black	Men	74	0.00141, 0.00162, 0.00186, 0.00214, 0.00247
	Black	Men	75	0.0015, 0.00174, 0.00202, 0.00234, 0.00271
	Black	Men	76	0.00162, 0.00189, 0.00219, 0.00255, 0.00296

Variable	Race/ethnicity	Sex	Age	Distribution
	Black	Men	77	0.00176, 0.00205, 0.00239, 0.00278, 0.00324
	Black	Men	78	0.00192, 0.00224, 0.00261, 0.00304, 0.00354
	Black	Men	79	0.00208, 0.00243, 0.00284, 0.00333, 0.0039
	Black	Men	80	0.00223, 0.00263, 0.00309, 0.00364, 0.00428
	Black	Men	81	0.00239, 0.00283, 0.00334, 0.00395, 0.00468
	Black	Men	82	0.00254, 0.00302, 0.00359, 0.00426, 0.00506
	Black	Men	83	0.0027, 0.00321, 0.00383, 0.00456, 0.00543
	Black	Men	84	0.00284, 0.0034, 0.00406, 0.00485, 0.0058
	Black	Women	30	0.0000105, 0.0000159, 0.0000241, 0.0000364, 0.0000553
	Black	Women	31	0.0000123, 0.0000185, 0.0000277, 0.0000416, 0.0000628
	Black	Women	32	0.0000141, 0.0000212, 0.0000318, 0.0000477, 0.0000719
	Black	Women	33	0.0000159, 0.0000241, 0.0000363, 0.0000547, 0.0000828
	Black	Women	34	0.0000178, 0.0000271, 0.0000412, 0.0000626, 0.0000954
	Black	Women	35	0.0000198, 0.0000304, 0.0000464, 0.0000709, 0.000109
	Black	Women	36	0.0000222, 0.000034, 0.0000518, 0.000079, 0.000121
	Black	Women	37	0.0000253, 0.0000382, 0.0000575, 0.0000865, 0.000131
	Black	Women	38	0.000029, 0.000043, 0.0000634, 0.0000935, 0.000139
	Black	Women	39	0.000033, 0.000048, 0.0000695, 0.000101, 0.000147
	Black	Women	40	0.0000369, 0.000053, 0.0000759, 0.000109, 0.000156
	Black	Women	41	0.000041, 0.0000582, 0.0000825, 0.000117, 0.000166
	Black	Women	42	0.0000458, 0.0000641, 0.0000894, 0.000125, 0.000175
	Black	Women	43	0.0000514, 0.0000707, 0.000097, 0.000133, 0.000183
	Black	Women	44	0.0000574, 0.0000779, 0.000105, 0.000143, 0.000194
	Black	Women	45	0.0000638, 0.0000858, 0.000115, 0.000154, 0.000208
	Black	Women	46	0.0000717, 0.0000952, 0.000126, 0.000167, 0.000222
	Black	Women	47	0.0000819, 0.000107, 0.000139, 0.000181, 0.000236
	Black	Women	48	0.0000939, 0.00012, 0.000153, 0.000195, 0.00025
	Black	Women	49	0.000106, 0.000134, 0.000169, 0.000213, 0.000268
	Black	Women	50	0.000118, 0.000149, 0.000186, 0.000233, 0.000292
	Black	Women	51	0.000131, 0.000164, 0.000204, 0.000254, 0.000317
	Black	Women	52	0.000146, 0.000181, 0.000223, 0.000275, 0.000339
	Black	Women	53	0.000162, 0.000198, 0.000242, 0.000296, 0.000363
	Black	Women	54	0.000177, 0.000216, 0.000263, 0.000321, 0.000392
	Black	Women	55	0.00019, 0.000233, 0.000285, 0.000348, 0.000426
	Black	Women	56	0.000207, 0.000253, 0.000309, 0.000377, 0.000462
	Black	Women	57	0.000228, 0.000277, 0.000335, 0.000406, 0.000494
	Black	Women	58	0.000254, 0.000304, 0.000364, 0.000436, 0.000522
	Black	Women	59	0.000283, 0.000334, 0.000395, 0.000466, 0.000551
	Black	Women	60	0.00031, 0.000364, 0.000427, 0.0005, 0.000587
	Black	Women	61	0.000333, 0.000391, 0.000458, 0.000537, 0.00063
	Black	Women	62	0.000354, 0.000416, 0.000489, 0.000574, 0.000675
	Black	Women	63	0.000374, 0.00044, 0.000518, 0.000609, 0.000718
	Black	Women	64	0.000397, 0.000467, 0.000548, 0.000643, 0.000756

Variable	Race/ethnicity	Sex	Age	Distribution
	Black	Women	65	0.000425, 0.000497, 0.00058, 0.000678, 0.000793
	Black	Women	66	0.000458, 0.000533, 0.000619, 0.000718, 0.000836
	Black	Women	67	0.000499, 0.000576, 0.000666, 0.000768, 0.000888
	Black	Women	68	0.000549, 0.00063, 0.000723, 0.00083, 0.000954
	Black	Women	69	0.000609, 0.000696, 0.000794, 0.000906, 0.00104
	Black	Women	70	0.000678, 0.000772, 0.000878, 0.000999, 0.00114
	Black	Women	71	0.000755, 0.000859, 0.000976, 0.00111, 0.00126
	Black	Women	72	0.000839, 0.000956, 0.00109, 0.00124, 0.00141
	Black	Women	73	0.000935, 0.00107, 0.00121, 0.00138, 0.00158
	Black	Women	74	0.00105, 0.00119, 0.00136, 0.00154, 0.00176
	Black	Women	75	0.00117, 0.00133, 0.00152, 0.00173, 0.00197
	Black	Women	76	0.0013, 0.00149, 0.0017, 0.00195, 0.00223
	Black	Women	77	0.00143, 0.00166, 0.00191, 0.00221, 0.00255
	Black	Women	78	0.00157, 0.00184, 0.00215, 0.00251, 0.00294
	Black	Women	79	0.00173, 0.00205, 0.00242, 0.00286, 0.00338
	Black	Women	80	0.00191, 0.00227, 0.00271, 0.00322, 0.00384
	Black	Women	81	0.00211, 0.00252, 0.00301, 0.0036, 0.00432
	Black	Women	82	0.0023, 0.00277, 0.00333, 0.00401, 0.00482
	Black	Women	83	0.0025, 0.00302, 0.00366, 0.00442, 0.00536
	Black	Women	84	0.00268, 0.00327, 0.00398, 0.00484, 0.00591
	Hispanic	Men	30	0.00000687, 0.00000981, 0.000014, 0.0000198, 0.0000283
	Hispanic	Men	31	0.00000833, 0.0000115, 0.0000158, 0.0000218, 0.0000301
	Hispanic	Men	32	0.00000996, 0.0000134, 0.000018, 0.0000241, 0.0000324
	Hispanic	Men	33	0.0000117, 0.0000154, 0.0000204, 0.0000268, 0.0000354
	Hispanic	Men	34	0.0000134, 0.0000176, 0.000023, 0.00003, 0.0000392
	Hispanic	Men	35	0.0000152, 0.0000198, 0.0000258, 0.0000335, 0.0000436
	Hispanic	Men	36	0.0000172, 0.0000222, 0.0000287, 0.0000371, 0.000048
	Hispanic	Men	37	0.0000193, 0.0000248, 0.0000318, 0.0000408, 0.0000523
	Hispanic	Men	38	0.0000217, 0.0000276, 0.000035, 0.0000445, 0.0000566
	Hispanic	Men	39	0.0000241, 0.0000305, 0.0000384, 0.0000484, 0.000061
	Hispanic	Men	40	0.0000267, 0.0000335, 0.0000418, 0.0000523, 0.0000656
	Hispanic	Men	41	0.0000296, 0.0000367, 0.0000455, 0.0000562, 0.0000697
	Hispanic	Men	42	0.000033, 0.0000404, 0.0000493, 0.0000602, 0.0000736
	Hispanic	Men	43	0.0000367, 0.0000443, 0.0000535, 0.0000646, 0.0000781
	Hispanic	Men	44	0.0000402, 0.0000484, 0.0000582, 0.00007, 0.0000843
	Hispanic	Men	45	0.0000441, 0.000053, 0.0000637, 0.0000764, 0.0000918
	Hispanic	Men	46	0.0000488, 0.0000584, 0.0000699, 0.0000837, 0.0001
	Hispanic	Men	47	0.0000543, 0.0000649, 0.0000772, 0.000092, 0.00011
	Hispanic	Men	48	0.000061, 0.0000724, 0.0000858, 0.000102, 0.000121
	Hispanic	Men	49	0.0000691, 0.0000815, 0.0000959, 0.000113, 0.000133
	Hispanic	Men	50	0.000079, 0.0000923, 0.000108, 0.000125, 0.000147
	Hispanic	Men	51	0.000091, 0.000105, 0.000121, 0.000139, 0.000161
	Hispanic	Men	52	0.000104, 0.000119, 0.000136, 0.000155, 0.000178

Variable	Race/ethnicity	Sex	Age	Distribution
	Hispanic	Men	53	0.000115, 0.000133, 0.000152, 0.000175, 0.000201
	Hispanic	Men	54	0.000125, 0.000145, 0.000169, 0.000197, 0.00023
	Hispanic	Men	55	0.000134, 0.000158, 0.000187, 0.000222, 0.000263
	Hispanic	Men	56	0.000144, 0.000172, 0.000206, 0.000246, 0.000294
	Hispanic	Men	57	0.000157, 0.000188, 0.000225, 0.000269, 0.000322
	Hispanic	Men	58	0.000172, 0.000205, 0.000245, 0.000291, 0.000347
	Hispanic	Men	59	0.00019, 0.000225, 0.000265, 0.000313, 0.00037
	Hispanic	Men	60	0.000209, 0.000245, 0.000286, 0.000334, 0.000391
	Hispanic	Men	61	0.000229, 0.000266, 0.000308, 0.000357, 0.000414
	Hispanic	Men	62	0.000248, 0.000287, 0.00033, 0.000381, 0.000439
	Hispanic	Men	63	0.000267, 0.000307, 0.000353, 0.000406, 0.000468
	Hispanic	Men	64	0.000285, 0.000329, 0.000378, 0.000435, 0.000501
	Hispanic	Men	65	0.000306, 0.000353, 0.000406, 0.000468, 0.000539
	Hispanic	Men	66	0.000329, 0.00038, 0.000439, 0.000506, 0.000586
	Hispanic	Men	67	0.000355, 0.000412, 0.000478, 0.000554, 0.000643
	Hispanic	Men	68	0.000386, 0.00045, 0.000524, 0.000611, 0.000713
	Hispanic	Men	69	0.000424, 0.000496, 0.000579, 0.000677, 0.000792
	Hispanic	Men	70	0.000471, 0.000551, 0.000644, 0.000752, 0.000881
	Hispanic	Men	71	0.000526, 0.000615, 0.000717, 0.000837, 0.000978
	Hispanic	Men	72	0.000589, 0.000687, 0.0008, 0.000932, 0.00109
	Hispanic	Men	73	0.000658, 0.000768, 0.000894, 0.00104, 0.00121
	Hispanic	Men	74	0.000735, 0.000857, 0.000998, 0.00116, 0.00135
	Hispanic	Men	75	0.000821, 0.000958, 0.00112, 0.0013, 0.00152
	Hispanic	Men	76	0.000918, 0.00107, 0.00125, 0.00146, 0.0017
	Hispanic	Men	77	0.00103, 0.0012, 0.0014, 0.00164, 0.00192
	Hispanic	Men	78	0.00114, 0.00134, 0.00157, 0.00185, 0.00217
	Hispanic	Men	79	0.00127, 0.0015, 0.00177, 0.00208, 0.00246
	Hispanic	Men	80	0.0014, 0.00166, 0.00198, 0.00235, 0.00279
	Hispanic	Men	81	0.00153, 0.00184, 0.0022, 0.00263, 0.00316
	Hispanic	Men	82	0.00166, 0.00201, 0.00243, 0.00293, 0.00355
	Hispanic	Men	83	0.00179, 0.00218, 0.00266, 0.00324, 0.00396
	Hispanic	Men	84	0.00191, 0.00235, 0.00289, 0.00355, 0.00438
	Hispanic	Women	30	0.00000395, 0.00000617, 0.00000958, 0.0000149, 0.0000232
	Hispanic	Women	31	0.00000472, 0.00000715, 0.0000108, 0.0000162, 0.0000246
	Hispanic	Women	32	0.00000556, 0.00000825, 0.0000122, 0.000018, 0.0000267
	Hispanic	Women	33	0.00000648, 0.00000947, 0.0000138, 0.0000201, 0.0000294
	Hispanic	Women	34	0.00000751, 0.0000109, 0.0000156, 0.0000225, 0.0000325
	Hispanic	Women	35	0.00000871, 0.0000124, 0.0000176, 0.000025, 0.0000356
	Hispanic	Women	36	0.0000101, 0.0000141, 0.0000197, 0.0000275, 0.0000385
	Hispanic	Women	37	0.0000117, 0.000016, 0.0000219, 0.00003, 0.0000412
	Hispanic	Women	38	0.0000133, 0.000018, 0.0000242, 0.0000326, 0.000044
	Hispanic	Women	39	0.0000151, 0.0000201, 0.0000266, 0.0000353, 0.0000469
	Hispanic	Women	40	0.0000169, 0.0000222, 0.000029, 0.000038, 0.0000498

Variable	Race/ethnicity	Sex	Age	Distribution
	Hispanic	Women	41	0.0000189, 0.0000245, 0.0000316, 0.0000408, 0.0000528
	Hispanic	Women	42	0.000021, 0.0000269, 0.0000343, 0.0000438, 0.000056
	Hispanic	Women	43	0.0000231, 0.0000294, 0.0000373, 0.0000473, 0.0000601
	Hispanic	Women	44	0.0000255, 0.0000322, 0.0000407, 0.0000513, 0.0000649
	Hispanic	Women	45	0.0000285, 0.0000357, 0.0000445, 0.0000555, 0.0000694
	Hispanic	Women	46	0.0000325, 0.0000399, 0.0000488, 0.0000598, 0.0000733
	Hispanic	Women	47	0.0000372, 0.0000448, 0.0000537, 0.0000645, 0.0000775
	Hispanic	Women	48	0.0000422, 0.00005, 0.0000592, 0.0000702, 0.0000833
	Hispanic	Women	49	0.0000468, 0.0000554, 0.0000655, 0.0000775, 0.0000917
	Hispanic	Women	50	0.0000512, 0.000061, 0.0000727, 0.0000865, 0.000103
	Hispanic	Women	51	0.0000555, 0.000067, 0.0000807, 0.0000972, 0.000117
	Hispanic	Women	52	0.0000601, 0.0000734, 0.0000895, 0.000109, 0.000133
	Hispanic	Women	53	0.0000649, 0.0000803, 0.0000991, 0.000122, 0.000151
	Hispanic	Women	54	0.0000703, 0.0000878, 0.000109, 0.000136, 0.00017
	Hispanic	Women	55	0.0000768, 0.0000963, 0.00012, 0.00015, 0.000188
	Hispanic	Women	56	0.0000845, 0.000106, 0.000132, 0.000165, 0.000206
	Hispanic	Women	57	0.0000932, 0.000116, 0.000144, 0.000179, 0.000223
	Hispanic	Women	58	0.000103, 0.000127, 0.000157, 0.000195, 0.000242
	Hispanic	Women	59	0.000112, 0.000139, 0.000172, 0.000212, 0.000262
	Hispanic	Women	60	0.000123, 0.000152, 0.000187, 0.00023, 0.000284
	Hispanic	Women	61	0.000134, 0.000165, 0.000203, 0.000249, 0.000306
	Hispanic	Women	62	0.000145, 0.000179, 0.000219, 0.000268, 0.000329
	Hispanic	Women	63	0.000157, 0.000193, 0.000236, 0.000288, 0.000353
	Hispanic	Women	64	0.000169, 0.000207, 0.000254, 0.00031, 0.000381
	Hispanic	Women	65	0.000182, 0.000224, 0.000274, 0.000336, 0.000412
	Hispanic	Women	66	0.000197, 0.000243, 0.000298, 0.000366, 0.00045
	Hispanic	Women	67	0.000216, 0.000266, 0.000327, 0.000402, 0.000496
	Hispanic	Women	68	0.000238, 0.000294, 0.000362, 0.000447, 0.000551
	Hispanic	Women	69	0.000268, 0.00033, 0.000406, 0.000499, 0.000615
	Hispanic	Women	70	0.000305, 0.000374, 0.000457, 0.000559, 0.000685
	Hispanic	Women	71	0.000353, 0.000428, 0.000518, 0.000627, 0.00076
	Hispanic	Women	72	0.000413, 0.000493, 0.000589, 0.000702, 0.000839
	Hispanic	Women	73	0.000483, 0.000569, 0.000669, 0.000787, 0.000928
	Hispanic	Women	74	0.000563, 0.000656, 0.000763, 0.000887, 0.00103
	Hispanic	Women	75	0.000651, 0.000753, 0.00087, 0.00101, 0.00116
	Hispanic	Women	76	0.000747, 0.000863, 0.000996, 0.00115, 0.00133
	Hispanic	Women	77	0.000856, 0.00099, 0.00114, 0.00132, 0.00153
	Hispanic	Women	78	0.000984, 0.00114, 0.00131, 0.00152, 0.00175
	Hispanic	Women	79	0.00113, 0.00131, 0.00151, 0.00174, 0.00201
	Hispanic	Women	80	0.0013, 0.0015, 0.00173, 0.00199, 0.0023
	Hispanic	Women	81	0.00146, 0.00169, 0.00196, 0.00228, 0.00264
	Hispanic	Women	82	0.0016, 0.00188, 0.00222, 0.0026, 0.00307
	Hispanic	Women	83	0.00172, 0.00207, 0.00248, 0.00297, 0.00356

Variable	Race/ethnicity	Sex	Age	Distribution
	Hispanic	Women	84	0.00183, 0.00224, 0.00274, 0.00335, 0.0041
	White	Men	30	0.00000544, 0.00000762, 0.0000106, 0.0000148, 0.0000208
	White	Men	31	0.00000644, 0.0000088, 0.000012, 0.0000163, 0.0000223
	White	Men	32	0.00000756, 0.0000101, 0.0000135, 0.000018, 0.0000241
	White	Men	33	0.0000088, 0.0000116, 0.0000152, 0.0000199, 0.0000262
	White	Men	34	0.0000101, 0.0000131, 0.000017, 0.000022, 0.0000285
	White	Men	35	0.0000115, 0.0000147, 0.0000189, 0.0000243, 0.0000312
	White	Men	36	0.0000128, 0.0000164, 0.0000209, 0.0000267, 0.0000341
	White	Men	37	0.0000143, 0.0000182, 0.0000231, 0.0000293, 0.0000373
	White	Men	38	0.0000157, 0.00002, 0.0000253, 0.0000321, 0.0000407
	White	Men	39	0.0000173, 0.0000219, 0.0000276, 0.0000349, 0.0000442
	White	Men	40	0.000019, 0.0000239, 0.00003, 0.0000377, 0.0000475
	White	Men	41	0.0000211, 0.0000262, 0.0000325, 0.0000404, 0.0000502
	White	Men	42	0.0000237, 0.000029, 0.0000352, 0.0000429, 0.0000523
	White	Men	43	0.0000267, 0.000032, 0.0000383, 0.0000458, 0.0000548
	White	Men	44	0.0000298, 0.0000353, 0.0000418, 0.0000494, 0.0000585
	White	Men	45	0.0000333, 0.0000391, 0.0000459, 0.0000538, 0.0000632
	White	Men	46	0.0000373, 0.0000435, 0.0000507, 0.000059, 0.0000688
	White	Men	47	0.0000418, 0.0000486, 0.0000563, 0.0000652, 0.0000757
	White	Men	48	0.0000466, 0.0000542, 0.0000629, 0.000073, 0.0000848
	White	Men	49	0.0000516, 0.0000604, 0.0000706, 0.0000824, 0.0000965
	White	Men	50	0.0000572, 0.0000675, 0.0000795, 0.0000935, 0.00011
	White	Men	51	0.0000642, 0.0000759, 0.0000896, 0.000106, 0.000125
	White	Men	52	0.0000727, 0.0000858, 0.000101, 0.000119, 0.00014
	White	Men	53	0.0000824, 0.0000967, 0.000113, 0.000133, 0.000156
	White	Men	54	0.0000924, 0.000108, 0.000127, 0.000148, 0.000173
	White	Men	55	0.000102, 0.00012, 0.000141, 0.000165, 0.000193
	White	Men	56	0.000112, 0.000132, 0.000155, 0.000183, 0.000215
	White	Men	57	0.000122, 0.000145, 0.000171, 0.000203, 0.000241
	White	Men	58	0.000132, 0.000158, 0.000188, 0.000225, 0.000268
	White	Men	59	0.000145, 0.000173, 0.000207, 0.000247, 0.000296
	White	Men	60	0.000159, 0.00019, 0.000227, 0.000271, 0.000324
	White	Men	61	0.000176, 0.000209, 0.000248, 0.000294, 0.000349
	White	Men	62	0.000197, 0.000231, 0.00027, 0.000316, 0.000371
	White	Men	63	0.00022, 0.000254, 0.000294, 0.000339, 0.000392
	White	Men	64	0.000246, 0.00028, 0.000319, 0.000363, 0.000413
	White	Men	65	0.000274, 0.000309, 0.000347, 0.000391, 0.00044
	White	Men	66	0.000304, 0.00034, 0.00038, 0.000425, 0.000476
	White	Men	67	0.000336, 0.000376, 0.00042, 0.00047, 0.000525
	White	Men	68	0.000372, 0.000418, 0.000468, 0.000525, 0.000589
	White	Men	69	0.000414, 0.000467, 0.000526, 0.000592, 0.000667
	White	Men	70	0.000464, 0.000525, 0.000593, 0.00067, 0.000758
	White	Men	71	0.000521, 0.000591, 0.00067, 0.00076, 0.000863

Variable	Race/ethnicity	Sex	Age	Distribution
	White	Men	72	0.000584, 0.000666, 0.000758, 0.000863, 0.000983
	White	Men	73	0.000654, 0.000748, 0.000856, 0.000979, 0.00112
	White	Men	74	0.00073, 0.000841, 0.000967, 0.00111, 0.00128
	White	Men	75	0.000816, 0.000945, 0.00109, 0.00126, 0.00146
	White	Men	76	0.000915, 0.00107, 0.00124, 0.00144, 0.00168
	White	Men	77	0.00103, 0.00121, 0.00141, 0.00165, 0.00194
	White	Men	78	0.00115, 0.00137, 0.00162, 0.00191, 0.00226
	White	Men	79	0.00129, 0.00155, 0.00185, 0.00222, 0.00266
	White	Men	80	0.00144, 0.00175, 0.00212, 0.00257, 0.00313
	White	Men	81	0.0016, 0.00197, 0.00241, 0.00296, 0.00364
	White	Men	82	0.00179, 0.00221, 0.00273, 0.00337, 0.00418
	White	Men	83	0.00197, 0.00246, 0.00306, 0.0038, 0.00474
	White	Men	84	0.00215, 0.0027, 0.00338, 0.00424, 0.00533
	White	Women	30	0.00000486, 0.00000695, 0.00000991, 0.0000141, 0.0000202
	White	Women	31	0.00000574, 0.00000806, 0.0000113, 0.0000158, 0.0000222
	White	Women	32	0.00000677, 0.00000933, 0.0000128, 0.0000176, 0.0000243
	White	Women	33	0.00000794, 0.0000108, 0.0000145, 0.0000197, 0.0000266
	White	Women	34	0.00000919, 0.0000123, 0.0000164, 0.0000218, 0.0000292
	White	Women	35	0.0000105, 0.0000139, 0.0000183, 0.0000241, 0.0000319
	White	Women	36	0.0000118, 0.0000155, 0.0000203, 0.0000265, 0.0000347
	White	Women	37	0.0000132, 0.0000172, 0.0000222, 0.0000288, 0.0000374
	White	Women	38	0.0000147, 0.0000189, 0.0000242, 0.0000311, 0.0000401
	White	Women	39	0.0000161, 0.0000206, 0.0000262, 0.0000334, 0.0000427
	White	Women	40	0.0000178, 0.0000224, 0.0000283, 0.0000356, 0.0000449
	White	Women	41	0.0000197, 0.0000245, 0.0000303, 0.0000375, 0.0000466
	White	Women	42	0.0000221, 0.0000268, 0.0000325, 0.0000393, 0.0000477
	White	Women	43	0.0000246, 0.0000293, 0.0000348, 0.0000414, 0.0000494
	White	Women	44	0.000027, 0.0000318, 0.0000376, 0.0000443, 0.0000523
	White	Women	45	0.0000293, 0.0000346, 0.0000407, 0.0000479, 0.0000565
	White	Women	46	0.000032, 0.0000377, 0.0000444, 0.0000523, 0.0000616
	White	Women	47	0.0000348, 0.0000412, 0.0000486, 0.0000574, 0.0000679
	White	Women	48	0.0000379, 0.0000451, 0.0000536, 0.0000636, 0.0000757
	White	Women	49	0.0000412, 0.0000494, 0.0000592, 0.0000709, 0.0000852
	White	Women	50	0.0000449, 0.0000543, 0.0000656, 0.0000792, 0.0000959
	White	Women	51	0.0000495, 0.0000601, 0.0000727, 0.0000881, 0.000107
	White	Women	52	0.0000553, 0.0000668, 0.0000805, 0.0000971, 0.000117
	White	Women	53	0.0000619, 0.0000742, 0.0000888, 0.000106, 0.000127
	White	Women	54	0.0000691, 0.0000822, 0.0000975, 0.000116, 0.000138
	White	Women	55	0.0000768, 0.0000906, 0.000107, 0.000125, 0.000148
	White	Women	56	0.0000852, 0.0000995, 0.000116, 0.000135, 0.000158
	White	Women	57	0.0000942, 0.000109, 0.000126, 0.000146, 0.000169
	White	Women	58	0.000104, 0.00012, 0.000138, 0.000158, 0.000182
	White	Women	59	0.000115, 0.000132, 0.00015, 0.000172, 0.000197

Variable	Race/ethnicity	Sex	Age	Distribution
	White	Women	60	0.000127, 0.000145, 0.000165, 0.000188, 0.000214
	White	Women	61	0.000139, 0.000159, 0.000181, 0.000205, 0.000234
	White	Women	62	0.000153, 0.000174, 0.000198, 0.000224, 0.000255
	White	Women	63	0.000168, 0.00019, 0.000216, 0.000245, 0.000278
	White	Women	64	0.000184, 0.000209, 0.000236, 0.000267, 0.000303
	White	Women	65	0.000203, 0.00023, 0.000259, 0.000292, 0.00033
	White	Women	66	0.000225, 0.000254, 0.000286, 0.000322, 0.000364
	White	Women	67	0.00025, 0.000282, 0.000319, 0.00036, 0.000407
	White	Women	68	0.00028, 0.000317, 0.000358, 0.000405, 0.000459
	White	Women	69	0.000318, 0.00036, 0.000407, 0.000461, 0.000522
	White	Women	70	0.000365, 0.000413, 0.000466, 0.000527, 0.000596
	White	Women	71	0.00042, 0.000475, 0.000536, 0.000606, 0.000685
	White	Women	72	0.000482, 0.000546, 0.000617, 0.000698, 0.000791
	White	Women	73	0.000553, 0.000627, 0.000711, 0.000806, 0.000914
	White	Women	74	0.000634, 0.00072, 0.000818, 0.000928, 0.00105
	White	Women	75	0.00073, 0.000829, 0.000941, 0.00107, 0.00121
	White	Women	76	0.00084, 0.000955, 0.00108, 0.00123, 0.0014
	White	Women	77	0.000964, 0.0011, 0.00125, 0.00143, 0.00163
	White	Women	78	0.0011, 0.00127, 0.00145, 0.00167, 0.00192
	White	Women	79	0.00126, 0.00146, 0.00169, 0.00196, 0.00227
	White	Women	80	0.00144, 0.00168, 0.00196, 0.00229, 0.00268
	White	Women	81	0.00165, 0.00194, 0.00227, 0.00267, 0.00314
	White	Women	82	0.00187, 0.00222, 0.00262, 0.00309, 0.00366
	White	Women	83	0.00211, 0.00251, 0.00298, 0.00354, 0.00421
	White	Women	84	0.00235, 0.00281, 0.00336, 0.004, 0.00479
Stroke mortality rates for 2028 (0.01, 0.2, 0.5, 0.8. 0.99 percentiles of the empirical distribution produced during forecasting)				
	Black	Men	30	0.0000147, 0.0000208, 0.0000295, 0.0000418, 0.0000594
	Black	Men	31	0.0000182, 0.0000251, 0.0000345, 0.0000474, 0.0000653
	Black	Men	32	0.000022, 0.0000298, 0.0000402, 0.0000542, 0.0000733
	Black	Men	33	0.0000259, 0.0000348, 0.0000465, 0.0000621, 0.0000833
	Black	Men	34	0.0000299, 0.0000399, 0.0000532, 0.0000709, 0.0000946
	Black	Men	35	0.000034, 0.0000453, 0.0000602, 0.0000801, 0.000107
	Black	Men	36	0.0000382, 0.0000508, 0.0000673, 0.0000892, 0.000118
	Black	Men	37	0.0000429, 0.0000565, 0.0000744, 0.0000978, 0.000129
	Black	Men	38	0.0000479, 0.0000625, 0.0000813, 0.000106, 0.000138
	Black	Men	39	0.0000532, 0.0000686, 0.0000883, 0.000114, 0.000147
	Black	Men	40	0.0000589, 0.0000751, 0.0000954, 0.000121, 0.000155
	Black	Men	41	0.0000657, 0.0000823, 0.000103, 0.000129, 0.000161
	Black	Men	42	0.0000742, 0.000091, 0.000111, 0.000137, 0.000167
	Black	Men	43	0.0000833, 0.000101, 0.000121, 0.000146, 0.000177
	Black	Men	44	0.0000912, 0.00011, 0.000133, 0.00016, 0.000194
	Black	Men	45	0.0000992, 0.000121, 0.000147, 0.000178, 0.000217
	Black	Men	46	0.00011, 0.000134, 0.000163, 0.000198, 0.000241

Variable	Race/ethnicity	Sex	Age	Distribution
	Black	Men	47	0.000122, 0.000149, 0.000181, 0.00022, 0.000269
	Black	Men	48	0.000135, 0.000166, 0.000203, 0.000249, 0.000305
	Black	Men	49	0.00015, 0.000186, 0.000229, 0.000283, 0.000349
	Black	Men	50	0.000171, 0.000211, 0.00026, 0.00032, 0.000394
	Black	Men	51	0.000201, 0.000244, 0.000296, 0.000358, 0.000435
	Black	Men	52	0.000238, 0.000283, 0.000336, 0.000399, 0.000474
	Black	Men	53	0.000277, 0.000325, 0.00038, 0.000445, 0.000521
	Black	Men	54	0.000313, 0.000365, 0.000425, 0.000495, 0.000577
	Black	Men	55	0.000347, 0.000404, 0.00047, 0.000547, 0.000637
	Black	Men	56	0.00038, 0.000443, 0.000515, 0.000599, 0.000699
	Black	Men	57	0.000412, 0.000481, 0.00056, 0.000652, 0.00076
	Black	Men	58	0.000448, 0.000521, 0.000605, 0.000702, 0.000817
	Black	Men	59	0.000485, 0.000562, 0.00065, 0.000753, 0.000873
	Black	Men	60	0.000517, 0.0006, 0.000696, 0.000808, 0.000938
	Black	Men	61	0.000544, 0.000635, 0.000741, 0.000865, 0.00101
	Black	Men	62	0.000573, 0.000671, 0.000785, 0.000918, 0.00107
	Black	Men	63	0.000612, 0.000712, 0.000827, 0.000961, 0.00112
	Black	Men	64	0.000657, 0.000757, 0.00087, 0.001, 0.00115
	Black	Men	65	0.000699, 0.000802, 0.000918, 0.00105, 0.00121
	Black	Men	66	0.000738, 0.000849, 0.000974, 0.00112, 0.00129
	Black	Men	67	0.000786, 0.000906, 0.00104, 0.0012, 0.00138
	Black	Men	68	0.000849, 0.000978, 0.00112, 0.00129, 0.00149
	Black	Men	69	0.000925, 0.00106, 0.00122, 0.0014, 0.0016
	Black	Men	70	0.00101, 0.00116, 0.00132, 0.00151, 0.00174
	Black	Men	71	0.00109, 0.00125, 0.00144, 0.00164, 0.00188
	Black	Men	72	0.00119, 0.00136, 0.00156, 0.00178, 0.00204
	Black	Men	73	0.00128, 0.00147, 0.00168, 0.00193, 0.00222
	Black	Men	74	0.00136, 0.00158, 0.00182, 0.0021, 0.00242
	Black	Men	75	0.00146, 0.00169, 0.00197, 0.00228, 0.00265
	Black	Men	76	0.00157, 0.00183, 0.00214, 0.00249, 0.0029
	Black	Men	77	0.00171, 0.002, 0.00233, 0.00272, 0.00317
	Black	Men	78	0.00186, 0.00218, 0.00254, 0.00297, 0.00348
	Black	Men	79	0.00201, 0.00236, 0.00277, 0.00325, 0.00383
	Black	Men	80	0.00216, 0.00256, 0.00302, 0.00356, 0.00421
	Black	Men	81	0.00231, 0.00275, 0.00326, 0.00387, 0.00459
	Black	Men	82	0.00246, 0.00294, 0.0035, 0.00417, 0.00497
	Black	Men	83	0.00261, 0.00312, 0.00373, 0.00446, 0.00534
	Black	Men	84	0.00274, 0.0033, 0.00395, 0.00474, 0.0057
	Black	Women	30	0.0000103, 0.0000158, 0.000024, 0.0000366, 0.0000561
	Black	Women	31	0.0000121, 0.0000183, 0.0000277, 0.0000418, 0.0000635
	Black	Women	32	0.0000139, 0.000021, 0.0000318, 0.0000479, 0.0000726
	Black	Women	33	0.0000157, 0.0000239, 0.0000362, 0.0000549, 0.0000835
	Black	Women	34	0.0000176, 0.0000269, 0.0000411, 0.0000627, 0.0000961

Variable	Race/ethnicity	Sex	Age	Distribution
	Black	Women	35	0.0000196, 0.0000302, 0.0000463, 0.000071, 0.000109
	Black	Women	36	0.0000219, 0.0000337, 0.0000516, 0.0000791, 0.000122
	Black	Women	37	0.0000249, 0.0000379, 0.0000572, 0.0000865, 0.000131
	Black	Women	38	0.0000285, 0.0000425, 0.000063, 0.0000934, 0.000139
	Black	Women	39	0.0000323, 0.0000473, 0.0000689, 0.000101, 0.000147
	Black	Women	40	0.000036, 0.0000521, 0.0000751, 0.000108, 0.000157
	Black	Women	41	0.0000399, 0.0000571, 0.0000814, 0.000116, 0.000166
	Black	Women	42	0.0000445, 0.0000627, 0.0000881, 0.000124, 0.000175
	Black	Women	43	0.0000499, 0.0000691, 0.0000954, 0.000132, 0.000182
	Black	Women	44	0.0000556, 0.000076, 0.000104, 0.000141, 0.000193
	Black	Women	45	0.0000618, 0.0000837, 0.000113, 0.000153, 0.000207
	Black	Women	46	0.0000695, 0.0000929, 0.000124, 0.000165, 0.000221
	Black	Women	47	0.0000794, 0.000104, 0.000136, 0.000178, 0.000234
	Black	Women	48	0.0000911, 0.000117, 0.00015, 0.000193, 0.000248
	Black	Women	49	0.000103, 0.000131, 0.000166, 0.00021, 0.000266
	Black	Women	50	0.000115, 0.000145, 0.000183, 0.00023, 0.00029
	Black	Women	51	0.000128, 0.00016, 0.000201, 0.000251, 0.000315
	Black	Women	52	0.000143, 0.000177, 0.00022, 0.000272, 0.000338
	Black	Women	53	0.000158, 0.000195, 0.000239, 0.000294, 0.000361
	Black	Women	54	0.000173, 0.000212, 0.00026, 0.000318, 0.000391
	Black	Women	55	0.000186, 0.000229, 0.000281, 0.000346, 0.000425
	Black	Women	56	0.000202, 0.000249, 0.000305, 0.000375, 0.000461
	Black	Women	57	0.000223, 0.000272, 0.000331, 0.000403, 0.000492
	Black	Women	58	0.000248, 0.000299, 0.000359, 0.000432, 0.00052
	Black	Women	59	0.000277, 0.000329, 0.000389, 0.000462, 0.000548
	Black	Women	60	0.000303, 0.000357, 0.00042, 0.000495, 0.000583
	Black	Women	61	0.000326, 0.000384, 0.000451, 0.00053, 0.000625
	Black	Women	62	0.000345, 0.000408, 0.00048, 0.000566, 0.000669
	Black	Women	63	0.000365, 0.000431, 0.000509, 0.0006, 0.00071
	Black	Women	64	0.000387, 0.000456, 0.000537, 0.000633, 0.000746
	Black	Women	65	0.000413, 0.000485, 0.000569, 0.000666, 0.000782
	Black	Women	66	0.000445, 0.00052, 0.000605, 0.000705, 0.000823
	Black	Women	67	0.000484, 0.000562, 0.000651, 0.000754, 0.000874
	Black	Women	68	0.000533, 0.000614, 0.000707, 0.000815, 0.000939
	Black	Women	69	0.000591, 0.000678, 0.000776, 0.000889, 0.00102
	Black	Women	70	0.000658, 0.000752, 0.000858, 0.00098, 0.00112
	Black	Women	71	0.000732, 0.000836, 0.000954, 0.00109, 0.00124
	Black	Women	72	0.000813, 0.00093, 0.00106, 0.00121, 0.00139
	Black	Women	73	0.000906, 0.00104, 0.00118, 0.00135, 0.00155
	Black	Women	74	0.00101, 0.00116, 0.00132, 0.00151, 0.00173
	Black	Women	75	0.00113, 0.0013, 0.00148, 0.00169, 0.00193
	Black	Women	76	0.00126, 0.00145, 0.00166, 0.0019, 0.00219
	Black	Women	77	0.00139, 0.00161, 0.00186, 0.00216, 0.0025

Variable	Race/ethnicity	Sex	Age	Distribution
	Black	Women	78	0.00152, 0.00179, 0.0021, 0.00246, 0.00289
	Black	Women	79	0.00167, 0.00199, 0.00235, 0.00279, 0.00331
	Black	Women	80	0.00185, 0.00221, 0.00264, 0.00315, 0.00377
	Black	Women	81	0.00204, 0.00245, 0.00294, 0.00352, 0.00424
	Black	Women	82	0.00223, 0.00269, 0.00325, 0.00392, 0.00473
	Black	Women	83	0.00241, 0.00293, 0.00356, 0.00432, 0.00526
	Black	Women	84	0.00258, 0.00317, 0.00387, 0.00473, 0.0058
	Hispanic	Men	30	0.00000674, 0.00000969, 0.0000139, 0.0000199, 0.0000286
	Hispanic	Men	31	0.00000818, 0.0000114, 0.0000158, 0.0000219, 0.0000304
	Hispanic	Men	32	0.0000098, 0.0000133, 0.0000179, 0.0000242, 0.0000327
	Hispanic	Men	33	0.0000115, 0.0000153, 0.0000203, 0.0000269, 0.0000357
	Hispanic	Men	34	0.0000132, 0.0000174, 0.0000229, 0.00003, 0.0000396
	Hispanic	Men	35	0.000015, 0.0000196, 0.0000257, 0.0000335, 0.0000439
	Hispanic	Men	36	0.0000169, 0.000022, 0.0000286, 0.0000371, 0.0000484
	Hispanic	Men	37	0.000019, 0.0000245, 0.0000316, 0.0000407, 0.0000527
	Hispanic	Men	38	0.0000212, 0.0000272, 0.0000347, 0.0000444, 0.0000569
	Hispanic	Men	39	0.0000235, 0.0000299, 0.000038, 0.0000481, 0.0000612
	Hispanic	Men	40	0.000026, 0.0000328, 0.0000413, 0.0000519, 0.0000655
	Hispanic	Men	41	0.0000288, 0.0000359, 0.0000447, 0.0000557, 0.0000694
	Hispanic	Men	42	0.0000322, 0.0000395, 0.0000484, 0.0000593, 0.0000729
	Hispanic	Men	43	0.0000357, 0.0000433, 0.0000524, 0.0000635, 0.000077
	Hispanic	Men	44	0.0000391, 0.0000473, 0.000057, 0.0000687, 0.0000829
	Hispanic	Men	45	0.0000429, 0.0000517, 0.0000622, 0.0000749, 0.0000903
	Hispanic	Men	46	0.0000474, 0.000057, 0.0000683, 0.0000819, 0.0000984
	Hispanic	Men	47	0.0000529, 0.0000632, 0.0000755, 0.0000901, 0.000108
	Hispanic	Men	48	0.0000594, 0.0000706, 0.0000839, 0.0000996, 0.000118
	Hispanic	Men	49	0.0000673, 0.0000795, 0.0000938, 0.000111, 0.000131
	Hispanic	Men	50	0.0000771, 0.0000902, 0.000105, 0.000123, 0.000144
	Hispanic	Men	51	0.0000889, 0.000103, 0.000119, 0.000137, 0.000158
	Hispanic	Men	52	0.000102, 0.000117, 0.000133, 0.000153, 0.000175
	Hispanic	Men	53	0.000113, 0.00013, 0.00015, 0.000172, 0.000198
	Hispanic	Men	54	0.000122, 0.000143, 0.000167, 0.000195, 0.000228
	Hispanic	Men	55	0.000131, 0.000156, 0.000184, 0.000219, 0.00026
	Hispanic	Men	56	0.000141, 0.00017, 0.000203, 0.000243, 0.000291
	Hispanic	Men	57	0.000154, 0.000185, 0.000222, 0.000266, 0.000319
	Hispanic	Men	58	0.000169, 0.000202, 0.000241, 0.000288, 0.000344
	Hispanic	Men	59	0.000186, 0.000221, 0.000261, 0.000309, 0.000366
	Hispanic	Men	60	0.000205, 0.000241, 0.000282, 0.00033, 0.000388
	Hispanic	Men	61	0.000224, 0.000261, 0.000303, 0.000352, 0.00041
	Hispanic	Men	62	0.000243, 0.000281, 0.000325, 0.000375, 0.000435
	Hispanic	Men	63	0.00026, 0.000301, 0.000347, 0.0004, 0.000463
	Hispanic	Men	64	0.000278, 0.000321, 0.000371, 0.000428, 0.000494
	Hispanic	Men	65	0.000298, 0.000344, 0.000397, 0.000459, 0.000531

Variable	Race/ethnicity	Sex	Age	Distribution
	Hispanic	Men	66	0.00032, 0.000371, 0.000429, 0.000497, 0.000576
	Hispanic	Men	67	0.000345, 0.000401, 0.000467, 0.000543, 0.000632
	Hispanic	Men	68	0.000375, 0.000438, 0.000512, 0.000598, 0.0007
	Hispanic	Men	69	0.000412, 0.000483, 0.000566, 0.000663, 0.000778
	Hispanic	Men	70	0.000457, 0.000536, 0.000629, 0.000737, 0.000865
	Hispanic	Men	71	0.000511, 0.000599, 0.000701, 0.00082, 0.000961
	Hispanic	Men	72	0.000572, 0.000669, 0.000781, 0.000913, 0.00107
	Hispanic	Men	73	0.000639, 0.000747, 0.000872, 0.00102, 0.00119
	Hispanic	Men	74	0.000713, 0.000834, 0.000973, 0.00114, 0.00133
	Hispanic	Men	75	0.000797, 0.000932, 0.00109, 0.00127, 0.00149
	Hispanic	Men	76	0.00089, 0.00104, 0.00122, 0.00142, 0.00167
	Hispanic	Men	77	0.000995, 0.00117, 0.00137, 0.0016, 0.00188
	Hispanic	Men	78	0.00111, 0.00131, 0.00154, 0.00181, 0.00213
	Hispanic	Men	79	0.00123, 0.00146, 0.00172, 0.00204, 0.00241
	Hispanic	Men	80	0.00136, 0.00162, 0.00193, 0.0023, 0.00274
	Hispanic	Men	81	0.00149, 0.00179, 0.00215, 0.00258, 0.0031
	Hispanic	Men	82	0.00161, 0.00196, 0.00237, 0.00287, 0.00348
	Hispanic	Men	83	0.00173, 0.00212, 0.0026, 0.00317, 0.00389
	Hispanic	Men	84	0.00185, 0.00229, 0.00282, 0.00348, 0.0043
	Hispanic	Women	30	0.00000389, 0.0000061, 0.00000954, 0.0000149, 0.0000234
	Hispanic	Women	31	0.00000465, 0.00000708, 0.0000107, 0.0000163, 0.0000248
	Hispanic	Women	32	0.00000549, 0.00000818, 0.0000121, 0.000018, 0.0000269
	Hispanic	Women	33	0.0000064, 0.0000094, 0.0000138, 0.0000201, 0.0000296
	Hispanic	Women	34	0.00000742, 0.0000108, 0.0000156, 0.0000225, 0.0000327
	Hispanic	Women	35	0.00000859, 0.0000123, 0.0000175, 0.000025, 0.0000358
	Hispanic	Women	36	0.00000994, 0.000014, 0.0000196, 0.0000275, 0.0000387
	Hispanic	Women	37	0.0000115, 0.0000158, 0.0000218, 0.00003, 0.0000414
	Hispanic	Women	38	0.0000131, 0.0000177, 0.000024, 0.0000325, 0.0000441
	Hispanic	Women	39	0.0000148, 0.0000197, 0.0000263, 0.0000351, 0.0000469
	Hispanic	Women	40	0.0000165, 0.0000218, 0.0000286, 0.0000377, 0.0000497
	Hispanic	Women	41	0.0000184, 0.000024, 0.0000311, 0.0000403, 0.0000524
	Hispanic	Women	42	0.0000205, 0.0000263, 0.0000337, 0.0000431, 0.0000554
	Hispanic	Women	43	0.0000225, 0.0000287, 0.0000365, 0.0000465, 0.0000592
	Hispanic	Women	44	0.0000248, 0.0000315, 0.0000398, 0.0000503, 0.0000638
	Hispanic	Women	45	0.0000277, 0.0000348, 0.0000435, 0.0000544, 0.0000682
	Hispanic	Women	46	0.0000316, 0.0000389, 0.0000477, 0.0000585, 0.000072
	Hispanic	Women	47	0.0000362, 0.0000436, 0.0000525, 0.0000631, 0.000076
	Hispanic	Women	48	0.000041, 0.0000488, 0.0000579, 0.0000687, 0.0000817
	Hispanic	Women	49	0.0000456, 0.0000541, 0.0000641, 0.0000759, 0.00009
	Hispanic	Women	50	0.0000499, 0.0000597, 0.0000711, 0.0000849, 0.000101
	Hispanic	Women	51	0.0000543, 0.0000656, 0.0000791, 0.0000954, 0.000115
	Hispanic	Women	52	0.0000588, 0.000072, 0.0000879, 0.000107, 0.000131
	Hispanic	Women	53	0.0000636, 0.0000788, 0.0000974, 0.00012, 0.000149

Variable	Race/ethnicity	Sex	Age	Distribution
	Hispanic	Women	54	0.000069, 0.0000863, 0.000108, 0.000134, 0.000168
	Hispanic	Women	55	0.0000754, 0.0000947, 0.000118, 0.000148, 0.000186
	Hispanic	Women	56	0.0000831, 0.000104, 0.00013, 0.000162, 0.000203
	Hispanic	Women	57	0.0000916, 0.000114, 0.000142, 0.000177, 0.000221
	Hispanic	Women	58	0.000101, 0.000125, 0.000155, 0.000192, 0.000239
	Hispanic	Women	59	0.00011, 0.000137, 0.000169, 0.000209, 0.000259
	Hispanic	Women	60	0.000121, 0.000149, 0.000184, 0.000227, 0.000281
	Hispanic	Women	61	0.000131, 0.000162, 0.000199, 0.000245, 0.000303
	Hispanic	Women	62	0.000142, 0.000175, 0.000215, 0.000264, 0.000325
	Hispanic	Women	63	0.000154, 0.000189, 0.000231, 0.000284, 0.000349
	Hispanic	Women	64	0.000165, 0.000203, 0.000249, 0.000305, 0.000375
	Hispanic	Women	65	0.000178, 0.000219, 0.000268, 0.000329, 0.000405
	Hispanic	Women	66	0.000192, 0.000237, 0.000291, 0.000358, 0.000442
	Hispanic	Women	67	0.00021, 0.000259, 0.00032, 0.000394, 0.000487
	Hispanic	Women	68	0.000232, 0.000287, 0.000354, 0.000437, 0.000541
	Hispanic	Women	69	0.00026, 0.000322, 0.000396, 0.000489, 0.000604
	Hispanic	Women	70	0.000297, 0.000365, 0.000447, 0.000548, 0.000673
	Hispanic	Women	71	0.000343, 0.000417, 0.000506, 0.000614, 0.000746
	Hispanic	Women	72	0.000401, 0.00048, 0.000575, 0.000687, 0.000824
	Hispanic	Women	73	0.000469, 0.000554, 0.000653, 0.000771, 0.00091
	Hispanic	Women	74	0.000546, 0.000638, 0.000744, 0.000868, 0.00101
	Hispanic	Women	75	0.000631, 0.000732, 0.000849, 0.000984, 0.00114
	Hispanic	Women	76	0.000724, 0.000839, 0.000971, 0.00112, 0.0013
	Hispanic	Women	77	0.00083, 0.000962, 0.00111, 0.00129, 0.0015
	Hispanic	Women	78	0.000954, 0.00111, 0.00128, 0.00148, 0.00172
	Hispanic	Women	79	0.0011, 0.00127, 0.00147, 0.0017, 0.00197
	Hispanic	Women	80	0.00126, 0.00146, 0.00168, 0.00195, 0.00226
	Hispanic	Women	81	0.00141, 0.00165, 0.00191, 0.00223, 0.0026
	Hispanic	Women	82	0.00155, 0.00183, 0.00216, 0.00255, 0.00301
	Hispanic	Women	83	0.00166, 0.00201, 0.00241, 0.0029, 0.0035
	Hispanic	Women	84	0.00177, 0.00218, 0.00267, 0.00327, 0.00402
	White	Men	30	0.00000533, 0.00000752, 0.0000106, 0.0000149, 0.000021
	White	Men	31	0.00000632, 0.0000087, 0.0000119, 0.0000164, 0.0000225
	White	Men	32	0.00000743, 0.00001, 0.0000135, 0.0000181, 0.0000244
	White	Men	33	0.00000866, 0.0000115, 0.0000151, 0.00002, 0.0000264
	White	Men	34	0.00000996, 0.000013, 0.0000169, 0.000022, 0.0000287
	White	Men	35	0.0000113, 0.0000146, 0.0000188, 0.0000243, 0.0000314
	White	Men	36	0.0000126, 0.0000162, 0.0000208, 0.0000267, 0.0000344
	White	Men	37	0.000014, 0.0000179, 0.0000229, 0.0000293, 0.0000376
	White	Men	38	0.0000154, 0.0000197, 0.0000251, 0.000032, 0.0000409
	White	Men	39	0.0000168, 0.0000215, 0.0000273, 0.0000347, 0.0000443
	White	Men	40	0.0000185, 0.0000234, 0.0000296, 0.0000374, 0.0000474
	White	Men	41	0.0000205, 0.0000257, 0.000032, 0.0000399, 0.0000499

Variable	Race/ethnicity	Sex	Age	Distribution
	White	Men	42	0.0000231, 0.0000283, 0.0000346, 0.0000423, 0.0000518
	White	Men	43	0.000026, 0.0000312, 0.0000375, 0.000045, 0.0000541
	White	Men	44	0.000029, 0.0000344, 0.0000409, 0.0000485, 0.0000576
	White	Men	45	0.0000323, 0.0000381, 0.0000448, 0.0000527, 0.0000622
	White	Men	46	0.0000362, 0.0000423, 0.0000495, 0.0000578, 0.0000676
	White	Men	47	0.0000406, 0.0000473, 0.0000549, 0.0000639, 0.0000743
	White	Men	48	0.0000453, 0.0000528, 0.0000614, 0.0000714, 0.0000832
	White	Men	49	0.0000502, 0.0000588, 0.0000689, 0.0000807, 0.0000947
	White	Men	50	0.0000557, 0.0000658, 0.0000777, 0.0000917, 0.000108
	White	Men	51	0.0000625, 0.0000741, 0.0000877, 0.000104, 0.000123
	White	Men	52	0.0000709, 0.0000838, 0.0000989, 0.000117, 0.000138
	White	Men	53	0.0000805, 0.0000947, 0.000111, 0.000131, 0.000154
	White	Men	54	0.0000903, 0.000106, 0.000124, 0.000146, 0.000171
	White	Men	55	0.0001, 0.000118, 0.000138, 0.000162, 0.000191
	White	Men	56	0.00011, 0.00013, 0.000153, 0.00018, 0.000213
	White	Men	57	0.000119, 0.000142, 0.000169, 0.0002, 0.000238
	White	Men	58	0.000129, 0.000155, 0.000185, 0.000222, 0.000266
	White	Men	59	0.000141, 0.00017, 0.000204, 0.000244, 0.000293
	White	Men	60	0.000155, 0.000186, 0.000223, 0.000267, 0.00032
	White	Men	61	0.000172, 0.000205, 0.000244, 0.00029, 0.000345
	White	Men	62	0.000192, 0.000226, 0.000265, 0.000312, 0.000367
	White	Men	63	0.000214, 0.000249, 0.000288, 0.000334, 0.000387
	White	Men	64	0.000239, 0.000274, 0.000312, 0.000357, 0.000408
	White	Men	65	0.000266, 0.000301, 0.00034, 0.000383, 0.000433
	White	Men	66	0.000295, 0.000332, 0.000372, 0.000417, 0.000469
	White	Men	67	0.000326, 0.000366, 0.000411, 0.00046, 0.000517
	White	Men	68	0.000361, 0.000407, 0.000457, 0.000515, 0.00058
	White	Men	69	0.000402, 0.000454, 0.000513, 0.00058, 0.000656
	White	Men	70	0.00045, 0.000511, 0.000579, 0.000657, 0.000746
	White	Men	71	0.000505, 0.000575, 0.000655, 0.000745, 0.000849
	White	Men	72	0.000566, 0.000648, 0.00074, 0.000845, 0.000967
	White	Men	73	0.000633, 0.000728, 0.000836, 0.000959, 0.0011
	White	Men	74	0.000707, 0.000817, 0.000943, 0.00109, 0.00126
	White	Men	75	0.000791, 0.000919, 0.00107, 0.00124, 0.00144
	White	Men	76	0.000887, 0.00104, 0.00121, 0.00141, 0.00165
	White	Men	77	0.000996, 0.00117, 0.00138, 0.00162, 0.00191
	White	Men	78	0.00112, 0.00133, 0.00158, 0.00187, 0.00222
	White	Men	79	0.00125, 0.0015, 0.00181, 0.00217, 0.00261
	White	Men	80	0.00139, 0.0017, 0.00207, 0.00252, 0.00308
	White	Men	81	0.00155, 0.00191, 0.00236, 0.0029, 0.00359
	White	Men	82	0.00173, 0.00215, 0.00267, 0.00331, 0.00411
	White	Men	83	0.00191, 0.00239, 0.00298, 0.00373, 0.00467
	White	Men	84	0.00208, 0.00262, 0.0033, 0.00416, 0.00524

Variable	Race/ethnicity	Sex	Age	Distribution
	White	Women	30	0.00000476, 0.00000686, 0.00000987, 0.0000142, 0.0000205
	White	Women	31	0.00000564, 0.00000797, 0.0000112, 0.0000158, 0.0000224
	White	Women	32	0.00000666, 0.00000924, 0.0000128, 0.0000177, 0.0000245
	White	Women	33	0.00000782, 0.0000107, 0.0000145, 0.0000197, 0.0000269
	White	Women	34	0.00000906, 0.0000122, 0.0000163, 0.0000219, 0.0000294
	White	Women	35	0.0000103, 0.0000137, 0.0000182, 0.0000242, 0.0000322
	White	Women	36	0.0000116, 0.0000153, 0.0000201, 0.0000265, 0.0000349
	White	Women	37	0.000013, 0.0000169, 0.0000221, 0.0000288, 0.0000376
	White	Women	38	0.0000143, 0.0000186, 0.000024, 0.000031, 0.0000402
	White	Women	39	0.0000158, 0.0000202, 0.000026, 0.0000333, 0.0000427
	White	Women	40	0.0000173, 0.000022, 0.0000279, 0.0000353, 0.0000449
	White	Women	41	0.0000192, 0.000024, 0.0000298, 0.0000371, 0.0000463
	White	Women	42	0.0000215, 0.0000262, 0.0000319, 0.0000388, 0.0000473
	White	Women	43	0.0000239, 0.0000286, 0.0000342, 0.0000408, 0.0000487
	White	Women	44	0.0000262, 0.0000311, 0.0000368, 0.0000435, 0.0000515
	White	Women	45	0.0000285, 0.0000337, 0.0000398, 0.000047, 0.0000556
	White	Women	46	0.0000311, 0.0000367, 0.0000434, 0.0000512, 0.0000605
	White	Women	47	0.0000339, 0.0000402, 0.0000475, 0.0000562, 0.0000666
	White	Women	48	0.0000369, 0.000044, 0.0000523, 0.0000623, 0.0000742
	White	Women	49	0.0000401, 0.0000482, 0.0000579, 0.0000695, 0.0000835
	White	Women	50	0.0000438, 0.0000531, 0.0000642, 0.0000777, 0.0000941
	White	Women	51	0.0000484, 0.0000588, 0.0000713, 0.0000864, 0.000105
	White	Women	52	0.0000541, 0.0000655, 0.000079, 0.0000954, 0.000115
	White	Women	53	0.0000607, 0.0000728, 0.0000873, 0.000105, 0.000126
	White	Women	54	0.0000677, 0.0000807, 0.0000959, 0.000114, 0.000136
	White	Women	55	0.0000753, 0.000089, 0.000105, 0.000124, 0.000146
	White	Women	56	0.0000835, 0.0000978, 0.000114, 0.000134, 0.000157
	White	Women	57	0.0000924, 0.000107, 0.000125, 0.000144, 0.000168
	White	Women	58	0.000102, 0.000118, 0.000136, 0.000156, 0.000181
	White	Women	59	0.000113, 0.000129, 0.000148, 0.00017, 0.000195
	White	Women	60	0.000124, 0.000142, 0.000162, 0.000185, 0.000212
	White	Women	61	0.000136, 0.000156, 0.000178, 0.000203, 0.000232
	White	Women	62	0.000149, 0.00017, 0.000194, 0.000221, 0.000253
	White	Women	63	0.000163, 0.000186, 0.000212, 0.000241, 0.000275
	White	Women	64	0.000179, 0.000204, 0.000232, 0.000263, 0.000299
	White	Women	65	0.000198, 0.000224, 0.000254, 0.000287, 0.000325
	White	Women	66	0.000218, 0.000247, 0.00028, 0.000316, 0.000358
	White	Women	67	0.000242, 0.000275, 0.000311, 0.000353, 0.0004
	White	Women	68	0.000272, 0.000309, 0.00035, 0.000397, 0.000451
	White	Women	69	0.000309, 0.000351, 0.000398, 0.000452, 0.000513
	White	Women	70	0.000354, 0.000402, 0.000455, 0.000516, 0.000586
	White	Women	71	0.000407, 0.000462, 0.000524, 0.000593, 0.000673
	White	Women	72	0.000467, 0.000531, 0.000603, 0.000684, 0.000777

Variable	Race/ethnicity	Sex	Age	Distribution
	White	Women	73	0.000535, 0.00061, 0.000694, 0.000789, 0.000898
	White	Women	74	0.000614, 0.0007, 0.000797, 0.000908, 0.00104
	White	Women	75	0.000707, 0.000806, 0.000917, 0.00104, 0.00119
	White	Women	76	0.000813, 0.000928, 0.00106, 0.0012, 0.00137
	White	Women	77	0.000933, 0.00107, 0.00122, 0.0014, 0.0016
	White	Women	78	0.00107, 0.00123, 0.00142, 0.00163, 0.00188
	White	Women	79	0.00122, 0.00142, 0.00165, 0.00191, 0.00223
	White	Women	80	0.00139, 0.00163, 0.00191, 0.00224, 0.00263
	White	Women	81	0.00159, 0.00188, 0.00222, 0.00261, 0.00309
	White	Women	82	0.00181, 0.00215, 0.00255, 0.00302, 0.00359
	White	Women	83	0.00204, 0.00244, 0.00291, 0.00346, 0.00413
	White	Women	84	0.00228, 0.00273, 0.00327, 0.00392, 0.0047
Stroke mortality rates for 2029 (0.01, 0.2, 0.5, 0.8. 0.99 percentiles of the empirical distribution produced during forecasting)				
	Black	Men	30	0.0000144, 0.0000206, 0.0000294, 0.0000419, 0.0000601
	Black	Men	31	0.0000179, 0.0000248, 0.0000343, 0.0000475, 0.000066
	Black	Men	32	0.0000216, 0.0000295, 0.00004, 0.0000543, 0.000074
	Black	Men	33	0.0000255, 0.0000344, 0.0000463, 0.0000623, 0.0000839
	Black	Men	34	0.0000295, 0.0000396, 0.000053, 0.000071, 0.0000953
	Black	Men	35	0.0000335, 0.0000449, 0.00006, 0.0000802, 0.000107
	Black	Men	36	0.0000376, 0.0000502, 0.0000669, 0.0000892, 0.000119
	Black	Men	37	0.0000421, 0.0000558, 0.0000738, 0.0000977, 0.00013
	Black	Men	38	0.0000469, 0.0000616, 0.0000806, 0.000106, 0.000139
	Black	Men	39	0.000052, 0.0000675, 0.0000873, 0.000113, 0.000147
	Black	Men	40	0.0000574, 0.0000736, 0.0000941, 0.00012, 0.000154
	Black	Men	41	0.000064, 0.0000806, 0.000101, 0.000127, 0.000161
	Black	Men	42	0.0000723, 0.000089, 0.000109, 0.000135, 0.000166
	Black	Men	43	0.0000811, 0.0000983, 0.000119, 0.000144, 0.000174
	Black	Men	44	0.0000887, 0.000108, 0.00013, 0.000157, 0.000191
	Black	Men	45	0.0000965, 0.000118, 0.000143, 0.000175, 0.000213
	Black	Men	46	0.000107, 0.00013, 0.000159, 0.000194, 0.000237
	Black	Men	47	0.000119, 0.000145, 0.000177, 0.000216, 0.000264
	Black	Men	48	0.000132, 0.000162, 0.000198, 0.000244, 0.000299
	Black	Men	49	0.000147, 0.000181, 0.000224, 0.000277, 0.000343
	Black	Men	50	0.000167, 0.000207, 0.000255, 0.000314, 0.000387
	Black	Men	51	0.000197, 0.000239, 0.00029, 0.000352, 0.000428
	Black	Men	52	0.000234, 0.000278, 0.00033, 0.000392, 0.000467
	Black	Men	53	0.000271, 0.000319, 0.000373, 0.000438, 0.000514
	Black	Men	54	0.000307, 0.000358, 0.000418, 0.000488, 0.00057
	Black	Men	55	0.00034, 0.000397, 0.000463, 0.00054, 0.00063
	Black	Men	56	0.000372, 0.000435, 0.000508, 0.000592, 0.000692
	Black	Men	57	0.000404, 0.000473, 0.000552, 0.000644, 0.000753
	Black	Men	58	0.000439, 0.000512, 0.000596, 0.000694, 0.00081
	Black	Men	59	0.000475, 0.000552, 0.000641, 0.000744, 0.000865

Variable	Race/ethnicity	Sex	Age	Distribution
	Black	Men	60	0.000506, 0.00059, 0.000686, 0.000798, 0.000929
	Black	Men	61	0.000532, 0.000623, 0.00073, 0.000854, 0.001
	Black	Men	62	0.00056, 0.000658, 0.000771, 0.000905, 0.00106
	Black	Men	63	0.000597, 0.000697, 0.000812, 0.000946, 0.0011
	Black	Men	64	0.00064, 0.00074, 0.000853, 0.000985, 0.00114
	Black	Men	65	0.00068, 0.000783, 0.000899, 0.00103, 0.00119
	Black	Men	66	0.000718, 0.000828, 0.000953, 0.0011, 0.00126
	Black	Men	67	0.000764, 0.000883, 0.00102, 0.00118, 0.00136
	Black	Men	68	0.000825, 0.000952, 0.0011, 0.00127, 0.00146
	Black	Men	69	0.000898, 0.00103, 0.00119, 0.00137, 0.00158
	Black	Men	70	0.000978, 0.00112, 0.00129, 0.00148, 0.00171
	Black	Men	71	0.00106, 0.00122, 0.0014, 0.00161, 0.00185
	Black	Men	72	0.00115, 0.00132, 0.00152, 0.00174, 0.002
	Black	Men	73	0.00124, 0.00143, 0.00164, 0.00189, 0.00218
	Black	Men	74	0.00132, 0.00153, 0.00177, 0.00205, 0.00238
	Black	Men	75	0.00141, 0.00165, 0.00192, 0.00223, 0.0026
	Black	Men	76	0.00152, 0.00178, 0.00208, 0.00243, 0.00285
	Black	Men	77	0.00166, 0.00194, 0.00227, 0.00266, 0.00311
	Black	Men	78	0.0018, 0.00212, 0.00248, 0.00291, 0.00341
	Black	Men	79	0.00195, 0.0023, 0.00271, 0.00318, 0.00375
	Black	Men	80	0.0021, 0.00248, 0.00294, 0.00348, 0.00413
	Black	Men	81	0.00224, 0.00267, 0.00318, 0.00378, 0.00451
	Black	Men	82	0.00239, 0.00286, 0.00341, 0.00408, 0.00488
	Black	Men	83	0.00253, 0.00303, 0.00364, 0.00436, 0.00524
	Black	Men	84	0.00266, 0.0032, 0.00386, 0.00464, 0.0056
	Black	Women	30	0.0000101, 0.0000156, 0.000024, 0.0000368, 0.0000568
	Black	Women	31	0.0000119, 0.0000182, 0.0000276, 0.000042, 0.0000642
	Black	Women	32	0.0000137, 0.0000209, 0.0000317, 0.0000481, 0.0000733
	Black	Women	33	0.0000156, 0.0000238, 0.0000362, 0.0000551, 0.0000842
	Black	Women	34	0.0000174, 0.0000268, 0.000041, 0.0000629, 0.0000968
	Black	Women	35	0.0000193, 0.0000299, 0.0000462, 0.0000711, 0.00011
	Black	Women	36	0.0000217, 0.0000335, 0.0000515, 0.0000791, 0.000122
	Black	Women	37	0.0000246, 0.0000375, 0.0000569, 0.0000864, 0.000132
	Black	Women	38	0.000028, 0.0000419, 0.0000625, 0.0000933, 0.00014
	Black	Women	39	0.0000316, 0.0000466, 0.0000683, 0.0001, 0.000148
	Black	Women	40	0.0000351, 0.0000512, 0.0000743, 0.000108, 0.000157
	Black	Women	41	0.0000388, 0.000056, 0.0000804, 0.000115, 0.000166
	Black	Women	42	0.0000432, 0.0000614, 0.0000868, 0.000123, 0.000174
	Black	Women	43	0.0000485, 0.0000676, 0.0000938, 0.00013, 0.000182
	Black	Women	44	0.000054, 0.0000742, 0.000102, 0.000139, 0.000192
	Black	Women	45	0.00006, 0.0000817, 0.000111, 0.000151, 0.000205
	Black	Women	46	0.0000674, 0.0000906, 0.000121, 0.000163, 0.000219
	Black	Women	47	0.0000771, 0.000102, 0.000134, 0.000176, 0.000231

Variable	Race/ethnicity	Sex	Age	Distribution
	Black	Women	48	0.0000885, 0.000114, 0.000147, 0.00019, 0.000245
	Black	Women	49	0.0001, 0.000128, 0.000163, 0.000207, 0.000264
	Black	Women	50	0.000112, 0.000142, 0.00018, 0.000227, 0.000288
	Black	Women	51	0.000125, 0.000157, 0.000197, 0.000248, 0.000313
	Black	Women	52	0.000139, 0.000174, 0.000216, 0.000269, 0.000336
	Black	Women	53	0.000155, 0.000191, 0.000236, 0.000291, 0.00036
	Black	Women	54	0.000169, 0.000208, 0.000256, 0.000315, 0.000389
	Black	Women	55	0.000182, 0.000225, 0.000278, 0.000343, 0.000424
	Black	Women	56	0.000198, 0.000244, 0.000301, 0.000372, 0.000459
	Black	Women	57	0.000218, 0.000267, 0.000327, 0.0004, 0.00049
	Black	Women	58	0.000243, 0.000294, 0.000355, 0.000428, 0.000517
	Black	Women	59	0.000271, 0.000323, 0.000384, 0.000457, 0.000545
	Black	Women	60	0.000297, 0.000351, 0.000414, 0.000489, 0.000579
	Black	Women	61	0.000318, 0.000376, 0.000444, 0.000524, 0.000619
	Black	Women	62	0.000337, 0.0004, 0.000473, 0.000559, 0.000662
	Black	Women	63	0.000356, 0.000422, 0.0005, 0.000592, 0.000702
	Black	Women	64	0.000377, 0.000446, 0.000527, 0.000622, 0.000736
	Black	Women	65	0.000402, 0.000474, 0.000557, 0.000655, 0.000771
	Black	Women	66	0.000433, 0.000507, 0.000592, 0.000692, 0.00081
	Black	Women	67	0.000471, 0.000548, 0.000637, 0.00074, 0.000861
	Black	Women	68	0.000517, 0.000598, 0.000691, 0.000799, 0.000925
	Black	Women	69	0.000573, 0.00066, 0.000759, 0.000872, 0.001
	Black	Women	70	0.000638, 0.000732, 0.000839, 0.000961, 0.0011
	Black	Women	71	0.00071, 0.000814, 0.000932, 0.00107, 0.00122
	Black	Women	72	0.000789, 0.000905, 0.00104, 0.00119, 0.00136
	Black	Women	73	0.000879, 0.00101, 0.00116, 0.00133, 0.00152
	Black	Women	74	0.000983, 0.00113, 0.00129, 0.00148, 0.0017
	Black	Women	75	0.0011, 0.00126, 0.00144, 0.00165, 0.0019
	Black	Women	76	0.00122, 0.00141, 0.00162, 0.00186, 0.00214
	Black	Women	77	0.00134, 0.00156, 0.00182, 0.00211, 0.00246
	Black	Women	78	0.00147, 0.00174, 0.00204, 0.0024, 0.00283
	Black	Women	79	0.00162, 0.00193, 0.00229, 0.00273, 0.00325
	Black	Women	80	0.00179, 0.00214, 0.00257, 0.00308, 0.00369
	Black	Women	81	0.00197, 0.00238, 0.00286, 0.00345, 0.00416
	Black	Women	82	0.00215, 0.00261, 0.00316, 0.00383, 0.00465
	Black	Women	83	0.00233, 0.00284, 0.00347, 0.00423, 0.00516
	Black	Women	84	0.0025, 0.00307, 0.00377, 0.00463, 0.00569
	Hispanic	Men	30	0.0000066, 0.00000957, 0.0000138, 0.00002, 0.000029
	Hispanic	Men	31	0.00000804, 0.0000113, 0.0000157, 0.0000219, 0.0000307
	Hispanic	Men	32	0.00000964, 0.0000131, 0.0000178, 0.0000242, 0.000033
	Hispanic	Men	33	0.0000113, 0.0000152, 0.0000202, 0.000027, 0.0000361
	Hispanic	Men	34	0.000013, 0.0000173, 0.0000228, 0.0000301, 0.0000399
	Hispanic	Men	35	0.0000147, 0.0000194, 0.0000255, 0.0000336, 0.0000443

Variable	Race/ethnicity	Sex	Age	Distribution
	Hispanic	Men	36	0.0000166, 0.0000217, 0.0000284, 0.0000372, 0.0000487
	Hispanic	Men	37	0.0000186, 0.0000242, 0.0000314, 0.0000407, 0.000053
	Hispanic	Men	38	0.0000207, 0.0000268, 0.0000344, 0.0000443, 0.0000571
	Hispanic	Men	39	0.000023, 0.0000294, 0.0000375, 0.0000479, 0.0000613
	Hispanic	Men	40	0.0000253, 0.0000322, 0.0000407, 0.0000516, 0.0000654
	Hispanic	Men	41	0.0000281, 0.0000352, 0.000044, 0.0000551, 0.0000691
	Hispanic	Men	42	0.0000313, 0.0000386, 0.0000475, 0.0000585, 0.0000721
	Hispanic	Men	43	0.0000348, 0.0000423, 0.0000514, 0.0000624, 0.000076
	Hispanic	Men	44	0.0000381, 0.0000461, 0.0000558, 0.0000674, 0.0000816
	Hispanic	Men	45	0.0000417, 0.0000504, 0.0000608, 0.0000734, 0.0000887
	Hispanic	Men	46	0.0000461, 0.0000555, 0.0000667, 0.0000802, 0.0000966
	Hispanic	Men	47	0.0000514, 0.0000616, 0.0000737, 0.0000882, 0.000106
	Hispanic	Men	48	0.0000578, 0.0000689, 0.000082, 0.0000975, 0.000116
	Hispanic	Men	49	0.0000656, 0.0000776, 0.0000917, 0.000108, 0.000128
	Hispanic	Men	50	0.0000752, 0.0000881, 0.000103, 0.000121, 0.000141
	Hispanic	Men	51	0.0000869, 0.000101, 0.000116, 0.000134, 0.000156
	Hispanic	Men	52	0.0000995, 0.000114, 0.000131, 0.00015, 0.000173
	Hispanic	Men	53	0.000111, 0.000128, 0.000147, 0.000169, 0.000196
	Hispanic	Men	54	0.00012, 0.00014, 0.000164, 0.000192, 0.000225
	Hispanic	Men	55	0.000129, 0.000153, 0.000182, 0.000216, 0.000257
	Hispanic	Men	56	0.000139, 0.000167, 0.0002, 0.000239, 0.000287
	Hispanic	Men	57	0.000151, 0.000182, 0.000218, 0.000262, 0.000316
	Hispanic	Men	58	0.000166, 0.000199, 0.000238, 0.000284, 0.000341
	Hispanic	Men	59	0.000183, 0.000217, 0.000257, 0.000305, 0.000363
	Hispanic	Men	60	0.000201, 0.000236, 0.000278, 0.000326, 0.000384
	Hispanic	Men	61	0.000219, 0.000256, 0.000298, 0.000348, 0.000406
	Hispanic	Men	62	0.000237, 0.000275, 0.000319, 0.00037, 0.00043
	Hispanic	Men	63	0.000254, 0.000294, 0.00034, 0.000394, 0.000457
	Hispanic	Men	64	0.000271, 0.000314, 0.000363, 0.00042, 0.000487
	Hispanic	Men	65	0.00029, 0.000336, 0.000389, 0.000451, 0.000522
	Hispanic	Men	66	0.000311, 0.000361, 0.00042, 0.000487, 0.000566
	Hispanic	Men	67	0.000335, 0.000391, 0.000456, 0.000532, 0.000621
	Hispanic	Men	68	0.000364, 0.000427, 0.0005, 0.000586, 0.000688
	Hispanic	Men	69	0.0004, 0.000471, 0.000553, 0.00065, 0.000765
	Hispanic	Men	70	0.000444, 0.000522, 0.000614, 0.000722, 0.00085
	Hispanic	Men	71	0.000496, 0.000583, 0.000684, 0.000803, 0.000944
	Hispanic	Men	72	0.000555, 0.000651, 0.000763, 0.000894, 0.00105
	Hispanic	Men	73	0.00062, 0.000727, 0.000851, 0.000996, 0.00117
	Hispanic	Men	74	0.000692, 0.000811, 0.00095, 0.00111, 0.0013
	Hispanic	Men	75	0.000773, 0.000906, 0.00106, 0.00124, 0.00146
	Hispanic	Men	76	0.000864, 0.00101, 0.00119, 0.00139, 0.00163
	Hispanic	Men	77	0.000964, 0.00113, 0.00133, 0.00157, 0.00184
	Hispanic	Men	78	0.00107, 0.00127, 0.0015, 0.00177, 0.00209

Variable	Race/ethnicity	Sex	Age	Distribution
	Hispanic	Men	79	0.00119, 0.00142, 0.00168, 0.00199, 0.00237
	Hispanic	Men	80	0.00132, 0.00157, 0.00188, 0.00225, 0.00269
	Hispanic	Men	81	0.00144, 0.00174, 0.00209, 0.00252, 0.00304
	Hispanic	Men	82	0.00156, 0.0019, 0.00231, 0.00281, 0.00342
	Hispanic	Men	83	0.00168, 0.00206, 0.00253, 0.00311, 0.00382
	Hispanic	Men	84	0.00179, 0.00222, 0.00275, 0.0034, 0.00422
	Hispanic	Women	30	0.00000382, 0.00000604, 0.0000095, 0.0000149, 0.0000236
	Hispanic	Women	31	0.00000458, 0.00000701, 0.0000107, 0.0000163, 0.000025
	Hispanic	Women	32	0.00000542, 0.00000811, 0.0000121, 0.0000181, 0.000027
	Hispanic	Women	33	0.00000633, 0.00000933, 0.0000137, 0.0000202, 0.0000298
	Hispanic	Women	34	0.00000734, 0.0000107, 0.0000155, 0.0000225, 0.0000328
	Hispanic	Women	35	0.00000849, 0.0000122, 0.0000175, 0.000025, 0.0000359
	Hispanic	Women	36	0.0000098, 0.0000138, 0.0000195, 0.0000275, 0.0000388
	Hispanic	Women	37	0.0000113, 0.0000156, 0.0000216, 0.0000299, 0.0000415
	Hispanic	Women	38	0.0000128, 0.0000175, 0.0000238, 0.0000324, 0.0000442
	Hispanic	Women	39	0.0000144, 0.0000194, 0.000026, 0.0000349, 0.0000469
	Hispanic	Women	40	0.0000161, 0.0000214, 0.0000283, 0.0000373, 0.0000495
	Hispanic	Women	41	0.000018, 0.0000235, 0.0000306, 0.0000398, 0.000052
	Hispanic	Women	42	0.00002, 0.0000257, 0.0000331, 0.0000425, 0.0000548
	Hispanic	Women	43	0.000022, 0.0000281, 0.0000358, 0.0000456, 0.0000583
	Hispanic	Women	44	0.0000242, 0.0000307, 0.0000389, 0.0000493, 0.0000627
	Hispanic	Women	45	0.000027, 0.0000339, 0.0000425, 0.0000533, 0.0000669
	Hispanic	Women	46	0.0000307, 0.0000379, 0.0000466, 0.0000573, 0.0000706
	Hispanic	Women	47	0.0000352, 0.0000425, 0.0000512, 0.0000617, 0.0000745
	Hispanic	Women	48	0.00004, 0.0000476, 0.0000566, 0.0000673, 0.0000801
	Hispanic	Women	49	0.0000445, 0.0000528, 0.0000627, 0.0000744, 0.0000883
	Hispanic	Women	50	0.0000487, 0.0000583, 0.0000697, 0.0000832, 0.0000996
	Hispanic	Women	51	0.0000531, 0.0000642, 0.0000775, 0.0000936, 0.000113
	Hispanic	Women	52	0.0000576, 0.0000706, 0.0000863, 0.000105, 0.000129
	Hispanic	Women	53	0.0000624, 0.0000774, 0.0000958, 0.000119, 0.000147
	Hispanic	Women	54	0.0000677, 0.0000848, 0.000106, 0.000132, 0.000166
	Hispanic	Women	55	0.0000741, 0.0000931, 0.000117, 0.000146, 0.000184
	Hispanic	Women	56	0.0000816, 0.000102, 0.000128, 0.00016, 0.000201
	Hispanic	Women	57	0.00009, 0.000112, 0.00014, 0.000175, 0.000218
	Hispanic	Women	58	0.0000989, 0.000123, 0.000153, 0.00019, 0.000237
	Hispanic	Women	59	0.000108, 0.000135, 0.000167, 0.000207, 0.000257
	Hispanic	Women	60	0.000118, 0.000147, 0.000181, 0.000224, 0.000278
	Hispanic	Women	61	0.000129, 0.000159, 0.000196, 0.000242, 0.000299
	Hispanic	Women	62	0.000139, 0.000172, 0.000211, 0.00026, 0.000321
	Hispanic	Women	63	0.00015, 0.000185, 0.000227, 0.000279, 0.000344
	Hispanic	Women	64	0.000161, 0.000198, 0.000244, 0.0003, 0.000369
	Hispanic	Women	65	0.000173, 0.000214, 0.000263, 0.000323, 0.000398
	Hispanic	Women	66	0.000187, 0.000231, 0.000285, 0.000351, 0.000434

Variable	Race/ethnicity	Sex	Age	Distribution
	Hispanic	Women	67	0.000204, 0.000253, 0.000312, 0.000386, 0.000477
	Hispanic	Women	68	0.000226, 0.00028, 0.000346, 0.000428, 0.000531
	Hispanic	Women	69	0.000253, 0.000313, 0.000387, 0.000478, 0.000592
	Hispanic	Women	70	0.000289, 0.000355, 0.000436, 0.000536, 0.00066
	Hispanic	Women	71	0.000334, 0.000407, 0.000494, 0.000601, 0.000732
	Hispanic	Women	72	0.000389, 0.000468, 0.000561, 0.000673, 0.000808
	Hispanic	Women	73	0.000455, 0.000539, 0.000637, 0.000754, 0.000893
	Hispanic	Women	74	0.000529, 0.00062, 0.000726, 0.000849, 0.000994
	Hispanic	Women	75	0.000611, 0.000712, 0.000828, 0.000962, 0.00112
	Hispanic	Women	76	0.000702, 0.000816, 0.000947, 0.0011, 0.00128
	Hispanic	Women	77	0.000804, 0.000935, 0.00109, 0.00126, 0.00147
	Hispanic	Women	78	0.000924, 0.00107, 0.00125, 0.00145, 0.00169
	Hispanic	Women	79	0.00106, 0.00124, 0.00143, 0.00166, 0.00193
	Hispanic	Women	80	0.00122, 0.00141, 0.00164, 0.00191, 0.00221
	Hispanic	Women	81	0.00137, 0.0016, 0.00187, 0.00218, 0.00255
	Hispanic	Women	82	0.0015, 0.00178, 0.00211, 0.00249, 0.00296
	Hispanic	Women	83	0.00161, 0.00195, 0.00235, 0.00284, 0.00343
	Hispanic	Women	84	0.00171, 0.00211, 0.0026, 0.0032, 0.00394
	White	Men	30	0.00000522, 0.00000742, 0.0000105, 0.0000149, 0.0000213
	White	Men	31	0.0000062, 0.0000086, 0.0000119, 0.0000164, 0.0000228
	White	Men	32	0.00000731, 0.00000991, 0.0000134, 0.0000181, 0.0000246
	White	Men	33	0.00000853, 0.0000114, 0.0000151, 0.00002, 0.0000266
	White	Men	34	0.00000981, 0.0000129, 0.0000169, 0.0000221, 0.000029
	White	Men	35	0.0000111, 0.0000144, 0.0000187, 0.0000243, 0.0000317
	White	Men	36	0.0000124, 0.000016, 0.0000207, 0.0000268, 0.0000347
	White	Men	37	0.0000137, 0.0000177, 0.0000227, 0.0000293, 0.0000378
	White	Men	38	0.000015, 0.0000193, 0.0000248, 0.0000319, 0.0000411
	White	Men	39	0.0000164, 0.0000211, 0.000027, 0.0000346, 0.0000443
	White	Men	40	0.000018, 0.0000229, 0.0000292, 0.0000371, 0.0000473
	White	Men	41	0.00002, 0.0000251, 0.0000315, 0.0000395, 0.0000496
	White	Men	42	0.0000224, 0.0000276, 0.0000339, 0.0000417, 0.0000513
	White	Men	43	0.0000252, 0.0000305, 0.0000367, 0.0000442, 0.0000534
	White	Men	44	0.0000281, 0.0000336, 0.00004, 0.0000476, 0.0000567
	White	Men	45	0.0000313, 0.0000371, 0.0000438, 0.0000517, 0.0000611
	White	Men	46	0.0000351, 0.0000412, 0.0000483, 0.0000566, 0.0000664
	White	Men	47	0.0000394, 0.000046, 0.0000536, 0.0000625, 0.000073
	White	Men	48	0.000044, 0.0000514, 0.0000599, 0.0000699, 0.0000817
	White	Men	49	0.0000488, 0.0000574, 0.0000673, 0.0000791, 0.000093
	White	Men	50	0.0000542, 0.0000642, 0.000076, 0.0000899, 0.000107
	White	Men	51	0.0000609, 0.0000724, 0.0000859, 0.000102, 0.000121
	White	Men	52	0.0000692, 0.000082, 0.000097, 0.000115, 0.000136
	White	Men	53	0.0000786, 0.0000927, 0.000109, 0.000129, 0.000152
	White	Men	54	0.0000882, 0.000104, 0.000122, 0.000144, 0.000169

Variable	Race/ethnicity	Sex	Age	Distribution
	White	Men	55	0.0000978, 0.000115, 0.000136, 0.00016, 0.000189
	White	Men	56	0.000107, 0.000127, 0.00015, 0.000178, 0.000211
	White	Men	57	0.000117, 0.000139, 0.000166, 0.000198, 0.000236
	White	Men	58	0.000127, 0.000152, 0.000182, 0.000219, 0.000263
	White	Men	59	0.000138, 0.000166, 0.0002, 0.000241, 0.000291
	White	Men	60	0.000152, 0.000183, 0.000219, 0.000264, 0.000317
	White	Men	61	0.000168, 0.000201, 0.000239, 0.000286, 0.000341
	White	Men	62	0.000187, 0.000221, 0.00026, 0.000307, 0.000363
	White	Men	63	0.000209, 0.000243, 0.000282, 0.000328, 0.000382
	White	Men	64	0.000233, 0.000267, 0.000306, 0.00035, 0.000402
	White	Men	65	0.000259, 0.000294, 0.000332, 0.000376, 0.000427
	White	Men	66	0.000287, 0.000323, 0.000364, 0.000409, 0.000461
	White	Men	67	0.000317, 0.000357, 0.000401, 0.000452, 0.000509
	White	Men	68	0.00035, 0.000396, 0.000447, 0.000505, 0.00057
	White	Men	69	0.00039, 0.000442, 0.000502, 0.000569, 0.000646
	White	Men	70	0.000436, 0.000497, 0.000566, 0.000644, 0.000734
	White	Men	71	0.00049, 0.00056, 0.000639, 0.00073, 0.000835
	White	Men	72	0.000549, 0.00063, 0.000722, 0.000828, 0.00095
	White	Men	73	0.000614, 0.000708, 0.000816, 0.000939, 0.00108
	White	Men	74	0.000685, 0.000795, 0.00092, 0.00107, 0.00124
	White	Men	75	0.000766, 0.000894, 0.00104, 0.00121, 0.00141
	White	Men	76	0.000859, 0.00101, 0.00118, 0.00138, 0.00162
	White	Men	77	0.000965, 0.00114, 0.00134, 0.00159, 0.00187
	White	Men	78	0.00108, 0.00129, 0.00154, 0.00183, 0.00219
	White	Men	79	0.00121, 0.00146, 0.00176, 0.00213, 0.00257
	White	Men	80	0.00135, 0.00165, 0.00202, 0.00247, 0.00303
	White	Men	81	0.0015, 0.00186, 0.0023, 0.00285, 0.00353
	White	Men	82	0.00167, 0.00209, 0.0026, 0.00324, 0.00404
	White	Men	83	0.00185, 0.00232, 0.00291, 0.00365, 0.00459
	White	Men	84	0.00201, 0.00255, 0.00322, 0.00407, 0.00515
	White	Women	30	0.00000466, 0.00000678, 0.00000982, 0.0000142, 0.0000207
	White	Women	31	0.00000554, 0.00000789, 0.0000112, 0.0000159, 0.0000226
	White	Women	32	0.00000656, 0.00000916, 0.0000127, 0.0000177, 0.0000247
	White	Women	33	0.00000771, 0.0000106, 0.0000144, 0.0000197, 0.0000271
	White	Women	34	0.00000893, 0.0000121, 0.0000163, 0.0000219, 0.0000296
	White	Women	35	0.0000102, 0.0000136, 0.0000181, 0.0000242, 0.0000324
	White	Women	36	0.0000114, 0.0000152, 0.00002, 0.0000265, 0.0000351
	White	Women	37	0.0000127, 0.0000167, 0.0000219, 0.0000288, 0.0000378
	White	Women	38	0.000014, 0.0000183, 0.0000238, 0.000031, 0.0000404
	White	Women	39	0.0000154, 0.0000199, 0.0000257, 0.0000331, 0.0000428
	White	Women	40	0.0000169, 0.0000216, 0.0000275, 0.0000351, 0.0000448
	White	Women	41	0.0000187, 0.0000235, 0.0000294, 0.0000367, 0.0000461
	White	Women	42	0.0000209, 0.0000256, 0.0000313, 0.0000383, 0.0000469

Variable	Race/ethnicity	Sex	Age	Distribution
	White	Women	43	0.0000233, 0.000028, 0.0000335, 0.0000401, 0.0000481
	White	Women	44	0.0000255, 0.0000303, 0.000036, 0.0000427, 0.0000508
	White	Women	45	0.0000277, 0.0000329, 0.0000389, 0.0000461, 0.0000547
	White	Women	46	0.0000302, 0.0000358, 0.0000424, 0.0000501, 0.0000595
	White	Women	47	0.000033, 0.0000392, 0.0000464, 0.000055, 0.0000653
	White	Women	48	0.0000359, 0.0000429, 0.0000511, 0.000061, 0.0000728
	White	Women	49	0.0000391, 0.0000471, 0.0000566, 0.0000681, 0.000082
	White	Women	50	0.0000428, 0.0000519, 0.0000629, 0.0000762, 0.0000924
	White	Women	51	0.0000474, 0.0000576, 0.0000699, 0.0000848, 0.000103
	White	Women	52	0.000053, 0.0000642, 0.0000776, 0.0000938, 0.000114
	White	Women	53	0.0000595, 0.0000715, 0.0000858, 0.000103, 0.000124
	White	Women	54	0.0000664, 0.0000793, 0.0000944, 0.000112, 0.000134
	White	Women	55	0.0000739, 0.0000874, 0.000103, 0.000122, 0.000144
	White	Women	56	0.0000819, 0.0000961, 0.000113, 0.000132, 0.000155
	White	Women	57	0.0000906, 0.000106, 0.000123, 0.000143, 0.000166
	White	Women	58	0.0001, 0.000116, 0.000134, 0.000155, 0.000179
	White	Women	59	0.00011, 0.000127, 0.000146, 0.000168, 0.000194
	White	Women	60	0.000121, 0.000139, 0.00016, 0.000183, 0.00021
	White	Women	61	0.000133, 0.000153, 0.000175, 0.0002, 0.000229
	White	Women	62	0.000146, 0.000167, 0.000191, 0.000218, 0.00025
	White	Women	63	0.000159, 0.000182, 0.000208, 0.000238, 0.000272
	White	Women	64	0.000175, 0.000199, 0.000227, 0.000258, 0.000295
	White	Women	65	0.000192, 0.000219, 0.000248, 0.000282, 0.000321
	White	Women	66	0.000212, 0.000241, 0.000273, 0.00031, 0.000352
	White	Women	67	0.000235, 0.000268, 0.000304, 0.000346, 0.000393
	White	Women	68	0.000264, 0.000301, 0.000342, 0.000389, 0.000444
	White	Women	69	0.000299, 0.000341, 0.000389, 0.000443, 0.000505
	White	Women	70	0.000343, 0.000391, 0.000445, 0.000506, 0.000577
	White	Women	71	0.000395, 0.000449, 0.000511, 0.000581, 0.000662
	White	Women	72	0.000453, 0.000517, 0.000588, 0.00067, 0.000764
	White	Women	73	0.000519, 0.000593, 0.000677, 0.000772, 0.000883
	White	Women	74	0.000595, 0.000681, 0.000778, 0.000889, 0.00102
	White	Women	75	0.000685, 0.000783, 0.000894, 0.00102, 0.00117
	White	Women	76	0.000788, 0.000902, 0.00103, 0.00118, 0.00135
	White	Women	77	0.000904, 0.00104, 0.00119, 0.00137, 0.00157
	White	Women	78	0.00103, 0.0012, 0.00138, 0.0016, 0.00185
	White	Women	79	0.00118, 0.00138, 0.00161, 0.00187, 0.00219
	White	Women	80	0.00135, 0.00159, 0.00187, 0.00219, 0.00258
	White	Women	81	0.00154, 0.00183, 0.00216, 0.00256, 0.00303
	White	Women	82	0.00176, 0.00209, 0.00249, 0.00296, 0.00352
	White	Women	83	0.00198, 0.00237, 0.00283, 0.00339, 0.00406
	White	Women	84	0.0022, 0.00265, 0.00319, 0.00383, 0.00461

Stroke mortality rates for 2030 (0.01, 0.2, 0.5, 0.8. 0.99 percentiles of the empirical distribution produced during forecasting)

Variable	Race/ethnicity	Sex	Age	Distribution
	Black	Men	30	0.0000141, 0.0000203, 0.0000292, 0.0000421, 0.0000607
	Black	Men	31	0.0000176, 0.0000245, 0.0000342, 0.0000477, 0.0000667
	Black	Men	32	0.0000213, 0.0000292, 0.0000399, 0.0000545, 0.0000747
	Black	Men	33	0.0000252, 0.0000341, 0.0000461, 0.0000624, 0.0000846
	Black	Men	34	0.0000291, 0.0000392, 0.0000528, 0.0000711, 0.000096
	Black	Men	35	0.000033, 0.0000444, 0.0000597, 0.0000803, 0.000108
	Black	Men	36	0.000037, 0.0000497, 0.0000666, 0.0000892, 0.00012
	Black	Men	37	0.0000413, 0.0000551, 0.0000733, 0.0000975, 0.00013
	Black	Men	38	0.0000459, 0.0000607, 0.0000799, 0.000105, 0.000139
	Black	Men	39	0.0000508, 0.0000663, 0.0000863, 0.000112, 0.000147
	Black	Men	40	0.000056, 0.0000722, 0.0000929, 0.000119, 0.000154
	Black	Men	41	0.0000623, 0.0000789, 0.0000998, 0.000126, 0.00016
	Black	Men	42	0.0000704, 0.0000871, 0.000108, 0.000133, 0.000164
	Black	Men	43	0.000079, 0.000096, 0.000117, 0.000141, 0.000172
	Black	Men	44	0.0000864, 0.000105, 0.000127, 0.000154, 0.000188
	Black	Men	45	0.0000939, 0.000115, 0.00014, 0.000171, 0.000209
	Black	Men	46	0.000104, 0.000127, 0.000155, 0.00019, 0.000232
	Black	Men	47	0.000116, 0.000142, 0.000173, 0.000211, 0.000259
	Black	Men	48	0.000128, 0.000158, 0.000194, 0.000238, 0.000294
	Black	Men	49	0.000143, 0.000177, 0.000219, 0.000271, 0.000336
	Black	Men	50	0.000164, 0.000202, 0.000249, 0.000308, 0.000381
	Black	Men	51	0.000193, 0.000234, 0.000285, 0.000346, 0.000421
	Black	Men	52	0.000229, 0.000273, 0.000324, 0.000386, 0.00046
	Black	Men	53	0.000266, 0.000313, 0.000367, 0.000431, 0.000507
	Black	Men	54	0.000301, 0.000352, 0.000412, 0.000481, 0.000563
	Black	Men	55	0.000334, 0.00039, 0.000456, 0.000533, 0.000623
	Black	Men	56	0.000365, 0.000428, 0.0005, 0.000585, 0.000684
	Black	Men	57	0.000397, 0.000465, 0.000544, 0.000636, 0.000745
	Black	Men	58	0.00043, 0.000503, 0.000588, 0.000686, 0.000802
	Black	Men	59	0.000465, 0.000543, 0.000632, 0.000735, 0.000857
	Black	Men	60	0.000495, 0.000579, 0.000675, 0.000788, 0.000921
	Black	Men	61	0.00052, 0.000612, 0.000718, 0.000843, 0.000991
	Black	Men	62	0.000547, 0.000645, 0.000758, 0.000892, 0.00105
	Black	Men	63	0.000583, 0.000682, 0.000797, 0.000931, 0.00109
	Black	Men	64	0.000624, 0.000723, 0.000836, 0.000968, 0.00112
	Black	Men	65	0.000662, 0.000764, 0.00088, 0.00101, 0.00117
	Black	Men	66	0.000699, 0.000808, 0.000932, 0.00108, 0.00124
	Black	Men	67	0.000743, 0.000861, 0.000996, 0.00115, 0.00134
	Black	Men	68	0.000801, 0.000928, 0.00107, 0.00124, 0.00144
	Black	Men	69	0.000872, 0.00101, 0.00116, 0.00134, 0.00155
	Black	Men	70	0.000949, 0.00109, 0.00126, 0.00145, 0.00168
	Black	Men	71	0.00103, 0.00119, 0.00137, 0.00158, 0.00182
	Black	Men	72	0.00111, 0.00129, 0.00148, 0.00171, 0.00197

Variable	Race/ethnicity	Sex	Age	Distribution
	Black	Men	73	0.0012, 0.00139, 0.0016, 0.00185, 0.00214
	Black	Men	74	0.00128, 0.00149, 0.00173, 0.00201, 0.00233
	Black	Men	75	0.00137, 0.0016, 0.00187, 0.00218, 0.00255
	Black	Men	76	0.00148, 0.00173, 0.00203, 0.00238, 0.00279
	Black	Men	77	0.00161, 0.00189, 0.00221, 0.0026, 0.00305
	Black	Men	78	0.00175, 0.00206, 0.00242, 0.00284, 0.00335
	Black	Men	79	0.00189, 0.00223, 0.00264, 0.00311, 0.00368
	Black	Men	80	0.00203, 0.00242, 0.00287, 0.00341, 0.00405
	Black	Men	81	0.00217, 0.0026, 0.0031, 0.0037, 0.00443
	Black	Men	82	0.00231, 0.00278, 0.00333, 0.00399, 0.00479
	Black	Men	83	0.00244, 0.00295, 0.00355, 0.00427, 0.00514
	Black	Men	84	0.00257, 0.00311, 0.00376, 0.00454, 0.00549
	Black	Women	30	0.00000994, 0.0000155, 0.0000239, 0.000037, 0.0000576
	Black	Women	31	0.0000117, 0.000018, 0.0000276, 0.0000422, 0.0000649
	Black	Women	32	0.0000135, 0.0000207, 0.0000316, 0.0000483, 0.000074
	Black	Women	33	0.0000154, 0.0000236, 0.0000361, 0.0000552, 0.0000848
	Black	Women	34	0.0000172, 0.0000266, 0.000041, 0.000063, 0.0000975
	Black	Women	35	0.0000191, 0.0000297, 0.000046, 0.0000712, 0.000111
	Black	Women	36	0.0000214, 0.0000332, 0.0000513, 0.0000792, 0.000123
	Black	Women	37	0.0000242, 0.0000371, 0.0000566, 0.0000864, 0.000132
	Black	Women	38	0.0000275, 0.0000414, 0.0000621, 0.0000931, 0.00014
	Black	Women	39	0.000031, 0.0000459, 0.0000677, 0.0000999, 0.000148
	Black	Women	40	0.0000343, 0.0000503, 0.0000735, 0.000107, 0.000157
	Black	Women	41	0.0000378, 0.0000549, 0.0000794, 0.000115, 0.000166
	Black	Women	42	0.0000421, 0.0000601, 0.0000855, 0.000122, 0.000174
	Black	Women	43	0.0000471, 0.000066, 0.0000923, 0.000129, 0.000181
	Black	Women	44	0.0000524, 0.0000725, 0.0001, 0.000138, 0.000191
	Black	Women	45	0.0000582, 0.0000797, 0.000109, 0.000149, 0.000203
	Black	Women	46	0.0000654, 0.0000884, 0.000119, 0.00016, 0.000217
	Black	Women	47	0.0000749, 0.0000991, 0.000131, 0.000173, 0.000229
	Black	Women	48	0.000086, 0.000112, 0.000144, 0.000187, 0.000243
	Black	Women	49	0.0000977, 0.000125, 0.00016, 0.000204, 0.000261
	Black	Women	50	0.000109, 0.000139, 0.000176, 0.000224, 0.000285
	Black	Women	51	0.000122, 0.000154, 0.000194, 0.000245, 0.000311
	Black	Women	52	0.000136, 0.00017, 0.000213, 0.000266, 0.000334
	Black	Women	53	0.000151, 0.000188, 0.000233, 0.000288, 0.000358
	Black	Women	54	0.000165, 0.000205, 0.000253, 0.000313, 0.000388
	Black	Women	55	0.000178, 0.000222, 0.000275, 0.00034, 0.000422
	Black	Women	56	0.000194, 0.00024, 0.000298, 0.000369, 0.000457
	Black	Women	57	0.000213, 0.000263, 0.000323, 0.000397, 0.000488
	Black	Women	58	0.000238, 0.000289, 0.00035, 0.000424, 0.000515
	Black	Women	59	0.000265, 0.000317, 0.000379, 0.000453, 0.000542
	Black	Women	60	0.00029, 0.000345, 0.000408, 0.000484, 0.000575

Variable	Race/ethnicity	Sex	Age	Distribution
	Black	Women	61	0.000311, 0.000369, 0.000437, 0.000518, 0.000614
	Black	Women	62	0.00033, 0.000392, 0.000465, 0.000551, 0.000655
	Black	Women	63	0.000347, 0.000413, 0.000491, 0.000583, 0.000693
	Black	Women	64	0.000368, 0.000436, 0.000517, 0.000612, 0.000727
	Black	Women	65	0.000392, 0.000463, 0.000546, 0.000643, 0.000759
	Black	Women	66	0.000421, 0.000495, 0.00058, 0.00068, 0.000798
	Black	Women	67	0.000458, 0.000534, 0.000623, 0.000726, 0.000847
	Black	Women	68	0.000502, 0.000583, 0.000676, 0.000784, 0.00091
	Black	Women	69	0.000557, 0.000643, 0.000741, 0.000855, 0.000988
	Black	Women	70	0.00062, 0.000713, 0.00082, 0.000942, 0.00108
	Black	Women	71	0.000689, 0.000792, 0.00091, 0.00105, 0.0012
	Black	Women	72	0.000765, 0.000881, 0.00101, 0.00116, 0.00134
	Black	Women	73	0.000852, 0.000981, 0.00113, 0.0013, 0.0015
	Black	Women	74	0.000952, 0.0011, 0.00126, 0.00145, 0.00167
	Black	Women	75	0.00106, 0.00123, 0.00141, 0.00162, 0.00186
	Black	Women	76	0.00118, 0.00137, 0.00158, 0.00182, 0.0021
	Black	Women	77	0.0013, 0.00152, 0.00177, 0.00206, 0.00241
	Black	Women	78	0.00143, 0.00169, 0.00199, 0.00235, 0.00277
	Black	Women	79	0.00157, 0.00187, 0.00224, 0.00267, 0.00319
	Black	Women	80	0.00173, 0.00208, 0.0025, 0.00301, 0.00362
	Black	Women	81	0.0019, 0.00231, 0.00279, 0.00337, 0.00408
	Black	Women	82	0.00208, 0.00253, 0.00308, 0.00374, 0.00456
	Black	Women	83	0.00225, 0.00276, 0.00338, 0.00413, 0.00507
	Black	Women	84	0.00241, 0.00298, 0.00367, 0.00452, 0.00559
	Hispanic	Men	30	0.00000647, 0.00000946, 0.0000138, 0.00002, 0.0000293
	Hispanic	Men	31	0.00000789, 0.0000111, 0.0000157, 0.000022, 0.000031
	Hispanic	Men	32	0.00000948, 0.000013, 0.0000178, 0.0000243, 0.0000333
	Hispanic	Men	33	0.0000112, 0.000015, 0.0000201, 0.000027, 0.0000364
	Hispanic	Men	34	0.0000128, 0.0000171, 0.0000227, 0.0000302, 0.0000402
	Hispanic	Men	35	0.0000145, 0.0000192, 0.0000254, 0.0000336, 0.0000446
	Hispanic	Men	36	0.0000163, 0.0000215, 0.0000283, 0.0000372, 0.0000491
	Hispanic	Men	37	0.0000182, 0.0000239, 0.0000312, 0.0000407, 0.0000533
	Hispanic	Men	38	0.0000203, 0.0000263, 0.0000341, 0.0000442, 0.0000573
	Hispanic	Men	39	0.0000224, 0.0000289, 0.0000371, 0.0000477, 0.0000614
	Hispanic	Men	40	0.0000247, 0.0000315, 0.0000402, 0.0000512, 0.0000654
	Hispanic	Men	41	0.0000273, 0.0000344, 0.0000433, 0.0000545, 0.0000687
	Hispanic	Men	42	0.0000305, 0.0000378, 0.0000467, 0.0000577, 0.0000714
	Hispanic	Men	43	0.0000338, 0.0000413, 0.0000504, 0.0000614, 0.0000749
	Hispanic	Men	44	0.0000371, 0.000045, 0.0000546, 0.0000661, 0.0000803
	Hispanic	Men	45	0.0000406, 0.0000492, 0.0000595, 0.0000719, 0.0000872
	Hispanic	Men	46	0.0000448, 0.0000541, 0.0000652, 0.0000786, 0.0000949
	Hispanic	Men	47	0.00005, 0.0000601, 0.000072, 0.0000863, 0.000104
	Hispanic	Men	48	0.0000563, 0.0000672, 0.0000801, 0.0000954, 0.000114

Variable	Race/ethnicity	Sex	Age	Distribution
	Hispanic	Men	49	0.0000639, 0.0000758, 0.0000897, 0.000106, 0.000126
	Hispanic	Men	50	0.0000734, 0.0000861, 0.000101, 0.000118, 0.000139
	Hispanic	Men	51	0.0000849, 0.0000984, 0.000114, 0.000132, 0.000153
	Hispanic	Men	52	0.0000973, 0.000112, 0.000129, 0.000148, 0.00017
	Hispanic	Men	53	0.000108, 0.000125, 0.000145, 0.000167, 0.000193
	Hispanic	Men	54	0.000117, 0.000138, 0.000161, 0.000189, 0.000222
	Hispanic	Men	55	0.000126, 0.00015, 0.000179, 0.000213, 0.000254
	Hispanic	Men	56	0.000136, 0.000164, 0.000197, 0.000236, 0.000284
	Hispanic	Men	57	0.000148, 0.000179, 0.000215, 0.000259, 0.000312
	Hispanic	Men	58	0.000163, 0.000195, 0.000234, 0.000281, 0.000337
	Hispanic	Men	59	0.000179, 0.000213, 0.000254, 0.000302, 0.000359
	Hispanic	Men	60	0.000197, 0.000232, 0.000274, 0.000322, 0.000381
	Hispanic	Men	61	0.000214, 0.000251, 0.000294, 0.000343, 0.000402
	Hispanic	Men	62	0.000231, 0.00027, 0.000314, 0.000365, 0.000425
	Hispanic	Men	63	0.000248, 0.000288, 0.000334, 0.000388, 0.000451
	Hispanic	Men	64	0.000264, 0.000307, 0.000356, 0.000413, 0.00048
	Hispanic	Men	65	0.000282, 0.000328, 0.000381, 0.000442, 0.000514
	Hispanic	Men	66	0.000303, 0.000353, 0.00041, 0.000477, 0.000556
	Hispanic	Men	67	0.000326, 0.000381, 0.000446, 0.000521, 0.00061
	Hispanic	Men	68	0.000354, 0.000416, 0.000489, 0.000574, 0.000675
	Hispanic	Men	69	0.000388, 0.000458, 0.00054, 0.000636, 0.000751
	Hispanic	Men	70	0.000431, 0.000509, 0.0006, 0.000707, 0.000835
	Hispanic	Men	71	0.000482, 0.000568, 0.000668, 0.000786, 0.000927
	Hispanic	Men	72	0.000539, 0.000634, 0.000745, 0.000875, 0.00103
	Hispanic	Men	73	0.000601, 0.000707, 0.00083, 0.000975, 0.00115
	Hispanic	Men	74	0.000671, 0.000789, 0.000926, 0.00109, 0.00128
	Hispanic	Men	75	0.000749, 0.000881, 0.00103, 0.00121, 0.00143
	Hispanic	Men	76	0.000837, 0.000986, 0.00116, 0.00136, 0.0016
	Hispanic	Men	77	0.000935, 0.0011, 0.0013, 0.00153, 0.00181
	Hispanic	Men	78	0.00104, 0.00123, 0.00146, 0.00173, 0.00205
	Hispanic	Men	79	0.00116, 0.00138, 0.00164, 0.00195, 0.00232
	Hispanic	Men	80	0.00128, 0.00153, 0.00183, 0.0022, 0.00264
	Hispanic	Men	81	0.0014, 0.00169, 0.00204, 0.00246, 0.00298
	Hispanic	Men	82	0.00151, 0.00185, 0.00225, 0.00275, 0.00335
	Hispanic	Men	83	0.00163, 0.00201, 0.00247, 0.00304, 0.00375
	Hispanic	Men	84	0.00174, 0.00216, 0.00268, 0.00333, 0.00414
	Hispanic	Women	30	0.00000376, 0.00000597, 0.00000945, 0.000015, 0.0000238
	Hispanic	Women	31	0.00000451, 0.00000695, 0.0000107, 0.0000163, 0.0000251
	Hispanic	Women	32	0.00000535, 0.00000805, 0.0000121, 0.0000181, 0.0000272
	Hispanic	Women	33	0.00000625, 0.00000926, 0.0000137, 0.0000202, 0.0000299
	Hispanic	Women	34	0.00000725, 0.0000106, 0.0000155, 0.0000226, 0.000033
	Hispanic	Women	35	0.00000838, 0.0000121, 0.0000174, 0.000025, 0.0000361
	Hispanic	Women	36	0.00000966, 0.0000137, 0.0000194, 0.0000275, 0.000039

Variable	Race/ethnicity	Sex	Age	Distribution
	Hispanic	Women	37	0.0000111, 0.0000154, 0.0000215, 0.0000299, 0.0000416
	Hispanic	Women	38	0.0000126, 0.0000172, 0.0000236, 0.0000322, 0.0000442
	Hispanic	Women	39	0.0000141, 0.0000191, 0.0000257, 0.0000346, 0.0000468
	Hispanic	Women	40	0.0000157, 0.000021, 0.0000279, 0.000037, 0.0000493
	Hispanic	Women	41	0.0000175, 0.000023, 0.0000301, 0.0000394, 0.0000517
	Hispanic	Women	42	0.0000195, 0.0000252, 0.0000325, 0.0000419, 0.0000541
	Hispanic	Women	43	0.0000214, 0.0000275, 0.0000351, 0.0000448, 0.0000575
	Hispanic	Women	44	0.0000235, 0.00003, 0.0000381, 0.0000484, 0.0000616
	Hispanic	Women	45	0.0000263, 0.0000331, 0.0000415, 0.0000522, 0.0000657
	Hispanic	Women	46	0.0000299, 0.0000369, 0.0000455, 0.0000561, 0.0000692
	Hispanic	Women	47	0.0000343, 0.0000415, 0.0000501, 0.0000604, 0.0000731
	Hispanic	Women	48	0.0000389, 0.0000464, 0.0000553, 0.0000658, 0.0000785
	Hispanic	Women	49	0.0000433, 0.0000516, 0.0000613, 0.0000728, 0.0000867
	Hispanic	Women	50	0.0000476, 0.000057, 0.0000682, 0.0000816, 0.0000978
	Hispanic	Women	51	0.0000519, 0.0000629, 0.000076, 0.0000919, 0.000111
	Hispanic	Women	52	0.0000564, 0.0000692, 0.0000847, 0.000104, 0.000127
	Hispanic	Women	53	0.0000612, 0.000076, 0.0000941, 0.000117, 0.000145
	Hispanic	Women	54	0.0000665, 0.0000833, 0.000104, 0.00013, 0.000163
	Hispanic	Women	55	0.0000727, 0.0000915, 0.000115, 0.000144, 0.000181
	Hispanic	Women	56	0.0000801, 0.000101, 0.000126, 0.000158, 0.000199
	Hispanic	Women	57	0.0000884, 0.000111, 0.000138, 0.000172, 0.000216
	Hispanic	Women	58	0.0000972, 0.000121, 0.000151, 0.000188, 0.000234
	Hispanic	Women	59	0.000106, 0.000132, 0.000164, 0.000204, 0.000254
	Hispanic	Women	60	0.000116, 0.000144, 0.000178, 0.000221, 0.000275
	Hispanic	Women	61	0.000126, 0.000156, 0.000193, 0.000239, 0.000296
	Hispanic	Women	62	0.000136, 0.000168, 0.000208, 0.000256, 0.000317
	Hispanic	Women	63	0.000147, 0.000181, 0.000223, 0.000275, 0.000339
	Hispanic	Women	64	0.000157, 0.000194, 0.000239, 0.000294, 0.000363
	Hispanic	Women	65	0.000169, 0.000209, 0.000257, 0.000317, 0.000391
	Hispanic	Women	66	0.000183, 0.000226, 0.000279, 0.000344, 0.000426
	Hispanic	Women	67	0.000199, 0.000247, 0.000305, 0.000378, 0.000468
	Hispanic	Women	68	0.00022, 0.000273, 0.000338, 0.000419, 0.000521
	Hispanic	Women	69	0.000246, 0.000306, 0.000378, 0.000468, 0.000581
	Hispanic	Women	70	0.000281, 0.000346, 0.000426, 0.000525, 0.000647
	Hispanic	Women	71	0.000325, 0.000396, 0.000483, 0.000588, 0.000718
	Hispanic	Women	72	0.000378, 0.000456, 0.000548, 0.000658, 0.000793
	Hispanic	Women	73	0.000442, 0.000525, 0.000622, 0.000738, 0.000876
	Hispanic	Women	74	0.000514, 0.000603, 0.000708, 0.00083, 0.000975
	Hispanic	Women	75	0.000593, 0.000692, 0.000807, 0.000941, 0.0011
	Hispanic	Women	76	0.00068, 0.000793, 0.000923, 0.00107, 0.00125
	Hispanic	Women	77	0.000779, 0.000909, 0.00106, 0.00123, 0.00144
	Hispanic	Women	78	0.000895, 0.00104, 0.00122, 0.00142, 0.00165
	Hispanic	Women	79	0.00103, 0.0012, 0.0014, 0.00163, 0.0019

Variable	Race/ethnicity	Sex	Age	Distribution
	Hispanic	Women	80	0.00118, 0.00137, 0.0016, 0.00186, 0.00217
	Hispanic	Women	81	0.00132, 0.00155, 0.00182, 0.00213, 0.0025
	Hispanic	Women	82	0.00145, 0.00173, 0.00205, 0.00244, 0.0029
	Hispanic	Women	83	0.00156, 0.00189, 0.00229, 0.00278, 0.00337
	Hispanic	Women	84	0.00166, 0.00205, 0.00253, 0.00313, 0.00387
	White	Men	30	0.00000511, 0.00000733, 0.0000105, 0.000015, 0.0000215
	White	Men	31	0.00000609, 0.00000851, 0.0000118, 0.0000165, 0.000023
	White	Men	32	0.00000719, 0.00000981, 0.0000134, 0.0000182, 0.0000248
	White	Men	33	0.0000084, 0.0000112, 0.000015, 0.0000201, 0.0000269
	White	Men	34	0.00000966, 0.0000128, 0.0000168, 0.0000221, 0.0000292
	White	Men	35	0.0000109, 0.0000143, 0.0000187, 0.0000244, 0.0000319
	White	Men	36	0.0000122, 0.0000158, 0.0000206, 0.0000268, 0.0000349
	White	Men	37	0.0000134, 0.0000174, 0.0000226, 0.0000293, 0.000038
	White	Men	38	0.0000147, 0.000019, 0.0000246, 0.0000318, 0.0000412
	White	Men	39	0.000016, 0.0000207, 0.0000267, 0.0000344, 0.0000444
	White	Men	40	0.0000175, 0.0000225, 0.0000288, 0.0000368, 0.0000472
	White	Men	41	0.0000194, 0.0000245, 0.000031, 0.000039, 0.0000493
	White	Men	42	0.0000218, 0.000027, 0.0000333, 0.0000411, 0.0000508
	White	Men	43	0.0000246, 0.0000297, 0.000036, 0.0000435, 0.0000527
	White	Men	44	0.0000273, 0.0000327, 0.0000391, 0.0000467, 0.0000558
	White	Men	45	0.0000304, 0.0000361, 0.0000428, 0.0000507, 0.0000601
	White	Men	46	0.0000341, 0.0000401, 0.0000472, 0.0000554, 0.0000652
	White	Men	47	0.0000383, 0.0000448, 0.0000524, 0.0000612, 0.0000716
	White	Men	48	0.0000427, 0.0000501, 0.0000585, 0.0000684, 0.0000801
	White	Men	49	0.0000474, 0.0000559, 0.0000658, 0.0000775, 0.0000913
	White	Men	50	0.0000527, 0.0000627, 0.0000743, 0.0000882, 0.000105
	White	Men	51	0.0000593, 0.0000707, 0.0000841, 0.0001, 0.000119
	White	Men	52	0.0000675, 0.0000802, 0.0000951, 0.000113, 0.000134
	White	Men	53	0.0000768, 0.0000908, 0.000107, 0.000126, 0.00015
	White	Men	54	0.0000863, 0.000102, 0.00012, 0.000141, 0.000167
	White	Men	55	0.0000957, 0.000113, 0.000134, 0.000158, 0.000187
	White	Men	56	0.000105, 0.000125, 0.000148, 0.000176, 0.000209
	White	Men	57	0.000114, 0.000137, 0.000163, 0.000195, 0.000233
	White	Men	58	0.000124, 0.000149, 0.000179, 0.000216, 0.00026
	White	Men	59	0.000135, 0.000163, 0.000197, 0.000238, 0.000288
	White	Men	60	0.000148, 0.000179, 0.000216, 0.00026, 0.000314
	White	Men	61	0.000164, 0.000197, 0.000235, 0.000282, 0.000338
	White	Men	62	0.000182, 0.000216, 0.000256, 0.000303, 0.000359
	White	Men	63	0.000203, 0.000238, 0.000277, 0.000323, 0.000377
	White	Men	64	0.000227, 0.000261, 0.0003, 0.000344, 0.000396
	White	Men	65	0.000252, 0.000287, 0.000325, 0.00037, 0.00042
	White	Men	66	0.000279, 0.000315, 0.000356, 0.000401, 0.000454
	White	Men	67	0.000307, 0.000347, 0.000392, 0.000443, 0.0005

Variable	Race/ethnicity	Sex	Age	Distribution
	White	Men	68	0.00034, 0.000385, 0.000437, 0.000495, 0.000561
	White	Men	69	0.000378, 0.000431, 0.00049, 0.000557, 0.000635
	White	Men	70	0.000423, 0.000484, 0.000552, 0.000631, 0.000722
	White	Men	71	0.000475, 0.000545, 0.000624, 0.000715, 0.000821
	White	Men	72	0.000533, 0.000613, 0.000705, 0.000811, 0.000934
	White	Men	73	0.000595, 0.000689, 0.000796, 0.00092, 0.00106
	White	Men	74	0.000664, 0.000773, 0.000898, 0.00104, 0.00121
	White	Men	75	0.000743, 0.000869, 0.00102, 0.00119, 0.00139
	White	Men	76	0.000833, 0.00098, 0.00115, 0.00135, 0.00159
	White	Men	77	0.000935, 0.00111, 0.00131, 0.00155, 0.00184
	White	Men	78	0.00105, 0.00126, 0.0015, 0.00179, 0.00215
	White	Men	79	0.00117, 0.00142, 0.00172, 0.00208, 0.00253
	White	Men	80	0.0013, 0.0016, 0.00197, 0.00242, 0.00298
	White	Men	81	0.00145, 0.00181, 0.00225, 0.00279, 0.00347
	White	Men	82	0.00162, 0.00203, 0.00254, 0.00317, 0.00397
	White	Men	83	0.00179, 0.00226, 0.00284, 0.00357, 0.00451
	White	Men	84	0.00195, 0.00248, 0.00314, 0.00398, 0.00506
	White	Women	30	0.00000457, 0.0000067, 0.00000978, 0.0000143, 0.0000209
	White	Women	31	0.00000545, 0.00000781, 0.0000111, 0.0000159, 0.0000228
	White	Women	32	0.00000646, 0.00000907, 0.0000127, 0.0000178, 0.0000249
	White	Women	33	0.00000761, 0.0000105, 0.0000144, 0.0000198, 0.0000273
	White	Women	34	0.00000881, 0.000012, 0.0000162, 0.000022, 0.0000298
	White	Women	35	0.00001, 0.0000135, 0.0000181, 0.0000242, 0.0000326
	White	Women	36	0.0000112, 0.000015, 0.0000199, 0.0000265, 0.0000354
	White	Women	37	0.0000125, 0.0000165, 0.0000218, 0.0000287, 0.000038
	White	Women	38	0.0000137, 0.000018, 0.0000236, 0.0000309, 0.0000405
	White	Women	39	0.000015, 0.0000196, 0.0000254, 0.0000329, 0.0000428
	White	Women	40	0.0000165, 0.0000212, 0.0000271, 0.0000348, 0.0000447
	White	Women	41	0.0000182, 0.000023, 0.0000289, 0.0000364, 0.0000458
	White	Women	42	0.0000204, 0.0000251, 0.0000308, 0.0000377, 0.0000464
	White	Women	43	0.0000227, 0.0000273, 0.0000328, 0.0000394, 0.0000475
	White	Women	44	0.0000248, 0.0000296, 0.0000352, 0.0000419, 0.00005
	White	Women	45	0.0000269, 0.000032, 0.000038, 0.0000452, 0.0000537
	White	Women	46	0.0000293, 0.0000349, 0.0000414, 0.0000491, 0.0000584
	White	Women	47	0.0000321, 0.0000382, 0.0000453, 0.0000539, 0.0000641
	White	Women	48	0.000035, 0.0000419, 0.00005, 0.0000597, 0.0000714
	White	Women	49	0.0000381, 0.000046, 0.0000554, 0.0000667, 0.0000804
	White	Women	50	0.0000418, 0.0000507, 0.0000616, 0.0000747, 0.0000907
	White	Women	51	0.0000463, 0.0000564, 0.0000685, 0.0000833, 0.000101
	White	Women	52	0.0000519, 0.0000629, 0.0000762, 0.0000922, 0.000112
	White	Women	53	0.0000583, 0.0000702, 0.0000843, 0.000101, 0.000122
	White	Women	54	0.0000651, 0.0000778, 0.0000929, 0.000111, 0.000132
	White	Women	55	0.0000725, 0.0000859, 0.000102, 0.00012, 0.000143

Variable	Race/ethnicity	Sex	Age	Distribution
	White	Women	56	0.0000804, 0.0000945, 0.000111, 0.00013, 0.000153
	White	Women	57	0.0000889, 0.000104, 0.000121, 0.000141, 0.000164
	White	Women	58	0.000098, 0.000114, 0.000132, 0.000153, 0.000177
	White	Women	59	0.000108, 0.000125, 0.000144, 0.000166, 0.000192
	White	Women	60	0.000119, 0.000137, 0.000157, 0.000181, 0.000209
	White	Women	61	0.00013, 0.00015, 0.000172, 0.000198, 0.000227
	White	Women	62	0.000142, 0.000163, 0.000188, 0.000215, 0.000247
	White	Women	63	0.000155, 0.000178, 0.000204, 0.000234, 0.000268
	White	Women	64	0.00017, 0.000195, 0.000222, 0.000254, 0.000291
	White	Women	65	0.000187, 0.000213, 0.000243, 0.000277, 0.000316
	White	Women	66	0.000206, 0.000235, 0.000267, 0.000304, 0.000347
	White	Women	67	0.000229, 0.000261, 0.000297, 0.000339, 0.000387
	White	Women	68	0.000256, 0.000293, 0.000334, 0.000382, 0.000436
	White	Women	69	0.00029, 0.000332, 0.00038, 0.000434, 0.000496
	White	Women	70	0.000333, 0.00038, 0.000434, 0.000496, 0.000567
	White	Women	71	0.000383, 0.000437, 0.000499, 0.00057, 0.000651
	White	Women	72	0.000439, 0.000503, 0.000574, 0.000656, 0.000751
	White	Women	73	0.000503, 0.000577, 0.00066, 0.000756, 0.000867
	White	Women	74	0.000577, 0.000662, 0.000759, 0.00087, 0.000998
	White	Women	75	0.000663, 0.000761, 0.000872, 0.000999, 0.00115
	White	Women	76	0.000763, 0.000876, 0.001, 0.00115, 0.00132
	White	Women	77	0.000876, 0.00101, 0.00116, 0.00134, 0.00154
	White	Women	78	0.001, 0.00116, 0.00135, 0.00156, 0.00181
	White	Women	79	0.00114, 0.00134, 0.00157, 0.00183, 0.00215
	White	Women	80	0.00131, 0.00154, 0.00182, 0.00215, 0.00254
	White	Women	81	0.0015, 0.00178, 0.00211, 0.0025, 0.00297
	White	Women	82	0.0017, 0.00203, 0.00243, 0.00289, 0.00346
	White	Women	83	0.00191, 0.0023, 0.00276, 0.00331, 0.00398
	White	Women	84	0.00213, 0.00258, 0.00311, 0.00375, 0.00453
Stroke mortality rates for 2031 (0.01, 0.2, 0.5, 0.8. 0.99 percentiles of the empirical distribution produced during forecasting)				
	Black	Men	30	0.0000138, 0.0000201, 0.0000291, 0.0000422, 0.0000614
	Black	Men	31	0.0000172, 0.0000243, 0.0000341, 0.0000478, 0.0000673
	Black	Men	32	0.000021, 0.0000289, 0.0000397, 0.0000546, 0.0000753
	Black	Men	33	0.0000248, 0.0000338, 0.000046, 0.0000625, 0.0000853
	Black	Men	34	0.0000286, 0.0000389, 0.0000526, 0.0000712, 0.0000967
	Black	Men	35	0.0000325, 0.000044, 0.0000595, 0.0000803, 0.000109
	Black	Men	36	0.0000364, 0.0000491, 0.0000662, 0.0000892, 0.000121
	Black	Men	37	0.0000405, 0.0000544, 0.0000728, 0.0000974, 0.000131
	Black	Men	38	0.000045, 0.0000598, 0.0000792, 0.000105, 0.000139
	Black	Men	39	0.0000496, 0.0000652, 0.0000854, 0.000112, 0.000147
	Black	Men	40	0.0000546, 0.0000708, 0.0000916, 0.000118, 0.000154
	Black	Men	41	0.0000607, 0.0000773, 0.0000982, 0.000125, 0.000159
	Black	Men	42	0.0000686, 0.0000852, 0.000106, 0.000131, 0.000163

Variable	Race/ethnicity	Sex	Age	Distribution
	Black	Men	43	0.0000769, 0.0000938, 0.000114, 0.000139, 0.00017
	Black	Men	44	0.0000841, 0.000102, 0.000125, 0.000152, 0.000185
	Black	Men	45	0.0000913, 0.000112, 0.000137, 0.000168, 0.000206
	Black	Men	46	0.000101, 0.000124, 0.000152, 0.000186, 0.000228
	Black	Men	47	0.000113, 0.000138, 0.000169, 0.000207, 0.000254
	Black	Men	48	0.000125, 0.000154, 0.00019, 0.000233, 0.000288
	Black	Men	49	0.000139, 0.000173, 0.000215, 0.000266, 0.00033
	Black	Men	50	0.00016, 0.000198, 0.000244, 0.000302, 0.000374
	Black	Men	51	0.000188, 0.00023, 0.000279, 0.000339, 0.000414
	Black	Men	52	0.000224, 0.000267, 0.000318, 0.000379, 0.000453
	Black	Men	53	0.000261, 0.000307, 0.000361, 0.000424, 0.0005
	Black	Men	54	0.000295, 0.000346, 0.000405, 0.000474, 0.000556
	Black	Men	55	0.000327, 0.000384, 0.000449, 0.000526, 0.000616
	Black	Men	56	0.000359, 0.000421, 0.000493, 0.000577, 0.000677
	Black	Men	57	0.000389, 0.000457, 0.000536, 0.000628, 0.000738
	Black	Men	58	0.000422, 0.000495, 0.000579, 0.000678, 0.000795
	Black	Men	59	0.000456, 0.000533, 0.000622, 0.000727, 0.00085
	Black	Men	60	0.000485, 0.000568, 0.000665, 0.000778, 0.000912
	Black	Men	61	0.000509, 0.0006, 0.000706, 0.000832, 0.000981
	Black	Men	62	0.000535, 0.000632, 0.000745, 0.000879, 0.00104
	Black	Men	63	0.000569, 0.000668, 0.000782, 0.000917, 0.00108
	Black	Men	64	0.000608, 0.000707, 0.00082, 0.000951, 0.00111
	Black	Men	65	0.000645, 0.000746, 0.000862, 0.000995, 0.00115
	Black	Men	66	0.00068, 0.000788, 0.000912, 0.00106, 0.00122
	Black	Men	67	0.000722, 0.000839, 0.000973, 0.00113, 0.00131
	Black	Men	68	0.000778, 0.000904, 0.00105, 0.00122, 0.00141
	Black	Men	69	0.000846, 0.000981, 0.00113, 0.00131, 0.00152
	Black	Men	70	0.000921, 0.00107, 0.00123, 0.00142, 0.00165
	Black	Men	71	0.000999, 0.00116, 0.00134, 0.00154, 0.00179
	Black	Men	72	0.00108, 0.00125, 0.00145, 0.00167, 0.00193
	Black	Men	73	0.00116, 0.00135, 0.00156, 0.00181, 0.0021
	Black	Men	74	0.00124, 0.00145, 0.00169, 0.00196, 0.00229
	Black	Men	75	0.00133, 0.00156, 0.00182, 0.00213, 0.0025
	Black	Men	76	0.00143, 0.00169, 0.00198, 0.00233, 0.00274
	Black	Men	77	0.00156, 0.00183, 0.00216, 0.00254, 0.00299
	Black	Men	78	0.00169, 0.002, 0.00236, 0.00278, 0.00328
	Black	Men	79	0.00183, 0.00217, 0.00257, 0.00305, 0.00361
	Black	Men	80	0.00197, 0.00235, 0.0028, 0.00333, 0.00398
	Black	Men	81	0.0021, 0.00252, 0.00302, 0.00362, 0.00434
	Black	Men	82	0.00224, 0.0027, 0.00324, 0.0039, 0.0047
	Black	Men	83	0.00237, 0.00286, 0.00346, 0.00417, 0.00505
	Black	Men	84	0.00249, 0.00302, 0.00366, 0.00444, 0.00539
	Black	Women	30	0.00000977, 0.0000153, 0.0000239, 0.0000372, 0.0000583

Variable	Race/ethnicity	Sex	Age	Distribution
	Black	Women	31	0.0000115, 0.0000179, 0.0000275, 0.0000424, 0.0000656
	Black	Women	32	0.0000134, 0.0000206, 0.0000316, 0.0000484, 0.0000746
	Black	Women	33	0.0000152, 0.0000235, 0.0000361, 0.0000554, 0.0000855
	Black	Women	34	0.000017, 0.0000264, 0.0000409, 0.0000632, 0.0000981
	Black	Women	35	0.0000189, 0.0000295, 0.0000459, 0.0000713, 0.000111
	Black	Women	36	0.0000211, 0.0000329, 0.0000511, 0.0000792, 0.000123
	Black	Women	37	0.0000239, 0.0000367, 0.0000563, 0.0000863, 0.000133
	Black	Women	38	0.0000271, 0.0000409, 0.0000617, 0.0000929, 0.000141
	Black	Women	39	0.0000304, 0.0000452, 0.0000671, 0.0000996, 0.000148
	Black	Women	40	0.0000335, 0.0000495, 0.0000727, 0.000107, 0.000157
	Black	Women	41	0.0000369, 0.0000538, 0.0000783, 0.000114, 0.000166
	Black	Women	42	0.0000409, 0.0000588, 0.0000843, 0.000121, 0.000173
	Black	Women	43	0.0000458, 0.0000646, 0.0000908, 0.000128, 0.00018
	Black	Women	44	0.0000509, 0.0000708, 0.0000982, 0.000136, 0.000189
	Black	Women	45	0.0000565, 0.0000778, 0.000107, 0.000146, 0.000202
	Black	Women	46	0.0000636, 0.0000863, 0.000117, 0.000158, 0.000214
	Black	Women	47	0.0000727, 0.0000968, 0.000128, 0.00017, 0.000227
	Black	Women	48	0.0000836, 0.000109, 0.000142, 0.000184, 0.00024
	Black	Women	49	0.000095, 0.000122, 0.000157, 0.000201, 0.000259
	Black	Women	50	0.000106, 0.000136, 0.000173, 0.000221, 0.000283
	Black	Women	51	0.000119, 0.000151, 0.000191, 0.000242, 0.000308
	Black	Women	52	0.000133, 0.000167, 0.00021, 0.000264, 0.000332
	Black	Women	53	0.000148, 0.000184, 0.000229, 0.000285, 0.000356
	Black	Women	54	0.000162, 0.000201, 0.00025, 0.00031, 0.000386
	Black	Women	55	0.000175, 0.000218, 0.000271, 0.000337, 0.000421
	Black	Women	56	0.00019, 0.000236, 0.000294, 0.000365, 0.000455
	Black	Women	57	0.000209, 0.000258, 0.000319, 0.000393, 0.000486
	Black	Women	58	0.000233, 0.000284, 0.000345, 0.00042, 0.000512
	Black	Women	59	0.000259, 0.000312, 0.000374, 0.000448, 0.000539
	Black	Women	60	0.000284, 0.000338, 0.000403, 0.000479, 0.000571
	Black	Women	61	0.000305, 0.000362, 0.000431, 0.000512, 0.000609
	Black	Women	62	0.000322, 0.000384, 0.000457, 0.000544, 0.000649
	Black	Women	63	0.000339, 0.000405, 0.000482, 0.000574, 0.000685
	Black	Women	64	0.000359, 0.000427, 0.000507, 0.000602, 0.000717
	Black	Women	65	0.000382, 0.000452, 0.000534, 0.000632, 0.000748
	Black	Women	66	0.00041, 0.000483, 0.000567, 0.000667, 0.000785
	Black	Women	67	0.000445, 0.000521, 0.000609, 0.000712, 0.000833
	Black	Women	68	0.000488, 0.000568, 0.000661, 0.000769, 0.000895
	Black	Women	69	0.00054, 0.000626, 0.000725, 0.000839, 0.000972
	Black	Women	70	0.000601, 0.000694, 0.000801, 0.000924, 0.00107
	Black	Women	71	0.000668, 0.000771, 0.000889, 0.00102, 0.00118
	Black	Women	72	0.000742, 0.000858, 0.000989, 0.00114, 0.00132
	Black	Women	73	0.000826, 0.000955, 0.0011, 0.00127, 0.00147

Variable	Race/ethnicity	Sex	Age	Distribution
	Black	Women	74	0.000923, 0.00107, 0.00123, 0.00142, 0.00164
	Black	Women	75	0.00103, 0.00119, 0.00137, 0.00158, 0.00183
	Black	Women	76	0.00115, 0.00133, 0.00154, 0.00178, 0.00206
	Black	Women	77	0.00126, 0.00148, 0.00173, 0.00202, 0.00236
	Black	Women	78	0.00138, 0.00164, 0.00194, 0.0023, 0.00272
	Black	Women	79	0.00152, 0.00182, 0.00218, 0.00261, 0.00312
	Black	Women	80	0.00167, 0.00202, 0.00244, 0.00294, 0.00355
	Black	Women	81	0.00184, 0.00224, 0.00272, 0.00329, 0.004
	Black	Women	82	0.00201, 0.00246, 0.003, 0.00366, 0.00447
	Black	Women	83	0.00217, 0.00268, 0.00329, 0.00404, 0.00497
	Black	Women	84	0.00233, 0.00289, 0.00357, 0.00442, 0.00548
	Hispanic	Men	30	0.00000635, 0.00000934, 0.0000137, 0.0000201, 0.0000296
	Hispanic	Men	31	0.00000776, 0.000011, 0.0000156, 0.0000221, 0.0000313
	Hispanic	Men	32	0.00000934, 0.0000129, 0.0000177, 0.0000244, 0.0000336
	Hispanic	Men	33	0.000011, 0.0000149, 0.0000201, 0.0000271, 0.0000366
	Hispanic	Men	34	0.0000127, 0.0000169, 0.0000226, 0.0000302, 0.0000405
	Hispanic	Men	35	0.0000143, 0.0000191, 0.0000253, 0.0000337, 0.0000449
	Hispanic	Men	36	0.000016, 0.0000212, 0.0000281, 0.0000372, 0.0000494
	Hispanic	Men	37	0.0000179, 0.0000236, 0.0000309, 0.0000406, 0.0000535
	Hispanic	Men	38	0.0000199, 0.0000259, 0.0000338, 0.000044, 0.0000575
	Hispanic	Men	39	0.0000219, 0.0000284, 0.0000367, 0.0000474, 0.0000615
	Hispanic	Men	40	0.0000241, 0.0000309, 0.0000396, 0.0000508, 0.0000652
	Hispanic	Men	41	0.0000266, 0.0000337, 0.0000426, 0.0000539, 0.0000683
	Hispanic	Men	42	0.0000297, 0.0000369, 0.0000458, 0.0000569, 0.0000707
	Hispanic	Men	43	0.000033, 0.0000404, 0.0000494, 0.0000603, 0.0000739
	Hispanic	Men	44	0.0000361, 0.0000439, 0.0000534, 0.0000649, 0.0000791
	Hispanic	Men	45	0.0000394, 0.0000479, 0.0000581, 0.0000705, 0.0000857
	Hispanic	Men	46	0.0000436, 0.0000528, 0.0000637, 0.0000769, 0.0000931
	Hispanic	Men	47	0.0000487, 0.0000586, 0.0000703, 0.0000845, 0.000102
	Hispanic	Men	48	0.0000548, 0.0000656, 0.0000783, 0.0000934, 0.000112
	Hispanic	Men	49	0.0000623, 0.000074, 0.0000877, 0.000104, 0.000123
	Hispanic	Men	50	0.0000716, 0.0000842, 0.0000989, 0.000116, 0.000137
	Hispanic	Men	51	0.0000829, 0.0000963, 0.000112, 0.00013, 0.000151
	Hispanic	Men	52	0.0000952, 0.00011, 0.000126, 0.000145, 0.000167
	Hispanic	Men	53	0.000106, 0.000123, 0.000142, 0.000164, 0.00019
	Hispanic	Men	54	0.000115, 0.000135, 0.000159, 0.000187, 0.000219
	Hispanic	Men	55	0.000124, 0.000148, 0.000176, 0.00021, 0.000251
	Hispanic	Men	56	0.000134, 0.000161, 0.000194, 0.000233, 0.000281
	Hispanic	Men	57	0.000146, 0.000176, 0.000212, 0.000256, 0.000309
	Hispanic	Men	58	0.00016, 0.000192, 0.000231, 0.000277, 0.000334
	Hispanic	Men	59	0.000176, 0.00021, 0.00025, 0.000298, 0.000356
	Hispanic	Men	60	0.000192, 0.000228, 0.000269, 0.000318, 0.000377
	Hispanic	Men	61	0.00021, 0.000246, 0.000289, 0.000339, 0.000398

Variable	Race/ethnicity	Sex	Age	Distribution
	Hispanic	Men	62	0.000226, 0.000264, 0.000308, 0.00036, 0.00042
	Hispanic	Men	63	0.000242, 0.000282, 0.000328, 0.000382, 0.000445
	Hispanic	Men	64	0.000257, 0.0003, 0.000349, 0.000406, 0.000473
	Hispanic	Men	65	0.000275, 0.00032, 0.000373, 0.000434, 0.000506
	Hispanic	Men	66	0.000294, 0.000344, 0.000401, 0.000468, 0.000547
	Hispanic	Men	67	0.000317, 0.000372, 0.000436, 0.00051, 0.000599
	Hispanic	Men	68	0.000344, 0.000405, 0.000478, 0.000562, 0.000663
	Hispanic	Men	69	0.000377, 0.000446, 0.000528, 0.000623, 0.000738
	Hispanic	Men	70	0.000419, 0.000496, 0.000586, 0.000692, 0.00082
	Hispanic	Men	71	0.000468, 0.000553, 0.000652, 0.00077, 0.00091
	Hispanic	Men	72	0.000523, 0.000617, 0.000727, 0.000856, 0.00101
	Hispanic	Men	73	0.000584, 0.000688, 0.00081, 0.000954, 0.00112
	Hispanic	Men	74	0.000651, 0.000768, 0.000904, 0.00106, 0.00125
	Hispanic	Men	75	0.000727, 0.000857, 0.00101, 0.00119, 0.0014
	Hispanic	Men	76	0.000812, 0.000959, 0.00113, 0.00133, 0.00157
	Hispanic	Men	77	0.000907, 0.00107, 0.00127, 0.0015, 0.00177
	Hispanic	Men	78	0.00101, 0.0012, 0.00142, 0.00169, 0.00201
	Hispanic	Men	79	0.00112, 0.00134, 0.0016, 0.00191, 0.00228
	Hispanic	Men	80	0.00124, 0.00149, 0.00179, 0.00215, 0.00259
	Hispanic	Men	81	0.00135, 0.00164, 0.00199, 0.00241, 0.00293
	Hispanic	Men	82	0.00147, 0.0018, 0.0022, 0.00269, 0.00329
	Hispanic	Men	83	0.00158, 0.00195, 0.00241, 0.00297, 0.00368
	Hispanic	Men	84	0.00168, 0.0021, 0.00261, 0.00325, 0.00406
	Hispanic	Women	30	0.0000037, 0.00000591, 0.00000941, 0.000015, 0.000024
	Hispanic	Women	31	0.00000445, 0.00000689, 0.0000106, 0.0000164, 0.0000253
	Hispanic	Women	32	0.00000528, 0.00000798, 0.000012, 0.0000181, 0.0000274
	Hispanic	Women	33	0.00000618, 0.0000092, 0.0000136, 0.0000202, 0.0000301
	Hispanic	Women	34	0.00000717, 0.0000105, 0.0000154, 0.0000226, 0.0000332
	Hispanic	Women	35	0.00000827, 0.000012, 0.0000173, 0.000025, 0.0000363
	Hispanic	Women	36	0.00000952, 0.0000136, 0.0000193, 0.0000274, 0.0000391
	Hispanic	Women	37	0.0000109, 0.0000153, 0.0000213, 0.0000298, 0.0000418
	Hispanic	Women	38	0.0000123, 0.000017, 0.0000234, 0.0000321, 0.0000443
	Hispanic	Women	39	0.0000138, 0.0000188, 0.0000254, 0.0000344, 0.0000468
	Hispanic	Women	40	0.0000154, 0.0000206, 0.0000275, 0.0000367, 0.0000492
	Hispanic	Women	41	0.0000171, 0.0000225, 0.0000296, 0.0000389, 0.0000513
	Hispanic	Women	42	0.000019, 0.0000246, 0.0000319, 0.0000413, 0.0000535
	Hispanic	Women	43	0.0000209, 0.0000268, 0.0000344, 0.0000441, 0.0000566
	Hispanic	Women	44	0.0000229, 0.0000293, 0.0000373, 0.0000475, 0.0000606
	Hispanic	Women	45	0.0000256, 0.0000323, 0.0000406, 0.0000511, 0.0000645
	Hispanic	Women	46	0.0000291, 0.000036, 0.0000445, 0.0000549, 0.0000679
	Hispanic	Women	47	0.0000334, 0.0000404, 0.0000489, 0.0000591, 0.0000717
	Hispanic	Women	48	0.0000379, 0.0000453, 0.000054, 0.0000645, 0.000077
	Hispanic	Women	49	0.0000422, 0.0000504, 0.0000599, 0.0000714, 0.0000851

Variable	Race/ethnicity	Sex	Age	Distribution
	Hispanic	Women	50	0.0000464, 0.0000557, 0.0000668, 0.00008, 0.000096
	Hispanic	Women	51	0.0000507, 0.0000615, 0.0000745, 0.0000902, 0.000109
	Hispanic	Women	52	0.0000553, 0.0000679, 0.0000831, 0.000102, 0.000125
	Hispanic	Women	53	0.00006, 0.0000746, 0.0000925, 0.000115, 0.000143
	Hispanic	Women	54	0.0000652, 0.0000819, 0.000103, 0.000128, 0.000161
	Hispanic	Women	55	0.0000714, 0.00009, 0.000113, 0.000142, 0.000179
	Hispanic	Women	56	0.0000787, 0.000099, 0.000124, 0.000156, 0.000196
	Hispanic	Women	57	0.0000868, 0.000109, 0.000136, 0.00017, 0.000213
	Hispanic	Women	58	0.0000954, 0.000119, 0.000149, 0.000185, 0.000231
	Hispanic	Women	59	0.000104, 0.00013, 0.000162, 0.000201, 0.000251
	Hispanic	Women	60	0.000114, 0.000142, 0.000176, 0.000218, 0.000272
	Hispanic	Women	61	0.000123, 0.000153, 0.00019, 0.000235, 0.000292
	Hispanic	Women	62	0.000133, 0.000165, 0.000204, 0.000253, 0.000313
	Hispanic	Women	63	0.000143, 0.000177, 0.000219, 0.00027, 0.000334
	Hispanic	Women	64	0.000154, 0.00019, 0.000234, 0.000289, 0.000357
	Hispanic	Women	65	0.000165, 0.000204, 0.000252, 0.000311, 0.000384
	Hispanic	Women	66	0.000178, 0.000221, 0.000273, 0.000337, 0.000418
	Hispanic	Women	67	0.000194, 0.000241, 0.000298, 0.00037, 0.00046
	Hispanic	Women	68	0.000214, 0.000266, 0.00033, 0.00041, 0.000511
	Hispanic	Women	69	0.00024, 0.000298, 0.00037, 0.000459, 0.00057
	Hispanic	Women	70	0.000273, 0.000338, 0.000416, 0.000514, 0.000635
	Hispanic	Women	71	0.000315, 0.000386, 0.000471, 0.000576, 0.000704
	Hispanic	Women	72	0.000367, 0.000444, 0.000535, 0.000644, 0.000778
	Hispanic	Women	73	0.000429, 0.000511, 0.000607, 0.000722, 0.00086
	Hispanic	Women	74	0.000498, 0.000587, 0.00069, 0.000812, 0.000957
	Hispanic	Women	75	0.000575, 0.000673, 0.000787, 0.00092, 0.00108
	Hispanic	Women	76	0.00066, 0.000771, 0.0009, 0.00105, 0.00123
	Hispanic	Women	77	0.000756, 0.000884, 0.00103, 0.00121, 0.00141
	Hispanic	Women	78	0.000868, 0.00102, 0.00119, 0.00139, 0.00162
	Hispanic	Women	79	0.000998, 0.00117, 0.00136, 0.00159, 0.00186
	Hispanic	Women	80	0.00114, 0.00134, 0.00156, 0.00182, 0.00214
	Hispanic	Women	81	0.00128, 0.00151, 0.00178, 0.00209, 0.00246
	Hispanic	Women	82	0.00141, 0.00168, 0.002, 0.00239, 0.00285
	Hispanic	Women	83	0.00151, 0.00184, 0.00223, 0.00272, 0.00331
	Hispanic	Women	84	0.00161, 0.002, 0.00247, 0.00306, 0.00379
	White	Men	30	0.00000501, 0.00000724, 0.0000104, 0.000015, 0.0000218
	White	Men	31	0.00000598, 0.00000841, 0.0000118, 0.0000165, 0.0000232
	White	Men	32	0.00000707, 0.00000972, 0.0000133, 0.0000182, 0.000025
	White	Men	33	0.00000827, 0.0000111, 0.000015, 0.0000201, 0.0000271
	White	Men	34	0.00000951, 0.0000126, 0.0000167, 0.0000222, 0.0000294
	White	Men	35	0.0000107, 0.0000141, 0.0000186, 0.0000244, 0.0000322
	White	Men	36	0.0000119, 0.0000157, 0.0000205, 0.0000268, 0.0000351
	White	Men	37	0.0000132, 0.0000172, 0.0000224, 0.0000292, 0.0000382

Variable	Race/ethnicity	Sex	Age	Distribution
	White	Men	38	0.0000144, 0.0000187, 0.0000244, 0.0000317, 0.0000413
	White	Men	39	0.0000156, 0.0000203, 0.0000264, 0.0000342, 0.0000444
	White	Men	40	0.0000171, 0.000022, 0.0000284, 0.0000365, 0.0000471
	White	Men	41	0.0000189, 0.000024, 0.0000304, 0.0000386, 0.000049
	White	Men	42	0.0000212, 0.0000264, 0.0000327, 0.0000405, 0.0000503
	White	Men	43	0.0000239, 0.000029, 0.0000352, 0.0000427, 0.000052
	White	Men	44	0.0000266, 0.0000319, 0.0000382, 0.0000458, 0.000055
	White	Men	45	0.0000295, 0.0000352, 0.0000418, 0.0000497, 0.0000591
	White	Men	46	0.0000331, 0.0000391, 0.000046, 0.0000543, 0.000064
	White	Men	47	0.0000372, 0.0000436, 0.0000511, 0.0000599, 0.0000703
	White	Men	48	0.0000415, 0.0000488, 0.0000572, 0.000067, 0.0000786
	White	Men	49	0.0000461, 0.0000545, 0.0000643, 0.0000759, 0.0000896
	White	Men	50	0.0000514, 0.0000612, 0.0000727, 0.0000864, 0.000103
	White	Men	51	0.0000579, 0.0000691, 0.0000824, 0.0000982, 0.000117
	White	Men	52	0.000066, 0.0000785, 0.0000933, 0.000111, 0.000132
	White	Men	53	0.0000751, 0.0000889, 0.000105, 0.000124, 0.000147
	White	Men	54	0.0000844, 0.0000999, 0.000118, 0.000139, 0.000165
	White	Men	55	0.0000936, 0.000111, 0.000131, 0.000156, 0.000185
	White	Men	56	0.000103, 0.000122, 0.000146, 0.000173, 0.000206
	White	Men	57	0.000112, 0.000134, 0.000161, 0.000192, 0.000231
	White	Men	58	0.000121, 0.000146, 0.000177, 0.000213, 0.000258
	White	Men	59	0.000132, 0.00016, 0.000194, 0.000235, 0.000285
	White	Men	60	0.000145, 0.000176, 0.000212, 0.000257, 0.000311
	White	Men	61	0.00016, 0.000193, 0.000231, 0.000278, 0.000334
	White	Men	62	0.000178, 0.000212, 0.000251, 0.000298, 0.000354
	White	Men	63	0.000198, 0.000232, 0.000272, 0.000318, 0.000372
	White	Men	64	0.000221, 0.000255, 0.000294, 0.000339, 0.000391
	White	Men	65	0.000245, 0.00028, 0.000318, 0.000363, 0.000414
	White	Men	66	0.000271, 0.000307, 0.000348, 0.000394, 0.000446
	White	Men	67	0.000298, 0.000338, 0.000383, 0.000434, 0.000492
	White	Men	68	0.00033, 0.000375, 0.000426, 0.000485, 0.000552
	White	Men	69	0.000367, 0.000419, 0.000478, 0.000546, 0.000625
	White	Men	70	0.00041, 0.000471, 0.00054, 0.000618, 0.00071
	White	Men	71	0.000461, 0.00053, 0.00061, 0.000701, 0.000807
	White	Men	72	0.000517, 0.000597, 0.000689, 0.000794, 0.000918
	White	Men	73	0.000577, 0.00067, 0.000777, 0.0009, 0.00105
	White	Men	74	0.000644, 0.000752, 0.000876, 0.00102, 0.00119
	White	Men	75	0.00072, 0.000845, 0.00099, 0.00116, 0.00136
	White	Men	76	0.000807, 0.000953, 0.00112, 0.00132, 0.00156
	White	Men	77	0.000906, 0.00108, 0.00128, 0.00152, 0.00181
	White	Men	78	0.00102, 0.00122, 0.00146, 0.00176, 0.00211
	White	Men	79	0.00114, 0.00138, 0.00168, 0.00204, 0.00248
	White	Men	80	0.00126, 0.00156, 0.00192, 0.00237, 0.00293

Variable	Race/ethnicity	Sex	Age	Distribution
	White	Men	81	0.00141, 0.00176, 0.00219, 0.00273, 0.00341
	White	Men	82	0.00157, 0.00198, 0.00248, 0.00311, 0.0039
	White	Men	83	0.00173, 0.00219, 0.00277, 0.0035, 0.00443
	White	Men	84	0.00189, 0.00241, 0.00307, 0.0039, 0.00497
	White	Women	30	0.00000449, 0.00000662, 0.00000973, 0.0000143, 0.0000211
	White	Women	31	0.00000536, 0.00000773, 0.0000111, 0.000016, 0.000023
	White	Women	32	0.00000637, 0.00000899, 0.0000127, 0.0000178, 0.0000251
	White	Women	33	0.0000075, 0.0000104, 0.0000144, 0.0000198, 0.0000275
	White	Women	34	0.00000869, 0.0000119, 0.0000162, 0.000022, 0.00003
	White	Women	35	0.00000987, 0.0000134, 0.000018, 0.0000243, 0.0000328
	White	Women	36	0.0000111, 0.0000148, 0.0000198, 0.0000265, 0.0000356
	White	Women	37	0.0000123, 0.0000163, 0.0000216, 0.0000287, 0.0000382
	White	Women	38	0.0000135, 0.0000178, 0.0000234, 0.0000308, 0.0000406
	White	Women	39	0.0000147, 0.0000192, 0.0000251, 0.0000328, 0.0000429
	White	Women	40	0.0000161, 0.0000208, 0.0000268, 0.0000345, 0.0000446
	White	Women	41	0.0000177, 0.0000225, 0.0000284, 0.000036, 0.0000456
	White	Women	42	0.0000198, 0.0000245, 0.0000302, 0.0000372, 0.0000459
	White	Women	43	0.0000221, 0.0000267, 0.0000322, 0.0000388, 0.0000468
	White	Women	44	0.0000241, 0.0000289, 0.0000345, 0.0000411, 0.0000492
	White	Women	45	0.0000262, 0.0000312, 0.0000372, 0.0000443, 0.0000528
	White	Women	46	0.0000285, 0.000034, 0.0000404, 0.0000481, 0.0000573
	White	Women	47	0.0000312, 0.0000372, 0.0000443, 0.0000527, 0.0000629
	White	Women	48	0.0000341, 0.0000408, 0.0000488, 0.0000584, 0.00007
	White	Women	49	0.0000372, 0.0000449, 0.0000541, 0.0000653, 0.0000789
	White	Women	50	0.0000408, 0.0000496, 0.0000603, 0.0000732, 0.0000891
	White	Women	51	0.0000453, 0.0000552, 0.0000672, 0.0000817, 0.0000997
	White	Women	52	0.0000508, 0.0000617, 0.0000748, 0.0000906, 0.00011
	White	Women	53	0.0000572, 0.0000689, 0.0000829, 0.0000997, 0.00012
	White	Women	54	0.0000639, 0.0000765, 0.0000914, 0.000109, 0.000131
	White	Women	55	0.0000711, 0.0000844, 0.0001, 0.000119, 0.000141
	White	Women	56	0.0000788, 0.0000929, 0.000109, 0.000129, 0.000152
	White	Women	57	0.0000871, 0.000102, 0.000119, 0.000139, 0.000163
	White	Women	58	0.0000961, 0.000112, 0.00013, 0.000151, 0.000176
	White	Women	59	0.000106, 0.000123, 0.000142, 0.000164, 0.00019
	White	Women	60	0.000116, 0.000134, 0.000155, 0.000179, 0.000207
	White	Women	61	0.000127, 0.000147, 0.000169, 0.000195, 0.000225
	White	Women	62	0.000139, 0.00016, 0.000184, 0.000212, 0.000245
	White	Women	63	0.000152, 0.000174, 0.0002, 0.00023, 0.000265
	White	Women	64	0.000166, 0.00019, 0.000218, 0.00025, 0.000287
	White	Women	65	0.000182, 0.000208, 0.000238, 0.000272, 0.000311
	White	Women	66	0.000201, 0.000229, 0.000262, 0.000298, 0.000341
	White	Women	67	0.000222, 0.000254, 0.000291, 0.000332, 0.00038
	White	Women	68	0.000249, 0.000285, 0.000327, 0.000374, 0.000429

Variable	Race/ethnicity	Sex	Age	Distribution
	White	Women	69	0.000282, 0.000323, 0.000371, 0.000425, 0.000488
	White	Women	70	0.000323, 0.00037, 0.000424, 0.000486, 0.000557
	White	Women	71	0.000371, 0.000426, 0.000487, 0.000558, 0.00064
	White	Women	72	0.000426, 0.000489, 0.000561, 0.000643, 0.000738
	White	Women	73	0.000488, 0.000561, 0.000644, 0.00074, 0.000851
	White	Women	74	0.000559, 0.000644, 0.00074, 0.000851, 0.00098
	White	Women	75	0.000643, 0.00074, 0.00085, 0.000977, 0.00112
	White	Women	76	0.00074, 0.000852, 0.000979, 0.00113, 0.0013
	White	Women	77	0.000849, 0.000981, 0.00113, 0.00131, 0.00151
	White	Women	78	0.00097, 0.00113, 0.00131, 0.00153, 0.00178
	White	Women	79	0.00111, 0.0013, 0.00153, 0.00179, 0.00211
	White	Women	80	0.00127, 0.0015, 0.00178, 0.0021, 0.00249
	White	Women	81	0.00145, 0.00173, 0.00206, 0.00245, 0.00292
	White	Women	82	0.00165, 0.00198, 0.00237, 0.00283, 0.0034
	White	Women	83	0.00185, 0.00224, 0.00269, 0.00324, 0.00391
	White	Women	84	0.00206, 0.0025, 0.00303, 0.00367, 0.00445
Stroke mortality rates for 2032 (0.01, 0.2, 0.5, 0.8. 0.99 percentiles of the empirical distribution produced during forecasting)				
	Black	Men	30	0.0000135, 0.0000198, 0.000029, 0.0000423, 0.000062
	Black	Men	31	0.0000169, 0.000024, 0.0000339, 0.0000479, 0.000068
	Black	Men	32	0.0000206, 0.0000286, 0.0000396, 0.0000548, 0.000076
	Black	Men	33	0.0000244, 0.0000335, 0.0000458, 0.0000627, 0.0000859
	Black	Men	34	0.0000282, 0.0000385, 0.0000524, 0.0000714, 0.0000974
	Black	Men	35	0.000032, 0.0000436, 0.0000592, 0.0000804, 0.00011
	Black	Men	36	0.0000358, 0.0000486, 0.0000659, 0.0000892, 0.000121
	Black	Men	37	0.0000398, 0.0000537, 0.0000723, 0.0000973, 0.000131
	Black	Men	38	0.0000441, 0.0000589, 0.0000784, 0.000105, 0.00014
	Black	Men	39	0.0000485, 0.0000641, 0.0000844, 0.000111, 0.000147
	Black	Men	40	0.0000533, 0.0000695, 0.0000904, 0.000118, 0.000153
	Black	Men	41	0.0000592, 0.0000757, 0.0000966, 0.000123, 0.000158
	Black	Men	42	0.0000668, 0.0000833, 0.000104, 0.000129, 0.000161
	Black	Men	43	0.0000749, 0.0000917, 0.000112, 0.000137, 0.000167
	Black	Men	44	0.0000818, 0.0001, 0.000122, 0.000149, 0.000182
	Black	Men	45	0.0000888, 0.000109, 0.000134, 0.000164, 0.000202
	Black	Men	46	0.0000982, 0.000121, 0.000148, 0.000182, 0.000224
	Black	Men	47	0.00011, 0.000135, 0.000165, 0.000202, 0.000249
	Black	Men	48	0.000122, 0.00015, 0.000185, 0.000228, 0.000282
	Black	Men	49	0.000136, 0.000169, 0.00021, 0.00026, 0.000324
	Black	Men	50	0.000156, 0.000193, 0.000239, 0.000296, 0.000367
	Black	Men	51	0.000184, 0.000225, 0.000274, 0.000333, 0.000407
	Black	Men	52	0.000219, 0.000262, 0.000313, 0.000373, 0.000446
	Black	Men	53	0.000256, 0.000301, 0.000355, 0.000418, 0.000493
	Black	Men	54	0.000289, 0.00034, 0.000399, 0.000468, 0.000549
	Black	Men	55	0.000321, 0.000377, 0.000442, 0.000519, 0.000609

Variable	Race/ethnicity	Sex	Age	Distribution
	Black	Men	56	0.000352, 0.000414, 0.000486, 0.00057, 0.00067
	Black	Men	57	0.000382, 0.000449, 0.000528, 0.000621, 0.000731
	Black	Men	58	0.000414, 0.000486, 0.000571, 0.00067, 0.000787
	Black	Men	59	0.000447, 0.000524, 0.000613, 0.000718, 0.000842
	Black	Men	60	0.000475, 0.000558, 0.000655, 0.000769, 0.000904
	Black	Men	61	0.000498, 0.000589, 0.000695, 0.000821, 0.00097
	Black	Men	62	0.000523, 0.000619, 0.000732, 0.000866, 0.00103
	Black	Men	63	0.000555, 0.000653, 0.000768, 0.000902, 0.00106
	Black	Men	64	0.000593, 0.000691, 0.000804, 0.000935, 0.00109
	Black	Men	65	0.000628, 0.000728, 0.000843, 0.000977, 0.00113
	Black	Men	66	0.000661, 0.000769, 0.000892, 0.00103, 0.0012
	Black	Men	67	0.000702, 0.000818, 0.000951, 0.00111, 0.00129
	Black	Men	68	0.000756, 0.000881, 0.00102, 0.00119, 0.00139
	Black	Men	69	0.000822, 0.000955, 0.00111, 0.00129, 0.0015
	Black	Men	70	0.000894, 0.00104, 0.0012, 0.0014, 0.00162
	Black	Men	71	0.00097, 0.00113, 0.0013, 0.00151, 0.00176
	Black	Men	72	0.00105, 0.00122, 0.00141, 0.00164, 0.0019
	Black	Men	73	0.00113, 0.00131, 0.00152, 0.00177, 0.00206
	Black	Men	74	0.0012, 0.00141, 0.00164, 0.00192, 0.00224
	Black	Men	75	0.00129, 0.00152, 0.00178, 0.00209, 0.00245
	Black	Men	76	0.00139, 0.00164, 0.00193, 0.00227, 0.00268
	Black	Men	77	0.00151, 0.00178, 0.0021, 0.00248, 0.00293
	Black	Men	78	0.00164, 0.00194, 0.0023, 0.00272, 0.00322
	Black	Men	79	0.00178, 0.00211, 0.00251, 0.00298, 0.00354
	Black	Men	80	0.00191, 0.00228, 0.00273, 0.00326, 0.0039
	Black	Men	81	0.00204, 0.00245, 0.00295, 0.00354, 0.00426
	Black	Men	82	0.00217, 0.00262, 0.00316, 0.00382, 0.00461
	Black	Men	83	0.00229, 0.00278, 0.00337, 0.00408, 0.00496
	Black	Men	84	0.00241, 0.00294, 0.00357, 0.00434, 0.0053
	Black	Women	30	0.0000096, 0.0000151, 0.0000238, 0.0000374, 0.000059
	Black	Women	31	0.0000114, 0.0000177, 0.0000274, 0.0000425, 0.0000662
	Black	Women	32	0.0000132, 0.0000204, 0.0000315, 0.0000486, 0.0000752
	Black	Women	33	0.000015, 0.0000233, 0.000036, 0.0000555, 0.0000861
	Black	Women	34	0.0000169, 0.0000263, 0.0000408, 0.0000633, 0.0000987
	Black	Women	35	0.0000187, 0.0000293, 0.0000458, 0.0000714, 0.000112
	Black	Women	36	0.0000209, 0.0000327, 0.0000509, 0.0000792, 0.000124
	Black	Women	37	0.0000235, 0.0000364, 0.000056, 0.0000862, 0.000133
	Black	Women	38	0.0000266, 0.0000404, 0.0000612, 0.0000927, 0.000141
	Black	Women	39	0.0000298, 0.0000446, 0.0000665, 0.0000992, 0.000149
	Black	Women	40	0.0000328, 0.0000486, 0.0000719, 0.000106, 0.000157
	Black	Women	41	0.000036, 0.0000528, 0.0000773, 0.000113, 0.000166
	Black	Women	42	0.0000399, 0.0000576, 0.000083, 0.00012, 0.000173
	Black	Women	43	0.0000446, 0.0000632, 0.0000893, 0.000126, 0.000179

Variable	Race/ethnicity	Sex	Age	Distribution
	Black	Women	44	0.0000495, 0.0000692, 0.0000964, 0.000134, 0.000188
	Black	Women	45	0.0000549, 0.000076, 0.000105, 0.000144, 0.0002
	Black	Women	46	0.0000618, 0.0000842, 0.000114, 0.000156, 0.000212
	Black	Women	47	0.0000707, 0.0000945, 0.000126, 0.000168, 0.000224
	Black	Women	48	0.0000814, 0.000106, 0.000139, 0.000181, 0.000237
	Black	Women	49	0.0000925, 0.000119, 0.000154, 0.000198, 0.000256
	Black	Women	50	0.000104, 0.000133, 0.00017, 0.000218, 0.00028
	Black	Women	51	0.000116, 0.000148, 0.000188, 0.000239, 0.000306
	Black	Women	52	0.00013, 0.000164, 0.000207, 0.000261, 0.000329
	Black	Women	53	0.000145, 0.000181, 0.000226, 0.000283, 0.000354
	Black	Women	54	0.000158, 0.000198, 0.000246, 0.000307, 0.000384
	Black	Women	55	0.000171, 0.000214, 0.000268, 0.000334, 0.000418
	Black	Women	56	0.000186, 0.000233, 0.00029, 0.000362, 0.000453
	Black	Women	57	0.000205, 0.000254, 0.000315, 0.00039, 0.000483
	Black	Women	58	0.000228, 0.000279, 0.000341, 0.000416, 0.000509
	Black	Women	59	0.000254, 0.000306, 0.000369, 0.000444, 0.000535
	Black	Women	60	0.000278, 0.000332, 0.000397, 0.000474, 0.000566
	Black	Women	61	0.000298, 0.000356, 0.000424, 0.000505, 0.000603
	Black	Women	62	0.000315, 0.000376, 0.000449, 0.000537, 0.000642
	Black	Women	63	0.000331, 0.000396, 0.000473, 0.000566, 0.000677
	Black	Women	64	0.00035, 0.000417, 0.000497, 0.000592, 0.000707
	Black	Women	65	0.000372, 0.000442, 0.000523, 0.00062, 0.000737
	Black	Women	66	0.000399, 0.000471, 0.000555, 0.000654, 0.000773
	Black	Women	67	0.000432, 0.000508, 0.000595, 0.000698, 0.00082
	Black	Women	68	0.000474, 0.000554, 0.000646, 0.000754, 0.000881
	Black	Women	69	0.000525, 0.00061, 0.000708, 0.000822, 0.000956
	Black	Women	70	0.000584, 0.000676, 0.000783, 0.000906, 0.00105
	Black	Women	71	0.000649, 0.000751, 0.000869, 0.001, 0.00116
	Black	Women	72	0.00072, 0.000835, 0.000966, 0.00112, 0.0013
	Black	Women	73	0.000802, 0.000929, 0.00108, 0.00125, 0.00144
	Black	Women	74	0.000895, 0.00104, 0.0012, 0.00139, 0.00161
	Black	Women	75	0.001, 0.00116, 0.00134, 0.00155, 0.00179
	Black	Women	76	0.00111, 0.00129, 0.0015, 0.00174, 0.00202
	Black	Women	77	0.00122, 0.00144, 0.00168, 0.00197, 0.00231
	Black	Women	78	0.00134, 0.00159, 0.00189, 0.00224, 0.00267
	Black	Women	79	0.00147, 0.00177, 0.00212, 0.00255, 0.00306
	Black	Women	80	0.00162, 0.00196, 0.00238, 0.00287, 0.00348
	Black	Women	81	0.00178, 0.00217, 0.00265, 0.00322, 0.00392
	Black	Women	82	0.00195, 0.00239, 0.00292, 0.00358, 0.00439
	Black	Women	83	0.0021, 0.0026, 0.0032, 0.00395, 0.00488
	Black	Women	84	0.00225, 0.0028, 0.00348, 0.00432, 0.00538
	Hispanic	Men	30	0.00000623, 0.00000923, 0.0000136, 0.0000201, 0.0000299
	Hispanic	Men	31	0.00000763, 0.0000109, 0.0000155, 0.0000221, 0.0000316

Variable	Race/ethnicity	Sex	Age	Distribution
	Hispanic	Men	32	0.00000919, 0.0000128, 0.0000177, 0.0000244, 0.0000339
	Hispanic	Men	33	0.0000108, 0.0000148, 0.00002, 0.0000271, 0.0000369
	Hispanic	Men	34	0.0000125, 0.0000168, 0.0000226, 0.0000303, 0.0000408
	Hispanic	Men	35	0.0000141, 0.0000189, 0.0000252, 0.0000337, 0.0000452
	Hispanic	Men	36	0.0000157, 0.000021, 0.000028, 0.0000372, 0.0000497
	Hispanic	Men	37	0.0000175, 0.0000232, 0.0000307, 0.0000406, 0.0000538
	Hispanic	Men	38	0.0000194, 0.0000256, 0.0000335, 0.0000439, 0.0000577
	Hispanic	Men	39	0.0000214, 0.0000279, 0.0000363, 0.0000472, 0.0000616
	Hispanic	Men	40	0.0000235, 0.0000303, 0.0000391, 0.0000504, 0.0000651
	Hispanic	Men	41	0.0000259, 0.000033, 0.000042, 0.0000533, 0.0000679
	Hispanic	Men	42	0.0000289, 0.0000361, 0.000045, 0.0000561, 0.00007
	Hispanic	Men	43	0.0000321, 0.0000395, 0.0000484, 0.0000593, 0.0000729
	Hispanic	Men	44	0.0000351, 0.0000429, 0.0000523, 0.0000637, 0.0000778
	Hispanic	Men	45	0.0000384, 0.0000467, 0.0000568, 0.0000691, 0.0000842
	Hispanic	Men	46	0.0000424, 0.0000514, 0.0000622, 0.0000753, 0.0000914
	Hispanic	Men	47	0.0000474, 0.0000571, 0.0000687, 0.0000827, 0.0000996
	Hispanic	Men	48	0.0000534, 0.000064, 0.0000765, 0.0000915, 0.00011
	Hispanic	Men	49	0.0000607, 0.0000722, 0.0000858, 0.000102, 0.000121
	Hispanic	Men	50	0.0000699, 0.0000823, 0.0000968, 0.000114, 0.000134
	Hispanic	Men	51	0.000081, 0.0000943, 0.00011, 0.000127, 0.000148
	Hispanic	Men	52	0.0000932, 0.000108, 0.000124, 0.000143, 0.000165
	Hispanic	Men	53	0.000104, 0.000121, 0.00014, 0.000162, 0.000188
	Hispanic	Men	54	0.000113, 0.000133, 0.000156, 0.000184, 0.000217
	Hispanic	Men	55	0.000121, 0.000145, 0.000174, 0.000207, 0.000248
	Hispanic	Men	56	0.000131, 0.000159, 0.000191, 0.00023, 0.000278
	Hispanic	Men	57	0.000143, 0.000173, 0.000209, 0.000253, 0.000306
	Hispanic	Men	58	0.000157, 0.000189, 0.000228, 0.000274, 0.00033
	Hispanic	Men	59	0.000172, 0.000206, 0.000246, 0.000294, 0.000353
	Hispanic	Men	60	0.000189, 0.000224, 0.000265, 0.000314, 0.000373
	Hispanic	Men	61	0.000205, 0.000242, 0.000284, 0.000334, 0.000394
	Hispanic	Men	62	0.000221, 0.000259, 0.000303, 0.000355, 0.000416
	Hispanic	Men	63	0.000236, 0.000276, 0.000322, 0.000376, 0.00044
	Hispanic	Men	64	0.000251, 0.000293, 0.000342, 0.000399, 0.000466
	Hispanic	Men	65	0.000268, 0.000313, 0.000365, 0.000426, 0.000498
	Hispanic	Men	66	0.000286, 0.000336, 0.000392, 0.000459, 0.000537
	Hispanic	Men	67	0.000308, 0.000362, 0.000426, 0.0005, 0.000588
	Hispanic	Men	68	0.000334, 0.000395, 0.000467, 0.000551, 0.000651
	Hispanic	Men	69	0.000367, 0.000435, 0.000515, 0.00061, 0.000724
	Hispanic	Men	70	0.000407, 0.000483, 0.000572, 0.000678, 0.000805
	Hispanic	Men	71	0.000454, 0.000538, 0.000637, 0.000754, 0.000893
	Hispanic	Men	72	0.000508, 0.000601, 0.00071, 0.000838, 0.000992
	Hispanic	Men	73	0.000567, 0.00067, 0.000791, 0.000933, 0.0011
	Hispanic	Men	74	0.000632, 0.000747, 0.000881, 0.00104, 0.00123

Variable	Race/ethnicity	Sex	Age	Distribution
	Hispanic	Men	75	0.000705, 0.000834, 0.000984, 0.00116, 0.00137
	Hispanic	Men	76	0.000788, 0.000932, 0.0011, 0.0013, 0.00154
	Hispanic	Men	77	0.00088, 0.00104, 0.00124, 0.00146, 0.00173
	Hispanic	Men	78	0.00098, 0.00117, 0.00139, 0.00165, 0.00197
	Hispanic	Men	79	0.00109, 0.0013, 0.00156, 0.00186, 0.00223
	Hispanic	Men	80	0.0012, 0.00145, 0.00174, 0.0021, 0.00254
	Hispanic	Men	81	0.00131, 0.0016, 0.00194, 0.00236, 0.00287
	Hispanic	Men	82	0.00142, 0.00175, 0.00214, 0.00263, 0.00323
	Hispanic	Men	83	0.00153, 0.0019, 0.00235, 0.00291, 0.00361
	Hispanic	Men	84	0.00163, 0.00204, 0.00255, 0.00318, 0.00398
	Hispanic	Women	30	0.00000363, 0.00000585, 0.00000937, 0.000015, 0.0000242
	Hispanic	Women	31	0.00000438, 0.00000682, 0.0000106, 0.0000164, 0.0000255
	Hispanic	Women	32	0.00000521, 0.00000792, 0.000012, 0.0000181, 0.0000276
	Hispanic	Women	33	0.00000611, 0.00000913, 0.0000136, 0.0000202, 0.0000303
	Hispanic	Women	34	0.00000708, 0.0000105, 0.0000154, 0.0000226, 0.0000333
	Hispanic	Women	35	0.00000817, 0.0000119, 0.0000173, 0.000025, 0.0000364
	Hispanic	Women	36	0.00000938, 0.0000134, 0.0000192, 0.0000274, 0.0000393
	Hispanic	Women	37	0.0000107, 0.0000151, 0.0000212, 0.0000297, 0.0000419
	Hispanic	Women	38	0.0000121, 0.0000168, 0.0000232, 0.000032, 0.0000443
	Hispanic	Women	39	0.0000135, 0.0000185, 0.0000251, 0.0000342, 0.0000467
	Hispanic	Women	40	0.000015, 0.0000202, 0.0000271, 0.0000364, 0.000049
	Hispanic	Women	41	0.0000167, 0.0000221, 0.0000291, 0.0000385, 0.0000509
	Hispanic	Women	42	0.0000185, 0.0000241, 0.0000313, 0.0000406, 0.0000529
	Hispanic	Women	43	0.0000204, 0.0000262, 0.0000337, 0.0000433, 0.0000557
	Hispanic	Women	44	0.0000223, 0.0000286, 0.0000365, 0.0000465, 0.0000595
	Hispanic	Women	45	0.0000249, 0.0000315, 0.0000397, 0.0000501, 0.0000633
	Hispanic	Women	46	0.0000283, 0.0000351, 0.0000434, 0.0000537, 0.0000666
	Hispanic	Women	47	0.0000325, 0.0000394, 0.0000478, 0.0000579, 0.0000703
	Hispanic	Women	48	0.0000369, 0.0000442, 0.0000528, 0.0000631, 0.0000755
	Hispanic	Women	49	0.0000412, 0.0000492, 0.0000586, 0.0000699, 0.0000835
	Hispanic	Women	50	0.0000453, 0.0000545, 0.0000654, 0.0000784, 0.0000943
	Hispanic	Women	51	0.0000496, 0.0000603, 0.000073, 0.0000886, 0.000108
	Hispanic	Women	52	0.0000542, 0.0000665, 0.0000816, 0.0001, 0.000123
	Hispanic	Women	53	0.0000589, 0.0000733, 0.000091, 0.000113, 0.000141
	Hispanic	Women	54	0.000064, 0.0000805, 0.000101, 0.000127, 0.000159
	Hispanic	Women	55	0.0000701, 0.0000885, 0.000111, 0.00014, 0.000177
	Hispanic	Women	56	0.0000773, 0.0000974, 0.000122, 0.000154, 0.000194
	Hispanic	Women	57	0.0000853, 0.000107, 0.000134, 0.000168, 0.000211
	Hispanic	Women	58	0.0000937, 0.000117, 0.000146, 0.000183, 0.000229
	Hispanic	Women	59	0.000102, 0.000128, 0.00016, 0.000199, 0.000248
	Hispanic	Women	60	0.000111, 0.000139, 0.000173, 0.000216, 0.000269
	Hispanic	Women	61	0.000121, 0.000151, 0.000187, 0.000232, 0.000289
	Hispanic	Women	62	0.000131, 0.000162, 0.000201, 0.000249, 0.000309

Variable	Race/ethnicity	Sex	Age	Distribution
	Hispanic	Women	63	0.00014, 0.000174, 0.000215, 0.000266, 0.000329
	Hispanic	Women	64	0.00015, 0.000186, 0.00023, 0.000284, 0.000352
	Hispanic	Women	65	0.000161, 0.000199, 0.000247, 0.000305, 0.000378
	Hispanic	Women	66	0.000173, 0.000215, 0.000267, 0.00033, 0.00041
	Hispanic	Women	67	0.000189, 0.000235, 0.000292, 0.000362, 0.000451
	Hispanic	Women	68	0.000208, 0.00026, 0.000323, 0.000402, 0.000501
	Hispanic	Women	69	0.000233, 0.00029, 0.000361, 0.000449, 0.000559
	Hispanic	Women	70	0.000266, 0.000329, 0.000407, 0.000503, 0.000623
	Hispanic	Women	71	0.000307, 0.000376, 0.00046, 0.000563, 0.000691
	Hispanic	Women	72	0.000357, 0.000432, 0.000522, 0.00063, 0.000763
	Hispanic	Women	73	0.000416, 0.000497, 0.000592, 0.000706, 0.000843
	Hispanic	Women	74	0.000483, 0.000571, 0.000673, 0.000794, 0.000938
	Hispanic	Women	75	0.000558, 0.000655, 0.000767, 0.0009, 0.00106
	Hispanic	Women	76	0.00064, 0.00075, 0.000878, 0.00103, 0.0012
	Hispanic	Women	77	0.000733, 0.000859, 0.00101, 0.00118, 0.00138
	Hispanic	Women	78	0.000841, 0.000987, 0.00116, 0.00136, 0.00159
	Hispanic	Women	79	0.000967, 0.00113, 0.00133, 0.00156, 0.00183
	Hispanic	Women	80	0.00111, 0.0013, 0.00152, 0.00178, 0.0021
	Hispanic	Women	81	0.00124, 0.00147, 0.00173, 0.00204, 0.00241
	Hispanic	Women	82	0.00136, 0.00163, 0.00195, 0.00233, 0.0028
	Hispanic	Women	83	0.00146, 0.00179, 0.00218, 0.00266, 0.00324
	Hispanic	Women	84	0.00156, 0.00194, 0.00241, 0.00299, 0.00372
	White	Men	30	0.00000491, 0.00000715, 0.0000104, 0.0000151, 0.000022
	White	Men	31	0.00000588, 0.00000832, 0.0000117, 0.0000166, 0.0000235
	White	Men	32	0.00000696, 0.00000962, 0.0000133, 0.0000183, 0.0000252
	White	Men	33	0.00000815, 0.000011, 0.0000149, 0.0000201, 0.0000273
	White	Men	34	0.00000937, 0.0000125, 0.0000167, 0.0000222, 0.0000297
	White	Men	35	0.0000106, 0.000014, 0.0000185, 0.0000245, 0.0000324
	White	Men	36	0.0000117, 0.0000155, 0.0000204, 0.0000268, 0.0000354
	White	Men	37	0.0000129, 0.000017, 0.0000223, 0.0000292, 0.0000384
	White	Men	38	0.0000141, 0.0000185, 0.0000242, 0.0000316, 0.0000415
	White	Men	39	0.0000153, 0.00002, 0.0000261, 0.000034, 0.0000445
	White	Men	40	0.0000166, 0.0000216, 0.000028, 0.0000362, 0.000047
	White	Men	41	0.0000184, 0.0000235, 0.0000299, 0.0000382, 0.0000487
	White	Men	42	0.0000207, 0.0000258, 0.0000321, 0.0000399, 0.0000498
	White	Men	43	0.0000232, 0.0000283, 0.0000345, 0.000042, 0.0000512
	White	Men	44	0.0000258, 0.0000311, 0.0000374, 0.0000449, 0.0000541
	White	Men	45	0.0000287, 0.0000342, 0.0000408, 0.0000487, 0.0000581
	White	Men	46	0.0000321, 0.000038, 0.000045, 0.0000531, 0.0000629
	White	Men	47	0.0000361, 0.0000425, 0.0000499, 0.0000586, 0.0000689
	White	Men	48	0.0000404, 0.0000475, 0.0000558, 0.0000656, 0.0000771
	White	Men	49	0.0000449, 0.0000532, 0.0000628, 0.0000743, 0.000088
	White	Men	50	0.00005, 0.0000597, 0.0000711, 0.0000847, 0.000101

Variable	Race/ethnicity	Sex	Age	Distribution
	White	Men	51	0.0000564, 0.0000675, 0.0000807, 0.0000964, 0.000115
	White	Men	52	0.0000644, 0.0000768, 0.0000915, 0.000109, 0.00013
	White	Men	53	0.0000734, 0.0000871, 0.000103, 0.000122, 0.000145
	White	Men	54	0.0000826, 0.0000979, 0.000116, 0.000137, 0.000163
	White	Men	55	0.0000916, 0.000109, 0.000129, 0.000153, 0.000182
	White	Men	56	0.000101, 0.00012, 0.000143, 0.000171, 0.000204
	White	Men	57	0.000109, 0.000132, 0.000158, 0.00019, 0.000228
	White	Men	58	0.000119, 0.000144, 0.000174, 0.00021, 0.000255
	White	Men	59	0.000129, 0.000157, 0.000191, 0.000232, 0.000282
	White	Men	60	0.000142, 0.000172, 0.000209, 0.000253, 0.000308
	White	Men	61	0.000157, 0.000189, 0.000227, 0.000274, 0.000331
	White	Men	62	0.000174, 0.000207, 0.000247, 0.000294, 0.00035
	White	Men	63	0.000193, 0.000227, 0.000267, 0.000313, 0.000368
	White	Men	64	0.000215, 0.000249, 0.000288, 0.000333, 0.000385
	White	Men	65	0.000238, 0.000273, 0.000312, 0.000356, 0.000407
	White	Men	66	0.000263, 0.000299, 0.00034, 0.000386, 0.000439
	White	Men	67	0.00029, 0.00033, 0.000374, 0.000425, 0.000484
	White	Men	68	0.00032, 0.000365, 0.000417, 0.000475, 0.000542
	White	Men	69	0.000356, 0.000408, 0.000467, 0.000535, 0.000614
	White	Men	70	0.000398, 0.000458, 0.000527, 0.000606, 0.000698
	White	Men	71	0.000447, 0.000516, 0.000595, 0.000687, 0.000793
	White	Men	72	0.000501, 0.000581, 0.000672, 0.000778, 0.000902
	White	Men	73	0.00056, 0.000652, 0.000758, 0.000882, 0.00103
	White	Men	74	0.000625, 0.000731, 0.000855, 0.001, 0.00117
	White	Men	75	0.000698, 0.000822, 0.000966, 0.00114, 0.00134
	White	Men	76	0.000783, 0.000927, 0.0011, 0.0013, 0.00153
	White	Men	77	0.000879, 0.00105, 0.00125, 0.00149, 0.00177
	White	Men	78	0.000986, 0.00119, 0.00143, 0.00172, 0.00207
	White	Men	79	0.0011, 0.00134, 0.00164, 0.002, 0.00244
	White	Men	80	0.00123, 0.00152, 0.00188, 0.00232, 0.00287
	White	Men	81	0.00137, 0.00171, 0.00214, 0.00267, 0.00335
	White	Men	82	0.00152, 0.00192, 0.00242, 0.00304, 0.00383
	White	Men	83	0.00168, 0.00213, 0.0027, 0.00342, 0.00435
	White	Men	84	0.00183, 0.00234, 0.00299, 0.00382, 0.00488
	White	Women	30	0.0000044, 0.00000654, 0.00000969, 0.0000144, 0.0000213
	White	Women	31	0.00000527, 0.00000765, 0.0000111, 0.000016, 0.0000232
	White	Women	32	0.00000627, 0.00000891, 0.0000126, 0.0000178, 0.0000253
	White	Women	33	0.0000074, 0.0000103, 0.0000143, 0.0000199, 0.0000277
	White	Women	34	0.00000857, 0.0000118, 0.0000161, 0.000022, 0.0000303
	White	Women	35	0.00000973, 0.0000132, 0.0000179, 0.0000243, 0.000033
	White	Women	36	0.0000109, 0.0000147, 0.0000197, 0.0000265, 0.0000358
	White	Women	37	0.000012, 0.0000161, 0.0000215, 0.0000287, 0.0000383
	White	Women	38	0.0000132, 0.0000175, 0.0000232, 0.0000307, 0.0000407

Variable	Race/ethnicity	Sex	Age	Distribution
	White	Women	39	0.0000144, 0.0000189, 0.0000248, 0.0000326, 0.0000429
	White	Women	40	0.0000157, 0.0000204, 0.0000264, 0.0000343, 0.0000445
	White	Women	41	0.0000173, 0.000022, 0.000028, 0.0000356, 0.0000453
	White	Women	42	0.0000193, 0.000024, 0.0000297, 0.0000367, 0.0000455
	White	Women	43	0.0000215, 0.0000261, 0.0000315, 0.0000381, 0.0000462
	White	Women	44	0.0000235, 0.0000282, 0.0000337, 0.0000404, 0.0000484
	White	Women	45	0.0000254, 0.0000304, 0.0000364, 0.0000434, 0.000052
	White	Women	46	0.0000277, 0.0000331, 0.0000395, 0.0000471, 0.0000563
	White	Women	47	0.0000304, 0.0000363, 0.0000433, 0.0000516, 0.0000617
	White	Women	48	0.0000332, 0.0000398, 0.0000477, 0.0000572, 0.0000686
	White	Women	49	0.0000362, 0.0000438, 0.000053, 0.000064, 0.0000774
	White	Women	50	0.0000398, 0.0000485, 0.000059, 0.0000718, 0.0000875
	White	Women	51	0.0000443, 0.000054, 0.0000658, 0.0000802, 0.0000979
	White	Women	52	0.0000498, 0.0000605, 0.0000734, 0.000089, 0.000108
	White	Women	53	0.000056, 0.0000676, 0.0000814, 0.0000981, 0.000118
	White	Women	54	0.0000627, 0.0000751, 0.0000899, 0.000108, 0.000129
	White	Women	55	0.0000697, 0.000083, 0.0000986, 0.000117, 0.000139
	White	Women	56	0.0000773, 0.0000913, 0.000108, 0.000127, 0.00015
	White	Women	57	0.0000855, 0.0001, 0.000117, 0.000137, 0.000161
	White	Women	58	0.0000942, 0.00011, 0.000128, 0.000149, 0.000174
	White	Women	59	0.000104, 0.00012, 0.00014, 0.000162, 0.000188
	White	Women	60	0.000114, 0.000132, 0.000153, 0.000177, 0.000205
	White	Women	61	0.000124, 0.000144, 0.000166, 0.000192, 0.000223
	White	Women	62	0.000136, 0.000157, 0.000181, 0.000209, 0.000242
	White	Women	63	0.000148, 0.000171, 0.000197, 0.000227, 0.000262
	White	Women	64	0.000162, 0.000186, 0.000214, 0.000246, 0.000283
	White	Women	65	0.000177, 0.000203, 0.000233, 0.000267, 0.000306
	White	Women	66	0.000195, 0.000224, 0.000256, 0.000293, 0.000335
	White	Women	67	0.000216, 0.000248, 0.000284, 0.000326, 0.000374
	White	Women	68	0.000241, 0.000278, 0.000319, 0.000367, 0.000422
	White	Women	69	0.000273, 0.000315, 0.000362, 0.000416, 0.00048
	White	Women	70	0.000313, 0.00036, 0.000414, 0.000476, 0.000548
	White	Women	71	0.00036, 0.000414, 0.000476, 0.000547, 0.000629
	White	Women	72	0.000413, 0.000476, 0.000547, 0.000629, 0.000725
	White	Women	73	0.000473, 0.000546, 0.000629, 0.000724, 0.000836
	White	Women	74	0.000542, 0.000626, 0.000722, 0.000832, 0.000961
	White	Women	75	0.000623, 0.000719, 0.000829, 0.000956, 0.0011
	White	Women	76	0.000717, 0.000828, 0.000955, 0.0011, 0.00127
	White	Women	77	0.000822, 0.000953, 0.0011, 0.00128, 0.00148
	White	Women	78	0.00094, 0.0011, 0.00128, 0.00149, 0.00174
	White	Women	79	0.00107, 0.00127, 0.00149, 0.00175, 0.00207
	White	Women	80	0.00123, 0.00146, 0.00173, 0.00205, 0.00244
	White	Women	81	0.0014, 0.00168, 0.00201, 0.0024, 0.00287

Variable	Race/ethnicity	Sex	Age	Distribution
	White	Women	82	0.0016, 0.00192, 0.00231, 0.00277, 0.00333
	White	Women	83	0.00179, 0.00217, 0.00262, 0.00317, 0.00384
	White	Women	84	0.002, 0.00243, 0.00295, 0.00359, 0.00437
Stroke mortality rates for 2033 (0.01, 0.2, 0.5, 0.8. 0.99 percentiles of the empirical distribution produced during forecasting)				
	Black	Men	30	0.0000133, 0.0000196, 0.0000288, 0.0000424, 0.0000627
	Black	Men	31	0.0000167, 0.0000238, 0.0000338, 0.0000481, 0.0000686
	Black	Men	32	0.0000203, 0.0000284, 0.0000394, 0.0000549, 0.0000766
	Black	Men	33	0.0000241, 0.0000332, 0.0000457, 0.0000628, 0.0000866
	Black	Men	34	0.0000279, 0.0000382, 0.0000523, 0.0000715, 0.000098
	Black	Men	35	0.0000315, 0.0000432, 0.0000589, 0.0000805, 0.00011
	Black	Men	36	0.0000352, 0.0000481, 0.0000655, 0.0000892, 0.000122
	Black	Men	37	0.0000391, 0.0000531, 0.0000718, 0.0000971, 0.000132
	Black	Men	38	0.0000432, 0.000058, 0.0000777, 0.000104, 0.00014
	Black	Men	39	0.0000474, 0.000063, 0.0000835, 0.000111, 0.000147
	Black	Men	40	0.000052, 0.0000682, 0.0000891, 0.000117, 0.000153
	Black	Men	41	0.0000577, 0.0000742, 0.0000951, 0.000122, 0.000157
	Black	Men	42	0.0000651, 0.0000815, 0.000102, 0.000127, 0.000159
	Black	Men	43	0.000073, 0.0000896, 0.00011, 0.000134, 0.000165
	Black	Men	44	0.0000797, 0.0000976, 0.000119, 0.000146, 0.000179
	Black	Men	45	0.0000864, 0.000107, 0.000131, 0.000161, 0.000198
	Black	Men	46	0.0000955, 0.000118, 0.000145, 0.000178, 0.000219
	Black	Men	47	0.000107, 0.000131, 0.000161, 0.000198, 0.000244
	Black	Men	48	0.000119, 0.000147, 0.000181, 0.000224, 0.000277
	Black	Men	49	0.000133, 0.000165, 0.000205, 0.000255, 0.000318
	Black	Men	50	0.000152, 0.000189, 0.000234, 0.00029, 0.00036
	Black	Men	51	0.00018, 0.00022, 0.000268, 0.000327, 0.0004
	Black	Men	52	0.000215, 0.000257, 0.000307, 0.000367, 0.000439
	Black	Men	53	0.000251, 0.000296, 0.000349, 0.000411, 0.000486
	Black	Men	54	0.000283, 0.000334, 0.000392, 0.000461, 0.000543
	Black	Men	55	0.000315, 0.000371, 0.000436, 0.000512, 0.000603
	Black	Men	56	0.000345, 0.000407, 0.000478, 0.000563, 0.000663
	Black	Men	57	0.000375, 0.000442, 0.000521, 0.000613, 0.000723
	Black	Men	58	0.000406, 0.000478, 0.000562, 0.000662, 0.00078
	Black	Men	59	0.000438, 0.000515, 0.000604, 0.000709, 0.000834
	Black	Men	60	0.000465, 0.000548, 0.000645, 0.000759, 0.000895
	Black	Men	61	0.000487, 0.000578, 0.000684, 0.00081, 0.00096
	Black	Men	62	0.000511, 0.000607, 0.00072, 0.000854, 0.00101
	Black	Men	63	0.000542, 0.00064, 0.000754, 0.000888, 0.00105
	Black	Men	64	0.000578, 0.000675, 0.000788, 0.000919, 0.00107
	Black	Men	65	0.000612, 0.000711, 0.000826, 0.000959, 0.00111
	Black	Men	66	0.000644, 0.00075, 0.000872, 0.00101, 0.00118
	Black	Men	67	0.000683, 0.000797, 0.00093, 0.00108, 0.00127
	Black	Men	68	0.000735, 0.000858, 0.001, 0.00117, 0.00136

Variable	Race/ethnicity	Sex	Age	Distribution
	Black	Men	69	0.000798, 0.00093, 0.00108, 0.00126, 0.00147
	Black	Men	70	0.000867, 0.00101, 0.00118, 0.00137, 0.00159
	Black	Men	71	0.000941, 0.0011, 0.00127, 0.00148, 0.00172
	Black	Men	72	0.00102, 0.00118, 0.00138, 0.0016, 0.00187
	Black	Men	73	0.00109, 0.00128, 0.00149, 0.00173, 0.00202
	Black	Men	74	0.00117, 0.00137, 0.0016, 0.00188, 0.0022
	Black	Men	75	0.00125, 0.00147, 0.00173, 0.00204, 0.00241
	Black	Men	76	0.00135, 0.00159, 0.00188, 0.00222, 0.00263
	Black	Men	77	0.00146, 0.00173, 0.00205, 0.00243, 0.00287
	Black	Men	78	0.00159, 0.00189, 0.00224, 0.00266, 0.00315
	Black	Men	79	0.00172, 0.00205, 0.00245, 0.00291, 0.00348
	Black	Men	80	0.00185, 0.00222, 0.00266, 0.00319, 0.00383
	Black	Men	81	0.00198, 0.00239, 0.00287, 0.00346, 0.00418
	Black	Men	82	0.0021, 0.00255, 0.00308, 0.00373, 0.00453
	Black	Men	83	0.00222, 0.0027, 0.00329, 0.00399, 0.00487
	Black	Men	84	0.00233, 0.00285, 0.00348, 0.00425, 0.0052
	Black	Women	30	0.00000944, 0.000015, 0.0000237, 0.0000375, 0.0000596
	Black	Women	31	0.0000112, 0.0000176, 0.0000274, 0.0000427, 0.0000669
	Black	Women	32	0.000013, 0.0000203, 0.0000315, 0.0000487, 0.0000759
	Black	Women	33	0.0000149, 0.0000232, 0.0000359, 0.0000557, 0.0000867
	Black	Women	34	0.0000167, 0.0000261, 0.0000407, 0.0000634, 0.0000993
	Black	Women	35	0.0000185, 0.0000291, 0.0000457, 0.0000715, 0.000113
	Black	Women	36	0.0000206, 0.0000324, 0.0000507, 0.0000792, 0.000124
	Black	Women	37	0.0000232, 0.000036, 0.0000557, 0.0000862, 0.000134
	Black	Women	38	0.0000262, 0.00004, 0.0000608, 0.0000925, 0.000141
	Black	Women	39	0.0000292, 0.0000439, 0.0000659, 0.0000989, 0.000149
	Black	Women	40	0.0000321, 0.0000478, 0.0000711, 0.000106, 0.000157
	Black	Women	41	0.0000351, 0.0000518, 0.0000763, 0.000112, 0.000166
	Black	Women	42	0.0000389, 0.0000565, 0.0000818, 0.000118, 0.000172
	Black	Women	43	0.0000434, 0.0000618, 0.0000878, 0.000125, 0.000178
	Black	Women	44	0.0000482, 0.0000676, 0.0000947, 0.000132, 0.000186
	Black	Women	45	0.0000534, 0.0000742, 0.000103, 0.000142, 0.000198
	Black	Women	46	0.00006, 0.0000822, 0.000112, 0.000153, 0.00021
	Black	Women	47	0.0000688, 0.0000922, 0.000123, 0.000165, 0.000221
	Black	Women	48	0.0000792, 0.000104, 0.000136, 0.000178, 0.000234
	Black	Women	49	0.0000901, 0.000117, 0.000151, 0.000195, 0.000253
	Black	Women	50	0.000101, 0.00013, 0.000167, 0.000215, 0.000277
	Black	Women	51	0.000113, 0.000145, 0.000185, 0.000236, 0.000303
	Black	Women	52	0.000127, 0.000161, 0.000204, 0.000258, 0.000327
	Black	Women	53	0.000142, 0.000178, 0.000223, 0.00028, 0.000351
	Black	Women	54	0.000155, 0.000194, 0.000243, 0.000304, 0.000381
	Black	Women	55	0.000168, 0.000211, 0.000264, 0.000331, 0.000416
	Black	Women	56	0.000182, 0.000229, 0.000287, 0.000359, 0.000451

Variable	Race/ethnicity	Sex	Age	Distribution
	Black	Women	57	0.000201, 0.00025, 0.000311, 0.000386, 0.00048
	Black	Women	58	0.000224, 0.000275, 0.000336, 0.000412, 0.000506
	Black	Women	59	0.000249, 0.000301, 0.000364, 0.000439, 0.000531
	Black	Women	60	0.000272, 0.000327, 0.000391, 0.000468, 0.000562
	Black	Women	61	0.000291, 0.000349, 0.000417, 0.000499, 0.000598
	Black	Women	62	0.000308, 0.000369, 0.000442, 0.000529, 0.000635
	Black	Women	63	0.000323, 0.000388, 0.000465, 0.000557, 0.000668
	Black	Women	64	0.000341, 0.000408, 0.000488, 0.000583, 0.000697
	Black	Women	65	0.000362, 0.000431, 0.000513, 0.000609, 0.000725
	Black	Women	66	0.000388, 0.00046, 0.000543, 0.000642, 0.00076
	Black	Women	67	0.00042, 0.000495, 0.000582, 0.000685, 0.000806
	Black	Women	68	0.00046, 0.00054, 0.000631, 0.000739, 0.000866
	Black	Women	69	0.00051, 0.000594, 0.000692, 0.000806, 0.00094
	Black	Women	70	0.000567, 0.000659, 0.000765, 0.000888, 0.00103
	Black	Women	71	0.00063, 0.000732, 0.000849, 0.000984, 0.00114
	Black	Women	72	0.000699, 0.000813, 0.000944, 0.0011, 0.00127
	Black	Women	73	0.000778, 0.000904, 0.00105, 0.00122, 0.00142
	Black	Women	74	0.000868, 0.00101, 0.00117, 0.00136, 0.00158
	Black	Women	75	0.00097, 0.00113, 0.00131, 0.00151, 0.00176
	Black	Women	76	0.00108, 0.00125, 0.00146, 0.0017, 0.00198
	Black	Women	77	0.00119, 0.00139, 0.00164, 0.00193, 0.00227
	Black	Women	78	0.0013, 0.00155, 0.00184, 0.00219, 0.00261
	Black	Women	79	0.00143, 0.00172, 0.00207, 0.00249, 0.003
	Black	Women	80	0.00157, 0.00191, 0.00232, 0.00281, 0.00342
	Black	Women	81	0.00173, 0.00211, 0.00258, 0.00315, 0.00385
	Black	Women	82	0.00188, 0.00232, 0.00285, 0.0035, 0.0043
	Black	Women	83	0.00203, 0.00252, 0.00312, 0.00386, 0.00478
	Black	Women	84	0.00218, 0.00272, 0.00339, 0.00422, 0.00527
	Hispanic	Men	30	0.00000611, 0.00000913, 0.0000136, 0.0000202, 0.0000302
	Hispanic	Men	31	0.0000075, 0.0000108, 0.0000155, 0.0000222, 0.0000319
	Hispanic	Men	32	0.00000905, 0.0000126, 0.0000176, 0.0000245, 0.0000342
	Hispanic	Men	33	0.0000107, 0.0000146, 0.0000199, 0.0000272, 0.0000372
	Hispanic	Men	34	0.0000123, 0.0000166, 0.0000225, 0.0000303, 0.0000411
	Hispanic	Men	35	0.0000139, 0.0000187, 0.0000251, 0.0000338, 0.0000455
	Hispanic	Men	36	0.0000155, 0.0000208, 0.0000278, 0.0000372, 0.00005
	Hispanic	Men	37	0.0000172, 0.000023, 0.0000305, 0.0000406, 0.0000541
	Hispanic	Men	38	0.000019, 0.0000252, 0.0000332, 0.0000438, 0.0000579
	Hispanic	Men	39	0.0000209, 0.0000274, 0.0000359, 0.000047, 0.0000616
	Hispanic	Men	40	0.0000229, 0.0000298, 0.0000386, 0.00005, 0.000065
	Hispanic	Men	41	0.0000253, 0.0000323, 0.0000413, 0.0000527, 0.0000675
	Hispanic	Men	42	0.0000282, 0.0000353, 0.0000442, 0.0000553, 0.0000693
	Hispanic	Men	43	0.0000313, 0.0000386, 0.0000474, 0.0000583, 0.0000719
	Hispanic	Men	44	0.0000342, 0.0000419, 0.0000512, 0.0000625, 0.0000765

Variable	Race/ethnicity	Sex	Age	Distribution
	Hispanic	Men	45	0.0000373, 0.0000456, 0.0000556, 0.0000677, 0.0000827
	Hispanic	Men	46	0.0000412, 0.0000501, 0.0000608, 0.0000738, 0.0000897
	Hispanic	Men	47	0.0000461, 0.0000557, 0.0000671, 0.0000809, 0.0000977
	Hispanic	Men	48	0.000052, 0.0000624, 0.0000747, 0.0000895, 0.000107
	Hispanic	Men	49	0.0000592, 0.0000705, 0.0000839, 0.0000998, 0.000119
	Hispanic	Men	50	0.0000682, 0.0000804, 0.0000948, 0.000112, 0.000132
	Hispanic	Men	51	0.0000792, 0.0000923, 0.000107, 0.000125, 0.000146
	Hispanic	Men	52	0.0000912, 0.000105, 0.000122, 0.00014, 0.000162
	Hispanic	Men	53	0.000102, 0.000118, 0.000137, 0.000159, 0.000185
	Hispanic	Men	54	0.000111, 0.000131, 0.000154, 0.000181, 0.000214
	Hispanic	Men	55	0.000119, 0.000143, 0.000171, 0.000204, 0.000245
	Hispanic	Men	56	0.000129, 0.000156, 0.000188, 0.000227, 0.000275
	Hispanic	Men	57	0.00014, 0.00017, 0.000206, 0.000249, 0.000302
	Hispanic	Men	58	0.000154, 0.000186, 0.000224, 0.000271, 0.000327
	Hispanic	Men	59	0.000169, 0.000203, 0.000243, 0.000291, 0.000349
	Hispanic	Men	60	0.000185, 0.00022, 0.000261, 0.00031, 0.00037
	Hispanic	Men	61	0.000201, 0.000237, 0.00028, 0.00033, 0.00039
	Hispanic	Men	62	0.000216, 0.000254, 0.000298, 0.00035, 0.000411
	Hispanic	Men	63	0.00023, 0.00027, 0.000316, 0.00037, 0.000434
	Hispanic	Men	64	0.000245, 0.000287, 0.000335, 0.000392, 0.00046
	Hispanic	Men	65	0.000261, 0.000305, 0.000357, 0.000418, 0.00049
	Hispanic	Men	66	0.000279, 0.000327, 0.000384, 0.00045, 0.000528
	Hispanic	Men	67	0.0003, 0.000353, 0.000416, 0.00049, 0.000578
	Hispanic	Men	68	0.000325, 0.000385, 0.000456, 0.000539, 0.00064
	Hispanic	Men	69	0.000356, 0.000424, 0.000503, 0.000598, 0.000711
	Hispanic	Men	70	0.000395, 0.00047, 0.000559, 0.000664, 0.00079
	Hispanic	Men	71	0.000441, 0.000524, 0.000622, 0.000738, 0.000877
	Hispanic	Men	72	0.000493, 0.000585, 0.000693, 0.00082, 0.000973
	Hispanic	Men	73	0.00055, 0.000652, 0.000772, 0.000913, 0.00108
	Hispanic	Men	74	0.000613, 0.000727, 0.00086, 0.00102, 0.00121
	Hispanic	Men	75	0.000684, 0.000811, 0.00096, 0.00114, 0.00135
	Hispanic	Men	76	0.000764, 0.000907, 0.00107, 0.00127, 0.00151
	Hispanic	Men	77	0.000853, 0.00101, 0.0012, 0.00143, 0.0017
	Hispanic	Men	78	0.00095, 0.00114, 0.00135, 0.00161, 0.00193
	Hispanic	Men	79	0.00105, 0.00127, 0.00152, 0.00182, 0.00219
	Hispanic	Men	80	0.00116, 0.00141, 0.0017, 0.00206, 0.00249
	Hispanic	Men	81	0.00127, 0.00155, 0.00189, 0.00231, 0.00282
	Hispanic	Men	82	0.00138, 0.0017, 0.00209, 0.00257, 0.00317
	Hispanic	Men	83	0.00148, 0.00184, 0.00229, 0.00284, 0.00354
	Hispanic	Men	84	0.00158, 0.00198, 0.00248, 0.00311, 0.00391
	Hispanic	Women	30	0.00000357, 0.00000579, 0.00000933, 0.000015, 0.0000243
	Hispanic	Women	31	0.00000432, 0.00000676, 0.0000105, 0.0000164, 0.0000257
	Hispanic	Women	32	0.00000514, 0.00000785, 0.0000119, 0.0000182, 0.0000277

Variable	Race/ethnicity	Sex	Age	Distribution
	Hispanic	Women	33	0.00000603, 0.00000906, 0.0000135, 0.0000203, 0.0000304
	Hispanic	Women	34	0.000007, 0.0000104, 0.0000153, 0.0000226, 0.0000335
	Hispanic	Women	35	0.00000807, 0.0000118, 0.0000172, 0.000025, 0.0000366
	Hispanic	Women	36	0.00000925, 0.0000133, 0.0000191, 0.0000274, 0.0000395
	Hispanic	Women	37	0.0000105, 0.0000149, 0.000021, 0.0000297, 0.000042
	Hispanic	Women	38	0.0000119, 0.0000165, 0.000023, 0.0000319, 0.0000444
	Hispanic	Women	39	0.0000132, 0.0000182, 0.0000249, 0.000034, 0.0000467
	Hispanic	Women	40	0.0000147, 0.0000198, 0.0000268, 0.0000361, 0.0000488
	Hispanic	Women	41	0.0000163, 0.0000216, 0.0000287, 0.000038, 0.0000505
	Hispanic	Women	42	0.0000181, 0.0000236, 0.0000307, 0.00004, 0.0000523
	Hispanic	Women	43	0.0000199, 0.0000257, 0.000033, 0.0000425, 0.0000549
	Hispanic	Women	44	0.0000218, 0.0000279, 0.0000357, 0.0000456, 0.0000585
	Hispanic	Women	45	0.0000242, 0.0000307, 0.0000388, 0.0000491, 0.0000622
	Hispanic	Women	46	0.0000276, 0.0000342, 0.0000424, 0.0000526, 0.0000654
	Hispanic	Women	47	0.0000316, 0.0000384, 0.0000467, 0.0000566, 0.0000689
	Hispanic	Women	48	0.0000359, 0.0000431, 0.0000516, 0.0000618, 0.0000741
	Hispanic	Women	49	0.0000401, 0.000048, 0.0000573, 0.0000685, 0.0000819
	Hispanic	Women	50	0.0000442, 0.0000533, 0.000064, 0.0000769, 0.0000926
	Hispanic	Women	51	0.0000485, 0.000059, 0.0000716, 0.0000869, 0.000106
	Hispanic	Women	52	0.000053, 0.0000653, 0.0000801, 0.0000984, 0.000121
	Hispanic	Women	53	0.0000577, 0.0000719, 0.0000894, 0.000111, 0.000138
	Hispanic	Women	54	0.0000628, 0.0000791, 0.0000993, 0.000125, 0.000157
	Hispanic	Women	55	0.0000689, 0.000087, 0.00011, 0.000138, 0.000175
	Hispanic	Women	56	0.000076, 0.0000958, 0.000121, 0.000152, 0.000192
	Hispanic	Women	57	0.0000838, 0.000105, 0.000132, 0.000166, 0.000208
	Hispanic	Women	58	0.000092, 0.000115, 0.000144, 0.000181, 0.000226
	Hispanic	Women	59	0.000101, 0.000126, 0.000157, 0.000196, 0.000246
	Hispanic	Women	60	0.000109, 0.000137, 0.000171, 0.000213, 0.000266
	Hispanic	Women	61	0.000118, 0.000148, 0.000184, 0.000229, 0.000286
	Hispanic	Women	62	0.000128, 0.000159, 0.000197, 0.000245, 0.000305
	Hispanic	Women	63	0.000137, 0.00017, 0.000211, 0.000261, 0.000325
	Hispanic	Women	64	0.000147, 0.000182, 0.000225, 0.000279, 0.000346
	Hispanic	Women	65	0.000157, 0.000195, 0.000241, 0.000299, 0.000371
	Hispanic	Women	66	0.000169, 0.00021, 0.000261, 0.000324, 0.000403
	Hispanic	Women	67	0.000184, 0.000229, 0.000285, 0.000355, 0.000442
	Hispanic	Women	68	0.000203, 0.000253, 0.000316, 0.000393, 0.000491
	Hispanic	Women	69	0.000227, 0.000283, 0.000353, 0.000439, 0.000549
	Hispanic	Women	70	0.000258, 0.000321, 0.000397, 0.000492, 0.000611
	Hispanic	Women	71	0.000298, 0.000366, 0.000449, 0.000551, 0.000678
	Hispanic	Women	72	0.000347, 0.000421, 0.000509, 0.000617, 0.000748
	Hispanic	Women	73	0.000404, 0.000484, 0.000578, 0.000691, 0.000827
	Hispanic	Women	74	0.000469, 0.000556, 0.000657, 0.000777, 0.00092
	Hispanic	Women	75	0.000541, 0.000637, 0.000748, 0.00088, 0.00104

Variable	Race/ethnicity	Sex	Age	Distribution
	Hispanic	Women	76	0.00062, 0.000729, 0.000856, 0.001, 0.00118
	Hispanic	Women	77	0.00071, 0.000836, 0.000981, 0.00115, 0.00136
	Hispanic	Women	78	0.000815, 0.00096, 0.00113, 0.00133, 0.00156
	Hispanic	Women	79	0.000937, 0.0011, 0.0013, 0.00152, 0.00179
	Hispanic	Women	80	0.00107, 0.00126, 0.00148, 0.00175, 0.00206
	Hispanic	Women	81	0.0012, 0.00143, 0.00169, 0.002, 0.00237
	Hispanic	Women	82	0.00132, 0.00159, 0.0019, 0.00228, 0.00275
	Hispanic	Women	83	0.00142, 0.00174, 0.00212, 0.0026, 0.00318
	Hispanic	Women	84	0.00151, 0.00188, 0.00235, 0.00292, 0.00365
	White	Men	30	0.00000481, 0.00000707, 0.0000103, 0.0000151, 0.0000222
	White	Men	31	0.00000578, 0.00000823, 0.0000117, 0.0000166, 0.0000237
	White	Men	32	0.00000685, 0.00000953, 0.0000132, 0.0000183, 0.0000255
	White	Men	33	0.00000803, 0.0000109, 0.0000149, 0.0000202, 0.0000275
	White	Men	34	0.00000923, 0.0000124, 0.0000166, 0.0000223, 0.0000299
	White	Men	35	0.0000104, 0.0000139, 0.0000184, 0.0000245, 0.0000326
	White	Men	36	0.0000115, 0.0000153, 0.0000203, 0.0000268, 0.0000356
	White	Men	37	0.0000127, 0.0000167, 0.0000221, 0.0000292, 0.0000386
	White	Men	38	0.0000138, 0.0000182, 0.0000239, 0.0000315, 0.0000416
	White	Men	39	0.0000149, 0.0000196, 0.0000258, 0.0000338, 0.0000445
	White	Men	40	0.0000162, 0.0000212, 0.0000276, 0.0000359, 0.0000469
	White	Men	41	0.0000179, 0.000023, 0.0000295, 0.0000377, 0.0000484
	White	Men	42	0.0000201, 0.0000252, 0.0000315, 0.0000393, 0.0000492
	White	Men	43	0.0000226, 0.0000277, 0.0000338, 0.0000413, 0.0000505
	White	Men	44	0.0000251, 0.0000303, 0.0000366, 0.0000441, 0.0000532
	White	Men	45	0.0000279, 0.0000334, 0.0000399, 0.0000477, 0.0000571
	White	Men	46	0.0000312, 0.0000371, 0.0000439, 0.000052, 0.0000618
	White	Men	47	0.0000351, 0.0000414, 0.0000487, 0.0000574, 0.0000676
	White	Men	48	0.0000393, 0.0000463, 0.0000545, 0.0000642, 0.0000757
	White	Men	49	0.0000437, 0.0000518, 0.0000614, 0.0000728, 0.0000864
	White	Men	50	0.0000487, 0.0000583, 0.0000696, 0.0000831, 0.0000993
	White	Men	51	0.0000551, 0.000066, 0.000079, 0.0000946, 0.000113
	White	Men	52	0.000063, 0.0000752, 0.0000897, 0.000107, 0.000128
	White	Men	53	0.0000718, 0.0000854, 0.000101, 0.00012, 0.000143
	White	Men	54	0.0000808, 0.000096, 0.000114, 0.000135, 0.000161
	White	Men	55	0.0000897, 0.000107, 0.000127, 0.000151, 0.00018
	White	Men	56	0.0000985, 0.000118, 0.000141, 0.000169, 0.000202
	White	Men	57	0.000107, 0.000129, 0.000156, 0.000187, 0.000226
	White	Men	58	0.000116, 0.000141, 0.000171, 0.000208, 0.000252
	White	Men	59	0.000126, 0.000154, 0.000188, 0.000229, 0.000279
	White	Men	60	0.000139, 0.000169, 0.000205, 0.00025, 0.000304
	White	Men	61	0.000153, 0.000185, 0.000224, 0.00027, 0.000327
	White	Men	62	0.00017, 0.000203, 0.000242, 0.000289, 0.000346
	White	Men	63	0.000189, 0.000222, 0.000261, 0.000308, 0.000363

Variable	Race/ethnicity	Sex	Age	Distribution
	White	Men	64	0.000209, 0.000243, 0.000282, 0.000327, 0.00038
	White	Men	65	0.000232, 0.000266, 0.000305, 0.000349, 0.000401
	White	Men	66	0.000256, 0.000292, 0.000332, 0.000379, 0.000432
	White	Men	67	0.000281, 0.000321, 0.000366, 0.000417, 0.000476
	White	Men	68	0.000311, 0.000356, 0.000407, 0.000466, 0.000533
	White	Men	69	0.000345, 0.000397, 0.000456, 0.000525, 0.000604
	White	Men	70	0.000386, 0.000446, 0.000515, 0.000594, 0.000686
	White	Men	71	0.000434, 0.000502, 0.000581, 0.000673, 0.000779
	White	Men	72	0.000486, 0.000565, 0.000656, 0.000762, 0.000886
	White	Men	73	0.000543, 0.000635, 0.00074, 0.000863, 0.00101
	White	Men	74	0.000606, 0.000712, 0.000834, 0.000978, 0.00115
	White	Men	75	0.000677, 0.000799, 0.000942, 0.00111, 0.00131
	White	Men	76	0.000759, 0.000901, 0.00107, 0.00127, 0.0015
	White	Men	77	0.000852, 0.00102, 0.00122, 0.00145, 0.00174
	White	Men	78	0.000956, 0.00116, 0.00139, 0.00168, 0.00203
	White	Men	79	0.00107, 0.00131, 0.0016, 0.00195, 0.00239
	White	Men	80	0.00119, 0.00148, 0.00183, 0.00227, 0.00282
	White	Men	81	0.00132, 0.00166, 0.00209, 0.00262, 0.00329
	White	Men	82	0.00148, 0.00187, 0.00236, 0.00298, 0.00377
	White	Men	83	0.00163, 0.00208, 0.00264, 0.00335, 0.00427
	White	Men	84	0.00178, 0.00228, 0.00292, 0.00373, 0.00479
	White	Women	30	0.00000432, 0.00000647, 0.00000965, 0.0000144, 0.0000215
	White	Women	31	0.00000518, 0.00000757, 0.000011, 0.000016, 0.0000234
	White	Women	32	0.00000618, 0.00000883, 0.0000126, 0.0000179, 0.0000255
	White	Women	33	0.0000073, 0.0000102, 0.0000143, 0.0000199, 0.0000278
	White	Women	34	0.00000845, 0.0000117, 0.000016, 0.0000221, 0.0000305
	White	Women	35	0.00000959, 0.0000131, 0.0000179, 0.0000243, 0.0000332
	White	Women	36	0.0000107, 0.0000145, 0.0000196, 0.0000265, 0.000036
	White	Women	37	0.0000118, 0.0000159, 0.0000213, 0.0000286, 0.0000385
	White	Women	38	0.0000129, 0.0000172, 0.000023, 0.0000306, 0.0000408
	White	Women	39	0.000014, 0.0000186, 0.0000245, 0.0000324, 0.0000429
	White	Women	40	0.0000153, 0.00002, 0.0000261, 0.000034, 0.0000444
	White	Women	41	0.0000169, 0.0000216, 0.0000276, 0.0000352, 0.000045
	White	Women	42	0.0000188, 0.0000234, 0.0000291, 0.0000362, 0.000045
	White	Women	43	0.0000209, 0.0000255, 0.0000309, 0.0000375, 0.0000456
	White	Women	44	0.0000228, 0.0000275, 0.000033, 0.0000396, 0.0000477
	White	Women	45	0.0000247, 0.0000297, 0.0000355, 0.0000426, 0.0000511
	White	Women	46	0.000027, 0.0000323, 0.0000386, 0.0000462, 0.0000553
	White	Women	47	0.0000295, 0.0000354, 0.0000423, 0.0000505, 0.0000605
	White	Women	48	0.0000323, 0.0000389, 0.0000466, 0.000056, 0.0000673
	White	Women	49	0.0000353, 0.0000428, 0.0000518, 0.0000626, 0.0000759
	White	Women	50	0.0000389, 0.0000474, 0.0000578, 0.0000704, 0.0000859
	White	Women	51	0.0000433, 0.0000529, 0.0000645, 0.0000787, 0.0000962

Variable	Race/ethnicity	Sex	Age	Distribution
	White	Women	52	0.0000488, 0.0000593, 0.000072, 0.0000875, 0.000106
	White	Women	53	0.0000549, 0.0000664, 0.00008, 0.0000965, 0.000117
	White	Women	54	0.0000614, 0.0000738, 0.0000884, 0.000106, 0.000127
	White	Women	55	0.0000684, 0.0000816, 0.0000971, 0.000116, 0.000138
	White	Women	56	0.0000759, 0.0000898, 0.000106, 0.000125, 0.000148
	White	Women	57	0.0000838, 0.0000985, 0.000116, 0.000136, 0.00016
	White	Women	58	0.0000923, 0.000108, 0.000126, 0.000147, 0.000172
	White	Women	59	0.000101, 0.000118, 0.000138, 0.00016, 0.000187
	White	Women	60	0.000111, 0.000129, 0.00015, 0.000174, 0.000203
	White	Women	61	0.000121, 0.000141, 0.000164, 0.00019, 0.000221
	White	Women	62	0.000132, 0.000154, 0.000178, 0.000206, 0.000239
	White	Women	63	0.000144, 0.000167, 0.000193, 0.000223, 0.000259
	White	Women	64	0.000157, 0.000182, 0.000209, 0.000241, 0.000279
	White	Women	65	0.000173, 0.000198, 0.000228, 0.000262, 0.000301
	White	Women	66	0.00019, 0.000218, 0.00025, 0.000287, 0.00033
	White	Women	67	0.00021, 0.000241, 0.000278, 0.000319, 0.000367
	White	Women	68	0.000234, 0.00027, 0.000312, 0.000359, 0.000414
	White	Women	69	0.000265, 0.000307, 0.000354, 0.000408, 0.000471
	White	Women	70	0.000304, 0.000351, 0.000405, 0.000466, 0.000539
	White	Women	71	0.000349, 0.000403, 0.000465, 0.000536, 0.000618
	White	Women	72	0.000401, 0.000463, 0.000534, 0.000616, 0.000712
	White	Women	73	0.000458, 0.000531, 0.000613, 0.000709, 0.000821
	White	Women	74	0.000525, 0.000609, 0.000704, 0.000814, 0.000944
	White	Women	75	0.000604, 0.000699, 0.000808, 0.000935, 0.00108
	White	Women	76	0.000695, 0.000805, 0.000931, 0.00108, 0.00125
	White	Women	77	0.000797, 0.000927, 0.00108, 0.00125, 0.00145
	White	Women	78	0.000911, 0.00107, 0.00125, 0.00146, 0.00171
	White	Women	79	0.00104, 0.00123, 0.00145, 0.00171, 0.00203
	White	Women	80	0.00119, 0.00142, 0.00169, 0.00201, 0.0024
	White	Women	81	0.00136, 0.00163, 0.00196, 0.00234, 0.00281
	White	Women	82	0.00155, 0.00187, 0.00225, 0.00271, 0.00327
	White	Women	83	0.00174, 0.00211, 0.00256, 0.0031, 0.00377
	White	Women	84	0.00193, 0.00236, 0.00288, 0.00351, 0.00428
Stroke mortality rates for 2034 (0.01, 0.2, 0.5, 0.8. 0.99 percentiles of the empirical distribution produced during forecasting)				
	Black	Men	30	0.000013, 0.0000194, 0.0000287, 0.0000425, 0.0000633
	Black	Men	31	0.0000164, 0.0000235, 0.0000337, 0.0000482, 0.0000692
	Black	Men	32	0.00002, 0.0000281, 0.0000393, 0.000055, 0.0000772
	Black	Men	33	0.0000238, 0.0000329, 0.0000455, 0.0000629, 0.0000872
	Black	Men	34	0.0000275, 0.0000379, 0.0000521, 0.0000716, 0.0000987
	Black	Men	35	0.0000311, 0.0000428, 0.0000587, 0.0000805, 0.000111
	Black	Men	36	0.0000347, 0.0000476, 0.0000651, 0.0000892, 0.000122
	Black	Men	37	0.0000384, 0.0000524, 0.0000713, 0.0000969, 0.000132
	Black	Men	38	0.0000423, 0.0000572, 0.000077, 0.000104, 0.00014

Variable	Race/ethnicity	Sex	Age	Distribution
	Black	Men	39	0.0000464, 0.0000619, 0.0000825, 0.00011, 0.000147
	Black	Men	40	0.0000507, 0.0000669, 0.0000879, 0.000116, 0.000152
	Black	Men	41	0.0000562, 0.0000726, 0.0000936, 0.000121, 0.000156
	Black	Men	42	0.0000634, 0.0000797, 0.0001, 0.000125, 0.000158
	Black	Men	43	0.0000711, 0.0000876, 0.000108, 0.000132, 0.000163
	Black	Men	44	0.0000776, 0.0000953, 0.000117, 0.000143, 0.000176
	Black	Men	45	0.0000841, 0.000104, 0.000128, 0.000158, 0.000195
	Black	Men	46	0.0000929, 0.000115, 0.000141, 0.000174, 0.000215
	Black	Men	47	0.000104, 0.000128, 0.000158, 0.000194, 0.000239
	Black	Men	48	0.000116, 0.000143, 0.000177, 0.000219, 0.000271
	Black	Men	49	0.000129, 0.000161, 0.000201, 0.00025, 0.000311
	Black	Men	50	0.000149, 0.000185, 0.000229, 0.000285, 0.000354
	Black	Men	51	0.000176, 0.000216, 0.000263, 0.000321, 0.000393
	Black	Men	52	0.00021, 0.000252, 0.000301, 0.00036, 0.000432
	Black	Men	53	0.000246, 0.00029, 0.000343, 0.000405, 0.000479
	Black	Men	54	0.000278, 0.000328, 0.000386, 0.000454, 0.000536
	Black	Men	55	0.000309, 0.000364, 0.000429, 0.000505, 0.000596
	Black	Men	56	0.000339, 0.0004, 0.000471, 0.000556, 0.000656
	Black	Men	57	0.000368, 0.000435, 0.000513, 0.000606, 0.000716
	Black	Men	58	0.000398, 0.00047, 0.000554, 0.000654, 0.000772
	Black	Men	59	0.000429, 0.000506, 0.000595, 0.000701, 0.000826
	Black	Men	60	0.000455, 0.000538, 0.000635, 0.00075, 0.000886
	Black	Men	61	0.000476, 0.000567, 0.000673, 0.000799, 0.00095
	Black	Men	62	0.000499, 0.000595, 0.000707, 0.000841, 0.001
	Black	Men	63	0.000529, 0.000626, 0.00074, 0.000874, 0.00103
	Black	Men	64	0.000563, 0.00066, 0.000772, 0.000903, 0.00106
	Black	Men	65	0.000596, 0.000694, 0.000808, 0.000941, 0.0011
	Black	Men	66	0.000626, 0.000731, 0.000853, 0.000994, 0.00116
	Black	Men	67	0.000664, 0.000777, 0.000909, 0.00106, 0.00124
	Black	Men	68	0.000714, 0.000836, 0.000977, 0.00114, 0.00134
	Black	Men	69	0.000775, 0.000906, 0.00106, 0.00123, 0.00144
	Black	Men	70	0.000842, 0.000984, 0.00115, 0.00134, 0.00156
	Black	Men	71	0.000913, 0.00107, 0.00124, 0.00145, 0.00169
	Black	Men	72	0.000987, 0.00115, 0.00134, 0.00157, 0.00183
	Black	Men	73	0.00106, 0.00124, 0.00145, 0.00169, 0.00198
	Black	Men	74	0.00113, 0.00133, 0.00156, 0.00184, 0.00216
	Black	Men	75	0.00121, 0.00143, 0.00169, 0.002, 0.00236
	Black	Men	76	0.00131, 0.00155, 0.00184, 0.00217, 0.00258
	Black	Men	77	0.00142, 0.00169, 0.002, 0.00237, 0.00282
	Black	Men	78	0.00154, 0.00184, 0.00218, 0.0026, 0.00309
	Black	Men	79	0.00167, 0.002, 0.00239, 0.00285, 0.00341
	Black	Men	80	0.00179, 0.00216, 0.00259, 0.00312, 0.00375
	Black	Men	81	0.00192, 0.00232, 0.0028, 0.00339, 0.0041

Variable	Race/ethnicity	Sex	Age	Distribution
	Black	Men	82	0.00204, 0.00248, 0.00301, 0.00365, 0.00444
	Black	Men	83	0.00215, 0.00263, 0.0032, 0.00391, 0.00477
	Black	Men	84	0.00226, 0.00277, 0.00339, 0.00416, 0.0051
	Black	Women	30	0.00000928, 0.0000149, 0.0000237, 0.0000377, 0.0000603
	Black	Women	31	0.000011, 0.0000174, 0.0000273, 0.0000428, 0.0000675
	Black	Women	32	0.0000129, 0.0000202, 0.0000314, 0.0000489, 0.0000765
	Black	Women	33	0.0000147, 0.000023, 0.0000359, 0.0000558, 0.0000873
	Black	Women	34	0.0000165, 0.000026, 0.0000406, 0.0000635, 0.0000999
	Black	Women	35	0.0000183, 0.0000289, 0.0000455, 0.0000716, 0.000113
	Black	Women	36	0.0000204, 0.0000321, 0.0000505, 0.0000792, 0.000125
	Black	Women	37	0.0000229, 0.0000357, 0.0000554, 0.000086, 0.000134
	Black	Women	38	0.0000257, 0.0000395, 0.0000604, 0.0000922, 0.000142
	Black	Women	39	0.0000286, 0.0000433, 0.0000653, 0.0000984, 0.000149
	Black	Women	40	0.0000314, 0.000047, 0.0000703, 0.000105, 0.000157
	Black	Women	41	0.0000343, 0.0000509, 0.0000753, 0.000111, 0.000165
	Black	Women	42	0.0000379, 0.0000553, 0.0000805, 0.000117, 0.000171
	Black	Women	43	0.0000423, 0.0000605, 0.0000863, 0.000123, 0.000176
	Black	Women	44	0.0000469, 0.0000661, 0.0000929, 0.000131, 0.000184
	Black	Women	45	0.0000519, 0.0000724, 0.000101, 0.00014, 0.000195
	Black	Women	46	0.0000584, 0.0000803, 0.00011, 0.000151, 0.000207
	Black	Women	47	0.0000669, 0.0000901, 0.000121, 0.000162, 0.000218
	Black	Women	48	0.0000771, 0.000102, 0.000134, 0.000176, 0.000232
	Black	Women	49	0.0000878, 0.000114, 0.000148, 0.000192, 0.00025
	Black	Women	50	0.0000985, 0.000127, 0.000164, 0.000212, 0.000274
	Black	Women	51	0.00011, 0.000142, 0.000182, 0.000233, 0.0003
	Black	Women	52	0.000124, 0.000158, 0.000201, 0.000255, 0.000324
	Black	Women	53	0.000139, 0.000175, 0.00022, 0.000277, 0.000349
	Black	Women	54	0.000152, 0.000191, 0.00024, 0.000301, 0.000379
	Black	Women	55	0.000164, 0.000207, 0.000261, 0.000328, 0.000414
	Black	Women	56	0.000179, 0.000225, 0.000283, 0.000356, 0.000448
	Black	Women	57	0.000197, 0.000246, 0.000307, 0.000382, 0.000478
	Black	Women	58	0.000219, 0.00027, 0.000332, 0.000408, 0.000502
	Black	Women	59	0.000244, 0.000296, 0.000359, 0.000434, 0.000527
	Black	Women	60	0.000267, 0.000321, 0.000385, 0.000463, 0.000557
	Black	Women	61	0.000285, 0.000343, 0.000411, 0.000493, 0.000592
	Black	Women	62	0.000301, 0.000362, 0.000435, 0.000522, 0.000628
	Black	Women	63	0.000316, 0.00038, 0.000457, 0.000548, 0.00066
	Black	Women	64	0.000333, 0.000399, 0.000478, 0.000573, 0.000687
	Black	Women	65	0.000353, 0.000421, 0.000502, 0.000598, 0.000714
	Black	Women	66	0.000378, 0.000449, 0.000532, 0.00063, 0.000748
	Black	Women	67	0.000409, 0.000483, 0.000569, 0.000671, 0.000793
	Black	Women	68	0.000447, 0.000526, 0.000617, 0.000724, 0.000852
	Black	Women	69	0.000495, 0.000579, 0.000677, 0.00079, 0.000925

Variable	Race/ethnicity	Sex	Age	Distribution
	Black	Women	70	0.00055, 0.000642, 0.000747, 0.00087, 0.00101
	Black	Women	71	0.000611, 0.000712, 0.000829, 0.000965, 0.00112
	Black	Women	72	0.000678, 0.000791, 0.000921, 0.00107, 0.00125
	Black	Women	73	0.000754, 0.00088, 0.00102, 0.00119, 0.00139
	Black	Women	74	0.000842, 0.000981, 0.00114, 0.00133, 0.00155
	Black	Women	75	0.00094, 0.00109, 0.00127, 0.00148, 0.00172
	Black	Women	76	0.00104, 0.00122, 0.00142, 0.00166, 0.00194
	Black	Women	77	0.00115, 0.00136, 0.0016, 0.00188, 0.00222
	Black	Women	78	0.00126, 0.0015, 0.0018, 0.00214, 0.00256
	Black	Women	79	0.00138, 0.00167, 0.00202, 0.00243, 0.00294
	Black	Women	80	0.00152, 0.00185, 0.00226, 0.00275, 0.00335
	Black	Women	81	0.00167, 0.00205, 0.00251, 0.00307, 0.00377
	Black	Women	82	0.00182, 0.00225, 0.00277, 0.00342, 0.00422
	Black	Women	83	0.00197, 0.00245, 0.00304, 0.00377, 0.00469
	Black	Women	84	0.0021, 0.00264, 0.0033, 0.00413, 0.00517
	Hispanic	Men	30	0.000006, 0.00000902, 0.0000135, 0.0000202, 0.0000304
	Hispanic	Men	31	0.00000737, 0.0000107, 0.0000154, 0.0000222, 0.0000322
	Hispanic	Men	32	0.00000891, 0.0000125, 0.0000175, 0.0000245, 0.0000345
	Hispanic	Men	33	0.0000105, 0.0000145, 0.0000199, 0.0000273, 0.0000375
	Hispanic	Men	34	0.0000121, 0.0000165, 0.0000224, 0.0000304, 0.0000414
	Hispanic	Men	35	0.0000137, 0.0000185, 0.000025, 0.0000338, 0.0000458
	Hispanic	Men	36	0.0000152, 0.0000206, 0.0000277, 0.0000372, 0.0000503
	Hispanic	Men	37	0.0000169, 0.0000227, 0.0000303, 0.0000405, 0.0000543
	Hispanic	Men	38	0.0000187, 0.0000248, 0.0000329, 0.0000436, 0.000058
	Hispanic	Men	39	0.0000204, 0.000027, 0.0000355, 0.0000467, 0.0000616
	Hispanic	Men	40	0.0000223, 0.0000292, 0.000038, 0.0000496, 0.0000648
	Hispanic	Men	41	0.0000246, 0.0000317, 0.0000406, 0.0000521, 0.0000671
	Hispanic	Men	42	0.0000275, 0.0000346, 0.0000434, 0.0000545, 0.0000686
	Hispanic	Men	43	0.0000305, 0.0000377, 0.0000465, 0.0000573, 0.0000709
	Hispanic	Men	44	0.0000333, 0.0000409, 0.0000501, 0.0000613, 0.0000753
	Hispanic	Men	45	0.0000363, 0.0000444, 0.0000543, 0.0000664, 0.0000813
	Hispanic	Men	46	0.0000401, 0.0000489, 0.0000594, 0.0000722, 0.000088
	Hispanic	Men	47	0.0000449, 0.0000543, 0.0000656, 0.0000792, 0.0000958
	Hispanic	Men	48	0.0000506, 0.0000609, 0.000073, 0.0000876, 0.000105
	Hispanic	Men	49	0.0000577, 0.0000689, 0.000082, 0.0000978, 0.000117
	Hispanic	Men	50	0.0000665, 0.0000786, 0.0000928, 0.000109, 0.000129
	Hispanic	Men	51	0.0000774, 0.0000903, 0.000105, 0.000123, 0.000143
	Hispanic	Men	52	0.0000893, 0.000103, 0.000119, 0.000138, 0.00016
	Hispanic	Men	53	0.0000998, 0.000116, 0.000135, 0.000157, 0.000182
	Hispanic	Men	54	0.000108, 0.000128, 0.000151, 0.000179, 0.000211
	Hispanic	Men	55	0.000117, 0.00014, 0.000168, 0.000202, 0.000242
	Hispanic	Men	56	0.000127, 0.000153, 0.000186, 0.000224, 0.000272
	Hispanic	Men	57	0.000138, 0.000167, 0.000203, 0.000246, 0.000299

Variable	Race/ethnicity	Sex	Age	Distribution
	Hispanic	Men	58	0.000151, 0.000183, 0.000221, 0.000267, 0.000324
	Hispanic	Men	59	0.000165, 0.000199, 0.000239, 0.000287, 0.000346
	Hispanic	Men	60	0.000181, 0.000216, 0.000257, 0.000307, 0.000366
	Hispanic	Men	61	0.000196, 0.000233, 0.000275, 0.000325, 0.000386
	Hispanic	Men	62	0.000211, 0.000249, 0.000293, 0.000345, 0.000406
	Hispanic	Men	63	0.000224, 0.000264, 0.00031, 0.000364, 0.000428
	Hispanic	Men	64	0.000238, 0.00028, 0.000329, 0.000385, 0.000453
	Hispanic	Men	65	0.000254, 0.000298, 0.00035, 0.00041, 0.000482
	Hispanic	Men	66	0.000271, 0.000319, 0.000375, 0.000441, 0.000519
	Hispanic	Men	67	0.000291, 0.000345, 0.000407, 0.00048, 0.000567
	Hispanic	Men	68	0.000316, 0.000375, 0.000445, 0.000528, 0.000628
	Hispanic	Men	69	0.000346, 0.000413, 0.000492, 0.000585, 0.000698
	Hispanic	Men	70	0.000384, 0.000458, 0.000546, 0.00065, 0.000776
	Hispanic	Men	71	0.000428, 0.000511, 0.000607, 0.000722, 0.000861
	Hispanic	Men	72	0.000479, 0.000569, 0.000676, 0.000803, 0.000955
	Hispanic	Men	73	0.000534, 0.000634, 0.000753, 0.000893, 0.00106
	Hispanic	Men	74	0.000595, 0.000707, 0.000839, 0.000995, 0.00118
	Hispanic	Men	75	0.000664, 0.000789, 0.000936, 0.00111, 0.00132
	Hispanic	Men	76	0.000741, 0.000882, 0.00105, 0.00124, 0.00148
	Hispanic	Men	77	0.000828, 0.000987, 0.00117, 0.0014, 0.00167
	Hispanic	Men	78	0.000922, 0.0011, 0.00132, 0.00158, 0.00189
	Hispanic	Men	79	0.00102, 0.00123, 0.00148, 0.00178, 0.00215
	Hispanic	Men	80	0.00113, 0.00137, 0.00166, 0.00201, 0.00244
	Hispanic	Men	81	0.00123, 0.00151, 0.00185, 0.00226, 0.00276
	Hispanic	Men	82	0.00134, 0.00165, 0.00204, 0.00251, 0.00311
	Hispanic	Men	83	0.00144, 0.00179, 0.00223, 0.00278, 0.00347
	Hispanic	Men	84	0.00153, 0.00193, 0.00242, 0.00304, 0.00383
	Hispanic	Women	30	0.00000352, 0.00000573, 0.00000929, 0.0000151, 0.0000245
	Hispanic	Women	31	0.00000426, 0.0000067, 0.0000105, 0.0000164, 0.0000258
	Hispanic	Women	32	0.00000508, 0.00000779, 0.0000119, 0.0000182, 0.0000279
	Hispanic	Women	33	0.00000596, 0.00000899, 0.0000135, 0.0000203, 0.0000306
	Hispanic	Women	34	0.00000692, 0.0000103, 0.0000153, 0.0000226, 0.0000337
	Hispanic	Women	35	0.00000797, 0.0000117, 0.0000171, 0.000025, 0.0000368
	Hispanic	Women	36	0.00000912, 0.0000132, 0.000019, 0.0000274, 0.0000396
	Hispanic	Women	37	0.0000104, 0.0000147, 0.0000209, 0.0000296, 0.0000421
	Hispanic	Women	38	0.0000117, 0.0000163, 0.0000228, 0.0000317, 0.0000444
	Hispanic	Women	39	0.000013, 0.0000179, 0.0000246, 0.0000338, 0.0000466
	Hispanic	Women	40	0.0000143, 0.0000195, 0.0000264, 0.0000358, 0.0000486
	Hispanic	Women	41	0.0000159, 0.0000212, 0.0000282, 0.0000376, 0.0000501
	Hispanic	Women	42	0.0000176, 0.0000231, 0.0000302, 0.0000394, 0.0000517
	Hispanic	Women	43	0.0000194, 0.0000251, 0.0000324, 0.0000418, 0.0000541
	Hispanic	Women	44	0.0000212, 0.0000273, 0.0000349, 0.0000448, 0.0000575
	Hispanic	Women	45	0.0000236, 0.0000299, 0.0000379, 0.0000481, 0.000061

Variable	Race/ethnicity	Sex	Age	Distribution
	Hispanic	Women	46	0.0000268, 0.0000334, 0.0000415, 0.0000515, 0.0000641
	Hispanic	Women	47	0.0000308, 0.0000375, 0.0000456, 0.0000554, 0.0000676
	Hispanic	Women	48	0.000035, 0.000042, 0.0000504, 0.0000605, 0.0000726
	Hispanic	Women	49	0.0000391, 0.0000469, 0.0000561, 0.0000671, 0.0000804
	Hispanic	Women	50	0.0000432, 0.0000521, 0.0000627, 0.0000754, 0.0000909
	Hispanic	Women	51	0.0000474, 0.0000578, 0.0000702, 0.0000853, 0.000104
	Hispanic	Women	52	0.000052, 0.000064, 0.0000787, 0.0000967, 0.000119
	Hispanic	Women	53	0.0000566, 0.0000706, 0.0000879, 0.000109, 0.000136
	Hispanic	Women	54	0.0000617, 0.0000777, 0.0000977, 0.000123, 0.000155
	Hispanic	Women	55	0.0000676, 0.0000856, 0.000108, 0.000136, 0.000173
	Hispanic	Women	56	0.0000746, 0.0000943, 0.000119, 0.00015, 0.000189
	Hispanic	Women	57	0.0000823, 0.000104, 0.00013, 0.000164, 0.000206
	Hispanic	Women	58	0.0000904, 0.000114, 0.000142, 0.000178, 0.000224
	Hispanic	Women	59	0.0000987, 0.000124, 0.000155, 0.000194, 0.000243
	Hispanic	Women	60	0.000107, 0.000134, 0.000168, 0.00021, 0.000263
	Hispanic	Women	61	0.000116, 0.000145, 0.000181, 0.000226, 0.000282
	Hispanic	Women	62	0.000125, 0.000156, 0.000194, 0.000241, 0.000301
	Hispanic	Women	63	0.000134, 0.000167, 0.000207, 0.000257, 0.00032
	Hispanic	Women	64	0.000143, 0.000178, 0.000221, 0.000274, 0.00034
	Hispanic	Women	65	0.000153, 0.00019, 0.000236, 0.000293, 0.000365
	Hispanic	Women	66	0.000165, 0.000205, 0.000255, 0.000317, 0.000395
	Hispanic	Women	67	0.000179, 0.000224, 0.000279, 0.000347, 0.000434
	Hispanic	Women	68	0.000197, 0.000247, 0.000308, 0.000385, 0.000482
	Hispanic	Women	69	0.000221, 0.000276, 0.000345, 0.00043, 0.000538
	Hispanic	Women	70	0.000251, 0.000313, 0.000388, 0.000482, 0.0006
	Hispanic	Women	71	0.00029, 0.000357, 0.000439, 0.00054, 0.000665
	Hispanic	Women	72	0.000337, 0.00041, 0.000497, 0.000604, 0.000734
	Hispanic	Women	73	0.000392, 0.000471, 0.000564, 0.000676, 0.000811
	Hispanic	Women	74	0.000455, 0.00054, 0.000641, 0.00076, 0.000902
	Hispanic	Women	75	0.000525, 0.000619, 0.00073, 0.00086, 0.00102
	Hispanic	Women	76	0.000601, 0.000709, 0.000834, 0.000981, 0.00116
	Hispanic	Women	77	0.000689, 0.000812, 0.000957, 0.00113, 0.00133
	Hispanic	Women	78	0.00079, 0.000933, 0.0011, 0.0013, 0.00153
	Hispanic	Women	79	0.000908, 0.00107, 0.00126, 0.00149, 0.00176
	Hispanic	Women	80	0.00104, 0.00123, 0.00145, 0.00171, 0.00202
	Hispanic	Women	81	0.00116, 0.00139, 0.00165, 0.00195, 0.00233
	Hispanic	Women	82	0.00128, 0.00154, 0.00185, 0.00223, 0.00269
	Hispanic	Women	83	0.00137, 0.00169, 0.00207, 0.00254, 0.00312
	Hispanic	Women	84	0.00146, 0.00183, 0.00229, 0.00286, 0.00358
	White	Men	30	0.00000472, 0.00000698, 0.0000103, 0.0000152, 0.0000225
	White	Men	31	0.00000568, 0.00000815, 0.0000116, 0.0000166, 0.0000239
	White	Men	32	0.00000675, 0.00000944, 0.0000132, 0.0000183, 0.0000257
	White	Men	33	0.00000791, 0.0000108, 0.0000148, 0.0000202, 0.0000277

Variable	Race/ethnicity	Sex	Age	Distribution
	White	Men	34	0.0000091, 0.0000123, 0.0000166, 0.0000223, 0.0000301
	White	Men	35	0.0000102, 0.0000137, 0.0000183, 0.0000245, 0.0000329
	White	Men	36	0.0000113, 0.0000151, 0.0000201, 0.0000268, 0.0000358
	White	Men	37	0.0000124, 0.0000165, 0.0000219, 0.0000291, 0.0000387
	White	Men	38	0.0000135, 0.0000179, 0.0000237, 0.0000314, 0.0000417
	White	Men	39	0.0000146, 0.0000193, 0.0000255, 0.0000336, 0.0000445
	White	Men	40	0.0000158, 0.0000208, 0.0000272, 0.0000356, 0.0000467
	White	Men	41	0.0000175, 0.0000225, 0.000029, 0.0000373, 0.0000481
	White	Men	42	0.0000196, 0.0000246, 0.0000309, 0.0000388, 0.0000487
	White	Men	43	0.000022, 0.000027, 0.0000331, 0.0000406, 0.0000498
	White	Men	44	0.0000244, 0.0000296, 0.0000358, 0.0000432, 0.0000524
	White	Men	45	0.0000271, 0.0000325, 0.000039, 0.0000467, 0.0000561
	White	Men	46	0.0000303, 0.0000361, 0.0000429, 0.0000509, 0.0000606
	White	Men	47	0.0000341, 0.0000403, 0.0000476, 0.0000561, 0.0000664
	White	Men	48	0.0000382, 0.0000451, 0.0000532, 0.0000628, 0.0000742
	White	Men	49	0.0000425, 0.0000506, 0.00006, 0.0000713, 0.0000847
	White	Men	50	0.0000475, 0.0000569, 0.0000681, 0.0000814, 0.0000976
	White	Men	51	0.0000538, 0.0000646, 0.0000774, 0.0000928, 0.000112
	White	Men	52	0.0000615, 0.0000737, 0.000088, 0.000105, 0.000126
	White	Men	53	0.0000702, 0.0000837, 0.0000996, 0.000119, 0.000141
	White	Men	54	0.0000791, 0.0000942, 0.000112, 0.000133, 0.000159
	White	Men	55	0.0000879, 0.000105, 0.000125, 0.000149, 0.000178
	White	Men	56	0.0000965, 0.000116, 0.000139, 0.000166, 0.000199
	White	Men	57	0.000105, 0.000127, 0.000153, 0.000185, 0.000223
	White	Men	58	0.000114, 0.000139, 0.000169, 0.000205, 0.000249
	White	Men	59	0.000124, 0.000151, 0.000185, 0.000226, 0.000276
	White	Men	60	0.000136, 0.000166, 0.000202, 0.000246, 0.000301
	White	Men	61	0.00015, 0.000182, 0.00022, 0.000266, 0.000323
	White	Men	62	0.000166, 0.000199, 0.000238, 0.000285, 0.000342
	White	Men	63	0.000184, 0.000217, 0.000256, 0.000303, 0.000358
	White	Men	64	0.000204, 0.000238, 0.000276, 0.000321, 0.000374
	White	Men	65	0.000226, 0.00026, 0.000299, 0.000343, 0.000395
	White	Men	66	0.000249, 0.000285, 0.000325, 0.000371, 0.000425
	White	Men	67	0.000273, 0.000313, 0.000358, 0.000409, 0.000468
	White	Men	68	0.000302, 0.000346, 0.000398, 0.000456, 0.000524
	White	Men	69	0.000335, 0.000387, 0.000446, 0.000514, 0.000593
	White	Men	70	0.000375, 0.000434, 0.000503, 0.000582, 0.000674
	White	Men	71	0.000421, 0.000489, 0.000568, 0.000659, 0.000766
	White	Men	72	0.000472, 0.00055, 0.000641, 0.000746, 0.00087
	White	Men	73	0.000527, 0.000617, 0.000722, 0.000845, 0.000989
	White	Men	74	0.000588, 0.000692, 0.000814, 0.000957, 0.00113
	White	Men	75	0.000657, 0.000778, 0.000919, 0.00109, 0.00129
	White	Men	76	0.000736, 0.000877, 0.00104, 0.00124, 0.00148

Variable	Race/ethnicity	Sex	Age	Distribution
	White	Men	77	0.000827, 0.000992, 0.00119, 0.00142, 0.00171
	White	Men	78	0.000927, 0.00112, 0.00136, 0.00164, 0.00199
	White	Men	79	0.00104, 0.00127, 0.00156, 0.00191, 0.00235
	White	Men	80	0.00115, 0.00144, 0.00179, 0.00222, 0.00277
	White	Men	81	0.00128, 0.00162, 0.00204, 0.00256, 0.00323
	White	Men	82	0.00143, 0.00182, 0.0023, 0.00291, 0.0037
	White	Men	83	0.00158, 0.00202, 0.00257, 0.00328, 0.00419
	White	Men	84	0.00172, 0.00222, 0.00284, 0.00365, 0.0047
	White	Women	30	0.00000424, 0.00000639, 0.0000096, 0.0000144, 0.0000217
	White	Women	31	0.0000051, 0.00000749, 0.000011, 0.0000161, 0.0000236
	White	Women	32	0.00000609, 0.00000875, 0.0000125, 0.0000179, 0.0000257
	White	Women	33	0.0000072, 0.0000101, 0.0000142, 0.0000199, 0.000028
	White	Women	34	0.00000834, 0.0000116, 0.000016, 0.0000221, 0.0000306
	White	Women	35	0.00000945, 0.000013, 0.0000178, 0.0000243, 0.0000334
	White	Women	36	0.0000105, 0.0000144, 0.0000195, 0.0000265, 0.0000362
	White	Women	37	0.0000116, 0.0000157, 0.0000212, 0.0000286, 0.0000387
	White	Women	38	0.0000127, 0.000017, 0.0000228, 0.0000305, 0.0000409
	White	Women	39	0.0000137, 0.0000183, 0.0000243, 0.0000322, 0.0000429
	White	Women	40	0.0000149, 0.0000196, 0.0000257, 0.0000337, 0.0000443
	White	Women	41	0.0000164, 0.0000211, 0.0000271, 0.0000348, 0.0000448
	White	Women	42	0.0000184, 0.0000229, 0.0000286, 0.0000357, 0.0000446
	White	Women	43	0.0000204, 0.0000249, 0.0000303, 0.0000369, 0.000045
	White	Women	44	0.0000222, 0.0000268, 0.0000323, 0.0000389, 0.0000469
	White	Women	45	0.0000241, 0.0000289, 0.0000348, 0.0000417, 0.0000502
	White	Women	46	0.0000262, 0.0000315, 0.0000377, 0.0000452, 0.0000543
	White	Women	47	0.0000287, 0.0000345, 0.0000413, 0.0000495, 0.0000593
	White	Women	48	0.0000315, 0.0000379, 0.0000456, 0.0000548, 0.000066
	White	Women	49	0.0000344, 0.0000418, 0.0000506, 0.0000614, 0.0000745
	White	Women	50	0.0000379, 0.0000464, 0.0000566, 0.000069, 0.0000843
	White	Women	51	0.0000423, 0.0000518, 0.0000633, 0.0000773, 0.0000946
	White	Women	52	0.0000478, 0.0000582, 0.0000707, 0.0000859, 0.000105
	White	Women	53	0.0000539, 0.0000651, 0.0000787, 0.000095, 0.000115
	White	Women	54	0.0000603, 0.0000725, 0.000087, 0.000104, 0.000126
	White	Women	55	0.0000671, 0.0000802, 0.0000956, 0.000114, 0.000136
	White	Women	56	0.0000745, 0.0000883, 0.000104, 0.000124, 0.000147
	White	Women	57	0.0000822, 0.0000969, 0.000114, 0.000134, 0.000158
	White	Women	58	0.0000905, 0.000106, 0.000124, 0.000145, 0.000171
	White	Women	59	0.0000994, 0.000116, 0.000136, 0.000158, 0.000185
	White	Women	60	0.000109, 0.000127, 0.000148, 0.000172, 0.000201
	White	Women	61	0.000119, 0.000138, 0.000161, 0.000187, 0.000219
	White	Women	62	0.000129, 0.000151, 0.000175, 0.000203, 0.000237
	White	Women	63	0.000141, 0.000163, 0.00019, 0.00022, 0.000255
	White	Women	64	0.000153, 0.000178, 0.000205, 0.000237, 0.000275

Variable	Race/ethnicity	Sex	Age	Distribution
	White	Women	65	0.000168, 0.000194, 0.000223, 0.000257, 0.000296
	White	Women	66	0.000185, 0.000213, 0.000245, 0.000281, 0.000324
	White	Women	67	0.000204, 0.000235, 0.000271, 0.000313, 0.000361
	White	Women	68	0.000228, 0.000263, 0.000304, 0.000352, 0.000407
	White	Women	69	0.000258, 0.000299, 0.000345, 0.0004, 0.000463
	White	Women	70	0.000295, 0.000342, 0.000395, 0.000457, 0.000529
	White	Women	71	0.000339, 0.000392, 0.000454, 0.000525, 0.000607
	White	Women	72	0.000389, 0.00045, 0.000521, 0.000603, 0.000699
	White	Women	73	0.000445, 0.000516, 0.000599, 0.000694, 0.000806
	White	Women	74	0.000509, 0.000592, 0.000687, 0.000797, 0.000926
	White	Women	75	0.000585, 0.00068, 0.000788, 0.000914, 0.00106
	White	Women	76	0.000673, 0.000782, 0.000907, 0.00105, 0.00122
	White	Women	77	0.000772, 0.000901, 0.00105, 0.00122, 0.00142
	White	Women	78	0.000883, 0.00104, 0.00122, 0.00143, 0.00168
	White	Women	79	0.00101, 0.0012, 0.00142, 0.00168, 0.00199
	White	Women	80	0.00115, 0.00138, 0.00165, 0.00197, 0.00235
	White	Women	81	0.00132, 0.00159, 0.00191, 0.00229, 0.00276
	White	Women	82	0.0015, 0.00181, 0.00219, 0.00265, 0.00321
	White	Women	83	0.00168, 0.00205, 0.00249, 0.00303, 0.0037
	White	Women	84	0.00187, 0.00229, 0.00281, 0.00343, 0.0042
Stroke mortality rates for 2035 (0.01, 0.2, 0.5, 0.8. 0.99 percentiles of the empirical distribution produced during forecasting)				
	Black	Men	30	0.0000128, 0.0000191, 0.0000286, 0.0000426, 0.0000639
	Black	Men	31	0.0000161, 0.0000233, 0.0000335, 0.0000483, 0.0000698
	Black	Men	32	0.0000197, 0.0000278, 0.0000392, 0.0000551, 0.0000778
	Black	Men	33	0.0000234, 0.0000327, 0.0000454, 0.000063, 0.0000878
	Black	Men	34	0.0000271, 0.0000376, 0.0000519, 0.0000717, 0.0000993
	Black	Men	35	0.0000306, 0.0000424, 0.0000584, 0.0000806, 0.000112
	Black	Men	36	0.0000341, 0.0000471, 0.0000648, 0.0000891, 0.000123
	Black	Men	37	0.0000377, 0.0000518, 0.0000708, 0.0000968, 0.000133
	Black	Men	38	0.0000415, 0.0000564, 0.0000763, 0.000103, 0.00014
	Black	Men	39	0.0000453, 0.0000609, 0.0000816, 0.000109, 0.000147
	Black	Men	40	0.0000495, 0.0000656, 0.0000868, 0.000115, 0.000152
	Black	Men	41	0.0000548, 0.0000712, 0.0000921, 0.000119, 0.000155
	Black	Men	42	0.0000618, 0.000078, 0.0000982, 0.000124, 0.000156
	Black	Men	43	0.0000693, 0.0000856, 0.000105, 0.00013, 0.000161
	Black	Men	44	0.0000755, 0.000093, 0.000114, 0.000141, 0.000173
	Black	Men	45	0.0000818, 0.000101, 0.000125, 0.000155, 0.000191
	Black	Men	46	0.0000904, 0.000112, 0.000138, 0.000171, 0.000211
	Black	Men	47	0.000101, 0.000125, 0.000154, 0.00019, 0.000234
	Black	Men	48	0.000113, 0.00014, 0.000173, 0.000214, 0.000266
	Black	Men	49	0.000126, 0.000158, 0.000196, 0.000245, 0.000305
	Black	Men	50	0.000145, 0.000181, 0.000225, 0.000279, 0.000347
	Black	Men	51	0.000172, 0.000211, 0.000258, 0.000315, 0.000386

Variable	Race/ethnicity	Sex	Age	Distribution
	Black	Men	52	0.000206, 0.000247, 0.000296, 0.000354, 0.000425
	Black	Men	53	0.000241, 0.000285, 0.000337, 0.000399, 0.000472
	Black	Men	54	0.000273, 0.000322, 0.00038, 0.000448, 0.000529
	Black	Men	55	0.000303, 0.000358, 0.000422, 0.000498, 0.000589
	Black	Men	56	0.000332, 0.000393, 0.000464, 0.000548, 0.000649
	Black	Men	57	0.000361, 0.000427, 0.000506, 0.000598, 0.000709
	Black	Men	58	0.00039, 0.000462, 0.000546, 0.000646, 0.000764
	Black	Men	59	0.00042, 0.000497, 0.000586, 0.000692, 0.000818
	Black	Men	60	0.000446, 0.000528, 0.000625, 0.00074, 0.000877
	Black	Men	61	0.000466, 0.000556, 0.000662, 0.000788, 0.00094
	Black	Men	62	0.000488, 0.000583, 0.000695, 0.000829, 0.00099
	Black	Men	63	0.000516, 0.000613, 0.000726, 0.00086, 0.00102
	Black	Men	64	0.000549, 0.000645, 0.000757, 0.000887, 0.00104
	Black	Men	65	0.00058, 0.000678, 0.000791, 0.000923, 0.00108
	Black	Men	66	0.00061, 0.000714, 0.000834, 0.000975, 0.00114
	Black	Men	67	0.000646, 0.000758, 0.000888, 0.00104, 0.00122
	Black	Men	68	0.000694, 0.000814, 0.000955, 0.00112, 0.00131
	Black	Men	69	0.000752, 0.000882, 0.00103, 0.00121, 0.00142
	Black	Men	70	0.000818, 0.000958, 0.00112, 0.00131, 0.00154
	Black	Men	71	0.000887, 0.00104, 0.00121, 0.00142, 0.00166
	Black	Men	72	0.000958, 0.00112, 0.00131, 0.00154, 0.0018
	Black	Men	73	0.00103, 0.00121, 0.00142, 0.00166, 0.00195
	Black	Men	74	0.0011, 0.0013, 0.00153, 0.0018, 0.00212
	Black	Men	75	0.00118, 0.00139, 0.00165, 0.00195, 0.00231
	Black	Men	76	0.00127, 0.00151, 0.00179, 0.00212, 0.00252
	Black	Men	77	0.00138, 0.00164, 0.00195, 0.00232, 0.00276
	Black	Men	78	0.0015, 0.00179, 0.00213, 0.00254, 0.00303
	Black	Men	79	0.00162, 0.00194, 0.00233, 0.00279, 0.00334
	Black	Men	80	0.00174, 0.0021, 0.00253, 0.00305, 0.00368
	Black	Men	81	0.00186, 0.00226, 0.00273, 0.00331, 0.00402
	Black	Men	82	0.00197, 0.00241, 0.00293, 0.00357, 0.00436
	Black	Men	83	0.00208, 0.00255, 0.00312, 0.00382, 0.00468
	Black	Men	84	0.00219, 0.00269, 0.00331, 0.00406, 0.005
	Black	Women	30	0.00000913, 0.0000147, 0.0000236, 0.0000378, 0.0000609
	Black	Women	31	0.0000109, 0.0000173, 0.0000272, 0.000043, 0.0000681
	Black	Women	32	0.0000127, 0.00002, 0.0000313, 0.000049, 0.0000771
	Black	Women	33	0.0000146, 0.0000229, 0.0000358, 0.0000559, 0.0000879
	Black	Women	34	0.0000163, 0.0000258, 0.0000405, 0.0000637, 0.0001
	Black	Women	35	0.0000181, 0.0000287, 0.0000454, 0.0000717, 0.000114
	Black	Women	36	0.0000201, 0.0000319, 0.0000503, 0.0000792, 0.000125
	Black	Women	37	0.0000226, 0.0000353, 0.0000551, 0.0000859, 0.000135
	Black	Women	38	0.0000253, 0.000039, 0.0000599, 0.000092, 0.000142
	Black	Women	39	0.0000281, 0.0000427, 0.0000647, 0.000098, 0.000149

Variable	Race/ethnicity	Sex	Age	Distribution
	Black	Women	40	0.0000307, 0.0000463, 0.0000695, 0.000104, 0.000157
	Black	Women	41	0.0000335, 0.00005, 0.0000743, 0.00011, 0.000165
	Black	Women	42	0.0000369, 0.0000542, 0.0000793, 0.000116, 0.00017
	Black	Women	43	0.0000412, 0.0000592, 0.0000849, 0.000122, 0.000175
	Black	Women	44	0.0000456, 0.0000646, 0.0000912, 0.000129, 0.000182
	Black	Women	45	0.0000505, 0.0000708, 0.0000988, 0.000138, 0.000193
	Black	Women	46	0.0000568, 0.0000784, 0.000108, 0.000148, 0.000205
	Black	Women	47	0.0000651, 0.0000879, 0.000118, 0.00016, 0.000216
	Black	Women	48	0.000075, 0.0000993, 0.000131, 0.000173, 0.000228
	Black	Women	49	0.0000855, 0.000112, 0.000145, 0.000189, 0.000247
	Black	Women	50	0.0000961, 0.000125, 0.000161, 0.000209, 0.000271
	Black	Women	51	0.000108, 0.000139, 0.000179, 0.00023, 0.000297
	Black	Women	52	0.000122, 0.000155, 0.000198, 0.000251, 0.000321
	Black	Women	53	0.000136, 0.000172, 0.000217, 0.000274, 0.000346
	Black	Women	54	0.000149, 0.000188, 0.000237, 0.000298, 0.000376
	Black	Women	55	0.000161, 0.000204, 0.000258, 0.000325, 0.000411
	Black	Women	56	0.000175, 0.000221, 0.000279, 0.000352, 0.000445
	Black	Women	57	0.000193, 0.000242, 0.000303, 0.000378, 0.000474
	Black	Women	58	0.000215, 0.000266, 0.000328, 0.000404, 0.000499
	Black	Women	59	0.000239, 0.000291, 0.000354, 0.00043, 0.000523
	Black	Women	60	0.000261, 0.000315, 0.00038, 0.000457, 0.000552
	Black	Women	61	0.000279, 0.000336, 0.000405, 0.000487, 0.000586
	Black	Women	62	0.000294, 0.000355, 0.000427, 0.000515, 0.000621
	Black	Women	63	0.000308, 0.000372, 0.000448, 0.00054, 0.000652
	Black	Women	64	0.000324, 0.00039, 0.000469, 0.000563, 0.000678
	Black	Women	65	0.000344, 0.000412, 0.000492, 0.000588, 0.000703
	Black	Women	66	0.000368, 0.000438, 0.00052, 0.000618, 0.000735
	Black	Women	67	0.000398, 0.000471, 0.000557, 0.000658, 0.000779
	Black	Women	68	0.000435, 0.000513, 0.000603, 0.00071, 0.000837
	Black	Women	69	0.000481, 0.000564, 0.000661, 0.000775, 0.000909
	Black	Women	70	0.000534, 0.000625, 0.00073, 0.000853, 0.000998
	Black	Women	71	0.000594, 0.000694, 0.00081, 0.000945, 0.00111
	Black	Women	72	0.000658, 0.00077, 0.0009, 0.00105, 0.00123
	Black	Women	73	0.000732, 0.000856, 0.001, 0.00117, 0.00137
	Black	Women	74	0.000817, 0.000954, 0.00111, 0.0013, 0.00152
	Black	Women	75	0.000912, 0.00106, 0.00124, 0.00145, 0.00169
	Black	Women	76	0.00101, 0.00119, 0.00139, 0.00162, 0.0019
	Black	Women	77	0.00111, 0.00132, 0.00156, 0.00184, 0.00218
	Black	Women	78	0.00122, 0.00146, 0.00175, 0.00209, 0.00251
	Black	Women	79	0.00134, 0.00162, 0.00196, 0.00238, 0.00288
	Black	Women	80	0.00147, 0.0018, 0.0022, 0.00268, 0.00328
	Black	Women	81	0.00162, 0.00199, 0.00245, 0.003, 0.0037
	Black	Women	82	0.00176, 0.00218, 0.0027, 0.00334, 0.00414

Variable	Race/ethnicity	Sex	Age	Distribution
	Black	Women	83	0.0019, 0.00237, 0.00296, 0.00368, 0.0046
	Black	Women	84	0.00203, 0.00256, 0.00321, 0.00403, 0.00507
	Hispanic	Men	30	0.00000589, 0.00000892, 0.0000135, 0.0000203, 0.0000307
	Hispanic	Men	31	0.00000725, 0.0000106, 0.0000153, 0.0000223, 0.0000324
	Hispanic	Men	32	0.00000878, 0.0000124, 0.0000175, 0.0000246, 0.0000347
	Hispanic	Men	33	0.0000104, 0.0000144, 0.0000198, 0.0000273, 0.0000378
	Hispanic	Men	34	0.000012, 0.0000164, 0.0000223, 0.0000305, 0.0000417
	Hispanic	Men	35	0.0000135, 0.0000183, 0.0000249, 0.0000338, 0.0000461
	Hispanic	Men	36	0.000015, 0.0000203, 0.0000275, 0.0000372, 0.0000505
	Hispanic	Men	37	0.0000166, 0.0000224, 0.0000301, 0.0000404, 0.0000545
	Hispanic	Men	38	0.0000183, 0.0000244, 0.0000326, 0.0000435, 0.0000582
	Hispanic	Men	39	0.00002, 0.0000265, 0.0000351, 0.0000464, 0.0000616
	Hispanic	Men	40	0.0000218, 0.0000286, 0.0000375, 0.0000492, 0.0000646
	Hispanic	Men	41	0.000024, 0.000031, 0.00004, 0.0000516, 0.0000666
	Hispanic	Men	42	0.0000268, 0.0000338, 0.0000426, 0.0000537, 0.0000678
	Hispanic	Men	43	0.0000297, 0.0000368, 0.0000456, 0.0000564, 0.0000699
	Hispanic	Men	44	0.0000324, 0.0000399, 0.000049, 0.0000602, 0.0000741
	Hispanic	Men	45	0.0000353, 0.0000433, 0.0000531, 0.000065, 0.0000798
	Hispanic	Men	46	0.000039, 0.0000476, 0.0000581, 0.0000707, 0.0000864
	Hispanic	Men	47	0.0000437, 0.0000529, 0.0000641, 0.0000775, 0.000094
	Hispanic	Men	48	0.0000493, 0.0000594, 0.0000714, 0.0000858, 0.000103
	Hispanic	Men	49	0.0000562, 0.0000672, 0.0000802, 0.0000958, 0.000114
	Hispanic	Men	50	0.0000649, 0.0000768, 0.0000908, 0.000107, 0.000127
	Hispanic	Men	51	0.0000756, 0.0000884, 0.000103, 0.000121, 0.000141
	Hispanic	Men	52	0.0000874, 0.000101, 0.000117, 0.000136, 0.000157
	Hispanic	Men	53	0.0000978, 0.000114, 0.000133, 0.000154, 0.00018
	Hispanic	Men	54	0.000106, 0.000126, 0.000149, 0.000176, 0.000208
	Hispanic	Men	55	0.000115, 0.000138, 0.000166, 0.000199, 0.000239
	Hispanic	Men	56	0.000124, 0.000151, 0.000183, 0.000221, 0.000269
	Hispanic	Men	57	0.000135, 0.000165, 0.0002, 0.000243, 0.000296
	Hispanic	Men	58	0.000148, 0.00018, 0.000218, 0.000264, 0.00032
	Hispanic	Men	59	0.000162, 0.000196, 0.000236, 0.000284, 0.000342
	Hispanic	Men	60	0.000177, 0.000212, 0.000253, 0.000303, 0.000362
	Hispanic	Men	61	0.000192, 0.000228, 0.000271, 0.000321, 0.000382
	Hispanic	Men	62	0.000206, 0.000244, 0.000288, 0.00034, 0.000402
	Hispanic	Men	63	0.000219, 0.000258, 0.000304, 0.000358, 0.000423
	Hispanic	Men	64	0.000233, 0.000274, 0.000322, 0.000379, 0.000446
	Hispanic	Men	65	0.000247, 0.000291, 0.000342, 0.000402, 0.000474
	Hispanic	Men	66	0.000264, 0.000312, 0.000367, 0.000432, 0.00051
	Hispanic	Men	67	0.000283, 0.000336, 0.000397, 0.00047, 0.000557
	Hispanic	Men	68	0.000307, 0.000366, 0.000435, 0.000517, 0.000616
	Hispanic	Men	69	0.000336, 0.000402, 0.00048, 0.000573, 0.000685
	Hispanic	Men	70	0.000373, 0.000446, 0.000533, 0.000636, 0.000761

Variable	Race/ethnicity	Sex	Age	Distribution
	Hispanic	Men	71	0.000416, 0.000497, 0.000593, 0.000707, 0.000845
	Hispanic	Men	72	0.000465, 0.000554, 0.00066, 0.000786, 0.000937
	Hispanic	Men	73	0.000518, 0.000617, 0.000735, 0.000874, 0.00104
	Hispanic	Men	74	0.000577, 0.000688, 0.000818, 0.000973, 0.00116
	Hispanic	Men	75	0.000644, 0.000767, 0.000912, 0.00109, 0.00129
	Hispanic	Men	76	0.000719, 0.000858, 0.00102, 0.00121, 0.00145
	Hispanic	Men	77	0.000803, 0.00096, 0.00114, 0.00137, 0.00163
	Hispanic	Men	78	0.000894, 0.00107, 0.00129, 0.00154, 0.00185
	Hispanic	Men	79	0.000992, 0.0012, 0.00145, 0.00174, 0.00211
	Hispanic	Men	80	0.00109, 0.00133, 0.00162, 0.00197, 0.00239
	Hispanic	Men	81	0.0012, 0.00147, 0.0018, 0.00221, 0.00271
	Hispanic	Men	82	0.0013, 0.00161, 0.00199, 0.00246, 0.00305
	Hispanic	Men	83	0.00139, 0.00174, 0.00218, 0.00272, 0.0034
	Hispanic	Men	84	0.00148, 0.00187, 0.00236, 0.00298, 0.00376
	Hispanic	Women	30	0.00000346, 0.00000567, 0.00000924, 0.0000151, 0.0000247
	Hispanic	Women	31	0.0000042, 0.00000664, 0.0000104, 0.0000164, 0.000026
	Hispanic	Women	32	0.00000502, 0.00000773, 0.0000119, 0.0000182, 0.000028
	Hispanic	Women	33	0.0000059, 0.00000893, 0.0000135, 0.0000203, 0.0000307
	Hispanic	Women	34	0.00000684, 0.0000102, 0.0000152, 0.0000226, 0.0000338
	Hispanic	Women	35	0.00000787, 0.0000116, 0.000017, 0.000025, 0.0000369
	Hispanic	Women	36	0.00000899, 0.0000131, 0.0000189, 0.0000274, 0.0000397
	Hispanic	Women	37	0.0000102, 0.0000146, 0.0000207, 0.0000295, 0.0000422
	Hispanic	Women	38	0.0000114, 0.0000161, 0.0000225, 0.0000316, 0.0000445
	Hispanic	Women	39	0.0000127, 0.0000176, 0.0000243, 0.0000336, 0.0000466
	Hispanic	Women	40	0.000014, 0.0000191, 0.000026, 0.0000354, 0.0000484
	Hispanic	Women	41	0.0000155, 0.0000208, 0.0000278, 0.0000371, 0.0000498
	Hispanic	Women	42	0.0000172, 0.0000226, 0.0000296, 0.0000388, 0.0000511
	Hispanic	Women	43	0.0000189, 0.0000245, 0.0000317, 0.000041, 0.0000532
	Hispanic	Women	44	0.0000207, 0.0000266, 0.0000342, 0.0000439, 0.0000565
	Hispanic	Women	45	0.000023, 0.0000292, 0.0000371, 0.0000471, 0.0000599
	Hispanic	Women	46	0.0000261, 0.0000325, 0.0000405, 0.0000504, 0.0000629
	Hispanic	Women	47	0.0000299, 0.0000365, 0.0000445, 0.0000543, 0.0000662
	Hispanic	Women	48	0.0000341, 0.000041, 0.0000493, 0.0000592, 0.0000712
	Hispanic	Women	49	0.0000381, 0.0000458, 0.0000548, 0.0000657, 0.0000788
	Hispanic	Women	50	0.0000421, 0.0000509, 0.0000613, 0.0000739, 0.0000893
	Hispanic	Women	51	0.0000464, 0.0000566, 0.0000688, 0.0000838, 0.000102
	Hispanic	Women	52	0.0000509, 0.0000628, 0.0000772, 0.000095, 0.000117
	Hispanic	Women	53	0.0000555, 0.0000693, 0.0000864, 0.000108, 0.000134
	Hispanic	Women	54	0.0000605, 0.0000764, 0.0000961, 0.000121, 0.000153
	Hispanic	Women	55	0.0000664, 0.0000841, 0.000106, 0.000134, 0.00017
	Hispanic	Women	56	0.0000733, 0.0000927, 0.000117, 0.000148, 0.000187
	Hispanic	Women	57	0.0000808, 0.000102, 0.000128, 0.000161, 0.000204
	Hispanic	Women	58	0.0000887, 0.000112, 0.00014, 0.000176, 0.000221

Variable	Race/ethnicity	Sex	Age	Distribution
	Hispanic	Women	59	0.0000968, 0.000122, 0.000153, 0.000191, 0.00024
	Hispanic	Women	60	0.000105, 0.000132, 0.000165, 0.000207, 0.00026
	Hispanic	Women	61	0.000114, 0.000143, 0.000178, 0.000223, 0.000279
	Hispanic	Women	62	0.000122, 0.000153, 0.000191, 0.000238, 0.000297
	Hispanic	Women	63	0.000131, 0.000163, 0.000203, 0.000253, 0.000315
	Hispanic	Women	64	0.00014, 0.000174, 0.000216, 0.000269, 0.000335
	Hispanic	Women	65	0.000149, 0.000186, 0.000231, 0.000288, 0.000358
	Hispanic	Women	66	0.000161, 0.0002, 0.00025, 0.000311, 0.000388
	Hispanic	Women	67	0.000174, 0.000218, 0.000272, 0.00034, 0.000426
	Hispanic	Women	68	0.000192, 0.000241, 0.000301, 0.000377, 0.000473
	Hispanic	Women	69	0.000215, 0.000269, 0.000337, 0.000421, 0.000528
	Hispanic	Women	70	0.000244, 0.000305, 0.000379, 0.000472, 0.000588
	Hispanic	Women	71	0.000282, 0.000348, 0.000429, 0.000528, 0.000652
	Hispanic	Women	72	0.000327, 0.000399, 0.000485, 0.000591, 0.00072
	Hispanic	Women	73	0.000381, 0.000458, 0.00055, 0.000661, 0.000795
	Hispanic	Women	74	0.000442, 0.000526, 0.000625, 0.000743, 0.000884
	Hispanic	Women	75	0.000509, 0.000602, 0.000712, 0.000841, 0.000995
	Hispanic	Women	76	0.000583, 0.000689, 0.000813, 0.000959, 0.00113
	Hispanic	Women	77	0.000668, 0.00079, 0.000933, 0.0011, 0.0013
	Hispanic	Women	78	0.000766, 0.000907, 0.00107, 0.00127, 0.0015
	Hispanic	Women	79	0.00088, 0.00104, 0.00123, 0.00146, 0.00173
	Hispanic	Women	80	0.00101, 0.00119, 0.00141, 0.00167, 0.00198
	Hispanic	Women	81	0.00113, 0.00135, 0.0016, 0.00191, 0.00228
	Hispanic	Women	82	0.00124, 0.0015, 0.00181, 0.00218, 0.00264
	Hispanic	Women	83	0.00133, 0.00164, 0.00202, 0.00248, 0.00306
	Hispanic	Women	84	0.00142, 0.00178, 0.00223, 0.00279, 0.00351
	White	Men	30	0.00000463, 0.0000069, 0.0000102, 0.0000152, 0.0000227
	White	Men	31	0.00000558, 0.00000806, 0.0000116, 0.0000167, 0.0000241
	White	Men	32	0.00000664, 0.00000935, 0.0000131, 0.0000184, 0.0000259
	White	Men	33	0.0000078, 0.0000107, 0.0000148, 0.0000203, 0.0000279
	White	Men	34	0.00000897, 0.0000122, 0.0000165, 0.0000223, 0.0000303
	White	Men	35	0.0000101, 0.0000136, 0.0000183, 0.0000245, 0.0000331
	White	Men	36	0.0000112, 0.000015, 0.00002, 0.0000268, 0.000036
	White	Men	37	0.0000122, 0.0000163, 0.0000218, 0.0000291, 0.0000389
	White	Men	38	0.0000132, 0.0000176, 0.0000235, 0.0000313, 0.0000418
	White	Men	39	0.0000143, 0.000019, 0.0000252, 0.0000334, 0.0000445
	White	Men	40	0.0000154, 0.0000204, 0.0000268, 0.0000353, 0.0000466
	White	Men	41	0.000017, 0.0000221, 0.0000285, 0.0000369, 0.0000478
	White	Men	42	0.0000191, 0.0000241, 0.0000303, 0.0000382, 0.0000482
	White	Men	43	0.0000214, 0.0000264, 0.0000324, 0.0000399, 0.0000491
	White	Men	44	0.0000237, 0.0000289, 0.000035, 0.0000424, 0.0000516
	White	Men	45	0.0000263, 0.0000317, 0.0000381, 0.0000458, 0.0000552
	White	Men	46	0.0000295, 0.0000352, 0.0000419, 0.0000499, 0.0000595

Variable	Race/ethnicity	Sex	Age	Distribution
	White	Men	47	0.0000332, 0.0000393, 0.0000465, 0.0000549, 0.0000651
	White	Men	48	0.0000372, 0.000044, 0.000052, 0.0000615, 0.0000728
	White	Men	49	0.0000414, 0.0000493, 0.0000587, 0.0000698, 0.0000832
	White	Men	50	0.0000463, 0.0000556, 0.0000666, 0.0000798, 0.0000958
	White	Men	51	0.0000525, 0.0000631, 0.0000759, 0.0000911, 0.00011
	White	Men	52	0.0000602, 0.0000721, 0.0000863, 0.000103, 0.000124
	White	Men	53	0.0000687, 0.0000821, 0.0000978, 0.000117, 0.000139
	White	Men	54	0.0000775, 0.0000924, 0.00011, 0.000131, 0.000157
	White	Men	55	0.0000861, 0.000103, 0.000123, 0.000147, 0.000176
	White	Men	56	0.0000946, 0.000114, 0.000137, 0.000164, 0.000197
	White	Men	57	0.000103, 0.000125, 0.000151, 0.000182, 0.000221
	White	Men	58	0.000112, 0.000136, 0.000166, 0.000202, 0.000247
	White	Men	59	0.000121, 0.000149, 0.000182, 0.000223, 0.000273
	White	Men	60	0.000133, 0.000163, 0.000199, 0.000243, 0.000298
	White	Men	61	0.000146, 0.000178, 0.000216, 0.000263, 0.000319
	White	Men	62	0.000162, 0.000195, 0.000234, 0.000281, 0.000338
	White	Men	63	0.000179, 0.000213, 0.000252, 0.000298, 0.000353
	White	Men	64	0.000199, 0.000232, 0.000271, 0.000316, 0.000368
	White	Men	65	0.00022, 0.000254, 0.000292, 0.000337, 0.000388
	White	Men	66	0.000242, 0.000277, 0.000318, 0.000364, 0.000418
	White	Men	67	0.000266, 0.000305, 0.000349, 0.000401, 0.00046
	White	Men	68	0.000293, 0.000337, 0.000388, 0.000447, 0.000515
	White	Men	69	0.000325, 0.000377, 0.000435, 0.000504, 0.000583
	White	Men	70	0.000364, 0.000423, 0.000491, 0.00057, 0.000662
	White	Men	71	0.000408, 0.000476, 0.000554, 0.000645, 0.000752
	White	Men	72	0.000458, 0.000536, 0.000625, 0.00073, 0.000854
	White	Men	73	0.000511, 0.000601, 0.000705, 0.000827, 0.000971
	White	Men	74	0.00057, 0.000673, 0.000794, 0.000936, 0.00111
	White	Men	75	0.000637, 0.000756, 0.000897, 0.00106, 0.00126
	White	Men	76	0.000714, 0.000853, 0.00102, 0.00121, 0.00145
	White	Men	77	0.000802, 0.000965, 0.00116, 0.00139, 0.00167
	White	Men	78	0.0009, 0.00109, 0.00133, 0.00161, 0.00196
	White	Men	79	0.00101, 0.00124, 0.00152, 0.00187, 0.0023
	White	Men	80	0.00112, 0.0014, 0.00174, 0.00217, 0.00272
	White	Men	81	0.00125, 0.00157, 0.00199, 0.00251, 0.00317
	White	Men	82	0.00139, 0.00177, 0.00224, 0.00285, 0.00363
	White	Men	83	0.00153, 0.00196, 0.00251, 0.00321, 0.00411
	White	Men	84	0.00167, 0.00215, 0.00277, 0.00357, 0.00461
	White	Women	30	0.00000416, 0.00000632, 0.00000956, 0.0000145, 0.0000219
	White	Women	31	0.00000502, 0.00000742, 0.0000109, 0.0000161, 0.0000238
	White	Women	32	0.00000601, 0.00000867, 0.0000125, 0.0000179, 0.0000259
	White	Women	33	0.00000711, 0.00001, 0.0000142, 0.00002, 0.0000282
	White	Women	34	0.00000823, 0.0000115, 0.0000159, 0.0000221, 0.0000308

Variable	Race/ethnicity	Sex	Age	Distribution
	White	Women	35	0.00000932, 0.0000129, 0.0000177, 0.0000244, 0.0000336
	White	Women	36	0.0000104, 0.0000142, 0.0000194, 0.0000265, 0.0000363
	White	Women	37	0.0000114, 0.0000155, 0.000021, 0.0000285, 0.0000388
	White	Women	38	0.0000124, 0.0000168, 0.0000226, 0.0000304, 0.000041
	White	Women	39	0.0000134, 0.000018, 0.000024, 0.000032, 0.0000429
	White	Women	40	0.0000146, 0.0000192, 0.0000254, 0.0000334, 0.0000442
	White	Women	41	0.000016, 0.0000207, 0.0000267, 0.0000344, 0.0000445
	White	Women	42	0.0000179, 0.0000224, 0.0000281, 0.0000352, 0.0000441
	White	Women	43	0.0000199, 0.0000243, 0.0000297, 0.0000362, 0.0000444
	White	Women	44	0.0000216, 0.0000262, 0.0000316, 0.0000382, 0.0000462
	White	Women	45	0.0000234, 0.0000282, 0.000034, 0.0000409, 0.0000493
	White	Women	46	0.0000255, 0.0000307, 0.0000369, 0.0000443, 0.0000533
	White	Women	47	0.000028, 0.0000336, 0.0000403, 0.0000484, 0.0000582
	White	Women	48	0.0000307, 0.000037, 0.0000445, 0.0000536, 0.0000647
	White	Women	49	0.0000336, 0.0000408, 0.0000495, 0.0000601, 0.000073
	White	Women	50	0.000037, 0.0000453, 0.0000554, 0.0000676, 0.0000828
	White	Women	51	0.0000414, 0.0000507, 0.000062, 0.0000759, 0.0000929
	White	Women	52	0.0000468, 0.000057, 0.0000694, 0.0000845, 0.000103
	White	Women	53	0.0000528, 0.0000639, 0.0000773, 0.0000935, 0.000113
	White	Women	54	0.0000591, 0.0000712, 0.0000856, 0.000103, 0.000124
	White	Women	55	0.0000658, 0.0000788, 0.0000941, 0.000112, 0.000135
	White	Women	56	0.000073, 0.0000868, 0.000103, 0.000122, 0.000145
	White	Women	57	0.0000807, 0.0000953, 0.000112, 0.000132, 0.000156
	White	Women	58	0.0000887, 0.000104, 0.000122, 0.000144, 0.000169
	White	Women	59	0.0000973, 0.000114, 0.000134, 0.000156, 0.000183
	White	Women	60	0.000106, 0.000125, 0.000146, 0.00017, 0.000199
	White	Women	61	0.000116, 0.000136, 0.000158, 0.000185, 0.000216
	White	Women	62	0.000126, 0.000147, 0.000172, 0.0002, 0.000234
	White	Women	63	0.000137, 0.00016, 0.000186, 0.000216, 0.000252
	White	Women	64	0.00015, 0.000174, 0.000201, 0.000233, 0.000271
	White	Women	65	0.000164, 0.000189, 0.000218, 0.000252, 0.000292
	White	Women	66	0.000179, 0.000207, 0.000239, 0.000276, 0.000319
	White	Women	67	0.000198, 0.000229, 0.000265, 0.000306, 0.000355
	White	Women	68	0.000221, 0.000257, 0.000297, 0.000345, 0.0004
	White	Women	69	0.00025, 0.000291, 0.000337, 0.000392, 0.000455
	White	Women	70	0.000286, 0.000333, 0.000386, 0.000448, 0.00052
	White	Women	71	0.000329, 0.000382, 0.000443, 0.000514, 0.000597
	White	Women	72	0.000377, 0.000438, 0.000509, 0.000591, 0.000687
	White	Women	73	0.000431, 0.000502, 0.000584, 0.000679, 0.000791
	White	Women	74	0.000494, 0.000575, 0.00067, 0.000779, 0.000908
	White	Women	75	0.000567, 0.000661, 0.000769, 0.000894, 0.00104
	White	Women	76	0.000653, 0.00076, 0.000885, 0.00103, 0.0012
	White	Women	77	0.000749, 0.000876, 0.00102, 0.00119, 0.0014

Variable	Race/ethnicity	Sex	Age	Distribution
	White	Women	78	0.000856, 0.00101, 0.00119, 0.0014, 0.00164
	White	Women	79	0.000978, 0.00116, 0.00138, 0.00164, 0.00195
	White	Women	80	0.00112, 0.00134, 0.00161, 0.00192, 0.00231
	White	Women	81	0.00128, 0.00154, 0.00186, 0.00224, 0.00271
	White	Women	82	0.00145, 0.00176, 0.00214, 0.00259, 0.00315
	White	Women	83	0.00163, 0.00199, 0.00243, 0.00297, 0.00363
	White	Women	84	0.00181, 0.00223, 0.00273, 0.00336, 0.00413
Stroke mortality rates for 2036 (0.01, 0.2, 0.5, 0.8. 0.99 percentiles of the empirical distribution produced during forecasting)				
	Black	Men	30	0.0000125, 0.0000189, 0.0000284, 0.0000427, 0.0000645
	Black	Men	31	0.0000158, 0.000023, 0.0000334, 0.0000484, 0.0000704
	Black	Men	32	0.0000194, 0.0000276, 0.000039, 0.0000552, 0.0000784
	Black	Men	33	0.0000231, 0.0000324, 0.0000452, 0.0000631, 0.0000884
	Black	Men	34	0.0000267, 0.0000372, 0.0000517, 0.0000718, 0.0000999
	Black	Men	35	0.0000302, 0.000042, 0.0000582, 0.0000807, 0.000112
	Black	Men	36	0.0000336, 0.0000466, 0.0000644, 0.0000891, 0.000124
	Black	Men	37	0.0000371, 0.0000511, 0.0000703, 0.0000966, 0.000133
	Black	Men	38	0.0000407, 0.0000556, 0.0000757, 0.000103, 0.000141
	Black	Men	39	0.0000444, 0.0000599, 0.0000807, 0.000109, 0.000147
	Black	Men	40	0.0000484, 0.0000644, 0.0000856, 0.000114, 0.000151
	Black	Men	41	0.0000535, 0.0000697, 0.0000907, 0.000118, 0.000154
	Black	Men	42	0.0000603, 0.0000763, 0.0000964, 0.000122, 0.000154
	Black	Men	43	0.0000675, 0.0000836, 0.000103, 0.000128, 0.000158
	Black	Men	44	0.0000735, 0.0000908, 0.000112, 0.000138, 0.00017
	Black	Men	45	0.0000796, 0.0000988, 0.000122, 0.000152, 0.000188
	Black	Men	46	0.000088, 0.000109, 0.000135, 0.000167, 0.000207
	Black	Men	47	0.0000985, 0.000122, 0.00015, 0.000186, 0.00023
	Black	Men	48	0.00011, 0.000136, 0.000169, 0.00021, 0.000261
	Black	Men	49	0.000123, 0.000154, 0.000192, 0.00024, 0.0003
	Black	Men	50	0.000142, 0.000177, 0.00022, 0.000274, 0.000341
	Black	Men	51	0.000169, 0.000207, 0.000253, 0.00031, 0.00038
	Black	Men	52	0.000202, 0.000242, 0.000291, 0.000348, 0.000418
	Black	Men	53	0.000236, 0.00028, 0.000331, 0.000392, 0.000465
	Black	Men	54	0.000267, 0.000316, 0.000374, 0.000441, 0.000522
	Black	Men	55	0.000297, 0.000352, 0.000416, 0.000492, 0.000582
	Black	Men	56	0.000326, 0.000387, 0.000457, 0.000541, 0.000642
	Black	Men	57	0.000354, 0.00042, 0.000498, 0.000591, 0.000701
	Black	Men	58	0.000383, 0.000454, 0.000538, 0.000638, 0.000757
	Black	Men	59	0.000412, 0.000488, 0.000578, 0.000684, 0.00081
	Black	Men	60	0.000437, 0.000519, 0.000616, 0.000731, 0.000868
	Black	Men	61	0.000456, 0.000545, 0.000651, 0.000777, 0.000929
	Black	Men	62	0.000477, 0.000571, 0.000683, 0.000816, 0.000978
	Black	Men	63	0.000504, 0.0006, 0.000712, 0.000846, 0.00101
	Black	Men	64	0.000536, 0.000631, 0.000741, 0.000872, 0.00103

Variable	Race/ethnicity	Sex	Age	Distribution
	Black	Men	65	0.000565, 0.000662, 0.000774, 0.000906, 0.00106
	Black	Men	66	0.000593, 0.000696, 0.000816, 0.000956, 0.00112
	Black	Men	67	0.000628, 0.000739, 0.000868, 0.00102, 0.0012
	Black	Men	68	0.000674, 0.000794, 0.000933, 0.0011, 0.00129
	Black	Men	69	0.000731, 0.000859, 0.00101, 0.00118, 0.00139
	Black	Men	70	0.000794, 0.000933, 0.00109, 0.00128, 0.00151
	Black	Men	71	0.000861, 0.00101, 0.00119, 0.00139, 0.00163
	Black	Men	72	0.00093, 0.00109, 0.00128, 0.0015, 0.00176
	Black	Men	73	0.000998, 0.00118, 0.00138, 0.00162, 0.00191
	Black	Men	74	0.00107, 0.00126, 0.00149, 0.00176, 0.00208
	Black	Men	75	0.00114, 0.00136, 0.00161, 0.00191, 0.00226
	Black	Men	76	0.00123, 0.00147, 0.00174, 0.00208, 0.00247
	Black	Men	77	0.00134, 0.00159, 0.0019, 0.00227, 0.0027
	Black	Men	78	0.00145, 0.00174, 0.00208, 0.00248, 0.00297
	Black	Men	79	0.00157, 0.00189, 0.00227, 0.00272, 0.00328
	Black	Men	80	0.00169, 0.00204, 0.00247, 0.00298, 0.00361
	Black	Men	81	0.0018, 0.00219, 0.00267, 0.00324, 0.00395
	Black	Men	82	0.00191, 0.00234, 0.00286, 0.00349, 0.00427
	Black	Men	83	0.00202, 0.00248, 0.00304, 0.00374, 0.0046
	Black	Men	84	0.00212, 0.00262, 0.00322, 0.00397, 0.00491
	Black	Women	30	0.00000898, 0.0000146, 0.0000235, 0.0000379, 0.0000616
	Black	Women	31	0.0000107, 0.0000171, 0.0000272, 0.0000431, 0.0000687
	Black	Women	32	0.0000126, 0.0000199, 0.0000312, 0.0000491, 0.0000776
	Black	Women	33	0.0000144, 0.0000227, 0.0000357, 0.0000561, 0.0000884
	Black	Women	34	0.0000162, 0.0000256, 0.0000404, 0.0000638, 0.000101
	Black	Women	35	0.0000179, 0.0000286, 0.0000453, 0.0000717, 0.000114
	Black	Women	36	0.0000199, 0.0000316, 0.0000501, 0.0000792, 0.000126
	Black	Women	37	0.0000222, 0.000035, 0.0000548, 0.0000858, 0.000135
	Black	Women	38	0.0000249, 0.0000386, 0.0000595, 0.0000917, 0.000142
	Black	Women	39	0.0000275, 0.0000421, 0.0000641, 0.0000976, 0.000149
	Black	Women	40	0.00003, 0.0000455, 0.0000687, 0.000104, 0.000157
	Black	Women	41	0.0000327, 0.000049, 0.0000733, 0.00011, 0.000164
	Black	Women	42	0.000036, 0.0000532, 0.0000781, 0.000115, 0.000169
	Black	Women	43	0.0000401, 0.000058, 0.0000834, 0.00012, 0.000173
	Black	Women	44	0.0000444, 0.0000632, 0.0000896, 0.000127, 0.000181
	Black	Women	45	0.0000492, 0.0000691, 0.0000969, 0.000136, 0.000191
	Black	Women	46	0.0000553, 0.0000765, 0.000106, 0.000146, 0.000202
	Black	Women	47	0.0000634, 0.0000859, 0.000116, 0.000157, 0.000213
	Black	Women	48	0.0000731, 0.000097, 0.000128, 0.00017, 0.000225
	Black	Women	49	0.0000834, 0.000109, 0.000143, 0.000186, 0.000244
	Black	Women	50	0.0000937, 0.000122, 0.000159, 0.000206, 0.000268
	Black	Women	51	0.000105, 0.000136, 0.000176, 0.000227, 0.000294
	Black	Women	52	0.000119, 0.000152, 0.000195, 0.000248, 0.000318

Variable	Race/ethnicity	Sex	Age	Distribution
	Black	Women	53	0.000133, 0.000169, 0.000214, 0.00027, 0.000343
	Black	Women	54	0.000146, 0.000185, 0.000234, 0.000295, 0.000373
	Black	Women	55	0.000158, 0.000201, 0.000254, 0.000322, 0.000408
	Black	Women	56	0.000172, 0.000218, 0.000276, 0.000349, 0.000442
	Black	Women	57	0.000189, 0.000238, 0.000299, 0.000375, 0.000471
	Black	Women	58	0.000211, 0.000261, 0.000323, 0.0004, 0.000495
	Black	Women	59	0.000234, 0.000286, 0.000349, 0.000425, 0.000519
	Black	Women	60	0.000256, 0.00031, 0.000374, 0.000452, 0.000547
	Black	Women	61	0.000273, 0.00033, 0.000398, 0.00048, 0.000581
	Black	Women	62	0.000287, 0.000348, 0.00042, 0.000508, 0.000614
	Black	Women	63	0.000301, 0.000364, 0.00044, 0.000532, 0.000643
	Black	Women	64	0.000317, 0.000382, 0.00046, 0.000554, 0.000668
	Black	Women	65	0.000335, 0.000402, 0.000482, 0.000577, 0.000692
	Black	Women	66	0.000358, 0.000427, 0.000509, 0.000606, 0.000723
	Black	Women	67	0.000387, 0.000459, 0.000544, 0.000645, 0.000766
	Black	Women	68	0.000423, 0.0005, 0.00059, 0.000696, 0.000823
	Black	Women	69	0.000467, 0.00055, 0.000646, 0.000759, 0.000894
	Black	Women	70	0.000519, 0.000609, 0.000713, 0.000836, 0.000981
	Black	Women	71	0.000576, 0.000676, 0.000791, 0.000926, 0.00109
	Black	Women	72	0.000639, 0.00075, 0.000879, 0.00103, 0.00121
	Black	Women	73	0.00071, 0.000833, 0.000976, 0.00114, 0.00134
	Black	Women	74	0.000792, 0.000928, 0.00109, 0.00127, 0.00149
	Black	Women	75	0.000884, 0.00104, 0.00121, 0.00141, 0.00166
	Black	Women	76	0.000981, 0.00115, 0.00135, 0.00159, 0.00187
	Black	Women	77	0.00108, 0.00128, 0.00152, 0.0018, 0.00213
	Black	Women	78	0.00118, 0.00142, 0.00171, 0.00205, 0.00246
	Black	Women	79	0.0013, 0.00158, 0.00191, 0.00232, 0.00282
	Black	Women	80	0.00143, 0.00175, 0.00214, 0.00262, 0.00322
	Black	Women	81	0.00157, 0.00194, 0.00238, 0.00294, 0.00362
	Black	Women	82	0.00171, 0.00212, 0.00263, 0.00326, 0.00405
	Black	Women	83	0.00184, 0.0023, 0.00288, 0.0036, 0.00451
	Black	Women	84	0.00197, 0.00248, 0.00313, 0.00394, 0.00497
	Hispanic	Men	30	0.00000578, 0.00000882, 0.0000134, 0.0000203, 0.000031
	Hispanic	Men	31	0.00000714, 0.0000105, 0.0000153, 0.0000223, 0.0000327
	Hispanic	Men	32	0.00000865, 0.0000123, 0.0000174, 0.0000246, 0.000035
	Hispanic	Men	33	0.0000102, 0.0000142, 0.0000197, 0.0000274, 0.000038
	Hispanic	Men	34	0.0000118, 0.0000162, 0.0000222, 0.0000305, 0.0000419
	Hispanic	Men	35	0.0000133, 0.0000182, 0.0000248, 0.0000339, 0.0000464
	Hispanic	Men	36	0.0000147, 0.0000201, 0.0000274, 0.0000372, 0.0000508
	Hispanic	Men	37	0.0000163, 0.0000221, 0.0000299, 0.0000404, 0.0000547
	Hispanic	Men	38	0.0000179, 0.0000241, 0.0000323, 0.0000434, 0.0000583
	Hispanic	Men	39	0.0000195, 0.0000261, 0.0000347, 0.0000462, 0.0000616
	Hispanic	Men	40	0.0000213, 0.0000281, 0.000037, 0.0000488, 0.0000645

Variable	Race/ethnicity	Sex	Age	Distribution
	Hispanic	Men	41	0.0000234, 0.0000304, 0.0000394, 0.000051, 0.0000662
	Hispanic	Men	42	0.0000261, 0.0000331, 0.0000419, 0.0000529, 0.0000671
	Hispanic	Men	43	0.0000289, 0.000036, 0.0000447, 0.0000554, 0.0000689
	Hispanic	Men	44	0.0000315, 0.0000389, 0.000048, 0.0000591, 0.0000729
	Hispanic	Men	45	0.0000344, 0.0000423, 0.0000519, 0.0000637, 0.0000784
	Hispanic	Men	46	0.000038, 0.0000465, 0.0000567, 0.0000693, 0.0000847
	Hispanic	Men	47	0.0000425, 0.0000516, 0.0000626, 0.0000759, 0.0000921
	Hispanic	Men	48	0.000048, 0.0000579, 0.0000697, 0.000084, 0.000101
	Hispanic	Men	49	0.0000548, 0.0000656, 0.0000785, 0.0000938, 0.000112
	Hispanic	Men	50	0.0000633, 0.0000751, 0.0000889, 0.000105, 0.000125
	Hispanic	Men	51	0.0000739, 0.0000865, 0.000101, 0.000118, 0.000139
	Hispanic	Men	52	0.0000855, 0.0000993, 0.000115, 0.000133, 0.000155
	Hispanic	Men	53	0.0000958, 0.000112, 0.00013, 0.000152, 0.000177
	Hispanic	Men	54	0.000104, 0.000124, 0.000146, 0.000173, 0.000206
	Hispanic	Men	55	0.000113, 0.000136, 0.000163, 0.000196, 0.000236
	Hispanic	Men	56	0.000122, 0.000148, 0.00018, 0.000219, 0.000266
	Hispanic	Men	57	0.000133, 0.000162, 0.000197, 0.00024, 0.000293
	Hispanic	Men	58	0.000145, 0.000177, 0.000215, 0.000261, 0.000317
	Hispanic	Men	59	0.000159, 0.000192, 0.000232, 0.00028, 0.000339
	Hispanic	Men	60	0.000174, 0.000208, 0.000249, 0.000299, 0.000358
	Hispanic	Men	61	0.000188, 0.000224, 0.000266, 0.000317, 0.000377
	Hispanic	Men	62	0.000201, 0.000239, 0.000283, 0.000335, 0.000397
	Hispanic	Men	63	0.000214, 0.000253, 0.000299, 0.000353, 0.000417
	Hispanic	Men	64	0.000227, 0.000268, 0.000316, 0.000372, 0.000439
	Hispanic	Men	65	0.000241, 0.000284, 0.000335, 0.000395, 0.000466
	Hispanic	Men	66	0.000257, 0.000304, 0.000359, 0.000424, 0.000501
	Hispanic	Men	67	0.000276, 0.000327, 0.000388, 0.00046, 0.000547
	Hispanic	Men	68	0.000298, 0.000356, 0.000425, 0.000507, 0.000605
	Hispanic	Men	69	0.000327, 0.000392, 0.000469, 0.000561, 0.000673
	Hispanic	Men	70	0.000362, 0.000435, 0.00052, 0.000623, 0.000747
	Hispanic	Men	71	0.000404, 0.000484, 0.000579, 0.000692, 0.000829
	Hispanic	Men	72	0.000451, 0.00054, 0.000644, 0.000769, 0.000919
	Hispanic	Men	73	0.000503, 0.000601, 0.000717, 0.000855, 0.00102
	Hispanic	Men	74	0.00056, 0.000669, 0.000798, 0.000951, 0.00114
	Hispanic	Men	75	0.000625, 0.000746, 0.00089, 0.00106, 0.00127
	Hispanic	Men	76	0.000698, 0.000834, 0.000995, 0.00119, 0.00142
	Hispanic	Men	77	0.000779, 0.000933, 0.00112, 0.00134, 0.0016
	Hispanic	Men	78	0.000868, 0.00104, 0.00125, 0.00151, 0.00181
	Hispanic	Men	79	0.000962, 0.00117, 0.00141, 0.0017, 0.00207
	Hispanic	Men	80	0.00106, 0.0013, 0.00158, 0.00192, 0.00235
	Hispanic	Men	81	0.00116, 0.00143, 0.00176, 0.00216, 0.00266
	Hispanic	Men	82	0.00126, 0.00156, 0.00194, 0.0024, 0.00299
	Hispanic	Men	83	0.00135, 0.00169, 0.00212, 0.00266, 0.00334

Variable	Race/ethnicity	Sex	Age	Distribution
	Hispanic	Men	84	0.00144, 0.00182, 0.0023, 0.00291, 0.00368
	Hispanic	Women	30	0.00000341, 0.00000561, 0.0000092, 0.0000151, 0.0000249
	Hispanic	Women	31	0.00000414, 0.00000658, 0.0000104, 0.0000165, 0.0000261
	Hispanic	Women	32	0.00000495, 0.00000767, 0.0000118, 0.0000182, 0.0000282
	Hispanic	Women	33	0.00000583, 0.00000886, 0.0000134, 0.0000203, 0.0000309
	Hispanic	Women	34	0.00000676, 0.0000101, 0.0000152, 0.0000226, 0.000034
	Hispanic	Women	35	0.00000777, 0.0000115, 0.000017, 0.000025, 0.0000371
	Hispanic	Women	36	0.00000887, 0.0000129, 0.0000188, 0.0000273, 0.0000399
	Hispanic	Women	37	0.00001, 0.0000144, 0.0000206, 0.0000295, 0.0000423
	Hispanic	Women	38	0.0000112, 0.0000159, 0.0000223, 0.0000315, 0.0000445
	Hispanic	Women	39	0.0000124, 0.0000173, 0.000024, 0.0000334, 0.0000465
	Hispanic	Women	40	0.0000137, 0.0000188, 0.0000257, 0.0000351, 0.0000482
	Hispanic	Women	41	0.0000151, 0.0000204, 0.0000273, 0.0000367, 0.0000494
	Hispanic	Women	42	0.0000168, 0.0000221, 0.0000291, 0.0000383, 0.0000505
	Hispanic	Women	43	0.0000184, 0.000024, 0.0000311, 0.0000403, 0.0000524
	Hispanic	Women	44	0.0000201, 0.000026, 0.0000334, 0.000043, 0.0000555
	Hispanic	Women	45	0.0000224, 0.0000285, 0.0000363, 0.0000461, 0.0000588
	Hispanic	Women	46	0.0000254, 0.0000317, 0.0000396, 0.0000494, 0.0000617
	Hispanic	Women	47	0.0000291, 0.0000356, 0.0000435, 0.0000531, 0.0000649
	Hispanic	Women	48	0.0000332, 0.00004, 0.0000481, 0.0000579, 0.0000698
	Hispanic	Women	49	0.0000372, 0.0000447, 0.0000536, 0.0000643, 0.0000774
	Hispanic	Women	50	0.0000411, 0.0000498, 0.0000601, 0.0000725, 0.0000877
	Hispanic	Women	51	0.0000454, 0.0000554, 0.0000675, 0.0000822, 0.0001
	Hispanic	Women	52	0.0000499, 0.0000615, 0.0000758, 0.0000933, 0.000115
	Hispanic	Women	53	0.0000545, 0.0000681, 0.0000849, 0.000106, 0.000132
	Hispanic	Women	54	0.0000594, 0.000075, 0.0000946, 0.000119, 0.000151
	Hispanic	Women	55	0.0000652, 0.0000827, 0.000105, 0.000133, 0.000168
	Hispanic	Women	56	0.000072, 0.0000912, 0.000115, 0.000146, 0.000185
	Hispanic	Women	57	0.0000794, 0.0001, 0.000126, 0.000159, 0.000201
	Hispanic	Women	58	0.0000871, 0.00011, 0.000138, 0.000174, 0.000219
	Hispanic	Women	59	0.000095, 0.00012, 0.00015, 0.000189, 0.000238
	Hispanic	Women	60	0.000103, 0.00013, 0.000163, 0.000204, 0.000257
	Hispanic	Women	61	0.000111, 0.00014, 0.000175, 0.00022, 0.000276
	Hispanic	Women	62	0.00012, 0.00015, 0.000187, 0.000234, 0.000293
	Hispanic	Women	63	0.000128, 0.00016, 0.000199, 0.000249, 0.000311
	Hispanic	Women	64	0.000136, 0.00017, 0.000212, 0.000264, 0.00033
	Hispanic	Women	65	0.000146, 0.000182, 0.000226, 0.000282, 0.000352
	Hispanic	Women	66	0.000157, 0.000196, 0.000244, 0.000304, 0.000381
	Hispanic	Women	67	0.00017, 0.000213, 0.000266, 0.000333, 0.000418
	Hispanic	Women	68	0.000187, 0.000235, 0.000294, 0.000369, 0.000464
	Hispanic	Women	69	0.000209, 0.000262, 0.000329, 0.000412, 0.000518
	Hispanic	Women	70	0.000238, 0.000297, 0.00037, 0.000462, 0.000577
	Hispanic	Women	71	0.000274, 0.000339, 0.000418, 0.000517, 0.000639

Variable	Race/ethnicity	Sex	Age	Distribution
	Hispanic	Women	72	0.000318, 0.000389, 0.000474, 0.000578, 0.000706
	Hispanic	Women	73	0.00037, 0.000446, 0.000537, 0.000646, 0.00078
	Hispanic	Women	74	0.000429, 0.000512, 0.00061, 0.000726, 0.000867
	Hispanic	Women	75	0.000494, 0.000586, 0.000694, 0.000822, 0.000975
	Hispanic	Women	76	0.000566, 0.00067, 0.000793, 0.000938, 0.00111
	Hispanic	Women	77	0.000648, 0.000768, 0.000909, 0.00108, 0.00128
	Hispanic	Women	78	0.000743, 0.000882, 0.00105, 0.00124, 0.00147
	Hispanic	Women	79	0.000854, 0.00101, 0.0012, 0.00142, 0.00169
	Hispanic	Women	80	0.000974, 0.00116, 0.00138, 0.00163, 0.00194
	Hispanic	Women	81	0.00109, 0.00131, 0.00156, 0.00187, 0.00224
	Hispanic	Women	82	0.0012, 0.00146, 0.00176, 0.00214, 0.00259
	Hispanic	Women	83	0.00129, 0.00159, 0.00197, 0.00243, 0.003
	Hispanic	Women	84	0.00137, 0.00173, 0.00217, 0.00273, 0.00344
	White	Men	30	0.00000454, 0.00000682, 0.0000102, 0.0000153, 0.0000229
	White	Men	31	0.00000549, 0.00000798, 0.0000116, 0.0000167, 0.0000243
	White	Men	32	0.00000654, 0.00000926, 0.0000131, 0.0000184, 0.0000261
	White	Men	33	0.00000769, 0.0000106, 0.0000147, 0.0000203, 0.0000281
	White	Men	34	0.00000884, 0.0000121, 0.0000164, 0.0000224, 0.0000305
	White	Men	35	0.00000994, 0.0000135, 0.0000182, 0.0000246, 0.0000333
	White	Men	36	0.000011, 0.0000148, 0.0000199, 0.0000268, 0.0000362
	White	Men	37	0.000012, 0.0000161, 0.0000216, 0.000029, 0.0000391
	White	Men	38	0.0000129, 0.0000174, 0.0000233, 0.0000312, 0.0000419
	White	Men	39	0.0000139, 0.0000186, 0.0000249, 0.0000332, 0.0000444
	White	Men	40	0.0000151, 0.00002, 0.0000265, 0.000035, 0.0000464
	White	Men	41	0.0000166, 0.0000216, 0.000028, 0.0000364, 0.0000474
	White	Men	42	0.0000186, 0.0000236, 0.0000298, 0.0000376, 0.0000477
	White	Men	43	0.0000209, 0.0000258, 0.0000318, 0.0000392, 0.0000484
	White	Men	44	0.0000231, 0.0000281, 0.0000342, 0.0000416, 0.0000507
	White	Men	45	0.0000256, 0.0000309, 0.0000372, 0.0000449, 0.0000542
	White	Men	46	0.0000286, 0.0000342, 0.0000409, 0.0000488, 0.0000584
	White	Men	47	0.0000322, 0.0000383, 0.0000454, 0.0000538, 0.0000638
	White	Men	48	0.0000362, 0.0000429, 0.0000508, 0.0000602, 0.0000714
	White	Men	49	0.0000403, 0.0000481, 0.0000574, 0.0000683, 0.0000816
	White	Men	50	0.0000451, 0.0000543, 0.0000652, 0.0000782, 0.0000941
	White	Men	51	0.0000512, 0.0000618, 0.0000743, 0.0000894, 0.000108
	White	Men	52	0.0000588, 0.0000706, 0.0000847, 0.000102, 0.000122
	White	Men	53	0.0000673, 0.0000805, 0.0000961, 0.000115, 0.000137
	White	Men	54	0.0000759, 0.0000907, 0.000108, 0.000129, 0.000155
	White	Men	55	0.0000844, 0.000101, 0.000121, 0.000145, 0.000174
	White	Men	56	0.0000927, 0.000112, 0.000134, 0.000162, 0.000195
	White	Men	57	0.000101, 0.000123, 0.000148, 0.00018, 0.000219
	White	Men	58	0.000109, 0.000134, 0.000163, 0.0002, 0.000244
	White	Men	59	0.000119, 0.000146, 0.000179, 0.00022, 0.00027

Variable	Race/ethnicity	Sex	Age	Distribution
	White	Men	60	0.00013, 0.00016, 0.000196, 0.00024, 0.000294
	White	Men	61	0.000143, 0.000175, 0.000213, 0.000259, 0.000316
	White	Men	62	0.000158, 0.000191, 0.00023, 0.000276, 0.000333
	White	Men	63	0.000175, 0.000208, 0.000247, 0.000293, 0.000348
	White	Men	64	0.000194, 0.000227, 0.000265, 0.00031, 0.000363
	White	Men	65	0.000214, 0.000247, 0.000286, 0.00033, 0.000382
	White	Men	66	0.000235, 0.000271, 0.000311, 0.000357, 0.000411
	White	Men	67	0.000258, 0.000297, 0.000341, 0.000393, 0.000452
	White	Men	68	0.000284, 0.000329, 0.000379, 0.000438, 0.000506
	White	Men	69	0.000316, 0.000367, 0.000425, 0.000493, 0.000573
	White	Men	70	0.000353, 0.000412, 0.000479, 0.000558, 0.000651
	White	Men	71	0.000396, 0.000464, 0.000541, 0.000632, 0.000739
	White	Men	72	0.000444, 0.000521, 0.000611, 0.000715, 0.000839
	White	Men	73	0.000496, 0.000585, 0.000688, 0.000809, 0.000953
	White	Men	74	0.000553, 0.000655, 0.000775, 0.000916, 0.00109
	White	Men	75	0.000618, 0.000736, 0.000875, 0.00104, 0.00124
	White	Men	76	0.000693, 0.00083, 0.000991, 0.00118, 0.00142
	White	Men	77	0.000778, 0.000939, 0.00113, 0.00136, 0.00164
	White	Men	78	0.000873, 0.00106, 0.00129, 0.00157, 0.00192
	White	Men	79	0.000975, 0.0012, 0.00148, 0.00183, 0.00226
	White	Men	80	0.00108, 0.00136, 0.0017, 0.00213, 0.00267
	White	Men	81	0.00121, 0.00153, 0.00194, 0.00245, 0.00311
	White	Men	82	0.00135, 0.00172, 0.00219, 0.00279, 0.00356
	White	Men	83	0.00149, 0.00191, 0.00245, 0.00314, 0.00403
	White	Men	84	0.00162, 0.00209, 0.00271, 0.00349, 0.00452
	White	Women	30	0.00000409, 0.00000625, 0.00000952, 0.0000145, 0.0000221
	White	Women	31	0.00000494, 0.00000735, 0.0000109, 0.0000161, 0.000024
	White	Women	32	0.00000592, 0.0000086, 0.0000124, 0.000018, 0.0000261
	White	Women	33	0.00000701, 0.00000997, 0.0000141, 0.00002, 0.0000284
	White	Women	34	0.00000812, 0.0000114, 0.0000159, 0.0000222, 0.000031
	White	Women	35	0.00000919, 0.0000127, 0.0000176, 0.0000244, 0.0000338
	White	Women	36	0.0000102, 0.0000141, 0.0000193, 0.0000265, 0.0000365
	White	Women	37	0.0000112, 0.0000153, 0.0000209, 0.0000285, 0.0000389
	White	Women	38	0.0000122, 0.0000165, 0.0000224, 0.0000303, 0.0000411
	White	Women	39	0.0000131, 0.0000177, 0.0000237, 0.0000319, 0.0000429
	White	Women	40	0.0000142, 0.0000189, 0.000025, 0.0000331, 0.000044
	White	Women	41	0.0000156, 0.0000203, 0.0000263, 0.000034, 0.0000442
	White	Women	42	0.0000174, 0.000022, 0.0000276, 0.0000347, 0.0000436
	White	Women	43	0.0000194, 0.0000238, 0.0000291, 0.0000356, 0.0000437
	White	Women	44	0.0000211, 0.0000255, 0.0000309, 0.0000375, 0.0000455
	White	Women	45	0.0000228, 0.0000275, 0.0000332, 0.0000401, 0.0000485
	White	Women	46	0.0000248, 0.0000299, 0.000036, 0.0000434, 0.0000523
	White	Women	47	0.0000272, 0.0000328, 0.0000394, 0.0000474, 0.0000571

Variable	Race/ethnicity	Sex	Age	Distribution
	White	Women	48	0.0000299, 0.0000361, 0.0000435, 0.0000525, 0.0000635
	White	Women	49	0.0000327, 0.0000399, 0.0000484, 0.0000589, 0.0000717
	White	Women	50	0.0000362, 0.0000443, 0.0000542, 0.0000663, 0.0000813
	White	Women	51	0.0000405, 0.0000497, 0.0000608, 0.0000744, 0.0000913
	White	Women	52	0.0000458, 0.0000559, 0.0000681, 0.000083, 0.000101
	White	Women	53	0.0000518, 0.0000628, 0.000076, 0.000092, 0.000112
	White	Women	54	0.000058, 0.0000699, 0.0000842, 0.000101, 0.000122
	White	Women	55	0.0000646, 0.0000774, 0.0000926, 0.000111, 0.000133
	White	Women	56	0.0000717, 0.0000853, 0.000101, 0.00012, 0.000143
	White	Women	57	0.0000791, 0.0000937, 0.000111, 0.000131, 0.000155
	White	Women	58	0.000087, 0.000103, 0.000121, 0.000142, 0.000167
	White	Women	59	0.0000954, 0.000112, 0.000132, 0.000154, 0.000181
	White	Women	60	0.000104, 0.000122, 0.000143, 0.000168, 0.000197
	White	Women	61	0.000114, 0.000133, 0.000156, 0.000183, 0.000214
	White	Women	62	0.000123, 0.000144, 0.000169, 0.000198, 0.000231
	White	Women	63	0.000134, 0.000156, 0.000183, 0.000213, 0.000249
	White	Women	64	0.000146, 0.00017, 0.000197, 0.000229, 0.000267
	White	Women	65	0.000159, 0.000185, 0.000214, 0.000248, 0.000287
	White	Women	66	0.000175, 0.000202, 0.000234, 0.000271, 0.000313
	White	Women	67	0.000193, 0.000223, 0.000259, 0.0003, 0.000348
	White	Women	68	0.000215, 0.00025, 0.000291, 0.000338, 0.000393
	White	Women	69	0.000243, 0.000283, 0.00033, 0.000384, 0.000447
	White	Women	70	0.000278, 0.000324, 0.000377, 0.000438, 0.000511
	White	Women	71	0.000319, 0.000372, 0.000432, 0.000503, 0.000586
	White	Women	72	0.000366, 0.000427, 0.000497, 0.000578, 0.000674
	White	Women	73	0.000418, 0.000489, 0.00057, 0.000664, 0.000776
	White	Women	74	0.000479, 0.00056, 0.000653, 0.000762, 0.000891
	White	Women	75	0.00055, 0.000643, 0.000749, 0.000874, 0.00102
	White	Women	76	0.000633, 0.000739, 0.000862, 0.00101, 0.00118
	White	Women	77	0.000726, 0.000851, 0.000997, 0.00117, 0.00137
	White	Women	78	0.00083, 0.000981, 0.00116, 0.00136, 0.00161
	White	Women	79	0.000948, 0.00113, 0.00135, 0.0016, 0.00191
	White	Women	80	0.00108, 0.0013, 0.00157, 0.00188, 0.00226
	White	Women	81	0.00124, 0.0015, 0.00181, 0.00219, 0.00265
	White	Women	82	0.00141, 0.00171, 0.00208, 0.00253, 0.00309
	White	Women	83	0.00158, 0.00194, 0.00237, 0.0029, 0.00356
	White	Women	84	0.00176, 0.00217, 0.00267, 0.00328, 0.00405
Stroke mortality rates for 2037 (0.01, 0.2, 0.5, 0.8. 0.99 percentiles of the empirical distribution produced during forecasting)				
	Black	Men	30	0.0000123, 0.0000187, 0.0000283, 0.0000428, 0.0000651
	Black	Men	31	0.0000156, 0.0000228, 0.0000332, 0.0000485, 0.000071
	Black	Men	32	0.0000191, 0.0000273, 0.0000389, 0.0000553, 0.000079
	Black	Men	33	0.0000228, 0.0000321, 0.000045, 0.0000632, 0.000089
	Black	Men	34	0.0000264, 0.0000369, 0.0000515, 0.0000718, 0.000101

Variable	Race/ethnicity	Sex	Age	Distribution
	Black	Men	35	0.0000298, 0.0000416, 0.0000579, 0.0000807, 0.000113
	Black	Men	36	0.0000331, 0.0000461, 0.0000641, 0.0000891, 0.000124
	Black	Men	37	0.0000364, 0.0000505, 0.0000698, 0.0000964, 0.000134
	Black	Men	38	0.0000399, 0.0000548, 0.000075, 0.000103, 0.000141
	Black	Men	39	0.0000434, 0.0000589, 0.0000798, 0.000108, 0.000147
	Black	Men	40	0.0000472, 0.0000632, 0.0000844, 0.000113, 0.000151
	Black	Men	41	0.0000522, 0.0000683, 0.0000892, 0.000117, 0.000153
	Black	Men	42	0.0000588, 0.0000747, 0.0000947, 0.00012, 0.000153
	Black	Men	43	0.0000658, 0.0000817, 0.000101, 0.000126, 0.000156
	Black	Men	44	0.0000716, 0.0000886, 0.00011, 0.000135, 0.000168
	Black	Men	45	0.0000775, 0.0000964, 0.00012, 0.000148, 0.000185
	Black	Men	46	0.0000856, 0.000106, 0.000132, 0.000164, 0.000203
	Black	Men	47	0.0000959, 0.000119, 0.000147, 0.000182, 0.000225
	Black	Men	48	0.000107, 0.000133, 0.000165, 0.000205, 0.000255
	Black	Men	49	0.00012, 0.00015, 0.000188, 0.000235, 0.000294
	Black	Men	50	0.000139, 0.000173, 0.000215, 0.000268, 0.000335
	Black	Men	51	0.000165, 0.000202, 0.000248, 0.000304, 0.000373
	Black	Men	52	0.000198, 0.000238, 0.000285, 0.000342, 0.000412
	Black	Men	53	0.000231, 0.000275, 0.000326, 0.000386, 0.000459
	Black	Men	54	0.000262, 0.000311, 0.000368, 0.000435, 0.000516
	Black	Men	55	0.000292, 0.000346, 0.00041, 0.000485, 0.000575
	Black	Men	56	0.00032, 0.00038, 0.000451, 0.000534, 0.000635
	Black	Men	57	0.000347, 0.000413, 0.000491, 0.000583, 0.000694
	Black	Men	58	0.000375, 0.000447, 0.00053, 0.00063, 0.000749
	Black	Men	59	0.000404, 0.00048, 0.000569, 0.000675, 0.000802
	Black	Men	60	0.000428, 0.00051, 0.000606, 0.000721, 0.00086
	Black	Men	61	0.000446, 0.000535, 0.000641, 0.000767, 0.000919
	Black	Men	62	0.000466, 0.00056, 0.000671, 0.000804, 0.000966
	Black	Men	63	0.000492, 0.000587, 0.000699, 0.000832, 0.000992
	Black	Men	64	0.000522, 0.000617, 0.000727, 0.000856, 0.00101
	Black	Men	65	0.000551, 0.000647, 0.000758, 0.000889, 0.00104
	Black	Men	66	0.000577, 0.000679, 0.000798, 0.000937, 0.0011
	Black	Men	67	0.000611, 0.00072, 0.000848, 0.000999, 0.00118
	Black	Men	68	0.000655, 0.000773, 0.000911, 0.00107, 0.00127
	Black	Men	69	0.00071, 0.000837, 0.000985, 0.00116, 0.00137
	Black	Men	70	0.000771, 0.000908, 0.00107, 0.00126, 0.00148
	Black	Men	71	0.000836, 0.000984, 0.00116, 0.00136, 0.0016
	Black	Men	72	0.000902, 0.00106, 0.00125, 0.00147, 0.00173
	Black	Men	73	0.000969, 0.00114, 0.00135, 0.00159, 0.00187
	Black	Men	74	0.00103, 0.00123, 0.00145, 0.00172, 0.00204
	Black	Men	75	0.00111, 0.00132, 0.00157, 0.00186, 0.00222
	Black	Men	76	0.00119, 0.00143, 0.0017, 0.00203, 0.00242
	Black	Men	77	0.0013, 0.00155, 0.00185, 0.00221, 0.00265

Variable	Race/ethnicity	Sex	Age	Distribution
	Black	Men	78	0.00141, 0.00169, 0.00202, 0.00243, 0.00291
	Black	Men	79	0.00152, 0.00184, 0.00221, 0.00266, 0.00321
	Black	Men	80	0.00163, 0.00198, 0.00241, 0.00291, 0.00354
	Black	Men	81	0.00175, 0.00213, 0.0026, 0.00317, 0.00387
	Black	Men	82	0.00185, 0.00228, 0.00279, 0.00342, 0.00419
	Black	Men	83	0.00195, 0.00241, 0.00297, 0.00365, 0.00451
	Black	Men	84	0.00205, 0.00254, 0.00314, 0.00389, 0.00482
	Black	Women	30	0.00000884, 0.0000144, 0.0000234, 0.0000381, 0.0000622
	Black	Women	31	0.0000106, 0.000017, 0.0000271, 0.0000432, 0.0000693
	Black	Women	32	0.0000124, 0.0000197, 0.0000312, 0.0000493, 0.0000782
	Black	Women	33	0.0000143, 0.0000226, 0.0000356, 0.0000562, 0.000089
	Black	Women	34	0.000016, 0.0000255, 0.0000403, 0.0000638, 0.000102
	Black	Women	35	0.0000177, 0.0000284, 0.0000451, 0.0000718, 0.000115
	Black	Women	36	0.0000197, 0.0000314, 0.0000499, 0.0000792, 0.000126
	Black	Women	37	0.0000219, 0.0000347, 0.0000545, 0.0000857, 0.000135
	Black	Women	38	0.0000245, 0.0000381, 0.000059, 0.0000914, 0.000142
	Black	Women	39	0.000027, 0.0000415, 0.0000635, 0.0000971, 0.000149
	Black	Women	40	0.0000294, 0.0000448, 0.0000679, 0.000103, 0.000157
	Black	Women	41	0.0000319, 0.0000482, 0.0000723, 0.000109, 0.000164
	Black	Women	42	0.0000352, 0.0000521, 0.0000769, 0.000113, 0.000168
	Black	Women	43	0.0000391, 0.0000567, 0.000082, 0.000118, 0.000172
	Black	Women	44	0.0000433, 0.0000618, 0.0000879, 0.000125, 0.000179
	Black	Women	45	0.0000478, 0.0000675, 0.000095, 0.000134, 0.000189
	Black	Women	46	0.0000538, 0.0000747, 0.000104, 0.000143, 0.000199
	Black	Women	47	0.0000617, 0.0000839, 0.000114, 0.000154, 0.00021
	Black	Women	48	0.0000712, 0.0000948, 0.000126, 0.000167, 0.000222
	Black	Women	49	0.0000813, 0.000107, 0.00014, 0.000183, 0.00024
	Black	Women	50	0.0000915, 0.000119, 0.000156, 0.000203, 0.000265
	Black	Women	51	0.000103, 0.000134, 0.000173, 0.000224, 0.000291
	Black	Women	52	0.000117, 0.00015, 0.000192, 0.000245, 0.000315
	Black	Women	53	0.000131, 0.000166, 0.000211, 0.000267, 0.00034
	Black	Women	54	0.000143, 0.000182, 0.00023, 0.000292, 0.00037
	Black	Women	55	0.000155, 0.000198, 0.000251, 0.000318, 0.000405
	Black	Women	56	0.000169, 0.000215, 0.000272, 0.000345, 0.000439
	Black	Women	57	0.000186, 0.000234, 0.000295, 0.000371, 0.000468
	Black	Women	58	0.000207, 0.000257, 0.000319, 0.000395, 0.000491
	Black	Women	59	0.00023, 0.000281, 0.000344, 0.00042, 0.000515
	Black	Women	60	0.00025, 0.000304, 0.000369, 0.000447, 0.000542
	Black	Women	61	0.000267, 0.000324, 0.000392, 0.000474, 0.000575
	Black	Women	62	0.000281, 0.000341, 0.000413, 0.0005, 0.000607
	Black	Women	63	0.000294, 0.000357, 0.000432, 0.000523, 0.000635
	Black	Women	64	0.000309, 0.000374, 0.000451, 0.000544, 0.000658
	Black	Women	65	0.000327, 0.000393, 0.000472, 0.000566, 0.000681

Variable	Race/ethnicity	Sex	Age	Distribution
	Black	Women	66	0.000349, 0.000417, 0.000498, 0.000594, 0.000711
	Black	Women	67	0.000376, 0.000448, 0.000532, 0.000632, 0.000753
	Black	Women	68	0.000411, 0.000487, 0.000576, 0.000682, 0.000809
	Black	Women	69	0.000454, 0.000536, 0.000631, 0.000744, 0.000878
	Black	Women	70	0.000504, 0.000593, 0.000697, 0.000819, 0.000964
	Black	Women	71	0.00056, 0.000658, 0.000773, 0.000907, 0.00107
	Black	Women	72	0.000621, 0.00073, 0.000858, 0.00101, 0.00119
	Black	Women	73	0.000689, 0.000811, 0.000953, 0.00112, 0.00132
	Black	Women	74	0.000768, 0.000903, 0.00106, 0.00124, 0.00146
	Black	Women	75	0.000857, 0.00101, 0.00118, 0.00138, 0.00162
	Black	Women	76	0.000951, 0.00112, 0.00132, 0.00155, 0.00183
	Black	Women	77	0.00105, 0.00125, 0.00148, 0.00176, 0.00209
	Black	Women	78	0.00115, 0.00138, 0.00166, 0.002, 0.00241
	Black	Women	79	0.00126, 0.00153, 0.00187, 0.00227, 0.00277
	Black	Women	80	0.00138, 0.0017, 0.00209, 0.00256, 0.00315
	Black	Women	81	0.00152, 0.00188, 0.00232, 0.00287, 0.00355
	Black	Women	82	0.00165, 0.00206, 0.00256, 0.00319, 0.00397
	Black	Women	83	0.00178, 0.00224, 0.0028, 0.00352, 0.00442
	Black	Women	84	0.0019, 0.00241, 0.00305, 0.00385, 0.00487
	Hispanic	Men	30	0.00000568, 0.00000872, 0.0000133, 0.0000204, 0.0000313
	Hispanic	Men	31	0.00000702, 0.0000104, 0.0000152, 0.0000224, 0.000033
	Hispanic	Men	32	0.00000852, 0.0000122, 0.0000173, 0.0000247, 0.0000353
	Hispanic	Men	33	0.0000101, 0.0000141, 0.0000197, 0.0000274, 0.0000383
	Hispanic	Men	34	0.0000116, 0.0000161, 0.0000222, 0.0000305, 0.0000422
	Hispanic	Men	35	0.0000131, 0.000018, 0.0000247, 0.0000339, 0.0000467
	Hispanic	Men	36	0.0000145, 0.0000199, 0.0000272, 0.0000372, 0.0000511
	Hispanic	Men	37	0.000016, 0.0000218, 0.0000297, 0.0000403, 0.0000549
	Hispanic	Men	38	0.0000176, 0.0000238, 0.000032, 0.0000432, 0.0000584
	Hispanic	Men	39	0.0000191, 0.0000256, 0.0000343, 0.0000459, 0.0000616
	Hispanic	Men	40	0.0000208, 0.0000276, 0.0000365, 0.0000484, 0.0000643
	Hispanic	Men	41	0.0000228, 0.0000298, 0.0000388, 0.0000504, 0.0000658
	Hispanic	Men	42	0.0000255, 0.0000324, 0.0000411, 0.0000522, 0.0000664
	Hispanic	Men	43	0.0000282, 0.0000352, 0.0000438, 0.0000545, 0.0000679
	Hispanic	Men	44	0.0000307, 0.000038, 0.0000469, 0.0000579, 0.0000717
	Hispanic	Men	45	0.0000334, 0.0000412, 0.0000508, 0.0000625, 0.0000771
	Hispanic	Men	46	0.0000369, 0.0000453, 0.0000554, 0.0000678, 0.0000832
	Hispanic	Men	47	0.0000414, 0.0000503, 0.0000611, 0.0000742, 0.0000903
	Hispanic	Men	48	0.0000468, 0.0000565, 0.0000682, 0.0000822, 0.0000993
	Hispanic	Men	49	0.0000534, 0.0000641, 0.0000767, 0.0000919, 0.00011
	Hispanic	Men	50	0.0000618, 0.0000734, 0.0000871, 0.000103, 0.000123
	Hispanic	Men	51	0.0000722, 0.0000847, 0.0000992, 0.000116, 0.000136
	Hispanic	Men	52	0.0000837, 0.0000973, 0.000113, 0.000131, 0.000153
	Hispanic	Men	53	0.0000939, 0.00011, 0.000128, 0.00015, 0.000175

Variable	Race/ethnicity	Sex	Age	Distribution
	Hispanic	Men	54	0.000102, 0.000121, 0.000144, 0.000171, 0.000203
	Hispanic	Men	55	0.00011, 0.000133, 0.000161, 0.000193, 0.000234
	Hispanic	Men	56	0.00012, 0.000146, 0.000177, 0.000216, 0.000263
	Hispanic	Men	57	0.00013, 0.000159, 0.000194, 0.000237, 0.000289
	Hispanic	Men	58	0.000143, 0.000174, 0.000211, 0.000257, 0.000314
	Hispanic	Men	59	0.000156, 0.000189, 0.000229, 0.000276, 0.000335
	Hispanic	Men	60	0.00017, 0.000205, 0.000246, 0.000295, 0.000355
	Hispanic	Men	61	0.000184, 0.00022, 0.000262, 0.000312, 0.000373
	Hispanic	Men	62	0.000197, 0.000234, 0.000278, 0.00033, 0.000392
	Hispanic	Men	63	0.000209, 0.000248, 0.000293, 0.000347, 0.000412
	Hispanic	Men	64	0.000221, 0.000262, 0.000309, 0.000366, 0.000433
	Hispanic	Men	65	0.000235, 0.000278, 0.000328, 0.000387, 0.000458
	Hispanic	Men	66	0.00025, 0.000297, 0.000351, 0.000415, 0.000492
	Hispanic	Men	67	0.000268, 0.000319, 0.000379, 0.000451, 0.000537
	Hispanic	Men	68	0.00029, 0.000347, 0.000415, 0.000496, 0.000594
	Hispanic	Men	69	0.000318, 0.000382, 0.000458, 0.000549, 0.00066
	Hispanic	Men	70	0.000352, 0.000423, 0.000508, 0.00061, 0.000733
	Hispanic	Men	71	0.000393, 0.000472, 0.000565, 0.000677, 0.000813
	Hispanic	Men	72	0.000438, 0.000525, 0.000629, 0.000752, 0.000902
	Hispanic	Men	73	0.000488, 0.000585, 0.000699, 0.000836, 0.001
	Hispanic	Men	74	0.000544, 0.000651, 0.000778, 0.00093, 0.00111
	Hispanic	Men	75	0.000606, 0.000726, 0.000867, 0.00104, 0.00124
	Hispanic	Men	76	0.000677, 0.000811, 0.00097, 0.00116, 0.00139
	Hispanic	Men	77	0.000756, 0.000908, 0.00109, 0.0013, 0.00157
	Hispanic	Men	78	0.000842, 0.00102, 0.00122, 0.00147, 0.00178
	Hispanic	Men	79	0.000934, 0.00113, 0.00137, 0.00167, 0.00202
	Hispanic	Men	80	0.00103, 0.00126, 0.00154, 0.00188, 0.0023
	Hispanic	Men	81	0.00113, 0.00139, 0.00171, 0.00211, 0.0026
	Hispanic	Men	82	0.00122, 0.00152, 0.00189, 0.00235, 0.00293
	Hispanic	Men	83	0.00131, 0.00165, 0.00207, 0.0026, 0.00327
	Hispanic	Men	84	0.00139, 0.00177, 0.00224, 0.00284, 0.00361
	Hispanic	Women	30	0.00000335, 0.00000555, 0.00000916, 0.0000151, 0.000025
	Hispanic	Women	31	0.00000408, 0.00000652, 0.0000104, 0.0000165, 0.0000263
	Hispanic	Women	32	0.00000489, 0.0000076, 0.0000118, 0.0000182, 0.0000283
	Hispanic	Women	33	0.00000576, 0.00000879, 0.0000134, 0.0000203, 0.000031
	Hispanic	Women	34	0.00000669, 0.0000101, 0.0000151, 0.0000227, 0.0000341
	Hispanic	Women	35	0.00000768, 0.0000114, 0.0000169, 0.000025, 0.0000372
	Hispanic	Women	36	0.00000875, 0.0000128, 0.0000187, 0.0000273, 0.00004
	Hispanic	Women	37	0.00000987, 0.0000142, 0.0000205, 0.0000294, 0.0000424
	Hispanic	Women	38	0.000011, 0.0000156, 0.0000221, 0.0000313, 0.0000445
	Hispanic	Women	39	0.0000122, 0.000017, 0.0000238, 0.0000332, 0.0000464
	Hispanic	Women	40	0.0000134, 0.0000184, 0.0000253, 0.0000348, 0.000048
	Hispanic	Women	41	0.0000148, 0.00002, 0.0000269, 0.0000362, 0.000049

Variable	Race/ethnicity	Sex	Age	Distribution
	Hispanic	Women	42	0.0000164, 0.0000217, 0.0000286, 0.0000377, 0.0000499
	Hispanic	Women	43	0.000018, 0.0000234, 0.0000305, 0.0000396, 0.0000516
	Hispanic	Women	44	0.0000196, 0.0000254, 0.0000327, 0.0000422, 0.0000546
	Hispanic	Women	45	0.0000218, 0.0000278, 0.0000354, 0.0000452, 0.0000577
	Hispanic	Women	46	0.0000247, 0.0000309, 0.0000387, 0.0000483, 0.0000605
	Hispanic	Women	47	0.0000284, 0.0000348, 0.0000425, 0.000052, 0.0000637
	Hispanic	Women	48	0.0000323, 0.000039, 0.000047, 0.0000567, 0.0000685
	Hispanic	Women	49	0.0000362, 0.0000436, 0.0000524, 0.000063, 0.0000759
	Hispanic	Women	50	0.0000402, 0.0000486, 0.0000588, 0.0000711, 0.0000861
	Hispanic	Women	51	0.0000444, 0.0000542, 0.0000661, 0.0000807, 0.0000986
	Hispanic	Women	52	0.0000488, 0.0000603, 0.0000744, 0.0000917, 0.000113
	Hispanic	Women	53	0.0000534, 0.0000668, 0.0000834, 0.000104, 0.00013
	Hispanic	Women	54	0.0000583, 0.0000737, 0.000093, 0.000117, 0.000148
	Hispanic	Women	55	0.000064, 0.0000814, 0.000103, 0.000131, 0.000166
	Hispanic	Women	56	0.0000707, 0.0000897, 0.000114, 0.000144, 0.000183
	Hispanic	Women	57	0.000078, 0.0000987, 0.000125, 0.000157, 0.000199
	Hispanic	Women	58	0.0000856, 0.000108, 0.000136, 0.000172, 0.000217
	Hispanic	Women	59	0.0000933, 0.000118, 0.000148, 0.000186, 0.000235
	Hispanic	Women	60	0.000101, 0.000128, 0.00016, 0.000202, 0.000254
	Hispanic	Women	61	0.000109, 0.000137, 0.000172, 0.000216, 0.000272
	Hispanic	Women	62	0.000117, 0.000147, 0.000184, 0.000231, 0.000289
	Hispanic	Women	63	0.000125, 0.000157, 0.000196, 0.000245, 0.000306
	Hispanic	Women	64	0.000133, 0.000167, 0.000208, 0.000259, 0.000324
	Hispanic	Women	65	0.000142, 0.000178, 0.000222, 0.000277, 0.000346
	Hispanic	Women	66	0.000153, 0.000191, 0.000239, 0.000298, 0.000374
	Hispanic	Women	67	0.000165, 0.000208, 0.00026, 0.000326, 0.00041
	Hispanic	Women	68	0.000182, 0.000229, 0.000288, 0.000361, 0.000455
	Hispanic	Women	69	0.000203, 0.000256, 0.000321, 0.000403, 0.000508
	Hispanic	Women	70	0.000231, 0.000289, 0.000362, 0.000452, 0.000566
	Hispanic	Women	71	0.000266, 0.00033, 0.000409, 0.000506, 0.000627
	Hispanic	Women	72	0.000309, 0.000379, 0.000463, 0.000565, 0.000692
	Hispanic	Women	73	0.000359, 0.000434, 0.000524, 0.000632, 0.000764
	Hispanic	Women	74	0.000416, 0.000498, 0.000595, 0.00071, 0.00085
	Hispanic	Women	75	0.000479, 0.00057, 0.000677, 0.000803, 0.000956
	Hispanic	Women	76	0.000549, 0.000652, 0.000773, 0.000916, 0.00109
	Hispanic	Women	77	0.000628, 0.000747, 0.000887, 0.00105, 0.00125
	Hispanic	Women	78	0.000721, 0.000858, 0.00102, 0.00121, 0.00144
	Hispanic	Women	79	0.000828, 0.000986, 0.00117, 0.00139, 0.00166
	Hispanic	Women	80	0.000944, 0.00113, 0.00134, 0.0016, 0.00191
	Hispanic	Women	81	0.00106, 0.00127, 0.00153, 0.00183, 0.0022
	Hispanic	Women	82	0.00116, 0.00141, 0.00172, 0.00209, 0.00254
	Hispanic	Women	83	0.00125, 0.00155, 0.00192, 0.00237, 0.00294
	Hispanic	Women	84	0.00133, 0.00168, 0.00212, 0.00267, 0.00337

Variable	Race/ethnicity	Sex	Age	Distribution
	White	Men	30	0.00000446, 0.00000674, 0.0000102, 0.0000153, 0.0000231
	White	Men	31	0.0000054, 0.0000079, 0.0000115, 0.0000168, 0.0000245
	White	Men	32	0.00000645, 0.00000918, 0.000013, 0.0000185, 0.0000263
	White	Men	33	0.00000758, 0.0000106, 0.0000146, 0.0000203, 0.0000283
	White	Men	34	0.00000872, 0.000012, 0.0000164, 0.0000224, 0.0000307
	White	Men	35	0.00000979, 0.0000133, 0.0000181, 0.0000246, 0.0000335
	White	Men	36	0.0000108, 0.0000146, 0.0000198, 0.0000268, 0.0000364
	White	Men	37	0.0000117, 0.0000159, 0.0000215, 0.000029, 0.0000392
	White	Men	38	0.0000127, 0.0000171, 0.0000231, 0.0000311, 0.0000419
	White	Men	39	0.0000136, 0.0000183, 0.0000246, 0.000033, 0.0000444
	White	Men	40	0.0000147, 0.0000196, 0.0000261, 0.0000347, 0.0000463
	White	Men	41	0.0000162, 0.0000212, 0.0000276, 0.000036, 0.0000471
	White	Men	42	0.0000181, 0.000023, 0.0000292, 0.0000371, 0.0000471
	White	Men	43	0.0000203, 0.0000252, 0.0000311, 0.0000385, 0.0000477
	White	Men	44	0.0000225, 0.0000275, 0.0000335, 0.0000408, 0.0000499
	White	Men	45	0.0000248, 0.0000301, 0.0000364, 0.000044, 0.0000533
	White	Men	46	0.0000278, 0.0000334, 0.0000399, 0.0000478, 0.0000574
	White	Men	47	0.0000313, 0.0000373, 0.0000443, 0.0000526, 0.0000626
	White	Men	48	0.0000352, 0.0000418, 0.0000496, 0.0000589, 0.00007
	White	Men	49	0.0000393, 0.000047, 0.0000561, 0.0000669, 0.0000801
	White	Men	50	0.000044, 0.000053, 0.0000638, 0.0000767, 0.0000924
	White	Men	51	0.00005, 0.0000604, 0.0000728, 0.0000877, 0.000106
	White	Men	52	0.0000575, 0.0000692, 0.0000831, 0.0000997, 0.00012
	White	Men	53	0.0000659, 0.0000789, 0.0000944, 0.000113, 0.000135
	White	Men	54	0.0000743, 0.000089, 0.000106, 0.000127, 0.000153
	White	Men	55	0.0000827, 0.0000993, 0.000119, 0.000143, 0.000172
	White	Men	56	0.0000909, 0.00011, 0.000132, 0.00016, 0.000193
	White	Men	57	0.0000989, 0.00012, 0.000146, 0.000178, 0.000216
	White	Men	58	0.000107, 0.000131, 0.000161, 0.000197, 0.000241
	White	Men	59	0.000116, 0.000143, 0.000176, 0.000217, 0.000267
	White	Men	60	0.000127, 0.000157, 0.000193, 0.000237, 0.000291
	White	Men	61	0.00014, 0.000171, 0.000209, 0.000255, 0.000312
	White	Men	62	0.000154, 0.000187, 0.000225, 0.000272, 0.000329
	White	Men	63	0.000171, 0.000204, 0.000242, 0.000288, 0.000343
	White	Men	64	0.000189, 0.000222, 0.00026, 0.000305, 0.000358
	White	Men	65	0.000208, 0.000242, 0.00028, 0.000324, 0.000376
	White	Men	66	0.000229, 0.000264, 0.000304, 0.00035, 0.000404
	White	Men	67	0.000251, 0.000289, 0.000334, 0.000385, 0.000444
	White	Men	68	0.000276, 0.00032, 0.000371, 0.000429, 0.000497
	White	Men	69	0.000307, 0.000357, 0.000415, 0.000483, 0.000563
	White	Men	70	0.000343, 0.000401, 0.000468, 0.000547, 0.000639
	White	Men	71	0.000385, 0.000451, 0.000528, 0.000619, 0.000725
	White	Men	72	0.000431, 0.000507, 0.000596, 0.0007, 0.000823

Variable	Race/ethnicity	Sex	Age	Distribution
	White	Men	73	0.000482, 0.000569, 0.000671, 0.000792, 0.000935
	White	Men	74	0.000537, 0.000637, 0.000756, 0.000896, 0.00106
	White	Men	75	0.000599, 0.000716, 0.000853, 0.00102, 0.00121
	White	Men	76	0.000672, 0.000807, 0.000967, 0.00116, 0.00139
	White	Men	77	0.000755, 0.000913, 0.0011, 0.00133, 0.00161
	White	Men	78	0.000847, 0.00103, 0.00126, 0.00154, 0.00188
	White	Men	79	0.000946, 0.00117, 0.00145, 0.00179, 0.00222
	White	Men	80	0.00105, 0.00132, 0.00166, 0.00208, 0.00261
	White	Men	81	0.00117, 0.00149, 0.00189, 0.0024, 0.00305
	White	Men	82	0.00131, 0.00167, 0.00214, 0.00273, 0.00349
	White	Men	83	0.00144, 0.00186, 0.00239, 0.00307, 0.00395
	White	Men	84	0.00157, 0.00204, 0.00264, 0.00342, 0.00444
	White	Women	30	0.00000402, 0.00000618, 0.00000947, 0.0000145, 0.0000223
	White	Women	31	0.00000486, 0.00000727, 0.0000108, 0.0000161, 0.0000241
	White	Women	32	0.00000584, 0.00000852, 0.0000124, 0.000018, 0.0000262
	White	Women	33	0.00000692, 0.00000988, 0.0000141, 0.00002, 0.0000286
	White	Women	34	0.00000802, 0.0000113, 0.0000158, 0.0000222, 0.0000312
	White	Women	35	0.00000906, 0.0000126, 0.0000176, 0.0000244, 0.000034
	White	Women	36	0.0000101, 0.0000139, 0.0000192, 0.0000265, 0.0000367
	White	Women	37	0.000011, 0.0000151, 0.0000207, 0.0000284, 0.0000391
	White	Women	38	0.0000119, 0.0000163, 0.0000222, 0.0000302, 0.0000412
	White	Women	39	0.0000128, 0.0000174, 0.0000235, 0.0000317, 0.0000429
	White	Women	40	0.0000139, 0.0000185, 0.0000247, 0.0000329, 0.0000439
	White	Women	41	0.0000152, 0.0000199, 0.0000259, 0.0000336, 0.0000439
	White	Women	42	0.000017, 0.0000215, 0.0000271, 0.0000342, 0.0000432
	White	Women	43	0.0000189, 0.0000232, 0.0000285, 0.000035, 0.0000431
	White	Women	44	0.0000205, 0.0000249, 0.0000303, 0.0000368, 0.0000448
	White	Women	45	0.0000221, 0.0000268, 0.0000325, 0.0000393, 0.0000477
	White	Women	46	0.0000241, 0.0000292, 0.0000352, 0.0000425, 0.0000513
	White	Women	47	0.0000265, 0.000032, 0.0000385, 0.0000464, 0.000056
	White	Women	48	0.0000291, 0.0000352, 0.0000425, 0.0000514, 0.0000622
	White	Women	49	0.0000319, 0.0000389, 0.0000474, 0.0000576, 0.0000703
	White	Women	50	0.0000353, 0.0000433, 0.0000531, 0.000065, 0.0000798
	White	Women	51	0.0000396, 0.0000486, 0.0000596, 0.0000731, 0.0000897
	White	Women	52	0.0000449, 0.0000548, 0.0000669, 0.0000816, 0.0000997
	White	Women	53	0.0000507, 0.0000616, 0.0000747, 0.0000905, 0.00011
	White	Women	54	0.0000569, 0.0000687, 0.0000828, 0.0000999, 0.000121
	White	Women	55	0.0000634, 0.0000761, 0.0000912, 0.000109, 0.000131
	White	Women	56	0.0000703, 0.0000839, 0.0000999, 0.000119, 0.000142
	White	Women	57	0.0000777, 0.0000921, 0.000109, 0.000129, 0.000153
	White	Women	58	0.0000853, 0.000101, 0.000119, 0.00014, 0.000166
	White	Women	59	0.0000935, 0.00011, 0.00013, 0.000152, 0.00018
	White	Women	60	0.000102, 0.00012, 0.000141, 0.000166, 0.000195

Variable	Race/ethnicity	Sex	Age	Distribution
	White	Women	61	0.000111, 0.000131, 0.000153, 0.00018, 0.000212
	White	Women	62	0.000121, 0.000142, 0.000166, 0.000195, 0.000229
	White	Women	63	0.000131, 0.000153, 0.000179, 0.00021, 0.000246
	White	Women	64	0.000142, 0.000166, 0.000193, 0.000225, 0.000263
	White	Women	65	0.000155, 0.00018, 0.000209, 0.000243, 0.000282
	White	Women	66	0.00017, 0.000197, 0.000229, 0.000265, 0.000308
	White	Women	67	0.000187, 0.000218, 0.000253, 0.000294, 0.000342
	White	Women	68	0.000209, 0.000243, 0.000284, 0.000331, 0.000386
	White	Women	69	0.000236, 0.000276, 0.000322, 0.000376, 0.000439
	White	Women	70	0.00027, 0.000315, 0.000368, 0.000429, 0.000502
	White	Women	71	0.00031, 0.000362, 0.000422, 0.000493, 0.000576
	White	Women	72	0.000355, 0.000415, 0.000485, 0.000566, 0.000662
	White	Women	73	0.000406, 0.000475, 0.000556, 0.00065, 0.000762
	White	Women	74	0.000464, 0.000544, 0.000637, 0.000745, 0.000874
	White	Women	75	0.000533, 0.000625, 0.000731, 0.000855, 0.001
	White	Women	76	0.000613, 0.000719, 0.000841, 0.000984, 0.00115
	White	Women	77	0.000704, 0.000828, 0.000972, 0.00114, 0.00134
	White	Women	78	0.000805, 0.000953, 0.00113, 0.00133, 0.00158
	White	Women	79	0.000919, 0.0011, 0.00131, 0.00157, 0.00187
	White	Women	80	0.00105, 0.00127, 0.00153, 0.00184, 0.00222
	White	Women	81	0.0012, 0.00146, 0.00177, 0.00214, 0.0026
	White	Women	82	0.00136, 0.00167, 0.00203, 0.00248, 0.00303
	White	Women	83	0.00153, 0.00188, 0.00231, 0.00284, 0.00349
	White	Women	84	0.0017, 0.0021, 0.0026, 0.00321, 0.00397
Total mortality rates for 2014 (0.01, 0.2, 0.5, 0.8. 0.99 percentiles of the empirical distribution produced during forecasting)				
	Black	Men	30	0.00228, 0.00228, 0.00228, 0.00228, 0.00228
	Black	Men	31	0.00234, 0.00234, 0.00234, 0.00234, 0.00234
	Black	Men	32	0.0024, 0.0024, 0.0024, 0.0024, 0.0024
	Black	Men	33	0.00248, 0.00248, 0.00248, 0.00248, 0.00248
	Black	Men	34	0.00256, 0.00256, 0.00256, 0.00256, 0.00256
	Black	Men	35	0.00266, 0.00266, 0.00266, 0.00266, 0.00266
	Black	Men	36	0.00277, 0.00277, 0.00277, 0.00277, 0.00277
	Black	Men	37	0.00289, 0.00289, 0.00289, 0.00289, 0.00289
	Black	Men	38	0.00301, 0.00301, 0.00301, 0.00301, 0.00301
	Black	Men	39	0.00313, 0.00313, 0.00313, 0.00313, 0.00313
	Black	Men	40	0.00327, 0.00327, 0.00327, 0.00327, 0.00327
	Black	Men	41	0.00342, 0.00342, 0.00342, 0.00342, 0.00342
	Black	Men	42	0.00361, 0.00361, 0.00361, 0.00361, 0.00361
	Black	Men	43	0.00383, 0.00383, 0.00383, 0.00383, 0.00383
	Black	Men	44	0.00411, 0.00411, 0.00411, 0.00411, 0.00411
	Black	Men	45	0.00444, 0.00444, 0.00444, 0.00444, 0.00444
	Black	Men	46	0.00483, 0.00483, 0.00483, 0.00483, 0.00483
	Black	Men	47	0.00529, 0.00529, 0.00529, 0.00529, 0.00529

Variable	Race/ethnicity	Sex	Age	Distribution
	Black	Men	48	0.0058, 0.0058, 0.0058, 0.0058, 0.0058
	Black	Men	49	0.00636, 0.00636, 0.00636, 0.00636, 0.00636
	Black	Men	50	0.00697, 0.00697, 0.00697, 0.00697, 0.00697
	Black	Men	51	0.00763, 0.00763, 0.00763, 0.00763, 0.00763
	Black	Men	52	0.00837, 0.00837, 0.00837, 0.00837, 0.00837
	Black	Men	53	0.00918, 0.00918, 0.00918, 0.00918, 0.00918
	Black	Men	54	0.0101, 0.0101, 0.0101, 0.0101, 0.0101
	Black	Men	55	0.0111, 0.0111, 0.0111, 0.0111, 0.0111
	Black	Men	56	0.0123, 0.0123, 0.0123, 0.0123, 0.0123
	Black	Men	57	0.0136, 0.0136, 0.0136, 0.0136, 0.0136
	Black	Men	58	0.015, 0.015, 0.015, 0.015, 0.015
	Black	Men	59	0.0164, 0.0164, 0.0164, 0.0164, 0.0164
	Black	Men	60	0.0178, 0.0178, 0.0178, 0.0178, 0.0178
	Black	Men	61	0.0192, 0.0192, 0.0192, 0.0192, 0.0192
	Black	Men	62	0.0205, 0.0205, 0.0205, 0.0205, 0.0205
	Black	Men	63	0.0217, 0.0217, 0.0217, 0.0217, 0.0217
	Black	Men	64	0.0229, 0.0229, 0.0229, 0.0229, 0.0229
	Black	Men	65	0.0241, 0.0241, 0.0241, 0.0241, 0.0241
	Black	Men	66	0.0254, 0.0254, 0.0254, 0.0254, 0.0254
	Black	Men	67	0.0269, 0.0269, 0.0269, 0.0269, 0.0269
	Black	Men	68	0.0286, 0.0286, 0.0286, 0.0286, 0.0286
	Black	Men	69	0.0306, 0.0306, 0.0306, 0.0306, 0.0306
	Black	Men	70	0.0328, 0.0328, 0.0328, 0.0328, 0.0328
	Black	Men	71	0.0353, 0.0353, 0.0353, 0.0353, 0.0353
	Black	Men	72	0.038, 0.038, 0.038, 0.038, 0.038
	Black	Men	73	0.0408, 0.0408, 0.0408, 0.0408, 0.0408
	Black	Men	74	0.0438, 0.0438, 0.0438, 0.0438, 0.0438
	Black	Men	75	0.0471, 0.0471, 0.0471, 0.0471, 0.0471
	Black	Men	76	0.0505, 0.0505, 0.0505, 0.0505, 0.0505
	Black	Men	77	0.0543, 0.0543, 0.0543, 0.0543, 0.0543
	Black	Men	78	0.0584, 0.0584, 0.0584, 0.0584, 0.0584
	Black	Men	79	0.063, 0.063, 0.063, 0.063, 0.063
	Black	Men	80	0.0681, 0.0681, 0.0681, 0.0681, 0.0681
	Black	Men	81	0.0736, 0.0736, 0.0736, 0.0736, 0.0736
	Black	Men	82	0.0793, 0.0793, 0.0793, 0.0793, 0.0793
	Black	Men	83	0.0851, 0.0851, 0.0851, 0.0851, 0.0851
	Black	Men	84	0.0907, 0.0907, 0.0907, 0.0907, 0.0907
	Black	Women	30	0.000954, 0.000954, 0.000954, 0.000954, 0.000954
	Black	Women	31	0.00103, 0.00103, 0.00103, 0.00103, 0.00103
	Black	Women	32	0.00112, 0.00112, 0.00112, 0.00112, 0.00112
	Black	Women	33	0.00122, 0.00122, 0.00122, 0.00122, 0.00122
	Black	Women	34	0.00132, 0.00132, 0.00132, 0.00132, 0.00132
	Black	Women	35	0.00142, 0.00142, 0.00142, 0.00142, 0.00142

Variable	Race/ethnicity	Sex	Age	Distribution
	Black	Women	36	0.00153, 0.00153, 0.00153, 0.00153, 0.00153
	Black	Women	37	0.00165, 0.00165, 0.00165, 0.00165, 0.00165
	Black	Women	38	0.00178, 0.00178, 0.00178, 0.00178, 0.00178
	Black	Women	39	0.00192, 0.00192, 0.00192, 0.00192, 0.00192
	Black	Women	40	0.00207, 0.00207, 0.00207, 0.00207, 0.00207
	Black	Women	41	0.00224, 0.00224, 0.00224, 0.00224, 0.00224
	Black	Women	42	0.00242, 0.00242, 0.00242, 0.00242, 0.00242
	Black	Women	43	0.00261, 0.00261, 0.00261, 0.00261, 0.00261
	Black	Women	44	0.00282, 0.00282, 0.00282, 0.00282, 0.00282
	Black	Women	45	0.00305, 0.00305, 0.00305, 0.00305, 0.00305
	Black	Women	46	0.00331, 0.00331, 0.00331, 0.00331, 0.00331
	Black	Women	47	0.00361, 0.00361, 0.00361, 0.00361, 0.00361
	Black	Women	48	0.00395, 0.00395, 0.00395, 0.00395, 0.00395
	Black	Women	49	0.00434, 0.00434, 0.00434, 0.00434, 0.00434
	Black	Women	50	0.00477, 0.00477, 0.00477, 0.00477, 0.00477
	Black	Women	51	0.00523, 0.00523, 0.00523, 0.00523, 0.00523
	Black	Women	52	0.00573, 0.00573, 0.00573, 0.00573, 0.00573
	Black	Women	53	0.00624, 0.00624, 0.00624, 0.00624, 0.00624
	Black	Women	54	0.00677, 0.00677, 0.00677, 0.00677, 0.00677
	Black	Women	55	0.00732, 0.00732, 0.00732, 0.00732, 0.00732
	Black	Women	56	0.00789, 0.00789, 0.00789, 0.00789, 0.00789
	Black	Women	57	0.00849, 0.00849, 0.00849, 0.00849, 0.00849
	Black	Women	58	0.00912, 0.00912, 0.00912, 0.00912, 0.00912
	Black	Women	59	0.00978, 0.00978, 0.00978, 0.00978, 0.00978
	Black	Women	60	0.0105, 0.0105, 0.0105, 0.0105, 0.0105
	Black	Women	61	0.0112, 0.0112, 0.0112, 0.0112, 0.0112
	Black	Women	62	0.0119, 0.0119, 0.0119, 0.0119, 0.0119
	Black	Women	63	0.0126, 0.0126, 0.0126, 0.0126, 0.0126
	Black	Women	64	0.0134, 0.0134, 0.0134, 0.0134, 0.0134
	Black	Women	65	0.0143, 0.0143, 0.0143, 0.0143, 0.0143
	Black	Women	66	0.0153, 0.0153, 0.0153, 0.0153, 0.0153
	Black	Women	67	0.0164, 0.0164, 0.0164, 0.0164, 0.0164
	Black	Women	68	0.0175, 0.0175, 0.0175, 0.0175, 0.0175
	Black	Women	69	0.0189, 0.0189, 0.0189, 0.0189, 0.0189
	Black	Women	70	0.0203, 0.0203, 0.0203, 0.0203, 0.0203
	Black	Women	71	0.0219, 0.0219, 0.0219, 0.0219, 0.0219
	Black	Women	72	0.0237, 0.0237, 0.0237, 0.0237, 0.0237
	Black	Women	73	0.0256, 0.0256, 0.0256, 0.0256, 0.0256
	Black	Women	74	0.0278, 0.0278, 0.0278, 0.0278, 0.0278
	Black	Women	75	0.0303, 0.0303, 0.0303, 0.0303, 0.0303
	Black	Women	76	0.0331, 0.0331, 0.0331, 0.0331, 0.0331
	Black	Women	77	0.0362, 0.0362, 0.0362, 0.0362, 0.0362
	Black	Women	78	0.0396, 0.0396, 0.0396, 0.0396, 0.0396

Variable	Race/ethnicity	Sex	Age	Distribution
	Black	Women	79	0.0434, 0.0434, 0.0434, 0.0434, 0.0434
	Black	Women	80	0.0475, 0.0475, 0.0475, 0.0475, 0.0475
	Black	Women	81	0.0519, 0.0519, 0.0519, 0.0519, 0.0519
	Black	Women	82	0.0565, 0.0565, 0.0565, 0.0565, 0.0565
	Black	Women	83	0.0612, 0.0612, 0.0612, 0.0612, 0.0612
	Black	Women	84	0.0659, 0.0659, 0.0659, 0.0659, 0.0659
	Hispanic	Men	30	0.00098, 0.00098, 0.00098, 0.00098, 0.00098
	Hispanic	Men	31	0.000994, 0.000994, 0.000994, 0.000994, 0.000994
	Hispanic	Men	32	0.00102, 0.00102, 0.00102, 0.00102, 0.00102
	Hispanic	Men	33	0.00105, 0.00105, 0.00105, 0.00105, 0.00105
	Hispanic	Men	34	0.00108, 0.00108, 0.00108, 0.00108, 0.00108
	Hispanic	Men	35	0.00113, 0.00113, 0.00113, 0.00113, 0.00113
	Hispanic	Men	36	0.00118, 0.00118, 0.00118, 0.00118, 0.00118
	Hispanic	Men	37	0.00124, 0.00124, 0.00124, 0.00124, 0.00124
	Hispanic	Men	38	0.0013, 0.0013, 0.0013, 0.0013, 0.0013
	Hispanic	Men	39	0.00137, 0.00137, 0.00137, 0.00137, 0.00137
	Hispanic	Men	40	0.00146, 0.00146, 0.00146, 0.00146, 0.00146
	Hispanic	Men	41	0.00156, 0.00156, 0.00156, 0.00156, 0.00156
	Hispanic	Men	42	0.00167, 0.00167, 0.00167, 0.00167, 0.00167
	Hispanic	Men	43	0.00181, 0.00181, 0.00181, 0.00181, 0.00181
	Hispanic	Men	44	0.00197, 0.00197, 0.00197, 0.00197, 0.00197
	Hispanic	Men	45	0.00215, 0.00215, 0.00215, 0.00215, 0.00215
	Hispanic	Men	46	0.00236, 0.00236, 0.00236, 0.00236, 0.00236
	Hispanic	Men	47	0.0026, 0.0026, 0.0026, 0.0026, 0.0026
	Hispanic	Men	48	0.00288, 0.00288, 0.00288, 0.00288, 0.00288
	Hispanic	Men	49	0.0032, 0.0032, 0.0032, 0.0032, 0.0032
	Hispanic	Men	50	0.00354, 0.00354, 0.00354, 0.00354, 0.00354
	Hispanic	Men	51	0.00391, 0.00391, 0.00391, 0.00391, 0.00391
	Hispanic	Men	52	0.00429, 0.00429, 0.00429, 0.00429, 0.00429
	Hispanic	Men	53	0.00468, 0.00468, 0.00468, 0.00468, 0.00468
	Hispanic	Men	54	0.00508, 0.00508, 0.00508, 0.00508, 0.00508
	Hispanic	Men	55	0.00551, 0.00551, 0.00551, 0.00551, 0.00551
	Hispanic	Men	56	0.00596, 0.00596, 0.00596, 0.00596, 0.00596
	Hispanic	Men	57	0.00646, 0.00646, 0.00646, 0.00646, 0.00646
	Hispanic	Men	58	0.007, 0.007, 0.007, 0.007, 0.007
	Hispanic	Men	59	0.00758, 0.00758, 0.00758, 0.00758, 0.00758
	Hispanic	Men	60	0.00819, 0.00819, 0.00819, 0.00819, 0.00819
	Hispanic	Men	61	0.00882, 0.00882, 0.00882, 0.00882, 0.00882
	Hispanic	Men	62	0.00945, 0.00945, 0.00945, 0.00945, 0.00945
	Hispanic	Men	63	0.0101, 0.0101, 0.0101, 0.0101, 0.0101
	Hispanic	Men	64	0.0108, 0.0108, 0.0108, 0.0108, 0.0108
	Hispanic	Men	65	0.0115, 0.0115, 0.0115, 0.0115, 0.0115
	Hispanic	Men	66	0.0123, 0.0123, 0.0123, 0.0123, 0.0123

Variable	Race/ethnicity	Sex	Age	Distribution
	Hispanic	Men	67	0.0132, 0.0132, 0.0132, 0.0132, 0.0132
	Hispanic	Men	68	0.0142, 0.0142, 0.0142, 0.0142, 0.0142
	Hispanic	Men	69	0.0154, 0.0154, 0.0154, 0.0154, 0.0154
	Hispanic	Men	70	0.0168, 0.0168, 0.0168, 0.0168, 0.0168
	Hispanic	Men	71	0.0183, 0.0183, 0.0183, 0.0183, 0.0183
	Hispanic	Men	72	0.0199, 0.0199, 0.0199, 0.0199, 0.0199
	Hispanic	Men	73	0.0218, 0.0218, 0.0218, 0.0218, 0.0218
	Hispanic	Men	74	0.0238, 0.0238, 0.0238, 0.0238, 0.0238
	Hispanic	Men	75	0.026, 0.026, 0.026, 0.026, 0.026
	Hispanic	Men	76	0.0285, 0.0285, 0.0285, 0.0285, 0.0285
	Hispanic	Men	77	0.0314, 0.0314, 0.0314, 0.0314, 0.0314
	Hispanic	Men	78	0.0347, 0.0347, 0.0347, 0.0347, 0.0347
	Hispanic	Men	79	0.0384, 0.0384, 0.0384, 0.0384, 0.0384
	Hispanic	Men	80	0.0427, 0.0427, 0.0427, 0.0427, 0.0427
	Hispanic	Men	81	0.0473, 0.0473, 0.0473, 0.0473, 0.0473
	Hispanic	Men	82	0.0522, 0.0522, 0.0522, 0.0522, 0.0522
	Hispanic	Men	83	0.0573, 0.0573, 0.0573, 0.0573, 0.0573
	Hispanic	Men	84	0.0623, 0.0623, 0.0623, 0.0623, 0.0623
	Hispanic	Women	30	0.000402, 0.000402, 0.000402, 0.000402, 0.000402
	Hispanic	Women	31	0.000415, 0.000415, 0.000415, 0.000415, 0.000415
	Hispanic	Women	32	0.000434, 0.000434, 0.000434, 0.000434, 0.000434
	Hispanic	Women	33	0.000462, 0.000462, 0.000462, 0.000462, 0.000462
	Hispanic	Women	34	0.000496, 0.000496, 0.000496, 0.000496, 0.000496
	Hispanic	Women	35	0.000536, 0.000536, 0.000536, 0.000536, 0.000536
	Hispanic	Women	36	0.00058, 0.00058, 0.00058, 0.00058, 0.00058
	Hispanic	Women	37	0.000628, 0.000628, 0.000628, 0.000628, 0.000628
	Hispanic	Women	38	0.000679, 0.000679, 0.000679, 0.000679, 0.000679
	Hispanic	Women	39	0.000735, 0.000735, 0.000735, 0.000735, 0.000735
	Hispanic	Women	40	0.000796, 0.000796, 0.000796, 0.000796, 0.000796
	Hispanic	Women	41	0.000862, 0.000862, 0.000862, 0.000862, 0.000862
	Hispanic	Women	42	0.000936, 0.000936, 0.000936, 0.000936, 0.000936
	Hispanic	Women	43	0.00102, 0.00102, 0.00102, 0.00102, 0.00102
	Hispanic	Women	44	0.00111, 0.00111, 0.00111, 0.00111, 0.00111
	Hispanic	Women	45	0.00121, 0.00121, 0.00121, 0.00121, 0.00121
	Hispanic	Women	46	0.00133, 0.00133, 0.00133, 0.00133, 0.00133
	Hispanic	Women	47	0.00146, 0.00146, 0.00146, 0.00146, 0.00146
	Hispanic	Women	48	0.0016, 0.0016, 0.0016, 0.0016, 0.0016
	Hispanic	Women	49	0.00176, 0.00176, 0.00176, 0.00176, 0.00176
	Hispanic	Women	50	0.00193, 0.00193, 0.00193, 0.00193, 0.00193
	Hispanic	Women	51	0.00211, 0.00211, 0.00211, 0.00211, 0.00211
	Hispanic	Women	52	0.0023, 0.0023, 0.0023, 0.0023, 0.0023
	Hispanic	Women	53	0.0025, 0.0025, 0.0025, 0.0025, 0.0025
	Hispanic	Women	54	0.00271, 0.00271, 0.00271, 0.00271, 0.00271

Variable	Race/ethnicity	Sex	Age	Distribution
	Hispanic	Women	55	0.00295, 0.00295, 0.00295, 0.00295, 0.00295
	Hispanic	Women	56	0.0032, 0.0032, 0.0032, 0.0032, 0.0032
	Hispanic	Women	57	0.00347, 0.00347, 0.00347, 0.00347, 0.00347
	Hispanic	Women	58	0.00377, 0.00377, 0.00377, 0.00377, 0.00377
	Hispanic	Women	59	0.00409, 0.00409, 0.00409, 0.00409, 0.00409
	Hispanic	Women	60	0.00444, 0.00444, 0.00444, 0.00444, 0.00444
	Hispanic	Women	61	0.00481, 0.00481, 0.00481, 0.00481, 0.00481
	Hispanic	Women	62	0.00521, 0.00521, 0.00521, 0.00521, 0.00521
	Hispanic	Women	63	0.00564, 0.00564, 0.00564, 0.00564, 0.00564
	Hispanic	Women	64	0.00612, 0.00612, 0.00612, 0.00612, 0.00612
	Hispanic	Women	65	0.00665, 0.00665, 0.00665, 0.00665, 0.00665
	Hispanic	Women	66	0.00725, 0.00725, 0.00725, 0.00725, 0.00725
	Hispanic	Women	67	0.00793, 0.00793, 0.00793, 0.00793, 0.00793
	Hispanic	Women	68	0.00868, 0.00868, 0.00868, 0.00868, 0.00868
	Hispanic	Women	69	0.00953, 0.00953, 0.00953, 0.00953, 0.00953
	Hispanic	Women	70	0.0105, 0.0105, 0.0105, 0.0105, 0.0105
	Hispanic	Women	71	0.0115, 0.0115, 0.0115, 0.0115, 0.0115
	Hispanic	Women	72	0.0127, 0.0127, 0.0127, 0.0127, 0.0127
	Hispanic	Women	73	0.014, 0.014, 0.014, 0.014, 0.014
	Hispanic	Women	74	0.0154, 0.0154, 0.0154, 0.0154, 0.0154
	Hispanic	Women	75	0.0171, 0.0171, 0.0171, 0.0171, 0.0171
	Hispanic	Women	76	0.019, 0.019, 0.019, 0.019, 0.019
	Hispanic	Women	77	0.0212, 0.0212, 0.0212, 0.0212, 0.0212
	Hispanic	Women	78	0.0238, 0.0238, 0.0238, 0.0238, 0.0238
	Hispanic	Women	79	0.0268, 0.0268, 0.0268, 0.0268, 0.0268
	Hispanic	Women	80	0.0302, 0.0302, 0.0302, 0.0302, 0.0302
	Hispanic	Women	81	0.034, 0.034, 0.034, 0.034, 0.034
	Hispanic	Women	82	0.0382, 0.0382, 0.0382, 0.0382, 0.0382
	Hispanic	Women	83	0.0425, 0.0425, 0.0425, 0.0425, 0.0425
	Hispanic	Women	84	0.0469, 0.0469, 0.0469, 0.0469, 0.0469
	White	Men	30	0.00158, 0.00158, 0.00158, 0.00158, 0.00158
	White	Men	31	0.00163, 0.00163, 0.00163, 0.00163, 0.00163
	White	Men	32	0.00168, 0.00168, 0.00168, 0.00168, 0.00168
	White	Men	33	0.00173, 0.00173, 0.00173, 0.00173, 0.00173
	White	Men	34	0.00178, 0.00178, 0.00178, 0.00178, 0.00178
	White	Men	35	0.00183, 0.00183, 0.00183, 0.00183, 0.00183
	White	Men	36	0.00189, 0.00189, 0.00189, 0.00189, 0.00189
	White	Men	37	0.00195, 0.00195, 0.00195, 0.00195, 0.00195
	White	Men	38	0.00203, 0.00203, 0.00203, 0.00203, 0.00203
	White	Men	39	0.00213, 0.00213, 0.00213, 0.00213, 0.00213
	White	Men	40	0.00224, 0.00224, 0.00224, 0.00224, 0.00224
	White	Men	41	0.00237, 0.00237, 0.00237, 0.00237, 0.00237
	White	Men	42	0.00253, 0.00253, 0.00253, 0.00253, 0.00253

Variable	Race/ethnicity	Sex	Age	Distribution
	White	Men	43	0.00271, 0.00271, 0.00271, 0.00271, 0.00271
	White	Men	44	0.00293, 0.00293, 0.00293, 0.00293, 0.00293
	White	Men	45	0.00319, 0.00319, 0.00319, 0.00319, 0.00319
	White	Men	46	0.0035, 0.0035, 0.0035, 0.0035, 0.0035
	White	Men	47	0.00385, 0.00385, 0.00385, 0.00385, 0.00385
	White	Men	48	0.00424, 0.00424, 0.00424, 0.00424, 0.00424
	White	Men	49	0.00467, 0.00467, 0.00467, 0.00467, 0.00467
	White	Men	50	0.00513, 0.00513, 0.00513, 0.00513, 0.00513
	White	Men	51	0.00562, 0.00562, 0.00562, 0.00562, 0.00562
	White	Men	52	0.00613, 0.00613, 0.00613, 0.00613, 0.00613
	White	Men	53	0.00666, 0.00666, 0.00666, 0.00666, 0.00666
	White	Men	54	0.00723, 0.00723, 0.00723, 0.00723, 0.00723
	White	Men	55	0.00782, 0.00782, 0.00782, 0.00782, 0.00782
	White	Men	56	0.00845, 0.00845, 0.00845, 0.00845, 0.00845
	White	Men	57	0.00912, 0.00912, 0.00912, 0.00912, 0.00912
	White	Men	58	0.00982, 0.00982, 0.00982, 0.00982, 0.00982
	White	Men	59	0.0105, 0.0105, 0.0105, 0.0105, 0.0105
	White	Men	60	0.0113, 0.0113, 0.0113, 0.0113, 0.0113
	White	Men	61	0.012, 0.012, 0.012, 0.012, 0.012
	White	Men	62	0.0128, 0.0128, 0.0128, 0.0128, 0.0128
	White	Men	63	0.0136, 0.0136, 0.0136, 0.0136, 0.0136
	White	Men	64	0.0145, 0.0145, 0.0145, 0.0145, 0.0145
	White	Men	65	0.0154, 0.0154, 0.0154, 0.0154, 0.0154
	White	Men	66	0.0165, 0.0165, 0.0165, 0.0165, 0.0165
	White	Men	67	0.0178, 0.0178, 0.0178, 0.0178, 0.0178
	White	Men	68	0.0193, 0.0193, 0.0193, 0.0193, 0.0193
	White	Men	69	0.021, 0.021, 0.021, 0.021, 0.021
	White	Men	70	0.023, 0.023, 0.023, 0.023, 0.023
	White	Men	71	0.0251, 0.0251, 0.0251, 0.0251, 0.0251
	White	Men	72	0.0275, 0.0275, 0.0275, 0.0275, 0.0275
	White	Men	73	0.0301, 0.0301, 0.0301, 0.0301, 0.0301
	White	Men	74	0.0329, 0.0329, 0.0329, 0.0329, 0.0329
	White	Men	75	0.0359, 0.0359, 0.0359, 0.0359, 0.0359
	White	Men	76	0.0393, 0.0393, 0.0393, 0.0393, 0.0393
	White	Men	77	0.0431, 0.0431, 0.0431, 0.0431, 0.0431
	White	Men	78	0.0476, 0.0476, 0.0476, 0.0476, 0.0476
	White	Men	79	0.0528, 0.0528, 0.0528, 0.0528, 0.0528
	White	Men	80	0.0587, 0.0587, 0.0587, 0.0587, 0.0587
	White	Men	81	0.0654, 0.0654, 0.0654, 0.0654, 0.0654
	White	Men	82	0.0727, 0.0727, 0.0727, 0.0727, 0.0727
	White	Men	83	0.0803, 0.0803, 0.0803, 0.0803, 0.0803
	White	Men	84	0.088, 0.088, 0.088, 0.088, 0.088
	White	Women	30	0.000741, 0.000741, 0.000741, 0.000741, 0.000741

Variable	Race/ethnicity	Sex	Age	Distribution
	White	Women	31	0.000789, 0.000789, 0.000789, 0.000789, 0.000789
	White	Women	32	0.000842, 0.000842, 0.000842, 0.000842, 0.000842
	White	Women	33	0.000901, 0.000901, 0.000901, 0.000901, 0.000901
	White	Women	34	0.000962, 0.000962, 0.000962, 0.000962, 0.000962
	White	Women	35	0.00103, 0.00103, 0.00103, 0.00103, 0.00103
	White	Women	36	0.00109, 0.00109, 0.00109, 0.00109, 0.00109
	White	Women	37	0.00117, 0.00117, 0.00117, 0.00117, 0.00117
	White	Women	38	0.00124, 0.00124, 0.00124, 0.00124, 0.00124
	White	Women	39	0.00133, 0.00133, 0.00133, 0.00133, 0.00133
	White	Women	40	0.00142, 0.00142, 0.00142, 0.00142, 0.00142
	White	Women	41	0.00153, 0.00153, 0.00153, 0.00153, 0.00153
	White	Women	42	0.00165, 0.00165, 0.00165, 0.00165, 0.00165
	White	Women	43	0.00178, 0.00178, 0.00178, 0.00178, 0.00178
	White	Women	44	0.00193, 0.00193, 0.00193, 0.00193, 0.00193
	White	Women	45	0.0021, 0.0021, 0.0021, 0.0021, 0.0021
	White	Women	46	0.00229, 0.00229, 0.00229, 0.00229, 0.00229
	White	Women	47	0.00251, 0.00251, 0.00251, 0.00251, 0.00251
	White	Women	48	0.00275, 0.00275, 0.00275, 0.00275, 0.00275
	White	Women	49	0.00301, 0.00301, 0.00301, 0.00301, 0.00301
	White	Women	50	0.00328, 0.00328, 0.00328, 0.00328, 0.00328
	White	Women	51	0.00357, 0.00357, 0.00357, 0.00357, 0.00357
	White	Women	52	0.00387, 0.00387, 0.00387, 0.00387, 0.00387
	White	Women	53	0.00418, 0.00418, 0.00418, 0.00418, 0.00418
	White	Women	54	0.00449, 0.00449, 0.00449, 0.00449, 0.00449
	White	Women	55	0.00481, 0.00481, 0.00481, 0.00481, 0.00481
	White	Women	56	0.00513, 0.00513, 0.00513, 0.00513, 0.00513
	White	Women	57	0.00548, 0.00548, 0.00548, 0.00548, 0.00548
	White	Women	58	0.00584, 0.00584, 0.00584, 0.00584, 0.00584
	White	Women	59	0.00622, 0.00622, 0.00622, 0.00622, 0.00622
	White	Women	60	0.00664, 0.00664, 0.00664, 0.00664, 0.00664
	White	Women	61	0.00711, 0.00711, 0.00711, 0.00711, 0.00711
	White	Women	62	0.00763, 0.00763, 0.00763, 0.00763, 0.00763
	White	Women	63	0.00822, 0.00822, 0.00822, 0.00822, 0.00822
	White	Women	64	0.0089, 0.0089, 0.0089, 0.0089, 0.0089
	White	Women	65	0.00968, 0.00968, 0.00968, 0.00968, 0.00968
	White	Women	66	0.0106, 0.0106, 0.0106, 0.0106, 0.0106
	White	Women	67	0.0116, 0.0116, 0.0116, 0.0116, 0.0116
	White	Women	68	0.0128, 0.0128, 0.0128, 0.0128, 0.0128
	White	Women	69	0.0141, 0.0141, 0.0141, 0.0141, 0.0141
	White	Women	70	0.0155, 0.0155, 0.0155, 0.0155, 0.0155
	White	Women	71	0.0171, 0.0171, 0.0171, 0.0171, 0.0171
	White	Women	72	0.0188, 0.0188, 0.0188, 0.0188, 0.0188
	White	Women	73	0.0207, 0.0207, 0.0207, 0.0207, 0.0207

Variable	Race/ethnicity	Sex	Age	Distribution
	White	Women	74	0.0228, 0.0228, 0.0228, 0.0228, 0.0228
	White	Women	75	0.0251, 0.0251, 0.0251, 0.0251, 0.0251
	White	Women	76	0.0277, 0.0277, 0.0277, 0.0277, 0.0277
	White	Women	77	0.0307, 0.0307, 0.0307, 0.0307, 0.0307
	White	Women	78	0.0342, 0.0342, 0.0342, 0.0342, 0.0342
	White	Women	79	0.0383, 0.0383, 0.0383, 0.0383, 0.0383
	White	Women	80	0.0429, 0.0429, 0.0429, 0.0429, 0.0429
	White	Women	81	0.048, 0.048, 0.048, 0.048, 0.048
	White	Women	82	0.0535, 0.0535, 0.0535, 0.0535, 0.0535
	White	Women	83	0.0594, 0.0594, 0.0594, 0.0594, 0.0594
	White	Women	84	0.0654, 0.0654, 0.0654, 0.0654, 0.0654
Total mortality rates for 2015 (0.01, 0.2, 0.5, 0.8. 0.99 percentiles of the empirical distribution produced during forecasting)				
	Black	Men	30	0.00248, 0.00248, 0.00248, 0.00248, 0.00248
	Black	Men	31	0.00253, 0.00253, 0.00253, 0.00253, 0.00253
	Black	Men	32	0.00257, 0.00257, 0.00257, 0.00257, 0.00257
	Black	Men	33	0.00264, 0.00264, 0.00264, 0.00264, 0.00264
	Black	Men	34	0.00273, 0.00273, 0.00273, 0.00273, 0.00273
	Black	Men	35	0.00285, 0.00285, 0.00285, 0.00285, 0.00285
	Black	Men	36	0.00297, 0.00297, 0.00297, 0.00297, 0.00297
	Black	Men	37	0.0031, 0.0031, 0.0031, 0.0031, 0.0031
	Black	Men	38	0.00322, 0.00322, 0.00322, 0.00322, 0.00322
	Black	Men	39	0.00333, 0.00333, 0.00333, 0.00333, 0.00333
	Black	Men	40	0.00346, 0.00346, 0.00346, 0.00346, 0.00346
	Black	Men	41	0.00361, 0.00361, 0.00361, 0.00361, 0.00361
	Black	Men	42	0.00379, 0.00379, 0.00379, 0.00379, 0.00379
	Black	Men	43	0.00401, 0.00401, 0.00401, 0.00401, 0.00401
	Black	Men	44	0.00427, 0.00427, 0.00427, 0.00427, 0.00427
	Black	Men	45	0.00457, 0.00457, 0.00457, 0.00457, 0.00457
	Black	Men	46	0.00491, 0.00491, 0.00491, 0.00491, 0.00491
	Black	Men	47	0.00531, 0.00531, 0.00531, 0.00531, 0.00531
	Black	Men	48	0.00579, 0.00579, 0.00579, 0.00579, 0.00579
	Black	Men	49	0.00635, 0.00635, 0.00635, 0.00635, 0.00635
	Black	Men	50	0.007, 0.007, 0.007, 0.007, 0.007
	Black	Men	51	0.00774, 0.00774, 0.00774, 0.00774, 0.00774
	Black	Men	52	0.00855, 0.00855, 0.00855, 0.00855, 0.00855
	Black	Men	53	0.00941, 0.00941, 0.00941, 0.00941, 0.00941
	Black	Men	54	0.0103, 0.0103, 0.0103, 0.0103, 0.0103
	Black	Men	55	0.0113, 0.0113, 0.0113, 0.0113, 0.0113
	Black	Men	56	0.0123, 0.0123, 0.0123, 0.0123, 0.0123
	Black	Men	57	0.0135, 0.0135, 0.0135, 0.0135, 0.0135
	Black	Men	58	0.0148, 0.0148, 0.0148, 0.0148, 0.0148
	Black	Men	59	0.0162, 0.0162, 0.0162, 0.0162, 0.0162
	Black	Men	60	0.0177, 0.0177, 0.0177, 0.0177, 0.0177

Variable	Race/ethnicity	Sex	Age	Distribution
	Black	Men	61	0.0193, 0.0193, 0.0193, 0.0193, 0.0193
	Black	Men	62	0.0208, 0.0208, 0.0208, 0.0208, 0.0208
	Black	Men	63	0.0222, 0.0222, 0.0222, 0.0222, 0.0222
	Black	Men	64	0.0235, 0.0235, 0.0235, 0.0235, 0.0235
	Black	Men	65	0.0246, 0.0246, 0.0246, 0.0246, 0.0246
	Black	Men	66	0.0258, 0.0258, 0.0258, 0.0258, 0.0258
	Black	Men	67	0.0271, 0.0271, 0.0271, 0.0271, 0.0271
	Black	Men	68	0.0286, 0.0286, 0.0286, 0.0286, 0.0286
	Black	Men	69	0.0304, 0.0304, 0.0304, 0.0304, 0.0304
	Black	Men	70	0.0326, 0.0326, 0.0326, 0.0326, 0.0326
	Black	Men	71	0.0351, 0.0351, 0.0351, 0.0351, 0.0351
	Black	Men	72	0.0379, 0.0379, 0.0379, 0.0379, 0.0379
	Black	Men	73	0.0409, 0.0409, 0.0409, 0.0409, 0.0409
	Black	Men	74	0.0441, 0.0441, 0.0441, 0.0441, 0.0441
	Black	Men	75	0.0475, 0.0475, 0.0475, 0.0475, 0.0475
	Black	Men	76	0.051, 0.051, 0.051, 0.051, 0.051
	Black	Men	77	0.0548, 0.0548, 0.0548, 0.0548, 0.0548
	Black	Men	78	0.059, 0.059, 0.059, 0.059, 0.059
	Black	Men	79	0.0636, 0.0636, 0.0636, 0.0636, 0.0636
	Black	Men	80	0.0687, 0.0687, 0.0687, 0.0687, 0.0687
	Black	Men	81	0.0742, 0.0742, 0.0742, 0.0742, 0.0742
	Black	Men	82	0.08, 0.08, 0.08, 0.08, 0.08
	Black	Men	83	0.0859, 0.0859, 0.0859, 0.0859, 0.0859
	Black	Men	84	0.0917, 0.0917, 0.0917, 0.0917, 0.0917
	Black	Women	30	0.000994, 0.000994, 0.000994, 0.000994, 0.000994
	Black	Women	31	0.00108, 0.00108, 0.00108, 0.00108, 0.00108
	Black	Women	32	0.00117, 0.00117, 0.00117, 0.00117, 0.00117
	Black	Women	33	0.00127, 0.00127, 0.00127, 0.00127, 0.00127
	Black	Women	34	0.00137, 0.00137, 0.00137, 0.00137, 0.00137
	Black	Women	35	0.00147, 0.00147, 0.00147, 0.00147, 0.00147
	Black	Women	36	0.00156, 0.00156, 0.00156, 0.00156, 0.00156
	Black	Women	37	0.00166, 0.00166, 0.00166, 0.00166, 0.00166
	Black	Women	38	0.00177, 0.00177, 0.00177, 0.00177, 0.00177
	Black	Women	39	0.00189, 0.00189, 0.00189, 0.00189, 0.00189
	Black	Women	40	0.00203, 0.00203, 0.00203, 0.00203, 0.00203
	Black	Women	41	0.0022, 0.0022, 0.0022, 0.0022, 0.0022
	Black	Women	42	0.00238, 0.00238, 0.00238, 0.00238, 0.00238
	Black	Women	43	0.00258, 0.00258, 0.00258, 0.00258, 0.00258
	Black	Women	44	0.00281, 0.00281, 0.00281, 0.00281, 0.00281
	Black	Women	45	0.00305, 0.00305, 0.00305, 0.00305, 0.00305
	Black	Women	46	0.00333, 0.00333, 0.00333, 0.00333, 0.00333
	Black	Women	47	0.00362, 0.00362, 0.00362, 0.00362, 0.00362
	Black	Women	48	0.00395, 0.00395, 0.00395, 0.00395, 0.00395

Variable	Race/ethnicity	Sex	Age	Distribution
	Black	Women	49	0.00431, 0.00431, 0.00431, 0.00431, 0.00431
	Black	Women	50	0.00471, 0.00471, 0.00471, 0.00471, 0.00471
	Black	Women	51	0.00514, 0.00514, 0.00514, 0.00514, 0.00514
	Black	Women	52	0.0056, 0.0056, 0.0056, 0.0056, 0.0056
	Black	Women	53	0.0061, 0.0061, 0.0061, 0.0061, 0.0061
	Black	Women	54	0.00663, 0.00663, 0.00663, 0.00663, 0.00663
	Black	Women	55	0.00721, 0.00721, 0.00721, 0.00721, 0.00721
	Black	Women	56	0.00782, 0.00782, 0.00782, 0.00782, 0.00782
	Black	Women	57	0.00847, 0.00847, 0.00847, 0.00847, 0.00847
	Black	Women	58	0.00914, 0.00914, 0.00914, 0.00914, 0.00914
	Black	Women	59	0.00982, 0.00982, 0.00982, 0.00982, 0.00982
	Black	Women	60	0.0105, 0.0105, 0.0105, 0.0105, 0.0105
	Black	Women	61	0.0112, 0.0112, 0.0112, 0.0112, 0.0112
	Black	Women	62	0.0119, 0.0119, 0.0119, 0.0119, 0.0119
	Black	Women	63	0.0125, 0.0125, 0.0125, 0.0125, 0.0125
	Black	Women	64	0.0133, 0.0133, 0.0133, 0.0133, 0.0133
	Black	Women	65	0.0141, 0.0141, 0.0141, 0.0141, 0.0141
	Black	Women	66	0.015, 0.015, 0.015, 0.015, 0.015
	Black	Women	67	0.016, 0.016, 0.016, 0.016, 0.016
	Black	Women	68	0.0173, 0.0173, 0.0173, 0.0173, 0.0173
	Black	Women	69	0.0187, 0.0187, 0.0187, 0.0187, 0.0187
	Black	Women	70	0.0203, 0.0203, 0.0203, 0.0203, 0.0203
	Black	Women	71	0.022, 0.022, 0.022, 0.022, 0.022
	Black	Women	72	0.0239, 0.0239, 0.0239, 0.0239, 0.0239
	Black	Women	73	0.026, 0.026, 0.026, 0.026, 0.026
	Black	Women	74	0.0281, 0.0281, 0.0281, 0.0281, 0.0281
	Black	Women	75	0.0305, 0.0305, 0.0305, 0.0305, 0.0305
	Black	Women	76	0.033, 0.033, 0.033, 0.033, 0.033
	Black	Women	77	0.0358, 0.0358, 0.0358, 0.0358, 0.0358
	Black	Women	78	0.0391, 0.0391, 0.0391, 0.0391, 0.0391
	Black	Women	79	0.0428, 0.0428, 0.0428, 0.0428, 0.0428
	Black	Women	80	0.0469, 0.0469, 0.0469, 0.0469, 0.0469
	Black	Women	81	0.0516, 0.0516, 0.0516, 0.0516, 0.0516
	Black	Women	82	0.0566, 0.0566, 0.0566, 0.0566, 0.0566
	Black	Women	83	0.0619, 0.0619, 0.0619, 0.0619, 0.0619
	Black	Women	84	0.0672, 0.0672, 0.0672, 0.0672, 0.0672
	Hispanic	Men	30	0.00106, 0.00106, 0.00106, 0.00106, 0.00106
	Hispanic	Men	31	0.00109, 0.00109, 0.00109, 0.00109, 0.00109
	Hispanic	Men	32	0.00112, 0.00112, 0.00112, 0.00112, 0.00112
	Hispanic	Men	33	0.00115, 0.00115, 0.00115, 0.00115, 0.00115
	Hispanic	Men	34	0.00118, 0.00118, 0.00118, 0.00118, 0.00118
	Hispanic	Men	35	0.00121, 0.00121, 0.00121, 0.00121, 0.00121
	Hispanic	Men	36	0.00124, 0.00124, 0.00124, 0.00124, 0.00124

Variable	Race/ethnicity	Sex	Age	Distribution
	Hispanic	Men	37	0.00129, 0.00129, 0.00129, 0.00129, 0.00129
	Hispanic	Men	38	0.00135, 0.00135, 0.00135, 0.00135, 0.00135
	Hispanic	Men	39	0.00142, 0.00142, 0.00142, 0.00142, 0.00142
	Hispanic	Men	40	0.00149, 0.00149, 0.00149, 0.00149, 0.00149
	Hispanic	Men	41	0.00159, 0.00159, 0.00159, 0.00159, 0.00159
	Hispanic	Men	42	0.00169, 0.00169, 0.00169, 0.00169, 0.00169
	Hispanic	Men	43	0.00182, 0.00182, 0.00182, 0.00182, 0.00182
	Hispanic	Men	44	0.00198, 0.00198, 0.00198, 0.00198, 0.00198
	Hispanic	Men	45	0.00216, 0.00216, 0.00216, 0.00216, 0.00216
	Hispanic	Men	46	0.00238, 0.00238, 0.00238, 0.00238, 0.00238
	Hispanic	Men	47	0.00263, 0.00263, 0.00263, 0.00263, 0.00263
	Hispanic	Men	48	0.00292, 0.00292, 0.00292, 0.00292, 0.00292
	Hispanic	Men	49	0.00322, 0.00322, 0.00322, 0.00322, 0.00322
	Hispanic	Men	50	0.00354, 0.00354, 0.00354, 0.00354, 0.00354
	Hispanic	Men	51	0.00388, 0.00388, 0.00388, 0.00388, 0.00388
	Hispanic	Men	52	0.00423, 0.00423, 0.00423, 0.00423, 0.00423
	Hispanic	Men	53	0.0046, 0.0046, 0.0046, 0.0046, 0.0046
	Hispanic	Men	54	0.00501, 0.00501, 0.00501, 0.00501, 0.00501
	Hispanic	Men	55	0.00544, 0.00544, 0.00544, 0.00544, 0.00544
	Hispanic	Men	56	0.00593, 0.00593, 0.00593, 0.00593, 0.00593
	Hispanic	Men	57	0.00646, 0.00646, 0.00646, 0.00646, 0.00646
	Hispanic	Men	58	0.00704, 0.00704, 0.00704, 0.00704, 0.00704
	Hispanic	Men	59	0.00766, 0.00766, 0.00766, 0.00766, 0.00766
	Hispanic	Men	60	0.00831, 0.00831, 0.00831, 0.00831, 0.00831
	Hispanic	Men	61	0.00898, 0.00898, 0.00898, 0.00898, 0.00898
	Hispanic	Men	62	0.00964, 0.00964, 0.00964, 0.00964, 0.00964
	Hispanic	Men	63	0.0103, 0.0103, 0.0103, 0.0103, 0.0103
	Hispanic	Men	64	0.011, 0.011, 0.011, 0.011, 0.011
	Hispanic	Men	65	0.0117, 0.0117, 0.0117, 0.0117, 0.0117
	Hispanic	Men	66	0.0125, 0.0125, 0.0125, 0.0125, 0.0125
	Hispanic	Men	67	0.0135, 0.0135, 0.0135, 0.0135, 0.0135
	Hispanic	Men	68	0.0145, 0.0145, 0.0145, 0.0145, 0.0145
	Hispanic	Men	69	0.0158, 0.0158, 0.0158, 0.0158, 0.0158
	Hispanic	Men	70	0.0172, 0.0172, 0.0172, 0.0172, 0.0172
	Hispanic	Men	71	0.0187, 0.0187, 0.0187, 0.0187, 0.0187
	Hispanic	Men	72	0.0204, 0.0204, 0.0204, 0.0204, 0.0204
	Hispanic	Men	73	0.0223, 0.0223, 0.0223, 0.0223, 0.0223
	Hispanic	Men	74	0.0243, 0.0243, 0.0243, 0.0243, 0.0243
	Hispanic	Men	75	0.0264, 0.0264, 0.0264, 0.0264, 0.0264
	Hispanic	Men	76	0.0289, 0.0289, 0.0289, 0.0289, 0.0289
	Hispanic	Men	77	0.0317, 0.0317, 0.0317, 0.0317, 0.0317
	Hispanic	Men	78	0.035, 0.035, 0.035, 0.035, 0.035
	Hispanic	Men	79	0.0388, 0.0388, 0.0388, 0.0388, 0.0388

Variable	Race/ethnicity	Sex	Age	Distribution
	Hispanic	Men	80	0.0433, 0.0433, 0.0433, 0.0433, 0.0433
	Hispanic	Men	81	0.0482, 0.0482, 0.0482, 0.0482, 0.0482
	Hispanic	Men	82	0.0536, 0.0536, 0.0536, 0.0536, 0.0536
	Hispanic	Men	83	0.0592, 0.0592, 0.0592, 0.0592, 0.0592
	Hispanic	Men	84	0.0647, 0.0647, 0.0647, 0.0647, 0.0647
	Hispanic	Women	30	0.000449, 0.000449, 0.000449, 0.000449, 0.000449
	Hispanic	Women	31	0.000477, 0.000477, 0.000477, 0.000477, 0.000477
	Hispanic	Women	32	0.000504, 0.000504, 0.000504, 0.000504, 0.000504
	Hispanic	Women	33	0.00053, 0.00053, 0.00053, 0.00053, 0.00053
	Hispanic	Women	34	0.000556, 0.000556, 0.000556, 0.000556, 0.000556
	Hispanic	Women	35	0.000583, 0.000583, 0.000583, 0.000583, 0.000583
	Hispanic	Women	36	0.000613, 0.000613, 0.000613, 0.000613, 0.000613
	Hispanic	Women	37	0.000647, 0.000647, 0.000647, 0.000647, 0.000647
	Hispanic	Women	38	0.000686, 0.000686, 0.000686, 0.000686, 0.000686
	Hispanic	Women	39	0.000732, 0.000732, 0.000732, 0.000732, 0.000732
	Hispanic	Women	40	0.000786, 0.000786, 0.000786, 0.000786, 0.000786
	Hispanic	Women	41	0.000848, 0.000848, 0.000848, 0.000848, 0.000848
	Hispanic	Women	42	0.00092, 0.00092, 0.00092, 0.00092, 0.00092
	Hispanic	Women	43	0.001, 0.001, 0.001, 0.001, 0.001
	Hispanic	Women	44	0.0011, 0.0011, 0.0011, 0.0011, 0.0011
	Hispanic	Women	45	0.00121, 0.00121, 0.00121, 0.00121, 0.00121
	Hispanic	Women	46	0.00134, 0.00134, 0.00134, 0.00134, 0.00134
	Hispanic	Women	47	0.00147, 0.00147, 0.00147, 0.00147, 0.00147
	Hispanic	Women	48	0.00162, 0.00162, 0.00162, 0.00162, 0.00162
	Hispanic	Women	49	0.00177, 0.00177, 0.00177, 0.00177, 0.00177
	Hispanic	Women	50	0.00193, 0.00193, 0.00193, 0.00193, 0.00193
	Hispanic	Women	51	0.0021, 0.0021, 0.0021, 0.0021, 0.0021
	Hispanic	Women	52	0.00229, 0.00229, 0.00229, 0.00229, 0.00229
	Hispanic	Women	53	0.00248, 0.00248, 0.00248, 0.00248, 0.00248
	Hispanic	Women	54	0.00268, 0.00268, 0.00268, 0.00268, 0.00268
	Hispanic	Women	55	0.0029, 0.0029, 0.0029, 0.0029, 0.0029
	Hispanic	Women	56	0.00315, 0.00315, 0.00315, 0.00315, 0.00315
	Hispanic	Women	57	0.00341, 0.00341, 0.00341, 0.00341, 0.00341
	Hispanic	Women	58	0.00371, 0.00371, 0.00371, 0.00371, 0.00371
	Hispanic	Women	59	0.00404, 0.00404, 0.00404, 0.00404, 0.00404
	Hispanic	Women	60	0.00441, 0.00441, 0.00441, 0.00441, 0.00441
	Hispanic	Women	61	0.00481, 0.00481, 0.00481, 0.00481, 0.00481
	Hispanic	Women	62	0.00525, 0.00525, 0.00525, 0.00525, 0.00525
	Hispanic	Women	63	0.00572, 0.00572, 0.00572, 0.00572, 0.00572
	Hispanic	Women	64	0.00622, 0.00622, 0.00622, 0.00622, 0.00622
	Hispanic	Women	65	0.00676, 0.00676, 0.00676, 0.00676, 0.00676
	Hispanic	Women	66	0.00736, 0.00736, 0.00736, 0.00736, 0.00736
	Hispanic	Women	67	0.00802, 0.00802, 0.00802, 0.00802, 0.00802

Variable	Race/ethnicity	Sex	Age	Distribution
	Hispanic	Women	68	0.00878, 0.00878, 0.00878, 0.00878, 0.00878
	Hispanic	Women	69	0.00964, 0.00964, 0.00964, 0.00964, 0.00964
	Hispanic	Women	70	0.0106, 0.0106, 0.0106, 0.0106, 0.0106
	Hispanic	Women	71	0.0117, 0.0117, 0.0117, 0.0117, 0.0117
	Hispanic	Women	72	0.013, 0.013, 0.013, 0.013, 0.013
	Hispanic	Women	73	0.0143, 0.0143, 0.0143, 0.0143, 0.0143
	Hispanic	Women	74	0.0158, 0.0158, 0.0158, 0.0158, 0.0158
	Hispanic	Women	75	0.0175, 0.0175, 0.0175, 0.0175, 0.0175
	Hispanic	Women	76	0.0194, 0.0194, 0.0194, 0.0194, 0.0194
	Hispanic	Women	77	0.0216, 0.0216, 0.0216, 0.0216, 0.0216
	Hispanic	Women	78	0.0241, 0.0241, 0.0241, 0.0241, 0.0241
	Hispanic	Women	79	0.0271, 0.0271, 0.0271, 0.0271, 0.0271
	Hispanic	Women	80	0.0306, 0.0306, 0.0306, 0.0306, 0.0306
	Hispanic	Women	81	0.0346, 0.0346, 0.0346, 0.0346, 0.0346
	Hispanic	Women	82	0.039, 0.039, 0.039, 0.039, 0.039
	Hispanic	Women	83	0.0437, 0.0437, 0.0437, 0.0437, 0.0437
	Hispanic	Women	84	0.0484, 0.0484, 0.0484, 0.0484, 0.0484
	White	Men	30	0.00171, 0.00171, 0.00171, 0.00171, 0.00171
	White	Men	31	0.00177, 0.00177, 0.00177, 0.00177, 0.00177
	White	Men	32	0.00182, 0.00182, 0.00182, 0.00182, 0.00182
	White	Men	33	0.00187, 0.00187, 0.00187, 0.00187, 0.00187
	White	Men	34	0.00192, 0.00192, 0.00192, 0.00192, 0.00192
	White	Men	35	0.00198, 0.00198, 0.00198, 0.00198, 0.00198
	White	Men	36	0.00203, 0.00203, 0.00203, 0.00203, 0.00203
	White	Men	37	0.00209, 0.00209, 0.00209, 0.00209, 0.00209
	White	Men	38	0.00216, 0.00216, 0.00216, 0.00216, 0.00216
	White	Men	39	0.00224, 0.00224, 0.00224, 0.00224, 0.00224
	White	Men	40	0.00234, 0.00234, 0.00234, 0.00234, 0.00234
	White	Men	41	0.00246, 0.00246, 0.00246, 0.00246, 0.00246
	White	Men	42	0.00261, 0.00261, 0.00261, 0.00261, 0.00261
	White	Men	43	0.00279, 0.00279, 0.00279, 0.00279, 0.00279
	White	Men	44	0.003, 0.003, 0.003, 0.003, 0.003
	White	Men	45	0.00324, 0.00324, 0.00324, 0.00324, 0.00324
	White	Men	46	0.00352, 0.00352, 0.00352, 0.00352, 0.00352
	White	Men	47	0.00383, 0.00383, 0.00383, 0.00383, 0.00383
	White	Men	48	0.0042, 0.0042, 0.0042, 0.0042, 0.0042
	White	Men	49	0.00462, 0.00462, 0.00462, 0.00462, 0.00462
	White	Men	50	0.00509, 0.00509, 0.00509, 0.00509, 0.00509
	White	Men	51	0.0056, 0.0056, 0.0056, 0.0056, 0.0056
	White	Men	52	0.00615, 0.00615, 0.00615, 0.00615, 0.00615
	White	Men	53	0.00671, 0.00671, 0.00671, 0.00671, 0.00671
	White	Men	54	0.00728, 0.00728, 0.00728, 0.00728, 0.00728
	White	Men	55	0.00786, 0.00786, 0.00786, 0.00786, 0.00786

Variable	Race/ethnicity	Sex	Age	Distribution
	White	Men	56	0.00846, 0.00846, 0.00846, 0.00846, 0.00846
	White	Men	57	0.00909, 0.00909, 0.00909, 0.00909, 0.00909
	White	Men	58	0.00977, 0.00977, 0.00977, 0.00977, 0.00977
	White	Men	59	0.0105, 0.0105, 0.0105, 0.0105, 0.0105
	White	Men	60	0.0113, 0.0113, 0.0113, 0.0113, 0.0113
	White	Men	61	0.0121, 0.0121, 0.0121, 0.0121, 0.0121
	White	Men	62	0.013, 0.013, 0.013, 0.013, 0.013
	White	Men	63	0.0138, 0.0138, 0.0138, 0.0138, 0.0138
	White	Men	64	0.0147, 0.0147, 0.0147, 0.0147, 0.0147
	White	Men	65	0.0156, 0.0156, 0.0156, 0.0156, 0.0156
	White	Men	66	0.0167, 0.0167, 0.0167, 0.0167, 0.0167
	White	Men	67	0.0179, 0.0179, 0.0179, 0.0179, 0.0179
	White	Men	68	0.0193, 0.0193, 0.0193, 0.0193, 0.0193
	White	Men	69	0.021, 0.021, 0.021, 0.021, 0.021
	White	Men	70	0.0229, 0.0229, 0.0229, 0.0229, 0.0229
	White	Men	71	0.0251, 0.0251, 0.0251, 0.0251, 0.0251
	White	Men	72	0.0275, 0.0275, 0.0275, 0.0275, 0.0275
	White	Men	73	0.0301, 0.0301, 0.0301, 0.0301, 0.0301
	White	Men	74	0.0329, 0.0329, 0.0329, 0.0329, 0.0329
	White	Men	75	0.036, 0.036, 0.036, 0.036, 0.036
	White	Men	76	0.0393, 0.0393, 0.0393, 0.0393, 0.0393
	White	Men	77	0.0432, 0.0432, 0.0432, 0.0432, 0.0432
	White	Men	78	0.0476, 0.0476, 0.0476, 0.0476, 0.0476
	White	Men	79	0.0528, 0.0528, 0.0528, 0.0528, 0.0528
	White	Men	80	0.0588, 0.0588, 0.0588, 0.0588, 0.0588
	White	Men	81	0.0657, 0.0657, 0.0657, 0.0657, 0.0657
	White	Men	82	0.0731, 0.0731, 0.0731, 0.0731, 0.0731
	White	Men	83	0.0809, 0.0809, 0.0809, 0.0809, 0.0809
	White	Men	84	0.0888, 0.0888, 0.0888, 0.0888, 0.0888
	White	Women	30	0.000807, 0.000807, 0.000807, 0.000807, 0.000807
	White	Women	31	0.000859, 0.000859, 0.000859, 0.000859, 0.000859
	White	Women	32	0.00091, 0.00091, 0.00091, 0.00091, 0.00091
	White	Women	33	0.000962, 0.000962, 0.000962, 0.000962, 0.000962
	White	Women	34	0.00101, 0.00101, 0.00101, 0.00101, 0.00101
	White	Women	35	0.00107, 0.00107, 0.00107, 0.00107, 0.00107
	White	Women	36	0.00113, 0.00113, 0.00113, 0.00113, 0.00113
	White	Women	37	0.0012, 0.0012, 0.0012, 0.0012, 0.0012
	White	Women	38	0.00127, 0.00127, 0.00127, 0.00127, 0.00127
	White	Women	39	0.00136, 0.00136, 0.00136, 0.00136, 0.00136
	White	Women	40	0.00145, 0.00145, 0.00145, 0.00145, 0.00145
	White	Women	41	0.00156, 0.00156, 0.00156, 0.00156, 0.00156
	White	Women	42	0.00168, 0.00168, 0.00168, 0.00168, 0.00168
	White	Women	43	0.00181, 0.00181, 0.00181, 0.00181, 0.00181

Variable	Race/ethnicity	Sex	Age	Distribution
	White	Women	44	0.00196, 0.00196, 0.00196, 0.00196, 0.00196
	White	Women	45	0.00213, 0.00213, 0.00213, 0.00213, 0.00213
	White	Women	46	0.00232, 0.00232, 0.00232, 0.00232, 0.00232
	White	Women	47	0.00252, 0.00252, 0.00252, 0.00252, 0.00252
	White	Women	48	0.00275, 0.00275, 0.00275, 0.00275, 0.00275
	White	Women	49	0.003, 0.003, 0.003, 0.003, 0.003
	White	Women	50	0.00328, 0.00328, 0.00328, 0.00328, 0.00328
	White	Women	51	0.00358, 0.00358, 0.00358, 0.00358, 0.00358
	White	Women	52	0.00389, 0.00389, 0.00389, 0.00389, 0.00389
	White	Women	53	0.00421, 0.00421, 0.00421, 0.00421, 0.00421
	White	Women	54	0.00454, 0.00454, 0.00454, 0.00454, 0.00454
	White	Women	55	0.00488, 0.00488, 0.00488, 0.00488, 0.00488
	White	Women	56	0.00521, 0.00521, 0.00521, 0.00521, 0.00521
	White	Women	57	0.00556, 0.00556, 0.00556, 0.00556, 0.00556
	White	Women	58	0.00592, 0.00592, 0.00592, 0.00592, 0.00592
	White	Women	59	0.00631, 0.00631, 0.00631, 0.00631, 0.00631
	White	Women	60	0.00673, 0.00673, 0.00673, 0.00673, 0.00673
	White	Women	61	0.00719, 0.00719, 0.00719, 0.00719, 0.00719
	White	Women	62	0.00771, 0.00771, 0.00771, 0.00771, 0.00771
	White	Women	63	0.00829, 0.00829, 0.00829, 0.00829, 0.00829
	White	Women	64	0.00895, 0.00895, 0.00895, 0.00895, 0.00895
	White	Women	65	0.00971, 0.00971, 0.00971, 0.00971, 0.00971
	White	Women	66	0.0106, 0.0106, 0.0106, 0.0106, 0.0106
	White	Women	67	0.0116, 0.0116, 0.0116, 0.0116, 0.0116
	White	Women	68	0.0127, 0.0127, 0.0127, 0.0127, 0.0127
	White	Women	69	0.0141, 0.0141, 0.0141, 0.0141, 0.0141
	White	Women	70	0.0155, 0.0155, 0.0155, 0.0155, 0.0155
	White	Women	71	0.0172, 0.0172, 0.0172, 0.0172, 0.0172
	White	Women	72	0.0189, 0.0189, 0.0189, 0.0189, 0.0189
	White	Women	73	0.0208, 0.0208, 0.0208, 0.0208, 0.0208
	White	Women	74	0.0229, 0.0229, 0.0229, 0.0229, 0.0229
	White	Women	75	0.0253, 0.0253, 0.0253, 0.0253, 0.0253
	White	Women	76	0.0279, 0.0279, 0.0279, 0.0279, 0.0279
	White	Women	77	0.0309, 0.0309, 0.0309, 0.0309, 0.0309
	White	Women	78	0.0344, 0.0344, 0.0344, 0.0344, 0.0344
	White	Women	79	0.0385, 0.0385, 0.0385, 0.0385, 0.0385
	White	Women	80	0.0432, 0.0432, 0.0432, 0.0432, 0.0432
	White	Women	81	0.0484, 0.0484, 0.0484, 0.0484, 0.0484
	White	Women	82	0.0542, 0.0542, 0.0542, 0.0542, 0.0542
	White	Women	83	0.0604, 0.0604, 0.0604, 0.0604, 0.0604
	White	Women	84	0.0668, 0.0668, 0.0668, 0.0668, 0.0668
Total mortality rates for 2016 (0.01, 0.2, 0.5, 0.8. 0.99 percentiles of the empirical distribution produced during forecasting)				
	Black	Men	30	0.0019, 0.0021, 0.00231, 0.00254, 0.0028

Variable	Race/ethnicity	Sex	Age	Distribution
	Black	Men	31	0.00195, 0.00214, 0.00235, 0.00257, 0.00282
	Black	Men	32	0.00199, 0.00218, 0.00239, 0.00261, 0.00286
	Black	Men	33	0.00204, 0.00223, 0.00244, 0.00267, 0.00293
	Black	Men	34	0.00211, 0.00231, 0.00252, 0.00276, 0.00302
	Black	Men	35	0.00219, 0.0024, 0.00262, 0.00287, 0.00314
	Black	Men	36	0.00229, 0.00251, 0.00274, 0.00299, 0.00327
	Black	Men	37	0.00241, 0.00263, 0.00286, 0.00311, 0.00339
	Black	Men	38	0.00253, 0.00275, 0.00298, 0.00324, 0.00352
	Black	Men	39	0.00263, 0.00286, 0.00311, 0.00339, 0.00369
	Black	Men	40	0.00272, 0.00298, 0.00326, 0.00356, 0.0039
	Black	Men	41	0.00283, 0.00312, 0.00343, 0.00377, 0.00415
	Black	Men	42	0.00298, 0.00329, 0.00364, 0.00402, 0.00445
	Black	Men	43	0.00316, 0.00351, 0.00389, 0.00432, 0.00479
	Black	Men	44	0.00338, 0.00376, 0.00419, 0.00466, 0.00519
	Black	Men	45	0.00364, 0.00406, 0.00453, 0.00505, 0.00564
	Black	Men	46	0.00396, 0.00441, 0.00492, 0.00548, 0.00612
	Black	Men	47	0.00435, 0.00483, 0.00536, 0.00595, 0.00661
	Black	Men	48	0.00481, 0.00531, 0.00586, 0.00645, 0.00712
	Black	Men	49	0.00536, 0.00587, 0.00642, 0.00702, 0.00768
	Black	Men	50	0.00597, 0.00649, 0.00705, 0.00765, 0.00832
	Black	Men	51	0.00665, 0.00718, 0.00775, 0.00835, 0.00902
	Black	Men	52	0.00742, 0.00795, 0.00851, 0.00911, 0.00976
	Black	Men	53	0.00827, 0.00879, 0.00934, 0.00993, 0.0106
	Black	Men	54	0.00917, 0.00969, 0.0102, 0.0108, 0.0114
	Black	Men	55	0.0101, 0.0106, 0.0112, 0.0118, 0.0124
	Black	Men	56	0.0111, 0.0117, 0.0122, 0.0128, 0.0135
	Black	Men	57	0.0122, 0.0128, 0.0134, 0.014, 0.0146
	Black	Men	58	0.0134, 0.014, 0.0146, 0.0152, 0.0158
	Black	Men	59	0.0147, 0.0153, 0.0159, 0.0165, 0.0172
	Black	Men	60	0.016, 0.0166, 0.0173, 0.018, 0.0187
	Black	Men	61	0.0172, 0.0179, 0.0187, 0.0195, 0.0203
	Black	Men	62	0.0185, 0.0193, 0.0201, 0.0209, 0.0219
	Black	Men	63	0.0197, 0.0206, 0.0214, 0.0224, 0.0234
	Black	Men	64	0.0209, 0.0218, 0.0228, 0.0238, 0.0248
	Black	Men	65	0.022, 0.023, 0.0241, 0.0251, 0.0263
	Black	Men	66	0.0233, 0.0243, 0.0254, 0.0266, 0.0278
	Black	Men	67	0.0245, 0.0257, 0.0269, 0.0281, 0.0294
	Black	Men	68	0.026, 0.0272, 0.0285, 0.0299, 0.0313
	Black	Men	69	0.0275, 0.0289, 0.0304, 0.0319, 0.0336
	Black	Men	70	0.0293, 0.0309, 0.0326, 0.0343, 0.0362
	Black	Men	71	0.0313, 0.0331, 0.035, 0.037, 0.0391
	Black	Men	72	0.0336, 0.0356, 0.0376, 0.0399, 0.0422
	Black	Men	73	0.0361, 0.0383, 0.0405, 0.0429, 0.0455

Variable	Race/ethnicity	Sex	Age	Distribution
	Black	Men	74	0.0391, 0.0413, 0.0436, 0.0461, 0.0488
	Black	Men	75	0.0424, 0.0446, 0.047, 0.0494, 0.0521
	Black	Men	76	0.046, 0.0483, 0.0506, 0.053, 0.0556
	Black	Men	77	0.0499, 0.0522, 0.0545, 0.057, 0.0595
	Black	Men	78	0.0539, 0.0563, 0.0588, 0.0615, 0.0642
	Black	Men	79	0.0582, 0.0608, 0.0636, 0.0665, 0.0696
	Black	Men	80	0.0628, 0.0658, 0.0689, 0.0722, 0.0756
	Black	Men	81	0.0677, 0.071, 0.0745, 0.0782, 0.0821
	Black	Men	82	0.0727, 0.0765, 0.0804, 0.0846, 0.089
	Black	Men	83	0.0777, 0.0819, 0.0864, 0.091, 0.096
	Black	Men	84	0.0824, 0.0871, 0.0921, 0.0974, 0.103
	Black	Women	30	0.000754, 0.000835, 0.000924, 0.00102, 0.00113
	Black	Women	31	0.00082, 0.000904, 0.000995, 0.0011, 0.00121
	Black	Women	32	0.000898, 0.000983, 0.00108, 0.00118, 0.00129
	Black	Women	33	0.000985, 0.00107, 0.00116, 0.00126, 0.00137
	Black	Women	34	0.00107, 0.00116, 0.00125, 0.00136, 0.00147
	Black	Women	35	0.00115, 0.00125, 0.00135, 0.00146, 0.00158
	Black	Women	36	0.00123, 0.00133, 0.00144, 0.00156, 0.00169
	Black	Women	37	0.00132, 0.00143, 0.00155, 0.00168, 0.00182
	Black	Women	38	0.00141, 0.00153, 0.00166, 0.0018, 0.00195
	Black	Women	39	0.00153, 0.00165, 0.00179, 0.00194, 0.00209
	Black	Women	40	0.00166, 0.00179, 0.00194, 0.00209, 0.00226
	Black	Women	41	0.00181, 0.00195, 0.00211, 0.00227, 0.00245
	Black	Women	42	0.00199, 0.00214, 0.00229, 0.00246, 0.00264
	Black	Women	43	0.00221, 0.00235, 0.00251, 0.00267, 0.00285
	Black	Women	44	0.00244, 0.00259, 0.00274, 0.0029, 0.00307
	Black	Women	45	0.00269, 0.00284, 0.00299, 0.00316, 0.00333
	Black	Women	46	0.00294, 0.00311, 0.00328, 0.00345, 0.00364
	Black	Women	47	0.0032, 0.00339, 0.00359, 0.00379, 0.00401
	Black	Women	48	0.00349, 0.0037, 0.00392, 0.00416, 0.00441
	Black	Women	49	0.00382, 0.00405, 0.00429, 0.00455, 0.00482
	Black	Women	50	0.0042, 0.00444, 0.00469, 0.00495, 0.00523
	Black	Women	51	0.00464, 0.00487, 0.00511, 0.00536, 0.00563
	Black	Women	52	0.00514, 0.00535, 0.00556, 0.00579, 0.00603
	Black	Women	53	0.00564, 0.00584, 0.00605, 0.00626, 0.00648
	Black	Women	54	0.00612, 0.00634, 0.00656, 0.0068, 0.00704
	Black	Women	55	0.00659, 0.00685, 0.00711, 0.00739, 0.00768
	Black	Women	56	0.00709, 0.00739, 0.00769, 0.00801, 0.00834
	Black	Women	57	0.00763, 0.00796, 0.00829, 0.00864, 0.009
	Black	Women	58	0.0082, 0.00855, 0.0089, 0.00927, 0.00966
	Black	Women	59	0.00878, 0.00914, 0.00952, 0.00992, 0.0103
	Black	Women	60	0.00935, 0.00975, 0.0102, 0.0106, 0.011
	Black	Women	61	0.00994, 0.0104, 0.0108, 0.0112, 0.0117

Variable	Race/ethnicity	Sex	Age	Distribution
	Black	Women	62	0.0105, 0.011, 0.0115, 0.0119, 0.0125
	Black	Women	63	0.0112, 0.0117, 0.0122, 0.0127, 0.0133
	Black	Women	64	0.0118, 0.0124, 0.013, 0.0136, 0.0142
	Black	Women	65	0.0126, 0.0132, 0.0138, 0.0145, 0.0152
	Black	Women	66	0.0134, 0.0141, 0.0148, 0.0156, 0.0164
	Black	Women	67	0.0145, 0.0152, 0.016, 0.0168, 0.0176
	Black	Women	68	0.0156, 0.0164, 0.0172, 0.0181, 0.019
	Black	Women	69	0.0169, 0.0178, 0.0187, 0.0196, 0.0206
	Black	Women	70	0.0183, 0.0192, 0.0202, 0.0213, 0.0224
	Black	Women	71	0.0197, 0.0208, 0.0219, 0.023, 0.0243
	Black	Women	72	0.0214, 0.0225, 0.0237, 0.025, 0.0263
	Black	Women	73	0.0231, 0.0244, 0.0257, 0.027, 0.0284
	Black	Women	74	0.0251, 0.0264, 0.0278, 0.0292, 0.0308
	Black	Women	75	0.0274, 0.0287, 0.0302, 0.0317, 0.0333
	Black	Women	76	0.0299, 0.0313, 0.0328, 0.0344, 0.0361
	Black	Women	77	0.0327, 0.0342, 0.0358, 0.0376, 0.0394
	Black	Women	78	0.0358, 0.0375, 0.0392, 0.0411, 0.0431
	Black	Women	79	0.0392, 0.0411, 0.0431, 0.0451, 0.0473
	Black	Women	80	0.0429, 0.0451, 0.0473, 0.0496, 0.0521
	Black	Women	81	0.0468, 0.0493, 0.0519, 0.0546, 0.0575
	Black	Women	82	0.0508, 0.0537, 0.0568, 0.06, 0.0635
	Black	Women	83	0.0547, 0.0582, 0.0618, 0.0657, 0.0699
	Black	Women	84	0.0586, 0.0626, 0.0669, 0.0715, 0.0765
	Hispanic	Men	30	0.000831, 0.00091, 0.000995, 0.00109, 0.00119
	Hispanic	Men	31	0.000846, 0.000926, 0.00101, 0.00111, 0.00121
	Hispanic	Men	32	0.000864, 0.000944, 0.00103, 0.00113, 0.00123
	Hispanic	Men	33	0.000879, 0.000962, 0.00105, 0.00115, 0.00126
	Hispanic	Men	34	0.000891, 0.00098, 0.00108, 0.00119, 0.0013
	Hispanic	Men	35	0.000909, 0.001, 0.00111, 0.00122, 0.00135
	Hispanic	Men	36	0.000942, 0.00104, 0.00115, 0.00127, 0.0014
	Hispanic	Men	37	0.000992, 0.00109, 0.0012, 0.00132, 0.00145
	Hispanic	Men	38	0.00105, 0.00116, 0.00127, 0.00139, 0.00152
	Hispanic	Men	39	0.00112, 0.00123, 0.00134, 0.00146, 0.0016
	Hispanic	Men	40	0.0012, 0.00131, 0.00143, 0.00156, 0.0017
	Hispanic	Men	41	0.00129, 0.00141, 0.00153, 0.00167, 0.00181
	Hispanic	Men	42	0.00141, 0.00153, 0.00165, 0.00179, 0.00194
	Hispanic	Men	43	0.00155, 0.00167, 0.0018, 0.00193, 0.00208
	Hispanic	Men	44	0.00173, 0.00185, 0.00197, 0.0021, 0.00224
	Hispanic	Men	45	0.00193, 0.00205, 0.00217, 0.0023, 0.00244
	Hispanic	Men	46	0.00214, 0.00227, 0.0024, 0.00255, 0.0027
	Hispanic	Men	47	0.00236, 0.00251, 0.00266, 0.00283, 0.00301
	Hispanic	Men	48	0.00259, 0.00277, 0.00295, 0.00314, 0.00335
	Hispanic	Men	49	0.00285, 0.00304, 0.00325, 0.00347, 0.00371

Variable	Race/ethnicity	Sex	Age	Distribution
	Hispanic	Men	50	0.00314, 0.00335, 0.00357, 0.0038, 0.00406
	Hispanic	Men	51	0.00348, 0.00369, 0.0039, 0.00413, 0.00437
	Hispanic	Men	52	0.00389, 0.00406, 0.00425, 0.00445, 0.00465
	Hispanic	Men	53	0.00434, 0.00448, 0.00462, 0.00477, 0.00492
	Hispanic	Men	54	0.00476, 0.00489, 0.00501, 0.00514, 0.00528
	Hispanic	Men	55	0.00511, 0.00527, 0.00543, 0.0056, 0.00577
	Hispanic	Men	56	0.00546, 0.00567, 0.00588, 0.0061, 0.00634
	Hispanic	Men	57	0.00586, 0.00611, 0.00637, 0.00664, 0.00692
	Hispanic	Men	58	0.00632, 0.0066, 0.00689, 0.00719, 0.00751
	Hispanic	Men	59	0.0068, 0.00712, 0.00745, 0.00779, 0.00815
	Hispanic	Men	60	0.00733, 0.00768, 0.00804, 0.00842, 0.00882
	Hispanic	Men	61	0.0079, 0.00827, 0.00866, 0.00906, 0.00949
	Hispanic	Men	62	0.00852, 0.0089, 0.0093, 0.00972, 0.0102
	Hispanic	Men	63	0.00917, 0.00957, 0.00998, 0.0104, 0.0109
	Hispanic	Men	64	0.00981, 0.0102, 0.0107, 0.0112, 0.0117
	Hispanic	Men	65	0.0105, 0.0109, 0.0115, 0.012, 0.0126
	Hispanic	Men	66	0.0111, 0.0117, 0.0123, 0.0129, 0.0136
	Hispanic	Men	67	0.0119, 0.0126, 0.0132, 0.014, 0.0147
	Hispanic	Men	68	0.0128, 0.0135, 0.0143, 0.0151, 0.016
	Hispanic	Men	69	0.0138, 0.0146, 0.0155, 0.0164, 0.0174
	Hispanic	Men	70	0.015, 0.0159, 0.0169, 0.0179, 0.019
	Hispanic	Men	71	0.0163, 0.0173, 0.0184, 0.0195, 0.0207
	Hispanic	Men	72	0.0178, 0.0189, 0.0201, 0.0213, 0.0226
	Hispanic	Men	73	0.0195, 0.0207, 0.0219, 0.0232, 0.0246
	Hispanic	Men	74	0.0214, 0.0226, 0.0239, 0.0253, 0.0267
	Hispanic	Men	75	0.0235, 0.0248, 0.0261, 0.0276, 0.0291
	Hispanic	Men	76	0.0259, 0.0272, 0.0287, 0.0302, 0.0318
	Hispanic	Men	77	0.0285, 0.03, 0.0316, 0.0332, 0.0349
	Hispanic	Men	78	0.0316, 0.0332, 0.0349, 0.0367, 0.0387
	Hispanic	Men	79	0.0351, 0.0369, 0.0388, 0.0408, 0.043
	Hispanic	Men	80	0.039, 0.0411, 0.0432, 0.0455, 0.0479
	Hispanic	Men	81	0.0434, 0.0457, 0.0482, 0.0507, 0.0534
	Hispanic	Men	82	0.048, 0.0506, 0.0534, 0.0564, 0.0595
	Hispanic	Men	83	0.0525, 0.0556, 0.0589, 0.0623, 0.066
	Hispanic	Men	84	0.0569, 0.0605, 0.0642, 0.0682, 0.0725
	Hispanic	Women	30	0.000335, 0.000374, 0.000418, 0.000466, 0.000521
	Hispanic	Women	31	0.000353, 0.000394, 0.000439, 0.00049, 0.000547
	Hispanic	Women	32	0.000374, 0.000416, 0.000462, 0.000513, 0.00057
	Hispanic	Women	33	0.000399, 0.000441, 0.000487, 0.000537, 0.000593
	Hispanic	Women	34	0.000424, 0.000467, 0.000513, 0.000564, 0.000621
	Hispanic	Women	35	0.000448, 0.000493, 0.000542, 0.000597, 0.000657
	Hispanic	Women	36	0.000472, 0.000522, 0.000576, 0.000636, 0.000702
	Hispanic	Women	37	0.000501, 0.000555, 0.000614, 0.00068, 0.000753

Variable	Race/ethnicity	Sex	Age	Distribution
	Hispanic	Women	38	0.000537, 0.000595, 0.000659, 0.000729, 0.000808
	Hispanic	Women	39	0.000583, 0.000643, 0.00071, 0.000783, 0.000865
	Hispanic	Women	40	0.000638, 0.0007, 0.000768, 0.000842, 0.000925
	Hispanic	Women	41	0.000704, 0.000766, 0.000834, 0.000908, 0.000989
	Hispanic	Women	42	0.00078, 0.000843, 0.00091, 0.000982, 0.00106
	Hispanic	Women	43	0.000866, 0.000929, 0.000996, 0.00107, 0.00115
	Hispanic	Women	44	0.000962, 0.00103, 0.0011, 0.00117, 0.00125
	Hispanic	Women	45	0.00107, 0.00113, 0.00121, 0.00128, 0.00137
	Hispanic	Women	46	0.00118, 0.00125, 0.00133, 0.00141, 0.0015
	Hispanic	Women	47	0.0013, 0.00138, 0.00147, 0.00156, 0.00165
	Hispanic	Women	48	0.00143, 0.00152, 0.00161, 0.00171, 0.00182
	Hispanic	Women	49	0.00156, 0.00166, 0.00176, 0.00188, 0.002
	Hispanic	Women	50	0.0017, 0.00181, 0.00193, 0.00205, 0.00219
	Hispanic	Women	51	0.00185, 0.00197, 0.0021, 0.00224, 0.00239
	Hispanic	Women	52	0.00201, 0.00215, 0.00229, 0.00244, 0.00261
	Hispanic	Women	53	0.00219, 0.00234, 0.00249, 0.00266, 0.00284
	Hispanic	Women	54	0.00238, 0.00254, 0.00271, 0.00289, 0.00308
	Hispanic	Women	55	0.00258, 0.00276, 0.00294, 0.00314, 0.00335
	Hispanic	Women	56	0.00281, 0.00299, 0.00319, 0.0034, 0.00362
	Hispanic	Women	57	0.00306, 0.00326, 0.00346, 0.00368, 0.00391
	Hispanic	Women	58	0.00334, 0.00354, 0.00375, 0.00398, 0.00422
	Hispanic	Women	59	0.00363, 0.00384, 0.00407, 0.00431, 0.00457
	Hispanic	Women	60	0.00393, 0.00417, 0.00442, 0.00469, 0.00497
	Hispanic	Women	61	0.00426, 0.00453, 0.0048, 0.00509, 0.00541
	Hispanic	Women	62	0.00462, 0.00491, 0.00522, 0.00554, 0.00589
	Hispanic	Women	63	0.00501, 0.00533, 0.00567, 0.00603, 0.00642
	Hispanic	Women	64	0.00543, 0.00579, 0.00617, 0.00657, 0.00701
	Hispanic	Women	65	0.00589, 0.00629, 0.00671, 0.00716, 0.00765
	Hispanic	Women	66	0.00641, 0.00685, 0.00732, 0.00781, 0.00835
	Hispanic	Women	67	0.00701, 0.00749, 0.00799, 0.00852, 0.0091
	Hispanic	Women	68	0.00771, 0.00821, 0.00874, 0.00931, 0.00992
	Hispanic	Women	69	0.0085, 0.00903, 0.00959, 0.0102, 0.0108
	Hispanic	Women	70	0.00938, 0.00995, 0.0106, 0.0112, 0.0119
	Hispanic	Women	71	0.0104, 0.011, 0.0116, 0.0123, 0.0131
	Hispanic	Women	72	0.0114, 0.0121, 0.0128, 0.0136, 0.0144
	Hispanic	Women	73	0.0126, 0.0134, 0.0142, 0.015, 0.0159
	Hispanic	Women	74	0.014, 0.0148, 0.0157, 0.0166, 0.0176
	Hispanic	Women	75	0.0156, 0.0165, 0.0174, 0.0184, 0.0194
	Hispanic	Women	76	0.0175, 0.0184, 0.0194, 0.0204, 0.0214
	Hispanic	Women	77	0.0197, 0.0206, 0.0216, 0.0227, 0.0238
	Hispanic	Women	78	0.0221, 0.0232, 0.0242, 0.0254, 0.0266
	Hispanic	Women	79	0.0249, 0.0261, 0.0273, 0.0286, 0.0299
	Hispanic	Women	80	0.028, 0.0293, 0.0308, 0.0323, 0.0338

Variable	Race/ethnicity	Sex	Age	Distribution
	Hispanic	Women	81	0.0313, 0.0329, 0.0346, 0.0364, 0.0383
	Hispanic	Women	82	0.0348, 0.0368, 0.0389, 0.0411, 0.0434
	Hispanic	Women	83	0.0384, 0.0408, 0.0433, 0.046, 0.0489
	Hispanic	Women	84	0.0421, 0.0449, 0.0479, 0.0511, 0.0545
	White	Men	30	0.00134, 0.00147, 0.00161, 0.00177, 0.00195
	White	Men	31	0.00138, 0.00151, 0.00166, 0.00182, 0.00199
	White	Men	32	0.00143, 0.00156, 0.0017, 0.00185, 0.00203
	White	Men	33	0.00147, 0.0016, 0.00174, 0.00189, 0.00205
	White	Men	34	0.00151, 0.00164, 0.00178, 0.00193, 0.00209
	White	Men	35	0.00155, 0.00168, 0.00182, 0.00197, 0.00214
	White	Men	36	0.00159, 0.00173, 0.00187, 0.00203, 0.0022
	White	Men	37	0.00164, 0.00178, 0.00193, 0.0021, 0.00227
	White	Men	38	0.00171, 0.00185, 0.00201, 0.00218, 0.00237
	White	Men	39	0.00179, 0.00194, 0.0021, 0.00228, 0.00248
	White	Men	40	0.00188, 0.00205, 0.00222, 0.00241, 0.00261
	White	Men	41	0.00201, 0.00218, 0.00236, 0.00255, 0.00277
	White	Men	42	0.00217, 0.00234, 0.00252, 0.00272, 0.00294
	White	Men	43	0.00236, 0.00254, 0.00272, 0.00292, 0.00314
	White	Men	44	0.00259, 0.00277, 0.00295, 0.00315, 0.00337
	White	Men	45	0.00285, 0.00303, 0.00322, 0.00343, 0.00365
	White	Men	46	0.00312, 0.00332, 0.00353, 0.00375, 0.00399
	White	Men	47	0.00341, 0.00363, 0.00387, 0.00413, 0.0044
	White	Men	48	0.00373, 0.00399, 0.00425, 0.00454, 0.00485
	White	Men	49	0.0041, 0.00438, 0.00467, 0.00499, 0.00533
	White	Men	50	0.00451, 0.00481, 0.00513, 0.00547, 0.00584
	White	Men	51	0.00497, 0.00529, 0.00562, 0.00597, 0.00635
	White	Men	52	0.0055, 0.00581, 0.00613, 0.00648, 0.00685
	White	Men	53	0.00606, 0.00636, 0.00667, 0.007, 0.00735
	White	Men	54	0.00662, 0.00692, 0.00723, 0.00755, 0.0079
	White	Men	55	0.00715, 0.00747, 0.0078, 0.00814, 0.00851
	White	Men	56	0.00768, 0.00803, 0.00839, 0.00877, 0.00917
	White	Men	57	0.00823, 0.00861, 0.00901, 0.00942, 0.00985
	White	Men	58	0.00881, 0.00923, 0.00965, 0.0101, 0.0106
	White	Men	59	0.00944, 0.00988, 0.0103, 0.0108, 0.0113
	White	Men	60	0.0101, 0.0106, 0.0111, 0.0116, 0.0121
	White	Men	61	0.0108, 0.0113, 0.0118, 0.0123, 0.0129
	White	Men	62	0.0116, 0.0121, 0.0126, 0.0131, 0.0137
	White	Men	63	0.0124, 0.0129, 0.0135, 0.014, 0.0146
	White	Men	64	0.0133, 0.0138, 0.0144, 0.015, 0.0156
	White	Men	65	0.0142, 0.0148, 0.0154, 0.016, 0.0167
	White	Men	66	0.0151, 0.0158, 0.0165, 0.0172, 0.018
	White	Men	67	0.0162, 0.017, 0.0178, 0.0186, 0.0194
	White	Men	68	0.0175, 0.0184, 0.0192, 0.0201, 0.0211

Variable	Race/ethnicity	Sex	Age	Distribution
	White	Men	69	0.019, 0.0199, 0.0209, 0.0219, 0.023
	White	Men	70	0.0207, 0.0217, 0.0228, 0.0239, 0.0252
	White	Men	71	0.0226, 0.0237, 0.0249, 0.0262, 0.0275
	White	Men	72	0.0247, 0.026, 0.0273, 0.0287, 0.0301
	White	Men	73	0.0271, 0.0284, 0.0299, 0.0313, 0.0329
	White	Men	74	0.0297, 0.0312, 0.0327, 0.0342, 0.0359
	White	Men	75	0.0327, 0.0342, 0.0358, 0.0374, 0.0392
	White	Men	76	0.036, 0.0376, 0.0392, 0.041, 0.0428
	White	Men	77	0.0397, 0.0414, 0.0432, 0.0451, 0.0471
	White	Men	78	0.0439, 0.0458, 0.0478, 0.0499, 0.0521
	White	Men	79	0.0487, 0.0509, 0.0532, 0.0555, 0.058
	White	Men	80	0.0542, 0.0567, 0.0593, 0.062, 0.0648
	White	Men	81	0.0603, 0.0632, 0.0662, 0.0693, 0.0726
	White	Men	82	0.0668, 0.0702, 0.0736, 0.0773, 0.0811
	White	Men	83	0.0735, 0.0774, 0.0814, 0.0856, 0.0901
	White	Men	84	0.0802, 0.0846, 0.0892, 0.0941, 0.0993
	White	Women	30	0.000648, 0.000702, 0.000759, 0.000822, 0.00089
	White	Women	31	0.000683, 0.000741, 0.000804, 0.000872, 0.000947
	White	Women	32	0.000723, 0.000784, 0.00085, 0.000922, 0.001
	White	Women	33	0.000768, 0.00083, 0.000898, 0.00097, 0.00105
	White	Women	34	0.000813, 0.000878, 0.000948, 0.00102, 0.0011
	White	Women	35	0.000857, 0.000927, 0.001, 0.00108, 0.00117
	White	Women	36	0.000905, 0.000981, 0.00106, 0.00115, 0.00125
	White	Women	37	0.00096, 0.00104, 0.00113, 0.00123, 0.00134
	White	Women	38	0.00102, 0.00111, 0.00121, 0.00132, 0.00144
	White	Women	39	0.00109, 0.00119, 0.0013, 0.00142, 0.00155
	White	Women	40	0.00117, 0.00128, 0.0014, 0.00153, 0.00167
	White	Women	41	0.00127, 0.00139, 0.00151, 0.00165, 0.00181
	White	Women	42	0.00138, 0.00151, 0.00164, 0.00179, 0.00195
	White	Women	43	0.00151, 0.00164, 0.00179, 0.00194, 0.00211
	White	Women	44	0.00165, 0.0018, 0.00195, 0.00212, 0.0023
	White	Women	45	0.00181, 0.00197, 0.00214, 0.00232, 0.00252
	White	Women	46	0.00199, 0.00216, 0.00234, 0.00254, 0.00276
	White	Women	47	0.00219, 0.00237, 0.00256, 0.00277, 0.003
	White	Women	48	0.00242, 0.0026, 0.0028, 0.00301, 0.00324
	White	Women	49	0.00266, 0.00285, 0.00305, 0.00327, 0.0035
	White	Women	50	0.0029, 0.00311, 0.00332, 0.00355, 0.0038
	White	Women	51	0.00315, 0.00337, 0.0036, 0.00385, 0.00412
	White	Women	52	0.0034, 0.00364, 0.00389, 0.00417, 0.00446
	White	Women	53	0.00365, 0.00391, 0.00419, 0.00449, 0.00482
	White	Women	54	0.00391, 0.00419, 0.0045, 0.00482, 0.00518
	White	Women	55	0.00419, 0.00448, 0.0048, 0.00514, 0.00551
	White	Women	56	0.0045, 0.00479, 0.0051, 0.00543, 0.00579

Variable	Race/ethnicity	Sex	Age	Distribution
	White	Women	57	0.00486, 0.00513, 0.00541, 0.00571, 0.00602
	White	Women	58	0.00524, 0.00548, 0.00573, 0.00599, 0.00627
	White	Women	59	0.00562, 0.00585, 0.00608, 0.00633, 0.00659
	White	Women	60	0.00597, 0.00622, 0.00648, 0.00675, 0.00704
	White	Women	61	0.00635, 0.00664, 0.00694, 0.00726, 0.00759
	White	Women	62	0.0068, 0.00713, 0.00748, 0.00785, 0.00823
	White	Women	63	0.00733, 0.00771, 0.00811, 0.00852, 0.00897
	White	Women	64	0.00796, 0.00838, 0.00883, 0.0093, 0.0098
	White	Women	65	0.00868, 0.00916, 0.00965, 0.0102, 0.0107
	White	Women	66	0.00951, 0.01, 0.0106, 0.0112, 0.0118
	White	Women	67	0.0104, 0.011, 0.0116, 0.0123, 0.013
	White	Women	68	0.0114, 0.0121, 0.0128, 0.0135, 0.0143
	White	Women	69	0.0125, 0.0133, 0.0141, 0.0149, 0.0158
	White	Women	70	0.0137, 0.0146, 0.0155, 0.0165, 0.0175
	White	Women	71	0.0151, 0.0161, 0.0171, 0.0182, 0.0194
	White	Women	72	0.0165, 0.0177, 0.0188, 0.0201, 0.0214
	White	Women	73	0.0182, 0.0194, 0.0207, 0.0221, 0.0236
	White	Women	74	0.0202, 0.0215, 0.0228, 0.0243, 0.0259
	White	Women	75	0.0225, 0.0238, 0.0252, 0.0267, 0.0283
	White	Women	76	0.0251, 0.0265, 0.0279, 0.0294, 0.031
	White	Women	77	0.0282, 0.0295, 0.031, 0.0325, 0.0341
	White	Women	78	0.0315, 0.033, 0.0345, 0.0361, 0.0378
	White	Women	79	0.0353, 0.0369, 0.0386, 0.0404, 0.0422
	White	Women	80	0.0396, 0.0414, 0.0433, 0.0453, 0.0474
	White	Women	81	0.0442, 0.0463, 0.0485, 0.0508, 0.0532
	White	Women	82	0.0493, 0.0517, 0.0542, 0.0568, 0.0596
	White	Women	83	0.0545, 0.0573, 0.0602, 0.0633, 0.0666
	White	Women	84	0.0598, 0.063, 0.0665, 0.0701, 0.0739
Total mortality rates for 2017 (0.01, 0.2, 0.5, 0.8. 0.99 percentiles of the empirical distribution produced during forecasting)				
	Black	Men	30	0.00186, 0.00208, 0.00231, 0.00257, 0.00286
	Black	Men	31	0.00192, 0.00212, 0.00235, 0.00259, 0.00287
	Black	Men	32	0.00197, 0.00217, 0.00239, 0.00263, 0.0029
	Black	Men	33	0.00202, 0.00222, 0.00244, 0.00269, 0.00296
	Black	Men	34	0.00208, 0.00229, 0.00252, 0.00277, 0.00305
	Black	Men	35	0.00215, 0.00237, 0.00261, 0.00287, 0.00317
	Black	Men	36	0.00225, 0.00247, 0.00272, 0.00299, 0.00329
	Black	Men	37	0.00235, 0.00258, 0.00283, 0.00311, 0.00341
	Black	Men	38	0.00245, 0.00269, 0.00295, 0.00323, 0.00354
	Black	Men	39	0.00254, 0.00279, 0.00307, 0.00337, 0.00371
	Black	Men	40	0.00262, 0.0029, 0.00321, 0.00354, 0.00392
	Black	Men	41	0.00272, 0.00303, 0.00337, 0.00375, 0.00418
	Black	Men	42	0.00285, 0.00319, 0.00357, 0.004, 0.00448
	Black	Men	43	0.00301, 0.00339, 0.00382, 0.00429, 0.00484

Variable	Race/ethnicity	Sex	Age	Distribution
	Black	Men	44	0.00321, 0.00363, 0.00411, 0.00464, 0.00525
	Black	Men	45	0.00345, 0.00392, 0.00445, 0.00504, 0.00573
	Black	Men	46	0.00374, 0.00426, 0.00483, 0.00548, 0.00623
	Black	Men	47	0.00411, 0.00466, 0.00527, 0.00596, 0.00675
	Black	Men	48	0.00456, 0.00513, 0.00576, 0.00647, 0.00728
	Black	Men	49	0.00509, 0.00567, 0.00632, 0.00704, 0.00785
	Black	Men	50	0.00568, 0.00629, 0.00695, 0.00768, 0.00849
	Black	Men	51	0.00635, 0.00697, 0.00764, 0.00838, 0.0092
	Black	Men	52	0.0071, 0.00773, 0.00841, 0.00914, 0.00995
	Black	Men	53	0.00793, 0.00856, 0.00924, 0.00996, 0.0108
	Black	Men	54	0.00882, 0.00946, 0.0101, 0.0108, 0.0116
	Black	Men	55	0.00976, 0.0104, 0.0111, 0.0118, 0.0126
	Black	Men	56	0.0108, 0.0114, 0.0121, 0.0129, 0.0137
	Black	Men	57	0.0119, 0.0126, 0.0133, 0.014, 0.0148
	Black	Men	58	0.0131, 0.0138, 0.0145, 0.0152, 0.016
	Black	Men	59	0.0144, 0.0151, 0.0158, 0.0165, 0.0173
	Black	Men	60	0.0156, 0.0163, 0.0171, 0.0179, 0.0188
	Black	Men	61	0.0168, 0.0177, 0.0185, 0.0194, 0.0203
	Black	Men	62	0.0181, 0.019, 0.0199, 0.0208, 0.0218
	Black	Men	63	0.0193, 0.0202, 0.0212, 0.0222, 0.0233
	Black	Men	64	0.0204, 0.0214, 0.0224, 0.0235, 0.0247
	Black	Men	65	0.0215, 0.0226, 0.0237, 0.0249, 0.0261
	Black	Men	66	0.0227, 0.0238, 0.025, 0.0263, 0.0276
	Black	Men	67	0.0239, 0.0252, 0.0265, 0.0278, 0.0293
	Black	Men	68	0.0253, 0.0267, 0.0281, 0.0296, 0.0312
	Black	Men	69	0.0268, 0.0283, 0.0299, 0.0316, 0.0334
	Black	Men	70	0.0286, 0.0303, 0.0321, 0.034, 0.036
	Black	Men	71	0.0305, 0.0324, 0.0344, 0.0366, 0.0389
	Black	Men	72	0.0327, 0.0348, 0.0371, 0.0394, 0.042
	Black	Men	73	0.0352, 0.0375, 0.0399, 0.0424, 0.0452
	Black	Men	74	0.038, 0.0404, 0.0429, 0.0456, 0.0484
	Black	Men	75	0.0413, 0.0437, 0.0462, 0.0489, 0.0517
	Black	Men	76	0.0448, 0.0472, 0.0497, 0.0524, 0.0552
	Black	Men	77	0.0486, 0.051, 0.0536, 0.0563, 0.0591
	Black	Men	78	0.0525, 0.0551, 0.0579, 0.0608, 0.0638
	Black	Men	79	0.0566, 0.0596, 0.0626, 0.0658, 0.0692
	Black	Men	80	0.0611, 0.0644, 0.0678, 0.0714, 0.0753
	Black	Men	81	0.0659, 0.0696, 0.0734, 0.0775, 0.0818
	Black	Men	82	0.0708, 0.0749, 0.0793, 0.0838, 0.0887
	Black	Men	83	0.0756, 0.0803, 0.0851, 0.0903, 0.0959
	Black	Men	84	0.0802, 0.0854, 0.0909, 0.0967, 0.103
	Black	Women	30	0.000729, 0.000819, 0.000919, 0.00103, 0.00116
	Black	Women	31	0.000796, 0.000888, 0.00099, 0.0011, 0.00123

Variable	Race/ethnicity	Sex	Age	Distribution
	Black	Women	32	0.000875, 0.000968, 0.00107, 0.00118, 0.00131
	Black	Women	33	0.000961, 0.00105, 0.00116, 0.00127, 0.00139
	Black	Women	34	0.00104, 0.00114, 0.00125, 0.00136, 0.00149
	Black	Women	35	0.00112, 0.00122, 0.00134, 0.00146, 0.0016
	Black	Women	36	0.00119, 0.00131, 0.00143, 0.00156, 0.00171
	Black	Women	37	0.00127, 0.00139, 0.00153, 0.00167, 0.00184
	Black	Women	38	0.00136, 0.00149, 0.00164, 0.00179, 0.00197
	Black	Women	39	0.00147, 0.00161, 0.00176, 0.00193, 0.00211
	Black	Women	40	0.00159, 0.00174, 0.0019, 0.00208, 0.00228
	Black	Women	41	0.00174, 0.0019, 0.00207, 0.00225, 0.00246
	Black	Women	42	0.00191, 0.00208, 0.00225, 0.00244, 0.00265
	Black	Women	43	0.00212, 0.00228, 0.00246, 0.00264, 0.00284
	Black	Women	44	0.00236, 0.00252, 0.00268, 0.00286, 0.00306
	Black	Women	45	0.0026, 0.00276, 0.00294, 0.00312, 0.00331
	Black	Women	46	0.00285, 0.00303, 0.00321, 0.00341, 0.00363
	Black	Women	47	0.0031, 0.0033, 0.00352, 0.00375, 0.004
	Black	Women	48	0.00338, 0.00361, 0.00386, 0.00412, 0.00441
	Black	Women	49	0.0037, 0.00395, 0.00423, 0.00451, 0.00483
	Black	Women	50	0.00408, 0.00434, 0.00462, 0.00492, 0.00524
	Black	Women	51	0.00452, 0.00478, 0.00505, 0.00534, 0.00564
	Black	Women	52	0.00502, 0.00526, 0.0055, 0.00576, 0.00604
	Black	Women	53	0.00553, 0.00576, 0.00599, 0.00623, 0.00649
	Black	Women	54	0.00601, 0.00626, 0.00651, 0.00677, 0.00704
	Black	Women	55	0.00648, 0.00676, 0.00705, 0.00735, 0.00767
	Black	Women	56	0.00697, 0.00729, 0.00762, 0.00797, 0.00833
	Black	Women	57	0.0075, 0.00785, 0.00822, 0.0086, 0.009
	Black	Women	58	0.00805, 0.00843, 0.00882, 0.00923, 0.00966
	Black	Women	59	0.0086, 0.00901, 0.00943, 0.00987, 0.0103
	Black	Women	60	0.00915, 0.00959, 0.01, 0.0105, 0.011
	Black	Women	61	0.00971, 0.0102, 0.0107, 0.0112, 0.0117
	Black	Women	62	0.0103, 0.0108, 0.0113, 0.0119, 0.0124
	Black	Women	63	0.0109, 0.0114, 0.012, 0.0126, 0.0132
	Black	Women	64	0.0115, 0.0121, 0.0128, 0.0134, 0.0141
	Black	Women	65	0.0122, 0.0129, 0.0136, 0.0144, 0.0152
	Black	Women	66	0.0131, 0.0138, 0.0146, 0.0154, 0.0163
	Black	Women	67	0.0141, 0.0149, 0.0157, 0.0166, 0.0175
	Black	Women	68	0.0152, 0.0161, 0.017, 0.0179, 0.0189
	Black	Women	69	0.0164, 0.0174, 0.0184, 0.0194, 0.0205
	Black	Women	70	0.0177, 0.0188, 0.0199, 0.021, 0.0222
	Black	Women	71	0.0192, 0.0203, 0.0215, 0.0228, 0.0241
	Black	Women	72	0.0208, 0.022, 0.0233, 0.0247, 0.0261
	Black	Women	73	0.0225, 0.0238, 0.0252, 0.0267, 0.0283
	Black	Women	74	0.0244, 0.0259, 0.0273, 0.0289, 0.0306

Variable	Race/ethnicity	Sex	Age	Distribution
	Black	Women	75	0.0266, 0.0281, 0.0297, 0.0313, 0.0331
	Black	Women	76	0.0291, 0.0306, 0.0323, 0.034, 0.0359
	Black	Women	77	0.0318, 0.0335, 0.0353, 0.0371, 0.0391
	Black	Women	78	0.0349, 0.0367, 0.0386, 0.0406, 0.0428
	Black	Women	79	0.0382, 0.0403, 0.0424, 0.0446, 0.047
	Black	Women	80	0.0419, 0.0442, 0.0466, 0.0491, 0.0518
	Black	Women	81	0.0457, 0.0483, 0.0511, 0.0541, 0.0572
	Black	Women	82	0.0495, 0.0527, 0.056, 0.0595, 0.0632
	Black	Women	83	0.0534, 0.0571, 0.0609, 0.0651, 0.0696
	Black	Women	84	0.0572, 0.0615, 0.066, 0.0709, 0.0761
	Hispanic	Men	30	0.000821, 0.000905, 0.000997, 0.0011, 0.00121
	Hispanic	Men	31	0.000836, 0.000922, 0.00102, 0.00112, 0.00123
	Hispanic	Men	32	0.000853, 0.00094, 0.00103, 0.00114, 0.00125
	Hispanic	Men	33	0.000865, 0.000956, 0.00106, 0.00117, 0.00129
	Hispanic	Men	34	0.000873, 0.000971, 0.00108, 0.0012, 0.00133
	Hispanic	Men	35	0.000887, 0.000992, 0.00111, 0.00124, 0.00138
	Hispanic	Men	36	0.000916, 0.00102, 0.00115, 0.00128, 0.00143
	Hispanic	Men	37	0.000961, 0.00107, 0.00119, 0.00133, 0.00148
	Hispanic	Men	38	0.00102, 0.00113, 0.00125, 0.00139, 0.00154
	Hispanic	Men	39	0.00108, 0.0012, 0.00133, 0.00147, 0.00162
	Hispanic	Men	40	0.00116, 0.00128, 0.00141, 0.00156, 0.00172
	Hispanic	Men	41	0.00124, 0.00137, 0.00151, 0.00167, 0.00184
	Hispanic	Men	42	0.00135, 0.00149, 0.00163, 0.00179, 0.00197
	Hispanic	Men	43	0.00149, 0.00163, 0.00177, 0.00193, 0.0021
	Hispanic	Men	44	0.00167, 0.0018, 0.00194, 0.00209, 0.00226
	Hispanic	Men	45	0.00187, 0.002, 0.00214, 0.00229, 0.00245
	Hispanic	Men	46	0.00208, 0.00222, 0.00237, 0.00253, 0.0027
	Hispanic	Men	47	0.0023, 0.00246, 0.00263, 0.00281, 0.00301
	Hispanic	Men	48	0.00253, 0.00271, 0.00291, 0.00312, 0.00335
	Hispanic	Men	49	0.00278, 0.00299, 0.00321, 0.00345, 0.00371
	Hispanic	Men	50	0.00307, 0.00329, 0.00353, 0.00379, 0.00407
	Hispanic	Men	51	0.00341, 0.00363, 0.00387, 0.00412, 0.00438
	Hispanic	Men	52	0.00382, 0.00401, 0.00422, 0.00443, 0.00466
	Hispanic	Men	53	0.00427, 0.00443, 0.00459, 0.00475, 0.00492
	Hispanic	Men	54	0.0047, 0.00484, 0.00498, 0.00512, 0.00527
	Hispanic	Men	55	0.00505, 0.00522, 0.0054, 0.00558, 0.00577
	Hispanic	Men	56	0.00539, 0.00562, 0.00585, 0.00609, 0.00634
	Hispanic	Men	57	0.00577, 0.00605, 0.00633, 0.00662, 0.00693
	Hispanic	Men	58	0.0062, 0.00652, 0.00684, 0.00718, 0.00754
	Hispanic	Men	59	0.00666, 0.00702, 0.00738, 0.00777, 0.00818
	Hispanic	Men	60	0.00716, 0.00755, 0.00796, 0.00839, 0.00885
	Hispanic	Men	61	0.0077, 0.00812, 0.00856, 0.00902, 0.00951
	Hispanic	Men	62	0.0083, 0.00873, 0.00918, 0.00966, 0.0102

Variable	Race/ethnicity	Sex	Age	Distribution
	Hispanic	Men	63	0.00893, 0.00938, 0.00984, 0.0103, 0.0108
	Hispanic	Men	64	0.00956, 0.01, 0.0105, 0.0111, 0.0116
	Hispanic	Men	65	0.0102, 0.0107, 0.0113, 0.0119, 0.0125
	Hispanic	Men	66	0.0108, 0.0115, 0.0121, 0.0128, 0.0135
	Hispanic	Men	67	0.0116, 0.0123, 0.013, 0.0138, 0.0146
	Hispanic	Men	68	0.0124, 0.0132, 0.0141, 0.015, 0.0159
	Hispanic	Men	69	0.0134, 0.0143, 0.0153, 0.0163, 0.0173
	Hispanic	Men	70	0.0146, 0.0156, 0.0166, 0.0177, 0.0189
	Hispanic	Men	71	0.0159, 0.017, 0.0181, 0.0193, 0.0206
	Hispanic	Men	72	0.0173, 0.0185, 0.0197, 0.021, 0.0225
	Hispanic	Men	73	0.0189, 0.0202, 0.0215, 0.0229, 0.0245
	Hispanic	Men	74	0.0207, 0.0221, 0.0235, 0.025, 0.0267
	Hispanic	Men	75	0.0228, 0.0242, 0.0257, 0.0273, 0.0291
	Hispanic	Men	76	0.0251, 0.0266, 0.0282, 0.0299, 0.0318
	Hispanic	Men	77	0.0277, 0.0294, 0.0311, 0.0329, 0.0349
	Hispanic	Men	78	0.0307, 0.0325, 0.0344, 0.0365, 0.0386
	Hispanic	Men	79	0.0341, 0.0361, 0.0383, 0.0405, 0.043
	Hispanic	Men	80	0.038, 0.0403, 0.0427, 0.0452, 0.0479
	Hispanic	Men	81	0.0423, 0.0448, 0.0475, 0.0504, 0.0534
	Hispanic	Men	82	0.0468, 0.0497, 0.0527, 0.056, 0.0594
	Hispanic	Men	83	0.0512, 0.0546, 0.0581, 0.0618, 0.0659
	Hispanic	Men	84	0.0555, 0.0593, 0.0634, 0.0677, 0.0724
	Hispanic	Women	30	0.000332, 0.000372, 0.000417, 0.000468, 0.000525
	Hispanic	Women	31	0.000349, 0.000392, 0.000439, 0.000491, 0.00055
	Hispanic	Women	32	0.000371, 0.000414, 0.000461, 0.000514, 0.000574
	Hispanic	Women	33	0.000395, 0.000438, 0.000486, 0.000538, 0.000597
	Hispanic	Women	34	0.000419, 0.000463, 0.000512, 0.000566, 0.000626
	Hispanic	Women	35	0.000441, 0.000488, 0.000541, 0.000598, 0.000663
	Hispanic	Women	36	0.000464, 0.000516, 0.000573, 0.000637, 0.000708
	Hispanic	Women	37	0.000492, 0.000548, 0.000611, 0.00068, 0.000759
	Hispanic	Women	38	0.000526, 0.000587, 0.000654, 0.000728, 0.000812
	Hispanic	Women	39	0.00057, 0.000633, 0.000703, 0.00078, 0.000867
	Hispanic	Women	40	0.000623, 0.000688, 0.000759, 0.000837, 0.000924
	Hispanic	Women	41	0.000687, 0.000752, 0.000823, 0.0009, 0.000986
	Hispanic	Women	42	0.000761, 0.000826, 0.000896, 0.000973, 0.00106
	Hispanic	Women	43	0.000844, 0.00091, 0.000981, 0.00106, 0.00114
	Hispanic	Women	44	0.000936, 0.001, 0.00108, 0.00116, 0.00124
	Hispanic	Women	45	0.00104, 0.00111, 0.00119, 0.00127, 0.00136
	Hispanic	Women	46	0.00115, 0.00123, 0.00131, 0.0014, 0.00149
	Hispanic	Women	47	0.00126, 0.00135, 0.00144, 0.00154, 0.00165
	Hispanic	Women	48	0.00139, 0.00148, 0.00159, 0.00169, 0.00181
	Hispanic	Women	49	0.00152, 0.00163, 0.00174, 0.00186, 0.00199
	Hispanic	Women	50	0.00166, 0.00178, 0.00191, 0.00204, 0.00219

Variable	Race/ethnicity	Sex	Age	Distribution
	Hispanic	Women	51	0.00182, 0.00195, 0.00208, 0.00223, 0.00239
	Hispanic	Women	52	0.00198, 0.00212, 0.00227, 0.00243, 0.0026
	Hispanic	Women	53	0.00217, 0.00232, 0.00247, 0.00264, 0.00283
	Hispanic	Women	54	0.00236, 0.00252, 0.00269, 0.00288, 0.00308
	Hispanic	Women	55	0.00256, 0.00274, 0.00292, 0.00313, 0.00335
	Hispanic	Women	56	0.00278, 0.00297, 0.00317, 0.00339, 0.00363
	Hispanic	Women	57	0.00302, 0.00323, 0.00344, 0.00367, 0.00392
	Hispanic	Women	58	0.00329, 0.00351, 0.00373, 0.00397, 0.00423
	Hispanic	Women	59	0.00358, 0.00381, 0.00405, 0.00431, 0.00458
	Hispanic	Women	60	0.00388, 0.00413, 0.00439, 0.00467, 0.00498
	Hispanic	Women	61	0.0042, 0.00447, 0.00476, 0.00508, 0.00541
	Hispanic	Women	62	0.00454, 0.00485, 0.00517, 0.00551, 0.00588
	Hispanic	Women	63	0.00492, 0.00526, 0.00561, 0.00599, 0.0064
	Hispanic	Women	64	0.00532, 0.0057, 0.0061, 0.00652, 0.00698
	Hispanic	Women	65	0.00577, 0.00618, 0.00663, 0.0071, 0.00762
	Hispanic	Women	66	0.00627, 0.00673, 0.00722, 0.00774, 0.00831
	Hispanic	Women	67	0.00685, 0.00735, 0.00787, 0.00844, 0.00905
	Hispanic	Women	68	0.00752, 0.00805, 0.00861, 0.00922, 0.00987
	Hispanic	Women	69	0.00828, 0.00885, 0.00945, 0.0101, 0.0108
	Hispanic	Women	70	0.00914, 0.00975, 0.0104, 0.0111, 0.0118
	Hispanic	Women	71	0.0101, 0.0107, 0.0114, 0.0122, 0.013
	Hispanic	Women	72	0.0111, 0.0119, 0.0126, 0.0135, 0.0143
	Hispanic	Women	73	0.0123, 0.0131, 0.014, 0.0149, 0.0158
	Hispanic	Women	74	0.0137, 0.0145, 0.0154, 0.0164, 0.0175
	Hispanic	Women	75	0.0152, 0.0162, 0.0171, 0.0182, 0.0193
	Hispanic	Women	76	0.0171, 0.0181, 0.0191, 0.0201, 0.0213
	Hispanic	Women	77	0.0192, 0.0202, 0.0213, 0.0224, 0.0236
	Hispanic	Women	78	0.0216, 0.0227, 0.0239, 0.0251, 0.0264
	Hispanic	Women	79	0.0243, 0.0256, 0.0269, 0.0283, 0.0298
	Hispanic	Women	80	0.0273, 0.0288, 0.0303, 0.0319, 0.0336
	Hispanic	Women	81	0.0305, 0.0323, 0.0341, 0.0361, 0.0381
	Hispanic	Women	82	0.034, 0.0361, 0.0383, 0.0406, 0.0432
	Hispanic	Women	83	0.0375, 0.04, 0.0427, 0.0455, 0.0486
	Hispanic	Women	84	0.041, 0.044, 0.0472, 0.0506, 0.0542
	White	Men	30	0.0013, 0.00145, 0.00161, 0.00179, 0.002
	White	Men	31	0.00135, 0.0015, 0.00166, 0.00184, 0.00204
	White	Men	32	0.0014, 0.00154, 0.0017, 0.00187, 0.00207
	White	Men	33	0.00144, 0.00158, 0.00174, 0.00191, 0.00209
	White	Men	34	0.00148, 0.00162, 0.00177, 0.00194, 0.00213
	White	Men	35	0.00151, 0.00166, 0.00181, 0.00199, 0.00218
	White	Men	36	0.00155, 0.0017, 0.00186, 0.00204, 0.00224
	White	Men	37	0.00159, 0.00175, 0.00192, 0.00211, 0.00231
	White	Men	38	0.00165, 0.00182, 0.00199, 0.00219, 0.00241

Variable	Race/ethnicity	Sex	Age	Distribution
	White	Men	39	0.00173, 0.0019, 0.00208, 0.00229, 0.00252
	White	Men	40	0.00182, 0.002, 0.00219, 0.00241, 0.00264
	White	Men	41	0.00194, 0.00213, 0.00233, 0.00255, 0.00279
	White	Men	42	0.00209, 0.00228, 0.00249, 0.00272, 0.00296
	White	Men	43	0.00228, 0.00247, 0.00268, 0.00291, 0.00316
	White	Men	44	0.0025, 0.0027, 0.00291, 0.00314, 0.00339
	White	Men	45	0.00274, 0.00295, 0.00318, 0.00342, 0.00368
	White	Men	46	0.003, 0.00324, 0.00348, 0.00375, 0.00404
	White	Men	47	0.00328, 0.00355, 0.00383, 0.00414, 0.00447
	White	Men	48	0.00359, 0.00389, 0.00422, 0.00456, 0.00494
	White	Men	49	0.00395, 0.00428, 0.00464, 0.00502, 0.00544
	White	Men	50	0.00436, 0.00471, 0.00509, 0.0055, 0.00595
	White	Men	51	0.00482, 0.00519, 0.00558, 0.006, 0.00646
	White	Men	52	0.00534, 0.00571, 0.0061, 0.00651, 0.00696
	White	Men	53	0.0059, 0.00626, 0.00663, 0.00703, 0.00746
	White	Men	54	0.00646, 0.00682, 0.00719, 0.00758, 0.008
	White	Men	55	0.00699, 0.00737, 0.00776, 0.00817, 0.00861
	White	Men	56	0.0075, 0.00792, 0.00834, 0.0088, 0.00928
	White	Men	57	0.00803, 0.00848, 0.00895, 0.00945, 0.00998
	White	Men	58	0.00859, 0.00908, 0.00959, 0.0101, 0.0107
	White	Men	59	0.00919, 0.00971, 0.0103, 0.0108, 0.0114
	White	Men	60	0.00984, 0.0104, 0.0109, 0.0115, 0.0122
	White	Men	61	0.0106, 0.0111, 0.0117, 0.0123, 0.0129
	White	Men	62	0.0113, 0.0119, 0.0125, 0.0131, 0.0137
	White	Men	63	0.0121, 0.0127, 0.0133, 0.0139, 0.0146
	White	Men	64	0.0129, 0.0135, 0.0142, 0.0148, 0.0155
	White	Men	65	0.0138, 0.0145, 0.0151, 0.0159, 0.0166
	White	Men	66	0.0147, 0.0155, 0.0162, 0.017, 0.0179
	White	Men	67	0.0158, 0.0166, 0.0175, 0.0184, 0.0193
	White	Men	68	0.0171, 0.018, 0.0189, 0.0199, 0.021
	White	Men	69	0.0185, 0.0195, 0.0206, 0.0217, 0.0229
	White	Men	70	0.0201, 0.0213, 0.0225, 0.0237, 0.0251
	White	Men	71	0.022, 0.0232, 0.0246, 0.0259, 0.0274
	White	Men	72	0.0241, 0.0254, 0.0269, 0.0284, 0.03
	White	Men	73	0.0264, 0.0278, 0.0294, 0.031, 0.0327
	White	Men	74	0.0289, 0.0305, 0.0321, 0.0339, 0.0357
	White	Men	75	0.0318, 0.0334, 0.0352, 0.037, 0.0389
	White	Men	76	0.035, 0.0368, 0.0386, 0.0405, 0.0426
	White	Men	77	0.0386, 0.0405, 0.0425, 0.0446, 0.0468
	White	Men	78	0.0428, 0.0449, 0.0471, 0.0493, 0.0518
	White	Men	79	0.0475, 0.0498, 0.0523, 0.0549, 0.0577
	White	Men	80	0.0528, 0.0555, 0.0584, 0.0614, 0.0645
	White	Men	81	0.0588, 0.0619, 0.0652, 0.0686, 0.0723

Variable	Race/ethnicity	Sex	Age	Distribution
	White	Men	82	0.0651, 0.0687, 0.0725, 0.0765, 0.0808
	White	Men	83	0.0716, 0.0758, 0.0802, 0.0849, 0.0899
	White	Men	84	0.0781, 0.0829, 0.0879, 0.0933, 0.0991
	White	Women	30	0.000637, 0.000695, 0.000759, 0.000828, 0.000904
	White	Women	31	0.000671, 0.000734, 0.000803, 0.000879, 0.000962
	White	Women	32	0.00071, 0.000777, 0.000849, 0.000928, 0.00102
	White	Women	33	0.000753, 0.000822, 0.000896, 0.000976, 0.00107
	White	Women	34	0.000795, 0.000867, 0.000945, 0.00103, 0.00112
	White	Women	35	0.000834, 0.000913, 0.000998, 0.00109, 0.00119
	White	Women	36	0.000876, 0.000963, 0.00106, 0.00116, 0.00128
	White	Women	37	0.000925, 0.00102, 0.00112, 0.00124, 0.00137
	White	Women	38	0.00098, 0.00109, 0.0012, 0.00133, 0.00147
	White	Women	39	0.00104, 0.00116, 0.00129, 0.00143, 0.00158
	White	Women	40	0.00112, 0.00124, 0.00138, 0.00154, 0.00171
	White	Women	41	0.00121, 0.00134, 0.00149, 0.00166, 0.00184
	White	Women	42	0.00131, 0.00145, 0.00161, 0.00179, 0.00199
	White	Women	43	0.00143, 0.00159, 0.00176, 0.00194, 0.00215
	White	Women	44	0.00157, 0.00173, 0.00192, 0.00212, 0.00235
	White	Women	45	0.00172, 0.0019, 0.0021, 0.00232, 0.00257
	White	Women	46	0.00188, 0.00208, 0.0023, 0.00254, 0.00281
	White	Women	47	0.00208, 0.00229, 0.00252, 0.00278, 0.00306
	White	Women	48	0.0023, 0.00252, 0.00276, 0.00302, 0.00331
	White	Women	49	0.00254, 0.00277, 0.00301, 0.00328, 0.00357
	White	Women	50	0.00278, 0.00302, 0.00328, 0.00356, 0.00387
	White	Women	51	0.00301, 0.00328, 0.00356, 0.00387, 0.00421
	White	Women	52	0.00324, 0.00354, 0.00385, 0.0042, 0.00458
	White	Women	53	0.00348, 0.0038, 0.00415, 0.00453, 0.00496
	White	Women	54	0.00373, 0.00408, 0.00445, 0.00486, 0.00531
	White	Women	55	0.00402, 0.00437, 0.00475, 0.00516, 0.00562
	White	Women	56	0.00436, 0.00469, 0.00505, 0.00544, 0.00586
	White	Women	57	0.00474, 0.00504, 0.00535, 0.00569, 0.00606
	White	Women	58	0.00513, 0.0054, 0.00567, 0.00596, 0.00627
	White	Women	59	0.0055, 0.00576, 0.00602, 0.0063, 0.00659
	White	Women	60	0.00585, 0.00613, 0.00641, 0.00671, 0.00703
	White	Women	61	0.00623, 0.00654, 0.00687, 0.00721, 0.00757
	White	Women	62	0.00666, 0.00702, 0.0074, 0.00779, 0.00821
	White	Women	63	0.00718, 0.00759, 0.00801, 0.00846, 0.00893
	White	Women	64	0.00779, 0.00824, 0.00872, 0.00922, 0.00975
	White	Women	65	0.0085, 0.009, 0.00952, 0.0101, 0.0107
	White	Women	66	0.00929, 0.00985, 0.0104, 0.0111, 0.0117
	White	Women	67	0.0102, 0.0108, 0.0115, 0.0122, 0.0129
	White	Women	68	0.0111, 0.0119, 0.0126, 0.0134, 0.0143
	White	Women	69	0.0122, 0.013, 0.0139, 0.0148, 0.0158

Variable	Race/ethnicity	Sex	Age	Distribution
	White	Women	70	0.0133, 0.0143, 0.0153, 0.0163, 0.0175
	White	Women	71	0.0146, 0.0157, 0.0168, 0.018, 0.0194
	White	Women	72	0.016, 0.0172, 0.0185, 0.0199, 0.0214
	White	Women	73	0.0177, 0.019, 0.0204, 0.0219, 0.0235
	White	Women	74	0.0196, 0.021, 0.0225, 0.0241, 0.0258
	White	Women	75	0.0218, 0.0232, 0.0248, 0.0264, 0.0282
	White	Women	76	0.0243, 0.0258, 0.0274, 0.0291, 0.0309
	White	Women	77	0.0273, 0.0288, 0.0304, 0.0322, 0.034
	White	Women	78	0.0306, 0.0322, 0.034, 0.0358, 0.0377
	White	Women	79	0.0343, 0.0361, 0.038, 0.04, 0.0421
	White	Women	80	0.0384, 0.0405, 0.0426, 0.0448, 0.0472
	White	Women	81	0.043, 0.0453, 0.0477, 0.0503, 0.053
	White	Women	82	0.0479, 0.0506, 0.0534, 0.0563, 0.0595
	White	Women	83	0.0529, 0.0561, 0.0593, 0.0628, 0.0665
	White	Women	84	0.0581, 0.0617, 0.0655, 0.0695, 0.0738
Total mortality rates for 2018 (0.01, 0.2, 0.5, 0.8. 0.99 percentiles of the empirical distribution produced during forecasting)				
	Black	Men	30	0.00183, 0.00205, 0.00231, 0.00259, 0.00292
	Black	Men	31	0.00189, 0.00211, 0.00235, 0.00261, 0.00291
	Black	Men	32	0.00195, 0.00216, 0.00239, 0.00264, 0.00293
	Black	Men	33	0.002, 0.00221, 0.00244, 0.0027, 0.00299
	Black	Men	34	0.00205, 0.00227, 0.00251, 0.00278, 0.00308
	Black	Men	35	0.00212, 0.00235, 0.0026, 0.00288, 0.00319
	Black	Men	36	0.0022, 0.00244, 0.0027, 0.00299, 0.00331
	Black	Men	37	0.00229, 0.00254, 0.0028, 0.0031, 0.00343
	Black	Men	38	0.00238, 0.00264, 0.00291, 0.00322, 0.00356
	Black	Men	39	0.00246, 0.00273, 0.00303, 0.00335, 0.00372
	Black	Men	40	0.00254, 0.00283, 0.00316, 0.00352, 0.00393
	Black	Men	41	0.00262, 0.00295, 0.00331, 0.00372, 0.00418
	Black	Men	42	0.00274, 0.0031, 0.00351, 0.00397, 0.00449
	Black	Men	43	0.00289, 0.00329, 0.00375, 0.00426, 0.00486
	Black	Men	44	0.00307, 0.00352, 0.00403, 0.00462, 0.00529
	Black	Men	45	0.00329, 0.00379, 0.00437, 0.00502, 0.00579
	Black	Men	46	0.00357, 0.00412, 0.00475, 0.00547, 0.00631
	Black	Men	47	0.00392, 0.00451, 0.00518, 0.00596, 0.00686
	Black	Men	48	0.00435, 0.00497, 0.00568, 0.00648, 0.00741
	Black	Men	49	0.00485, 0.0055, 0.00623, 0.00706, 0.008
	Black	Men	50	0.00543, 0.00611, 0.00685, 0.0077, 0.00865
	Black	Men	51	0.00608, 0.00678, 0.00755, 0.0084, 0.00937
	Black	Men	52	0.00682, 0.00753, 0.00831, 0.00917, 0.0101
	Black	Men	53	0.00764, 0.00836, 0.00914, 0.00999, 0.0109
	Black	Men	54	0.00853, 0.00926, 0.01, 0.0109, 0.0118
	Black	Men	55	0.00948, 0.0102, 0.011, 0.0118, 0.0128
	Black	Men	56	0.0105, 0.0112, 0.012, 0.0129, 0.0138

Variable	Race/ethnicity	Sex	Age	Distribution
	Black	Men	57	0.0116, 0.0124, 0.0132, 0.014, 0.0149
	Black	Men	58	0.0128, 0.0136, 0.0144, 0.0152, 0.0161
	Black	Men	59	0.0141, 0.0148, 0.0156, 0.0165, 0.0174
	Black	Men	60	0.0153, 0.0161, 0.017, 0.0179, 0.0188
	Black	Men	61	0.0165, 0.0174, 0.0183, 0.0193, 0.0203
	Black	Men	62	0.0177, 0.0187, 0.0196, 0.0206, 0.0217
	Black	Men	63	0.0189, 0.0199, 0.0209, 0.022, 0.0231
	Black	Men	64	0.0199, 0.021, 0.0221, 0.0233, 0.0245
	Black	Men	65	0.021, 0.0222, 0.0234, 0.0246, 0.026
	Black	Men	66	0.0221, 0.0234, 0.0247, 0.026, 0.0274
	Black	Men	67	0.0233, 0.0247, 0.0261, 0.0275, 0.0291
	Black	Men	68	0.0247, 0.0261, 0.0277, 0.0293, 0.031
	Black	Men	69	0.0262, 0.0278, 0.0295, 0.0313, 0.0332
	Black	Men	70	0.0278, 0.0297, 0.0316, 0.0336, 0.0358
	Black	Men	71	0.0298, 0.0318, 0.0339, 0.0362, 0.0386
	Black	Men	72	0.0319, 0.0341, 0.0365, 0.039, 0.0417
	Black	Men	73	0.0343, 0.0367, 0.0392, 0.0419, 0.0449
	Black	Men	74	0.0371, 0.0396, 0.0422, 0.045, 0.0481
	Black	Men	75	0.0402, 0.0427, 0.0454, 0.0483, 0.0513
	Black	Men	76	0.0436, 0.0462, 0.0489, 0.0517, 0.0548
	Black	Men	77	0.0473, 0.05, 0.0527, 0.0556, 0.0587
	Black	Men	78	0.0512, 0.054, 0.0569, 0.06, 0.0634
	Black	Men	79	0.0552, 0.0583, 0.0616, 0.0651, 0.0688
	Black	Men	80	0.0596, 0.0631, 0.0668, 0.0707, 0.0748
	Black	Men	81	0.0643, 0.0682, 0.0723, 0.0767, 0.0814
	Black	Men	82	0.0691, 0.0735, 0.0781, 0.0831, 0.0884
	Black	Men	83	0.0737, 0.0787, 0.084, 0.0896, 0.0956
	Black	Men	84	0.0782, 0.0838, 0.0896, 0.0959, 0.103
	Black	Women	30	0.000704, 0.000803, 0.000916, 0.00104, 0.00119
	Black	Women	31	0.000773, 0.000873, 0.000986, 0.00111, 0.00126
	Black	Women	32	0.000853, 0.000954, 0.00107, 0.00119, 0.00133
	Black	Women	33	0.000939, 0.00104, 0.00115, 0.00127, 0.00141
	Black	Women	34	0.00102, 0.00112, 0.00124, 0.00137, 0.00151
	Black	Women	35	0.00109, 0.0012, 0.00133, 0.00146, 0.00162
	Black	Women	36	0.00116, 0.00128, 0.00142, 0.00157, 0.00173
	Black	Women	37	0.00123, 0.00137, 0.00151, 0.00167, 0.00186
	Black	Women	38	0.00132, 0.00146, 0.00162, 0.00179, 0.00198
	Black	Women	39	0.00142, 0.00157, 0.00174, 0.00192, 0.00213
	Black	Women	40	0.00153, 0.0017, 0.00187, 0.00207, 0.00229
	Black	Women	41	0.00167, 0.00184, 0.00203, 0.00224, 0.00247
	Black	Women	42	0.00184, 0.00202, 0.00221, 0.00242, 0.00265
	Black	Women	43	0.00205, 0.00222, 0.00241, 0.00262, 0.00284
	Black	Women	44	0.00228, 0.00245, 0.00263, 0.00283, 0.00305

Variable	Race/ethnicity	Sex	Age	Distribution
	Black	Women	45	0.00252, 0.00269, 0.00288, 0.00308, 0.0033
	Black	Women	46	0.00276, 0.00295, 0.00316, 0.00338, 0.00361
	Black	Women	47	0.003, 0.00322, 0.00346, 0.00371, 0.00399
	Black	Women	48	0.00327, 0.00353, 0.0038, 0.00409, 0.0044
	Black	Women	49	0.00359, 0.00387, 0.00416, 0.00449, 0.00483
	Black	Women	50	0.00396, 0.00425, 0.00456, 0.0049, 0.00526
	Black	Women	51	0.0044, 0.00469, 0.00499, 0.00531, 0.00566
	Black	Women	52	0.0049, 0.00517, 0.00545, 0.00574, 0.00605
	Black	Women	53	0.00542, 0.00567, 0.00593, 0.00621, 0.0065
	Black	Women	54	0.00591, 0.00617, 0.00645, 0.00674, 0.00704
	Black	Women	55	0.00638, 0.00668, 0.00699, 0.00732, 0.00767
	Black	Women	56	0.00686, 0.00721, 0.00756, 0.00793, 0.00833
	Black	Women	57	0.00738, 0.00776, 0.00815, 0.00856, 0.00899
	Black	Women	58	0.00792, 0.00832, 0.00874, 0.00918, 0.00965
	Black	Women	59	0.00844, 0.00888, 0.00934, 0.00982, 0.0103
	Black	Women	60	0.00897, 0.00944, 0.00994, 0.0105, 0.011
	Black	Women	61	0.00951, 0.01, 0.0105, 0.0111, 0.0117
	Black	Women	62	0.0101, 0.0106, 0.0112, 0.0118, 0.0124
	Black	Women	63	0.0106, 0.0112, 0.0118, 0.0125, 0.0132
	Black	Women	64	0.0113, 0.0119, 0.0126, 0.0133, 0.0141
	Black	Women	65	0.012, 0.0127, 0.0134, 0.0142, 0.0151
	Black	Women	66	0.0128, 0.0136, 0.0144, 0.0153, 0.0162
	Black	Women	67	0.0137, 0.0146, 0.0155, 0.0164, 0.0174
	Black	Women	68	0.0148, 0.0157, 0.0167, 0.0177, 0.0188
	Black	Women	69	0.016, 0.017, 0.0181, 0.0192, 0.0204
	Black	Women	70	0.0173, 0.0184, 0.0195, 0.0208, 0.0221
	Black	Women	71	0.0187, 0.0199, 0.0212, 0.0225, 0.024
	Black	Women	72	0.0202, 0.0215, 0.0229, 0.0244, 0.0259
	Black	Women	73	0.0219, 0.0233, 0.0248, 0.0264, 0.0281
	Black	Women	74	0.0238, 0.0253, 0.0269, 0.0285, 0.0303
	Black	Women	75	0.0259, 0.0275, 0.0292, 0.0309, 0.0328
	Black	Women	76	0.0283, 0.03, 0.0317, 0.0336, 0.0356
	Black	Women	77	0.031, 0.0328, 0.0347, 0.0367, 0.0388
	Black	Women	78	0.034, 0.036, 0.038, 0.0401, 0.0424
	Black	Women	79	0.0373, 0.0395, 0.0417, 0.0441, 0.0467
	Black	Women	80	0.0409, 0.0433, 0.0459, 0.0486, 0.0515
	Black	Women	81	0.0446, 0.0474, 0.0504, 0.0535, 0.0569
	Black	Women	82	0.0484, 0.0517, 0.0551, 0.0588, 0.0628
	Black	Women	83	0.0522, 0.056, 0.0601, 0.0644, 0.0692
	Black	Women	84	0.0559, 0.0603, 0.065, 0.0701, 0.0757
	Hispanic	Men	30	0.000814, 0.000902, 0.001, 0.00111, 0.00123
	Hispanic	Men	31	0.000829, 0.000919, 0.00102, 0.00113, 0.00125
	Hispanic	Men	32	0.000845, 0.000937, 0.00104, 0.00115, 0.00127

Variable	Race/ethnicity	Sex	Age	Distribution
	Hispanic	Men	33	0.000854, 0.00095, 0.00106, 0.00118, 0.00131
	Hispanic	Men	34	0.000858, 0.000963, 0.00108, 0.00121, 0.00136
	Hispanic	Men	35	0.000869, 0.000981, 0.00111, 0.00125, 0.00141
	Hispanic	Men	36	0.000894, 0.00101, 0.00114, 0.00129, 0.00146
	Hispanic	Men	37	0.000936, 0.00105, 0.00119, 0.00134, 0.00151
	Hispanic	Men	38	0.00099, 0.00111, 0.00124, 0.00139, 0.00156
	Hispanic	Men	39	0.00105, 0.00118, 0.00131, 0.00147, 0.00164
	Hispanic	Men	40	0.00112, 0.00125, 0.0014, 0.00156, 0.00174
	Hispanic	Men	41	0.0012, 0.00134, 0.00149, 0.00166, 0.00185
	Hispanic	Men	42	0.00131, 0.00145, 0.00161, 0.00179, 0.00198
	Hispanic	Men	43	0.00145, 0.00159, 0.00175, 0.00192, 0.00212
	Hispanic	Men	44	0.00162, 0.00176, 0.00192, 0.00208, 0.00226
	Hispanic	Men	45	0.00182, 0.00196, 0.00211, 0.00227, 0.00245
	Hispanic	Men	46	0.00203, 0.00218, 0.00234, 0.00251, 0.0027
	Hispanic	Men	47	0.00224, 0.00241, 0.00259, 0.00279, 0.003
	Hispanic	Men	48	0.00247, 0.00266, 0.00287, 0.0031, 0.00335
	Hispanic	Men	49	0.00272, 0.00294, 0.00317, 0.00343, 0.00371
	Hispanic	Men	50	0.003, 0.00324, 0.00349, 0.00377, 0.00407
	Hispanic	Men	51	0.00334, 0.00358, 0.00383, 0.0041, 0.00439
	Hispanic	Men	52	0.00375, 0.00396, 0.00418, 0.00441, 0.00466
	Hispanic	Men	53	0.00422, 0.00438, 0.00455, 0.00473, 0.00492
	Hispanic	Men	54	0.00465, 0.0048, 0.00495, 0.0051, 0.00526
	Hispanic	Men	55	0.00499, 0.00518, 0.00537, 0.00556, 0.00576
	Hispanic	Men	56	0.00532, 0.00556, 0.00581, 0.00607, 0.00634
	Hispanic	Men	57	0.00569, 0.00598, 0.00628, 0.0066, 0.00694
	Hispanic	Men	58	0.0061, 0.00644, 0.00679, 0.00716, 0.00755
	Hispanic	Men	59	0.00654, 0.00692, 0.00732, 0.00774, 0.0082
	Hispanic	Men	60	0.00701, 0.00744, 0.00788, 0.00835, 0.00886
	Hispanic	Men	61	0.00754, 0.00799, 0.00846, 0.00897, 0.00951
	Hispanic	Men	62	0.00811, 0.00858, 0.00907, 0.00959, 0.0101
	Hispanic	Men	63	0.00872, 0.0092, 0.00971, 0.0102, 0.0108
	Hispanic	Men	64	0.00933, 0.00984, 0.0104, 0.0109, 0.0115
	Hispanic	Men	65	0.00994, 0.0105, 0.0111, 0.0117, 0.0124
	Hispanic	Men	66	0.0106, 0.0112, 0.0119, 0.0126, 0.0134
	Hispanic	Men	67	0.0113, 0.012, 0.0128, 0.0136, 0.0145
	Hispanic	Men	68	0.0121, 0.013, 0.0138, 0.0148, 0.0158
	Hispanic	Men	69	0.0131, 0.014, 0.015, 0.0161, 0.0172
	Hispanic	Men	70	0.0142, 0.0152, 0.0163, 0.0175, 0.0187
	Hispanic	Men	71	0.0155, 0.0166, 0.0178, 0.0191, 0.0204
	Hispanic	Men	72	0.0169, 0.0181, 0.0194, 0.0208, 0.0223
	Hispanic	Men	73	0.0184, 0.0198, 0.0212, 0.0227, 0.0243
	Hispanic	Men	74	0.0202, 0.0216, 0.0231, 0.0248, 0.0265
	Hispanic	Men	75	0.0222, 0.0237, 0.0253, 0.0271, 0.0289

Variable	Race/ethnicity	Sex	Age	Distribution
	Hispanic	Men	76	0.0244, 0.0261, 0.0278, 0.0296, 0.0316
	Hispanic	Men	77	0.027, 0.0288, 0.0306, 0.0326, 0.0348
	Hispanic	Men	78	0.0299, 0.0319, 0.0339, 0.0362, 0.0385
	Hispanic	Men	79	0.0332, 0.0354, 0.0377, 0.0402, 0.0429
	Hispanic	Men	80	0.037, 0.0395, 0.0421, 0.0448, 0.0478
	Hispanic	Men	81	0.0413, 0.044, 0.0469, 0.0499, 0.0533
	Hispanic	Men	82	0.0457, 0.0488, 0.052, 0.0555, 0.0593
	Hispanic	Men	83	0.0501, 0.0536, 0.0573, 0.0613, 0.0657
	Hispanic	Men	84	0.0543, 0.0583, 0.0626, 0.0672, 0.0722
	Hispanic	Women	30	0.00033, 0.000371, 0.000417, 0.000468, 0.000527
	Hispanic	Women	31	0.000348, 0.000391, 0.000438, 0.000491, 0.000552
	Hispanic	Women	32	0.000369, 0.000413, 0.000461, 0.000515, 0.000575
	Hispanic	Women	33	0.000393, 0.000437, 0.000485, 0.000539, 0.000599
	Hispanic	Women	34	0.000415, 0.000461, 0.000511, 0.000566, 0.000628
	Hispanic	Women	35	0.000436, 0.000485, 0.000539, 0.000598, 0.000666
	Hispanic	Women	36	0.000457, 0.000511, 0.00057, 0.000636, 0.000711
	Hispanic	Women	37	0.000483, 0.000542, 0.000606, 0.000679, 0.000761
	Hispanic	Women	38	0.000516, 0.000579, 0.000648, 0.000725, 0.000813
	Hispanic	Women	39	0.000558, 0.000623, 0.000695, 0.000776, 0.000866
	Hispanic	Women	40	0.000609, 0.000676, 0.000749, 0.000831, 0.000922
	Hispanic	Women	41	0.00067, 0.000738, 0.000811, 0.000892, 0.000982
	Hispanic	Women	42	0.000741, 0.000809, 0.000883, 0.000963, 0.00105
	Hispanic	Women	43	0.000822, 0.000891, 0.000965, 0.00105, 0.00113
	Hispanic	Women	44	0.000913, 0.000984, 0.00106, 0.00114, 0.00123
	Hispanic	Women	45	0.00101, 0.00109, 0.00117, 0.00125, 0.00135
	Hispanic	Women	46	0.00112, 0.0012, 0.00129, 0.00138, 0.00148
	Hispanic	Women	47	0.00123, 0.00132, 0.00142, 0.00152, 0.00163
	Hispanic	Women	48	0.00136, 0.00146, 0.00156, 0.00168, 0.0018
	Hispanic	Women	49	0.00149, 0.0016, 0.00172, 0.00185, 0.00198
	Hispanic	Women	50	0.00163, 0.00175, 0.00188, 0.00203, 0.00218
	Hispanic	Women	51	0.00179, 0.00192, 0.00206, 0.00222, 0.00238
	Hispanic	Women	52	0.00196, 0.0021, 0.00225, 0.00241, 0.00259
	Hispanic	Women	53	0.00214, 0.0023, 0.00246, 0.00263, 0.00281
	Hispanic	Women	54	0.00233, 0.0025, 0.00267, 0.00286, 0.00306
	Hispanic	Women	55	0.00253, 0.00271, 0.00291, 0.00311, 0.00333
	Hispanic	Women	56	0.00275, 0.00295, 0.00315, 0.00338, 0.00362
	Hispanic	Women	57	0.00299, 0.0032, 0.00342, 0.00366, 0.00391
	Hispanic	Women	58	0.00325, 0.00347, 0.00371, 0.00396, 0.00423
	Hispanic	Women	59	0.00353, 0.00377, 0.00402, 0.00429, 0.00458
	Hispanic	Women	60	0.00382, 0.00408, 0.00436, 0.00465, 0.00497
	Hispanic	Women	61	0.00413, 0.00442, 0.00472, 0.00505, 0.0054
	Hispanic	Women	62	0.00447, 0.00478, 0.00512, 0.00548, 0.00586
	Hispanic	Women	63	0.00483, 0.00518, 0.00555, 0.00594, 0.00637

Variable	Race/ethnicity	Sex	Age	Distribution
	Hispanic	Women	64	0.00523, 0.00561, 0.00602, 0.00646, 0.00694
	Hispanic	Women	65	0.00565, 0.00608, 0.00654, 0.00703, 0.00756
	Hispanic	Women	66	0.00614, 0.00661, 0.00712, 0.00766, 0.00825
	Hispanic	Women	67	0.0067, 0.00721, 0.00776, 0.00835, 0.00899
	Hispanic	Women	68	0.00735, 0.0079, 0.00849, 0.00912, 0.0098
	Hispanic	Women	69	0.00809, 0.00868, 0.00931, 0.00998, 0.0107
	Hispanic	Women	70	0.00891, 0.00955, 0.0102, 0.011, 0.0117
	Hispanic	Women	71	0.00983, 0.0105, 0.0113, 0.0121, 0.0129
	Hispanic	Women	72	0.0109, 0.0116, 0.0124, 0.0133, 0.0142
	Hispanic	Women	73	0.012, 0.0128, 0.0137, 0.0147, 0.0157
	Hispanic	Women	74	0.0133, 0.0142, 0.0152, 0.0162, 0.0173
	Hispanic	Women	75	0.0149, 0.0158, 0.0169, 0.0179, 0.0191
	Hispanic	Women	76	0.0167, 0.0177, 0.0188, 0.0199, 0.0211
	Hispanic	Women	77	0.0187, 0.0198, 0.021, 0.0222, 0.0234
	Hispanic	Women	78	0.0211, 0.0223, 0.0235, 0.0248, 0.0262
	Hispanic	Women	79	0.0237, 0.0251, 0.0265, 0.028, 0.0295
	Hispanic	Women	80	0.0267, 0.0282, 0.0298, 0.0316, 0.0334
	Hispanic	Women	81	0.0298, 0.0317, 0.0336, 0.0357, 0.0379
	Hispanic	Women	82	0.0332, 0.0354, 0.0377, 0.0402, 0.0429
	Hispanic	Women	83	0.0366, 0.0392, 0.0421, 0.0451, 0.0483
	Hispanic	Women	84	0.0401, 0.0432, 0.0465, 0.05, 0.0539
	White	Men	30	0.00127, 0.00143, 0.00161, 0.00182, 0.00205
	White	Men	31	0.00132, 0.00148, 0.00166, 0.00186, 0.00208
	White	Men	32	0.00137, 0.00152, 0.0017, 0.00189, 0.00211
	White	Men	33	0.00141, 0.00156, 0.00173, 0.00192, 0.00213
	White	Men	34	0.00144, 0.0016, 0.00177, 0.00196, 0.00217
	White	Men	35	0.00147, 0.00163, 0.0018, 0.002, 0.00221
	White	Men	36	0.0015, 0.00167, 0.00185, 0.00205, 0.00227
	White	Men	37	0.00155, 0.00172, 0.00191, 0.00212, 0.00235
	White	Men	38	0.0016, 0.00178, 0.00198, 0.00219, 0.00244
	White	Men	39	0.00167, 0.00186, 0.00206, 0.00229, 0.00254
	White	Men	40	0.00176, 0.00196, 0.00217, 0.0024, 0.00267
	White	Men	41	0.00188, 0.00208, 0.0023, 0.00254, 0.00281
	White	Men	42	0.00202, 0.00223, 0.00245, 0.0027, 0.00298
	White	Men	43	0.0022, 0.00241, 0.00264, 0.00289, 0.00317
	White	Men	44	0.00241, 0.00263, 0.00287, 0.00312, 0.0034
	White	Men	45	0.00265, 0.00288, 0.00313, 0.0034, 0.0037
	White	Men	46	0.0029, 0.00316, 0.00344, 0.00374, 0.00407
	White	Men	47	0.00317, 0.00347, 0.00378, 0.00413, 0.00451
	White	Men	48	0.00348, 0.00381, 0.00417, 0.00456, 0.005
	White	Men	49	0.00383, 0.00419, 0.00459, 0.00503, 0.00551
	White	Men	50	0.00423, 0.00462, 0.00505, 0.00552, 0.00603
	White	Men	51	0.00469, 0.0051, 0.00554, 0.00602, 0.00655

Variable	Race/ethnicity	Sex	Age	Distribution
	White	Men	52	0.0052, 0.00561, 0.00605, 0.00653, 0.00704
	White	Men	53	0.00576, 0.00616, 0.00659, 0.00705, 0.00754
	White	Men	54	0.00631, 0.00672, 0.00714, 0.00759, 0.00808
	White	Men	55	0.00684, 0.00726, 0.00771, 0.00818, 0.00869
	White	Men	56	0.00734, 0.0078, 0.00829, 0.00881, 0.00937
	White	Men	57	0.00785, 0.00836, 0.00889, 0.00947, 0.0101
	White	Men	58	0.00839, 0.00894, 0.00952, 0.0101, 0.0108
	White	Men	59	0.00897, 0.00955, 0.0102, 0.0108, 0.0115
	White	Men	60	0.00961, 0.0102, 0.0108, 0.0115, 0.0122
	White	Men	61	0.0103, 0.0109, 0.0116, 0.0122, 0.013
	White	Men	62	0.011, 0.0117, 0.0123, 0.013, 0.0137
	White	Men	63	0.0118, 0.0124, 0.0131, 0.0138, 0.0145
	White	Men	64	0.0126, 0.0133, 0.014, 0.0147, 0.0155
	White	Men	65	0.0134, 0.0142, 0.0149, 0.0157, 0.0165
	White	Men	66	0.0144, 0.0152, 0.016, 0.0169, 0.0178
	White	Men	67	0.0154, 0.0163, 0.0172, 0.0182, 0.0192
	White	Men	68	0.0166, 0.0176, 0.0186, 0.0197, 0.0209
	White	Men	69	0.018, 0.0191, 0.0203, 0.0215, 0.0228
	White	Men	70	0.0196, 0.0208, 0.0221, 0.0235, 0.025
	White	Men	71	0.0214, 0.0228, 0.0242, 0.0257, 0.0273
	White	Men	72	0.0234, 0.0249, 0.0264, 0.0281, 0.0298
	White	Men	73	0.0257, 0.0272, 0.0289, 0.0307, 0.0326
	White	Men	74	0.0281, 0.0298, 0.0316, 0.0335, 0.0355
	White	Men	75	0.0309, 0.0327, 0.0346, 0.0366, 0.0387
	White	Men	76	0.0341, 0.036, 0.038, 0.04, 0.0423
	White	Men	77	0.0376, 0.0397, 0.0418, 0.0441, 0.0464
	White	Men	78	0.0417, 0.0439, 0.0463, 0.0488, 0.0514
	White	Men	79	0.0463, 0.0488, 0.0515, 0.0543, 0.0573
	White	Men	80	0.0515, 0.0544, 0.0575, 0.0607, 0.0641
	White	Men	81	0.0573, 0.0606, 0.0642, 0.0679, 0.0718
	White	Men	82	0.0635, 0.0674, 0.0714, 0.0757, 0.0804
	White	Men	83	0.0698, 0.0743, 0.079, 0.084, 0.0894
	White	Men	84	0.0761, 0.0812, 0.0867, 0.0924, 0.0986
	White	Women	30	0.000627, 0.000689, 0.000757, 0.000832, 0.000915
	White	Women	31	0.00066, 0.000728, 0.000802, 0.000884, 0.000974
	White	Women	32	0.000698, 0.00077, 0.000847, 0.000933, 0.00103
	White	Women	33	0.00074, 0.000813, 0.000893, 0.000981, 0.00108
	White	Women	34	0.000778, 0.000856, 0.000941, 0.00103, 0.00114
	White	Women	35	0.000813, 0.000899, 0.000993, 0.0011, 0.00121
	White	Women	36	0.00085, 0.000945, 0.00105, 0.00117, 0.0013
	White	Women	37	0.000894, 0.000999, 0.00111, 0.00124, 0.00139
	White	Women	38	0.000944, 0.00106, 0.00119, 0.00133, 0.00149
	White	Women	39	0.001, 0.00113, 0.00127, 0.00143, 0.00161

Variable	Race/ethnicity	Sex	Age	Distribution
	White	Women	40	0.00107, 0.00121, 0.00136, 0.00153, 0.00173
	White	Women	41	0.00115, 0.0013, 0.00147, 0.00165, 0.00187
	White	Women	42	0.00125, 0.00141, 0.00159, 0.00179, 0.00201
	White	Women	43	0.00136, 0.00153, 0.00172, 0.00194, 0.00218
	White	Women	44	0.00149, 0.00168, 0.00188, 0.00211, 0.00238
	White	Women	45	0.00163, 0.00184, 0.00206, 0.00231, 0.0026
	White	Women	46	0.00179, 0.00202, 0.00226, 0.00254, 0.00285
	White	Women	47	0.00198, 0.00222, 0.00248, 0.00277, 0.0031
	White	Women	48	0.0022, 0.00244, 0.00272, 0.00302, 0.00335
	White	Women	49	0.00244, 0.00269, 0.00297, 0.00327, 0.00362
	White	Women	50	0.00267, 0.00294, 0.00324, 0.00356, 0.00392
	White	Women	51	0.0029, 0.00319, 0.00352, 0.00388, 0.00427
	White	Women	52	0.00311, 0.00345, 0.00381, 0.00421, 0.00466
	White	Women	53	0.00334, 0.00371, 0.00411, 0.00456, 0.00506
	White	Women	54	0.00359, 0.00398, 0.00441, 0.00488, 0.00541
	White	Women	55	0.00389, 0.00428, 0.00471, 0.00518, 0.0057
	White	Women	56	0.00424, 0.00461, 0.00501, 0.00544, 0.00591
	White	Women	57	0.00463, 0.00496, 0.00531, 0.00568, 0.00608
	White	Women	58	0.00503, 0.00532, 0.00562, 0.00594, 0.00628
	White	Women	59	0.0054, 0.00568, 0.00596, 0.00626, 0.00658
	White	Women	60	0.00574, 0.00604, 0.00635, 0.00668, 0.00702
	White	Women	61	0.00611, 0.00645, 0.0068, 0.00716, 0.00756
	White	Women	62	0.00654, 0.00692, 0.00731, 0.00773, 0.00818
	White	Women	63	0.00704, 0.00747, 0.00791, 0.00838, 0.00889
	White	Women	64	0.00764, 0.00811, 0.0086, 0.00913, 0.00969
	White	Women	65	0.00832, 0.00884, 0.00939, 0.00998, 0.0106
	White	Women	66	0.00908, 0.00967, 0.0103, 0.0109, 0.0116
	White	Women	67	0.00993, 0.0106, 0.0113, 0.012, 0.0128
	White	Women	68	0.0109, 0.0116, 0.0124, 0.0133, 0.0142
	White	Women	69	0.0119, 0.0127, 0.0137, 0.0146, 0.0157
	White	Women	70	0.013, 0.014, 0.015, 0.0162, 0.0174
	White	Women	71	0.0142, 0.0153, 0.0165, 0.0179, 0.0193
	White	Women	72	0.0156, 0.0168, 0.0182, 0.0197, 0.0213
	White	Women	73	0.0172, 0.0185, 0.02, 0.0216, 0.0234
	White	Women	74	0.019, 0.0205, 0.0221, 0.0238, 0.0257
	White	Women	75	0.0211, 0.0227, 0.0244, 0.0261, 0.0281
	White	Women	76	0.0236, 0.0252, 0.0269, 0.0288, 0.0307
	White	Women	77	0.0265, 0.0282, 0.0299, 0.0318, 0.0338
	White	Women	78	0.0297, 0.0315, 0.0334, 0.0354, 0.0375
	White	Women	79	0.0333, 0.0353, 0.0374, 0.0396, 0.0419
	White	Women	80	0.0374, 0.0396, 0.0419, 0.0444, 0.047
	White	Women	81	0.0418, 0.0444, 0.047, 0.0498, 0.0528
	White	Women	82	0.0466, 0.0495, 0.0526, 0.0558, 0.0593

Variable	Race/ethnicity	Sex	Age	Distribution
	White	Women	83	0.0515, 0.0549, 0.0584, 0.0622, 0.0663
	White	Women	84	0.0566, 0.0604, 0.0645, 0.0689, 0.0736
Total mortality rates for 2019 (0.01, 0.2, 0.5, 0.8. 0.99 percentiles of the empirical distribution produced during forecasting)				
	Black	Men	30	0.00179, 0.00203, 0.00231, 0.00262, 0.00297
	Black	Men	31	0.00186, 0.00209, 0.00235, 0.00263, 0.00296
	Black	Men	32	0.00192, 0.00215, 0.00239, 0.00266, 0.00297
	Black	Men	33	0.00197, 0.00219, 0.00244, 0.00271, 0.00302
	Black	Men	34	0.00202, 0.00225, 0.00251, 0.00279, 0.00311
	Black	Men	35	0.00208, 0.00232, 0.00259, 0.00289, 0.00322
	Black	Men	36	0.00216, 0.00241, 0.00268, 0.00299, 0.00333
	Black	Men	37	0.00224, 0.0025, 0.00278, 0.00309, 0.00345
	Black	Men	38	0.00232, 0.00259, 0.00288, 0.0032, 0.00357
	Black	Men	39	0.0024, 0.00268, 0.00299, 0.00333, 0.00372
	Black	Men	40	0.00246, 0.00277, 0.00311, 0.00349, 0.00393
	Black	Men	41	0.00254, 0.00288, 0.00326, 0.00369, 0.00419
	Black	Men	42	0.00264, 0.00302, 0.00345, 0.00394, 0.0045
	Black	Men	43	0.00278, 0.0032, 0.00368, 0.00423, 0.00487
	Black	Men	44	0.00295, 0.00342, 0.00396, 0.00458, 0.00532
	Black	Men	45	0.00316, 0.00368, 0.00429, 0.00499, 0.00583
	Black	Men	46	0.00342, 0.004, 0.00467, 0.00545, 0.00638
	Black	Men	47	0.00375, 0.00438, 0.0051, 0.00595, 0.00694
	Black	Men	48	0.00416, 0.00483, 0.00559, 0.00648, 0.00752
	Black	Men	49	0.00465, 0.00535, 0.00615, 0.00706, 0.00812
	Black	Men	50	0.00521, 0.00595, 0.00677, 0.00771, 0.00879
	Black	Men	51	0.00585, 0.00661, 0.00746, 0.00842, 0.00952
	Black	Men	52	0.00657, 0.00736, 0.00822, 0.00919, 0.0103
	Black	Men	53	0.00739, 0.00818, 0.00905, 0.01, 0.0111
	Black	Men	54	0.00828, 0.00908, 0.00995, 0.0109, 0.0119
	Black	Men	55	0.00923, 0.01, 0.0109, 0.0119, 0.0129
	Black	Men	56	0.0103, 0.0111, 0.0119, 0.0129, 0.0139
	Black	Men	57	0.0114, 0.0122, 0.0131, 0.014, 0.015
	Black	Men	58	0.0126, 0.0134, 0.0143, 0.0152, 0.0162
	Black	Men	59	0.0138, 0.0147, 0.0155, 0.0164, 0.0174
	Black	Men	60	0.015, 0.0159, 0.0168, 0.0178, 0.0189
	Black	Men	61	0.0162, 0.0171, 0.0181, 0.0192, 0.0203
	Black	Men	62	0.0174, 0.0184, 0.0194, 0.0205, 0.0216
	Black	Men	63	0.0185, 0.0195, 0.0206, 0.0218, 0.023
	Black	Men	64	0.0195, 0.0206, 0.0218, 0.0231, 0.0244
	Black	Men	65	0.0206, 0.0218, 0.023, 0.0244, 0.0258
	Black	Men	66	0.0216, 0.0229, 0.0243, 0.0257, 0.0272
	Black	Men	67	0.0228, 0.0242, 0.0257, 0.0272, 0.0289
	Black	Men	68	0.0241, 0.0256, 0.0272, 0.029, 0.0308
	Black	Men	69	0.0255, 0.0272, 0.029, 0.031, 0.033

Variable	Race/ethnicity	Sex	Age	Distribution
	Black	Men	70	0.0272, 0.0291, 0.0311, 0.0332, 0.0356
	Black	Men	71	0.029, 0.0311, 0.0334, 0.0358, 0.0384
	Black	Men	72	0.0311, 0.0334, 0.0359, 0.0385, 0.0414
	Black	Men	73	0.0335, 0.036, 0.0386, 0.0414, 0.0445
	Black	Men	74	0.0362, 0.0388, 0.0415, 0.0445, 0.0477
	Black	Men	75	0.0392, 0.0419, 0.0447, 0.0477, 0.0509
	Black	Men	76	0.0426, 0.0453, 0.0481, 0.0511, 0.0543
	Black	Men	77	0.0461, 0.0489, 0.0518, 0.0549, 0.0582
	Black	Men	78	0.0499, 0.0529, 0.056, 0.0593, 0.0629
	Black	Men	79	0.0539, 0.0572, 0.0607, 0.0644, 0.0683
	Black	Men	80	0.0582, 0.0619, 0.0658, 0.0699, 0.0744
	Black	Men	81	0.0627, 0.0669, 0.0713, 0.0759, 0.081
	Black	Men	82	0.0674, 0.0721, 0.077, 0.0823, 0.088
	Black	Men	83	0.072, 0.0772, 0.0828, 0.0887, 0.0952
	Black	Men	84	0.0763, 0.0822, 0.0884, 0.0951, 0.102
	Black	Women	30	0.000681, 0.000789, 0.000913, 0.00106, 0.00122
	Black	Women	31	0.000751, 0.00086, 0.000983, 0.00112, 0.00129
	Black	Women	32	0.000832, 0.000941, 0.00106, 0.0012, 0.00136
	Black	Women	33	0.000918, 0.00103, 0.00115, 0.00128, 0.00143
	Black	Women	34	0.000997, 0.00111, 0.00123, 0.00137, 0.00153
	Black	Women	35	0.00106, 0.00119, 0.00132, 0.00147, 0.00164
	Black	Women	36	0.00113, 0.00126, 0.00141, 0.00157, 0.00176
	Black	Women	37	0.00119, 0.00134, 0.0015, 0.00167, 0.00188
	Black	Women	38	0.00127, 0.00143, 0.0016, 0.00179, 0.002
	Black	Women	39	0.00137, 0.00153, 0.00171, 0.00191, 0.00214
	Black	Women	40	0.00148, 0.00165, 0.00184, 0.00206, 0.0023
	Black	Women	41	0.00161, 0.0018, 0.002, 0.00222, 0.00247
	Black	Women	42	0.00178, 0.00197, 0.00217, 0.0024, 0.00265
	Black	Women	43	0.00198, 0.00216, 0.00237, 0.00259, 0.00284
	Black	Women	44	0.0022, 0.00239, 0.00259, 0.0028, 0.00304
	Black	Women	45	0.00244, 0.00263, 0.00283, 0.00305, 0.00328
	Black	Women	46	0.00267, 0.00288, 0.0031, 0.00334, 0.0036
	Black	Women	47	0.00291, 0.00315, 0.0034, 0.00368, 0.00398
	Black	Women	48	0.00318, 0.00345, 0.00374, 0.00405, 0.0044
	Black	Women	49	0.00349, 0.00378, 0.00411, 0.00446, 0.00484
	Black	Women	50	0.00385, 0.00417, 0.00451, 0.00487, 0.00527
	Black	Women	51	0.00429, 0.00461, 0.00493, 0.00529, 0.00567
	Black	Women	52	0.0048, 0.00509, 0.00539, 0.00572, 0.00606
	Black	Women	53	0.00531, 0.00559, 0.00588, 0.00618, 0.00651
	Black	Women	54	0.0058, 0.00609, 0.00639, 0.00671, 0.00705
	Black	Women	55	0.00628, 0.0066, 0.00694, 0.00729, 0.00767
	Black	Women	56	0.00676, 0.00712, 0.0075, 0.0079, 0.00833
	Black	Women	57	0.00727, 0.00767, 0.00808, 0.00852, 0.00899

Variable	Race/ethnicity	Sex	Age	Distribution
	Black	Women	58	0.00779, 0.00822, 0.00867, 0.00914, 0.00965
	Black	Women	59	0.0083, 0.00877, 0.00926, 0.00977, 0.0103
	Black	Women	60	0.0088, 0.00931, 0.00984, 0.0104, 0.011
	Black	Women	61	0.00932, 0.00986, 0.0104, 0.011, 0.0117
	Black	Women	62	0.00985, 0.0104, 0.011, 0.0117, 0.0124
	Black	Women	63	0.0104, 0.011, 0.0117, 0.0124, 0.0131
	Black	Women	64	0.011, 0.0117, 0.0124, 0.0132, 0.014
	Black	Women	65	0.0117, 0.0124, 0.0132, 0.0141, 0.015
	Black	Women	66	0.0125, 0.0133, 0.0142, 0.0151, 0.0161
	Black	Women	67	0.0134, 0.0143, 0.0152, 0.0162, 0.0173
	Black	Women	68	0.0144, 0.0154, 0.0164, 0.0175, 0.0187
	Black	Women	69	0.0156, 0.0166, 0.0178, 0.019, 0.0203
	Black	Women	70	0.0168, 0.018, 0.0192, 0.0205, 0.022
	Black	Women	71	0.0182, 0.0195, 0.0208, 0.0222, 0.0238
	Black	Women	72	0.0197, 0.0211, 0.0225, 0.0241, 0.0257
	Black	Women	73	0.0213, 0.0228, 0.0244, 0.026, 0.0278
	Black	Women	74	0.0232, 0.0248, 0.0264, 0.0282, 0.0301
	Black	Women	75	0.0253, 0.0269, 0.0287, 0.0305, 0.0325
	Black	Women	76	0.0276, 0.0294, 0.0312, 0.0332, 0.0353
	Black	Women	77	0.0303, 0.0321, 0.0341, 0.0362, 0.0384
	Black	Women	78	0.0332, 0.0352, 0.0374, 0.0397, 0.0421
	Black	Women	79	0.0364, 0.0387, 0.0411, 0.0436, 0.0463
	Black	Women	80	0.0399, 0.0425, 0.0452, 0.048, 0.0511
	Black	Women	81	0.0436, 0.0465, 0.0496, 0.0529, 0.0564
	Black	Women	82	0.0473, 0.0507, 0.0543, 0.0582, 0.0624
	Black	Women	83	0.051, 0.055, 0.0592, 0.0637, 0.0687
	Black	Women	84	0.0547, 0.0592, 0.0641, 0.0694, 0.0752
	Hispanic	Men	30	0.000808, 0.0009, 0.001, 0.00111, 0.00124
	Hispanic	Men	31	0.000824, 0.000917, 0.00102, 0.00113, 0.00126
	Hispanic	Men	32	0.000839, 0.000934, 0.00104, 0.00116, 0.00129
	Hispanic	Men	33	0.000844, 0.000946, 0.00106, 0.00119, 0.00133
	Hispanic	Men	34	0.000846, 0.000956, 0.00108, 0.00122, 0.00138
	Hispanic	Men	35	0.000854, 0.000972, 0.00111, 0.00126, 0.00143
	Hispanic	Men	36	0.000876, 0.000999, 0.00114, 0.0013, 0.00148
	Hispanic	Men	37	0.000915, 0.00104, 0.00118, 0.00134, 0.00152
	Hispanic	Men	38	0.000966, 0.00109, 0.00123, 0.00139, 0.00158
	Hispanic	Men	39	0.00103, 0.00116, 0.0013, 0.00146, 0.00165
	Hispanic	Men	40	0.00109, 0.00123, 0.00138, 0.00155, 0.00175
	Hispanic	Men	41	0.00117, 0.00131, 0.00147, 0.00166, 0.00186
	Hispanic	Men	42	0.00127, 0.00142, 0.00159, 0.00178, 0.00199
	Hispanic	Men	43	0.0014, 0.00156, 0.00172, 0.00191, 0.00212
	Hispanic	Men	44	0.00157, 0.00173, 0.00189, 0.00207, 0.00226
	Hispanic	Men	45	0.00177, 0.00192, 0.00208, 0.00226, 0.00245

Variable	Race/ethnicity	Sex	Age	Distribution
	Hispanic	Men	46	0.00197, 0.00213, 0.00231, 0.00249, 0.00269
	Hispanic	Men	47	0.00219, 0.00237, 0.00256, 0.00277, 0.00299
	Hispanic	Men	48	0.00241, 0.00262, 0.00284, 0.00308, 0.00334
	Hispanic	Men	49	0.00266, 0.00289, 0.00314, 0.00341, 0.0037
	Hispanic	Men	50	0.00294, 0.00319, 0.00346, 0.00375, 0.00406
	Hispanic	Men	51	0.00328, 0.00353, 0.00379, 0.00408, 0.00438
	Hispanic	Men	52	0.0037, 0.00392, 0.00415, 0.00439, 0.00466
	Hispanic	Men	53	0.00416, 0.00434, 0.00452, 0.00471, 0.00491
	Hispanic	Men	54	0.0046, 0.00475, 0.00491, 0.00508, 0.00525
	Hispanic	Men	55	0.00494, 0.00513, 0.00533, 0.00554, 0.00575
	Hispanic	Men	56	0.00526, 0.00551, 0.00577, 0.00604, 0.00633
	Hispanic	Men	57	0.00562, 0.00592, 0.00624, 0.00658, 0.00693
	Hispanic	Men	58	0.00601, 0.00637, 0.00674, 0.00713, 0.00755
	Hispanic	Men	59	0.00643, 0.00684, 0.00726, 0.00771, 0.0082
	Hispanic	Men	60	0.00688, 0.00733, 0.00781, 0.00831, 0.00885
	Hispanic	Men	61	0.00738, 0.00787, 0.00837, 0.00891, 0.00949
	Hispanic	Men	62	0.00794, 0.00844, 0.00896, 0.00952, 0.0101
	Hispanic	Men	63	0.00853, 0.00904, 0.00958, 0.0101, 0.0107
	Hispanic	Men	64	0.00912, 0.00966, 0.0102, 0.0108, 0.0115
	Hispanic	Men	65	0.00971, 0.0103, 0.0109, 0.0116, 0.0123
	Hispanic	Men	66	0.0103, 0.011, 0.0117, 0.0125, 0.0133
	Hispanic	Men	67	0.011, 0.0118, 0.0126, 0.0135, 0.0144
	Hispanic	Men	68	0.0119, 0.0127, 0.0136, 0.0146, 0.0157
	Hispanic	Men	69	0.0128, 0.0138, 0.0148, 0.0159, 0.017
	Hispanic	Men	70	0.0139, 0.0149, 0.0161, 0.0173, 0.0186
	Hispanic	Men	71	0.0151, 0.0163, 0.0175, 0.0188, 0.0203
	Hispanic	Men	72	0.0164, 0.0177, 0.0191, 0.0206, 0.0221
	Hispanic	Men	73	0.018, 0.0193, 0.0208, 0.0224, 0.0242
	Hispanic	Men	74	0.0197, 0.0212, 0.0228, 0.0245, 0.0264
	Hispanic	Men	75	0.0216, 0.0232, 0.0249, 0.0268, 0.0288
	Hispanic	Men	76	0.0238, 0.0255, 0.0274, 0.0293, 0.0315
	Hispanic	Men	77	0.0263, 0.0282, 0.0302, 0.0323, 0.0347
	Hispanic	Men	78	0.0291, 0.0312, 0.0334, 0.0358, 0.0384
	Hispanic	Men	79	0.0324, 0.0347, 0.0372, 0.0398, 0.0427
	Hispanic	Men	80	0.0362, 0.0388, 0.0415, 0.0444, 0.0476
	Hispanic	Men	81	0.0403, 0.0432, 0.0462, 0.0495, 0.053
	Hispanic	Men	82	0.0447, 0.0479, 0.0513, 0.055, 0.059
	Hispanic	Men	83	0.0489, 0.0526, 0.0566, 0.0608, 0.0654
	Hispanic	Men	84	0.0531, 0.0573, 0.0617, 0.0666, 0.0718
	Hispanic	Women	30	0.000329, 0.000371, 0.000417, 0.000469, 0.000527
	Hispanic	Women	31	0.000348, 0.00039, 0.000438, 0.000491, 0.000552
	Hispanic	Women	32	0.000368, 0.000412, 0.000461, 0.000515, 0.000576
	Hispanic	Women	33	0.000391, 0.000435, 0.000484, 0.000539, 0.0006

Variable	Race/ethnicity	Sex	Age	Distribution
	Hispanic	Women	34	0.000412, 0.000459, 0.000509, 0.000566, 0.000629
	Hispanic	Women	35	0.000432, 0.000482, 0.000537, 0.000598, 0.000667
	Hispanic	Women	36	0.000452, 0.000506, 0.000567, 0.000635, 0.000712
	Hispanic	Women	37	0.000476, 0.000536, 0.000602, 0.000676, 0.000761
	Hispanic	Women	38	0.000507, 0.000571, 0.000642, 0.000721, 0.000812
	Hispanic	Women	39	0.000547, 0.000613, 0.000687, 0.00077, 0.000864
	Hispanic	Women	40	0.000596, 0.000664, 0.000739, 0.000823, 0.000918
	Hispanic	Women	41	0.000655, 0.000724, 0.000799, 0.000883, 0.000976
	Hispanic	Women	42	0.000723, 0.000793, 0.000869, 0.000952, 0.00104
	Hispanic	Women	43	0.000802, 0.000873, 0.000949, 0.00103, 0.00112
	Hispanic	Women	44	0.000891, 0.000964, 0.00104, 0.00113, 0.00122
	Hispanic	Women	45	0.000988, 0.00107, 0.00115, 0.00124, 0.00133
	Hispanic	Women	46	0.00109, 0.00118, 0.00127, 0.00136, 0.00147
	Hispanic	Women	47	0.00121, 0.0013, 0.0014, 0.0015, 0.00162
	Hispanic	Women	48	0.00133, 0.00143, 0.00154, 0.00166, 0.00179
	Hispanic	Women	49	0.00146, 0.00157, 0.0017, 0.00183, 0.00197
	Hispanic	Women	50	0.0016, 0.00173, 0.00186, 0.00201, 0.00217
	Hispanic	Women	51	0.00176, 0.0019, 0.00204, 0.0022, 0.00237
	Hispanic	Women	52	0.00193, 0.00208, 0.00223, 0.0024, 0.00258
	Hispanic	Women	53	0.00212, 0.00227, 0.00244, 0.00261, 0.0028
	Hispanic	Women	54	0.00231, 0.00248, 0.00265, 0.00284, 0.00305
	Hispanic	Women	55	0.00251, 0.00269, 0.00289, 0.00309, 0.00332
	Hispanic	Women	56	0.00272, 0.00292, 0.00313, 0.00336, 0.00361
	Hispanic	Women	57	0.00296, 0.00317, 0.0034, 0.00364, 0.0039
	Hispanic	Women	58	0.00321, 0.00344, 0.00368, 0.00394, 0.00422
	Hispanic	Women	59	0.00348, 0.00373, 0.00399, 0.00427, 0.00457
	Hispanic	Women	60	0.00376, 0.00403, 0.00432, 0.00463, 0.00496
	Hispanic	Women	61	0.00407, 0.00436, 0.00468, 0.00501, 0.00538
	Hispanic	Women	62	0.0044, 0.00472, 0.00506, 0.00543, 0.00583
	Hispanic	Women	63	0.00475, 0.00511, 0.00548, 0.00589, 0.00633
	Hispanic	Women	64	0.00513, 0.00552, 0.00594, 0.0064, 0.00689
	Hispanic	Women	65	0.00555, 0.00598, 0.00645, 0.00696, 0.0075
	Hispanic	Women	66	0.00601, 0.0065, 0.00701, 0.00757, 0.00818
	Hispanic	Women	67	0.00656, 0.00708, 0.00765, 0.00825, 0.00892
	Hispanic	Women	68	0.00718, 0.00775, 0.00836, 0.00901, 0.00973
	Hispanic	Women	69	0.0079, 0.00851, 0.00916, 0.00987, 0.0106
	Hispanic	Women	70	0.00871, 0.00937, 0.0101, 0.0108, 0.0117
	Hispanic	Women	71	0.0096, 0.0103, 0.0111, 0.0119, 0.0128
	Hispanic	Women	72	0.0106, 0.0114, 0.0122, 0.0131, 0.0141
	Hispanic	Women	73	0.0117, 0.0126, 0.0135, 0.0145, 0.0156
	Hispanic	Women	74	0.013, 0.014, 0.015, 0.016, 0.0172
	Hispanic	Women	75	0.0145, 0.0155, 0.0166, 0.0177, 0.0189
	Hispanic	Women	76	0.0163, 0.0173, 0.0185, 0.0196, 0.0209

Variable	Race/ethnicity	Sex	Age	Distribution
	Hispanic	Women	77	0.0183, 0.0194, 0.0206, 0.0219, 0.0232
	Hispanic	Women	78	0.0206, 0.0218, 0.0231, 0.0245, 0.026
	Hispanic	Women	79	0.0232, 0.0246, 0.0261, 0.0276, 0.0293
	Hispanic	Women	80	0.0261, 0.0277, 0.0294, 0.0312, 0.0331
	Hispanic	Women	81	0.0292, 0.0311, 0.0331, 0.0353, 0.0376
	Hispanic	Women	82	0.0325, 0.0347, 0.0372, 0.0398, 0.0426
	Hispanic	Women	83	0.0358, 0.0385, 0.0414, 0.0446, 0.048
	Hispanic	Women	84	0.0392, 0.0424, 0.0458, 0.0495, 0.0535
	White	Men	30	0.00124, 0.00142, 0.00161, 0.00184, 0.00209
	White	Men	31	0.00129, 0.00146, 0.00166, 0.00187, 0.00212
	White	Men	32	0.00134, 0.00151, 0.0017, 0.00191, 0.00214
	White	Men	33	0.00138, 0.00155, 0.00173, 0.00193, 0.00217
	White	Men	34	0.00141, 0.00158, 0.00176, 0.00197, 0.0022
	White	Men	35	0.00144, 0.00161, 0.0018, 0.00201, 0.00225
	White	Men	36	0.00146, 0.00164, 0.00184, 0.00206, 0.00231
	White	Men	37	0.0015, 0.00169, 0.00189, 0.00212, 0.00238
	White	Men	38	0.00155, 0.00174, 0.00196, 0.0022, 0.00247
	White	Men	39	0.00162, 0.00182, 0.00204, 0.00229, 0.00256
	White	Men	40	0.00171, 0.00191, 0.00214, 0.00239, 0.00268
	White	Men	41	0.00182, 0.00203, 0.00226, 0.00253, 0.00282
	White	Men	42	0.00195, 0.00217, 0.00242, 0.00269, 0.00299
	White	Men	43	0.00213, 0.00235, 0.0026, 0.00287, 0.00318
	White	Men	44	0.00234, 0.00257, 0.00282, 0.0031, 0.00341
	White	Men	45	0.00257, 0.00281, 0.00308, 0.00338, 0.0037
	White	Men	46	0.00281, 0.00309, 0.00339, 0.00372, 0.00409
	White	Men	47	0.00307, 0.00339, 0.00373, 0.00412, 0.00454
	White	Men	48	0.00337, 0.00373, 0.00412, 0.00455, 0.00504
	White	Men	49	0.00371, 0.00411, 0.00454, 0.00503, 0.00556
	White	Men	50	0.00411, 0.00454, 0.005, 0.00552, 0.00609
	White	Men	51	0.00456, 0.00501, 0.00549, 0.00602, 0.00661
	White	Men	52	0.00507, 0.00552, 0.006, 0.00653, 0.00711
	White	Men	53	0.00562, 0.00607, 0.00654, 0.00705, 0.0076
	White	Men	54	0.00618, 0.00662, 0.00709, 0.00759, 0.00814
	White	Men	55	0.0067, 0.00716, 0.00766, 0.00818, 0.00875
	White	Men	56	0.00719, 0.0077, 0.00824, 0.00881, 0.00944
	White	Men	57	0.00768, 0.00824, 0.00883, 0.00947, 0.0102
	White	Men	58	0.0082, 0.0088, 0.00945, 0.0101, 0.0109
	White	Men	59	0.00877, 0.00941, 0.0101, 0.0108, 0.0116
	White	Men	60	0.00939, 0.01, 0.0107, 0.0115, 0.0123
	White	Men	61	0.0101, 0.0107, 0.0114, 0.0122, 0.013
	White	Men	62	0.0108, 0.0115, 0.0122, 0.0129, 0.0137
	White	Men	63	0.0115, 0.0122, 0.0129, 0.0137, 0.0145
	White	Men	64	0.0123, 0.013, 0.0138, 0.0145, 0.0154

Variable	Race/ethnicity	Sex	Age	Distribution
	White	Men	65	0.0131, 0.0139, 0.0147, 0.0155, 0.0164
	White	Men	66	0.014, 0.0149, 0.0157, 0.0167, 0.0177
	White	Men	67	0.015, 0.016, 0.017, 0.018, 0.0191
	White	Men	68	0.0162, 0.0173, 0.0184, 0.0195, 0.0208
	White	Men	69	0.0176, 0.0187, 0.02, 0.0213, 0.0227
	White	Men	70	0.0191, 0.0204, 0.0218, 0.0232, 0.0248
	White	Men	71	0.0209, 0.0223, 0.0238, 0.0254, 0.0271
	White	Men	72	0.0228, 0.0244, 0.026, 0.0278, 0.0296
	White	Men	73	0.025, 0.0267, 0.0284, 0.0303, 0.0323
	White	Men	74	0.0274, 0.0292, 0.0311, 0.0331, 0.0352
	White	Men	75	0.0301, 0.032, 0.034, 0.0361, 0.0384
	White	Men	76	0.0332, 0.0352, 0.0373, 0.0395, 0.0419
	White	Men	77	0.0367, 0.0389, 0.0411, 0.0435, 0.0461
	White	Men	78	0.0406, 0.043, 0.0455, 0.0482, 0.051
	White	Men	79	0.0451, 0.0478, 0.0507, 0.0537, 0.0569
	White	Men	80	0.0502, 0.0533, 0.0566, 0.06, 0.0637
	White	Men	81	0.0559, 0.0595, 0.0632, 0.0671, 0.0714
	White	Men	82	0.062, 0.0661, 0.0704, 0.0749, 0.0799
	White	Men	83	0.0682, 0.0729, 0.0779, 0.0832, 0.0889
	White	Men	84	0.0743, 0.0797, 0.0854, 0.0915, 0.0981
	White	Women	30	0.000617, 0.000683, 0.000756, 0.000836, 0.000926
	White	Women	31	0.00065, 0.000722, 0.0008, 0.000888, 0.000985
	White	Women	32	0.000688, 0.000763, 0.000845, 0.000937, 0.00104
	White	Women	33	0.000728, 0.000805, 0.000891, 0.000985, 0.00109
	White	Women	34	0.000763, 0.000846, 0.000937, 0.00104, 0.00115
	White	Women	35	0.000795, 0.000886, 0.000987, 0.0011, 0.00123
	White	Women	36	0.000828, 0.000929, 0.00104, 0.00117, 0.00131
	White	Women	37	0.000867, 0.000978, 0.0011, 0.00124, 0.00141
	White	Women	38	0.000912, 0.00103, 0.00117, 0.00133, 0.00151
	White	Women	39	0.000963, 0.0011, 0.00125, 0.00142, 0.00162
	White	Women	40	0.00103, 0.00117, 0.00134, 0.00153, 0.00175
	White	Women	41	0.0011, 0.00126, 0.00144, 0.00165, 0.00188
	White	Women	42	0.00119, 0.00137, 0.00156, 0.00178, 0.00203
	White	Women	43	0.0013, 0.00149, 0.00169, 0.00193, 0.0022
	White	Women	44	0.00143, 0.00162, 0.00185, 0.0021, 0.00239
	White	Women	45	0.00156, 0.00178, 0.00202, 0.0023, 0.00262
	White	Women	46	0.00172, 0.00195, 0.00222, 0.00252, 0.00287
	White	Women	47	0.0019, 0.00215, 0.00244, 0.00276, 0.00313
	White	Women	48	0.00211, 0.00238, 0.00267, 0.003, 0.00338
	White	Women	49	0.00235, 0.00262, 0.00293, 0.00326, 0.00365
	White	Women	50	0.00258, 0.00287, 0.00319, 0.00355, 0.00396
	White	Women	51	0.0028, 0.00312, 0.00348, 0.00388, 0.00432
	White	Women	52	0.00301, 0.00337, 0.00377, 0.00422, 0.00473

Variable	Race/ethnicity	Sex	Age	Distribution
	White	Women	53	0.00322, 0.00363, 0.00407, 0.00457, 0.00514
	White	Women	54	0.00348, 0.0039, 0.00437, 0.0049, 0.00549
	White	Women	55	0.00378, 0.0042, 0.00467, 0.00518, 0.00576
	White	Women	56	0.00414, 0.00454, 0.00496, 0.00543, 0.00594
	White	Women	57	0.00454, 0.00489, 0.00526, 0.00566, 0.00609
	White	Women	58	0.00495, 0.00525, 0.00557, 0.00591, 0.00628
	White	Women	59	0.00531, 0.0056, 0.00591, 0.00623, 0.00658
	White	Women	60	0.00564, 0.00596, 0.00629, 0.00664, 0.00701
	White	Women	61	0.006, 0.00636, 0.00672, 0.00711, 0.00753
	White	Women	62	0.00642, 0.00681, 0.00723, 0.00767, 0.00814
	White	Women	63	0.00691, 0.00735, 0.00781, 0.00831, 0.00884
	White	Women	64	0.00749, 0.00797, 0.00849, 0.00904, 0.00963
	White	Women	65	0.00815, 0.00869, 0.00926, 0.00987, 0.0105
	White	Women	66	0.00889, 0.00949, 0.0101, 0.0108, 0.0116
	White	Women	67	0.0097, 0.0104, 0.0111, 0.0119, 0.0127
	White	Women	68	0.0106, 0.0114, 0.0122, 0.0131, 0.0141
	White	Women	69	0.0115, 0.0125, 0.0134, 0.0145, 0.0156
	White	Women	70	0.0126, 0.0137, 0.0148, 0.016, 0.0173
	White	Women	71	0.0138, 0.015, 0.0163, 0.0177, 0.0192
	White	Women	72	0.0151, 0.0165, 0.0179, 0.0195, 0.0212
	White	Women	73	0.0167, 0.0181, 0.0197, 0.0214, 0.0233
	White	Women	74	0.0184, 0.02, 0.0217, 0.0235, 0.0255
	White	Women	75	0.0205, 0.0222, 0.0239, 0.0258, 0.0279
	White	Women	76	0.023, 0.0247, 0.0265, 0.0284, 0.0306
	White	Women	77	0.0258, 0.0275, 0.0294, 0.0315, 0.0336
	White	Women	78	0.0289, 0.0308, 0.0328, 0.035, 0.0373
	White	Women	79	0.0325, 0.0346, 0.0368, 0.0391, 0.0416
	White	Women	80	0.0364, 0.0388, 0.0412, 0.0439, 0.0467
	White	Women	81	0.0408, 0.0434, 0.0463, 0.0493, 0.0525
	White	Women	82	0.0454, 0.0485, 0.0518, 0.0552, 0.059
	White	Women	83	0.0502, 0.0538, 0.0576, 0.0616, 0.066
	White	Women	84	0.0552, 0.0592, 0.0636, 0.0682, 0.0733
Total mortality rates for 2020 (0.01, 0.2, 0.5, 0.8. 0.99 percentiles of the empirical distribution produced during forecasting)				
	Black	Men	30	0.00176, 0.00201, 0.00231, 0.00264, 0.00303
	Black	Men	31	0.00184, 0.00208, 0.00235, 0.00265, 0.003
	Black	Men	32	0.0019, 0.00213, 0.00239, 0.00268, 0.003
	Black	Men	33	0.00195, 0.00218, 0.00244, 0.00273, 0.00305
	Black	Men	34	0.002, 0.00224, 0.0025, 0.0028, 0.00314
	Black	Men	35	0.00205, 0.0023, 0.00258, 0.00289, 0.00324
	Black	Men	36	0.00212, 0.00238, 0.00267, 0.00299, 0.00335
	Black	Men	37	0.0022, 0.00246, 0.00276, 0.00309, 0.00346
	Black	Men	38	0.00227, 0.00254, 0.00285, 0.00319, 0.00358
	Black	Men	39	0.00233, 0.00262, 0.00295, 0.00331, 0.00373

Variable	Race/ethnicity	Sex	Age	Distribution
	Black	Men	40	0.00239, 0.00271, 0.00306, 0.00347, 0.00393
	Black	Men	41	0.00246, 0.00281, 0.00321, 0.00366, 0.00418
	Black	Men	42	0.00255, 0.00294, 0.00339, 0.0039, 0.0045
	Black	Men	43	0.00268, 0.00311, 0.00362, 0.0042, 0.00488
	Black	Men	44	0.00284, 0.00333, 0.00389, 0.00455, 0.00533
	Black	Men	45	0.00303, 0.00358, 0.00422, 0.00496, 0.00586
	Black	Men	46	0.00328, 0.00388, 0.00459, 0.00543, 0.00643
	Black	Men	47	0.0036, 0.00425, 0.00502, 0.00593, 0.00701
	Black	Men	48	0.00399, 0.0047, 0.00551, 0.00647, 0.00761
	Black	Men	49	0.00447, 0.00521, 0.00607, 0.00706, 0.00823
	Black	Men	50	0.00502, 0.0058, 0.00669, 0.00772, 0.00892
	Black	Men	51	0.00564, 0.00646, 0.00738, 0.00844, 0.00966
	Black	Men	52	0.00635, 0.0072, 0.00814, 0.00921, 0.0104
	Black	Men	53	0.00716, 0.00802, 0.00897, 0.01, 0.0112
	Black	Men	54	0.00805, 0.00892, 0.00986, 0.0109, 0.0121
	Black	Men	55	0.00901, 0.00988, 0.0108, 0.0119, 0.013
	Black	Men	56	0.01, 0.0109, 0.0119, 0.0129, 0.014
	Black	Men	57	0.0112, 0.012, 0.013, 0.014, 0.0151
	Black	Men	58	0.0124, 0.0132, 0.0142, 0.0151, 0.0162
	Black	Men	59	0.0136, 0.0145, 0.0154, 0.0164, 0.0175
	Black	Men	60	0.0147, 0.0157, 0.0167, 0.0177, 0.0189
	Black	Men	61	0.0159, 0.0169, 0.0179, 0.019, 0.0202
	Black	Men	62	0.0171, 0.0181, 0.0192, 0.0203, 0.0215
	Black	Men	63	0.0181, 0.0192, 0.0204, 0.0216, 0.0229
	Black	Men	64	0.0191, 0.0203, 0.0215, 0.0228, 0.0242
	Black	Men	65	0.0201, 0.0214, 0.0227, 0.0241, 0.0256
	Black	Men	66	0.0212, 0.0225, 0.0239, 0.0254, 0.027
	Black	Men	67	0.0223, 0.0237, 0.0253, 0.0269, 0.0287
	Black	Men	68	0.0235, 0.0251, 0.0268, 0.0286, 0.0306
	Black	Men	69	0.0249, 0.0267, 0.0286, 0.0306, 0.0328
	Black	Men	70	0.0265, 0.0285, 0.0306, 0.0329, 0.0353
	Black	Men	71	0.0283, 0.0305, 0.0328, 0.0353, 0.0381
	Black	Men	72	0.0304, 0.0328, 0.0353, 0.038, 0.041
	Black	Men	73	0.0327, 0.0352, 0.038, 0.0409, 0.0441
	Black	Men	74	0.0353, 0.038, 0.0408, 0.0439, 0.0472
	Black	Men	75	0.0383, 0.041, 0.0439, 0.047, 0.0504
	Black	Men	76	0.0415, 0.0443, 0.0473, 0.0504, 0.0538
	Black	Men	77	0.045, 0.0479, 0.051, 0.0543, 0.0577
	Black	Men	78	0.0487, 0.0518, 0.0551, 0.0586, 0.0624
	Black	Men	79	0.0526, 0.0561, 0.0597, 0.0636, 0.0678
	Black	Men	80	0.0568, 0.0607, 0.0648, 0.0692, 0.0739
	Black	Men	81	0.0612, 0.0656, 0.0702, 0.0751, 0.0805
	Black	Men	82	0.0658, 0.0707, 0.0759, 0.0815, 0.0875

Variable	Race/ethnicity	Sex	Age	Distribution
	Black	Men	83	0.0703, 0.0757, 0.0816, 0.0879, 0.0948
	Black	Men	84	0.0745, 0.0806, 0.0872, 0.0942, 0.102
	Black	Women	30	0.000661, 0.000776, 0.000911, 0.00107, 0.00126
	Black	Women	31	0.000731, 0.000847, 0.000981, 0.00114, 0.00132
	Black	Women	32	0.000812, 0.000929, 0.00106, 0.00121, 0.00138
	Black	Women	33	0.000898, 0.00101, 0.00114, 0.00129, 0.00146
	Black	Women	34	0.000975, 0.0011, 0.00123, 0.00138, 0.00155
	Black	Women	35	0.00104, 0.00117, 0.00131, 0.00148, 0.00166
	Black	Women	36	0.0011, 0.00124, 0.0014, 0.00157, 0.00178
	Black	Women	37	0.00116, 0.00131, 0.00148, 0.00168, 0.0019
	Black	Women	38	0.00123, 0.0014, 0.00158, 0.00178, 0.00202
	Black	Women	39	0.00132, 0.00149, 0.00169, 0.00191, 0.00216
	Black	Women	40	0.00143, 0.00161, 0.00182, 0.00205, 0.00231
	Black	Women	41	0.00156, 0.00175, 0.00196, 0.00221, 0.00248
	Black	Women	42	0.00172, 0.00191, 0.00213, 0.00238, 0.00265
	Black	Women	43	0.00191, 0.00211, 0.00233, 0.00257, 0.00283
	Black	Women	44	0.00213, 0.00233, 0.00254, 0.00277, 0.00303
	Black	Women	45	0.00237, 0.00257, 0.00278, 0.00301, 0.00327
	Black	Women	46	0.00259, 0.00281, 0.00305, 0.0033, 0.00358
	Black	Women	47	0.00283, 0.00308, 0.00335, 0.00364, 0.00397
	Black	Women	48	0.00309, 0.00337, 0.00368, 0.00402, 0.0044
	Black	Women	49	0.00339, 0.00371, 0.00405, 0.00443, 0.00484
	Black	Women	50	0.00375, 0.00409, 0.00445, 0.00484, 0.00528
	Black	Women	51	0.00419, 0.00453, 0.00488, 0.00526, 0.00568
	Black	Women	52	0.0047, 0.00501, 0.00534, 0.00569, 0.00607
	Black	Women	53	0.00521, 0.00551, 0.00583, 0.00616, 0.00651
	Black	Women	54	0.00571, 0.00602, 0.00634, 0.00668, 0.00705
	Black	Women	55	0.00618, 0.00652, 0.00688, 0.00726, 0.00766
	Black	Women	56	0.00666, 0.00704, 0.00744, 0.00787, 0.00832
	Black	Women	57	0.00716, 0.00758, 0.00802, 0.00848, 0.00898
	Black	Women	58	0.00767, 0.00812, 0.0086, 0.0091, 0.00964
	Black	Women	59	0.00816, 0.00865, 0.00917, 0.00972, 0.0103
	Black	Women	60	0.00864, 0.00918, 0.00974, 0.0103, 0.011
	Black	Women	61	0.00913, 0.00971, 0.0103, 0.011, 0.0117
	Black	Women	62	0.00965, 0.0103, 0.0109, 0.0116, 0.0123
	Black	Women	63	0.0102, 0.0108, 0.0115, 0.0123, 0.0131
	Black	Women	64	0.0108, 0.0115, 0.0122, 0.013, 0.0139
	Black	Women	65	0.0114, 0.0122, 0.013, 0.0139, 0.0149
	Black	Women	66	0.0122, 0.013, 0.0139, 0.0149, 0.016
	Black	Women	67	0.0131, 0.014, 0.015, 0.0161, 0.0172
	Black	Women	68	0.0141, 0.0151, 0.0162, 0.0173, 0.0186
	Black	Women	69	0.0152, 0.0163, 0.0175, 0.0187, 0.0201
	Black	Women	70	0.0164, 0.0176, 0.0189, 0.0203, 0.0218

Variable	Race/ethnicity	Sex	Age	Distribution
	Black	Women	71	0.0177, 0.0191, 0.0205, 0.022, 0.0236
	Black	Women	72	0.0192, 0.0206, 0.0221, 0.0238, 0.0255
	Black	Women	73	0.0208, 0.0223, 0.024, 0.0257, 0.0276
	Black	Women	74	0.0226, 0.0242, 0.026, 0.0278, 0.0298
	Black	Women	75	0.0247, 0.0264, 0.0282, 0.0301, 0.0322
	Black	Women	76	0.027, 0.0288, 0.0307, 0.0327, 0.035
	Black	Women	77	0.0296, 0.0315, 0.0336, 0.0357, 0.0381
	Black	Women	78	0.0324, 0.0346, 0.0368, 0.0392, 0.0417
	Black	Women	79	0.0356, 0.038, 0.0404, 0.0431, 0.0459
	Black	Women	80	0.039, 0.0417, 0.0445, 0.0474, 0.0507
	Black	Women	81	0.0426, 0.0457, 0.0489, 0.0523, 0.056
	Black	Women	82	0.0463, 0.0498, 0.0535, 0.0575, 0.0619
	Black	Women	83	0.0499, 0.054, 0.0583, 0.0631, 0.0682
	Black	Women	84	0.0535, 0.0582, 0.0632, 0.0687, 0.0747
	Hispanic	Men	30	0.000804, 0.000899, 0.001, 0.00112, 0.00125
	Hispanic	Men	31	0.00082, 0.000916, 0.00102, 0.00114, 0.00127
	Hispanic	Men	32	0.000834, 0.000932, 0.00104, 0.00116, 0.0013
	Hispanic	Men	33	0.000837, 0.000942, 0.00106, 0.00119, 0.00134
	Hispanic	Men	34	0.000836, 0.000951, 0.00108, 0.00123, 0.0014
	Hispanic	Men	35	0.000841, 0.000964, 0.0011, 0.00126, 0.00145
	Hispanic	Men	36	0.000861, 0.000989, 0.00113, 0.0013, 0.0015
	Hispanic	Men	37	0.000896, 0.00103, 0.00117, 0.00134, 0.00154
	Hispanic	Men	38	0.000945, 0.00108, 0.00123, 0.00139, 0.00159
	Hispanic	Men	39	0.001, 0.00114, 0.00129, 0.00146, 0.00166
	Hispanic	Men	40	0.00106, 0.0012, 0.00136, 0.00154, 0.00175
	Hispanic	Men	41	0.00113, 0.00129, 0.00146, 0.00165, 0.00187
	Hispanic	Men	42	0.00123, 0.00139, 0.00157, 0.00177, 0.00199
	Hispanic	Men	43	0.00136, 0.00152, 0.0017, 0.0019, 0.00212
	Hispanic	Men	44	0.00153, 0.00169, 0.00186, 0.00205, 0.00226
	Hispanic	Men	45	0.00173, 0.00188, 0.00205, 0.00224, 0.00244
	Hispanic	Men	46	0.00193, 0.00209, 0.00227, 0.00247, 0.00268
	Hispanic	Men	47	0.00214, 0.00232, 0.00252, 0.00274, 0.00298
	Hispanic	Men	48	0.00236, 0.00257, 0.0028, 0.00305, 0.00332
	Hispanic	Men	49	0.00261, 0.00284, 0.0031, 0.00338, 0.00369
	Hispanic	Men	50	0.00289, 0.00315, 0.00342, 0.00372, 0.00405
	Hispanic	Men	51	0.00323, 0.00349, 0.00376, 0.00405, 0.00438
	Hispanic	Men	52	0.00364, 0.00387, 0.00411, 0.00437, 0.00465
	Hispanic	Men	53	0.00411, 0.0043, 0.00449, 0.00469, 0.0049
	Hispanic	Men	54	0.00455, 0.00471, 0.00488, 0.00505, 0.00524
	Hispanic	Men	55	0.00488, 0.00509, 0.00529, 0.00551, 0.00574
	Hispanic	Men	56	0.0052, 0.00546, 0.00573, 0.00602, 0.00632
	Hispanic	Men	57	0.00555, 0.00587, 0.0062, 0.00655, 0.00692
	Hispanic	Men	58	0.00593, 0.0063, 0.00669, 0.0071, 0.00754

Variable	Race/ethnicity	Sex	Age	Distribution
	Hispanic	Men	59	0.00633, 0.00675, 0.0072, 0.00767, 0.00819
	Hispanic	Men	60	0.00676, 0.00723, 0.00773, 0.00826, 0.00884
	Hispanic	Men	61	0.00725, 0.00775, 0.00828, 0.00885, 0.00947
	Hispanic	Men	62	0.00778, 0.0083, 0.00885, 0.00944, 0.0101
	Hispanic	Men	63	0.00836, 0.00889, 0.00945, 0.01, 0.0107
	Hispanic	Men	64	0.00893, 0.00949, 0.0101, 0.0107, 0.0114
	Hispanic	Men	65	0.0095, 0.0101, 0.0108, 0.0115, 0.0122
	Hispanic	Men	66	0.0101, 0.0108, 0.0115, 0.0123, 0.0132
	Hispanic	Men	67	0.0108, 0.0116, 0.0124, 0.0133, 0.0143
	Hispanic	Men	68	0.0116, 0.0125, 0.0134, 0.0144, 0.0155
	Hispanic	Men	69	0.0125, 0.0135, 0.0145, 0.0157, 0.0169
	Hispanic	Men	70	0.0136, 0.0146, 0.0158, 0.0171, 0.0184
	Hispanic	Men	71	0.0147, 0.0159, 0.0172, 0.0186, 0.0201
	Hispanic	Men	72	0.0161, 0.0174, 0.0188, 0.0203, 0.022
	Hispanic	Men	73	0.0175, 0.019, 0.0205, 0.0222, 0.024
	Hispanic	Men	74	0.0192, 0.0207, 0.0224, 0.0242, 0.0262
	Hispanic	Men	75	0.0211, 0.0227, 0.0245, 0.0265, 0.0286
	Hispanic	Men	76	0.0232, 0.025, 0.0269, 0.029, 0.0313
	Hispanic	Men	77	0.0257, 0.0276, 0.0297, 0.032, 0.0345
	Hispanic	Men	78	0.0285, 0.0306, 0.033, 0.0355, 0.0382
	Hispanic	Men	79	0.0317, 0.0341, 0.0367, 0.0395, 0.0425
	Hispanic	Men	80	0.0354, 0.0381, 0.0409, 0.044, 0.0473
	Hispanic	Men	81	0.0394, 0.0424, 0.0456, 0.049, 0.0528
	Hispanic	Men	82	0.0437, 0.047, 0.0506, 0.0545, 0.0587
	Hispanic	Men	83	0.0479, 0.0517, 0.0558, 0.0602, 0.065
	Hispanic	Men	84	0.0519, 0.0563, 0.0609, 0.066, 0.0715
	Hispanic	Women	30	0.000329, 0.00037, 0.000417, 0.000469, 0.000528
	Hispanic	Women	31	0.000347, 0.00039, 0.000438, 0.000491, 0.000552
	Hispanic	Women	32	0.000368, 0.000412, 0.00046, 0.000514, 0.000576
	Hispanic	Women	33	0.00039, 0.000434, 0.000484, 0.000538, 0.0006
	Hispanic	Women	34	0.00041, 0.000457, 0.000508, 0.000565, 0.00063
	Hispanic	Women	35	0.000428, 0.000478, 0.000535, 0.000597, 0.000668
	Hispanic	Women	36	0.000446, 0.000502, 0.000564, 0.000634, 0.000713
	Hispanic	Women	37	0.000469, 0.000529, 0.000597, 0.000674, 0.000761
	Hispanic	Women	38	0.000498, 0.000563, 0.000635, 0.000717, 0.00081
	Hispanic	Women	39	0.000536, 0.000604, 0.000679, 0.000764, 0.000861
	Hispanic	Women	40	0.000583, 0.000652, 0.000729, 0.000816, 0.000913
	Hispanic	Women	41	0.000639, 0.00071, 0.000788, 0.000874, 0.000971
	Hispanic	Women	42	0.000705, 0.000777, 0.000855, 0.000941, 0.00104
	Hispanic	Women	43	0.000782, 0.000855, 0.000934, 0.00102, 0.00111
	Hispanic	Women	44	0.00087, 0.000944, 0.00102, 0.00111, 0.00121
	Hispanic	Women	45	0.000966, 0.00104, 0.00113, 0.00122, 0.00132
	Hispanic	Women	46	0.00107, 0.00115, 0.00125, 0.00134, 0.00145

Variable	Race/ethnicity	Sex	Age	Distribution
	Hispanic	Women	47	0.00118, 0.00128, 0.00138, 0.00149, 0.0016
	Hispanic	Women	48	0.0013, 0.00141, 0.00152, 0.00164, 0.00177
	Hispanic	Women	49	0.00143, 0.00155, 0.00167, 0.00181, 0.00196
	Hispanic	Women	50	0.00157, 0.0017, 0.00184, 0.00199, 0.00216
	Hispanic	Women	51	0.00173, 0.00187, 0.00202, 0.00218, 0.00236
	Hispanic	Women	52	0.00191, 0.00206, 0.00221, 0.00238, 0.00256
	Hispanic	Women	53	0.0021, 0.00225, 0.00242, 0.00259, 0.00278
	Hispanic	Women	54	0.00229, 0.00246, 0.00264, 0.00283, 0.00303
	Hispanic	Women	55	0.00249, 0.00267, 0.00287, 0.00308, 0.00331
	Hispanic	Women	56	0.00269, 0.0029, 0.00311, 0.00334, 0.00359
	Hispanic	Women	57	0.00293, 0.00314, 0.00337, 0.00362, 0.00389
	Hispanic	Women	58	0.00318, 0.00341, 0.00366, 0.00392, 0.00421
	Hispanic	Women	59	0.00344, 0.00369, 0.00396, 0.00424, 0.00456
	Hispanic	Women	60	0.00371, 0.00399, 0.00428, 0.0046, 0.00494
	Hispanic	Women	61	0.004, 0.00431, 0.00463, 0.00498, 0.00536
	Hispanic	Women	62	0.00432, 0.00466, 0.00501, 0.00539, 0.0058
	Hispanic	Women	63	0.00467, 0.00503, 0.00542, 0.00584, 0.00629
	Hispanic	Women	64	0.00504, 0.00544, 0.00587, 0.00633, 0.00683
	Hispanic	Women	65	0.00544, 0.00589, 0.00636, 0.00688, 0.00744
	Hispanic	Women	66	0.00589, 0.00639, 0.00691, 0.00748, 0.00811
	Hispanic	Women	67	0.00642, 0.00696, 0.00753, 0.00816, 0.00884
	Hispanic	Women	68	0.00703, 0.00761, 0.00823, 0.00891, 0.00964
	Hispanic	Women	69	0.00772, 0.00835, 0.00902, 0.00975, 0.0105
	Hispanic	Women	70	0.0085, 0.00919, 0.00991, 0.0107, 0.0116
	Hispanic	Women	71	0.00937, 0.0101, 0.0109, 0.0118, 0.0127
	Hispanic	Women	72	0.0103, 0.0112, 0.012, 0.013, 0.014
	Hispanic	Women	73	0.0114, 0.0123, 0.0133, 0.0143, 0.0155
	Hispanic	Women	74	0.0127, 0.0137, 0.0147, 0.0158, 0.017
	Hispanic	Women	75	0.0142, 0.0152, 0.0163, 0.0175, 0.0188
	Hispanic	Women	76	0.0159, 0.017, 0.0182, 0.0194, 0.0207
	Hispanic	Women	77	0.0179, 0.0191, 0.0203, 0.0216, 0.023
	Hispanic	Women	78	0.0201, 0.0214, 0.0228, 0.0242, 0.0258
	Hispanic	Women	79	0.0227, 0.0241, 0.0257, 0.0273, 0.0291
	Hispanic	Women	80	0.0255, 0.0272, 0.0289, 0.0308, 0.0329
	Hispanic	Women	81	0.0285, 0.0305, 0.0326, 0.0349, 0.0373
	Hispanic	Women	82	0.0318, 0.0341, 0.0366, 0.0393, 0.0422
	Hispanic	Women	83	0.035, 0.0378, 0.0408, 0.0441, 0.0476
	Hispanic	Women	84	0.0383, 0.0416, 0.0451, 0.049, 0.0532
	White	Men	30	0.00122, 0.0014, 0.00161, 0.00185, 0.00214
	White	Men	31	0.00127, 0.00145, 0.00165, 0.00189, 0.00216
	White	Men	32	0.00132, 0.00149, 0.00169, 0.00192, 0.00218
	White	Men	33	0.00135, 0.00153, 0.00172, 0.00195, 0.0022
	White	Men	34	0.00138, 0.00156, 0.00175, 0.00198, 0.00223

Variable	Race/ethnicity	Sex	Age	Distribution
	White	Men	35	0.0014, 0.00158, 0.00179, 0.00202, 0.00228
	White	Men	36	0.00143, 0.00161, 0.00183, 0.00206, 0.00234
	White	Men	37	0.00146, 0.00166, 0.00187, 0.00212, 0.00241
	White	Men	38	0.00151, 0.00171, 0.00194, 0.00219, 0.00249
	White	Men	39	0.00157, 0.00178, 0.00202, 0.00228, 0.00258
	White	Men	40	0.00166, 0.00187, 0.00211, 0.00238, 0.00269
	White	Men	41	0.00176, 0.00198, 0.00223, 0.00251, 0.00283
	White	Men	42	0.00189, 0.00212, 0.00238, 0.00267, 0.00299
	White	Men	43	0.00206, 0.0023, 0.00256, 0.00285, 0.00318
	White	Men	44	0.00226, 0.00251, 0.00278, 0.00307, 0.00341
	White	Men	45	0.00249, 0.00275, 0.00304, 0.00335, 0.0037
	White	Men	46	0.00272, 0.00302, 0.00334, 0.00369, 0.00409
	White	Men	47	0.00298, 0.00331, 0.00368, 0.00409, 0.00455
	White	Men	48	0.00327, 0.00365, 0.00407, 0.00454, 0.00506
	White	Men	49	0.00361, 0.00403, 0.00449, 0.00501, 0.0056
	White	Men	50	0.004, 0.00445, 0.00495, 0.00551, 0.00614
	White	Men	51	0.00444, 0.00492, 0.00544, 0.00602, 0.00666
	White	Men	52	0.00495, 0.00543, 0.00595, 0.00653, 0.00716
	White	Men	53	0.0055, 0.00597, 0.00649, 0.00704, 0.00766
	White	Men	54	0.00605, 0.00653, 0.00704, 0.00759, 0.00819
	White	Men	55	0.00656, 0.00707, 0.0076, 0.00818, 0.0088
	White	Men	56	0.00704, 0.00759, 0.00818, 0.00881, 0.0095
	White	Men	57	0.00752, 0.00812, 0.00877, 0.00947, 0.0102
	White	Men	58	0.00803, 0.00868, 0.00937, 0.0101, 0.0109
	White	Men	59	0.00858, 0.00926, 0.01, 0.0108, 0.0116
	White	Men	60	0.00918, 0.00989, 0.0106, 0.0114, 0.0123
	White	Men	61	0.00984, 0.0106, 0.0113, 0.0121, 0.013
	White	Men	62	0.0106, 0.0113, 0.012, 0.0128, 0.0137
	White	Men	63	0.0113, 0.012, 0.0128, 0.0136, 0.0144
	White	Men	64	0.012, 0.0128, 0.0136, 0.0144, 0.0153
	White	Men	65	0.0128, 0.0136, 0.0145, 0.0154, 0.0163
	White	Men	66	0.0137, 0.0146, 0.0155, 0.0165, 0.0176
	White	Men	67	0.0147, 0.0157, 0.0167, 0.0178, 0.019
	White	Men	68	0.0158, 0.0169, 0.0181, 0.0193, 0.0207
	White	Men	69	0.0171, 0.0184, 0.0197, 0.021, 0.0226
	White	Men	70	0.0186, 0.02, 0.0214, 0.023, 0.0246
	White	Men	71	0.0204, 0.0218, 0.0234, 0.0251, 0.0269
	White	Men	72	0.0223, 0.0239, 0.0256, 0.0274, 0.0294
	White	Men	73	0.0244, 0.0261, 0.028, 0.0299, 0.0321
	White	Men	74	0.0267, 0.0286, 0.0306, 0.0327, 0.0349
	White	Men	75	0.0294, 0.0314, 0.0335, 0.0357, 0.0381
	White	Men	76	0.0324, 0.0345, 0.0367, 0.039, 0.0416
	White	Men	77	0.0358, 0.0381, 0.0405, 0.043, 0.0457

Variable	Race/ethnicity	Sex	Age	Distribution
	White	Men	78	0.0397, 0.0422, 0.0448, 0.0476, 0.0506
	White	Men	79	0.0441, 0.0469, 0.0499, 0.0531, 0.0565
	White	Men	80	0.0491, 0.0523, 0.0557, 0.0593, 0.0632
	White	Men	81	0.0546, 0.0583, 0.0622, 0.0664, 0.0709
	White	Men	82	0.0605, 0.0648, 0.0693, 0.0741, 0.0793
	White	Men	83	0.0666, 0.0715, 0.0767, 0.0823, 0.0884
	White	Men	84	0.0726, 0.0782, 0.0841, 0.0906, 0.0976
	White	Women	30	0.000609, 0.000678, 0.000754, 0.000839, 0.000935
	White	Women	31	0.000641, 0.000716, 0.000799, 0.000891, 0.000995
	White	Women	32	0.000678, 0.000756, 0.000843, 0.00094, 0.00105
	White	Women	33	0.000716, 0.000798, 0.000888, 0.000988, 0.0011
	White	Women	34	0.00075, 0.000837, 0.000933, 0.00104, 0.00116
	White	Women	35	0.000778, 0.000874, 0.000982, 0.0011, 0.00124
	White	Women	36	0.000807, 0.000914, 0.00103, 0.00117, 0.00133
	White	Women	37	0.000842, 0.00096, 0.00109, 0.00124, 0.00142
	White	Women	38	0.000882, 0.00101, 0.00116, 0.00133, 0.00152
	White	Women	39	0.000929, 0.00107, 0.00123, 0.00142, 0.00164
	White	Women	40	0.000987, 0.00114, 0.00132, 0.00152, 0.00176
	White	Women	41	0.00106, 0.00123, 0.00142, 0.00164, 0.0019
	White	Women	42	0.00115, 0.00133, 0.00153, 0.00177, 0.00204
	White	Women	43	0.00125, 0.00144, 0.00166, 0.00191, 0.00221
	White	Women	44	0.00137, 0.00158, 0.00181, 0.00209, 0.0024
	White	Women	45	0.0015, 0.00173, 0.00199, 0.00229, 0.00263
	White	Women	46	0.00165, 0.0019, 0.00218, 0.00251, 0.00289
	White	Women	47	0.00182, 0.00209, 0.0024, 0.00274, 0.00315
	White	Women	48	0.00203, 0.00231, 0.00263, 0.00299, 0.0034
	White	Women	49	0.00227, 0.00256, 0.00288, 0.00325, 0.00367
	White	Women	50	0.00249, 0.00281, 0.00315, 0.00354, 0.00398
	White	Women	51	0.00271, 0.00305, 0.00344, 0.00387, 0.00436
	White	Women	52	0.00291, 0.0033, 0.00373, 0.00422, 0.00478
	White	Women	53	0.00312, 0.00355, 0.00403, 0.00457, 0.0052
	White	Women	54	0.00337, 0.00382, 0.00433, 0.0049, 0.00556
	White	Women	55	0.00368, 0.00413, 0.00463, 0.00518, 0.00581
	White	Women	56	0.00405, 0.00447, 0.00492, 0.00542, 0.00597
	White	Women	57	0.00446, 0.00483, 0.00522, 0.00564, 0.0061
	White	Women	58	0.00487, 0.00519, 0.00552, 0.00588, 0.00627
	White	Women	59	0.00522, 0.00553, 0.00586, 0.0062, 0.00657
	White	Women	60	0.00555, 0.00588, 0.00623, 0.0066, 0.00699
	White	Women	61	0.0059, 0.00627, 0.00665, 0.00706, 0.00751
	White	Women	62	0.0063, 0.00671, 0.00715, 0.00761, 0.0081
	White	Women	63	0.00678, 0.00724, 0.00772, 0.00823, 0.00878
	White	Women	64	0.00734, 0.00784, 0.00838, 0.00894, 0.00955
	White	Women	65	0.00798, 0.00854, 0.00913, 0.00976, 0.0104

Variable	Race/ethnicity	Sex	Age	Distribution
	White	Women	66	0.00869, 0.00932, 0.00998, 0.0107, 0.0115
	White	Women	67	0.00948, 0.0102, 0.0109, 0.0118, 0.0126
	White	Women	68	0.0103, 0.0111, 0.012, 0.013, 0.014
	White	Women	69	0.0113, 0.0122, 0.0132, 0.0143, 0.0155
	White	Women	70	0.0123, 0.0134, 0.0145, 0.0158, 0.0172
	White	Women	71	0.0134, 0.0147, 0.016, 0.0174, 0.019
	White	Women	72	0.0147, 0.0161, 0.0176, 0.0192, 0.021
	White	Women	73	0.0162, 0.0177, 0.0194, 0.0211, 0.0231
	White	Women	74	0.0179, 0.0196, 0.0213, 0.0232, 0.0253
	White	Women	75	0.02, 0.0217, 0.0235, 0.0255, 0.0277
	White	Women	76	0.0223, 0.0241, 0.026, 0.0281, 0.0304
	White	Women	77	0.0251, 0.0269, 0.0289, 0.0311, 0.0334
	White	Women	78	0.0282, 0.0302, 0.0323, 0.0346, 0.037
	White	Women	79	0.0316, 0.0338, 0.0362, 0.0387, 0.0414
	White	Women	80	0.0355, 0.038, 0.0406, 0.0434, 0.0464
	White	Women	81	0.0398, 0.0426, 0.0456, 0.0487, 0.0522
	White	Women	82	0.0443, 0.0475, 0.051, 0.0546, 0.0586
	White	Women	83	0.049, 0.0527, 0.0567, 0.061, 0.0656
	White	Women	84	0.0538, 0.0581, 0.0626, 0.0676, 0.0729
Total mortality rates for 2021 (0.01, 0.2, 0.5, 0.8. 0.99 percentiles of the empirical distribution produced during forecasting)				
	Black	Men	30	0.00173, 0.002, 0.00231, 0.00266, 0.00308
	Black	Men	31	0.00181, 0.00206, 0.00235, 0.00267, 0.00304
	Black	Men	32	0.00188, 0.00212, 0.00239, 0.00269, 0.00304
	Black	Men	33	0.00193, 0.00217, 0.00244, 0.00274, 0.00308
	Black	Men	34	0.00197, 0.00222, 0.0025, 0.00281, 0.00317
	Black	Men	35	0.00202, 0.00228, 0.00257, 0.0029, 0.00327
	Black	Men	36	0.00208, 0.00235, 0.00265, 0.00299, 0.00337
	Black	Men	37	0.00215, 0.00243, 0.00273, 0.00308, 0.00347
	Black	Men	38	0.00222, 0.0025, 0.00282, 0.00318, 0.00358
	Black	Men	39	0.00227, 0.00257, 0.00291, 0.00329, 0.00373
	Black	Men	40	0.00233, 0.00265, 0.00302, 0.00344, 0.00392
	Black	Men	41	0.00239, 0.00275, 0.00316, 0.00363, 0.00418
	Black	Men	42	0.00247, 0.00287, 0.00333, 0.00387, 0.00449
	Black	Men	43	0.00259, 0.00304, 0.00355, 0.00416, 0.00488
	Black	Men	44	0.00274, 0.00324, 0.00382, 0.00452, 0.00534
	Black	Men	45	0.00292, 0.00348, 0.00414, 0.00493, 0.00588
	Black	Men	46	0.00316, 0.00378, 0.00452, 0.0054, 0.00647
	Black	Men	47	0.00346, 0.00414, 0.00495, 0.00591, 0.00707
	Black	Men	48	0.00384, 0.00457, 0.00544, 0.00646, 0.00769
	Black	Men	49	0.0043, 0.00508, 0.00599, 0.00706, 0.00834
	Black	Men	50	0.00484, 0.00566, 0.00661, 0.00772, 0.00903
	Black	Men	51	0.00545, 0.00632, 0.0073, 0.00845, 0.00979
	Black	Men	52	0.00615, 0.00705, 0.00807, 0.00923, 0.0106

Variable	Race/ethnicity	Sex	Age	Distribution
	Black	Men	53	0.00695, 0.00787, 0.00889, 0.0101, 0.0114
	Black	Men	54	0.00784, 0.00877, 0.00979, 0.0109, 0.0122
	Black	Men	55	0.0088, 0.00973, 0.0108, 0.0119, 0.0131
	Black	Men	56	0.00984, 0.0108, 0.0118, 0.0129, 0.0141
	Black	Men	57	0.011, 0.0119, 0.0129, 0.014, 0.0152
	Black	Men	58	0.0122, 0.0131, 0.0141, 0.0151, 0.0163
	Black	Men	59	0.0134, 0.0143, 0.0153, 0.0163, 0.0175
	Black	Men	60	0.0145, 0.0155, 0.0165, 0.0176, 0.0188
	Black	Men	61	0.0156, 0.0167, 0.0178, 0.0189, 0.0202
	Black	Men	62	0.0168, 0.0178, 0.019, 0.0201, 0.0214
	Black	Men	63	0.0178, 0.0189, 0.0201, 0.0214, 0.0227
	Black	Men	64	0.0187, 0.02, 0.0212, 0.0226, 0.024
	Black	Men	65	0.0197, 0.021, 0.0224, 0.0238, 0.0254
	Black	Men	66	0.0207, 0.0221, 0.0236, 0.0251, 0.0268
	Black	Men	67	0.0218, 0.0233, 0.0249, 0.0266, 0.0285
	Black	Men	68	0.023, 0.0246, 0.0264, 0.0283, 0.0304
	Black	Men	69	0.0243, 0.0262, 0.0282, 0.0303, 0.0326
	Black	Men	70	0.0259, 0.0279, 0.0301, 0.0325, 0.035
	Black	Men	71	0.0277, 0.0299, 0.0323, 0.0349, 0.0377
	Black	Men	72	0.0297, 0.0321, 0.0347, 0.0376, 0.0407
	Black	Men	73	0.0319, 0.0345, 0.0374, 0.0404, 0.0437
	Black	Men	74	0.0345, 0.0372, 0.0402, 0.0433, 0.0468
	Black	Men	75	0.0374, 0.0402, 0.0432, 0.0464, 0.0499
	Black	Men	76	0.0406, 0.0434, 0.0465, 0.0498, 0.0533
	Black	Men	77	0.044, 0.047, 0.0502, 0.0536, 0.0572
	Black	Men	78	0.0476, 0.0508, 0.0542, 0.0579, 0.0619
	Black	Men	79	0.0514, 0.055, 0.0588, 0.0629, 0.0673
	Black	Men	80	0.0555, 0.0595, 0.0638, 0.0684, 0.0734
	Black	Men	81	0.0598, 0.0644, 0.0692, 0.0744, 0.08
	Black	Men	82	0.0643, 0.0694, 0.0748, 0.0806, 0.087
	Black	Men	83	0.0686, 0.0743, 0.0805, 0.0871, 0.0943
	Black	Men	84	0.0728, 0.0791, 0.086, 0.0934, 0.101
	Black	Women	30	0.000641, 0.000764, 0.000909, 0.00108, 0.00129
	Black	Women	31	0.000712, 0.000836, 0.000979, 0.00115, 0.00135
	Black	Women	32	0.000794, 0.000917, 0.00106, 0.00122, 0.00141
	Black	Women	33	0.000879, 0.001, 0.00114, 0.0013, 0.00148
	Black	Women	34	0.000955, 0.00108, 0.00122, 0.00139, 0.00157
	Black	Women	35	0.00102, 0.00115, 0.00131, 0.00148, 0.00168
	Black	Women	36	0.00107, 0.00122, 0.00139, 0.00158, 0.0018
	Black	Women	37	0.00113, 0.00129, 0.00147, 0.00168, 0.00191
	Black	Women	38	0.0012, 0.00137, 0.00156, 0.00178, 0.00204
	Black	Women	39	0.00128, 0.00146, 0.00167, 0.0019, 0.00217
	Black	Women	40	0.00138, 0.00157, 0.00179, 0.00204, 0.00232

Variable	Race/ethnicity	Sex	Age	Distribution
	Black	Women	41	0.0015, 0.00171, 0.00193, 0.00219, 0.00249
	Black	Women	42	0.00166, 0.00187, 0.0021, 0.00236, 0.00265
	Black	Women	43	0.00185, 0.00206, 0.00229, 0.00254, 0.00283
	Black	Women	44	0.00207, 0.00227, 0.0025, 0.00274, 0.00301
	Black	Women	45	0.0023, 0.00251, 0.00273, 0.00298, 0.00325
	Black	Women	46	0.00252, 0.00275, 0.003, 0.00327, 0.00357
	Black	Women	47	0.00275, 0.00301, 0.0033, 0.00361, 0.00395
	Black	Women	48	0.003, 0.0033, 0.00363, 0.00399, 0.00439
	Black	Women	49	0.0033, 0.00363, 0.004, 0.0044, 0.00484
	Black	Women	50	0.00366, 0.00401, 0.0044, 0.00482, 0.00528
	Black	Women	51	0.0041, 0.00445, 0.00483, 0.00524, 0.00569
	Black	Women	52	0.0046, 0.00493, 0.00529, 0.00567, 0.00608
	Black	Women	53	0.00512, 0.00544, 0.00578, 0.00613, 0.00652
	Black	Women	54	0.00562, 0.00595, 0.00629, 0.00666, 0.00705
	Black	Women	55	0.00609, 0.00645, 0.00683, 0.00723, 0.00766
	Black	Women	56	0.00657, 0.00697, 0.00739, 0.00784, 0.00831
	Black	Women	57	0.00706, 0.0075, 0.00796, 0.00845, 0.00897
	Black	Women	58	0.00756, 0.00803, 0.00853, 0.00906, 0.00963
	Black	Women	59	0.00803, 0.00855, 0.00909, 0.00968, 0.0103
	Black	Women	60	0.00849, 0.00905, 0.00965, 0.0103, 0.011
	Black	Women	61	0.00896, 0.00957, 0.0102, 0.0109, 0.0116
	Black	Women	62	0.00946, 0.0101, 0.0108, 0.0115, 0.0123
	Black	Women	63	0.00998, 0.0107, 0.0114, 0.0122, 0.013
	Black	Women	64	0.0105, 0.0113, 0.0121, 0.0129, 0.0138
	Black	Women	65	0.0112, 0.012, 0.0128, 0.0138, 0.0148
	Black	Women	66	0.0119, 0.0128, 0.0137, 0.0148, 0.0159
	Black	Women	67	0.0127, 0.0137, 0.0148, 0.0159, 0.0171
	Black	Women	68	0.0137, 0.0148, 0.0159, 0.0171, 0.0185
	Black	Women	69	0.0148, 0.016, 0.0172, 0.0185, 0.02
	Black	Women	70	0.016, 0.0173, 0.0186, 0.0201, 0.0216
	Black	Women	71	0.0173, 0.0187, 0.0201, 0.0217, 0.0234
	Black	Women	72	0.0187, 0.0202, 0.0218, 0.0235, 0.0253
	Black	Women	73	0.0203, 0.0219, 0.0236, 0.0254, 0.0274
	Black	Women	74	0.0221, 0.0237, 0.0255, 0.0275, 0.0296
	Black	Women	75	0.0241, 0.0258, 0.0277, 0.0297, 0.0319
	Black	Women	76	0.0263, 0.0282, 0.0302, 0.0323, 0.0346
	Black	Women	77	0.0289, 0.0309, 0.033, 0.0353, 0.0377
	Black	Women	78	0.0317, 0.0339, 0.0362, 0.0387, 0.0413
	Black	Women	79	0.0348, 0.0372, 0.0398, 0.0425, 0.0455
	Black	Women	80	0.0382, 0.0409, 0.0438, 0.0469, 0.0502
	Black	Women	81	0.0417, 0.0448, 0.0481, 0.0517, 0.0555
	Black	Women	82	0.0453, 0.0489, 0.0527, 0.0569, 0.0614
	Black	Women	83	0.0488, 0.053, 0.0575, 0.0624, 0.0677

Variable	Race/ethnicity	Sex	Age	Distribution
	Black	Women	84	0.0524, 0.0571, 0.0623, 0.0679, 0.0741
	Hispanic	Men	30	0.000801, 0.000898, 0.00101, 0.00113, 0.00126
	Hispanic	Men	31	0.000817, 0.000915, 0.00102, 0.00115, 0.00128
	Hispanic	Men	32	0.000829, 0.000931, 0.00104, 0.00117, 0.00131
	Hispanic	Men	33	0.00083, 0.000939, 0.00106, 0.0012, 0.00136
	Hispanic	Men	34	0.000826, 0.000946, 0.00108, 0.00123, 0.00141
	Hispanic	Men	35	0.00083, 0.000957, 0.0011, 0.00127, 0.00147
	Hispanic	Men	36	0.000847, 0.000979, 0.00113, 0.00131, 0.00151
	Hispanic	Men	37	0.00088, 0.00101, 0.00117, 0.00135, 0.00155
	Hispanic	Men	38	0.000925, 0.00106, 0.00122, 0.00139, 0.0016
	Hispanic	Men	39	0.000978, 0.00112, 0.00128, 0.00146, 0.00166
	Hispanic	Men	40	0.00104, 0.00118, 0.00135, 0.00154, 0.00175
	Hispanic	Men	41	0.00111, 0.00126, 0.00144, 0.00164, 0.00187
	Hispanic	Men	42	0.0012, 0.00136, 0.00155, 0.00175, 0.00199
	Hispanic	Men	43	0.00133, 0.00149, 0.00168, 0.00188, 0.00212
	Hispanic	Men	44	0.00149, 0.00166, 0.00183, 0.00203, 0.00225
	Hispanic	Men	45	0.00169, 0.00185, 0.00202, 0.00221, 0.00243
	Hispanic	Men	46	0.00188, 0.00206, 0.00224, 0.00244, 0.00266
	Hispanic	Men	47	0.00209, 0.00228, 0.00249, 0.00271, 0.00296
	Hispanic	Men	48	0.00231, 0.00253, 0.00277, 0.00302, 0.00331
	Hispanic	Men	49	0.00256, 0.0028, 0.00306, 0.00336, 0.00368
	Hispanic	Men	50	0.00284, 0.0031, 0.00339, 0.0037, 0.00404
	Hispanic	Men	51	0.00318, 0.00344, 0.00372, 0.00403, 0.00437
	Hispanic	Men	52	0.00359, 0.00383, 0.00408, 0.00435, 0.00464
	Hispanic	Men	53	0.00406, 0.00425, 0.00445, 0.00466, 0.00488
	Hispanic	Men	54	0.0045, 0.00467, 0.00484, 0.00503, 0.00522
	Hispanic	Men	55	0.00483, 0.00504, 0.00526, 0.00549, 0.00572
	Hispanic	Men	56	0.00514, 0.00541, 0.00569, 0.00599, 0.00631
	Hispanic	Men	57	0.00548, 0.00581, 0.00615, 0.00652, 0.00691
	Hispanic	Men	58	0.00585, 0.00623, 0.00664, 0.00707, 0.00753
	Hispanic	Men	59	0.00624, 0.00668, 0.00714, 0.00763, 0.00817
	Hispanic	Men	60	0.00665, 0.00714, 0.00766, 0.00821, 0.00882
	Hispanic	Men	61	0.00711, 0.00764, 0.00819, 0.00879, 0.00944
	Hispanic	Men	62	0.00764, 0.00817, 0.00875, 0.00936, 0.01
	Hispanic	Men	63	0.00819, 0.00874, 0.00932, 0.00995, 0.0106
	Hispanic	Men	64	0.00874, 0.00933, 0.00994, 0.0106, 0.0113
	Hispanic	Men	65	0.0093, 0.00994, 0.0106, 0.0113, 0.0121
	Hispanic	Men	66	0.00989, 0.0106, 0.0114, 0.0122, 0.0131
	Hispanic	Men	67	0.0106, 0.0114, 0.0122, 0.0132, 0.0142
	Hispanic	Men	68	0.0113, 0.0122, 0.0132, 0.0142, 0.0154
	Hispanic	Men	69	0.0122, 0.0132, 0.0143, 0.0155, 0.0167
	Hispanic	Men	70	0.0133, 0.0144, 0.0156, 0.0169, 0.0183
	Hispanic	Men	71	0.0144, 0.0156, 0.0169, 0.0184, 0.0199

Variable	Race/ethnicity	Sex	Age	Distribution
	Hispanic	Men	72	0.0157, 0.017, 0.0185, 0.0201, 0.0218
	Hispanic	Men	73	0.0171, 0.0186, 0.0202, 0.0219, 0.0238
	Hispanic	Men	74	0.0187, 0.0203, 0.022, 0.0239, 0.026
	Hispanic	Men	75	0.0206, 0.0223, 0.0241, 0.0262, 0.0284
	Hispanic	Men	76	0.0227, 0.0245, 0.0265, 0.0287, 0.0311
	Hispanic	Men	77	0.0251, 0.0271, 0.0293, 0.0317, 0.0342
	Hispanic	Men	78	0.0278, 0.0301, 0.0325, 0.0351, 0.0379
	Hispanic	Men	79	0.031, 0.0335, 0.0362, 0.0391, 0.0422
	Hispanic	Men	80	0.0346, 0.0374, 0.0403, 0.0436, 0.0471
	Hispanic	Men	81	0.0386, 0.0417, 0.045, 0.0485, 0.0524
	Hispanic	Men	82	0.0428, 0.0462, 0.0499, 0.054, 0.0583
	Hispanic	Men	83	0.0469, 0.0508, 0.055, 0.0596, 0.0646
	Hispanic	Men	84	0.0508, 0.0553, 0.0601, 0.0653, 0.0711
	Hispanic	Women	30	0.000328, 0.00037, 0.000416, 0.000469, 0.000528
	Hispanic	Women	31	0.000347, 0.00039, 0.000438, 0.000491, 0.000552
	Hispanic	Women	32	0.000368, 0.000411, 0.00046, 0.000514, 0.000576
	Hispanic	Women	33	0.000389, 0.000433, 0.000483, 0.000538, 0.0006
	Hispanic	Women	34	0.000407, 0.000455, 0.000507, 0.000565, 0.000631
	Hispanic	Women	35	0.000424, 0.000475, 0.000532, 0.000597, 0.000669
	Hispanic	Women	36	0.000441, 0.000497, 0.000561, 0.000632, 0.000713
	Hispanic	Women	37	0.000462, 0.000524, 0.000593, 0.000671, 0.00076
	Hispanic	Women	38	0.00049, 0.000555, 0.000629, 0.000713, 0.000808
	Hispanic	Women	39	0.000525, 0.000594, 0.000671, 0.000758, 0.000857
	Hispanic	Women	40	0.00057, 0.000641, 0.000719, 0.000808, 0.000908
	Hispanic	Women	41	0.000624, 0.000696, 0.000776, 0.000864, 0.000964
	Hispanic	Women	42	0.000688, 0.000761, 0.000841, 0.00093, 0.00103
	Hispanic	Women	43	0.000763, 0.000837, 0.000918, 0.00101, 0.0011
	Hispanic	Women	44	0.000849, 0.000925, 0.00101, 0.0011, 0.00119
	Hispanic	Women	45	0.000944, 0.00102, 0.00111, 0.0012, 0.0013
	Hispanic	Women	46	0.00105, 0.00113, 0.00123, 0.00133, 0.00144
	Hispanic	Women	47	0.00116, 0.00125, 0.00136, 0.00147, 0.00159
	Hispanic	Women	48	0.00128, 0.00138, 0.0015, 0.00162, 0.00176
	Hispanic	Women	49	0.0014, 0.00152, 0.00165, 0.00179, 0.00195
	Hispanic	Women	50	0.00155, 0.00168, 0.00182, 0.00198, 0.00215
	Hispanic	Women	51	0.00171, 0.00185, 0.002, 0.00217, 0.00235
	Hispanic	Women	52	0.00189, 0.00204, 0.00219, 0.00236, 0.00255
	Hispanic	Women	53	0.00208, 0.00223, 0.0024, 0.00258, 0.00277
	Hispanic	Women	54	0.00227, 0.00244, 0.00262, 0.00281, 0.00301
	Hispanic	Women	55	0.00246, 0.00265, 0.00285, 0.00306, 0.00329
	Hispanic	Women	56	0.00267, 0.00287, 0.00309, 0.00333, 0.00358
	Hispanic	Women	57	0.00289, 0.00312, 0.00335, 0.0036, 0.00388
	Hispanic	Women	58	0.00314, 0.00338, 0.00363, 0.0039, 0.00419
	Hispanic	Women	59	0.00339, 0.00365, 0.00392, 0.00422, 0.00454

Variable	Race/ethnicity	Sex	Age	Distribution
	Hispanic	Women	60	0.00366, 0.00394, 0.00424, 0.00457, 0.00492
	Hispanic	Women	61	0.00394, 0.00425, 0.00458, 0.00494, 0.00533
	Hispanic	Women	62	0.00425, 0.00459, 0.00495, 0.00534, 0.00577
	Hispanic	Women	63	0.00459, 0.00496, 0.00535, 0.00578, 0.00624
	Hispanic	Women	64	0.00495, 0.00535, 0.00579, 0.00626, 0.00678
	Hispanic	Women	65	0.00534, 0.00579, 0.00627, 0.0068, 0.00738
	Hispanic	Women	66	0.00578, 0.00628, 0.00681, 0.0074, 0.00804
	Hispanic	Women	67	0.00628, 0.00683, 0.00742, 0.00806, 0.00876
	Hispanic	Women	68	0.00687, 0.00747, 0.00811, 0.0088, 0.00956
	Hispanic	Women	69	0.00755, 0.00819, 0.00888, 0.00963, 0.0105
	Hispanic	Women	70	0.00831, 0.00901, 0.00976, 0.0106, 0.0115
	Hispanic	Women	71	0.00916, 0.00992, 0.0107, 0.0116, 0.0126
	Hispanic	Women	72	0.0101, 0.0109, 0.0118, 0.0128, 0.0139
	Hispanic	Women	73	0.0112, 0.0121, 0.0131, 0.0141, 0.0153
	Hispanic	Women	74	0.0124, 0.0134, 0.0145, 0.0156, 0.0169
	Hispanic	Women	75	0.0139, 0.0149, 0.016, 0.0173, 0.0186
	Hispanic	Women	76	0.0155, 0.0167, 0.0179, 0.0191, 0.0205
	Hispanic	Women	77	0.0175, 0.0187, 0.02, 0.0213, 0.0228
	Hispanic	Women	78	0.0197, 0.021, 0.0224, 0.0239, 0.0256
	Hispanic	Women	79	0.0222, 0.0237, 0.0253, 0.027, 0.0288
	Hispanic	Women	80	0.0249, 0.0267, 0.0285, 0.0305, 0.0326
	Hispanic	Women	81	0.0279, 0.03, 0.0321, 0.0345, 0.037
	Hispanic	Women	82	0.0311, 0.0335, 0.0361, 0.0389, 0.0419
	Hispanic	Women	83	0.0343, 0.0372, 0.0403, 0.0436, 0.0472
	Hispanic	Women	84	0.0375, 0.0409, 0.0445, 0.0484, 0.0528
	White	Men	30	0.00119, 0.00138, 0.00161, 0.00187, 0.00218
	White	Men	31	0.00124, 0.00143, 0.00165, 0.00191, 0.0022
	White	Men	32	0.00129, 0.00148, 0.00169, 0.00193, 0.00221
	White	Men	33	0.00133, 0.00151, 0.00172, 0.00196, 0.00223
	White	Men	34	0.00135, 0.00154, 0.00175, 0.00199, 0.00226
	White	Men	35	0.00137, 0.00156, 0.00178, 0.00202, 0.0023
	White	Men	36	0.00139, 0.00159, 0.00181, 0.00207, 0.00236
	White	Men	37	0.00142, 0.00163, 0.00186, 0.00212, 0.00243
	White	Men	38	0.00147, 0.00168, 0.00192, 0.00219, 0.00251
	White	Men	39	0.00153, 0.00175, 0.00199, 0.00227, 0.00259
	White	Men	40	0.00161, 0.00183, 0.00208, 0.00237, 0.0027
	White	Men	41	0.00171, 0.00194, 0.0022, 0.00249, 0.00283
	White	Men	42	0.00183, 0.00207, 0.00234, 0.00264, 0.00299
	White	Men	43	0.002, 0.00224, 0.00252, 0.00282, 0.00317
	White	Men	44	0.00219, 0.00245, 0.00273, 0.00304, 0.0034
	White	Men	45	0.00241, 0.00269, 0.00299, 0.00332, 0.0037
	White	Men	46	0.00264, 0.00295, 0.00329, 0.00366, 0.00409
	White	Men	47	0.00289, 0.00324, 0.00363, 0.00406, 0.00456

Variable	Race/ethnicity	Sex	Age	Distribution
	White	Men	48	0.00318, 0.00357, 0.00402, 0.00451, 0.00508
	White	Men	49	0.00351, 0.00395, 0.00444, 0.00499, 0.00562
	White	Men	50	0.00389, 0.00437, 0.0049, 0.00549, 0.00617
	White	Men	51	0.00433, 0.00483, 0.00539, 0.006, 0.0067
	White	Men	52	0.00483, 0.00534, 0.0059, 0.00652, 0.0072
	White	Men	53	0.00538, 0.00588, 0.00643, 0.00703, 0.0077
	White	Men	54	0.00593, 0.00644, 0.00698, 0.00758, 0.00823
	White	Men	55	0.00644, 0.00697, 0.00754, 0.00816, 0.00884
	White	Men	56	0.00691, 0.00749, 0.00812, 0.0088, 0.00954
	White	Men	57	0.00737, 0.00801, 0.0087, 0.00945, 0.0103
	White	Men	58	0.00786, 0.00855, 0.0093, 0.0101, 0.011
	White	Men	59	0.0084, 0.00913, 0.00991, 0.0108, 0.0117
	White	Men	60	0.00899, 0.00974, 0.0105, 0.0114, 0.0123
	White	Men	61	0.00964, 0.0104, 0.0112, 0.012, 0.013
	White	Men	62	0.0103, 0.0111, 0.0119, 0.0127, 0.0136
	White	Men	63	0.011, 0.0118, 0.0126, 0.0134, 0.0143
	White	Men	64	0.0118, 0.0126, 0.0134, 0.0142, 0.0152
	White	Men	65	0.0125, 0.0134, 0.0143, 0.0152, 0.0162
	White	Men	66	0.0134, 0.0143, 0.0153, 0.0163, 0.0174
	White	Men	67	0.0143, 0.0154, 0.0164, 0.0176, 0.0189
	White	Men	68	0.0154, 0.0166, 0.0178, 0.0191, 0.0205
	White	Men	69	0.0167, 0.018, 0.0194, 0.0208, 0.0224
	White	Men	70	0.0182, 0.0196, 0.0211, 0.0227, 0.0245
	White	Men	71	0.0199, 0.0214, 0.023, 0.0248, 0.0267
	White	Men	72	0.0217, 0.0234, 0.0252, 0.0271, 0.0292
	White	Men	73	0.0238, 0.0256, 0.0275, 0.0296, 0.0318
	White	Men	74	0.0261, 0.028, 0.0301, 0.0323, 0.0346
	White	Men	75	0.0287, 0.0307, 0.0329, 0.0352, 0.0377
	White	Men	76	0.0316, 0.0338, 0.0361, 0.0386, 0.0412
	White	Men	77	0.035, 0.0373, 0.0398, 0.0424, 0.0453
	White	Men	78	0.0387, 0.0413, 0.0441, 0.047, 0.0502
	White	Men	79	0.043, 0.046, 0.0491, 0.0524, 0.056
	White	Men	80	0.0479, 0.0513, 0.0548, 0.0586, 0.0627
	White	Men	81	0.0534, 0.0572, 0.0613, 0.0656, 0.0703
	White	Men	82	0.0592, 0.0636, 0.0683, 0.0733, 0.0788
	White	Men	83	0.0651, 0.0702, 0.0756, 0.0814, 0.0878
	White	Men	84	0.0709, 0.0767, 0.0829, 0.0896, 0.0969
	White	Women	30	0.000601, 0.000673, 0.000752, 0.000842, 0.000943
	White	Women	31	0.000633, 0.00071, 0.000797, 0.000894, 0.001
	White	Women	32	0.000668, 0.00075, 0.000841, 0.000943, 0.00106
	White	Women	33	0.000706, 0.000791, 0.000885, 0.000991, 0.00111
	White	Women	34	0.000737, 0.000828, 0.000929, 0.00104, 0.00117
	White	Women	35	0.000762, 0.000863, 0.000976, 0.0011, 0.00125

Variable	Race/ethnicity	Sex	Age	Distribution
	White	Women	36	0.000788, 0.0009, 0.00103, 0.00117, 0.00134
	White	Women	37	0.000819, 0.000942, 0.00108, 0.00124, 0.00143
	White	Women	38	0.000855, 0.00099, 0.00114, 0.00132, 0.00153
	White	Women	39	0.000898, 0.00105, 0.00122, 0.00141, 0.00165
	White	Women	40	0.000951, 0.00111, 0.0013, 0.00151, 0.00177
	White	Women	41	0.00102, 0.00119, 0.00139, 0.00163, 0.0019
	White	Women	42	0.0011, 0.00129, 0.0015, 0.00175, 0.00205
	White	Women	43	0.0012, 0.0014, 0.00163, 0.0019, 0.00221
	White	Women	44	0.00131, 0.00153, 0.00178, 0.00207, 0.00241
	White	Women	45	0.00144, 0.00168, 0.00195, 0.00227, 0.00264
	White	Women	46	0.00158, 0.00184, 0.00214, 0.00249, 0.0029
	White	Women	47	0.00176, 0.00204, 0.00236, 0.00272, 0.00316
	White	Women	48	0.00196, 0.00226, 0.00259, 0.00297, 0.00341
	White	Women	49	0.00219, 0.0025, 0.00284, 0.00323, 0.00368
	White	Women	50	0.00242, 0.00274, 0.00311, 0.00352, 0.004
	White	Women	51	0.00263, 0.00299, 0.00339, 0.00386, 0.00439
	White	Women	52	0.00282, 0.00323, 0.00369, 0.00421, 0.00482
	White	Women	53	0.00303, 0.00348, 0.00399, 0.00457, 0.00525
	White	Women	54	0.00328, 0.00375, 0.00429, 0.0049, 0.00561
	White	Women	55	0.00359, 0.00406, 0.00459, 0.00518, 0.00585
	White	Women	56	0.00397, 0.0044, 0.00488, 0.00541, 0.006
	White	Women	57	0.00439, 0.00477, 0.00517, 0.00562, 0.0061
	White	Women	58	0.00479, 0.00512, 0.00548, 0.00585, 0.00626
	White	Women	59	0.00514, 0.00547, 0.0058, 0.00617, 0.00655
	White	Women	60	0.00546, 0.0058, 0.00617, 0.00656, 0.00697
	White	Women	61	0.0058, 0.00618, 0.00658, 0.00701, 0.00748
	White	Women	62	0.00619, 0.00662, 0.00706, 0.00754, 0.00806
	White	Women	63	0.00666, 0.00713, 0.00762, 0.00815, 0.00872
	White	Women	64	0.0072, 0.00772, 0.00826, 0.00885, 0.00948
	White	Women	65	0.00782, 0.00839, 0.009, 0.00965, 0.0104
	White	Women	66	0.00851, 0.00915, 0.00983, 0.0106, 0.0114
	White	Women	67	0.00926, 0.00999, 0.0108, 0.0116, 0.0125
	White	Women	68	0.0101, 0.0109, 0.0118, 0.0128, 0.0139
	White	Women	69	0.011, 0.012, 0.013, 0.0142, 0.0154
	White	Women	70	0.012, 0.0131, 0.0143, 0.0156, 0.0171
	White	Women	71	0.0131, 0.0144, 0.0157, 0.0172, 0.0189
	White	Women	72	0.0144, 0.0158, 0.0173, 0.019, 0.0209
	White	Women	73	0.0158, 0.0173, 0.019, 0.0209, 0.0229
	White	Women	74	0.0175, 0.0191, 0.021, 0.0229, 0.0251
	White	Women	75	0.0194, 0.0212, 0.0231, 0.0252, 0.0275
	White	Women	76	0.0217, 0.0236, 0.0256, 0.0278, 0.0301
	White	Women	77	0.0244, 0.0264, 0.0284, 0.0307, 0.0331
	White	Women	78	0.0274, 0.0295, 0.0318, 0.0341, 0.0367

Variable	Race/ethnicity	Sex	Age	Distribution
	White	Women	79	0.0308, 0.0331, 0.0356, 0.0382, 0.0411
	White	Women	80	0.0346, 0.0372, 0.0399, 0.0429, 0.0461
	White	Women	81	0.0388, 0.0417, 0.0448, 0.0482, 0.0518
	White	Women	82	0.0432, 0.0466, 0.0502, 0.0541, 0.0583
	White	Women	83	0.0478, 0.0517, 0.0559, 0.0603, 0.0652
	White	Women	84	0.0525, 0.057, 0.0617, 0.0669, 0.0725
Total mortality rates for 2022 (0.01, 0.2, 0.5, 0.8. 0.99 percentiles of the empirical distribution produced during forecasting)				
	Black	Men	30	0.0017, 0.00198, 0.00231, 0.00268, 0.00313
	Black	Men	31	0.00179, 0.00205, 0.00235, 0.00269, 0.00309
	Black	Men	32	0.00186, 0.00211, 0.00239, 0.00271, 0.00307
	Black	Men	33	0.0019, 0.00215, 0.00244, 0.00275, 0.00311
	Black	Men	34	0.00194, 0.0022, 0.00249, 0.00282, 0.00319
	Black	Men	35	0.00199, 0.00226, 0.00256, 0.0029, 0.00329
	Black	Men	36	0.00205, 0.00232, 0.00263, 0.00299, 0.00339
	Black	Men	37	0.00211, 0.00239, 0.00271, 0.00307, 0.00348
	Black	Men	38	0.00217, 0.00246, 0.00279, 0.00316, 0.00359
	Black	Men	39	0.00222, 0.00253, 0.00287, 0.00327, 0.00373
	Black	Men	40	0.00226, 0.0026, 0.00298, 0.00341, 0.00392
	Black	Men	41	0.00232, 0.00269, 0.00311, 0.0036, 0.00417
	Black	Men	42	0.0024, 0.00281, 0.00328, 0.00383, 0.00449
	Black	Men	43	0.0025, 0.00296, 0.00349, 0.00413, 0.00488
	Black	Men	44	0.00264, 0.00315, 0.00376, 0.00448, 0.00535
	Black	Men	45	0.00282, 0.00339, 0.00408, 0.0049, 0.0059
	Black	Men	46	0.00304, 0.00368, 0.00445, 0.00537, 0.0065
	Black	Men	47	0.00333, 0.00403, 0.00487, 0.00589, 0.00713
	Black	Men	48	0.0037, 0.00446, 0.00536, 0.00645, 0.00777
	Black	Men	49	0.00415, 0.00496, 0.00592, 0.00705, 0.00843
	Black	Men	50	0.00468, 0.00554, 0.00654, 0.00772, 0.00914
	Black	Men	51	0.00528, 0.00618, 0.00723, 0.00846, 0.00991
	Black	Men	52	0.00597, 0.00691, 0.00799, 0.00924, 0.0107
	Black	Men	53	0.00677, 0.00773, 0.00882, 0.0101, 0.0115
	Black	Men	54	0.00765, 0.00863, 0.00972, 0.0109, 0.0123
	Black	Men	55	0.00862, 0.0096, 0.0107, 0.0119, 0.0132
	Black	Men	56	0.00965, 0.0106, 0.0117, 0.0129, 0.0142
	Black	Men	57	0.0108, 0.0118, 0.0128, 0.014, 0.0152
	Black	Men	58	0.012, 0.0129, 0.014, 0.0151, 0.0163
	Black	Men	59	0.0131, 0.0141, 0.0152, 0.0163, 0.0175
	Black	Men	60	0.0142, 0.0153, 0.0164, 0.0176, 0.0188
	Black	Men	61	0.0154, 0.0164, 0.0176, 0.0188, 0.0201
	Black	Men	62	0.0165, 0.0176, 0.0187, 0.02, 0.0213
	Black	Men	63	0.0175, 0.0186, 0.0198, 0.0212, 0.0226
	Black	Men	64	0.0184, 0.0196, 0.0209, 0.0223, 0.0239
	Black	Men	65	0.0193, 0.0206, 0.022, 0.0235, 0.0252

Variable	Race/ethnicity	Sex	Age	Distribution
	Black	Men	66	0.0203, 0.0217, 0.0232, 0.0248, 0.0266
	Black	Men	67	0.0213, 0.0229, 0.0245, 0.0263, 0.0282
	Black	Men	68	0.0225, 0.0242, 0.026, 0.028, 0.0301
	Black	Men	69	0.0238, 0.0257, 0.0277, 0.0299, 0.0323
	Black	Men	70	0.0253, 0.0274, 0.0297, 0.0321, 0.0347
	Black	Men	71	0.027, 0.0293, 0.0318, 0.0345, 0.0374
	Black	Men	72	0.029, 0.0315, 0.0342, 0.0371, 0.0403
	Black	Men	73	0.0312, 0.0339, 0.0367, 0.0399, 0.0433
	Black	Men	74	0.0337, 0.0365, 0.0395, 0.0428, 0.0463
	Black	Men	75	0.0365, 0.0394, 0.0425, 0.0458, 0.0495
	Black	Men	76	0.0396, 0.0426, 0.0457, 0.0491, 0.0528
	Black	Men	77	0.043, 0.0461, 0.0494, 0.0529, 0.0567
	Black	Men	78	0.0465, 0.0498, 0.0534, 0.0572, 0.0613
	Black	Men	79	0.0502, 0.0539, 0.0579, 0.0621, 0.0667
	Black	Men	80	0.0542, 0.0584, 0.0628, 0.0676, 0.0728
	Black	Men	81	0.0585, 0.0632, 0.0682, 0.0736, 0.0794
	Black	Men	82	0.0629, 0.0681, 0.0737, 0.0798, 0.0865
	Black	Men	83	0.0671, 0.073, 0.0793, 0.0862, 0.0938
	Black	Men	84	0.0712, 0.0777, 0.0848, 0.0925, 0.101
	Black	Women	30	0.000624, 0.000753, 0.000908, 0.00109, 0.00132
	Black	Women	31	0.000695, 0.000825, 0.000978, 0.00116, 0.00138
	Black	Women	32	0.000777, 0.000907, 0.00106, 0.00123, 0.00143
	Black	Women	33	0.000862, 0.000992, 0.00114, 0.00131, 0.0015
	Black	Women	34	0.000936, 0.00107, 0.00122, 0.00139, 0.00159
	Black	Women	35	0.000994, 0.00114, 0.0013, 0.00149, 0.0017
	Black	Women	36	0.00104, 0.0012, 0.00138, 0.00158, 0.00182
	Black	Women	37	0.0011, 0.00127, 0.00146, 0.00168, 0.00193
	Black	Women	38	0.00116, 0.00134, 0.00154, 0.00178, 0.00205
	Black	Women	39	0.00124, 0.00143, 0.00164, 0.00189, 0.00218
	Black	Women	40	0.00134, 0.00154, 0.00176, 0.00203, 0.00233
	Black	Women	41	0.00145, 0.00167, 0.0019, 0.00217, 0.00249
	Black	Women	42	0.00161, 0.00182, 0.00206, 0.00234, 0.00265
	Black	Women	43	0.00179, 0.00201, 0.00225, 0.00251, 0.00282
	Black	Women	44	0.00201, 0.00222, 0.00245, 0.00271, 0.003
	Black	Women	45	0.00223, 0.00245, 0.00269, 0.00295, 0.00323
	Black	Women	46	0.00245, 0.00269, 0.00295, 0.00323, 0.00355
	Black	Women	47	0.00268, 0.00295, 0.00325, 0.00357, 0.00394
	Black	Women	48	0.00292, 0.00324, 0.00358, 0.00396, 0.00438
	Black	Women	49	0.00321, 0.00356, 0.00395, 0.00437, 0.00484
	Black	Women	50	0.00357, 0.00394, 0.00435, 0.00479, 0.00529
	Black	Women	51	0.00401, 0.00438, 0.00478, 0.00521, 0.00569
	Black	Women	52	0.00451, 0.00486, 0.00524, 0.00564, 0.00608
	Black	Women	53	0.00503, 0.00537, 0.00573, 0.00611, 0.00652

Variable	Race/ethnicity	Sex	Age	Distribution
	Black	Women	54	0.00553, 0.00587, 0.00624, 0.00663, 0.00705
	Black	Women	55	0.006, 0.00638, 0.00678, 0.0072, 0.00766
	Black	Women	56	0.00647, 0.00689, 0.00733, 0.0078, 0.00831
	Black	Women	57	0.00696, 0.00742, 0.0079, 0.00841, 0.00896
	Black	Women	58	0.00745, 0.00794, 0.00846, 0.00902, 0.00962
	Black	Women	59	0.0079, 0.00845, 0.00902, 0.00963, 0.0103
	Black	Women	60	0.00834, 0.00893, 0.00956, 0.0102, 0.011
	Black	Women	61	0.0088, 0.00943, 0.0101, 0.0108, 0.0116
	Black	Women	62	0.00928, 0.00994, 0.0107, 0.0114, 0.0122
	Black	Women	63	0.00978, 0.0105, 0.0112, 0.012, 0.0129
	Black	Women	64	0.0103, 0.0111, 0.0119, 0.0128, 0.0137
	Black	Women	65	0.0109, 0.0118, 0.0127, 0.0136, 0.0147
	Black	Women	66	0.0116, 0.0125, 0.0135, 0.0146, 0.0157
	Black	Women	67	0.0124, 0.0135, 0.0145, 0.0157, 0.017
	Black	Women	68	0.0134, 0.0145, 0.0157, 0.0169, 0.0183
	Black	Women	69	0.0144, 0.0156, 0.0169, 0.0183, 0.0198
	Black	Women	70	0.0156, 0.0169, 0.0183, 0.0198, 0.0215
	Black	Women	71	0.0169, 0.0183, 0.0198, 0.0214, 0.0232
	Black	Women	72	0.0183, 0.0198, 0.0214, 0.0232, 0.0251
	Black	Women	73	0.0198, 0.0214, 0.0232, 0.0251, 0.0271
	Black	Women	74	0.0215, 0.0233, 0.0251, 0.0271, 0.0293
	Black	Women	75	0.0235, 0.0253, 0.0273, 0.0294, 0.0316
	Black	Women	76	0.0257, 0.0276, 0.0297, 0.0319, 0.0343
	Black	Women	77	0.0282, 0.0303, 0.0325, 0.0348, 0.0374
	Black	Women	78	0.031, 0.0332, 0.0356, 0.0382, 0.0409
	Black	Women	79	0.034, 0.0365, 0.0392, 0.042, 0.0451
	Black	Women	80	0.0373, 0.0401, 0.0431, 0.0463, 0.0498
	Black	Women	81	0.0408, 0.044, 0.0474, 0.0511, 0.0551
	Black	Women	82	0.0443, 0.048, 0.052, 0.0562, 0.0609
	Black	Women	83	0.0478, 0.0521, 0.0567, 0.0617, 0.0672
	Black	Women	84	0.0513, 0.0561, 0.0614, 0.0672, 0.0736
	Hispanic	Men	30	0.000798, 0.000897, 0.00101, 0.00113, 0.00127
	Hispanic	Men	31	0.000814, 0.000915, 0.00103, 0.00115, 0.00129
	Hispanic	Men	32	0.000825, 0.000929, 0.00104, 0.00117, 0.00132
	Hispanic	Men	33	0.000824, 0.000937, 0.00106, 0.00121, 0.00137
	Hispanic	Men	34	0.000818, 0.000941, 0.00108, 0.00124, 0.00143
	Hispanic	Men	35	0.000819, 0.00095, 0.0011, 0.00128, 0.00148
	Hispanic	Men	36	0.000834, 0.00097, 0.00113, 0.00131, 0.00152
	Hispanic	Men	37	0.000864, 0.001, 0.00116, 0.00135, 0.00156
	Hispanic	Men	38	0.000907, 0.00105, 0.00121, 0.00139, 0.0016
	Hispanic	Men	39	0.000957, 0.0011, 0.00126, 0.00145, 0.00167
	Hispanic	Men	40	0.00101, 0.00116, 0.00133, 0.00153, 0.00176
	Hispanic	Men	41	0.00108, 0.00124, 0.00142, 0.00163, 0.00187

Variable	Race/ethnicity	Sex	Age	Distribution
	Hispanic	Men	42	0.00117, 0.00134, 0.00152, 0.00174, 0.00199
	Hispanic	Men	43	0.00129, 0.00146, 0.00165, 0.00187, 0.00211
	Hispanic	Men	44	0.00146, 0.00162, 0.00181, 0.00201, 0.00224
	Hispanic	Men	45	0.00165, 0.00181, 0.00199, 0.00219, 0.00241
	Hispanic	Men	46	0.00184, 0.00202, 0.00221, 0.00242, 0.00265
	Hispanic	Men	47	0.00205, 0.00224, 0.00245, 0.00269, 0.00294
	Hispanic	Men	48	0.00227, 0.00249, 0.00273, 0.00299, 0.00329
	Hispanic	Men	49	0.00251, 0.00276, 0.00303, 0.00333, 0.00366
	Hispanic	Men	50	0.00279, 0.00306, 0.00335, 0.00367, 0.00403
	Hispanic	Men	51	0.00313, 0.0034, 0.00369, 0.00401, 0.00435
	Hispanic	Men	52	0.00354, 0.00379, 0.00404, 0.00432, 0.00462
	Hispanic	Men	53	0.00401, 0.00421, 0.00442, 0.00463, 0.00486
	Hispanic	Men	54	0.00445, 0.00463, 0.00481, 0.005, 0.0052
	Hispanic	Men	55	0.00478, 0.005, 0.00522, 0.00546, 0.00571
	Hispanic	Men	56	0.00509, 0.00537, 0.00566, 0.00596, 0.00629
	Hispanic	Men	57	0.00542, 0.00576, 0.00611, 0.00649, 0.00689
	Hispanic	Men	58	0.00578, 0.00617, 0.00659, 0.00703, 0.00751
	Hispanic	Men	59	0.00615, 0.0066, 0.00708, 0.00759, 0.00815
	Hispanic	Men	60	0.00655, 0.00705, 0.00759, 0.00816, 0.00879
	Hispanic	Men	61	0.00699, 0.00753, 0.00811, 0.00872, 0.0094
	Hispanic	Men	62	0.00749, 0.00805, 0.00864, 0.00928, 0.00996
	Hispanic	Men	63	0.00803, 0.0086, 0.0092, 0.00985, 0.0105
	Hispanic	Men	64	0.00856, 0.00917, 0.0098, 0.0105, 0.0112
	Hispanic	Men	65	0.0091, 0.00976, 0.0105, 0.0112, 0.012
	Hispanic	Men	66	0.00968, 0.0104, 0.0112, 0.012, 0.013
	Hispanic	Men	67	0.0103, 0.0112, 0.012, 0.013, 0.014
	Hispanic	Men	68	0.0111, 0.012, 0.013, 0.0141, 0.0152
	Hispanic	Men	69	0.012, 0.013, 0.0141, 0.0153, 0.0166
	Hispanic	Men	70	0.013, 0.0141, 0.0153, 0.0166, 0.0181
	Hispanic	Men	71	0.0141, 0.0153, 0.0167, 0.0182, 0.0198
	Hispanic	Men	72	0.0153, 0.0167, 0.0182, 0.0198, 0.0216
	Hispanic	Men	73	0.0167, 0.0182, 0.0199, 0.0216, 0.0236
	Hispanic	Men	74	0.0183, 0.0199, 0.0217, 0.0236, 0.0258
	Hispanic	Men	75	0.0201, 0.0219, 0.0238, 0.0259, 0.0281
	Hispanic	Men	76	0.0221, 0.0241, 0.0261, 0.0284, 0.0308
	Hispanic	Men	77	0.0245, 0.0266, 0.0288, 0.0313, 0.034
	Hispanic	Men	78	0.0272, 0.0295, 0.032, 0.0347, 0.0377
	Hispanic	Men	79	0.0303, 0.0329, 0.0356, 0.0386, 0.0419
	Hispanic	Men	80	0.0339, 0.0367, 0.0398, 0.0431, 0.0467
	Hispanic	Men	81	0.0378, 0.041, 0.0444, 0.048, 0.0521
	Hispanic	Men	82	0.0419, 0.0454, 0.0493, 0.0534, 0.0579
	Hispanic	Men	83	0.0459, 0.05, 0.0543, 0.059, 0.0642
	Hispanic	Men	84	0.0498, 0.0544, 0.0593, 0.0647, 0.0706

Variable	Race/ethnicity	Sex	Age	Distribution
	Hispanic	Women	30	0.000328, 0.00037, 0.000416, 0.000469, 0.000528
	Hispanic	Women	31	0.000347, 0.00039, 0.000437, 0.000491, 0.000552
	Hispanic	Women	32	0.000367, 0.000411, 0.00046, 0.000514, 0.000575
	Hispanic	Women	33	0.000388, 0.000433, 0.000482, 0.000538, 0.0006
	Hispanic	Women	34	0.000405, 0.000453, 0.000506, 0.000565, 0.000631
	Hispanic	Women	35	0.00042, 0.000472, 0.00053, 0.000596, 0.00067
	Hispanic	Women	36	0.000436, 0.000493, 0.000557, 0.00063, 0.000714
	Hispanic	Women	37	0.000455, 0.000518, 0.000588, 0.000668, 0.00076
	Hispanic	Women	38	0.000481, 0.000548, 0.000623, 0.000708, 0.000806
	Hispanic	Women	39	0.000515, 0.000585, 0.000663, 0.000752, 0.000853
	Hispanic	Women	40	0.000558, 0.000629, 0.00071, 0.0008, 0.000903
	Hispanic	Women	41	0.00061, 0.000683, 0.000764, 0.000855, 0.000957
	Hispanic	Women	42	0.000672, 0.000746, 0.000828, 0.000919, 0.00102
	Hispanic	Women	43	0.000745, 0.00082, 0.000903, 0.000993, 0.00109
	Hispanic	Women	44	0.00083, 0.000907, 0.00099, 0.00108, 0.00118
	Hispanic	Women	45	0.000923, 0.001, 0.00109, 0.00119, 0.00129
	Hispanic	Women	46	0.00102, 0.00111, 0.00121, 0.00131, 0.00142
	Hispanic	Women	47	0.00113, 0.00123, 0.00133, 0.00145, 0.00157
	Hispanic	Women	48	0.00125, 0.00136, 0.00148, 0.00161, 0.00175
	Hispanic	Women	49	0.00138, 0.0015, 0.00163, 0.00178, 0.00194
	Hispanic	Women	50	0.00152, 0.00165, 0.0018, 0.00196, 0.00214
	Hispanic	Women	51	0.00168, 0.00183, 0.00198, 0.00215, 0.00234
	Hispanic	Women	52	0.00186, 0.00201, 0.00217, 0.00235, 0.00254
	Hispanic	Women	53	0.00206, 0.00221, 0.00238, 0.00256, 0.00275
	Hispanic	Women	54	0.00225, 0.00242, 0.0026, 0.00279, 0.003
	Hispanic	Women	55	0.00244, 0.00263, 0.00283, 0.00304, 0.00327
	Hispanic	Women	56	0.00264, 0.00285, 0.00307, 0.00331, 0.00357
	Hispanic	Women	57	0.00286, 0.00309, 0.00333, 0.00359, 0.00387
	Hispanic	Women	58	0.0031, 0.00334, 0.0036, 0.00388, 0.00418
	Hispanic	Women	59	0.00335, 0.00361, 0.00389, 0.0042, 0.00453
	Hispanic	Women	60	0.0036, 0.00389, 0.0042, 0.00454, 0.00491
	Hispanic	Women	61	0.00388, 0.0042, 0.00454, 0.00491, 0.00531
	Hispanic	Women	62	0.00418, 0.00453, 0.0049, 0.0053, 0.00573
	Hispanic	Women	63	0.00451, 0.00489, 0.00529, 0.00572, 0.0062
	Hispanic	Women	64	0.00486, 0.00527, 0.00571, 0.00619, 0.00672
	Hispanic	Women	65	0.00523, 0.00569, 0.00619, 0.00672, 0.00731
	Hispanic	Women	66	0.00566, 0.00617, 0.00671, 0.00731, 0.00796
	Hispanic	Women	67	0.00616, 0.00671, 0.00731, 0.00796, 0.00868
	Hispanic	Women	68	0.00673, 0.00733, 0.00798, 0.00869, 0.00947
	Hispanic	Women	69	0.00739, 0.00804, 0.00875, 0.00951, 0.0104
	Hispanic	Women	70	0.00813, 0.00884, 0.00961, 0.0104, 0.0114
	Hispanic	Women	71	0.00895, 0.00973, 0.0106, 0.0115, 0.0125
	Hispanic	Women	72	0.00987, 0.0107, 0.0117, 0.0127, 0.0138

Variable	Race/ethnicity	Sex	Age	Distribution
	Hispanic	Women	73	0.0109, 0.0119, 0.0129, 0.014, 0.0152
	Hispanic	Women	74	0.0121, 0.0131, 0.0142, 0.0154, 0.0167
	Hispanic	Women	75	0.0135, 0.0146, 0.0158, 0.017, 0.0184
	Hispanic	Women	76	0.0152, 0.0163, 0.0176, 0.0189, 0.0203
	Hispanic	Women	77	0.0171, 0.0183, 0.0196, 0.0211, 0.0226
	Hispanic	Women	78	0.0192, 0.0206, 0.0221, 0.0236, 0.0253
	Hispanic	Women	79	0.0217, 0.0232, 0.0249, 0.0266, 0.0285
	Hispanic	Women	80	0.0244, 0.0262, 0.0281, 0.0301, 0.0323
	Hispanic	Women	81	0.0273, 0.0294, 0.0317, 0.034, 0.0366
	Hispanic	Women	82	0.0304, 0.0329, 0.0356, 0.0384, 0.0415
	Hispanic	Women	83	0.0336, 0.0365, 0.0397, 0.0431, 0.0468
	Hispanic	Women	84	0.0368, 0.0402, 0.0439, 0.0479, 0.0523
	White	Men	30	0.00117, 0.00137, 0.00161, 0.00188, 0.00221
	White	Men	31	0.00122, 0.00142, 0.00165, 0.00192, 0.00223
	White	Men	32	0.00127, 0.00146, 0.00169, 0.00194, 0.00224
	White	Men	33	0.00131, 0.0015, 0.00172, 0.00197, 0.00225
	White	Men	34	0.00133, 0.00152, 0.00174, 0.00199, 0.00228
	White	Men	35	0.00134, 0.00154, 0.00177, 0.00203, 0.00233
	White	Men	36	0.00136, 0.00157, 0.0018, 0.00207, 0.00238
	White	Men	37	0.00139, 0.0016, 0.00184, 0.00212, 0.00245
	White	Men	38	0.00143, 0.00165, 0.0019, 0.00219, 0.00252
	White	Men	39	0.00149, 0.00171, 0.00197, 0.00226, 0.0026
	White	Men	40	0.00156, 0.00179, 0.00205, 0.00235, 0.0027
	White	Men	41	0.00166, 0.00189, 0.00216, 0.00247, 0.00283
	White	Men	42	0.00178, 0.00202, 0.0023, 0.00262, 0.00298
	White	Men	43	0.00193, 0.00219, 0.00247, 0.0028, 0.00316
	White	Men	44	0.00213, 0.00239, 0.00268, 0.00301, 0.00339
	White	Men	45	0.00234, 0.00262, 0.00294, 0.00329, 0.00368
	White	Men	46	0.00257, 0.00288, 0.00323, 0.00363, 0.00408
	White	Men	47	0.00281, 0.00317, 0.00358, 0.00403, 0.00455
	White	Men	48	0.00309, 0.0035, 0.00396, 0.00448, 0.00508
	White	Men	49	0.00341, 0.00387, 0.00439, 0.00497, 0.00564
	White	Men	50	0.00379, 0.00429, 0.00484, 0.00547, 0.00619
	White	Men	51	0.00423, 0.00475, 0.00533, 0.00599, 0.00673
	White	Men	52	0.00472, 0.00526, 0.00585, 0.0065, 0.00723
	White	Men	53	0.00527, 0.0058, 0.00638, 0.00702, 0.00773
	White	Men	54	0.00581, 0.00635, 0.00693, 0.00756, 0.00826
	White	Men	55	0.00632, 0.00688, 0.00749, 0.00815, 0.00887
	White	Men	56	0.00678, 0.00739, 0.00806, 0.00878, 0.00958
	White	Men	57	0.00723, 0.0079, 0.00864, 0.00944, 0.0103
	White	Men	58	0.00771, 0.00844, 0.00923, 0.0101, 0.011
	White	Men	59	0.00823, 0.009, 0.00982, 0.0107, 0.0117
	White	Men	60	0.00881, 0.00959, 0.0104, 0.0113, 0.0124

Variable	Race/ethnicity	Sex	Age	Distribution
	White	Men	61	0.00944, 0.0102, 0.0111, 0.012, 0.013
	White	Men	62	0.0101, 0.0109, 0.0117, 0.0126, 0.0136
	White	Men	63	0.0108, 0.0116, 0.0124, 0.0133, 0.0143
	White	Men	64	0.0115, 0.0123, 0.0132, 0.0141, 0.0151
	White	Men	65	0.0123, 0.0131, 0.014, 0.015, 0.0161
	White	Men	66	0.0131, 0.014, 0.015, 0.0161, 0.0173
	White	Men	67	0.014, 0.0151, 0.0162, 0.0174, 0.0187
	White	Men	68	0.0151, 0.0163, 0.0175, 0.0189, 0.0204
	White	Men	69	0.0163, 0.0176, 0.019, 0.0206, 0.0222
	White	Men	70	0.0178, 0.0192, 0.0208, 0.0224, 0.0243
	White	Men	71	0.0194, 0.021, 0.0227, 0.0245, 0.0265
	White	Men	72	0.0212, 0.0229, 0.0248, 0.0268, 0.0289
	White	Men	73	0.0232, 0.0251, 0.0271, 0.0292, 0.0315
	White	Men	74	0.0255, 0.0274, 0.0296, 0.0318, 0.0343
	White	Men	75	0.028, 0.0301, 0.0323, 0.0348, 0.0374
	White	Men	76	0.0309, 0.0331, 0.0355, 0.038, 0.0408
	White	Men	77	0.0341, 0.0366, 0.0391, 0.0419, 0.0449
	White	Men	78	0.0378, 0.0405, 0.0434, 0.0464, 0.0497
	White	Men	79	0.0421, 0.0451, 0.0483, 0.0518, 0.0555
	White	Men	80	0.0469, 0.0503, 0.054, 0.0579, 0.0622
	White	Men	81	0.0522, 0.0561, 0.0603, 0.0649, 0.0698
	White	Men	82	0.0579, 0.0624, 0.0673, 0.0725, 0.0782
	White	Men	83	0.0636, 0.0689, 0.0745, 0.0805, 0.0871
	White	Men	84	0.0694, 0.0753, 0.0817, 0.0887, 0.0963
	White	Women	30	0.000593, 0.000668, 0.000751, 0.000844, 0.00095
	White	Women	31	0.000625, 0.000705, 0.000795, 0.000896, 0.00101
	White	Women	32	0.00066, 0.000744, 0.000839, 0.000945, 0.00107
	White	Women	33	0.000696, 0.000784, 0.000882, 0.000993, 0.00112
	White	Women	34	0.000725, 0.00082, 0.000925, 0.00104, 0.00118
	White	Women	35	0.000747, 0.000852, 0.00097, 0.0011, 0.00126
	White	Women	36	0.00077, 0.000886, 0.00102, 0.00117, 0.00135
	White	Women	37	0.000798, 0.000925, 0.00107, 0.00124, 0.00144
	White	Women	38	0.000831, 0.00097, 0.00113, 0.00132, 0.00154
	White	Women	39	0.00087, 0.00102, 0.0012, 0.00141, 0.00165
	White	Women	40	0.000919, 0.00108, 0.00128, 0.0015, 0.00178
	White	Women	41	0.000982, 0.00116, 0.00137, 0.00161, 0.00191
	White	Women	42	0.00106, 0.00125, 0.00148, 0.00174, 0.00205
	White	Women	43	0.00116, 0.00136, 0.0016, 0.00188, 0.00221
	White	Women	44	0.00127, 0.00149, 0.00175, 0.00205, 0.00241
	White	Women	45	0.00139, 0.00163, 0.00191, 0.00225, 0.00264
	White	Women	46	0.00153, 0.00179, 0.0021, 0.00247, 0.0029
	White	Women	47	0.00169, 0.00198, 0.00231, 0.0027, 0.00316
	White	Women	48	0.0019, 0.0022, 0.00255, 0.00295, 0.00342

Variable	Race/ethnicity	Sex	Age	Distribution
	White	Women	49	0.00212, 0.00244, 0.0028, 0.00321, 0.00369
	White	Women	50	0.00235, 0.00269, 0.00307, 0.00351, 0.00401
	White	Women	51	0.00255, 0.00293, 0.00335, 0.00384, 0.00441
	White	Women	52	0.00274, 0.00316, 0.00365, 0.00421, 0.00485
	White	Women	53	0.00295, 0.00341, 0.00395, 0.00457, 0.00529
	White	Women	54	0.00319, 0.00369, 0.00425, 0.0049, 0.00565
	White	Women	55	0.00351, 0.004, 0.00455, 0.00517, 0.00589
	White	Women	56	0.00389, 0.00434, 0.00484, 0.00539, 0.00601
	White	Women	57	0.00431, 0.00471, 0.00513, 0.00559, 0.0061
	White	Women	58	0.00472, 0.00506, 0.00543, 0.00583, 0.00625
	White	Women	59	0.00506, 0.0054, 0.00575, 0.00613, 0.00654
	White	Women	60	0.00537, 0.00573, 0.00611, 0.00652, 0.00695
	White	Women	61	0.0057, 0.0061, 0.00652, 0.00696, 0.00744
	White	Women	62	0.00608, 0.00652, 0.00698, 0.00748, 0.00802
	White	Women	63	0.00654, 0.00702, 0.00753, 0.00807, 0.00866
	White	Women	64	0.00707, 0.00759, 0.00815, 0.00875, 0.0094
	White	Women	65	0.00766, 0.00825, 0.00887, 0.00954, 0.0103
	White	Women	66	0.00833, 0.00899, 0.00969, 0.0104, 0.0113
	White	Women	67	0.00906, 0.00981, 0.0106, 0.0115, 0.0124
	White	Women	68	0.00984, 0.0107, 0.0116, 0.0127, 0.0138
	White	Women	69	0.0107, 0.0117, 0.0128, 0.014, 0.0153
	White	Women	70	0.0117, 0.0128, 0.0141, 0.0154, 0.017
	White	Women	71	0.0128, 0.0141, 0.0155, 0.017, 0.0188
	White	Women	72	0.014, 0.0154, 0.017, 0.0188, 0.0207
	White	Women	73	0.0154, 0.017, 0.0187, 0.0206, 0.0228
	White	Women	74	0.017, 0.0187, 0.0206, 0.0226, 0.0249
	White	Women	75	0.0189, 0.0207, 0.0227, 0.0249, 0.0273
	White	Women	76	0.0212, 0.0231, 0.0252, 0.0274, 0.0299
	White	Women	77	0.0238, 0.0258, 0.028, 0.0303, 0.0329
	White	Women	78	0.0268, 0.0289, 0.0312, 0.0337, 0.0364
	White	Women	79	0.0301, 0.0325, 0.035, 0.0377, 0.0407
	White	Women	80	0.0338, 0.0365, 0.0393, 0.0424, 0.0457
	White	Women	81	0.0379, 0.0409, 0.0441, 0.0476, 0.0515
	White	Women	82	0.0422, 0.0457, 0.0494, 0.0535, 0.0578
	White	Women	83	0.0467, 0.0507, 0.055, 0.0597, 0.0648
	White	Women	84	0.0513, 0.0559, 0.0608, 0.0662, 0.0721
Total mortality rates for 2023 (0.01, 0.2, 0.5, 0.8. 0.99 percentiles of the empirical distribution produced during forecasting)				
	Black	Men	30	0.00167, 0.00196, 0.00231, 0.00271, 0.00318
	Black	Men	31	0.00176, 0.00204, 0.00235, 0.00271, 0.00313
	Black	Men	32	0.00184, 0.0021, 0.00239, 0.00272, 0.0031
	Black	Men	33	0.00188, 0.00214, 0.00243, 0.00276, 0.00314
	Black	Men	34	0.00192, 0.00219, 0.00249, 0.00283, 0.00322
	Black	Men	35	0.00196, 0.00224, 0.00255, 0.0029, 0.00331

Variable	Race/ethnicity	Sex	Age	Distribution
	Black	Men	36	0.00201, 0.0023, 0.00262, 0.00298, 0.0034
	Black	Men	37	0.00207, 0.00236, 0.00269, 0.00306, 0.00349
	Black	Men	38	0.00212, 0.00242, 0.00276, 0.00315, 0.00359
	Black	Men	39	0.00217, 0.00248, 0.00284, 0.00325, 0.00372
	Black	Men	40	0.0022, 0.00255, 0.00294, 0.00339, 0.00391
	Black	Men	41	0.00225, 0.00263, 0.00306, 0.00357, 0.00416
	Black	Men	42	0.00232, 0.00274, 0.00323, 0.0038, 0.00448
	Black	Men	43	0.00242, 0.00289, 0.00344, 0.00409, 0.00487
	Black	Men	44	0.00255, 0.00308, 0.0037, 0.00444, 0.00535
	Black	Men	45	0.00272, 0.00331, 0.00401, 0.00486, 0.00591
	Black	Men	46	0.00293, 0.00359, 0.00438, 0.00534, 0.00653
	Black	Men	47	0.00321, 0.00393, 0.0048, 0.00586, 0.00718
	Black	Men	48	0.00357, 0.00435, 0.00529, 0.00643, 0.00783
	Black	Men	49	0.00401, 0.00485, 0.00585, 0.00705, 0.00851
	Black	Men	50	0.00453, 0.00542, 0.00647, 0.00772, 0.00924
	Black	Men	51	0.00512, 0.00606, 0.00716, 0.00846, 0.01
	Black	Men	52	0.0058, 0.00679, 0.00793, 0.00925, 0.0108
	Black	Men	53	0.00659, 0.0076, 0.00875, 0.0101, 0.0116
	Black	Men	54	0.00748, 0.0085, 0.00965, 0.011, 0.0124
	Black	Men	55	0.00844, 0.00947, 0.0106, 0.0119, 0.0133
	Black	Men	56	0.00948, 0.0105, 0.0116, 0.0129, 0.0143
	Black	Men	57	0.0106, 0.0116, 0.0127, 0.0139, 0.0153
	Black	Men	58	0.0118, 0.0128, 0.0139, 0.015, 0.0163
	Black	Men	59	0.0129, 0.014, 0.015, 0.0162, 0.0175
	Black	Men	60	0.014, 0.0151, 0.0162, 0.0175, 0.0188
	Black	Men	61	0.0151, 0.0162, 0.0174, 0.0187, 0.02
	Black	Men	62	0.0162, 0.0173, 0.0185, 0.0198, 0.0212
	Black	Men	63	0.0171, 0.0183, 0.0196, 0.0209, 0.0224
	Black	Men	64	0.018, 0.0193, 0.0206, 0.0221, 0.0237
	Black	Men	65	0.0189, 0.0203, 0.0217, 0.0233, 0.025
	Black	Men	66	0.0198, 0.0213, 0.0229, 0.0245, 0.0264
	Black	Men	67	0.0208, 0.0224, 0.0241, 0.026, 0.028
	Black	Men	68	0.022, 0.0237, 0.0256, 0.0276, 0.0299
	Black	Men	69	0.0233, 0.0252, 0.0273, 0.0295, 0.032
	Black	Men	70	0.0247, 0.0269, 0.0292, 0.0317, 0.0344
	Black	Men	71	0.0264, 0.0288, 0.0313, 0.0341, 0.0371
	Black	Men	72	0.0283, 0.0309, 0.0336, 0.0366, 0.0399
	Black	Men	73	0.0305, 0.0332, 0.0361, 0.0393, 0.0429
	Black	Men	74	0.0329, 0.0358, 0.0388, 0.0422, 0.0459
	Black	Men	75	0.0357, 0.0386, 0.0418, 0.0452, 0.049
	Black	Men	76	0.0387, 0.0417, 0.045, 0.0485, 0.0523
	Black	Men	77	0.042, 0.0452, 0.0486, 0.0522, 0.0562
	Black	Men	78	0.0454, 0.0489, 0.0525, 0.0565, 0.0608

Variable	Race/ethnicity	Sex	Age	Distribution
	Black	Men	79	0.0491, 0.0529, 0.057, 0.0614, 0.0662
	Black	Men	80	0.053, 0.0573, 0.0619, 0.0668, 0.0723
	Black	Men	81	0.0572, 0.062, 0.0672, 0.0727, 0.0789
	Black	Men	82	0.0615, 0.0669, 0.0727, 0.079, 0.0859
	Black	Men	83	0.0656, 0.0717, 0.0782, 0.0854, 0.0932
	Black	Men	84	0.0696, 0.0763, 0.0836, 0.0916, 0.1
	Black	Women	30	0.000608, 0.000743, 0.000907, 0.00111, 0.00135
	Black	Women	31	0.000679, 0.000815, 0.000977, 0.00117, 0.00141
	Black	Women	32	0.000762, 0.000897, 0.00105, 0.00124, 0.00146
	Black	Women	33	0.000846, 0.000981, 0.00114, 0.00132, 0.00153
	Black	Women	34	0.000919, 0.00106, 0.00122, 0.0014, 0.00161
	Black	Women	35	0.000974, 0.00112, 0.00129, 0.00149, 0.00172
	Black	Women	36	0.00102, 0.00118, 0.00137, 0.00158, 0.00184
	Black	Women	37	0.00107, 0.00124, 0.00144, 0.00168, 0.00195
	Black	Women	38	0.00113, 0.00131, 0.00153, 0.00177, 0.00206
	Black	Women	39	0.0012, 0.0014, 0.00162, 0.00189, 0.00219
	Black	Women	40	0.00129, 0.0015, 0.00174, 0.00201, 0.00234
	Black	Women	41	0.00141, 0.00163, 0.00187, 0.00216, 0.00249
	Black	Women	42	0.00156, 0.00178, 0.00203, 0.00232, 0.00265
	Black	Women	43	0.00174, 0.00196, 0.00221, 0.00249, 0.00281
	Black	Women	44	0.00195, 0.00217, 0.00241, 0.00268, 0.00298
	Black	Women	45	0.00217, 0.00239, 0.00264, 0.00291, 0.00321
	Black	Women	46	0.00239, 0.00263, 0.0029, 0.0032, 0.00353
	Black	Women	47	0.00261, 0.00289, 0.0032, 0.00354, 0.00392
	Black	Women	48	0.00285, 0.00317, 0.00353, 0.00392, 0.00437
	Black	Women	49	0.00313, 0.0035, 0.00389, 0.00434, 0.00484
	Black	Women	50	0.00349, 0.00387, 0.0043, 0.00476, 0.00529
	Black	Women	51	0.00392, 0.00431, 0.00473, 0.00519, 0.0057
	Black	Women	52	0.00443, 0.0048, 0.00519, 0.00562, 0.00608
	Black	Women	53	0.00495, 0.0053, 0.00568, 0.00608, 0.00652
	Black	Women	54	0.00544, 0.00581, 0.00619, 0.0066, 0.00704
	Black	Women	55	0.00592, 0.00631, 0.00673, 0.00717, 0.00765
	Black	Women	56	0.00639, 0.00682, 0.00728, 0.00777, 0.0083
	Black	Women	57	0.00687, 0.00734, 0.00784, 0.00837, 0.00895
	Black	Women	58	0.00734, 0.00785, 0.0084, 0.00898, 0.0096
	Black	Women	59	0.00778, 0.00835, 0.00894, 0.00958, 0.0103
	Black	Women	60	0.00821, 0.00882, 0.00947, 0.0102, 0.0109
	Black	Women	61	0.00864, 0.0093, 0.00999, 0.0107, 0.0116
	Black	Women	62	0.0091, 0.00979, 0.0105, 0.0113, 0.0122
	Black	Women	63	0.00958, 0.0103, 0.0111, 0.0119, 0.0129
	Black	Women	64	0.0101, 0.0109, 0.0117, 0.0127, 0.0136
	Black	Women	65	0.0107, 0.0115, 0.0125, 0.0135, 0.0146
	Black	Women	66	0.0114, 0.0123, 0.0133, 0.0144, 0.0156

Variable	Race/ethnicity	Sex	Age	Distribution
	Black	Women	67	0.0122, 0.0132, 0.0143, 0.0155, 0.0168
	Black	Women	68	0.0131, 0.0142, 0.0154, 0.0167, 0.0182
	Black	Women	69	0.0141, 0.0153, 0.0167, 0.0181, 0.0197
	Black	Women	70	0.0152, 0.0166, 0.018, 0.0196, 0.0213
	Black	Women	71	0.0165, 0.0179, 0.0195, 0.0212, 0.023
	Black	Women	72	0.0179, 0.0194, 0.0211, 0.0229, 0.0249
	Black	Women	73	0.0193, 0.021, 0.0228, 0.0247, 0.0269
	Black	Women	74	0.021, 0.0228, 0.0247, 0.0267, 0.029
	Black	Women	75	0.0229, 0.0248, 0.0268, 0.029, 0.0313
	Black	Women	76	0.0251, 0.0271, 0.0292, 0.0315, 0.0339
	Black	Women	77	0.0276, 0.0297, 0.0319, 0.0344, 0.037
	Black	Women	78	0.0303, 0.0326, 0.0351, 0.0377, 0.0405
	Black	Women	79	0.0333, 0.0358, 0.0386, 0.0415, 0.0447
	Black	Women	80	0.0365, 0.0394, 0.0425, 0.0458, 0.0493
	Black	Women	81	0.0399, 0.0432, 0.0467, 0.0505, 0.0546
	Black	Women	82	0.0434, 0.0471, 0.0512, 0.0556, 0.0604
	Black	Women	83	0.0468, 0.0511, 0.0558, 0.061, 0.0666
	Black	Women	84	0.0502, 0.0552, 0.0605, 0.0665, 0.073
	Hispanic	Men	30	0.000796, 0.000896, 0.00101, 0.00114, 0.00128
	Hispanic	Men	31	0.000812, 0.000914, 0.00103, 0.00116, 0.0013
	Hispanic	Men	32	0.000822, 0.000928, 0.00105, 0.00118, 0.00133
	Hispanic	Men	33	0.000819, 0.000934, 0.00106, 0.00121, 0.00138
	Hispanic	Men	34	0.000811, 0.000937, 0.00108, 0.00125, 0.00144
	Hispanic	Men	35	0.00081, 0.000944, 0.0011, 0.00128, 0.00149
	Hispanic	Men	36	0.000822, 0.000962, 0.00112, 0.00131, 0.00153
	Hispanic	Men	37	0.00085, 0.000992, 0.00116, 0.00135, 0.00157
	Hispanic	Men	38	0.00089, 0.00103, 0.0012, 0.00139, 0.00161
	Hispanic	Men	39	0.000938, 0.00108, 0.00125, 0.00144, 0.00167
	Hispanic	Men	40	0.00099, 0.00114, 0.00132, 0.00152, 0.00175
	Hispanic	Men	41	0.00105, 0.00122, 0.0014, 0.00161, 0.00186
	Hispanic	Men	42	0.00114, 0.00131, 0.0015, 0.00172, 0.00198
	Hispanic	Men	43	0.00126, 0.00143, 0.00163, 0.00185, 0.0021
	Hispanic	Men	44	0.00142, 0.00159, 0.00178, 0.00199, 0.00223
	Hispanic	Men	45	0.00161, 0.00178, 0.00196, 0.00217, 0.00239
	Hispanic	Men	46	0.0018, 0.00198, 0.00218, 0.00239, 0.00263
	Hispanic	Men	47	0.002, 0.0022, 0.00242, 0.00266, 0.00292
	Hispanic	Men	48	0.00222, 0.00245, 0.00269, 0.00297, 0.00327
	Hispanic	Men	49	0.00246, 0.00271, 0.00299, 0.0033, 0.00364
	Hispanic	Men	50	0.00274, 0.00301, 0.00331, 0.00364, 0.00401
	Hispanic	Men	51	0.00308, 0.00335, 0.00365, 0.00398, 0.00434
	Hispanic	Men	52	0.00349, 0.00374, 0.00401, 0.0043, 0.0046
	Hispanic	Men	53	0.00397, 0.00417, 0.00438, 0.00461, 0.00484
	Hispanic	Men	54	0.0044, 0.00459, 0.00478, 0.00497, 0.00518

Variable	Race/ethnicity	Sex	Age	Distribution
	Hispanic	Men	55	0.00473, 0.00495, 0.00519, 0.00543, 0.00569
	Hispanic	Men	56	0.00503, 0.00532, 0.00562, 0.00593, 0.00627
	Hispanic	Men	57	0.00535, 0.0057, 0.00607, 0.00646, 0.00688
	Hispanic	Men	58	0.0057, 0.00611, 0.00654, 0.00699, 0.00749
	Hispanic	Men	59	0.00606, 0.00653, 0.00702, 0.00755, 0.00812
	Hispanic	Men	60	0.00644, 0.00696, 0.00751, 0.00811, 0.00876
	Hispanic	Men	61	0.00687, 0.00743, 0.00802, 0.00866, 0.00935
	Hispanic	Men	62	0.00736, 0.00793, 0.00854, 0.00919, 0.00991
	Hispanic	Men	63	0.00788, 0.00846, 0.00908, 0.00975, 0.0105
	Hispanic	Men	64	0.00839, 0.00901, 0.00966, 0.0104, 0.0111
	Hispanic	Men	65	0.00891, 0.00959, 0.0103, 0.0111, 0.0119
	Hispanic	Men	66	0.00947, 0.0102, 0.011, 0.0119, 0.0128
	Hispanic	Men	67	0.0101, 0.0109, 0.0118, 0.0128, 0.0139
	Hispanic	Men	68	0.0108, 0.0118, 0.0128, 0.0139, 0.0151
	Hispanic	Men	69	0.0117, 0.0127, 0.0139, 0.0151, 0.0164
	Hispanic	Men	70	0.0127, 0.0138, 0.0151, 0.0164, 0.0179
	Hispanic	Men	71	0.0138, 0.015, 0.0164, 0.0179, 0.0196
	Hispanic	Men	72	0.015, 0.0164, 0.0179, 0.0196, 0.0214
	Hispanic	Men	73	0.0163, 0.0179, 0.0195, 0.0214, 0.0234
	Hispanic	Men	74	0.0179, 0.0195, 0.0214, 0.0233, 0.0255
	Hispanic	Men	75	0.0196, 0.0214, 0.0234, 0.0255, 0.0279
	Hispanic	Men	76	0.0216, 0.0236, 0.0257, 0.028, 0.0306
	Hispanic	Men	77	0.024, 0.0261, 0.0284, 0.0309, 0.0337
	Hispanic	Men	78	0.0266, 0.029, 0.0315, 0.0343, 0.0374
	Hispanic	Men	79	0.0297, 0.0323, 0.0351, 0.0382, 0.0416
	Hispanic	Men	80	0.0331, 0.0361, 0.0392, 0.0426, 0.0464
	Hispanic	Men	81	0.037, 0.0402, 0.0437, 0.0475, 0.0517
	Hispanic	Men	82	0.041, 0.0447, 0.0486, 0.0528, 0.0575
	Hispanic	Men	83	0.045, 0.0491, 0.0536, 0.0584, 0.0638
	Hispanic	Men	84	0.0488, 0.0534, 0.0585, 0.064, 0.0701
	Hispanic	Women	30	0.000328, 0.000369, 0.000416, 0.000469, 0.000528
	Hispanic	Women	31	0.000347, 0.00039, 0.000437, 0.000491, 0.000552
	Hispanic	Women	32	0.000367, 0.000411, 0.000459, 0.000514, 0.000575
	Hispanic	Women	33	0.000386, 0.000432, 0.000482, 0.000537, 0.0006
	Hispanic	Women	34	0.000403, 0.000451, 0.000504, 0.000564, 0.000632
	Hispanic	Women	35	0.000416, 0.000469, 0.000528, 0.000595, 0.00067
	Hispanic	Women	36	0.00043, 0.000489, 0.000554, 0.000628, 0.000714
	Hispanic	Women	37	0.000449, 0.000512, 0.000583, 0.000665, 0.000758
	Hispanic	Women	38	0.000473, 0.00054, 0.000617, 0.000703, 0.000803
	Hispanic	Women	39	0.000505, 0.000576, 0.000655, 0.000745, 0.000849
	Hispanic	Women	40	0.000546, 0.000618, 0.0007, 0.000792, 0.000897
	Hispanic	Women	41	0.000596, 0.00067, 0.000753, 0.000845, 0.00095
	Hispanic	Women	42	0.000656, 0.000731, 0.000815, 0.000907, 0.00101

Variable	Race/ethnicity	Sex	Age	Distribution
	Hispanic	Women	43	0.000727, 0.000804, 0.000888, 0.00098, 0.00108
	Hispanic	Women	44	0.000811, 0.000889, 0.000973, 0.00107, 0.00117
	Hispanic	Women	45	0.000903, 0.000985, 0.00107, 0.00117, 0.00127
	Hispanic	Women	46	0.001, 0.00109, 0.00119, 0.00129, 0.0014
	Hispanic	Women	47	0.00111, 0.00121, 0.00131, 0.00143, 0.00156
	Hispanic	Women	48	0.00123, 0.00134, 0.00146, 0.00159, 0.00173
	Hispanic	Women	49	0.00135, 0.00148, 0.00161, 0.00176, 0.00192
	Hispanic	Women	50	0.00149, 0.00163, 0.00178, 0.00194, 0.00212
	Hispanic	Women	51	0.00166, 0.0018, 0.00196, 0.00213, 0.00232
	Hispanic	Women	52	0.00184, 0.00199, 0.00215, 0.00233, 0.00252
	Hispanic	Women	53	0.00204, 0.00219, 0.00236, 0.00254, 0.00273
	Hispanic	Women	54	0.00223, 0.0024, 0.00258, 0.00277, 0.00298
	Hispanic	Women	55	0.00242, 0.00261, 0.00281, 0.00302, 0.00326
	Hispanic	Women	56	0.00262, 0.00282, 0.00305, 0.00329, 0.00355
	Hispanic	Women	57	0.00283, 0.00306, 0.0033, 0.00357, 0.00385
	Hispanic	Women	58	0.00307, 0.00331, 0.00357, 0.00386, 0.00417
	Hispanic	Women	59	0.00331, 0.00357, 0.00386, 0.00417, 0.00451
	Hispanic	Women	60	0.00355, 0.00385, 0.00417, 0.00451, 0.00489
	Hispanic	Women	61	0.00382, 0.00414, 0.00449, 0.00487, 0.00528
	Hispanic	Women	62	0.00412, 0.00447, 0.00484, 0.00525, 0.0057
	Hispanic	Women	63	0.00443, 0.00481, 0.00522, 0.00566, 0.00615
	Hispanic	Women	64	0.00477, 0.00519, 0.00564, 0.00613, 0.00666
	Hispanic	Women	65	0.00514, 0.0056, 0.0061, 0.00664, 0.00724
	Hispanic	Women	66	0.00555, 0.00606, 0.00662, 0.00722, 0.00789
	Hispanic	Women	67	0.00603, 0.00659, 0.0072, 0.00786, 0.0086
	Hispanic	Women	68	0.00659, 0.0072, 0.00786, 0.00858, 0.00938
	Hispanic	Women	69	0.00723, 0.00789, 0.00861, 0.0094, 0.0103
	Hispanic	Women	70	0.00795, 0.00867, 0.00945, 0.0103, 0.0112
	Hispanic	Women	71	0.00875, 0.00954, 0.0104, 0.0113, 0.0124
	Hispanic	Women	72	0.00965, 0.0105, 0.0115, 0.0125, 0.0136
	Hispanic	Women	73	0.0107, 0.0116, 0.0127, 0.0138, 0.015
	Hispanic	Women	74	0.0118, 0.0129, 0.014, 0.0152, 0.0165
	Hispanic	Women	75	0.0132, 0.0143, 0.0155, 0.0168, 0.0182
	Hispanic	Women	76	0.0148, 0.016, 0.0173, 0.0186, 0.0201
	Hispanic	Women	77	0.0167, 0.018, 0.0193, 0.0208, 0.0224
	Hispanic	Women	78	0.0188, 0.0202, 0.0217, 0.0233, 0.0251
	Hispanic	Women	79	0.0212, 0.0228, 0.0245, 0.0263, 0.0283
	Hispanic	Women	80	0.0239, 0.0257, 0.0276, 0.0297, 0.032
	Hispanic	Women	81	0.0268, 0.0289, 0.0312, 0.0336, 0.0363
	Hispanic	Women	82	0.0298, 0.0323, 0.035, 0.038, 0.0412
	Hispanic	Women	83	0.0329, 0.0359, 0.0391, 0.0426, 0.0465
	Hispanic	Women	84	0.036, 0.0395, 0.0433, 0.0474, 0.0519
	White	Men	30	0.00114, 0.00136, 0.0016, 0.0019, 0.00225

Variable	Race/ethnicity	Sex	Age	Distribution
	White	Men	31	0.0012, 0.00141, 0.00165, 0.00193, 0.00227
	White	Men	32	0.00125, 0.00145, 0.00168, 0.00195, 0.00227
	White	Men	33	0.00128, 0.00148, 0.00171, 0.00197, 0.00228
	White	Men	34	0.0013, 0.0015, 0.00173, 0.002, 0.00231
	White	Men	35	0.00132, 0.00152, 0.00176, 0.00203, 0.00235
	White	Men	36	0.00133, 0.00154, 0.00179, 0.00207, 0.00241
	White	Men	37	0.00135, 0.00157, 0.00183, 0.00212, 0.00247
	White	Men	38	0.00139, 0.00162, 0.00188, 0.00218, 0.00253
	White	Men	39	0.00144, 0.00168, 0.00194, 0.00225, 0.00261
	White	Men	40	0.00152, 0.00175, 0.00202, 0.00234, 0.0027
	White	Men	41	0.00161, 0.00185, 0.00213, 0.00245, 0.00282
	White	Men	42	0.00172, 0.00198, 0.00226, 0.00259, 0.00297
	White	Men	43	0.00188, 0.00214, 0.00243, 0.00277, 0.00315
	White	Men	44	0.00207, 0.00234, 0.00264, 0.00298, 0.00337
	White	Men	45	0.00227, 0.00256, 0.00289, 0.00325, 0.00367
	White	Men	46	0.00249, 0.00282, 0.00318, 0.00359, 0.00406
	White	Men	47	0.00273, 0.0031, 0.00352, 0.004, 0.00454
	White	Men	48	0.003, 0.00343, 0.00391, 0.00445, 0.00508
	White	Men	49	0.00332, 0.0038, 0.00433, 0.00494, 0.00564
	White	Men	50	0.00369, 0.00421, 0.00479, 0.00545, 0.00621
	White	Men	51	0.00413, 0.00467, 0.00528, 0.00596, 0.00675
	White	Men	52	0.00462, 0.00517, 0.00579, 0.00648, 0.00726
	White	Men	53	0.00516, 0.00571, 0.00632, 0.007, 0.00775
	White	Men	54	0.0057, 0.00626, 0.00687, 0.00754, 0.00828
	White	Men	55	0.00621, 0.00679, 0.00743, 0.00812, 0.00889
	White	Men	56	0.00666, 0.0073, 0.008, 0.00876, 0.00961
	White	Men	57	0.00709, 0.0078, 0.00857, 0.00942, 0.0104
	White	Men	58	0.00756, 0.00832, 0.00915, 0.0101, 0.0111
	White	Men	59	0.00807, 0.00887, 0.00974, 0.0107, 0.0117
	White	Men	60	0.00864, 0.00945, 0.0103, 0.0113, 0.0124
	White	Men	61	0.00925, 0.0101, 0.0109, 0.0119, 0.0129
	White	Men	62	0.00991, 0.0107, 0.0116, 0.0125, 0.0135
	White	Men	63	0.0106, 0.0114, 0.0123, 0.0132, 0.0142
	White	Men	64	0.0113, 0.0121, 0.013, 0.014, 0.015
	White	Men	65	0.012, 0.0129, 0.0138, 0.0149, 0.016
	White	Men	66	0.0128, 0.0138, 0.0148, 0.0159, 0.0172
	White	Men	67	0.0137, 0.0148, 0.0159, 0.0172, 0.0186
	White	Men	68	0.0147, 0.016, 0.0173, 0.0187, 0.0202
	White	Men	69	0.016, 0.0173, 0.0187, 0.0203, 0.022
	White	Men	70	0.0174, 0.0188, 0.0204, 0.0222, 0.0241
	White	Men	71	0.0189, 0.0206, 0.0223, 0.0242, 0.0263
	White	Men	72	0.0207, 0.0225, 0.0244, 0.0264, 0.0287
	White	Men	73	0.0227, 0.0246, 0.0266, 0.0288, 0.0313

Variable	Race/ethnicity	Sex	Age	Distribution
	White	Men	74	0.0249, 0.0269, 0.0291, 0.0314, 0.034
	White	Men	75	0.0273, 0.0295, 0.0318, 0.0343, 0.037
	White	Men	76	0.0302, 0.0325, 0.0349, 0.0375, 0.0404
	White	Men	77	0.0334, 0.0358, 0.0385, 0.0413, 0.0444
	White	Men	78	0.037, 0.0397, 0.0427, 0.0458, 0.0493
	White	Men	79	0.0411, 0.0442, 0.0476, 0.0511, 0.055
	White	Men	80	0.0458, 0.0494, 0.0532, 0.0572, 0.0617
	White	Men	81	0.0511, 0.0551, 0.0594, 0.0641, 0.0692
	White	Men	82	0.0566, 0.0613, 0.0663, 0.0716, 0.0775
	White	Men	83	0.0623, 0.0676, 0.0734, 0.0796, 0.0865
	White	Men	84	0.0679, 0.074, 0.0805, 0.0877, 0.0956
	White	Women	30	0.000586, 0.000663, 0.000749, 0.000846, 0.000957
	White	Women	31	0.000617, 0.0007, 0.000793, 0.000898, 0.00102
	White	Women	32	0.000652, 0.000739, 0.000837, 0.000947, 0.00107
	White	Women	33	0.000687, 0.000778, 0.000879, 0.000994, 0.00113
	White	Women	34	0.000714, 0.000811, 0.000921, 0.00105, 0.00119
	White	Women	35	0.000733, 0.000841, 0.000964, 0.00111, 0.00127
	White	Women	36	0.000753, 0.000873, 0.00101, 0.00117, 0.00136
	White	Women	37	0.000778, 0.000909, 0.00106, 0.00124, 0.00145
	White	Women	38	0.000807, 0.000951, 0.00112, 0.00131, 0.00155
	White	Women	39	0.000843, 0.000999, 0.00118, 0.0014, 0.00166
	White	Women	40	0.000888, 0.00106, 0.00126, 0.00149, 0.00178
	White	Women	41	0.000948, 0.00113, 0.00134, 0.0016, 0.00191
	White	Women	42	0.00102, 0.00122, 0.00145, 0.00172, 0.00205
	White	Women	43	0.00112, 0.00132, 0.00157, 0.00186, 0.00221
	White	Women	44	0.00122, 0.00145, 0.00171, 0.00203, 0.00241
	White	Women	45	0.00134, 0.00159, 0.00188, 0.00222, 0.00264
	White	Women	46	0.00147, 0.00175, 0.00207, 0.00244, 0.0029
	White	Women	47	0.00164, 0.00193, 0.00228, 0.00268, 0.00316
	White	Women	48	0.00184, 0.00215, 0.00251, 0.00293, 0.00342
	White	Women	49	0.00206, 0.00239, 0.00276, 0.00319, 0.00369
	White	Women	50	0.00228, 0.00263, 0.00303, 0.00348, 0.00402
	White	Women	51	0.00248, 0.00287, 0.00331, 0.00382, 0.00442
	White	Women	52	0.00267, 0.0031, 0.00361, 0.00419, 0.00488
	White	Women	53	0.00287, 0.00335, 0.00391, 0.00456, 0.00533
	White	Women	54	0.00311, 0.00362, 0.00421, 0.00489, 0.00569
	White	Women	55	0.00343, 0.00394, 0.00451, 0.00516, 0.00591
	White	Women	56	0.00382, 0.00428, 0.0048, 0.00537, 0.00602
	White	Women	57	0.00424, 0.00465, 0.00509, 0.00557, 0.0061
	White	Women	58	0.00465, 0.00501, 0.00539, 0.00579, 0.00624
	White	Women	59	0.00499, 0.00534, 0.0057, 0.0061, 0.00652
	White	Women	60	0.00529, 0.00566, 0.00605, 0.00647, 0.00693
	White	Women	61	0.00561, 0.00602, 0.00645, 0.00691, 0.00741

Variable	Race/ethnicity	Sex	Age	Distribution
	White	Women	62	0.00598, 0.00643, 0.0069, 0.00741, 0.00797
	White	Women	63	0.00642, 0.00691, 0.00743, 0.00799, 0.0086
	White	Women	64	0.00693, 0.00747, 0.00804, 0.00865, 0.00932
	White	Women	65	0.00751, 0.00811, 0.00874, 0.00942, 0.0102
	White	Women	66	0.00816, 0.00882, 0.00954, 0.0103, 0.0112
	White	Women	67	0.00886, 0.00962, 0.0104, 0.0113, 0.0123
	White	Women	68	0.00961, 0.0105, 0.0115, 0.0125, 0.0137
	White	Women	69	0.0105, 0.0115, 0.0126, 0.0138, 0.0152
	White	Women	70	0.0114, 0.0126, 0.0138, 0.0153, 0.0168
	White	Women	71	0.0124, 0.0138, 0.0152, 0.0168, 0.0186
	White	Women	72	0.0136, 0.0151, 0.0167, 0.0185, 0.0205
	White	Women	73	0.015, 0.0166, 0.0184, 0.0204, 0.0226
	White	Women	74	0.0166, 0.0183, 0.0202, 0.0224, 0.0247
	White	Women	75	0.0184, 0.0203, 0.0223, 0.0246, 0.027
	White	Women	76	0.0206, 0.0226, 0.0247, 0.027, 0.0296
	White	Women	77	0.0232, 0.0253, 0.0275, 0.0299, 0.0326
	White	Women	78	0.0261, 0.0283, 0.0307, 0.0333, 0.0361
	White	Women	79	0.0294, 0.0318, 0.0344, 0.0373, 0.0404
	White	Women	80	0.033, 0.0357, 0.0387, 0.0419, 0.0454
	White	Women	81	0.037, 0.0401, 0.0435, 0.0471, 0.051
	White	Women	82	0.0413, 0.0448, 0.0487, 0.0528, 0.0574
	White	Women	83	0.0457, 0.0498, 0.0542, 0.059, 0.0644
	White	Women	84	0.0502, 0.0548, 0.0599, 0.0655, 0.0716
Total mortality rates for 2024 (0.01, 0.2, 0.5, 0.8. 0.99 percentiles of the empirical distribution produced during forecasting)				
	Black	Men	30	0.00164, 0.00195, 0.00231, 0.00273, 0.00323
	Black	Men	31	0.00174, 0.00202, 0.00235, 0.00273, 0.00317
	Black	Men	32	0.00182, 0.00209, 0.00239, 0.00273, 0.00314
	Black	Men	33	0.00186, 0.00213, 0.00243, 0.00277, 0.00317
	Black	Men	34	0.0019, 0.00217, 0.00248, 0.00284, 0.00325
	Black	Men	35	0.00194, 0.00222, 0.00254, 0.00291, 0.00333
	Black	Men	36	0.00198, 0.00227, 0.0026, 0.00298, 0.00342
	Black	Men	37	0.00203, 0.00233, 0.00267, 0.00305, 0.0035
	Black	Men	38	0.00208, 0.00239, 0.00273, 0.00313, 0.00359
	Black	Men	39	0.00212, 0.00244, 0.00281, 0.00323, 0.00372
	Black	Men	40	0.00215, 0.0025, 0.0029, 0.00336, 0.0039
	Black	Men	41	0.00219, 0.00257, 0.00302, 0.00354, 0.00415
	Black	Men	42	0.00226, 0.00268, 0.00318, 0.00376, 0.00447
	Black	Men	43	0.00235, 0.00282, 0.00338, 0.00405, 0.00486
	Black	Men	44	0.00247, 0.003, 0.00364, 0.00441, 0.00535
	Black	Men	45	0.00263, 0.00322, 0.00395, 0.00483, 0.00592
	Black	Men	46	0.00283, 0.0035, 0.00431, 0.00531, 0.00655
	Black	Men	47	0.00311, 0.00384, 0.00473, 0.00584, 0.00722
	Black	Men	48	0.00345, 0.00425, 0.00522, 0.00641, 0.00789

Variable	Race/ethnicity	Sex	Age	Distribution
	Black	Men	49	0.00389, 0.00474, 0.00578, 0.00703, 0.00859
	Black	Men	50	0.00439, 0.00531, 0.0064, 0.00772, 0.00933
	Black	Men	51	0.00498, 0.00595, 0.0071, 0.00847, 0.0101
	Black	Men	52	0.00565, 0.00667, 0.00786, 0.00926, 0.0109
	Black	Men	53	0.00643, 0.00748, 0.00869, 0.0101, 0.0117
	Black	Men	54	0.00732, 0.00838, 0.00958, 0.011, 0.0125
	Black	Men	55	0.00828, 0.00935, 0.0105, 0.0119, 0.0134
	Black	Men	56	0.00932, 0.0104, 0.0116, 0.0129, 0.0143
	Black	Men	57	0.0104, 0.0115, 0.0126, 0.0139, 0.0153
	Black	Men	58	0.0116, 0.0126, 0.0138, 0.015, 0.0163
	Black	Men	59	0.0127, 0.0138, 0.0149, 0.0161, 0.0175
	Black	Men	60	0.0138, 0.0149, 0.0161, 0.0174, 0.0188
	Black	Men	61	0.0148, 0.016, 0.0172, 0.0185, 0.02
	Black	Men	62	0.0159, 0.0171, 0.0183, 0.0196, 0.0211
	Black	Men	63	0.0168, 0.018, 0.0193, 0.0207, 0.0222
	Black	Men	64	0.0177, 0.019, 0.0204, 0.0219, 0.0235
	Black	Men	65	0.0185, 0.0199, 0.0214, 0.023, 0.0247
	Black	Men	66	0.0194, 0.0209, 0.0225, 0.0243, 0.0261
	Black	Men	67	0.0204, 0.022, 0.0238, 0.0257, 0.0277
	Black	Men	68	0.0215, 0.0233, 0.0252, 0.0273, 0.0296
	Black	Men	69	0.0227, 0.0247, 0.0269, 0.0292, 0.0317
	Black	Men	70	0.0242, 0.0264, 0.0287, 0.0313, 0.0341
	Black	Men	71	0.0258, 0.0282, 0.0308, 0.0336, 0.0367
	Black	Men	72	0.0277, 0.0303, 0.0331, 0.0361, 0.0395
	Black	Men	73	0.0298, 0.0326, 0.0355, 0.0388, 0.0424
	Black	Men	74	0.0322, 0.0351, 0.0382, 0.0416, 0.0454
	Black	Men	75	0.0349, 0.0379, 0.0411, 0.0446, 0.0484
	Black	Men	76	0.0378, 0.0409, 0.0443, 0.0479, 0.0518
	Black	Men	77	0.041, 0.0443, 0.0478, 0.0515, 0.0556
	Black	Men	78	0.0444, 0.048, 0.0517, 0.0558, 0.0602
	Black	Men	79	0.048, 0.0519, 0.0561, 0.0606, 0.0656
	Black	Men	80	0.0519, 0.0562, 0.061, 0.0661, 0.0717
	Black	Men	81	0.056, 0.0609, 0.0662, 0.0719, 0.0783
	Black	Men	82	0.0601, 0.0657, 0.0716, 0.0781, 0.0853
	Black	Men	83	0.0642, 0.0704, 0.0771, 0.0845, 0.0926
	Black	Men	84	0.0681, 0.075, 0.0825, 0.0907, 0.0999
	Black	Women	30	0.000593, 0.000734, 0.000906, 0.00112, 0.00138
	Black	Women	31	0.000664, 0.000806, 0.000976, 0.00118, 0.00144
	Black	Women	32	0.000747, 0.000888, 0.00105, 0.00125, 0.00149
	Black	Women	33	0.000832, 0.000972, 0.00113, 0.00132, 0.00155
	Black	Women	34	0.000903, 0.00105, 0.00121, 0.00141, 0.00163
	Black	Women	35	0.000955, 0.00111, 0.00129, 0.0015, 0.00174
	Black	Women	36	0.000998, 0.00117, 0.00136, 0.00159, 0.00185

Variable	Race/ethnicity	Sex	Age	Distribution
	Black	Women	37	0.00104, 0.00122, 0.00143, 0.00168, 0.00197
	Black	Women	38	0.0011, 0.00129, 0.00151, 0.00177, 0.00208
	Black	Women	39	0.00117, 0.00137, 0.0016, 0.00188, 0.0022
	Black	Women	40	0.00126, 0.00147, 0.00171, 0.002, 0.00234
	Black	Women	41	0.00137, 0.00159, 0.00184, 0.00214, 0.00249
	Black	Women	42	0.00151, 0.00174, 0.002, 0.0023, 0.00264
	Black	Women	43	0.00169, 0.00191, 0.00217, 0.00246, 0.0028
	Black	Women	44	0.00189, 0.00212, 0.00237, 0.00265, 0.00297
	Black	Women	45	0.00211, 0.00234, 0.0026, 0.00288, 0.00319
	Black	Women	46	0.00232, 0.00258, 0.00285, 0.00316, 0.00351
	Black	Women	47	0.00254, 0.00283, 0.00315, 0.0035, 0.0039
	Black	Women	48	0.00277, 0.00311, 0.00348, 0.00389, 0.00436
	Black	Women	49	0.00306, 0.00343, 0.00384, 0.00431, 0.00483
	Black	Women	50	0.00341, 0.00381, 0.00425, 0.00474, 0.00529
	Black	Women	51	0.00384, 0.00424, 0.00468, 0.00516, 0.0057
	Black	Women	52	0.00435, 0.00473, 0.00514, 0.00559, 0.00608
	Black	Women	53	0.00487, 0.00524, 0.00563, 0.00605, 0.00651
	Black	Women	54	0.00536, 0.00574, 0.00614, 0.00657, 0.00704
	Black	Women	55	0.00584, 0.00624, 0.00668, 0.00714, 0.00764
	Black	Women	56	0.0063, 0.00675, 0.00723, 0.00774, 0.00829
	Black	Women	57	0.00678, 0.00726, 0.00778, 0.00834, 0.00894
	Black	Women	58	0.00724, 0.00777, 0.00833, 0.00893, 0.00959
	Black	Women	59	0.00767, 0.00825, 0.00887, 0.00953, 0.0102
	Black	Women	60	0.00807, 0.00871, 0.00938, 0.0101, 0.0109
	Black	Women	61	0.00849, 0.00917, 0.00989, 0.0107, 0.0115
	Black	Women	62	0.00894, 0.00965, 0.0104, 0.0112, 0.0121
	Black	Women	63	0.0094, 0.0102, 0.011, 0.0118, 0.0128
	Black	Women	64	0.00989, 0.0107, 0.0116, 0.0125, 0.0136
	Black	Women	65	0.0104, 0.0113, 0.0123, 0.0133, 0.0145
	Black	Women	66	0.0111, 0.0121, 0.0131, 0.0143, 0.0155
	Black	Women	67	0.0119, 0.0129, 0.0141, 0.0153, 0.0167
	Black	Women	68	0.0128, 0.0139, 0.0152, 0.0165, 0.018
	Black	Women	69	0.0138, 0.015, 0.0164, 0.0179, 0.0195
	Black	Women	70	0.0149, 0.0163, 0.0177, 0.0193, 0.0211
	Black	Women	71	0.0161, 0.0176, 0.0192, 0.0209, 0.0228
	Black	Women	72	0.0174, 0.019, 0.0207, 0.0226, 0.0247
	Black	Women	73	0.0189, 0.0206, 0.0224, 0.0244, 0.0266
	Black	Women	74	0.0205, 0.0223, 0.0243, 0.0264, 0.0287
	Black	Women	75	0.0224, 0.0243, 0.0264, 0.0286, 0.031
	Black	Women	76	0.0246, 0.0266, 0.0287, 0.0311, 0.0336
	Black	Women	77	0.027, 0.0291, 0.0314, 0.0339, 0.0366
	Black	Women	78	0.0296, 0.032, 0.0345, 0.0372, 0.0401
	Black	Women	79	0.0326, 0.0352, 0.038, 0.041, 0.0442

Variable	Race/ethnicity	Sex	Age	Distribution
	Black	Women	80	0.0358, 0.0387, 0.0418, 0.0452, 0.0489
	Black	Women	81	0.0391, 0.0424, 0.046, 0.0499, 0.0541
	Black	Women	82	0.0425, 0.0463, 0.0504, 0.0549, 0.0599
	Black	Women	83	0.0458, 0.0503, 0.055, 0.0603, 0.0661
	Black	Women	84	0.0492, 0.0542, 0.0597, 0.0657, 0.0724
	Hispanic	Men	30	0.000794, 0.000896, 0.00101, 0.00114, 0.00129
	Hispanic	Men	31	0.00081, 0.000914, 0.00103, 0.00116, 0.00131
	Hispanic	Men	32	0.00082, 0.000927, 0.00105, 0.00118, 0.00134
	Hispanic	Men	33	0.000814, 0.000932, 0.00106, 0.00122, 0.00139
	Hispanic	Men	34	0.000804, 0.000933, 0.00108, 0.00125, 0.00145
	Hispanic	Men	35	0.000801, 0.000938, 0.0011, 0.00128, 0.0015
	Hispanic	Men	36	0.000811, 0.000953, 0.00112, 0.00131, 0.00154
	Hispanic	Men	37	0.000837, 0.000981, 0.00115, 0.00134, 0.00158
	Hispanic	Men	38	0.000875, 0.00102, 0.00119, 0.00138, 0.00161
	Hispanic	Men	39	0.000919, 0.00107, 0.00124, 0.00144, 0.00167
	Hispanic	Men	40	0.000969, 0.00112, 0.0013, 0.00151, 0.00175
	Hispanic	Men	41	0.00103, 0.00119, 0.00138, 0.0016, 0.00186
	Hispanic	Men	42	0.00111, 0.00129, 0.00148, 0.00171, 0.00197
	Hispanic	Men	43	0.00123, 0.00141, 0.0016, 0.00183, 0.00209
	Hispanic	Men	44	0.00139, 0.00156, 0.00175, 0.00197, 0.00221
	Hispanic	Men	45	0.00157, 0.00175, 0.00193, 0.00214, 0.00237
	Hispanic	Men	46	0.00176, 0.00195, 0.00214, 0.00236, 0.00261
	Hispanic	Men	47	0.00196, 0.00217, 0.00239, 0.00263, 0.0029
	Hispanic	Men	48	0.00218, 0.00241, 0.00266, 0.00294, 0.00325
	Hispanic	Men	49	0.00242, 0.00267, 0.00296, 0.00327, 0.00362
	Hispanic	Men	50	0.00269, 0.00297, 0.00328, 0.00362, 0.00399
	Hispanic	Men	51	0.00303, 0.00331, 0.00362, 0.00395, 0.00432
	Hispanic	Men	52	0.00345, 0.0037, 0.00398, 0.00427, 0.00459
	Hispanic	Men	53	0.00392, 0.00413, 0.00435, 0.00458, 0.00482
	Hispanic	Men	54	0.00436, 0.00455, 0.00474, 0.00494, 0.00516
	Hispanic	Men	55	0.00468, 0.00491, 0.00515, 0.0054, 0.00567
	Hispanic	Men	56	0.00498, 0.00527, 0.00558, 0.0059, 0.00625
	Hispanic	Men	57	0.00529, 0.00565, 0.00602, 0.00642, 0.00686
	Hispanic	Men	58	0.00563, 0.00605, 0.00649, 0.00696, 0.00747
	Hispanic	Men	59	0.00598, 0.00645, 0.00696, 0.0075, 0.0081
	Hispanic	Men	60	0.00635, 0.00687, 0.00744, 0.00805, 0.00872
	Hispanic	Men	61	0.00676, 0.00733, 0.00793, 0.00859, 0.00931
	Hispanic	Men	62	0.00723, 0.00781, 0.00844, 0.00911, 0.00984
	Hispanic	Men	63	0.00773, 0.00833, 0.00896, 0.00965, 0.0104
	Hispanic	Men	64	0.00823, 0.00886, 0.00953, 0.0102, 0.011
	Hispanic	Men	65	0.00873, 0.00942, 0.0102, 0.0109, 0.0118
	Hispanic	Men	66	0.00927, 0.01, 0.0109, 0.0117, 0.0127
	Hispanic	Men	67	0.00989, 0.0107, 0.0117, 0.0127, 0.0138

Variable	Race/ethnicity	Sex	Age	Distribution
	Hispanic	Men	68	0.0106, 0.0116, 0.0126, 0.0137, 0.015
	Hispanic	Men	69	0.0114, 0.0125, 0.0136, 0.0149, 0.0163
	Hispanic	Men	70	0.0124, 0.0136, 0.0148, 0.0162, 0.0178
	Hispanic	Men	71	0.0135, 0.0148, 0.0162, 0.0177, 0.0194
	Hispanic	Men	72	0.0146, 0.0161, 0.0176, 0.0193, 0.0212
	Hispanic	Men	73	0.016, 0.0175, 0.0192, 0.0211, 0.0232
	Hispanic	Men	74	0.0175, 0.0192, 0.021, 0.0231, 0.0253
	Hispanic	Men	75	0.0192, 0.021, 0.023, 0.0252, 0.0276
	Hispanic	Men	76	0.0212, 0.0232, 0.0253, 0.0277, 0.0303
	Hispanic	Men	77	0.0234, 0.0256, 0.028, 0.0306, 0.0334
	Hispanic	Men	78	0.026, 0.0285, 0.0311, 0.0339, 0.0371
	Hispanic	Men	79	0.029, 0.0317, 0.0346, 0.0378, 0.0413
	Hispanic	Men	80	0.0325, 0.0354, 0.0387, 0.0422, 0.046
	Hispanic	Men	81	0.0363, 0.0396, 0.0431, 0.047, 0.0513
	Hispanic	Men	82	0.0402, 0.0439, 0.0479, 0.0523, 0.0571
	Hispanic	Men	83	0.0441, 0.0483, 0.0528, 0.0578, 0.0633
	Hispanic	Men	84	0.0478, 0.0525, 0.0577, 0.0633, 0.0696
	Hispanic	Women	30	0.000327, 0.000369, 0.000416, 0.000468, 0.000528
	Hispanic	Women	31	0.000346, 0.000389, 0.000437, 0.000491, 0.000552
	Hispanic	Women	32	0.000366, 0.00041, 0.000459, 0.000513, 0.000575
	Hispanic	Women	33	0.000385, 0.000431, 0.000481, 0.000537, 0.0006
	Hispanic	Women	34	0.000401, 0.000449, 0.000503, 0.000564, 0.000632
	Hispanic	Women	35	0.000413, 0.000466, 0.000526, 0.000594, 0.000671
	Hispanic	Women	36	0.000425, 0.000484, 0.000551, 0.000627, 0.000713
	Hispanic	Women	37	0.000442, 0.000506, 0.000579, 0.000661, 0.000757
	Hispanic	Women	38	0.000465, 0.000533, 0.00061, 0.000699, 0.000801
	Hispanic	Women	39	0.000496, 0.000567, 0.000647, 0.000739, 0.000845
	Hispanic	Women	40	0.000534, 0.000608, 0.00069, 0.000784, 0.000891
	Hispanic	Women	41	0.000582, 0.000657, 0.000741, 0.000836, 0.000943
	Hispanic	Women	42	0.00064, 0.000717, 0.000801, 0.000896, 0.001
	Hispanic	Women	43	0.00071, 0.000788, 0.000873, 0.000967, 0.00107
	Hispanic	Women	44	0.000792, 0.000871, 0.000957, 0.00105, 0.00116
	Hispanic	Women	45	0.000883, 0.000966, 0.00105, 0.00115, 0.00126
	Hispanic	Women	46	0.000981, 0.00107, 0.00117, 0.00127, 0.00139
	Hispanic	Women	47	0.00109, 0.00119, 0.00129, 0.00141, 0.00154
	Hispanic	Women	48	0.0012, 0.00131, 0.00144, 0.00157, 0.00172
	Hispanic	Women	49	0.00133, 0.00145, 0.00159, 0.00174, 0.00191
	Hispanic	Women	50	0.00147, 0.00161, 0.00176, 0.00193, 0.00211
	Hispanic	Women	51	0.00163, 0.00178, 0.00194, 0.00212, 0.00231
	Hispanic	Women	52	0.00182, 0.00197, 0.00213, 0.00231, 0.00251
	Hispanic	Women	53	0.00202, 0.00217, 0.00234, 0.00252, 0.00272
	Hispanic	Women	54	0.00221, 0.00238, 0.00256, 0.00275, 0.00296
	Hispanic	Women	55	0.0024, 0.00258, 0.00279, 0.003, 0.00324

Variable	Race/ethnicity	Sex	Age	Distribution
	Hispanic	Women	56	0.00259, 0.0028, 0.00303, 0.00327, 0.00354
	Hispanic	Women	57	0.0028, 0.00303, 0.00328, 0.00355, 0.00384
	Hispanic	Women	58	0.00303, 0.00328, 0.00355, 0.00384, 0.00415
	Hispanic	Women	59	0.00326, 0.00354, 0.00383, 0.00415, 0.00449
	Hispanic	Women	60	0.0035, 0.0038, 0.00413, 0.00448, 0.00487
	Hispanic	Women	61	0.00376, 0.00409, 0.00445, 0.00483, 0.00525
	Hispanic	Women	62	0.00405, 0.00441, 0.00479, 0.0052, 0.00566
	Hispanic	Women	63	0.00436, 0.00474, 0.00516, 0.00561, 0.0061
	Hispanic	Women	64	0.00469, 0.00511, 0.00556, 0.00606, 0.0066
	Hispanic	Women	65	0.00504, 0.00551, 0.00601, 0.00656, 0.00717
	Hispanic	Women	66	0.00544, 0.00596, 0.00652, 0.00713, 0.00781
	Hispanic	Women	67	0.00591, 0.00647, 0.00709, 0.00777, 0.00851
	Hispanic	Women	68	0.00645, 0.00707, 0.00774, 0.00848, 0.00929
	Hispanic	Women	69	0.00707, 0.00774, 0.00848, 0.00928, 0.0102
	Hispanic	Women	70	0.00777, 0.00851, 0.00931, 0.0102, 0.0111
	Hispanic	Women	71	0.00856, 0.00936, 0.0102, 0.0112, 0.0122
	Hispanic	Women	72	0.00943, 0.0103, 0.0113, 0.0123, 0.0135
	Hispanic	Women	73	0.0104, 0.0114, 0.0124, 0.0136, 0.0149
	Hispanic	Women	74	0.0116, 0.0126, 0.0138, 0.015, 0.0164
	Hispanic	Women	75	0.0129, 0.0141, 0.0153, 0.0166, 0.018
	Hispanic	Women	76	0.0145, 0.0157, 0.017, 0.0184, 0.0199
	Hispanic	Women	77	0.0163, 0.0176, 0.019, 0.0205, 0.0221
	Hispanic	Women	78	0.0184, 0.0198, 0.0214, 0.023, 0.0248
	Hispanic	Women	79	0.0208, 0.0224, 0.0241, 0.026, 0.028
	Hispanic	Women	80	0.0234, 0.0252, 0.0272, 0.0294, 0.0317
	Hispanic	Women	81	0.0262, 0.0284, 0.0307, 0.0332, 0.036
	Hispanic	Women	82	0.0292, 0.0318, 0.0345, 0.0375, 0.0408
	Hispanic	Women	83	0.0322, 0.0353, 0.0385, 0.0421, 0.0461
	Hispanic	Women	84	0.0353, 0.0388, 0.0426, 0.0468, 0.0515
	White	Men	30	0.00112, 0.00134, 0.0016, 0.00191, 0.00228
	White	Men	31	0.00118, 0.00139, 0.00164, 0.00194, 0.0023
	White	Men	32	0.00123, 0.00144, 0.00168, 0.00196, 0.0023
	White	Men	33	0.00126, 0.00147, 0.00171, 0.00198, 0.0023
	White	Men	34	0.00128, 0.00149, 0.00173, 0.00201, 0.00233
	White	Men	35	0.00129, 0.0015, 0.00175, 0.00204, 0.00237
	White	Men	36	0.0013, 0.00152, 0.00177, 0.00207, 0.00242
	White	Men	37	0.00132, 0.00155, 0.00181, 0.00212, 0.00248
	White	Men	38	0.00135, 0.00159, 0.00186, 0.00217, 0.00254
	White	Men	39	0.00141, 0.00164, 0.00192, 0.00224, 0.00261
	White	Men	40	0.00148, 0.00172, 0.002, 0.00232, 0.0027
	White	Men	41	0.00156, 0.00181, 0.0021, 0.00243, 0.00282
	White	Men	42	0.00167, 0.00193, 0.00223, 0.00257, 0.00296
	White	Men	43	0.00182, 0.00209, 0.00239, 0.00274, 0.00314

Variable	Race/ethnicity	Sex	Age	Distribution
	White	Men	44	0.00201, 0.00228, 0.00259, 0.00295, 0.00335
	White	Men	45	0.00221, 0.00251, 0.00284, 0.00322, 0.00365
	White	Men	46	0.00242, 0.00276, 0.00313, 0.00356, 0.00405
	White	Men	47	0.00266, 0.00304, 0.00347, 0.00396, 0.00453
	White	Men	48	0.00292, 0.00336, 0.00385, 0.00442, 0.00507
	White	Men	49	0.00324, 0.00372, 0.00427, 0.00491, 0.00564
	White	Men	50	0.0036, 0.00413, 0.00473, 0.00542, 0.00621
	White	Men	51	0.00403, 0.00459, 0.00522, 0.00594, 0.00676
	White	Men	52	0.00452, 0.00509, 0.00573, 0.00645, 0.00727
	White	Men	53	0.00506, 0.00563, 0.00627, 0.00697, 0.00776
	White	Men	54	0.0056, 0.00618, 0.00681, 0.00751, 0.00829
	White	Men	55	0.0061, 0.00671, 0.00737, 0.0081, 0.00891
	White	Men	56	0.00654, 0.00721, 0.00793, 0.00873, 0.00962
	White	Men	57	0.00697, 0.0077, 0.0085, 0.00939, 0.0104
	White	Men	58	0.00742, 0.00821, 0.00907, 0.01, 0.0111
	White	Men	59	0.00792, 0.00875, 0.00965, 0.0106, 0.0118
	White	Men	60	0.00847, 0.00931, 0.0102, 0.0112, 0.0124
	White	Men	61	0.00907, 0.00991, 0.0108, 0.0118, 0.0129
	White	Men	62	0.00972, 0.0105, 0.0114, 0.0124, 0.0135
	White	Men	63	0.0104, 0.0112, 0.0121, 0.013, 0.0141
	White	Men	64	0.011, 0.0119, 0.0128, 0.0138, 0.0149
	White	Men	65	0.0117, 0.0127, 0.0136, 0.0147, 0.0158
	White	Men	66	0.0125, 0.0135, 0.0146, 0.0157, 0.017
	White	Men	67	0.0134, 0.0145, 0.0157, 0.017, 0.0184
	White	Men	68	0.0144, 0.0156, 0.017, 0.0184, 0.02
	White	Men	69	0.0156, 0.017, 0.0185, 0.0201, 0.0218
	White	Men	70	0.017, 0.0185, 0.0201, 0.0219, 0.0239
	White	Men	71	0.0185, 0.0202, 0.022, 0.0239, 0.0261
	White	Men	72	0.0202, 0.022, 0.024, 0.0261, 0.0284
	White	Men	73	0.0221, 0.0241, 0.0262, 0.0285, 0.031
	White	Men	74	0.0243, 0.0264, 0.0286, 0.031, 0.0337
	White	Men	75	0.0267, 0.0289, 0.0313, 0.0338, 0.0366
	White	Men	76	0.0295, 0.0318, 0.0343, 0.037, 0.04
	White	Men	77	0.0326, 0.0352, 0.0379, 0.0408, 0.044
	White	Men	78	0.0361, 0.039, 0.042, 0.0452, 0.0488
	White	Men	79	0.0402, 0.0434, 0.0468, 0.0505, 0.0545
	White	Men	80	0.0448, 0.0485, 0.0523, 0.0565, 0.0611
	White	Men	81	0.05, 0.0541, 0.0585, 0.0633, 0.0686
	White	Men	82	0.0554, 0.0602, 0.0653, 0.0708, 0.0769
	White	Men	83	0.0609, 0.0664, 0.0723, 0.0787, 0.0858
	White	Men	84	0.0664, 0.0726, 0.0794, 0.0868, 0.0949
	White	Women	30	0.000579, 0.000658, 0.000747, 0.000847, 0.000963
	White	Women	31	0.00061, 0.000695, 0.000791, 0.0009, 0.00103

Variable	Race/ethnicity	Sex	Age	Distribution
	White	Women	32	0.000644, 0.000734, 0.000834, 0.000949, 0.00108
	White	Women	33	0.000678, 0.000771, 0.000876, 0.000995, 0.00113
	White	Women	34	0.000703, 0.000804, 0.000917, 0.00105, 0.0012
	White	Women	35	0.00072, 0.000831, 0.000959, 0.00111, 0.00128
	White	Women	36	0.000737, 0.00086, 0.001, 0.00117, 0.00136
	White	Women	37	0.000759, 0.000894, 0.00105, 0.00123, 0.00145
	White	Women	38	0.000786, 0.000932, 0.0011, 0.00131, 0.00155
	White	Women	39	0.000818, 0.000977, 0.00117, 0.00139, 0.00166
	White	Women	40	0.00086, 0.00103, 0.00124, 0.00148, 0.00178
	White	Women	41	0.000917, 0.0011, 0.00132, 0.00159, 0.00191
	White	Women	42	0.000989, 0.00119, 0.00142, 0.0017, 0.00204
	White	Women	43	0.00108, 0.00129, 0.00154, 0.00184, 0.00221
	White	Women	44	0.00118, 0.00141, 0.00168, 0.00201, 0.0024
	White	Women	45	0.00129, 0.00154, 0.00184, 0.0022, 0.00263
	White	Women	46	0.00142, 0.0017, 0.00203, 0.00242, 0.00289
	White	Women	47	0.00158, 0.00188, 0.00224, 0.00266, 0.00316
	White	Women	48	0.00178, 0.0021, 0.00247, 0.0029, 0.00342
	White	Women	49	0.002, 0.00233, 0.00272, 0.00316, 0.00369
	White	Women	50	0.00222, 0.00258, 0.00299, 0.00346, 0.00402
	White	Women	51	0.00242, 0.00281, 0.00327, 0.0038, 0.00443
	White	Women	52	0.0026, 0.00305, 0.00357, 0.00418, 0.0049
	White	Women	53	0.0028, 0.00329, 0.00387, 0.00455, 0.00536
	White	Women	54	0.00304, 0.00356, 0.00417, 0.00488, 0.00572
	White	Women	55	0.00336, 0.00388, 0.00447, 0.00515, 0.00594
	White	Women	56	0.00375, 0.00423, 0.00476, 0.00535, 0.00603
	White	Women	57	0.00418, 0.00459, 0.00505, 0.00554, 0.0061
	White	Women	58	0.00458, 0.00495, 0.00534, 0.00576, 0.00622
	White	Women	59	0.00492, 0.00527, 0.00565, 0.00606, 0.0065
	White	Women	60	0.0052, 0.00559, 0.006, 0.00643, 0.00691
	White	Women	61	0.00552, 0.00594, 0.00638, 0.00686, 0.00738
	White	Women	62	0.00588, 0.00634, 0.00682, 0.00735, 0.00792
	White	Women	63	0.00631, 0.00681, 0.00734, 0.00791, 0.00854
	White	Women	64	0.00681, 0.00735, 0.00793, 0.00856, 0.00924
	White	Women	65	0.00737, 0.00797, 0.00861, 0.00931, 0.0101
	White	Women	66	0.00799, 0.00867, 0.0094, 0.0102, 0.0111
	White	Women	67	0.00867, 0.00944, 0.0103, 0.0112, 0.0122
	White	Women	68	0.0094, 0.0103, 0.0113, 0.0124, 0.0135
	White	Women	69	0.0102, 0.0112, 0.0124, 0.0136, 0.015
	White	Women	70	0.0111, 0.0123, 0.0136, 0.0151, 0.0167
	White	Women	71	0.0121, 0.0135, 0.015, 0.0166, 0.0185
	White	Women	72	0.0133, 0.0148, 0.0164, 0.0183, 0.0203
	White	Women	73	0.0146, 0.0163, 0.0181, 0.0201, 0.0224
	White	Women	74	0.0162, 0.0179, 0.0199, 0.0221, 0.0245

Variable	Race/ethnicity	Sex	Age	Distribution
	White	Women	75	0.018, 0.0199, 0.0219, 0.0242, 0.0268
	White	Women	76	0.0201, 0.0221, 0.0243, 0.0267, 0.0293
	White	Women	77	0.0226, 0.0247, 0.027, 0.0295, 0.0323
	White	Women	78	0.0255, 0.0278, 0.0302, 0.0329, 0.0358
	White	Women	79	0.0287, 0.0312, 0.0339, 0.0368, 0.04
	White	Women	80	0.0322, 0.0351, 0.0381, 0.0414, 0.045
	White	Women	81	0.0362, 0.0394, 0.0428, 0.0465, 0.0506
	White	Women	82	0.0403, 0.044, 0.0479, 0.0522, 0.057
	White	Women	83	0.0446, 0.0488, 0.0534, 0.0584, 0.0639
	White	Women	84	0.049, 0.0538, 0.059, 0.0648, 0.0711
Total mortality rates for 2025 (0.01, 0.2, 0.5, 0.8. 0.99 percentiles of the empirical distribution produced during forecasting)				
	Black	Men	30	0.00162, 0.00193, 0.00231, 0.00275, 0.00328
	Black	Men	31	0.00172, 0.00201, 0.00235, 0.00274, 0.00321
	Black	Men	32	0.0018, 0.00208, 0.00239, 0.00275, 0.00317
	Black	Men	33	0.00185, 0.00212, 0.00243, 0.00279, 0.0032
	Black	Men	34	0.00188, 0.00216, 0.00248, 0.00284, 0.00327
	Black	Men	35	0.00191, 0.0022, 0.00253, 0.00291, 0.00335
	Black	Men	36	0.00195, 0.00225, 0.00259, 0.00298, 0.00343
	Black	Men	37	0.002, 0.0023, 0.00265, 0.00304, 0.00351
	Black	Men	38	0.00204, 0.00235, 0.0027, 0.00311, 0.00359
	Black	Men	39	0.00207, 0.0024, 0.00277, 0.00321, 0.00371
	Black	Men	40	0.0021, 0.00245, 0.00286, 0.00333, 0.00389
	Black	Men	41	0.00213, 0.00252, 0.00297, 0.0035, 0.00414
	Black	Men	42	0.00219, 0.00262, 0.00313, 0.00373, 0.00446
	Black	Men	43	0.00228, 0.00276, 0.00333, 0.00401, 0.00485
	Black	Men	44	0.00239, 0.00293, 0.00358, 0.00437, 0.00534
	Black	Men	45	0.00254, 0.00315, 0.00388, 0.00479, 0.00592
	Black	Men	46	0.00274, 0.00341, 0.00424, 0.00528, 0.00657
	Black	Men	47	0.003, 0.00375, 0.00467, 0.00581, 0.00725
	Black	Men	48	0.00334, 0.00416, 0.00515, 0.00639, 0.00794
	Black	Men	49	0.00377, 0.00464, 0.00571, 0.00702, 0.00865
	Black	Men	50	0.00427, 0.0052, 0.00633, 0.00771, 0.00941
	Black	Men	51	0.00484, 0.00584, 0.00703, 0.00847, 0.0102
	Black	Men	52	0.00551, 0.00656, 0.00779, 0.00927, 0.011
	Black	Men	53	0.00628, 0.00737, 0.00862, 0.0101, 0.0118
	Black	Men	54	0.00716, 0.00826, 0.00952, 0.011, 0.0126
	Black	Men	55	0.00813, 0.00923, 0.0105, 0.0119, 0.0135
	Black	Men	56	0.00916, 0.0103, 0.0115, 0.0129, 0.0144
	Black	Men	57	0.0103, 0.0114, 0.0126, 0.0139, 0.0154
	Black	Men	58	0.0114, 0.0125, 0.0137, 0.0149, 0.0163
	Black	Men	59	0.0126, 0.0136, 0.0148, 0.0161, 0.0175
	Black	Men	60	0.0136, 0.0147, 0.0159, 0.0173, 0.0187
	Black	Men	61	0.0146, 0.0158, 0.017, 0.0184, 0.0199

Variable	Race/ethnicity	Sex	Age	Distribution
	Black	Men	62	0.0156, 0.0168, 0.0181, 0.0195, 0.0209
	Black	Men	63	0.0165, 0.0178, 0.0191, 0.0205, 0.0221
	Black	Men	64	0.0173, 0.0187, 0.0201, 0.0216, 0.0233
	Black	Men	65	0.0181, 0.0196, 0.0211, 0.0227, 0.0245
	Black	Men	66	0.019, 0.0205, 0.0222, 0.024, 0.0259
	Black	Men	67	0.02, 0.0216, 0.0234, 0.0254, 0.0275
	Black	Men	68	0.021, 0.0229, 0.0248, 0.027, 0.0293
	Black	Men	69	0.0222, 0.0243, 0.0264, 0.0288, 0.0314
	Black	Men	70	0.0237, 0.0259, 0.0283, 0.0309, 0.0338
	Black	Men	71	0.0253, 0.0277, 0.0303, 0.0332, 0.0364
	Black	Men	72	0.0271, 0.0297, 0.0326, 0.0357, 0.0391
	Black	Men	73	0.0291, 0.0319, 0.035, 0.0383, 0.042
	Black	Men	74	0.0314, 0.0344, 0.0376, 0.0411, 0.0449
	Black	Men	75	0.0341, 0.0371, 0.0404, 0.044, 0.0479
	Black	Men	76	0.037, 0.0401, 0.0435, 0.0472, 0.0512
	Black	Men	77	0.0401, 0.0435, 0.047, 0.0509, 0.0551
	Black	Men	78	0.0435, 0.0471, 0.0509, 0.0551, 0.0596
	Black	Men	79	0.047, 0.051, 0.0553, 0.0599, 0.065
	Black	Men	80	0.0507, 0.0552, 0.0601, 0.0653, 0.0711
	Black	Men	81	0.0548, 0.0598, 0.0652, 0.0711, 0.0777
	Black	Men	82	0.0588, 0.0645, 0.0706, 0.0773, 0.0847
	Black	Men	83	0.0628, 0.0691, 0.076, 0.0836, 0.092
	Black	Men	84	0.0666, 0.0736, 0.0813, 0.0898, 0.0993
	Black	Women	30	0.000579, 0.000725, 0.000906, 0.00113, 0.00142
	Black	Women	31	0.000651, 0.000798, 0.000976, 0.00119, 0.00146
	Black	Women	32	0.000734, 0.00088, 0.00105, 0.00126, 0.00151
	Black	Women	33	0.000818, 0.000963, 0.00113, 0.00133, 0.00157
	Black	Women	34	0.000887, 0.00104, 0.00121, 0.00141, 0.00165
	Black	Women	35	0.000937, 0.0011, 0.00128, 0.0015, 0.00176
	Black	Women	36	0.000977, 0.00115, 0.00135, 0.00159, 0.00187
	Black	Women	37	0.00102, 0.0012, 0.00142, 0.00168, 0.00198
	Black	Women	38	0.00107, 0.00127, 0.0015, 0.00177, 0.00209
	Black	Women	39	0.00114, 0.00134, 0.00158, 0.00187, 0.00221
	Black	Women	40	0.00122, 0.00144, 0.00169, 0.00199, 0.00235
	Black	Women	41	0.00133, 0.00155, 0.00182, 0.00213, 0.00249
	Black	Women	42	0.00146, 0.0017, 0.00196, 0.00227, 0.00264
	Black	Women	43	0.00164, 0.00187, 0.00214, 0.00244, 0.00278
	Black	Women	44	0.00184, 0.00207, 0.00233, 0.00262, 0.00295
	Black	Women	45	0.00206, 0.00229, 0.00255, 0.00284, 0.00317
	Black	Women	46	0.00226, 0.00252, 0.00281, 0.00313, 0.00348
	Black	Women	47	0.00247, 0.00277, 0.0031, 0.00347, 0.00388
	Black	Women	48	0.00271, 0.00305, 0.00343, 0.00386, 0.00434
	Black	Women	49	0.00298, 0.00337, 0.0038, 0.00428, 0.00483

Variable	Race/ethnicity	Sex	Age	Distribution
	Black	Women	50	0.00333, 0.00374, 0.0042, 0.00471, 0.00529
	Black	Women	51	0.00377, 0.00418, 0.00463, 0.00513, 0.00569
	Black	Women	52	0.00427, 0.00467, 0.00509, 0.00556, 0.00608
	Black	Women	53	0.00479, 0.00517, 0.00558, 0.00602, 0.00651
	Black	Women	54	0.00528, 0.00568, 0.0061, 0.00654, 0.00703
	Black	Women	55	0.00576, 0.00618, 0.00663, 0.00711, 0.00763
	Black	Women	56	0.00622, 0.00668, 0.00718, 0.0077, 0.00828
	Black	Women	57	0.00669, 0.00719, 0.00773, 0.0083, 0.00893
	Black	Women	58	0.00714, 0.00769, 0.00827, 0.00889, 0.00957
	Black	Women	59	0.00756, 0.00816, 0.00879, 0.00948, 0.0102
	Black	Women	60	0.00795, 0.0086, 0.0093, 0.01, 0.0109
	Black	Women	61	0.00835, 0.00904, 0.00979, 0.0106, 0.0115
	Black	Women	62	0.00877, 0.0095, 0.0103, 0.0111, 0.0121
	Black	Women	63	0.00922, 0.00999, 0.0108, 0.0117, 0.0127
	Black	Women	64	0.00969, 0.0105, 0.0114, 0.0124, 0.0135
	Black	Women	65	0.0102, 0.0111, 0.0121, 0.0132, 0.0144
	Black	Women	66	0.0109, 0.0119, 0.0129, 0.0141, 0.0154
	Black	Women	67	0.0116, 0.0127, 0.0139, 0.0151, 0.0165
	Black	Women	68	0.0125, 0.0137, 0.0149, 0.0163, 0.0179
	Black	Women	69	0.0135, 0.0148, 0.0161, 0.0177, 0.0193
	Black	Women	70	0.0146, 0.0159, 0.0175, 0.0191, 0.0209
	Black	Women	71	0.0157, 0.0172, 0.0189, 0.0206, 0.0226
	Black	Women	72	0.017, 0.0186, 0.0204, 0.0223, 0.0244
	Black	Women	73	0.0185, 0.0202, 0.0221, 0.0241, 0.0264
	Black	Women	74	0.0201, 0.0219, 0.0239, 0.026, 0.0284
	Black	Women	75	0.0219, 0.0238, 0.0259, 0.0282, 0.0307
	Black	Women	76	0.024, 0.0261, 0.0283, 0.0306, 0.0332
	Black	Women	77	0.0264, 0.0286, 0.0309, 0.0334, 0.0362
	Black	Women	78	0.029, 0.0314, 0.0339, 0.0367, 0.0397
	Black	Women	79	0.0319, 0.0345, 0.0374, 0.0404, 0.0438
	Black	Women	80	0.035, 0.038, 0.0412, 0.0446, 0.0484
	Black	Women	81	0.0383, 0.0417, 0.0453, 0.0493, 0.0536
	Black	Women	82	0.0416, 0.0455, 0.0497, 0.0543, 0.0594
	Black	Women	83	0.0449, 0.0494, 0.0542, 0.0596, 0.0655
	Black	Women	84	0.0482, 0.0533, 0.0588, 0.065, 0.0719
	Hispanic	Men	30	0.000792, 0.000895, 0.00101, 0.00114, 0.00129
	Hispanic	Men	31	0.000809, 0.000914, 0.00103, 0.00116, 0.00131
	Hispanic	Men	32	0.000817, 0.000926, 0.00105, 0.00119, 0.00135
	Hispanic	Men	33	0.00081, 0.00093, 0.00106, 0.00122, 0.0014
	Hispanic	Men	34	0.000798, 0.000929, 0.00108, 0.00125, 0.00146
	Hispanic	Men	35	0.000793, 0.000932, 0.00109, 0.00129, 0.00151
	Hispanic	Men	36	0.000801, 0.000945, 0.00111, 0.00131, 0.00155
	Hispanic	Men	37	0.000824, 0.000971, 0.00114, 0.00134, 0.00158

Variable	Race/ethnicity	Sex	Age	Distribution
	Hispanic	Men	38	0.00086, 0.00101, 0.00118, 0.00138, 0.00161
	Hispanic	Men	39	0.000902, 0.00105, 0.00123, 0.00143, 0.00167
	Hispanic	Men	40	0.000949, 0.00111, 0.00129, 0.0015, 0.00175
	Hispanic	Men	41	0.00101, 0.00117, 0.00136, 0.00159, 0.00185
	Hispanic	Men	42	0.00109, 0.00126, 0.00146, 0.00169, 0.00196
	Hispanic	Men	43	0.0012, 0.00138, 0.00158, 0.00181, 0.00208
	Hispanic	Men	44	0.00136, 0.00153, 0.00173, 0.00195, 0.00219
	Hispanic	Men	45	0.00154, 0.00171, 0.0019, 0.00212, 0.00235
	Hispanic	Men	46	0.00173, 0.00191, 0.00211, 0.00234, 0.00258
	Hispanic	Men	47	0.00192, 0.00213, 0.00235, 0.0026, 0.00288
	Hispanic	Men	48	0.00214, 0.00237, 0.00262, 0.00291, 0.00323
	Hispanic	Men	49	0.00237, 0.00263, 0.00292, 0.00324, 0.0036
	Hispanic	Men	50	0.00265, 0.00293, 0.00324, 0.00359, 0.00397
	Hispanic	Men	51	0.00299, 0.00327, 0.00358, 0.00392, 0.0043
	Hispanic	Men	52	0.0034, 0.00366, 0.00394, 0.00424, 0.00457
	Hispanic	Men	53	0.00388, 0.00409, 0.00432, 0.00455, 0.0048
	Hispanic	Men	54	0.00431, 0.00451, 0.00471, 0.00491, 0.00513
	Hispanic	Men	55	0.00463, 0.00487, 0.00511, 0.00537, 0.00564
	Hispanic	Men	56	0.00492, 0.00522, 0.00554, 0.00587, 0.00623
	Hispanic	Men	57	0.00523, 0.0056, 0.00598, 0.00639, 0.00683
	Hispanic	Men	58	0.00557, 0.00599, 0.00644, 0.00692, 0.00744
	Hispanic	Men	59	0.0059, 0.00638, 0.0069, 0.00746, 0.00807
	Hispanic	Men	60	0.00625, 0.00679, 0.00737, 0.008, 0.00869
	Hispanic	Men	61	0.00665, 0.00723, 0.00785, 0.00852, 0.00926
	Hispanic	Men	62	0.0071, 0.0077, 0.00833, 0.00902, 0.00978
	Hispanic	Men	63	0.00759, 0.00819, 0.00884, 0.00954, 0.0103
	Hispanic	Men	64	0.00807, 0.00871, 0.00939, 0.0101, 0.0109
	Hispanic	Men	65	0.00856, 0.00925, 0.01, 0.0108, 0.0117
	Hispanic	Men	66	0.00908, 0.00986, 0.0107, 0.0116, 0.0126
	Hispanic	Men	67	0.00968, 0.0105, 0.0115, 0.0125, 0.0136
	Hispanic	Men	68	0.0104, 0.0113, 0.0124, 0.0135, 0.0148
	Hispanic	Men	69	0.0112, 0.0123, 0.0134, 0.0147, 0.0161
	Hispanic	Men	70	0.0121, 0.0133, 0.0146, 0.016, 0.0176
	Hispanic	Men	71	0.0132, 0.0145, 0.0159, 0.0175, 0.0192
	Hispanic	Men	72	0.0143, 0.0158, 0.0173, 0.0191, 0.021
	Hispanic	Men	73	0.0156, 0.0172, 0.0189, 0.0208, 0.0229
	Hispanic	Men	74	0.0171, 0.0188, 0.0207, 0.0228, 0.0251
	Hispanic	Men	75	0.0188, 0.0206, 0.0227, 0.0249, 0.0274
	Hispanic	Men	76	0.0207, 0.0227, 0.0249, 0.0274, 0.03
	Hispanic	Men	77	0.0229, 0.0252, 0.0276, 0.0302, 0.0331
	Hispanic	Men	78	0.0255, 0.0279, 0.0306, 0.0335, 0.0367
	Hispanic	Men	79	0.0284, 0.0312, 0.0341, 0.0374, 0.0409
	Hispanic	Men	80	0.0318, 0.0348, 0.0381, 0.0417, 0.0456

Variable	Race/ethnicity	Sex	Age	Distribution
	Hispanic	Men	81	0.0355, 0.0389, 0.0425, 0.0465, 0.0509
	Hispanic	Men	82	0.0394, 0.0432, 0.0472, 0.0517, 0.0566
	Hispanic	Men	83	0.0432, 0.0475, 0.0521, 0.0572, 0.0628
	Hispanic	Men	84	0.0469, 0.0517, 0.0569, 0.0627, 0.0691
	Hispanic	Women	30	0.000327, 0.000369, 0.000415, 0.000468, 0.000528
	Hispanic	Women	31	0.000346, 0.000389, 0.000437, 0.00049, 0.000551
	Hispanic	Women	32	0.000366, 0.00041, 0.000459, 0.000513, 0.000575
	Hispanic	Women	33	0.000384, 0.00043, 0.00048, 0.000537, 0.0006
	Hispanic	Women	34	0.000398, 0.000447, 0.000502, 0.000563, 0.000632
	Hispanic	Women	35	0.000409, 0.000463, 0.000524, 0.000593, 0.000671
	Hispanic	Women	36	0.000421, 0.00048, 0.000548, 0.000625, 0.000713
	Hispanic	Women	37	0.000436, 0.0005, 0.000574, 0.000658, 0.000756
	Hispanic	Women	38	0.000457, 0.000526, 0.000604, 0.000694, 0.000798
	Hispanic	Women	39	0.000486, 0.000558, 0.000639, 0.000732, 0.00084
	Hispanic	Women	40	0.000523, 0.000597, 0.000681, 0.000776, 0.000885
	Hispanic	Women	41	0.000569, 0.000645, 0.00073, 0.000826, 0.000936
	Hispanic	Women	42	0.000625, 0.000703, 0.000789, 0.000885, 0.000994
	Hispanic	Women	43	0.000694, 0.000772, 0.000858, 0.000954, 0.00106
	Hispanic	Women	44	0.000774, 0.000854, 0.000941, 0.00104, 0.00114
	Hispanic	Women	45	0.000864, 0.000947, 0.00104, 0.00114, 0.00125
	Hispanic	Women	46	0.000961, 0.00105, 0.00115, 0.00126, 0.00137
	Hispanic	Women	47	0.00107, 0.00117, 0.00127, 0.00139, 0.00153
	Hispanic	Women	48	0.00118, 0.00129, 0.00142, 0.00155, 0.0017
	Hispanic	Women	49	0.0013, 0.00143, 0.00157, 0.00172, 0.00189
	Hispanic	Women	50	0.00144, 0.00159, 0.00174, 0.00191, 0.0021
	Hispanic	Women	51	0.00161, 0.00176, 0.00192, 0.0021, 0.0023
	Hispanic	Women	52	0.0018, 0.00195, 0.00211, 0.00229, 0.00249
	Hispanic	Women	53	0.002, 0.00215, 0.00232, 0.0025, 0.0027
	Hispanic	Women	54	0.00219, 0.00236, 0.00254, 0.00273, 0.00294
	Hispanic	Women	55	0.00237, 0.00256, 0.00277, 0.00298, 0.00322
	Hispanic	Women	56	0.00257, 0.00278, 0.00301, 0.00325, 0.00352
	Hispanic	Women	57	0.00277, 0.00301, 0.00326, 0.00353, 0.00382
	Hispanic	Women	58	0.003, 0.00325, 0.00352, 0.00381, 0.00413
	Hispanic	Women	59	0.00322, 0.0035, 0.0038, 0.00412, 0.00448
	Hispanic	Women	60	0.00345, 0.00376, 0.00409, 0.00445, 0.00484
	Hispanic	Women	61	0.00371, 0.00404, 0.0044, 0.00479, 0.00523
	Hispanic	Women	62	0.00399, 0.00435, 0.00473, 0.00516, 0.00562
	Hispanic	Women	63	0.00429, 0.00467, 0.00509, 0.00555, 0.00605
	Hispanic	Women	64	0.0046, 0.00503, 0.00549, 0.00599, 0.00654
	Hispanic	Women	65	0.00495, 0.00542, 0.00593, 0.00648, 0.0071
	Hispanic	Women	66	0.00534, 0.00586, 0.00642, 0.00704, 0.00773
	Hispanic	Women	67	0.00579, 0.00636, 0.00698, 0.00767, 0.00843
	Hispanic	Women	68	0.00631, 0.00694, 0.00762, 0.00837, 0.0092

Variable	Race/ethnicity	Sex	Age	Distribution
	Hispanic	Women	69	0.00692, 0.0076, 0.00834, 0.00916, 0.0101
	Hispanic	Women	70	0.0076, 0.00835, 0.00916, 0.01, 0.011
	Hispanic	Women	71	0.00837, 0.00919, 0.0101, 0.011, 0.0121
	Hispanic	Women	72	0.00922, 0.0101, 0.0111, 0.0122, 0.0133
	Hispanic	Women	73	0.0102, 0.0112, 0.0122, 0.0134, 0.0147
	Hispanic	Women	74	0.0113, 0.0124, 0.0135, 0.0148, 0.0162
	Hispanic	Women	75	0.0126, 0.0138, 0.015, 0.0164, 0.0178
	Hispanic	Women	76	0.0142, 0.0154, 0.0167, 0.0181, 0.0197
	Hispanic	Women	77	0.016, 0.0173, 0.0187, 0.0202, 0.0219
	Hispanic	Women	78	0.018, 0.0195, 0.021, 0.0227, 0.0246
	Hispanic	Women	79	0.0203, 0.022, 0.0237, 0.0256, 0.0277
	Hispanic	Women	80	0.0229, 0.0248, 0.0268, 0.029, 0.0314
	Hispanic	Women	81	0.0257, 0.0279, 0.0303, 0.0328, 0.0357
	Hispanic	Women	82	0.0286, 0.0312, 0.034, 0.0371, 0.0404
	Hispanic	Women	83	0.0316, 0.0347, 0.038, 0.0416, 0.0457
	Hispanic	Women	84	0.0346, 0.0382, 0.042, 0.0463, 0.0511
	White	Men	30	0.0011, 0.00133, 0.0016, 0.00192, 0.00231
	White	Men	31	0.00116, 0.00138, 0.00164, 0.00195, 0.00232
	White	Men	32	0.00121, 0.00142, 0.00167, 0.00197, 0.00232
	White	Men	33	0.00124, 0.00146, 0.0017, 0.00199, 0.00233
	White	Men	34	0.00126, 0.00147, 0.00172, 0.00201, 0.00235
	White	Men	35	0.00127, 0.00149, 0.00174, 0.00204, 0.00239
	White	Men	36	0.00127, 0.0015, 0.00176, 0.00207, 0.00244
	White	Men	37	0.00129, 0.00152, 0.00179, 0.00211, 0.00249
	White	Men	38	0.00132, 0.00156, 0.00183, 0.00216, 0.00255
	White	Men	39	0.00137, 0.00161, 0.00189, 0.00222, 0.00261
	White	Men	40	0.00144, 0.00168, 0.00197, 0.0023, 0.0027
	White	Men	41	0.00152, 0.00177, 0.00206, 0.00241, 0.00281
	White	Men	42	0.00163, 0.00189, 0.00219, 0.00254, 0.00295
	White	Men	43	0.00177, 0.00204, 0.00235, 0.00271, 0.00312
	White	Men	44	0.00195, 0.00223, 0.00255, 0.00291, 0.00333
	White	Men	45	0.00215, 0.00245, 0.00279, 0.00318, 0.00363
	White	Men	46	0.00236, 0.0027, 0.00308, 0.00352, 0.00402
	White	Men	47	0.00258, 0.00297, 0.00342, 0.00392, 0.00451
	White	Men	48	0.00285, 0.00329, 0.0038, 0.00438, 0.00506
	White	Men	49	0.00315, 0.00365, 0.00422, 0.00487, 0.00564
	White	Men	50	0.00352, 0.00406, 0.00468, 0.00539, 0.00622
	White	Men	51	0.00394, 0.00451, 0.00516, 0.00591, 0.00677
	White	Men	52	0.00443, 0.00502, 0.00568, 0.00643, 0.00728
	White	Men	53	0.00496, 0.00555, 0.00621, 0.00694, 0.00777
	White	Men	54	0.0055, 0.0061, 0.00675, 0.00748, 0.0083
	White	Men	55	0.00599, 0.00662, 0.00731, 0.00807, 0.00892
	White	Men	56	0.00643, 0.00712, 0.00787, 0.00871, 0.00964

Variable	Race/ethnicity	Sex	Age	Distribution
	White	Men	57	0.00684, 0.0076, 0.00844, 0.00936, 0.0104
	White	Men	58	0.00729, 0.0081, 0.009, 0.00999, 0.0111
	White	Men	59	0.00778, 0.00863, 0.00956, 0.0106, 0.0118
	White	Men	60	0.00831, 0.00918, 0.0101, 0.0112, 0.0123
	White	Men	61	0.0089, 0.00977, 0.0107, 0.0117, 0.0129
	White	Men	62	0.00953, 0.0104, 0.0113, 0.0123, 0.0134
	White	Men	63	0.0102, 0.011, 0.0119, 0.0129, 0.014
	White	Men	64	0.0108, 0.0117, 0.0126, 0.0136, 0.0148
	White	Men	65	0.0115, 0.0124, 0.0134, 0.0145, 0.0157
	White	Men	66	0.0122, 0.0133, 0.0144, 0.0156, 0.0169
	White	Men	67	0.0131, 0.0142, 0.0155, 0.0168, 0.0182
	White	Men	68	0.0141, 0.0154, 0.0167, 0.0182, 0.0198
	White	Men	69	0.0152, 0.0166, 0.0182, 0.0198, 0.0217
	White	Men	70	0.0166, 0.0181, 0.0198, 0.0216, 0.0236
	White	Men	71	0.0181, 0.0198, 0.0216, 0.0236, 0.0258
	White	Men	72	0.0198, 0.0216, 0.0236, 0.0258, 0.0282
	White	Men	73	0.0216, 0.0236, 0.0257, 0.0281, 0.0307
	White	Men	74	0.0237, 0.0258, 0.0281, 0.0306, 0.0333
	White	Men	75	0.0261, 0.0283, 0.0308, 0.0334, 0.0363
	White	Men	76	0.0288, 0.0312, 0.0338, 0.0365, 0.0396
	White	Men	77	0.0319, 0.0345, 0.0373, 0.0403, 0.0435
	White	Men	78	0.0353, 0.0382, 0.0413, 0.0447, 0.0483
	White	Men	79	0.0393, 0.0426, 0.0461, 0.0498, 0.054
	White	Men	80	0.0439, 0.0476, 0.0515, 0.0558, 0.0605
	White	Men	81	0.0489, 0.0531, 0.0577, 0.0626, 0.068
	White	Men	82	0.0542, 0.0591, 0.0643, 0.07, 0.0763
	White	Men	83	0.0596, 0.0652, 0.0713, 0.0778, 0.0851
	White	Men	84	0.065, 0.0713, 0.0782, 0.0858, 0.0942
	White	Women	30	0.000573, 0.000654, 0.000745, 0.000849, 0.000968
	White	Women	31	0.000604, 0.000691, 0.000789, 0.000902, 0.00103
	White	Women	32	0.000637, 0.000728, 0.000832, 0.000951, 0.00109
	White	Women	33	0.00067, 0.000765, 0.000873, 0.000996, 0.00114
	White	Women	34	0.000693, 0.000796, 0.000913, 0.00105, 0.0012
	White	Women	35	0.000708, 0.000822, 0.000953, 0.0011, 0.00128
	White	Women	36	0.000723, 0.000848, 0.000994, 0.00117, 0.00137
	White	Women	37	0.000742, 0.000879, 0.00104, 0.00123, 0.00146
	White	Women	38	0.000765, 0.000914, 0.00109, 0.0013, 0.00155
	White	Women	39	0.000794, 0.000956, 0.00115, 0.00138, 0.00166
	White	Women	40	0.000833, 0.00101, 0.00122, 0.00147, 0.00178
	White	Women	41	0.000887, 0.00107, 0.0013, 0.00157, 0.0019
	White	Women	42	0.000957, 0.00116, 0.0014, 0.00169, 0.00204
	White	Women	43	0.00104, 0.00126, 0.00151, 0.00182, 0.0022
	White	Women	44	0.00114, 0.00137, 0.00165, 0.00198, 0.00239

Variable	Race/ethnicity	Sex	Age	Distribution
	White	Women	45	0.00125, 0.0015, 0.00181, 0.00218, 0.00262
	White	Women	46	0.00138, 0.00166, 0.00199, 0.00239, 0.00288
	White	Women	47	0.00153, 0.00184, 0.0022, 0.00263, 0.00315
	White	Women	48	0.00173, 0.00205, 0.00243, 0.00288, 0.00341
	White	Women	49	0.00194, 0.00228, 0.00268, 0.00314, 0.00369
	White	Women	50	0.00216, 0.00253, 0.00295, 0.00344, 0.00402
	White	Women	51	0.00235, 0.00276, 0.00323, 0.00378, 0.00444
	White	Women	52	0.00253, 0.00299, 0.00353, 0.00416, 0.00491
	White	Women	53	0.00273, 0.00323, 0.00383, 0.00453, 0.00538
	White	Women	54	0.00297, 0.00351, 0.00413, 0.00487, 0.00574
	White	Women	55	0.00329, 0.00382, 0.00443, 0.00513, 0.00595
	White	Women	56	0.00369, 0.00417, 0.00472, 0.00533, 0.00604
	White	Women	57	0.00412, 0.00454, 0.00501, 0.00552, 0.00609
	White	Women	58	0.00452, 0.00489, 0.0053, 0.00573, 0.00621
	White	Women	59	0.00485, 0.00521, 0.0056, 0.00602, 0.00648
	White	Women	60	0.00513, 0.00552, 0.00594, 0.00639, 0.00688
	White	Women	61	0.00543, 0.00586, 0.00631, 0.00681, 0.00734
	White	Women	62	0.00578, 0.00625, 0.00675, 0.00728, 0.00787
	White	Women	63	0.0062, 0.0067, 0.00724, 0.00783, 0.00847
	White	Women	64	0.00668, 0.00723, 0.00782, 0.00846, 0.00916
	White	Women	65	0.00722, 0.00783, 0.00849, 0.0092, 0.00998
	White	Women	66	0.00782, 0.00851, 0.00926, 0.0101, 0.0109
	White	Women	67	0.00848, 0.00927, 0.0101, 0.0111, 0.0121
	White	Women	68	0.00919, 0.0101, 0.0111, 0.0122, 0.0134
	White	Women	69	0.00997, 0.011, 0.0122, 0.0135, 0.0149
	White	Women	70	0.0109, 0.0121, 0.0134, 0.0149, 0.0165
	White	Women	71	0.0118, 0.0132, 0.0147, 0.0164, 0.0183
	White	Women	72	0.013, 0.0145, 0.0162, 0.018, 0.0202
	White	Women	73	0.0143, 0.0159, 0.0178, 0.0198, 0.0221
	White	Women	74	0.0158, 0.0176, 0.0196, 0.0218, 0.0243
	White	Women	75	0.0175, 0.0195, 0.0216, 0.0239, 0.0265
	White	Women	76	0.0196, 0.0217, 0.0239, 0.0263, 0.0291
	White	Women	77	0.0221, 0.0242, 0.0266, 0.0291, 0.032
	White	Women	78	0.0249, 0.0272, 0.0297, 0.0324, 0.0355
	White	Women	79	0.028, 0.0306, 0.0333, 0.0363, 0.0396
	White	Women	80	0.0315, 0.0344, 0.0375, 0.0408, 0.0446
	White	Women	81	0.0354, 0.0386, 0.0421, 0.046, 0.0502
	White	Women	82	0.0394, 0.0432, 0.0472, 0.0516, 0.0565
	White	Women	83	0.0437, 0.0479, 0.0526, 0.0577, 0.0634
	White	Women	84	0.048, 0.0528, 0.0582, 0.064, 0.0706
Total mortality rates for 2026 (0.01, 0.2, 0.5, 0.8. 0.99 percentiles of the empirical distribution produced during forecasting)				
	Black	Men	30	0.0016, 0.00192, 0.00231, 0.00277, 0.00333
	Black	Men	31	0.0017, 0.002, 0.00235, 0.00276, 0.00324

Variable	Race/ethnicity	Sex	Age	Distribution
	Black	Men	32	0.00178, 0.00207, 0.00239, 0.00276, 0.0032
	Black	Men	33	0.00183, 0.00211, 0.00243, 0.0028, 0.00322
	Black	Men	34	0.00186, 0.00214, 0.00247, 0.00285, 0.00329
	Black	Men	35	0.00189, 0.00218, 0.00252, 0.00291, 0.00337
	Black	Men	36	0.00192, 0.00223, 0.00257, 0.00298, 0.00345
	Black	Men	37	0.00196, 0.00227, 0.00263, 0.00303, 0.00351
	Black	Men	38	0.002, 0.00231, 0.00268, 0.0031, 0.00359
	Black	Men	39	0.00202, 0.00236, 0.00274, 0.00318, 0.00371
	Black	Men	40	0.00205, 0.0024, 0.00282, 0.0033, 0.00388
	Black	Men	41	0.00208, 0.00247, 0.00293, 0.00347, 0.00412
	Black	Men	42	0.00213, 0.00256, 0.00308, 0.00369, 0.00444
	Black	Men	43	0.00221, 0.00269, 0.00327, 0.00398, 0.00484
	Black	Men	44	0.00232, 0.00286, 0.00352, 0.00433, 0.00533
	Black	Men	45	0.00246, 0.00307, 0.00382, 0.00475, 0.00592
	Black	Men	46	0.00265, 0.00333, 0.00418, 0.00524, 0.00658
	Black	Men	47	0.00291, 0.00366, 0.0046, 0.00578, 0.00728
	Black	Men	48	0.00324, 0.00406, 0.00509, 0.00637, 0.00799
	Black	Men	49	0.00365, 0.00455, 0.00564, 0.007, 0.00871
	Black	Men	50	0.00415, 0.0051, 0.00627, 0.0077, 0.00948
	Black	Men	51	0.00471, 0.00574, 0.00697, 0.00846, 0.0103
	Black	Men	52	0.00537, 0.00645, 0.00773, 0.00927, 0.0111
	Black	Men	53	0.00615, 0.00726, 0.00856, 0.0101, 0.0119
	Black	Men	54	0.00702, 0.00815, 0.00945, 0.011, 0.0127
	Black	Men	55	0.00798, 0.00912, 0.0104, 0.0119, 0.0136
	Black	Men	56	0.00901, 0.0101, 0.0114, 0.0128, 0.0145
	Black	Men	57	0.0101, 0.0112, 0.0125, 0.0138, 0.0154
	Black	Men	58	0.0113, 0.0124, 0.0136, 0.0149, 0.0164
	Black	Men	59	0.0124, 0.0135, 0.0147, 0.016, 0.0175
	Black	Men	60	0.0134, 0.0145, 0.0158, 0.0172, 0.0187
	Black	Men	61	0.0144, 0.0156, 0.0169, 0.0183, 0.0198
	Black	Men	62	0.0154, 0.0166, 0.0179, 0.0193, 0.0208
	Black	Men	63	0.0162, 0.0175, 0.0189, 0.0203, 0.0219
	Black	Men	64	0.017, 0.0184, 0.0198, 0.0214, 0.0231
	Black	Men	65	0.0178, 0.0192, 0.0208, 0.0225, 0.0243
	Black	Men	66	0.0186, 0.0202, 0.0219, 0.0237, 0.0256
	Black	Men	67	0.0195, 0.0212, 0.0231, 0.025, 0.0272
	Black	Men	68	0.0206, 0.0224, 0.0244, 0.0266, 0.029
	Black	Men	69	0.0218, 0.0238, 0.026, 0.0285, 0.0311
	Black	Men	70	0.0231, 0.0254, 0.0278, 0.0305, 0.0335
	Black	Men	71	0.0247, 0.0272, 0.0298, 0.0328, 0.036
	Black	Men	72	0.0265, 0.0291, 0.032, 0.0352, 0.0387
	Black	Men	73	0.0285, 0.0313, 0.0344, 0.0378, 0.0416
	Black	Men	74	0.0307, 0.0337, 0.037, 0.0405, 0.0444

Variable	Race/ethnicity	Sex	Age	Distribution
	Black	Men	75	0.0333, 0.0364, 0.0397, 0.0434, 0.0474
	Black	Men	76	0.0362, 0.0394, 0.0428, 0.0466, 0.0507
	Black	Men	77	0.0393, 0.0426, 0.0463, 0.0502, 0.0545
	Black	Men	78	0.0425, 0.0462, 0.0501, 0.0544, 0.059
	Black	Men	79	0.046, 0.05, 0.0544, 0.0592, 0.0644
	Black	Men	80	0.0497, 0.0542, 0.0592, 0.0645, 0.0705
	Black	Men	81	0.0536, 0.0587, 0.0643, 0.0703, 0.077
	Black	Men	82	0.0576, 0.0634, 0.0696, 0.0765, 0.0841
	Black	Men	83	0.0615, 0.0679, 0.075, 0.0827, 0.0914
	Black	Men	84	0.0652, 0.0723, 0.0802, 0.0889, 0.0986
	Black	Women	30	0.000566, 0.000717, 0.000905, 0.00114, 0.00145
	Black	Women	31	0.000638, 0.00079, 0.000976, 0.00121, 0.00149
	Black	Women	32	0.000721, 0.000872, 0.00105, 0.00127, 0.00154
	Black	Women	33	0.000805, 0.000955, 0.00113, 0.00134, 0.00159
	Black	Women	34	0.000873, 0.00103, 0.00121, 0.00142, 0.00167
	Black	Women	35	0.00092, 0.00109, 0.00128, 0.00151, 0.00178
	Black	Women	36	0.000957, 0.00114, 0.00134, 0.00159, 0.00189
	Black	Women	37	0.000996, 0.00119, 0.00141, 0.00168, 0.00199
	Black	Women	38	0.00104, 0.00124, 0.00148, 0.00176, 0.0021
	Black	Women	39	0.00111, 0.00132, 0.00157, 0.00186, 0.00222
	Black	Women	40	0.00118, 0.00141, 0.00167, 0.00198, 0.00235
	Black	Women	41	0.00129, 0.00152, 0.00179, 0.00211, 0.00249
	Black	Women	42	0.00142, 0.00166, 0.00193, 0.00225, 0.00263
	Black	Women	43	0.00159, 0.00183, 0.0021, 0.00241, 0.00277
	Black	Women	44	0.00179, 0.00203, 0.00229, 0.00259, 0.00293
	Black	Women	45	0.002, 0.00224, 0.00251, 0.00281, 0.00315
	Black	Women	46	0.00221, 0.00247, 0.00276, 0.00309, 0.00346
	Black	Women	47	0.00241, 0.00272, 0.00305, 0.00343, 0.00386
	Black	Women	48	0.00264, 0.00299, 0.00338, 0.00382, 0.00433
	Black	Women	49	0.00292, 0.00331, 0.00375, 0.00425, 0.00482
	Black	Women	50	0.00326, 0.00368, 0.00415, 0.00468, 0.00528
	Black	Women	51	0.00369, 0.00412, 0.00458, 0.0051, 0.00569
	Black	Women	52	0.0042, 0.0046, 0.00505, 0.00553, 0.00607
	Black	Women	53	0.00471, 0.00511, 0.00554, 0.006, 0.0065
	Black	Women	54	0.00521, 0.00561, 0.00605, 0.00651, 0.00702
	Black	Women	55	0.00568, 0.00612, 0.00658, 0.00708, 0.00762
	Black	Women	56	0.00614, 0.00662, 0.00712, 0.00767, 0.00826
	Black	Women	57	0.0066, 0.00712, 0.00767, 0.00826, 0.00891
	Black	Women	58	0.00705, 0.00761, 0.0082, 0.00885, 0.00955
	Black	Women	59	0.00745, 0.00806, 0.00872, 0.00943, 0.0102
	Black	Women	60	0.00782, 0.00849, 0.00921, 0.00999, 0.0108
	Black	Women	61	0.00821, 0.00892, 0.00969, 0.0105, 0.0114
	Black	Women	62	0.00862, 0.00936, 0.0102, 0.011, 0.012

Variable	Race/ethnicity	Sex	Age	Distribution
	Black	Women	63	0.00904, 0.00983, 0.0107, 0.0116, 0.0126
	Black	Women	64	0.0095, 0.0103, 0.0113, 0.0123, 0.0134
	Black	Women	65	0.01, 0.0109, 0.0119, 0.013, 0.0142
	Black	Women	66	0.0106, 0.0116, 0.0127, 0.0139, 0.0152
	Black	Women	67	0.0114, 0.0125, 0.0137, 0.015, 0.0164
	Black	Women	68	0.0122, 0.0134, 0.0147, 0.0161, 0.0177
	Black	Women	69	0.0132, 0.0145, 0.0159, 0.0174, 0.0192
	Black	Women	70	0.0142, 0.0156, 0.0172, 0.0189, 0.0207
	Black	Women	71	0.0154, 0.0169, 0.0186, 0.0204, 0.0224
	Black	Women	72	0.0166, 0.0183, 0.0201, 0.022, 0.0242
	Black	Women	73	0.018, 0.0198, 0.0217, 0.0238, 0.0261
	Black	Women	74	0.0196, 0.0215, 0.0235, 0.0257, 0.0281
	Black	Women	75	0.0214, 0.0234, 0.0255, 0.0278, 0.0304
	Black	Women	76	0.0235, 0.0256, 0.0278, 0.0302, 0.0329
	Black	Women	77	0.0258, 0.028, 0.0304, 0.033, 0.0358
	Black	Women	78	0.0284, 0.0308, 0.0334, 0.0362, 0.0393
	Black	Women	79	0.0312, 0.0339, 0.0368, 0.0399, 0.0433
	Black	Women	80	0.0343, 0.0373, 0.0406, 0.0441, 0.0479
	Black	Women	81	0.0375, 0.0409, 0.0446, 0.0487, 0.0531
	Black	Women	82	0.0408, 0.0447, 0.049, 0.0537, 0.0588
	Black	Women	83	0.044, 0.0485, 0.0535, 0.0589, 0.065
	Black	Women	84	0.0472, 0.0524, 0.058, 0.0643, 0.0713
	Hispanic	Men	30	0.00079, 0.000895, 0.00101, 0.00115, 0.0013
	Hispanic	Men	31	0.000807, 0.000913, 0.00103, 0.00117, 0.00132
	Hispanic	Men	32	0.000815, 0.000926, 0.00105, 0.00119, 0.00135
	Hispanic	Men	33	0.000806, 0.000928, 0.00107, 0.00122, 0.00141
	Hispanic	Men	34	0.000792, 0.000925, 0.00108, 0.00126, 0.00147
	Hispanic	Men	35	0.000785, 0.000927, 0.00109, 0.00129, 0.00152
	Hispanic	Men	36	0.000791, 0.000938, 0.00111, 0.00131, 0.00156
	Hispanic	Men	37	0.000812, 0.000961, 0.00113, 0.00134, 0.00159
	Hispanic	Men	38	0.000845, 0.000995, 0.00117, 0.00137, 0.00162
	Hispanic	Men	39	0.000885, 0.00104, 0.00121, 0.00142, 0.00166
	Hispanic	Men	40	0.000929, 0.00109, 0.00127, 0.00149, 0.00174
	Hispanic	Men	41	0.000984, 0.00115, 0.00135, 0.00157, 0.00184
	Hispanic	Men	42	0.00106, 0.00124, 0.00144, 0.00168, 0.00195
	Hispanic	Men	43	0.00118, 0.00136, 0.00156, 0.00179, 0.00206
	Hispanic	Men	44	0.00133, 0.00151, 0.0017, 0.00192, 0.00218
	Hispanic	Men	45	0.00151, 0.00168, 0.00188, 0.00209, 0.00233
	Hispanic	Men	46	0.00169, 0.00188, 0.00208, 0.00231, 0.00256
	Hispanic	Men	47	0.00189, 0.00209, 0.00232, 0.00257, 0.00286
	Hispanic	Men	48	0.0021, 0.00233, 0.00259, 0.00288, 0.0032
	Hispanic	Men	49	0.00233, 0.00259, 0.00289, 0.00321, 0.00358
	Hispanic	Men	50	0.0026, 0.00289, 0.00321, 0.00356, 0.00395

Variable	Race/ethnicity	Sex	Age	Distribution
	Hispanic	Men	51	0.00294, 0.00323, 0.00355, 0.0039, 0.00428
	Hispanic	Men	52	0.00336, 0.00362, 0.00391, 0.00421, 0.00455
	Hispanic	Men	53	0.00383, 0.00405, 0.00428, 0.00452, 0.00478
	Hispanic	Men	54	0.00427, 0.00447, 0.00467, 0.00488, 0.00511
	Hispanic	Men	55	0.00459, 0.00483, 0.00508, 0.00534, 0.00562
	Hispanic	Men	56	0.00487, 0.00518, 0.0055, 0.00584, 0.00621
	Hispanic	Men	57	0.00518, 0.00555, 0.00594, 0.00636, 0.00681
	Hispanic	Men	58	0.0055, 0.00593, 0.00639, 0.00688, 0.00742
	Hispanic	Men	59	0.00582, 0.00631, 0.00684, 0.00741, 0.00804
	Hispanic	Men	60	0.00616, 0.00671, 0.0073, 0.00794, 0.00865
	Hispanic	Men	61	0.00654, 0.00713, 0.00776, 0.00845, 0.00921
	Hispanic	Men	62	0.00698, 0.00759, 0.00823, 0.00894, 0.00971
	Hispanic	Men	63	0.00745, 0.00807, 0.00873, 0.00944, 0.0102
	Hispanic	Men	64	0.00792, 0.00856, 0.00926, 0.01, 0.0108
	Hispanic	Men	65	0.00839, 0.00909, 0.00985, 0.0107, 0.0116
	Hispanic	Men	66	0.0089, 0.00968, 0.0105, 0.0115, 0.0125
	Hispanic	Men	67	0.00948, 0.0104, 0.0113, 0.0123, 0.0135
	Hispanic	Men	68	0.0102, 0.0111, 0.0122, 0.0134, 0.0147
	Hispanic	Men	69	0.011, 0.012, 0.0132, 0.0145, 0.016
	Hispanic	Men	70	0.0119, 0.0131, 0.0144, 0.0158, 0.0174
	Hispanic	Men	71	0.0129, 0.0142, 0.0157, 0.0172, 0.019
	Hispanic	Men	72	0.014, 0.0155, 0.0171, 0.0188, 0.0208
	Hispanic	Men	73	0.0153, 0.0169, 0.0186, 0.0206, 0.0227
	Hispanic	Men	74	0.0167, 0.0184, 0.0204, 0.0225, 0.0248
	Hispanic	Men	75	0.0184, 0.0202, 0.0223, 0.0246, 0.0271
	Hispanic	Men	76	0.0203, 0.0223, 0.0245, 0.027, 0.0297
	Hispanic	Men	77	0.0225, 0.0247, 0.0271, 0.0298, 0.0328
	Hispanic	Men	78	0.025, 0.0274, 0.0301, 0.0331, 0.0364
	Hispanic	Men	79	0.0279, 0.0306, 0.0336, 0.0369, 0.0406
	Hispanic	Men	80	0.0312, 0.0342, 0.0376, 0.0412, 0.0453
	Hispanic	Men	81	0.0348, 0.0382, 0.0419, 0.046, 0.0504
	Hispanic	Men	82	0.0386, 0.0424, 0.0466, 0.0511, 0.0562
	Hispanic	Men	83	0.0424, 0.0467, 0.0514, 0.0565, 0.0623
	Hispanic	Men	84	0.046, 0.0508, 0.0561, 0.062, 0.0685
	Hispanic	Women	30	0.000326, 0.000368, 0.000415, 0.000468, 0.000528
	Hispanic	Women	31	0.000346, 0.000389, 0.000437, 0.00049, 0.000551
	Hispanic	Women	32	0.000366, 0.00041, 0.000458, 0.000513, 0.000574
	Hispanic	Women	33	0.000383, 0.000429, 0.00048, 0.000536, 0.0006
	Hispanic	Women	34	0.000396, 0.000445, 0.000501, 0.000562, 0.000633
	Hispanic	Women	35	0.000406, 0.00046, 0.000522, 0.000591, 0.000671
	Hispanic	Women	36	0.000416, 0.000476, 0.000544, 0.000622, 0.000713
	Hispanic	Women	37	0.00043, 0.000495, 0.000569, 0.000655, 0.000754
	Hispanic	Women	38	0.00045, 0.000519, 0.000598, 0.000689, 0.000795

Variable	Race/ethnicity	Sex	Age	Distribution
	Hispanic	Women	39	0.000477, 0.000549, 0.000631, 0.000726, 0.000836
	Hispanic	Women	40	0.000512, 0.000587, 0.000671, 0.000768, 0.000879
	Hispanic	Women	41	0.000557, 0.000633, 0.000719, 0.000816, 0.000928
	Hispanic	Women	42	0.000611, 0.000689, 0.000776, 0.000874, 0.000985
	Hispanic	Women	43	0.000678, 0.000757, 0.000844, 0.000941, 0.00105
	Hispanic	Women	44	0.000757, 0.000837, 0.000925, 0.00102, 0.00113
	Hispanic	Women	45	0.000845, 0.000929, 0.00102, 0.00112, 0.00123
	Hispanic	Women	46	0.000941, 0.00103, 0.00113, 0.00124, 0.00136
	Hispanic	Women	47	0.00104, 0.00115, 0.00126, 0.00138, 0.00151
	Hispanic	Women	48	0.00116, 0.00127, 0.0014, 0.00153, 0.00169
	Hispanic	Women	49	0.00128, 0.00141, 0.00155, 0.00171, 0.00188
	Hispanic	Women	50	0.00142, 0.00156, 0.00172, 0.00189, 0.00208
	Hispanic	Women	51	0.00158, 0.00174, 0.0019, 0.00208, 0.00228
	Hispanic	Women	52	0.00177, 0.00193, 0.0021, 0.00228, 0.00248
	Hispanic	Women	53	0.00197, 0.00213, 0.0023, 0.00248, 0.00268
	Hispanic	Women	54	0.00217, 0.00234, 0.00252, 0.00271, 0.00292
	Hispanic	Women	55	0.00235, 0.00254, 0.00275, 0.00296, 0.0032
	Hispanic	Women	56	0.00254, 0.00275, 0.00298, 0.00323, 0.0035
	Hispanic	Women	57	0.00275, 0.00298, 0.00323, 0.00351, 0.00381
	Hispanic	Women	58	0.00296, 0.00322, 0.00349, 0.00379, 0.00412
	Hispanic	Women	59	0.00318, 0.00346, 0.00377, 0.0041, 0.00446
	Hispanic	Women	60	0.00341, 0.00372, 0.00405, 0.00442, 0.00482
	Hispanic	Women	61	0.00365, 0.00399, 0.00436, 0.00476, 0.0052
	Hispanic	Women	62	0.00392, 0.00429, 0.00468, 0.00511, 0.00558
	Hispanic	Women	63	0.00421, 0.0046, 0.00503, 0.00549, 0.006
	Hispanic	Women	64	0.00452, 0.00495, 0.00541, 0.00592, 0.00648
	Hispanic	Women	65	0.00485, 0.00533, 0.00584, 0.00641, 0.00703
	Hispanic	Women	66	0.00523, 0.00576, 0.00633, 0.00695, 0.00765
	Hispanic	Women	67	0.00567, 0.00625, 0.00688, 0.00757, 0.00834
	Hispanic	Women	68	0.00618, 0.00682, 0.0075, 0.00826, 0.00911
	Hispanic	Women	69	0.00677, 0.00746, 0.00822, 0.00904, 0.00996
	Hispanic	Women	70	0.00744, 0.00819, 0.00902, 0.00992, 0.0109
	Hispanic	Women	71	0.00819, 0.00901, 0.00991, 0.0109, 0.012
	Hispanic	Women	72	0.00902, 0.00993, 0.0109, 0.012, 0.0132
	Hispanic	Women	73	0.00997, 0.011, 0.012, 0.0132, 0.0145
	Hispanic	Women	74	0.0111, 0.0121, 0.0133, 0.0146, 0.016
	Hispanic	Women	75	0.0124, 0.0135, 0.0148, 0.0161, 0.0176
	Hispanic	Women	76	0.0139, 0.0151, 0.0165, 0.0179, 0.0195
	Hispanic	Women	77	0.0156, 0.017, 0.0184, 0.02, 0.0217
	Hispanic	Women	78	0.0176, 0.0191, 0.0207, 0.0224, 0.0243
	Hispanic	Women	79	0.0199, 0.0216, 0.0234, 0.0253, 0.0274
	Hispanic	Women	80	0.0224, 0.0243, 0.0264, 0.0286, 0.0311
	Hispanic	Women	81	0.0252, 0.0274, 0.0298, 0.0324, 0.0353

Variable	Race/ethnicity	Sex	Age	Distribution
	Hispanic	Women	82	0.028, 0.0307, 0.0335, 0.0366, 0.0401
	Hispanic	Women	83	0.031, 0.0341, 0.0374, 0.0411, 0.0453
	Hispanic	Women	84	0.0339, 0.0375, 0.0414, 0.0458, 0.0506
	White	Men	30	0.00108, 0.00132, 0.00159, 0.00193, 0.00234
	White	Men	31	0.00114, 0.00137, 0.00164, 0.00196, 0.00235
	White	Men	32	0.00119, 0.00141, 0.00167, 0.00198, 0.00234
	White	Men	33	0.00123, 0.00144, 0.0017, 0.00199, 0.00235
	White	Men	34	0.00124, 0.00146, 0.00171, 0.00201, 0.00237
	White	Men	35	0.00124, 0.00147, 0.00173, 0.00204, 0.00241
	White	Men	36	0.00125, 0.00148, 0.00175, 0.00207, 0.00245
	White	Men	37	0.00126, 0.0015, 0.00178, 0.00211, 0.00251
	White	Men	38	0.00129, 0.00153, 0.00181, 0.00215, 0.00256
	White	Men	39	0.00133, 0.00158, 0.00187, 0.00221, 0.00261
	White	Men	40	0.0014, 0.00165, 0.00194, 0.00228, 0.00269
	White	Men	41	0.00148, 0.00173, 0.00203, 0.00238, 0.0028
	White	Men	42	0.00158, 0.00185, 0.00215, 0.00251, 0.00293
	White	Men	43	0.00172, 0.00199, 0.00231, 0.00267, 0.0031
	White	Men	44	0.00189, 0.00218, 0.0025, 0.00288, 0.00331
	White	Men	45	0.00209, 0.0024, 0.00274, 0.00314, 0.0036
	White	Men	46	0.00229, 0.00264, 0.00303, 0.00348, 0.004
	White	Men	47	0.00252, 0.00291, 0.00336, 0.00388, 0.00449
	White	Men	48	0.00277, 0.00322, 0.00374, 0.00434, 0.00505
	White	Men	49	0.00308, 0.00358, 0.00416, 0.00484, 0.00563
	White	Men	50	0.00343, 0.00399, 0.00462, 0.00535, 0.00622
	White	Men	51	0.00385, 0.00444, 0.00511, 0.00588, 0.00677
	White	Men	52	0.00433, 0.00494, 0.00562, 0.0064, 0.00729
	White	Men	53	0.00487, 0.00547, 0.00615, 0.00691, 0.00778
	White	Men	54	0.0054, 0.00602, 0.0067, 0.00745, 0.0083
	White	Men	55	0.00589, 0.00654, 0.00725, 0.00804, 0.00892
	White	Men	56	0.00632, 0.00703, 0.00781, 0.00867, 0.00965
	White	Men	57	0.00673, 0.00751, 0.00837, 0.00933, 0.0104
	White	Men	58	0.00716, 0.008, 0.00892, 0.00996, 0.0111
	White	Men	59	0.00764, 0.00851, 0.00948, 0.0106, 0.0118
	White	Men	60	0.00816, 0.00905, 0.01, 0.0111, 0.0123
	White	Men	61	0.00874, 0.00962, 0.0106, 0.0116, 0.0128
	White	Men	62	0.00935, 0.0102, 0.0112, 0.0122, 0.0133
	White	Men	63	0.00998, 0.0108, 0.0118, 0.0128, 0.0139
	White	Men	64	0.0106, 0.0115, 0.0125, 0.0135, 0.0146
	White	Men	65	0.0113, 0.0122, 0.0132, 0.0143, 0.0156
	White	Men	66	0.012, 0.013, 0.0142, 0.0154, 0.0167
	White	Men	67	0.0128, 0.014, 0.0152, 0.0166, 0.0181
	White	Men	68	0.0138, 0.0151, 0.0165, 0.018, 0.0197
	White	Men	69	0.0149, 0.0163, 0.0179, 0.0196, 0.0215

Variable	Race/ethnicity	Sex	Age	Distribution
	White	Men	70	0.0162, 0.0178, 0.0195, 0.0214, 0.0234
	White	Men	71	0.0177, 0.0194, 0.0213, 0.0233, 0.0256
	White	Men	72	0.0193, 0.0212, 0.0232, 0.0254, 0.0279
	White	Men	73	0.0211, 0.0231, 0.0253, 0.0277, 0.0303
	White	Men	74	0.0232, 0.0253, 0.0276, 0.0302, 0.033
	White	Men	75	0.0255, 0.0278, 0.0302, 0.0329, 0.0359
	White	Men	76	0.0282, 0.0306, 0.0332, 0.0361, 0.0392
	White	Men	77	0.0312, 0.0338, 0.0366, 0.0397, 0.0431
	White	Men	78	0.0346, 0.0375, 0.0407, 0.0441, 0.0478
	White	Men	79	0.0385, 0.0418, 0.0453, 0.0492, 0.0534
	White	Men	80	0.0429, 0.0467, 0.0507, 0.0551, 0.06
	White	Men	81	0.0479, 0.0522, 0.0568, 0.0618, 0.0674
	White	Men	82	0.0531, 0.058, 0.0634, 0.0692, 0.0756
	White	Men	83	0.0584, 0.0641, 0.0702, 0.077, 0.0844
	White	Men	84	0.0636, 0.0701, 0.0771, 0.0848, 0.0934
	White	Women	30	0.000567, 0.00065, 0.000743, 0.00085, 0.000973
	White	Women	31	0.000597, 0.000686, 0.000787, 0.000903, 0.00104
	White	Women	32	0.00063, 0.000724, 0.00083, 0.000952, 0.00109
	White	Women	33	0.000662, 0.00076, 0.00087, 0.000997, 0.00114
	White	Women	34	0.000684, 0.000789, 0.000909, 0.00105, 0.00121
	White	Women	35	0.000696, 0.000813, 0.000947, 0.0011, 0.00129
	White	Women	36	0.000709, 0.000837, 0.000987, 0.00116, 0.00137
	White	Women	37	0.000725, 0.000865, 0.00103, 0.00123, 0.00146
	White	Women	38	0.000746, 0.000897, 0.00108, 0.00129, 0.00156
	White	Women	39	0.000772, 0.000936, 0.00113, 0.00137, 0.00166
	White	Women	40	0.000808, 0.000985, 0.0012, 0.00146, 0.00178
	White	Women	41	0.000859, 0.00105, 0.00128, 0.00156, 0.0019
	White	Women	42	0.000926, 0.00113, 0.00137, 0.00167, 0.00203
	White	Women	43	0.00101, 0.00122, 0.00148, 0.0018, 0.00219
	White	Women	44	0.0011, 0.00134, 0.00162, 0.00196, 0.00238
	White	Women	45	0.00121, 0.00147, 0.00178, 0.00215, 0.00261
	White	Women	46	0.00133, 0.00162, 0.00196, 0.00237, 0.00287
	White	Women	47	0.00149, 0.00179, 0.00216, 0.0026, 0.00314
	White	Women	48	0.00168, 0.002, 0.00239, 0.00285, 0.0034
	White	Women	49	0.00189, 0.00224, 0.00264, 0.00311, 0.00368
	White	Women	50	0.00211, 0.00248, 0.00291, 0.00341, 0.00401
	White	Women	51	0.0023, 0.00271, 0.00319, 0.00376, 0.00444
	White	Women	52	0.00247, 0.00294, 0.00349, 0.00414, 0.00492
	White	Women	53	0.00266, 0.00318, 0.00379, 0.00452, 0.00539
	White	Women	54	0.00291, 0.00345, 0.00409, 0.00485, 0.00576
	White	Women	55	0.00323, 0.00377, 0.00439, 0.00511, 0.00596
	White	Women	56	0.00363, 0.00412, 0.00468, 0.00531, 0.00604
	White	Women	57	0.00406, 0.00449, 0.00496, 0.00549, 0.00608

Variable	Race/ethnicity	Sex	Age	Distribution
	White	Women	58	0.00446, 0.00484, 0.00525, 0.0057, 0.00619
	White	Women	59	0.00478, 0.00515, 0.00555, 0.00599, 0.00646
	White	Women	60	0.00505, 0.00545, 0.00588, 0.00635, 0.00685
	White	Women	61	0.00535, 0.00578, 0.00625, 0.00675, 0.0073
	White	Women	62	0.00569, 0.00616, 0.00667, 0.00722, 0.00782
	White	Women	63	0.00609, 0.0066, 0.00715, 0.00775, 0.0084
	White	Women	64	0.00656, 0.00712, 0.00772, 0.00837, 0.00908
	White	Women	65	0.00708, 0.0077, 0.00837, 0.00909, 0.00988
	White	Women	66	0.00767, 0.00836, 0.00912, 0.00994, 0.0108
	White	Women	67	0.0083, 0.0091, 0.00997, 0.0109, 0.012
	White	Women	68	0.00898, 0.00991, 0.0109, 0.012, 0.0133
	White	Women	69	0.00974, 0.0108, 0.012, 0.0133, 0.0148
	White	Women	70	0.0106, 0.0118, 0.0132, 0.0147, 0.0164
	White	Women	71	0.0116, 0.0129, 0.0145, 0.0162, 0.0181
	White	Women	72	0.0127, 0.0142, 0.0159, 0.0178, 0.02
	White	Women	73	0.0139, 0.0156, 0.0175, 0.0196, 0.0219
	White	Women	74	0.0154, 0.0172, 0.0192, 0.0215, 0.024
	White	Women	75	0.0171, 0.0191, 0.0212, 0.0236, 0.0263
	White	Women	76	0.0192, 0.0212, 0.0235, 0.026, 0.0288
	White	Women	77	0.0216, 0.0237, 0.0261, 0.0287, 0.0317
	White	Women	78	0.0243, 0.0267, 0.0292, 0.032, 0.0351
	White	Women	79	0.0274, 0.03, 0.0328, 0.0359, 0.0393
	White	Women	80	0.0308, 0.0337, 0.0369, 0.0403, 0.0442
	White	Women	81	0.0346, 0.0379, 0.0415, 0.0454, 0.0497
	White	Women	82	0.0386, 0.0424, 0.0465, 0.051, 0.056
	White	Women	83	0.0427, 0.0471, 0.0518, 0.0571, 0.0629
	White	Women	84	0.0469, 0.0519, 0.0573, 0.0633, 0.07
Total mortality rates for 2027 (0.01, 0.2, 0.5, 0.8. 0.99 percentiles of the empirical distribution produced during forecasting)				
	Black	Men	30	0.00158, 0.00191, 0.00231, 0.00279, 0.00337
	Black	Men	31	0.00168, 0.00199, 0.00235, 0.00277, 0.00328
	Black	Men	32	0.00177, 0.00206, 0.00239, 0.00277, 0.00323
	Black	Men	33	0.00181, 0.0021, 0.00243, 0.00281, 0.00325
	Black	Men	34	0.00184, 0.00213, 0.00247, 0.00286, 0.00332
	Black	Men	35	0.00186, 0.00217, 0.00251, 0.00292, 0.00339
	Black	Men	36	0.0019, 0.0022, 0.00256, 0.00297, 0.00346
	Black	Men	37	0.00193, 0.00224, 0.00261, 0.00302, 0.00352
	Black	Men	38	0.00196, 0.00228, 0.00265, 0.00308, 0.00359
	Black	Men	39	0.00198, 0.00232, 0.00271, 0.00316, 0.0037
	Black	Men	40	0.002, 0.00236, 0.00278, 0.00328, 0.00387
	Black	Men	41	0.00202, 0.00242, 0.00288, 0.00344, 0.00411
	Black	Men	42	0.00207, 0.00251, 0.00303, 0.00366, 0.00442
	Black	Men	43	0.00215, 0.00263, 0.00322, 0.00394, 0.00482
	Black	Men	44	0.00225, 0.0028, 0.00346, 0.00429, 0.00532

Variable	Race/ethnicity	Sex	Age	Distribution
	Black	Men	45	0.00239, 0.003, 0.00376, 0.00471, 0.00592
	Black	Men	46	0.00257, 0.00326, 0.00412, 0.0052, 0.00659
	Black	Men	47	0.00282, 0.00358, 0.00454, 0.00575, 0.0073
	Black	Men	48	0.00314, 0.00398, 0.00502, 0.00634, 0.00803
	Black	Men	49	0.00355, 0.00445, 0.00558, 0.00698, 0.00877
	Black	Men	50	0.00403, 0.00501, 0.00621, 0.00769, 0.00955
	Black	Men	51	0.00459, 0.00564, 0.0069, 0.00846, 0.0104
	Black	Men	52	0.00525, 0.00635, 0.00767, 0.00926, 0.0112
	Black	Men	53	0.00602, 0.00716, 0.0085, 0.0101, 0.012
	Black	Men	54	0.00689, 0.00805, 0.00939, 0.011, 0.0128
	Black	Men	55	0.00785, 0.00901, 0.0103, 0.0119, 0.0136
	Black	Men	56	0.00887, 0.01, 0.0113, 0.0128, 0.0145
	Black	Men	57	0.00997, 0.0111, 0.0124, 0.0138, 0.0154
	Black	Men	58	0.0111, 0.0122, 0.0135, 0.0148, 0.0164
	Black	Men	59	0.0122, 0.0133, 0.0146, 0.0159, 0.0174
	Black	Men	60	0.0132, 0.0144, 0.0157, 0.0171, 0.0186
	Black	Men	61	0.0141, 0.0154, 0.0167, 0.0181, 0.0197
	Black	Men	62	0.0151, 0.0164, 0.0177, 0.0191, 0.0207
	Black	Men	63	0.0159, 0.0172, 0.0186, 0.0201, 0.0217
	Black	Men	64	0.0167, 0.0181, 0.0195, 0.0211, 0.0229
	Black	Men	65	0.0174, 0.0189, 0.0205, 0.0222, 0.0241
	Black	Men	66	0.0182, 0.0198, 0.0215, 0.0234, 0.0254
	Black	Men	67	0.0191, 0.0209, 0.0227, 0.0247, 0.0269
	Black	Men	68	0.0201, 0.022, 0.0241, 0.0263, 0.0288
	Black	Men	69	0.0213, 0.0234, 0.0256, 0.0281, 0.0308
	Black	Men	70	0.0226, 0.0249, 0.0274, 0.0301, 0.0332
	Black	Men	71	0.0242, 0.0267, 0.0294, 0.0323, 0.0357
	Black	Men	72	0.0259, 0.0286, 0.0315, 0.0347, 0.0383
	Black	Men	73	0.0278, 0.0307, 0.0338, 0.0373, 0.0411
	Black	Men	74	0.0301, 0.0331, 0.0363, 0.04, 0.044
	Black	Men	75	0.0326, 0.0357, 0.0391, 0.0428, 0.0469
	Black	Men	76	0.0354, 0.0386, 0.0421, 0.0459, 0.0502
	Black	Men	77	0.0384, 0.0418, 0.0455, 0.0495, 0.0539
	Black	Men	78	0.0416, 0.0453, 0.0493, 0.0537, 0.0585
	Black	Men	79	0.045, 0.0491, 0.0536, 0.0584, 0.0638
	Black	Men	80	0.0486, 0.0533, 0.0583, 0.0638, 0.0698
	Black	Men	81	0.0525, 0.0577, 0.0633, 0.0695, 0.0764
	Black	Men	82	0.0564, 0.0622, 0.0686, 0.0756, 0.0834
	Black	Men	83	0.0602, 0.0668, 0.0739, 0.0819, 0.0907
	Black	Men	84	0.0638, 0.0711, 0.0791, 0.088, 0.098
	Black	Women	30	0.000555, 0.000709, 0.000905, 0.00116, 0.00148
	Black	Women	31	0.000626, 0.000782, 0.000976, 0.00122, 0.00152
	Black	Women	32	0.000709, 0.000865, 0.00105, 0.00128, 0.00156

Variable	Race/ethnicity	Sex	Age	Distribution
	Black	Women	33	0.000793, 0.000947, 0.00113, 0.00135, 0.00161
	Black	Women	34	0.00086, 0.00102, 0.00121, 0.00143, 0.00169
	Black	Women	35	0.000904, 0.00107, 0.00127, 0.00151, 0.00179
	Black	Women	36	0.000938, 0.00112, 0.00134, 0.00159, 0.0019
	Black	Women	37	0.000974, 0.00117, 0.0014, 0.00167, 0.00201
	Black	Women	38	0.00102, 0.00122, 0.00147, 0.00176, 0.00211
	Black	Women	39	0.00108, 0.00129, 0.00155, 0.00185, 0.00222
	Black	Women	40	0.00115, 0.00138, 0.00164, 0.00196, 0.00235
	Black	Women	41	0.00125, 0.00149, 0.00176, 0.00209, 0.00249
	Black	Women	42	0.00138, 0.00162, 0.0019, 0.00223, 0.00262
	Black	Women	43	0.00155, 0.00179, 0.00207, 0.00238, 0.00276
	Black	Women	44	0.00174, 0.00198, 0.00225, 0.00256, 0.00291
	Black	Women	45	0.00195, 0.0022, 0.00247, 0.00278, 0.00313
	Black	Women	46	0.00215, 0.00242, 0.00272, 0.00306, 0.00344
	Black	Women	47	0.00235, 0.00266, 0.00301, 0.0034, 0.00384
	Black	Women	48	0.00258, 0.00293, 0.00334, 0.00379, 0.00431
	Black	Women	49	0.00285, 0.00325, 0.0037, 0.00422, 0.00481
	Black	Women	50	0.00319, 0.00362, 0.0041, 0.00465, 0.00527
	Black	Women	51	0.00362, 0.00406, 0.00454, 0.00508, 0.00568
	Black	Women	52	0.00412, 0.00454, 0.005, 0.00551, 0.00607
	Black	Women	53	0.00464, 0.00505, 0.00549, 0.00597, 0.00649
	Black	Women	54	0.00514, 0.00555, 0.006, 0.00648, 0.00701
	Black	Women	55	0.00561, 0.00606, 0.00653, 0.00705, 0.00761
	Black	Women	56	0.00607, 0.00655, 0.00707, 0.00764, 0.00825
	Black	Women	57	0.00652, 0.00705, 0.00761, 0.00823, 0.00889
	Black	Women	58	0.00696, 0.00753, 0.00814, 0.0088, 0.00953
	Black	Women	59	0.00735, 0.00797, 0.00865, 0.00938, 0.0102
	Black	Women	60	0.0077, 0.00839, 0.00912, 0.00993, 0.0108
	Black	Women	61	0.00807, 0.0088, 0.00959, 0.0104, 0.0114
	Black	Women	62	0.00847, 0.00923, 0.01, 0.0109, 0.0119
	Black	Women	63	0.00887, 0.00968, 0.0105, 0.0115, 0.0125
	Black	Women	64	0.00931, 0.0102, 0.0111, 0.0121, 0.0133
	Black	Women	65	0.00981, 0.0108, 0.0118, 0.0129, 0.0141
	Black	Women	66	0.0104, 0.0114, 0.0125, 0.0138, 0.0151
	Black	Women	67	0.0111, 0.0122, 0.0135, 0.0148, 0.0163
	Black	Women	68	0.012, 0.0132, 0.0145, 0.0159, 0.0176
	Black	Women	69	0.0129, 0.0142, 0.0156, 0.0172, 0.019
	Black	Women	70	0.0139, 0.0153, 0.0169, 0.0186, 0.0205
	Black	Women	71	0.015, 0.0166, 0.0183, 0.0201, 0.0222
	Black	Women	72	0.0163, 0.0179, 0.0197, 0.0217, 0.0239
	Black	Women	73	0.0176, 0.0194, 0.0213, 0.0235, 0.0258
	Black	Women	74	0.0192, 0.021, 0.0231, 0.0253, 0.0278
	Black	Women	75	0.0209, 0.0229, 0.0251, 0.0274, 0.03

Variable	Race/ethnicity	Sex	Age	Distribution
	Black	Women	76	0.023, 0.0251, 0.0273, 0.0298, 0.0325
	Black	Women	77	0.0253, 0.0275, 0.0299, 0.0326, 0.0354
	Black	Women	78	0.0278, 0.0302, 0.0329, 0.0357, 0.0389
	Black	Women	79	0.0306, 0.0333, 0.0362, 0.0394, 0.0429
	Black	Women	80	0.0336, 0.0366, 0.0399, 0.0435, 0.0475
	Black	Women	81	0.0368, 0.0402, 0.044, 0.0481, 0.0526
	Black	Women	82	0.04, 0.0439, 0.0483, 0.053, 0.0583
	Black	Women	83	0.0431, 0.0477, 0.0527, 0.0582, 0.0644
	Black	Women	84	0.0463, 0.0515, 0.0572, 0.0635, 0.0707
	Hispanic	Men	30	0.000789, 0.000894, 0.00101, 0.00115, 0.0013
	Hispanic	Men	31	0.000806, 0.000913, 0.00103, 0.00117, 0.00132
	Hispanic	Men	32	0.000813, 0.000925, 0.00105, 0.00119, 0.00136
	Hispanic	Men	33	0.000803, 0.000926, 0.00107, 0.00123, 0.00141
	Hispanic	Men	34	0.000787, 0.000922, 0.00108, 0.00126, 0.00148
	Hispanic	Men	35	0.000778, 0.000922, 0.00109, 0.00129, 0.00153
	Hispanic	Men	36	0.000782, 0.00093, 0.00111, 0.00131, 0.00156
	Hispanic	Men	37	0.0008, 0.000951, 0.00113, 0.00134, 0.00159
	Hispanic	Men	38	0.000831, 0.000982, 0.00116, 0.00137, 0.00162
	Hispanic	Men	39	0.000868, 0.00102, 0.0012, 0.00141, 0.00166
	Hispanic	Men	40	0.000911, 0.00107, 0.00126, 0.00148, 0.00174
	Hispanic	Men	41	0.000963, 0.00113, 0.00133, 0.00156, 0.00183
	Hispanic	Men	42	0.00104, 0.00122, 0.00142, 0.00166, 0.00194
	Hispanic	Men	43	0.00115, 0.00133, 0.00153, 0.00177, 0.00205
	Hispanic	Men	44	0.0013, 0.00148, 0.00168, 0.0019, 0.00216
	Hispanic	Men	45	0.00148, 0.00165, 0.00185, 0.00206, 0.00231
	Hispanic	Men	46	0.00166, 0.00185, 0.00205, 0.00228, 0.00254
	Hispanic	Men	47	0.00185, 0.00206, 0.00229, 0.00254, 0.00283
	Hispanic	Men	48	0.00206, 0.00229, 0.00256, 0.00285, 0.00318
	Hispanic	Men	49	0.00229, 0.00256, 0.00285, 0.00318, 0.00356
	Hispanic	Men	50	0.00256, 0.00285, 0.00317, 0.00353, 0.00393
	Hispanic	Men	51	0.0029, 0.00319, 0.00351, 0.00387, 0.00426
	Hispanic	Men	52	0.00331, 0.00358, 0.00387, 0.00418, 0.00453
	Hispanic	Men	53	0.00379, 0.00401, 0.00425, 0.00449, 0.00476
	Hispanic	Men	54	0.00423, 0.00443, 0.00464, 0.00485, 0.00509
	Hispanic	Men	55	0.00454, 0.00478, 0.00504, 0.00531, 0.0056
	Hispanic	Men	56	0.00482, 0.00513, 0.00546, 0.00581, 0.00619
	Hispanic	Men	57	0.00512, 0.0055, 0.0059, 0.00632, 0.00679
	Hispanic	Men	58	0.00544, 0.00587, 0.00634, 0.00684, 0.00739
	Hispanic	Men	59	0.00575, 0.00625, 0.00678, 0.00737, 0.008
	Hispanic	Men	60	0.00607, 0.00663, 0.00723, 0.00789, 0.00861
	Hispanic	Men	61	0.00644, 0.00704, 0.00768, 0.00838, 0.00915
	Hispanic	Men	62	0.00686, 0.00748, 0.00814, 0.00885, 0.00964
	Hispanic	Men	63	0.00731, 0.00794, 0.00861, 0.00934, 0.0101

Variable	Race/ethnicity	Sex	Age	Distribution
	Hispanic	Men	64	0.00777, 0.00842, 0.00913, 0.0099, 0.0107
	Hispanic	Men	65	0.00822, 0.00894, 0.00971, 0.0105, 0.0115
	Hispanic	Men	66	0.00872, 0.00951, 0.0104, 0.0113, 0.0123
	Hispanic	Men	67	0.00928, 0.0102, 0.0111, 0.0122, 0.0134
	Hispanic	Men	68	0.00995, 0.0109, 0.012, 0.0132, 0.0145
	Hispanic	Men	69	0.0107, 0.0118, 0.013, 0.0143, 0.0158
	Hispanic	Men	70	0.0116, 0.0128, 0.0141, 0.0156, 0.0172
	Hispanic	Men	71	0.0126, 0.0139, 0.0154, 0.017, 0.0188
	Hispanic	Men	72	0.0137, 0.0152, 0.0168, 0.0186, 0.0206
	Hispanic	Men	73	0.0149, 0.0166, 0.0183, 0.0203, 0.0225
	Hispanic	Men	74	0.0163, 0.0181, 0.02, 0.0222, 0.0246
	Hispanic	Men	75	0.018, 0.0199, 0.022, 0.0243, 0.0268
	Hispanic	Men	76	0.0198, 0.0219, 0.0242, 0.0267, 0.0294
	Hispanic	Men	77	0.022, 0.0243, 0.0267, 0.0294, 0.0325
	Hispanic	Men	78	0.0245, 0.027, 0.0297, 0.0327, 0.0361
	Hispanic	Men	79	0.0273, 0.0301, 0.0331, 0.0365, 0.0402
	Hispanic	Men	80	0.0306, 0.0336, 0.037, 0.0407, 0.0448
	Hispanic	Men	81	0.0341, 0.0376, 0.0413, 0.0454, 0.05
	Hispanic	Men	82	0.0379, 0.0417, 0.0459, 0.0505, 0.0557
	Hispanic	Men	83	0.0415, 0.0459, 0.0507, 0.0559, 0.0618
	Hispanic	Men	84	0.0451, 0.05, 0.0553, 0.0613, 0.068
	Hispanic	Women	30	0.000326, 0.000368, 0.000415, 0.000468, 0.000528
	Hispanic	Women	31	0.000346, 0.000389, 0.000436, 0.00049, 0.000551
	Hispanic	Women	32	0.000365, 0.000409, 0.000458, 0.000512, 0.000574
	Hispanic	Women	33	0.000382, 0.000428, 0.000479, 0.000536, 0.0006
	Hispanic	Women	34	0.000394, 0.000444, 0.000499, 0.000562, 0.000633
	Hispanic	Women	35	0.000402, 0.000457, 0.00052, 0.00059, 0.000671
	Hispanic	Women	36	0.000411, 0.000472, 0.000541, 0.00062, 0.000712
	Hispanic	Women	37	0.000424, 0.000489, 0.000565, 0.000651, 0.000752
	Hispanic	Women	38	0.000442, 0.000512, 0.000592, 0.000684, 0.000791
	Hispanic	Women	39	0.000468, 0.000541, 0.000624, 0.000719, 0.000831
	Hispanic	Women	40	0.000502, 0.000577, 0.000662, 0.00076, 0.000873
	Hispanic	Women	41	0.000544, 0.000621, 0.000708, 0.000807, 0.00092
	Hispanic	Women	42	0.000597, 0.000675, 0.000763, 0.000862, 0.000976
	Hispanic	Women	43	0.000662, 0.000742, 0.00083, 0.000928, 0.00104
	Hispanic	Women	44	0.00074, 0.000821, 0.000909, 0.00101, 0.00112
	Hispanic	Women	45	0.000827, 0.000911, 0.001, 0.0011, 0.00122
	Hispanic	Women	46	0.000922, 0.00101, 0.00111, 0.00122, 0.00134
	Hispanic	Women	47	0.00102, 0.00113, 0.00124, 0.00136, 0.00149
	Hispanic	Women	48	0.00113, 0.00125, 0.00138, 0.00152, 0.00167
	Hispanic	Women	49	0.00126, 0.00139, 0.00153, 0.00169, 0.00187
	Hispanic	Women	50	0.0014, 0.00154, 0.0017, 0.00187, 0.00207
	Hispanic	Women	51	0.00156, 0.00171, 0.00188, 0.00207, 0.00227

Variable	Race/ethnicity	Sex	Age	Distribution
	Hispanic	Women	52	0.00175, 0.00191, 0.00208, 0.00226, 0.00246
	Hispanic	Women	53	0.00195, 0.00211, 0.00228, 0.00246, 0.00266
	Hispanic	Women	54	0.00215, 0.00232, 0.0025, 0.00269, 0.00291
	Hispanic	Women	55	0.00233, 0.00252, 0.00273, 0.00295, 0.00319
	Hispanic	Women	56	0.00252, 0.00273, 0.00296, 0.00321, 0.00349
	Hispanic	Women	57	0.00272, 0.00295, 0.00321, 0.00349, 0.00379
	Hispanic	Women	58	0.00293, 0.00319, 0.00347, 0.00377, 0.0041
	Hispanic	Women	59	0.00314, 0.00343, 0.00374, 0.00407, 0.00444
	Hispanic	Women	60	0.00336, 0.00368, 0.00402, 0.00439, 0.0048
	Hispanic	Women	61	0.0036, 0.00394, 0.00431, 0.00472, 0.00517
	Hispanic	Women	62	0.00386, 0.00423, 0.00463, 0.00506, 0.00554
	Hispanic	Women	63	0.00414, 0.00454, 0.00497, 0.00543, 0.00595
	Hispanic	Women	64	0.00444, 0.00487, 0.00534, 0.00585, 0.00642
	Hispanic	Women	65	0.00476, 0.00524, 0.00576, 0.00633, 0.00696
	Hispanic	Women	66	0.00513, 0.00566, 0.00623, 0.00687, 0.00757
	Hispanic	Women	67	0.00556, 0.00614, 0.00677, 0.00748, 0.00826
	Hispanic	Women	68	0.00606, 0.00669, 0.00739, 0.00816, 0.00902
	Hispanic	Women	69	0.00663, 0.00733, 0.00809, 0.00893, 0.00986
	Hispanic	Women	70	0.00728, 0.00804, 0.00887, 0.00979, 0.0108
	Hispanic	Women	71	0.00801, 0.00884, 0.00975, 0.0108, 0.0119
	Hispanic	Women	72	0.00882, 0.00974, 0.0107, 0.0118, 0.0131
	Hispanic	Women	73	0.00975, 0.0107, 0.0118, 0.013, 0.0144
	Hispanic	Women	74	0.0108, 0.0119, 0.0131, 0.0144, 0.0158
	Hispanic	Women	75	0.0121, 0.0133, 0.0145, 0.0159, 0.0174
	Hispanic	Women	76	0.0136, 0.0148, 0.0162, 0.0176, 0.0193
	Hispanic	Women	77	0.0153, 0.0167, 0.0181, 0.0197, 0.0214
	Hispanic	Women	78	0.0173, 0.0188, 0.0204, 0.0221, 0.024
	Hispanic	Women	79	0.0195, 0.0212, 0.023, 0.025, 0.0272
	Hispanic	Women	80	0.022, 0.0239, 0.026, 0.0283, 0.0308
	Hispanic	Women	81	0.0247, 0.0269, 0.0294, 0.032, 0.035
	Hispanic	Women	82	0.0275, 0.0301, 0.033, 0.0362, 0.0397
	Hispanic	Women	83	0.0304, 0.0335, 0.0369, 0.0407, 0.0448
	Hispanic	Women	84	0.0332, 0.0369, 0.0409, 0.0453, 0.0502
	White	Men	30	0.00107, 0.0013, 0.00159, 0.00194, 0.00237
	White	Men	31	0.00112, 0.00136, 0.00163, 0.00197, 0.00238
	White	Men	32	0.00117, 0.0014, 0.00167, 0.00198, 0.00237
	White	Men	33	0.00121, 0.00143, 0.00169, 0.002, 0.00237
	White	Men	34	0.00122, 0.00144, 0.00171, 0.00202, 0.00239
	White	Men	35	0.00122, 0.00145, 0.00172, 0.00204, 0.00242
	White	Men	36	0.00122, 0.00146, 0.00174, 0.00207, 0.00247
	White	Men	37	0.00123, 0.00147, 0.00176, 0.0021, 0.00251
	White	Men	38	0.00126, 0.0015, 0.00179, 0.00214, 0.00256
	White	Men	39	0.0013, 0.00155, 0.00184, 0.00219, 0.00261

Variable	Race/ethnicity	Sex	Age	Distribution
	White	Men	40	0.00136, 0.00161, 0.00191, 0.00226, 0.00268
	White	Men	41	0.00144, 0.0017, 0.002, 0.00236, 0.00278
	White	Men	42	0.00154, 0.00181, 0.00212, 0.00248, 0.00292
	White	Men	43	0.00167, 0.00195, 0.00227, 0.00264, 0.00308
	White	Men	44	0.00184, 0.00213, 0.00246, 0.00284, 0.00329
	White	Men	45	0.00203, 0.00234, 0.0027, 0.0031, 0.00358
	White	Men	46	0.00223, 0.00258, 0.00298, 0.00344, 0.00397
	White	Men	47	0.00245, 0.00285, 0.00331, 0.00384, 0.00447
	White	Men	48	0.0027, 0.00316, 0.00369, 0.0043, 0.00503
	White	Men	49	0.003, 0.00351, 0.00411, 0.0048, 0.00562
	White	Men	50	0.00336, 0.00392, 0.00456, 0.00532, 0.00621
	White	Men	51	0.00377, 0.00437, 0.00505, 0.00584, 0.00677
	White	Men	52	0.00425, 0.00486, 0.00556, 0.00636, 0.00729
	White	Men	53	0.00478, 0.0054, 0.00609, 0.00688, 0.00778
	White	Men	54	0.00531, 0.00594, 0.00664, 0.00742, 0.0083
	White	Men	55	0.0058, 0.00646, 0.00719, 0.008, 0.00892
	White	Men	56	0.00622, 0.00694, 0.00775, 0.00864, 0.00965
	White	Men	57	0.00662, 0.00742, 0.0083, 0.00929, 0.0104
	White	Men	58	0.00704, 0.0079, 0.00885, 0.00992, 0.0111
	White	Men	59	0.00751, 0.0084, 0.00939, 0.0105, 0.0118
	White	Men	60	0.00802, 0.00893, 0.00993, 0.011, 0.0123
	White	Men	61	0.00858, 0.00948, 0.0105, 0.0116, 0.0128
	White	Men	62	0.00918, 0.0101, 0.011, 0.0121, 0.0132
	White	Men	63	0.00979, 0.0107, 0.0116, 0.0127, 0.0138
	White	Men	64	0.0104, 0.0113, 0.0123, 0.0133, 0.0145
	White	Men	65	0.011, 0.012, 0.013, 0.0142, 0.0154
	White	Men	66	0.0117, 0.0128, 0.0139, 0.0152, 0.0165
	White	Men	67	0.0125, 0.0137, 0.015, 0.0164, 0.0179
	White	Men	68	0.0135, 0.0148, 0.0162, 0.0178, 0.0195
	White	Men	69	0.0146, 0.016, 0.0176, 0.0193, 0.0212
	White	Men	70	0.0158, 0.0174, 0.0192, 0.0211, 0.0232
	White	Men	71	0.0173, 0.019, 0.0209, 0.023, 0.0253
	White	Men	72	0.0189, 0.0208, 0.0228, 0.0251, 0.0276
	White	Men	73	0.0206, 0.0227, 0.0249, 0.0273, 0.03
	White	Men	74	0.0226, 0.0248, 0.0272, 0.0298, 0.0326
	White	Men	75	0.0249, 0.0272, 0.0297, 0.0325, 0.0355
	White	Men	76	0.0275, 0.03, 0.0327, 0.0356, 0.0387
	White	Men	77	0.0305, 0.0332, 0.036, 0.0392, 0.0426
	White	Men	78	0.0338, 0.0368, 0.04, 0.0435, 0.0473
	White	Men	79	0.0377, 0.041, 0.0446, 0.0486, 0.0529
	White	Men	80	0.042, 0.0458, 0.05, 0.0544, 0.0594
	White	Men	81	0.0469, 0.0512, 0.0559, 0.0611, 0.0667
	White	Men	82	0.052, 0.057, 0.0624, 0.0683, 0.0749

Variable	Race/ethnicity	Sex	Age	Distribution
	White	Men	83	0.0572, 0.0629, 0.0692, 0.0761, 0.0837
	White	Men	84	0.0623, 0.0689, 0.076, 0.0839, 0.0927
	White	Women	30	0.000561, 0.000645, 0.000741, 0.000851, 0.000978
	White	Women	31	0.000591, 0.000682, 0.000785, 0.000904, 0.00104
	White	Women	32	0.000624, 0.000719, 0.000828, 0.000953, 0.0011
	White	Women	33	0.000655, 0.000754, 0.000867, 0.000998, 0.00115
	White	Women	34	0.000675, 0.000782, 0.000905, 0.00105, 0.00121
	White	Women	35	0.000685, 0.000804, 0.000941, 0.0011, 0.00129
	White	Women	36	0.000695, 0.000826, 0.000979, 0.00116, 0.00138
	White	Women	37	0.000709, 0.000851, 0.00102, 0.00122, 0.00146
	White	Women	38	0.000728, 0.000881, 0.00106, 0.00129, 0.00156
	White	Women	39	0.000751, 0.000917, 0.00112, 0.00136, 0.00166
	White	Women	40	0.000785, 0.000963, 0.00118, 0.00145, 0.00177
	White	Women	41	0.000833, 0.00102, 0.00126, 0.00154, 0.00189
	White	Women	42	0.000896, 0.0011, 0.00135, 0.00165, 0.00202
	White	Women	43	0.000975, 0.00119, 0.00146, 0.00178, 0.00218
	White	Women	44	0.00107, 0.0013, 0.00159, 0.00194, 0.00237
	White	Women	45	0.00117, 0.00143, 0.00174, 0.00213, 0.0026
	White	Women	46	0.00129, 0.00158, 0.00192, 0.00234, 0.00286
	White	Women	47	0.00144, 0.00175, 0.00212, 0.00257, 0.00313
	White	Women	48	0.00163, 0.00196, 0.00235, 0.00282, 0.00339
	White	Women	49	0.00184, 0.00219, 0.0026, 0.00308, 0.00367
	White	Women	50	0.00205, 0.00243, 0.00287, 0.00339, 0.004
	White	Women	51	0.00224, 0.00266, 0.00315, 0.00374, 0.00444
	White	Women	52	0.00242, 0.00289, 0.00345, 0.00412, 0.00493
	White	Women	53	0.0026, 0.00313, 0.00375, 0.0045, 0.00541
	White	Women	54	0.00285, 0.0034, 0.00405, 0.00483, 0.00578
	White	Women	55	0.00317, 0.00372, 0.00435, 0.00509, 0.00597
	White	Women	56	0.00357, 0.00407, 0.00464, 0.00529, 0.00604
	White	Women	57	0.004, 0.00444, 0.00492, 0.00546, 0.00607
	White	Women	58	0.0044, 0.00479, 0.00521, 0.00567, 0.00617
	White	Women	59	0.00471, 0.0051, 0.00551, 0.00595, 0.00643
	White	Women	60	0.00498, 0.00539, 0.00583, 0.0063, 0.00682
	White	Women	61	0.00526, 0.00571, 0.00618, 0.0067, 0.00726
	White	Women	62	0.00559, 0.00607, 0.00659, 0.00715, 0.00777
	White	Women	63	0.00598, 0.0065, 0.00706, 0.00767, 0.00834
	White	Women	64	0.00644, 0.007, 0.00761, 0.00827, 0.009
	White	Women	65	0.00695, 0.00757, 0.00825, 0.00898, 0.00979
	White	Women	66	0.00751, 0.00822, 0.00898, 0.00981, 0.0107
	White	Women	67	0.00812, 0.00893, 0.00981, 0.0108, 0.0119
	White	Women	68	0.00879, 0.00972, 0.0108, 0.0119, 0.0132
	White	Women	69	0.00952, 0.0106, 0.0118, 0.0131, 0.0146
	White	Women	70	0.0104, 0.0116, 0.013, 0.0145, 0.0162

Variable	Race/ethnicity	Sex	Age	Distribution
	White	Women	71	0.0113, 0.0127, 0.0142, 0.016, 0.0179
	White	Women	72	0.0124, 0.0139, 0.0156, 0.0176, 0.0198
	White	Women	73	0.0136, 0.0153, 0.0172, 0.0193, 0.0217
	White	Women	74	0.015, 0.0168, 0.0189, 0.0212, 0.0238
	White	Women	75	0.0167, 0.0187, 0.0208, 0.0233, 0.026
	White	Women	76	0.0187, 0.0208, 0.0231, 0.0256, 0.0285
	White	Women	77	0.0211, 0.0233, 0.0257, 0.0284, 0.0313
	White	Women	78	0.0237, 0.0261, 0.0287, 0.0316, 0.0348
	White	Women	79	0.0268, 0.0294, 0.0323, 0.0354, 0.0389
	White	Women	80	0.0301, 0.0331, 0.0363, 0.0398, 0.0437
	White	Women	81	0.0338, 0.0372, 0.0408, 0.0448, 0.0493
	White	Women	82	0.0378, 0.0416, 0.0458, 0.0504, 0.0555
	White	Women	83	0.0418, 0.0462, 0.051, 0.0564, 0.0623
	White	Women	84	0.0459, 0.051, 0.0565, 0.0626, 0.0695
Total mortality rates for 2028 (0.01, 0.2, 0.5, 0.8. 0.99 percentiles of the empirical distribution produced during forecasting)				
	Black	Men	30	0.00156, 0.0019, 0.00231, 0.00281, 0.00342
	Black	Men	31	0.00167, 0.00198, 0.00235, 0.00279, 0.00332
	Black	Men	32	0.00175, 0.00205, 0.00239, 0.00279, 0.00325
	Black	Men	33	0.0018, 0.00209, 0.00242, 0.00282, 0.00327
	Black	Men	34	0.00182, 0.00212, 0.00246, 0.00287, 0.00334
	Black	Men	35	0.00184, 0.00215, 0.0025, 0.00292, 0.00341
	Black	Men	36	0.00187, 0.00218, 0.00255, 0.00297, 0.00347
	Black	Men	37	0.0019, 0.00222, 0.00258, 0.00301, 0.00352
	Black	Men	38	0.00192, 0.00225, 0.00262, 0.00307, 0.00358
	Black	Men	39	0.00194, 0.00228, 0.00267, 0.00314, 0.00369
	Black	Men	40	0.00195, 0.00232, 0.00274, 0.00325, 0.00385
	Black	Men	41	0.00197, 0.00237, 0.00284, 0.00341, 0.00409
	Black	Men	42	0.00202, 0.00245, 0.00298, 0.00362, 0.0044
	Black	Men	43	0.00209, 0.00257, 0.00317, 0.0039, 0.00481
	Black	Men	44	0.00219, 0.00273, 0.00341, 0.00425, 0.00531
	Black	Men	45	0.00232, 0.00293, 0.0037, 0.00467, 0.00591
	Black	Men	46	0.00249, 0.00318, 0.00406, 0.00517, 0.0066
	Black	Men	47	0.00273, 0.0035, 0.00447, 0.00571, 0.00732
	Black	Men	48	0.00305, 0.00389, 0.00496, 0.00631, 0.00806
	Black	Men	49	0.00345, 0.00437, 0.00552, 0.00696, 0.00881
	Black	Men	50	0.00393, 0.00492, 0.00614, 0.00767, 0.00961
	Black	Men	51	0.00448, 0.00555, 0.00684, 0.00845, 0.0104
	Black	Men	52	0.00513, 0.00626, 0.00761, 0.00926, 0.0113
	Black	Men	53	0.00589, 0.00706, 0.00844, 0.0101, 0.0121
	Black	Men	54	0.00676, 0.00795, 0.00933, 0.011, 0.0129
	Black	Men	55	0.00772, 0.00891, 0.0103, 0.0118, 0.0137
	Black	Men	56	0.00874, 0.00994, 0.0113, 0.0128, 0.0145
	Black	Men	57	0.00984, 0.011, 0.0123, 0.0138, 0.0154

Variable	Race/ethnicity	Sex	Age	Distribution
	Black	Men	58	0.011, 0.0121, 0.0134, 0.0148, 0.0164
	Black	Men	59	0.012, 0.0132, 0.0145, 0.0159, 0.0174
	Black	Men	60	0.013, 0.0142, 0.0155, 0.017, 0.0186
	Black	Men	61	0.0139, 0.0152, 0.0165, 0.018, 0.0196
	Black	Men	62	0.0149, 0.0161, 0.0175, 0.0189, 0.0205
	Black	Men	63	0.0157, 0.017, 0.0184, 0.0199, 0.0216
	Black	Men	64	0.0164, 0.0178, 0.0193, 0.0209, 0.0227
	Black	Men	65	0.0171, 0.0186, 0.0202, 0.0219, 0.0238
	Black	Men	66	0.0179, 0.0195, 0.0212, 0.0231, 0.0251
	Black	Men	67	0.0187, 0.0205, 0.0224, 0.0244, 0.0267
	Black	Men	68	0.0197, 0.0216, 0.0237, 0.026, 0.0285
	Black	Men	69	0.0208, 0.0229, 0.0252, 0.0277, 0.0305
	Black	Men	70	0.0222, 0.0245, 0.027, 0.0297, 0.0328
	Black	Men	71	0.0237, 0.0262, 0.0289, 0.0319, 0.0353
	Black	Men	72	0.0253, 0.028, 0.031, 0.0343, 0.0379
	Black	Men	73	0.0272, 0.0301, 0.0333, 0.0368, 0.0407
	Black	Men	74	0.0294, 0.0324, 0.0357, 0.0394, 0.0435
	Black	Men	75	0.0319, 0.035, 0.0384, 0.0422, 0.0464
	Black	Men	76	0.0346, 0.0379, 0.0414, 0.0453, 0.0496
	Black	Men	77	0.0376, 0.041, 0.0448, 0.0489, 0.0534
	Black	Men	78	0.0407, 0.0445, 0.0485, 0.053, 0.0579
	Black	Men	79	0.0441, 0.0482, 0.0528, 0.0577, 0.0632
	Black	Men	80	0.0476, 0.0523, 0.0574, 0.063, 0.0692
	Black	Men	81	0.0514, 0.0567, 0.0624, 0.0687, 0.0758
	Black	Men	82	0.0553, 0.0612, 0.0676, 0.0748, 0.0828
	Black	Men	83	0.059, 0.0656, 0.0729, 0.081, 0.0901
	Black	Men	84	0.0625, 0.0699, 0.078, 0.0871, 0.0973
	Black	Women	30	0.000544, 0.000702, 0.000905, 0.00117, 0.00151
	Black	Women	31	0.000615, 0.000776, 0.000976, 0.00123, 0.00155
	Black	Women	32	0.000698, 0.000858, 0.00105, 0.00129, 0.00158
	Black	Women	33	0.000781, 0.00094, 0.00113, 0.00136, 0.00163
	Black	Women	34	0.000847, 0.00101, 0.0012, 0.00143, 0.00171
	Black	Women	35	0.000889, 0.00106, 0.00127, 0.00151, 0.00181
	Black	Women	36	0.000921, 0.00111, 0.00133, 0.0016, 0.00192
	Black	Women	37	0.000954, 0.00115, 0.00139, 0.00167, 0.00202
	Black	Women	38	0.000995, 0.0012, 0.00145, 0.00175, 0.00212
	Black	Women	39	0.00105, 0.00127, 0.00153, 0.00184, 0.00223
	Black	Women	40	0.00112, 0.00135, 0.00162, 0.00195, 0.00235
	Black	Women	41	0.00122, 0.00145, 0.00174, 0.00207, 0.00248
	Black	Women	42	0.00134, 0.00159, 0.00187, 0.00221, 0.00261
	Black	Women	43	0.00151, 0.00175, 0.00203, 0.00236, 0.00274
	Black	Women	44	0.0017, 0.00194, 0.00222, 0.00253, 0.00289
	Black	Women	45	0.0019, 0.00215, 0.00243, 0.00274, 0.0031

Variable	Race/ethnicity	Sex	Age	Distribution
	Black	Women	46	0.0021, 0.00237, 0.00268, 0.00302, 0.00341
	Black	Women	47	0.0023, 0.00261, 0.00296, 0.00336, 0.00382
	Black	Women	48	0.00252, 0.00288, 0.00329, 0.00376, 0.0043
	Black	Women	49	0.00279, 0.00319, 0.00366, 0.00418, 0.0048
	Black	Women	50	0.00313, 0.00356, 0.00406, 0.00462, 0.00527
	Black	Women	51	0.00356, 0.004, 0.00449, 0.00505, 0.00568
	Black	Women	52	0.00406, 0.00449, 0.00496, 0.00548, 0.00606
	Black	Women	53	0.00457, 0.00499, 0.00544, 0.00594, 0.00648
	Black	Women	54	0.00507, 0.0055, 0.00596, 0.00645, 0.007
	Black	Women	55	0.00554, 0.00599, 0.00648, 0.00701, 0.00759
	Black	Women	56	0.00599, 0.00649, 0.00702, 0.0076, 0.00823
	Black	Women	57	0.00644, 0.00698, 0.00756, 0.00819, 0.00887
	Black	Women	58	0.00687, 0.00745, 0.00808, 0.00876, 0.00951
	Black	Women	59	0.00725, 0.00789, 0.00858, 0.00933, 0.0101
	Black	Women	60	0.00759, 0.00829, 0.00904, 0.00987, 0.0108
	Black	Women	61	0.00794, 0.00868, 0.00949, 0.0104, 0.0113
	Black	Women	62	0.00832, 0.00909, 0.00993, 0.0109, 0.0119
	Black	Women	63	0.00871, 0.00953, 0.0104, 0.0114, 0.0125
	Black	Women	64	0.00913, 0.01, 0.011, 0.012, 0.0132
	Black	Women	65	0.00961, 0.0106, 0.0116, 0.0127, 0.014
	Black	Women	66	0.0102, 0.0112, 0.0124, 0.0136, 0.015
	Black	Women	67	0.0109, 0.012, 0.0132, 0.0146, 0.0161
	Black	Women	68	0.0117, 0.0129, 0.0143, 0.0157, 0.0174
	Black	Women	69	0.0126, 0.0139, 0.0154, 0.017, 0.0188
	Black	Women	70	0.0136, 0.015, 0.0166, 0.0184, 0.0204
	Black	Women	71	0.0147, 0.0163, 0.018, 0.0199, 0.022
	Black	Women	72	0.0159, 0.0176, 0.0194, 0.0214, 0.0237
	Black	Women	73	0.0172, 0.019, 0.021, 0.0231, 0.0256
	Black	Women	74	0.0187, 0.0206, 0.0227, 0.025, 0.0275
	Black	Women	75	0.0205, 0.0225, 0.0247, 0.0271, 0.0297
	Black	Women	76	0.0225, 0.0246, 0.0269, 0.0294, 0.0322
	Black	Women	77	0.0247, 0.027, 0.0294, 0.0321, 0.0351
	Black	Women	78	0.0272, 0.0297, 0.0324, 0.0353, 0.0385
	Black	Women	79	0.0299, 0.0327, 0.0357, 0.0389, 0.0425
	Black	Women	80	0.0329, 0.036, 0.0393, 0.043, 0.047
	Black	Women	81	0.036, 0.0395, 0.0433, 0.0475, 0.0521
	Black	Women	82	0.0392, 0.0432, 0.0476, 0.0524, 0.0578
	Black	Women	83	0.0423, 0.0469, 0.0519, 0.0575, 0.0638
	Black	Women	84	0.0454, 0.0506, 0.0564, 0.0628, 0.0701
	Hispanic	Men	30	0.000787, 0.000894, 0.00101, 0.00115, 0.00131
	Hispanic	Men	31	0.000805, 0.000913, 0.00103, 0.00117, 0.00133
	Hispanic	Men	32	0.000811, 0.000924, 0.00105, 0.0012, 0.00136
	Hispanic	Men	33	0.0008, 0.000924, 0.00107, 0.00123, 0.00142

Variable	Race/ethnicity	Sex	Age	Distribution
	Hispanic	Men	34	0.000782, 0.000918, 0.00108, 0.00126, 0.00148
	Hispanic	Men	35	0.000771, 0.000916, 0.00109, 0.00129, 0.00153
	Hispanic	Men	36	0.000773, 0.000923, 0.0011, 0.00131, 0.00157
	Hispanic	Men	37	0.000789, 0.000941, 0.00112, 0.00133, 0.00159
	Hispanic	Men	38	0.000818, 0.00097, 0.00115, 0.00136, 0.00161
	Hispanic	Men	39	0.000853, 0.00101, 0.00119, 0.0014, 0.00166
	Hispanic	Men	40	0.000893, 0.00105, 0.00124, 0.00146, 0.00173
	Hispanic	Men	41	0.000943, 0.00111, 0.00131, 0.00154, 0.00182
	Hispanic	Men	42	0.00102, 0.00119, 0.0014, 0.00164, 0.00193
	Hispanic	Men	43	0.00113, 0.00131, 0.00151, 0.00175, 0.00203
	Hispanic	Men	44	0.00127, 0.00145, 0.00165, 0.00188, 0.00214
	Hispanic	Men	45	0.00145, 0.00162, 0.00182, 0.00204, 0.00229
	Hispanic	Men	46	0.00163, 0.00181, 0.00202, 0.00225, 0.00251
	Hispanic	Men	47	0.00181, 0.00202, 0.00226, 0.00251, 0.0028
	Hispanic	Men	48	0.00202, 0.00226, 0.00252, 0.00282, 0.00315
	Hispanic	Men	49	0.00225, 0.00252, 0.00282, 0.00315, 0.00353
	Hispanic	Men	50	0.00252, 0.00281, 0.00314, 0.0035, 0.00391
	Hispanic	Men	51	0.00286, 0.00315, 0.00348, 0.00384, 0.00424
	Hispanic	Men	52	0.00327, 0.00355, 0.00384, 0.00416, 0.0045
	Hispanic	Men	53	0.00375, 0.00398, 0.00421, 0.00446, 0.00473
	Hispanic	Men	54	0.00418, 0.00439, 0.0046, 0.00482, 0.00506
	Hispanic	Men	55	0.00449, 0.00474, 0.00501, 0.00528, 0.00558
	Hispanic	Men	56	0.00477, 0.00509, 0.00542, 0.00578, 0.00616
	Hispanic	Men	57	0.00506, 0.00545, 0.00585, 0.00629, 0.00676
	Hispanic	Men	58	0.00537, 0.00581, 0.00629, 0.0068, 0.00736
	Hispanic	Men	59	0.00568, 0.00618, 0.00673, 0.00732, 0.00797
	Hispanic	Men	60	0.00599, 0.00655, 0.00716, 0.00783, 0.00857
	Hispanic	Men	61	0.00634, 0.00694, 0.0076, 0.00831, 0.0091
	Hispanic	Men	62	0.00675, 0.00737, 0.00804, 0.00877, 0.00957
	Hispanic	Men	63	0.00718, 0.00782, 0.0085, 0.00924, 0.0101
	Hispanic	Men	64	0.00762, 0.00829, 0.009, 0.00978, 0.0106
	Hispanic	Men	65	0.00806, 0.00878, 0.00957, 0.0104, 0.0114
	Hispanic	Men	66	0.00854, 0.00934, 0.0102, 0.0112, 0.0122
	Hispanic	Men	67	0.00909, 0.00999, 0.011, 0.012, 0.0132
	Hispanic	Men	68	0.00974, 0.0107, 0.0118, 0.013, 0.0144
	Hispanic	Men	69	0.0105, 0.0116, 0.0128, 0.0142, 0.0156
	Hispanic	Men	70	0.0114, 0.0126, 0.0139, 0.0154, 0.0171
	Hispanic	Men	71	0.0123, 0.0137, 0.0152, 0.0168, 0.0186
	Hispanic	Men	72	0.0134, 0.0149, 0.0165, 0.0183, 0.0204
	Hispanic	Men	73	0.0146, 0.0162, 0.018, 0.02, 0.0223
	Hispanic	Men	74	0.016, 0.0178, 0.0197, 0.0219, 0.0243
	Hispanic	Men	75	0.0176, 0.0195, 0.0216, 0.0239, 0.0266
	Hispanic	Men	76	0.0194, 0.0215, 0.0238, 0.0263, 0.0291

Variable	Race/ethnicity	Sex	Age	Distribution
	Hispanic	Men	77	0.0215, 0.0238, 0.0263, 0.0291, 0.0322
	Hispanic	Men	78	0.024, 0.0265, 0.0293, 0.0323, 0.0357
	Hispanic	Men	79	0.0268, 0.0296, 0.0326, 0.036, 0.0398
	Hispanic	Men	80	0.03, 0.0331, 0.0365, 0.0402, 0.0444
	Hispanic	Men	81	0.0335, 0.0369, 0.0407, 0.0449, 0.0496
	Hispanic	Men	82	0.0371, 0.041, 0.0453, 0.05, 0.0552
	Hispanic	Men	83	0.0407, 0.0451, 0.05, 0.0553, 0.0612
	Hispanic	Men	84	0.0442, 0.0491, 0.0546, 0.0606, 0.0674
	Hispanic	Women	30	0.000325, 0.000367, 0.000415, 0.000468, 0.000528
	Hispanic	Women	31	0.000345, 0.000388, 0.000436, 0.00049, 0.000551
	Hispanic	Women	32	0.000365, 0.000409, 0.000457, 0.000512, 0.000574
	Hispanic	Women	33	0.000381, 0.000427, 0.000478, 0.000535, 0.0006
	Hispanic	Women	34	0.000392, 0.000442, 0.000498, 0.000561, 0.000633
	Hispanic	Women	35	0.000399, 0.000455, 0.000517, 0.000589, 0.000672
	Hispanic	Women	36	0.000406, 0.000468, 0.000538, 0.000618, 0.000712
	Hispanic	Women	37	0.000418, 0.000484, 0.00056, 0.000648, 0.000751
	Hispanic	Women	38	0.000435, 0.000505, 0.000586, 0.000679, 0.000788
	Hispanic	Women	39	0.000459, 0.000532, 0.000616, 0.000713, 0.000826
	Hispanic	Women	40	0.000492, 0.000567, 0.000653, 0.000751, 0.000866
	Hispanic	Women	41	0.000532, 0.00061, 0.000697, 0.000797, 0.000913
	Hispanic	Women	42	0.000583, 0.000662, 0.000751, 0.000851, 0.000966
	Hispanic	Women	43	0.000647, 0.000727, 0.000816, 0.000916, 0.00103
	Hispanic	Women	44	0.000724, 0.000805, 0.000894, 0.000993, 0.0011
	Hispanic	Women	45	0.00081, 0.000894, 0.000986, 0.00109, 0.0012
	Hispanic	Women	46	0.000903, 0.000994, 0.00109, 0.0012, 0.00133
	Hispanic	Women	47	0.001, 0.00111, 0.00122, 0.00134, 0.00148
	Hispanic	Women	48	0.00111, 0.00123, 0.00136, 0.0015, 0.00165
	Hispanic	Women	49	0.00123, 0.00137, 0.00151, 0.00167, 0.00185
	Hispanic	Women	50	0.00137, 0.00152, 0.00168, 0.00186, 0.00206
	Hispanic	Women	51	0.00154, 0.00169, 0.00186, 0.00205, 0.00225
	Hispanic	Women	52	0.00173, 0.00189, 0.00206, 0.00224, 0.00245
	Hispanic	Women	53	0.00193, 0.00209, 0.00226, 0.00245, 0.00265
	Hispanic	Women	54	0.00213, 0.0023, 0.00248, 0.00267, 0.00289
	Hispanic	Women	55	0.00231, 0.0025, 0.00271, 0.00293, 0.00317
	Hispanic	Women	56	0.00249, 0.00271, 0.00294, 0.00319, 0.00347
	Hispanic	Women	57	0.00269, 0.00293, 0.00319, 0.00347, 0.00378
	Hispanic	Women	58	0.0029, 0.00316, 0.00344, 0.00375, 0.00409
	Hispanic	Women	59	0.00311, 0.00339, 0.00371, 0.00405, 0.00442
	Hispanic	Women	60	0.00332, 0.00363, 0.00398, 0.00436, 0.00478
	Hispanic	Women	61	0.00354, 0.00389, 0.00427, 0.00468, 0.00514
	Hispanic	Women	62	0.0038, 0.00417, 0.00457, 0.00501, 0.0055
	Hispanic	Women	63	0.00407, 0.00447, 0.0049, 0.00538, 0.0059
	Hispanic	Women	64	0.00436, 0.0048, 0.00527, 0.00578, 0.00636

Variable	Race/ethnicity	Sex	Age	Distribution
	Hispanic	Women	65	0.00468, 0.00515, 0.00568, 0.00625, 0.00689
	Hispanic	Women	66	0.00503, 0.00556, 0.00614, 0.00678, 0.00749
	Hispanic	Women	67	0.00545, 0.00603, 0.00667, 0.00738, 0.00817
	Hispanic	Women	68	0.00593, 0.00657, 0.00728, 0.00805, 0.00892
	Hispanic	Women	69	0.00649, 0.00719, 0.00796, 0.00881, 0.00976
	Hispanic	Women	70	0.00713, 0.00789, 0.00873, 0.00966, 0.0107
	Hispanic	Women	71	0.00784, 0.00868, 0.0096, 0.0106, 0.0118
	Hispanic	Women	72	0.00863, 0.00955, 0.0106, 0.0117, 0.0129
	Hispanic	Women	73	0.00953, 0.0105, 0.0116, 0.0129, 0.0142
	Hispanic	Women	74	0.0106, 0.0117, 0.0129, 0.0142, 0.0157
	Hispanic	Women	75	0.0118, 0.013, 0.0143, 0.0157, 0.0172
	Hispanic	Women	76	0.0133, 0.0146, 0.0159, 0.0174, 0.019
	Hispanic	Women	77	0.015, 0.0164, 0.0178, 0.0194, 0.0212
	Hispanic	Women	78	0.0169, 0.0184, 0.0201, 0.0218, 0.0238
	Hispanic	Women	79	0.0191, 0.0208, 0.0226, 0.0247, 0.0269
	Hispanic	Women	80	0.0215, 0.0235, 0.0256, 0.0279, 0.0305
	Hispanic	Women	81	0.0242, 0.0265, 0.0289, 0.0316, 0.0346
	Hispanic	Women	82	0.0269, 0.0296, 0.0326, 0.0358, 0.0393
	Hispanic	Women	83	0.0298, 0.0329, 0.0364, 0.0402, 0.0444
	Hispanic	Women	84	0.0326, 0.0363, 0.0403, 0.0447, 0.0497
	White	Men	30	0.00105, 0.00129, 0.00159, 0.00195, 0.0024
	White	Men	31	0.00111, 0.00134, 0.00163, 0.00198, 0.0024
	White	Men	32	0.00116, 0.00139, 0.00166, 0.00199, 0.00239
	White	Men	33	0.00119, 0.00142, 0.00169, 0.002, 0.00239
	White	Men	34	0.0012, 0.00143, 0.0017, 0.00202, 0.0024
	White	Men	35	0.0012, 0.00143, 0.00171, 0.00204, 0.00244
	White	Men	36	0.0012, 0.00144, 0.00172, 0.00207, 0.00248
	White	Men	37	0.00121, 0.00145, 0.00174, 0.00209, 0.00252
	White	Men	38	0.00123, 0.00148, 0.00177, 0.00213, 0.00256
	White	Men	39	0.00127, 0.00152, 0.00182, 0.00217, 0.00261
	White	Men	40	0.00133, 0.00158, 0.00188, 0.00224, 0.00267
	White	Men	41	0.0014, 0.00166, 0.00197, 0.00233, 0.00277
	White	Men	42	0.00149, 0.00177, 0.00208, 0.00245, 0.0029
	White	Men	43	0.00163, 0.00191, 0.00223, 0.00261, 0.00306
	White	Men	44	0.00179, 0.00208, 0.00242, 0.00281, 0.00326
	White	Men	45	0.00198, 0.00229, 0.00265, 0.00306, 0.00355
	White	Men	46	0.00217, 0.00253, 0.00293, 0.0034, 0.00395
	White	Men	47	0.00239, 0.00279, 0.00326, 0.0038, 0.00444
	White	Men	48	0.00264, 0.0031, 0.00363, 0.00426, 0.00501
	White	Men	49	0.00293, 0.00345, 0.00405, 0.00476, 0.0056
	White	Men	50	0.00328, 0.00385, 0.00451, 0.00528, 0.0062
	White	Men	51	0.00369, 0.0043, 0.005, 0.00581, 0.00676
	White	Men	52	0.00416, 0.00479, 0.00551, 0.00633, 0.00728

Variable	Race/ethnicity	Sex	Age	Distribution
	White	Men	53	0.00469, 0.00532, 0.00604, 0.00685, 0.00777
	White	Men	54	0.00522, 0.00586, 0.00658, 0.00738, 0.0083
	White	Men	55	0.0057, 0.00638, 0.00713, 0.00797, 0.00891
	White	Men	56	0.00612, 0.00686, 0.00768, 0.0086, 0.00965
	White	Men	57	0.00651, 0.00733, 0.00823, 0.00925, 0.0104
	White	Men	58	0.00693, 0.0078, 0.00878, 0.00987, 0.0111
	White	Men	59	0.00738, 0.00829, 0.00931, 0.0104, 0.0117
	White	Men	60	0.00788, 0.0088, 0.00983, 0.011, 0.0123
	White	Men	61	0.00843, 0.00934, 0.0104, 0.0115, 0.0127
	White	Men	62	0.00901, 0.00991, 0.0109, 0.012, 0.0132
	White	Men	63	0.0096, 0.0105, 0.0115, 0.0125, 0.0137
	White	Men	64	0.0102, 0.0111, 0.0121, 0.0132, 0.0144
	White	Men	65	0.0108, 0.0118, 0.0129, 0.014, 0.0153
	White	Men	66	0.0115, 0.0126, 0.0137, 0.015, 0.0164
	White	Men	67	0.0123, 0.0135, 0.0147, 0.0162, 0.0177
	White	Men	68	0.0132, 0.0145, 0.0159, 0.0175, 0.0193
	White	Men	69	0.0143, 0.0157, 0.0173, 0.0191, 0.021
	White	Men	70	0.0155, 0.0171, 0.0189, 0.0208, 0.023
	White	Men	71	0.0169, 0.0186, 0.0206, 0.0227, 0.0251
	White	Men	72	0.0184, 0.0204, 0.0224, 0.0247, 0.0273
	White	Men	73	0.0202, 0.0222, 0.0245, 0.027, 0.0297
	White	Men	74	0.0221, 0.0243, 0.0267, 0.0294, 0.0323
	White	Men	75	0.0244, 0.0267, 0.0292, 0.032, 0.0351
	White	Men	76	0.0269, 0.0294, 0.0321, 0.0351, 0.0383
	White	Men	77	0.0298, 0.0325, 0.0355, 0.0386, 0.0422
	White	Men	78	0.0331, 0.0361, 0.0394, 0.0429, 0.0468
	White	Men	79	0.0369, 0.0403, 0.0439, 0.0479, 0.0524
	White	Men	80	0.0412, 0.045, 0.0492, 0.0538, 0.0588
	White	Men	81	0.0459, 0.0503, 0.0551, 0.0603, 0.0661
	White	Men	82	0.0509, 0.056, 0.0615, 0.0675, 0.0742
	White	Men	83	0.056, 0.0618, 0.0682, 0.0752, 0.083
	White	Men	84	0.0611, 0.0677, 0.0749, 0.0829, 0.0919
	White	Women	30	0.000556, 0.000642, 0.000739, 0.000852, 0.000983
	White	Women	31	0.000586, 0.000678, 0.000783, 0.000905, 0.00105
	White	Women	32	0.000618, 0.000714, 0.000825, 0.000953, 0.0011
	White	Women	33	0.000648, 0.000749, 0.000864, 0.000998, 0.00115
	White	Women	34	0.000666, 0.000775, 0.000901, 0.00105, 0.00122
	White	Women	35	0.000675, 0.000795, 0.000936, 0.0011, 0.0013
	White	Women	36	0.000682, 0.000815, 0.000971, 0.00116, 0.00138
	White	Women	37	0.000694, 0.000838, 0.00101, 0.00122, 0.00147
	White	Women	38	0.00071, 0.000865, 0.00105, 0.00128, 0.00156
	White	Women	39	0.000731, 0.000899, 0.0011, 0.00135, 0.00166
	White	Women	40	0.000763, 0.000942, 0.00116, 0.00143, 0.00177

Variable	Race/ethnicity	Sex	Age	Distribution
	White	Women	41	0.000808, 0.001, 0.00123, 0.00152, 0.00189
	White	Women	42	0.000869, 0.00107, 0.00132, 0.00163, 0.00201
	White	Women	43	0.000945, 0.00116, 0.00143, 0.00176, 0.00216
	White	Women	44	0.00103, 0.00127, 0.00156, 0.00191, 0.00235
	White	Women	45	0.00113, 0.00139, 0.00171, 0.0021, 0.00258
	White	Women	46	0.00125, 0.00154, 0.00189, 0.00231, 0.00284
	White	Women	47	0.0014, 0.00171, 0.00209, 0.00255, 0.00311
	White	Women	48	0.00158, 0.00192, 0.00231, 0.00279, 0.00338
	White	Women	49	0.00179, 0.00215, 0.00256, 0.00306, 0.00365
	White	Women	50	0.002, 0.00238, 0.00283, 0.00336, 0.00399
	White	Women	51	0.00219, 0.00261, 0.00311, 0.00371, 0.00443
	White	Women	52	0.00236, 0.00284, 0.00341, 0.0041, 0.00493
	White	Women	53	0.00255, 0.00308, 0.00371, 0.00448, 0.00541
	White	Women	54	0.00279, 0.00335, 0.00402, 0.00482, 0.00579
	White	Women	55	0.00311, 0.00367, 0.00431, 0.00507, 0.00598
	White	Women	56	0.00351, 0.00402, 0.0046, 0.00526, 0.00603
	White	Women	57	0.00394, 0.00439, 0.00488, 0.00543, 0.00605
	White	Women	58	0.00434, 0.00474, 0.00517, 0.00563, 0.00615
	White	Women	59	0.00465, 0.00504, 0.00546, 0.00591, 0.00641
	White	Women	60	0.0049, 0.00532, 0.00577, 0.00626, 0.00679
	White	Women	61	0.00518, 0.00563, 0.00612, 0.00665, 0.00722
	White	Women	62	0.0055, 0.00599, 0.00652, 0.00709, 0.00771
	White	Women	63	0.00588, 0.00641, 0.00697, 0.00759, 0.00827
	White	Women	64	0.00632, 0.00689, 0.00751, 0.00818, 0.00891
	White	Women	65	0.00682, 0.00745, 0.00813, 0.00887, 0.00969
	White	Women	66	0.00736, 0.00807, 0.00884, 0.00969, 0.0106
	White	Women	67	0.00795, 0.00877, 0.00966, 0.0106, 0.0117
	White	Women	68	0.00859, 0.00954, 0.0106, 0.0117, 0.013
	White	Women	69	0.00931, 0.0104, 0.0116, 0.013, 0.0145
	White	Women	70	0.0101, 0.0114, 0.0128, 0.0143, 0.0161
	White	Women	71	0.011, 0.0124, 0.014, 0.0158, 0.0178
	White	Women	72	0.0121, 0.0136, 0.0154, 0.0173, 0.0196
	White	Women	73	0.0133, 0.015, 0.0169, 0.019, 0.0215
	White	Women	74	0.0147, 0.0165, 0.0186, 0.0209, 0.0235
	White	Women	75	0.0163, 0.0183, 0.0205, 0.0229, 0.0257
	White	Women	76	0.0183, 0.0204, 0.0227, 0.0253, 0.0282
	White	Women	77	0.0206, 0.0228, 0.0253, 0.028, 0.031
	White	Women	78	0.0232, 0.0256, 0.0283, 0.0312, 0.0344
	White	Women	79	0.0262, 0.0288, 0.0317, 0.0349, 0.0385
	White	Women	80	0.0295, 0.0325, 0.0357, 0.0393, 0.0433
	White	Women	81	0.0331, 0.0365, 0.0402, 0.0443, 0.0488
	White	Women	82	0.0369, 0.0408, 0.0451, 0.0498, 0.055
	White	Women	83	0.0409, 0.0454, 0.0503, 0.0557, 0.0618

Variable	Race/ethnicity	Sex	Age	Distribution
	White	Women	84	0.0449, 0.05, 0.0557, 0.0619, 0.0689
Total mortality rates for 2029 (0.01, 0.2, 0.5, 0.8. 0.99 percentiles of the empirical distribution produced during forecasting)				
	Black	Men	30	0.00154, 0.00189, 0.00231, 0.00282, 0.00346
	Black	Men	31	0.00165, 0.00197, 0.00235, 0.0028, 0.00335
	Black	Men	32	0.00174, 0.00204, 0.00239, 0.0028, 0.00328
	Black	Men	33	0.00178, 0.00208, 0.00242, 0.00282, 0.0033
	Black	Men	34	0.0018, 0.00211, 0.00246, 0.00287, 0.00336
	Black	Men	35	0.00182, 0.00213, 0.0025, 0.00292, 0.00342
	Black	Men	36	0.00184, 0.00216, 0.00253, 0.00297, 0.00348
	Black	Men	37	0.00187, 0.00219, 0.00256, 0.003, 0.00352
	Black	Men	38	0.00189, 0.00222, 0.0026, 0.00305, 0.00358
	Black	Men	39	0.0019, 0.00224, 0.00264, 0.00311, 0.00368
	Black	Men	40	0.00191, 0.00227, 0.00271, 0.00322, 0.00384
	Black	Men	41	0.00193, 0.00232, 0.0028, 0.00337, 0.00407
	Black	Men	42	0.00196, 0.0024, 0.00293, 0.00358, 0.00438
	Black	Men	43	0.00203, 0.00252, 0.00312, 0.00386, 0.00479
	Black	Men	44	0.00212, 0.00267, 0.00335, 0.00421, 0.00529
	Black	Men	45	0.00225, 0.00287, 0.00364, 0.00463, 0.0059
	Black	Men	46	0.00242, 0.00311, 0.00399, 0.00513, 0.0066
	Black	Men	47	0.00265, 0.00343, 0.00441, 0.00568, 0.00733
	Black	Men	48	0.00297, 0.00381, 0.0049, 0.00628, 0.00808
	Black	Men	49	0.00336, 0.00428, 0.00545, 0.00694, 0.00885
	Black	Men	50	0.00383, 0.00483, 0.00608, 0.00766, 0.00966
	Black	Men	51	0.00438, 0.00546, 0.00678, 0.00844, 0.0105
	Black	Men	52	0.00502, 0.00616, 0.00755, 0.00925, 0.0114
	Black	Men	53	0.00578, 0.00697, 0.00838, 0.0101, 0.0122
	Black	Men	54	0.00664, 0.00785, 0.00927, 0.0109, 0.0129
	Black	Men	55	0.0076, 0.00882, 0.0102, 0.0118, 0.0137
	Black	Men	56	0.00862, 0.00983, 0.0112, 0.0128, 0.0146
	Black	Men	57	0.0097, 0.0109, 0.0122, 0.0137, 0.0155
	Black	Men	58	0.0108, 0.012, 0.0133, 0.0147, 0.0164
	Black	Men	59	0.0119, 0.0131, 0.0144, 0.0158, 0.0174
	Black	Men	60	0.0128, 0.014, 0.0154, 0.0169, 0.0185
	Black	Men	61	0.0137, 0.015, 0.0164, 0.0179, 0.0195
	Black	Men	62	0.0146, 0.0159, 0.0173, 0.0188, 0.0204
	Black	Men	63	0.0154, 0.0167, 0.0181, 0.0197, 0.0214
	Black	Men	64	0.0161, 0.0175, 0.019, 0.0206, 0.0225
	Black	Men	65	0.0168, 0.0183, 0.0199, 0.0217, 0.0236
	Black	Men	66	0.0175, 0.0191, 0.0209, 0.0228, 0.0249
	Black	Men	67	0.0184, 0.0201, 0.022, 0.0241, 0.0264
	Black	Men	68	0.0193, 0.0212, 0.0233, 0.0256, 0.0282
	Black	Men	69	0.0204, 0.0225, 0.0248, 0.0274, 0.0302
	Black	Men	70	0.0217, 0.024, 0.0265, 0.0294, 0.0325

Variable	Race/ethnicity	Sex	Age	Distribution
	Black	Men	71	0.0231, 0.0257, 0.0284, 0.0315, 0.0349
	Black	Men	72	0.0248, 0.0275, 0.0305, 0.0338, 0.0375
	Black	Men	73	0.0266, 0.0295, 0.0327, 0.0363, 0.0402
	Black	Men	74	0.0288, 0.0318, 0.0352, 0.0389, 0.043
	Black	Men	75	0.0312, 0.0344, 0.0378, 0.0416, 0.0459
	Black	Men	76	0.0339, 0.0372, 0.0408, 0.0447, 0.0491
	Black	Men	77	0.0368, 0.0403, 0.0441, 0.0482, 0.0528
	Black	Men	78	0.0399, 0.0437, 0.0478, 0.0523, 0.0573
	Black	Men	79	0.0431, 0.0474, 0.052, 0.057, 0.0626
	Black	Men	80	0.0466, 0.0514, 0.0566, 0.0622, 0.0686
	Black	Men	81	0.0504, 0.0557, 0.0615, 0.0679, 0.0751
	Black	Men	82	0.0541, 0.0601, 0.0667, 0.0739, 0.0821
	Black	Men	83	0.0578, 0.0645, 0.0719, 0.0801, 0.0894
	Black	Men	84	0.0613, 0.0687, 0.0769, 0.0862, 0.0966
	Black	Women	30	0.000533, 0.000696, 0.000905, 0.00118, 0.00154
	Black	Women	31	0.000605, 0.000769, 0.000976, 0.00124, 0.00157
	Black	Women	32	0.000688, 0.000852, 0.00105, 0.0013, 0.00161
	Black	Women	33	0.00077, 0.000933, 0.00113, 0.00136, 0.00165
	Black	Women	34	0.000835, 0.001, 0.0012, 0.00144, 0.00173
	Black	Women	35	0.000875, 0.00105, 0.00126, 0.00152, 0.00183
	Black	Women	36	0.000904, 0.00109, 0.00132, 0.0016, 0.00193
	Black	Women	37	0.000934, 0.00114, 0.00138, 0.00167, 0.00203
	Black	Women	38	0.000972, 0.00118, 0.00144, 0.00175, 0.00213
	Black	Women	39	0.00102, 0.00124, 0.00151, 0.00183, 0.00223
	Black	Women	40	0.00109, 0.00132, 0.0016, 0.00194, 0.00235
	Black	Women	41	0.00118, 0.00142, 0.00171, 0.00206, 0.00248
	Black	Women	42	0.00131, 0.00155, 0.00184, 0.00219, 0.0026
	Black	Women	43	0.00146, 0.00171, 0.002, 0.00233, 0.00273
	Black	Women	44	0.00166, 0.0019, 0.00218, 0.0025, 0.00287
	Black	Women	45	0.00186, 0.00211, 0.00239, 0.00271, 0.00308
	Black	Women	46	0.00205, 0.00233, 0.00264, 0.00299, 0.00339
	Black	Women	47	0.00224, 0.00256, 0.00292, 0.00333, 0.0038
	Black	Women	48	0.00246, 0.00283, 0.00324, 0.00372, 0.00428
	Black	Women	49	0.00273, 0.00314, 0.00361, 0.00415, 0.00478
	Black	Women	50	0.00306, 0.00351, 0.00401, 0.00459, 0.00526
	Black	Women	51	0.00349, 0.00394, 0.00445, 0.00502, 0.00567
	Black	Women	52	0.00399, 0.00443, 0.00491, 0.00545, 0.00605
	Black	Women	53	0.00451, 0.00494, 0.0054, 0.00591, 0.00647
	Black	Women	54	0.005, 0.00544, 0.00591, 0.00642, 0.00699
	Black	Women	55	0.00547, 0.00594, 0.00644, 0.00698, 0.00758
	Black	Women	56	0.00592, 0.00643, 0.00697, 0.00757, 0.00822
	Black	Women	57	0.00636, 0.00691, 0.00751, 0.00815, 0.00885
	Black	Women	58	0.00678, 0.00738, 0.00802, 0.00872, 0.00948

Variable	Race/ethnicity	Sex	Age	Distribution
	Black	Women	59	0.00715, 0.0078, 0.00851, 0.00927, 0.0101
	Black	Women	60	0.00748, 0.00819, 0.00896, 0.0098, 0.0107
	Black	Women	61	0.00781, 0.00857, 0.00939, 0.0103, 0.0113
	Black	Women	62	0.00818, 0.00897, 0.00982, 0.0108, 0.0118
	Black	Women	63	0.00855, 0.00938, 0.0103, 0.0113, 0.0124
	Black	Women	64	0.00896, 0.00985, 0.0108, 0.0119, 0.0131
	Black	Women	65	0.00942, 0.0104, 0.0114, 0.0126, 0.0139
	Black	Women	66	0.00999, 0.011, 0.0122, 0.0134, 0.0149
	Black	Women	67	0.0107, 0.0118, 0.013, 0.0144, 0.016
	Black	Women	68	0.0114, 0.0127, 0.014, 0.0156, 0.0172
	Black	Women	69	0.0123, 0.0137, 0.0152, 0.0168, 0.0186
	Black	Women	70	0.0133, 0.0148, 0.0164, 0.0182, 0.0202
	Black	Women	71	0.0144, 0.016, 0.0177, 0.0196, 0.0218
	Black	Women	72	0.0156, 0.0172, 0.0191, 0.0212, 0.0235
	Black	Women	73	0.0169, 0.0187, 0.0206, 0.0228, 0.0253
	Black	Women	74	0.0183, 0.0202, 0.0223, 0.0247, 0.0272
	Black	Women	75	0.02, 0.0221, 0.0243, 0.0267, 0.0294
	Black	Women	76	0.022, 0.0241, 0.0264, 0.029, 0.0318
	Black	Women	77	0.0242, 0.0265, 0.029, 0.0317, 0.0347
	Black	Women	78	0.0266, 0.0291, 0.0318, 0.0348, 0.0381
	Black	Women	79	0.0293, 0.0321, 0.0351, 0.0384, 0.042
	Black	Women	80	0.0323, 0.0354, 0.0387, 0.0424, 0.0465
	Black	Women	81	0.0353, 0.0388, 0.0427, 0.0469, 0.0516
	Black	Women	82	0.0384, 0.0424, 0.0469, 0.0518, 0.0572
	Black	Women	83	0.0414, 0.0461, 0.0512, 0.0569, 0.0632
	Black	Women	84	0.0445, 0.0497, 0.0556, 0.0621, 0.0694
	Hispanic	Men	30	0.000786, 0.000894, 0.00101, 0.00115, 0.00131
	Hispanic	Men	31	0.000804, 0.000913, 0.00103, 0.00117, 0.00133
	Hispanic	Men	32	0.00081, 0.000924, 0.00105, 0.0012, 0.00137
	Hispanic	Men	33	0.000797, 0.000922, 0.00107, 0.00123, 0.00143
	Hispanic	Men	34	0.000777, 0.000915, 0.00108, 0.00126, 0.00149
	Hispanic	Men	35	0.000764, 0.000911, 0.00109, 0.00129, 0.00154
	Hispanic	Men	36	0.000764, 0.000916, 0.0011, 0.00131, 0.00157
	Hispanic	Men	37	0.000779, 0.000932, 0.00111, 0.00133, 0.00159
	Hispanic	Men	38	0.000805, 0.000959, 0.00114, 0.00135, 0.00161
	Hispanic	Men	39	0.000838, 0.000994, 0.00118, 0.00139, 0.00165
	Hispanic	Men	40	0.000875, 0.00104, 0.00123, 0.00145, 0.00172
	Hispanic	Men	41	0.000924, 0.00109, 0.00129, 0.00153, 0.00181
	Hispanic	Men	42	0.000995, 0.00117, 0.00138, 0.00162, 0.00191
	Hispanic	Men	43	0.0011, 0.00128, 0.00149, 0.00173, 0.00201
	Hispanic	Men	44	0.00125, 0.00142, 0.00162, 0.00185, 0.00212
	Hispanic	Men	45	0.00142, 0.00159, 0.00179, 0.00201, 0.00226
	Hispanic	Men	46	0.00159, 0.00178, 0.00199, 0.00222, 0.00249

Variable	Race/ethnicity	Sex	Age	Distribution
	Hispanic	Men	47	0.00178, 0.00199, 0.00222, 0.00248, 0.00278
	Hispanic	Men	48	0.00198, 0.00222, 0.00249, 0.00279, 0.00313
	Hispanic	Men	49	0.00221, 0.00248, 0.00278, 0.00312, 0.00351
	Hispanic	Men	50	0.00248, 0.00278, 0.0031, 0.00347, 0.00389
	Hispanic	Men	51	0.00282, 0.00312, 0.00345, 0.00381, 0.00422
	Hispanic	Men	52	0.00323, 0.00351, 0.0038, 0.00413, 0.00448
	Hispanic	Men	53	0.00371, 0.00394, 0.00418, 0.00443, 0.00471
	Hispanic	Men	54	0.00414, 0.00435, 0.00457, 0.00479, 0.00503
	Hispanic	Men	55	0.00445, 0.0047, 0.00497, 0.00525, 0.00555
	Hispanic	Men	56	0.00472, 0.00504, 0.00538, 0.00575, 0.00614
	Hispanic	Men	57	0.00501, 0.0054, 0.00581, 0.00625, 0.00674
	Hispanic	Men	58	0.00531, 0.00576, 0.00624, 0.00676, 0.00733
	Hispanic	Men	59	0.0056, 0.00612, 0.00667, 0.00727, 0.00794
	Hispanic	Men	60	0.0059, 0.00647, 0.00709, 0.00777, 0.00852
	Hispanic	Men	61	0.00625, 0.00685, 0.00752, 0.00824, 0.00904
	Hispanic	Men	62	0.00664, 0.00726, 0.00794, 0.00868, 0.0095
	Hispanic	Men	63	0.00706, 0.0077, 0.00839, 0.00914, 0.00997
	Hispanic	Men	64	0.00748, 0.00815, 0.00888, 0.00967, 0.0105
	Hispanic	Men	65	0.0079, 0.00863, 0.00942, 0.0103, 0.0112
	Hispanic	Men	66	0.00837, 0.00918, 0.0101, 0.011, 0.0121
	Hispanic	Men	67	0.00891, 0.00981, 0.0108, 0.0119, 0.0131
	Hispanic	Men	68	0.00954, 0.0105, 0.0116, 0.0129, 0.0142
	Hispanic	Men	69	0.0103, 0.0114, 0.0126, 0.014, 0.0155
	Hispanic	Men	70	0.0111, 0.0124, 0.0137, 0.0152, 0.0169
	Hispanic	Men	71	0.0121, 0.0134, 0.0149, 0.0166, 0.0184
	Hispanic	Men	72	0.0131, 0.0146, 0.0163, 0.0181, 0.0202
	Hispanic	Men	73	0.0143, 0.0159, 0.0177, 0.0198, 0.022
	Hispanic	Men	74	0.0156, 0.0174, 0.0194, 0.0216, 0.024
	Hispanic	Men	75	0.0172, 0.0191, 0.0213, 0.0236, 0.0263
	Hispanic	Men	76	0.019, 0.0211, 0.0234, 0.026, 0.0288
	Hispanic	Men	77	0.0211, 0.0234, 0.0259, 0.0287, 0.0318
	Hispanic	Men	78	0.0235, 0.026, 0.0288, 0.0319, 0.0353
	Hispanic	Men	79	0.0262, 0.0291, 0.0322, 0.0356, 0.0394
	Hispanic	Men	80	0.0294, 0.0325, 0.036, 0.0398, 0.044
	Hispanic	Men	81	0.0328, 0.0363, 0.0402, 0.0444, 0.0491
	Hispanic	Men	82	0.0364, 0.0403, 0.0446, 0.0494, 0.0547
	Hispanic	Men	83	0.04, 0.0444, 0.0493, 0.0547, 0.0607
	Hispanic	Men	84	0.0433, 0.0483, 0.0538, 0.06, 0.0669
	Hispanic	Women	30	0.000325, 0.000367, 0.000414, 0.000468, 0.000528
	Hispanic	Women	31	0.000345, 0.000388, 0.000436, 0.000489, 0.00055
	Hispanic	Women	32	0.000364, 0.000408, 0.000457, 0.000512, 0.000573
	Hispanic	Women	33	0.00038, 0.000426, 0.000477, 0.000535, 0.0006
	Hispanic	Women	34	0.000389, 0.00044, 0.000497, 0.00056, 0.000633

Variable	Race/ethnicity	Sex	Age	Distribution
	Hispanic	Women	35	0.000395, 0.000452, 0.000515, 0.000588, 0.000672
	Hispanic	Women	36	0.000402, 0.000464, 0.000534, 0.000616, 0.000711
	Hispanic	Women	37	0.000412, 0.000479, 0.000555, 0.000644, 0.000749
	Hispanic	Women	38	0.000428, 0.000498, 0.00058, 0.000674, 0.000785
	Hispanic	Women	39	0.000451, 0.000524, 0.000608, 0.000706, 0.000821
	Hispanic	Women	40	0.000482, 0.000557, 0.000644, 0.000743, 0.00086
	Hispanic	Women	41	0.000521, 0.000598, 0.000686, 0.000787, 0.000905
	Hispanic	Women	42	0.00057, 0.000649, 0.000739, 0.00084, 0.000957
	Hispanic	Women	43	0.000632, 0.000713, 0.000802, 0.000903, 0.00102
	Hispanic	Women	44	0.000708, 0.000789, 0.000879, 0.000979, 0.00109
	Hispanic	Women	45	0.000792, 0.000877, 0.00097, 0.00107, 0.00119
	Hispanic	Women	46	0.000884, 0.000976, 0.00108, 0.00119, 0.00131
	Hispanic	Women	47	0.000984, 0.00109, 0.0012, 0.00132, 0.00146
	Hispanic	Women	48	0.00109, 0.00121, 0.00134, 0.00148, 0.00164
	Hispanic	Women	49	0.00121, 0.00135, 0.00149, 0.00165, 0.00184
	Hispanic	Women	50	0.00135, 0.0015, 0.00166, 0.00184, 0.00204
	Hispanic	Women	51	0.00152, 0.00167, 0.00184, 0.00203, 0.00224
	Hispanic	Women	52	0.00171, 0.00187, 0.00204, 0.00222, 0.00243
	Hispanic	Women	53	0.00191, 0.00207, 0.00224, 0.00243, 0.00263
	Hispanic	Women	54	0.00211, 0.00228, 0.00246, 0.00266, 0.00287
	Hispanic	Women	55	0.00229, 0.00248, 0.00269, 0.00291, 0.00315
	Hispanic	Women	56	0.00247, 0.00269, 0.00292, 0.00318, 0.00346
	Hispanic	Women	57	0.00266, 0.0029, 0.00316, 0.00345, 0.00376
	Hispanic	Women	58	0.00287, 0.00313, 0.00342, 0.00373, 0.00407
	Hispanic	Women	59	0.00307, 0.00336, 0.00367, 0.00402, 0.0044
	Hispanic	Women	60	0.00327, 0.00359, 0.00394, 0.00433, 0.00475
	Hispanic	Women	61	0.00349, 0.00384, 0.00422, 0.00464, 0.00511
	Hispanic	Women	62	0.00374, 0.00411, 0.00452, 0.00497, 0.00546
	Hispanic	Women	63	0.00401, 0.00441, 0.00484, 0.00532, 0.00585
	Hispanic	Women	64	0.00429, 0.00472, 0.0052, 0.00572, 0.0063
	Hispanic	Women	65	0.00459, 0.00507, 0.0056, 0.00617, 0.00682
	Hispanic	Women	66	0.00494, 0.00547, 0.00605, 0.00669, 0.00742
	Hispanic	Women	67	0.00534, 0.00593, 0.00657, 0.00728, 0.00808
	Hispanic	Women	68	0.00581, 0.00646, 0.00716, 0.00795, 0.00883
	Hispanic	Women	69	0.00636, 0.00706, 0.00784, 0.0087, 0.00966
	Hispanic	Women	70	0.00698, 0.00775, 0.0086, 0.00954, 0.0106
	Hispanic	Women	71	0.00767, 0.00852, 0.00944, 0.0105, 0.0116
	Hispanic	Women	72	0.00845, 0.00937, 0.0104, 0.0115, 0.0128
	Hispanic	Women	73	0.00933, 0.0103, 0.0115, 0.0127, 0.0141
	Hispanic	Women	74	0.0104, 0.0115, 0.0127, 0.014, 0.0155
	Hispanic	Women	75	0.0116, 0.0128, 0.014, 0.0155, 0.017
	Hispanic	Women	76	0.013, 0.0143, 0.0157, 0.0172, 0.0188
	Hispanic	Women	77	0.0147, 0.016, 0.0175, 0.0192, 0.021

Variable	Race/ethnicity	Sex	Age	Distribution
	Hispanic	Women	78	0.0166, 0.0181, 0.0197, 0.0215, 0.0235
	Hispanic	Women	79	0.0187, 0.0204, 0.0223, 0.0243, 0.0266
	Hispanic	Women	80	0.0211, 0.0231, 0.0252, 0.0276, 0.0302
	Hispanic	Women	81	0.0237, 0.026, 0.0285, 0.0312, 0.0343
	Hispanic	Women	82	0.0264, 0.0291, 0.0321, 0.0353, 0.0389
	Hispanic	Women	83	0.0292, 0.0324, 0.0358, 0.0397, 0.044
	Hispanic	Women	84	0.032, 0.0356, 0.0397, 0.0442, 0.0493
	White	Men	30	0.00104, 0.00128, 0.00158, 0.00196, 0.00242
	White	Men	31	0.00109, 0.00133, 0.00163, 0.00198, 0.00242
	White	Men	32	0.00114, 0.00138, 0.00166, 0.002, 0.00241
	White	Men	33	0.00118, 0.00141, 0.00168, 0.00201, 0.0024
	White	Men	34	0.00118, 0.00142, 0.00169, 0.00202, 0.00242
	White	Men	35	0.00118, 0.00142, 0.0017, 0.00204, 0.00245
	White	Men	36	0.00118, 0.00142, 0.00171, 0.00206, 0.00249
	White	Men	37	0.00118, 0.00143, 0.00173, 0.00209, 0.00253
	White	Men	38	0.0012, 0.00145, 0.00175, 0.00212, 0.00256
	White	Men	39	0.00124, 0.00149, 0.00179, 0.00216, 0.0026
	White	Men	40	0.00129, 0.00155, 0.00185, 0.00222, 0.00266
	White	Men	41	0.00136, 0.00163, 0.00194, 0.00231, 0.00275
	White	Men	42	0.00145, 0.00173, 0.00205, 0.00243, 0.00288
	White	Men	43	0.00158, 0.00186, 0.00219, 0.00258, 0.00303
	White	Men	44	0.00174, 0.00204, 0.00237, 0.00277, 0.00323
	White	Men	45	0.00193, 0.00224, 0.0026, 0.00302, 0.00352
	White	Men	46	0.00212, 0.00247, 0.00288, 0.00336, 0.00392
	White	Men	47	0.00233, 0.00273, 0.00321, 0.00376, 0.00441
	White	Men	48	0.00257, 0.00304, 0.00358, 0.00422, 0.00498
	White	Men	49	0.00286, 0.00338, 0.004, 0.00472, 0.00558
	White	Men	50	0.00321, 0.00378, 0.00445, 0.00524, 0.00618
	White	Men	51	0.00361, 0.00423, 0.00494, 0.00577, 0.00675
	White	Men	52	0.00408, 0.00472, 0.00545, 0.00629, 0.00728
	White	Men	53	0.00461, 0.00525, 0.00598, 0.00681, 0.00776
	White	Men	54	0.00514, 0.00579, 0.00652, 0.00735, 0.00829
	White	Men	55	0.00562, 0.00631, 0.00707, 0.00793, 0.00891
	White	Men	56	0.00602, 0.00678, 0.00762, 0.00857, 0.00964
	White	Men	57	0.00641, 0.00724, 0.00817, 0.00921, 0.0104
	White	Men	58	0.00681, 0.0077, 0.0087, 0.00983, 0.0111
	White	Men	59	0.00726, 0.00818, 0.00922, 0.0104, 0.0117
	White	Men	60	0.00774, 0.00869, 0.00973, 0.0109, 0.0122
	White	Men	61	0.00828, 0.00921, 0.0102, 0.0114, 0.0127
	White	Men	62	0.00885, 0.00976, 0.0108, 0.0119, 0.0131
	White	Men	63	0.00942, 0.0103, 0.0113, 0.0124, 0.0136
	White	Men	64	0.01, 0.0109, 0.0119, 0.013, 0.0143
	White	Men	65	0.0106, 0.0116, 0.0127, 0.0138, 0.0151

Variable	Race/ethnicity	Sex	Age	Distribution
	White	Men	66	0.0113, 0.0123, 0.0135, 0.0148, 0.0162
	White	Men	67	0.012, 0.0132, 0.0145, 0.016, 0.0176
	White	Men	68	0.0129, 0.0142, 0.0157, 0.0173, 0.0191
	White	Men	69	0.0139, 0.0154, 0.017, 0.0188, 0.0208
	White	Men	70	0.0151, 0.0168, 0.0186, 0.0205, 0.0227
	White	Men	71	0.0165, 0.0183, 0.0202, 0.0224, 0.0248
	White	Men	72	0.018, 0.02, 0.0221, 0.0244, 0.027
	White	Men	73	0.0197, 0.0218, 0.0241, 0.0266, 0.0294
	White	Men	74	0.0216, 0.0239, 0.0263, 0.029, 0.0319
	White	Men	75	0.0238, 0.0262, 0.0288, 0.0316, 0.0347
	White	Men	76	0.0263, 0.0289, 0.0316, 0.0346, 0.0379
	White	Men	77	0.0292, 0.0319, 0.0349, 0.0381, 0.0417
	White	Men	78	0.0324, 0.0354, 0.0387, 0.0423, 0.0463
	White	Men	79	0.0361, 0.0395, 0.0432, 0.0473, 0.0518
	White	Men	80	0.0403, 0.0442, 0.0484, 0.0531, 0.0582
	White	Men	81	0.045, 0.0494, 0.0543, 0.0596, 0.0655
	White	Men	82	0.0499, 0.055, 0.0606, 0.0667, 0.0735
	White	Men	83	0.0549, 0.0608, 0.0672, 0.0743, 0.0822
	White	Men	84	0.0598, 0.0665, 0.0738, 0.082, 0.0911
	White	Women	30	0.000551, 0.000638, 0.000737, 0.000852, 0.000987
	White	Women	31	0.00058, 0.000674, 0.000781, 0.000906, 0.00105
	White	Women	32	0.000612, 0.00071, 0.000823, 0.000954, 0.00111
	White	Women	33	0.000641, 0.000744, 0.000862, 0.000998, 0.00116
	White	Women	34	0.000658, 0.000769, 0.000897, 0.00105, 0.00122
	White	Women	35	0.000665, 0.000787, 0.00093, 0.0011, 0.0013
	White	Women	36	0.00067, 0.000804, 0.000964, 0.00115, 0.00139
	White	Women	37	0.00068, 0.000825, 0.000999, 0.00121, 0.00147
	White	Women	38	0.000694, 0.00085, 0.00104, 0.00127, 0.00156
	White	Women	39	0.000713, 0.000881, 0.00109, 0.00134, 0.00166
	White	Women	40	0.000741, 0.000922, 0.00114, 0.00142, 0.00176
	White	Women	41	0.000784, 0.000976, 0.00121, 0.00151, 0.00188
	White	Women	42	0.000843, 0.00105, 0.0013, 0.00161, 0.002
	White	Women	43	0.000916, 0.00114, 0.0014, 0.00174, 0.00215
	White	Women	44	0.001, 0.00124, 0.00153, 0.00189, 0.00234
	White	Women	45	0.0011, 0.00136, 0.00168, 0.00207, 0.00256
	White	Women	46	0.00121, 0.0015, 0.00185, 0.00229, 0.00283
	White	Women	47	0.00136, 0.00167, 0.00205, 0.00252, 0.0031
	White	Women	48	0.00154, 0.00187, 0.00228, 0.00276, 0.00336
	White	Women	49	0.00175, 0.0021, 0.00252, 0.00303, 0.00364
	White	Women	50	0.00196, 0.00234, 0.00279, 0.00333, 0.00398
	White	Women	51	0.00214, 0.00257, 0.00308, 0.00369, 0.00442
	White	Women	52	0.00231, 0.00279, 0.00337, 0.00407, 0.00493
	White	Women	53	0.00249, 0.00303, 0.00368, 0.00446, 0.00542

Variable	Race/ethnicity	Sex	Age	Distribution
	White	Women	54	0.00273, 0.0033, 0.00398, 0.0048, 0.00579
	White	Women	55	0.00306, 0.00362, 0.00427, 0.00505, 0.00598
	White	Women	56	0.00345, 0.00397, 0.00456, 0.00524, 0.00603
	White	Women	57	0.00389, 0.00434, 0.00484, 0.0054, 0.00604
	White	Women	58	0.00428, 0.00469, 0.00512, 0.0056, 0.00613
	White	Women	59	0.00459, 0.00498, 0.00541, 0.00587, 0.00638
	White	Women	60	0.00483, 0.00526, 0.00572, 0.00621, 0.00676
	White	Women	61	0.0051, 0.00556, 0.00606, 0.00659, 0.00718
	White	Women	62	0.00541, 0.00591, 0.00644, 0.00702, 0.00766
	White	Women	63	0.00578, 0.00631, 0.00689, 0.00751, 0.0082
	White	Women	64	0.00621, 0.00678, 0.00741, 0.00808, 0.00883
	White	Women	65	0.00669, 0.00732, 0.00801, 0.00876, 0.00959
	White	Women	66	0.00722, 0.00793, 0.00871, 0.00956, 0.0105
	White	Women	67	0.00779, 0.00861, 0.00951, 0.0105, 0.0116
	White	Women	68	0.00841, 0.00936, 0.0104, 0.0116, 0.0129
	White	Women	69	0.0091, 0.0102, 0.0114, 0.0128, 0.0143
	White	Women	70	0.00989, 0.0111, 0.0125, 0.0141, 0.0159
	White	Women	71	0.0108, 0.0122, 0.0138, 0.0156, 0.0176
	White	Women	72	0.0118, 0.0134, 0.0151, 0.0171, 0.0194
	White	Women	73	0.0129, 0.0147, 0.0166, 0.0188, 0.0213
	White	Women	74	0.0143, 0.0162, 0.0183, 0.0206, 0.0233
	White	Women	75	0.0159, 0.0179, 0.0201, 0.0226, 0.0255
	White	Women	76	0.0178, 0.02, 0.0223, 0.0249, 0.0279
	White	Women	77	0.0201, 0.0224, 0.0248, 0.0276, 0.0307
	White	Women	78	0.0227, 0.0251, 0.0278, 0.0307, 0.034
	White	Women	79	0.0256, 0.0283, 0.0312, 0.0345, 0.0381
	White	Women	80	0.0288, 0.0319, 0.0352, 0.0388, 0.0429
	White	Women	81	0.0324, 0.0358, 0.0396, 0.0437, 0.0483
	White	Women	82	0.0362, 0.0401, 0.0444, 0.0492, 0.0545
	White	Women	83	0.0401, 0.0446, 0.0495, 0.0551, 0.0613
	White	Women	84	0.044, 0.0492, 0.0548, 0.0612, 0.0683
Total mortality rates for 2030 (0.01, 0.2, 0.5, 0.8. 0.99 percentiles of the empirical distribution produced during forecasting)				
	Black	Men	30	0.00152, 0.00188, 0.00231, 0.00284, 0.0035
	Black	Men	31	0.00164, 0.00196, 0.00235, 0.00282, 0.00338
	Black	Men	32	0.00172, 0.00203, 0.00239, 0.00281, 0.00331
	Black	Men	33	0.00177, 0.00207, 0.00242, 0.00283, 0.00332
	Black	Men	34	0.00178, 0.00209, 0.00245, 0.00288, 0.00338
	Black	Men	35	0.0018, 0.00212, 0.00249, 0.00292, 0.00344
	Black	Men	36	0.00182, 0.00214, 0.00252, 0.00296, 0.00349
	Black	Men	37	0.00184, 0.00216, 0.00255, 0.00299, 0.00353
	Black	Men	38	0.00185, 0.00219, 0.00257, 0.00303, 0.00358
	Black	Men	39	0.00186, 0.00221, 0.00261, 0.00309, 0.00367
	Black	Men	40	0.00186, 0.00223, 0.00267, 0.00319, 0.00382

Variable	Race/ethnicity	Sex	Age	Distribution
	Black	Men	41	0.00188, 0.00228, 0.00276, 0.00334, 0.00405
	Black	Men	42	0.00191, 0.00235, 0.00289, 0.00355, 0.00436
	Black	Men	43	0.00198, 0.00246, 0.00307, 0.00382, 0.00476
	Black	Men	44	0.00206, 0.00261, 0.0033, 0.00416, 0.00527
	Black	Men	45	0.00218, 0.0028, 0.00359, 0.00459, 0.00589
	Black	Men	46	0.00235, 0.00304, 0.00394, 0.00509, 0.00659
	Black	Men	47	0.00258, 0.00335, 0.00435, 0.00564, 0.00734
	Black	Men	48	0.00288, 0.00374, 0.00483, 0.00625, 0.0081
	Black	Men	49	0.00327, 0.00421, 0.00539, 0.00691, 0.00889
	Black	Men	50	0.00374, 0.00475, 0.00602, 0.00764, 0.00971
	Black	Men	51	0.00428, 0.00537, 0.00673, 0.00842, 0.0106
	Black	Men	52	0.00492, 0.00608, 0.00749, 0.00924, 0.0114
	Black	Men	53	0.00567, 0.00688, 0.00832, 0.0101, 0.0122
	Black	Men	54	0.00653, 0.00776, 0.00921, 0.0109, 0.013
	Black	Men	55	0.00748, 0.00872, 0.0102, 0.0118, 0.0138
	Black	Men	56	0.00849, 0.00974, 0.0111, 0.0128, 0.0146
	Black	Men	57	0.00957, 0.0108, 0.0122, 0.0137, 0.0155
	Black	Men	58	0.0107, 0.0119, 0.0132, 0.0147, 0.0163
	Black	Men	59	0.0117, 0.0129, 0.0142, 0.0157, 0.0173
	Black	Men	60	0.0126, 0.0139, 0.0152, 0.0168, 0.0184
	Black	Men	61	0.0135, 0.0148, 0.0162, 0.0177, 0.0194
	Black	Men	62	0.0144, 0.0157, 0.0171, 0.0186, 0.0202
	Black	Men	63	0.0151, 0.0165, 0.0179, 0.0195, 0.0212
	Black	Men	64	0.0158, 0.0172, 0.0187, 0.0204, 0.0222
	Black	Men	65	0.0165, 0.018, 0.0196, 0.0214, 0.0234
	Black	Men	66	0.0172, 0.0188, 0.0206, 0.0225, 0.0246
	Black	Men	67	0.018, 0.0198, 0.0217, 0.0238, 0.0261
	Black	Men	68	0.0189, 0.0208, 0.023, 0.0253, 0.0279
	Black	Men	69	0.02, 0.0221, 0.0245, 0.027, 0.0299
	Black	Men	70	0.0212, 0.0236, 0.0261, 0.029, 0.0322
	Black	Men	71	0.0227, 0.0252, 0.028, 0.0311, 0.0346
	Black	Men	72	0.0243, 0.027, 0.03, 0.0333, 0.0371
	Black	Men	73	0.0261, 0.029, 0.0322, 0.0358, 0.0398
	Black	Men	74	0.0281, 0.0312, 0.0346, 0.0383, 0.0425
	Black	Men	75	0.0305, 0.0337, 0.0372, 0.041, 0.0453
	Black	Men	76	0.0331, 0.0365, 0.0401, 0.0441, 0.0485
	Black	Men	77	0.036, 0.0395, 0.0434, 0.0476, 0.0522
	Black	Men	78	0.0391, 0.0429, 0.047, 0.0516, 0.0567
	Black	Men	79	0.0423, 0.0465, 0.0512, 0.0563, 0.0619
	Black	Men	80	0.0457, 0.0505, 0.0557, 0.0615, 0.0679
	Black	Men	81	0.0493, 0.0547, 0.0606, 0.0671, 0.0744
	Black	Men	82	0.053, 0.0591, 0.0657, 0.0731, 0.0814
	Black	Men	83	0.0566, 0.0634, 0.0709, 0.0792, 0.0887

Variable	Race/ethnicity	Sex	Age	Distribution
	Black	Men	84	0.06, 0.0675, 0.0759, 0.0852, 0.0959
	Black	Women	30	0.000524, 0.00069, 0.000906, 0.00119, 0.00157
	Black	Women	31	0.000595, 0.000763, 0.000976, 0.00125, 0.0016
	Black	Women	32	0.000678, 0.000846, 0.00105, 0.00131, 0.00163
	Black	Women	33	0.00076, 0.000927, 0.00113, 0.00137, 0.00167
	Black	Women	34	0.000823, 0.000994, 0.0012, 0.00144, 0.00174
	Black	Women	35	0.000862, 0.00104, 0.00126, 0.00152, 0.00184
	Black	Women	36	0.000888, 0.00108, 0.00131, 0.0016, 0.00195
	Black	Women	37	0.000915, 0.00112, 0.00137, 0.00167, 0.00204
	Black	Women	38	0.00095, 0.00116, 0.00142, 0.00174, 0.00213
	Black	Women	39	0.000998, 0.00122, 0.00149, 0.00182, 0.00223
	Black	Women	40	0.00106, 0.0013, 0.00158, 0.00192, 0.00235
	Black	Women	41	0.00115, 0.00139, 0.00169, 0.00204, 0.00247
	Black	Women	42	0.00127, 0.00152, 0.00181, 0.00217, 0.00259
	Black	Women	43	0.00143, 0.00168, 0.00197, 0.00231, 0.00271
	Black	Women	44	0.00161, 0.00186, 0.00214, 0.00247, 0.00285
	Black	Women	45	0.00181, 0.00207, 0.00235, 0.00268, 0.00305
	Black	Women	46	0.002, 0.00228, 0.00259, 0.00295, 0.00336
	Black	Women	47	0.00219, 0.00251, 0.00288, 0.00329, 0.00377
	Black	Women	48	0.00241, 0.00278, 0.0032, 0.00369, 0.00426
	Black	Women	49	0.00267, 0.00309, 0.00357, 0.00412, 0.00477
	Black	Women	50	0.003, 0.00345, 0.00397, 0.00456, 0.00525
	Black	Women	51	0.00343, 0.00389, 0.0044, 0.00499, 0.00566
	Black	Women	52	0.00393, 0.00437, 0.00487, 0.00542, 0.00604
	Black	Women	53	0.00444, 0.00488, 0.00536, 0.00588, 0.00646
	Black	Women	54	0.00493, 0.00538, 0.00587, 0.00639, 0.00697
	Black	Women	55	0.0054, 0.00588, 0.00639, 0.00695, 0.00756
	Black	Women	56	0.00585, 0.00637, 0.00692, 0.00753, 0.0082
	Black	Women	57	0.00629, 0.00685, 0.00745, 0.00811, 0.00883
	Black	Women	58	0.0067, 0.00731, 0.00796, 0.00867, 0.00946
	Black	Women	59	0.00705, 0.00772, 0.00844, 0.00922, 0.0101
	Black	Women	60	0.00737, 0.00809, 0.00888, 0.00974, 0.0107
	Black	Women	61	0.00769, 0.00846, 0.00929, 0.0102, 0.0112
	Black	Women	62	0.00804, 0.00884, 0.00971, 0.0107, 0.0117
	Black	Women	63	0.0084, 0.00924, 0.0102, 0.0112, 0.0123
	Black	Women	64	0.00879, 0.00969, 0.0107, 0.0117, 0.0129
	Black	Women	65	0.00924, 0.0102, 0.0113, 0.0124, 0.0138
	Black	Women	66	0.00978, 0.0108, 0.012, 0.0133, 0.0147
	Black	Women	67	0.0104, 0.0116, 0.0129, 0.0143, 0.0158
	Black	Women	68	0.0112, 0.0125, 0.0138, 0.0154, 0.0171
	Black	Women	69	0.0121, 0.0134, 0.0149, 0.0166, 0.0185
	Black	Women	70	0.013, 0.0145, 0.0161, 0.0179, 0.02
	Black	Women	71	0.0141, 0.0157, 0.0174, 0.0194, 0.0215

Variable	Race/ethnicity	Sex	Age	Distribution
	Black	Women	72	0.0152, 0.0169, 0.0188, 0.0209, 0.0232
	Black	Women	73	0.0165, 0.0183, 0.0203, 0.0225, 0.025
	Black	Women	74	0.0179, 0.0199, 0.022, 0.0243, 0.0269
	Black	Women	75	0.0196, 0.0216, 0.0239, 0.0263, 0.0291
	Black	Women	76	0.0215, 0.0237, 0.026, 0.0286, 0.0315
	Black	Women	77	0.0237, 0.026, 0.0285, 0.0312, 0.0343
	Black	Women	78	0.0261, 0.0286, 0.0313, 0.0343, 0.0376
	Black	Women	79	0.0287, 0.0315, 0.0346, 0.0379, 0.0416
	Black	Women	80	0.0316, 0.0347, 0.0381, 0.0419, 0.046
	Black	Women	81	0.0346, 0.0382, 0.042, 0.0463, 0.0511
	Black	Women	82	0.0376, 0.0417, 0.0462, 0.0511, 0.0567
	Black	Women	83	0.0406, 0.0453, 0.0505, 0.0562, 0.0627
	Black	Women	84	0.0436, 0.0489, 0.0548, 0.0614, 0.0688
	Hispanic	Men	30	0.000785, 0.000893, 0.00102, 0.00115, 0.00131
	Hispanic	Men	31	0.000803, 0.000913, 0.00104, 0.00117, 0.00133
	Hispanic	Men	32	0.000808, 0.000923, 0.00105, 0.0012, 0.00137
	Hispanic	Men	33	0.000794, 0.00092, 0.00107, 0.00123, 0.00143
	Hispanic	Men	34	0.000773, 0.000912, 0.00107, 0.00127, 0.00149
	Hispanic	Men	35	0.000758, 0.000907, 0.00108, 0.00129, 0.00155
	Hispanic	Men	36	0.000756, 0.000909, 0.00109, 0.00131, 0.00158
	Hispanic	Men	37	0.000769, 0.000923, 0.00111, 0.00133, 0.00159
	Hispanic	Men	38	0.000793, 0.000947, 0.00113, 0.00135, 0.00161
	Hispanic	Men	39	0.000823, 0.00098, 0.00116, 0.00138, 0.00165
	Hispanic	Men	40	0.000858, 0.00102, 0.00121, 0.00144, 0.00171
	Hispanic	Men	41	0.000905, 0.00108, 0.00128, 0.00151, 0.0018
	Hispanic	Men	42	0.000974, 0.00115, 0.00136, 0.0016, 0.0019
	Hispanic	Men	43	0.00108, 0.00126, 0.00147, 0.00171, 0.00199
	Hispanic	Men	44	0.00122, 0.0014, 0.0016, 0.00183, 0.00209
	Hispanic	Men	45	0.00139, 0.00156, 0.00176, 0.00199, 0.00224
	Hispanic	Men	46	0.00156, 0.00175, 0.00196, 0.00219, 0.00246
	Hispanic	Men	47	0.00174, 0.00196, 0.00219, 0.00245, 0.00275
	Hispanic	Men	48	0.00195, 0.00219, 0.00246, 0.00276, 0.0031
	Hispanic	Men	49	0.00217, 0.00245, 0.00275, 0.00309, 0.00348
	Hispanic	Men	50	0.00244, 0.00274, 0.00307, 0.00344, 0.00386
	Hispanic	Men	51	0.00278, 0.00308, 0.00341, 0.00378, 0.00419
	Hispanic	Men	52	0.00319, 0.00347, 0.00377, 0.0041, 0.00446
	Hispanic	Men	53	0.00367, 0.0039, 0.00414, 0.0044, 0.00468
	Hispanic	Men	54	0.0041, 0.00431, 0.00453, 0.00476, 0.00501
	Hispanic	Men	55	0.00441, 0.00466, 0.00493, 0.00522, 0.00553
	Hispanic	Men	56	0.00467, 0.005, 0.00535, 0.00572, 0.00612
	Hispanic	Men	57	0.00496, 0.00535, 0.00577, 0.00622, 0.00671
	Hispanic	Men	58	0.00525, 0.0057, 0.00619, 0.00672, 0.0073
	Hispanic	Men	59	0.00553, 0.00605, 0.00661, 0.00722, 0.0079

Variable	Race/ethnicity	Sex	Age	Distribution
	Hispanic	Men	60	0.00582, 0.0064, 0.00703, 0.00771, 0.00848
	Hispanic	Men	61	0.00615, 0.00677, 0.00743, 0.00817, 0.00899
	Hispanic	Men	62	0.00653, 0.00716, 0.00785, 0.0086, 0.00943
	Hispanic	Men	63	0.00694, 0.00758, 0.00828, 0.00904, 0.00988
	Hispanic	Men	64	0.00734, 0.00802, 0.00875, 0.00955, 0.0104
	Hispanic	Men	65	0.00775, 0.00849, 0.00929, 0.0102, 0.0111
	Hispanic	Men	66	0.0082, 0.00902, 0.00991, 0.0109, 0.012
	Hispanic	Men	67	0.00873, 0.00964, 0.0106, 0.0117, 0.0129
	Hispanic	Men	68	0.00934, 0.0104, 0.0115, 0.0127, 0.0141
	Hispanic	Men	69	0.0101, 0.0112, 0.0124, 0.0138, 0.0153
	Hispanic	Men	70	0.0109, 0.0121, 0.0135, 0.015, 0.0167
	Hispanic	Men	71	0.0118, 0.0132, 0.0147, 0.0164, 0.0183
	Hispanic	Men	72	0.0129, 0.0143, 0.016, 0.0179, 0.0199
	Hispanic	Men	73	0.014, 0.0156, 0.0175, 0.0195, 0.0218
	Hispanic	Men	74	0.0153, 0.0171, 0.0191, 0.0213, 0.0238
	Hispanic	Men	75	0.0168, 0.0188, 0.0209, 0.0233, 0.026
	Hispanic	Men	76	0.0186, 0.0207, 0.023, 0.0256, 0.0285
	Hispanic	Men	77	0.0207, 0.023, 0.0255, 0.0283, 0.0315
	Hispanic	Men	78	0.023, 0.0256, 0.0284, 0.0315, 0.035
	Hispanic	Men	79	0.0257, 0.0286, 0.0317, 0.0351, 0.039
	Hispanic	Men	80	0.0288, 0.032, 0.0354, 0.0393, 0.0436
	Hispanic	Men	81	0.0322, 0.0357, 0.0396, 0.0438, 0.0486
	Hispanic	Men	82	0.0357, 0.0397, 0.044, 0.0488, 0.0542
	Hispanic	Men	83	0.0392, 0.0437, 0.0486, 0.054, 0.0602
	Hispanic	Men	84	0.0425, 0.0475, 0.0531, 0.0593, 0.0663
	Hispanic	Women	30	0.000324, 0.000367, 0.000414, 0.000467, 0.000528
	Hispanic	Women	31	0.000345, 0.000388, 0.000436, 0.000489, 0.00055
	Hispanic	Women	32	0.000364, 0.000408, 0.000457, 0.000511, 0.000573
	Hispanic	Women	33	0.000378, 0.000425, 0.000477, 0.000534, 0.0006
	Hispanic	Women	34	0.000387, 0.000438, 0.000495, 0.00056, 0.000634
	Hispanic	Women	35	0.000392, 0.000449, 0.000513, 0.000587, 0.000672
	Hispanic	Women	36	0.000397, 0.00046, 0.000531, 0.000614, 0.00071
	Hispanic	Women	37	0.000406, 0.000473, 0.000551, 0.000641, 0.000747
	Hispanic	Women	38	0.000421, 0.000492, 0.000574, 0.000669, 0.000781
	Hispanic	Women	39	0.000443, 0.000516, 0.000601, 0.0007, 0.000816
	Hispanic	Women	40	0.000472, 0.000548, 0.000635, 0.000735, 0.000853
	Hispanic	Women	41	0.00051, 0.000587, 0.000676, 0.000778, 0.000897
	Hispanic	Women	42	0.000557, 0.000637, 0.000727, 0.000829, 0.000947
	Hispanic	Women	43	0.000618, 0.000699, 0.000789, 0.00089, 0.00101
	Hispanic	Women	44	0.000692, 0.000774, 0.000864, 0.000964, 0.00108
	Hispanic	Women	45	0.000776, 0.00086, 0.000953, 0.00106, 0.00117
	Hispanic	Women	46	0.000866, 0.000958, 0.00106, 0.00117, 0.00129
	Hispanic	Women	47	0.000964, 0.00107, 0.00118, 0.00131, 0.00145

Variable	Race/ethnicity	Sex	Age	Distribution
	Hispanic	Women	48	0.00107, 0.00119, 0.00132, 0.00146, 0.00162
	Hispanic	Women	49	0.00119, 0.00133, 0.00147, 0.00164, 0.00182
	Hispanic	Women	50	0.00133, 0.00148, 0.00164, 0.00182, 0.00203
	Hispanic	Women	51	0.00149, 0.00165, 0.00182, 0.00201, 0.00223
	Hispanic	Women	52	0.00169, 0.00185, 0.00202, 0.00221, 0.00241
	Hispanic	Women	53	0.00189, 0.00205, 0.00222, 0.00241, 0.00261
	Hispanic	Women	54	0.00209, 0.00226, 0.00244, 0.00264, 0.00285
	Hispanic	Women	55	0.00227, 0.00246, 0.00267, 0.00289, 0.00313
	Hispanic	Women	56	0.00244, 0.00266, 0.0029, 0.00316, 0.00344
	Hispanic	Women	57	0.00263, 0.00288, 0.00314, 0.00343, 0.00374
	Hispanic	Women	58	0.00284, 0.0031, 0.00339, 0.0037, 0.00405
	Hispanic	Women	59	0.00303, 0.00333, 0.00364, 0.00399, 0.00438
	Hispanic	Women	60	0.00323, 0.00355, 0.00391, 0.0043, 0.00473
	Hispanic	Women	61	0.00344, 0.00379, 0.00418, 0.0046, 0.00507
	Hispanic	Women	62	0.00368, 0.00406, 0.00447, 0.00492, 0.00542
	Hispanic	Women	63	0.00394, 0.00434, 0.00478, 0.00526, 0.0058
	Hispanic	Women	64	0.00421, 0.00465, 0.00513, 0.00565, 0.00624
	Hispanic	Women	65	0.00451, 0.00499, 0.00552, 0.0061, 0.00675
	Hispanic	Women	66	0.00484, 0.00537, 0.00596, 0.00661, 0.00734
	Hispanic	Women	67	0.00523, 0.00582, 0.00647, 0.00719, 0.008
	Hispanic	Women	68	0.0057, 0.00634, 0.00705, 0.00785, 0.00874
	Hispanic	Women	69	0.00623, 0.00694, 0.00771, 0.00858, 0.00956
	Hispanic	Women	70	0.00683, 0.00761, 0.00846, 0.00941, 0.0105
	Hispanic	Women	71	0.00751, 0.00836, 0.00929, 0.0103, 0.0115
	Hispanic	Women	72	0.00826, 0.0092, 0.0102, 0.0114, 0.0127
	Hispanic	Women	73	0.00913, 0.0101, 0.0113, 0.0125, 0.0139
	Hispanic	Women	74	0.0101, 0.0112, 0.0125, 0.0138, 0.0153
	Hispanic	Women	75	0.0113, 0.0125, 0.0138, 0.0153, 0.0169
	Hispanic	Women	76	0.0127, 0.014, 0.0154, 0.0169, 0.0186
	Hispanic	Women	77	0.0144, 0.0157, 0.0173, 0.0189, 0.0207
	Hispanic	Women	78	0.0162, 0.0178, 0.0194, 0.0212, 0.0233
	Hispanic	Women	79	0.0183, 0.0201, 0.0219, 0.024, 0.0263
	Hispanic	Women	80	0.0207, 0.0227, 0.0248, 0.0272, 0.0298
	Hispanic	Women	81	0.0232, 0.0256, 0.0281, 0.0309, 0.0339
	Hispanic	Women	82	0.0259, 0.0286, 0.0316, 0.0349, 0.0386
	Hispanic	Women	83	0.0286, 0.0318, 0.0353, 0.0392, 0.0436
	Hispanic	Women	84	0.0314, 0.0351, 0.0391, 0.0437, 0.0489
	White	Men	30	0.00102, 0.00127, 0.00158, 0.00196, 0.00245
	White	Men	31	0.00108, 0.00132, 0.00162, 0.00199, 0.00244
	White	Men	32	0.00113, 0.00137, 0.00165, 0.002, 0.00242
	White	Men	33	0.00116, 0.0014, 0.00168, 0.00201, 0.00242
	White	Men	34	0.00117, 0.0014, 0.00169, 0.00202, 0.00243
	White	Men	35	0.00116, 0.0014, 0.00169, 0.00204, 0.00246

Variable	Race/ethnicity	Sex	Age	Distribution
	White	Men	36	0.00115, 0.0014, 0.0017, 0.00206, 0.0025
	White	Men	37	0.00116, 0.00141, 0.00171, 0.00208, 0.00253
	White	Men	38	0.00117, 0.00143, 0.00173, 0.00211, 0.00256
	White	Men	39	0.00121, 0.00146, 0.00177, 0.00214, 0.00259
	White	Men	40	0.00126, 0.00152, 0.00183, 0.0022, 0.00265
	White	Men	41	0.00133, 0.00159, 0.00191, 0.00228, 0.00274
	White	Men	42	0.00142, 0.00169, 0.00201, 0.0024, 0.00286
	White	Men	43	0.00154, 0.00182, 0.00215, 0.00254, 0.00301
	White	Men	44	0.0017, 0.00199, 0.00233, 0.00273, 0.00321
	White	Men	45	0.00188, 0.00219, 0.00256, 0.00298, 0.00349
	White	Men	46	0.00206, 0.00242, 0.00283, 0.00331, 0.00388
	White	Men	47	0.00227, 0.00268, 0.00316, 0.00372, 0.00438
	White	Men	48	0.00251, 0.00298, 0.00353, 0.00418, 0.00496
	White	Men	49	0.0028, 0.00332, 0.00394, 0.00468, 0.00556
	White	Men	50	0.00314, 0.00372, 0.0044, 0.0052, 0.00617
	White	Men	51	0.00354, 0.00416, 0.00488, 0.00573, 0.00674
	White	Men	52	0.00401, 0.00465, 0.0054, 0.00626, 0.00726
	White	Men	53	0.00453, 0.00518, 0.00592, 0.00677, 0.00775
	White	Men	54	0.00505, 0.00572, 0.00647, 0.00731, 0.00827
	White	Men	55	0.00553, 0.00623, 0.00701, 0.00789, 0.00889
	White	Men	56	0.00593, 0.0067, 0.00756, 0.00853, 0.00963
	White	Men	57	0.00631, 0.00715, 0.0081, 0.00917, 0.0104
	White	Men	58	0.0067, 0.00761, 0.00863, 0.00978, 0.0111
	White	Men	59	0.00714, 0.00808, 0.00914, 0.0103, 0.0117
	White	Men	60	0.00761, 0.00857, 0.00964, 0.0108, 0.0122
	White	Men	61	0.00813, 0.00908, 0.0101, 0.0113, 0.0126
	White	Men	62	0.00869, 0.00961, 0.0106, 0.0118, 0.013
	White	Men	63	0.00925, 0.0102, 0.0112, 0.0123, 0.0135
	White	Men	64	0.0098, 0.0107, 0.0118, 0.0129, 0.0141
	White	Men	65	0.0104, 0.0114, 0.0125, 0.0137, 0.015
	White	Men	66	0.011, 0.0121, 0.0133, 0.0146, 0.0161
	White	Men	67	0.0118, 0.013, 0.0143, 0.0158, 0.0174
	White	Men	68	0.0126, 0.014, 0.0154, 0.0171, 0.0189
	White	Men	69	0.0136, 0.0151, 0.0168, 0.0186, 0.0206
	White	Men	70	0.0148, 0.0165, 0.0183, 0.0203, 0.0225
	White	Men	71	0.0162, 0.0179, 0.0199, 0.0221, 0.0245
	White	Men	72	0.0176, 0.0196, 0.0217, 0.0241, 0.0267
	White	Men	73	0.0193, 0.0214, 0.0237, 0.0262, 0.0291
	White	Men	74	0.0212, 0.0234, 0.0258, 0.0286, 0.0316
	White	Men	75	0.0233, 0.0257, 0.0283, 0.0311, 0.0343
	White	Men	76	0.0258, 0.0283, 0.0311, 0.0341, 0.0375
	White	Men	77	0.0285, 0.0313, 0.0343, 0.0376, 0.0412
	White	Men	78	0.0317, 0.0348, 0.0381, 0.0418, 0.0458

Variable	Race/ethnicity	Sex	Age	Distribution
	White	Men	79	0.0353, 0.0388, 0.0426, 0.0467, 0.0513
	White	Men	80	0.0395, 0.0434, 0.0477, 0.0524, 0.0576
	White	Men	81	0.0441, 0.0485, 0.0534, 0.0588, 0.0648
	White	Men	82	0.0489, 0.0541, 0.0597, 0.0659, 0.0728
	White	Men	83	0.0538, 0.0597, 0.0662, 0.0734, 0.0815
	White	Men	84	0.0586, 0.0654, 0.0728, 0.081, 0.0903
	White	Women	30	0.000546, 0.000634, 0.000735, 0.000853, 0.00099
	White	Women	31	0.000575, 0.00067, 0.000779, 0.000907, 0.00106
	White	Women	32	0.000606, 0.000706, 0.000821, 0.000955, 0.00111
	White	Women	33	0.000634, 0.000739, 0.000859, 0.000998, 0.00116
	White	Women	34	0.00065, 0.000763, 0.000893, 0.00105, 0.00123
	White	Women	35	0.000655, 0.000779, 0.000925, 0.0011, 0.00131
	White	Women	36	0.000659, 0.000794, 0.000956, 0.00115, 0.00139
	White	Women	37	0.000666, 0.000813, 0.000989, 0.0012, 0.00147
	White	Women	38	0.000678, 0.000835, 0.00103, 0.00126, 0.00155
	White	Women	39	0.000695, 0.000863, 0.00107, 0.00133, 0.00165
	White	Women	40	0.000721, 0.000902, 0.00113, 0.0014, 0.00176
	White	Women	41	0.000762, 0.000954, 0.00119, 0.00149, 0.00187
	White	Women	42	0.000818, 0.00102, 0.00128, 0.00159, 0.00199
	White	Women	43	0.000889, 0.00111, 0.00138, 0.00171, 0.00214
	White	Women	44	0.000972, 0.00121, 0.0015, 0.00186, 0.00232
	White	Women	45	0.00107, 0.00133, 0.00165, 0.00205, 0.00255
	White	Women	46	0.00118, 0.00147, 0.00182, 0.00226, 0.00281
	White	Women	47	0.00132, 0.00163, 0.00202, 0.00249, 0.00308
	White	Women	48	0.0015, 0.00183, 0.00224, 0.00273, 0.00334
	White	Women	49	0.00171, 0.00206, 0.00249, 0.003, 0.00362
	White	Women	50	0.00191, 0.0023, 0.00275, 0.0033, 0.00397
	White	Women	51	0.00209, 0.00252, 0.00304, 0.00366, 0.00441
	White	Women	52	0.00226, 0.00275, 0.00334, 0.00405, 0.00493
	White	Women	53	0.00244, 0.00298, 0.00364, 0.00444, 0.00542
	White	Women	54	0.00268, 0.00325, 0.00394, 0.00477, 0.00579
	White	Women	55	0.003, 0.00357, 0.00424, 0.00503, 0.00598
	White	Women	56	0.0034, 0.00393, 0.00452, 0.00521, 0.00602
	White	Women	57	0.00383, 0.00429, 0.0048, 0.00538, 0.00602
	White	Women	58	0.00423, 0.00464, 0.00508, 0.00557, 0.00611
	White	Women	59	0.00453, 0.00493, 0.00536, 0.00584, 0.00636
	White	Women	60	0.00477, 0.0052, 0.00566, 0.00617, 0.00673
	White	Women	61	0.00503, 0.00549, 0.00599, 0.00654, 0.00714
	White	Women	62	0.00533, 0.00583, 0.00637, 0.00696, 0.00761
	White	Women	63	0.00569, 0.00622, 0.0068, 0.00743, 0.00813
	White	Women	64	0.0061, 0.00668, 0.0073, 0.00799, 0.00875
	White	Women	65	0.00656, 0.0072, 0.00789, 0.00865, 0.0095
	White	Women	66	0.00707, 0.00779, 0.00858, 0.00944, 0.0104

Variable	Race/ethnicity	Sex	Age	Distribution
	White	Women	67	0.00763, 0.00846, 0.00936, 0.0104, 0.0115
	White	Women	68	0.00823, 0.00919, 0.0103, 0.0114, 0.0128
	White	Women	69	0.0089, 0.01, 0.0112, 0.0126, 0.0142
	White	Women	70	0.00967, 0.0109, 0.0123, 0.0139, 0.0158
	White	Women	71	0.0105, 0.012, 0.0135, 0.0153, 0.0174
	White	Women	72	0.0115, 0.0131, 0.0149, 0.0169, 0.0192
	White	Women	73	0.0126, 0.0144, 0.0163, 0.0185, 0.021
	White	Women	74	0.014, 0.0158, 0.0179, 0.0203, 0.023
	White	Women	75	0.0156, 0.0176, 0.0198, 0.0223, 0.0252
	White	Women	76	0.0174, 0.0196, 0.0219, 0.0246, 0.0276
	White	Women	77	0.0196, 0.0219, 0.0244, 0.0272, 0.0303
	White	Women	78	0.0222, 0.0246, 0.0273, 0.0303, 0.0337
	White	Women	79	0.025, 0.0277, 0.0307, 0.034, 0.0377
	White	Women	80	0.0282, 0.0313, 0.0346, 0.0383, 0.0424
	White	Women	81	0.0317, 0.0352, 0.039, 0.0432, 0.0479
	White	Women	82	0.0354, 0.0394, 0.0437, 0.0486, 0.054
	White	Women	83	0.0392, 0.0438, 0.0488, 0.0544, 0.0607
	White	Women	84	0.0431, 0.0483, 0.054, 0.0605, 0.0677
Total mortality rates for 2031 (0.01, 0.2, 0.5, 0.8. 0.99 percentiles of the empirical distribution produced during forecasting)				
	Black	Men	30	0.00151, 0.00187, 0.00231, 0.00286, 0.00354
	Black	Men	31	0.00162, 0.00196, 0.00235, 0.00283, 0.00342
	Black	Men	32	0.00171, 0.00202, 0.00239, 0.00282, 0.00333
	Black	Men	33	0.00175, 0.00206, 0.00242, 0.00284, 0.00334
	Black	Men	34	0.00177, 0.00208, 0.00245, 0.00288, 0.0034
	Black	Men	35	0.00178, 0.0021, 0.00248, 0.00292, 0.00345
	Black	Men	36	0.00179, 0.00212, 0.0025, 0.00296, 0.0035
	Black	Men	37	0.00181, 0.00214, 0.00253, 0.00298, 0.00353
	Black	Men	38	0.00182, 0.00215, 0.00255, 0.00301, 0.00357
	Black	Men	39	0.00182, 0.00217, 0.00258, 0.00307, 0.00365
	Black	Men	40	0.00182, 0.00219, 0.00263, 0.00316, 0.0038
	Black	Men	41	0.00183, 0.00223, 0.00272, 0.00331, 0.00403
	Black	Men	42	0.00187, 0.00231, 0.00284, 0.00351, 0.00434
	Black	Men	43	0.00192, 0.00241, 0.00302, 0.00378, 0.00474
	Black	Men	44	0.00201, 0.00256, 0.00325, 0.00412, 0.00525
	Black	Men	45	0.00212, 0.00274, 0.00353, 0.00455, 0.00587
	Black	Men	46	0.00228, 0.00298, 0.00388, 0.00504, 0.00658
	Black	Men	47	0.00251, 0.00328, 0.00429, 0.0056, 0.00734
	Black	Men	48	0.00281, 0.00366, 0.00477, 0.00622, 0.00812
	Black	Men	49	0.00319, 0.00413, 0.00533, 0.00689, 0.00891
	Black	Men	50	0.00365, 0.00467, 0.00596, 0.00761, 0.00975
	Black	Men	51	0.00419, 0.00529, 0.00667, 0.0084, 0.0106
	Black	Men	52	0.00482, 0.00599, 0.00744, 0.00923, 0.0115
	Black	Men	53	0.00557, 0.00679, 0.00827, 0.0101, 0.0123

Variable	Race/ethnicity	Sex	Age	Distribution
	Black	Men	54	0.00642, 0.00767, 0.00915, 0.0109, 0.013
	Black	Men	55	0.00737, 0.00863, 0.0101, 0.0118, 0.0138
	Black	Men	56	0.00838, 0.00964, 0.0111, 0.0127, 0.0146
	Black	Men	57	0.00945, 0.0107, 0.0121, 0.0137, 0.0155
	Black	Men	58	0.0105, 0.0118, 0.0131, 0.0146, 0.0163
	Black	Men	59	0.0116, 0.0128, 0.0141, 0.0156, 0.0173
	Black	Men	60	0.0124, 0.0137, 0.0151, 0.0167, 0.0184
	Black	Men	61	0.0133, 0.0146, 0.016, 0.0176, 0.0193
	Black	Men	62	0.0142, 0.0155, 0.0169, 0.0184, 0.0201
	Black	Men	63	0.0149, 0.0162, 0.0177, 0.0193, 0.021
	Black	Men	64	0.0155, 0.0169, 0.0185, 0.0202, 0.022
	Black	Men	65	0.0161, 0.0177, 0.0193, 0.0211, 0.0231
	Black	Men	66	0.0168, 0.0185, 0.0203, 0.0222, 0.0244
	Black	Men	67	0.0176, 0.0194, 0.0214, 0.0235, 0.0259
	Black	Men	68	0.0185, 0.0205, 0.0226, 0.025, 0.0276
	Black	Men	69	0.0196, 0.0217, 0.0241, 0.0267, 0.0296
	Black	Men	70	0.0208, 0.0231, 0.0257, 0.0286, 0.0318
	Black	Men	71	0.0222, 0.0247, 0.0275, 0.0307, 0.0342
	Black	Men	72	0.0237, 0.0265, 0.0295, 0.0329, 0.0367
	Black	Men	73	0.0255, 0.0284, 0.0317, 0.0353, 0.0393
	Black	Men	74	0.0275, 0.0306, 0.034, 0.0378, 0.042
	Black	Men	75	0.0299, 0.0331, 0.0366, 0.0405, 0.0448
	Black	Men	76	0.0324, 0.0358, 0.0394, 0.0435, 0.048
	Black	Men	77	0.0353, 0.0388, 0.0427, 0.0469, 0.0516
	Black	Men	78	0.0382, 0.0421, 0.0463, 0.0509, 0.0561
	Black	Men	79	0.0414, 0.0457, 0.0504, 0.0556, 0.0613
	Black	Men	80	0.0448, 0.0496, 0.0549, 0.0607, 0.0673
	Black	Men	81	0.0484, 0.0538, 0.0597, 0.0663, 0.0738
	Black	Men	82	0.052, 0.0581, 0.0648, 0.0723, 0.0807
	Black	Men	83	0.0555, 0.0623, 0.0699, 0.0784, 0.088
	Black	Men	84	0.0588, 0.0664, 0.0748, 0.0843, 0.0952
	Black	Women	30	0.000515, 0.000684, 0.000906, 0.0012, 0.00159
	Black	Women	31	0.000586, 0.000758, 0.000977, 0.00126, 0.00163
	Black	Women	32	0.000669, 0.00084, 0.00105, 0.00132, 0.00165
	Black	Women	33	0.000751, 0.000921, 0.00113, 0.00138, 0.00169
	Black	Women	34	0.000812, 0.000987, 0.0012, 0.00145, 0.00176
	Black	Women	35	0.000849, 0.00103, 0.00126, 0.00153, 0.00186
	Black	Women	36	0.000872, 0.00107, 0.00131, 0.0016, 0.00196
	Black	Women	37	0.000897, 0.0011, 0.00136, 0.00167, 0.00205
	Black	Women	38	0.00093, 0.00115, 0.00141, 0.00174, 0.00214
	Black	Women	39	0.000974, 0.0012, 0.00148, 0.00181, 0.00224
	Black	Women	40	0.00104, 0.00127, 0.00156, 0.00191, 0.00235
	Black	Women	41	0.00112, 0.00137, 0.00166, 0.00202, 0.00246

Variable	Race/ethnicity	Sex	Age	Distribution
	Black	Women	42	0.00124, 0.00149, 0.00179, 0.00214, 0.00258
	Black	Women	43	0.00139, 0.00164, 0.00193, 0.00228, 0.00269
	Black	Women	44	0.00157, 0.00182, 0.00211, 0.00244, 0.00283
	Black	Women	45	0.00177, 0.00202, 0.00231, 0.00265, 0.00303
	Black	Women	46	0.00196, 0.00224, 0.00255, 0.00292, 0.00334
	Black	Women	47	0.00214, 0.00247, 0.00283, 0.00326, 0.00375
	Black	Women	48	0.00235, 0.00273, 0.00316, 0.00365, 0.00424
	Black	Women	49	0.00261, 0.00304, 0.00352, 0.00409, 0.00475
	Black	Women	50	0.00294, 0.0034, 0.00392, 0.00453, 0.00523
	Black	Women	51	0.00337, 0.00383, 0.00436, 0.00496, 0.00565
	Black	Women	52	0.00387, 0.00432, 0.00482, 0.00539, 0.00602
	Black	Women	53	0.00438, 0.00483, 0.00531, 0.00585, 0.00644
	Black	Women	54	0.00487, 0.00533, 0.00582, 0.00636, 0.00696
	Black	Women	55	0.00534, 0.00582, 0.00635, 0.00692, 0.00755
	Black	Women	56	0.00578, 0.00631, 0.00688, 0.0075, 0.00818
	Black	Women	57	0.00621, 0.00678, 0.0074, 0.00807, 0.00881
	Black	Women	58	0.00662, 0.00723, 0.0079, 0.00863, 0.00943
	Black	Women	59	0.00696, 0.00764, 0.00837, 0.00917, 0.0101
	Black	Women	60	0.00726, 0.008, 0.0088, 0.00968, 0.0107
	Black	Women	61	0.00757, 0.00835, 0.0092, 0.0101, 0.0112
	Black	Women	62	0.0079, 0.00871, 0.0096, 0.0106, 0.0117
	Black	Women	63	0.00825, 0.0091, 0.01, 0.0111, 0.0122
	Black	Women	64	0.00862, 0.00953, 0.0105, 0.0116, 0.0128
	Black	Women	65	0.00906, 0.01, 0.0111, 0.0123, 0.0136
	Black	Women	66	0.00959, 0.0107, 0.0118, 0.0131, 0.0146
	Black	Women	67	0.0102, 0.0114, 0.0127, 0.0141, 0.0157
	Black	Women	68	0.011, 0.0122, 0.0136, 0.0152, 0.0169
	Black	Women	69	0.0118, 0.0132, 0.0147, 0.0164, 0.0183
	Black	Women	70	0.0127, 0.0142, 0.0159, 0.0177, 0.0198
	Black	Women	71	0.0138, 0.0154, 0.0171, 0.0191, 0.0213
	Black	Women	72	0.0149, 0.0166, 0.0185, 0.0206, 0.023
	Black	Women	73	0.0161, 0.018, 0.02, 0.0222, 0.0247
	Black	Women	74	0.0175, 0.0195, 0.0216, 0.024, 0.0266
	Black	Women	75	0.0192, 0.0212, 0.0235, 0.026, 0.0287
	Black	Women	76	0.0211, 0.0232, 0.0256, 0.0282, 0.0311
	Black	Women	77	0.0232, 0.0255, 0.028, 0.0308, 0.0339
	Black	Women	78	0.0256, 0.0281, 0.0308, 0.0339, 0.0372
	Black	Women	79	0.0282, 0.031, 0.034, 0.0374, 0.0411
	Black	Women	80	0.031, 0.0341, 0.0376, 0.0414, 0.0456
	Black	Women	81	0.0339, 0.0375, 0.0414, 0.0457, 0.0506
	Black	Women	82	0.0369, 0.041, 0.0455, 0.0505, 0.0561
	Black	Women	83	0.0398, 0.0445, 0.0497, 0.0555, 0.0621
	Black	Women	84	0.0428, 0.0481, 0.054, 0.0607, 0.0682

Variable	Race/ethnicity	Sex	Age	Distribution
	Hispanic	Men	30	0.000784, 0.000893, 0.00102, 0.00116, 0.00132
	Hispanic	Men	31	0.000803, 0.000912, 0.00104, 0.00118, 0.00134
	Hispanic	Men	32	0.000807, 0.000923, 0.00105, 0.0012, 0.00137
	Hispanic	Men	33	0.000791, 0.000919, 0.00107, 0.00124, 0.00144
	Hispanic	Men	34	0.000768, 0.000909, 0.00107, 0.00127, 0.0015
	Hispanic	Men	35	0.000752, 0.000902, 0.00108, 0.00129, 0.00155
	Hispanic	Men	36	0.000748, 0.000903, 0.00109, 0.00131, 0.00158
	Hispanic	Men	37	0.000759, 0.000914, 0.0011, 0.00132, 0.00159
	Hispanic	Men	38	0.000781, 0.000936, 0.00112, 0.00134, 0.00161
	Hispanic	Men	39	0.000809, 0.000966, 0.00115, 0.00137, 0.00164
	Hispanic	Men	40	0.000842, 0.001, 0.0012, 0.00143, 0.0017
	Hispanic	Men	41	0.000886, 0.00106, 0.00126, 0.0015, 0.00179
	Hispanic	Men	42	0.000953, 0.00113, 0.00134, 0.00159, 0.00188
	Hispanic	Men	43	0.00106, 0.00124, 0.00144, 0.00169, 0.00197
	Hispanic	Men	44	0.0012, 0.00137, 0.00157, 0.0018, 0.00207
	Hispanic	Men	45	0.00136, 0.00154, 0.00174, 0.00196, 0.00222
	Hispanic	Men	46	0.00153, 0.00172, 0.00193, 0.00217, 0.00243
	Hispanic	Men	47	0.00171, 0.00192, 0.00216, 0.00242, 0.00272
	Hispanic	Men	48	0.00191, 0.00215, 0.00242, 0.00273, 0.00307
	Hispanic	Men	49	0.00214, 0.00241, 0.00272, 0.00306, 0.00346
	Hispanic	Men	50	0.0024, 0.0027, 0.00304, 0.00341, 0.00384
	Hispanic	Men	51	0.00274, 0.00304, 0.00338, 0.00375, 0.00417
	Hispanic	Men	52	0.00315, 0.00343, 0.00374, 0.00407, 0.00443
	Hispanic	Men	53	0.00363, 0.00386, 0.00411, 0.00437, 0.00465
	Hispanic	Men	54	0.00406, 0.00428, 0.0045, 0.00473, 0.00498
	Hispanic	Men	55	0.00436, 0.00462, 0.0049, 0.00519, 0.0055
	Hispanic	Men	56	0.00463, 0.00496, 0.00531, 0.00568, 0.00609
	Hispanic	Men	57	0.0049, 0.0053, 0.00573, 0.00619, 0.00669
	Hispanic	Men	58	0.00519, 0.00565, 0.00614, 0.00668, 0.00727
	Hispanic	Men	59	0.00546, 0.00599, 0.00656, 0.00718, 0.00786
	Hispanic	Men	60	0.00574, 0.00632, 0.00696, 0.00766, 0.00843
	Hispanic	Men	61	0.00606, 0.00668, 0.00736, 0.0081, 0.00893
	Hispanic	Men	62	0.00642, 0.00706, 0.00775, 0.00852, 0.00936
	Hispanic	Men	63	0.00682, 0.00747, 0.00817, 0.00894, 0.0098
	Hispanic	Men	64	0.00721, 0.00789, 0.00863, 0.00944, 0.0103
	Hispanic	Men	65	0.0076, 0.00834, 0.00915, 0.01, 0.011
	Hispanic	Men	66	0.00804, 0.00886, 0.00976, 0.0107, 0.0118
	Hispanic	Men	67	0.00855, 0.00946, 0.0105, 0.0116, 0.0128
	Hispanic	Men	68	0.00915, 0.0102, 0.0113, 0.0125, 0.0139
	Hispanic	Men	69	0.00986, 0.011, 0.0122, 0.0136, 0.0152
	Hispanic	Men	70	0.0107, 0.0119, 0.0133, 0.0148, 0.0165
	Hispanic	Men	71	0.0116, 0.0129, 0.0145, 0.0161, 0.0181
	Hispanic	Men	72	0.0126, 0.0141, 0.0158, 0.0176, 0.0197

Variable	Race/ethnicity	Sex	Age	Distribution
	Hispanic	Men	73	0.0137, 0.0154, 0.0172, 0.0192, 0.0215
	Hispanic	Men	74	0.015, 0.0168, 0.0188, 0.021, 0.0235
	Hispanic	Men	75	0.0165, 0.0184, 0.0206, 0.023, 0.0257
	Hispanic	Men	76	0.0182, 0.0204, 0.0227, 0.0253, 0.0282
	Hispanic	Men	77	0.0203, 0.0226, 0.0251, 0.028, 0.0311
	Hispanic	Men	78	0.0226, 0.0251, 0.0279, 0.0311, 0.0346
	Hispanic	Men	79	0.0252, 0.0281, 0.0312, 0.0347, 0.0386
	Hispanic	Men	80	0.0283, 0.0314, 0.0349, 0.0388, 0.0431
	Hispanic	Men	81	0.0316, 0.0351, 0.039, 0.0433, 0.0482
	Hispanic	Men	82	0.0351, 0.039, 0.0434, 0.0482, 0.0537
	Hispanic	Men	83	0.0385, 0.0429, 0.0479, 0.0534, 0.0596
	Hispanic	Men	84	0.0417, 0.0468, 0.0524, 0.0586, 0.0657
	Hispanic	Women	30	0.000324, 0.000366, 0.000414, 0.000467, 0.000528
	Hispanic	Women	31	0.000345, 0.000388, 0.000435, 0.000489, 0.00055
	Hispanic	Women	32	0.000363, 0.000407, 0.000456, 0.000511, 0.000573
	Hispanic	Women	33	0.000377, 0.000424, 0.000476, 0.000534, 0.0006
	Hispanic	Women	34	0.000385, 0.000436, 0.000494, 0.000559, 0.000634
	Hispanic	Women	35	0.000389, 0.000446, 0.000511, 0.000585, 0.000672
	Hispanic	Women	36	0.000393, 0.000456, 0.000528, 0.000612, 0.00071
	Hispanic	Women	37	0.000401, 0.000468, 0.000546, 0.000637, 0.000745
	Hispanic	Women	38	0.000414, 0.000485, 0.000568, 0.000664, 0.000778
	Hispanic	Women	39	0.000435, 0.000508, 0.000594, 0.000693, 0.000811
	Hispanic	Women	40	0.000462, 0.000538, 0.000626, 0.000727, 0.000847
	Hispanic	Women	41	0.000499, 0.000576, 0.000666, 0.000769, 0.000889
	Hispanic	Women	42	0.000545, 0.000625, 0.000715, 0.000818, 0.000938
	Hispanic	Women	43	0.000604, 0.000685, 0.000775, 0.000878, 0.000995
	Hispanic	Women	44	0.000677, 0.000759, 0.000849, 0.00095, 0.00106
	Hispanic	Women	45	0.000759, 0.000844, 0.000937, 0.00104, 0.00116
	Hispanic	Women	46	0.000849, 0.000941, 0.00104, 0.00115, 0.00128
	Hispanic	Women	47	0.000945, 0.00105, 0.00116, 0.00129, 0.00143
	Hispanic	Women	48	0.00105, 0.00117, 0.0013, 0.00144, 0.00161
	Hispanic	Women	49	0.00117, 0.00131, 0.00145, 0.00162, 0.00181
	Hispanic	Women	50	0.00131, 0.00146, 0.00162, 0.00181, 0.00201
	Hispanic	Women	51	0.00147, 0.00163, 0.00181, 0.002, 0.00221
	Hispanic	Women	52	0.00167, 0.00183, 0.002, 0.00219, 0.0024
	Hispanic	Women	53	0.00188, 0.00203, 0.00221, 0.00239, 0.00259
	Hispanic	Women	54	0.00207, 0.00224, 0.00242, 0.00262, 0.00283
	Hispanic	Women	55	0.00225, 0.00244, 0.00265, 0.00287, 0.00312
	Hispanic	Women	56	0.00242, 0.00264, 0.00288, 0.00314, 0.00342
	Hispanic	Women	57	0.00261, 0.00285, 0.00312, 0.00341, 0.00373
	Hispanic	Women	58	0.0028, 0.00307, 0.00336, 0.00368, 0.00403
	Hispanic	Women	59	0.00299, 0.00329, 0.00361, 0.00397, 0.00436
	Hispanic	Women	60	0.00318, 0.00351, 0.00387, 0.00427, 0.0047

Variable	Race/ethnicity	Sex	Age	Distribution
	Hispanic	Women	61	0.00339, 0.00375, 0.00414, 0.00456, 0.00504
	Hispanic	Women	62	0.00363, 0.004, 0.00442, 0.00487, 0.00538
	Hispanic	Women	63	0.00388, 0.00428, 0.00472, 0.00521, 0.00575
	Hispanic	Women	64	0.00414, 0.00458, 0.00506, 0.00558, 0.00617
	Hispanic	Women	65	0.00442, 0.00491, 0.00544, 0.00602, 0.00668
	Hispanic	Women	66	0.00475, 0.00528, 0.00587, 0.00652, 0.00726
	Hispanic	Women	67	0.00513, 0.00572, 0.00637, 0.0071, 0.00791
	Hispanic	Women	68	0.00558, 0.00623, 0.00694, 0.00774, 0.00864
	Hispanic	Women	69	0.0061, 0.00681, 0.00759, 0.00847, 0.00946
	Hispanic	Women	70	0.00669, 0.00747, 0.00833, 0.00928, 0.0104
	Hispanic	Women	71	0.00735, 0.0082, 0.00915, 0.0102, 0.0114
	Hispanic	Women	72	0.00809, 0.00903, 0.0101, 0.0112, 0.0125
	Hispanic	Women	73	0.00893, 0.00996, 0.0111, 0.0123, 0.0138
	Hispanic	Women	74	0.00992, 0.011, 0.0123, 0.0136, 0.0151
	Hispanic	Women	75	0.0111, 0.0123, 0.0136, 0.015, 0.0167
	Hispanic	Women	76	0.0125, 0.0138, 0.0151, 0.0167, 0.0184
	Hispanic	Women	77	0.0141, 0.0155, 0.017, 0.0186, 0.0205
	Hispanic	Women	78	0.0159, 0.0174, 0.0191, 0.021, 0.023
	Hispanic	Women	79	0.0179, 0.0197, 0.0216, 0.0237, 0.026
	Hispanic	Women	80	0.0203, 0.0223, 0.0245, 0.0269, 0.0295
	Hispanic	Women	81	0.0228, 0.0251, 0.0277, 0.0305, 0.0336
	Hispanic	Women	82	0.0254, 0.0281, 0.0311, 0.0345, 0.0382
	Hispanic	Women	83	0.0281, 0.0313, 0.0348, 0.0388, 0.0432
	Hispanic	Women	84	0.0308, 0.0345, 0.0386, 0.0432, 0.0484
	White	Men	30	0.00101, 0.00126, 0.00158, 0.00197, 0.00247
	White	Men	31	0.00106, 0.00131, 0.00162, 0.00199, 0.00246
	White	Men	32	0.00112, 0.00136, 0.00165, 0.00201, 0.00244
	White	Men	33	0.00115, 0.00138, 0.00167, 0.00201, 0.00243
	White	Men	34	0.00115, 0.00139, 0.00168, 0.00203, 0.00245
	White	Men	35	0.00114, 0.00139, 0.00168, 0.00204, 0.00248
	White	Men	36	0.00113, 0.00138, 0.00169, 0.00205, 0.00251
	White	Men	37	0.00113, 0.00139, 0.00169, 0.00207, 0.00254
	White	Men	38	0.00115, 0.0014, 0.00171, 0.00209, 0.00256
	White	Men	39	0.00118, 0.00144, 0.00175, 0.00212, 0.00259
	White	Men	40	0.00123, 0.00149, 0.0018, 0.00218, 0.00264
	White	Men	41	0.00129, 0.00156, 0.00187, 0.00226, 0.00272
	White	Men	42	0.00138, 0.00165, 0.00198, 0.00237, 0.00284
	White	Men	43	0.0015, 0.00178, 0.00212, 0.00251, 0.00298
	White	Men	44	0.00165, 0.00195, 0.00229, 0.0027, 0.00318
	White	Men	45	0.00183, 0.00215, 0.00251, 0.00295, 0.00346
	White	Men	46	0.00201, 0.00237, 0.00278, 0.00327, 0.00385
	White	Men	47	0.00221, 0.00262, 0.0031, 0.00367, 0.00435
	White	Men	48	0.00245, 0.00292, 0.00347, 0.00413, 0.00493

Variable	Race/ethnicity	Sex	Age	Distribution
	White	Men	49	0.00273, 0.00326, 0.00389, 0.00464, 0.00554
	White	Men	50	0.00307, 0.00365, 0.00434, 0.00516, 0.00615
	White	Men	51	0.00347, 0.0041, 0.00483, 0.00569, 0.00673
	White	Men	52	0.00393, 0.00459, 0.00534, 0.00622, 0.00725
	White	Men	53	0.00445, 0.00511, 0.00587, 0.00673, 0.00774
	White	Men	54	0.00497, 0.00565, 0.00641, 0.00727, 0.00826
	White	Men	55	0.00545, 0.00616, 0.00695, 0.00785, 0.00888
	White	Men	56	0.00584, 0.00662, 0.0075, 0.00849, 0.00962
	White	Men	57	0.00621, 0.00707, 0.00803, 0.00913, 0.0104
	White	Men	58	0.0066, 0.00752, 0.00855, 0.00973, 0.0111
	White	Men	59	0.00702, 0.00798, 0.00906, 0.0103, 0.0117
	White	Men	60	0.00748, 0.00845, 0.00954, 0.0108, 0.0122
	White	Men	61	0.00799, 0.00895, 0.01, 0.0112, 0.0125
	White	Men	62	0.00853, 0.00947, 0.0105, 0.0116, 0.0129
	White	Men	63	0.00908, 0.01, 0.011, 0.0121, 0.0134
	White	Men	64	0.00962, 0.0106, 0.0116, 0.0127, 0.014
	White	Men	65	0.0102, 0.0112, 0.0123, 0.0135, 0.0148
	White	Men	66	0.0108, 0.0119, 0.0131, 0.0144, 0.0159
	White	Men	67	0.0115, 0.0127, 0.0141, 0.0156, 0.0172
	White	Men	68	0.0124, 0.0137, 0.0152, 0.0169, 0.0187
	White	Men	69	0.0133, 0.0149, 0.0165, 0.0183, 0.0204
	White	Men	70	0.0145, 0.0162, 0.018, 0.02, 0.0223
	White	Men	71	0.0158, 0.0176, 0.0196, 0.0218, 0.0243
	White	Men	72	0.0173, 0.0192, 0.0214, 0.0238, 0.0264
	White	Men	73	0.0189, 0.021, 0.0233, 0.0259, 0.0288
	White	Men	74	0.0207, 0.0229, 0.0254, 0.0282, 0.0312
	White	Men	75	0.0228, 0.0252, 0.0278, 0.0307, 0.0339
	White	Men	76	0.0252, 0.0278, 0.0305, 0.0336, 0.037
	White	Men	77	0.0279, 0.0307, 0.0337, 0.0371, 0.0408
	White	Men	78	0.0311, 0.0341, 0.0375, 0.0412, 0.0453
	White	Men	79	0.0346, 0.0381, 0.0419, 0.0461, 0.0507
	White	Men	80	0.0387, 0.0426, 0.047, 0.0517, 0.057
	White	Men	81	0.0432, 0.0477, 0.0526, 0.0581, 0.0642
	White	Men	82	0.0479, 0.0531, 0.0588, 0.0651, 0.0721
	White	Men	83	0.0527, 0.0587, 0.0652, 0.0725, 0.0807
	White	Men	84	0.0575, 0.0642, 0.0717, 0.0801, 0.0896
	White	Women	30	0.000541, 0.000631, 0.000733, 0.000853, 0.000994
	White	Women	31	0.00057, 0.000666, 0.000778, 0.000907, 0.00106
	White	Women	32	0.000601, 0.000702, 0.000819, 0.000955, 0.00112
	White	Women	33	0.000628, 0.000734, 0.000856, 0.000998, 0.00117
	White	Women	34	0.000643, 0.000756, 0.000889, 0.00104, 0.00123
	White	Women	35	0.000646, 0.000771, 0.000919, 0.0011, 0.00131
	White	Women	36	0.000648, 0.000784, 0.000949, 0.00115, 0.00139

Variable	Race/ethnicity	Sex	Age	Distribution
	White	Women	37	0.000653, 0.000801, 0.000979, 0.0012, 0.00147
	White	Women	38	0.000663, 0.000821, 0.00101, 0.00125, 0.00155
	White	Women	39	0.000677, 0.000847, 0.00106, 0.00132, 0.00165
	White	Women	40	0.000702, 0.000883, 0.00111, 0.00139, 0.00175
	White	Women	41	0.00074, 0.000933, 0.00117, 0.00147, 0.00186
	White	Women	42	0.000794, 0.000999, 0.00125, 0.00157, 0.00198
	White	Women	43	0.000862, 0.00108, 0.00135, 0.00169, 0.00212
	White	Women	44	0.000943, 0.00118, 0.00147, 0.00184, 0.0023
	White	Women	45	0.00104, 0.0013, 0.00162, 0.00202, 0.00253
	White	Women	46	0.00115, 0.00143, 0.00179, 0.00223, 0.00279
	White	Women	47	0.00129, 0.0016, 0.00198, 0.00246, 0.00306
	White	Women	48	0.00146, 0.0018, 0.0022, 0.0027, 0.00332
	White	Women	49	0.00167, 0.00202, 0.00245, 0.00297, 0.0036
	White	Women	50	0.00187, 0.00226, 0.00272, 0.00327, 0.00395
	White	Women	51	0.00205, 0.00248, 0.003, 0.00363, 0.0044
	White	Women	52	0.00221, 0.0027, 0.0033, 0.00402, 0.00492
	White	Women	53	0.00239, 0.00294, 0.0036, 0.00441, 0.00542
	White	Women	54	0.00263, 0.00321, 0.0039, 0.00475, 0.00579
	White	Women	55	0.00295, 0.00352, 0.0042, 0.00501, 0.00598
	White	Women	56	0.00335, 0.00388, 0.00449, 0.00519, 0.00601
	White	Women	57	0.00378, 0.00425, 0.00477, 0.00535, 0.006
	White	Women	58	0.00417, 0.00459, 0.00504, 0.00554, 0.00609
	White	Women	59	0.00447, 0.00488, 0.00532, 0.0058, 0.00633
	White	Women	60	0.0047, 0.00514, 0.00561, 0.00613, 0.0067
	White	Women	61	0.00495, 0.00542, 0.00593, 0.00649, 0.0071
	White	Women	62	0.00524, 0.00575, 0.00629, 0.00689, 0.00755
	White	Women	63	0.00559, 0.00613, 0.00671, 0.00735, 0.00806
	White	Women	64	0.00599, 0.00657, 0.0072, 0.0079, 0.00866
	White	Women	65	0.00644, 0.00708, 0.00778, 0.00855, 0.0094
	White	Women	66	0.00694, 0.00766, 0.00845, 0.00932, 0.0103
	White	Women	67	0.00747, 0.0083, 0.00922, 0.0102, 0.0114
	White	Women	68	0.00806, 0.00902, 0.0101, 0.0113, 0.0126
	White	Women	69	0.00871, 0.00982, 0.0111, 0.0125, 0.0141
	White	Women	70	0.00945, 0.0107, 0.0121, 0.0137, 0.0156
	White	Women	71	0.0103, 0.0117, 0.0133, 0.0151, 0.0172
	White	Women	72	0.0113, 0.0128, 0.0146, 0.0166, 0.019
	White	Women	73	0.0124, 0.0141, 0.016, 0.0183, 0.0208
	White	Women	74	0.0137, 0.0155, 0.0176, 0.02, 0.0228
	White	Women	75	0.0152, 0.0172, 0.0195, 0.022, 0.0249
	White	Women	76	0.017, 0.0192, 0.0216, 0.0242, 0.0273
	White	Women	77	0.0192, 0.0215, 0.024, 0.0268, 0.03
	White	Women	78	0.0217, 0.0242, 0.0269, 0.0299, 0.0333
	White	Women	79	0.0245, 0.0272, 0.0302, 0.0335, 0.0373

Variable	Race/ethnicity	Sex	Age	Distribution
	White	Women	80	0.0276, 0.0307, 0.0341, 0.0378, 0.042
	White	Women	81	0.031, 0.0345, 0.0384, 0.0426, 0.0474
	White	Women	82	0.0347, 0.0387, 0.0431, 0.048, 0.0535
	White	Women	83	0.0384, 0.043, 0.0481, 0.0537, 0.0602
	White	Women	84	0.0422, 0.0474, 0.0532, 0.0598, 0.0671
Total mortality rates for 2032 (0.01, 0.2, 0.5, 0.8. 0.99 percentiles of the empirical distribution produced during forecasting)				
	Black	Men	30	0.00149, 0.00186, 0.00231, 0.00287, 0.00358
	Black	Men	31	0.00161, 0.00195, 0.00236, 0.00285, 0.00345
	Black	Men	32	0.0017, 0.00202, 0.00239, 0.00283, 0.00336
	Black	Men	33	0.00174, 0.00205, 0.00242, 0.00285, 0.00336
	Black	Men	34	0.00175, 0.00207, 0.00245, 0.00289, 0.00342
	Black	Men	35	0.00176, 0.00209, 0.00247, 0.00292, 0.00347
	Black	Men	36	0.00177, 0.0021, 0.00249, 0.00295, 0.0035
	Black	Men	37	0.00178, 0.00211, 0.00251, 0.00297, 0.00353
	Black	Men	38	0.00179, 0.00213, 0.00252, 0.003, 0.00356
	Black	Men	39	0.00179, 0.00214, 0.00255, 0.00304, 0.00364
	Black	Men	40	0.00178, 0.00215, 0.0026, 0.00313, 0.00379
	Black	Men	41	0.00179, 0.00219, 0.00268, 0.00327, 0.00401
	Black	Men	42	0.00182, 0.00226, 0.0028, 0.00347, 0.00431
	Black	Men	43	0.00187, 0.00236, 0.00297, 0.00374, 0.00471
	Black	Men	44	0.00195, 0.0025, 0.00319, 0.00408, 0.00522
	Black	Men	45	0.00206, 0.00268, 0.00348, 0.0045, 0.00585
	Black	Men	46	0.00222, 0.00292, 0.00382, 0.005, 0.00657
	Black	Men	47	0.00244, 0.00322, 0.00423, 0.00556, 0.00734
	Black	Men	48	0.00273, 0.00359, 0.00471, 0.00618, 0.00813
	Black	Men	49	0.00311, 0.00405, 0.00527, 0.00686, 0.00894
	Black	Men	50	0.00357, 0.00459, 0.00591, 0.00759, 0.00978
	Black	Men	51	0.0041, 0.00521, 0.00661, 0.00839, 0.0107
	Black	Men	52	0.00472, 0.00591, 0.00738, 0.00921, 0.0115
	Black	Men	53	0.00547, 0.00671, 0.00821, 0.0101, 0.0123
	Black	Men	54	0.00632, 0.00759, 0.0091, 0.0109, 0.0131
	Black	Men	55	0.00726, 0.00854, 0.01, 0.0118, 0.0139
	Black	Men	56	0.00827, 0.00955, 0.011, 0.0127, 0.0147
	Black	Men	57	0.00933, 0.0106, 0.012, 0.0136, 0.0155
	Black	Men	58	0.0104, 0.0117, 0.013, 0.0146, 0.0163
	Black	Men	59	0.0114, 0.0127, 0.014, 0.0156, 0.0173
	Black	Men	60	0.0123, 0.0136, 0.015, 0.0165, 0.0183
	Black	Men	61	0.0131, 0.0144, 0.0159, 0.0174, 0.0192
	Black	Men	62	0.0139, 0.0153, 0.0167, 0.0182, 0.02
	Black	Men	63	0.0146, 0.016, 0.0175, 0.0191, 0.0209
	Black	Men	64	0.0152, 0.0167, 0.0182, 0.0199, 0.0218
	Black	Men	65	0.0158, 0.0174, 0.019, 0.0209, 0.0229
	Black	Men	66	0.0165, 0.0182, 0.02, 0.0219, 0.0241

Variable	Race/ethnicity	Sex	Age	Distribution
	Black	Men	67	0.0173, 0.0191, 0.021, 0.0232, 0.0256
	Black	Men	68	0.0181, 0.0201, 0.0223, 0.0247, 0.0273
	Black	Men	69	0.0192, 0.0213, 0.0237, 0.0263, 0.0293
	Black	Men	70	0.0203, 0.0227, 0.0253, 0.0282, 0.0315
	Black	Men	71	0.0217, 0.0243, 0.0271, 0.0303, 0.0338
	Black	Men	72	0.0232, 0.026, 0.029, 0.0324, 0.0363
	Black	Men	73	0.025, 0.0279, 0.0312, 0.0348, 0.0389
	Black	Men	74	0.0269, 0.03, 0.0334, 0.0372, 0.0415
	Black	Men	75	0.0292, 0.0324, 0.036, 0.0399, 0.0443
	Black	Men	76	0.0318, 0.0351, 0.0388, 0.0429, 0.0474
	Black	Men	77	0.0345, 0.0381, 0.042, 0.0463, 0.0511
	Black	Men	78	0.0375, 0.0413, 0.0456, 0.0503, 0.0555
	Black	Men	79	0.0406, 0.0449, 0.0496, 0.0548, 0.0607
	Black	Men	80	0.0439, 0.0487, 0.0541, 0.06, 0.0666
	Black	Men	81	0.0474, 0.0529, 0.0589, 0.0656, 0.0731
	Black	Men	82	0.051, 0.0571, 0.0639, 0.0714, 0.08
	Black	Men	83	0.0544, 0.0613, 0.0689, 0.0775, 0.0873
	Black	Men	84	0.0577, 0.0653, 0.0738, 0.0834, 0.0944
	Black	Women	30	0.000506, 0.000678, 0.000906, 0.00121, 0.00162
	Black	Women	31	0.000578, 0.000752, 0.000977, 0.00127, 0.00165
	Black	Women	32	0.000661, 0.000835, 0.00105, 0.00133, 0.00168
	Black	Women	33	0.000742, 0.000915, 0.00113, 0.00139, 0.00171
	Black	Women	34	0.000802, 0.00098, 0.00119, 0.00146, 0.00178
	Black	Women	35	0.000837, 0.00102, 0.00125, 0.00153, 0.00187
	Black	Women	36	0.000858, 0.00106, 0.0013, 0.0016, 0.00197
	Black	Women	37	0.00088, 0.00109, 0.00135, 0.00166, 0.00206
	Black	Women	38	0.00091, 0.00113, 0.0014, 0.00173, 0.00215
	Black	Women	39	0.000952, 0.00118, 0.00146, 0.0018, 0.00224
	Black	Women	40	0.00101, 0.00125, 0.00154, 0.0019, 0.00234
	Black	Women	41	0.00109, 0.00134, 0.00164, 0.002, 0.00245
	Black	Women	42	0.00121, 0.00146, 0.00176, 0.00212, 0.00257
	Black	Women	43	0.00135, 0.00161, 0.0019, 0.00225, 0.00267
	Black	Women	44	0.00154, 0.00179, 0.00207, 0.00241, 0.0028
	Black	Women	45	0.00173, 0.00198, 0.00228, 0.00261, 0.003
	Black	Women	46	0.00191, 0.00219, 0.00251, 0.00288, 0.00331
	Black	Women	47	0.0021, 0.00242, 0.00279, 0.00322, 0.00372
	Black	Women	48	0.0023, 0.00268, 0.00312, 0.00362, 0.00421
	Black	Women	49	0.00256, 0.00299, 0.00348, 0.00405, 0.00473
	Black	Women	50	0.00289, 0.00335, 0.00388, 0.0045, 0.00522
	Black	Women	51	0.00331, 0.00378, 0.00432, 0.00493, 0.00563
	Black	Women	52	0.00381, 0.00427, 0.00478, 0.00536, 0.00601
	Black	Women	53	0.00432, 0.00477, 0.00527, 0.00582, 0.00643
	Black	Women	54	0.00481, 0.00527, 0.00578, 0.00633, 0.00694

Variable	Race/ethnicity	Sex	Age	Distribution
	Black	Women	55	0.00527, 0.00577, 0.0063, 0.00688, 0.00753
	Black	Women	56	0.00572, 0.00625, 0.00683, 0.00746, 0.00816
	Black	Women	57	0.00614, 0.00672, 0.00735, 0.00803, 0.00879
	Black	Women	58	0.00654, 0.00716, 0.00784, 0.00858, 0.0094
	Black	Women	59	0.00687, 0.00755, 0.0083, 0.00912, 0.01
	Black	Women	60	0.00716, 0.0079, 0.00872, 0.00961, 0.0106
	Black	Women	61	0.00745, 0.00824, 0.00911, 0.0101, 0.0111
	Black	Women	62	0.00777, 0.00859, 0.00949, 0.0105, 0.0116
	Black	Women	63	0.00811, 0.00896, 0.0099, 0.0109, 0.0121
	Black	Women	64	0.00846, 0.00938, 0.0104, 0.0115, 0.0127
	Black	Women	65	0.00888, 0.00987, 0.011, 0.0122, 0.0135
	Black	Women	66	0.00939, 0.0105, 0.0116, 0.013, 0.0144
	Black	Women	67	0.01, 0.0112, 0.0125, 0.0139, 0.0155
	Black	Women	68	0.0107, 0.012, 0.0134, 0.015, 0.0167
	Black	Women	69	0.0116, 0.0129, 0.0145, 0.0162, 0.0181
	Black	Women	70	0.0125, 0.014, 0.0156, 0.0175, 0.0196
	Black	Women	71	0.0135, 0.0151, 0.0169, 0.0188, 0.0211
	Black	Women	72	0.0146, 0.0163, 0.0182, 0.0203, 0.0227
	Black	Women	73	0.0158, 0.0176, 0.0196, 0.0219, 0.0245
	Black	Women	74	0.0172, 0.0191, 0.0213, 0.0237, 0.0263
	Black	Women	75	0.0188, 0.0208, 0.0231, 0.0256, 0.0284
	Black	Women	76	0.0206, 0.0228, 0.0252, 0.0278, 0.0307
	Black	Women	77	0.0227, 0.025, 0.0276, 0.0304, 0.0335
	Black	Women	78	0.025, 0.0276, 0.0304, 0.0334, 0.0368
	Black	Women	79	0.0276, 0.0304, 0.0335, 0.0369, 0.0407
	Black	Women	80	0.0304, 0.0335, 0.037, 0.0408, 0.0451
	Black	Women	81	0.0333, 0.0369, 0.0408, 0.0452, 0.0501
	Black	Women	82	0.0362, 0.0403, 0.0448, 0.0499, 0.0556
	Black	Women	83	0.0391, 0.0438, 0.049, 0.0549, 0.0615
	Black	Women	84	0.042, 0.0473, 0.0533, 0.06, 0.0676
	Hispanic	Men	30	0.000783, 0.000892, 0.00102, 0.00116, 0.00132
	Hispanic	Men	31	0.000802, 0.000912, 0.00104, 0.00118, 0.00134
	Hispanic	Men	32	0.000806, 0.000922, 0.00105, 0.0012, 0.00138
	Hispanic	Men	33	0.000789, 0.000917, 0.00107, 0.00124, 0.00144
	Hispanic	Men	34	0.000764, 0.000906, 0.00107, 0.00127, 0.0015
	Hispanic	Men	35	0.000746, 0.000897, 0.00108, 0.00129, 0.00155
	Hispanic	Men	36	0.00074, 0.000896, 0.00108, 0.00131, 0.00158
	Hispanic	Men	37	0.000749, 0.000905, 0.00109, 0.00132, 0.00159
	Hispanic	Men	38	0.000769, 0.000925, 0.00111, 0.00133, 0.0016
	Hispanic	Men	39	0.000795, 0.000953, 0.00114, 0.00136, 0.00163
	Hispanic	Men	40	0.000826, 0.000989, 0.00118, 0.00141, 0.00169
	Hispanic	Men	41	0.000869, 0.00104, 0.00124, 0.00148, 0.00177
	Hispanic	Men	42	0.000933, 0.00111, 0.00132, 0.00157, 0.00187

Variable	Race/ethnicity	Sex	Age	Distribution
	Hispanic	Men	43	0.00103, 0.00121, 0.00142, 0.00167, 0.00195
	Hispanic	Men	44	0.00117, 0.00135, 0.00155, 0.00178, 0.00205
	Hispanic	Men	45	0.00133, 0.00151, 0.00171, 0.00193, 0.00219
	Hispanic	Men	46	0.0015, 0.00169, 0.0019, 0.00214, 0.00241
	Hispanic	Men	47	0.00168, 0.00189, 0.00213, 0.00239, 0.0027
	Hispanic	Men	48	0.00188, 0.00212, 0.00239, 0.0027, 0.00304
	Hispanic	Men	49	0.0021, 0.00237, 0.00268, 0.00303, 0.00343
	Hispanic	Men	50	0.00237, 0.00267, 0.003, 0.00338, 0.00381
	Hispanic	Men	51	0.0027, 0.00301, 0.00334, 0.00372, 0.00415
	Hispanic	Men	52	0.00311, 0.0034, 0.0037, 0.00404, 0.00441
	Hispanic	Men	53	0.00359, 0.00383, 0.00408, 0.00434, 0.00463
	Hispanic	Men	54	0.00402, 0.00424, 0.00446, 0.0047, 0.00496
	Hispanic	Men	55	0.00432, 0.00458, 0.00486, 0.00516, 0.00547
	Hispanic	Men	56	0.00458, 0.00492, 0.00527, 0.00565, 0.00607
	Hispanic	Men	57	0.00485, 0.00525, 0.00568, 0.00615, 0.00666
	Hispanic	Men	58	0.00513, 0.0056, 0.0061, 0.00664, 0.00724
	Hispanic	Men	59	0.0054, 0.00593, 0.0065, 0.00713, 0.00783
	Hispanic	Men	60	0.00566, 0.00625, 0.00689, 0.0076, 0.00839
	Hispanic	Men	61	0.00597, 0.00659, 0.00728, 0.00803, 0.00887
	Hispanic	Men	62	0.00632, 0.00696, 0.00766, 0.00843, 0.00929
	Hispanic	Men	63	0.0067, 0.00735, 0.00806, 0.00884, 0.00971
	Hispanic	Men	64	0.00708, 0.00776, 0.00851, 0.00932, 0.0102
	Hispanic	Men	65	0.00746, 0.0082, 0.00901, 0.00991, 0.0109
	Hispanic	Men	66	0.00788, 0.00871, 0.00961, 0.0106, 0.0117
	Hispanic	Men	67	0.00838, 0.0093, 0.0103, 0.0114, 0.0127
	Hispanic	Men	68	0.00897, 0.00999, 0.0111, 0.0124, 0.0138
	Hispanic	Men	69	0.00966, 0.0108, 0.012, 0.0134, 0.015
	Hispanic	Men	70	0.0104, 0.0117, 0.0131, 0.0146, 0.0164
	Hispanic	Men	71	0.0113, 0.0127, 0.0142, 0.0159, 0.0179
	Hispanic	Men	72	0.0123, 0.0138, 0.0155, 0.0174, 0.0195
	Hispanic	Men	73	0.0134, 0.0151, 0.0169, 0.019, 0.0213
	Hispanic	Men	74	0.0147, 0.0165, 0.0185, 0.0207, 0.0233
	Hispanic	Men	75	0.0162, 0.0181, 0.0203, 0.0227, 0.0254
	Hispanic	Men	76	0.0179, 0.02, 0.0223, 0.0249, 0.0279
	Hispanic	Men	77	0.0199, 0.0222, 0.0247, 0.0276, 0.0308
	Hispanic	Men	78	0.0221, 0.0247, 0.0275, 0.0307, 0.0342
	Hispanic	Men	79	0.0247, 0.0276, 0.0308, 0.0343, 0.0382
	Hispanic	Men	80	0.0277, 0.0309, 0.0344, 0.0383, 0.0427
	Hispanic	Men	81	0.031, 0.0345, 0.0385, 0.0428, 0.0477
	Hispanic	Men	82	0.0344, 0.0384, 0.0428, 0.0477, 0.0532
	Hispanic	Men	83	0.0377, 0.0422, 0.0472, 0.0528, 0.0591
	Hispanic	Men	84	0.0409, 0.046, 0.0516, 0.0579, 0.0651
	Hispanic	Women	30	0.000323, 0.000366, 0.000413, 0.000467, 0.000528

Variable	Race/ethnicity	Sex	Age	Distribution
	Hispanic	Women	31	0.000344, 0.000387, 0.000435, 0.000488, 0.000549
	Hispanic	Women	32	0.000363, 0.000407, 0.000456, 0.00051, 0.000572
	Hispanic	Women	33	0.000376, 0.000423, 0.000475, 0.000534, 0.0006
	Hispanic	Women	34	0.000383, 0.000435, 0.000493, 0.000558, 0.000634
	Hispanic	Women	35	0.000386, 0.000443, 0.000509, 0.000584, 0.000671
	Hispanic	Women	36	0.000389, 0.000452, 0.000525, 0.000609, 0.000709
	Hispanic	Women	37	0.000395, 0.000463, 0.000542, 0.000634, 0.000743
	Hispanic	Women	38	0.000408, 0.000479, 0.000562, 0.000659, 0.000774
	Hispanic	Women	39	0.000427, 0.000501, 0.000586, 0.000687, 0.000806
	Hispanic	Women	40	0.000453, 0.000529, 0.000617, 0.000719, 0.00084
	Hispanic	Women	41	0.000488, 0.000566, 0.000655, 0.000759, 0.000881
	Hispanic	Women	42	0.000533, 0.000613, 0.000703, 0.000807, 0.000928
	Hispanic	Women	43	0.000591, 0.000672, 0.000762, 0.000866, 0.000984
	Hispanic	Women	44	0.000662, 0.000744, 0.000835, 0.000936, 0.00105
	Hispanic	Women	45	0.000743, 0.000828, 0.000922, 0.00103, 0.00114
	Hispanic	Women	46	0.000831, 0.000923, 0.00102, 0.00114, 0.00126
	Hispanic	Women	47	0.000927, 0.00103, 0.00114, 0.00127, 0.00141
	Hispanic	Women	48	0.00103, 0.00115, 0.00128, 0.00143, 0.00159
	Hispanic	Women	49	0.00115, 0.00129, 0.00144, 0.0016, 0.00179
	Hispanic	Women	50	0.00129, 0.00144, 0.0016, 0.00179, 0.002
	Hispanic	Women	51	0.00145, 0.00161, 0.00179, 0.00198, 0.0022
	Hispanic	Women	52	0.00165, 0.00181, 0.00198, 0.00217, 0.00238
	Hispanic	Women	53	0.00186, 0.00202, 0.00219, 0.00237, 0.00258
	Hispanic	Women	54	0.00205, 0.00222, 0.0024, 0.0026, 0.00281
	Hispanic	Women	55	0.00223, 0.00242, 0.00263, 0.00285, 0.0031
	Hispanic	Women	56	0.0024, 0.00262, 0.00286, 0.00312, 0.00341
	Hispanic	Women	57	0.00258, 0.00283, 0.0031, 0.00339, 0.00371
	Hispanic	Women	58	0.00277, 0.00304, 0.00334, 0.00366, 0.00402
	Hispanic	Women	59	0.00296, 0.00326, 0.00358, 0.00394, 0.00434
	Hispanic	Women	60	0.00314, 0.00347, 0.00384, 0.00423, 0.00468
	Hispanic	Women	61	0.00334, 0.0037, 0.00409, 0.00453, 0.00501
	Hispanic	Women	62	0.00357, 0.00395, 0.00437, 0.00483, 0.00534
	Hispanic	Women	63	0.00381, 0.00422, 0.00466, 0.00515, 0.0057
	Hispanic	Women	64	0.00407, 0.00451, 0.00499, 0.00552, 0.00611
	Hispanic	Women	65	0.00434, 0.00483, 0.00536, 0.00595, 0.00661
	Hispanic	Women	66	0.00466, 0.00519, 0.00578, 0.00644, 0.00718
	Hispanic	Women	67	0.00503, 0.00562, 0.00627, 0.007, 0.00782
	Hispanic	Women	68	0.00547, 0.00612, 0.00684, 0.00764, 0.00855
	Hispanic	Women	69	0.00598, 0.00669, 0.00748, 0.00836, 0.00935
	Hispanic	Women	70	0.00655, 0.00733, 0.00819, 0.00916, 0.0103
	Hispanic	Women	71	0.0072, 0.00805, 0.009, 0.0101, 0.0113
	Hispanic	Women	72	0.00792, 0.00886, 0.0099, 0.0111, 0.0124
	Hispanic	Women	73	0.00874, 0.00977, 0.0109, 0.0122, 0.0136

Variable	Race/ethnicity	Sex	Age	Distribution
	Hispanic	Women	74	0.00971, 0.0108, 0.012, 0.0134, 0.015
	Hispanic	Women	75	0.0109, 0.0121, 0.0134, 0.0148, 0.0165
	Hispanic	Women	76	0.0122, 0.0135, 0.0149, 0.0165, 0.0182
	Hispanic	Women	77	0.0138, 0.0152, 0.0167, 0.0184, 0.0202
	Hispanic	Women	78	0.0156, 0.0171, 0.0188, 0.0207, 0.0227
	Hispanic	Women	79	0.0176, 0.0194, 0.0213, 0.0234, 0.0257
	Hispanic	Women	80	0.0199, 0.0219, 0.0241, 0.0265, 0.0292
	Hispanic	Women	81	0.0223, 0.0247, 0.0272, 0.0301, 0.0332
	Hispanic	Women	82	0.0249, 0.0277, 0.0307, 0.034, 0.0378
	Hispanic	Women	83	0.0275, 0.0308, 0.0343, 0.0383, 0.0428
	Hispanic	Women	84	0.0302, 0.0339, 0.038, 0.0427, 0.048
	White	Men	30	0.000993, 0.00125, 0.00157, 0.00198, 0.00249
	White	Men	31	0.00105, 0.0013, 0.00162, 0.002, 0.00248
	White	Men	32	0.0011, 0.00135, 0.00165, 0.00201, 0.00246
	White	Men	33	0.00113, 0.00137, 0.00166, 0.00202, 0.00245
	White	Men	34	0.00114, 0.00138, 0.00167, 0.00203, 0.00246
	White	Men	35	0.00113, 0.00137, 0.00167, 0.00204, 0.00249
	White	Men	36	0.00111, 0.00137, 0.00167, 0.00205, 0.00251
	White	Men	37	0.00111, 0.00137, 0.00168, 0.00206, 0.00254
	White	Men	38	0.00112, 0.00138, 0.00169, 0.00208, 0.00256
	White	Men	39	0.00115, 0.00141, 0.00172, 0.00211, 0.00258
	White	Men	40	0.0012, 0.00146, 0.00177, 0.00215, 0.00262
	White	Men	41	0.00126, 0.00153, 0.00184, 0.00223, 0.0027
	White	Men	42	0.00134, 0.00162, 0.00194, 0.00234, 0.00281
	White	Men	43	0.00146, 0.00174, 0.00208, 0.00248, 0.00296
	White	Men	44	0.00161, 0.00191, 0.00225, 0.00266, 0.00315
	White	Men	45	0.00178, 0.0021, 0.00247, 0.00291, 0.00342
	White	Men	46	0.00196, 0.00232, 0.00274, 0.00323, 0.00382
	White	Men	47	0.00216, 0.00257, 0.00306, 0.00363, 0.00432
	White	Men	48	0.00239, 0.00286, 0.00342, 0.00409, 0.0049
	White	Men	49	0.00267, 0.0032, 0.00384, 0.00459, 0.00551
	White	Men	50	0.003, 0.00359, 0.00429, 0.00512, 0.00613
	White	Men	51	0.0034, 0.00403, 0.00477, 0.00565, 0.00671
	White	Men	52	0.00386, 0.00452, 0.00529, 0.00618, 0.00723
	White	Men	53	0.00438, 0.00505, 0.00581, 0.00669, 0.00772
	White	Men	54	0.0049, 0.00558, 0.00635, 0.00723, 0.00824
	White	Men	55	0.00537, 0.00609, 0.0069, 0.00781, 0.00886
	White	Men	56	0.00576, 0.00655, 0.00744, 0.00845, 0.0096
	White	Men	57	0.00612, 0.00699, 0.00797, 0.00909, 0.0104
	White	Men	58	0.0065, 0.00743, 0.00848, 0.00968, 0.0111
	White	Men	59	0.00691, 0.00788, 0.00897, 0.0102, 0.0116
	White	Men	60	0.00736, 0.00834, 0.00944, 0.0107, 0.0121
	White	Men	61	0.00786, 0.00883, 0.0099, 0.0111, 0.0125

Variable	Race/ethnicity	Sex	Age	Distribution
	White	Men	62	0.00838, 0.00933, 0.0104, 0.0115, 0.0128
	White	Men	63	0.00891, 0.00985, 0.0109, 0.012, 0.0133
	White	Men	64	0.00944, 0.0104, 0.0114, 0.0126, 0.0139
	White	Men	65	0.00998, 0.011, 0.0121, 0.0133, 0.0147
	White	Men	66	0.0106, 0.0117, 0.0129, 0.0143, 0.0157
	White	Men	67	0.0113, 0.0125, 0.0139, 0.0154, 0.017
	White	Men	68	0.0121, 0.0135, 0.015, 0.0166, 0.0185
	White	Men	69	0.0131, 0.0146, 0.0162, 0.0181, 0.0202
	White	Men	70	0.0142, 0.0158, 0.0177, 0.0197, 0.022
	White	Men	71	0.0155, 0.0173, 0.0193, 0.0215, 0.024
	White	Men	72	0.0169, 0.0188, 0.021, 0.0234, 0.0262
	White	Men	73	0.0185, 0.0206, 0.0229, 0.0255, 0.0284
	White	Men	74	0.0202, 0.0225, 0.025, 0.0278, 0.0309
	White	Men	75	0.0223, 0.0247, 0.0273, 0.0303, 0.0335
	White	Men	76	0.0247, 0.0272, 0.03, 0.0331, 0.0366
	White	Men	77	0.0274, 0.0301, 0.0332, 0.0366, 0.0403
	White	Men	78	0.0304, 0.0335, 0.0369, 0.0406, 0.0448
	White	Men	79	0.0339, 0.0374, 0.0412, 0.0455, 0.0502
	White	Men	80	0.0379, 0.0419, 0.0462, 0.0511, 0.0564
	White	Men	81	0.0423, 0.0469, 0.0518, 0.0574, 0.0635
	White	Men	82	0.047, 0.0522, 0.0579, 0.0643, 0.0714
	White	Men	83	0.0517, 0.0577, 0.0643, 0.0717, 0.08
	White	Men	84	0.0563, 0.0631, 0.0707, 0.0792, 0.0888
	White	Women	30	0.000537, 0.000627, 0.000732, 0.000853, 0.000997
	White	Women	31	0.000566, 0.000663, 0.000776, 0.000908, 0.00106
	White	Women	32	0.000596, 0.000698, 0.000816, 0.000955, 0.00112
	White	Women	33	0.000622, 0.000729, 0.000853, 0.000998, 0.00117
	White	Women	34	0.000635, 0.00075, 0.000885, 0.00104, 0.00123
	White	Women	35	0.000637, 0.000763, 0.000914, 0.00109, 0.00131
	White	Women	36	0.000637, 0.000775, 0.000941, 0.00114, 0.00139
	White	Women	37	0.000641, 0.000789, 0.00097, 0.00119, 0.00147
	White	Women	38	0.000648, 0.000807, 0.001, 0.00124, 0.00155
	White	Women	39	0.000661, 0.000831, 0.00104, 0.00131, 0.00164
	White	Women	40	0.000683, 0.000864, 0.00109, 0.00138, 0.00174
	White	Women	41	0.00072, 0.000912, 0.00115, 0.00146, 0.00185
	White	Women	42	0.000772, 0.000976, 0.00123, 0.00155, 0.00196
	White	Women	43	0.000838, 0.00106, 0.00133, 0.00167, 0.0021
	White	Women	44	0.000916, 0.00115, 0.00145, 0.00181, 0.00228
	White	Women	45	0.00101, 0.00127, 0.00159, 0.00199, 0.00251
	White	Women	46	0.00111, 0.0014, 0.00175, 0.0022, 0.00277
	White	Women	47	0.00125, 0.00156, 0.00195, 0.00243, 0.00304
	White	Women	48	0.00142, 0.00176, 0.00217, 0.00267, 0.0033
	White	Women	49	0.00163, 0.00198, 0.00241, 0.00294, 0.00358

Variable	Race/ethnicity	Sex	Age	Distribution
	White	Women	50	0.00183, 0.00222, 0.00268, 0.00324, 0.00393
	White	Women	51	0.002, 0.00244, 0.00297, 0.0036, 0.00439
	White	Women	52	0.00217, 0.00266, 0.00326, 0.004, 0.00491
	White	Women	53	0.00235, 0.0029, 0.00357, 0.00439, 0.00541
	White	Women	54	0.00258, 0.00316, 0.00387, 0.00473, 0.00579
	White	Women	55	0.0029, 0.00348, 0.00416, 0.00498, 0.00597
	White	Women	56	0.0033, 0.00384, 0.00445, 0.00516, 0.00599
	White	Women	57	0.00373, 0.0042, 0.00473, 0.00532, 0.00599
	White	Women	58	0.00412, 0.00454, 0.005, 0.0055, 0.00606
	White	Women	59	0.00441, 0.00482, 0.00527, 0.00576, 0.0063
	White	Women	60	0.00463, 0.00508, 0.00556, 0.00608, 0.00666
	White	Women	61	0.00488, 0.00535, 0.00587, 0.00643, 0.00706
	White	Women	62	0.00516, 0.00567, 0.00622, 0.00683, 0.0075
	White	Women	63	0.0055, 0.00604, 0.00663, 0.00727, 0.00799
	White	Women	64	0.00589, 0.00647, 0.00711, 0.0078, 0.00858
	White	Women	65	0.00632, 0.00696, 0.00767, 0.00844, 0.0093
	White	Women	66	0.0068, 0.00753, 0.00832, 0.0092, 0.0102
	White	Women	67	0.00732, 0.00816, 0.00907, 0.0101, 0.0112
	White	Women	68	0.00789, 0.00885, 0.00993, 0.0111, 0.0125
	White	Women	69	0.00852, 0.00964, 0.0109, 0.0123, 0.0139
	White	Women	70	0.00924, 0.0105, 0.0119, 0.0136, 0.0154
	White	Women	71	0.0101, 0.0115, 0.0131, 0.0149, 0.017
	White	Women	72	0.011, 0.0126, 0.0144, 0.0164, 0.0188
	White	Women	73	0.0121, 0.0138, 0.0158, 0.018, 0.0206
	White	Women	74	0.0133, 0.0152, 0.0173, 0.0198, 0.0225
	White	Women	75	0.0149, 0.0169, 0.0191, 0.0217, 0.0246
	White	Women	76	0.0167, 0.0188, 0.0212, 0.0239, 0.027
	White	Women	77	0.0188, 0.0211, 0.0236, 0.0264, 0.0297
	White	Women	78	0.0212, 0.0237, 0.0264, 0.0295, 0.0329
	White	Women	79	0.024, 0.0267, 0.0297, 0.0331, 0.0369
	White	Women	80	0.027, 0.0301, 0.0335, 0.0373, 0.0415
	White	Women	81	0.0304, 0.0339, 0.0378, 0.0421, 0.0469
	White	Women	82	0.034, 0.038, 0.0424, 0.0474, 0.053
	White	Women	83	0.0376, 0.0422, 0.0474, 0.0531, 0.0596
	White	Women	84	0.0414, 0.0466, 0.0525, 0.0591, 0.0665
Total mortality rates for 2033 (0.01, 0.2, 0.5, 0.8. 0.99 percentiles of the empirical distribution produced during forecasting)				
	Black	Men	30	0.00148, 0.00185, 0.00231, 0.00289, 0.00362
	Black	Men	31	0.0016, 0.00194, 0.00236, 0.00286, 0.00348
	Black	Men	32	0.00169, 0.00201, 0.00239, 0.00284, 0.00338
	Black	Men	33	0.00173, 0.00204, 0.00242, 0.00286, 0.00338
	Black	Men	34	0.00174, 0.00206, 0.00244, 0.00289, 0.00343
	Black	Men	35	0.00174, 0.00207, 0.00246, 0.00292, 0.00348
	Black	Men	36	0.00175, 0.00208, 0.00248, 0.00295, 0.00351

Variable	Race/ethnicity	Sex	Age	Distribution
	Black	Men	37	0.00175, 0.00209, 0.00249, 0.00296, 0.00353
	Black	Men	38	0.00176, 0.0021, 0.0025, 0.00298, 0.00356
	Black	Men	39	0.00175, 0.0021, 0.00252, 0.00302, 0.00363
	Black	Men	40	0.00174, 0.00212, 0.00256, 0.0031, 0.00377
	Black	Men	41	0.00175, 0.00215, 0.00264, 0.00324, 0.00398
	Black	Men	42	0.00177, 0.00221, 0.00276, 0.00343, 0.00429
	Black	Men	43	0.00182, 0.00231, 0.00292, 0.0037, 0.00469
	Black	Men	44	0.0019, 0.00245, 0.00314, 0.00404, 0.0052
	Black	Men	45	0.00201, 0.00263, 0.00342, 0.00446, 0.00583
	Black	Men	46	0.00216, 0.00286, 0.00376, 0.00496, 0.00655
	Black	Men	47	0.00237, 0.00315, 0.00417, 0.00552, 0.00733
	Black	Men	48	0.00266, 0.00353, 0.00466, 0.00615, 0.00814
	Black	Men	49	0.00304, 0.00398, 0.00521, 0.00682, 0.00895
	Black	Men	50	0.00349, 0.00452, 0.00585, 0.00756, 0.00981
	Black	Men	51	0.00401, 0.00513, 0.00655, 0.00836, 0.0107
	Black	Men	52	0.00463, 0.00583, 0.00732, 0.0092, 0.0116
	Black	Men	53	0.00537, 0.00663, 0.00816, 0.01, 0.0124
	Black	Men	54	0.00622, 0.00751, 0.00904, 0.0109, 0.0131
	Black	Men	55	0.00716, 0.00846, 0.00997, 0.0118, 0.0139
	Black	Men	56	0.00816, 0.00946, 0.0109, 0.0127, 0.0147
	Black	Men	57	0.00921, 0.0105, 0.0119, 0.0136, 0.0155
	Black	Men	58	0.0103, 0.0115, 0.0129, 0.0145, 0.0163
	Black	Men	59	0.0113, 0.0125, 0.0139, 0.0155, 0.0172
	Black	Men	60	0.0121, 0.0134, 0.0148, 0.0164, 0.0182
	Black	Men	61	0.0129, 0.0142, 0.0157, 0.0173, 0.0191
	Black	Men	62	0.0137, 0.015, 0.0165, 0.0181, 0.0198
	Black	Men	63	0.0144, 0.0157, 0.0172, 0.0189, 0.0207
	Black	Men	64	0.0149, 0.0164, 0.018, 0.0197, 0.0216
	Black	Men	65	0.0155, 0.0171, 0.0188, 0.0206, 0.0227
	Black	Men	66	0.0162, 0.0178, 0.0197, 0.0217, 0.0239
	Black	Men	67	0.0169, 0.0187, 0.0207, 0.0229, 0.0253
	Black	Men	68	0.0178, 0.0197, 0.0219, 0.0243, 0.0271
	Black	Men	69	0.0188, 0.0209, 0.0233, 0.026, 0.029
	Black	Men	70	0.0199, 0.0223, 0.0249, 0.0278, 0.0312
	Black	Men	71	0.0213, 0.0238, 0.0267, 0.0298, 0.0335
	Black	Men	72	0.0227, 0.0255, 0.0286, 0.032, 0.0359
	Black	Men	73	0.0244, 0.0274, 0.0306, 0.0343, 0.0384
	Black	Men	74	0.0264, 0.0295, 0.0329, 0.0367, 0.041
	Black	Men	75	0.0286, 0.0318, 0.0354, 0.0393, 0.0438
	Black	Men	76	0.0311, 0.0345, 0.0382, 0.0423, 0.0469
	Black	Men	77	0.0338, 0.0374, 0.0413, 0.0456, 0.0505
	Black	Men	78	0.0367, 0.0406, 0.0449, 0.0496, 0.0549
	Black	Men	79	0.0397, 0.0441, 0.0489, 0.0541, 0.0601

Variable	Race/ethnicity	Sex	Age	Distribution
	Black	Men	80	0.043, 0.0479, 0.0533, 0.0592, 0.066
	Black	Men	81	0.0465, 0.0519, 0.058, 0.0648, 0.0724
	Black	Men	82	0.05, 0.0561, 0.063, 0.0706, 0.0793
	Black	Men	83	0.0533, 0.0602, 0.0679, 0.0766, 0.0865
	Black	Men	84	0.0566, 0.0642, 0.0728, 0.0825, 0.0937
	Black	Women	30	0.000498, 0.000673, 0.000907, 0.00122, 0.00165
	Black	Women	31	0.00057, 0.000747, 0.000978, 0.00128, 0.00168
	Black	Women	32	0.000653, 0.00083, 0.00105, 0.00133, 0.0017
	Black	Women	33	0.000733, 0.000909, 0.00113, 0.00139, 0.00173
	Black	Women	34	0.000792, 0.000973, 0.00119, 0.00146, 0.00179
	Black	Women	35	0.000825, 0.00102, 0.00125, 0.00153, 0.00189
	Black	Women	36	0.000844, 0.00105, 0.00129, 0.0016, 0.00199
	Black	Women	37	0.000863, 0.00108, 0.00134, 0.00166, 0.00207
	Black	Women	38	0.000891, 0.00111, 0.00138, 0.00172, 0.00215
	Black	Women	39	0.00093, 0.00116, 0.00144, 0.00179, 0.00224
	Black	Women	40	0.000985, 0.00122, 0.00152, 0.00188, 0.00234
	Black	Women	41	0.00106, 0.00131, 0.00161, 0.00198, 0.00245
	Black	Women	42	0.00117, 0.00143, 0.00173, 0.0021, 0.00255
	Black	Women	43	0.00132, 0.00157, 0.00187, 0.00223, 0.00266
	Black	Women	44	0.0015, 0.00175, 0.00204, 0.00238, 0.00278
	Black	Women	45	0.00169, 0.00194, 0.00224, 0.00258, 0.00298
	Black	Women	46	0.00187, 0.00215, 0.00248, 0.00285, 0.00328
	Black	Women	47	0.00205, 0.00238, 0.00275, 0.00319, 0.0037
	Black	Women	48	0.00225, 0.00263, 0.00307, 0.00359, 0.00419
	Black	Women	49	0.00251, 0.00294, 0.00344, 0.00402, 0.00471
	Black	Women	50	0.00283, 0.0033, 0.00384, 0.00447, 0.0052
	Black	Women	51	0.00325, 0.00373, 0.00428, 0.0049, 0.00562
	Black	Women	52	0.00375, 0.00422, 0.00474, 0.00533, 0.00599
	Black	Women	53	0.00426, 0.00472, 0.00523, 0.00579, 0.00641
	Black	Women	54	0.00475, 0.00522, 0.00573, 0.0063, 0.00692
	Black	Women	55	0.00521, 0.00571, 0.00626, 0.00685, 0.00751
	Black	Women	56	0.00565, 0.00619, 0.00678, 0.00742, 0.00814
	Black	Women	57	0.00607, 0.00666, 0.0073, 0.00799, 0.00877
	Black	Women	58	0.00646, 0.00709, 0.00778, 0.00854, 0.00938
	Black	Women	59	0.00678, 0.00748, 0.00823, 0.00906, 0.00999
	Black	Women	60	0.00706, 0.00781, 0.00864, 0.00955, 0.0106
	Black	Women	61	0.00734, 0.00814, 0.00901, 0.00998, 0.0111
	Black	Women	62	0.00764, 0.00847, 0.00938, 0.0104, 0.0115
	Black	Women	63	0.00796, 0.00883, 0.00978, 0.0108, 0.012
	Black	Women	64	0.00831, 0.00923, 0.0102, 0.0114, 0.0126
	Black	Women	65	0.00871, 0.0097, 0.0108, 0.012, 0.0134
	Black	Women	66	0.00921, 0.0103, 0.0115, 0.0128, 0.0143
	Black	Women	67	0.00981, 0.011, 0.0123, 0.0137, 0.0154

Variable	Race/ethnicity	Sex	Age	Distribution
	Black	Women	68	0.0105, 0.0118, 0.0132, 0.0148, 0.0166
	Black	Women	69	0.0113, 0.0127, 0.0142, 0.016, 0.0179
	Black	Women	70	0.0122, 0.0137, 0.0154, 0.0172, 0.0194
	Black	Women	71	0.0132, 0.0148, 0.0166, 0.0186, 0.0209
	Black	Women	72	0.0143, 0.016, 0.0179, 0.02, 0.0225
	Black	Women	73	0.0154, 0.0173, 0.0193, 0.0216, 0.0242
	Black	Women	74	0.0168, 0.0187, 0.0209, 0.0233, 0.026
	Black	Women	75	0.0184, 0.0204, 0.0227, 0.0252, 0.0281
	Black	Women	76	0.0202, 0.0224, 0.0248, 0.0274, 0.0304
	Black	Women	77	0.0222, 0.0246, 0.0271, 0.03, 0.0331
	Black	Women	78	0.0245, 0.0271, 0.0299, 0.033, 0.0364
	Black	Women	79	0.027, 0.0299, 0.033, 0.0364, 0.0402
	Black	Women	80	0.0298, 0.033, 0.0364, 0.0403, 0.0446
	Black	Women	81	0.0326, 0.0362, 0.0402, 0.0446, 0.0495
	Black	Women	82	0.0355, 0.0396, 0.0442, 0.0493, 0.055
	Black	Women	83	0.0383, 0.0431, 0.0483, 0.0542, 0.0609
	Black	Women	84	0.0412, 0.0465, 0.0525, 0.0593, 0.067
	Hispanic	Men	30	0.000782, 0.000892, 0.00102, 0.00116, 0.00132
	Hispanic	Men	31	0.000801, 0.000912, 0.00104, 0.00118, 0.00134
	Hispanic	Men	32	0.000805, 0.000922, 0.00105, 0.00121, 0.00138
	Hispanic	Men	33	0.000786, 0.000916, 0.00107, 0.00124, 0.00144
	Hispanic	Men	34	0.00076, 0.000903, 0.00107, 0.00127, 0.00151
	Hispanic	Men	35	0.000741, 0.000892, 0.00107, 0.00129, 0.00156
	Hispanic	Men	36	0.000733, 0.000889, 0.00108, 0.0013, 0.00158
	Hispanic	Men	37	0.000739, 0.000896, 0.00108, 0.00131, 0.00159
	Hispanic	Men	38	0.000757, 0.000914, 0.0011, 0.00132, 0.0016
	Hispanic	Men	39	0.000782, 0.000939, 0.00113, 0.00135, 0.00162
	Hispanic	Men	40	0.000811, 0.000974, 0.00117, 0.0014, 0.00168
	Hispanic	Men	41	0.000851, 0.00102, 0.00122, 0.00147, 0.00176
	Hispanic	Men	42	0.000914, 0.00109, 0.0013, 0.00155, 0.00185
	Hispanic	Men	43	0.00101, 0.00119, 0.0014, 0.00164, 0.00193
	Hispanic	Men	44	0.00115, 0.00132, 0.00152, 0.00176, 0.00203
	Hispanic	Men	45	0.0013, 0.00148, 0.00168, 0.00191, 0.00217
	Hispanic	Men	46	0.00147, 0.00166, 0.00187, 0.00211, 0.00238
	Hispanic	Men	47	0.00165, 0.00186, 0.0021, 0.00236, 0.00267
	Hispanic	Men	48	0.00184, 0.00209, 0.00236, 0.00267, 0.00302
	Hispanic	Men	49	0.00206, 0.00234, 0.00265, 0.003, 0.0034
	Hispanic	Men	50	0.00233, 0.00263, 0.00297, 0.00335, 0.00379
	Hispanic	Men	51	0.00266, 0.00297, 0.00331, 0.00369, 0.00412
	Hispanic	Men	52	0.00308, 0.00336, 0.00367, 0.00401, 0.00438
	Hispanic	Men	53	0.00355, 0.00379, 0.00404, 0.00431, 0.0046
	Hispanic	Men	54	0.00398, 0.0042, 0.00443, 0.00467, 0.00493
	Hispanic	Men	55	0.00428, 0.00455, 0.00483, 0.00513, 0.00545

Variable	Race/ethnicity	Sex	Age	Distribution
	Hispanic	Men	56	0.00453, 0.00487, 0.00523, 0.00562, 0.00604
	Hispanic	Men	57	0.0048, 0.00521, 0.00564, 0.00612, 0.00663
	Hispanic	Men	58	0.00507, 0.00554, 0.00605, 0.0066, 0.00721
	Hispanic	Men	59	0.00533, 0.00586, 0.00644, 0.00708, 0.00779
	Hispanic	Men	60	0.00559, 0.00618, 0.00683, 0.00754, 0.00834
	Hispanic	Men	61	0.00588, 0.00651, 0.0072, 0.00796, 0.00881
	Hispanic	Men	62	0.00622, 0.00686, 0.00757, 0.00835, 0.00922
	Hispanic	Men	63	0.00658, 0.00724, 0.00796, 0.00875, 0.00962
	Hispanic	Men	64	0.00695, 0.00764, 0.00839, 0.00921, 0.0101
	Hispanic	Men	65	0.00732, 0.00807, 0.00888, 0.00978, 0.0108
	Hispanic	Men	66	0.00773, 0.00856, 0.00946, 0.0105, 0.0116
	Hispanic	Men	67	0.00821, 0.00913, 0.0101, 0.0113, 0.0125
	Hispanic	Men	68	0.00879, 0.00981, 0.0109, 0.0122, 0.0136
	Hispanic	Men	69	0.00946, 0.0106, 0.0118, 0.0132, 0.0148
	Hispanic	Men	70	0.0102, 0.0115, 0.0129, 0.0144, 0.0162
	Hispanic	Men	71	0.0111, 0.0125, 0.014, 0.0157, 0.0177
	Hispanic	Men	72	0.0121, 0.0136, 0.0153, 0.0171, 0.0193
	Hispanic	Men	73	0.0131, 0.0148, 0.0166, 0.0187, 0.0211
	Hispanic	Men	74	0.0144, 0.0162, 0.0182, 0.0204, 0.023
	Hispanic	Men	75	0.0158, 0.0178, 0.0199, 0.0224, 0.0251
	Hispanic	Men	76	0.0175, 0.0196, 0.022, 0.0246, 0.0276
	Hispanic	Men	77	0.0195, 0.0218, 0.0243, 0.0272, 0.0305
	Hispanic	Men	78	0.0217, 0.0243, 0.0271, 0.0303, 0.0339
	Hispanic	Men	79	0.0243, 0.0271, 0.0303, 0.0338, 0.0378
	Hispanic	Men	80	0.0272, 0.0304, 0.0339, 0.0378, 0.0423
	Hispanic	Men	81	0.0304, 0.034, 0.0379, 0.0423, 0.0472
	Hispanic	Men	82	0.0338, 0.0378, 0.0422, 0.0471, 0.0526
	Hispanic	Men	83	0.037, 0.0415, 0.0466, 0.0522, 0.0585
	Hispanic	Men	84	0.0402, 0.0452, 0.0509, 0.0573, 0.0645
	Hispanic	Women	30	0.000323, 0.000365, 0.000413, 0.000467, 0.000528
	Hispanic	Women	31	0.000344, 0.000387, 0.000435, 0.000488, 0.000549
	Hispanic	Women	32	0.000363, 0.000407, 0.000455, 0.00051, 0.000572
	Hispanic	Women	33	0.000375, 0.000422, 0.000474, 0.000533, 0.0006
	Hispanic	Women	34	0.000381, 0.000433, 0.000491, 0.000558, 0.000634
	Hispanic	Women	35	0.000382, 0.000441, 0.000507, 0.000583, 0.000671
	Hispanic	Women	36	0.000384, 0.000448, 0.000522, 0.000607, 0.000708
	Hispanic	Women	37	0.00039, 0.000458, 0.000537, 0.00063, 0.000741
	Hispanic	Women	38	0.000401, 0.000473, 0.000556, 0.000654, 0.000771
	Hispanic	Women	39	0.000419, 0.000493, 0.000579, 0.00068, 0.0008
	Hispanic	Women	40	0.000444, 0.00052, 0.000608, 0.000712, 0.000833
	Hispanic	Women	41	0.000478, 0.000556, 0.000645, 0.00075, 0.000872
	Hispanic	Women	42	0.000521, 0.000601, 0.000692, 0.000797, 0.000919
	Hispanic	Women	43	0.000578, 0.000659, 0.00075, 0.000853, 0.000973

Variable	Race/ethnicity	Sex	Age	Distribution
	Hispanic	Women	44	0.000648, 0.00073, 0.000821, 0.000923, 0.00104
	Hispanic	Women	45	0.000728, 0.000813, 0.000906, 0.00101, 0.00113
	Hispanic	Women	46	0.000815, 0.000907, 0.00101, 0.00112, 0.00125
	Hispanic	Women	47	0.000909, 0.00101, 0.00113, 0.00125, 0.0014
	Hispanic	Women	48	0.00101, 0.00113, 0.00126, 0.00141, 0.00158
	Hispanic	Women	49	0.00113, 0.00127, 0.00142, 0.00159, 0.00178
	Hispanic	Women	50	0.00127, 0.00142, 0.00159, 0.00177, 0.00198
	Hispanic	Women	51	0.00143, 0.00159, 0.00177, 0.00196, 0.00218
	Hispanic	Women	52	0.00163, 0.00179, 0.00196, 0.00215, 0.00237
	Hispanic	Women	53	0.00184, 0.002, 0.00217, 0.00235, 0.00256
	Hispanic	Women	54	0.00203, 0.0022, 0.00238, 0.00258, 0.0028
	Hispanic	Women	55	0.0022, 0.0024, 0.00261, 0.00283, 0.00308
	Hispanic	Women	56	0.00237, 0.0026, 0.00284, 0.0031, 0.00339
	Hispanic	Women	57	0.00256, 0.0028, 0.00307, 0.00337, 0.0037
	Hispanic	Women	58	0.00274, 0.00302, 0.00331, 0.00364, 0.004
	Hispanic	Women	59	0.00292, 0.00323, 0.00355, 0.00392, 0.00432
	Hispanic	Women	60	0.0031, 0.00343, 0.0038, 0.0042, 0.00466
	Hispanic	Women	61	0.0033, 0.00366, 0.00405, 0.00449, 0.00498
	Hispanic	Women	62	0.00351, 0.0039, 0.00432, 0.00478, 0.0053
	Hispanic	Women	63	0.00375, 0.00416, 0.0046, 0.00509, 0.00564
	Hispanic	Women	64	0.004, 0.00444, 0.00492, 0.00545, 0.00605
	Hispanic	Women	65	0.00427, 0.00475, 0.00528, 0.00587, 0.00654
	Hispanic	Women	66	0.00457, 0.00511, 0.0057, 0.00636, 0.0071
	Hispanic	Women	67	0.00493, 0.00552, 0.00618, 0.00691, 0.00774
	Hispanic	Women	68	0.00536, 0.00601, 0.00673, 0.00754, 0.00845
	Hispanic	Women	69	0.00585, 0.00657, 0.00736, 0.00825, 0.00925
	Hispanic	Women	70	0.00642, 0.0072, 0.00807, 0.00904, 0.0101
	Hispanic	Women	71	0.00705, 0.0079, 0.00885, 0.00992, 0.0111
	Hispanic	Women	72	0.00775, 0.00869, 0.00974, 0.0109, 0.0122
	Hispanic	Women	73	0.00856, 0.00959, 0.0107, 0.012, 0.0134
	Hispanic	Women	74	0.0095, 0.0106, 0.0119, 0.0132, 0.0148
	Hispanic	Women	75	0.0106, 0.0118, 0.0131, 0.0146, 0.0163
	Hispanic	Women	76	0.012, 0.0132, 0.0147, 0.0162, 0.018
	Hispanic	Women	77	0.0135, 0.0149, 0.0164, 0.0181, 0.02
	Hispanic	Women	78	0.0152, 0.0168, 0.0185, 0.0204, 0.0225
	Hispanic	Women	79	0.0172, 0.019, 0.0209, 0.0231, 0.0254
	Hispanic	Women	80	0.0195, 0.0215, 0.0237, 0.0262, 0.0289
	Hispanic	Women	81	0.0219, 0.0243, 0.0268, 0.0297, 0.0329
	Hispanic	Women	82	0.0244, 0.0272, 0.0302, 0.0336, 0.0374
	Hispanic	Women	83	0.027, 0.0302, 0.0338, 0.0378, 0.0424
	Hispanic	Women	84	0.0296, 0.0333, 0.0375, 0.0422, 0.0475
	White	Men	30	0.00098, 0.00124, 0.00157, 0.00198, 0.00251
	White	Men	31	0.00104, 0.0013, 0.00161, 0.00201, 0.0025

Variable	Race/ethnicity	Sex	Age	Distribution
	White	Men	32	0.00109, 0.00134, 0.00164, 0.00201, 0.00247
	White	Men	33	0.00112, 0.00136, 0.00166, 0.00202, 0.00246
	White	Men	34	0.00112, 0.00137, 0.00166, 0.00203, 0.00247
	White	Men	35	0.00111, 0.00136, 0.00166, 0.00203, 0.00249
	White	Men	36	0.0011, 0.00135, 0.00166, 0.00204, 0.00252
	White	Men	37	0.00109, 0.00135, 0.00166, 0.00205, 0.00254
	White	Men	38	0.0011, 0.00136, 0.00168, 0.00207, 0.00255
	White	Men	39	0.00113, 0.00139, 0.0017, 0.00209, 0.00257
	White	Men	40	0.00117, 0.00143, 0.00175, 0.00213, 0.00261
	White	Men	41	0.00123, 0.00149, 0.00181, 0.0022, 0.00268
	White	Men	42	0.00131, 0.00158, 0.00191, 0.00231, 0.00279
	White	Men	43	0.00142, 0.00171, 0.00204, 0.00244, 0.00293
	White	Men	44	0.00157, 0.00187, 0.00221, 0.00262, 0.00312
	White	Men	45	0.00174, 0.00205, 0.00243, 0.00287, 0.00339
	White	Men	46	0.00191, 0.00227, 0.00269, 0.00319, 0.00378
	White	Men	47	0.00211, 0.00252, 0.00301, 0.00359, 0.00429
	White	Men	48	0.00234, 0.00281, 0.00337, 0.00405, 0.00486
	White	Men	49	0.00261, 0.00315, 0.00378, 0.00455, 0.00548
	White	Men	50	0.00294, 0.00353, 0.00424, 0.00508, 0.0061
	White	Men	51	0.00333, 0.00397, 0.00472, 0.00561, 0.00668
	White	Men	52	0.00379, 0.00446, 0.00523, 0.00614, 0.00721
	White	Men	53	0.00431, 0.00498, 0.00576, 0.00665, 0.0077
	White	Men	54	0.00482, 0.00551, 0.0063, 0.00719, 0.00822
	White	Men	55	0.00529, 0.00602, 0.00684, 0.00777, 0.00884
	White	Men	56	0.00568, 0.00647, 0.00738, 0.0084, 0.00959
	White	Men	57	0.00603, 0.00691, 0.0079, 0.00904, 0.0104
	White	Men	58	0.0064, 0.00734, 0.00841, 0.00963, 0.011
	White	Men	59	0.0068, 0.00778, 0.00889, 0.0102, 0.0116
	White	Men	60	0.00724, 0.00823, 0.00935, 0.0106, 0.0121
	White	Men	61	0.00773, 0.0087, 0.0098, 0.011, 0.0124
	White	Men	62	0.00824, 0.00919, 0.0102, 0.0114, 0.0127
	White	Men	63	0.00875, 0.0097, 0.0107, 0.0119, 0.0132
	White	Men	64	0.00926, 0.0102, 0.0113, 0.0125, 0.0138
	White	Men	65	0.00979, 0.0108, 0.0119, 0.0132, 0.0146
	White	Men	66	0.0104, 0.0115, 0.0127, 0.0141, 0.0156
	White	Men	67	0.011, 0.0123, 0.0136, 0.0152, 0.0168
	White	Men	68	0.0118, 0.0132, 0.0147, 0.0164, 0.0183
	White	Men	69	0.0128, 0.0143, 0.016, 0.0179, 0.02
	White	Men	70	0.0139, 0.0156, 0.0174, 0.0195, 0.0218
	White	Men	71	0.0151, 0.0169, 0.019, 0.0212, 0.0238
	White	Men	72	0.0165, 0.0185, 0.0207, 0.0231, 0.0259
	White	Men	73	0.0181, 0.0202, 0.0225, 0.0251, 0.0281
	White	Men	74	0.0198, 0.0221, 0.0246, 0.0274, 0.0305

Variable	Race/ethnicity	Sex	Age	Distribution
	White	Men	75	0.0218, 0.0242, 0.0269, 0.0298, 0.0331
	White	Men	76	0.0241, 0.0267, 0.0295, 0.0327, 0.0362
	White	Men	77	0.0268, 0.0296, 0.0327, 0.036, 0.0398
	White	Men	78	0.0298, 0.0329, 0.0363, 0.0401, 0.0443
	White	Men	79	0.0332, 0.0367, 0.0406, 0.0449, 0.0496
	White	Men	80	0.0371, 0.0411, 0.0455, 0.0504, 0.0558
	White	Men	81	0.0415, 0.046, 0.0511, 0.0566, 0.0629
	White	Men	82	0.0461, 0.0513, 0.0571, 0.0635, 0.0707
	White	Men	83	0.0507, 0.0567, 0.0634, 0.0708, 0.0792
	White	Men	84	0.0552, 0.0621, 0.0697, 0.0782, 0.0879
	White	Women	30	0.000532, 0.000624, 0.00073, 0.000854, 0.001
	White	Women	31	0.000561, 0.000659, 0.000774, 0.000908, 0.00107
	White	Women	32	0.000591, 0.000694, 0.000814, 0.000955, 0.00112
	White	Women	33	0.000616, 0.000725, 0.00085, 0.000998, 0.00117
	White	Women	34	0.000628, 0.000745, 0.000881, 0.00104, 0.00124
	White	Women	35	0.000628, 0.000756, 0.000909, 0.00109, 0.00131
	White	Women	36	0.000627, 0.000766, 0.000934, 0.00114, 0.00139
	White	Women	37	0.000628, 0.000778, 0.00096, 0.00119, 0.00147
	White	Women	38	0.000634, 0.000793, 0.00099, 0.00124, 0.00155
	White	Women	39	0.000645, 0.000815, 0.00103, 0.00129, 0.00163
	White	Women	40	0.000666, 0.000846, 0.00107, 0.00136, 0.00173
	White	Women	41	0.0007, 0.000892, 0.00113, 0.00144, 0.00183
	White	Women	42	0.00075, 0.000953, 0.00121, 0.00153, 0.00195
	White	Women	43	0.000814, 0.00103, 0.0013, 0.00165, 0.00209
	White	Women	44	0.00089, 0.00112, 0.00142, 0.00179, 0.00226
	White	Women	45	0.000978, 0.00124, 0.00156, 0.00197, 0.00248
	White	Women	46	0.00108, 0.00137, 0.00172, 0.00217, 0.00274
	White	Women	47	0.00122, 0.00153, 0.00192, 0.0024, 0.00301
	White	Women	48	0.00139, 0.00172, 0.00213, 0.00264, 0.00328
	White	Women	49	0.00159, 0.00195, 0.00238, 0.00291, 0.00356
	White	Women	50	0.00179, 0.00218, 0.00264, 0.00321, 0.00391
	White	Women	51	0.00196, 0.0024, 0.00293, 0.00358, 0.00437
	White	Women	52	0.00212, 0.00262, 0.00323, 0.00397, 0.0049
	White	Women	53	0.0023, 0.00285, 0.00353, 0.00436, 0.00541
	White	Women	54	0.00254, 0.00312, 0.00383, 0.0047, 0.00579
	White	Women	55	0.00286, 0.00344, 0.00413, 0.00496, 0.00596
	White	Women	56	0.00326, 0.00379, 0.00441, 0.00513, 0.00598
	White	Women	57	0.00368, 0.00416, 0.00469, 0.00529, 0.00597
	White	Women	58	0.00407, 0.00449, 0.00496, 0.00547, 0.00604
	White	Women	59	0.00435, 0.00477, 0.00522, 0.00572, 0.00627
	White	Women	60	0.00457, 0.00502, 0.0055, 0.00604, 0.00663
	White	Women	61	0.00481, 0.00529, 0.00581, 0.00638, 0.00701
	White	Women	62	0.00508, 0.00559, 0.00615, 0.00676, 0.00744

Variable	Race/ethnicity	Sex	Age	Distribution
	White	Women	63	0.00541, 0.00595, 0.00654, 0.0072, 0.00792
	White	Women	64	0.00578, 0.00637, 0.00701, 0.00771, 0.0085
	White	Women	65	0.0062, 0.00685, 0.00756, 0.00834, 0.0092
	White	Women	66	0.00667, 0.0074, 0.0082, 0.00908, 0.0101
	White	Women	67	0.00717, 0.00801, 0.00893, 0.00996, 0.0111
	White	Women	68	0.00772, 0.00869, 0.00977, 0.011, 0.0124
	White	Women	69	0.00834, 0.00946, 0.0107, 0.0121, 0.0138
	White	Women	70	0.00904, 0.0103, 0.0117, 0.0134, 0.0153
	White	Women	71	0.00984, 0.0113, 0.0129, 0.0147, 0.0169
	White	Women	72	0.0108, 0.0123, 0.0141, 0.0162, 0.0186
	White	Women	73	0.0118, 0.0135, 0.0155, 0.0178, 0.0204
	White	Women	74	0.013, 0.0149, 0.017, 0.0195, 0.0223
	White	Women	75	0.0145, 0.0165, 0.0188, 0.0214, 0.0243
	White	Women	76	0.0163, 0.0184, 0.0208, 0.0235, 0.0267
	White	Women	77	0.0184, 0.0207, 0.0232, 0.0261, 0.0293
	White	Women	78	0.0208, 0.0232, 0.026, 0.0291, 0.0326
	White	Women	79	0.0235, 0.0262, 0.0293, 0.0326, 0.0365
	White	Women	80	0.0265, 0.0296, 0.033, 0.0368, 0.0411
	White	Women	81	0.0298, 0.0333, 0.0372, 0.0415, 0.0464
	White	Women	82	0.0333, 0.0373, 0.0418, 0.0468, 0.0524
	White	Women	83	0.0369, 0.0415, 0.0467, 0.0524, 0.059
	White	Women	84	0.0405, 0.0458, 0.0517, 0.0584, 0.0659
Total mortality rates for 2034 (0.01, 0.2, 0.5, 0.8. 0.99 percentiles of the empirical distribution produced during forecasting)				
	Black	Men	30	0.00146, 0.00184, 0.00231, 0.0029, 0.00365
	Black	Men	31	0.00159, 0.00194, 0.00236, 0.00287, 0.00351
	Black	Men	32	0.00168, 0.002, 0.00239, 0.00285, 0.00341
	Black	Men	33	0.00171, 0.00204, 0.00242, 0.00287, 0.0034
	Black	Men	34	0.00172, 0.00205, 0.00244, 0.0029, 0.00345
	Black	Men	35	0.00172, 0.00206, 0.00245, 0.00292, 0.00349
	Black	Men	36	0.00173, 0.00206, 0.00246, 0.00294, 0.00352
	Black	Men	37	0.00173, 0.00207, 0.00247, 0.00295, 0.00353
	Black	Men	38	0.00173, 0.00207, 0.00247, 0.00296, 0.00355
	Black	Men	39	0.00172, 0.00207, 0.00249, 0.003, 0.00361
	Black	Men	40	0.00171, 0.00208, 0.00253, 0.00308, 0.00375
	Black	Men	41	0.00171, 0.00211, 0.0026, 0.00321, 0.00396
	Black	Men	42	0.00173, 0.00217, 0.00272, 0.0034, 0.00426
	Black	Men	43	0.00178, 0.00227, 0.00288, 0.00366, 0.00466
	Black	Men	44	0.00185, 0.0024, 0.00309, 0.00399, 0.00517
	Black	Men	45	0.00196, 0.00257, 0.00337, 0.00442, 0.0058
	Black	Men	46	0.0021, 0.0028, 0.00371, 0.00491, 0.00653
	Black	Men	47	0.00231, 0.00309, 0.00412, 0.00548, 0.00732
	Black	Men	48	0.0026, 0.00346, 0.0046, 0.00611, 0.00814
	Black	Men	49	0.00296, 0.00391, 0.00516, 0.00679, 0.00897

Variable	Race/ethnicity	Sex	Age	Distribution
	Black	Men	50	0.00341, 0.00445, 0.00579, 0.00754, 0.00983
	Black	Men	51	0.00393, 0.00506, 0.0065, 0.00834, 0.0107
	Black	Men	52	0.00455, 0.00576, 0.00727, 0.00918, 0.0116
	Black	Men	53	0.00528, 0.00655, 0.0081, 0.01, 0.0124
	Black	Men	54	0.00613, 0.00743, 0.00898, 0.0109, 0.0132
	Black	Men	55	0.00706, 0.00838, 0.00991, 0.0117, 0.0139
	Black	Men	56	0.00806, 0.00937, 0.0109, 0.0126, 0.0147
	Black	Men	57	0.0091, 0.0104, 0.0119, 0.0135, 0.0155
	Black	Men	58	0.0102, 0.0114, 0.0129, 0.0145, 0.0163
	Black	Men	59	0.0111, 0.0124, 0.0138, 0.0154, 0.0172
	Black	Men	60	0.0119, 0.0132, 0.0147, 0.0163, 0.0182
	Black	Men	61	0.0127, 0.0141, 0.0155, 0.0172, 0.019
	Black	Men	62	0.0135, 0.0148, 0.0163, 0.0179, 0.0197
	Black	Men	63	0.0141, 0.0155, 0.017, 0.0187, 0.0205
	Black	Men	64	0.0147, 0.0161, 0.0177, 0.0195, 0.0214
	Black	Men	65	0.0152, 0.0168, 0.0185, 0.0204, 0.0224
	Black	Men	66	0.0159, 0.0175, 0.0194, 0.0214, 0.0236
	Black	Men	67	0.0166, 0.0184, 0.0204, 0.0226, 0.0251
	Black	Men	68	0.0174, 0.0194, 0.0216, 0.024, 0.0268
	Black	Men	69	0.0184, 0.0206, 0.023, 0.0257, 0.0287
	Black	Men	70	0.0195, 0.0219, 0.0245, 0.0275, 0.0308
	Black	Men	71	0.0208, 0.0234, 0.0262, 0.0294, 0.0331
	Black	Men	72	0.0223, 0.025, 0.0281, 0.0316, 0.0355
	Black	Men	73	0.0239, 0.0269, 0.0301, 0.0338, 0.038
	Black	Men	74	0.0258, 0.0289, 0.0324, 0.0362, 0.0406
	Black	Men	75	0.028, 0.0312, 0.0348, 0.0388, 0.0433
	Black	Men	76	0.0304, 0.0338, 0.0375, 0.0417, 0.0463
	Black	Men	77	0.0331, 0.0367, 0.0407, 0.045, 0.0499
	Black	Men	78	0.036, 0.0399, 0.0442, 0.0489, 0.0542
	Black	Men	79	0.039, 0.0433, 0.0481, 0.0535, 0.0594
	Black	Men	80	0.0422, 0.0471, 0.0525, 0.0585, 0.0653
	Black	Men	81	0.0456, 0.0511, 0.0572, 0.064, 0.0717
	Black	Men	82	0.049, 0.0552, 0.0621, 0.0698, 0.0786
	Black	Men	83	0.0523, 0.0592, 0.067, 0.0758, 0.0858
	Black	Men	84	0.0555, 0.0631, 0.0718, 0.0816, 0.0929
	Black	Women	30	0.000491, 0.000668, 0.000907, 0.00123, 0.00168
	Black	Women	31	0.000562, 0.000743, 0.000979, 0.00129, 0.0017
	Black	Women	32	0.000645, 0.000825, 0.00105, 0.00134, 0.00172
	Black	Women	33	0.000725, 0.000904, 0.00113, 0.0014, 0.00175
	Black	Women	34	0.000783, 0.000966, 0.00119, 0.00147, 0.00181
	Black	Women	35	0.000814, 0.00101, 0.00124, 0.00154, 0.0019
	Black	Women	36	0.00083, 0.00104, 0.00129, 0.0016, 0.002
	Black	Women	37	0.000848, 0.00106, 0.00133, 0.00166, 0.00208

Variable	Race/ethnicity	Sex	Age	Distribution
	Black	Women	38	0.000873, 0.00109, 0.00137, 0.00172, 0.00215
	Black	Women	39	0.000909, 0.00114, 0.00143, 0.00178, 0.00224
	Black	Women	40	0.000962, 0.0012, 0.0015, 0.00187, 0.00233
	Black	Women	41	0.00104, 0.00129, 0.00159, 0.00197, 0.00244
	Black	Women	42	0.00115, 0.0014, 0.0017, 0.00208, 0.00254
	Black	Women	43	0.00129, 0.00154, 0.00184, 0.0022, 0.00264
	Black	Women	44	0.00146, 0.00171, 0.00201, 0.00235, 0.00276
	Black	Women	45	0.00165, 0.00191, 0.0022, 0.00255, 0.00295
	Black	Women	46	0.00183, 0.00211, 0.00244, 0.00281, 0.00326
	Black	Women	47	0.002, 0.00233, 0.00271, 0.00315, 0.00367
	Black	Women	48	0.00221, 0.00259, 0.00303, 0.00355, 0.00417
	Black	Women	49	0.00246, 0.00289, 0.0034, 0.00399, 0.00469
	Black	Women	50	0.00278, 0.00325, 0.0038, 0.00443, 0.00519
	Black	Women	51	0.0032, 0.00368, 0.00423, 0.00487, 0.0056
	Black	Women	52	0.00369, 0.00417, 0.0047, 0.0053, 0.00598
	Black	Women	53	0.0042, 0.00467, 0.00519, 0.00576, 0.00639
	Black	Women	54	0.00469, 0.00517, 0.00569, 0.00627, 0.00691
	Black	Women	55	0.00515, 0.00566, 0.00621, 0.00682, 0.00749
	Black	Women	56	0.00559, 0.00614, 0.00673, 0.00739, 0.00812
	Black	Women	57	0.006, 0.0066, 0.00724, 0.00795, 0.00874
	Black	Women	58	0.00639, 0.00703, 0.00773, 0.00849, 0.00935
	Black	Women	59	0.0067, 0.0074, 0.00816, 0.00901, 0.00995
	Black	Women	60	0.00696, 0.00772, 0.00856, 0.00949, 0.0105
	Black	Women	61	0.00723, 0.00803, 0.00892, 0.00991, 0.011
	Black	Women	62	0.00752, 0.00836, 0.00928, 0.0103, 0.0114
	Black	Women	63	0.00783, 0.0087, 0.00966, 0.0107, 0.0119
	Black	Women	64	0.00816, 0.00908, 0.0101, 0.0112, 0.0125
	Black	Women	65	0.00854, 0.00954, 0.0106, 0.0119, 0.0133
	Black	Women	66	0.00902, 0.0101, 0.0113, 0.0127, 0.0142
	Black	Women	67	0.00961, 0.0108, 0.0121, 0.0136, 0.0152
	Black	Women	68	0.0103, 0.0116, 0.013, 0.0146, 0.0164
	Black	Women	69	0.0111, 0.0125, 0.014, 0.0158, 0.0178
	Black	Women	70	0.0119, 0.0135, 0.0151, 0.017, 0.0192
	Black	Women	71	0.0129, 0.0145, 0.0163, 0.0184, 0.0207
	Black	Women	72	0.0139, 0.0157, 0.0176, 0.0198, 0.0222
	Black	Women	73	0.0151, 0.017, 0.019, 0.0213, 0.0239
	Black	Women	74	0.0164, 0.0184, 0.0206, 0.023, 0.0257
	Black	Women	75	0.018, 0.02, 0.0223, 0.0249, 0.0277
	Black	Women	76	0.0198, 0.022, 0.0244, 0.027, 0.03
	Black	Women	77	0.0218, 0.0241, 0.0267, 0.0295, 0.0327
	Black	Women	78	0.024, 0.0266, 0.0294, 0.0325, 0.036
	Black	Women	79	0.0265, 0.0294, 0.0325, 0.0359, 0.0398
	Black	Women	80	0.0292, 0.0324, 0.0359, 0.0398, 0.0441

Variable	Race/ethnicity	Sex	Age	Distribution
	Black	Women	81	0.032, 0.0356, 0.0396, 0.044, 0.049
	Black	Women	82	0.0348, 0.039, 0.0435, 0.0487, 0.0545
	Black	Women	83	0.0376, 0.0423, 0.0476, 0.0536, 0.0603
	Black	Women	84	0.0404, 0.0457, 0.0518, 0.0586, 0.0664
	Hispanic	Men	30	0.000781, 0.000891, 0.00102, 0.00116, 0.00132
	Hispanic	Men	31	0.000801, 0.000912, 0.00104, 0.00118, 0.00134
	Hispanic	Men	32	0.000804, 0.000921, 0.00105, 0.00121, 0.00138
	Hispanic	Men	33	0.000784, 0.000914, 0.00106, 0.00124, 0.00145
	Hispanic	Men	34	0.000756, 0.0009, 0.00107, 0.00127, 0.00151
	Hispanic	Men	35	0.000735, 0.000888, 0.00107, 0.00129, 0.00156
	Hispanic	Men	36	0.000726, 0.000883, 0.00107, 0.0013, 0.00158
	Hispanic	Men	37	0.00073, 0.000888, 0.00108, 0.00131, 0.00159
	Hispanic	Men	38	0.000746, 0.000903, 0.00109, 0.00132, 0.00159
	Hispanic	Men	39	0.000768, 0.000926, 0.00111, 0.00134, 0.00162
	Hispanic	Men	40	0.000796, 0.000959, 0.00115, 0.00139, 0.00167
	Hispanic	Men	41	0.000834, 0.001, 0.00121, 0.00145, 0.00175
	Hispanic	Men	42	0.000895, 0.00107, 0.00128, 0.00153, 0.00183
	Hispanic	Men	43	0.000991, 0.00117, 0.00138, 0.00162, 0.00191
	Hispanic	Men	44	0.00112, 0.0013, 0.0015, 0.00173, 0.00201
	Hispanic	Men	45	0.00128, 0.00146, 0.00165, 0.00188, 0.00214
	Hispanic	Men	46	0.00144, 0.00163, 0.00184, 0.00208, 0.00235
	Hispanic	Men	47	0.00162, 0.00183, 0.00207, 0.00233, 0.00264
	Hispanic	Men	48	0.00181, 0.00205, 0.00233, 0.00264, 0.00299
	Hispanic	Men	49	0.00203, 0.00231, 0.00262, 0.00297, 0.00338
	Hispanic	Men	50	0.00229, 0.0026, 0.00294, 0.00332, 0.00376
	Hispanic	Men	51	0.00262, 0.00294, 0.00328, 0.00366, 0.0041
	Hispanic	Men	52	0.00304, 0.00333, 0.00364, 0.00398, 0.00435
	Hispanic	Men	53	0.00352, 0.00376, 0.00401, 0.00428, 0.00457
	Hispanic	Men	54	0.00394, 0.00416, 0.0044, 0.00464, 0.0049
	Hispanic	Men	55	0.00424, 0.00451, 0.00479, 0.0051, 0.00542
	Hispanic	Men	56	0.00449, 0.00483, 0.0052, 0.00559, 0.00602
	Hispanic	Men	57	0.00475, 0.00516, 0.0056, 0.00608, 0.00661
	Hispanic	Men	58	0.00502, 0.00549, 0.006, 0.00656, 0.00718
	Hispanic	Men	59	0.00527, 0.0058, 0.00639, 0.00704, 0.00775
	Hispanic	Men	60	0.00551, 0.00611, 0.00676, 0.00749, 0.0083
	Hispanic	Men	61	0.00579, 0.00643, 0.00712, 0.00789, 0.00876
	Hispanic	Men	62	0.00612, 0.00677, 0.00748, 0.00827, 0.00914
	Hispanic	Men	63	0.00647, 0.00713, 0.00786, 0.00865, 0.00953
	Hispanic	Men	64	0.00682, 0.00752, 0.00827, 0.0091, 0.01
	Hispanic	Men	65	0.00718, 0.00793, 0.00875, 0.00966, 0.0107
	Hispanic	Men	66	0.00758, 0.00841, 0.00932, 0.0103, 0.0115
	Hispanic	Men	67	0.00805, 0.00897, 0.00999, 0.0111, 0.0124
	Hispanic	Men	68	0.00861, 0.00963, 0.0108, 0.012, 0.0135

Variable	Race/ethnicity	Sex	Age	Distribution
	Hispanic	Men	69	0.00927, 0.0104, 0.0117, 0.0131, 0.0147
	Hispanic	Men	70	0.01, 0.0113, 0.0127, 0.0142, 0.016
	Hispanic	Men	71	0.0109, 0.0122, 0.0138, 0.0155, 0.0175
	Hispanic	Men	72	0.0118, 0.0133, 0.015, 0.0169, 0.0191
	Hispanic	Men	73	0.0129, 0.0145, 0.0164, 0.0185, 0.0208
	Hispanic	Men	74	0.0141, 0.0159, 0.0179, 0.0202, 0.0227
	Hispanic	Men	75	0.0155, 0.0175, 0.0196, 0.0221, 0.0248
	Hispanic	Men	76	0.0172, 0.0193, 0.0216, 0.0243, 0.0273
	Hispanic	Men	77	0.0191, 0.0214, 0.024, 0.0268, 0.0301
	Hispanic	Men	78	0.0213, 0.0238, 0.0267, 0.0299, 0.0335
	Hispanic	Men	79	0.0238, 0.0267, 0.0298, 0.0334, 0.0374
	Hispanic	Men	80	0.0267, 0.0299, 0.0334, 0.0374, 0.0418
	Hispanic	Men	81	0.0299, 0.0334, 0.0374, 0.0418, 0.0467
	Hispanic	Men	82	0.0331, 0.0371, 0.0416, 0.0465, 0.0521
	Hispanic	Men	83	0.0363, 0.0409, 0.0459, 0.0515, 0.058
	Hispanic	Men	84	0.0394, 0.0445, 0.0502, 0.0566, 0.0639
	Hispanic	Women	30	0.000322, 0.000365, 0.000413, 0.000467, 0.000528
	Hispanic	Women	31	0.000344, 0.000387, 0.000434, 0.000488, 0.000549
	Hispanic	Women	32	0.000362, 0.000406, 0.000455, 0.00051, 0.000571
	Hispanic	Women	33	0.000374, 0.000421, 0.000474, 0.000533, 0.0006
	Hispanic	Women	34	0.000379, 0.000431, 0.00049, 0.000557, 0.000634
	Hispanic	Women	35	0.000379, 0.000438, 0.000505, 0.000581, 0.000671
	Hispanic	Women	36	0.00038, 0.000444, 0.000518, 0.000605, 0.000707
	Hispanic	Women	37	0.000385, 0.000453, 0.000533, 0.000627, 0.000738
	Hispanic	Women	38	0.000395, 0.000467, 0.00055, 0.000649, 0.000767
	Hispanic	Women	39	0.000412, 0.000486, 0.000572, 0.000674, 0.000795
	Hispanic	Women	40	0.000435, 0.000512, 0.0006, 0.000704, 0.000827
	Hispanic	Women	41	0.000467, 0.000546, 0.000636, 0.000741, 0.000864
	Hispanic	Women	42	0.00051, 0.00059, 0.000681, 0.000786, 0.000909
	Hispanic	Women	43	0.000565, 0.000646, 0.000737, 0.000841, 0.000961
	Hispanic	Women	44	0.000634, 0.000716, 0.000807, 0.000909, 0.00103
	Hispanic	Women	45	0.000713, 0.000797, 0.000891, 0.000996, 0.00111
	Hispanic	Women	46	0.000798, 0.00089, 0.000992, 0.0011, 0.00123
	Hispanic	Women	47	0.000892, 0.000995, 0.00111, 0.00124, 0.00138
	Hispanic	Women	48	0.000995, 0.00111, 0.00125, 0.00139, 0.00156
	Hispanic	Women	49	0.00111, 0.00125, 0.0014, 0.00157, 0.00176
	Hispanic	Women	50	0.00125, 0.0014, 0.00157, 0.00176, 0.00197
	Hispanic	Women	51	0.00141, 0.00157, 0.00175, 0.00195, 0.00217
	Hispanic	Women	52	0.00161, 0.00177, 0.00194, 0.00214, 0.00235
	Hispanic	Women	53	0.00182, 0.00198, 0.00215, 0.00234, 0.00254
	Hispanic	Women	54	0.00201, 0.00218, 0.00236, 0.00256, 0.00278
	Hispanic	Women	55	0.00218, 0.00238, 0.00259, 0.00281, 0.00306
	Hispanic	Women	56	0.00235, 0.00257, 0.00282, 0.00308, 0.00337

Variable	Race/ethnicity	Sex	Age	Distribution
	Hispanic	Women	57	0.00253, 0.00278, 0.00305, 0.00335, 0.00368
	Hispanic	Women	58	0.00271, 0.00299, 0.00329, 0.00362, 0.00398
	Hispanic	Women	59	0.00289, 0.00319, 0.00353, 0.00389, 0.0043
	Hispanic	Women	60	0.00306, 0.0034, 0.00376, 0.00417, 0.00463
	Hispanic	Women	61	0.00325, 0.00361, 0.00401, 0.00445, 0.00495
	Hispanic	Women	62	0.00346, 0.00384, 0.00427, 0.00473, 0.00526
	Hispanic	Women	63	0.00369, 0.0041, 0.00454, 0.00504, 0.00559
	Hispanic	Women	64	0.00393, 0.00437, 0.00485, 0.00539, 0.00599
	Hispanic	Women	65	0.00419, 0.00467, 0.0052, 0.0058, 0.00647
	Hispanic	Women	66	0.00449, 0.00502, 0.00561, 0.00627, 0.00702
	Hispanic	Women	67	0.00484, 0.00543, 0.00609, 0.00682, 0.00765
	Hispanic	Women	68	0.00525, 0.0059, 0.00663, 0.00744, 0.00836
	Hispanic	Women	69	0.00574, 0.00645, 0.00724, 0.00814, 0.00915
	Hispanic	Women	70	0.00628, 0.00707, 0.00794, 0.00892, 0.01
	Hispanic	Women	71	0.0069, 0.00776, 0.00871, 0.00979, 0.011
	Hispanic	Women	72	0.00759, 0.00853, 0.00958, 0.0108, 0.0121
	Hispanic	Women	73	0.00838, 0.00941, 0.0106, 0.0118, 0.0133
	Hispanic	Women	74	0.0093, 0.0104, 0.0117, 0.013, 0.0146
	Hispanic	Women	75	0.0104, 0.0116, 0.0129, 0.0144, 0.0161
	Hispanic	Women	76	0.0117, 0.013, 0.0144, 0.016, 0.0178
	Hispanic	Women	77	0.0132, 0.0146, 0.0162, 0.0179, 0.0198
	Hispanic	Women	78	0.0149, 0.0165, 0.0182, 0.0201, 0.0222
	Hispanic	Women	79	0.0169, 0.0187, 0.0206, 0.0228, 0.0252
	Hispanic	Women	80	0.0191, 0.0211, 0.0234, 0.0258, 0.0286
	Hispanic	Women	81	0.0215, 0.0238, 0.0264, 0.0293, 0.0325
	Hispanic	Women	82	0.024, 0.0267, 0.0298, 0.0332, 0.037
	Hispanic	Women	83	0.0265, 0.0297, 0.0333, 0.0374, 0.0419
	Hispanic	Women	84	0.029, 0.0328, 0.037, 0.0417, 0.047
	White	Men	30	0.000968, 0.00123, 0.00156, 0.00199, 0.00253
	White	Men	31	0.00103, 0.00129, 0.00161, 0.00201, 0.00252
	White	Men	32	0.00108, 0.00133, 0.00164, 0.00202, 0.00249
	White	Men	33	0.00111, 0.00135, 0.00165, 0.00202, 0.00247
	White	Men	34	0.00111, 0.00136, 0.00166, 0.00203, 0.00248
	White	Men	35	0.00109, 0.00135, 0.00165, 0.00203, 0.0025
	White	Men	36	0.00108, 0.00133, 0.00165, 0.00204, 0.00252
	White	Men	37	0.00107, 0.00133, 0.00165, 0.00204, 0.00254
	White	Men	38	0.00108, 0.00134, 0.00166, 0.00205, 0.00255
	White	Men	39	0.0011, 0.00136, 0.00168, 0.00207, 0.00256
	White	Men	40	0.00114, 0.0014, 0.00172, 0.00211, 0.00259
	White	Men	41	0.0012, 0.00146, 0.00179, 0.00218, 0.00266
	White	Men	42	0.00128, 0.00155, 0.00188, 0.00228, 0.00277
	White	Men	43	0.00139, 0.00167, 0.00201, 0.00241, 0.0029
	White	Men	44	0.00153, 0.00183, 0.00217, 0.00259, 0.00308

Variable	Race/ethnicity	Sex	Age	Distribution
	White	Men	45	0.00169, 0.00201, 0.00238, 0.00283, 0.00336
	White	Men	46	0.00187, 0.00222, 0.00265, 0.00315, 0.00375
	White	Men	47	0.00206, 0.00247, 0.00296, 0.00354, 0.00425
	White	Men	48	0.00228, 0.00276, 0.00332, 0.004, 0.00483
	White	Men	49	0.00255, 0.00309, 0.00373, 0.00451, 0.00545
	White	Men	50	0.00288, 0.00347, 0.00418, 0.00504, 0.00607
	White	Men	51	0.00327, 0.00391, 0.00467, 0.00557, 0.00666
	White	Men	52	0.00373, 0.0044, 0.00518, 0.0061, 0.00719
	White	Men	53	0.00424, 0.00492, 0.0057, 0.00661, 0.00768
	White	Men	54	0.00475, 0.00545, 0.00624, 0.00715, 0.00819
	White	Men	55	0.00521, 0.00595, 0.00678, 0.00773, 0.00882
	White	Men	56	0.0056, 0.0064, 0.00732, 0.00836, 0.00956
	White	Men	57	0.00594, 0.00683, 0.00784, 0.009, 0.0103
	White	Men	58	0.00631, 0.00726, 0.00834, 0.00958, 0.011
	White	Men	59	0.0067, 0.00769, 0.00881, 0.0101, 0.0116
	White	Men	60	0.00713, 0.00813, 0.00926, 0.0105, 0.012
	White	Men	61	0.0076, 0.00858, 0.00969, 0.0109, 0.0124
	White	Men	62	0.0081, 0.00906, 0.0101, 0.0113, 0.0127
	White	Men	63	0.0086, 0.00955, 0.0106, 0.0118, 0.0131
	White	Men	64	0.00909, 0.0101, 0.0111, 0.0123, 0.0136
	White	Men	65	0.0096, 0.0106, 0.0118, 0.013, 0.0144
	White	Men	66	0.0102, 0.0113, 0.0125, 0.0139, 0.0154
	White	Men	67	0.0108, 0.0121, 0.0134, 0.015, 0.0167
	White	Men	68	0.0116, 0.013, 0.0145, 0.0162, 0.0181
	White	Men	69	0.0125, 0.014, 0.0157, 0.0176, 0.0198
	White	Men	70	0.0136, 0.0153, 0.0171, 0.0192, 0.0216
	White	Men	71	0.0148, 0.0166, 0.0187, 0.0209, 0.0235
	White	Men	72	0.0162, 0.0181, 0.0203, 0.0228, 0.0256
	White	Men	73	0.0177, 0.0198, 0.0222, 0.0248, 0.0278
	White	Men	74	0.0194, 0.0216, 0.0242, 0.027, 0.0302
	White	Men	75	0.0213, 0.0238, 0.0264, 0.0294, 0.0328
	White	Men	76	0.0236, 0.0262, 0.0291, 0.0322, 0.0357
	White	Men	77	0.0262, 0.029, 0.0321, 0.0355, 0.0394
	White	Men	78	0.0292, 0.0323, 0.0357, 0.0395, 0.0438
	White	Men	79	0.0325, 0.0361, 0.04, 0.0443, 0.0491
	White	Men	80	0.0364, 0.0404, 0.0448, 0.0497, 0.0552
	White	Men	81	0.0407, 0.0452, 0.0503, 0.0559, 0.0622
	White	Men	82	0.0452, 0.0504, 0.0562, 0.0627, 0.07
	White	Men	83	0.0497, 0.0557, 0.0624, 0.07, 0.0785
	White	Men	84	0.0542, 0.061, 0.0687, 0.0773, 0.0871
	White	Women	30	0.000528, 0.000621, 0.000728, 0.000854, 0.001
	White	Women	31	0.000557, 0.000656, 0.000772, 0.000908, 0.00107
	White	Women	32	0.000586, 0.00069, 0.000812, 0.000955, 0.00113

Variable	Race/ethnicity	Sex	Age	Distribution
	White	Women	33	0.000611, 0.00072, 0.000847, 0.000997, 0.00118
	White	Women	34	0.000622, 0.000739, 0.000877, 0.00104, 0.00124
	White	Women	35	0.00062, 0.000749, 0.000903, 0.00109, 0.00132
	White	Women	36	0.000617, 0.000757, 0.000927, 0.00114, 0.00139
	White	Women	37	0.000617, 0.000767, 0.000951, 0.00118, 0.00147
	White	Women	38	0.000621, 0.00078, 0.000978, 0.00123, 0.00154
	White	Women	39	0.00063, 0.0008, 0.00101, 0.00128, 0.00163
	White	Women	40	0.000649, 0.000829, 0.00106, 0.00135, 0.00172
	White	Women	41	0.000681, 0.000872, 0.00111, 0.00142, 0.00182
	White	Women	42	0.000729, 0.000932, 0.00119, 0.00151, 0.00193
	White	Women	43	0.000791, 0.00101, 0.00128, 0.00162, 0.00207
	White	Women	44	0.000865, 0.0011, 0.00139, 0.00176, 0.00224
	White	Women	45	0.00095, 0.00121, 0.00153, 0.00194, 0.00246
	White	Women	46	0.00105, 0.00134, 0.00169, 0.00214, 0.00272
	White	Women	47	0.00119, 0.0015, 0.00188, 0.00237, 0.00299
	White	Women	48	0.00135, 0.00169, 0.0021, 0.00261, 0.00326
	White	Women	49	0.00155, 0.00191, 0.00234, 0.00288, 0.00354
	White	Women	50	0.00175, 0.00214, 0.00261, 0.00318, 0.00389
	White	Women	51	0.00192, 0.00236, 0.00289, 0.00355, 0.00435
	White	Women	52	0.00208, 0.00258, 0.00319, 0.00394, 0.00489
	White	Women	53	0.00226, 0.00281, 0.00349, 0.00434, 0.0054
	White	Women	54	0.00249, 0.00308, 0.0038, 0.00468, 0.00578
	White	Women	55	0.00281, 0.0034, 0.00409, 0.00493, 0.00595
	White	Women	56	0.00321, 0.00375, 0.00438, 0.00511, 0.00597
	White	Women	57	0.00364, 0.00412, 0.00465, 0.00525, 0.00594
	White	Women	58	0.00402, 0.00445, 0.00492, 0.00543, 0.00601
	White	Women	59	0.0043, 0.00472, 0.00518, 0.00569, 0.00625
	White	Women	60	0.00451, 0.00496, 0.00545, 0.00599, 0.0066
	White	Women	61	0.00474, 0.00522, 0.00575, 0.00633, 0.00697
	White	Women	62	0.005, 0.00552, 0.00608, 0.0067, 0.00739
	White	Women	63	0.00532, 0.00586, 0.00646, 0.00712, 0.00785
	White	Women	64	0.00568, 0.00627, 0.00691, 0.00762, 0.00841
	White	Women	65	0.00609, 0.00674, 0.00745, 0.00823, 0.00911
	White	Women	66	0.00654, 0.00727, 0.00807, 0.00896, 0.00996
	White	Women	67	0.00703, 0.00787, 0.0088, 0.00983, 0.011
	White	Women	68	0.00756, 0.00853, 0.00962, 0.0108, 0.0122
	White	Women	69	0.00816, 0.00928, 0.0105, 0.012, 0.0136
	White	Women	70	0.00885, 0.0101, 0.0116, 0.0132, 0.0151
	White	Women	71	0.00963, 0.0111, 0.0127, 0.0145, 0.0167
	White	Women	72	0.0105, 0.0121, 0.0139, 0.016, 0.0184
	White	Women	73	0.0115, 0.0133, 0.0152, 0.0175, 0.0201
	White	Women	74	0.0128, 0.0146, 0.0168, 0.0192, 0.022
	White	Women	75	0.0142, 0.0162, 0.0185, 0.0211, 0.0241

Variable	Race/ethnicity	Sex	Age	Distribution
	White	Women	76	0.0159, 0.0181, 0.0205, 0.0232, 0.0263
	White	Women	77	0.018, 0.0203, 0.0228, 0.0257, 0.029
	White	Women	78	0.0203, 0.0228, 0.0256, 0.0287, 0.0322
	White	Women	79	0.023, 0.0257, 0.0288, 0.0322, 0.0361
	White	Women	80	0.0259, 0.029, 0.0325, 0.0363, 0.0407
	White	Women	81	0.0292, 0.0327, 0.0366, 0.041, 0.0459
	White	Women	82	0.0326, 0.0366, 0.0411, 0.0462, 0.0519
	White	Women	83	0.0361, 0.0408, 0.046, 0.0518, 0.0585
	White	Women	84	0.0397, 0.045, 0.0509, 0.0577, 0.0653
Total mortality rates for 2035 (0.01, 0.2, 0.5, 0.8. 0.99 percentiles of the empirical distribution produced during forecasting)				
	Black	Men	30	0.00145, 0.00184, 0.00231, 0.00292, 0.00369
	Black	Men	31	0.00157, 0.00193, 0.00236, 0.00288, 0.00353
	Black	Men	32	0.00167, 0.002, 0.00239, 0.00286, 0.00343
	Black	Men	33	0.0017, 0.00203, 0.00241, 0.00287, 0.00342
	Black	Men	34	0.00171, 0.00204, 0.00243, 0.0029, 0.00347
	Black	Men	35	0.00171, 0.00204, 0.00244, 0.00292, 0.0035
	Black	Men	36	0.0017, 0.00205, 0.00245, 0.00293, 0.00352
	Black	Men	37	0.0017, 0.00204, 0.00245, 0.00294, 0.00352
	Black	Men	38	0.0017, 0.00204, 0.00245, 0.00294, 0.00354
	Black	Men	39	0.00168, 0.00204, 0.00246, 0.00297, 0.0036
	Black	Men	40	0.00167, 0.00204, 0.0025, 0.00305, 0.00373
	Black	Men	41	0.00167, 0.00207, 0.00256, 0.00317, 0.00394
	Black	Men	42	0.00169, 0.00213, 0.00267, 0.00336, 0.00423
	Black	Men	43	0.00173, 0.00222, 0.00283, 0.00362, 0.00463
	Black	Men	44	0.0018, 0.00235, 0.00305, 0.00395, 0.00514
	Black	Men	45	0.00191, 0.00252, 0.00332, 0.00437, 0.00577
	Black	Men	46	0.00205, 0.00274, 0.00365, 0.00487, 0.00651
	Black	Men	47	0.00225, 0.00303, 0.00406, 0.00544, 0.00731
	Black	Men	48	0.00253, 0.0034, 0.00454, 0.00607, 0.00813
	Black	Men	49	0.0029, 0.00385, 0.0051, 0.00675, 0.00897
	Black	Men	50	0.00334, 0.00438, 0.00573, 0.00751, 0.00985
	Black	Men	51	0.00386, 0.00499, 0.00644, 0.00832, 0.0108
	Black	Men	52	0.00447, 0.00568, 0.00721, 0.00916, 0.0116
	Black	Men	53	0.0052, 0.00647, 0.00804, 0.01, 0.0124
	Black	Men	54	0.00604, 0.00735, 0.00893, 0.0108, 0.0132
	Black	Men	55	0.00697, 0.00829, 0.00985, 0.0117, 0.0139
	Black	Men	56	0.00796, 0.00928, 0.0108, 0.0126, 0.0147
	Black	Men	57	0.00899, 0.0103, 0.0118, 0.0135, 0.0155
	Black	Men	58	0.01, 0.0113, 0.0128, 0.0144, 0.0162
	Black	Men	59	0.011, 0.0123, 0.0137, 0.0153, 0.0171
	Black	Men	60	0.0118, 0.0131, 0.0146, 0.0162, 0.0181
	Black	Men	61	0.0125, 0.0139, 0.0154, 0.017, 0.0189
	Black	Men	62	0.0133, 0.0146, 0.0161, 0.0177, 0.0195

Variable	Race/ethnicity	Sex	Age	Distribution
	Black	Men	63	0.0139, 0.0153, 0.0168, 0.0185, 0.0203
	Black	Men	64	0.0144, 0.0159, 0.0175, 0.0192, 0.0212
	Black	Men	65	0.015, 0.0165, 0.0182, 0.0201, 0.0222
	Black	Men	66	0.0156, 0.0172, 0.0191, 0.0211, 0.0234
	Black	Men	67	0.0163, 0.0181, 0.0201, 0.0223, 0.0248
	Black	Men	68	0.0171, 0.0191, 0.0213, 0.0237, 0.0265
	Black	Men	69	0.018, 0.0202, 0.0226, 0.0253, 0.0284
	Black	Men	70	0.0191, 0.0215, 0.0241, 0.0271, 0.0305
	Black	Men	71	0.0204, 0.023, 0.0258, 0.029, 0.0327
	Black	Men	72	0.0218, 0.0246, 0.0277, 0.0311, 0.0351
	Black	Men	73	0.0234, 0.0264, 0.0296, 0.0333, 0.0375
	Black	Men	74	0.0253, 0.0284, 0.0318, 0.0357, 0.0401
	Black	Men	75	0.0274, 0.0307, 0.0342, 0.0382, 0.0428
	Black	Men	76	0.0298, 0.0332, 0.0369, 0.0411, 0.0458
	Black	Men	77	0.0324, 0.036, 0.04, 0.0444, 0.0493
	Black	Men	78	0.0352, 0.0392, 0.0435, 0.0483, 0.0536
	Black	Men	79	0.0382, 0.0426, 0.0474, 0.0528, 0.0588
	Black	Men	80	0.0413, 0.0463, 0.0517, 0.0578, 0.0647
	Black	Men	81	0.0447, 0.0502, 0.0563, 0.0632, 0.071
	Black	Men	82	0.0481, 0.0543, 0.0612, 0.069, 0.0779
	Black	Men	83	0.0513, 0.0582, 0.0661, 0.0749, 0.085
	Black	Men	84	0.0544, 0.0621, 0.0708, 0.0807, 0.0922
	Black	Women	30	0.000484, 0.000664, 0.000908, 0.00124, 0.0017
	Black	Women	31	0.000555, 0.000738, 0.000979, 0.0013, 0.00173
	Black	Women	32	0.000638, 0.000821, 0.00105, 0.00135, 0.00174
	Black	Women	33	0.000717, 0.000899, 0.00112, 0.00141, 0.00176
	Black	Women	34	0.000774, 0.00096, 0.00119, 0.00147, 0.00182
	Black	Women	35	0.000803, 0.000999, 0.00124, 0.00154, 0.00191
	Black	Women	36	0.000818, 0.00102, 0.00128, 0.0016, 0.00201
	Black	Women	37	0.000833, 0.00105, 0.00132, 0.00166, 0.00209
	Black	Women	38	0.000855, 0.00108, 0.00136, 0.00171, 0.00216
	Black	Women	39	0.000889, 0.00112, 0.00141, 0.00177, 0.00224
	Black	Women	40	0.000939, 0.00118, 0.00148, 0.00185, 0.00233
	Black	Women	41	0.00101, 0.00126, 0.00157, 0.00195, 0.00243
	Black	Women	42	0.00112, 0.00137, 0.00168, 0.00206, 0.00252
	Black	Women	43	0.00126, 0.00151, 0.00181, 0.00218, 0.00262
	Black	Women	44	0.00143, 0.00168, 0.00197, 0.00232, 0.00273
	Black	Women	45	0.00161, 0.00187, 0.00217, 0.00252, 0.00292
	Black	Women	46	0.00178, 0.00207, 0.0024, 0.00278, 0.00323
	Black	Women	47	0.00196, 0.00229, 0.00267, 0.00312, 0.00364
	Black	Women	48	0.00216, 0.00254, 0.00299, 0.00352, 0.00414
	Black	Women	49	0.00241, 0.00284, 0.00335, 0.00396, 0.00467
	Black	Women	50	0.00273, 0.0032, 0.00376, 0.0044, 0.00517

Variable	Race/ethnicity	Sex	Age	Distribution
	Black	Women	51	0.00315, 0.00363, 0.00419, 0.00484, 0.00559
	Black	Women	52	0.00364, 0.00412, 0.00466, 0.00526, 0.00596
	Black	Women	53	0.00415, 0.00462, 0.00514, 0.00572, 0.00638
	Black	Women	54	0.00463, 0.00512, 0.00565, 0.00623, 0.00689
	Black	Women	55	0.00509, 0.00561, 0.00617, 0.00678, 0.00747
	Black	Women	56	0.00553, 0.00608, 0.00669, 0.00735, 0.00809
	Black	Women	57	0.00594, 0.00654, 0.00719, 0.00791, 0.00872
	Black	Women	58	0.00631, 0.00696, 0.00767, 0.00845, 0.00932
	Black	Women	59	0.00661, 0.00732, 0.0081, 0.00896, 0.00992
	Black	Women	60	0.00686, 0.00763, 0.00848, 0.00942, 0.0105
	Black	Women	61	0.00712, 0.00793, 0.00883, 0.00983, 0.011
	Black	Women	62	0.0074, 0.00824, 0.00917, 0.0102, 0.0114
	Black	Women	63	0.00769, 0.00857, 0.00954, 0.0106, 0.0118
	Black	Women	64	0.00801, 0.00894, 0.00997, 0.0111, 0.0124
	Black	Women	65	0.00838, 0.00939, 0.0105, 0.0117, 0.0131
	Black	Women	66	0.00885, 0.00993, 0.0111, 0.0125, 0.014
	Black	Women	67	0.00942, 0.0106, 0.0119, 0.0134, 0.0151
	Black	Women	68	0.0101, 0.0114, 0.0128, 0.0144, 0.0163
	Black	Women	69	0.0108, 0.0122, 0.0138, 0.0156, 0.0176
	Black	Women	70	0.0117, 0.0132, 0.0149, 0.0168, 0.019
	Black	Women	71	0.0126, 0.0143, 0.0161, 0.0181, 0.0204
	Black	Women	72	0.0137, 0.0154, 0.0173, 0.0195, 0.022
	Black	Women	73	0.0148, 0.0166, 0.0187, 0.021, 0.0237
	Black	Women	74	0.0161, 0.018, 0.0202, 0.0227, 0.0255
	Black	Women	75	0.0176, 0.0197, 0.022, 0.0245, 0.0274
	Black	Women	76	0.0194, 0.0215, 0.024, 0.0266, 0.0297
	Black	Women	77	0.0213, 0.0237, 0.0263, 0.0291, 0.0323
	Black	Women	78	0.0236, 0.0261, 0.0289, 0.0321, 0.0356
	Black	Women	79	0.026, 0.0288, 0.032, 0.0354, 0.0393
	Black	Women	80	0.0286, 0.0318, 0.0353, 0.0393, 0.0436
	Black	Women	81	0.0314, 0.035, 0.039, 0.0435, 0.0485
	Black	Women	82	0.0342, 0.0383, 0.0429, 0.0481, 0.0539
	Black	Women	83	0.0369, 0.0416, 0.0469, 0.0529, 0.0598
	Black	Women	84	0.0396, 0.045, 0.051, 0.0579, 0.0657
	Hispanic	Men	30	0.00078, 0.000891, 0.00102, 0.00116, 0.00133
	Hispanic	Men	31	0.0008, 0.000912, 0.00104, 0.00118, 0.00135
	Hispanic	Men	32	0.000803, 0.000921, 0.00105, 0.00121, 0.00138
	Hispanic	Men	33	0.000782, 0.000913, 0.00106, 0.00124, 0.00145
	Hispanic	Men	34	0.000753, 0.000897, 0.00107, 0.00127, 0.00151
	Hispanic	Men	35	0.00073, 0.000883, 0.00107, 0.00129, 0.00156
	Hispanic	Men	36	0.000719, 0.000876, 0.00107, 0.0013, 0.00158
	Hispanic	Men	37	0.000721, 0.000879, 0.00107, 0.0013, 0.00159
	Hispanic	Men	38	0.000735, 0.000892, 0.00108, 0.00131, 0.00159

Variable	Race/ethnicity	Sex	Age	Distribution
	Hispanic	Men	39	0.000756, 0.000914, 0.0011, 0.00133, 0.00161
	Hispanic	Men	40	0.000781, 0.000944, 0.00114, 0.00137, 0.00166
	Hispanic	Men	41	0.000818, 0.000987, 0.00119, 0.00143, 0.00173
	Hispanic	Men	42	0.000877, 0.00105, 0.00126, 0.00151, 0.00181
	Hispanic	Men	43	0.00097, 0.00115, 0.00136, 0.0016, 0.00189
	Hispanic	Men	44	0.0011, 0.00128, 0.00148, 0.00171, 0.00198
	Hispanic	Men	45	0.00125, 0.00143, 0.00163, 0.00185, 0.00212
	Hispanic	Men	46	0.00142, 0.0016, 0.00181, 0.00205, 0.00233
	Hispanic	Men	47	0.00159, 0.0018, 0.00204, 0.00231, 0.00261
	Hispanic	Men	48	0.00178, 0.00202, 0.00229, 0.0026, 0.00296
	Hispanic	Men	49	0.002, 0.00227, 0.00259, 0.00294, 0.00335
	Hispanic	Men	50	0.00226, 0.00256, 0.0029, 0.00329, 0.00373
	Hispanic	Men	51	0.00259, 0.0029, 0.00325, 0.00363, 0.00407
	Hispanic	Men	52	0.003, 0.00329, 0.0036, 0.00395, 0.00433
	Hispanic	Men	53	0.00348, 0.00372, 0.00398, 0.00425, 0.00455
	Hispanic	Men	54	0.0039, 0.00413, 0.00436, 0.00461, 0.00487
	Hispanic	Men	55	0.0042, 0.00447, 0.00476, 0.00507, 0.0054
	Hispanic	Men	56	0.00444, 0.00479, 0.00516, 0.00556, 0.00599
	Hispanic	Men	57	0.0047, 0.00512, 0.00556, 0.00605, 0.00658
	Hispanic	Men	58	0.00496, 0.00544, 0.00596, 0.00652, 0.00715
	Hispanic	Men	59	0.0052, 0.00574, 0.00634, 0.00699, 0.00772
	Hispanic	Men	60	0.00544, 0.00604, 0.0067, 0.00743, 0.00825
	Hispanic	Men	61	0.00571, 0.00634, 0.00705, 0.00782, 0.0087
	Hispanic	Men	62	0.00602, 0.00668, 0.00739, 0.00818, 0.00907
	Hispanic	Men	63	0.00636, 0.00703, 0.00775, 0.00855, 0.00945
	Hispanic	Men	64	0.0067, 0.0074, 0.00816, 0.00899, 0.00992
	Hispanic	Men	65	0.00705, 0.0078, 0.00862, 0.00953, 0.0106
	Hispanic	Men	66	0.00743, 0.00826, 0.00918, 0.0102, 0.0113
	Hispanic	Men	67	0.00789, 0.00881, 0.00983, 0.011, 0.0123
	Hispanic	Men	68	0.00844, 0.00946, 0.0106, 0.0119, 0.0133
	Hispanic	Men	69	0.00908, 0.0102, 0.0115, 0.0129, 0.0145
	Hispanic	Men	70	0.00982, 0.0111, 0.0125, 0.014, 0.0158
	Hispanic	Men	71	0.0106, 0.012, 0.0136, 0.0153, 0.0173
	Hispanic	Men	72	0.0116, 0.0131, 0.0148, 0.0167, 0.0189
	Hispanic	Men	73	0.0126, 0.0143, 0.0161, 0.0182, 0.0206
	Hispanic	Men	74	0.0138, 0.0156, 0.0176, 0.0199, 0.0225
	Hispanic	Men	75	0.0152, 0.0171, 0.0193, 0.0218, 0.0246
	Hispanic	Men	76	0.0168, 0.0189, 0.0213, 0.0239, 0.0269
	Hispanic	Men	77	0.0187, 0.021, 0.0236, 0.0265, 0.0298
	Hispanic	Men	78	0.0209, 0.0234, 0.0263, 0.0295, 0.0331
	Hispanic	Men	79	0.0233, 0.0262, 0.0294, 0.033, 0.037
	Hispanic	Men	80	0.0262, 0.0294, 0.0329, 0.0369, 0.0414
	Hispanic	Men	81	0.0293, 0.0329, 0.0368, 0.0412, 0.0462

Variable	Race/ethnicity	Sex	Age	Distribution
	Hispanic	Men	82	0.0325, 0.0365, 0.041, 0.046, 0.0516
	Hispanic	Men	83	0.0357, 0.0402, 0.0452, 0.0509, 0.0574
	Hispanic	Men	84	0.0387, 0.0438, 0.0495, 0.056, 0.0633
	Hispanic	Women	30	0.000322, 0.000365, 0.000412, 0.000466, 0.000528
	Hispanic	Women	31	0.000344, 0.000386, 0.000434, 0.000488, 0.000548
	Hispanic	Women	32	0.000362, 0.000406, 0.000454, 0.000509, 0.000571
	Hispanic	Women	33	0.000373, 0.00042, 0.000473, 0.000532, 0.0006
	Hispanic	Women	34	0.000377, 0.000429, 0.000489, 0.000556, 0.000634
	Hispanic	Women	35	0.000376, 0.000435, 0.000502, 0.00058, 0.000671
	Hispanic	Women	36	0.000376, 0.000441, 0.000515, 0.000603, 0.000706
	Hispanic	Women	37	0.00038, 0.000448, 0.000529, 0.000623, 0.000736
	Hispanic	Women	38	0.000389, 0.000461, 0.000545, 0.000644, 0.000763
	Hispanic	Women	39	0.000404, 0.000478, 0.000565, 0.000667, 0.00079
	Hispanic	Women	40	0.000427, 0.000503, 0.000592, 0.000696, 0.00082
	Hispanic	Women	41	0.000458, 0.000536, 0.000626, 0.000731, 0.000856
	Hispanic	Women	42	0.000499, 0.000578, 0.00067, 0.000775, 0.000899
	Hispanic	Women	43	0.000553, 0.000633, 0.000725, 0.000829, 0.00095
	Hispanic	Women	44	0.00062, 0.000702, 0.000793, 0.000896, 0.00101
	Hispanic	Women	45	0.000698, 0.000782, 0.000876, 0.000981, 0.0011
	Hispanic	Women	46	0.000782, 0.000874, 0.000976, 0.00109, 0.00122
	Hispanic	Women	47	0.000875, 0.000978, 0.00109, 0.00122, 0.00137
	Hispanic	Women	48	0.000976, 0.0011, 0.00123, 0.00138, 0.00154
	Hispanic	Women	49	0.00109, 0.00123, 0.00138, 0.00155, 0.00175
	Hispanic	Women	50	0.00123, 0.00138, 0.00155, 0.00174, 0.00195
	Hispanic	Women	51	0.00139, 0.00155, 0.00173, 0.00193, 0.00215
	Hispanic	Women	52	0.00159, 0.00175, 0.00193, 0.00212, 0.00233
	Hispanic	Women	53	0.0018, 0.00196, 0.00213, 0.00232, 0.00252
	Hispanic	Women	54	0.00199, 0.00216, 0.00235, 0.00254, 0.00276
	Hispanic	Women	55	0.00216, 0.00236, 0.00257, 0.0028, 0.00305
	Hispanic	Women	56	0.00233, 0.00255, 0.0028, 0.00306, 0.00336
	Hispanic	Women	57	0.0025, 0.00276, 0.00303, 0.00333, 0.00366
	Hispanic	Women	58	0.00269, 0.00296, 0.00326, 0.00359, 0.00396
	Hispanic	Women	59	0.00286, 0.00316, 0.0035, 0.00387, 0.00428
	Hispanic	Women	60	0.00302, 0.00336, 0.00373, 0.00414, 0.0046
	Hispanic	Women	61	0.0032, 0.00357, 0.00397, 0.00441, 0.00491
	Hispanic	Women	62	0.00341, 0.00379, 0.00422, 0.00469, 0.00521
	Hispanic	Women	63	0.00363, 0.00404, 0.00448, 0.00498, 0.00554
	Hispanic	Women	64	0.00386, 0.0043, 0.00479, 0.00532, 0.00593
	Hispanic	Women	65	0.00411, 0.0046, 0.00513, 0.00573, 0.0064
	Hispanic	Women	66	0.0044, 0.00494, 0.00553, 0.00619, 0.00694
	Hispanic	Women	67	0.00475, 0.00534, 0.00599, 0.00673, 0.00757
	Hispanic	Women	68	0.00515, 0.0058, 0.00653, 0.00734, 0.00827
	Hispanic	Women	69	0.00562, 0.00633, 0.00713, 0.00803, 0.00905

Variable	Race/ethnicity	Sex	Age	Distribution
	Hispanic	Women	70	0.00616, 0.00694, 0.00781, 0.0088, 0.00991
	Hispanic	Women	71	0.00676, 0.00762, 0.00857, 0.00965, 0.0109
	Hispanic	Women	72	0.00743, 0.00837, 0.00942, 0.0106, 0.012
	Hispanic	Women	73	0.0082, 0.00923, 0.0104, 0.0117, 0.0131
	Hispanic	Women	74	0.0091, 0.0102, 0.0115, 0.0129, 0.0144
	Hispanic	Women	75	0.0102, 0.0114, 0.0127, 0.0142, 0.0159
	Hispanic	Women	76	0.0115, 0.0128, 0.0142, 0.0158, 0.0175
	Hispanic	Women	77	0.0129, 0.0144, 0.0159, 0.0176, 0.0195
	Hispanic	Women	78	0.0146, 0.0162, 0.0179, 0.0198, 0.022
	Hispanic	Women	79	0.0166, 0.0183, 0.0203, 0.0225, 0.0249
	Hispanic	Women	80	0.0187, 0.0208, 0.023, 0.0255, 0.0283
	Hispanic	Women	81	0.0211, 0.0234, 0.0261, 0.0289, 0.0322
	Hispanic	Women	82	0.0235, 0.0263, 0.0294, 0.0328, 0.0366
	Hispanic	Women	83	0.026, 0.0292, 0.0329, 0.0369, 0.0415
	Hispanic	Women	84	0.0285, 0.0322, 0.0364, 0.0412, 0.0466
	White	Men	30	0.000957, 0.00122, 0.00156, 0.00199, 0.00255
	White	Men	31	0.00102, 0.00128, 0.0016, 0.00201, 0.00253
	White	Men	32	0.00107, 0.00132, 0.00163, 0.00202, 0.0025
	White	Men	33	0.00109, 0.00134, 0.00165, 0.00202, 0.00249
	White	Men	34	0.00109, 0.00134, 0.00165, 0.00203, 0.00249
	White	Men	35	0.00108, 0.00133, 0.00164, 0.00203, 0.00251
	White	Men	36	0.00106, 0.00132, 0.00164, 0.00203, 0.00253
	White	Men	37	0.00105, 0.00131, 0.00163, 0.00203, 0.00254
	White	Men	38	0.00105, 0.00132, 0.00164, 0.00204, 0.00254
	White	Men	39	0.00108, 0.00134, 0.00166, 0.00205, 0.00255
	White	Men	40	0.00112, 0.00138, 0.0017, 0.00209, 0.00257
	White	Men	41	0.00117, 0.00143, 0.00176, 0.00215, 0.00264
	White	Men	42	0.00124, 0.00152, 0.00185, 0.00225, 0.00274
	White	Men	43	0.00135, 0.00163, 0.00197, 0.00238, 0.00287
	White	Men	44	0.00149, 0.00179, 0.00213, 0.00255, 0.00305
	White	Men	45	0.00165, 0.00197, 0.00234, 0.00279, 0.00332
	White	Men	46	0.00182, 0.00218, 0.0026, 0.0031, 0.00371
	White	Men	47	0.00201, 0.00242, 0.00291, 0.0035, 0.00421
	White	Men	48	0.00223, 0.0027, 0.00327, 0.00396, 0.0048
	White	Men	49	0.0025, 0.00304, 0.00368, 0.00446, 0.00542
	White	Men	50	0.00282, 0.00342, 0.00413, 0.00499, 0.00605
	White	Men	51	0.00321, 0.00385, 0.00461, 0.00553, 0.00664
	White	Men	52	0.00366, 0.00434, 0.00512, 0.00605, 0.00716
	White	Men	53	0.00417, 0.00486, 0.00565, 0.00657, 0.00765
	White	Men	54	0.00468, 0.00539, 0.00618, 0.0071, 0.00817
	White	Men	55	0.00514, 0.00588, 0.00672, 0.00768, 0.00879
	White	Men	56	0.00552, 0.00633, 0.00726, 0.00831, 0.00954
	White	Men	57	0.00586, 0.00675, 0.00777, 0.00895, 0.0103

Variable	Race/ethnicity	Sex	Age	Distribution
	White	Men	58	0.00621, 0.00717, 0.00827, 0.00953, 0.011
	White	Men	59	0.0066, 0.00759, 0.00873, 0.01, 0.0115
	White	Men	60	0.00701, 0.00802, 0.00916, 0.0105, 0.012
	White	Men	61	0.00747, 0.00847, 0.00958, 0.0108, 0.0123
	White	Men	62	0.00796, 0.00893, 0.01, 0.0112, 0.0126
	White	Men	63	0.00844, 0.0094, 0.0105, 0.0116, 0.0129
	White	Men	64	0.00892, 0.0099, 0.011, 0.0122, 0.0135
	White	Men	65	0.00941, 0.0104, 0.0116, 0.0128, 0.0143
	White	Men	66	0.00996, 0.0111, 0.0123, 0.0137, 0.0153
	White	Men	67	0.0106, 0.0118, 0.0132, 0.0148, 0.0165
	White	Men	68	0.0114, 0.0127, 0.0143, 0.016, 0.0179
	White	Men	69	0.0123, 0.0138, 0.0155, 0.0174, 0.0196
	White	Men	70	0.0133, 0.015, 0.0168, 0.0189, 0.0213
	White	Men	71	0.0145, 0.0163, 0.0184, 0.0206, 0.0232
	White	Men	72	0.0158, 0.0178, 0.02, 0.0225, 0.0253
	White	Men	73	0.0173, 0.0194, 0.0218, 0.0244, 0.0275
	White	Men	74	0.019, 0.0212, 0.0238, 0.0266, 0.0298
	White	Men	75	0.0209, 0.0233, 0.026, 0.029, 0.0324
	White	Men	76	0.0231, 0.0257, 0.0286, 0.0318, 0.0353
	White	Men	77	0.0257, 0.0285, 0.0316, 0.035, 0.0389
	White	Men	78	0.0286, 0.0317, 0.0352, 0.039, 0.0433
	White	Men	79	0.0319, 0.0354, 0.0393, 0.0437, 0.0485
	White	Men	80	0.0357, 0.0397, 0.0441, 0.0491, 0.0546
	White	Men	81	0.0399, 0.0445, 0.0495, 0.0552, 0.0616
	White	Men	82	0.0443, 0.0496, 0.0554, 0.0619, 0.0693
	White	Men	83	0.0487, 0.0548, 0.0615, 0.0691, 0.0777
	White	Men	84	0.0531, 0.06, 0.0677, 0.0764, 0.0863
	White	Women	30	0.000524, 0.000617, 0.000726, 0.000854, 0.00101
	White	Women	31	0.000553, 0.000653, 0.00077, 0.000908, 0.00107
	White	Women	32	0.000582, 0.000687, 0.00081, 0.000955, 0.00113
	White	Women	33	0.000606, 0.000716, 0.000845, 0.000997, 0.00118
	White	Women	34	0.000615, 0.000734, 0.000874, 0.00104, 0.00124
	White	Women	35	0.000612, 0.000742, 0.000898, 0.00109, 0.00132
	White	Women	36	0.000607, 0.000748, 0.00092, 0.00113, 0.00139
	White	Women	37	0.000606, 0.000756, 0.000941, 0.00117, 0.00146
	White	Women	38	0.000608, 0.000768, 0.000967, 0.00122, 0.00154
	White	Women	39	0.000616, 0.000785, 0.000999, 0.00127, 0.00162
	White	Women	40	0.000633, 0.000812, 0.00104, 0.00133, 0.00171
	White	Women	41	0.000663, 0.000854, 0.0011, 0.00141, 0.00181
	White	Women	42	0.000709, 0.000911, 0.00117, 0.00149, 0.00192
	White	Women	43	0.000769, 0.000984, 0.00126, 0.0016, 0.00205
	White	Women	44	0.000841, 0.00107, 0.00137, 0.00174, 0.00222
	White	Women	45	0.000925, 0.00118, 0.0015, 0.00191, 0.00244

Variable	Race/ethnicity	Sex	Age	Distribution
	White	Women	46	0.00103, 0.00131, 0.00166, 0.00211, 0.0027
	White	Women	47	0.00116, 0.00146, 0.00185, 0.00234, 0.00297
	White	Women	48	0.00132, 0.00165, 0.00207, 0.00258, 0.00323
	White	Women	49	0.00152, 0.00187, 0.00231, 0.00285, 0.00352
	White	Women	50	0.00171, 0.0021, 0.00257, 0.00315, 0.00387
	White	Women	51	0.00188, 0.00232, 0.00286, 0.00352, 0.00434
	White	Women	52	0.00204, 0.00254, 0.00316, 0.00392, 0.00487
	White	Women	53	0.00222, 0.00277, 0.00346, 0.00431, 0.00539
	White	Women	54	0.00245, 0.00304, 0.00376, 0.00465, 0.00577
	White	Women	55	0.00277, 0.00335, 0.00406, 0.0049, 0.00594
	White	Women	56	0.00317, 0.00371, 0.00434, 0.00508, 0.00595
	White	Women	57	0.00359, 0.00407, 0.00461, 0.00522, 0.00592
	White	Women	58	0.00397, 0.0044, 0.00488, 0.0054, 0.00599
	White	Women	59	0.00424, 0.00467, 0.00513, 0.00565, 0.00622
	White	Women	60	0.00444, 0.0049, 0.0054, 0.00595, 0.00656
	White	Women	61	0.00467, 0.00515, 0.00569, 0.00627, 0.00693
	White	Women	62	0.00492, 0.00544, 0.00601, 0.00663, 0.00733
	White	Women	63	0.00523, 0.00578, 0.00638, 0.00704, 0.00778
	White	Women	64	0.00558, 0.00617, 0.00682, 0.00753, 0.00833
	White	Women	65	0.00598, 0.00663, 0.00734, 0.00813, 0.00901
	White	Women	66	0.00641, 0.00714, 0.00795, 0.00885, 0.00985
	White	Women	67	0.00689, 0.00773, 0.00866, 0.0097, 0.0109
	White	Women	68	0.00741, 0.00838, 0.00947, 0.0107, 0.0121
	White	Women	69	0.00799, 0.00911, 0.0104, 0.0118, 0.0135
	White	Women	70	0.00866, 0.00993, 0.0114, 0.013, 0.0149
	White	Women	71	0.00942, 0.0108, 0.0125, 0.0143, 0.0165
	White	Women	72	0.0103, 0.0119, 0.0137, 0.0157, 0.0181
	White	Women	73	0.0113, 0.013, 0.015, 0.0173, 0.0199
	White	Women	74	0.0125, 0.0143, 0.0165, 0.0189, 0.0218
	White	Women	75	0.0139, 0.0159, 0.0182, 0.0208, 0.0238
	White	Women	76	0.0156, 0.0177, 0.0201, 0.0229, 0.026
	White	Women	77	0.0176, 0.0199, 0.0224, 0.0253, 0.0287
	White	Women	78	0.0199, 0.0224, 0.0252, 0.0283, 0.0318
	White	Women	79	0.0225, 0.0252, 0.0283, 0.0318, 0.0357
	White	Women	80	0.0254, 0.0285, 0.032, 0.0358, 0.0402
	White	Women	81	0.0286, 0.0321, 0.036, 0.0404, 0.0454
	White	Women	82	0.032, 0.036, 0.0405, 0.0456, 0.0514
	White	Women	83	0.0354, 0.0401, 0.0453, 0.0512, 0.0579
	White	Women	84	0.0389, 0.0442, 0.0502, 0.057, 0.0647
Total mortality rates for 2036 (0.01, 0.2, 0.5, 0.8. 0.99 percentiles of the empirical distribution produced during forecasting)				
	Black	Men	30	0.00144, 0.00183, 0.00231, 0.00293, 0.00372
	Black	Men	31	0.00156, 0.00192, 0.00236, 0.0029, 0.00356
	Black	Men	32	0.00166, 0.00199, 0.00239, 0.00287, 0.00345

Variable	Race/ethnicity	Sex	Age	Distribution
	Black	Men	33	0.00169, 0.00202, 0.00241, 0.00288, 0.00344
	Black	Men	34	0.00169, 0.00203, 0.00243, 0.0029, 0.00348
	Black	Men	35	0.00169, 0.00203, 0.00244, 0.00292, 0.00351
	Black	Men	36	0.00168, 0.00203, 0.00244, 0.00293, 0.00353
	Black	Men	37	0.00168, 0.00202, 0.00243, 0.00292, 0.00352
	Black	Men	38	0.00167, 0.00201, 0.00243, 0.00292, 0.00353
	Black	Men	39	0.00165, 0.00201, 0.00243, 0.00295, 0.00358
	Black	Men	40	0.00164, 0.00201, 0.00246, 0.00302, 0.0037
	Black	Men	41	0.00163, 0.00203, 0.00253, 0.00314, 0.00391
	Black	Men	42	0.00165, 0.00209, 0.00263, 0.00332, 0.0042
	Black	Men	43	0.00169, 0.00217, 0.00279, 0.00357, 0.00459
	Black	Men	44	0.00176, 0.0023, 0.003, 0.00391, 0.00511
	Black	Men	45	0.00186, 0.00247, 0.00327, 0.00432, 0.00574
	Black	Men	46	0.002, 0.00268, 0.0036, 0.00482, 0.00649
	Black	Men	47	0.0022, 0.00297, 0.004, 0.00539, 0.00729
	Black	Men	48	0.00247, 0.00334, 0.00448, 0.00603, 0.00813
	Black	Men	49	0.00283, 0.00378, 0.00504, 0.00672, 0.00898
	Black	Men	50	0.00327, 0.00431, 0.00568, 0.00747, 0.00986
	Black	Men	51	0.00378, 0.00492, 0.00639, 0.00829, 0.0108
	Black	Men	52	0.00439, 0.00561, 0.00716, 0.00913, 0.0117
	Black	Men	53	0.00512, 0.0064, 0.00799, 0.00997, 0.0125
	Black	Men	54	0.00595, 0.00728, 0.00887, 0.0108, 0.0132
	Black	Men	55	0.00688, 0.00822, 0.0098, 0.0117, 0.0139
	Black	Men	56	0.00786, 0.0092, 0.0108, 0.0126, 0.0147
	Black	Men	57	0.00889, 0.0102, 0.0117, 0.0135, 0.0155
	Black	Men	58	0.00992, 0.0112, 0.0127, 0.0143, 0.0162
	Black	Men	59	0.0108, 0.0121, 0.0136, 0.0152, 0.0171
	Black	Men	60	0.0116, 0.013, 0.0145, 0.0161, 0.018
	Black	Men	61	0.0123, 0.0137, 0.0152, 0.0169, 0.0188
	Black	Men	62	0.0131, 0.0144, 0.0159, 0.0176, 0.0194
	Black	Men	63	0.0137, 0.015, 0.0166, 0.0183, 0.0201
	Black	Men	64	0.0142, 0.0156, 0.0172, 0.019, 0.021
	Black	Men	65	0.0147, 0.0162, 0.018, 0.0199, 0.022
	Black	Men	66	0.0153, 0.0169, 0.0188, 0.0208, 0.0231
	Black	Men	67	0.0159, 0.0178, 0.0198, 0.022, 0.0245
	Black	Men	68	0.0167, 0.0187, 0.0209, 0.0234, 0.0262
	Black	Men	69	0.0176, 0.0198, 0.0223, 0.025, 0.0281
	Black	Men	70	0.0187, 0.0211, 0.0238, 0.0267, 0.0301
	Black	Men	71	0.02, 0.0225, 0.0254, 0.0287, 0.0323
	Black	Men	72	0.0214, 0.0241, 0.0272, 0.0307, 0.0347
	Black	Men	73	0.0229, 0.0259, 0.0292, 0.0329, 0.0371
	Black	Men	74	0.0247, 0.0278, 0.0313, 0.0352, 0.0396
	Black	Men	75	0.0268, 0.0301, 0.0337, 0.0377, 0.0422

Variable	Race/ethnicity	Sex	Age	Distribution
	Black	Men	76	0.0292, 0.0326, 0.0363, 0.0405, 0.0452
	Black	Men	77	0.0318, 0.0354, 0.0394, 0.0438, 0.0488
	Black	Men	78	0.0345, 0.0385, 0.0428, 0.0476, 0.053
	Black	Men	79	0.0374, 0.0418, 0.0467, 0.0521, 0.0582
	Black	Men	80	0.0405, 0.0455, 0.0509, 0.0571, 0.064
	Black	Men	81	0.0438, 0.0494, 0.0555, 0.0625, 0.0703
	Black	Men	82	0.0471, 0.0534, 0.0603, 0.0682, 0.0772
	Black	Men	83	0.0503, 0.0573, 0.0651, 0.074, 0.0843
	Black	Men	84	0.0534, 0.0611, 0.0698, 0.0798, 0.0914
	Black	Women	30	0.000477, 0.00066, 0.000909, 0.00125, 0.00173
	Black	Women	31	0.000548, 0.000734, 0.00098, 0.00131, 0.00175
	Black	Women	32	0.000631, 0.000816, 0.00105, 0.00136, 0.00176
	Black	Women	33	0.00071, 0.000894, 0.00112, 0.00141, 0.00178
	Black	Women	34	0.000766, 0.000954, 0.00119, 0.00148, 0.00184
	Black	Women	35	0.000793, 0.000991, 0.00124, 0.00154, 0.00193
	Black	Women	36	0.000805, 0.00101, 0.00127, 0.0016, 0.00202
	Black	Women	37	0.000818, 0.00104, 0.00131, 0.00165, 0.00209
	Black	Women	38	0.000838, 0.00106, 0.00135, 0.0017, 0.00216
	Black	Women	39	0.00087, 0.0011, 0.00139, 0.00176, 0.00223
	Black	Women	40	0.000917, 0.00116, 0.00146, 0.00184, 0.00232
	Black	Women	41	0.000988, 0.00124, 0.00154, 0.00193, 0.00241
	Black	Women	42	0.00109, 0.00134, 0.00165, 0.00203, 0.00251
	Black	Women	43	0.00123, 0.00148, 0.00178, 0.00215, 0.0026
	Black	Women	44	0.00139, 0.00165, 0.00194, 0.00229, 0.00271
	Black	Women	45	0.00157, 0.00183, 0.00213, 0.00248, 0.00289
	Black	Women	46	0.00175, 0.00203, 0.00236, 0.00275, 0.0032
	Black	Women	47	0.00192, 0.00225, 0.00263, 0.00308, 0.00362
	Black	Women	48	0.00212, 0.0025, 0.00295, 0.00348, 0.00412
	Black	Women	49	0.00236, 0.0028, 0.00331, 0.00392, 0.00465
	Black	Women	50	0.00268, 0.00316, 0.00372, 0.00437, 0.00515
	Black	Women	51	0.00309, 0.00359, 0.00415, 0.0048, 0.00557
	Black	Women	52	0.00359, 0.00407, 0.00462, 0.00523, 0.00594
	Black	Women	53	0.0041, 0.00457, 0.0051, 0.00569, 0.00636
	Black	Women	54	0.00458, 0.00507, 0.00561, 0.0062, 0.00687
	Black	Women	55	0.00504, 0.00556, 0.00612, 0.00675, 0.00744
	Black	Women	56	0.00546, 0.00603, 0.00664, 0.00732, 0.00807
	Black	Women	57	0.00587, 0.00648, 0.00714, 0.00787, 0.00869
	Black	Women	58	0.00624, 0.00689, 0.00761, 0.0084, 0.00929
	Black	Women	59	0.00653, 0.00725, 0.00803, 0.0089, 0.00988
	Black	Women	60	0.00677, 0.00755, 0.00841, 0.00936, 0.0104
	Black	Women	61	0.00701, 0.00783, 0.00874, 0.00975, 0.0109
	Black	Women	62	0.00728, 0.00813, 0.00907, 0.0101, 0.0113
	Black	Women	63	0.00756, 0.00844, 0.00942, 0.0105, 0.0117

Variable	Race/ethnicity	Sex	Age	Distribution
	Black	Women	64	0.00786, 0.0088, 0.00984, 0.011, 0.0123
	Black	Women	65	0.00822, 0.00923, 0.0103, 0.0116, 0.013
	Black	Women	66	0.00868, 0.00977, 0.011, 0.0123, 0.0139
	Black	Women	67	0.00923, 0.0104, 0.0117, 0.0132, 0.0149
	Black	Women	68	0.00988, 0.0112, 0.0126, 0.0142, 0.0161
	Black	Women	69	0.0106, 0.012, 0.0136, 0.0154, 0.0174
	Black	Women	70	0.0115, 0.013, 0.0147, 0.0166, 0.0188
	Black	Women	71	0.0124, 0.014, 0.0158, 0.0179, 0.0202
	Black	Women	72	0.0134, 0.0151, 0.017, 0.0192, 0.0217
	Black	Women	73	0.0145, 0.0163, 0.0184, 0.0207, 0.0234
	Black	Women	74	0.0157, 0.0177, 0.0199, 0.0224, 0.0252
	Black	Women	75	0.0172, 0.0193, 0.0216, 0.0242, 0.0271
	Black	Women	76	0.019, 0.0211, 0.0236, 0.0263, 0.0293
	Black	Women	77	0.0209, 0.0233, 0.0259, 0.0287, 0.032
	Black	Women	78	0.0231, 0.0257, 0.0285, 0.0316, 0.0351
	Black	Women	79	0.0255, 0.0283, 0.0315, 0.035, 0.0389
	Black	Women	80	0.0281, 0.0313, 0.0348, 0.0387, 0.0432
	Black	Women	81	0.0308, 0.0344, 0.0384, 0.0429, 0.048
	Black	Women	82	0.0335, 0.0377, 0.0423, 0.0475, 0.0534
	Black	Women	83	0.0362, 0.041, 0.0463, 0.0523, 0.0592
	Black	Women	84	0.0389, 0.0443, 0.0503, 0.0572, 0.0651
	Hispanic	Men	30	0.000779, 0.000891, 0.00102, 0.00116, 0.00133
	Hispanic	Men	31	0.0008, 0.000912, 0.00104, 0.00118, 0.00135
	Hispanic	Men	32	0.000802, 0.00092, 0.00105, 0.00121, 0.00139
	Hispanic	Men	33	0.000779, 0.000911, 0.00106, 0.00124, 0.00145
	Hispanic	Men	34	0.000749, 0.000894, 0.00107, 0.00127, 0.00152
	Hispanic	Men	35	0.000724, 0.000879, 0.00106, 0.00129, 0.00156
	Hispanic	Men	36	0.000712, 0.00087, 0.00106, 0.0013, 0.00158
	Hispanic	Men	37	0.000713, 0.000871, 0.00106, 0.0013, 0.00158
	Hispanic	Men	38	0.000725, 0.000882, 0.00107, 0.0013, 0.00158
	Hispanic	Men	39	0.000743, 0.000901, 0.00109, 0.00132, 0.0016
	Hispanic	Men	40	0.000767, 0.000929, 0.00112, 0.00136, 0.00165
	Hispanic	Men	41	0.000802, 0.000971, 0.00117, 0.00142, 0.00172
	Hispanic	Men	42	0.000859, 0.00103, 0.00124, 0.00149, 0.0018
	Hispanic	Men	43	0.00095, 0.00113, 0.00133, 0.00158, 0.00187
	Hispanic	Men	44	0.00108, 0.00125, 0.00145, 0.00169, 0.00196
	Hispanic	Men	45	0.00123, 0.0014, 0.0016, 0.00183, 0.00209
	Hispanic	Men	46	0.00139, 0.00158, 0.00179, 0.00203, 0.0023
	Hispanic	Men	47	0.00156, 0.00177, 0.00201, 0.00228, 0.00258
	Hispanic	Men	48	0.00175, 0.00199, 0.00226, 0.00257, 0.00293
	Hispanic	Men	49	0.00196, 0.00224, 0.00255, 0.00291, 0.00332
	Hispanic	Men	50	0.00222, 0.00253, 0.00287, 0.00326, 0.00371
	Hispanic	Men	51	0.00255, 0.00287, 0.00321, 0.0036, 0.00404

Variable	Race/ethnicity	Sex	Age	Distribution
	Hispanic	Men	52	0.00297, 0.00326, 0.00357, 0.00392, 0.0043
	Hispanic	Men	53	0.00344, 0.00369, 0.00394, 0.00422, 0.00452
	Hispanic	Men	54	0.00387, 0.00409, 0.00433, 0.00458, 0.00485
	Hispanic	Men	55	0.00416, 0.00443, 0.00472, 0.00503, 0.00537
	Hispanic	Men	56	0.0044, 0.00475, 0.00512, 0.00552, 0.00596
	Hispanic	Men	57	0.00465, 0.00507, 0.00552, 0.00601, 0.00655
	Hispanic	Men	58	0.00491, 0.00539, 0.00591, 0.00648, 0.00712
	Hispanic	Men	59	0.00514, 0.00569, 0.00628, 0.00694, 0.00768
	Hispanic	Men	60	0.00537, 0.00597, 0.00663, 0.00737, 0.0082
	Hispanic	Men	61	0.00562, 0.00626, 0.00697, 0.00776, 0.00864
	Hispanic	Men	62	0.00593, 0.00658, 0.0073, 0.0081, 0.009
	Hispanic	Men	63	0.00626, 0.00692, 0.00765, 0.00846, 0.00936
	Hispanic	Men	64	0.00658, 0.00728, 0.00804, 0.00888, 0.00982
	Hispanic	Men	65	0.00692, 0.00767, 0.0085, 0.00941, 0.0104
	Hispanic	Men	66	0.00729, 0.00812, 0.00904, 0.0101, 0.0112
	Hispanic	Men	67	0.00774, 0.00866, 0.00968, 0.0108, 0.0121
	Hispanic	Men	68	0.00827, 0.0093, 0.0104, 0.0117, 0.0132
	Hispanic	Men	69	0.0089, 0.01, 0.0113, 0.0127, 0.0143
	Hispanic	Men	70	0.00962, 0.0109, 0.0123, 0.0138, 0.0156
	Hispanic	Men	71	0.0104, 0.0118, 0.0133, 0.0151, 0.0171
	Hispanic	Men	72	0.0113, 0.0128, 0.0145, 0.0164, 0.0186
	Hispanic	Men	73	0.0123, 0.014, 0.0158, 0.0179, 0.0203
	Hispanic	Men	74	0.0135, 0.0153, 0.0173, 0.0196, 0.0222
	Hispanic	Men	75	0.0149, 0.0168, 0.019, 0.0215, 0.0243
	Hispanic	Men	76	0.0165, 0.0186, 0.0209, 0.0236, 0.0266
	Hispanic	Men	77	0.0183, 0.0206, 0.0232, 0.0261, 0.0294
	Hispanic	Men	78	0.0205, 0.023, 0.0259, 0.0291, 0.0327
	Hispanic	Men	79	0.0229, 0.0258, 0.029, 0.0325, 0.0366
	Hispanic	Men	80	0.0257, 0.0289, 0.0324, 0.0364, 0.0409
	Hispanic	Men	81	0.0288, 0.0323, 0.0363, 0.0407, 0.0458
	Hispanic	Men	82	0.0319, 0.0359, 0.0404, 0.0454, 0.0511
	Hispanic	Men	83	0.035, 0.0395, 0.0446, 0.0503, 0.0568
	Hispanic	Men	84	0.038, 0.0431, 0.0488, 0.0553, 0.0627
	Hispanic	Women	30	0.000321, 0.000364, 0.000412, 0.000466, 0.000528
	Hispanic	Women	31	0.000343, 0.000386, 0.000434, 0.000487, 0.000548
	Hispanic	Women	32	0.000361, 0.000405, 0.000454, 0.000509, 0.000571
	Hispanic	Women	33	0.000372, 0.000419, 0.000472, 0.000532, 0.000599
	Hispanic	Women	34	0.000375, 0.000428, 0.000487, 0.000556, 0.000634
	Hispanic	Women	35	0.000373, 0.000432, 0.0005, 0.000579, 0.000671
	Hispanic	Women	36	0.000372, 0.000437, 0.000512, 0.0006, 0.000705
	Hispanic	Women	37	0.000375, 0.000444, 0.000524, 0.00062, 0.000734
	Hispanic	Women	38	0.000383, 0.000455, 0.000539, 0.000639, 0.000759
	Hispanic	Women	39	0.000397, 0.000471, 0.000558, 0.000661, 0.000784

Variable	Race/ethnicity	Sex	Age	Distribution
	Hispanic	Women	40	0.000419, 0.000495, 0.000583, 0.000688, 0.000813
	Hispanic	Women	41	0.000448, 0.000526, 0.000616, 0.000722, 0.000848
	Hispanic	Women	42	0.000488, 0.000567, 0.000659, 0.000765, 0.00089
	Hispanic	Women	43	0.000541, 0.000621, 0.000713, 0.000817, 0.000939
	Hispanic	Women	44	0.000607, 0.000688, 0.000779, 0.000883, 0.001
	Hispanic	Women	45	0.000684, 0.000768, 0.000861, 0.000966, 0.00109
	Hispanic	Women	46	0.000767, 0.000858, 0.00096, 0.00107, 0.0012
	Hispanic	Women	47	0.000858, 0.000961, 0.00108, 0.0012, 0.00135
	Hispanic	Women	48	0.000959, 0.00108, 0.00121, 0.00136, 0.00153
	Hispanic	Women	49	0.00107, 0.00121, 0.00136, 0.00153, 0.00173
	Hispanic	Women	50	0.00121, 0.00136, 0.00153, 0.00172, 0.00194
	Hispanic	Women	51	0.00137, 0.00153, 0.00171, 0.00191, 0.00214
	Hispanic	Women	52	0.00157, 0.00173, 0.00191, 0.0021, 0.00232
	Hispanic	Women	53	0.00178, 0.00194, 0.00211, 0.0023, 0.00251
	Hispanic	Women	54	0.00198, 0.00214, 0.00233, 0.00252, 0.00274
	Hispanic	Women	55	0.00215, 0.00234, 0.00255, 0.00278, 0.00303
	Hispanic	Women	56	0.00231, 0.00253, 0.00278, 0.00304, 0.00334
	Hispanic	Women	57	0.00248, 0.00273, 0.00301, 0.00331, 0.00365
	Hispanic	Women	58	0.00266, 0.00293, 0.00324, 0.00357, 0.00394
	Hispanic	Women	59	0.00282, 0.00313, 0.00347, 0.00384, 0.00426
	Hispanic	Women	60	0.00298, 0.00332, 0.0037, 0.00411, 0.00458
	Hispanic	Women	61	0.00316, 0.00352, 0.00393, 0.00438, 0.00488
	Hispanic	Women	62	0.00336, 0.00374, 0.00417, 0.00464, 0.00517
	Hispanic	Women	63	0.00357, 0.00398, 0.00443, 0.00493, 0.00549
	Hispanic	Women	64	0.0038, 0.00424, 0.00472, 0.00526, 0.00587
	Hispanic	Women	65	0.00404, 0.00452, 0.00506, 0.00565, 0.00633
	Hispanic	Women	66	0.00432, 0.00485, 0.00545, 0.00611, 0.00687
	Hispanic	Women	67	0.00465, 0.00524, 0.0059, 0.00664, 0.00748
	Hispanic	Women	68	0.00505, 0.0057, 0.00642, 0.00724, 0.00817
	Hispanic	Women	69	0.00551, 0.00622, 0.00702, 0.00792, 0.00895
	Hispanic	Women	70	0.00603, 0.00681, 0.00769, 0.00868, 0.0098
	Hispanic	Women	71	0.00662, 0.00748, 0.00844, 0.00952, 0.0108
	Hispanic	Women	72	0.00728, 0.00822, 0.00927, 0.0105, 0.0118
	Hispanic	Women	73	0.00803, 0.00906, 0.0102, 0.0115, 0.013
	Hispanic	Women	74	0.00891, 0.01, 0.0113, 0.0127, 0.0143
	Hispanic	Women	75	0.00997, 0.0112, 0.0125, 0.014, 0.0157
	Hispanic	Women	76	0.0112, 0.0125, 0.0139, 0.0155, 0.0173
	Hispanic	Women	77	0.0127, 0.0141, 0.0156, 0.0174, 0.0193
	Hispanic	Women	78	0.0144, 0.0159, 0.0176, 0.0196, 0.0217
	Hispanic	Women	79	0.0162, 0.018, 0.02, 0.0222, 0.0246
	Hispanic	Women	80	0.0184, 0.0204, 0.0227, 0.0252, 0.028
	Hispanic	Women	81	0.0207, 0.023, 0.0257, 0.0286, 0.0319
	Hispanic	Women	82	0.0231, 0.0259, 0.0289, 0.0324, 0.0363

Variable	Race/ethnicity	Sex	Age	Distribution
	Hispanic	Women	83	0.0255, 0.0288, 0.0324, 0.0365, 0.0411
	Hispanic	Women	84	0.028, 0.0317, 0.0359, 0.0407, 0.0461
	White	Men	30	0.000946, 0.00121, 0.00156, 0.002, 0.00256
	White	Men	31	0.001, 0.00127, 0.0016, 0.00202, 0.00255
	White	Men	32	0.00106, 0.00131, 0.00163, 0.00202, 0.00251
	White	Men	33	0.00108, 0.00133, 0.00164, 0.00202, 0.0025
	White	Men	34	0.00108, 0.00133, 0.00164, 0.00203, 0.0025
	White	Men	35	0.00106, 0.00132, 0.00164, 0.00203, 0.00252
	White	Men	36	0.00104, 0.0013, 0.00162, 0.00203, 0.00253
	White	Men	37	0.00103, 0.00129, 0.00162, 0.00202, 0.00254
	White	Men	38	0.00103, 0.00129, 0.00162, 0.00202, 0.00254
	White	Men	39	0.00105, 0.00131, 0.00163, 0.00203, 0.00253
	White	Men	40	0.00109, 0.00135, 0.00167, 0.00206, 0.00256
	White	Men	41	0.00114, 0.00141, 0.00173, 0.00213, 0.00262
	White	Men	42	0.00121, 0.00149, 0.00182, 0.00222, 0.00272
	White	Men	43	0.00132, 0.0016, 0.00194, 0.00235, 0.00285
	White	Men	44	0.00146, 0.00175, 0.0021, 0.00251, 0.00302
	White	Men	45	0.00161, 0.00193, 0.0023, 0.00275, 0.00329
	White	Men	46	0.00178, 0.00214, 0.00256, 0.00306, 0.00367
	White	Men	47	0.00196, 0.00237, 0.00286, 0.00346, 0.00418
	White	Men	48	0.00218, 0.00265, 0.00322, 0.00391, 0.00476
	White	Men	49	0.00245, 0.00298, 0.00363, 0.00442, 0.00539
	White	Men	50	0.00277, 0.00336, 0.00408, 0.00495, 0.00602
	White	Men	51	0.00315, 0.00379, 0.00456, 0.00549, 0.00661
	White	Men	52	0.0036, 0.00428, 0.00507, 0.00601, 0.00714
	White	Men	53	0.00411, 0.0048, 0.00559, 0.00653, 0.00762
	White	Men	54	0.00462, 0.00532, 0.00613, 0.00706, 0.00814
	White	Men	55	0.00507, 0.00582, 0.00667, 0.00764, 0.00877
	White	Men	56	0.00544, 0.00626, 0.0072, 0.00827, 0.00952
	White	Men	57	0.00577, 0.00668, 0.00771, 0.0089, 0.0103
	White	Men	58	0.00612, 0.00709, 0.0082, 0.00947, 0.011
	White	Men	59	0.0065, 0.0075, 0.00865, 0.00997, 0.0115
	White	Men	60	0.00691, 0.00792, 0.00907, 0.0104, 0.0119
	White	Men	61	0.00735, 0.00835, 0.00947, 0.0107, 0.0122
	White	Men	62	0.00782, 0.0088, 0.00988, 0.0111, 0.0125
	White	Men	63	0.0083, 0.00926, 0.0103, 0.0115, 0.0128
	White	Men	64	0.00876, 0.00974, 0.0108, 0.012, 0.0134
	White	Men	65	0.00924, 0.0103, 0.0114, 0.0127, 0.0141
	White	Men	66	0.00977, 0.0109, 0.0121, 0.0135, 0.0151
	White	Men	67	0.0104, 0.0116, 0.013, 0.0146, 0.0163
	White	Men	68	0.0111, 0.0125, 0.0141, 0.0158, 0.0177
	White	Men	69	0.012, 0.0135, 0.0152, 0.0172, 0.0193
	White	Men	70	0.013, 0.0147, 0.0166, 0.0187, 0.0211

Variable	Race/ethnicity	Sex	Age	Distribution
	White	Men	71	0.0142, 0.016, 0.0181, 0.0204, 0.023
	White	Men	72	0.0155, 0.0175, 0.0197, 0.0222, 0.025
	White	Men	73	0.0169, 0.019, 0.0214, 0.0241, 0.0271
	White	Men	74	0.0185, 0.0208, 0.0234, 0.0262, 0.0294
	White	Men	75	0.0204, 0.0229, 0.0256, 0.0286, 0.032
	White	Men	76	0.0226, 0.0252, 0.0281, 0.0313, 0.0349
	White	Men	77	0.0251, 0.028, 0.0311, 0.0345, 0.0384
	White	Men	78	0.028, 0.0311, 0.0346, 0.0384, 0.0428
	White	Men	79	0.0312, 0.0348, 0.0387, 0.0431, 0.048
	White	Men	80	0.035, 0.039, 0.0435, 0.0484, 0.054
	White	Men	81	0.0391, 0.0437, 0.0488, 0.0545, 0.0609
	White	Men	82	0.0434, 0.0487, 0.0546, 0.0612, 0.0686
	White	Men	83	0.0478, 0.0539, 0.0606, 0.0683, 0.0769
	White	Men	84	0.0521, 0.059, 0.0667, 0.0755, 0.0855
	White	Women	30	0.00052, 0.000614, 0.000724, 0.000854, 0.00101
	White	Women	31	0.000549, 0.00065, 0.000768, 0.000908, 0.00108
	White	Women	32	0.000577, 0.000683, 0.000808, 0.000955, 0.00113
	White	Women	33	0.000601, 0.000712, 0.000842, 0.000996, 0.00118
	White	Women	34	0.000609, 0.000728, 0.00087, 0.00104, 0.00124
	White	Women	35	0.000604, 0.000735, 0.000893, 0.00108, 0.00132
	White	Women	36	0.000598, 0.000739, 0.000913, 0.00113, 0.00139
	White	Women	37	0.000595, 0.000745, 0.000932, 0.00117, 0.00146
	White	Women	38	0.000596, 0.000755, 0.000955, 0.00121, 0.00153
	White	Women	39	0.000602, 0.000771, 0.000985, 0.00126, 0.00161
	White	Women	40	0.000617, 0.000796, 0.00102, 0.00132, 0.0017
	White	Women	41	0.000646, 0.000835, 0.00108, 0.00139, 0.00179
	White	Women	42	0.00069, 0.00089, 0.00115, 0.00147, 0.0019
	White	Women	43	0.000748, 0.000962, 0.00123, 0.00158, 0.00203
	White	Women	44	0.000818, 0.00105, 0.00134, 0.00171, 0.0022
	White	Women	45	0.0009, 0.00115, 0.00147, 0.00188, 0.00241
	White	Women	46	0.000999, 0.00128, 0.00163, 0.00209, 0.00267
	White	Women	47	0.00113, 0.00143, 0.00182, 0.00231, 0.00294
	White	Women	48	0.00129, 0.00162, 0.00203, 0.00255, 0.00321
	White	Women	49	0.00148, 0.00184, 0.00228, 0.00282, 0.00349
	White	Women	50	0.00168, 0.00207, 0.00254, 0.00312, 0.00385
	White	Women	51	0.00185, 0.00229, 0.00282, 0.00349, 0.00432
	White	Women	52	0.00201, 0.0025, 0.00312, 0.00389, 0.00486
	White	Women	53	0.00218, 0.00273, 0.00342, 0.00429, 0.00538
	White	Women	54	0.00241, 0.003, 0.00373, 0.00463, 0.00576
	White	Women	55	0.00273, 0.00331, 0.00402, 0.00488, 0.00593
	White	Women	56	0.00312, 0.00367, 0.0043, 0.00505, 0.00593
	White	Women	57	0.00355, 0.00403, 0.00458, 0.00519, 0.0059
	White	Women	58	0.00392, 0.00436, 0.00484, 0.00537, 0.00596

Variable	Race/ethnicity	Sex	Age	Distribution
	White	Women	59	0.00419, 0.00462, 0.00509, 0.00561, 0.00619
	White	Women	60	0.00438, 0.00485, 0.00535, 0.00591, 0.00653
	White	Women	61	0.0046, 0.00509, 0.00563, 0.00622, 0.00688
	White	Women	62	0.00485, 0.00537, 0.00594, 0.00657, 0.00727
	White	Women	63	0.00514, 0.00569, 0.0063, 0.00697, 0.00771
	White	Women	64	0.00549, 0.00608, 0.00673, 0.00744, 0.00825
	White	Women	65	0.00587, 0.00652, 0.00723, 0.00803, 0.00891
	White	Women	66	0.00629, 0.00702, 0.00783, 0.00873, 0.00975
	White	Women	67	0.00675, 0.00759, 0.00852, 0.00957, 0.0108
	White	Women	68	0.00725, 0.00823, 0.00932, 0.0106, 0.012
	White	Women	69	0.00782, 0.00894, 0.0102, 0.0117, 0.0133
	White	Women	70	0.00847, 0.00974, 0.0112, 0.0128, 0.0148
	White	Women	71	0.00922, 0.0106, 0.0123, 0.0141, 0.0163
	White	Women	72	0.0101, 0.0116, 0.0134, 0.0155, 0.0179
	White	Women	73	0.011, 0.0128, 0.0147, 0.017, 0.0197
	White	Women	74	0.0122, 0.0141, 0.0162, 0.0186, 0.0215
	White	Women	75	0.0136, 0.0156, 0.0179, 0.0205, 0.0235
	White	Women	76	0.0152, 0.0174, 0.0198, 0.0225, 0.0257
	White	Women	77	0.0172, 0.0195, 0.0221, 0.025, 0.0283
	White	Women	78	0.0195, 0.022, 0.0247, 0.0279, 0.0314
	White	Women	79	0.022, 0.0248, 0.0279, 0.0313, 0.0352
	White	Women	80	0.0249, 0.028, 0.0315, 0.0353, 0.0398
	White	Women	81	0.028, 0.0315, 0.0355, 0.0399, 0.045
	White	Women	82	0.0313, 0.0354, 0.0399, 0.045, 0.0508
	White	Women	83	0.0347, 0.0394, 0.0446, 0.0505, 0.0573
	White	Women	84	0.0382, 0.0435, 0.0495, 0.0563, 0.0641
Total mortality rates for 2037 (0.01, 0.2, 0.5, 0.8. 0.99 percentiles of the empirical distribution produced during forecasting)				
	Black	Men	30	0.00143, 0.00182, 0.00232, 0.00294, 0.00375
	Black	Men	31	0.00155, 0.00192, 0.00236, 0.00291, 0.00359
	Black	Men	32	0.00165, 0.00199, 0.00239, 0.00288, 0.00347
	Black	Men	33	0.00168, 0.00202, 0.00241, 0.00289, 0.00346
	Black	Men	34	0.00168, 0.00202, 0.00242, 0.00291, 0.0035
	Black	Men	35	0.00167, 0.00202, 0.00243, 0.00292, 0.00352
	Black	Men	36	0.00166, 0.00201, 0.00242, 0.00292, 0.00353
	Black	Men	37	0.00165, 0.002, 0.00241, 0.00291, 0.00352
	Black	Men	38	0.00164, 0.00199, 0.0024, 0.0029, 0.00352
	Black	Men	39	0.00162, 0.00198, 0.0024, 0.00292, 0.00356
	Black	Men	40	0.0016, 0.00198, 0.00243, 0.00299, 0.00368
	Black	Men	41	0.0016, 0.002, 0.00249, 0.00311, 0.00388
	Black	Men	42	0.00161, 0.00205, 0.00259, 0.00328, 0.00417
	Black	Men	43	0.00165, 0.00213, 0.00274, 0.00353, 0.00456
	Black	Men	44	0.00172, 0.00225, 0.00295, 0.00386, 0.00507
	Black	Men	45	0.00181, 0.00242, 0.00321, 0.00428, 0.00571

Variable	Race/ethnicity	Sex	Age	Distribution
	Black	Men	46	0.00195, 0.00263, 0.00355, 0.00478, 0.00646
	Black	Men	47	0.00214, 0.00291, 0.00395, 0.00535, 0.00727
	Black	Men	48	0.00242, 0.00328, 0.00443, 0.00598, 0.00811
	Black	Men	49	0.00277, 0.00372, 0.00499, 0.00668, 0.00898
	Black	Men	50	0.0032, 0.00425, 0.00562, 0.00744, 0.00987
	Black	Men	51	0.00371, 0.00485, 0.00633, 0.00826, 0.0108
	Black	Men	52	0.00432, 0.00554, 0.00711, 0.00911, 0.0117
	Black	Men	53	0.00504, 0.00633, 0.00794, 0.00995, 0.0125
	Black	Men	54	0.00587, 0.0072, 0.00882, 0.0108, 0.0132
	Black	Men	55	0.00679, 0.00814, 0.00974, 0.0116, 0.014
	Black	Men	56	0.00777, 0.00912, 0.0107, 0.0125, 0.0147
	Black	Men	57	0.00879, 0.0101, 0.0116, 0.0134, 0.0154
	Black	Men	58	0.00981, 0.0111, 0.0126, 0.0143, 0.0162
	Black	Men	59	0.0107, 0.012, 0.0135, 0.0151, 0.017
	Black	Men	60	0.0114, 0.0128, 0.0143, 0.016, 0.0179
	Black	Men	61	0.0122, 0.0135, 0.0151, 0.0168, 0.0187
	Black	Men	62	0.0129, 0.0142, 0.0157, 0.0174, 0.0192
	Black	Men	63	0.0134, 0.0148, 0.0164, 0.0181, 0.0199
	Black	Men	64	0.0139, 0.0154, 0.017, 0.0188, 0.0208
	Black	Men	65	0.0144, 0.016, 0.0177, 0.0196, 0.0217
	Black	Men	66	0.015, 0.0167, 0.0185, 0.0206, 0.0229
	Black	Men	67	0.0156, 0.0175, 0.0195, 0.0217, 0.0243
	Black	Men	68	0.0164, 0.0184, 0.0206, 0.0231, 0.0259
	Black	Men	69	0.0173, 0.0195, 0.0219, 0.0247, 0.0278
	Black	Men	70	0.0183, 0.0207, 0.0234, 0.0264, 0.0298
	Black	Men	71	0.0195, 0.0221, 0.025, 0.0283, 0.032
	Black	Men	72	0.0209, 0.0237, 0.0268, 0.0303, 0.0343
	Black	Men	73	0.0225, 0.0254, 0.0287, 0.0324, 0.0366
	Black	Men	74	0.0242, 0.0273, 0.0308, 0.0347, 0.0391
	Black	Men	75	0.0263, 0.0295, 0.0331, 0.0372, 0.0417
	Black	Men	76	0.0286, 0.032, 0.0357, 0.0399, 0.0447
	Black	Men	77	0.0311, 0.0347, 0.0387, 0.0432, 0.0482
	Black	Men	78	0.0338, 0.0378, 0.0421, 0.047, 0.0524
	Black	Men	79	0.0367, 0.0411, 0.046, 0.0514, 0.0575
	Black	Men	80	0.0398, 0.0447, 0.0502, 0.0563, 0.0633
	Black	Men	81	0.043, 0.0485, 0.0547, 0.0617, 0.0697
	Black	Men	82	0.0462, 0.0525, 0.0595, 0.0674, 0.0764
	Black	Men	83	0.0494, 0.0563, 0.0642, 0.0732, 0.0835
	Black	Men	84	0.0524, 0.0601, 0.0689, 0.0789, 0.0906
	Black	Women	30	0.000471, 0.000655, 0.000909, 0.00126, 0.00176
	Black	Women	31	0.000542, 0.00073, 0.000981, 0.00132, 0.00177
	Black	Women	32	0.000624, 0.000812, 0.00105, 0.00137, 0.00178
	Black	Women	33	0.000703, 0.00089, 0.00112, 0.00142, 0.0018

Variable	Race/ethnicity	Sex	Age	Distribution
	Black	Women	34	0.000758, 0.000948, 0.00118, 0.00148, 0.00185
	Black	Women	35	0.000783, 0.000983, 0.00123, 0.00154, 0.00194
	Black	Women	36	0.000793, 0.001, 0.00127, 0.0016, 0.00203
	Black	Women	37	0.000804, 0.00102, 0.0013, 0.00165, 0.0021
	Black	Women	38	0.000822, 0.00105, 0.00133, 0.0017, 0.00216
	Black	Women	39	0.000851, 0.00108, 0.00138, 0.00175, 0.00223
	Black	Women	40	0.000896, 0.00114, 0.00144, 0.00182, 0.00231
	Black	Women	41	0.000965, 0.00121, 0.00152, 0.00191, 0.0024
	Black	Women	42	0.00106, 0.00132, 0.00163, 0.00201, 0.00249
	Black	Women	43	0.0012, 0.00145, 0.00176, 0.00212, 0.00258
	Black	Women	44	0.00136, 0.00161, 0.00191, 0.00226, 0.00268
	Black	Women	45	0.00154, 0.0018, 0.0021, 0.00245, 0.00287
	Black	Women	46	0.00171, 0.00199, 0.00233, 0.00271, 0.00317
	Black	Women	47	0.00188, 0.00221, 0.0026, 0.00305, 0.00359
	Black	Women	48	0.00207, 0.00246, 0.00291, 0.00345, 0.00409
	Black	Women	49	0.00231, 0.00275, 0.00327, 0.00389, 0.00463
	Black	Women	50	0.00263, 0.00311, 0.00367, 0.00434, 0.00513
	Black	Women	51	0.00305, 0.00354, 0.00411, 0.00477, 0.00555
	Black	Women	52	0.00353, 0.00402, 0.00457, 0.0052, 0.00592
	Black	Women	53	0.00404, 0.00453, 0.00506, 0.00566, 0.00634
	Black	Women	54	0.00453, 0.00502, 0.00557, 0.00617, 0.00684
	Black	Women	55	0.00498, 0.00551, 0.00608, 0.00671, 0.00742
	Black	Women	56	0.00541, 0.00597, 0.00659, 0.00728, 0.00804
	Black	Women	57	0.00581, 0.00642, 0.00709, 0.00783, 0.00866
	Black	Women	58	0.00617, 0.00683, 0.00755, 0.00836, 0.00925
	Black	Women	59	0.00645, 0.00717, 0.00797, 0.00885, 0.00984
	Black	Women	60	0.00668, 0.00746, 0.00833, 0.0093, 0.0104
	Black	Women	61	0.00691, 0.00774, 0.00865, 0.00968, 0.0108
	Black	Women	62	0.00717, 0.00802, 0.00896, 0.01, 0.0112
	Black	Women	63	0.00743, 0.00832, 0.0093, 0.0104, 0.0116
	Black	Women	64	0.00772, 0.00866, 0.0097, 0.0109, 0.0122
	Black	Women	65	0.00807, 0.00908, 0.0102, 0.0115, 0.0129
	Black	Women	66	0.00851, 0.0096, 0.0108, 0.0122, 0.0138
	Black	Women	67	0.00905, 0.0102, 0.0116, 0.0131, 0.0148
	Black	Women	68	0.00968, 0.011, 0.0124, 0.0141, 0.0159
	Black	Women	69	0.0104, 0.0118, 0.0134, 0.0152, 0.0172
	Black	Women	70	0.0112, 0.0127, 0.0144, 0.0164, 0.0186
	Black	Women	71	0.0121, 0.0137, 0.0156, 0.0176, 0.02
	Black	Women	72	0.0131, 0.0148, 0.0168, 0.019, 0.0215
	Black	Women	73	0.0142, 0.016, 0.0181, 0.0204, 0.0231
	Black	Women	74	0.0154, 0.0174, 0.0196, 0.022, 0.0249
	Black	Women	75	0.0169, 0.0189, 0.0212, 0.0238, 0.0268
	Black	Women	76	0.0186, 0.0208, 0.0232, 0.0259, 0.029

Variable	Race/ethnicity	Sex	Age	Distribution
	Black	Women	77	0.0205, 0.0228, 0.0254, 0.0283, 0.0316
	Black	Women	78	0.0226, 0.0252, 0.028, 0.0312, 0.0347
	Black	Women	79	0.025, 0.0278, 0.031, 0.0345, 0.0384
	Black	Women	80	0.0275, 0.0307, 0.0343, 0.0382, 0.0427
	Black	Women	81	0.0302, 0.0338, 0.0379, 0.0424, 0.0475
	Black	Women	82	0.0329, 0.037, 0.0417, 0.0469, 0.0528
	Black	Women	83	0.0355, 0.0403, 0.0456, 0.0517, 0.0586
	Black	Women	84	0.0381, 0.0435, 0.0496, 0.0565, 0.0645
	Hispanic	Men	30	0.000778, 0.00089, 0.00102, 0.00116, 0.00133
	Hispanic	Men	31	0.000799, 0.000911, 0.00104, 0.00118, 0.00135
	Hispanic	Men	32	0.000801, 0.00092, 0.00105, 0.00121, 0.00139
	Hispanic	Men	33	0.000777, 0.00091, 0.00106, 0.00124, 0.00145
	Hispanic	Men	34	0.000746, 0.000892, 0.00106, 0.00127, 0.00152
	Hispanic	Men	35	0.000719, 0.000874, 0.00106, 0.00129, 0.00156
	Hispanic	Men	36	0.000705, 0.000864, 0.00106, 0.00129, 0.00158
	Hispanic	Men	37	0.000704, 0.000863, 0.00105, 0.00129, 0.00158
	Hispanic	Men	38	0.000714, 0.000871, 0.00106, 0.00129, 0.00158
	Hispanic	Men	39	0.000731, 0.000888, 0.00108, 0.00131, 0.00159
	Hispanic	Men	40	0.000753, 0.000915, 0.00111, 0.00134, 0.00163
	Hispanic	Men	41	0.000786, 0.000955, 0.00116, 0.0014, 0.0017
	Hispanic	Men	42	0.000842, 0.00102, 0.00122, 0.00147, 0.00178
	Hispanic	Men	43	0.000931, 0.00111, 0.00131, 0.00156, 0.00185
	Hispanic	Men	44	0.00106, 0.00123, 0.00143, 0.00166, 0.00194
	Hispanic	Men	45	0.0012, 0.00138, 0.00158, 0.0018, 0.00207
	Hispanic	Men	46	0.00136, 0.00155, 0.00176, 0.002, 0.00227
	Hispanic	Men	47	0.00153, 0.00174, 0.00198, 0.00225, 0.00256
	Hispanic	Men	48	0.00172, 0.00196, 0.00223, 0.00254, 0.0029
	Hispanic	Men	49	0.00193, 0.00221, 0.00252, 0.00288, 0.00329
	Hispanic	Men	50	0.00219, 0.0025, 0.00284, 0.00323, 0.00368
	Hispanic	Men	51	0.00252, 0.00283, 0.00318, 0.00357, 0.00402
	Hispanic	Men	52	0.00293, 0.00322, 0.00354, 0.00389, 0.00427
	Hispanic	Men	53	0.00341, 0.00365, 0.00391, 0.00419, 0.00449
	Hispanic	Men	54	0.00383, 0.00406, 0.0043, 0.00455, 0.00482
	Hispanic	Men	55	0.00412, 0.00439, 0.00469, 0.005, 0.00534
	Hispanic	Men	56	0.00436, 0.00471, 0.00509, 0.00549, 0.00594
	Hispanic	Men	57	0.0046, 0.00503, 0.00548, 0.00598, 0.00652
	Hispanic	Men	58	0.00485, 0.00534, 0.00586, 0.00644, 0.00708
	Hispanic	Men	59	0.00508, 0.00563, 0.00623, 0.00689, 0.00764
	Hispanic	Men	60	0.0053, 0.0059, 0.00657, 0.00732, 0.00815
	Hispanic	Men	61	0.00554, 0.00619, 0.0069, 0.00769, 0.00858
	Hispanic	Men	62	0.00584, 0.00649, 0.00722, 0.00802, 0.00892
	Hispanic	Men	63	0.00615, 0.00682, 0.00755, 0.00836, 0.00927
	Hispanic	Men	64	0.00647, 0.00716, 0.00793, 0.00878, 0.00972

Variable	Race/ethnicity	Sex	Age	Distribution
	Hispanic	Men	65	0.00679, 0.00754, 0.00837, 0.00929, 0.0103
	Hispanic	Men	66	0.00715, 0.00798, 0.0089, 0.00993, 0.0111
	Hispanic	Men	67	0.00759, 0.00851, 0.00953, 0.0107, 0.012
	Hispanic	Men	68	0.00811, 0.00913, 0.0103, 0.0116, 0.013
	Hispanic	Men	69	0.00872, 0.00985, 0.0111, 0.0126, 0.0142
	Hispanic	Men	70	0.00943, 0.0107, 0.0121, 0.0137, 0.0155
	Hispanic	Men	71	0.0102, 0.0116, 0.0131, 0.0149, 0.0169
	Hispanic	Men	72	0.0111, 0.0126, 0.0143, 0.0162, 0.0184
	Hispanic	Men	73	0.0121, 0.0137, 0.0156, 0.0177, 0.0201
	Hispanic	Men	74	0.0132, 0.015, 0.017, 0.0193, 0.0219
	Hispanic	Men	75	0.0146, 0.0165, 0.0187, 0.0212, 0.024
	Hispanic	Men	76	0.0162, 0.0183, 0.0206, 0.0233, 0.0263
	Hispanic	Men	77	0.018, 0.0203, 0.0229, 0.0258, 0.0291
	Hispanic	Men	78	0.0201, 0.0226, 0.0255, 0.0287, 0.0324
	Hispanic	Men	79	0.0225, 0.0253, 0.0285, 0.0321, 0.0362
	Hispanic	Men	80	0.0252, 0.0284, 0.032, 0.036, 0.0405
	Hispanic	Men	81	0.0282, 0.0318, 0.0358, 0.0402, 0.0453
	Hispanic	Men	82	0.0313, 0.0353, 0.0398, 0.0448, 0.0506
	Hispanic	Men	83	0.0344, 0.0389, 0.044, 0.0497, 0.0563
	Hispanic	Men	84	0.0373, 0.0424, 0.0481, 0.0547, 0.0621
	Hispanic	Women	30	0.000321, 0.000364, 0.000412, 0.000466, 0.000528
	Hispanic	Women	31	0.000343, 0.000386, 0.000433, 0.000487, 0.000548
	Hispanic	Women	32	0.000361, 0.000405, 0.000454, 0.000508, 0.00057
	Hispanic	Women	33	0.000371, 0.000418, 0.000471, 0.000531, 0.000599
	Hispanic	Women	34	0.000373, 0.000426, 0.000486, 0.000555, 0.000634
	Hispanic	Women	35	0.00037, 0.00043, 0.000498, 0.000577, 0.00067
	Hispanic	Women	36	0.000368, 0.000433, 0.000509, 0.000598, 0.000704
	Hispanic	Women	37	0.00037, 0.000439, 0.00052, 0.000616, 0.000732
	Hispanic	Women	38	0.000377, 0.000449, 0.000534, 0.000634, 0.000755
	Hispanic	Women	39	0.00039, 0.000464, 0.000551, 0.000655, 0.000779
	Hispanic	Women	40	0.000411, 0.000486, 0.000575, 0.00068, 0.000806
	Hispanic	Women	41	0.000439, 0.000517, 0.000607, 0.000713, 0.00084
	Hispanic	Women	42	0.000477, 0.000557, 0.000648, 0.000755, 0.00088
	Hispanic	Women	43	0.000529, 0.000609, 0.000701, 0.000806, 0.000928
	Hispanic	Women	44	0.000594, 0.000675, 0.000766, 0.000869, 0.000988
	Hispanic	Women	45	0.00067, 0.000753, 0.000847, 0.000952, 0.00107
	Hispanic	Women	46	0.000752, 0.000843, 0.000944, 0.00106, 0.00119
	Hispanic	Women	47	0.000842, 0.000945, 0.00106, 0.00119, 0.00133
	Hispanic	Women	48	0.000941, 0.00106, 0.00119, 0.00134, 0.00151
	Hispanic	Women	49	0.00105, 0.00119, 0.00135, 0.00152, 0.00172
	Hispanic	Women	50	0.00119, 0.00134, 0.00151, 0.00171, 0.00192
	Hispanic	Women	51	0.00135, 0.00152, 0.0017, 0.0019, 0.00212
	Hispanic	Women	52	0.00155, 0.00171, 0.00189, 0.00208, 0.0023

Variable	Race/ethnicity	Sex	Age	Distribution
	Hispanic	Women	53	0.00176, 0.00192, 0.00209, 0.00228, 0.00249
	Hispanic	Women	54	0.00196, 0.00213, 0.00231, 0.00251, 0.00272
	Hispanic	Women	55	0.00213, 0.00232, 0.00253, 0.00276, 0.00301
	Hispanic	Women	56	0.00229, 0.00251, 0.00276, 0.00302, 0.00332
	Hispanic	Women	57	0.00245, 0.00271, 0.00298, 0.00329, 0.00363
	Hispanic	Women	58	0.00263, 0.00291, 0.00321, 0.00355, 0.00393
	Hispanic	Women	59	0.00279, 0.0031, 0.00344, 0.00381, 0.00424
	Hispanic	Women	60	0.00294, 0.00328, 0.00366, 0.00408, 0.00455
	Hispanic	Women	61	0.00311, 0.00348, 0.00389, 0.00434, 0.00485
	Hispanic	Women	62	0.00331, 0.00369, 0.00412, 0.0046, 0.00513
	Hispanic	Women	63	0.00351, 0.00392, 0.00437, 0.00487, 0.00544
	Hispanic	Women	64	0.00373, 0.00417, 0.00466, 0.0052, 0.00581
	Hispanic	Women	65	0.00397, 0.00445, 0.00498, 0.00558, 0.00626
	Hispanic	Women	66	0.00424, 0.00477, 0.00537, 0.00603, 0.00679
	Hispanic	Women	67	0.00456, 0.00515, 0.00581, 0.00655, 0.0074
	Hispanic	Women	68	0.00495, 0.0056, 0.00632, 0.00715, 0.00808
	Hispanic	Women	69	0.0054, 0.00611, 0.00691, 0.00781, 0.00884
	Hispanic	Women	70	0.00591, 0.00669, 0.00757, 0.00856, 0.00969
	Hispanic	Women	71	0.00648, 0.00734, 0.0083, 0.00939, 0.0106
	Hispanic	Women	72	0.00713, 0.00807, 0.00912, 0.0103, 0.0117
	Hispanic	Women	73	0.00786, 0.00889, 0.01, 0.0113, 0.0128
	Hispanic	Women	74	0.00873, 0.00984, 0.0111, 0.0125, 0.0141
	Hispanic	Women	75	0.00977, 0.011, 0.0123, 0.0138, 0.0155
	Hispanic	Women	76	0.011, 0.0123, 0.0137, 0.0153, 0.0171
	Hispanic	Women	77	0.0124, 0.0138, 0.0154, 0.0171, 0.0191
	Hispanic	Women	78	0.0141, 0.0156, 0.0174, 0.0193, 0.0214
	Hispanic	Women	79	0.0159, 0.0177, 0.0197, 0.0219, 0.0243
	Hispanic	Women	80	0.018, 0.0201, 0.0223, 0.0248, 0.0277
	Hispanic	Women	81	0.0203, 0.0227, 0.0253, 0.0282, 0.0315
	Hispanic	Women	82	0.0226, 0.0254, 0.0285, 0.032, 0.0359
	Hispanic	Women	83	0.025, 0.0283, 0.0319, 0.036, 0.0407
	Hispanic	Women	84	0.0275, 0.0312, 0.0354, 0.0402, 0.0457
	White	Men	30	0.000935, 0.00121, 0.00155, 0.002, 0.00258
	White	Men	31	0.000994, 0.00126, 0.0016, 0.00202, 0.00256
	White	Men	32	0.00105, 0.00131, 0.00163, 0.00202, 0.00253
	White	Men	33	0.00107, 0.00133, 0.00164, 0.00202, 0.00251
	White	Men	34	0.00107, 0.00132, 0.00164, 0.00203, 0.00251
	White	Men	35	0.00105, 0.00131, 0.00163, 0.00202, 0.00252
	White	Men	36	0.00103, 0.00129, 0.00161, 0.00202, 0.00253
	White	Men	37	0.00101, 0.00127, 0.0016, 0.00201, 0.00254
	White	Men	38	0.00101, 0.00127, 0.0016, 0.00201, 0.00253
	White	Men	39	0.00103, 0.00129, 0.00161, 0.00201, 0.00252
	White	Men	40	0.00107, 0.00133, 0.00165, 0.00204, 0.00254

Variable	Race/ethnicity	Sex	Age	Distribution
	White	Men	41	0.00111, 0.00138, 0.0017, 0.0021, 0.0026
	White	Men	42	0.00118, 0.00145, 0.00178, 0.00219, 0.00269
	White	Men	43	0.00129, 0.00157, 0.0019, 0.00231, 0.00282
	White	Men	44	0.00142, 0.00171, 0.00206, 0.00248, 0.00299
	White	Men	45	0.00157, 0.00189, 0.00226, 0.00271, 0.00325
	White	Men	46	0.00174, 0.00209, 0.00251, 0.00302, 0.00364
	White	Men	47	0.00192, 0.00233, 0.00282, 0.00341, 0.00414
	White	Men	48	0.00213, 0.00261, 0.00317, 0.00387, 0.00472
	White	Men	49	0.00239, 0.00293, 0.00358, 0.00437, 0.00535
	White	Men	50	0.00271, 0.00331, 0.00403, 0.0049, 0.00598
	White	Men	51	0.00309, 0.00374, 0.00451, 0.00544, 0.00658
	White	Men	52	0.00354, 0.00422, 0.00502, 0.00597, 0.00711
	White	Men	53	0.00404, 0.00474, 0.00554, 0.00648, 0.00759
	White	Men	54	0.00455, 0.00526, 0.00608, 0.00701, 0.00811
	White	Men	55	0.005, 0.00575, 0.00661, 0.00759, 0.00874
	White	Men	56	0.00537, 0.00619, 0.00714, 0.00822, 0.00949
	White	Men	57	0.0057, 0.0066, 0.00765, 0.00885, 0.0103
	White	Men	58	0.00604, 0.00701, 0.00813, 0.00942, 0.0109
	White	Men	59	0.0064, 0.00741, 0.00857, 0.00991, 0.0115
	White	Men	60	0.0068, 0.00782, 0.00898, 0.0103, 0.0119
	White	Men	61	0.00723, 0.00824, 0.00937, 0.0107, 0.0121
	White	Men	62	0.00769, 0.00867, 0.00976, 0.011, 0.0124
	White	Men	63	0.00815, 0.00911, 0.0102, 0.0114, 0.0127
	White	Men	64	0.0086, 0.00958, 0.0107, 0.0119, 0.0132
	White	Men	65	0.00906, 0.0101, 0.0112, 0.0125, 0.014
	White	Men	66	0.00958, 0.0107, 0.012, 0.0134, 0.0149
	White	Men	67	0.0102, 0.0114, 0.0128, 0.0144, 0.0161
	White	Men	68	0.0109, 0.0123, 0.0138, 0.0156, 0.0175
	White	Men	69	0.0118, 0.0133, 0.015, 0.0169, 0.0191
	White	Men	70	0.0128, 0.0144, 0.0163, 0.0184, 0.0209
	White	Men	71	0.0139, 0.0157, 0.0178, 0.0201, 0.0227
	White	Men	72	0.0151, 0.0171, 0.0193, 0.0218, 0.0247
	White	Men	73	0.0166, 0.0187, 0.0211, 0.0238, 0.0268
	White	Men	74	0.0182, 0.0204, 0.023, 0.0258, 0.0291
	White	Men	75	0.02, 0.0224, 0.0251, 0.0282, 0.0316
	White	Men	76	0.0222, 0.0248, 0.0276, 0.0309, 0.0345
	White	Men	77	0.0246, 0.0275, 0.0306, 0.0341, 0.038
	White	Men	78	0.0274, 0.0306, 0.034, 0.0379, 0.0423
	White	Men	79	0.0306, 0.0342, 0.0381, 0.0425, 0.0474
	White	Men	80	0.0343, 0.0383, 0.0428, 0.0478, 0.0534
	White	Men	81	0.0383, 0.0429, 0.0481, 0.0538, 0.0603
	White	Men	82	0.0426, 0.0479, 0.0538, 0.0604, 0.0679
	White	Men	83	0.0469, 0.053, 0.0598, 0.0674, 0.0762

Variable	Race/ethnicity	Sex	Age	Distribution
	White	Men	84	0.0511, 0.058, 0.0658, 0.0746, 0.0847
	White	Women	30	0.000517, 0.000611, 0.000723, 0.000854, 0.00101
	White	Women	31	0.000545, 0.000647, 0.000766, 0.000908, 0.00108
	White	Women	32	0.000573, 0.00068, 0.000806, 0.000955, 0.00113
	White	Women	33	0.000596, 0.000708, 0.000839, 0.000995, 0.00118
	White	Women	34	0.000603, 0.000723, 0.000866, 0.00104, 0.00124
	White	Women	35	0.000597, 0.000728, 0.000888, 0.00108, 0.00132
	White	Women	36	0.000589, 0.000731, 0.000906, 0.00112, 0.00139
	White	Women	37	0.000584, 0.000735, 0.000923, 0.00116, 0.00146
	White	Women	38	0.000584, 0.000743, 0.000944, 0.0012, 0.00153
	White	Women	39	0.000588, 0.000757, 0.000971, 0.00125, 0.0016
	White	Women	40	0.000602, 0.00078, 0.00101, 0.0013, 0.00169
	White	Women	41	0.00063, 0.000818, 0.00106, 0.00137, 0.00178
	White	Women	42	0.000672, 0.000871, 0.00113, 0.00145, 0.00188
	White	Women	43	0.000728, 0.00094, 0.00121, 0.00156, 0.00201
	White	Women	44	0.000796, 0.00103, 0.00132, 0.00169, 0.00218
	White	Women	45	0.000876, 0.00113, 0.00145, 0.00186, 0.00239
	White	Women	46	0.000973, 0.00125, 0.0016, 0.00206, 0.00265
	White	Women	47	0.0011, 0.0014, 0.00179, 0.00228, 0.00292
	White	Women	48	0.00126, 0.00159, 0.002, 0.00252, 0.00318
	White	Women	49	0.00145, 0.00181, 0.00224, 0.00279, 0.00347
	White	Women	50	0.00164, 0.00203, 0.00251, 0.00309, 0.00383
	White	Women	51	0.00181, 0.00225, 0.00279, 0.00346, 0.0043
	White	Women	52	0.00197, 0.00247, 0.00309, 0.00386, 0.00484
	White	Women	53	0.00214, 0.0027, 0.00339, 0.00426, 0.00536
	White	Women	54	0.00237, 0.00296, 0.00369, 0.0046, 0.00575
	White	Women	55	0.00269, 0.00328, 0.00399, 0.00485, 0.00591
	White	Women	56	0.00308, 0.00363, 0.00427, 0.00502, 0.00591
	White	Women	57	0.0035, 0.00399, 0.00454, 0.00516, 0.00588
	White	Women	58	0.00388, 0.00431, 0.0048, 0.00533, 0.00593
	White	Women	59	0.00413, 0.00457, 0.00505, 0.00557, 0.00616
	White	Women	60	0.00433, 0.00479, 0.0053, 0.00586, 0.00649
	White	Women	61	0.00453, 0.00503, 0.00557, 0.00617, 0.00684
	White	Women	62	0.00477, 0.0053, 0.00587, 0.0065, 0.00722
	White	Women	63	0.00506, 0.00561, 0.00622, 0.00689, 0.00764
	White	Women	64	0.00539, 0.00598, 0.00663, 0.00735, 0.00816
	White	Women	65	0.00576, 0.00641, 0.00713, 0.00792, 0.00882
	White	Women	66	0.00617, 0.0069, 0.00771, 0.00862, 0.00964
	White	Women	67	0.00662, 0.00746, 0.00839, 0.00944, 0.0106
	White	Women	68	0.00711, 0.00808, 0.00917, 0.0104, 0.0118
	White	Women	69	0.00766, 0.00877, 0.01, 0.0115, 0.0132
	White	Women	70	0.00829, 0.00956, 0.011, 0.0127, 0.0146
	White	Women	71	0.00902, 0.0104, 0.0121, 0.0139, 0.0161

Variable	Race/ethnicity	Sex	Age	Distribution
	White	Women	72	0.00984, 0.0114, 0.0132, 0.0153, 0.0177
	White	Women	73	0.0108, 0.0125, 0.0145, 0.0168, 0.0194
	White	Women	74	0.0119, 0.0138, 0.0159, 0.0184, 0.0212
	White	Women	75	0.0133, 0.0153, 0.0176, 0.0202, 0.0232
	White	Women	76	0.0149, 0.017, 0.0195, 0.0222, 0.0254
	White	Women	77	0.0168, 0.0191, 0.0217, 0.0246, 0.028
	White	Women	78	0.0191, 0.0215, 0.0243, 0.0275, 0.0311
	White	Women	79	0.0216, 0.0243, 0.0274, 0.0309, 0.0348
	White	Women	80	0.0244, 0.0275, 0.031, 0.0349, 0.0393
	White	Women	81	0.0275, 0.031, 0.0349, 0.0394, 0.0445
	White	Women	82	0.0307, 0.0348, 0.0393, 0.0444, 0.0503
	White	Women	83	0.034, 0.0387, 0.0439, 0.0499, 0.0567
	White	Women	84	0.0374, 0.0427, 0.0487, 0.0556, 0.0635
